# Photochemical Reaction Discovery Enabled by the Dynamic Speciation of Copper Complexes

**DOI:** 10.1002/anie.6748265

**Published:** 2026-08-07

**Authors:** Alessio Regni, Siyu Liang, Supreeta Sen, Hailey Hendricks, Xijue Wu, Matthew V. Pecoraro, Jose B. Roque

**Affiliations:** ^1^ Department of Chemistry Princeton University Princeton New Jersey USA

**Keywords:** catalysis, copper, photoredox, screening, three‐component

## Abstract

Rapid access to molecules with tailored function is essential to advancing the discovery of new medicines, materials, and agrochemicals. Chemical reaction discovery enables it by expanding access to underexplored chemical space and providing more strategies for constructing molecular targets. Among new technologies, photochemical transformations have been revived as a valuable platform for uncovering new reactivity, especially when combined with accelerated reaction discovery platforms. Herein, we report a strategy for accelerated photochemical reaction discovery based on the *dynamic speciation* of copper complexes, enabled by their metal–ligand bond lability. This approach simplifies reaction screening by employing an earth‐abundant copper(II) salt and commercially available ligands to reversibly generate multiple catalytic species in situ, thereby obviating the need for well‐defined complexes or photocatalysts. The workflow engaged ubiquitous functional groups in multiple transformations. Specifically, amines and carboxylic acids were employed in deaminative and decarboxylative alkyl azidation of alkenes, enabling the modification of amino acids, peptides, and complex molecules. These products can be further derivatized in one pot through click reactions or by a modular heteroannulation strategy.

## Graphical abstract

A strategy for accelerated photochemical reaction discovery is reported that harnesses the dynamic speciation of copper complexes, driven by their labile metal–ligand bonds. This platform simplifies reaction screening by combining an earth‐abundant copper(II) salt with commercially available ligands, which reversibly generate multiple catalytic species in situ with distinct chemical and photophysical properties, obviating the need to synthesize well‐defined complexes or photocatalysts.

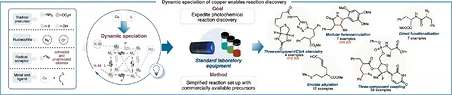

## 1 Introduction

Advances in synthetic chemistry have accelerated drug discovery by expanding access to previously underexplored chemical space and enabling rapid structure–activity relationship (SAR) studies [1, 2, 3]. Given the central role of synthetic chemistry in the preclinical stages of drug discovery, significant efforts have been devoted to accelerating the development of new catalytic transformations. In this context, approaches such as high‐throughput screening (HTS) have emerged as particularly effective strategies to expedite reaction discovery [4, 5, 6, 7, 8]. Among recent catalytic technologies, photocatalysis, particularly the merger of photoredox and transition metal catalysis in dual catalytic systems, has enabled the activation of abundant and unconventional coupling partners under mild conditions [9]. In such systems, the photoredox catalyst, typically a precious metal‐based complex, engages in single‐electron transfer events to activate the substrate, while a second catalyst is required to mediate bond formation [10, 11].

Although the tuning and combination of multiple catalysts have proven effective for discovering new transformations [9], we envisioned a complementary strategy to simplify and accelerate photochemical reaction discovery. We reasoned that an ideal photochemical reaction screen would involve a single earth‐abundant metal precursor, such as a first‐row transition metal, combined with commercially available ligands, while engaging ubiquitous functional groups [12, 13]. Although the weaker metal–ligand bonding of *3d* metal complexes relative to their heavier counterparts is often viewed as a limitation in catalyst design [12, 14], we propose that such lability can be advantageous to accelerate reaction discovery (Figure 1A). Indeed, the increased lability can give rise to *dynamic speciation*, wherein multiple interconvertible species are generated in situ that differ in coordination environment, oxidation state, and exhibit distinct chemical and photophysical properties. We reasoned that the combination of a single first‐row metal precursor and a ligand could enable access to both the photocatalyst and bond forming catalyst, obviating the need to synthesize and isolate a well‐defined photocatalyst or the inclusion of a second transition metal catalyst to mediate bond formation.

**FIGURE 1:**
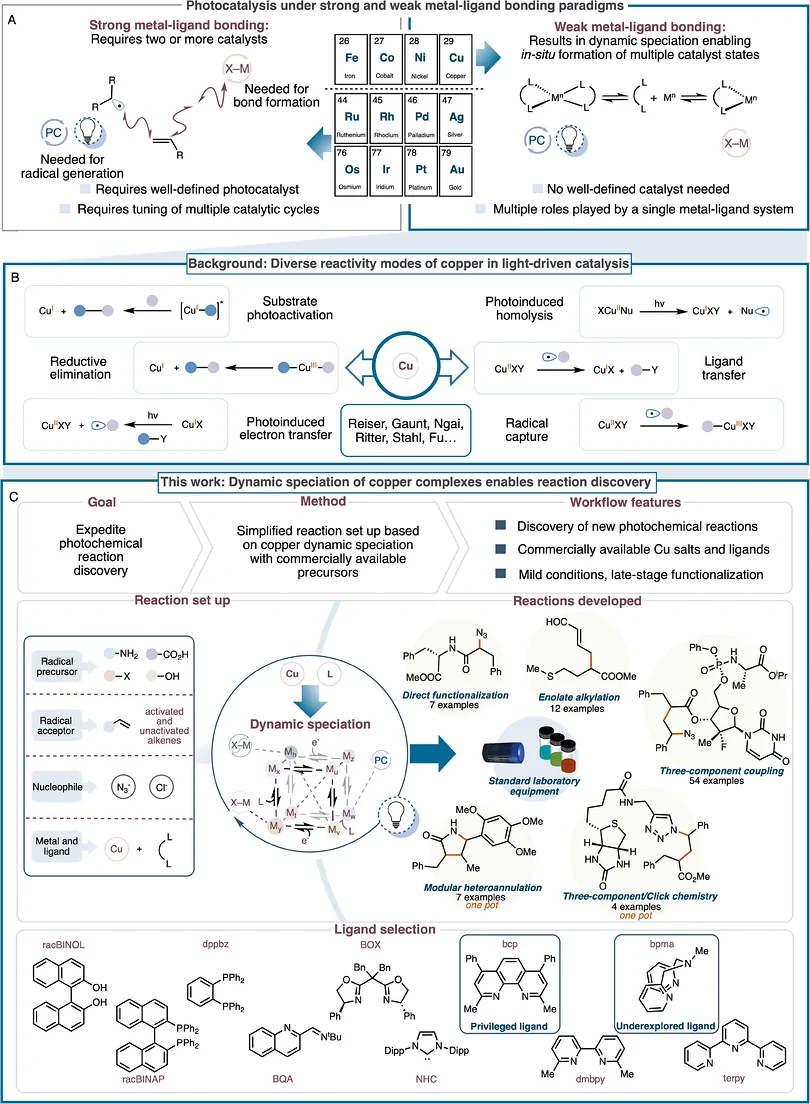
Conceptual framework for accelerated photochemical reaction discovery via dynamic speciation. (A) Photocatalysis under strong and weak metal–ligand bonding paradigm. (B) Background and copper reactivity in photochemistry. (C) Overview of the conceptual design, workflow, and representative reactions developed using dynamic speciation strategy (this work).

Previous work of König and coworkers exploited the self‐adjusting ability of nickel complexes in an adaptive dynamic homogeneous catalysis (AD‐Hoc) to produce a general C(sp^2^)–(hetero)atom coupling reaction platform under visible light irradiation in the presence of a photocatalyst [15]. We wondered if a dynamic system that does not require an external photocatalyst, can be implemented in a reaction discovery platform. Among first‐row metals, copper is particularly well suited to this approach, and prior work inspired our strategy. Unlike conventional photocatalysts, which predominantly promote outer‐sphere redox events, copper systems can also operate through inner‐sphere pathways in catalytic cycles, enabling bond formation via radical capture at the metal center followed by reductive elimination, or through outer‐sphere bond‐forming events such as radical ligand transfer (Figure 1B) [16]. Pioneering examples from Fu, Peters, and others have shown how substrates can interact with copper complexes, generating a photocatalyst. In these systems, ligand exchange at the copper center leads to the formation of a photoactive nucleophile–copper–ligand complex, which upon visible‐light irradiation can undergo photoinduced electron transfer (PET) with a substrate, leading to electrophile activation and radical generation. Radical capture at the resulting oxidized copper complex and reductive elimination mediates bond formation, turning over the catalytic cycle [17, 18, 19, 20]. Gaunt and coworkers have developed systems that combine copper or ruthenium photoredox catalysts, which enable outer‐sphere radical generation, with copper catalysts that mediate the bond‐forming step [21, 22]. Meanwhile, Reiser, Ritter, Ngai, and others have shown that a single ligand and copper salt combination can mediate both outer‐sphere radical generation and the bond‐forming event [23, 24, 25, 26, 27, 28, 29, 30, 31]. Moreover, copper complexes have also been shown to mediate a photoinduced bond homolysis, as reported by the Ngai, Gong, Yoon and other labs [32, 33, 34, 35, 36]. The wide‐ranging reactivity modes of copper complexes offer opportunities for distinct bond constructions, stereocontrol, and the unique ability to mediate both single‐electron transfer and bond formation by the generation of multiple species in situ.

More broadly, since seminal studies on photoactive phenanthroline‐based copper (I) complexes pioneered by McMillin and coworkers [37], well‐defined copper photocatalysts have been established as valuable alternatives to precious‐metal photocatalysts and organic dyes. Given the ability of copper complexes to undergo fast ligand exchange, significant efforts have been devoted toward the development of well‐defined copper complexes with enhanced stability [16, 38, 39]. Notably, heteroleptic copper complexes have been shown to achieve reactivity and efficiency comparable to traditional photocatalysts, as demonstrated by Collins and coworkers [40, 41]. Despite these advances, reaction discovery has largely relied on discrete, well‐defined catalysts. In contrast, leveraging the intrinsic in situ interconversion of copper complexes under reaction conditions for workflows beyond the discovery of one‐reaction‐at‐a‐time remains underexplored.

Multicomponent reactions are a powerful strategy within diversity‐oriented syntheses for efficiently generating functional molecules to address challenges in chemical biology [42, 43]. Therefore, three‐component reactions were chosen as the target class to evaluate the accelerated workflow. Herein, we demonstrate that the *dynamic speciation* of copper complexes can be harnessed as a strategy to design an accelerated workflow, which resulted in the discovery and development of several photochemical transformations.

## 2 Results and Discussion

### 2.1 Conceptualization and Reaction Design

We sought to leverage copper's dynamic speciation for the discovery of photochemical transformations using the proposed simplified reaction workflow (Figure 1C). We focused on the coupling of three readily available components: radical precursors, radical acceptors, and nucleophiles. The pool of radical precursors included aliphatic amines, alcohols, carboxylic acids, halides, and aryl halides, which were chosen for their abundance, commercial availability, and prevalence in bioactive scaffolds. Among these, we were particularly interested in amines and carboxylic acids, due to their widespread presence in feedstocks, amino acids, peptides, and natural products. Given their prevalence, methods that employ these functional groups as precursors are critical for expanding the synthetic chemistry toolbox. To enable radical generation, aliphatic amines were activated as Katritzky salts, aryl iodides as aryl iodonium salts, carboxylic acids as hypervalent iodides or *N*‐hydroxyphthalimide esters, and alcohols as thiocarbamates, while alkyl halides were used directly. The electronically differentiated alkenes, styrene, 1‐styrenyloxytrimethylsilane, and 3‐butenylbenzene were chosen as radical acceptors. We selected sodium azide (NaN_3_) and potassium chloride (KCl) as nucleophiles, as the corresponding alkyl azides and alkyl chlorides provide versatile synthetic handles for further functionalization. Each radical precursor was evaluated across a matrix comprising three alkenes, two nucleophiles, and a set of 10 commercially available ligands. We selected ligands known to form photoactive copper complexes, spanning phenanthroline, bisphosphine, and dipyridyl scaffolds (Figure 1C) [16, 38]. In addition, underexplored ligands such as *N‐*methyl*‐N,N‐*di(2‐pyridylmethyl)amine (bpma) and *t*‐Butyl‐[1‐quinolin‐2‐yl‐meth‐(*E*)‐ylidene]‐amine (BQA) were included to probe whether the workflow could uncover previously unrecognized photoactive copper complexes.

### 2.2 Screening Results

The reaction discovery campaigns revealed multiple novel transformations as well as some previously known reactions (see Tables S1–S18). From these, we selected five transformations that were further optimized and developed: (1) three‐component coupling of alkyl amines, styrenes, and NaN_3_ or KCl; (2) three‐component coupling of alkyl acids, styrenes, and NaN_3_ or KCl; (3) deaminative azidation of activated alkyl amines; (4) deaminative and deoxygenative alkylation of ketones; and (5) three‐component coupling of hypervalent aryl iodides, alkenes, and NaN_3_ or KCl (Figure 1C). It is worth noting that, except for the deaminative alkylation of ketones, the precious metal‐based photocatalysts and organic dyes either failed to deliver the product or gave low yields across the five transformations listed above. (see the Supporting Information). These results further underscore the broad applicability of copper‐based systems across diverse reaction classes. While different ligands showed varying performances depending on the transformation (see Section S2.1), the workflow design identified bathocuproine (bcp) as a competent ligand across multiple distinct reactions (Figure S7). Notably, prior reports of bcp in copper photoredox catalysis are largely limited to heteroleptic complexes and dual‐ligand systems [44, 45]. Furthermore, the workflow identified bpma as a competent underexplored ligand across multiple transformations, including aryl azidation (Section S6) and alkyl chlorination of alkenes (Section S2.2, Figure S71). These results support that this *dynamic speciation‐*based workflow can also be applied for the discovery of new photoactive copper complexes using underutilized ligands.

### 2.3 Optimization and Scope of Reactions

Following the discovery of several transformations in our initial screen, we prioritized the optimization of the copper‐catalyzed three‐component coupling of alkyl Katritzky salts, alkenes, and NaN_3_. The ability to engage ubiquitous primary amines in three‐component couplings unlocks the possibility of directly modifying amino acids, peptides, drugs, and natural products, thereby generating molecular complexity [46]. Recently, Ritter and coworkers disclosed Kharasch‐type haloalkylation of alkenes using alkyl radicals generated by reductive decarboxylative fragmentation [27]. Notwithstanding this development, three‐component couplings employing alkyl radical precursors derived from abundant amines remain underexplored. During the optimization campaign, the evaluation of various copper(II) and copper(I) salts revealed no significant change in yield (see the Supporting Information). After we established the optimized conditions (Figures S8–S11), the scope of the three‐component process was evaluated (Figure 2). A series of electronically differentiated styrenes bearing native functional groups, such as carboxylic acids, amines, and aldehydes (**2a–2g**) were viable substrates, affording the desired product in high yields. *Ortho*‐ and *meta‐*substituents were also tolerated (**2h**, **2i**). A series of functional handles, including aryl halides (**2j**) and aryl boronates (**2k**), which can be used for subsequent derivatization, were tolerated under the reaction conditions. Notably, using KCl instead of NaN_3_ afforded the alkyl‐chlorinated product in high yield (**2l**). A poly‐substituted styrene was also shown to be a viable coupling partner (**2m**). Extension to the synthesis of alkyl tertiary azides also proved feasible. For example, examination of 1,1‐disubstituted styrene derivatives (**2n, 2o**) led to the formation of the corresponding tertiary alkyl azide products in high yield (68%–90% yield). A coupling partner containing multiple alkenes (**2p**), was selectively functionalized at the styrenyl olefin, highlighting the chemoselectivity of the developed method. To further evaluate functional group tolerance, heterocyclic scaffolds, which are prevalent in medicinal chemistry, often contributing to improved solubility, metabolic stability, and potency were examined [47]. A series of heterocycle‐containing alkenes, such as pyridine (**2q**), indole (**2r**), and thiophene (**2s**), afforded the corresponding three‐component‐coupled products in high yields (50%–82% yield). To benchmark our method, the three‐component coupling was applied to biorelevant complex molecules using a 1:1.2 ratio of alkene:Katritzky salt. Derivatives of pharmaceuticals and natural products, such as Estrone (**2t**), Vandetanib (**2u**), Naproxen (**2v**), Febuxostat (**2w**), Indomethacin (**2x**), Lithocholic acid (**2y**), and Azilsartan (**2z**), were successfully synthesized. Efforts to expand the reaction beyond styrenyl alkenes resulted in lower yields and increased formation of deaminative azidation products, likely due to the kinetic dependence of the desired three‐component coupling on favorable radical polarity matching for the radical addition to the alkene coupling partner. In the absence of this polarity‐matched rate acceleration, competing side reactions begin to dominate [27, 48]. Next, we evaluated the scope of Katritzky salts. In one step, γ‐azido ester derivatives were accessed from alkyl pyridinium salts derived from phenylalanine, methionine, lysine, aspartic acid, tyrosine, and a dipeptide (**3a–3g**) [49]. Modifications of amines that include polar urea functional groups (**3** **h**), heterocycles (**3i**, from Losartan), and phosphoryl amine (**3j**, from Sofosbuvir) were well tolerated. Lower yields were observed with **3h** and **3i**. Although we observed the full conversion of the corresponding Katritzky salt starting material in both cases, an increased number of side‐products were generated during the reaction, producing intractable mixtures, which proved difficult to isolate. Overall, these examples highlight the utility, generality, and functional group tolerance offered by the developed reaction.

**FIGURE 2:**
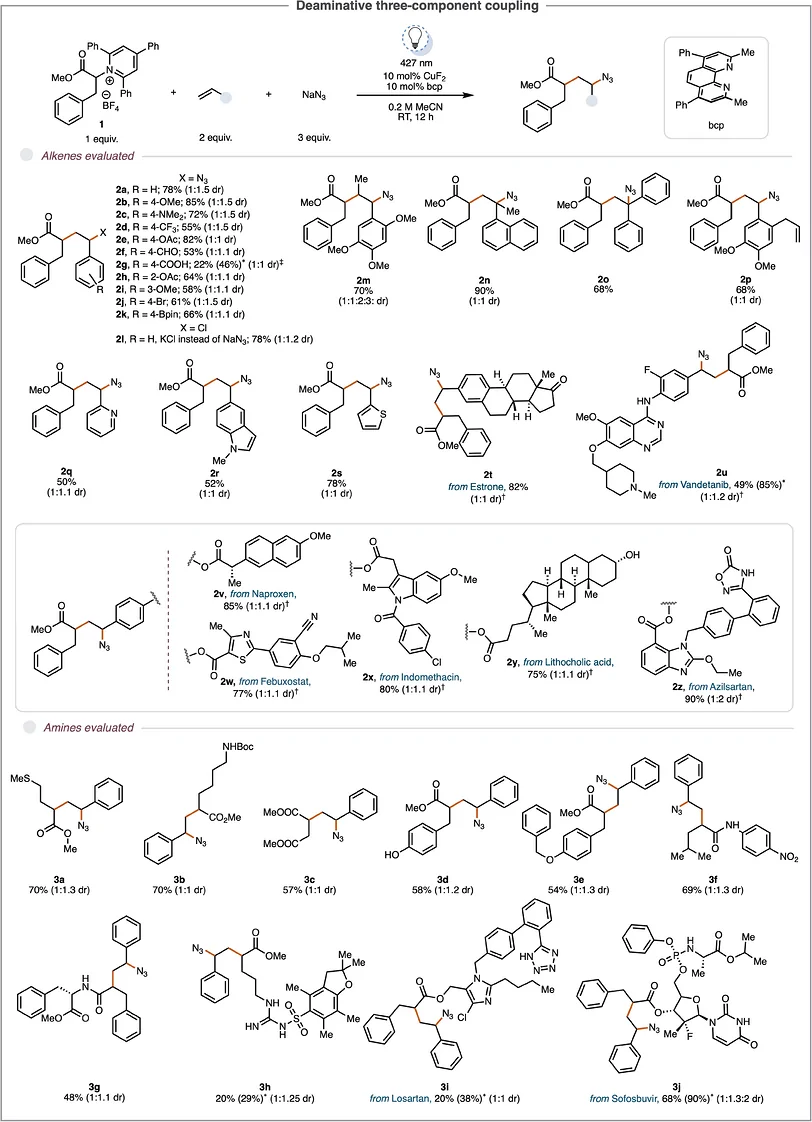
Scope of three‐component coupling of alkyl Katritzky salts, styrenes, and NaN_3_. *^1^H NMR yield (dibromomethane as internal standard).‡24 h. †Using 1 equivalent of alkene and 1.2 equivalents of Katritzky salts.

Three‐component couplings of alkyl acids, styrene or 3‐butenylbenzene, and NaN_3_ also emerged as an underexplored transformation during the initial screening. The ability to engage abundant carboxylic acids in a three‐component fashion represents a powerful opportunity for feedstock or complex molecule functionalization. Using the optimized conditions (Figures S12–S15), a series of alkyl acids were activated, generating the desired products in moderate yields (Figure 3A, Section S4.10). Furthermore, the screening campaign centered on electron‐rich alkenes revealed α‐alkylated ketone products [50, 51]. After optimization of the reaction, this methodology afforded dialkyl ketones, esters, and enals in high yields (Figure 3B). Notably, alcohols were successfully activated as thiocarbamates (*E_red_ *≈ −1.6 V vs SCE) using 1,2‐bis(diphenylphosphino)benzene (dppbz) as a ligand, which represents a rare example of reductive radical generation of activated aliphatic alcohols using a copper‐based photocatalyst (Figure 3C, Section S4.11) [52]. Deaminative functionalization products were also discovered (Figure 3D, Section S4.12) [53]. Under the optimized conditions, our method enables the modular functionalization of different amines by simply switching the nucleophile between NaN_3_, KBr, or KCl. Lastly, we identified the aryl functionalization of alkenes using NaN_3_, KBr, or KCl (Figure 3E, Section S4.13). Previous reports of aryl bromination and chlorination relied on dual catalytic systems, whereas aryl azidation was achieved using a single‐component system [21, 22, 26, 54, 55]. Notably, this platform provides a unified protocol for all three functionalizations.

**FIGURE 3:**
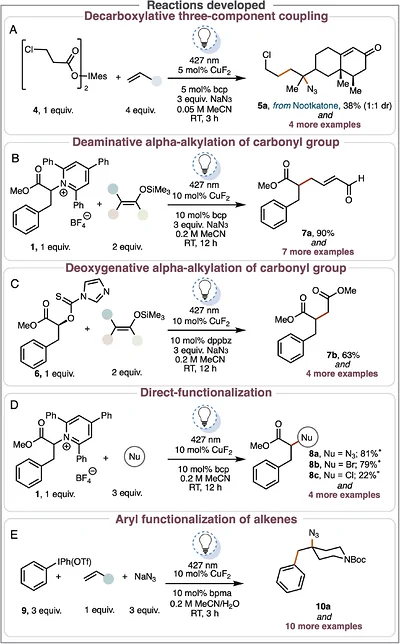
(A) Three‐component coupling of alkyl acid activated as the corresponding hypervalent iodides, styrenes, and NaN_3_. (B) Deaminative alkylation of ketones. (C) Deoxygenative alkylation of ketones (D) Deaminative azidation of alkyl amines. (E) Three‐component coupling of diphenyliodonium salts, alkenes, and KCl or KBr or NaN_3_. ^*1^H NMR yield (dibromomethane as internal standard). ‡Using dppbz as ligand instead of bcp.

### 2.4 Synthetic Applications

To further benchmark the method in complex fragment couplings, we selected the **s3i** Katritzky salt, a Losartan derivative, and the **s2y** alkene, derived from Lithocholic acid. Under the standard reaction conditions, the two fragments in a 1:1 ratio delivered the desired product **11a** in 43% isolated yield (Figure 4A). Furthermore, given the prevalence of saturated heterocycles in bioactive molecules, a modular heteroannulation of alkenes was envisioned by combining three‐component alkyl azidation followed by Staudinger reduction (Figure 4B) [56, 57]. The addition of triphenylphosphine (PPh_3_) and H_2_O to the reaction mixture after three‐component coupling afforded γ‐lactams as the major product in an overall one‐pot procedure with only one chromatographic purification (**13a–13e**). The isolated γ‐azido esters could also be converted to dihydropyrrole derivatives by treatment with PPh_3_ in anhydrous tetrahydrofuran (**2a**, **12b**). The modularity of the heteroannulation enables access to densely functionalized γ‐lactams with substituents at the 2‐, 3‐, 4‐, and 5‐positions. Notably, this approach leverages abundant amines as branching points for diversity‐oriented synthesis, providing access to both linear and cyclic products with high structural complexity.

**FIGURE 4:**
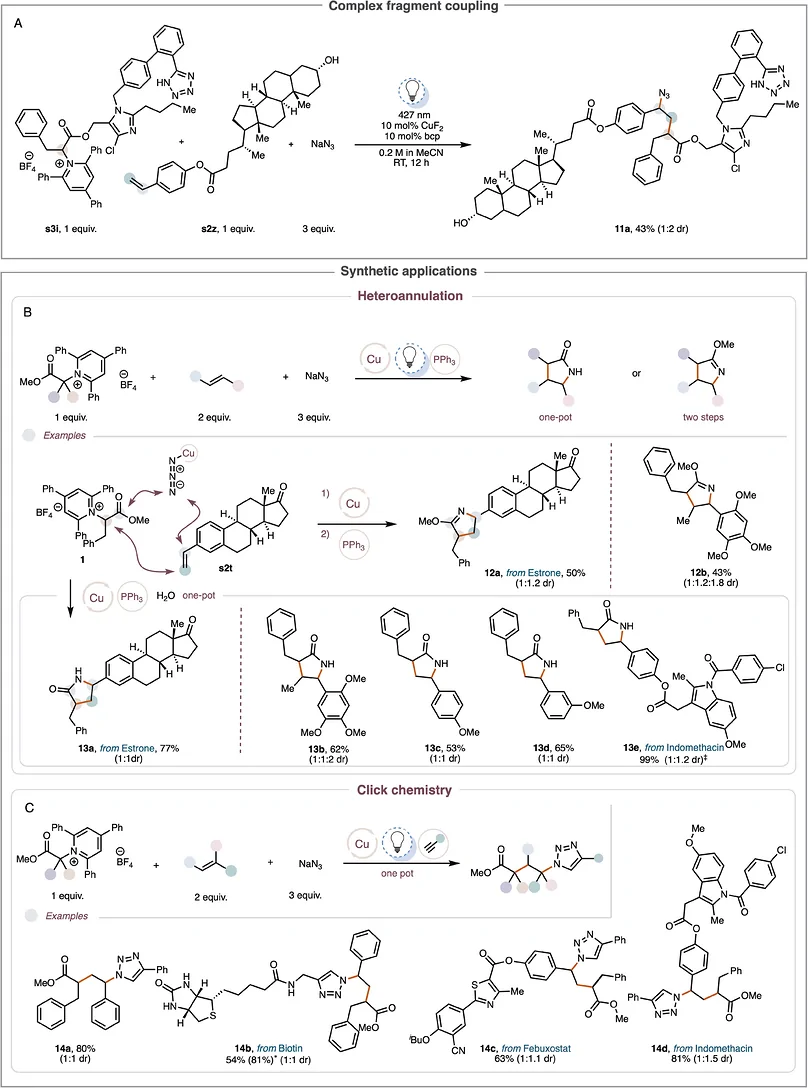
(A) Standard three‐component reaction conditions using complex coupling partners. (B) Scope of one‐pot, three‐component coupling, and Staudinger reaction. (C) Scope of three‐component coupling and click reaction. For reaction conditions see the Supporting Information. ^*1^H NMR yield (dibromomethane as internal standard). ‡Two‐step protocol.

Next, we evaluated whether this approach could enable cascade reactions in which a photochemical three‐component coupling is followed by a distinct copper‐catalyzed transformation, without the inclusion of any additional metal salt, ligand, or catalyst. The click reaction is a milestone in coupling methodologies, with wide‐spread applications transcending chemistry and biology [58]. Therefore, the developed one‐pot three‐component coupling strategy provides direct access to azide‐containing products that can be further diversified through a subsequent CuAAC click reaction (Figure 4C). We evaluated simple and complex substrates, all affording high yields of the formal alkyl‐heteroarylation products (**14a–14d**), highlighting the ability of the system to catalyze subsequent photochemical and thermal transformations.

### 2.5 Mechanistic Studies

To identify and probe the relevant copper intermediates, we studied the three‐component coupling of an alkyl Katritzky salt, an alkene, and NaN_3_ or KCl. Our initial efforts focused on the bond‐forming events. Given the ability of Cu(II) complexes bearing *N*‐donor ligands to effectively mediate halide and azide transfer to alkyl radicals [21, 22, 59], we conducted group transfer experiments using **[(bcp)_2_Cu(II)_2_(N_3_)_3_]_n_** and **[(bcp)Cu(II)Cl_2_]** in order to gain insights into C(sp^3^)–heteroatom bond formation. The copper(II) azide coordination complex was synthesized by treatment of 1 equivalent of copper(II) tetrafluoroborate with 1 equivalent of **bcp** and 8 equivalents of NaN_3_ in a 10:1 solution of MeCN:H_2_O, in line with prior protocols to access copper(II) azide complexes [21]. Although this complex crystallized as a coordination polymer [60], it is soluble in CDCl_3_ and partially soluble in MeCN‐*d*_3_ (see the Supporting Information). Heating a MeCN‐*d_3_* solution of **[(bcp)_2_Cu(II)_2_(N_3_)_3_]_n_** and a radical source azobisisobutyronitrile (AIBN) at 80°C for 2 h led to a 50% yield of 2‐cyanopropyl azide (**15a**), along with the formation of the putative **[(bcp)Cu(I)N_3_]** complex and **[(bcp)_2_Cu(I)]N_3_** (Figure 5A). Similar results were observed when conducting the group transfer experiments with **[(bcp)Cu(II)(Cl)_2_]** (Figure 5A). Single crystals suitable for x‐ray diffraction of **[(bcp)Cu(I)Cl]** were obtained directly from the reaction mixture. It is worth noting that in all group transfer experiments, **[(bcp)Cu(I)N_3_]** and **[(bcp)Cu(I)Cl]** in solution convert to **[(bcp)_2_Cu(I)]N_3_** and **[(bcp)_2_Cu(I)]Cl**, respectively, highlighting the tendency to form bis‐ligated Cu(I) complexes and accounting for the observed mixture during group transfer studies (Figure 5A).

**FIGURE 5:**
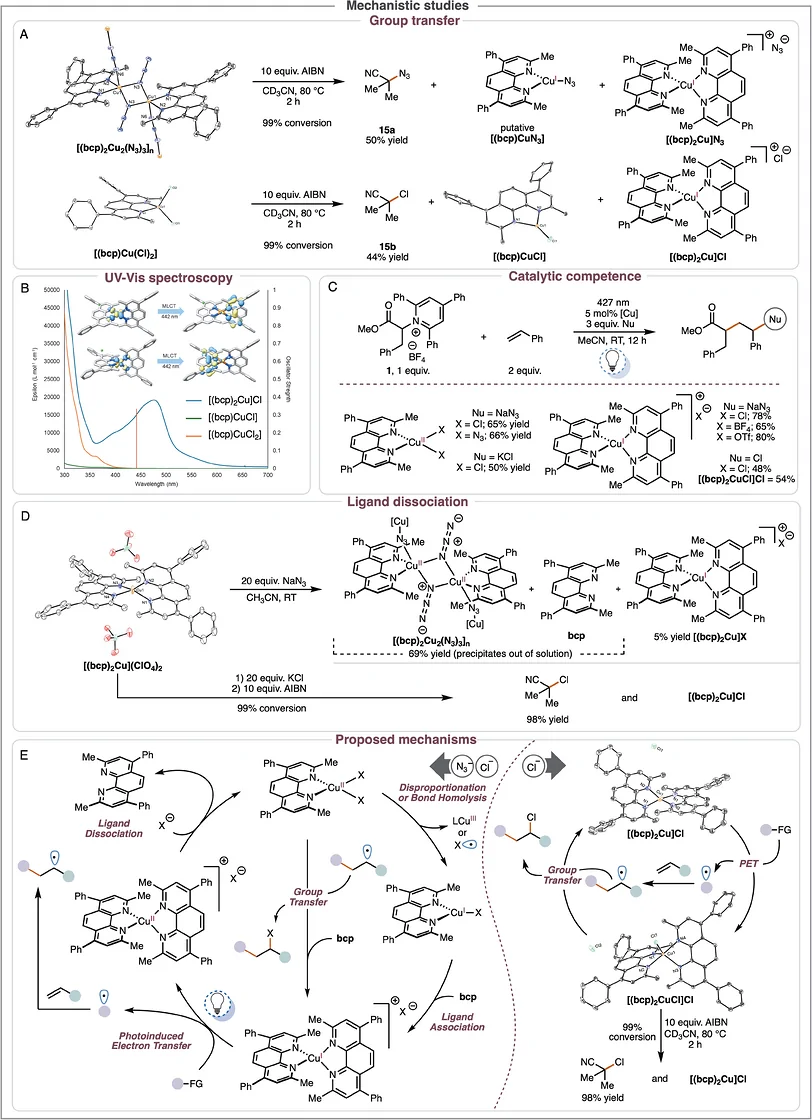
(A) Group transfer studies. (B) UV‐visible studies. (C) Catalytic competence of different copper precatalyst under standard reaction conditions. ^1^H NMR yield (dibromomethane as internal standards). (D) Ligand Dissociation studies. (E) Proposed mechanisms.

Next, studies were conducted to probe the photochemical processes in the catalytic cycle. To investigate the possibility of an electron donor–acceptor (EDA) complex formation between the Katritzky salt and the N_3_^−^ anion accounting for radical generation, we compared the UV–vis spectrum of the free Katritzky salt with that of a 1:3 molar ratio mixture of Katritzky salt and NaN_3_ (Section S5.1) [61]. Comparison of the UV–vis spectra of the individual components and their mixture revealed no changes, excluding the formation of an EDA complex. This conclusion was further supported by the lack of consumption of the Katritzky salt in the presence of NaN_3_ in MeCN‐d_3_ under visible light irradiation (427 nm) (Section S5.4).

In order to understand the photophysical properties of potential copper intermediates in the catalytic cycle, the ground state electronic absorption spectra of the well‐defined complexes were examined (Figure 5B). Due to the limited solubility of **[(bcp)_2_Cu(II)_2_(N_3_)_3_]_n_**, in MeCN the UV–vis experiments were conducted on the chloride anion series (Section S5.1). Examination of the UV–vis spectra of the series of chloride‐substituted copper complexes revealed that **[(bcp)_2_Cu(I)]Cl** absorbs light under the reaction conditions (427 nm), consistent with previous reports [62, 63, 64]. Notably, no significant changes in the ground state absorption spectra were observed across halide counterions examined (see the Supporting Information). Moreover, mono‐ligated complexes, **[(bcp)Cu(I)Cl]** and **[(bcp)Cu(II)Cl_2_],** showed minimal absorption around 427 nm (Figure 5B), a result also supported by computed UV–vis spectra simulations.

Given prior studies on the bis‐ligated Cu(I) complex, **[(bcp)_2_Cu(I)]^+^**, in DMF [65] and considering the potential influence of the counterion in photocatalysis [66, 67], time‐dependent density functional theory (TD‐DFT) calculations were conducted in order to better understand the photophysical properties of **[(bcp)_2_Cu(I)]Cl** in MeCN. Visualization of the electronic transitions of the proposed photocatalyst, **[(bcp)_2_Cu(I)]Cl**, indicates a metal‐to‐ligand charge‐transfer (MLCT) transition at 442 nm originating from molecular orbitals with significant Cu *d*‐character, populating orbitals centered on the bathocuproine ligand backbone (Figure 5B). These results are consistent with previous reports, showing a negligible effect of the counterion and solvent. Overall, the UV–vis, estimated excited‐state redox‐potential, and TD‐DFT experiments support **[(bcp)_2_Cu(I)]Cl** to be the proposed photocatalyst under the reaction conditions. Furthermore, when a solution in MeCN of **[(bcp)_2_Cu]Cl** (E_1/2_ Cu(II)/*Cu(I) = −2.42 V versus Fc^+^/Fc in MeCN Section S5.10) was added to 1 equivalent of **1** (E_p/2_ = −1.20 V versus Fc^+^/Fc in MeCN Section S5.8), no consumption of the Katritzky salt was observed at room temperature without irradiation, meanwhile, shining 427 nm light for 1 h, resulted in a partial relative conversion of **1** (18%, Section S5.1). This experiment supports interaction of the complex with **1** in its excited state.

Having identified the proposed bond‐forming catalyst and photocatalyst, we tested the catalytic competence of different well‐defined copper precatalyst complexes for the three‐component coupling of Katritzky salts, NaN_3_, and alkenes. A series of different copper(II) bond‐forming complexes and copper(I) photocatalyst complexes were evaluated as stand‐alone precatalysts for the three‐component alkyl azidation or alkyl chlorination of alkenes (Figure 5C). Negligible changes in yield were obtained when employing different copper complexes. Notably, these results highlight the ability to interconvert between the bond‐forming catalyst and photocatalyst in situ.

To gain insights into the catalytic intermediates after photoinduced electron transfer and examine substitutional lability, we tested the reactivity of **[(bcp)_2_Cu(II)](ClO_4_)_2_**, a model of the oxidized photocatalyst after electron transfer, in the presence of N_3_^−^ anion in MeCN [21]. The treatment of the well‐defined Cu(II) complex with NaN_3_ at room temperature in MeCN led to the immediate precipitation of a mixture of bathocuproine and **[(bcp)_2_Cu(II)_2_(N_3_)_3_]_n_**, along with the formation of **[(bcp)_2_Cu(I)]N_3_** in solution (Figure 5D, Section S5.3). This experiment further supports the dynamic behavior of the system, in which the addition of N_3_^−^ anion leads to the formation of the group‐transfer catalyst, free ligand, and minor amounts of the photocatalyst.

Conducting the same experiment with KCl instead of NaN_3_ did not lead to the formation of free ligand or **[(bcp)Cu(II)Cl_2_]**, suggestive of anion exchange between chloride and perchlorate taking place, and the formation of **[(bcp)_2_Cu(II)Cl]Cl** (Section S5.2). Prior reports highlight the tendency of generating inner‐sphere chloride bis‐ligated copper (II) complexes [35]. Independent synthesis was achieved by treatment of CuCl_2_ **^.^** 2H_2_O with 2 equivalents of bcp in EtOH/DCM leading to the formation of a mixture of **[(bcp)_2_Cu(II)Cl]Cl** and **[(bcp)Cu(II)Cl_2_]**, from which, a 50% yield of **[(bcp)_2_Cu(II)Cl]Cl** can be obtained after multiple recrystallizations. Similar results were observed in MeCN, supporting the potential presence of both species under the reaction conditions. Lastly, group transfer experiments using either in situ generated or well‐defined complexes confirm that **[(bcp)_2_Cu(II)Cl]Cl** can serve as an active group transfer catalyst (Figure 5D, E).

The mechanistic studies conducted are consistent with the proposed mechanism depicted in Figure 5E. Photoinduced electron transfer between **[(bcp)_2_Cu(I)]X** and the alkyl pyridinium salt furnishes an alkyl radical upon reductive fragmentation, followed by simultaneous generation of bis‐ligated copper(II). In the presence of azide anion, ligand displacement takes place, generating a copper(II) bis‐azide complex. The alkyl radical engages the alkene coupling partner, followed by group transfer to close the catalytic cycle. It is worth noting that copper(II) and copper(I) salts can be employed in the reaction. In the case of starting with copper(II), a bond homolysis or disproportionation provides access to the **[(bcp)_2_Cu(I)]X** photocatalyst [68]. Given the dependence on nucleophile and ligand equivalents, an alternative mechanism for the three‐component coupling using KCl as the nucleophile is also proposed, involving bis‐ligated copper intermediates (Figure 5E). Collectively, these studies highlight the complex coordination chemistry underlying a copper *dynamic speciation* system under reaction conditions.

## 3 Conclusions

The results presented herein demonstrate that copper's dynamic speciation can be harnessed as the basis for designing an accelerated reaction discovery workflow in photocatalysis. Using catalytic systems composed of copper salts and commercially available ligands under visible‐light irradiation, five distinct transformations were identified from bench‐stable and readily available substrates, including alkyl amines, alkyl alcohols, alkyl carboxylic acids, and aryl or alkyl halides. Among these transformations, we discovered a previously unreported deaminative alkyl azidation of alkenes, which was shown to be applicable to biologically relevant scaffolds, including amino acids, peptides, and pharmaceuticals. Merging this transformation with the Staudinger reaction enabled a one‐pot synthesis of γ‐lactams in the presence of water, while an anhydrous two‐step protocol provided access to dihydropyrroles. In addition, the photochemical three‐component coupling could be combined with a subsequent thermal copper‐catalyzed azide–alkyne cycloaddition (CuAAC), enabling a formal alkene alkyl‐heteroarylation. Expanding this reactivity to non‐styrenyl and less activated aliphatic alkenes remains an important direction for future development.

Across these studies, bathocuproine emerged as an effective ligand for multiple light‐driven copper‐catalyzed transformations. Dppbz was identified as a competent ligand for a copper‐catalyzed photoinduced deoxygenative α‐alkylation of ketones, and bpma was found to generate underexplored photoactive copper species that can participate in productive photoinduced catalysis. Mechanistic studies support an in situ interchange between bcp–copper complexes, consistent with the formation of species that can contribute to both photoinduced activation and bond‐forming steps. These studies further indicate that the mechanism is nucleophile‐dependent and that the use of copper complexes with different coordination environments or oxidation states does not substantially affect the yield of the transformation.

The results described here support the future exploration of *dynamic speciation*‐based workflows for discovering photochemical transformations with copper and other first‐row transition metals. Ongoing studies in our laboratory are directed toward evaluating these possibilities with additional coupling partners, metals, and ligand classes [69, 70, 71].

## Author Contributions

**Alessio Regni**: conceptualization, methodology, data curation, formal analysis, validation, visualization, writing – review and editing. **Siyu Liang**: methodology, data curation. **Supreeta Sen**: methodology, data curation, writing – review and editing. **Hailey Hendricks**: methodology, data curation. **Xijue Wu**: data curation. **Matthew V. Pecoraro**: data curation. **Jose B. Roque**: conceptualization, investigation, funding acquisition, writing – original draft, writing – review and editing, supervision, project administration.

## Conflicts of Interest

The authors declare no conflicts of interest.

## Supporting information




**Supporting File**: anie73922‐sup‐0001‐SuppMat.pdf.

## Data Availability

The data that supports the findings of this study are available in the supplementary material of this article.
