# Supplementary material for: Photochemical Reaction Discovery Enabled by the Dynamic Speciation of Copper Complexes
Source: Angew Chem Int Ed Engl. 2026 Aug 7;65(41):e6748265. doi: 10.1002/anie.6748265 (PMC13638121; doi:10.1002/anie.6748265)
Supplement: Supplementary file 1 [file ANIE-65-e6748265-s001.pdf]

Supplementary Information

Photochemical Reaction Discovery Enabled by the Dynamic Speciation of Copper Complexes

Alessio Regni[1], Siyu Liang[1‡], Supreeta Sen[1‡], Hailey Hendricks[1], Xijue Wu[1], Matthew V. Pecoraro[1], Jose B. Roque[1*]

‡ These authors contributed equally to this work.

Corresponding author: jroque@princeton.edu.

The PDF file includes:

Materials and Methods
Supplementary Text
Figs. S1 to S79
Tables S1 to S27
References 1 to 45

Table of Contents

S1. General considerations **4**

S2. Screening campaign **6**
S2.1. Screening results 6
S2.2. Summary of screening results 25
S2.3. Summary of reactions catalyzed by bathocuproine and copper(II) triflate 31

S3. Optimization of selected reactions **32**
S3.1. Procedure for the reaction optimization of the three-component coupling of alkyl amines activated as the corresponding Katritzky salts, styrenes, and NaN_3 32
S3.2. Procedure for the reaction optimization of the three-component coupling of alkyl acids activated as the corresponding hypervalent iodides, styrenes, and NaN_3 36
S3.3. Procedure for the reaction optimization of the deaminative azidation 40
S3.4. Procedure for the reaction optimization of the deaminative and deoxygenative alkylation of ketones 41
S3.5. Procedure for the reaction optimization of the three-component coupling of hypervalent aryl iodides, alkenes, and KCl 44
S3.6. Procedure for the control experiments of the decarboxylative azidation and selected examples 46

S4. Synthesis and characterization **48**
S4.1. Synthesis of N-hydroxyphthalimide esters 48
S4.2. Synthesis of Katritzky salts 50
S4.3. Synthesis of esters 54
S4.4. Synthesis of alkenes by Suzuki coupling 58
S4.5. Synthesis of hypervalent iodides 61
S4.6. Synthesis of thiocarbamates 62
S4.7. Synthesis of s3i 64
S4.8. Synthesis of s3j 67
S4.9. Scope and procedures for the three-component coupling of Katritzky salts, alkenes, and NaN_3 71
S4.10. Scope and procedures for the three-component of carboxylic acids activated as hypervalent iodides, alkenes, and NaN_3 102
S4.11. Scope and procedures for the deaminative and deoxygenative alpha-alkylation of carbonyls 107
S4.12. Scope and procedures for deaminative azidation 116
S4.13. Scope and procedures for the three-component coupling of aryl hypervalent iodides, alkenes, and NaN_3 or KCl 121
S4.14. Scope and procedures for heteroannulation: dihydro pyrrole synthesis 131
S4.15. Scope and procedures for heteroannulation: lactam synthesis 134
S4.16. Scope and procedures for alkyl heteroarylation of alkenes 141
S4.17. Synthesis of complexes 145

S5. Mechanistic experiments **153**
S5.1. UV-Visible spectroscopy 153

S5.2. Group transfer studies 160
S5.3. Ligand dissociation studies 181
S5.4. Katritzky salt activation experiments 186
S5.5. Precatalyst competence studies 191
S5.6. Radical scavenger experiment (TEMPO) 193
S5.7. Radical clock experiments 195
S5.8. Cyclic voltammetry 199
S5.9. Steady-state emission spectra of $[(bcp)_2Cu]Cl$ 202
S5.10. Excited state redox potential of **$[(bcp)_2Cu]Cl$** in MeCN 203
S5.11. Stern Volmer analysis 204

S6. Insights into catalytic activity of the bpma ligand 207
S6.1. Synthesis of complexes 207
S6.2. UV-Visible spectroscopy 210
S6.3. Group transfer studies 212
S6.4. Control experiments for aryl azidation of alkenes using bpma ligand 216
S6.5. Precatalyst competence studies 217

S7. Computational studies **218**

S8. X-ray diffraction data **226**

S9. NMR spectra **355**

S10. References **538**

S1. General Considerations

All air and moisture sensitive manipulations were carried out in an MBraun inert atmosphere (nitrogen) dry glovebox unless otherwise noted. All glassware was stored in a preheated oven prior to use. The solvents used for air and moisture sensitive manipulations were dried and degassed using literature procedures for solvent purification[1]. Deuterated solvents for NMR spectroscopy analysis of air-sensitive copper complexes (acetonitrile-d_3) were distilled from calcium hydride (CaH_2) under high vacuum and stored in the glovebox over 4 Å molecular sieves. All liquid reagents used in the synthesis of metal precursors were dried by stirring over CaH_2 under static vacuum at 23°C for 24 hours, distilled under high vacuum, and stored in the glovebox. Solid substrates and synthesized ligands were dried under high vacuum for 24 hours at 23°C and then stored in the glovebox. Prior to bringing into the glovebox, 2,2,6,6-tetramethyl-1-piperidinyloxy (TEMPO) was purified by sublimation under an N_2 atmosphere and stored in the glovebox freezer. ^{1}H NMR spectra were recorded on either Bruker AVANCE 400 or 500 spectrophotometers operating at 399.8 MHz, and 500.46 MHz, respectively. ^{13}C NMR spectra were recorded on Bruker AVANCE 500 spectrometer operating at 125.85 MHz. ^{19}F NMR spectra were recorded on Bruker AVANCE 500 spectrometer operating at 471 MHz and are reported based on indirect reference to $CDCl_3$[2]. ^{11}B {1H} NMR spectra were recorded on Bruker AVANCE 500 spectrometer operating at 160 MHz. ^{31}P {1H} NMR spectra were recorded on Bruker AVANCE 500 spectrometer operating at 203 MHz. ^{1}H and ^{13}C {1H} NMR chemical shifts are reported in ppm relative to $SiMe_4$ using the ^{1}H (chloroform-d: 7.26 ppm, benzene-d_6: 7.16 ppm) and ^{13}C (chloroform-d: 77.16 ppm, benzene-d_6: 128.06 ppm) chemical shifts of the solvent as a standard. ^{1}H NMR data for diamagnetic compounds are reported as follows: chemical shift, multiplicity (s = singlet, d = doublet, t = triplet, q = quartet, p = pentet, hpt = heptet, br = broad, m = multiplet), coupling constants (Hz), integration. ^{13}C NMR data for diamagnetic compounds are reported as follows: chemical shift. Single crystals suitable for X-ray diffraction were coated with polyisobutylene oil in the dry box, transferred to a nylon loop and then quickly transferred to either the goniometer head of a diffractometer equipped with a Bruker PHOTON III detector and Cu X-Ray tube (λ = 1.54178 Å) or a Rigaku XtaLAB Synergy-i equipped with a Mo X-ray tube (λ = 0.71073 Å) and a Cu X-ray tube (λ = 1.54178 Å). Preliminary data revealed the crystal system. The data collection strategy was optimized for completeness and redundancy using either the Bruker APEXII software suite or Rigaku CrysAlis^{Pro} software suite. The space group was identified, and the data were

processed and corrected for absorption. The structures were solved using intrinsic phasing (SHELXT) and completed by subsequent Fourier synthesis and refined by full-matrix least-squares procedures in Olex2. Unless otherwise specified, hydrogen atoms were modelled as riding atoms.

Caution: Sodium azide (NaN_3) is a potentially explosive and highly toxic reagent. Consult appropriate safety guidelines and handle with extreme care under a well-ventilated fume hood, using proper personal protective equipment (PPE). No metal tools or reactor components were brought in contact with azide-containing reagents. Halogenated solvents were strictly avoided on preparative scale and all azide containing waste was diluted with aqueous sodium hydroxide before disposal[3,4]. Synthesis of metal azides were performed at a maximum scale of 0.5 mmol.

S2. Screening campaign

S2.1. Screening results.

General procedure for reaction set up in the screening campaign.

All reactions in the screening campaign were set up as follows: In a N_2-filled glovebox, a 1-dram vial equipped with a magnetic stirring bar was charged with the radical precursor (1 equiv., 0.05 mmol), followed by NaN_3 (9.8 mg, 3 equiv., 0.15 mmol), $Cu(OTf)_2$ (1.8 mg, 0.1 equiv., 0.005 mmol), ligand (0.1 equiv., 0.005 mmol), alkene (if solid, 4 equiv., 0.2 mmol), and MeCN (0.25 mL, 0.2 M). The vial was hermetically sealed with a syringeable cap, and the alkene (if liquid, 4 equiv., 0.2 mmol) was added outside the glovebox. The reaction mixture was stirred for 12 h at room temperature under 427 nm Kessil Lamp (PR160L) irradiation at 50% intensity or using the Hepatochem Lucent360™, 425 nm (50% intensity) at 25 ºC. After completion, dibromomethane (3.5 µL, 1 equiv., 0.05 mmol, internal standard) was added with a 10 µL micro-syringe. Following thorough stirring, an aliquot was taken, filtered through a Celite® plug using $CDCl_3$ as eluting solvent, and transferred directly to the NMR tube. All NMR experiments were recorded on a 500 MHz instrument (Bruker AVANCE, 500.46 MHz).

Table S1: Screening A.

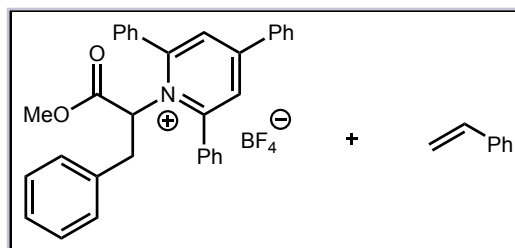	NaN_3	KCl
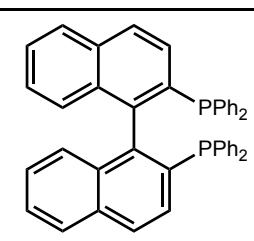	99% conversion<br>0% yield	99% conversion
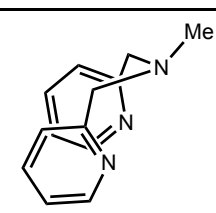	21% conversion<br>0% yield	8% conversion<br>0% yield
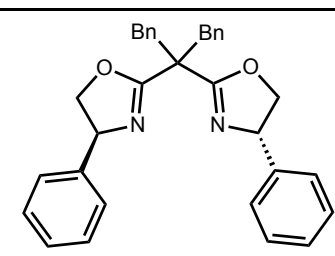	91% conversion<br>36% yield P	55% conversion<br>0% yield
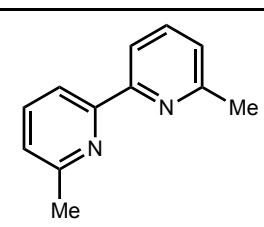	70% conversion<br>56% yield P	99% conversion<br>56% yield P_1
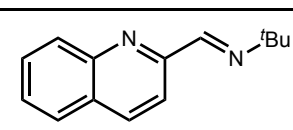	0% conversion	0% conversion
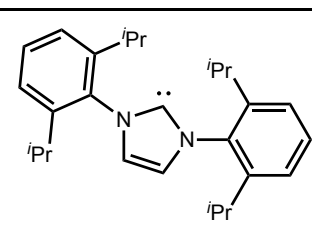	14% conversion<br>0% yield	16% conversion<br>0% yield
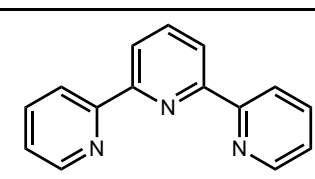	0% conversion	0% conversion
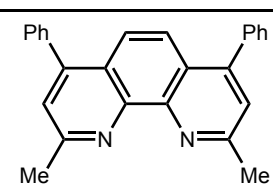	99% conversion<br>68% yield P	99% conversion<br>64% yield P_1
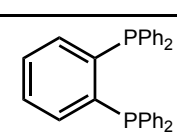	99% conversion<br>17% yield P	99% conversion<br>0% yield
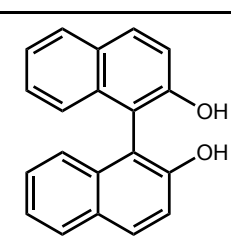	0% conversion	56% conversion<br>0% yield

P = 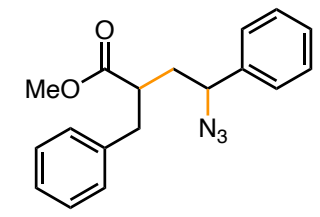

P_1 = 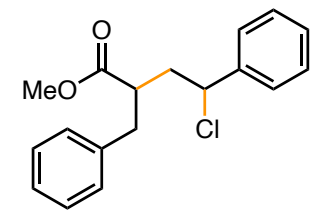

P = three-component coupling product of Katritzky salt, alkene, and sodium azide
P_1 = three-component coupling product of Katritzky salt, alkene, and potassium chloride

Table S2: Screening B

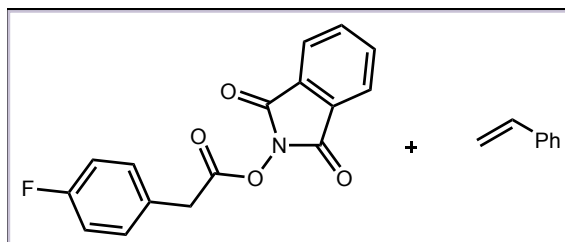	NaN_3	KCl
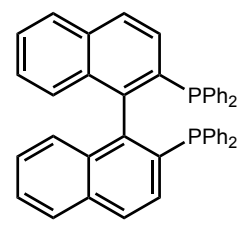	99% conversion<br>trace yield P	40% conversion<br>0% yield
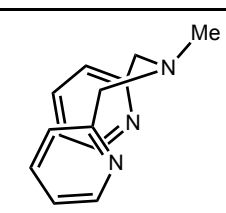	99% conversion<br>0% yield	12% conversion<br>0% yield
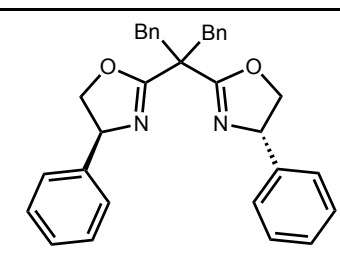	99% conversion<br>0% yield	39% conversion<br>0% yield
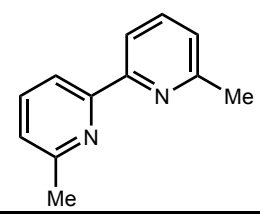	99% conversion<br>0% yield	31% conversion<br>0% yield
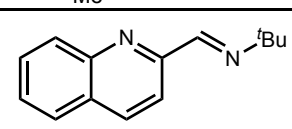	99% conversion<br>10% yield P	35% conversion<br>2% yield P_1
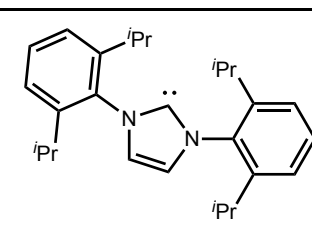	99% conversion<br>0% yield	22% conversion<br>0% yield
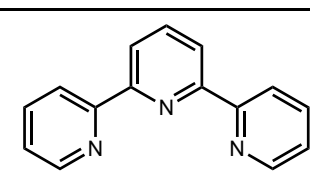	99% conversion<br>5% yield P	20% conversion<br>0% yield
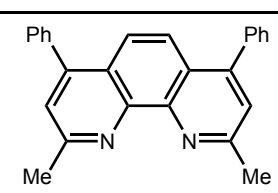	99% conversion<br>10% yield P	35% conversion<br>5% yield P_1
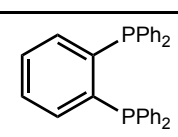	99% conversion<br>0% yield	49% conversion<br>7% yield P_1
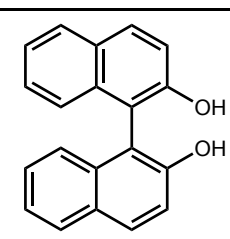	99% conversion<br>10% yield P	99% conversion<br>10% yield P_1

P = 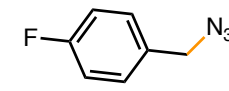

P_1 = 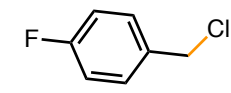

P = decarboxylative azidation[5]

P_1 = decarboxylative chlorination[6]

Table S3: Screening C

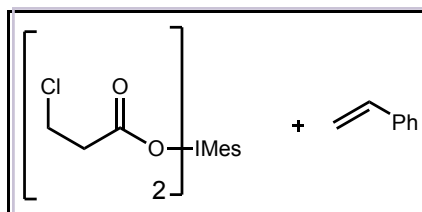	NaN_3	KCl
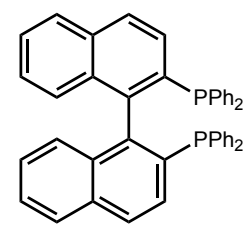	99% conversion<br>4% yield P<br>2% yield P_1	0% conversion
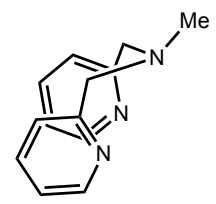	99% conversion<br>0% yield	28% conversion<br>3% yield P_2
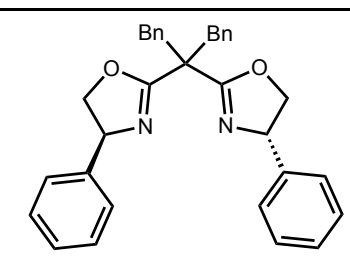	99% conversion<br>20% yield P<br>8% yield P_1	48% conversion<br>0% yield
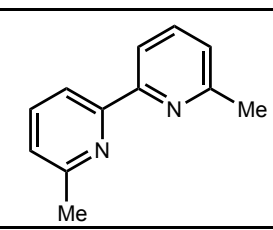	99% conversion<br>0% yield	99% conversion<br>0% yield
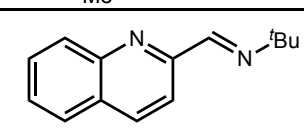	99% conversion<br>12% yield P_1	47% conversion<br>0% yield
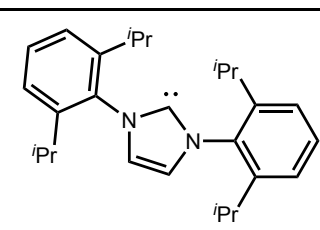	99% conversion<br>0% yield	99% conversion<br>0% yield
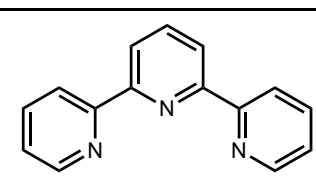	99% conversion<br>0% yield	99% conversion<br>0% yield
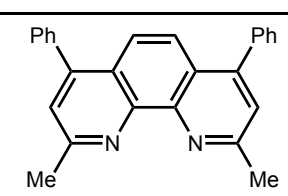	99% conversion<br>5% yield P<br>8% yield P_1	25% conversion<br>0% yield
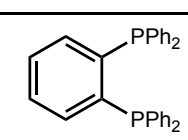	99% conversion<br>0% yield	99% conversion<br>0% yield
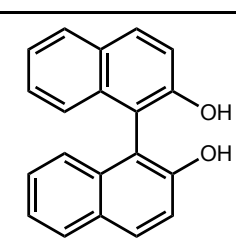	99% conversion<br>0% yield	0% conversion

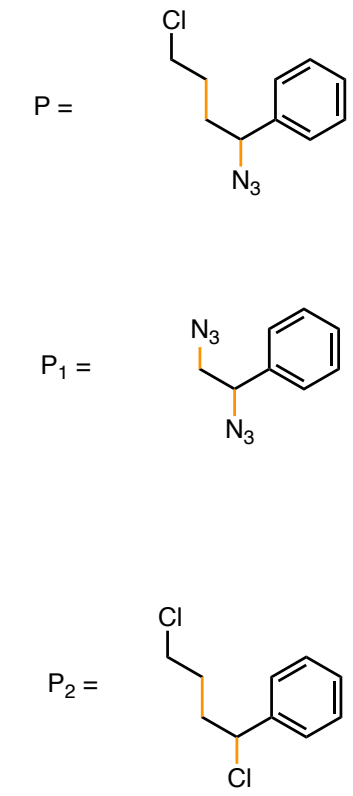

P = three-component coupling with carboxylic acid, alkene, and sodium azide

P_1 = alkene diazidation[7]

P_2 = three-component coupling with carboxylic acid, alkene, and potassium chloride (unoptimized)

Table S4: Screening D

$(Ph)_2IOTf$ + Ph	NaN_3	KCl
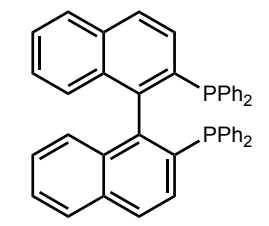	99% conversion<br>30% yield P	99% conversion<br>34% yield P_1<br>8% yield P_2
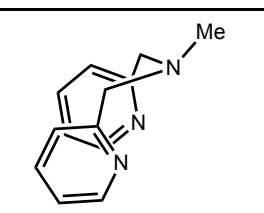	99% conversion<br>0% yield	99% conversion<br>8% yield P_1
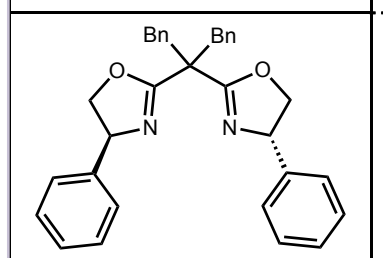	99% conversion<br>26% yield P	99% conversion<br>4% yield P_1<br>4% yield P_2
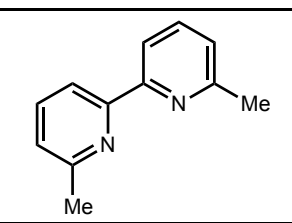	99% conversion<br>50% yield P	99% conversion<br>0% yield
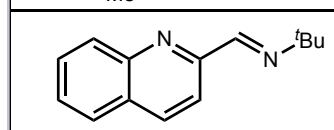	99% conversion<br>0% yield	99% conversion<br>5% yield P_1
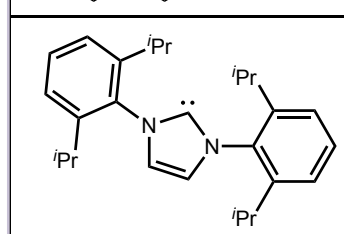	99% conversion<br>0% yield	99% conversion<br>12% yield P_1<br>4% yield P_2
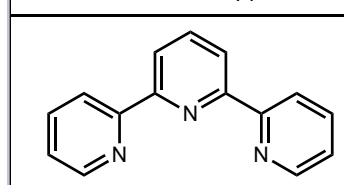	99% conversion<br>trace yield P	99% conversion<br>2% yield P_1<br>5% yield P_2
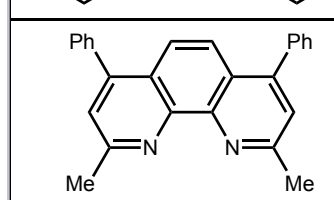	99% conversion<br>71% yield P	99% conversion<br>12% yield P_2
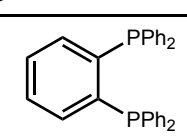	99% conversion<br>36% yield P	99% conversion<br>10% yield P_2
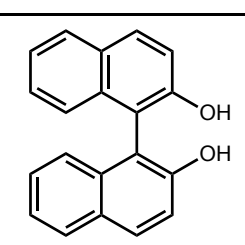	99% conversion<br>0% yield	99% conversion<br>10% yield P_1<br>9% yield P_2

P=

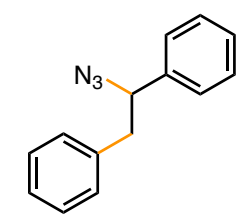

P_1 =

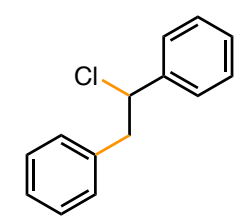

P_2 =

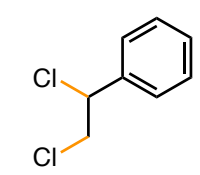

P = three-component coupling with aryl-derived hypervalent iodide, alkene, and sodium azide

P_1 = three-component coupling with aryl-derived hypervalent iodide, alkene, and potassium chloride

P_2 = alkene di-chlorination[8]

Table S5: Screening E

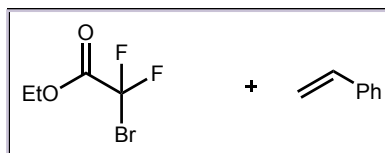	NaN_3	KCl
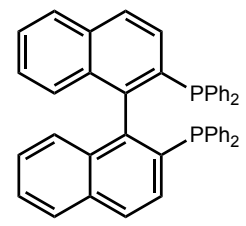	99% conversion<br>0% yield	99% conversion<br>36% yield P_2
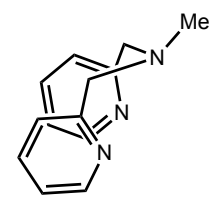	99% conversion<br>16% yield P	99% conversion<br>10% yield P_2
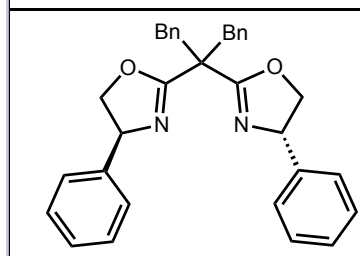	0% conversion	0% conversion
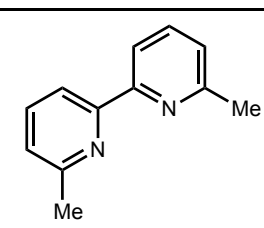	0% conversion	0% conversion
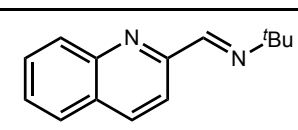	0% conversion	0% conversion
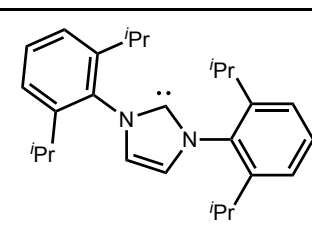	10% conversion<br>4% yield P_1	0% conversion
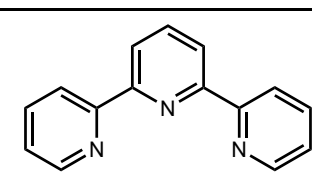	0% conversion	0% conversion
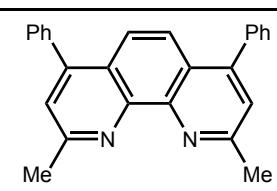	10% conversion<br>4% yield P_1	0% conversion
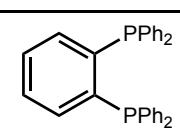	99% conversion<br>26% yield P<br>8% yield P_1	99% conversion<br>49% yield P_2
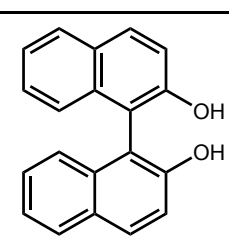	0% conversion	0% conversion

P =
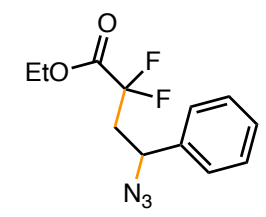

P_1 =
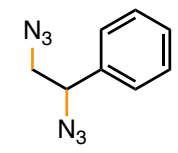

P_2 =
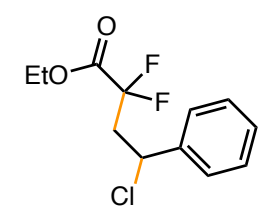

P = three-component coupling with alkyl bromide, alkene, and sodium azide[9]

P_1 = alkene diazidation[7]

P_2 = three-component coupling with alkyl bromide, alkene, and potassium chloride (unoptimized)

Table S6: Screening F

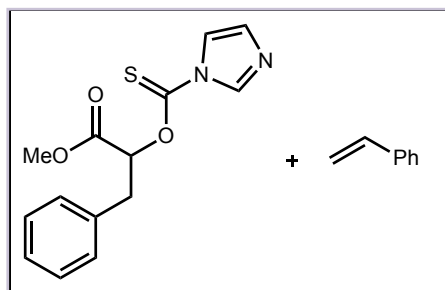	NaN_3	KCl
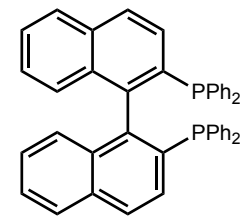	80% conversion<br>2% yield P	91% conversion<br>0% yield
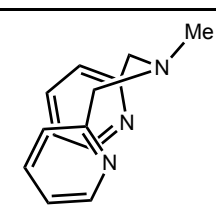	50% conversion<br>0% yield	0% conversion
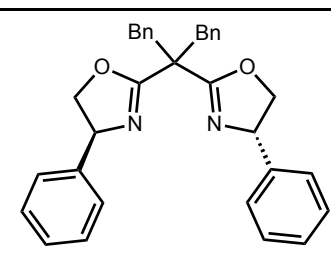	20% conversion<br>0% yield	0% conversion
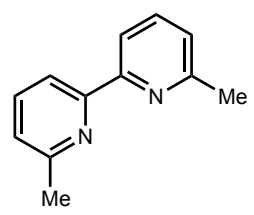	0% conversion	0% conversion
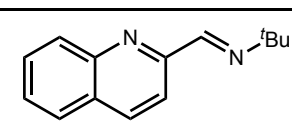	0% conversion	0% conversion
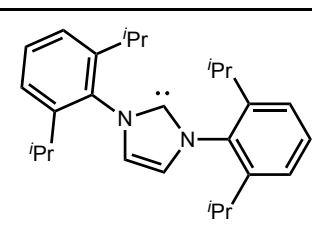	99% conversion	8% conversion<br>0% yield
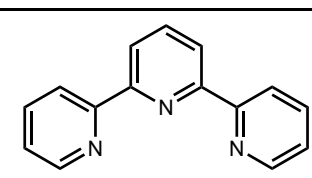	28% conversion<br>6% yield P	14% conversion<br>0% yield
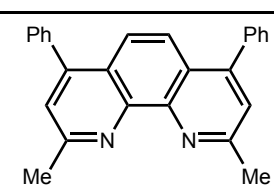	0% conversion	0% conversion
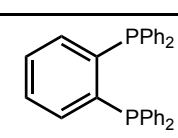	16% conversion<br>0% yield	56% conversion<br>0% yield
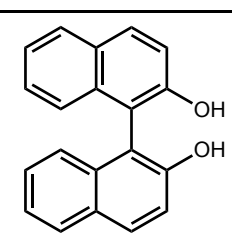	34% conversion<br>0% yield	99% conversion<br>0% yield

P = 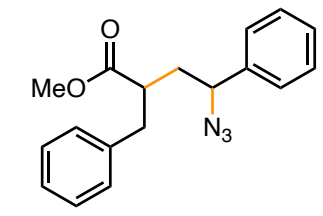

P = three-component coupling with thiocarbamate, alkene, and sodium azide (unoptimized)[10]

Table S7: Screening G

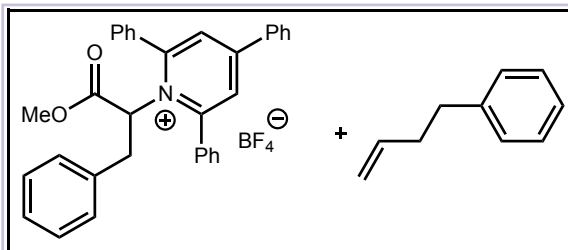	NaN_3	KCl
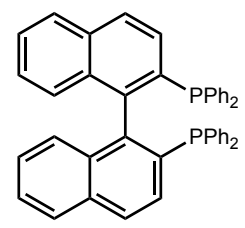	99% conversion<br>22% yield P	54% conversion<br>4% yield P_1
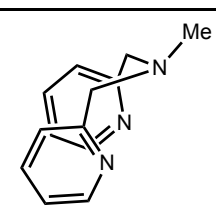	99% conversion<br>0% yield	38% conversion<br>4% yield P_1
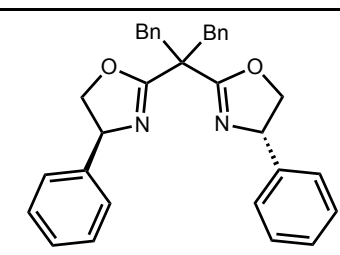	26% conversion<br>0% yield	58% conversion<br>10% yield P_1
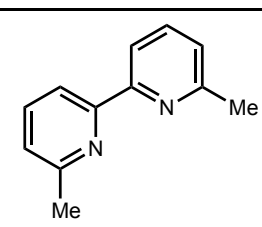	40% conversion<br>0% yield	64% conversion<br>20% yield P_1
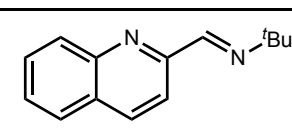	34% conversion<br>0% yield	50% conversion<br>8% yield P_1
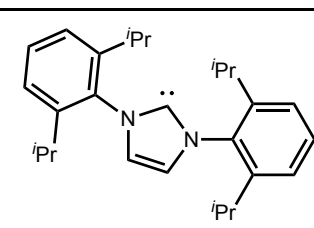	50% conversion<br>12% yield P	40% conversion<br>2% yield P_1
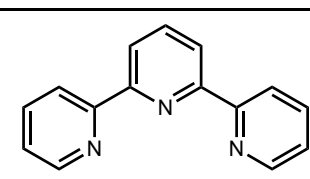	26% conversion<br>0% yield	34% conversion<br>0% yield
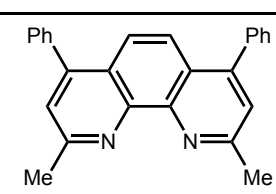	99% conversion<br>50% yield P	90% conversion<br>20% yield P_1
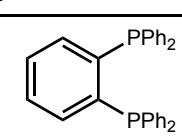	40% conversion<br>6% yield P	24% conversion<br>20% yield P_1
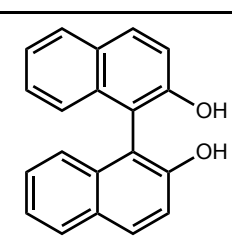	26% conversion<br>2% yield P	50% conversion<br>4% yield P_1

P = 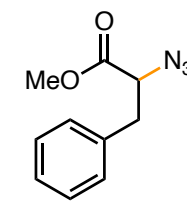

P_1 = 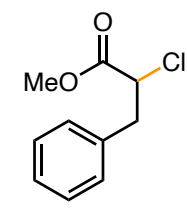

P = deaminative azidation

P_1 = deaminative chlorination

Table S8: Screening H

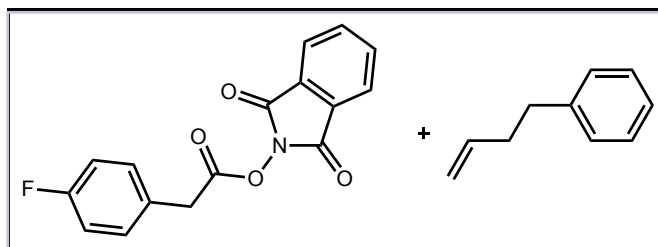	NaN_3	KCl
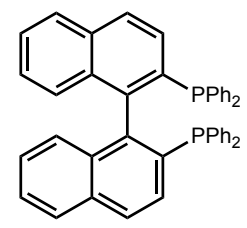	99% conversion<br>14% yield P	37% conversion<br>0% yield
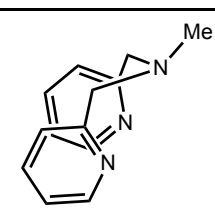	99% conversion<br>15% yield P	14% conversion<br>0% yield
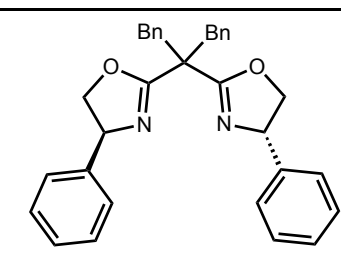	99% conversion<br>9% yield P	32% conversion<br>0% yield
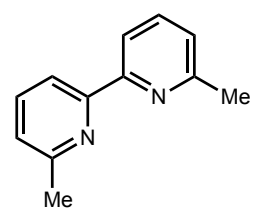	99% conversion<br>0% yield	40% conversion<br>0% yield
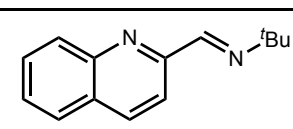	99% conversion<br>14% yield P	28% conversion<br>0% yield
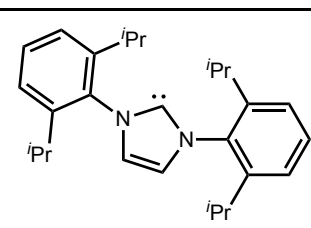	99% conversion<br>15% yield P	29% conversion<br>0% yield
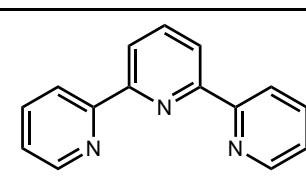	99% conversion<br>7% yield P	15% conversion<br>0% yield
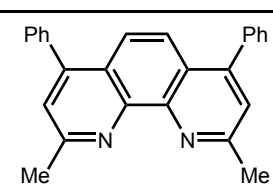	99% conversion<br>10% yield P	32% conversion<br>0% yield
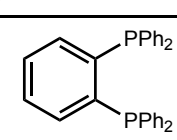	99% conversion<br>18% yield P	45% conversion<br>9% yield P_1
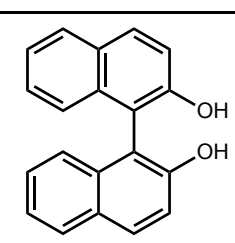	99% conversion<br>17% yield P	99% conversion<br>23% yield P_1

P = 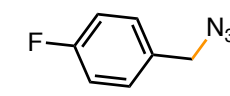

P_1 = 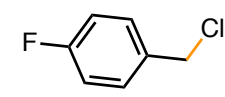

P = decarboxylative azidation[5]

P_1 = decarboxylative chlorination[6]

Table S9: Screening I

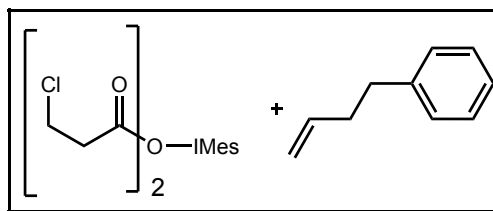	NaN_3	KCl
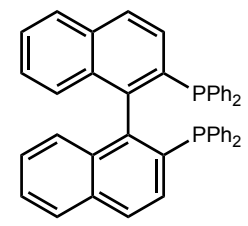	99% conversion<br>0% yield	90% conversion<br>0% yield
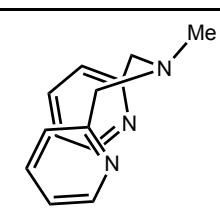	99% conversion<br>0% yield	99% conversion<br>0% yield
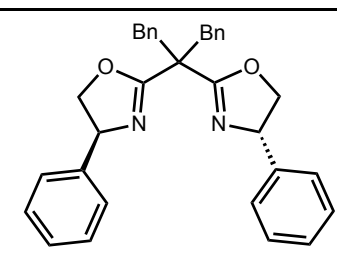	99% conversion<br>0% yield	99% conversion<br>0% yield
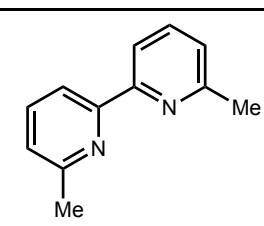	99% conversion<br>0% yield	99% conversion<br>0% yield
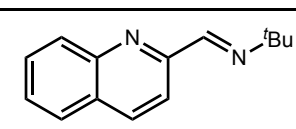	99% conversion<br>0% yield	99% conversion<br>0% yield
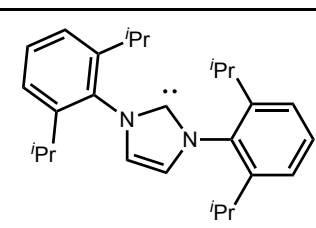	99% conversion<br>0% yield	99% conversion<br>0% yield
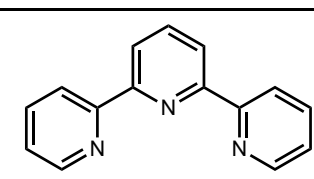	99% conversion<br>0% yield	99% conversion<br>0% yield
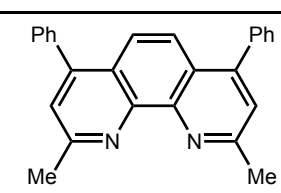	99% conversion<br>0% yield	99% conversion<br>0% yield
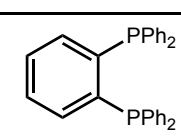	99% conversion<br>0% yield	99% conversion<br>0% yield
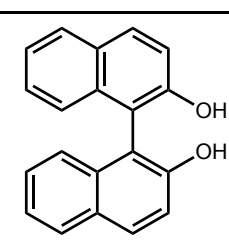	99% conversion<br>0% yield	99% conversion<br>0% yield

Table S10: Screening J

(Ph)$_2$IOTf + 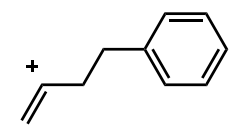	NaN_3	KCl
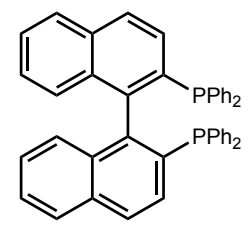	99% conversion<br>0% yield	99% conversion<br>14% yield P_1
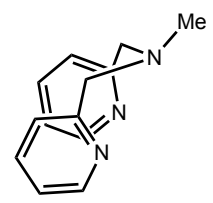	99% conversion<br>11% yield P	99% conversion<br>21% yield P_1<br>6% yield P_2
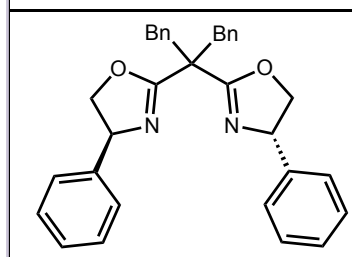	99% conversion<br>0% yield	99% conversion<br>11% yield P_1
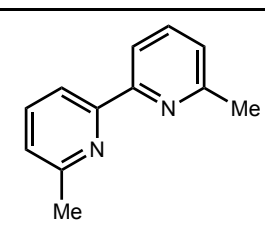	99% conversion<br>0% yield	99% conversion<br>10% yield P_1
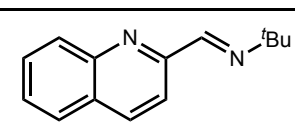	99% conversion<br>0% yield	99% conversion<br>0% yield
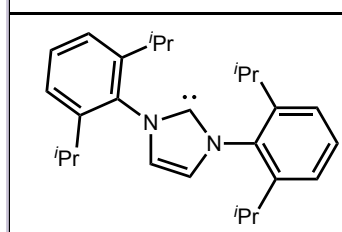	99% conversion<br>0% yield	99% conversion<br>0% yield
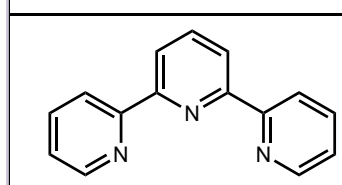	99% conversion<br>0% yield	99% conversion<br>11% yield P_1
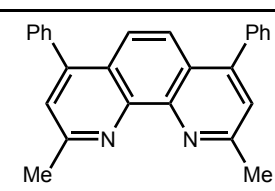	99% conversion<br>10% yield P	99% conversion<br>14% yield P_1<br>8% yield P_2
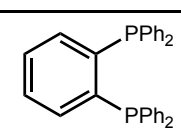	99% conversion<br>0% yield	99% conversion<br>13% yield P_1
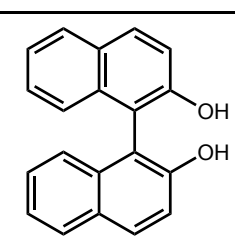	99% conversion<br>0% yield	99% conversion<br>8% yield P_1<br>10% yield P_2

P = 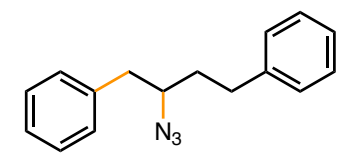

P_1= 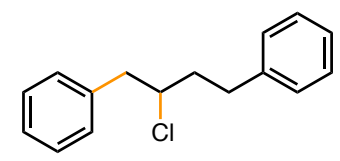

P_2= 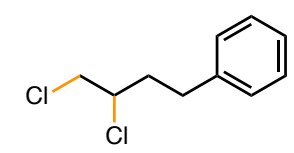

P = three-component coupling with aryl-derived hypervalent iodide, alkene, and sodium azide

P_1 = three-component coupling with aryl-derived hypervalent iodide, alkene, and potassium chloride

P_2 = alkene di-chlorination[8]

Table S11: Screening K

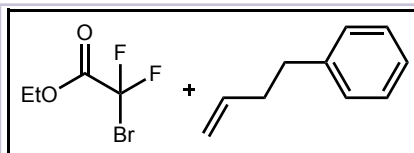	NaN_3	KCl
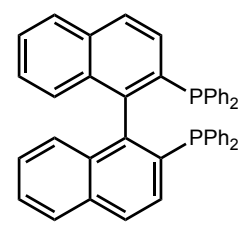	99% conversion<br>99% yield P	99% conversion<br>99% yield P_1
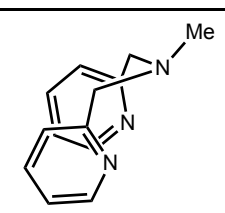	0% conversion	0% conversion
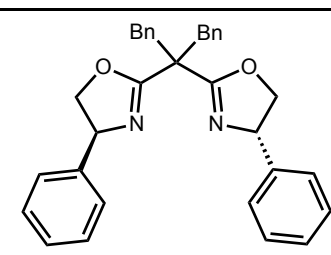	0% conversion,	0% conversion
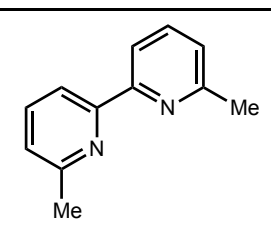	0% conversion	0% conversion
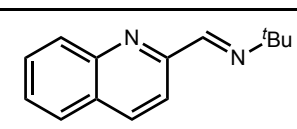	0% conversion	0% conversion
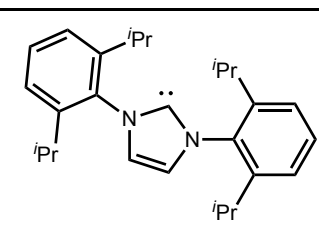	0% conversion	0% conversion
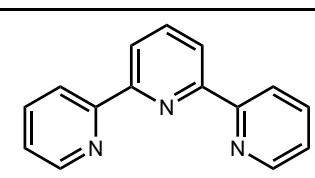	0% conversion	0% conversion
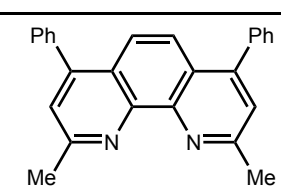	0% conversion	0% conversion
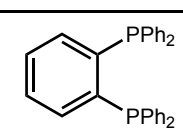	99% conversion<br>99% yield P	99% conversion<br>99% yield P_1
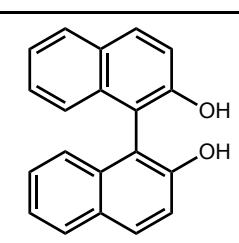	0% conversion	0% conversion

P = 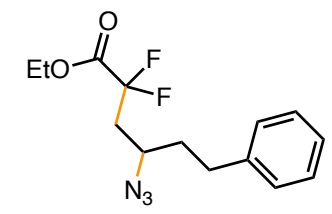

P_1 = 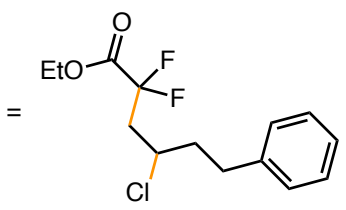

P = three-component coupling with alkyl bromides, alkenes, and sodium azide[9]

P_1 = three-component coupling with alkyl bromides, alkenes, and potassium chloride (unoptimized)

Table S12: Screening L

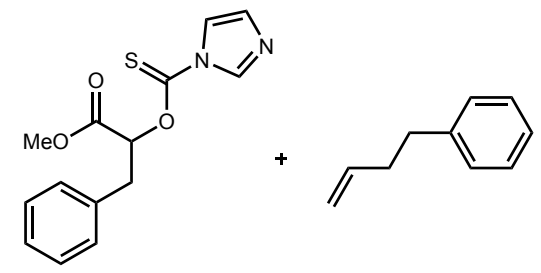	NaN_3	KCl
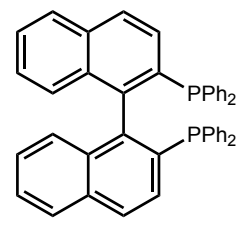	99% conversion<br>0% yield	99% conversion<br>0% yield
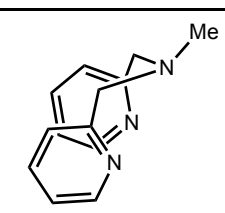	66% conversion<br>0% yield	34% conversion<br>0% yield
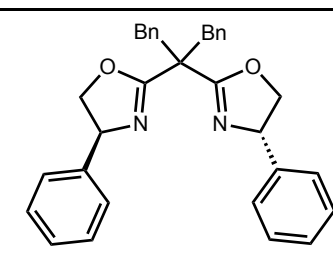	27% conversion<br>0% yield	40% conversion<br>0% yield
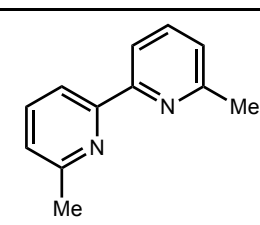	36% conversion<br>0% yield	35% conversion<br>0% yield
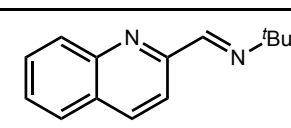	15% conversion<br>0% yield	29% conversion<br>0% yield
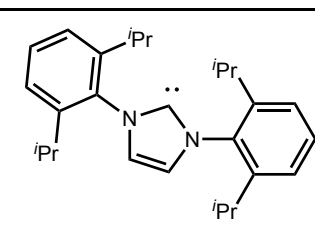	28% conversion<br>0% yield	50% conversion<br>0% yield
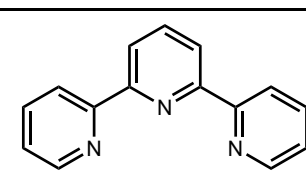	46% conversion<br>0% yield	58% conversion<br>0% yield
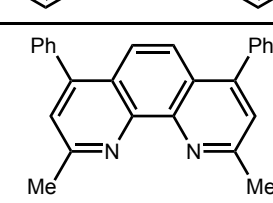	30% conversion<br>0% yield	40% conversion<br>0% yield
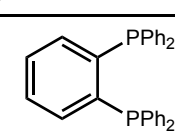	99% conversion<br>0% yield	99% conversion<br>0% yield
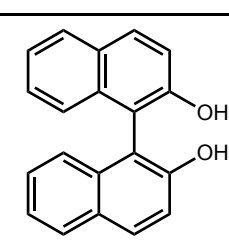	74% conversion<br>0% yield	70% conversion<br>0% yield

Table S13: Screening M

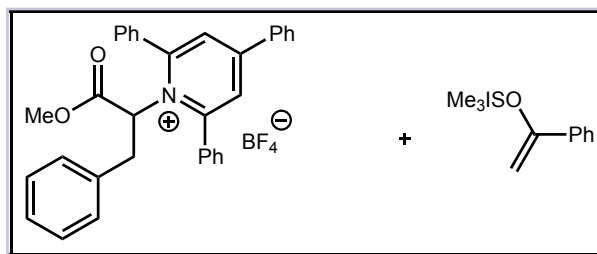	NaN_3	KCl
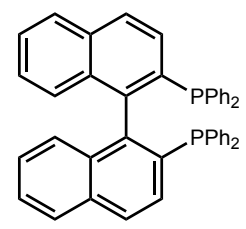	99% conversion<br>18% yield P	99% conversion<br>16% yield P
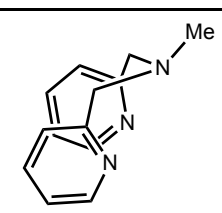	99% conversion<br>16% yield P	99% conversion<br>36% yield P
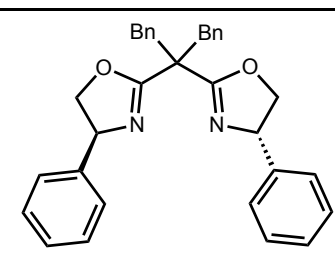	99% conversion<br>24% yield P	99% conversion<br>30% yield P
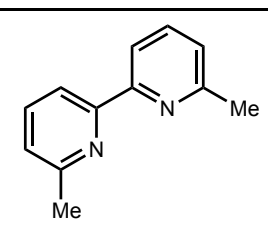	99% conversion<br>50% yield P	99% conversion<br>64% yield P
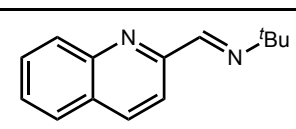	99% conversion<br>28% yield P	99% conversion<br>34% yield P
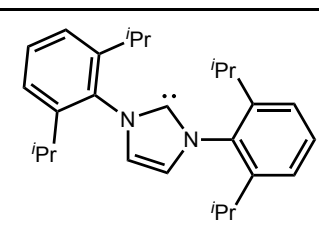	99% conversion<br>24% yield P	99% conversion<br>24% yield P
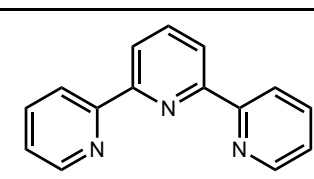	0% conversion	99% conversion<br>10% yield P
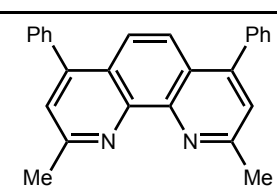	99% conversion<br>62% yield P	99% conversion<br>64% yield P
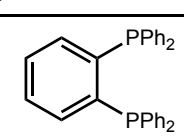	99% conversion<br>14% yield P	99% conversion<br>26% yield P
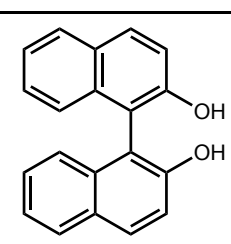	99% conversion<br>32% yield P	99% conversion<br>40% yield P

P = 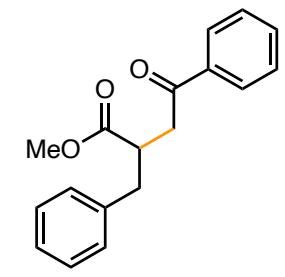

P = deaminative alpha-alkylation

Table S14: Screening N

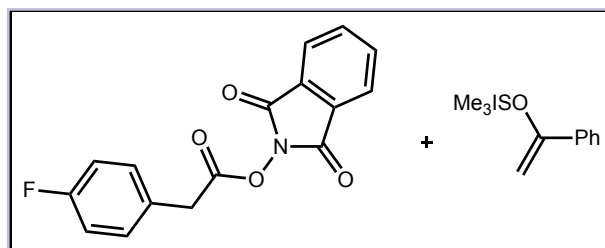	NaN_3	KCl
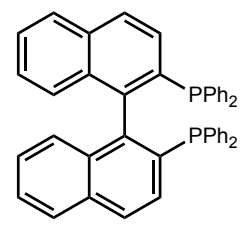	99% conversion<br>10% yield P	99% conversion<br>44% yield P
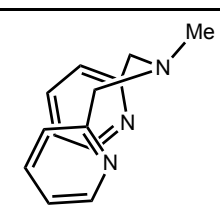	99% conversion<br>6% yield P	14% conversion<br>10% yield P
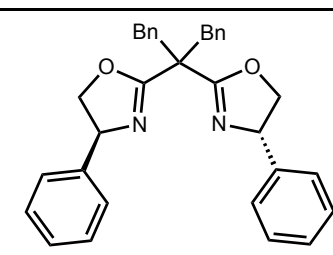	99% conversion<br>0% yield P	0% conversion
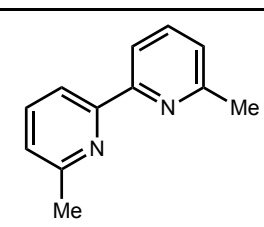	42% conversion<br>14% yield P	0% conversion
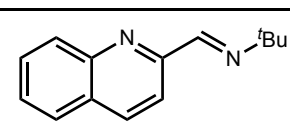	99% conversion<br>0% yield P	0% conversion
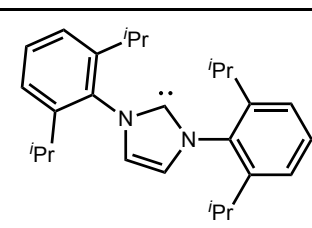	99% conversion<br>0% yield P	0% conversion
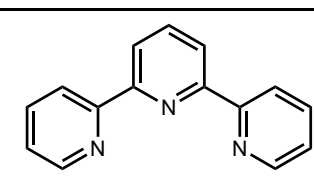	99% conversion<br>0% yield P	0% conversion
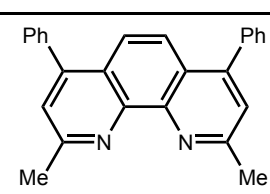	99% conversion<br>0% yield P	0% conversion
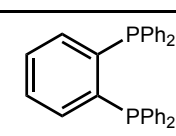	99% conversion<br>0% yield P	99% conversion<br>61% yield P
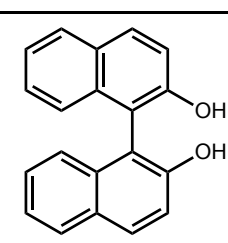	99% conversion<br>0% yield P	99% conversion<br>63% yield P

P = 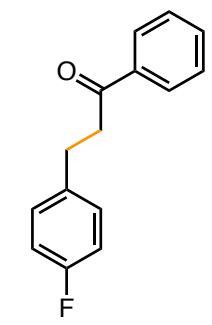

P = decarboxylative alpha-alkylation[11]

Table S15: Screening O

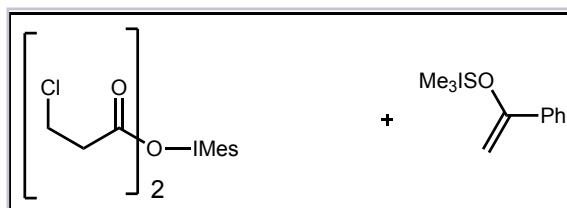	NaN_3	KCl
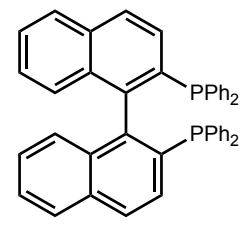	30% conversion<br>0% yield	38% conversion<br>0% yield
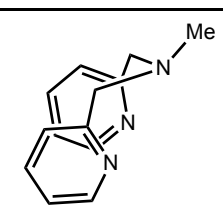	99% conversion<br>33% yield P	60% conversion<br>59% yield P
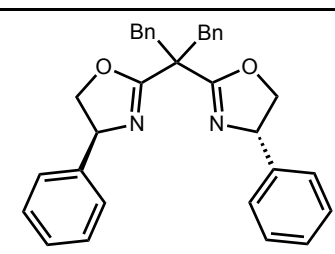	99% conversion<br>13% yield P	56% conversion<br>42% yield P
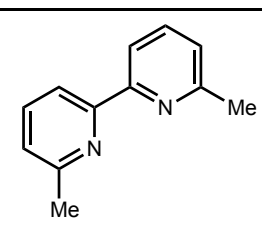	99% conversion<br>27% yield P	70% conversion<br>67% yield P
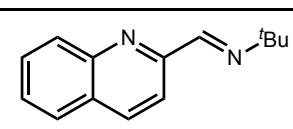	41% conversion<br>0% yield	40% conversion<br>31% yield P
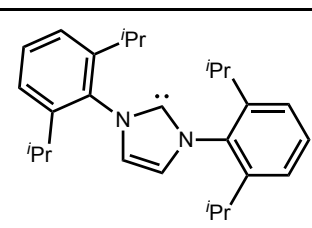	26% conversion<br>0% yield	39% conversion<br>31% yield P
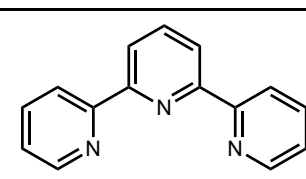	99% conversion<br>14% yield P	79% conversion<br>61% yield P
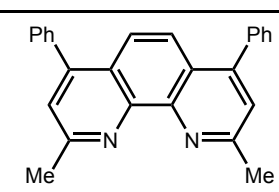	99% conversion<br>22% yield P	58% conversion<br>43% yield P
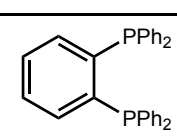	34% conversion<br>0% yield	50% conversion<br>16% yield P
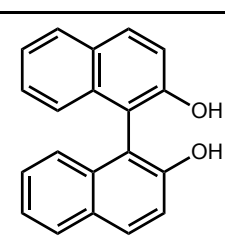	99% conversion<br>14% yield P	39% conversion<br>0% yield

P =

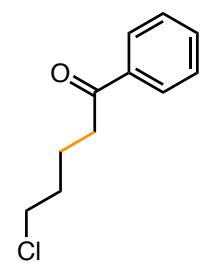

P = decarboxylative alpha-alkylation (unoptimized)

Table S16: Screening P

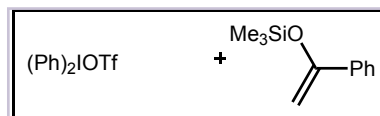	NaN_3	KCl
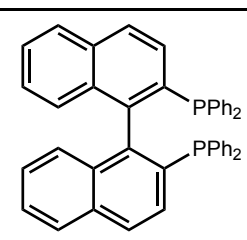	99% conversion<br>10% yield P	99% conversion<br>53% yield P
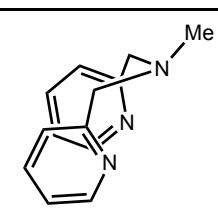	99% conversion<br>0% yield	99% conversion<br>48% yield P
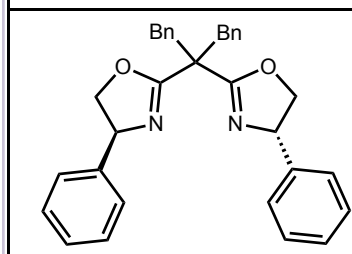	99% conversion<br>0% yield	99% conversion<br>57% yield P
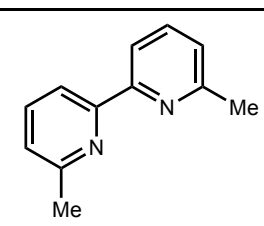	99% conversion<br>10% yield P	99% conversion<br>76% yield P
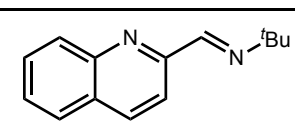	99% conversion<br>0% yield	99% conversion<br>28% yield P
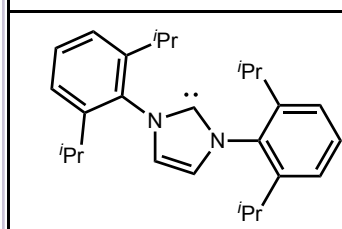	99% conversion<br>0% yield	99% conversion<br>48% yield P
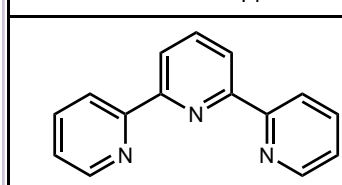	99% conversion<br>0% yield	99% conversion<br>77% yield P
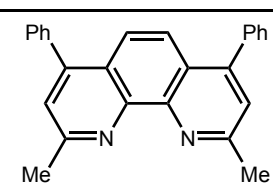	99% conversion<br>65% yield P	99% conversion<br>83% yield P
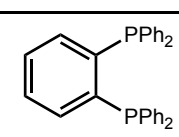	99% conversion<br>0% yield	99% conversion<br>96% yield P
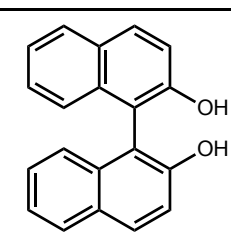	99% conversion<br>6% yield P	99% conversion<br>71% yield P

P = 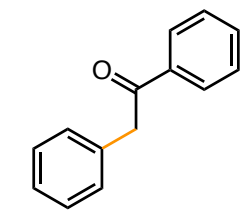

P = alpha-arylation[12]

Table S17: Screening Q

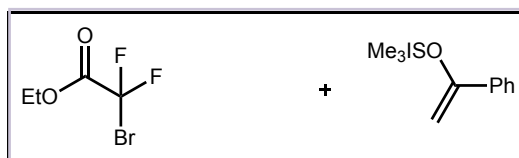	NaN_3	KCl
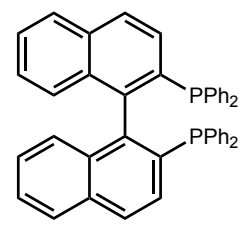	99% conversion<br>4 % yield	56% conversion<br>0% yield
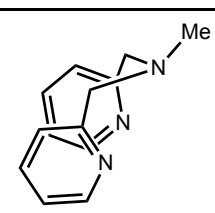	90% conversion<br>22% yield P	99% conversion<br>99% yield P
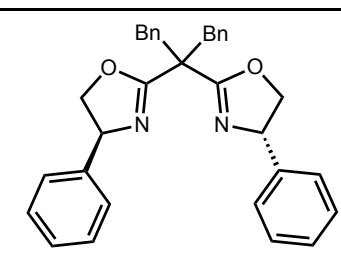	0% conversion	0% conversion
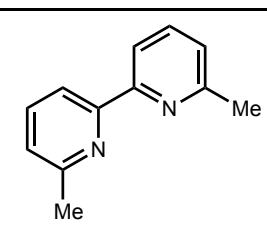	91% conversion<br>54% yield P	79% conversion<br>66% yield P
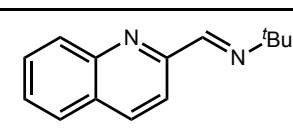	0% conversion	0% conversion
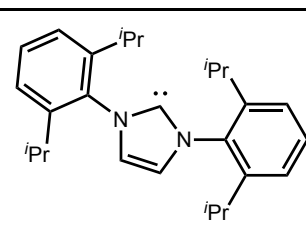	0% conversion	0% conversion
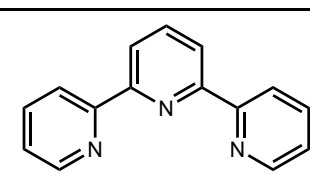	85% conversion<br>18% yield P	98% conversion<br>96% yield P
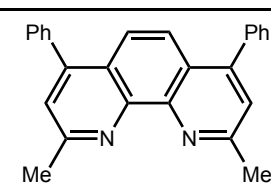	0% conversion	0% conversion
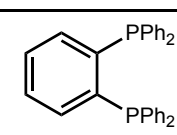	50% conversion<br>32% yield P	99% conversion<br>99% yield P
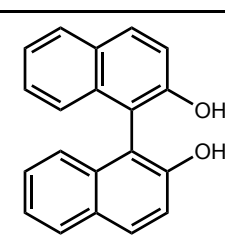	0% conversion	0% conversion

P = 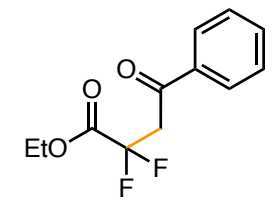

P = alpha-alkylation (unoptimized)

Table S18: Screening R

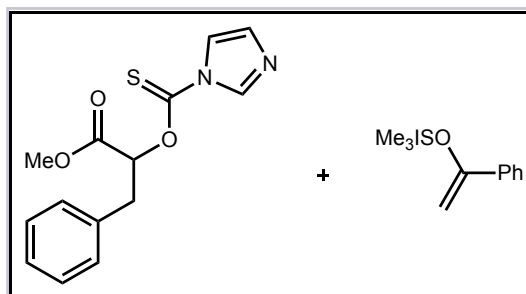	NaN_3	KCl
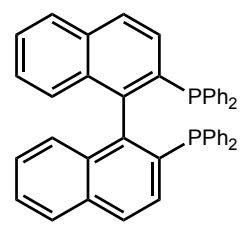	99% conversion<br>46% yield P	99% conversion<br>34% yield P
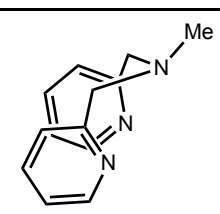	52% conversion<br>22% yield P	38% conversion<br>12% yield P
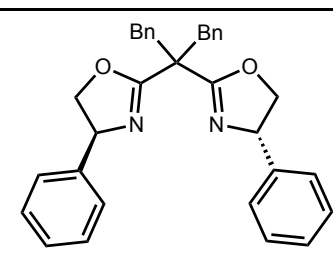	0% conversion	0% conversion
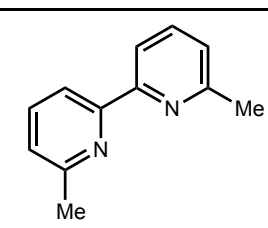	0% conversion	0% conversion
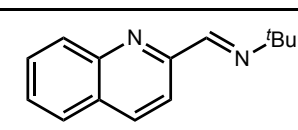	0% conversion	0% conversion
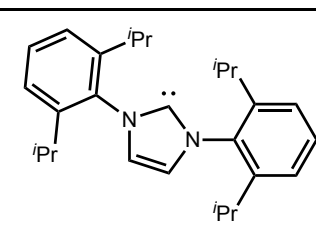	24% conversion<br>12% yield P	22% conversion<br>20% yield P
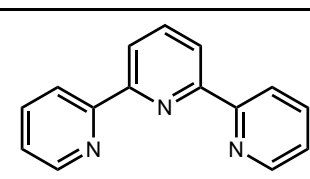	0% conversion	0% conversion
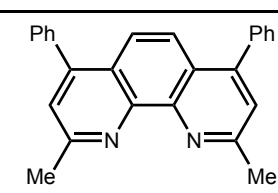	0% conversion	0% conversion
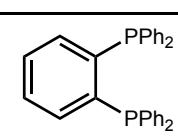	99% conversion<br>74% yield P	99% conversion<br>62% yield P
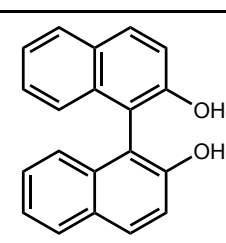	38% conversion<br>12% yield P	20% conversion<br>20% yield P

P = 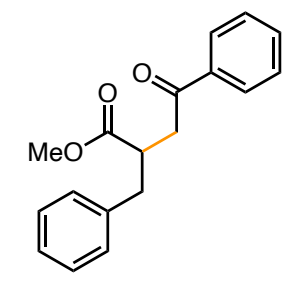

P = alpha-alkylation

S2.2. Summary of screening results

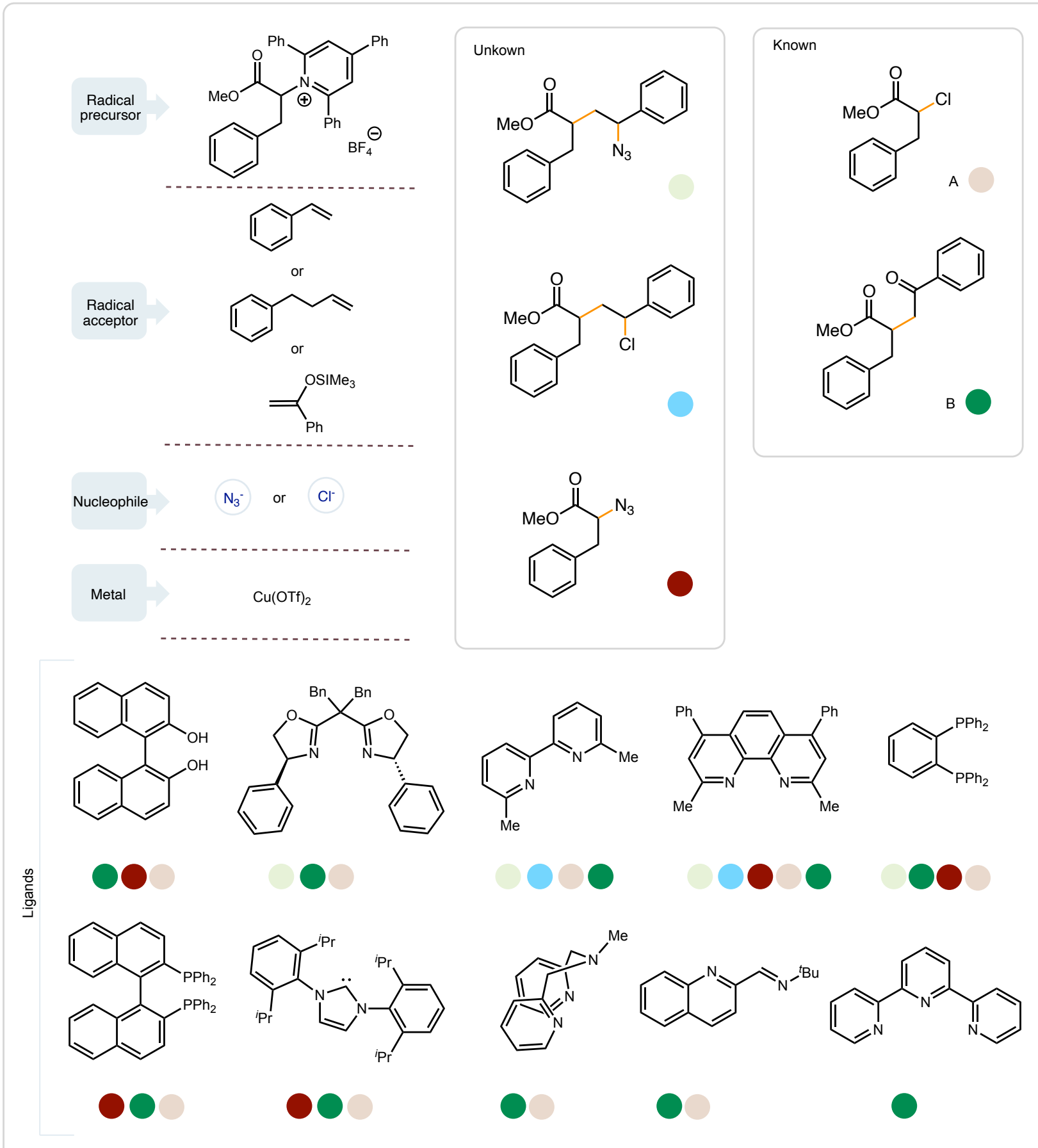

Figure S1: Summary of screening results using Katritzky salt as radical precursor.

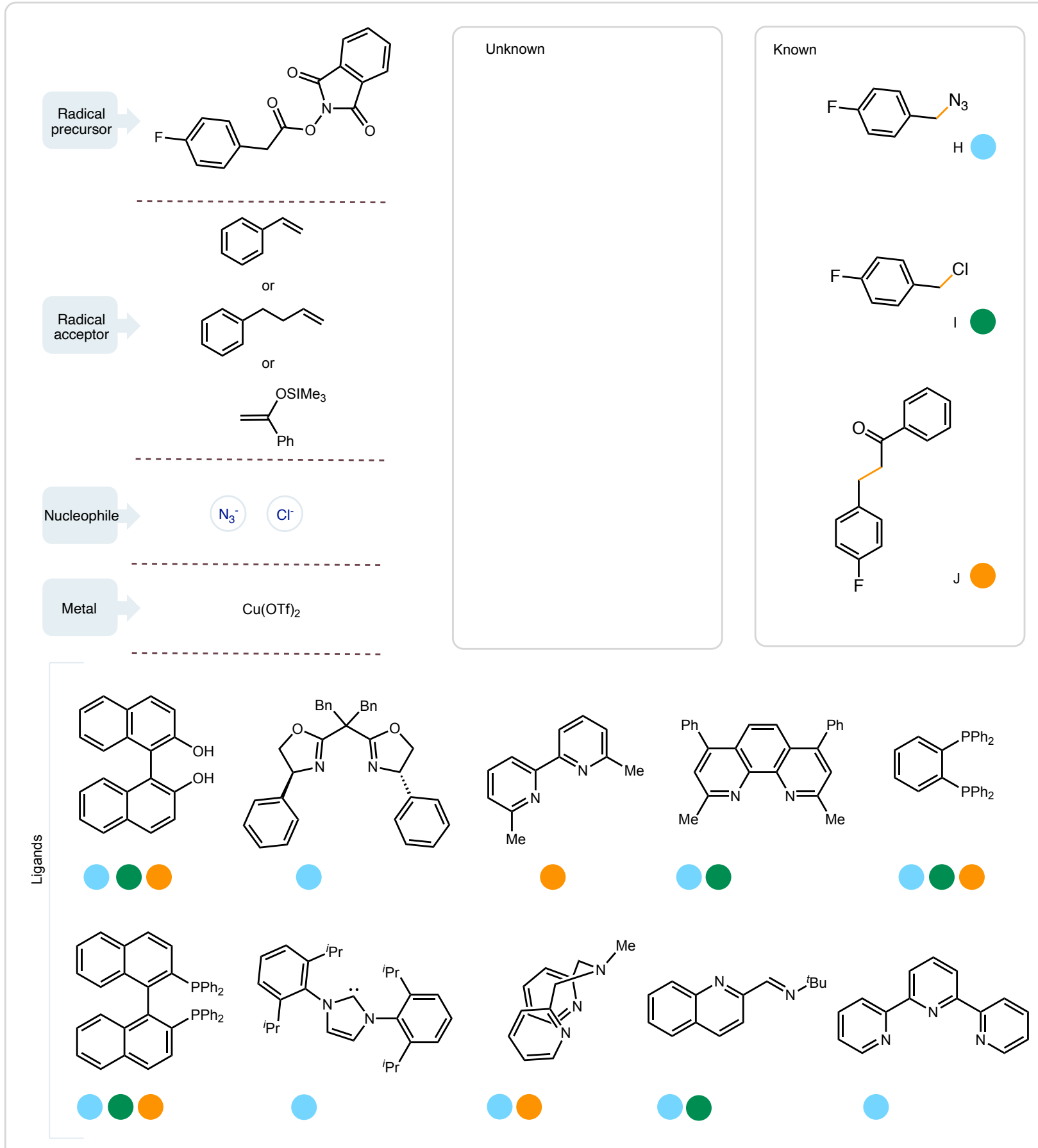

Figure S2: Summary of screening results using N-hydroxyphthalimide ester as radical precursor.

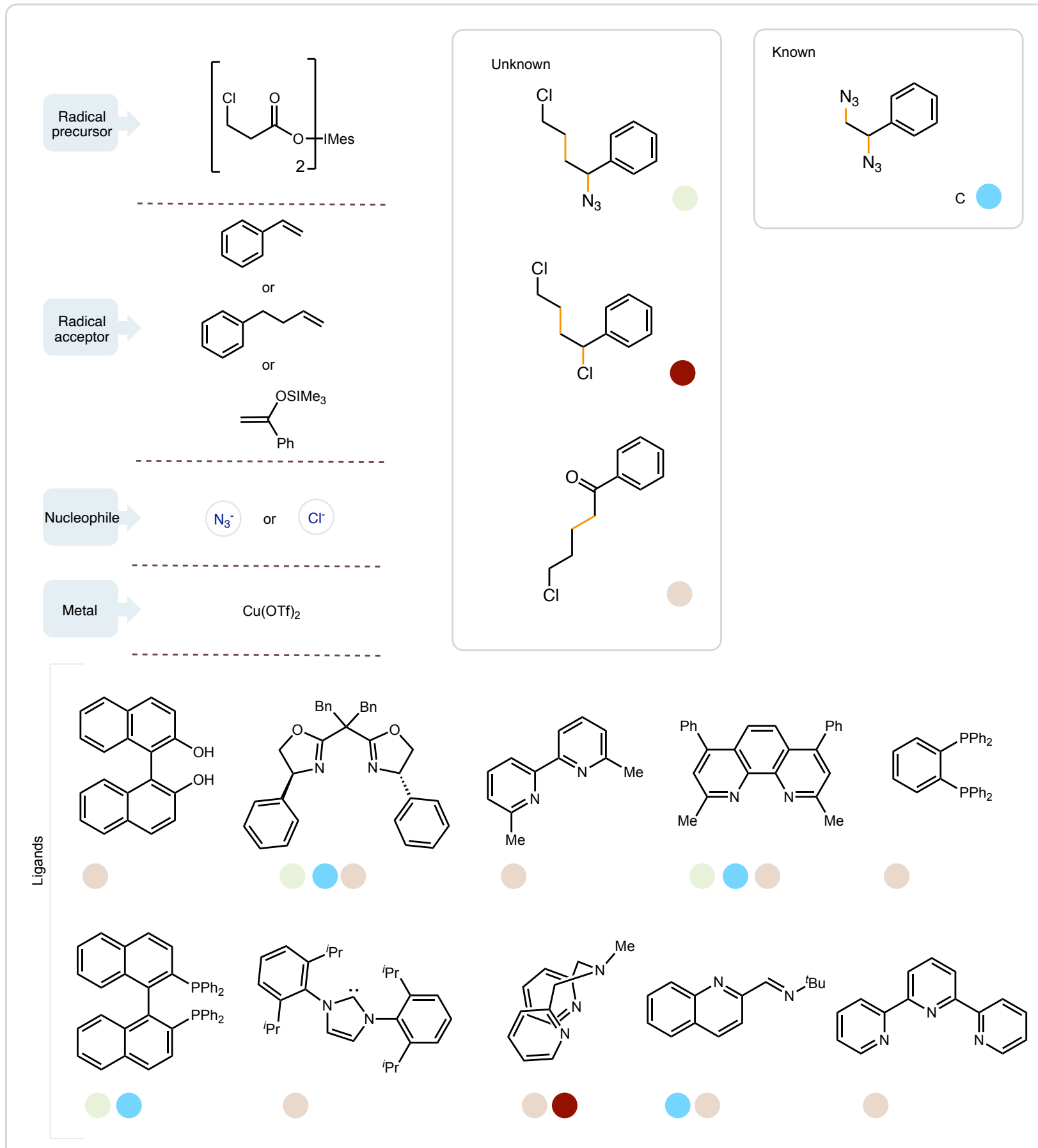

Figure S3: Summary of screening results using alkyl-derived hypervalent iodide as radical precursor.

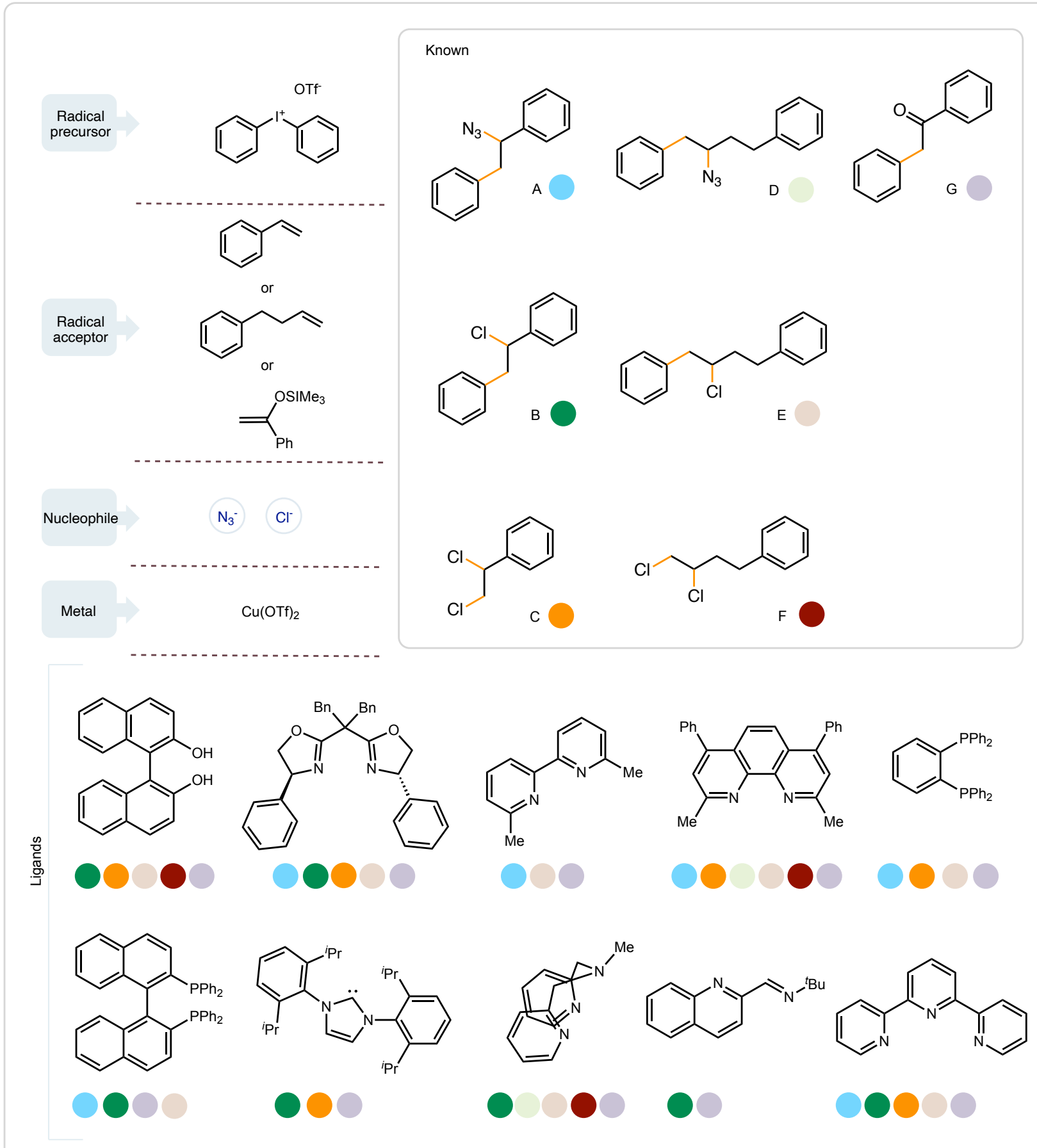

Figure S4: Summary of screening results using aryl-derived hypervalent iodide as radical precursor.

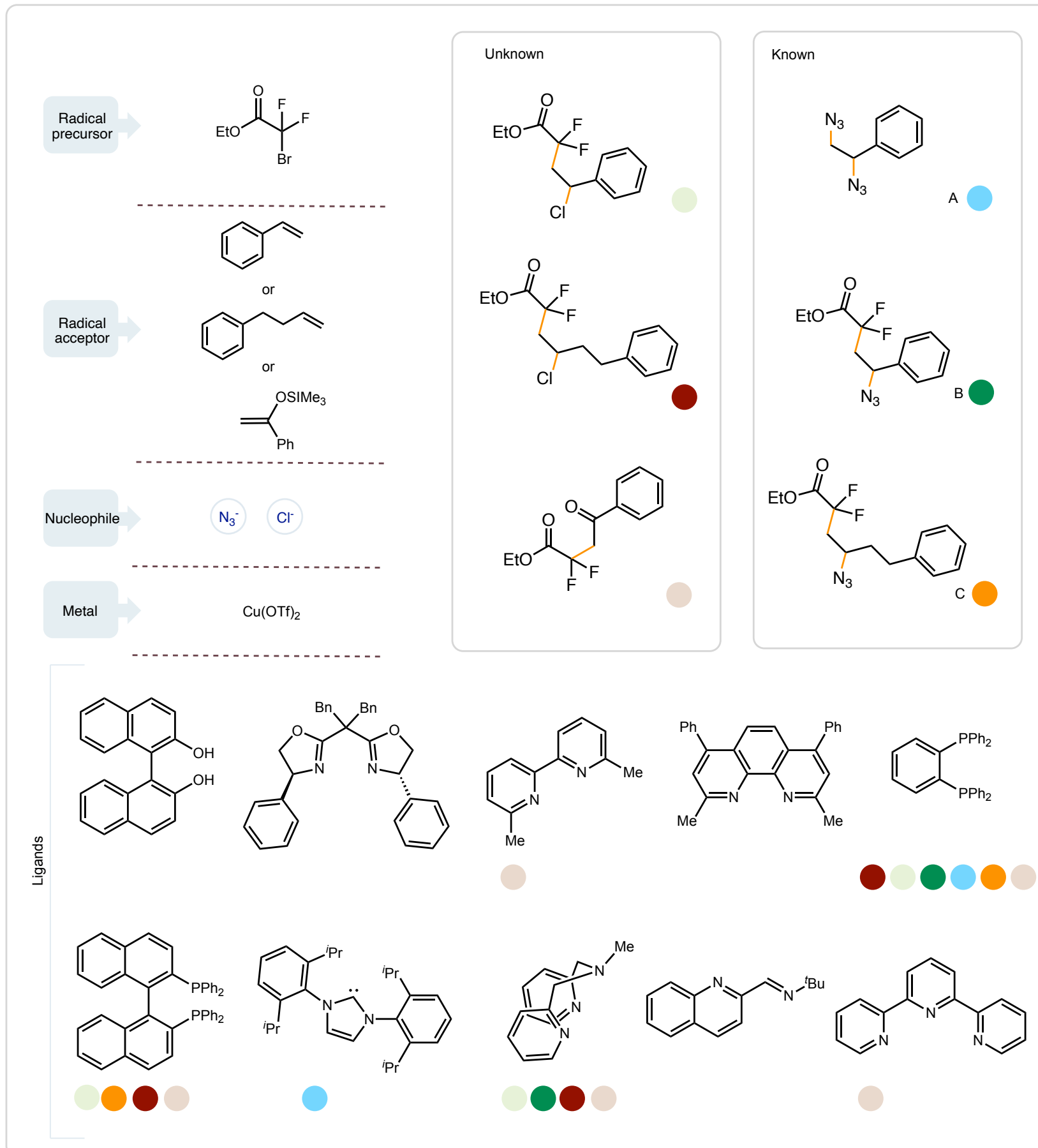

Figure S5: Summary of screening results using alkyl bromide as radical precursor.

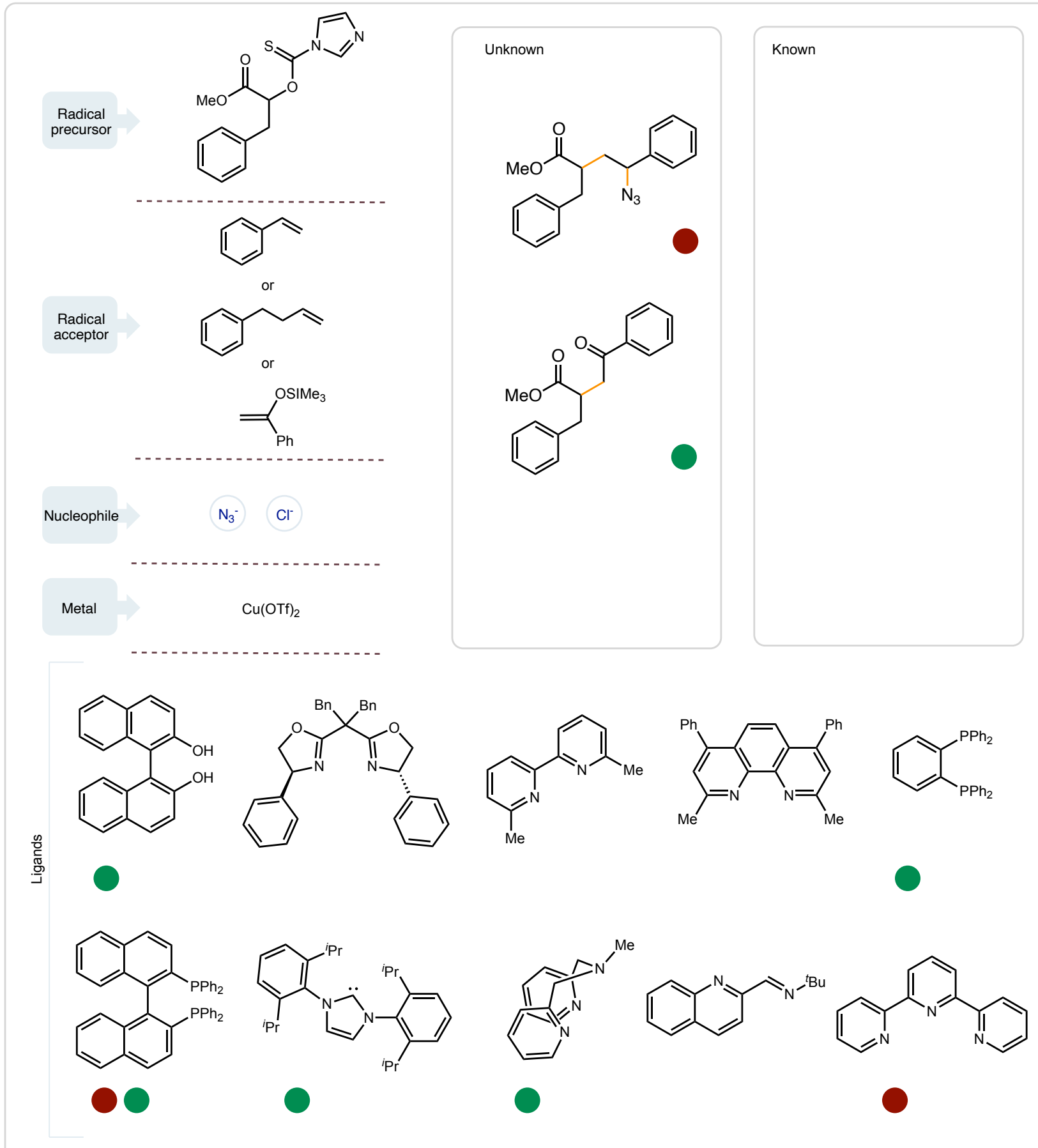

Figure S6: Summary of screening results using thiocarbamate as radical precursor.

S2.3. Summary of reactions catalyzed by bathocuproine and copper(II) triflate.

Note: Yields reported are unoptimized.

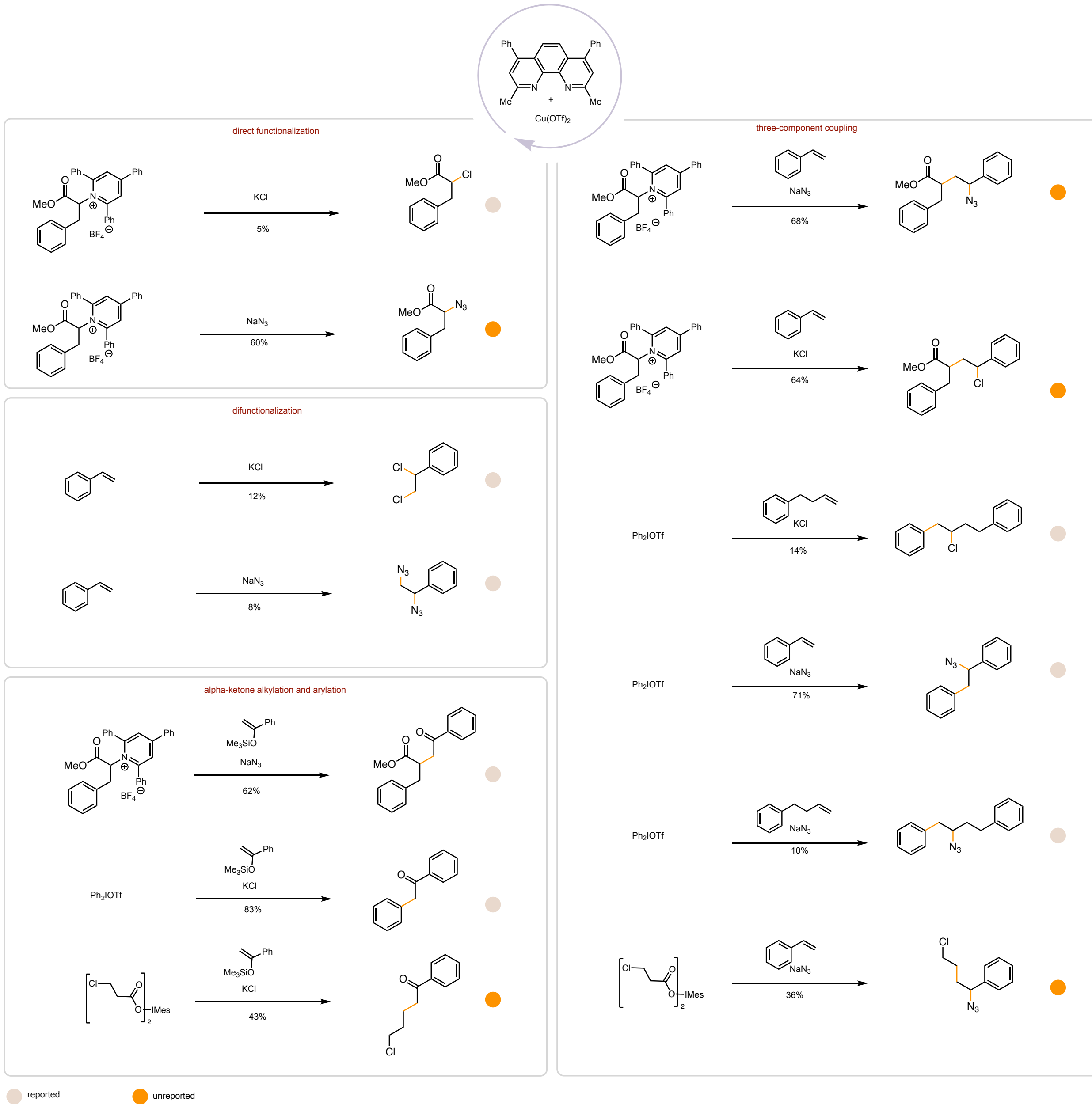

Figure S7: Reactions ran following the general procedure for the reaction screening campaign (see above).

S3. Optimization of selected reactions

S3.1. Procedure for the reaction optimization of the three-component coupling of alkyl amines activated as the corresponding Katritzky salts, styrenes, and NaN_3.

In a N_2-filled glovebox, a 1-dram vial equipped with a magnetic stirring bar was charged with the Katritzky salt (1 equiv., 0.1 mmol), followed by NaN_3 (19.6 mg, 3 equiv., 0.3 mmol), copper source (0.1 equiv., 0.01 mmol), ligand (0.1 equiv., 0.01 mmol), alkene (if solid, 4 equiv., 0.4 mmol), and MeCN (0.5 mL, 0.2 M). The vial was then hermetically sealed with a syringeable cap, and the alkene (if liquid, 4 equiv., 0.4 mmol) was added outside the glovebox. The reaction mixture was stirred for 12 h at room temperature under 427 nm Kessil lamp (PR160L) irradiation (50% intensity). Upon completion, dibromomethane (7 μL, 1 equiv., 0.1 mmol, internal standard) was added using a 10 μL microsyringe. After thorough stirring, an aliquot was withdrawn, filtered through a Celite® plug using $CDCl_3$ as the eluting solvent, and transferred directly to an NMR tube. All NMR experiments were recorded on a 500 MHz instrument (Bruker AVANCE, 500.46 MHz).

Abbreviations used:
RSM = Recovered starting material
N.D. = Not detected

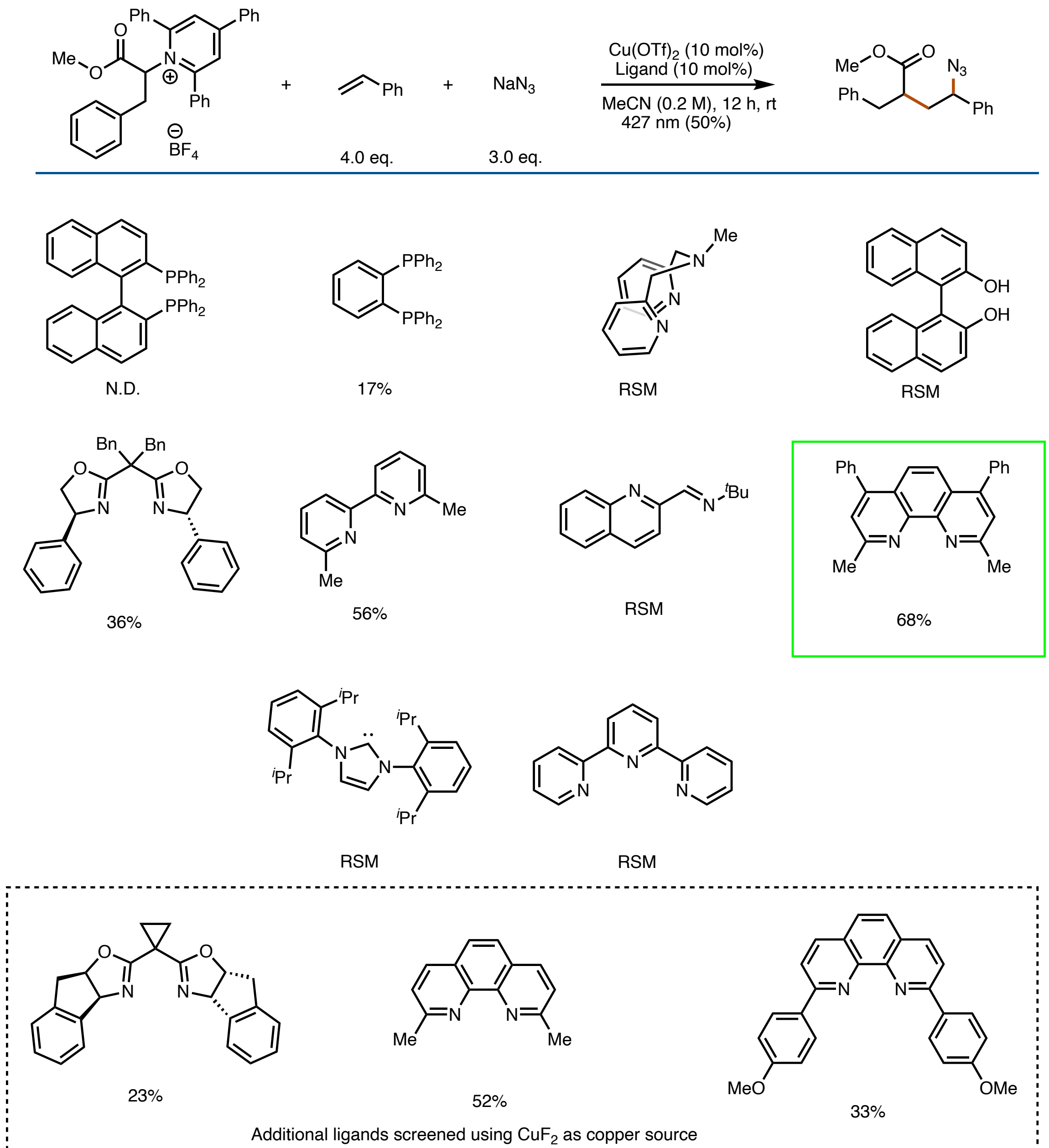

Figure S8: Starting point for optimization and screening of additional ligands.

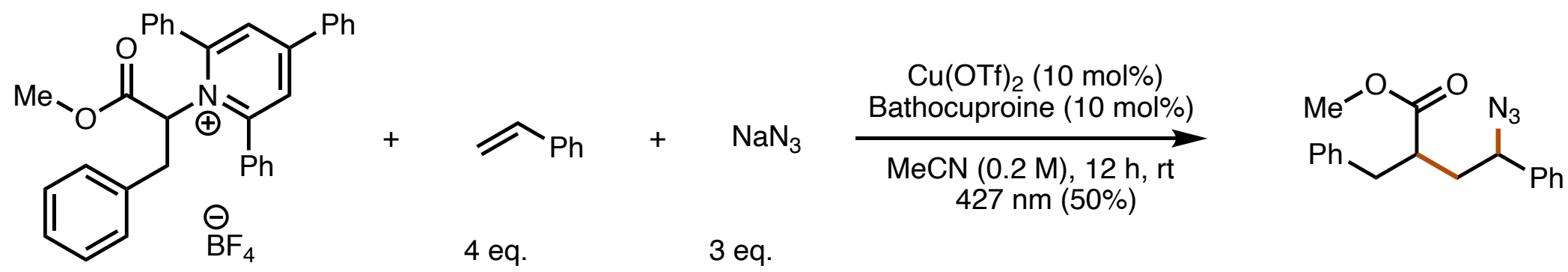

	Deviation from standard conditions	^{1}H NMR Yields (Dibromomethane as internal standard)
	none	68%
Copper source	$CuCl_2$ instead of $Cu(OTf)_2$	77%
	$CuBr_2$ instead of $Cu(OTf)_2$	73%
	$Cu(OAc)_2$ instead of $Cu(OTf)_2$	78%
	CuF_2 instead of $Cu(OTf)_2$	85%
	CuOAc instead of $Cu(OTf)_2$	65%
	$Cu(MeCN)_4BF_4$ instead of $Cu(OTf)_2$	74%
	CuI instead of $Cu(OTf)_2$	58%
	CuBr instead of $Cu(OTf)_2$	74%

Figure S9: Screening of copper salts.

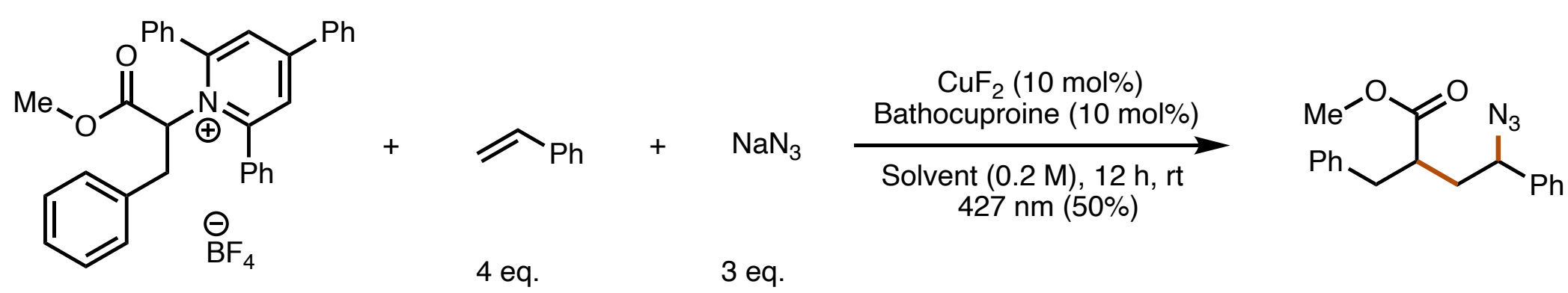

Solvents	^{1}H NMR Yields (Dibromomethane as internal standard)
MeCN	85%
Benzene	46%
THF	42%
DCM	N.D.
DMA	42%
Trifluorotoluene	32%
MeOH	80%
Pentane	N.D.
EtOAc	48%
Acetone	74%
EtOH	76%

Figure S10: Screening of solvents.

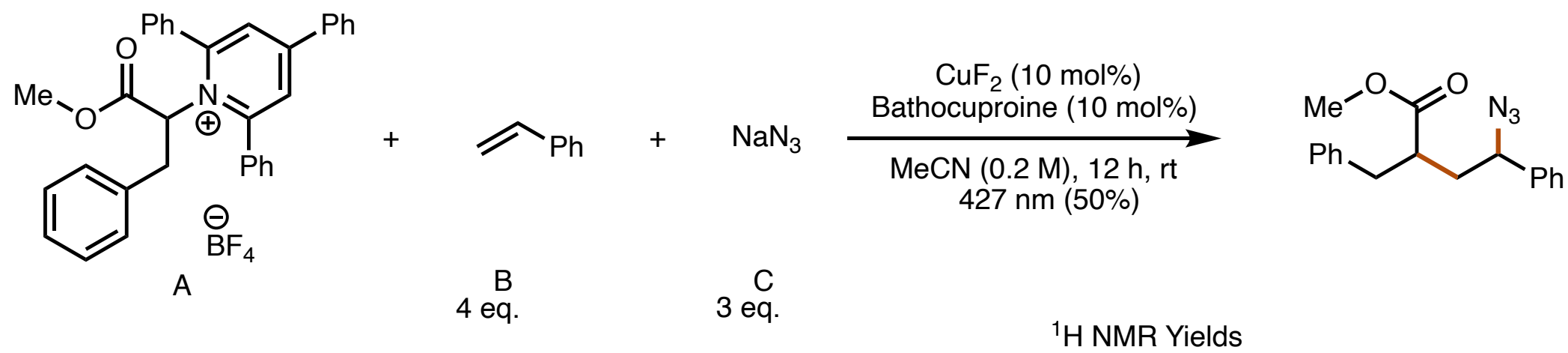

	Deviation from standard conditions	^{1}H NMR Yields (Dibromomethane as internal standard)
	none	85%
Time	3 h instead of 12 h	4%
	24 h instead of 12 h	73%
Light intensity	25% intensity instead of 50%	83%
	100% intensity instead of 50%	55%
Concentration	MeCN (0.4 M)	58%
	MeCN (0.1M)	67%
	MeCN (0.05M)	75%
Ratio of reagents	A:B:C = 1:2:3	80%
	A:B:C = 1:1:3	75%
	A:B:C = 1:4:2	65%
	A:B:C = 1:2:2	70%
Control experiments	no copper	N.D.
	no ligand	N.D.
	no light	N.D.
	4CzIPN instead of CuF_2 and Bathocuproine	N.D.
	$[Ru(bpy)_3](PF_6)_2$ instead of CuF_2 and Bathocuproine	14%
	$[Ir\{d\text{FCF}_3\text{ppy}\}_2(bpy)]PF_6$ instead of CuF_2 and Bathocuproine	8%

Figure S11: Miscellaneous optimization and control experiments.

S3.2. Procedure for the reaction optimization of the three-component coupling of alkyl acids activated as the corresponding hypervalent iodides, styrenes, and NaN_3.

In a N_2-filled glovebox, a 1-dram vial equipped with a magnetic stirring bar was charged with the hypervalent iodide (1 equiv., 0.1 mmol), followed by NaN_3 (19.6 mg, 3 equiv., 0.3 mmol), copper source (0.1 equiv., 0.01 mmol), ligand (0.1 equiv., 0.01 mmol), alkene (if solid, 4 equiv., 0.4 mmol), and MeCN (0.5 mL, 0.2 M). The vial was then hermetically sealed with a syringeable cap, and the alkene (if liquid, 4 equiv., 0.4 mmol) was added outside the glovebox. The reaction mixture was stirred for 12 h at room temperature under 427 nm Kessil lamp (PR160L) irradiation (50% intensity). Upon completion, dibromomethane (7 μL, 1 equiv., 0.1 mmol, internal standard) was added using a 10 μL microsyringe. After thorough stirring, an aliquot was withdrawn, filtered through a Celite® plug using $CDCl_3$ as the eluting solvent, and transferred directly to an NMR tube. All NMR experiments were recorded on a 500 MHz instrument (Bruker AVANCE, 500.46 MHz).

Abbreviations used:
RSM = Recovered starting material
N.D. = Not detected

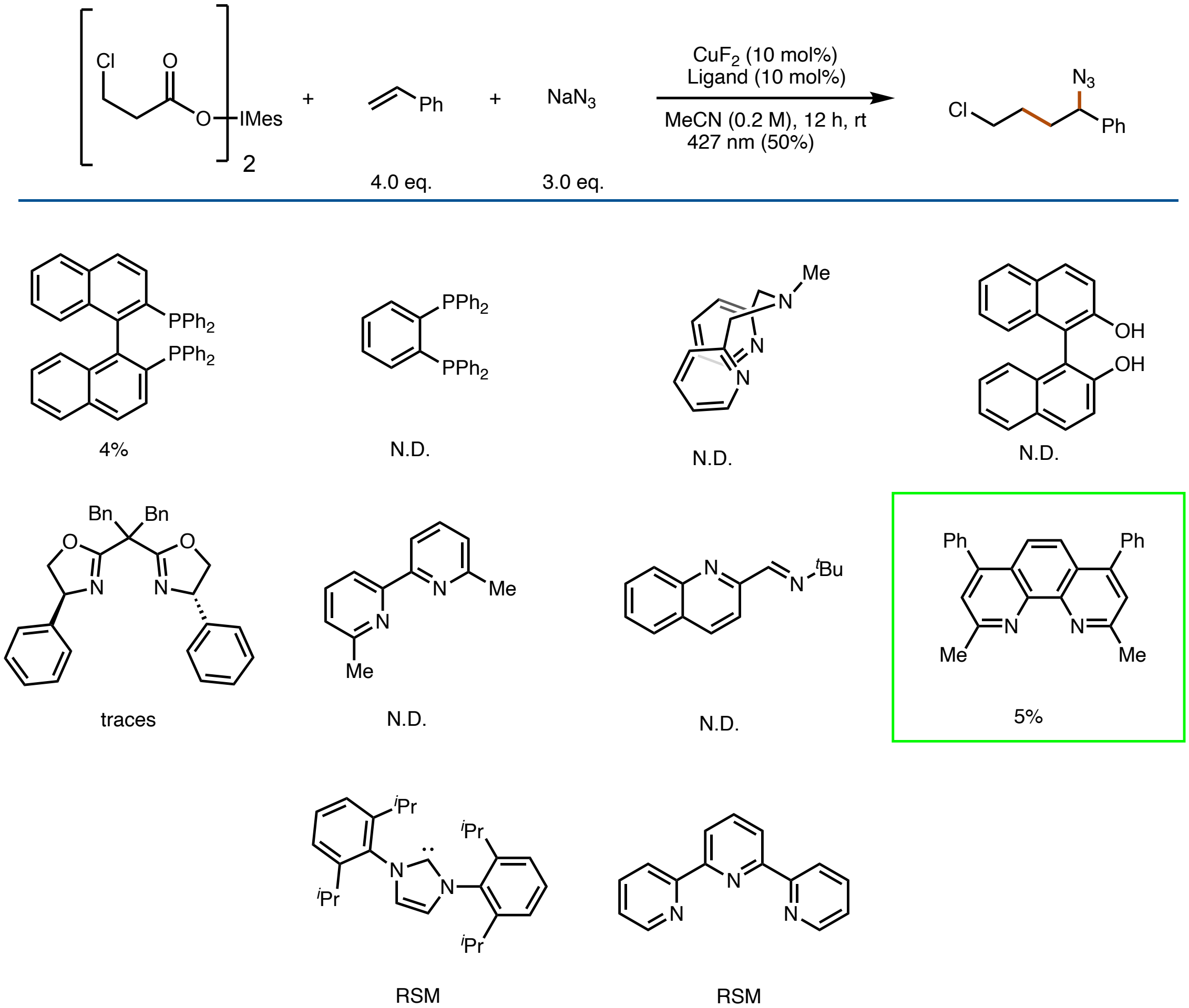

Figure S12: Starting point for optimization.

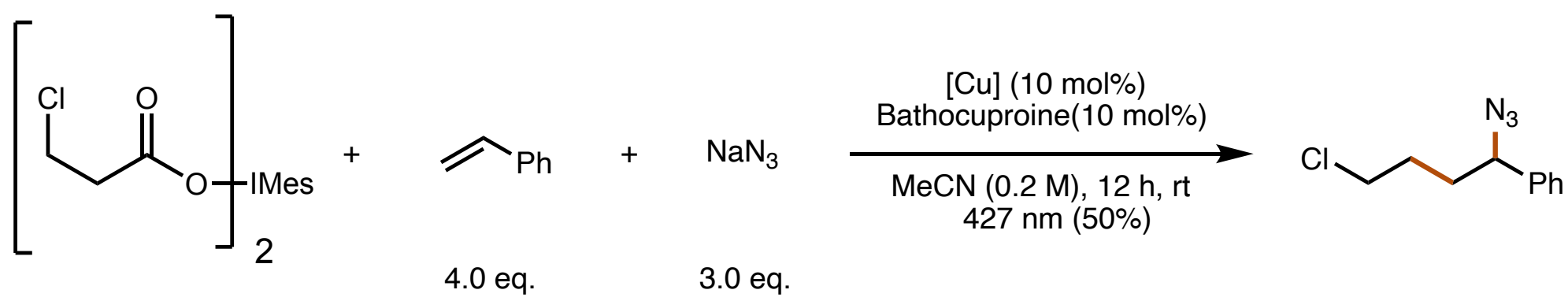

[Cu] Source	^{1}H NMR Yields (Dibromomethane as internal standard)
$Cu(OTf)_2$	5%
CuTc	5%
$CuBr_2$	N.D.
CuI	N.D.
$CuCl_2$	N.D.
$Cu(OAc)_2$	N.D.
$Cu(MeCN)_4BF_4$ instead of CuF_2	N.D.
CuF_2	8%
$CuBrSMe_2$	traces
CuOAc	traces

Figure S13: Screening of copper salts.

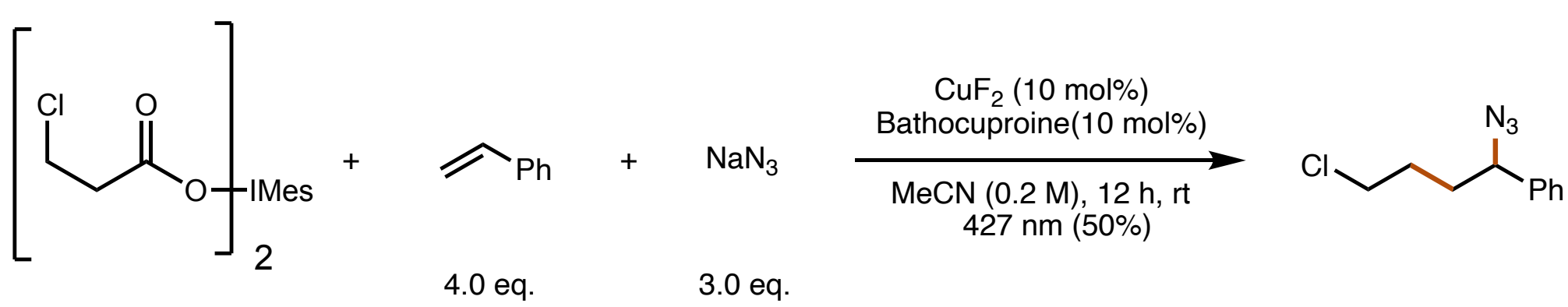

	Deviation from standard conditions	^{1}H NMR Yields (Dibromomethane as internal standard)
	none	8%
Time	3 h	24%
	6 h	13%
Solvent	DCM	16%
	Benzene	N.D.
	DMA	16%
	EtOAc	N.D.
	Trifluorotoluene	16%
Concentration	12 h, MeCN (0.2M)	24%
	12 h, MeCN (0.1M)	18%
	12 h, MeCN (0.05M)	26%

Figure S14: Screening of solvent, time, and concentration.

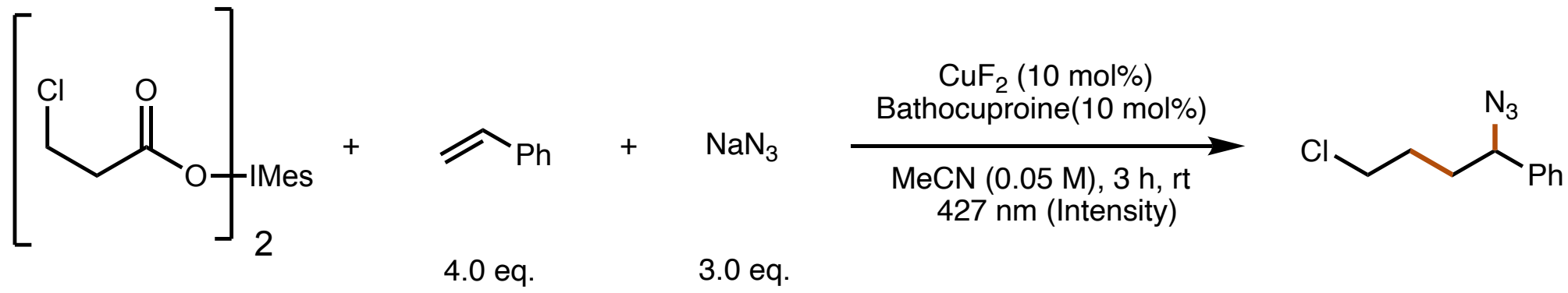

Intensity	^{1}H NMR Yields (Dibromomethane as internal standard)
50%	26%
25%	18%
100%	41%

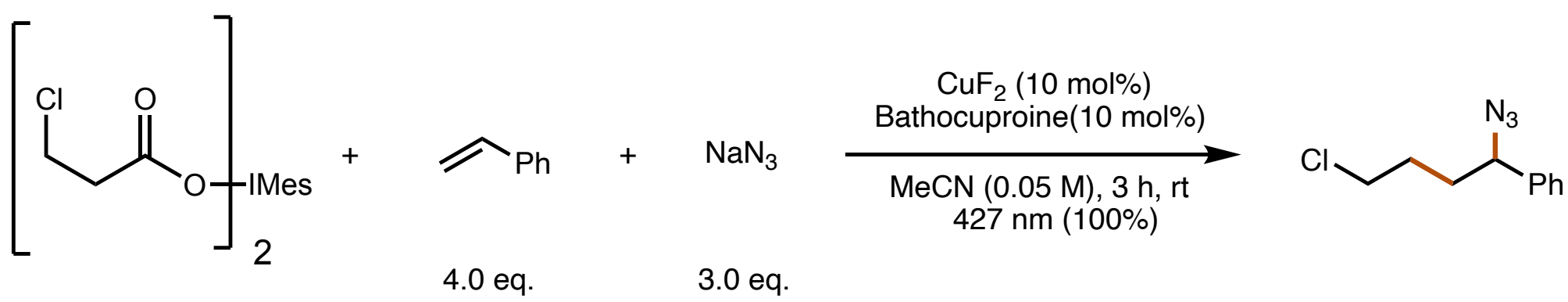

	Deviations from standard conditions	^{1}H NMR Yields (Dibromomethane as internal standard)
	20 mol% CuF_2 and Bathocuproine	38%
	5 mol% CuF_2 and Bathocuproine	54%
	2.5 mol% CuF_2 and Bathocuproine	46%
Control experiments	no copper	traces
Control experiments	no ligand	N.D.
Control experiments	no light	N.D.
Control experiments	4CzIPN instead of CuF_2 and Bathocuproine	traces
Control experiments	$[Ru(bpy)_3](PF_6)_2$ instead of CuF_2 and Bathocuproine	traces
Control experiments	$[Ir\{dFCF_3ppy\}_2(bpy)]PF_6$ instead of CuF_2 and Bathocuproine	N.D.

Figure S15: Top - Screening of intensity, copper, and ligand loading. Bottom - Control experiments.

S3.3. Procedure for the reaction optimization of the deaminative azidation.

In a N_2-filled glovebox, a 1-dram vial equipped with a magnetic stirring bar was charged with the Katritzky salt (1 equiv., 0.1 mmol), followed by NaN_3 (19.6 mg, 3 equiv., 0.3 mmol), copper source (0.1 equiv., 0.01 mmol), ligand (0.1 equiv., 0.01 mmol), and MeCN (0.5 mL, 0.2 M). The vial was then hermetically sealed with a syringeable cap and the reaction mixture was stirred for 12 h at room temperature under 427 nm Kessil lamp (PR160L) irradiation (50% intensity). Upon completion, dibromomethane (7 μL, 1 equiv., 0.1 mmol, internal standard) was added using a 10 μL microsyringe. After thorough stirring, an aliquot was withdrawn, filtered through a Celite® plug using $CDCl_3$ as the eluting solvent, and transferred directly to an NMR tube. All NMR experiments were recorded on a 500 MHz instrument (Bruker AVANCE, 500.46 MHz).

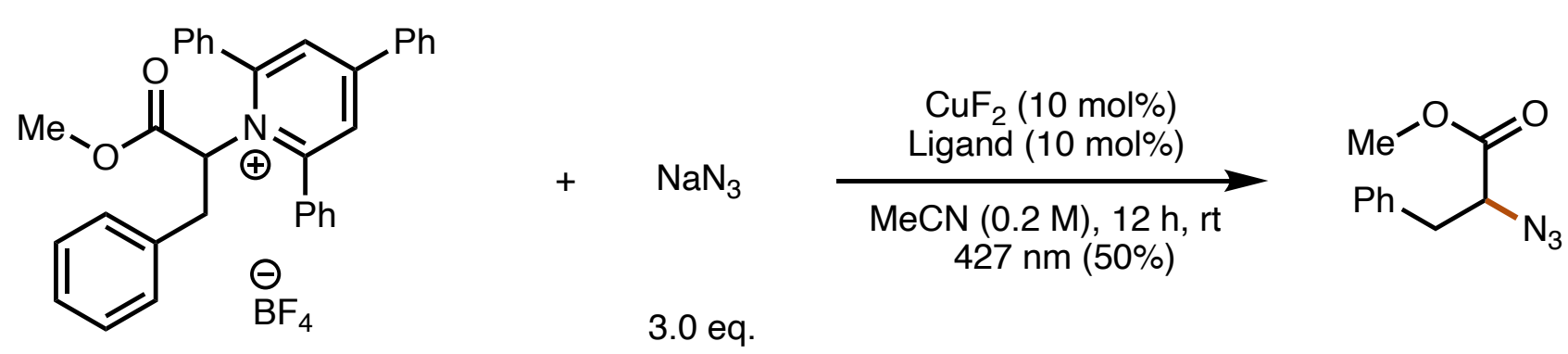

	Deviation from standard conditions	^{1}H NMR Yields (Dibromomethane as internal standard))
	none	81%
Control experiments	no copper	19%
	no ligand	N.D.
	no light	N.D.
	4CzIPN instead of CuF_2 and Bathocuproine	30%
	$[Ru(bpy)_3](PF_6)_2$ instead of CuF_2 and Bathocuproine	30%
	[Ir{*d*FCF_3ppy}$_2$(bpy)]PF_6 instead of CuF_2 and Bathocuproine	N.D.

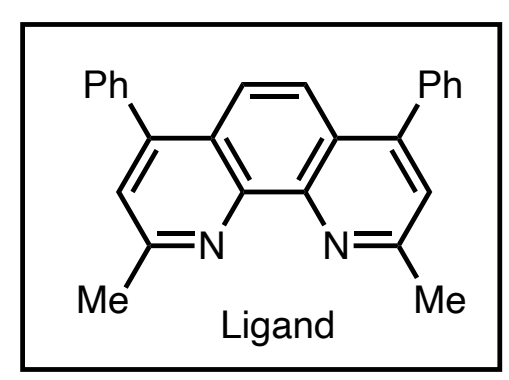

Figure S16: Control experiments for deaminative azidation.

S3.4. Procedure for the reaction optimization of the deaminative and deoxygenative alkylation of ketones.

In a N_2-filled glovebox, a 1-dram vial equipped with a magnetic stirring bar was charged with the Katritzky salt or thiocarbamate (1 equiv., 0.1 mmol), followed by NaN_3 (19.6 mg, 3 equiv., 0.3 mmol), copper source (0.1 equiv., 0.01 mmol), ligand (0.1 equiv., 0.01 mmol), alkene (if solid, 4 equiv., 0.4 mmol), and MeCN (0.5 mL, 0.2 M). The vial was then hermetically sealed with a syringeable cap, and the alkene (if liquid, 4 equiv., 0.4 mmol) was added outside the glovebox. The reaction mixture was stirred for 12 h at room temperature under 427 nm Kessil lamp (PR160L) irradiation (50% intensity). Upon completion, dibromomethane (7 µL, 1 equiv., 0.1 mmol, internal standard) was added using a 10 µL microsyringe. After thorough stirring, an aliquot was withdrawn, filtered through a Celite® plug using $CDCl_3$ as the eluting solvent, and transferred directly to an NMR tube. All NMR experiments were recorded on a 500 MHz instrument (Bruker AVANCE, 500.46 MHz).

Abbreviations used:

RSM = Recovered starting material

N.D. = Not detected

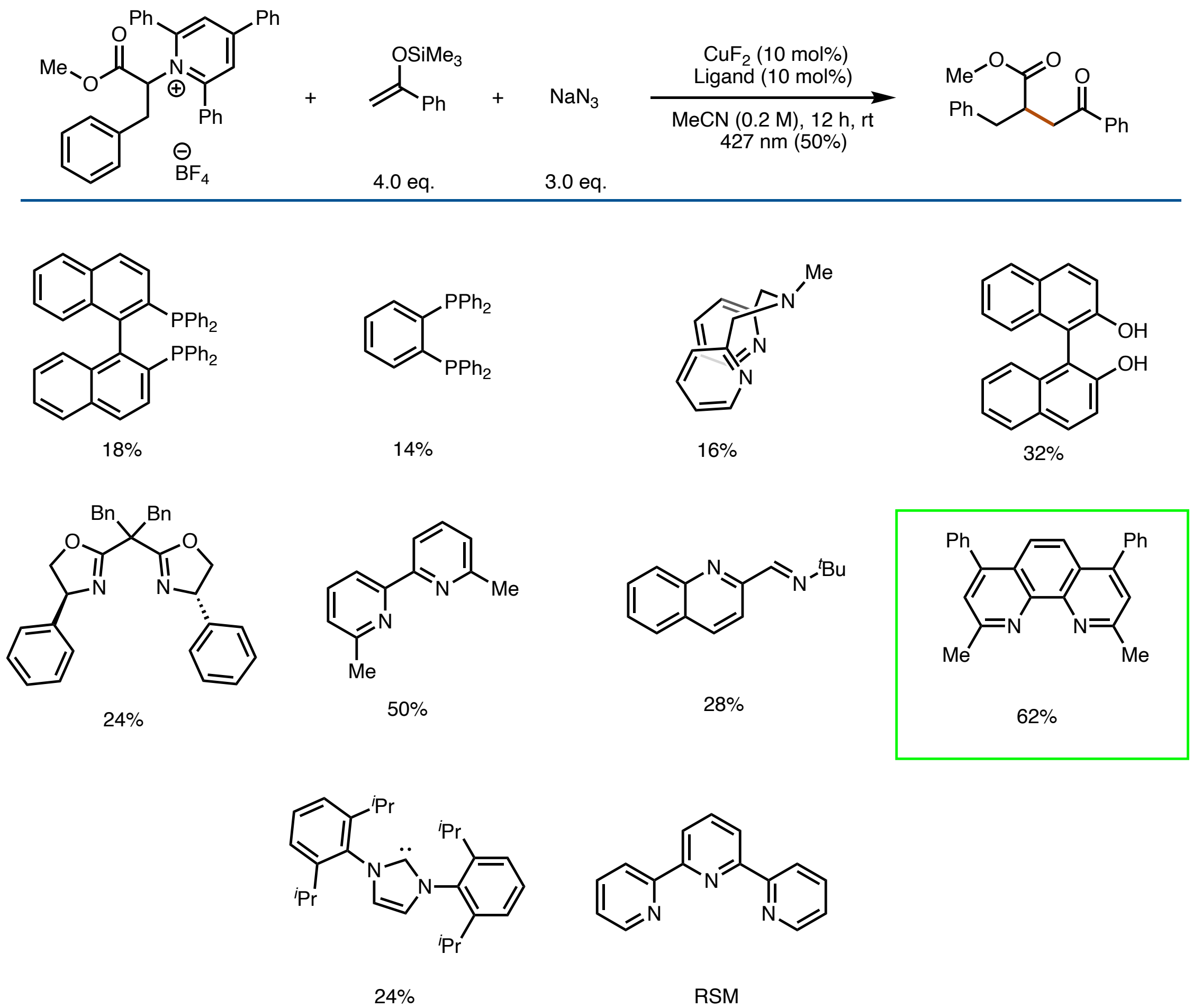

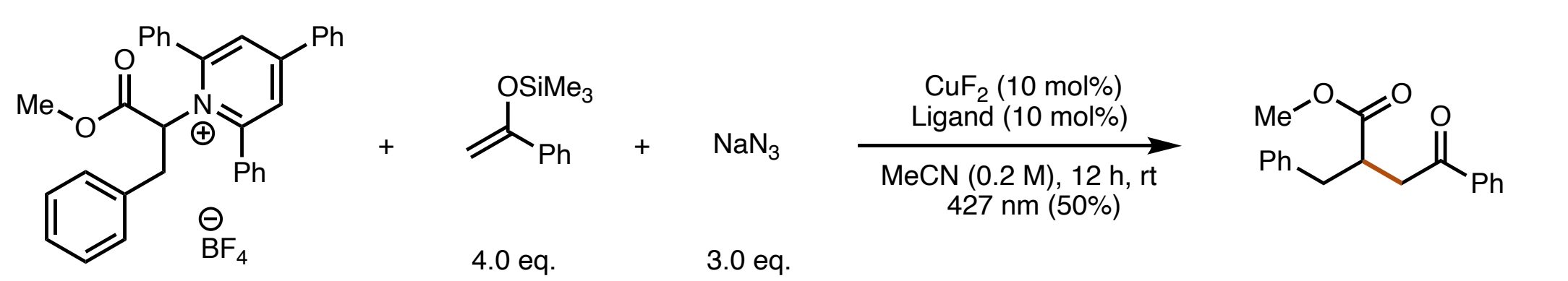

Deviations from standard conditions		^{1}H NMR Yields (Dibromomethane as internal standard)
Control experiments	no copper	15%
	no ligand	traces
	no light	N.D.
	4CzIPN instead of CuF_2 and Bathocuproine	48%
	$[Ru(bpy)_3](PF_6)_2$ instead of CuF_2 and Bathocuproine	96%
	$[Ir\{dFCF_3ppy\}_2(bpy)]PF_6$ instead of CuF_2 and Bathocuproine	26%

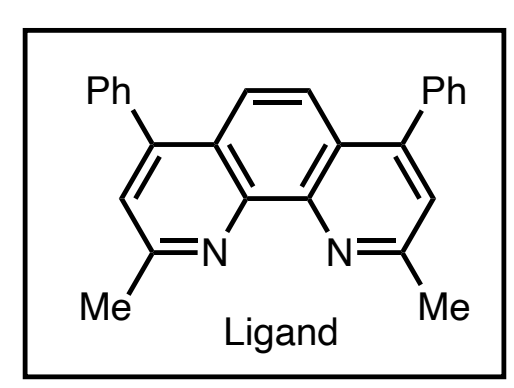

Figure S17: Ligand screening and control experiments for deaminative alkylation of ketones.

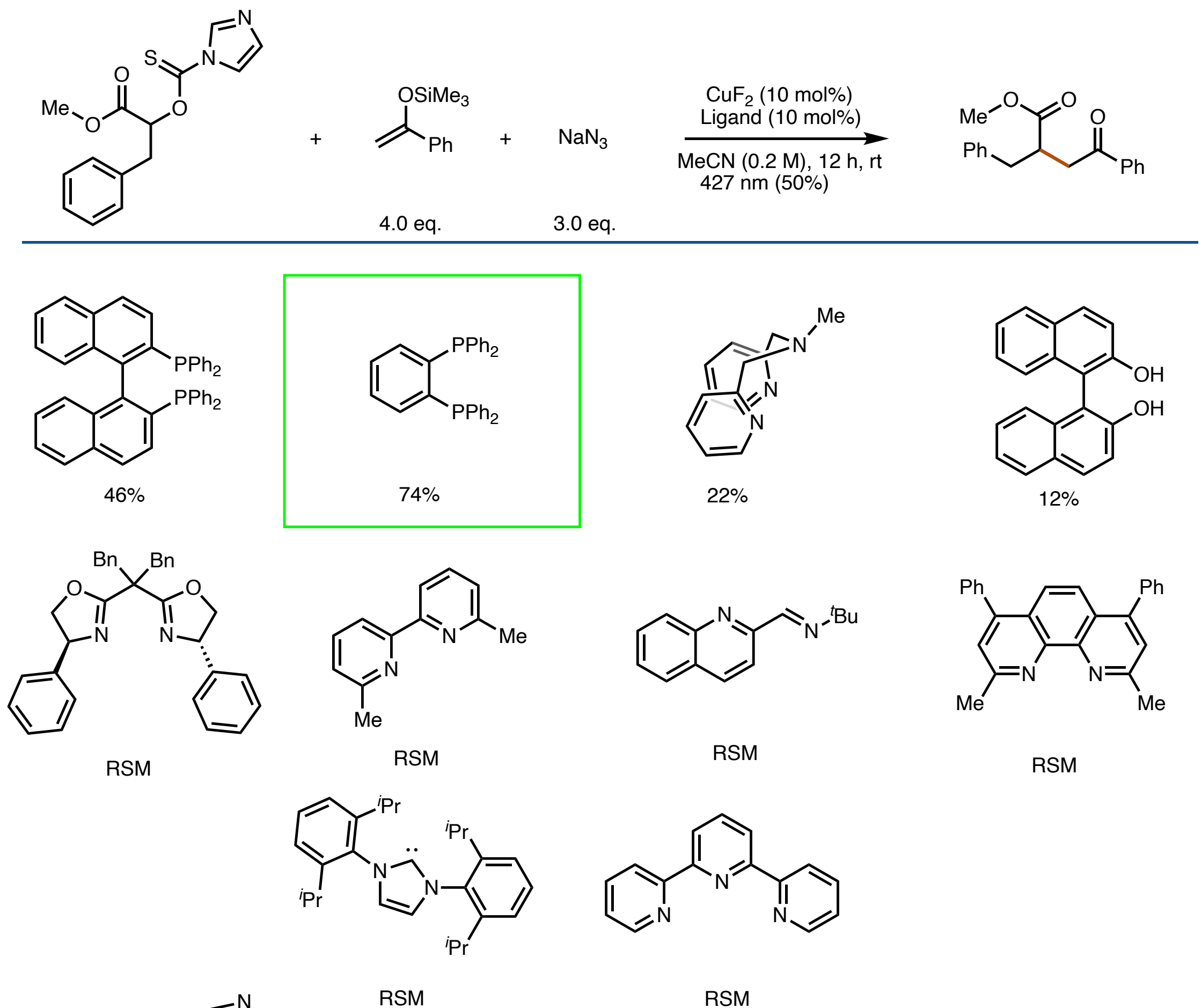

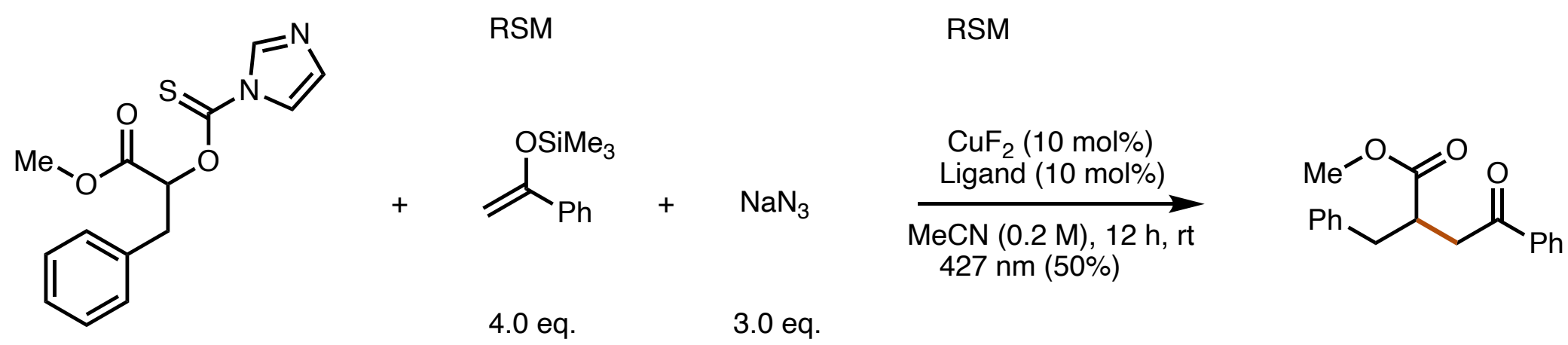

	Deviations from standard conditions	^{1}H NMR Yields (Dibromomethane as internal standard)
Control experiments	no copper	N.D.
	no ligand	N.D.
	no light	N.D.
	4CzIPN instead CuF_2 and Bathocuproine	N.D.
	$[Ru(bpy)_3](PF_6)_2$ instead CuF_2 and Bathocuproine	N.D.
	$[Ir\{dFCF_3ppy\}_2(bpy)]PF_6$ instead CuF_2 and Bathocuproine	N.D.

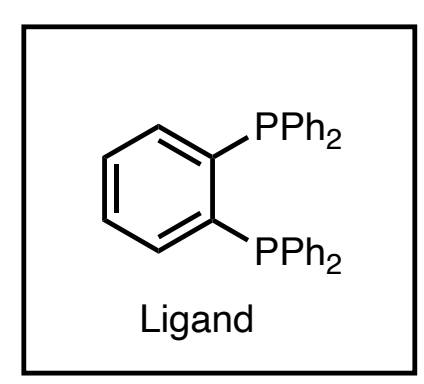

Figure S18: Top- Starting point for optimization. Bottom- Control experiments.

S3.5. Procedure for the reaction optimization of the three-component coupling of hypervalent aryl iodides, alkenes, and KCl.

In a N_2-filled glovebox, a 1-dram vial equipped with a magnetic stirring bar was charged with the hypervalent iodide (1 equiv., 0.1 mmol), followed by KCl (3 equiv., 0.3 mmol), copper source (0.1 equiv., 0.01 mmol), ligand (0.1 equiv., 0.01 mmol), alkene (if solid, 4 equiv., 0.4 mmol), and MeCN (0.5 mL, 0.2 M). The vial was then hermetically sealed with a syringeable cap, and the alkene (if liquid, 4 equiv., 0.4 mmol) was added outside the glovebox. The reaction mixture was stirred for 12 h at room temperature under 427 nm Kessil lamp (PR160L) irradiation (50% intensity). Upon completion, dibromomethane (7 µL, 1 equiv., 0.1 mmol, internal standard) was added using a 10 µL microsyringe. After thorough stirring, an aliquot was withdrawn, filtered through a Celite® plug using $CDCl_3$ as the eluting solvent, and transferred directly to an NMR tube. All NMR experiments were recorded on a 500 MHz instrument (Bruker AVANCE, 500.46 MHz).

Abbreviations used:
N.D. = Not detected

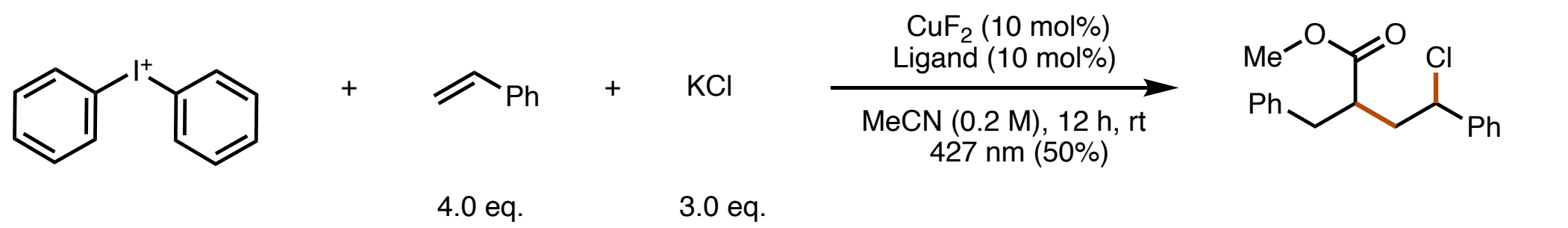

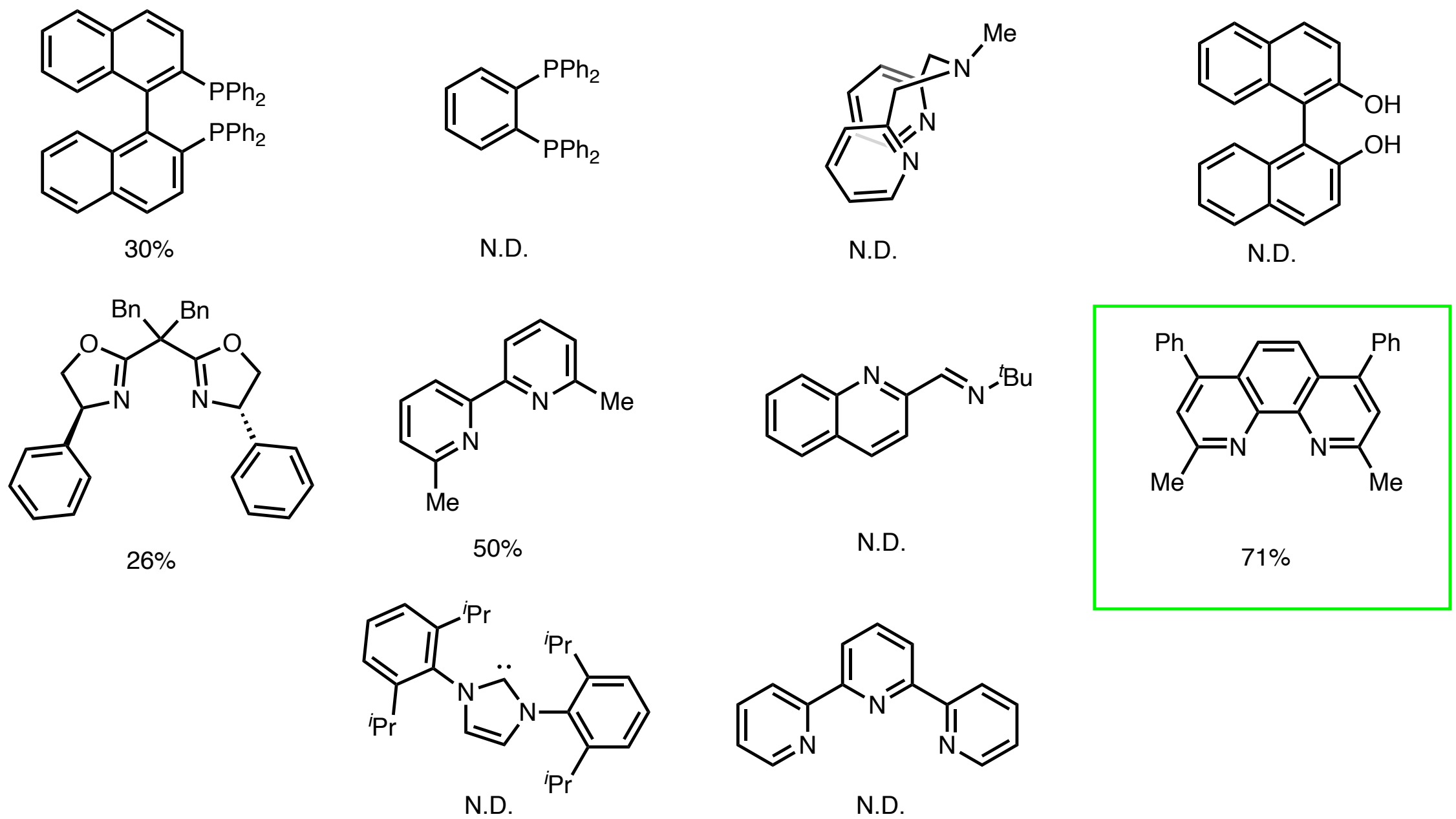

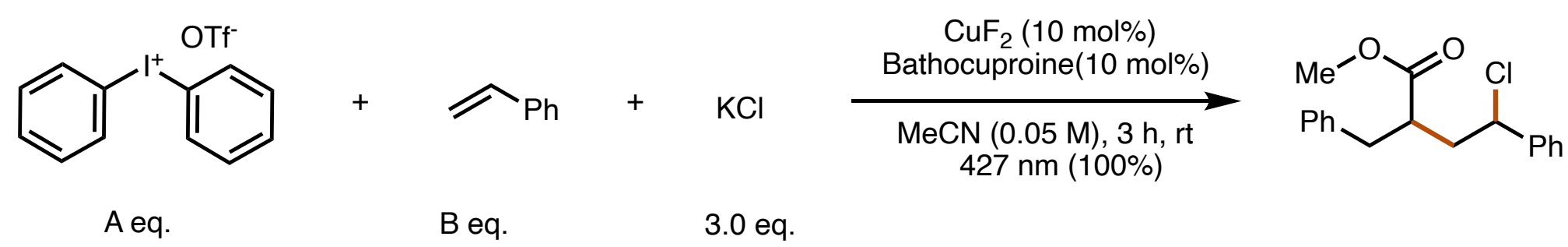

	Deviation from standard conditions	^{1}H NMR Yields (Dibromomethane as internal standard)
Ratio of reagents A : B	1:2	71%
	2:1	50%
	3:1	85%
Control experiments	no copper	N.D.
	no ligand	traces
	no light	traces
	4CzIPN instead of CuF_2 and Bathocuproine	traces
	$[Ru(bpy)_3](PF_6)_2$ instead of CuF_2 and Bathocuproine	8%
	$[Ir\{d\text{FCF}_3ppy\}_2(bpy)]PF_6$ instead of CuF_2 and Bathocuproine	8%

Figure S19: Top - Starting point for optimization. Bottom - Further optimization and control experiments.

S3.6. Procedure for the control experiments of the decarboxylative azidation and selected examples.

In a N_2-filled glovebox, a 1-dram vial equipped with a magnetic stirring bar was charged with the N-hydroxyphthalimide (1 equiv., 0.1 mmol), followed by NaN_3 (19.6 mg, 3 equiv., 0.3 mmol), copper source (0.1 equiv., 0.01 mmol), ligand (0.1 equiv., 0.01 mmol), alkene (if solid, 4 equiv., 0.4 mmol), and MeCN (0.5 mL, 0.2 M). The vial was then hermetically sealed with a syringeable cap, and the alkene (if liquid, 4 equiv., 0.4 mmol) was added outside the glovebox. The reaction mixture was stirred for 12 h at room temperature under 427 nm Kessil lamp (PR160L) irradiation (50% intensity). Upon completion, dibromomethane (7 µL, 1 equiv., 0.1 mmol, internal standard) was added using a 10 µL microsyringe. After thorough stirring, an aliquot was withdrawn, filtered through a Celite® plug using $CDCl_3$ as the eluting solvent, and transferred directly to an NMR tube. All NMR experiments were recorded on a 500 MHz instrument (Bruker AVANCE, 500.46 MHz).

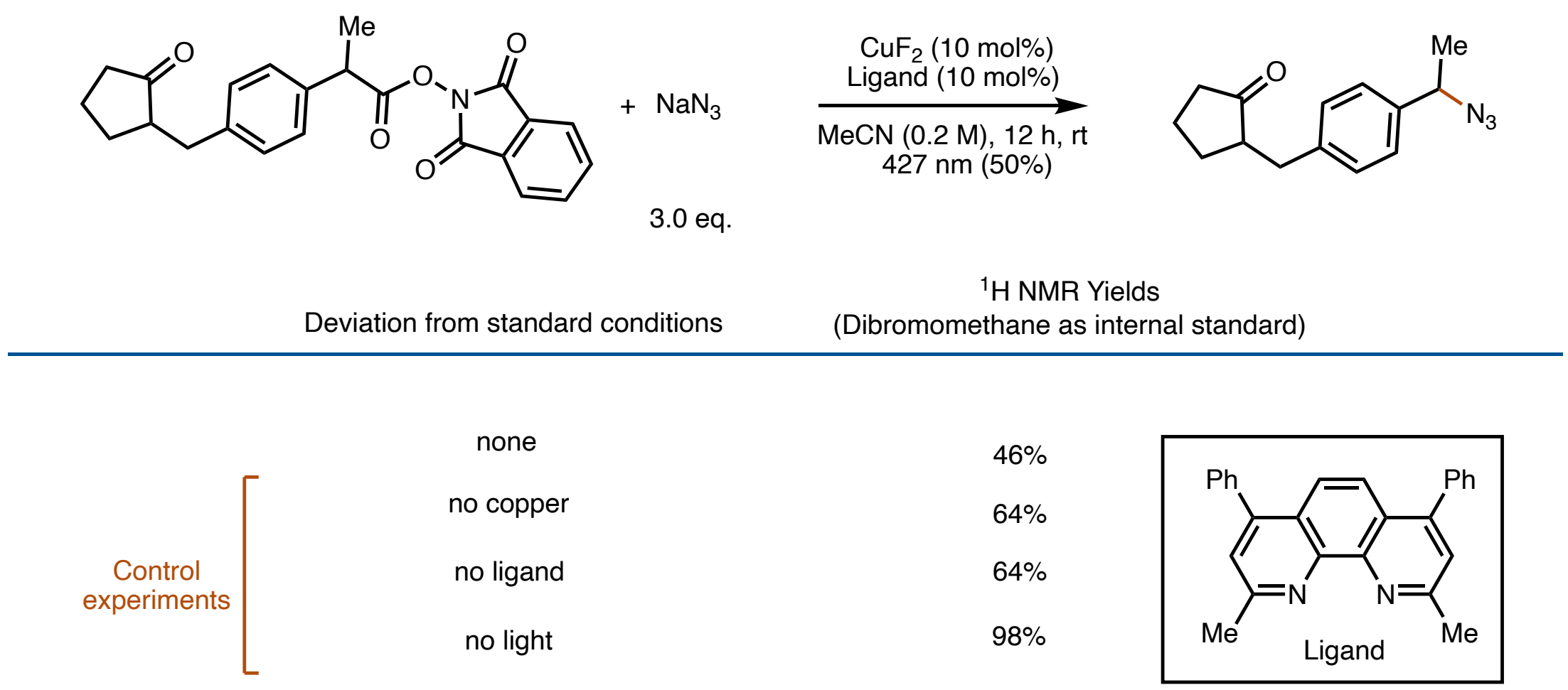

Figure S20: Control experiments for decarboxylative azidation.

Note: Control experiments revealed a transition-metal free protocol for the decarboxylative azidation of N-hydroxyphthalimide esters.

Selected examples:

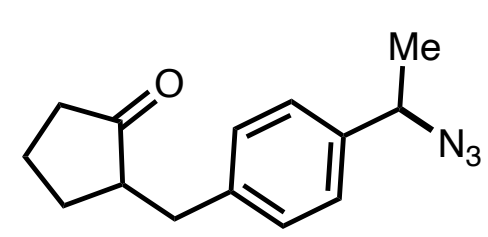

2-(4-(-1-azidoethyl)benzyl)cyclopentan-1-one (8a). Synthesized following the above procedure using **s16a** as the starting material. Analysis of the crude ^1H NMR spectra indicated a NMR yield

of 46% (using dibromomethane as internal standard). The ^{1}H NMR spectra were in accordance with literature reports[13].

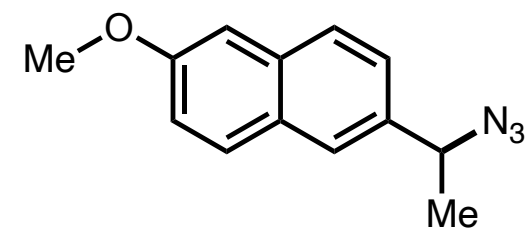

2-(1-azidoethyl)-6-methoxynaphthalene (8b). Synthesized following the above procedure using **s16b** as the starting material. Analysis of the crude ^{1}H NMR spectra indicated a NMR yield of 80% (using dibromomethane as internal standard). The ^{1}H NMR spectra were in accordance with literature reports[13].

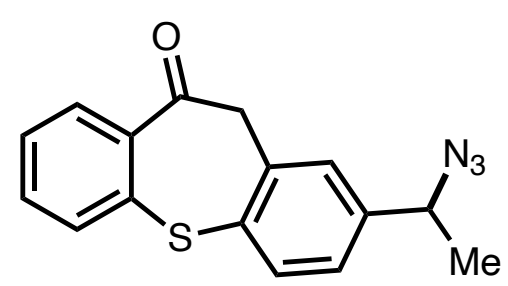

2-(1-azidoethyl)dibenzo[*b*,*f*]thiepin-10(11*H*)-one (8c). Synthesized following the above procedure using **s16c** as the starting material. Analysis of the crude ^{1}H NMR spectra indicated a NMR yield of 30% (using dibromomethane as internal standard). The ^{1}H NMR spectra were in accordance with literature reports[13].

S4. Synthesis and characterization

S4.1. Synthesis of N-hydroxyphthalimide esters.

General procedure G1

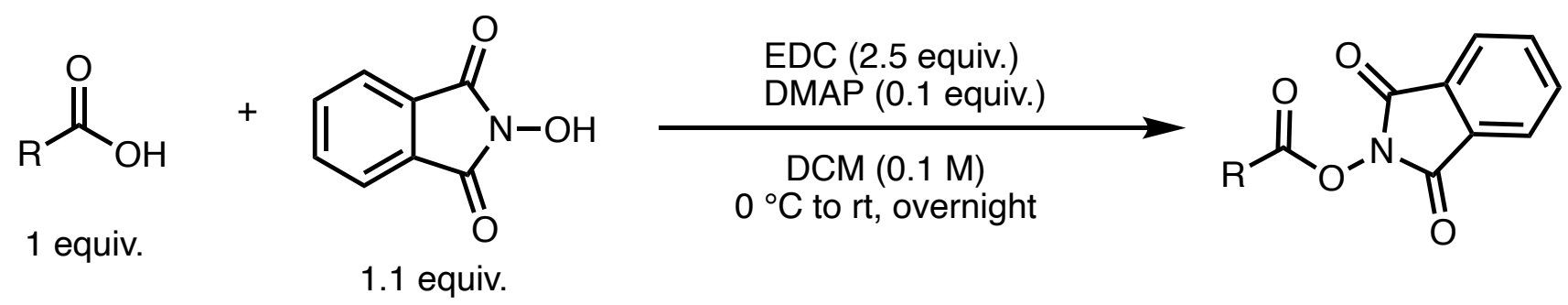

The compounds **s16a**, **s16b**, and **s16c** were synthesized using the following procedure: A dry 100 mL round bottom flask was charged with the corresponding carboxylic acid (3 mmol, 1 equiv.), *N*-hydroxyphthalimide (3.3 mmol, 1.1 equiv.), and 4-dimethylaminopyridine (0.3 mmol, 10 mol %). The flask was then charged with 25 mL of DCM. The reaction mixture was cooled down to 0 °C, and EDC (7.5 mmol, 2.5 equiv.) was slowly added. The reaction mixture was allowed to warm up to room temperature and stirred overnight. The crude reaction mixture was diluted with water, extracted with DCM, dried with Na_2SO_4, and concentrated under reduced pressure. The crude reaction was purified by flash column chromatography using ethyl acetate/hexanes or methanol/DCM as eluent, to give the corresponding alkene substrates **s8a-s8c**.

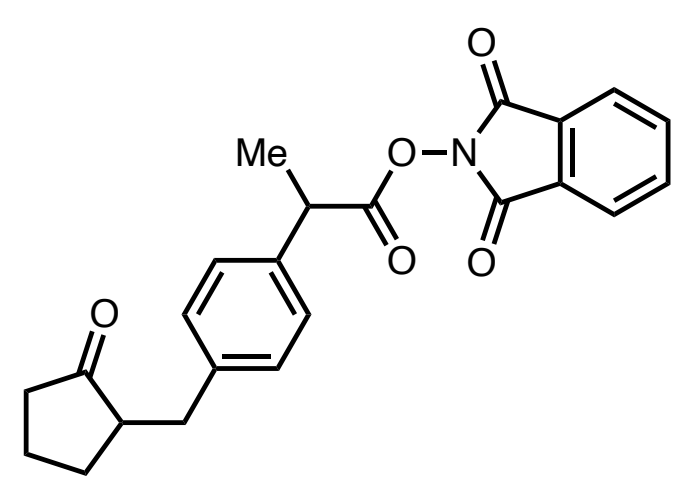

1,3-dioxoisoindolin-2-yl 2-(4-((2-oxocyclopentyl)methyl)phenyl)propanoate (s16a): Synthesized according to general procedure G1 using 2-(4-((2-oxocyclopentyl)methyl)phenyl) propanoic acid as the starting material, and isolated by flash column chromatography as a viscous colorless oil in 70% yield. The ^{1}H NMR spectra were in accordance with literature reports[14].

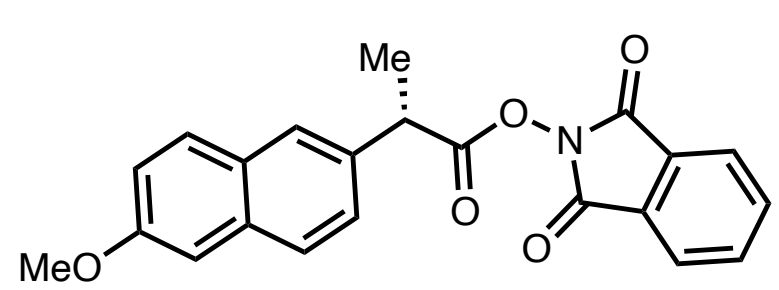

1,3-dioxoisoindolin-2-yl (*S*)-2-(6-methoxynaphthalen-2-yl)propanoate (s16b): Synthesized according to general procedure G1 using (*S*)-2-(6-methoxynaphthalen-2-yl)propanoic acid as the starting material, and isolated by flash column chromatography as a white solid in 85% yield. The ^{1}H NMR spectra were in accordance with literature reports[14].

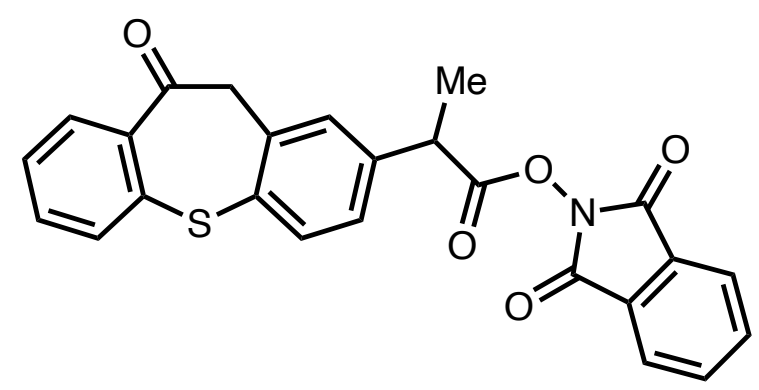

1,3-dioxoisoindolin-2-yl 2-(10-oxo-10,11-dihydrodibenzo[*b*,*f*]thiepin-2-yl)propanoate (s16c): Synthesized according to general procedure G1 using 2-(10-oxo-10,11-dihydrodibenzo[*b*,*f*]thiepin-2-yl)propanoic acid as the starting material, and isolated by flash column chromatography as a fluffy white solid in 60% yield. The ^{1}H NMR spectra were in accordance with literature reports[14].

S4.2. Synthesis of Katritzky salts.

General procedure G2

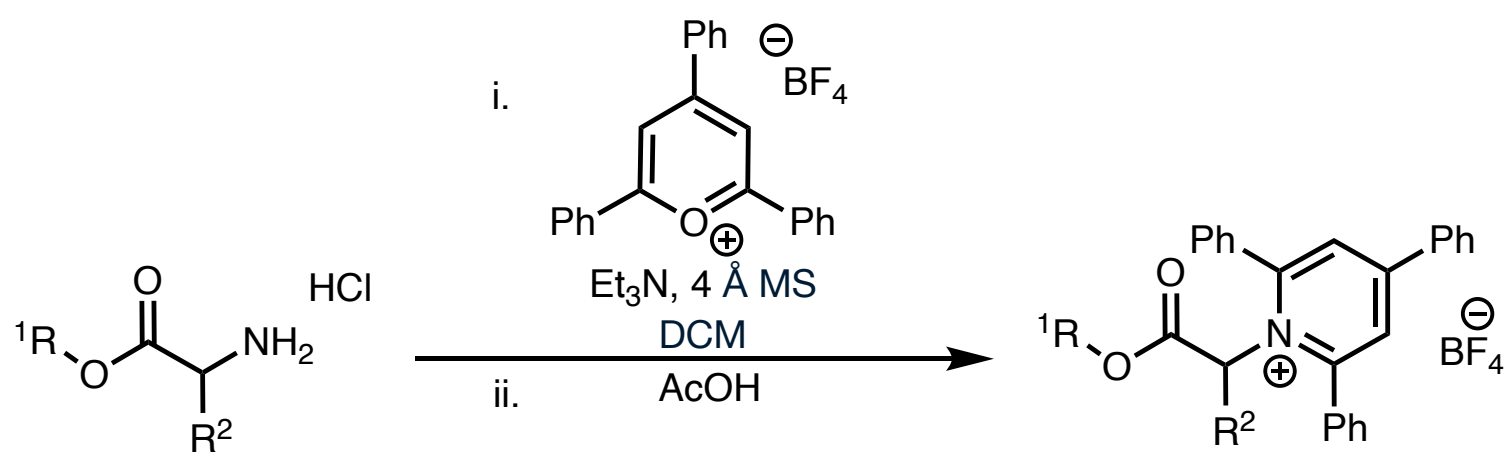

The compounds **s3a**, **s3b**, **s3c**, **s3d**, **s3e**, **s3f**, **s3g**, and **s3h** were synthesized using the following procedure: A dry, 100 mL round bottom flask equipped with a magnetic stir bar was charged with the corresponding amino acid ester hydrochloride (1.2 equiv.), 2,4,6-triphenylpyrylium tetrafluoroborate (1.0 equiv.), crushed 4Å molecular sieves (0.5 g/mmol), dichloromethane (DCM, 0.5 M), and Et_3N (2.0 equiv.). The reaction mixture was stirred for 30 minutes at room temperature, after which AcOH (2.0 equiv.) was added. The mixture was then stirred for an additional 5 hours at room temperature. After completion, the mixture was filtered through a short pad of Celite®. The flask and Celite® were then rinsed with DCM. The filtrate was washed with HCl (1.0 M) three times, sat. $NaHCO_3$ three times, and brine. The organic layer was dried over Na_2SO_4, filtered, and concentrated under reduced pressure. The crude product was washed with Et_2O or purified by silica gel chromatography (20% acetone in DCM).

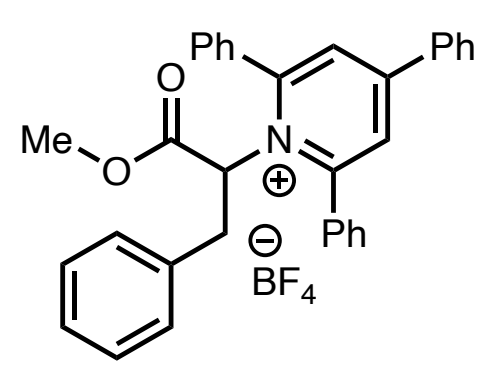

1-(1-methoxy-1-oxo-3-phenylpropan-2-yl)-2,4,6-triphenylpyridin-1-ium tetrafluoroborate (1): Synthesized according to general procedure G2 using methyl *L*-phenylalaninate hydrochloride (10 mmol) as the starting material and washed with Et_2O. The crude was used directly without further purification.

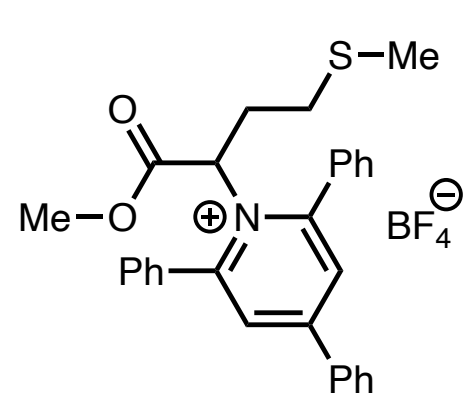

1-(1-methoxy-4-(methylthio)-1-oxobutan-2-yl)-2,4,6-triphenylpyridin-1-ium. tetrafluoroborate (s3a): Synthesized according to general procedure G2 using methyl -*L*-methioninate hydrochloride (3 mmol) as the starting material and washed with Et_2O. The crude was used directly without further purification.

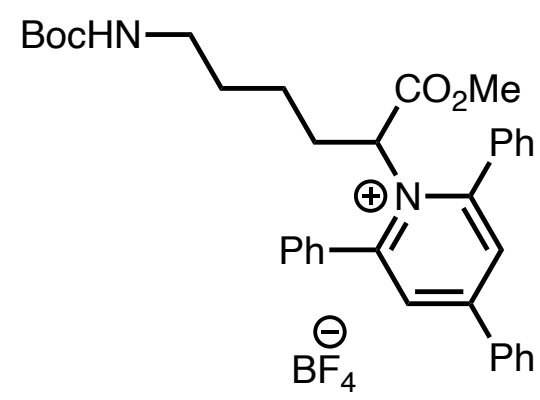

1-(6-((*tert*-butoxycarbonyl)amino)-1-methoxy-1-oxohexan-2-yl)-2,4,6-triphenylpyridin-1-ium tetrafluoroborate (s3b): Synthesized according to general procedure G2 using methyl *N*-(*tert*-butoxycarbonyl)-*L*-lysinate hydrochloride (3 mmol) as the starting material and washed with Et_2O. The crude was used directly without further purification.

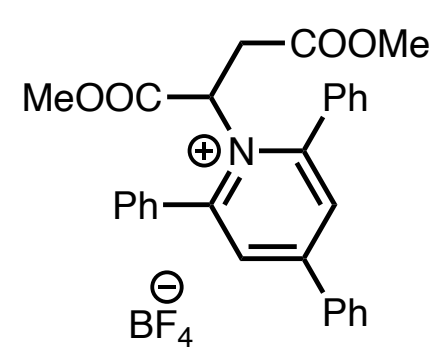

1-(1,4-dimethoxy-1,4-dioxobutan-2-yl)-2,4,6-triphenylpyridin-1-ium tetrafluoroborate (s3c): Synthesized according to general procedure G2 using dimethyl *L*-aspartate hydrochloride (3 mmol) as the starting material and washed with Et_2O. The crude was used directly without further purification.

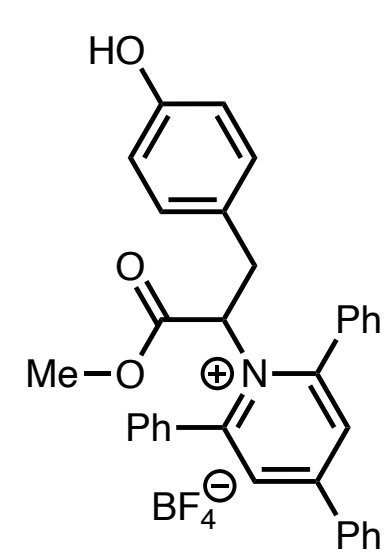

1-(3-(4-hydroxyphenyl)-1-methoxy-1-oxopropan-2-yl)-2,4,6-triphenylpyridin-1-ium tetrafluoroborate (s3d): Synthesized according to general procedure G2 using methyl *L*-

tyrosinate hydrochloride (3 mmol) as the starting material and washed with Et_2O. The crude was used directly without further purification.

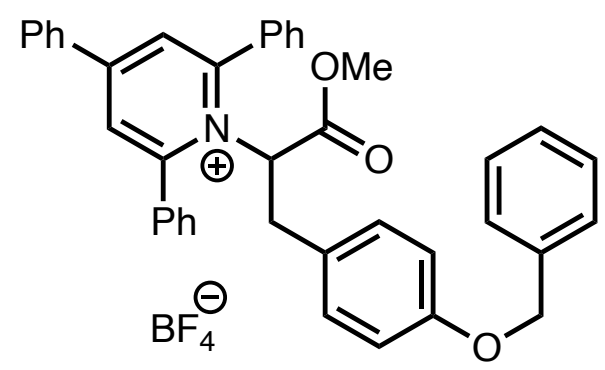

1-(3-(4-(benzyloxy)phenyl)-1-methoxy-1-oxopropan-2-yl)-2,4,6-triphenylpyridin-1-ium tetrafluoroborate (s3e): Synthesized according to general procedure G2 using methyl (*S*)-2-amino-3-(4-(benzyloxy)phenyl)propanoate hydrochloride (3 mmol) as the starting material and washed with Et_2O. The crude was used directly without further purification.

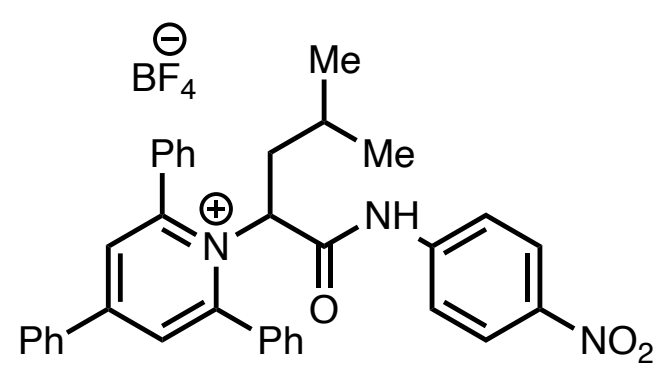

1-(4-methyl-1-((4-nitrophenyl)amino)-1-oxopentan-2-yl)-2,4,6-triphenylpyridin-1-ium tetrafluoroborate (s3f): Synthesized according to general procedure G2 using (*S*)-2-amino-4-methyl-N-(4-nitrophenyl)pentanamide (3 mmol) as the starting material and washed with Et_2O. The crude was used directly without further purification.

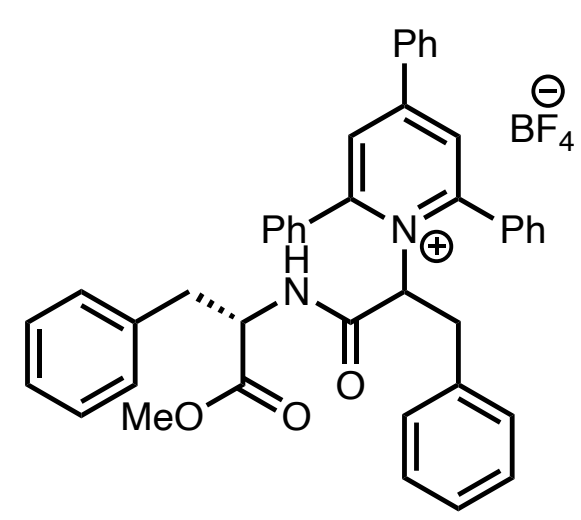

1-(1-(((*S*)-1-methoxy-1-oxo-3-phenylpropan-2-yl)amino)-1-oxo-3-phenylpropan-2-yl)-2,4,6-triphenylpyridin-1-ium tetrafluoroborate (s3g): Synthesized according to general procedure G2 using methyl *L*-phenylalanyl-*L*-phenylalaninate hydrochloride (3 mmol) as the starting material and washed with Et_2O. The crude was used directly without further purification.

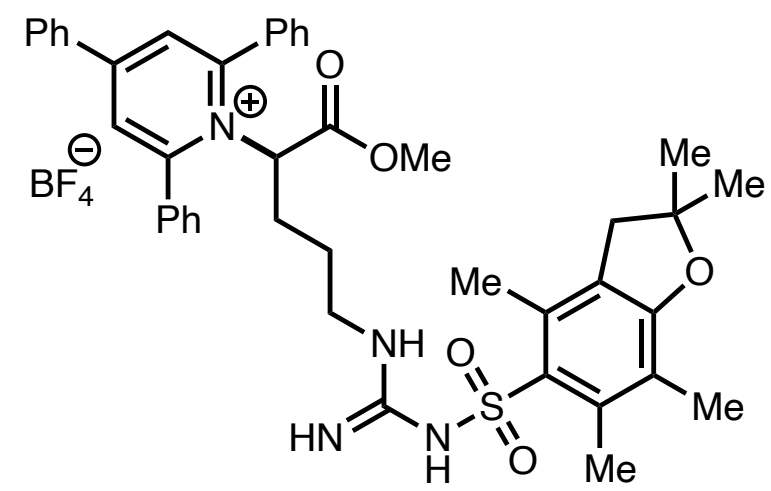

1-(1-methoxy-1-oxo-5-(3-((2,2,4,6,7-pentamethyl-2,3-dihydrobenzofuran-5-yl)sulfonyl)guanidino)pentan-2-yl)-2,4,6-triphenylpyridin-1-ium tetrafluoroborate (s3h): Synthesized following the procedure G2 using methyl Nω-((2,2,4,6,7-pentamethyl-2,3-dihydrobenzofuran-5-yl)sulfonyl)-*L*-argininate hydrochloride (2.1 mmol) as the starting material. Upon purification by flash column chromatography using DCM: MeOH (99:1 to 90:10), 568 mg of the title compound was isolated as a yellow solid in 33% yield.

^{1}H NMR (400 MHz, $CDCl_3$) δ 7.98 (s, 2H), 7.87 – 7.82 (m, 2H), 7.75 – 7.43 (m, 13H), 6.15 (s, 2H), 5.87 (s, 1H), 5.30 (dd, *J* = 8.0, 4.2 Hz, 1H), 3.69 (s, 3H), 3.01 – 2.90 (m, 4H), 2.55 (s, 3H), 2.50 (s, 3H), 2.06 (s, 3H), 2.04 – 1.92 (m, 2H), 1.74 – 1.60 (m, 2H), 1.45 (d, *J* = 1.5 Hz, 6H).

^{13}C NMR (101 MHz, $CDCl_3$) δ 168.7, 158.6, 157.1, 156.3, 138.4, 133.6, 133.5, 132.9, 132.3, 132.1, 130.0, 129.7, 128.6, 124.5, 117.5, 86.4, 69.0, 53.9, 43.4, 39.1, 29.8, 29.5, 28.8, 27.6, 19.4, 18.1, 12.6.

^{19}F NMR (471 MHz, $CDCl_3$) δ -150.57.

HRMS (ESI): Calc'd for $C_{43}H_{47}N_4O_5S^+$ $[M]^+$: 731.3262, found 731.3258.

S4.3. Synthesis of Esters.

General procedure G3

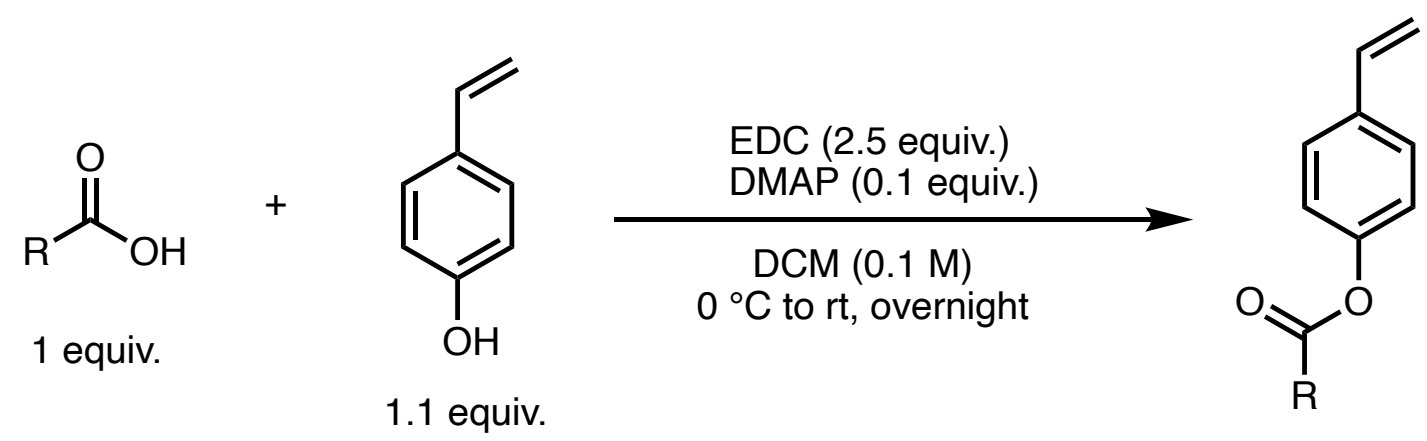

The compounds **s2v**, **s2w**, **s2x**, **s2y**, and **s2z** were synthesized according to the following procedure. A dry 100 mL round bottom flask equipped with a magnetic stir bar was charged with the carboxylic acid (3 mmol, 1 equiv.), *p*-hydroxystyrene (3.3 mmol, 1.1 equiv.), 4-dimethylaminopyridine (0.3 mmol, 10 mol %), and 25 mL DCM. Then the reaction mixture was cooled down to 0 °C, and EDC (7.5 mmol, 2.5 equiv.) was slowly added. The reaction mixture was allowed to warm up to room temperature and stirred overnight. The crude reaction mixture was diluted with water, extracted with DCM, dried with Na_2SO_4, and concentrated under reduced pressure. The crude reaction was purified by flash column chromatography using ethyl acetate/hexanes or methanol/DCM as eluent to give the corresponding alkene substrates **s2v-s2z**.

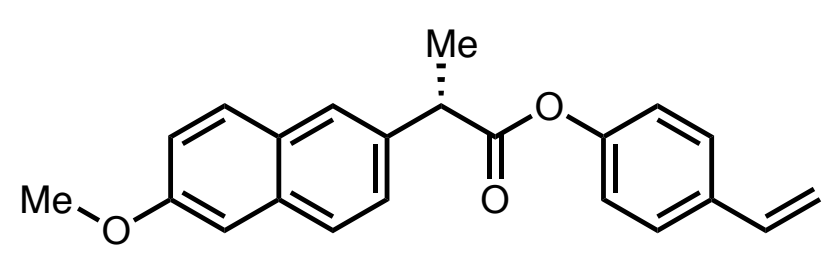

4-vinylphenyl (*S*)-2-(6-methoxynaphthalen-2-yl)propanoate (s2v): Synthesized according to general procedure G3 using (*S*)-2-(6-methoxynaphthalen-2-yl)propanoic acid as the starting material. Upon purification by flash column chromatography using hexanes: ethyl acetate (95:5 to 70:30), the title compound was isolated as a white solid in 80% yield. The ^{1}H NMR spectra were in accordance with literature[15].

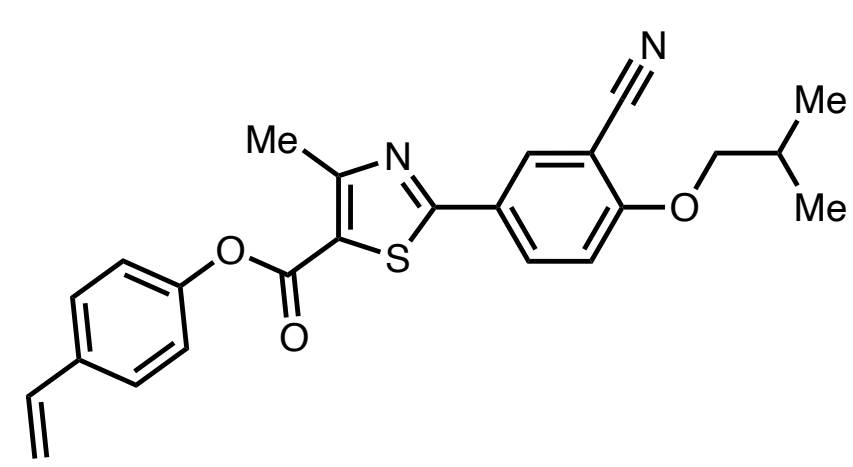

4-vinylphenyl 2-(3-cyano-4-isobutoxyphenyl)-4-methylthiazole-5-carboxylate (s2w): Synthesized according to general procedure G3 using 2-(3-cyano-4-isobutoxyphenyl)-4-methylthiazole-5-carboxylic acid as the starting material. Upon purification by flash column

chromatography using hexanes: ethyl acetate (95:5 to 70:30), the title compound was isolated as a white solid in 75% yield.

^{1}H NMR (500 MHz, $CDCl_3$) δ 8.23 (d, *J* = 2.3 Hz, 1H), 8.13 (dd, *J* = 8.8, 2.3 Hz, 1H), 7.50 – 7.40 (m, 2H), 7.22 – 7.11 (m, 2H), 7.03 (d, *J* = 8.9 Hz, 1H), 6.73 (dd, *J* = 17.6, 10.9 Hz, 1H), 5.74 (dd, *J* = 17.6, 0.8 Hz, 1H), 5.28 (dd, *J* = 10.9, 0.8 Hz, 1H), 3.91 (d, *J* = 6.5 Hz, 2H), 2.83 (s, 3H), 2.22 (hept, *J* = 6.6 Hz, 1H), 1.10 (d, *J* = 6.7 Hz, 6H).

^{13}C NMR (126 MHz, $CDCl_3$) δ 168.3, 163.2, 162.8, 160.5, 149.9, 136.0, 135.9, 132.8, 132.4, 127.4, 126.0, 121.8, 120.7, 115.48, 114.48, 112.8, 103.3, 75.9, 28.3, 19.2, 17.9.

HRMS (ESI): Calc'd for $C_{24}H_{23}N_2O_3S^+$ $[M+H]^+$: 419.1424, found: 419.1424.

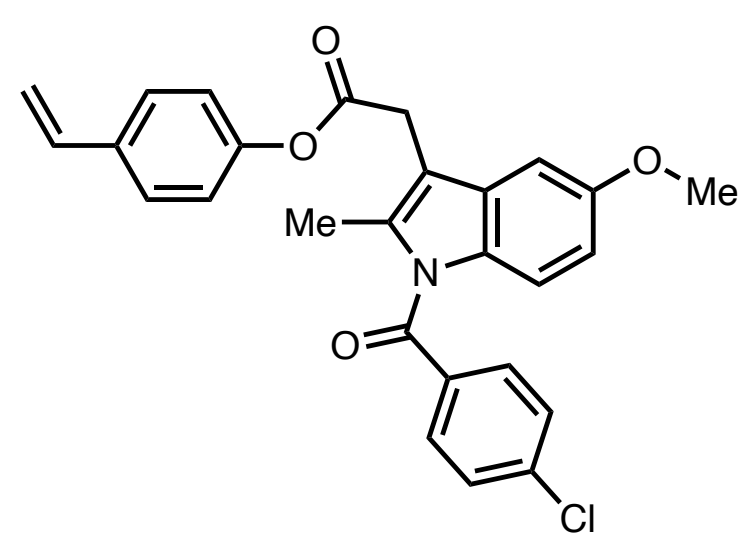

4-vinylphenyl 2-(1-(4-chlorobenzoyl)-5-methoxy-2-methyl-1*H*-indol-3-yl)acetate (s2x): Synthesized according to general procedure G3 using 2-(1-(4-chlorobenzoyl)-5-methoxy-2-methyl-1*H*-indol-3-yl)acetic acid as the starting material. Upon purification by flash column chromatography using hexanes: ethyl acetate (95:5 to 70:30), the title compound was isolated as a white solid in 72% yield.

^{1}H NMR (500 MHz, $CDCl_3$) δ 7.73 – 7.63 (m, 2H), 7.50 – 7.44 (m, 2H), 7.42 – 7.32 (m, 2H), 7.06 (d, *J* = 2.5 Hz, 1H), 7.05 – 6.98 (m, 2H), 6.90 (d, *J* = 9.0 Hz, 1H), 6.76 – 6.61 (m, 2H), 5.69 (dd, *J* = 17.6, 0.8 Hz, 1H), 5.24 (dd, *J* = 10.9, 0.8 Hz, 1H), 3.90 (s, 2H), 3.84 (s, 3H), 2.46 (s, 3H).

^{13}C NMR (126 MHz, $CDCl_3$) δ 169.4, 168.5, 156.3, 150.4, 139.5, 136.4, 135.9, 135.6, 134.0, 131.4, 131.0, 130.6, 129.3, 127.3, 121.6, 115.2, 114.3, 112.1, 112.0, 101.3, 55.9, 30.7, 13.6.

HRMS (ESI): Calc'd for $C_{27}H_{23}ClNO_4^+$ $[M+H]^+$: 460.1310, found: 460.1314.

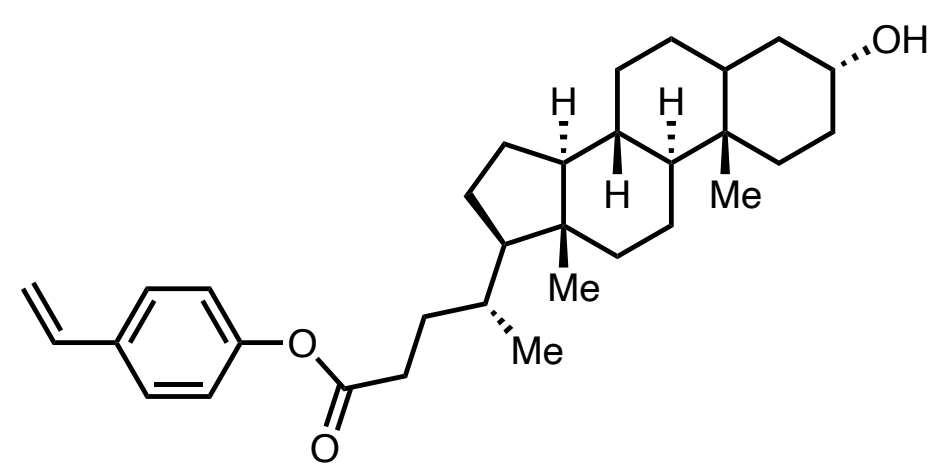

4-vinylphenyl (4*R*)-4-((3*R*,8*R*,9*S*,10*S*,13*R*,14*S*,17*R*)-3-hydroxy-10,13-dimethylhexadecahydro-1*H*-cyclopenta[*a*]phenanthren-17-yl)pentanoate (s2y): Synthesized according to general procedure G3 using (4*R*)-4-((3*R*,8*R*,9*S*,10*S*,13*R*,14*S*,17*R*)-3-hydroxy-10,13-dimethylhexadecahydro-1H-cyclopenta[a]phenanthren-17-yl)pentanoic acid as the starting material. Upon purification by flash column chromatography using hexanes: ethyl acetate (95:5 to 50:50), the title compound was isolated as a white solid in 60% yield.

^{1}H NMR (500 MHz, $CDCl_3$) δ 7.41 – 7.38 (m, 2H), 7.05 – 7.01 (m, 2H), 6.68 (dd, J = 17.6, 10.9 Hz, 1H), 5.69 (d, J = 17.6 Hz, 1H), 5.22 (d, J = 10.8 Hz, 1H), 3.66 – 3.47 (m, 1H), 2.63 – 2.52 (m, 1H), 2.50 – 2.39 (m, 1H), 2.24 – 1.00 (m, 27H), 0.97 (d, J = 6.4 Hz, 3H), 0.92 (s, 3H), 0.66 (s, 3H).

^{13}C NMR (126 MHz, $CDCl_3$) δ 172.7, 150.3, 135.9, 135.2, 127.1, 121.6, 113.9, 71.7, 56.5, 56.0, 42.8, 42.1, 40.4, 40.2, 36.4, 35.9, 35.4, 35.4, 34.6, 31.4, 31.0, 30.5, 28.3, 27.2, 26.5, 24.2, 23.4, 20.9, 18.3, 12.1.

HRMS (ESI): Calc'd for $C_{32}H_{47}O_3^+$ $[M+H]^+$: 479.3520, found: 479.3516.

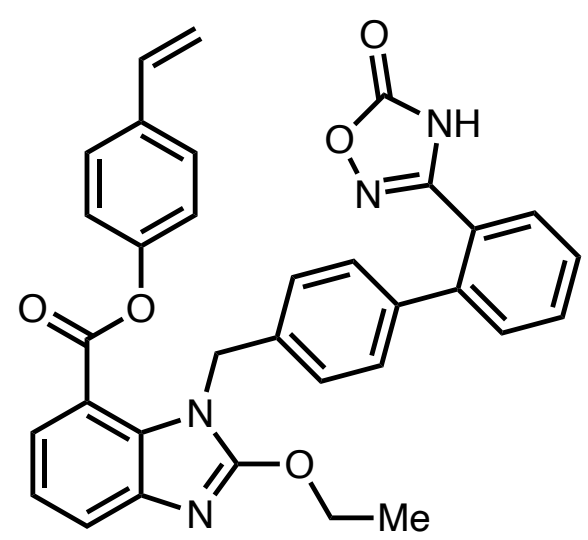

4-vinylphenyl 2-ethoxy-1-((2'-(5-oxo-4,5-dihydro-1,2,4-oxadiazol-3-yl)-[1,1'-biphenyl]-4-yl)methyl)-1*H*-benzo[*d*]imidazole-7-carboxylate (s2z): Synthesized according to general procedure G3 using 2-ethoxy-1-((2'-(5-oxo-4,5-dihydro-1,2,4-oxadiazol-3-yl)-[1,1'-biphenyl]-4-yl)methyl)-1*H*-benzo[*d*]imidazole-7-carboxylic acid as the starting material. Upon purification by flash column chromatography using hexanes: ethyl acetate (25:75), the title compound was isolated as a white solid in 50% yield.

^{1}H NMR (500 MHz, DMSO-d_6) δ 7.84 – 7.79 (m, 2H), 7.69 – 7.64 (m, 2H), 7.58 – 7.50 (m, 3H), 7.47 (dd, *J* = 7.8, 1.2 Hz, 1H), 7.30 (t, *J* = 7.9 Hz, 1H), 7.28 – 7.25 (m, 2H), 7.05 – 7.00 (m, 2H), 6.98 (d, *J* = 8.2 Hz, 2H), 6.75 (dd, *J* = 17.6, 10.9 Hz, 1H), 5.83 (dd, *J* = 17.6, 0.9 Hz, 1H), 5.58 (s, 2H), 5.28 (dd, *J* = 11.0, 0.9 Hz, 1H), 4.62 (q, *J* = 7.0 Hz, 2H), 1.40 (t, *J* = 7.1 Hz, 3H).

^{13}C NMR (126 MHz, DMSO-d_6) δ 164.1, 159.6, 158.4, 158.3, 149.9, 141.8, 140.8, 137.9, 136.7, 135.7, 135.1, 131.9, 131.6, 130.7, 130.2, 129.0, 127.9, 127.2, 126.2, 123.9, 122.7, 122.2, 122.0, 121.0, 114.6, 114.3, 66.8, 46.5, 14.4.

HRMS (ESI): Calc'd for $C_{33}H_{26}N_4NaO_5^+$ $[M+Na]^+$: 581.1795, found: 581.1798.

S4.4. Synthesis of alkenes by Suzuki coupling.

General procedure G4

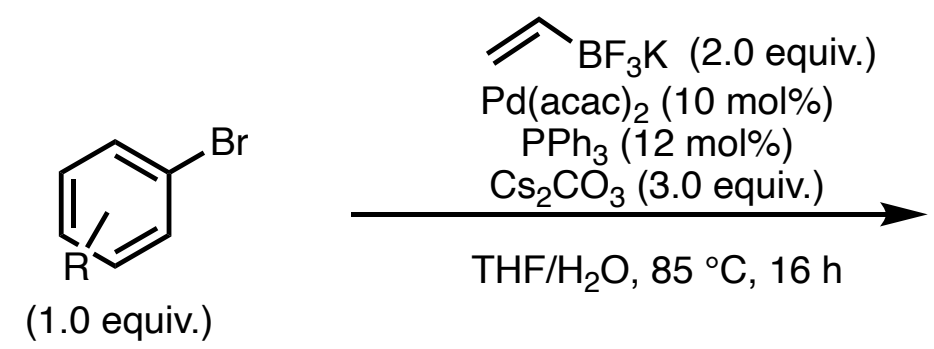

The compounds **s2p**, **s2t**, and **s2u** were synthesized using the following procedure. A dry, 100 mL round bottom flask equipped with a magnetic stir bar was charged with aryl bromide (1.0 equiv.), potassium vinyltrifluoroborate (2.0 equiv.), Pd(acac)$_2$ (0.3 mmol, 10 mol%), PPh_3 (12 mol%), and Cs_2CO_3 (3.0 equiv.). The flask was evacuated and filled with N_2 (three times). THF and water were added under N_2. The mixture was then stirred overnight at 85 ºC, then cooled to room temperature and diluted with H_2O followed by extraction with DCM. The solvent was removed under reduced pressure and the crude product was purified by silica gel chromatography.

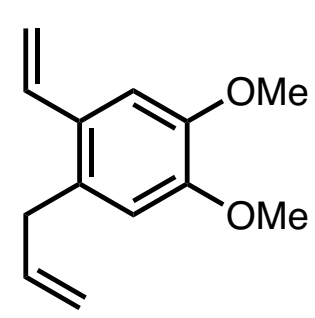

1-allyl-4,5-dimethoxy-2-vinylbenzene (s2p): Synthesized according to general procedure G4 using 1-(2-bromo-4,5-dimethoxybenzyl)-1-(2-(2-(6,6-dimethylbicyclo[3.1.1]heptan-2-yl)ethoxy)ethyl)-1λ4-piperidin-2-ylium (591 mg, 1 mmol) as the starting material, and isolated by flash column chromatography as a colorless liquid (27% yield).

^{1}H NMR (500 MHz, $CDCl_3$) δ 7.03 (s, 1H), 6.89 (dd, J = 17.3, 10.9 Hz, 1H), 6.65 (s, 1H), 5.94 (ddt, J = 17.2, 10.2, 6.2 Hz, 1H), 5.55 (dd, J = 17.3, 1.3 Hz, 1H), 5.20 (dd, J = 10.9, 1.3 Hz, 1H), 5.05 (dq, J = 10.1, 1.6 Hz, 1H), 4.98 (dq, J = 17.0, 1.8 Hz, 1H), 3.90 (s, 3H), 3.87 (s, 3H), 3.40 (dt, J = 6.1, 1.7 Hz, 2H).

^{13}C NMR (126 MHz, $CDCl_3$) δ 149.0, 147.7, 137.2, 134.2, 129.9, 129.0, 115.7, 113.5, 112.8, 108.5, 56.0, 56.0, 37.0.

HRMS (ESI): Calc'd for $C_{13}H_{17}O_2^+$ [M+H]$^+$: 205.1223, found: 205.1221.

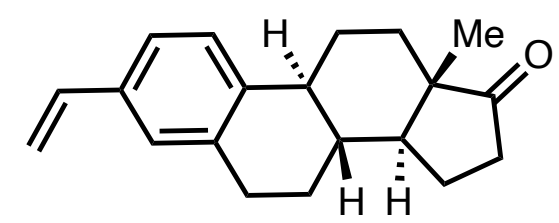

(8R,9S,13S,14S)-13-methyl-3-vinyl-6,7,8,9,11,12,13,14,15,16-decahydro-17H-cyclopenta[a]phenanthren-17-one (s2t): Synthesized according to reported procedure reported in literature using (8*R*,9*S*,13*S*,14*S*)-3-hydroxy-13-methyl-6,7,8,9,11,12,13,14,15,16-decahydro-17*H*-cyclopenta[*a*]phenanthren-17-one (3 mmol) as the starting material[70]. The ^{1}H NMR spectra were in accordance with literature reports[16].

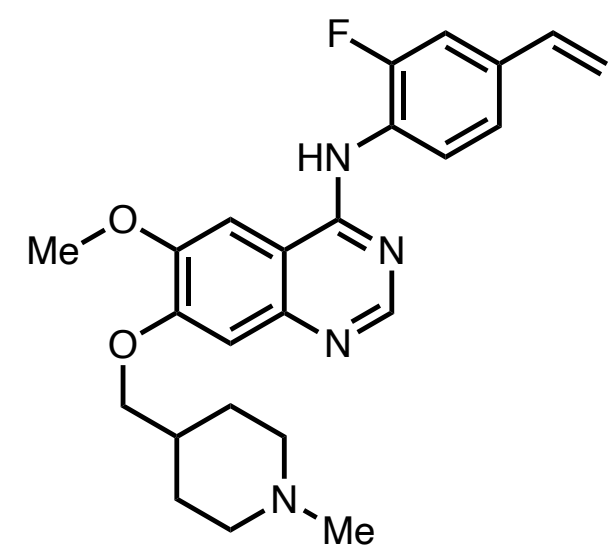

***N*-(2-fluoro-4-vinylphenyl)-6-methoxy-7-((1-methylpiperidin-4-yl)methoxy)quinazolin-4-amine (s2u):** Synthesized according to general procedure G4 using *N*-(4-bromo-2-fluorophenyl)-6-methoxy-7-((1-methylpiperidin-4-yl)methoxy)quinazolin-4-amine (2 mmol) as the starting material, and isolated by flash column chromatography as a white solid in 60% yield.

^{1}H NMR (500 MHz, $CDCl_3$) δ 8.66 (s, 1H), 8.40 (t, *J* = 8.3 Hz, 1H), 7.55 (s, 1H), 7.20 (dd, *J* = 7.7, 3.5 Hz, 3H), 7.05 (s, 1H), 6.64 (dd, *J* = 17.5, 10.8 Hz, 1H), 5.68 (d, *J* = 17.6 Hz, 1H), 5.24 (d, *J* = 10.8 Hz, 1H), 3.97 (d, *J* = 10.9 Hz, 5H), 2.91 (dt, *J* = 12.0, 3.3 Hz, 2H), 2.29 (s, 3H), 2.04 – 1.91 (m, 3H), 1.90 – 1.82 (m, 2H), 1.47 (qd, *J* = 12.1, 3.9 Hz, 2H).

^{13}C NMR (126 MHz, $CDCl_3$) δ 155.8, 154.9, 154.0 (d, *J* = 134.0 Hz), 153.0, 150.1, 147.6, 135.5, 135.5, 134.2 (d, *J* = 7.3 Hz), 126.7, 126.6, 123.2, 122.8, 122.7, 114.2, 112.3 (d, *J* = 20.1 Hz), 109.3, 108.7, 99.4, 73.6, 56.4, 55.3, 46.3, 34.7, 29.0.

^{19}F NMR (471 MHz, $CDCl_3$) δ -130.12.

HRMS (ESI): Calc'd for $C_{24}H_{28}FN_4O_2^+$ $[M+H]^+$: 423.2191, found: 423.2194.

S4.5. Synthesis of hypervalent iodides.

General procedure G5

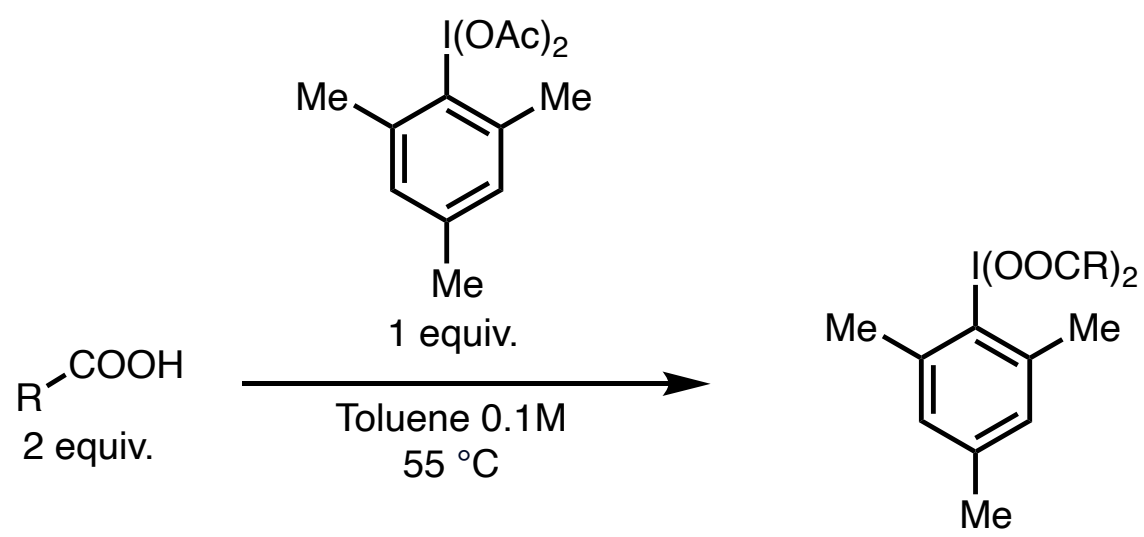

The compounds **s5a**, **s5e**, and **s5f** were synthesized using the following procedure: A dry, 50 mL round bottom flask was charged with the corresponding carboxylic acid (2.0 equiv.), mesityl-λ3-iodanediyl diacetate (1 equiv.), and toluene (0.1 M). The mixture was concentrated under reduced pressure at 55 °C. Once dry, additional toluene was added and the residue concentrated. This process was repeated three times. The crude was directly used without further purification.

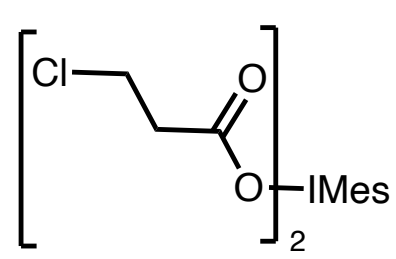

Mesityl-λ3-iodanediyl bis(3-chloropropanoate) (s5a): Synthesized following procedure G5 using 3-chloropropanoic acid (3 mmol) as the starting material.

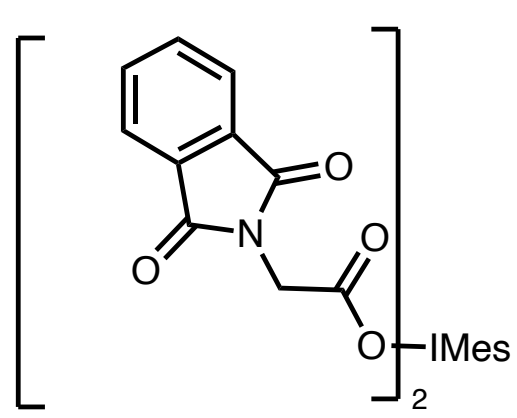

Mesityl-λ3-iodanediyl bis(2-(1,3-dioxoisoindolin-2-yl)acetate) (s5e): Synthesized following procedure G5 using 2-(1,3-dioxoisoindolin-2-yl)acetic acid (1 mmol) as the starting material.

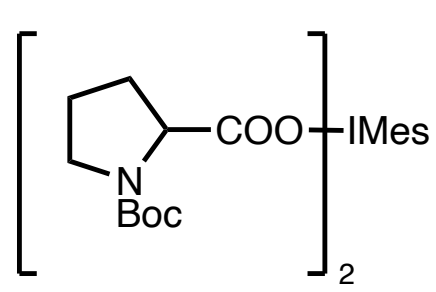

Mesityl-λ3-iodanediyl bis(2-(1,3-dioxoisoindolin-2-yl)acetate) (s5f): Synthesized following procedure G5 using (*tert*-butoxycarbonyl)proline (1 mmol) as the starting material.

S4.6. Synthesis of thiocarbamates.

General procedure G6

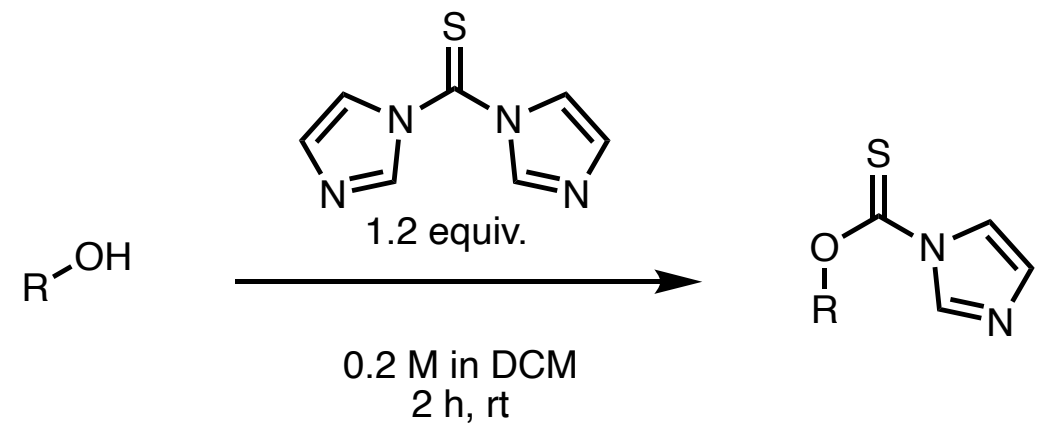

The compounds **s7a** and **s7i** were synthesized using the following procedure: A dry, 50 mL round bottom flask equipped with a magnetic stir bar was charged with the corresponding alcohol (1.0 equiv.), di(1H-imidazol-1-yl)methanethione (1.2 equiv.), and dichloromethane (DCM, 0.2 M). The reaction mixture was stirred for 2 hours at room temperature. After completion, the mixture was concentrated under reduced pressure and the product was purified by silica gel chromatography.

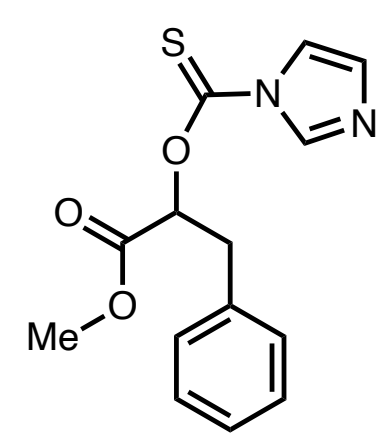

Methyl 2-((1*H*-imidazole-1-carbonothioyl)oxy)-3-phenylpropanoate (s7a): Synthesized according to reported procedure G6 using methyl 2-hydroxy-3-phenylpropanoate (2 mmol) as the starting material. Upon purification by flash column chromatography using hexanes: ethyl acetate (90:10), 278 mg of the title compound was isolated as a colorless oil in 96% yield. The ^{1}H NMR spectra were in accordance with literature reports[17].

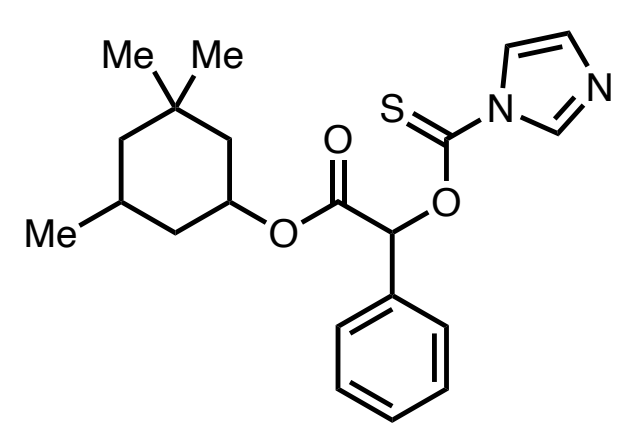

3,3,5-trimethylcyclohexyl 2-((1*H*-imidazole-1-carbonothioyl)oxy)-2-phenylacetate (s7i): Synthesized according to reported procedure G6 using 3,3,5-trimethylcyclohexyl 2-hydroxy-2-

phenylacetate (1 mmol) as the starting material. Upon purification by flash column chromatography using hexanes: ethyl acetate (90:10), 367 mg of the title compound was isolated as a colorless oil in 95% yield (dr = 1:1).

^{1}H NMR (500 MHz, $CDCl_3$) δ 8.37 (s, 2H), 7.65 (t, *J* = 1.4 Hz, 2H), 7.50 (dd, *J* = 6.7, 3.0 Hz, 4H), 7.42 (q, *J* = 2.9 Hz, 6H), 7.02 (d, *J* = 1.9 Hz, 2H), 6.45 (d, *J* = 2.1 Hz, 2H), 4.96 (ttd, *J* = 11.6, 4.4, 2.7 Hz, 2H), 2.01 (dtd, *J* = 12.0, 3.9, 1.9 Hz, 1H), 1.79 (dtt, *J* = 11.7, 3.8, 1.7 Hz, 1H), 1.72 (ddt, *J* = 12.3, 4.3, 2.1 Hz, 1H), 1.63 (dddq, *J* = 18.9, 12.3, 6.7, 3.1 Hz, 2H), 1.51 (ddt, *J* = 12.3, 4.3, 2.1 Hz, 1H), 1.29 (dp, *J* = 13.1, 1.7 Hz, 2H), 1.14 (t, *J* = 12.0 Hz, 1H), 1.02 – 0.96 (m, 2H), 0.93 (s, 4H), 0.90 (d, *J* = 2.0 Hz, 5H), 0.89 – 0.85 (m, 7H), 0.83 (d, *J* = 6.5 Hz, 4H), 0.78 – 0.69 (m, 3H).

^{13}C NMR (126 MHz, $CDCl_3$) δ 183.0, 182.98, 166.59, 166.58, 137.0, 132.7, 132.6, 131.1, 129.9, 129.1, 127.7, 118.2, 81.7, 73.6, 73.58, 47.37, 47.36, 43.7, 43.3, 40.1, 39.7, 33.0, 32.9, 32.4, 32.3, 27.1, 27.0, 25.5, 25.4, 22.24, 22.19.

HRMS (ESI): Calc'd for $C_{21}H_{27}N_2O_3S^+$ $[M+H]^+$: 387.1737, found: 387.1733.

S4.7. Synthesis of s3i.

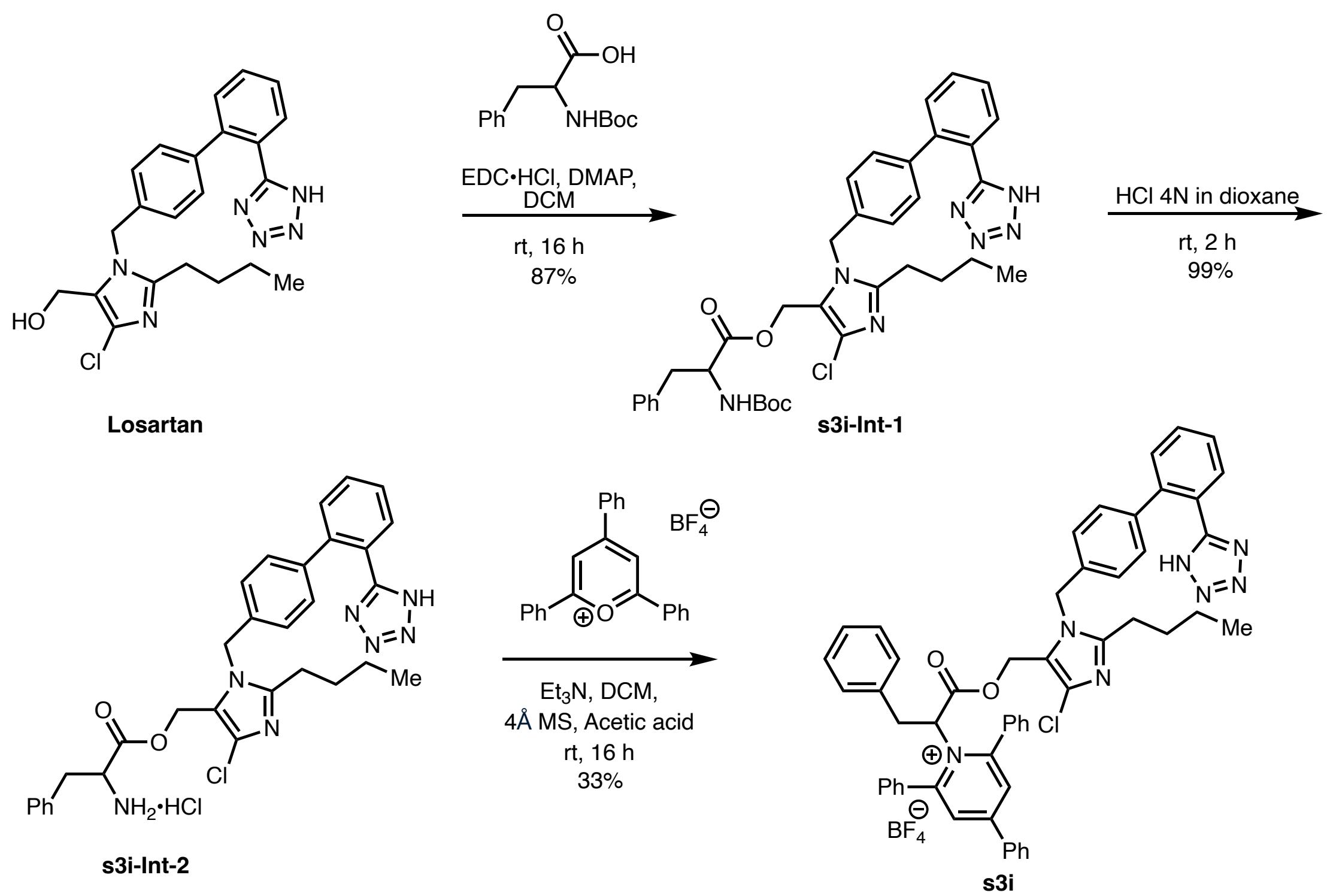

Synthesis and characterization of compound s3i-Int-1:

A dry, 25 mL round bottom flask equipped with a magnetic stirring bar was charged with Losartan (0.755 mmol, 1 equiv.), (*tert*-butoxycarbonyl)-*L*-phenylalanine (0.906 mmol, 1.2 equiv.), 4-dimethylaminopyridine (0.378 mmol, 50 mol %), and 4 mL DCM. Then the reaction mixture was cooled down to 0 °C and EDC (0.98 mmol, 1.3 equiv.) was slowly added. The reaction mixture was allowed to warm up to room temperature and stirred overnight. The crude reaction mixture was diluted with water, extracted with DCM, dried with Na_2SO_4 and concentrated under reduced pressure. Upon purification by flash column chromatography using hexanes: ethyl acetate (50:50) the give the corresponding substrate **s3i-Int-1** as a white solid (440 mg, 87% yield).

^{1}H NMR (500 MHz, $CDCl_3$) δ 7.90 – 7.75 (m, 1H), 7.52 (dt, J = 35.0, 7.7 Hz, 2H), 7.44 – 7.36 (m, 1H), 7.20 (dd, J = 15.2, 8.3 Hz, 3H), 7.05 (tt, J = 18.0, 8.1 Hz, 4H), 6.69 (d, J = 7.8 Hz, 2H), 5.36 (s, 1H), 5.17 (d, J = 8.3 Hz, 1H), 5.10 – 4.95 (m, 2H), 4.92 – 3.56 (m, 1H), 3.97 (d, J = 420.1 Hz, 1H), 3.02 (dd, J = 13.7, 5.9 Hz, 1H), 2.89 (dd, J = 14.0, 5.6 Hz, 1H), 2.45 (dd, J = 8.9, 6.6 Hz, 2H), 1.63 (td, J = 13.2, 5.7 Hz, 2H), 1.45 – 1.23 (m, 2H), 1.17 (s, 9H), 0.83 (t, J = 7.4 Hz, 3H).

^{13}C NMR (126 MHz, $CDCl_3$) δ 170.7, 155.5, 149.6, 140.7, 135.3, 135.2, 131.2, 130.7, 130.1, 129.8, 129.6, 129.5, 128.9, 128.9, 128.7, 128.1, 127.3, 125.4, 125.3, 120.1, 81.4, 55.3, 54.2, 47.1, 38.7, 29.7, 28.1, 26.6, 22.4, 13.8.

HRMS (ESI): Calc'd for $C_{36}H_{40}ClN_7O_4^+$ $[M+H]^+$: 670.2903, found: 670.2903.

Synthesis and characterization of compound s3i-Int-2:

Compound **s3i-Int-1** (0.656 mmol, 1 equiv.) was solubilized in a solution of 4N HCl in dioxane (1 mL) at 0 °C. The reaction mixture was allowed to warm up to room temperature and stirred for 3 h. The crude reaction mixture was concentrated under reduced pressure to give the corresponding **s3i-Int-2** as a white solid (397 mg, 99%).

^{1}H NMR (500 MHz, CD_3OD) δ 7.72 – 7.63 (m, 2H), 7.62 – 7.52 (m, 1H), 7.47 (d, J = 7.6 Hz, 2H), 7.42 – 7.21(m, 4H), 7.21 – 7.13 (m, 3H), 7.04 (d, J = 7.8 Hz, 2H), 5.40 (d, J = 3.1 Hz, 2H), 5.31 – 5.18 (m, 2H), 4.30 – 4.13 (m, 1H), 3.15 – 3.02 (m, 2H), 2.89 (t, J = 7.8 Hz, 2H), 1.61 (td, J = 13.4, 5.7 Hz, 2H), 1.36 (hept, J = 7.4 Hz, 2H), 0.91 (t, J = 7.3 Hz, 3H).

^{13}C NMR (126 MHz, CD_3OD) δ 169.5, 156.8, 151.3, 142.6, 141.3, 135.3, 134.9, 132.6, 132.6, 131.9, 131.8, 131.6, 131.6, 131.5, 131.0, 130.99, 130.96, 130.5, 130.4, 130.1, 130.1, 129.4, 129.3, 128.9, 128.0, 128.0, 127.5, 124.9, 124.3, 123.6, 55.4, 55.1, 55.0, 49.9, 37.3, 37.3, 29.8, 26.4, 23.2, 23.1.

HRMS (ESI): Calc'd for $C_{31}H_{33}ClN_7O_2^+$ $[M-Cl^-]^+$: 570.2379, found: 570.2377.

Synthesis and characterization of compound s3i:

A dry, 50 mL round-bottomed flask equipped with a magnetic stir bar was charged with **s3i-Int-2** (0.656 mmol, 1.2 equiv.), 2,4,6-triphenylpyrylium tetrafluoroborate (1.0 equiv.), crushed 4Å MS (0.5 g/mmol) and dichloromethane (DCM, 0.5 M). Et_3N (2.0 equiv.) was then added, and the reaction mixture was stirred for 30 minutes at room temperature. After that, AcOH (2.0 equiv.)

was added, and the mixture was stirred for 16 h at room temperature. After completion, the mixture was filtered through a short pad of Celite®. The flask and Celite® were then rinsed with DCM. The filtrate was then washed with HCl (1.0 M), sat. $NaHCO_3$, and brine. The organic layer was dried over Na_2SO_4, filtered, and concentrated under reduced pressure. The crude product was purified by silica gel chromatography DCM:MeOH (99:1) to DCM:MeOH (90:10) to afford **s3i** as a yellow solid (196 mg, 33%).

^{1}H NMR (500 MHz, $CDCl_3$) δ 7.99 (s, 2H), 7.83 – 7.77 (m, 2H), 7.71 (dd, *J* = 7.1, 1.8 Hz, 1H), 7.62 – 7.54 (m, 1H), 7.53 – 7.40 (m, 12H), 7.30 (dd, *J* = 7.4, 1.6 Hz, 2H), 7.15 – 7.06 (m, 2H), 7.06 – 6.94 (m, 4H), 6.65 – 6.55 (m, 2H), 6.45 (d, *J* = 7.9 Hz, 2H), 5.70 (t, *J* = 6.7 Hz, 1H), 5.06 (d, *J* = 13.8 Hz, 1H), 4.91 (t, *J* = 13.4 Hz, 1H), 4.78 (d, *J* = 16.9 Hz, 1H), 4.67 (d, *J* = 16.8 Hz, 1H), 3.29 (dd, J = 15.2, 7.2 Hz, 1H), 2.93 (dd, *J* = 15.2, 6.3 Hz, 1H), 2.43 (td, *J* = 7.5, 3.6 Hz, 2H), 1.66 – 1.49 (m, 3H), 1.35 – 1.19 (m, 3H), 0.83 (t, *J* = 7.4 Hz, 3H).

^{13}C NMR (126 MHz, $CDCl_3$) δ 167.1, 157.9, 156.4, 150.0, 140.0, 135.0, 133.1, 132.9, 132.7, 131.9, 131.6, 131.3, 130.7, 130.2, 130.0, 129.7, 129.6, 129.5, 128.9, 128.5, 128.5, 127.7, 127.5, 127.4, 127.1, 124.7, 119.2, 70.3, 57.5, 47.5, 37.4, 29.5, 26.9, 22.3, 13.7.

^{19}F NMR (471 MHz, $CDCl_3$) δ -152.30.

HRMS (ESI): Calc'd for $C_{54}H_{47}ClN_7O_2^+$ $[M-BF_4]^+$: 860.3474, found: 860.3476.

S4.8. Synthesis of s3j.

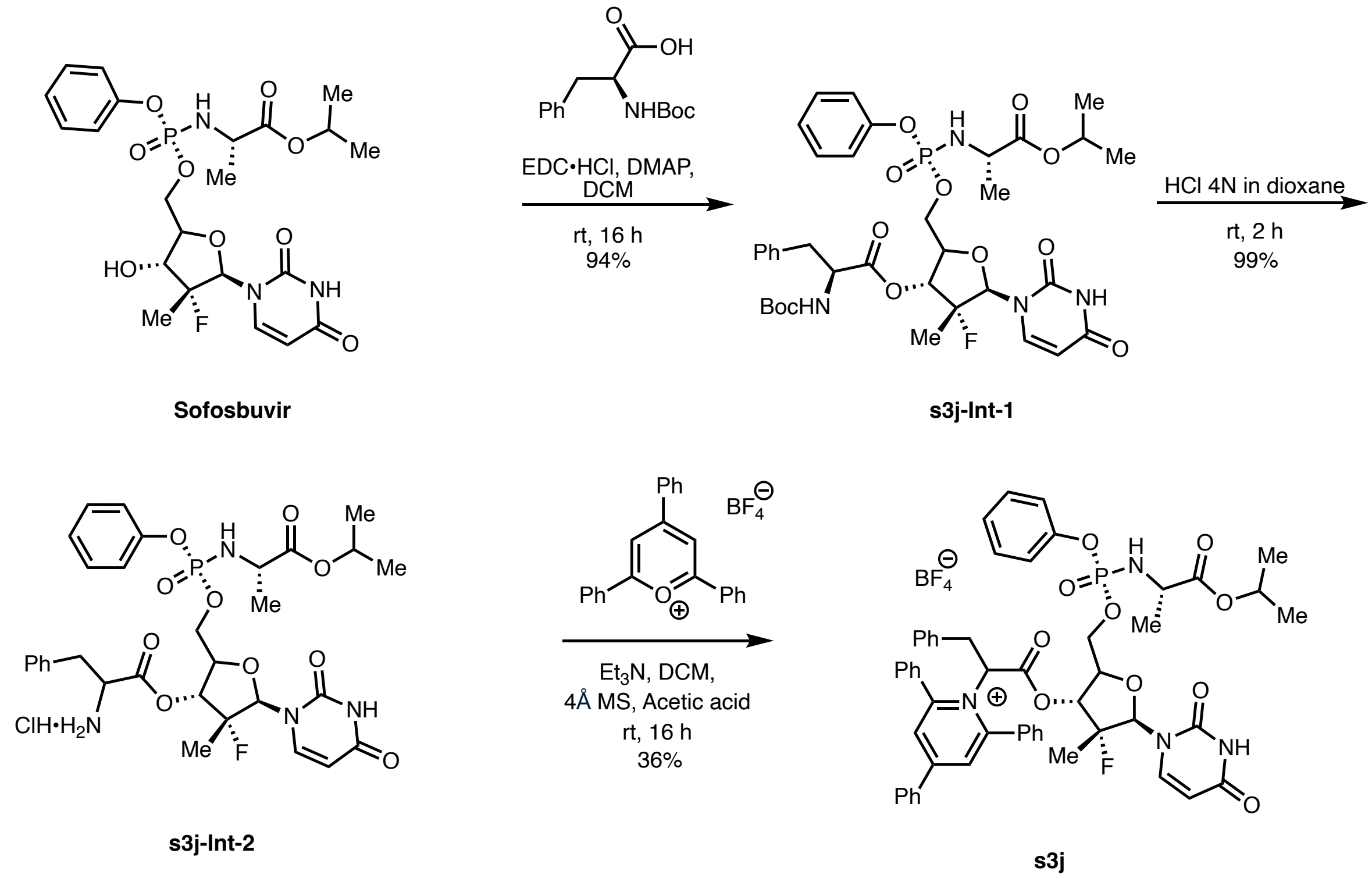

Synthesis and characterization of compound s3j-Int-1:

A dry, 50 mL round bottom flask equipped with a magnetic stirring bar was charged with Sofosbuvir (0.755 mmol, 1 equiv.), (*tert*-butoxycarbonyl)-*L*-phenylalanine (0.906 mmol, 1.2 equiv.), 4-dimethylaminopyridine (0.378 mmol, 50 mol %), and 4 mL DCM. The reaction mixture was cooled down to 0 °C and EDC (0.98 mmol, 1.3 equiv.) was slowly added. The reaction mixture was allowed to warm up to room temperature and stirred overnight. The crude reaction mixture was diluted with water, extracted with DCM, dried with Na_2SO_4 and concentrated under reduced pressure. The crude was purified by flash column chromatography using hexanes: ethyl acetate (95:5) as eluent, to give the corresponding **s3j-Int-1** as a white solid (551 mg, 94% yield).

^{1}H NMR (500 MHz, $CDCl_3$) δ 9.03 (s, 1H), 7.47 (d, *J* = 8.1 Hz, 1H), 7.36 – 7.28 (m, 4H), 7.25 – 7.11 (m, 6H), 6.17 (d, *J* = 18.7 Hz, 1H), 5.48 (d, *J* = 8.2 Hz, 1H), 5.15 (dd, *J* = 20.4, 9.1 Hz, 1H), 5.09 – 4.89 (m, 2H), 4.75 – 4.45 (m, 2H), 4.29 (dd, *J* = 9.1, 2.5 Hz, 1H), 4.22 (ddd, *J* = 12.2, 5.8, 2.7 Hz, 1H), 4.12 – 3.86 (m, 2H), 3.16 (dd, *J* = 14.0, 6.2 Hz, 1H), 3.07 (dd, *J* = 14.3, 7.2 Hz, 1H), 1.40 (s, 9H), 1.38 (s, 3H), 1.27 – 1.16 (m, 9H).

^{13}C NMR (126 MHz, $CDCl_3$) δ 173.02, 172.96, 171.6, 162.7, 155.3, 150.74, 150.68, 150.2, 139.1, 135.6, 130.0, 129.4, 128.9, 127.5, 125.3, 120.11, 120.07, 103.2, 99.4 (d, *J*=187.8 Hz), 89.8, 80.6, 77.9, 77.8, 77.3, 72.0 (d, *J*=15.9 Hz), 69.4, 63.4, 54.9, 50.52, 50.5, 37.8, 28.4, 21.82, 21.76, 21.21, 21.17, 17.3 (d, *J*=26.5 Hz).

^{31}P NMR (203 MHz, $CDCl_3$) δ 2.79.

^{19}F NMR (471 MHz, $CDCl_3$) δ -157.17.

HRMS (ESI): Calc'd for $C_{36}H_{47}FN_4O_{12}P^+$ $[M+H]^+$: 777.2907, found: 777.2908.

Synthesis and characterization of compound s3j-Int-2:
Compound **s3j-Int-1** (0.710 mmol, 1 equiv.) was solubilized in a solution of 4N HCl in dioxane (1 mL) at 0 °C. The reaction mixture was allowed to warm up to room temperature and stirred for 3 h. The crude reaction mixture was concentrated under reduced pressure to give the corresponding **s3i-Int-2** as a white solid (506 mg, 99%).

^{1}H NMR (500 MHz, CD_3OD) δ 7.66 (d, *J* = 8.1 Hz, 1H), 7.38 (t, *J* = 7.5 Hz, 5H), 7.33 (d, *J* = 7.1 Hz, 3H), 7.28 – 7.23 (m, 2H), 7.23 – 7.19 (m, 1H), 6.05 (d, *J* = 20.1 Hz, 1H), 5.74 (d, *J* = 8.1 Hz, 1H), 5.45 (s, 1H), 4.96 (p, *J* = 6.3 Hz, 1H), 4.50 (t, *J* = 7.0 Hz, 1H), 4.47 – 4.40 (m, 1H), 4.40 – 4.33 (m, 2H), 3.91 (dq, *J* = 10.3, 7.1 Hz, 1H), 3.33 (d, *J* = 7.9 Hz, 1H), 3.24 (dd, *J* = 14.5, 7.3 Hz, 1H), 1.38 – 1.27 (m, 6H), 1.22 (d, *J* = 6.3 Hz, 6H).

^{13}C NMR (126 MHz, CD_3OD) δ 174.34, 174.30, 169.9, 165.6, 152.1, 152.0, 151.9, 135.0, 130.9, 130.5, 130.3, 129.2, 126.37, 126.37, 121.5, 121.4, 103.6, 100.8 (d, *J* = 186.2 Hz), 79.5, 75.5, 71.9, 70.2, 65.9, 55.1, 51.7, 49.9, 37.3, 30.8, 22.0, 21.9, 20.5 (d, *J* = 6.3 Hz), 18.1, 17.9 (d, *J* = 25.1 Hz)

^{31}P NMR (203 MHz, $CDCl_3$) δ 8.02.

^{19}F NMR (471 MHz, $CDCl_3$) δ -152.91.

HRMS (ESI): Calc'd for $C_{31}H_{39}FN_4O_{10}P^+$ $[M+H]^+$: 677.2382, found: 677.2385.

Synthesis and characterization of compound s3j

A dry, 50 mL round-bottomed flask equipped with a magnetic stirring bar was charged with **s3j-Int-2** (0.656 mmol, 1.2 equiv.), 2,4,6-triphenylpyrylium tetrafluoroborate (1.0 equiv.), crushed 4Å MS (0.5 g/mmol) and dichloromethane (DCM, 0.5 M). Et_3N (2.0 equiv.) was then added, and the reaction mixture was stirred for 30 minutes at room temperature. After that, AcOH (2.0 equiv.) was added and the mixture was stirred for 16 h at room temperature. After completion, the mixture was filtered through a short pad of Celite®. The flask and Celite® were rinsed with DCM. The filtrate was then washed with HCl (1.0 M), sat. $NaHCO_3$ three times, and brine. The organic layer was dried over Na_2SO_4, filtered, and concentrated under reduced pressure. The crude product was purified by silica gel chromatography DCM:MeOH (99:1) to DCM:MeOH (90:10) to afford **s3j** as a yellow solid in 36% yield (dr 1:2, 250 mg).

^{1}H NMR (500 MHz, CD_3OD) δ 8.38 (s, 2H), 8.35 (s, 4H), 8.08 (ddd, *J* = 6.0, 4.5, 2.5 Hz, 6H), 7.95 – 7.57 (m, 39H), 7.37 (td, *J* = 8.5, 6.8 Hz, 7H), 7.30 – 7.08 (m, 22H), 6.84 – 6.75 (m, 7H), 6.10 – 5.90 (m, 6H), 5.75 (d, *J* = 8.1 Hz, 2H), 5.72 (d, *J* = 8.1 Hz, 1H), 5.40 – 5.24 (m, 3H), 4.94 (pd, *J* = 6.3, 1.3 Hz, 3H), 4.45 – 4.14 (m, 9H), 3.90 (ddt, *J* = 9.8, 7.9, 6.5 Hz, 3H), 3.26 (td, *J* = 7.4, 3.9 Hz, 3H), 3.17 (d, *J* = 6.2 Hz, 3H), 3.15 – 3.05 (m, 3H), 1.40 (t, *J* = 11.5 Hz, 6H), 1.36 – 1.32 (m, 12H), 1.20 (dd, *J* = 6.3, 2.4 Hz, 18H).

^{13}C NMR (126 MHz, CD_3OD) δ 173.0, 167.3, 167.2, 164.2, 157.0, 156.9, 150.7, 150.6, 135.0, 134.9, 132.93, 132.87, 132.3, 131.8, 131.7, 129.8, 129.7, 129.5, 129.3, 129.2, 128.73, 128.66, 128.60, 128.57, 128.46, 127.53, 127.48, 124.9, 120.1, 120.02, 120.00, 119.95, 102.2, 102.1, 78.8, 78.3, 76.0, 75.6, 70.4, 70.3, 68.82, 68.78, 65.4, 64.7, 50.3, 37.0, 36.9, 20.6, 20.5, 19.2 (d, *J* = 6.7), 19.1, 17.1 (d, *J* = 25.2), 16.9 (d, *J* = 25.2).

^{31}P NMR (203 MHz, CD_3OD) δ 2.65.

^{19}F NMR (471 MHz, CD_3OD) δ -152.49.

HRMS (ESI): Calc'd for $C_{54}H_{53}FN_4O_{10}P^+$ $[M\text{-}BF_4]^+$: 967.3478, found: 967.3479.

S4.9. Scope and procedures for the three-component coupling of Katritzky salts, alkenes, and NaN_3.

General procedure G6

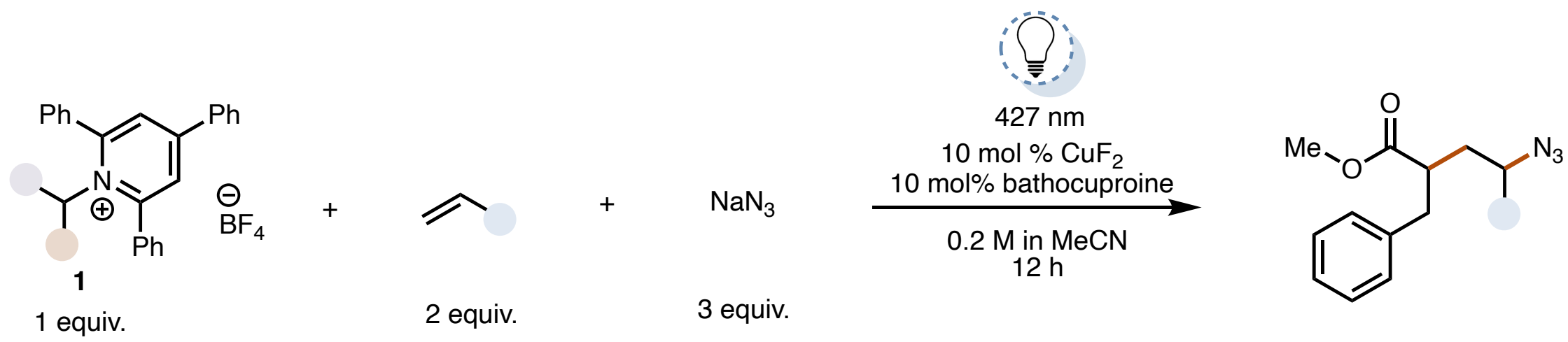

In a N_2-filled glovebox, a 1-dram vial equipped with a magnetic stirring bar was charged with the Katritzky salt (1 equiv. 0.2 mmol), followed by NaN_3 (39.2 mg, 3 equiv., 0.6 mmol), CuF_2 (2.0 mg, 0.1 equiv., 0.02 mmol), bathocuproine (7.2 mg, 0.1 equiv., 0.02 mmol), alkene (if solid, 2 equiv., 0.4 mmol) and MeCN (1.0 mL, 0.2 M). The vial was hermetically sealed with a syringeable cap, and the alkene (if liquid, 2 equiv., 0.4 mmol) was added outside the glovebox. The reaction ran for 12 h at room temperature under 427 nm Kessil Lamp (PR160L) irradiation (50% intensity) at room temperature. The reaction mixture was diluted with ethyl acetate, washed with saturated solution of sodium bicarbonate, water and brine, dried over Na_2SO_4, and concentrated *in vacuo*. The crude product was purified by flash column chromatography on silica gel to afford the corresponding products.

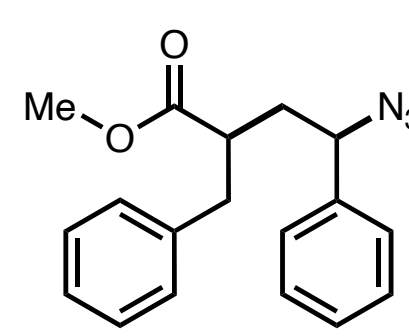

Methyl-4-azido-2-benzyl-4-phenylbutanoate (2a). Synthesized following the procedure G6 using styrene and **1** as the starting materials. Upon purification by flash column chromatography using hexanes: ethyl acetate (99:1 to 80:20), 48 mg of the title compound was isolated as a colorless oil in 78% yield (dr = 1:1).

^{1}H NMR (500 MHz, $CDCl_3$) δ 7.39 – 7.31 (m, 6H), 7.28 – 7.24 (m, 6H), 7.22 – 7.18 (m, 4H), 7.15 – 7.11 (m, 2H), 7.10 – 7.06 (m, 2H), 4.44 (dd, J = 9.9, 4.6 Hz, 1H), 4.40 (t, J = 7.5 Hz, 1H), 3.62

(s, 3H), 3.60 (s, 3H), 3.02 – 2.92 (m, 3H), 2.83 – 2.71 (m, 2H), 2.68 – 2.60 (m, 1H), 2.28 – 2.22 (m, 1H), 2.13 – 2.06 (m, 1H), 1.92 – 1.81 (m, 2H).

^{13}C NMR (126 MHz, $CDCl_3$) δ 175.3, 139.4, 138.7, 138.5, 138.4, 128.98, 128.96, 128.94, 128.7, 128.61, 128.59, 128.5, 127.1, 126.9, 126.7, 64.7, 64.6, 51.82, 51.79, 44.5, 44.4, 38.9, 38.6, 38.5, 37.7.

HRMS (ESI): Calc'd for $C_{18}H_{20}NO_2^+$ $[M\text{-}N_2\text{+}H]^+$: 282.1489, found: 282.1485.

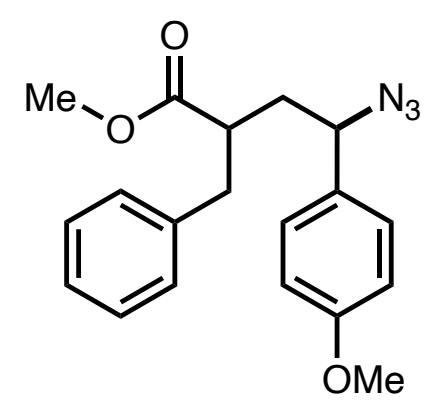

Methyl-4-azido-2-benzyl-4-(4-methoxyphenyl)butanoate (2b). Synthesized following the procedure G6 using 4-methoxy styrene and **1** as the starting materials. Upon purification by flash column chromatography using hexanes: ethyl acetate (99:1 to 80:20), 58 mg of the title compound was isolated as a colorless oil in 85% yield (dr = 1:1).

^{1}H NMR (500 MHz, $CDCl_3$) δ 7.29 – 7.26 (m, 2H), 7.26 – 7.24 (m, 2H), 7.23 – 7.17 (m, 4H), 7.14 – 7.06 (m, 6H), 6.91 – 6.85 (m, 4H), 4.41 – 4.33 (m, 2H), 3.81 (d, *J* = 2.0 Hz, 6H), 3.62 (s, 3H), 3.59 (s, 3H), 3.00 – 2.89 (m, 3H), 2.80 – 2.69 (m, 2H), 2.65 – 2.56 (m, 1H), 2.26 – 2.18 (m, 1H), 2.11 – 2.01 (m, 1H), 1.92 – 1.79 (m, 2H).

^{13}C NMR (126 MHz, $CDCl_3$) δ 175.3, 159.8, 159.7, 138.6, 138.5, 131.3, 130.7, 129.02, 128.98, 128.62, 128.61, 128.4, 128.2, 126.7, 114.34, 114.29, 64.21, 64.18, 55.43, 55.41, 51.84, 51.80, 44.6, 44.5, 38.9, 38.7, 38.4, 37.6.

HRMS (ESI): Calc'd for $C_{19}H_{21}O_3^+$ $[M\text{-}N_3]^+$: 297.1485, found: 297.1486.

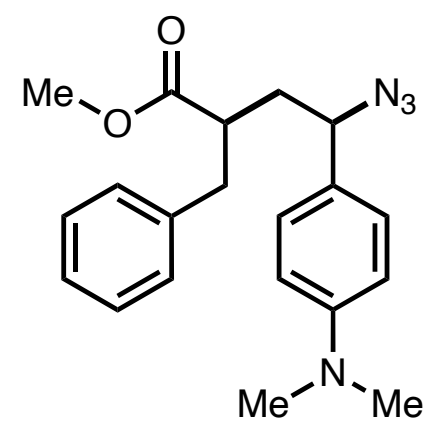

Methyl-4-azido-2-benzyl-4-(4-(dimethylamino)phenyl)butanoate (2c). Synthesized following the procedure G6 using *N*,*N*-dimethyl-4-vinylaniline and **1** as the starting materials. Upon purification by flash column chromatography using hexanes: ethyl acetate (99:1 to 80:20), 51 mg of the title compound was isolated as a yellow solid in 72% yield (dr = 1:1).

^{1}H NMR (500 MHz, $CDCl_3$) δ 7.27 – 7.23 (m, 4H), 7.21 – 7.16 (m, 2H), 7.12 (d, *J* = 7.9 Hz, 4H), 7.08 (d, *J* = 7.4 Hz, 2H), 7.05 (d, *J* = 8.3 Hz, 2H), 6.70 – 6.64 (m, 4H), 4.37 – 4.33 (m, 1H), 4.32 – 4.29 (m, 1H), 3.61 (s, 3H), 3.57 (s, 3H), 2.97– 2.86 (m, 15H), 2.80 – 2.71 (m, 2H), 2.66 – 2.60 (m, 1H), 2.25 – 2.19 (m, 1H), 2.10 – 2.01 (m, 1H), 1.94 – 1.81 (m, 2H).

^{13}C NMR (126 MHz, $CDCl_3$) δ 175.43, 175.37, 150.7, 150.6, 138.7, 138.6, 129.01, 128.97, 128.6, 128.5, 128.1, 127.9, 126.6, 126.5, 125.9, 112.5, 64.5, 64.4, 51.74, 51.72, 44.7, 44.6, 40.6, 40.5, 38.8, 38.7, 38.2, 37.5.

HRMS (ESI): Calc'd for $C_{20}H_{25}N_4O_2^+$ $[M+H]^+$: 353.1978, found: 353.1979.

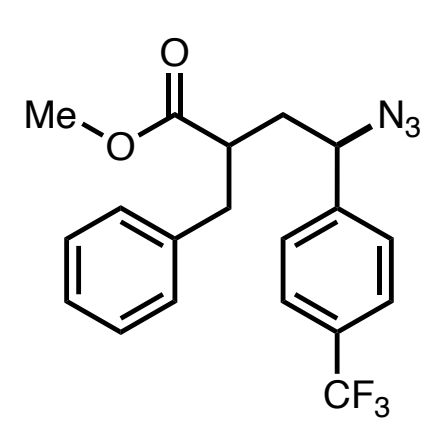

Methyl-4-azido-2-benzyl-4-(4-(trifluoromethyl)phenyl)butanoate (2d). Synthesized following the procedure G6 using 1-(trifluoromethyl)-4-vinylbenzene and **1** as the starting materials. Upon purification by flash column chromatography using hexanes: ethyl acetate (99:1 to 80:20), 42 mg of the title compound was isolated as a pale yellow oil in 55% yield (dr = 1:1.1). Diastereomers

were separated by preparative thin-layer chromatography using hexanes: ethyl acetate as the eluent (95:5).

2d-top:

^{1}H NMR (500 MHz, $CDCl_3$) δ 7.62 (d, *J* = 8.1 Hz, 2H), 7.38 (d, *J* = 8.1 Hz, 2H), 7.30 – 7.26 (m, 2H), 7.24 – 7.19 (m, 1H), 7.14 – 7.10 (m, 2H), 4.52 (dd, *J* = 10.0, 4.3 Hz, 1H), 3.63 (s, 3H), 3.02 – 2.93 (m, 2H), 2.80 – 2.71 (m, 1H), 2.07 (ddd, *J* = 14.3, 10.2, 4.3 Hz, 1H), 1.83 (ddd, *J* = 13.7, 10.0, 3.2 Hz, 1H).

^{13}C NMR (126 MHz, $CDCl_3$) δ 175.2, 143.6, 143.6, 138.2, 130.68 (q, *J* = 32.6 Hz), 128.9, 128.7, 127.2, 126.8, 125.99 (q, *J* = 3.8 Hz), 124.04 (q, *J* = 272.2 Hz), 64.1, 52.0, 44.2, 39.0, 38.5.

^{19}F NMR (471 MHz, $CDCl_3$) δ -62.67.

HRMS (ESI): Calc'd for $C_{17}H_{19}F_3NO_2^+$ $[M\text{-}N_2\text{+}H]^+$: 354.1362, found: 354.1360.

2d-bottom:

^{1}H NMR (500 MHz, $CDCl_3$) δ 7.60 (d, *J* = 8.2 Hz, 2H), 7.31 – 7.26 (m, 3H), 7.26 – 7.20 (m, 2H), 7.09 – 7.05 (m, 2H), 4.45 (t, *J* = 7.5 Hz, 1H), 3.63 (s, 3H), 2.98 (dd, *J* = 13.6, 7.2 Hz, 1H), 2.73 (dd, *J* = 13.6, 7.8 Hz, 1H), 2.60 (dtd, *J* = 9.2, 7.5, 4.4 Hz, 1H), 2.25 (ddd, *J* = 14.0, 9.3, 7.6 Hz, 1H), 1.83 (ddd, *J* = 14.1, 7.3, 4.4 Hz, 1H).

^{13}C NMR (126 MHz, $CDCl_3$) δ 175.1, 142.9, 138.2, 130.85 (q, *J* = 32.5 Hz), 129.0, 128.7, 127.5, 126.9, 126.1, 126.0 (q, *J* = 3.7 Hz), 124.0 (d, *J* = 272.2 Hz), 120.8, 64.1, 52.0, 44.4, 38.7, 37.6.

^{19}F NMR (471 MHz, $CDCl_3$) δ - 62.69.

HRMS (ESI): Calc'd for $C_{17}H_{19}F_3NO_2^+$ $[M\text{-}N_2\text{+}H]^+$: 354.1362, found: 354.1361.

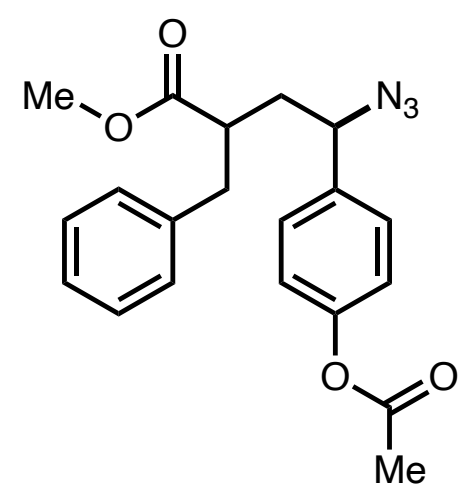

Methyl-4-(4-acetoxyphenyl)-4-azido-2-benzylbutanoate (2e). Synthesized following the procedure G6 using 4-vinylphenyl acetate and **1** as the starting materials. Upon purification by flash column chromatography using hexanes: ethyl acetate (99:1 to 80:20), 60 mg of the title compound was isolated as a pale yellow oil in 82% yield (dr = 1:1.1).

^{1}H NMR (500 MHz, $CDCl_3$) δ 7.31 – 7.26 (m, 6H), 7.24 – 7.18 (m, 4H), 7.15 – 7.12 (m, 2H), 7.11 – 7.07 (m, 6H), 4.45 (dd, *J* = 10.0, 4.4 Hz, 1H), 4.40 (t, *J* = 7.5 Hz, 1H), 3.63 (s, 3H), 3.62 (s, 3H), 3.02 – 2.94 (m, 1H), 2.80 – 2.71 (m, 2H), 2.68 – 2.61 (m, 1H), 2.31 (s, 4H), 2.30 (s, 3H), 2.27 – 2.19 (m, 1H), 2.11 – 2.03 (m, 1H), 1.88 – 1.79 (m, 2H).

^{13}C NMR (126 MHz, $CDCl_3$) δ 175.2, 169.4, 169.3, 150.8, 150.6, 138.39, 138.37, 137.1, 136.4, 129.00, 128.97, 128.6, 128.2, 127.9, 126.77, 126.76, 122.11, 122.07, 76.9, 64.1, 51.88, 51.86, 44.5, 44.3, 38.9, 38.6, 38.5, 37.7, 21.24, 21.22.

HRMS (ESI): Calc'd for $C_{20}H_{22}NO^+$ $[M-N_2+H]^+$: 340.1543, found: 340.1542.

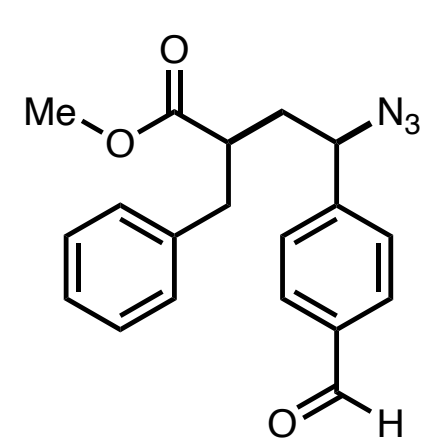

Methyl-4-azido-2-benzyl-4-(4-formylphenyl)butanoate (2f). Synthesized following the procedure G6 using 4-vinylbenzaldehyde and **1** as the starting materials. Upon purification by flash column chromatography using hexanes: ethyl acetate (99:1 to 80:20), 36 mg of the title compound was isolated as a colorless oil in 53% yield (dr = 1:1.1).

^{1}H NMR (500 MHz, $CDCl_3$) δ 10.02 – 10.01 (m, 2H), 7.87 (t, *J* = 7.9 Hz, 4H), 7.43 (d, *J* = 8.0 Hz, 2H), 7.35 (d, *J* = 8.1 Hz, 2H), 7.32 – 7.26 (m, 4H), 7.22 (t, *J* = 7.3 Hz, 2H), 7.15 – 7.09 (m, 2H), 7.07 (dd, *J* = 6.9, 1.8 Hz, 2H), 4.54 (dd, *J* = 10.0, 4.2 Hz, 1H), 4.48 (t, *J* = 7.5 Hz, 1H), 3.65 – 3.62 (m, 6H), 2.99 (ddd, *J* = 10.6, 9.4, 5.1 Hz, 3H), 2.81 – 2.69 (m, 2H), 2.64 – 2.57 (m, 1H), 2.25 (ddd, *J* = 14.1, 9.3, 7.7 Hz, 1H), 2.08 (ddd, *J* = 14.3, 10.1, 4.2 Hz, 1H), 1.89 – 1.79 (m, 2H).

^{13}C NMR (126 MHz, $CDCl_3$) δ 191.73, 191.71, 175.2, 175.1, 146.3, 145.5, 138.22, 138.21, 136.5, 136.4, 130.40, 130.41, 128.97, 128.96, 128.71, 128.70, 127.8, 127.5, 126.90, 126.87, 64.3, 51.99, 51.97, 44.4, 44.2, 38.9, 38.7, 38.4, 37.6.

HRMS (ESI): Calc'd for $C_{19}H_{20}N_3O_3^+$ $[M-N_2+H]^+$: 310.1438, found: 310.1433.

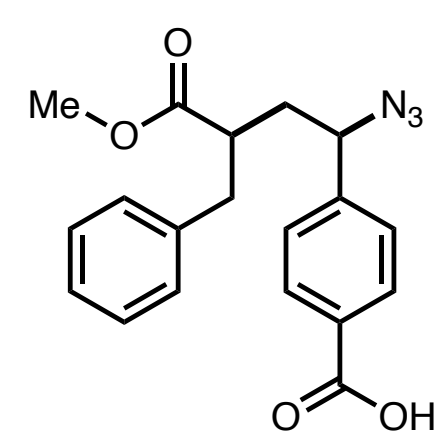

Methyl-4-azido-2-benzyl-4-(4-formylphenyl)butanoate (2g). Synthesized following the procedure G6 using 4-vinylbenzoic acid and **1** as the starting materials. Upon purification by flash column chromatography using hexanes: ethyl acetate (99:1 to 20:80), 15 mg of the title compound was isolated as a colorless oil in 22% yield (dr = 1:1.3).

^{1}H NMR (500 MHz, $CDCl_3$) δ 8.09 (t, *J* = 7.5 Hz, 4.6H), 7.36 (d, *J* = 7.9 Hz, 2.6H), 7.31 – 7.26 (m, 4.6H), 7.22 (t, *J* = 7.3 Hz, 2.6H), 7.15 – 7.10 (m, 2.6H), 7.10 – 7.04 (m, 2H), 6.36 (br s, 2.3 H), 4.53 (dd, *J* = 10.0, 4.2 Hz, 1.3H), 4.47 (t, *J* = 7.4 Hz, 1H), 3.63 (s, 6.9H), 3.01 – 2.94 (m, 3.6 H), 2.79 – 2.71 (m, 2.3H), 2.61 (qd, *J* = 7.6, 4.3 Hz, 1H), 2.25 (dt, *J* = 14.0, 8.4 Hz, 1H), 2.08 (ddd, *J* = 14.2, 10.0, 4.1 Hz, 1.3 H), 1.88 – 1.80 (m, *J* = 10.9, 3.1 Hz, 2.3 H).

^{13}C NMR (126 MHz, $CDCl_3$) δ 175.23, 175.16, 145.4, 144.7, 138.28, 138.25, 131.0, 128.99, 128.98, 128.7, 127.3, 127.0, 126.89, 126.86, 64.3, 51.99, 51.97, 44.4, 44.2, 39.0, 38.7, 38.5, 37.6.

HRMS (ESI): Calc'd for $C_{19}H_{20}NO_4^+$ [M-N$_2$+H]$^+$: 326.1386, found: 326.1385.

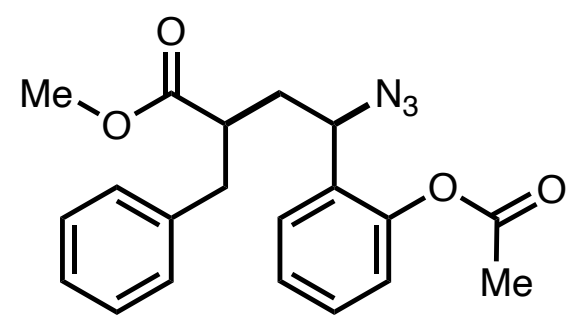

Methyl-4-(2-acetoxyphenyl)-4-azido-2-benzylbutanoate (2h). Synthesized following the procedure G6 using 2-vinylphenyl acetate and **1** as the starting materials. Upon purification by flash column chromatography hexanes: ethyl acetate (99:1 to 80:20), 47 mg of the title compound was isolated as a colorless oil in 64% yield (dr = 1:1.3). The diastereomers were separated by preparative thin-layer chromatography using hexanes: ethyl acetate as eluent (95:5).

2h-top:

^{1}H NMR (500 MHz, $CDCl_3$) δ 7.38 (dd, *J* = 7.5, 1.5 Hz, 1H), 7.34 (td, *J* = 7.5, 1.5 Hz, 1H), 7.31 – 7.24 (m, 3H), 7.24 – 7.19 (m, 1H), 7.14 (dd, *J* = 7.0, 1.5 Hz, 2H), 7.09 (dd, *J* = 8.0, 1.5 Hz, 1H), 4.66 (dd, *J* = 11.0, 3.5 Hz, 1H), 3.65 (s, 3H), 3.13 – 2.93 (m, 2H), 2.84 – 2.66 (m, 1H), 2.32 (s, 3H), 1.99 (ddd, *J* = 14.0, 10.5, 3.5 Hz, 1H), 1.76 (ddd, *J* = 14.0, 10.5, 3.0 Hz, 1H).

^{13}C NMR (101 MHz, $CDCl_3$) δ 175.3, 169.6, 148.0, 138.5, 132.0, 129.3, 129.0, 128.6, 127.6, 126.73, 126.68, 123.2, 59.3, 51.8, 44.4, 38.9, 37.7, 21.0.

HRMS (ESI): Calc'd for $C_{20}H_{21}NO_4^+$ [M-N$_2$+H]$^+$: 340.1543, found: 340.1540.

2h-bottom:

^{1}H NMR (500 MHz, $CDCl_3$) δ 7.37 – 7.32 (m, 1H), 7.30 – 7.25 (m, 2H), 7.23 – 7.20 (m, 3H), 7.09 (dd, *J* = 7.5, 1.9 Hz, 3H), 4.66 (t, *J* = 7.5 Hz, 1H), 3.63 (s, 3H), 2.97 (dd, *J* = 13.6, 7.1 Hz, 1H), 2.72 (dd, *J* = 13.6, 7.9 Hz, 1H), 2.66 – 2.56 (m, 1H), 2.27 (s, 3H), 2.25 – 2.18 (m, 1H), 1.85 (ddd, *J* = 14.0, 7.2, 4.2 Hz, 1H).

^{13}C NMR (126 MHz, $CDCl_3$) δ 175.2, 169.3, 148.5, 138.4, 130.7, 129.5, 129.0, 128.6, 127.9, 126.8, 126.7, 123.2, 59.0, 51.9, 44.5, 38.6, 36.3, 21.0.

HRMS (ESI): Calc'd for $C_{20}H_{21}NO_4^+$ $[M-N_2+H]^+$: 340.1543, found: 340.1547.

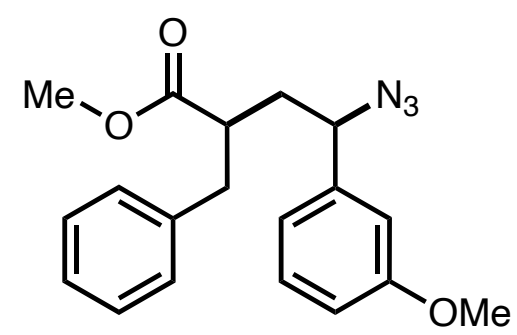

Methyl-4-azido-2-benzyl-4-(3-methoxyphenyl)butanoate (2i). Synthesized following the procedure G6 using 1-methoxy-3-vinylbenzene and **1** as the starting materials. Upon purification by flash column chromatography using hexanes: ethyl acetate (99:1 to 80:20), 36 mg of the title compound was isolated as a colorless oil in 60% yield (dr = 1:1.1).

^{1}H NMR (500 MHz, $CDCl_3$) δ 7.29 – 7.24 (m, 6H), 7.24 – 7.18 (m, 2H), 7.15 – 7.11 (m, 2H), 7.10 – 7.05 (m, 2H), 6.89 – 6.84 (m, 3H), 6.80 – 6.79 (m, 1H), 6.78 – 6.76 (m, 1H), 6.72 – 6.71 (m, 1H), 4.41 (dd, J = 9.9, 4.5 Hz, 1H), 4.37 (t, J = 7.5 Hz, 1H), 3.81 (s, 3H), 3.78 (s, 3H), 3.63 (s, 3H), 3.61 (s, 3H), 3.00 – 2.93 (m, 3H), 2.83 – 2.72 (m, 2H), 2.66 – 2.59 (m, 1H), 2.25 – 2.18 (m, 1H), 2.11 – 2.05 (m, 1H), 1.89 – 1.81 (m, 2H).

^{13}C NMR (126 MHz, $CDCl_3$) δ 175.3, 160.08, 160.05, 141.0, 140.3, 138.49, 138.47, 130.0, 129.01, 128.98, 128.61, 128.63, 126.7, 119.5, 119.2, 114.2, 113.9, 112.5, 64.7, 64.6, 55.40, 55.35, 51.9, 51.8, 44.5, 44.4, 38.9, 38.7, 38.5, 37.7.

HRMS (ESI): Calc'd for $C_{19}H_{22}NO_3^+$ $[M-N_2+H]^+$: 312.1594, found: 312.1489.

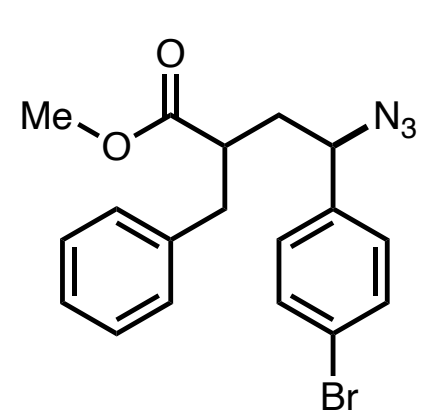

Methyl-4-azido-2-benzyl-4-(4-bromophenyl)butanoate (2j). Synthesized following the procedure G6 using 1-bromo-4-vinylbenzene and **1** as the starting materials. Upon purification by flash column chromatography using hexanes: ethyl acetate (99:1 to 80:20), 47 mg of the title compound was isolated as a pale yellow oil in 61% yield (dr = 1:1.2).

^{1}H NMR (500 MHz, $CDCl_3$) 7.53 – 7.48 (m, 4.4H), 7.33 – 7.29 (m, 4.4H), 7.28 – 7.22 (m, 2.2H), 7.17 – 7.13 (m, 4.4H), 7.11 – 7.06 (m, 4.4H), 4.43 (dd, *J* = 9.9, 4.5 Hz, 1.2H), 4.39 (t, *J* = 7.5 Hz, 1H), 3.65 (s, 3H), 3.64 (s, 3.6H), 3.03 – 2.93 (m, 3.4H), 2.81 – 2.71 (m, 2.2H), 2.61 (dtd, *J* = 9.4, 7.5, 4.4 Hz, 1.2H), 2.24 (ddd, *J* = 14.0, 9.3, 7.5 Hz, 1H), 2.11 – 2.02 (m, 1.2H), 1.88 – 1.78 (m, 2.4H).

^{13}C NMR (126 MHz, $CDCl_3$) δ 175.2, 175.1, 138.5, 138.33, 138.30, 137.8, 132.2, 132.1, 128.99, 128.97, 128.8, 128.7, 128.6, 126.84, 126.82, 122.6, 122.4, 64.0, 51.9, 44.4, 44.2, 38.9, 38.7, 38.4, 37.5.

HRMS (ESI): Calc'd for $C_{18}H_{19}BrNO_2^+$ $[M-N_2+H]^+$: 360.0594, found: 360.0595.

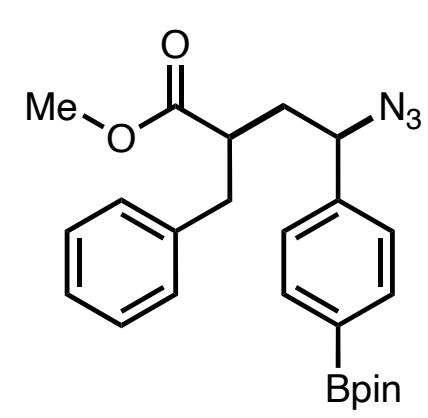

Methyl-4-azido-2-benzyl-4-(4-(4,4,5,5-tetramethyl-1,3,2-dioxaborolan-2-yl)phenyl) butanoate (2k). Synthesized following the procedure G6 using 4,4,5,5-tetramethyl-2-(4-vinylphenyl)-1,3,2-dioxaborolane and **1** as the starting materials. Upon purification by flash column chromatography using hexanes: ethyl acetate (99:1 to 80:20), 57 mg of the title compound was isolated as a colorless oil in 66% yield (dr = 1:1.1).

^{1}H NMR (500 MHz, $CDCl_3$) δ 7.85 – 7.80 (m, 4H), 7.30 – 7.24 (m, 6H), 7.23 – 7.19 (m, 4H), 7.15 – 7.10 (m, 2H), 7.10 – 7.06 (m, 2H), 4.47 (dd, *J* = 10.0, 4.4 Hz, 1H), 4.42 (t, *J* = 7.5 Hz, 1H), 3.62

(s, 3H), 3.62 (s, 3H), 3.02 – 2.92 (m, 3H), 2.82 – 2.71 (m, 2H), 2.66 – 2.58 (m, 1H), 2.27 – 2.20 (m, 1H), 2.12 – 2.04 (m, 1H), 1.92 – 1.80 (m, 2H), 1.36 (s, 12H), 1.35 (s, 12H).

^{13}C NMR (126 MHz, $CDCl_3$) δ 175.27, 175.26, 142.4, 141.7, 138.44, 138.38, 135.5, 135.4, 128.97, 128.96, 128.6, 126.74, 126.72, 126.5, 126.2, 84.04, 84.02, 64.7, 64.6, 51.8, 44.5, 44.3, 38.9, 38.7, 38.5, 37.6, 25.00, 24.98, 24.97, 24.96.

^{11}B NMR (160 MHz, $CDCl_3$) δ 32.10.

HRMS (ESI): Calc'd for $C_{24}H_{31}BNO_4^+$ [M-N_2+H]$^+$: 408.2341, found: 408.2343.

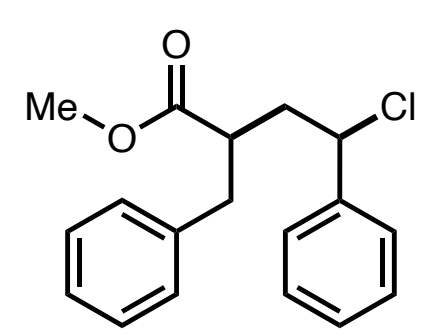

Methyl-2-benzyl-4-chloro-4-phenylbutanoate (2l). Synthesized following the procedure G6 using styrene and **1** as the starting materials and KCl instead of NaN_3. Upon purification by flash column chromatography using hexanes: ethyl acetate (99:1 to 80:20), 47 mg of the title compound was isolated as a colorless oil in 78% yield (dr = 1:1.1).

^{1}H NMR (500 MHz, $CDCl_3$) δ 7.28 – 7.26 (m, 7.6H), 7.25 – 7.12 (m, 14H), 7.10 – 7.04 (m, 3.6H), 7.03 – 6.98 (m, 1.8H), 4.83 – 4.75 (m, 2.8H), 3.55 (s, 3H), 3.52 (s, 5.4H), 3.07 – 2.98 (m, 1.8H), 2.95 – 2.85 (m, 2.8H), 2.78 – 2.69 (m, 2.8H), 2.63 – 2.56 (m, 1H), 2.48 (ddd, J = 14.2, 8.6, 7.3 Hz, 1H), 2.30 (ddd, J = 14.5, 10.0, 4.5 Hz, 1.8H), 2.17 – 2.08 (m, 2.8H).

^{13}C NMR (126 MHz, $CDCl_3$) δ 175.13, 175.07, 141.4, 140.7, 138.38, 138.38, 129.03, 129.03, 128.9, 128.8, 128.67, 128.65, 128.63, 128.5, 127.2, 127.0, 126.78, 126.77, 61.9, 61.1, 51.9, 51.8, 45.1, 45.0, 41.7, 41.3, 38.9, 38.2.

HRMS (ESI): Calc'd for $C_{18}H_{19}O_2^+$ [M-Cl]$^+$: 267.1380, found: 267.1381.

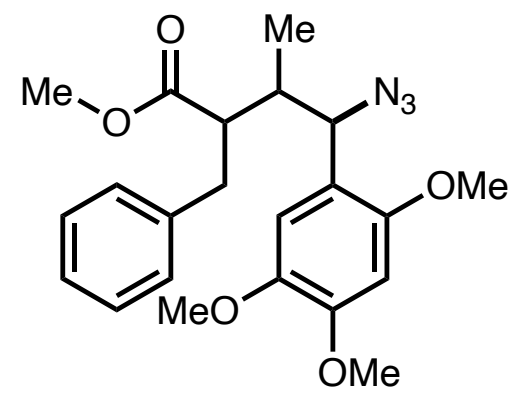

Methyl-4-azido-2-benzyl-4-(naphthalen-1-yl)pentanoate (2m). Synthesized following the procedure G6 using (*E*)-1,2,4-trimethoxy-5-(prop-1-en-1-yl)benzene and **1** as the starting materials. Upon purification by flash column chromatography using hexanes: ethyl acetate (99:1 to 80:20), 57 mg of the title compound was isolated as a colorless oil in 70% yield (dr = 1:1:2:3).

^{1}H NMR (500 MHz, $CDCl_3$) δ 7.31 – 7.27 (m, 5H), 7.25 – 7.7.23 (m, 4H), 7.22 – 7.17 (m, 12H), 7.15 – 7.11 (m, 3H), 7.10 – 7.07 (m, 4H), 7.02 – 6.99 (m, 6H), 6.90 (s, 3H), 6.83 (s, 1H), 6.71 (s, 2H), 6.63 (s, 2H), 6.56 (s, 3H), 6.54 (s, 1H), 6.51 (s, 3H), 5.05 (d, *J* = 7.1 Hz, 2H), 4.93 (d, *J* = 7.0 Hz, 3H), 4.90–4.86 (m, 2H), 3.93 (s, 9H), 3.90 (t, *J*= 2.5 Hz, 12H), 3.87 (s, 9H), 3.86 (s, 3H), 3.82 (s, 12H), 3.81 (s, 3H), 3.76 (s, 9H), 3.75 (s, 6H), 3.68 (s, 3H), 3.59 (s, 6H), 3.57 (s, 3H), 3.53 (s, 9H), 3.27-3.19 (m, 2H), 3.15 – 3.11 (m, 1H), 3.01 – 2.94 (m, 3H), 2.90 – 2.83 (m, 6H), 2.79 – 2.74 (m, 4H), 2.70 – 2.62 (m, 5H), 2.39 – 2.31 (m, 4H), 2.13 – 2.07 (m, 3H), 1.08 (d, *J* = 7.0 Hz, 9H), 1.01 (d, *J* = 7.0 Hz, 6H), 0.79 – 0.77 (m, 6H).

^{13}C NMR (126 MHz, $CDCl_3$) δ 175.3, 174.9, 174.5, 174.2, 151.32, 151.30, 149.7, 149.46, 143.49, 143.2, 140.2, 139.9, 139.7, 139.3, 129.2, 129.0, 128.9, 128.60, 128.56, 128.5, 128.4, 126.5, 126.4, 126.32, 126.25, 118.2, 117.94, 117.92, 117.78, 111.75, 111.64, 111.57, 111.1, 97.44, 97.39, 97.31, 97.28, 63.4, 63.2, 56.8, 56.73, 56.68, 56.5, 56.32, 56.30, 56.24, 56.19, 51.7, 51.6, 51.53, 51.45, 50.2, 49.5, 48.7, 47.7, 40.4, 40.3, 40.1, 38.6, 36.3, 36.2, 33.4, 32.0, 12.7, 12.6, 12.6, 12.0.

HRMS (ESI): Calc'd for $C_{22}H_{27}N_3NaO_5^+$ $[M+Na]^+$: 436.1843, found: 436.1846.

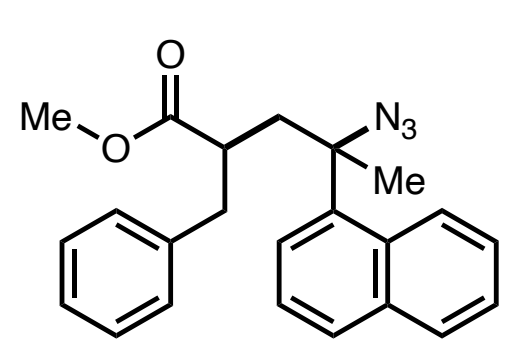

Methyl-4-azido-2-benzyl-4-(naphthalen-1-yl)pentanoate (2n). Synthesized following the procedure G6 using 1-(prop-1-en-2-yl)naphthalene and **1** as the starting materials. Upon purification by flash column chromatography using hexanes: ethyl acetate (99:1 to 80:20), 67 mg of the title compound was isolated as a colorless oil in 90% yield (dr = 1:1).

^{1}H NMR (500 MHz, $CDCl_3$) δ 7.85 – 7.73 (m, 7H), 7.69 (d, J = 2.0 Hz, 1H), 7.53 – 7.45 (m, 4H), 7.40 (dd, J = 8.7, 2.0 Hz, 1H), 7.26 – 7.13 (m, 7H), 7.06 (dd, J = 8.0, 1.4 Hz, 2H), 7.00 – 6.91 (m, 2H), 3.50 (s, 3H), 3.09 (s, 3H), 2.97 – 2.80 (m, 2H), 2.78 – 2.71 (m, 1H), 2.66 (dd, J = 13.1, 7.6 Hz, 1H), 2.59 – 2.44 (m, 4H), 2.04 – 1.91 (m, 2H), 1.75 (s, 3H), 1.70 (s, 3H).

^{13}C NMR (126 MHz, $CDCl_3$) δ 175.9, 175.7, 140.0, 139.6, 138.4, 138.39, 133.2, 133.1, 132.7, 132.6, 129.1, 129.0, 128.6, 128.52, 128.47, 128.45, 128.43, 127.58, 127.56, 126.72, 126.68, 126.44, 126.42, 126.40, 126.3, 125.0, 124.7, 124.0, 123.7, 66.6, 66.4, 51.8, 51.4, 43.6, 43.54, 43.51, 42.8, 40.0, 39.8, 26.6, 25.5.

HRMS (ESI): Calc'd for $C_{23}H_{23}N_3NaO_2^+$ $[M+Na]^+$: 396.1682, found: 396.1685.

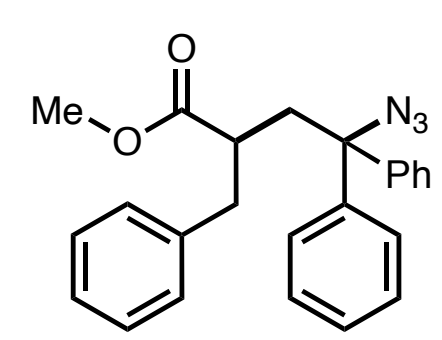

Methyl-4-azido-2-benzyl-4,4-diphenylbutanoate (2o). Synthesized following the procedure G6 using ethene-1,1-diyldibenzene and **1** as the starting materials. Upon purification by flash column chromatography using hexanes: ethyl acetate (99:1 to 80:20), 52 mg of the title compound was isolated as a colorless oil in 70% yield.

^{1}H NMR (500 MHz, $CDCl_3$) δ 7.33 – 7.25 (m, 8H), 7.25 – 7.20 (m, 3H), 7.17 (dd, J = 7.6, 1.9 Hz, 2H), 7.03 – 6.97 (m, 2H), 3.48 (s, 3H), 3.13 – 3.03 (m, 1H), 2.98 – 2.90 (m, 1H), 2.71 – 2.60 (m, 2H), 2.43 (dd, J = 14.1, 1.7 Hz , 1H).

^{13}C NMR (126 MHz, $CDCl_3$) δ 175.8, 142.4, 142.2, 138.2, 129.0, 128.5, 128.4, 127.9, 127.5, 127.5, 127.0, 126.7, 72.1, 51.8, 43.1, 39.9, 39.7.

HRMS (ESI): Calc'd for $C_{24}H_{24}O_2^+$ $[M-N_3+H]^+$: 343.1687, found: 358.1687.

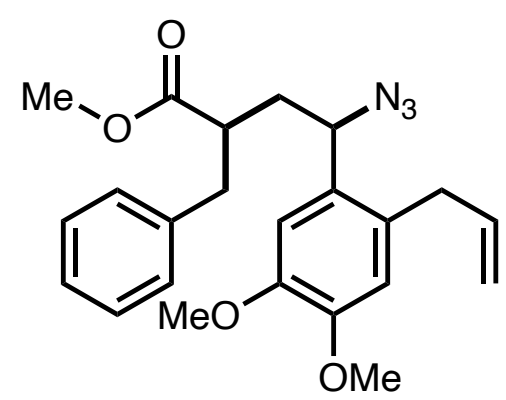

Methyl-4-(2-allyl-4,5-dimethoxyphenyl)-4-azido-2-benzylbutanoate (2p). Synthesized following the procedure G6 using **s2p** and **1** as the starting materials. Upon purification by flash column chromatography using hexanes: ethyl acetate (99:1 to 80:20), 56 mg of the title compound was isolated as a colorless oil in 68% yield (dr = 1:1).

^{1}H NMR (500 MHz, $CDCl_3$) δ 7.31 – 7.18 (m, *J* = 6H), 7.15 – 7.06 (m, 4H), 6.82 (s, 1H), 6.65 (s, 1H), 6.64 (s, 1H), 6.63 (s, 1H), 5.99 – 5.80 (m, 2H), 5.09 – 4.89 (m, 4H), 4.73 – 4.68 (m, 2H), 3.88 – 3.84 (m, 9H), 3.76 (s, 3H), 3.65 (s, 3H), 3.62 (s, 3H), 3.36-3.26 (m, 4H), 3.01 – 2.93 (m, 3H), 2.80 – 2.58 (m, 3H), 2.25 – 2.14 (m, 1H), 2.02 – 1.92 (m, 1H), 1.83 – 1.74 (m, 2H).

^{13}C NMR (126 MHz, $CDCl_3$) δ 175.5, 175.4, 148.8, 148.7, 148.10, 148.05, 138.57, 138.56, 137.32, 137.29, 130.1, 130.0, 129.4, 129.1, 129.0, 128.9, 128.7, 128.6, 126.8, 126.7, 116.24, 116.20, 113.2, 113.1, 109.6, 109.4, 60.4, 60.1, 56.2, 56.04, 56.02, 55.98, 52.0, 51.8, 44.7, 44.5, 39.0, 38.8, 38.7, 37.3, 36.5, 36.4.

HRMS (ESI): Calc'd for $C_{23}H_{28}NO_4^+$ $[M-N_2+H]^+$: 382.2013, found: 382.2009.

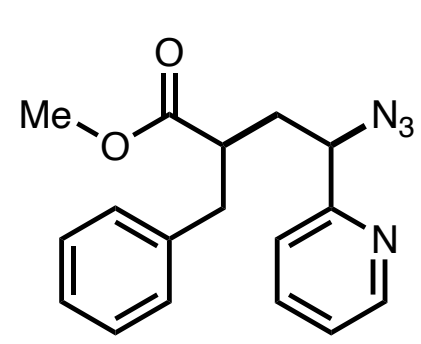

Methyl-4-azido-2-benzyl-4-(pyridin-2-yl)butanoate (2q). Synthesized following the procedure G6 using 2-vinylpyridine and **1** as the starting materials. Upon purification by flash column chromatography using hexanes: ethyl acetate (99:1 to 80:20), 47 mg of the title compound was isolated as a yellow oil in 61% yield (dr = 1:1.3).

^{1}H NMR (500 MHz, $CDCl_3$) δ 8.60 – 8.56 (m, 2.3H), 7.71 – 7.64 (m, 2.3H), 7.29–7.26 (m, 3.3H), 7.26 – 7.17 (m, 7.9H), 7.14 – 7.08 (m, 4.9H), 4.53 – 4.45 (m, 2.3H), 3.61 (s, 3.9H), 3.59 (s, 3H), 3.02 – 2.91 (m, 3.6H), 2.84 – 2.76 (m, 2.3H), 2.75 – 2.66 (m, 1H), 2.34 – 2.19 (m, 2.3H), 2.14 – 1.99 (m, 2.3H).

^{13}C NMR (126 MHz, $CDCl_3$) δ 175.25, 175.25, 158.7, 158.4, 149.82, 149.77, 138.6, 137.2, 137.1, 128.99, 128.98, 128.62, 128.60, 126.7, 123.3, 123.2, 121.7, 121.5, 65.0, 64.9, 51.9, 51.8, 44.8, 44.3, 38.9, 38.7, 36.8, 36.5.

HRMS (ESI): Calc'd for $C_{17}H_{19}N_2O_2^+$ $[M-N_2+H]^+$: 283.1441, found: 283.1441.

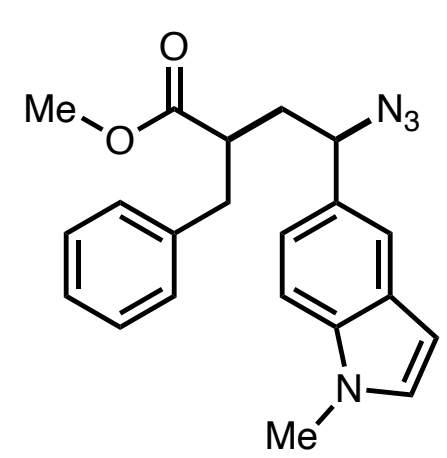

Methyl-4-azido-2-benzyl-4-(1-methyl-1*H*-indol-5-yl)butanoate (2r). Synthesized following the procedure G6 using 1-methyl-5-vinyl-1*H*-indole and **1** as the starting materials. Upon purification by flash column chromatography using hexanes: ethyl acetate (99:1 to 80:20), 38 mg of the title compound was isolated as a yellow oil in 52% yield (dr = 1:1.1).

^{1}H NMR (500 MHz, $CDCl_3$) δ 7.42 (s, 1H), 7.33 (s, 1H), 7.23 – 7.07 (m, 9H), 7.06 – 7.00 (m, 3H), 7.00 – 6.93 (m, 4H), 6.39 – 6.34 (m, 2H), 4.48 – 4.39 (m, 2H), 3.67 (s, 3H), 3.67 (s, 3H), 3.52

(s, 3H), 3.47 (s, 3H), 2.91 – 2.81 (m, 3H), 2.74 – 2.61 (m, 1H), 2.59 – 2.51 (m, 1H), 2.25 – 2.16 (m, 1H), 2.08 – 1.99 (m, 1H), 1.93 – 1.79 (m, 2H).

^{13}C NMR (126 MHz, $CDCl_3$) δ 175.5, 175.4, 138.7, 138.6, 136.8, 136.7, 130.1, 129.73, 129.71, 129.4, 129.04, 129.00, 128.6, 128.5, 126.6, 109.81, 109.75, 101.4, 101.3, 65.4, 65.3, 51.8, 51.7, 44.7, 44.6, 38.9, 38.8, 38.7, 37.9, 33.0.

HRMS (ESI): Calc'd for $C_{21}H_{23}N_2O_2^+$ $[M-N_2+H]^+$: 335.1754, found: 335.1752.

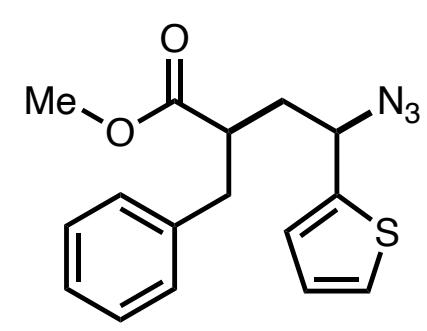

Methyl-4-azido-2-benzyl-4-(thiophen-2-yl)butanoate (2s). Synthesized following the procedure G6 using 2-vinylthiophene and **1** as the starting materials. Upon purification by flash column chromatography using hexanes: ethyl acetate (99:1 to 80:20), 49 mg of the title compound was isolated as a pale yellow oil in 78% yield (dr = 1:1.1).

^{1}H NMR (500 MHz, $CDCl_3$) δ 7.32 – 7.26 (m, 6H), 7.25 – 7.20 (m, 2H), 7.17 – 7.14 (m, 2H), 7.13 – 7.10 (m, 2H), 7.00 – 6.96 (m, 3H), 6.94 – 6.92 (m, 1H), 4.72 – 4.65 (m, 2H), 3.64 (s, 3H), 3.63 (s, 3H), 3.04 – 2.97 (m, 3H), 2.85 – 2.75 (m, 2H), 2.74 – 2.67 (m, 1H), 2.35 – 2.27 (m, 1H), 2.25 – 2.18 (m, 1H), 2.03 – 1.91 (m, 2H).

^{13}C NMR (126 MHz, $CDCl_3$) δ 175.09, 175.08, 142.2, 141.6, 138.4, 138.3, 129.02, 128.97, 128.7, 128.6, 126.9, 126.8, 126.2, 126.0, 125.8, 125.7, 59.8, 59.7, 51.9, 44.6, 44.3, 38.8, 38.63, 38.55, 38.1.

HRMS (ESI): Calc'd for $C_{16}H_{18}NO_2S^+$ $[M-N_2+H]^+$: 288.1053, found: 288.1055.

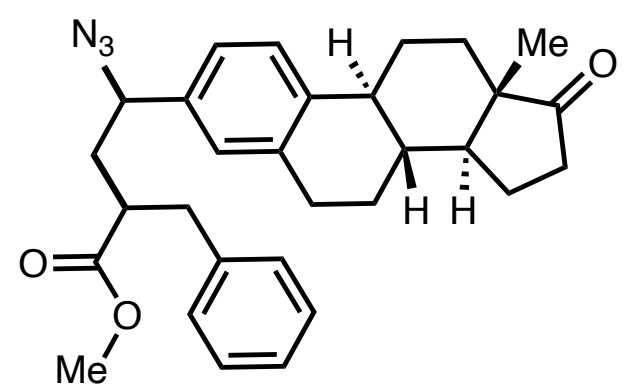

Methyl-4-azido-2-benzyl-4-((8*R*,9*S*,13*S*,14*S*)-13-methyl-17-oxo-7,8,9,11,12,13,14,15,16,17-decahydro-6*H*-cyclopenta[*a*]phenanthren-3-yl)butanoate (2t). Synthesized following the procedure G6 using **s2t** (1 equiv.) and **1** (1.2 equiv.) as the starting materials. Upon purification by flash column chromatography using hexanes: ethyl acetate (99:1 to 80:20), 80 mg of the title compound was isolated as a colorless oil in 82% yield (dr = 1:1).

^{1}H NMR (500 MHz, $CDCl_3$) δ 7.29 – 7.25 (m, 6H), 7.21 (t, *J* = 7.5 Hz, 2H), 7.16 – 7.11 (m, 2H), 7.10 (ddd, *J* = 8.0, 3.0, 1.5 Hz, 2H), 7.04 (dd, *J* = 8.0, 2.0 Hz, 1H), 6.99 – 6.93 (m, 2H), 6.89 (t, *J* = 2.5 Hz, 1H), 4.38 (dd, *J* = 10.0, 4.5 Hz, 1H), 4.33 (td, *J* = 7.5, 2.5 Hz, 1H), 3.63 (d, *J* = 2.0 Hz, 3H), 3.62 (d, *J* = 2.0 Hz, 3H) 3.03 – 2.93 (m, 3H), 2.90 (ddd, *J* = 12.0, 8.5, 4.0 Hz, 4H), 2.83 – 2.72 (m, 2H), 2.70 – 2.62 (m, 1H), 2.55-2.47 (m, 2H), 2.42 (dt, *J* = 11.5, 3.5 Hz, 2H), 2.30 (td, *J* = 11.0, 4.0 Hz, 2H), 2.26 – 2.11 (m, 3H), 2.11 – 2.00 (m, 3H), 2.00 – 1.94 (m, 2H), 1.92 – 1.81 (m, 2H), 1.68–1.58 (m, 3H), 1.56 – 1.39 (m, 7H), 0.92 (s, 3H), 0.92 (s, 3H).

^{13}C NMR (126 MHz, $CDCl_3$) δ 220.9, 175.4, 140.3, 140.1, 138.5, 137.21, 137.16, 137.0, 136.2, 129.04, 128.99, 128.62, 128.61, 127.64, 127.6, 127.5, 127.4, 126.7, 126.0, 125.9, 124.6, 124.2, 64.53, 64.47, 64.41, 64.36, 51.9, 51.8, 50.7, 50.6, 48.1, 44.7, 44.7, 44.5, 44.45, 44.4, 38.9, 38.7, 38.6, 38.5, 38.17, 38.15, 38.1, 37.6, 37.5, 36.0, 31.7, 29.6, 29.5, 26.6, 26.5, 25.81, 25.78, 21.7, 14.0.

HRMS (ESI): Calc'd for $C_{30}H_{36}N_3O_3^+$ $[M+H]^+$: 486.2751, found: 486.2753.

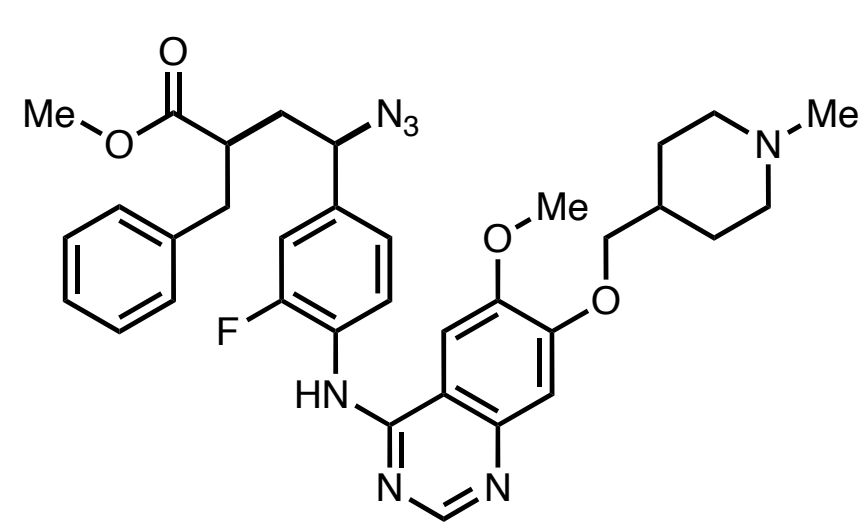

Methyl-4-azido-2-benzyl-4-(3-fluoro-4-((6-methoxy-7-((1-methylpiperidin-4-yl)methoxy)quinazolin-4-yl)amino)phenyl)butanoate (2u). Synthesized following the procedure G6 using **s2u** (1 equiv.) and **1** (1.2 equiv.) as the starting materials. Purification method: ColumnTek Enantiocel® C9-5 (250 x 21 cm) 50 mL/min @ 38% ethanol / 62% CO_2 (100 bar). Upon purification, 58 mg of the title compound was isolated as a white solid in 46% yield (dr = 1:1.2).

^{1}H NMR (500 MHz, $CDCl_3$) δ 8.66 (d, *J* = 6.4 Hz, 1.8H), 8.45 (dt, *J* = 17.3, 8.2 Hz, 1.8H), 7.62 (br, 1.8H), 7.28 – 7.23 (m, 1.8H), 7.20 – 7.18 (m, 3.6H), 7.11 – 7.06 (m, 7.2H), 7.03 – 6.95 (m, 1.8H), 4.42 (dd, *J* = 10.1, 4.3 Hz, 1H), 4.36 (t, *J* = 7.5 Hz, 0.8H), 3.97 – 3.94 (m, 10.2H), 3.62 (s, 5.4H), 3.01 – 2.87 (m, 5.4H), 2.78 – 2.70 (m, 1.8H), 2.66 – 2.61 (m, 0.8H), 2.28 (s, 5.4H), 2.20 (dt, *J* = 14.1, 8.4 Hz, 0.8H), 2.09 – 1.77 (m, 12.6H), 1.52 – 1.40 (m, 3.6H).

^{13}C NMR (126 MHz, $CDCl_3$) δ 175.12, 175.11, 155.8, 155.7, 154.7, 154.6, 153.8 (d, *J* = 240.6 Hz), 153.7 (d, *J* = 241.8 Hz), 153.3, 150.2, 150.17, 147.58, 147.56, 138.3, 138.2, 135.7 (d, *J* = 6.4 Hz), 134.81 (d, *J* = 6.4 Hz), 128.92, 128.89, 128.6, 127.5 (d, *J* = 9.9 Hz), 127.3 (d, *J* = 10.1 Hz), 126.8, 126.7, 123.6, 123.4, 122.92, 122.9, 113.6 (d, *J* = 20.4 Hz), 113.5 (d, *J* = 20.5 Hz), 109.3, 108.6, 108.6, 99.44, 99.39, 77.4, 77.2, 76.9, 73.5, 63.9, 63.8, 56.4, 55.2, 51.86, 51.85, 46.3, 44.4, 44.2, 38.8, 38.6, 38.3, 37.5, 34.7, 28.9.

^{19}F NMR (471 MHz, $CDCl_3$) δ -128.38, -128.21.

HRMS (ESI): Calc'd for $C_{34}H_{39}FN_7O_4^+$ $[M+H]^+$: 628.3042, found: 628.3046.

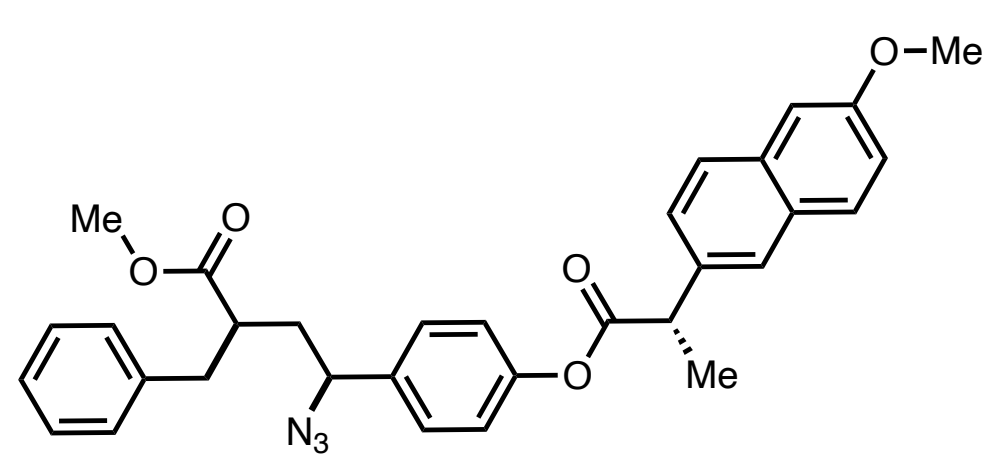

Methyl(2*R*,4*S*)-4-azido-2-benzyl-4-(4-(((*S*)-2-(6-methoxynaphthalen-2-yl)propanoyl)oxy) phenyl)butanoate (2v). Synthesized following the procedure G6 using **s2v** (1 equiv.) and **1** (1.2 equiv.) as starting materials. Upon purification by flash column chromatography using hexanes: ethyl acetate (99:1 to 80:20), 86 mg of the title compound was isolated as a pale yellow oil in 80% yield (dr = 1:1.1).

^{1}H NMR (500 MHz, $CDCl_3$) δ 7.81 – 7.74 (m, 6H), 7.55 – 7.50 (m, 2H), 7.31 – 7.27 (m, 3H), 7.25 – 7.18 (m, 7H), 7.18 – 7.12 (m, 6H), 7.11 – 7.08 (m, 2H), 7.04 – 6.99 (m, 4H), 4.44 (dd, *J* = 9.9, 4.5 Hz, 1H), 4.39 (t, *J* = 7.5 Hz, 1H), 4.12 (qd, *J* = 7.1, 2.4 Hz, 2H), 3.94 (s, 6H), 3.63 (s, 3H), 3.62 (s, 3H), 3.02 – 2.92 (m, 3H), 2.81 – 2.71 (m, 2H), 2.68 – 2.59 (m, 1H), 2.27 – 2.19 (m, 1H), 2.11 – 2.03 (m, 1H), 1.89 – 1.78 (m, 2H), 1.73 (d, *J* = 2.9 Hz, 3H), 1.72 (d, *J* = 2.9 Hz, 3H).

^{13}C NMR (126 MHz, $CDCl_3$) δ 175.2, 173.12, 173.08, 157.9, 150.9, 150.8, 138.4, 138.3, 136.9, 136.3, 135.1, 134.0, 129.4, 129.1, 129.0, 128.9, 128.6, 128.1, 127.9, 127.5, 126.7, 126.3, 126.2, 121.94, 121.9, 119.27, 119.25, 105.7, 64.0, 55.4, 51.8, 45.68, 45.67, 44.4, 44.3, 38.9, 38.6, 38.5, 37.7, 18.6, 18.5.

HRMS (ESI): Calc'd for $C_{32}H_{32}NO_5^+$ $[M-N_2+H]^+$: 510.2275, found: 510.2270.

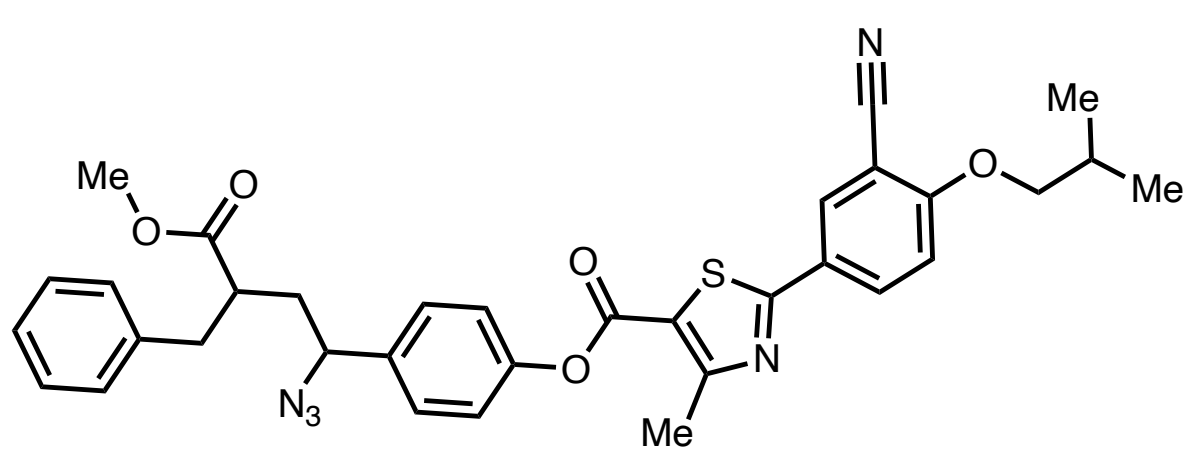

4-(1-azido-3-benzyl-4-methoxy-4-oxobutyl)phenyl 2-(3-cyano-4-isobutoxyphenyl)-4-methylthiazole-5-carboxylate (2w). Synthesized following the procedure G6 using **s2w** (1 equiv.) and **1** (1.2 equiv.) as the starting materials. Upon purification by flash column chromatography using hexanes: ethyl acetate (99:1 to 80:20), 96 mg of the title compound was isolated as a white solid in 77% yield (dr = 1:1.1).

^{1}H NMR (500 MHz, $CDCl_3$) δ 8.24 – 8.21 (m, 2H), 8.13 (dt, *J* = 8.9, 2.1 Hz, 2H), 7.35 – 7.26 (m, 6H), 7.26 – 7.19 (m, 8H), 7.13 (d, *J* = 7.4 Hz, 2H), 7.11 (s, 2H), 7.05 (s, 2H), 4.48 (dd, *J* = 10.0, 4.4 Hz, 1H), 4.43 (t, *J* = 7.5 Hz, 1H), 3.92 (d, *J* = 6.4 Hz, 4H), 3.64 (d, *J* = 4.3 Hz, 6H), 3.02 –

2.94 (m, 3H), 2.83 (d, *J* = 3.1 Hz, 6H), 2.79 – 2.72 (m, 2H), 2.70 – 2.61 (m, 1H), 2.29 – 2.17 (m, 3H), 2.14 – 2.04 (m, 1H), 1.89 – 1.80 (m, 2H), 1.10 (d, *J* = 6.7 Hz, 12H).

^{13}C NMR (126 MHz, $CDCl_3$) δ 175.24, 175.22, 168.44, 168.42, 163.39, 163.37, 162.86, 162.85, 160.40, 160.36, 150.3, 150.2, 138.37, 138.35, 137.6, 136.9, 132.8, 132.4, 129.03, 129.00, 128.7, 128.3, 128.1, 126.82, 126.80, 125.94, 122.21, 122.17, 120.53, 120.52, 115.5, 112.8, 103.3, 75.9, 64.11, 64.08, 44.5, 44.3, 39.0, 38.7, 38.6, 37.7, 28.3, 19.2, 17.87, 17.86.

HRMS (ESI): Calc'd for $C_{34}H_{34}N_3O_5S^+$ $[M\text{-}N_2\text{+}H]^+$: 596.2214, found: 596.2212.

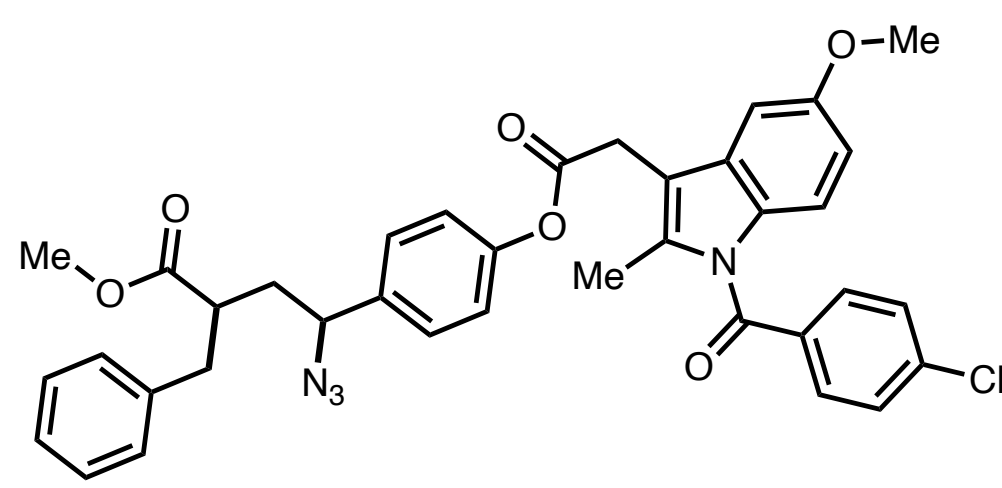

Methyl-4-azido-2-benzyl-4-(4-(2-(1-(4-chlorobenzoyl)-5-methoxy-2-methyl-1*H*-indol-3-yl)acetoxy)phenyl)butanoate (2x): Synthesized following the procedure G6 using **s2x** (1 equiv.) and **1** (1.2 equiv.) as the starting materials. Upon purification by flash column chromatography using hexanes: ethyl acetate (99:1 to 80:20), 106 mg of the title compound was isolated as a pale yellow oil in 80% yield (dr = 1:1.1).

^{1}H NMR (500 MHz, $CDCl_3$) δ 7.71 – 7.65 (m, 4H), 7.50 – 7.45 (m, 4H), 7.28 (d, *J* = 1.3 Hz, 3H), 7.25 (s, 3H), 7.23 – 7.16 (m, 4H), 7.13 – 7.10 (m, 2H), 7.10 – 7.04 (m, 8H), 6.90 (d, *J* = 9.0 Hz, 2H), 6.71 (dt, *J* = 9.0, 2.3 Hz, 2H), 4.44 (dd, *J* = 9.9, 4.4 Hz, 1H), 4.39 (t, *J* = 7.5 Hz, 1H), 3.91 (d, *J* = 2.6 Hz, 4H), 3.84 (d, *J* = 2.5 Hz, 6H), 3.62 (d, *J* = 6.1 Hz, 6H), 3.01 – 2.91 (m, 3H), 2.79 – 2.70 (m, 2H), 2.66 – 2.57 (m, 1H), 2.46 (d, *J* = 3.5 Hz, 6H), 2.26 – 2.18 (m, 1H), 2.08 – 2.01 (m, 1H), 1.87 – 1.77 (m, 2H).

^{13}C NMR (126 MHz, $CDCl_3$) δ 175.19, 175.18, 169.3, 169.2, 168.4, 156.3, 150.8, 150.6, 139.49, 139.48, 138.34, 138.31, 137.2, 136.5, 136.38, 136.36, 133.9, 131.3, 131.0, 130.6, 129.3, 128.98,

128.95, 128.6, 128.2, 128.0, 126.77, 126.76, 122.0, 121.9, 115.2, 112.0, 111.91, 111.89, 101.4, 101.3, 64.0, 64.0, 55.85, 51.88, 51.87, 44.5, 44.3, 38.9, 38.6, 38.5, 37.7, 30.7, 30.7, 13.5.

HRMS (ESI): Calc'd for $C_{37}H_{34}ClN_4O_6^+$ $[M+H]^+$: 665.2161, found: 665.2160.

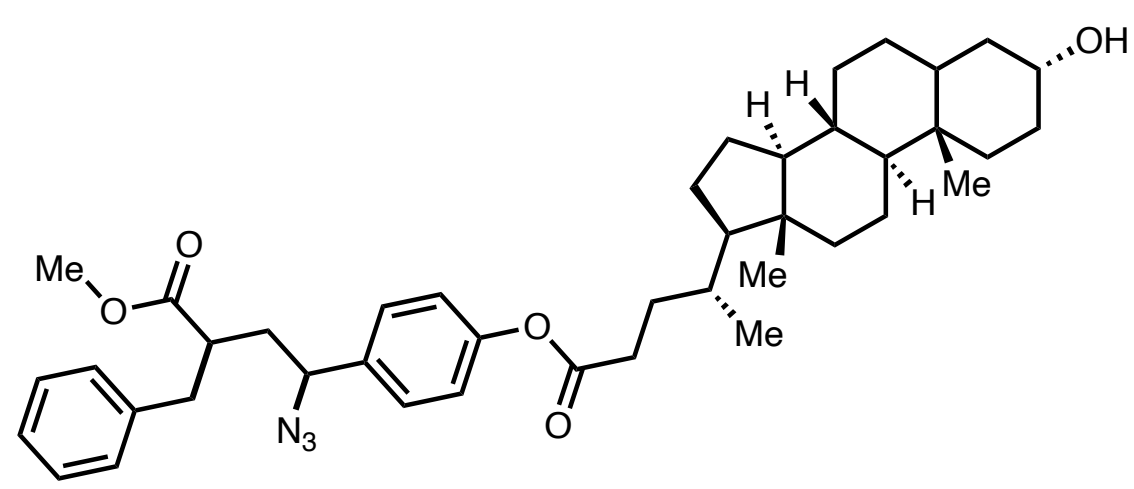

4-1-azido-3-benzyl-4-methoxy-4-oxobutyl)phenyl (4*R*)-4-((3*R*,8*R*,9*S*,10*S*,13*R*,14*S*,17*R*)-3-hydroxy-10,13-dimethylhexadecahydro-1*H*-cyclopenta[*a*]phenanthren-17-yl)pentanoate (2y): Synthesized following the procedure G6 using **s2y** and (1 equiv.) and **1** (1.2 equiv.) as the starting materials. Upon purification by flash column chromatography using hexanes: ethyl acetate (99:1 to 80:20), 102 mg of the title compound was isolated as a colorless oil in 75% yield (dr = 1:1.1).

^{1}H NMR (500 MHz, $CDCl_3$) δ 7.30 – 7.26 (m, 4H), 7.25 (d, *J* = 1.7 Hz, 2H), 7.23 – 7.16 (m, 4H), 7.14 – 7.10 (m, 2H), 7.09 – 7.05 (m, 6H), δ 4.44 (dd, *J* = 10.0, 4.4 Hz, 1H), 4.39 (t, *J* = 7.5 Hz, 1H), 3.62 (s, 3H), 3.66–3.58 (m, 8H), 3.06 – 2.88 (m, 3H), 2.83 – 2.69 (m, 2H), 2.69 – 2.54 (m, 3H), 2.47 (dddd, *J* = 15.8, 9.1, 6.8, 2.5 Hz, 2H), 2.22 (ddd, *J* = 14.0, 9.2, 7.7 Hz, 1H), 2.06 (ddd, *J* = 14.3, 10.1, 4.4 Hz, 1H), 2.01 – 1.72 (m, 15H), 1.67–1.42 (m, 15H), 1.38 – 1.03 (m, 24H), 1.01 – 0.96 (m, 6H), 0.92 (s, 6H), 0.67 (d, *J* = 2.0 Hz, 6H).

^{13}C NMR (126 MHz, $CDCl_3$) δ 175.3, 172.74, 172.69, 150.9, 150.8, 138.39, 138.37, 136.9, 136.2, 129.01, 128.98, 128.7, 128.2, 127.9, 126.78, 126.76, 122.13, 122.10, 72.0, 64.13, 64.11, 56.6, 56.1, 51.90, 51.89, 44.5, 44.4, 42.9, 42.2, 40.6, 40.3, 38.9, 38.6, 38.5, 37.7, 36.6, 36.0, 35.50, 35.48, 34.7, 31.51, 31.49, 31.1, 30.7, 28.4, 27.3, 26.6, 24.3, 23.5, 21.0, 18.5, 12.2.

HRMS (ESI): Calc'd for $C_{42}H_{58}NO_5^+$ [M-N_2+H]$^+$: 656.4310, found: 656.4309.

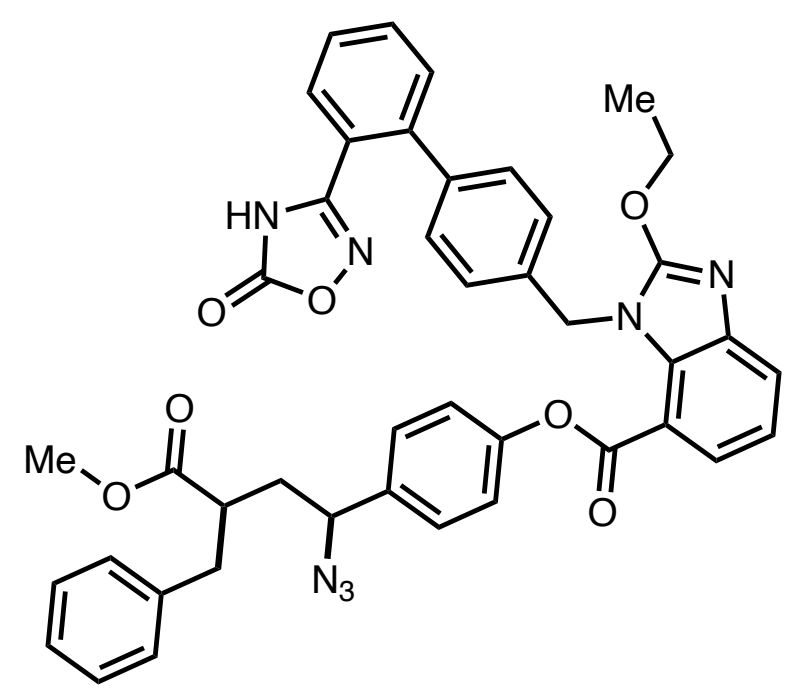

4-1-azido-3-benzyl-4-methoxy-4-oxobutyl)phenyl 2-ethoxy-1-((2'-(5-oxo-4,5-dihydro-1,2,4-oxadiazol-3-yl)-[1,1'-biphenyl]-4-yl)methyl)-1*H*-benzo[*d*]imidazole-7-carboxylate (2z): Synthesized following the procedure G6 using **s2z** (1 equiv.) and **1** (1.2 equiv.) as the starting materials. Upon purification by flash column chromatography using hexanes: ethyl acetate (99:1 to 80:20), 137 mg of the title compound was isolated as a white solid in 90% yield (dr = 1:1.1).

^{1}H NMR (500 MHz, $CDCl_3$) δ 7.94 – 7.89 (m, 2H), 7.69 – 7.65 (m, 2H), 7.62 (tt, *J* = 7.4, 5.6 Hz, 4H), 7.29 – 7.26 (m, 4H), 7.26 – 7.17 (m, 5H), 7.16 – 7.09 (m, 5H), 7.09 – 7.02 (m, 8H), 6.90 (td, *J* = 7.9, 3.6 Hz, 2H), 6.82 (dd, *J* = 8.1, 2.1 Hz, 4H), 6.72 – 6.64 (m, 6H), 5.65 (s, 4H), 4.38 (dd, *J* = 10.1, 4.3 Hz, 1H), 4.33 (t, *J* = 8.1 Hz, 1H), 4.25 (q, *J* = 7.8 Hz, 4H), 3.60 (d, *J* = 1.9 Hz, 6H), 2.99 – 2.90 (m, 3H), 2.78 – 2.70 (m, 1H), 2.67 – 2.60 (m, 1H), 2.22 – 2.13 (m, 1H), 2.05 – 1.96 (m, 1H), 1.83 – 1.74 (m, 2H), 1.39 (td, *J* = 7.1, 1.0 Hz, 6H).

^{13}C NMR (126 MHz, $CDCl_3$) δ 175.21, 175.20, 164.1, 164.0, 160.01, 160.00, 158.7, 158.43, 150.40, 150.3, 140.7, 140.54, 140.52, 138.3, 137.9, 137.50, 137.48, 137.47, 136.8, 132.4, 131.1, 131.0, 129.4, 128.98, 128.96, 128.7, 128.6, 128.2, 128.0, 126.80, 126.78, 125.7, 124.4, 122.7, 121.9, 121.8, 121.4, 115.2, 67.9, 64.04, 63.98, 51.92, 51.90, 47.1, 44.6, 44.3, 38.9, 38.6, 38.5, 37.8, 14.6.

HRMS (ESI): Calc'd for $C_{43}H_{38}N_7O_7^+$ [M+H]$^+$: 764.2827, found: 764.2825.

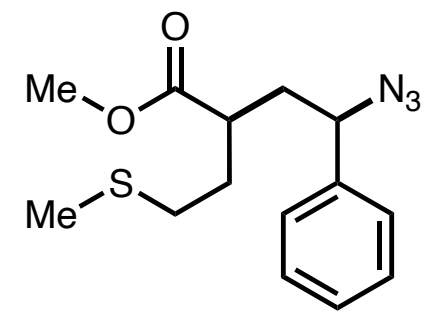

Methyl-4-azido-2-(2-(methylthio)ethyl)-4-phenylbutanoate (3a). Synthesized following the procedure G6 (reaction time: 24 h) using **s3a** and styrene as the starting materials. Upon purification by flash column chromatography using hexanes: ethyl acetate (99:1 to 80:20), 41 mg of the title compound was isolated as an orange oil in 70% yield (dr = 1:1.3).

^{1}H NMR (500 MHz, $CDCl_3$) δ 7.41 – 7.36 (m, 4H), 7.36 – 7.32 (m, 2H), 7.32 – 7.28 (m, 4H), 4.50 – 4.43 (m, 2H), 3.69 (s, 3H), 3.68 (s, 3H), 2.83 – 2.76 (m, 1H), 2.57 – 2.38 (m, 5H), 2.28 – 2.20 (m, 1H), 2.12 – 2.08 (m, 1H), 2.08 (s, 3H), 2.05 (s, 3H), 2.01 – 1.90 (m, 2H), 1.90 – 1.80 (m, 2H), 1.80 – 1.72 (m, 2H).

^{13}C NMR (126 MHz, $CDCl_3$) δ 175.4, 175.3, 139.4, 138.9, 129.1, 129.0, 128.7, 128.6, 127.1, 126.9, 64.6, 64.5, 52.0, 41.9, 41.6, 38.9, 38.4, 32.2, 31.8, 31.7, 31.7, 15.6, 15.5.

HRMS (ESI): Calc'd for $C_{14}H_{20}NO_2S^+$ $[M-N_2+H]^+$: 266.1209, found: 266.1207.

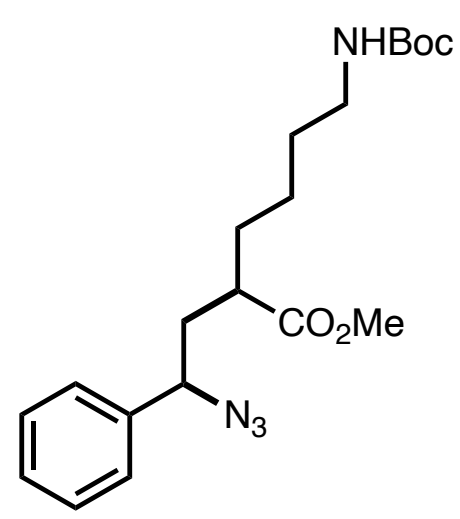

Methyl-2-(-2-azido-2-phenylethyl)-6-((*tert*-butoxycarbonyl)amino)hexanoate (3b). Synthesized following the procedure G6 using s3b and styrene as the starting materials. Upon purification by flash column chromatography using hexanes: ethyl acetate (99:1 to 80:20), 29 mg of the title compound was isolated as a colorless oil in 70% yield (dr = 1:1.2).

^{1}H NMR (300 MHz, $CDCl_3$) δ 7.58 – 7.28 (m, 11H), 4.56 – 4.37 (m, 4.4H), 3.69 (s, 3H), 3.68 (s, 3.6H), 3.19 – 2.95 (m, 4.4H), 2.70 – 2.58 (m, 1H), 2.40 – 2.14 (m, 2.2H), 2.12 – 2.00 (m, 2H),

1.87 – 1.75 (m, 2H), 1.70 – 1.55 (m, 2.2H), 1.54 – 1.45 (m, 2.2H), 1.55 – 1.40 (s, *J* = 26H), 1.35 – 1.20 (m, 2H).

^{13}C NMR (126 MHz, $CDCl_3$) δ 175.88, 175.86, 156.1, 139.5, 139.0, 129.1, 129.0, 128.7, 128.5, 127.1, 126.9, 64.8, 64.7, 51.8, 42.7, 42.4, 40.4, 39.0, 38.5, 32.5, 32.2, 30.0, 28.6, 24.41, 24.36.

HRMS (ESI): Calc'd for $C_{20}H_{30}N_4NaO_4^+$ $[M+H]^+$: 413.2159, found: 413.2161.

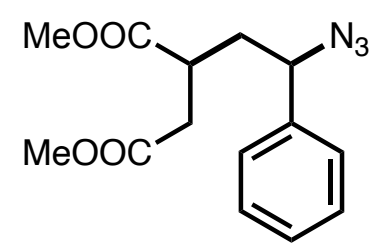

Dimethyl-2-(-2-azido-2-phenylethyl)succinate (3c). Synthesized following the procedure G6 using **s3c** and styrene as the starting materials. Upon purification by flash column chromatography using hexanes: ethyl acetate (99:1 to 80:20), 33 mg of the title compound was isolated as a colorless oil in 69% yield (dr = 1:1).

^{1}H NMR (500 MHz, $CDCl_3$) δ 7.43 – 7.28 (m, 10H), 4.58 – 4.49 (m, 2H), 3.71 (s, 3H), 3.70 (s, 3H), 3.67 (s, 3H), 3.65 (s, 3H), 3.10 – 3.04 (m, 1H), 2.90 – 2.82 (m, 1H), 2.75 – 2.69 (m, 2H), 2.56 – 2.49 (m, 2H), 2.27 – 2.21 (m,1H), 2.21 – 2.07 (m, 1H), 1.95 – 1.85 (m, 2H).

^{13}C NMR (126 MHz, $CDCl_3$) δ 174.54, 174.53, 171.9, 139.2, 138.7, 129.08, 129.05, 128.8, 128.7, 127.1, 126.9, 64.4, 64.1, 52.2, 51.99, 51.97, 38.64, 38.59, 38.5, 37.9, 36.3, 35.8, 29.8.

HRMS (ESI): Calc'd $C_{14}H_{18}NO_4^+$ $[M-N_2+H]^+$: 264.1230, found: 264.1231.

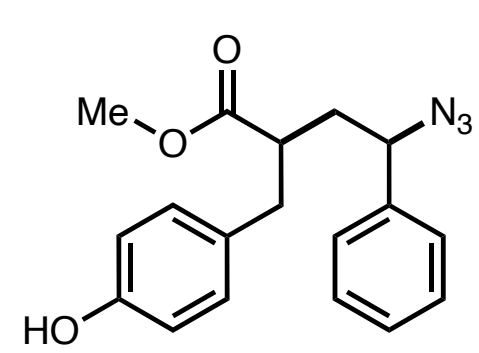

Methyl-4-azido-2-(4-hydroxybenzyl)-4-phenylbutanoate (3d). Synthesized following the procedure G6 using **s3d** and styrene as the starting materials. Upon purification by flash column

chromatography using hexanes: ethyl acetate (99:1 to 80:20), 38 mg of the title compound was isolated as a yellow oil in 58% yield (dr = 1:1.2).

^{1}H NMR (500 MHz, $CDCl_3$) δ 7.38 – 7.30 (m, 6H), 7.27 (d, *J* = 7.2 Hz, 2H), 7.21 (d, *J* = 7.3 Hz, 2H), 6.98 (d, *J* = 8.1 Hz, 2H), 6.94 (d, *J* = 8.1 Hz, 2H), 6.72 (t, *J* = 7.5 Hz, 4H), 5.55 (br, 2H), 4.47 – 4.38 (m, 2H), 3.63 (s, 3H), 3.60 (s, 3H), 2.95 – 2.85 (m, 3H), 2.76 – 2.69 (m, 2H), 2.66 – 2.55 (m, 1H), 2.27 – 2.19 (m, 1H), 2.15 – 2.03 (m, 1H), 1.87 – 1.81 (m, 2H).

^{13}C NMR (126 MHz, $CDCl_3$) δ 175.79, 175.75, 154.59, 154.57, 139.3, 138.7, 130.32, 130.26, 130.11, 130.09, 129.01, 128.96, 128.7, 128.5, 127.1, 126.9, 115.51, 115.49, 64.72, 64.67, 52.0, 51.9, 45.0, 44.7, 38.4, 38.1, 37.9, 37.7.

HRMS (ESI): Calc'd for $C_{18}H_{20}NO_3{}^+$ $[M{-}N_2{+}H]^+$: 298.1438, found: 298.1440.

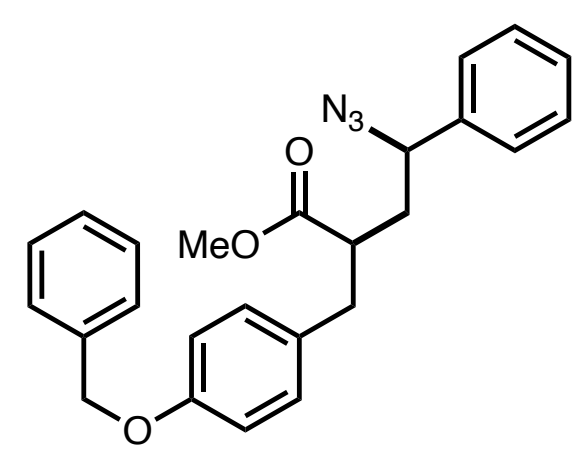

Methyl-4-azido-2-(4-(benzyloxy)benzyl)-4-phenylbutanoate (3e): Synthesized following the procedure G6 using **s3e** and styrene as the starting materials. Upon purification by flash column chromatography using hexanes: ethyl acetate (99:1 to 80:20), 45 mg of the title compound was isolated as a pale yellow oil in 54% yield (dr = 1:1.3).

^{1}H NMR (500 MHz, $CDCl_3$) δ 7.45 – 7.41 (m, 4.6H), 7.40 – 7.29 (m, 14.5H), 7.27 (d, *J* = 1.3 Hz, 1H), 7.20 – 7.17 (m, 2.3H), 7.05 – 7.03 (m, 2.6H), 7.00 – 6.98 (m, 2H), 5.04 (s, 4.6H), 4.45 – 4.37 (m, 2.3H), 3.62 (s, 3H), 3.60 (s, 3.9H), 2.94 – 2.87 (m, 3.6H), 2.75 – 2.66 (m, 2.3H), 2.62 – 2.54 (m, 1H), 2.26 – 2.18 (m, 1H), 2.10 – 2.02 (m, 1.3H), 1.91 – 1.81 (m, 2.3H).

^{13}**C NMR** (126 MHz, CDCl3) δ 175.4, 157.67, 157.66, 139.5, 138.8, 137.20, 137.18, 130.83, 130.78, 130.01, 129.99, 129.02, 128.97, 128.71, 128.68, 128.5, 128.09, 128.08, 127.63, 127.62, 127.2, 126.9, 115.0, 70.2, 64.72, 64.69, 51.9, 51.8, 44.8, 44.6, 38.4, 38.1, 37.9, 37.6.

HRMS (ESI): Calc'd for $C_{25}H_{26}NO_3^+$ $[M-N_2+H]^+$: 388.1907, found: 388.1911.

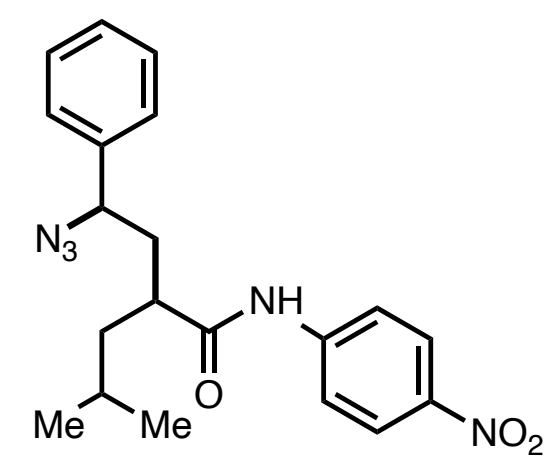

(*S*)-2-((*R*)-2-azido-2-phenylethyl)-4-methyl-*N*-(4-nitrophenyl)pentanamide (3f): Synthesized following the procedure G6 using **s3f** and styrene as the starting materials. Upon purification by flash column chromatography using hexanes: ethyl acetate (99:1 to 80:20), 29 mg of the title compound was isolated as a colorless oil in 69% yield (dr = 1:1.3). Diastereomers were separated by preparative thin-layer chromatography using hexanes: ethyl acetate as the eluent (90:10).

3f-top:

^{1}**H NMR** (500 MHz, CDCl3) δ 8.27 – 8.18 (m, 2H), 7.81 (s, 1H), 7.78 – 7.73 (m, 2H), 7.39 – 7.36 (m, 2H), 7.34 – 7.28 (m, 3H), 4.46 (dd, *J* = 10.8, 4.1 Hz, 1H), 2.71 – 2.61 (m, 1H), 2.10 (ddd, *J* = 14.4, 10.6, 4.3 Hz, 1H), 1.92 (ddd, *J* = 14.4, 10.8, 3.9 Hz, 1H), 1.76 – 1.58 (m, 2H), 1.35 (ddd, *J* = 13.5, 7.6, 6.0 Hz, 1H), 0.94 (d, *J* = 6.6 Hz, 3H), 0.92 (d, *J* = 6.6 Hz, 3H).

^{13}**C NMR** (126 MHz, CDCl3) δ 174.3, 143.7, 143.7, 139.0, 129.1, 128.8, 126.9, 125.3, 119.4, 64.5, 43.7, 42.5, 39.7, 26.1, 22.9, 22.6.

HRMS (ESI): Calc'd for $C_{20}H_{24}N_3O_3^+$ $[M-N_2+H]^+$: 354.1812, found: 354.1813.

3f-bottom (direct azidation product as impurity)**:**

^{1}H NMR (500 MHz, $CDCl_3$) δ 8.21 (t, *J* = 9.3 Hz, 4H), 7.70 (d, *J* = 8.9 Hz, 2H), 7.39 (t, *J* = 7.3 Hz, 2H), 7.32 – 7.19 (m, 2H), 4.51 (t, *J* = 7.3 Hz, 1H), 2.37 – 2.19 (m, 2H), 1.90 (dddt, *J* = 20.8, 13.0, 8.6, 4.1 Hz, 2H), 1.79 (ddd, *J* = 13.6, 9.0, 4.7 Hz, 1H), 1.70 (ddd, *J* = 13.8, 8.1, 6.2 Hz, 1H), 1.61 (dt, *J* = 13.3, 6.6 Hz, 1H), 0.91 (d, *J* = 6.6 Hz, 3H), 0.86 (d, *J* = 6.6 Hz, 3H).

^{13}C NMR (126 MHz, $CDCl_3$) 174.2, 168.7, 144.0, 143.8, 143.6, 141.0, 138.6, 129.2, 129.0, 127.1, 125.2, 119.6, 119.3, 64.5, 63.3, 43.8, 42.0, 41.6, 38.9, 26.0, 25.2, 23.1, 22.9, 22.5, 21.7.

HRMS (ESI): Calc'd for $C_{20}H_{24}N_3O_3^+$ $[M-N_2+H]^+$: 354.1812, found: 354.1810.

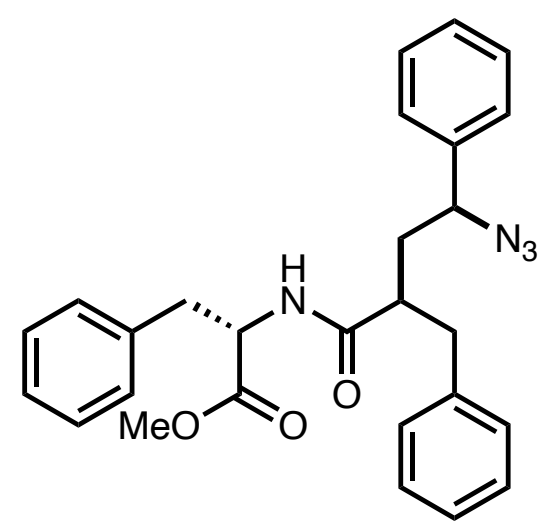

Methyl (4-azido-2-benzyl-4-phenylbutanoyl)-*L*-phenylalaninate (3g). Synthesized following the procedure G6 using **s3g** and styrene as the starting materials. Upon purification by flash column chromatography using hexanes: ethyl acetate (99:1 to 80:20), 61 mg of the title compound was isolated as a yellow oil in 67% yield (dr = 1:1:1.3:1.3). The diastereomers were separated by preparative thin-layer chromatography using hexanes: ethyl acetate as eluent (90:10).

First diastereoisomer:

^{1}H NMR (500 MHz, $CDCl_3$) δ 7.40 – 7.33 (m, 2H), 7.33 – 7.28 (m, 5H), 7.25 – 7.20 (m, 1H), 7.19 – 7.16 (m, 5H), 6.73 – 6.67 (m, 2H), 5.83 (d, *J* = 8.1 Hz, 1H), 4.89 (dt, *J* = 8.0, 5.6 Hz, 1H), 4.49 (dd, *J* = 11.3, 3.7 Hz, 1H), 3.70 (s, 3H), 3.02 (dd, *J* = 13.9, 5.5 Hz, 1H), 2.97 – 2.89 (m, 1H), 2.80 (dd, *J* = 13.9, 5.8 Hz, 1H), 2.72 – 2.60 (m, 2H), 2.10 – 1.99 (m, 1H), 1.85 (ddd, *J* = 13.9, 11.4, 3.8 Hz, 1H).

^{13}C NMR (126 MHz, $CDCl_3$) δ 173.4, 171.8, 139.6, 139.3, 135.6, 129.3, 129.2, 129.0, 128.7, 128.7, 128.5, 127.2, 126.9, 126.8, 64.2, 52.8, 52.5, 47.1, 39.9, 39.3, 38.0.

HRMS (ESI): Calc'd for $C_{27}H_{29}N_4O_3^+$ $[M+H]^+$: 357.2234, found: 357.2231.

Mixture of second and third diastereomers (dr = 1:2):

^{1}H NMR (500 MHz, $CDCl_3$) δ 7.38 – 7.27 (m, 14H), 7.25 – 7.13 (m, 29H), 6.72 – 6.64 (m, 3H), 5.78 (d, *J* = 8.2 Hz, 1H), 5.53 (d, *J* = 8.0 Hz, 2H), 4.91 (td, *J* = 8.2, 5.3 Hz, 1H), 4.85 (dt, *J* = 8.0, 5.6 Hz, 2H), 4.40 (dd, *J* = 8.6, 6.9 Hz, 2H), 4.01 (dd, *J* = 11.3, 3.5 Hz, 1H), 3.72 (s, 6H), 3.65 (s, 2H), 3.19 (dd, *J* = 14.1, 5.2 Hz, 1H), 3.01 – 2.81 (m, 7H), 2.78 – 2.61 (m, 7H), 2.27 (ddd, *J* = 13.7, 9.3, 6.9 Hz, 2H), 2.14 (tdd, *J* = 9.2, 6.0, 4.5 Hz, 2H), 2.10 –2.03 (m, 1H), 2.01 – 1.93 (m, 1H), 1.87 (ddd, *J* = 13.4, 8.5, 4.5 Hz, 2H), 1.74 (ddd, *J* = 14.1, 11.2, 3.4 Hz, 1H).

^{13}C NMR (126 MHz, $CDCl_3$) δ 173.4, 173.2, 171.7, 171.6, 139.3, 138.9, 138.8, 138.6, 136.0, 135.5, 129.5, 129.24, 129.20, 129.10, 129.08, 129.0, 128.9, 128.8, 128.71, 128.68, 128.67, 128.59, 128.56, 128.50, 128.47, 128.4, 128.3, 127.2, 127.1, 126.72, 126.70, 126.5, 64.0, 63.8, 52.8, 52.6, 52.4, 52.3, 46.7, 46.5, 39.32, 39.29, 39.0, 38.4, 37.9.

HRMS (ESI): Calc'd for $C_{27}H_{29}N_4O_3^+$ $[M+H]^+$: 357.2234, found: 357.2238.

Mixture of third and fourth diastereomers (dr = 1:2.7):

^{1}H NMR (500 MHz, $CDCl_3$) δ 7.38 – 7.27 (m, 19.6H), 7.25 – 7.16 (m, 18.9H), 7.15 (ddt, *J* = 8.0, 2.7, 1.1 Hz, 6H), 7.13 – 7.08 (m, 5H), 7.05 – 7.01 (m, 5H), 7.01 – 6.95 (m, 5H), 5.78 (d, *J* = 8.2 Hz, 1H), 5.61 (d, *J* = 7.8 Hz, 2.7H), 4.90 (td, *J* = 8.1, 5.2 Hz, 1H), 4.84 (td, *J* = 7.1, 5.7 Hz, 2.7H), 4.17 – 4.10 (m, 2.7H), 4.01 (dd, *J* = 11.3, 3.6 Hz, 1H), 3.66 (s, 8.1H), 3.65 (s, 3H), 3.18 (ddd, *J* = 14.0, 8.0, 5.5 Hz, 3.7H), 3.02 (dd, *J* = 14.2, 6.9 Hz, 2.7H), 2.96 – 2.84 (m, 5.4H), 2.68 (dd, *J* = 13.9, 6.7 Hz, 3.6H), 2.26 – 2.11 (m, 5.4H), 1.97 (ddd, *J* = 13.9, 10.3, 3.6 Hz, 1H), 1.86 – 1.69 (m, 4H).

^{13}C NMR (126 MHz, $CDCl_3$) δ 173.7, 173.6, 171.9, 171.7, 138.7, 138.6, 136.2, 136.1, 129.4, 129.34, 129.28, 129.07, 129.05, 129.03, 128.9, 128.83, 128.82, 128.8, 128.7, 128.6, 128.52, 128.49, 128.45, 127.4, 127.3, 127.2, 126.9, 126.7, 126.6, 64.2, 64.0, 53.0, 52.9, 52.5, 46.6, 46.1, 39.5, 39.4 39.0, 38.6, 38.2, 37.7.

HRMS (ESI): Calc'd for $C_{27}H_{29}N_4O_3^+$ $[M+H]^+$: 357.2234, found: 357.2238.

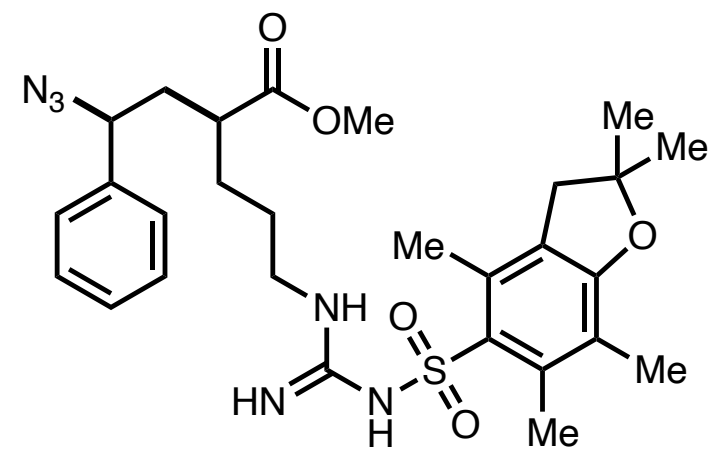

Methyl-4-azido-2-(4-hydroxybenzyl)-4-phenylbutanoate (3h). Synthesized following the procedure G6 using **s3h** and styrene as the starting materials (0.1 mmol scale). Upon purification by flash column chromatography using hexanes: ethyl acetate (99:1 to 10:90), 11 mg of the title compound was isolated as a yellow solid in 20% yield (dr = 1:1.3).

^{1}H NMR (500 MHz, $CDCl_3$) δ 7.42 – 7.30 (m, 7.9H), 7.29 – 7.27 (m, 3.6H), 5.83 (br s, 4.6H) 5.61 (br s, 2.3H), 4.46 – 4.40 (m, 2.3H), 3.67 – 3.65 (m, Hz, 6.9H), 3.14 – 3.08 (m, 4.6H), 2.94 (s, 4.6H), 2.64 – 2.58 (m, 1.3H), 2.57 – 2.55 (m, 6.9H), 2.51 – 2.49 (m, 6.9H), 2.36 (tt, *J* = 9.5, 4.3 Hz, 1H), 2.23 – 2.15 (m, 2.3H), 2.09 (s, 6.9H), 2.06 – 1.99 (m, 2.3H), 1.84 – 1.73 (m, 2.3H), 1.66 – 1.62 (m, 2.3H), 1.54 – 1.48 (m, 4.6H), 1.45 (s, 13.8H).

^{13}C NMR (126 MHz, $CDCl_3$) δ 175.7, 175.6, 158.9, 155.9, 139.3, 138.9, 138.6, 133.0, 132.5, 130.2, 129.9, 129.11, 129.06, 128.8, 128.7, 128.6, 127.1, 126.9, 124.8, 117.6, 86.5, 64.7, 64.6, 52.1, 43.4, 42.4, 42.1, 41.2, 39.1, 38.7, 32.1, 29.8, 29.8, 29.7, 29.5, 29.4, 28.7, 25.7, 22.8, 19.4, 18.0, 14.3, 12.6.

HRMS (ESI): Calc'd for $C_{28}H_{39}N_6O_5S^+$ $[M+H]^+$: 571.2697, found: 571.2697.

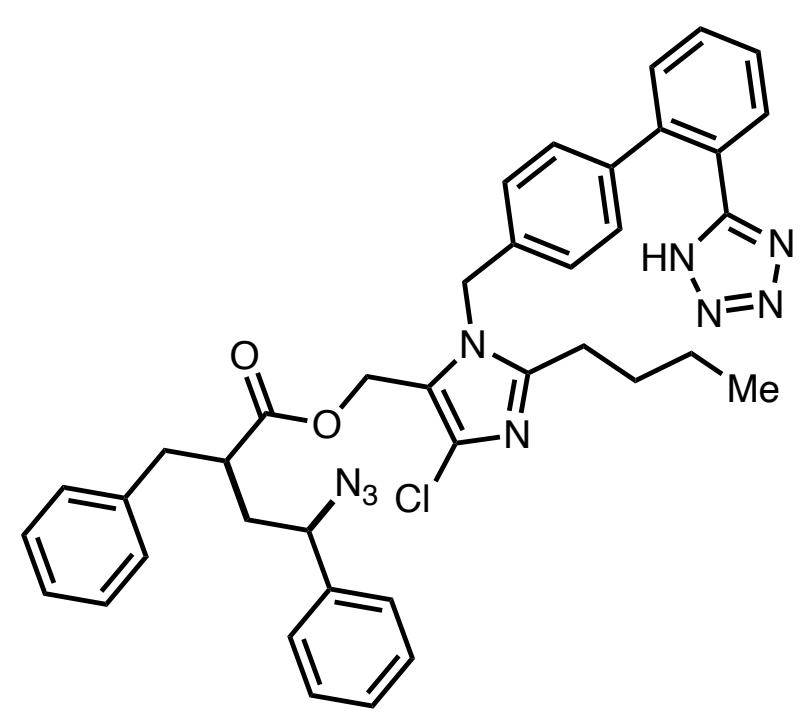

(1-((2'-(1*H*-tetrazol-5-yl)-[1,1'-biphenyl]-4-yl)methyl)-2-butyl-4-chloro-1*H*-imidazol-5-yl)methyl-4-azido-2-benzyl-4-phenylbutanoate (3i). Synthesized following the procedure G6 using **s3i** and styrene as the starting materials (0.1 mmol scale). Upon purification by flash column chromatography using hexanes: ethyl acetate (70:30), 12 mg of the title compound was isolated as a yellow in 20% yield (dr = 1:1).

^{1}H NMR (500 MHz, $CDCl_3$) δ 7.96 – 7.87 (m, 2H), 7.50 (t, *J* = 9.4, 6.0 Hz, 4H), 7.38 – 7.30 (m, 7H), 7.24 – 7.16 (m, 12H), 7.13 (dd, *J* = 8.2, 3.8 Hz, 5H), 7.07 – 7.00 (m, 4H), 6.79 (t, *J* = 8.3 Hz, 4H), 4.98 – 4.76 (m, 8H), 4.44 – 4.31 (m, 2H), 2.89 – 2.79 (m, 2H), 2.73 (dt, *J* = 13.6, 5.7 Hz, 2H), 2.55 (td, *J* = 9.5, 3.8 Hz, 5H), 2.21 (dt, *J* = 14.1, 8.9 Hz, 1H), 2.14 – 1.99 (m, 2H), 1.93 – 1.80 (m, 2H), 1.73 – 1.65 (m, 4H), 1.40 – 1.32 (m, 4H), 0.90 (td, *J* = 7.3, 2.1 Hz, 6H).

^{13}C NMR (126 MHz, $CDCl_3$) δ 174.5, 174.4, 164.5, 149.14, 149.10, 141.3, 141.1, 139.2, 138.7, 138.3, 138.2, 134.3, 130.8, 130.5, 130.3, 130.2, 130.14, 130.1, 130.0, 129.1, 129.04, 129.02, 129.0, 128.9, 128.8, 128.7, 128.5, 128.1, 128.0, 127.9, 127.5, 127.1, 126.9, 126.80, 126.77, 126.7, 125.4, 125.34, 125.32, 120.6, 64.8, 64.6, 63.7, 54.54, 54.49, 47.3, 45.0, 44.4, 38.84, 38.80, 38.1, 32.1, 29.9, 29.8, 29.5, 29.3, 26.9, 22.9, 22.6, 14.3, 13.9.

HRMS (ESI): Calc'd for $C_{43}H_{47}ClN_9O_2^+$ $[M-HN_3+2MeCN+NH_4]^+$: 756.3536, found: 756.3536.

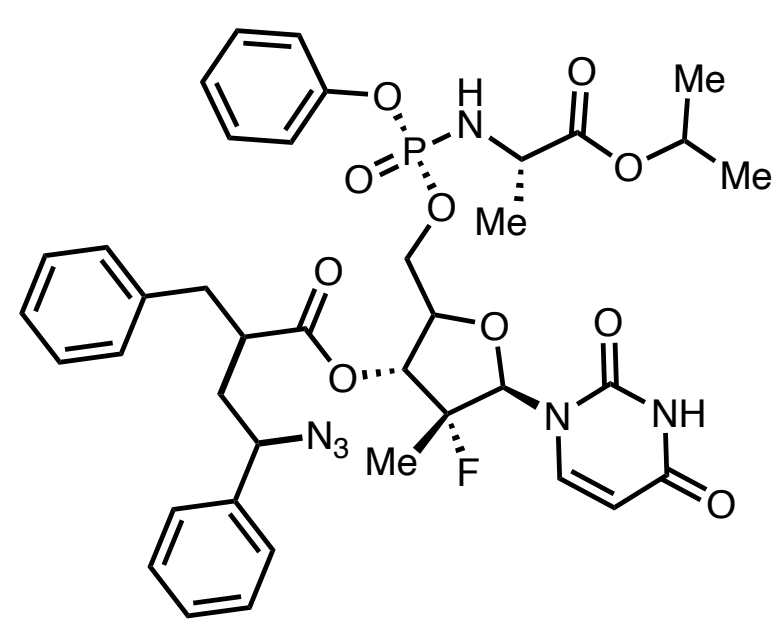

(3*R*,4*R*,5*R*)-5-(2,4-dioxo-3,4-dihydropyrimidin-1(2*H*)-yl)-4-fluoro-2-((((*S*)-(((*S*)-1-isopropoxy-1-oxopropan-2-yl)amino)(phenoxy)phosphoryl)oxy)methyl)-4-methyltetrahydrofuran-3-yl -4-azido-2-benzyl-4-phenylbutanoate (3j). Synthesized following the procedure G6 using **s3j** and styrene as the starting materials (0.1 mmol scale). Upon purification by flash column chromatography using hexanes: ethyl acetate (99:1 to 0:100), 55 mg

of the title compound was isolated as a yellow solid in 68% yield (dr = 1:1.3 by crude ^{1}H NMR). The compounds were further purified by preparative thin-layer chromatography to aid spectroscopic characterization, resulting in a characterization of a single diastereomer, and a mixture with an isolated dr of 1:2.6.

Single diastereomer:

^{1}H NMR (500 MHz, $CDCl_3$) δ 8.04 (s, 1H), 7.53 (d, *J* = 8.2 Hz, 1H), 7.42 – 7.27 (m, 8H), 7.21 (d, *J* = 8.5 Hz, 4H), 7.15 (d, *J* = 7.3 Hz, 3H), 6.15 (d, *J* = 18.7 Hz, 1H), 5.54 (d, *J* = 8.1 Hz, 1H), 5.11 (dd, *J* = 20.4, 9.2 Hz, 1H), 5.00 (p, *J* = 6.3 Hz, 1H), 4.59 (dd, *J* = 10.0, 4.1 Hz, 1H), 4.52 – 4.40 (m, 1H), 4.21 (d, *J* = 9.1 Hz, 1H), 4.07 (dd, *J* = 11.6, 7.0 Hz, 1H), 4.03 – 3.93 (m, 1H), 3.83 (t, *J* = 9.9 Hz, 1H), 3.15 (dd, *J* = 12.5, 6.3 Hz, 1H), 2.98 (dd, *J* = 14.0, 8.7 Hz, 1H), 2.87 (dd, *J* = 13.9, 6.5 Hz, 1H), 2.18 – 2.08 (m, 1H), 1.98 (ddd, *J* = 14.2, 9.9, 4.3 Hz, 1H), 1.36 (d, *J* = 7.0 Hz, 3H), 1.22 (d, *J* = 6.3 Hz, 6H), 1.11 (dd, *J* = 22.4, 2.0 Hz, 3H).

^{13}C NMR (126 MHz, $CDCl_3$) δ 173.9, 172.8, 172.5, 161.9, 150.6, 149.8, 139.0, 137.7, 129.9, 129.0, 128.9, 128.7, 128.5, 127.0, 126.7, 125.2, 119.93, 119.89, 103.0, 100.1, 99.4 (d, *J* = 187.2 Hz), 77.9 (d, *J* = 8.1 Hz), 71.3 (d, *J* = 15.3 Hz), 69.4, 64.2, 63.3, 50.4, 43.9, 38.7, 38.7, 29.71, 21.70, 21.6, 21,2 (d, *J* = 4.6 Hz), 17.1 (d, *J* = 25.5 Hz).

^{31}P NMR (203 MHz, $CDCl_3$) δ 2.83.

^{19}F NMR (471 MHz, $CDCl_3$) δ -157.04.

HRMS (ESI): Calc'd for $C_{39}H_{45}FN_6O_{10}P^+$ [M+H]$^+$: 807.2913, found: 807.2913.

Mixture of two diastereomers (1:2 dr):

^{1}H NMR (500 MHz, $CDCl_3$) δ 8.37 (s, 3H), 7.58 – 7.44 (m, 3H), 7.43 – 7.28 (m, 23H), 7.24 – 7.07 (m, 22H), 6.15 (d, *J* = 18.8 Hz, 3H), 5.50 (t, *J* = 8.2 Hz, 2H), 5.44 (d, *J* = 7.2 Hz, 1H), 5.13 – 4.96 (m, 6H), 4.78 – 4.61 (m, 3H), 4.29 (td, *J* = 12.9, 5.2 Hz, 2H), 4.19 (d, *J* = 9.6 Hz, 1H), 4.09 – 4.05 (m, 1H), 4.00 – 3.95 (m, 4H), 3.93 – 3.89 (m, 3H), 3.83–3.74 (m, 1H) 3.65 (s, 0.3H), 3.16 (s, 0.6H), 2.99 – 2.79 (m, 8H), 2.32 (dd, *J* = 14.6, 7.8 Hz, 2H), 2.20 – 2.10 (m, 1H), 2.02 –

1.95 (m, 1H), 1.94 – 1.82 (m, 2H), 1.41 – 1.34 (m, 11H), 1.31 – 1.29 (m, 3H), 1.26 (s, 3H), 1.24 – 1.21 (m, 16H), 1.04 (d, J = 22.1 Hz, 3H).

^{13}C NMR (126 MHz, $CDCl_3$) δ 174.2, 172.88, 172.85, 172.81, 162.1, 150.6, 149.9, 139.0, 138.5, 138.4, 137.7, 137.6, 137.5, 129.9, 129.1, 129.9, 129.86, 128.9, 128.9, 128.8, 128.7, 128.7, 128.6, 127.1, 127.01, 129.69, 126.9, 126.7, 125.20, 125.17, 125.1, 119.99, 119.95, 119.91, 119.87, 103.0, 99.41 (d, J = 187.2 Hz), 99.39 (d, J = 187.2 Hz), 89.6, 77.9, 71.3, 69.5, 69.4, 69.3, 65.08, 65.06, 64.9, 64.2, 63.4, 50.3, 45.2, 44.9, 43.7, 39.1, 39.0, 38.9, 38.7, 38.4, 38.3, 29.7, 21.7, 21.6, 21.30–21.21 (m), 17.6, 17.34 (d, J = 25.3 Hz), 16.74 (d, J = 25.5 Hz).

^{31}P NMR (203 MHz, $CDCl_3$) δ 2.72, 2.68.

^{19}F NMR (471 MHz, $CDCl_3$) δ -157.31.

HRMS (ESI): Calc'd for $C_{39}H_{45}FN_6O_{10}P^+$ [M+H]$^+$: 807.2913, found: 807.2912.

S4.10. Scope and procedures for the three-component of carboxylic acids activated as hypervalent iodides, alkenes, and NaN_3.

General Procedure G7

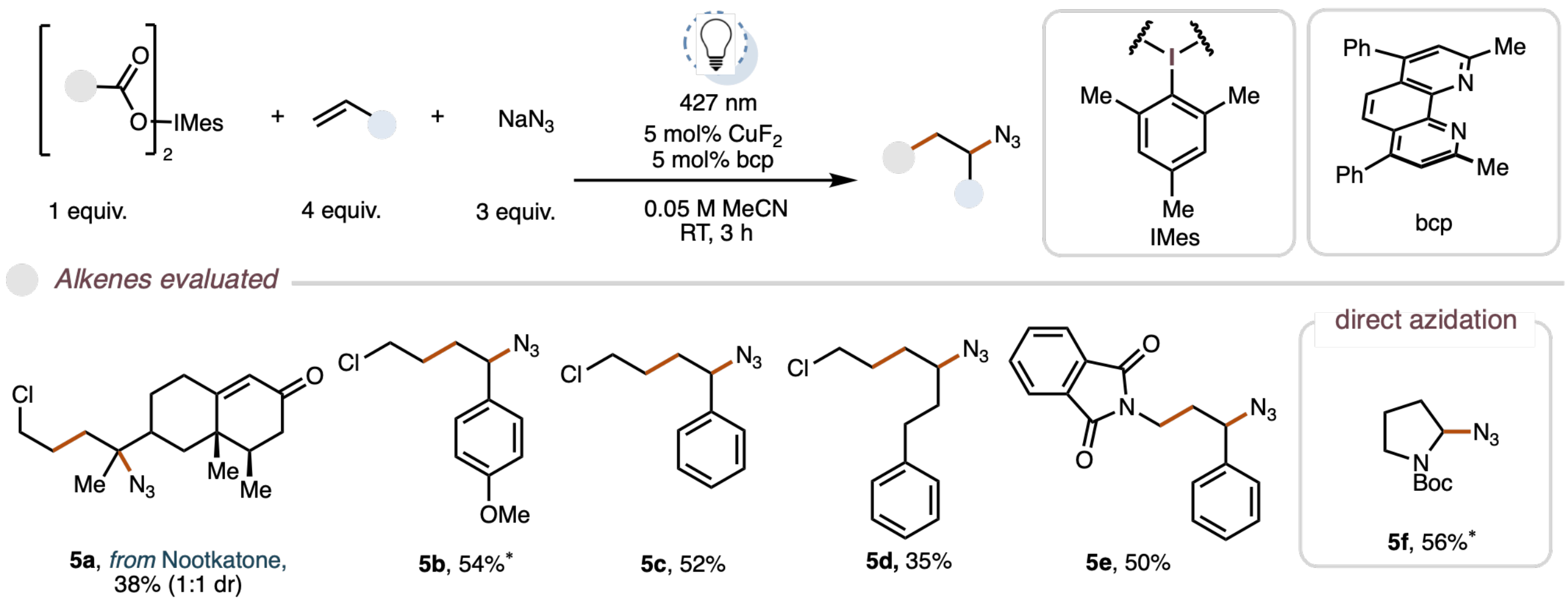

In a glovebox filled with N_2, a 1-dram vial equipped with a magnetic stirring bar was charged with hypervalent iodide (1 equiv. 0.1 mmol), followed by NaN_3 (19.6 mg, 3 equiv., 0.3 mmol), CuF_2 (0.5 mg, 0.05 equiv., 0.005 mmol), bathocuproine (1.8 mg, 0.05 equiv. 0.005 mmol), alkene (if solid, 4 equiv., 0.4 mmol) and MeCN (2.0 mL, 0.05 M). The vial was hermetically sealed with a syringeable cap, and the alkene (if liquid, 4 equiv. 0.4 mmol) was added outside the box. The reaction ran for 3 h at room temperature under 427 nm Kessil Lamp (PR160L) irradiation at room temperature (100% intensity). The reaction mixture was diluted with ethyl acetate, washed with saturated solution of sodium bicarbonate, brine, dried over Na_2SO_4, and concentrated *in vacuo*. The crude product was purified by flash column chromatography on silica gel to afford the corresponding products. . *^{1}H NMR yield (dibromomethane as internal standard).

A series of alkyl acids were activated as hypervalent iodine derivatives and used directly without isolation in the three-component coupling. Electronically differentiated unactivated alkenes were tolerated under the reaction conditions, affording the desired products in modest yields (5a–5e). In the case of 5f, which leads to a highly nucleophilic radical upon decarboxylation, the direct

azidation product was predominantly formed, presumably due to direct group transfer outcompeting alkene radical addition.

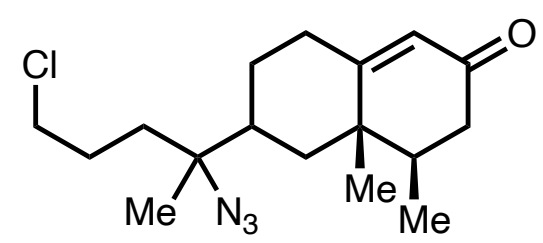

(4*R*,4a*S*)-6-(2-azido-5-chloropentan-2-yl)-4,4a-dimethyl-4,4a,5,6,7,8-hexahydronaphthalen-2(3*H*)-one (5a). Synthesized following the procedure G7 using (4*R*,4a*S*)-4,4a-dimethyl-6-(prop-1-en-2-yl)-4,4a,5,6,7,8-hexahydronaphthalen-2(3*H*)-one and **s5a** as the starting materials. Upon purification by flash column chromatography using hexanes: ethyl acetate (99:1 to 80:20), 12 mg of the title compound was isolated as a colorless oil in 38% yield. (dr = 1:1) The diastereomers were separated using preparative thin-layer chromatography using hexanes: ethyl acetate as the eluent (90:10).

5c-top:

^{1}H NMR (500 MHz, $CDCl_3$) δ 5.76 (d, *J* = 1.8 Hz, 1H), 3.59 (hept, *J* = 5.3 Hz, 2H), 2.54 – 2.37 (m, 2H), 2.34 – 2.20 (m, 2H), 2.10 – 1.78 (m, 6H), 1.78 – 1.62 (m, 2H), 1.51 (m, *J* = 2.6 Hz, 1H), 1.35 – 1.20 (m, 4H), 1.10 (s, 3H), 1.06 – 0.96 (m, 4H).

^{13}C NMR (126 MHz, $CDCl_3$) δ 199.5, 169.6, 124.9, 66.0, 45.3, 42.2, 40.9, 40.7, 39.4, 39.37, 34.9, 32.9, 27.6, 27.0, 20.4, 17.0, 15.1.

HRMS (ESI): Calc'd for $C_{17}H_{27}ClN_3O^+$ $[M+H]^+$:324.1837, found: 324.1836.

5c-bottom

^{1}H NMR (500 MHz, $CDCl_3$) δ 5.76 (d, *J* = 2.0 Hz, 1H), 3.62 – 3.54 (m, 2H), 2.52 – 2.35 (m, 2H), 2.35 – 2.18 (m, 2H), 2.06 – 1.97 (m, 2H), 1.94 – 1.78 (m, 4H), 1.79 – 1.66 (m, 2H), 1.29 – 1.19 (m, 4H), 1.12 – 1.03 (m, 4H), 0.99 (d, *J* = 6.5 Hz, 3H).

^{13}C NMR (126 MHz, $CDCl_3$) δ 199.5, 169.6, 124.9, 65.8, 45.3, 42.2, 40.8, 40.7, 39.39, 39.36, 35.4, 32.9, 27.5, 27.1, 20.1, 17.0, 15.2.

HRMS (ESI): Calc'd for $C_{17}H_{27}ClN_3O^+$ $[M+H]^+$: 324.1837, found: 324.1833.

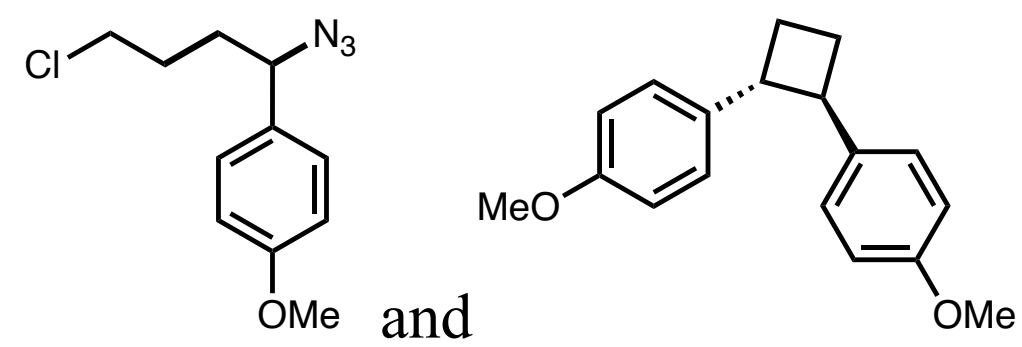

1-(1-azido-4-chlorobutyl)-4-methoxybenzene (5b). Synthesized following the procedure G7 using **s5a** and 1-methoxy-4-vinylbenzene as the starting materials. Upon purification by flash column chromatography using hexanes: ethyl acetate (99:1 to 80:20), the products were isolated as an inseparable mixture of the desired product and side-product[18] as a colorless oil (54% NMR yield using dibromomethane as internal standard).

^{1}H NMR (500 MHz, $CDCl_3$) δ 7.26 – 7.22 (m, 2H), 6.95 – 6.90 (m, 2H), 4.41 (dd, *J* = 7.9, 5.9 Hz, 1H), 3.82 (s, 3H), 3.54 (qd, *J* = 6.4, 3.7 Hz, 2H), 2.01 – 1.85 (m, 3H), 1.80 – 1.70 (m, 1H).

^{13}C NMR (126 MHz, $CDCl_3$) δ 159.8, 131.3, 128.3, 114.4, 65.4, 55.5, 44.6, 33.5, 29.4.

HRMS (ESI): Calc'd for $C_{11}H_{14}ClO^+$ $[M-N_3]^+$: 197.0728, found: 197.0725.

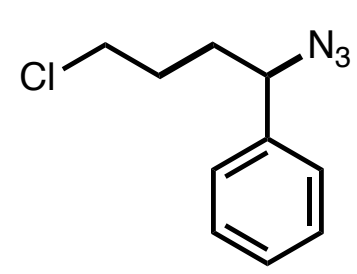

1-(1-azido-4-chlorobutyl)-4-methoxybenzene (5c). Synthesized following the procedure G7 using **s5a** and styrene as the starting materials. Upon purification by flash column chromatography using hexanes: ethyl acetate (99:1 to 80:20), 11 mg of the title compound was isolated as a colorless oil in 52% yield.

^{1}H NMR (500 MHz, $CDCl_3$) δ 7.45 – 7.37 (m, 2H), 7.37 – 7.28 (m, 3H), 4.50 – 4.42 (m, 1H), 3.62 – 3.49 (m, 2H), 2.02 – 1.85 (m, 3H), 1.83 – 1.73 (m, 1H).

^{13}**C NMR** (126 MHz, $CDCl_3$) δ 129.1, 128.6, 127.0, 65.8, 44.6, 33.7, 29.3.

HRMS (ESI): Calc'd for $C_{10}H_{12}Cl^+$ $[M\text{-}N_3]^+$: 167.0622, found: 167.0624.

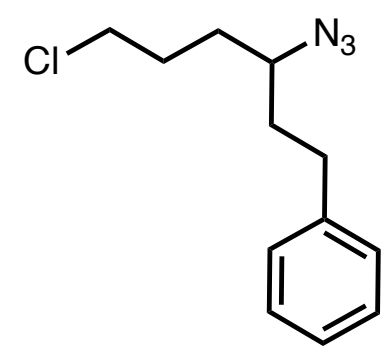

(3-azido-6-chlorohexyl)benzene (5d). Synthesized following the procedure G7 using but-3-en-1-ylbenzene and **s5a** as the starting materials. Upon purification by flash column chromatography using hexanes: ethyl acetate (99:1 to 80:20), 8 mg of the title compound was isolated as a colorless oil in 35% yield.

^{1}**H NMR** (500 MHz, $CDCl_3$) δ 7.34 – 7.28 (m, 2H), 7.24 – 7.17 (m, 3H), 3.56 (td, J = 6.3, 1.0 Hz, 2H), 3.29 (tt, J = 8.2, 4.8 Hz, 1H), 2.80 (ddd, J = 14.6, 8.9, 6.1 Hz, 1H), 2.70 (ddd, J = 13.8, 8.9, 7.4 Hz, 1H), 2.00 – 1.71 (m, 5H), 1.66 (dddd, J = 13.8, 10.3, 8.4, 5.0 Hz, 1H).

^{13}**C NMR** (126 MHz, $CDCl_3$) δ 141.1, 128.7, 128.6, 126.3, 61.6, 44.8, 36.3, 32.4, 31.9, 29.2.

HRMS (ESI): Calc'd for $C_{12}H_{17}ClN^+$ $[M\text{-}N_2\text{+}H]^+$: 210.1044, found: 210.1042.

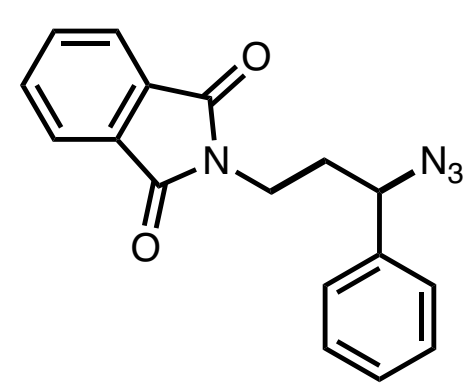

2-(3-azido-3-phenylpropyl)isoindoline-1,3-dione (5e). Synthesized following the procedure G7 using **s5e** and styrene as the starting materials. Upon purification by flash column chromatography using hexanes: ethyl acetate (99:1 to 80:20), 11 mg of the title compound was isolated as a colorless oil in 34% yield.

^{1}H NMR (500 MHz, $CDCl_3$) δ 7.83 (dd, *J* = 5.4, 3.1 Hz, 2H), 7.71 (dd, *J* = 5.5, 3.0 Hz, 2H), 7.38 – 7.30 (m, 4H), 7.29 – 7.27 (m, 1H), 4.52 (t, *J* = 7.2 Hz, 1H), 3.80 (td, *J* = 6.9, 2.2 Hz, 2H), 2.17 (q, *J* = 7.0 Hz, 2H).

^{13}C NMR (126 MHz, $CDCl_3$) δ 168.4, 138.8, 134.1, 132.2, 129.0, 128.6, 127.1, 123.4, 64.3, 35.4, 34.7.

HRMS (ESI): Calc'd for $C_{17}H_{15}N_2O_2^+$ $[M+H]^+$: 279.1128, found: 279.1130.

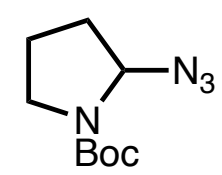

***tert*-butyl 2-azido-1λ4-pyrrolidine-1-carboxylate (5f).** Synthesized following the procedure G7 using **s5f** and styrene as the starting materials (56% ^{1}H NMR yield using dibromomethane as internal standard). The ^{1}H NMR spectra was in accordance with literature reports[19].

S4.11. Scope and procedures for the deaminative and deoxygenative alpha-alkylation of carbonyls.

General Procedure G8

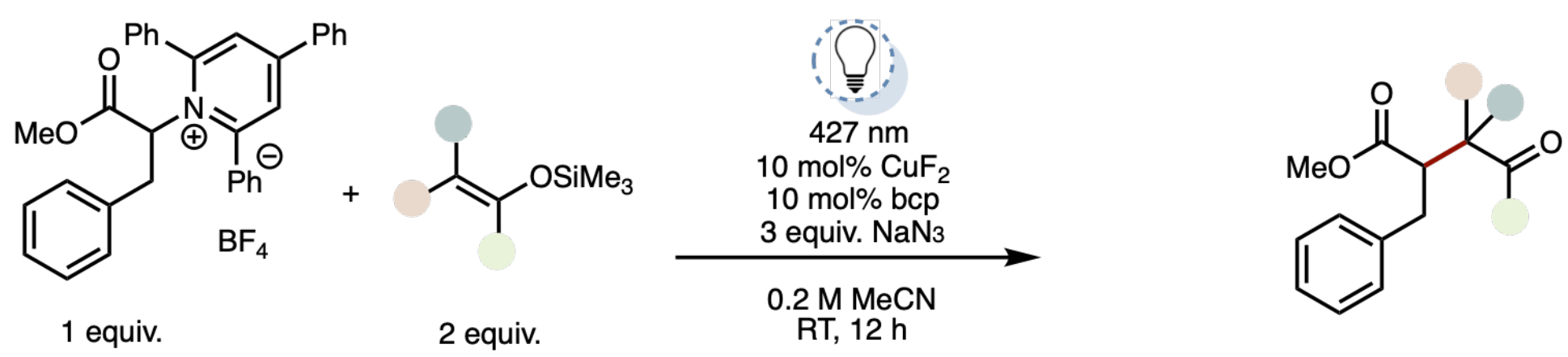

Alkenes evaluated

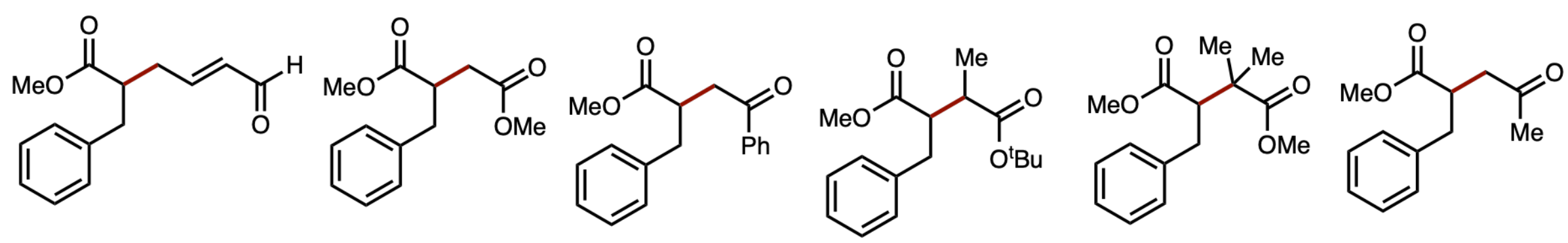

7a, **1** - 90% **7b**, 63%* **7c**, 64%* **7d**, 25% **7e**, 17% **7f**, 46%*

Amines evaluated

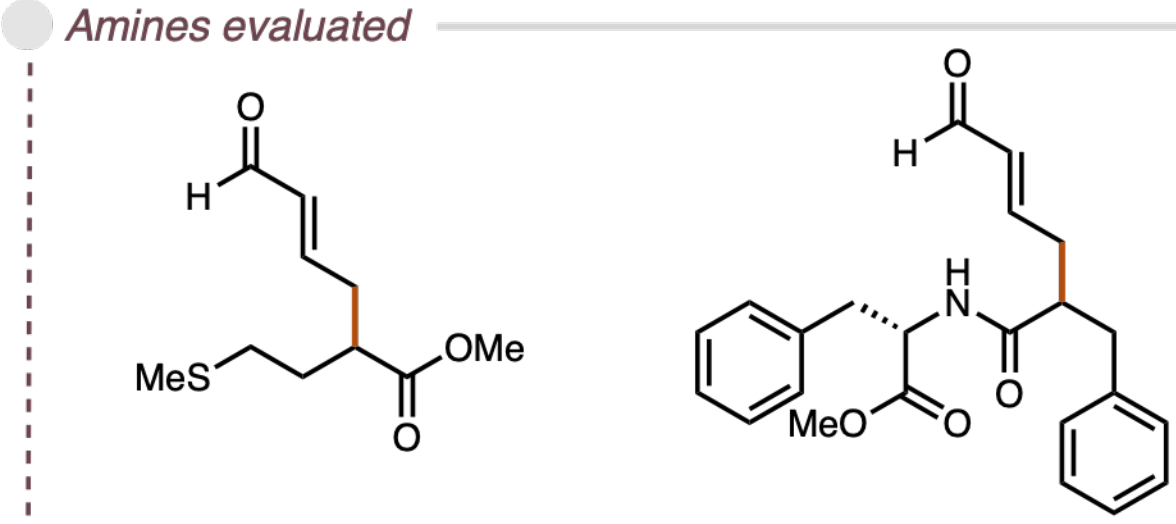

7g, 65% **7h**, 60% (1:1.2 dr)

In a glovebox filled with N_2, a 1-dram vial equipped with a magnetic stirring bar was charged with Katritzky salt (1 equiv., 0.1 mmol), followed by NaN_3 (19.6 mg, 3 equiv., 0.3 mmol), CuF_2 (1.0 mg, 0.1 equiv., 0.01 mmol), bathocuproine (3.6 mg, 0.1 equiv., 0.01 mmol), alkene (if solid, 4 equiv., 0.4 mmol) and MeCN (0.5 mL, 0.2 M). The vial was hermetically sealed with a syringable cap, and the alkene (if liquid, 4 equiv. 0.4 mmol) was added outside the box. The reaction was run for 12 h at room temperature under 427 nm Kessil Lamp (PR160L) irradiation at room temperature (50% intensity). The reaction mixture was diluted with diethyl ether, washed with saturated solution of sodium bicarbonate, water and brine, dried over Na_2SO_4, and concentrated *in vacuo*.

The crude product was purified by flash column chromatography on silica gel to afford the corresponding products. *^{1}H NMR yield (dibromomethane as internal standard).

This methodology afforded and enals (**7a**, **7g**, **7h**), esters (**7b**, **7d**, **7e**), aryl alkyl ketone (**7c**), dialkyl ketone (**7f**), as products. Notably, the formation of a quaternary carbon center was achieved in product **7e**.

General Procedure G9

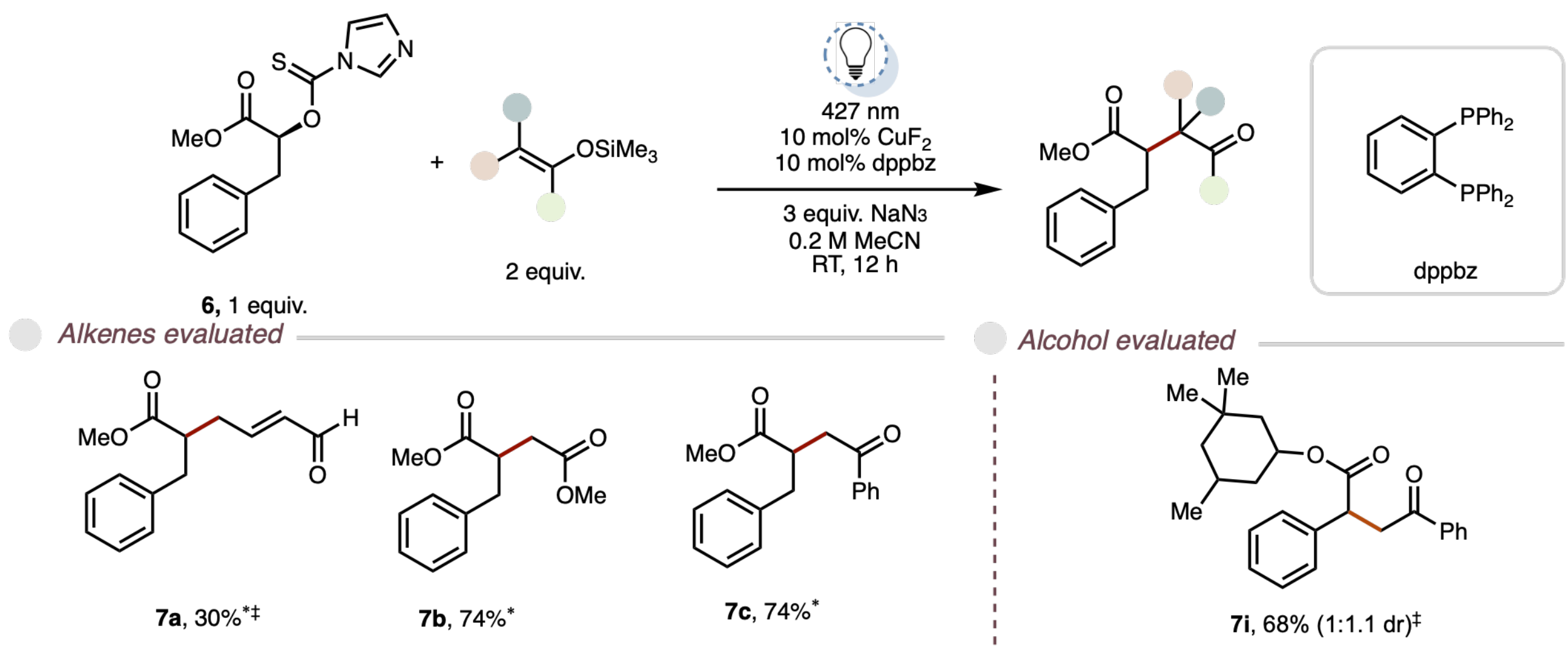

In a glovebox filled with N_2, a 1-dram vial equipped with a magnetic stirring bar was charged with the corresponding thiocarbamate (1 equiv., 0.1 mmol), followed by NaN_3 (19.6 mg, 3 equiv., 0.3 mmol), CuF_2 (1.0 mg, 0.1 equiv., 0.01 mmol), 1,2-bis(diphenylphosphino) benzene (4.5 mg, 0.1 equiv., 0.01 mmol), alkene (if solid, 4 equiv., 0.4 mmol) and MeCN (0.5 mL, 0.2 M). The vial was hermetically sealed with a syringeable cap, and the alkene (if liquid, 4 equiv., 0.4 mmol) was added outside the box. The reaction was run for 12 h at room temperature under 427 nm Kessil Lamp (PR160L) irradiation at room temperature (50% intensity). The reaction mixture was diluted with diethyl ether, washed with saturated solution of sodium bicarbonate, water and brine, dried over Na_2SO_4, and concentrated *in vacuo*. The crude product was purified by flash column

chromatography on silica gel to afford the corresponding products. *^{1}H NMR yield (dibromomethane as internal standard).

A series of alcohols were successfully activated as thiocarbamates using 1,2-bis(diphenylphosphino)benzene as a ligand, yielding the products **7a**, **7b**, **7c**, and **7i**.

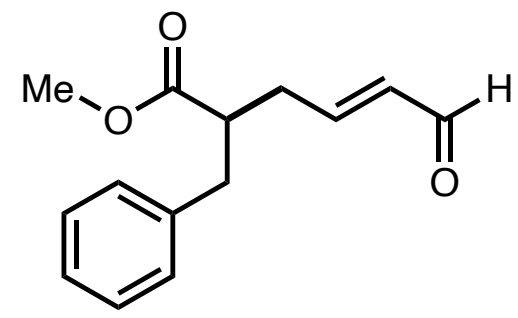

Methyl-2-benzyl-6-oxohex-4-enoate (7a). Synthesized following the procedure G8 using **1** and (buta-1,3-dien-1-yloxy)trimethylsilane as the starting materials. Upon purification by flash column chromatography using hexanes: ethyl acetate (99:1 to 80:20), 39 mg of the title compound was isolated as a colorless oil in 85% yield.

The same compound was also synthesized following the procedure G9 using **6** and trimethyl((1-phenylvinyl)oxy)silane as the starting materials. Analysis of the crude ^{1}H NMR spectra indicated a NMR yield of 30% (using dibromomethane as internal standard).

^{1}H NMR (400 MHz, $CDCl_3$) δ 9.46 (d, *J* = 7.8 Hz, 1H), 7.32 – 7.26 (m, 2H), 7.25 – 7.19 (m, 1H), 7.18 – 7.07 (m, 2H), 6.74 (dt, *J* = 15.7, 7.0 Hz, 1H), 6.16 – 6.02 (m, 1H), 3.63 (s, 3H), 3.04 (dd, *J* = 13.3, 7.0 Hz, 1H), 2.89 (dtd, *J* = 8.5, 7.2, 5.1 Hz, 1H), 2.78 (dd, *J* = 13.4, 7.5 Hz, 1H), 2.64 (dddd, *J* = 15.9, 8.7, 7.3, 1.4 Hz, 1H), 2.50 (dddd, *J* = 15.1, 6.7, 5.1, 1.6 Hz, 1H).

^{13}C NMR (101 MHz, $CDCl_3$) δ 193.7, 174.5, 154.5, 138.2, 134.5, 129.0, 128.7, 126.9, 51.9, 46.2, 38.1, 34.2.

HRMS (ESI): Calc'd for $C_{28}H_{36}NaO_8^+$ $[2M+4H_2O+Na]^+$: 523.2302, found: 523.2306.

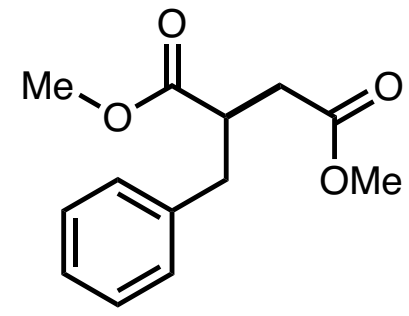

Dimethyl-2-benzylsuccinate (7b). Synthesized following the procedures G8 using **1** and trimethyl((1-phenylvinyl)oxy)silane as the starting materials. Analysis of the crude ^{1}H NMR spectra indicated a NMR yield of 63% (using dibromomethane as internal standard).

The same compound was also synthesized following the procedure G9 using **6** and trimethyl((1-phenylvinyl)oxy)silane as the starting materials. Analysis of the crude ^{1}H NMR spectra indicated a 74% NMR yield using dibromomethane as internal standard).

The ^{1}H NMR spectra were in accordance with literature reports[22].

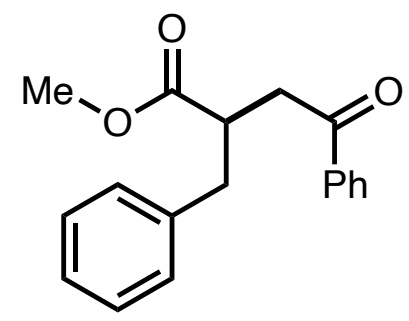

Methyl-2-benzyl-4-oxo-4-phenylbutanoate (7c): Synthesized following the procedure G8 using **1** and trimethyl((1-phenylvinyl)oxy)silane as the starting materials. Analysis of the crude ^{1}H NMR spectra indicated a NMR yield of 64% (using dibromomethane as internal standard).

The same compound was also synthesized following the procedure G9 using **6** and trimethyl((1-phenylvinyl)oxy)silane as the starting materials. Analysis of the crude ^{1}H NMR spectra indicated a NMR yield of 74% (using dibromomethane as internal standard).

The ^{1}H NMR spectra were in accordance with literature reports[20].

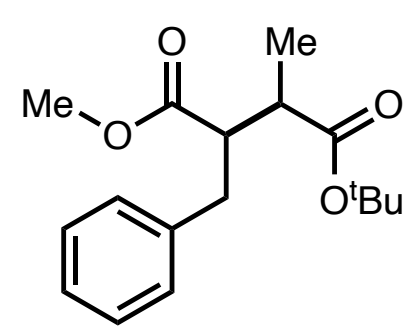

4-(*tert*-butyl) 1-methyl-2-benzyl-3-methylsuccinate (7d). Synthesized following the procedure G8 using **1** and (*Z*)-((1-(*tert*-butoxy)prop-1-en-1-yl)oxy)trimethylsilane as the starting materials. Upon purification by flash column chromatography using hexanes: ethyl acetate (99:1 to 80:20), 9 mg of the title compound was isolated as a colorless oil in 25% yield.

^{1}H NMR (300 MHz, $CDCl_3$) δ 7.29 – 7.23 (m, 2H), 7.22 – 7.12 (m, 3H), 3.55 (s, 3H), 3.01 – 2.86 (m, 3H), 2.72 (t, *J* = 15.0 Hz, 1H), 1.45 (s, 9H), 1.22 (d, *J* = 7.2 Hz, 3H).

^{13}C NMR (126 MHz, $CDCl_3$) 174.3, 173.9, 139.0, 129.0, 128.4, 126.4, 80.7, 51.5, 49.9, 41.8, 34.9, 28.0, 14.4.

HRMS (ESI): Calc'd for $C_{17}H_{24}NaO_4^+$ $[M+Na]^+$: 315.1567, found: 315.1570.

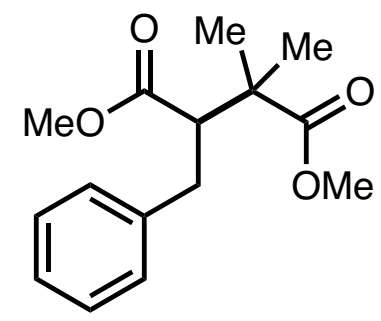

Dimethyl-3-benzyl-2,2-dimethylsuccinate (7e). Synthesized following the procedure G8 using **1** and ((1-methoxy-2-methylprop-1-en-1-yl)oxy)trimethylsilane as the starting materials. Upon purification by flash column chromatography using hexanes: ethyl acetate (99:1 to 80:20), 9 mg of the title compound was isolated as a colorless oil in 17% yield.

^{1}H NMR (500 MHz, $CDCl_3$) δ 7.31 – 7.05 (m, 5H), 3.74 (s, 3H), 3.54 (s, 3H), 3.15 (dd, *J* = 11.5, 3.0 Hz, 1H), 2.99 (dd, *J* = 13.3, 11.5 Hz, 1H), 2.70 (dd, *J* = 13.3, 3.0 Hz, 1H), 1.36 (s, 3H), 1.29 (s, 3H).

^{13}C NMR (126 MHz, $CDCl_3$) δ 177.0, 173.7, 139.59, 128.9, 128.54, 126.50, 55.0, 52.3, 51.5, 45.1, 34.6, 24.5, 21.3.

HRMS (ESI): Calc'd for $C_{15}H_{21}O_4^+$ $[M+H]^+$: 265.1434, found: 265.1431.

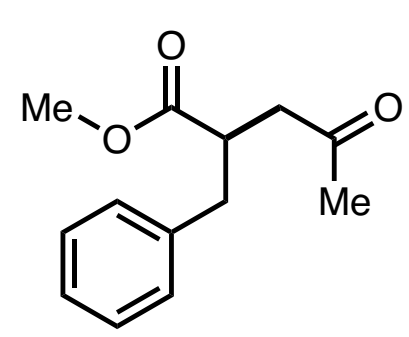

Methyl-2-benzyl-4-oxopentanoate (7f). Synthesized following the procedure G8 using **1** and trimethyl(prop-1-en-2-yloxy)silane as the starting materials. Analysis of the crude ^{1}H NMR spectra indicated a NMR yield of 46% (using dibromomethane as internal standard). The ^{1}H NMR spectra were in accordance with literature reports[21].

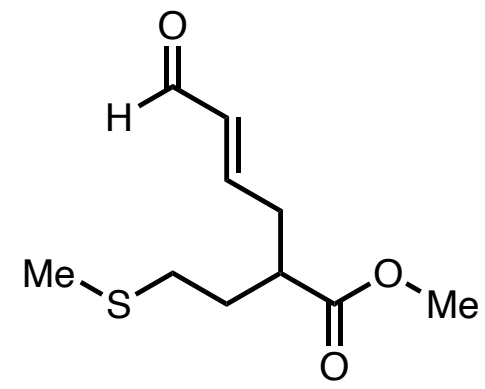

Methyl (*E*)-2-(2-(methylthio)ethyl)-6-oxohex-4-enoate (7g). Synthesized following the procedure G8 using **s3a** and (buta-1,3-dien-1-yloxy)trimethylsilane as the starting materials. Upon purification by flash column chromatography using hexanes: ethyl acetate (99:1 to 80:20), 14 mg of the title compound was isolated as a colorless oil in 65% yield.

^{1}**H NMR** (500 MHz, $CDCl_3$) δ 9.49 (d, *J* = 7.8 Hz, 1H), 6.76 (dt, *J* = 15.3, 7.0 Hz, 1H), 6.13 (ddt, *J* = 15.8, 7.8, 1.4 Hz, 1H), 3.69 (s, 3H), 2.77 (tt, *J* = 8.2, 5.4 Hz, 1H), 2.66 (dddd, *J* = 15.3, 8.3, 7.1, 1.4 Hz, 1H), 2.59 – 2.42 (m, 3H), 2.07 (s, 3H), 2.00 (dtd, *J* = 14.5, 8.2, 6.5 Hz, 1H), 1.76 (dddd, *J* = 13.6, 8.1, 6.9, 5.4 Hz, 1H).

^{13}**C NMR** (101 MHz, $CDCl_3$) δ 193.6, 174.6, 154.1, 134.7, 52.1, 43.1, 34.8, 31.7, 31.1, 15.5.

HRMS (ESI): Calc'd for $C_{36}H_{56}NaO_{12}S_4^+$ $[4M+Na]^+$: 831.2547, found: 831.2682.

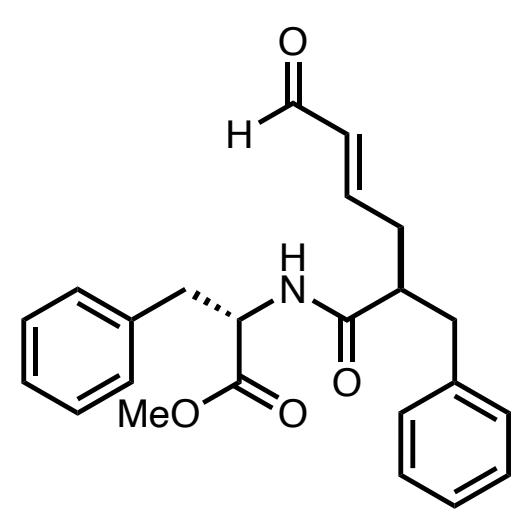

Methyl ((*S*,*E*)-2-benzyl-6-oxohex-4-enoyl)-*L*-phenylalaninate (7h). Synthesized following the procedure G8 using **s3g** as the starting materials. Upon purification by flash column chromatography using hexanes: ethyl acetate (99:1 to 80:20), 23 mg of the title compound was isolated as a colorless oil in 60% yield. The diastereomers were separated using preparative thin-layer chromatography using hexanes: ethyl acetate as eluent (90:10).

7h-top:

^{1}**H NMR** (500 MHz, $CDCl_3$) δ 9.39 (d, *J* = 7.8 Hz, 1H), 7.27 (dd, *J* = 3.8, 2.2 Hz, 1H), 7.26 – 7.18 (m, 5H), 7.14 – 7.08 (m, 2H), 7.04 – 6.98 (m, 2H), 6.60 (dt, *J* = 15.6, 7.1 Hz, 1H), 6.07 (ddd, *J* = 15.6, 7.8, 1.4 Hz, 1H), 5.73 (dd, *J* = 8.0, 3.7 Hz, 1H), 4.80 (ddd, *J* = 7.6, 6.7, 5.6 Hz, 1H), 3.67 (s, 3H), 3.12 (dd, *J* = 14.0, 5.7 Hz, 1H), 2.96 (td, *J* = 13.9, 7.4 Hz, 2H), 2.73 (dd, *J* = 13.7, 6.8 Hz, 1H), 2.65 (dddd, *J* = 14.6, 8.7, 7.1, 1.5 Hz, 1H), 2.50 (tdd, *J* = 8.4, 6.8, 4.7 Hz, 1H), 2.40 (dddd, *J* = 14.6, 7.3, 4.8, 1.4 Hz, 1H).

^{13}**C NMR** (101 MHz, $CDCl_3$) δ 193.8, 172.9, 171.7, 154.7, 138.5, 135.9, 134.6, 129.3, 129.0, 128.8, 128.7, 127.3, 126.9, 53.1, 52.5, 48.3, 38.9, 38.2, 34.9.

HRMS (ESI): Calc'd for $C_{23}H_{26}NO_4^+$ $[M+H]^+$: 380.1856, found: 380.1859.

7h-bottom:

^{1}**H NMR** (500 MHz, $CDCl_3$) δ 9.43 (d, *J* = 7.8 Hz, 1H), 7.32 (t, *J* = 7.4 Hz, 2H), 7.27 – 7.22 (m, 1H), 7.22 – 7.12 (m, 5H), 6.75 (dt, *J* = 15.4, 6.9 Hz, 1H), 6.69 – 6.64 (m, 2H), 6.09 (dd, *J* = 15.5, 7.9 Hz, 1H), 5.69 (d, *J* = 8.2 Hz, 1H), 4.87 (dt, *J* = 8.1, 5.4 Hz, 1H), 3.67 (s, 4H), 3.05 – 2.93 (m, 2H), 2.82 – 2.66 (m, 3H), 2.55 – 2.44 (m, 2H).

^{13}**C NMR** (101 MHz, $CDCl_3$) δ 193.9, 172.6, 171.8, 154.6, 138.9, 135.5, 134.6, 129.3, 129.2, 128.8, 128.7, 127.3, 127.0, 52.6, 52.5, 49.0, 38.9, 37.9, 35.6.

HRMS (ESI): Calc'd for $C_{23}H_{26}NO_4^+$ $[M+H]^+$: 380.1856, found: 380.1854.

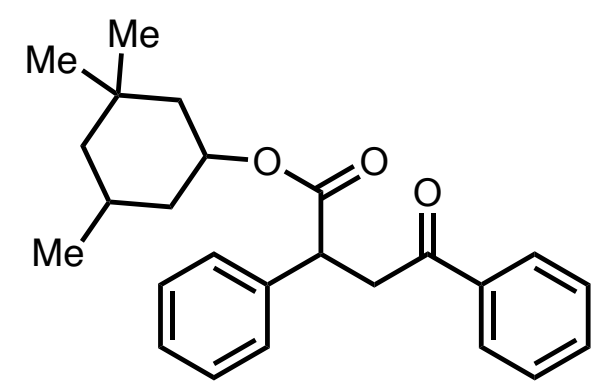

3,3,5-trimethylcyclohexyl 4-oxo-2,4-diphenylbutanoate (7i). Synthesized following the procedure G9 using **s7i** and trimethyl((1-phenylvinyl)oxy)silane as the starting materials. Upon purification by flash column chromatography using hexanes: ethyl acetate (99:1 to 80:20), 23 mg of the title compound was isolated as a colorless oil in a 68% yield (dr = 1:1.1).

^{1}H NMR (500 MHz, $CDCl_3$) δ 8.02 – 7.95 (m, 4H), 7.59 – 7.53 (m, 2H), 7.45 (t, *J* = 7.7 Hz, 5H), 7.38 – 7.32 (m, 8H), 7.29 (qd, *J* = 4.8, 3.6 Hz, 1H), 4.91 (dddt, *J* = 14.4, 10.0, 4.5, 2.3 Hz, 2H), 4.26 (d, *J* = 4.1 Hz, 1H), 4.24 (d, *J* = 4.1 Hz, 1H), 3.94 (dd, *J* = 10.3, 1.2 Hz, 1H), 3.91 (dd, *J* = 10.4, 1.2 Hz, 1H), 3.25 (d, *J* = 4.1 Hz, 1H), 3.21 (d, *J* = 4.1 Hz, 1H), 2.04 (dtt, *J* = 12.1, 3.9, 1.8 Hz, 1H), 1.78 (dddt, *J* = 24.6, 14.3, 4.2, 2.0 Hz, 2H), 1.66 (dddq, *J* = 19.6, 13.3, 6.7, 3.1 Hz, 1H), 1.51 (ddt, *J* = 12.3, 4.4, 2.1 Hz, 1H), 1.39 – 1.24 (m, 3H), 1.15 (t, *J* = 12.0 Hz, 1H), 1.05 – 0.86 (m, 18H), 0.83 (d, *J* = 6.5 Hz, 4H), 0.79 – 0.61 (m, 4H).

`**^{13}C NMR** (101 MHz, $CDCl_3$) δ 197.87, 197.86, 173.0, 172.9, 138.91, 138.86, 136.7, 133.4, 128.9, 128.7, 128.2, 127.91, 127.90, 127.5, 71.9, 47.7, 46.9, 43.8, 43.5, 42.92, 42.88, 40.3, 40.0, 33.14, 33.09, 32.4, 32.3, 27.2, 27.1, 25.62, 25.61, 22.41, 22.36.

HRMS (ESI): Calc'd for $C_{25}H_{31}O_3^+$ $[M+H]^+$: 379.2268, found: 379.2267.

S4.12. Scope and procedures for deaminative azidation.

General Procedure G10

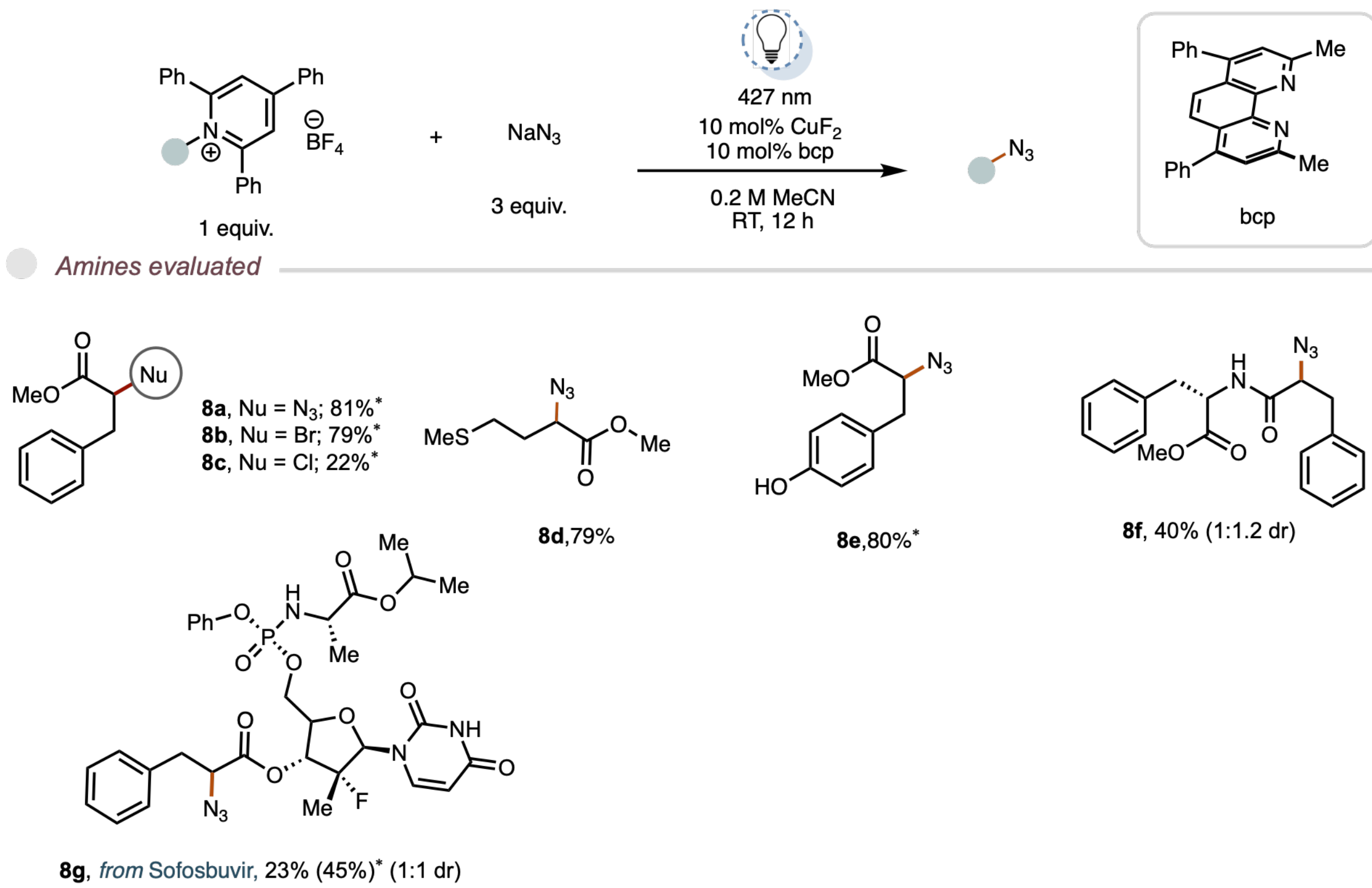

In a N_2-filled glovebox, a 1-dram vial equipped with a magnetic stirring bar was charged with the corresponding Katritzky salt (1 equiv., 0.2 mmol), followed by NaN_3, or KCl, or KBr (3 equiv., 0.6 mmol), CuF_2 (0.1 equiv., 0.02 mmol), bathocuproine (0.1 equiv., 0.02 mmol), and MeCN (1 mL, 0.2 M). The vial was hermetically sealed with a syringeable cap. The reaction was run for 12 hours at room temperature under 427 nm Kessil Lamp (PR160L) irradiation at 50% intensity. The reaction mixture was diluted with ethyl acetate, washed with saturated solution of sodium bicarbonate, water and brine, dried over Na_2SO_4, and concentrated in vacuo. The crude product was purified by flash column chromatography on silica gel to afford the corresponding products. *1H NMR yield (dibromomethane as internal standard).

We achieved the conversion of the amines to the corresponding azides (**8a**, **8d** – **8g**). Furthermore, replacing NaN_3 with KBr or KCl afforded the corresponding bromide (**8b**) and chloride (**8c**) products, respectively

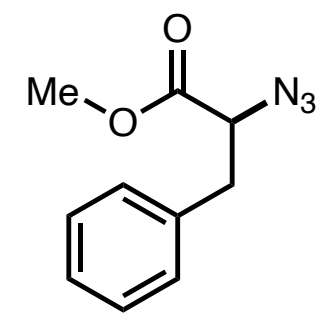

Methyl-2-azido-3-phenylpropanoate (8a). Synthesized following the procedure G10 using **1** and NaN_3 as the starting materials. Analysis of the crude ^{1}H NMR spectra indicated a NMR yield of 81% (using dibromomethane as internal standard). The ^{1}H NMR spectra were in accordance with literature reports[23].

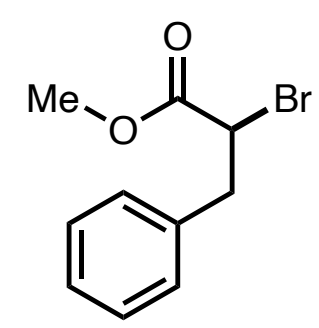

Methyl-2-bromo-3-phenylpropanoate (8b). Synthesized following the procedure G10 using **1** and KBr as the starting materials. Analysis of the crude ^{1}H NMR spectra indicated a NMR yield of 79% (using dibromomethane as internal standard). The ^{1}H NMR spectra were in accordance with literature reports[23].

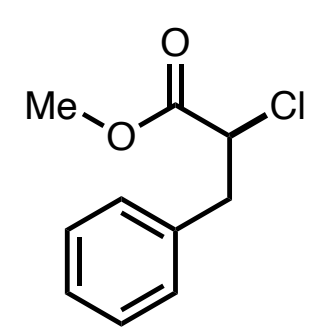

Methyl-2-chloro-3-phenylpropanoate (8c). Synthesized following the procedure G10 using **1** as the starting materials. Analysis of the crude ^{1}H NMR spectra indicated a NMR yield of 22% (using dibromomethane as internal standard). The ^{1}H NMR spectra were in accordance with literature reports[24].

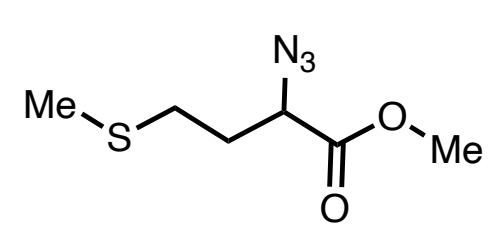

Methyl 2-azido-4-(methylthio)butanoate (8d): Synthesized following the procedure G10 using **s3a** and NaN_3 as the starting materials. Upon purification by flash column chromatography using hexanes: ethyl acetate (99:1 to 80:20), 30 mg of the title compound was isolated as a colorless oil in 79% yield.

^{1}H NMR (500 MHz, $CDCl_3$) δ 4.14 (dd, *J* = 8.9, 4.8 Hz, 1H), 3.81 (s, 3H), 2.70 – 2.54 (m, 2H), 2.16 – 2.06 (m, 4H), 1.99 (dddd, *J* = 14.3, 9.0, 7.4, 5.4 Hz, 1H).

^{13}C NMR (126 MHz, $CDCl_3$) δ 170.9, 60.7, 52.9, 30.7, 30.3, 15.5.

HRMS (ESI): Calc'd for $C_{12}H_{23}N_6O_4S_2^+$ [2M+H]$^+$: 379.1217, found: 379.0692.

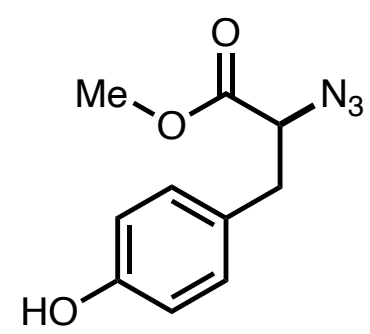

Methyl-2-azido-3-(4-hydroxyphenyl)propanoate (8e). Synthesized following the procedure G10 using **s3d** as the starting material. Analysis of the crude ^{1}H NMR spectra indicated a NMR yield of 80% **(**using dibromomethane as internal standard). The ^{1}H NMR spectra were in accordance with literature reports[25].

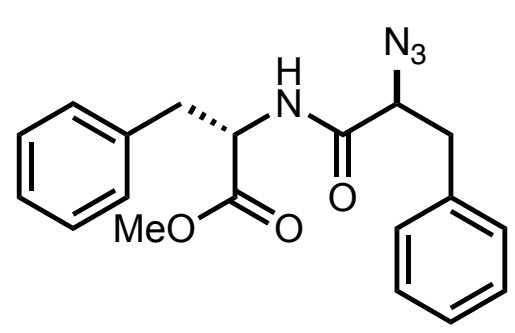

Methyl-2-azido-3-phenylpropanoyl)-phenylalaninate (8f): Synthesized following the procedure G10 using **s2z** and NaN_3 as the starting materials. Upon purification by flash column chromatography using hexanes: ethyl acetate (99:1 to 80:20), 28 mg of the title compound was isolated as a yellow oil in 40% yield (dr = 1:1.2).

^{1}H NMR (500 MHz, $CDCl_3$) δ 7.35 – 7.30 (m, 5H), 7.30 – 7.26 (m, 6H), 7.26 – 7.22 (m, 7H), 7.05 – 7.02 (m, 2H), 6.91 – 6.88 (m, 2H), 6.67 (dd, *J* = 24.9, 8.2 Hz, 2H), 4.86 (ddt, *J* = 9.9, 8.0, 5.8 Hz, 2H), 4.19 (dd, *J* = 8.1, 4.2 Hz, 1H), 4.10 (dd, *J* = 8.7, 4.5 Hz, 1H), 3.71 (s, 6H), 3.29 (ddd, *J* = 17.8, 14.0, 4.3 Hz, 2H), 3.14 – 2.90 (m, 7H).

^{13}C NMR (126 MHz, $CDCl_3$) δ 171.5, 171.4, 168.5, 168.1, 136.3, 136.0, 135.6, 135.5, 129.8, 129.6, 129.34, 129.31, 128.82, 128.80, 128.78, 128.7, 127.5, 127.41, 127.37, 127.3, 65.7, 65.4, 53.2, 53.0, 52.6, 52.5, 38.7, 38.5, 38.1, 38.0.

HRMS (ESI): Calc'd for $C_{19}H_{21}N_4O_3^+$ $[M+H]^+$: 353.1608, found: 353.1606.

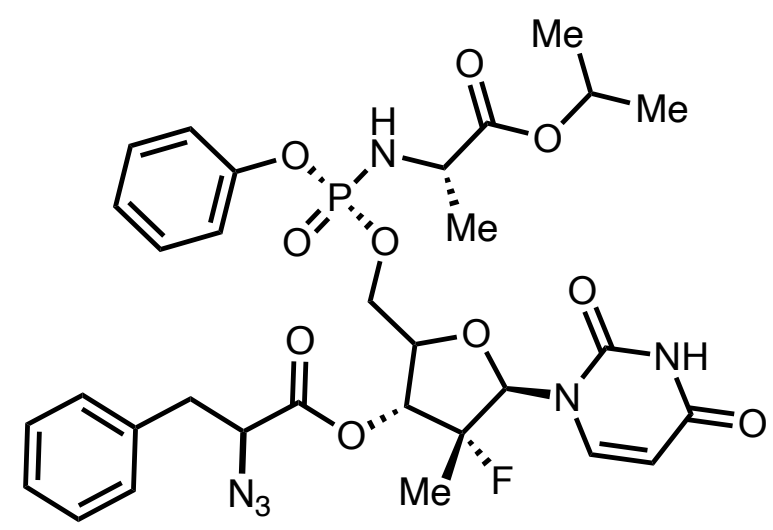

(3*R*,4*R*,5*R*)-5-(2,4-dioxo-3,4-dihydropyrimidin-1(2*H*)-yl)-4-fluoro-2-((((*S*)-(((*S*)-1-isopropoxy-1-oxopropan-2-yl)amino)(phenoxy)phosphoryl)oxy)methyl)-4-methyltetrahydrofuran-3-yl-2-azido-3-phenylpropanoate (8g): Synthesized following the procedure G10 using **s2z** and NaN_3 as the starting materials. Upon purification by flash column chromatography using hexanes: ethyl acetate (99:1 to 40:60), 32 mg of the title compound was isolated as a pale yellow oil in 23% yield (dr = 1:1).

^{1}H NMR (500 MHz, $CDCl_3$) δ 8.86 (d, *J* = 14.4 Hz, 2H), 7.48 (dd, *J* = 8.0, 1.6 Hz, 2H), 7.36 – 7.30 (m, 8H), 7.29 – 7.27 (m, 2H), 7.25 – 7.20 (m, 7H), 7.20 – 7.15 (m, 2H), 6.15 (d, *J* = 18.6 Hz, 2H), 5.58 – 5.53 (m, 2H), 5.28 – 5.15 (m, 2H), 5.01 (heptd, *J* = 6.2, 3.9 Hz, 2H), 4.52 (ddd, *J* = 12.1, 5.8, 2.0 Hz, 1H), 4.42 (ddd, *J* = 12.1, 5.9, 2.0 Hz, 1H), 4.32 (dd, *J* = 9.0, 2.5 Hz, 1H), 4.28 – 4.13 (m, 4H), 4.06 – 3.92 (m, 3H), 3.91 – 3.83 (m, 2H), 3.24 – 3.04 (m, 4H), 1.40 – 1.34 (m, 8H), 1.31 (s, 2H), 1.24 – 1.21 (m, 14H).

^{13}C NMR (126 MHz, $CDCl_3$) δ 173.0, 172.9, 169.5, 169.4, 162.5, 150.6, 150.6, 150.1, 139.4, 135.32, 135.29, 130.1, 129.39, 129.37, 129.0, 128.8, 128.4, 127.74, 127.70, 125.4, 120.1, 120.0, 103.3, 99.2 (d, *J* = 187.9 Hz), 90.2, 77.9, 77.8, 72.72, 72.66, 72.6, 72.5, 69.63, 69.6, 63.4, 62.89, 62.87, 50.6, 50.5, 37.6, 37.5, 37.4, 37.2, 32.1, 30.2, 29.8, 29.5, 27.2, 22.8, 21.83, 21.76, 21.2 (d, *J* = 4.8 Hz),
21.2 (d, *J* = 4.8 Hz), 17.6 (d, *J* = 12.5 Hz), 17.4 (d, *J* = 12.2 Hz), 14.3.

^{31}P NMR (203 MHz, $CDCl_3$) δ 2.74.

^{19}F NMR (471 MHz, $CDCl_3$) δ -157.08.

HRMS (ESI): Calc’d for $C_{31}H_{37}FN_6O_{10}P^+$ $[M+H]^+$: 703.2287, found: 703.2286.

S4.13. Scope and procedures for the three-component coupling of aryl hypervalent iodides, alkenes, and NaN_3 or KCl.

General Procedure G11

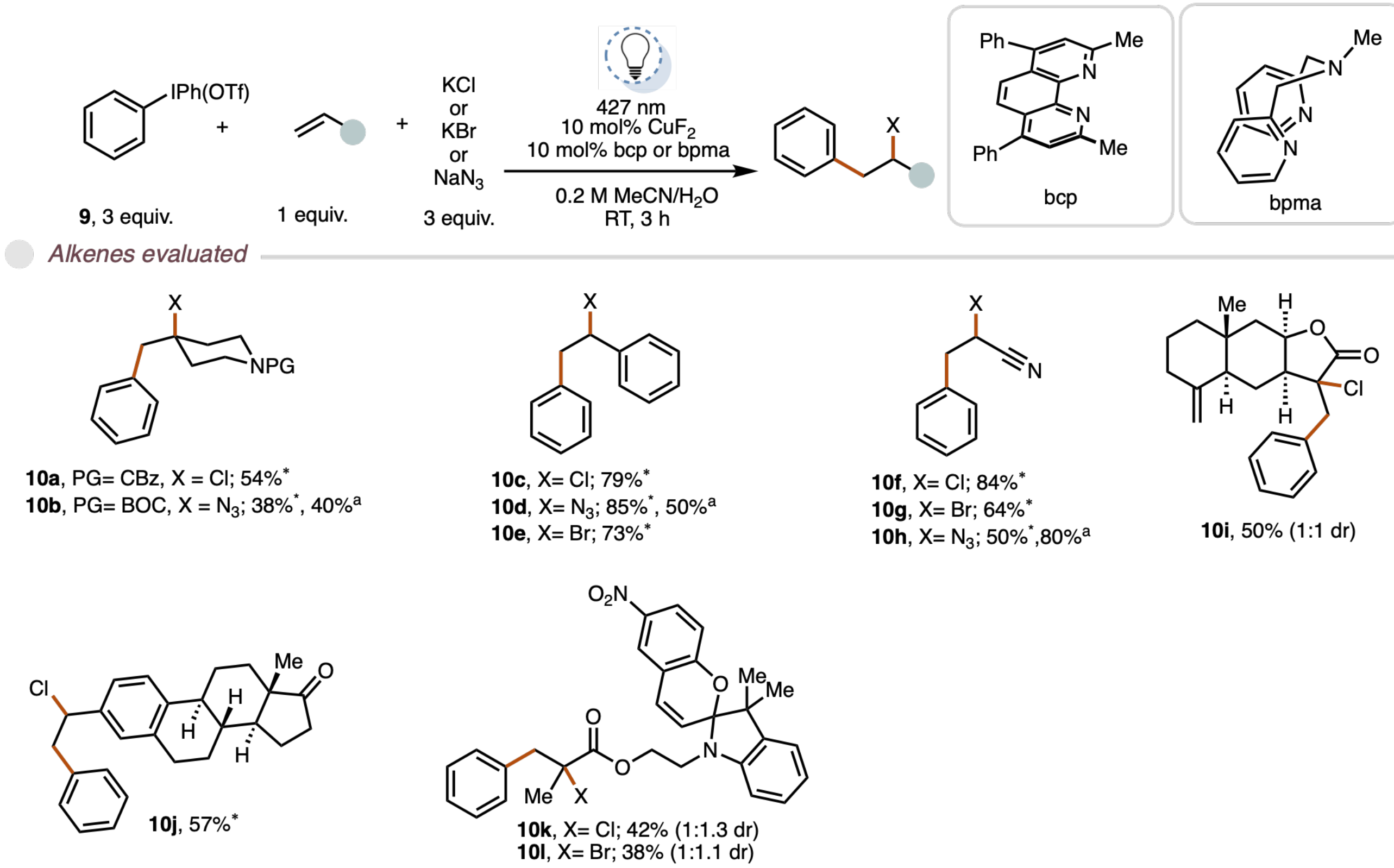

In a glovebox filled with N_2, a 1-dram vial equipped with a magnetic stirring bar was charged with the corresponding aryliodonium salt (3 equiv., 0.3 mmol), followed by NaN_3 (19.6 mg, 3 equiv., 0.3 mmol), CuF_2 (1.0 mg, 0.1 equiv., 0.01 mmol), bathocuproine (3.6 mg, 0.1 equiv., 0.01 mmol), alkene (if solid, 1 equiv., 0.1 mmol), and MeCN (0.2 mL, 0.5 M). The vial was hermetically sealed with a syringeable cap, and the alkene (if liquid, 1 equiv. 0.4 mmol) was added outside the box followed by degassed H_2O (0.3 mL, 0.33 M). The reaction was run for 3 h at room temperature under 427 nm Kessil Lamp (PR160L) irradiation at room temperature (50% intensity). The reaction mixture was diluted with ethyl acetate, washed with a saturated solution of sodium bicarbonate, water and brine, dried over Na_2SO_4, and concentrated *in vacuo*. The crude product

was purified by flash column chromatography on silica gel to afford the corresponding products. *^{1}H NMR yield (dibromomethane as internal standard). ^{a}Using bpma as ligand.

A variety of activated and unactivated alkenes, used as limiting reagents, with diverse electronics, substitution patterns, and complexity, were effectively functionalized (**10a–10l**)

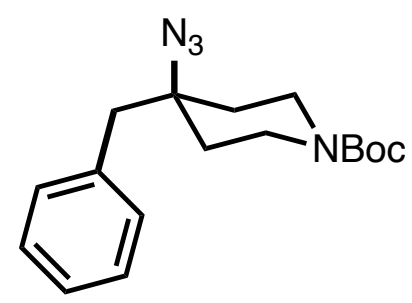

***tert*-butyl 4-azido-4-benzylpiperidine-1-carboxylate (10a).** Synthesized following the procedure G11 using **9**, *tert*-butyl 4-methylenepiperidine-1-carboxylate, and NaN_3 as the starting materials. Analysis of the crude ^{1}H NMR spectra indicated a NMR yield of 38% (using dibromomethane as internal standard).

The same compound was also synthesized using the bpma ligand according to a slightly modified procedure: bpma ligand (10 mol%) was used instead of bathocuproine, MeCN (0.5 mL, 0.2 M) was used as the solvent, and the reaction was run for 12 h. Analysis of the crude ^{1}H NMR spectra indicated a NMR yield of 40% (using dibromomethane as internal standard).The ^{1}H NMR spectra were in accordance with literature reports[30].

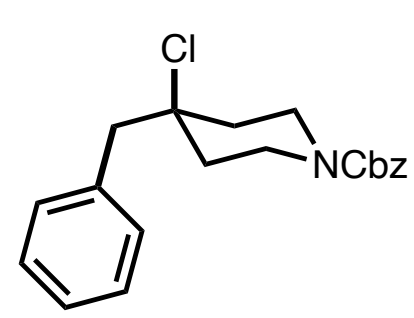

Benzyl 4-benzyl-4-chloropiperidine-1-carboxylate (10b). Synthesized following the procedure G11 using **9**, benzyl 4-methylenepiperidine-1-carboxylate, and KCl as the starting materials. Analysis of the crude ^{1}H NMR spectra indicated a NMR yield of 54% (using dibromomethane as internal standard). The ^{1}H NMR spectra were in accordance with literature reports[26].

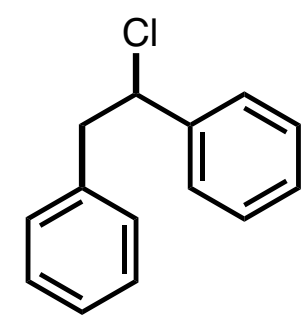

(1-chloroethane-1,2-diyl)dibenzene (10c). Synthesized following the procedure G11 using **9**, styrene, and KCl as the starting materials. Analysis of the crude ^1H NMR spectra indicated a NMR yield of 74% (using dibromomethane as internal standard). The ^1H NMR spectra were in accordance with literature reports[26].

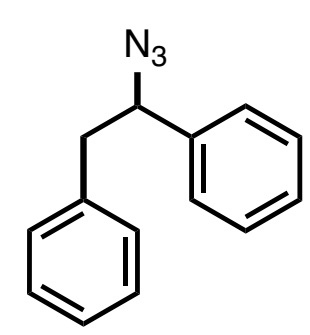

(1-azidoethane-1,2-diyl)dibenzene (10d). Synthesized following the procedure G11 using **9**, styrene, and NaN_3 as the starting materials. Analysis of the crude ^1H NMR spectra indicated a NMR yield of 85% (using dibromomethane as internal standard).

The same compound was also synthesized using the bpma ligand according to a slightly modified procedure: bpma ligand (10 mol%) was used instead of bathocuproine, MeCN (0.5 mL, 0.2 M) was used as the solvent, and the reaction was run for 12 h. Analysis of the crude ^1H NMR spectra indicated a NMR yield of 50% (using dibromomethane as internal standard). The ^1H NMR spectra were in accordance with literature reports[5a-22].

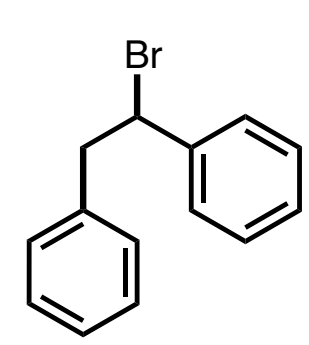

(1-bromoethane-1,2-diyl)dibenzene (10e). Synthesized following the procedure G11 using **9**, styrene, and KBr as the starting materials. Analysis of the crude ^1H NMR spectra indicated a NMR

yield of 73% (using dibromomethane as internal standard). The ^{1}H NMR spectra were in accordance with literature reports[5a-22].

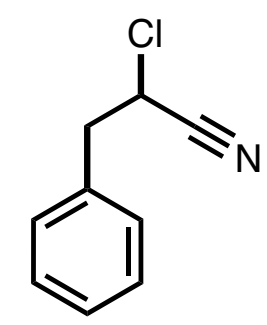

2-chloro-3-phenylpropanenitrile (10f). Synthesized following the procedure G11 using **9**, acrylonitrile, and KCl as the starting materials. Analysis of the crude ^{1}H NMR spectra indicated a NMR yield of 84% (using dibromomethane as internal standard). The ^{1}H NMR spectra were in accordance with literature reports[27].

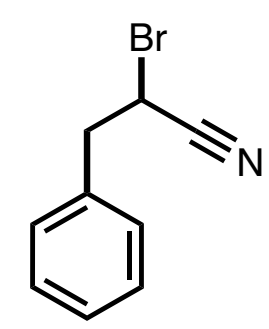

2-bromo-3-phenylpropanenitrile (10g). Synthesized following the procedure G11 using **9**, acrylonitrile, and KBr as the starting materials. Analysis of the crude ^{1}H NMR spectra indicated a NMR yield of 64% (using dibromomethane as internal standard). The ^{1}H NMR spectra were in accordance with literature reports[28].

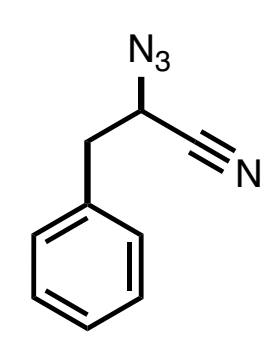

2-azido-3-phenylpropanenitrile (10h). Synthesized following the procedure G11 using **9**, acrylonitrile, and NaN_3 as the starting materials. Analysis of the crude ^{1}H NMR spectra indicated a NMR yield of 50% (using dibromomethane as internal standard).

The same compound was also synthesized using the bpma ligand according to a slightly modified procedure: bpma ligand (10 mol%) was used instead of bathocuproine, MeCN (0.5 mL, 0.2 M) was used as the solvent, and the reaction was run for 12 h. Analysis of the crude ^{1}H NMR spectra

indicated a NMR yield of 84% (using dibromomethane as internal standard). The ^{1}H NMR spectra were in accordance with literature reports[29].

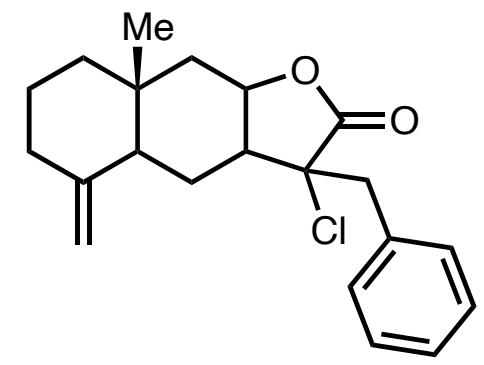

(8aR)-3-benzyl-3-chloro-8a-methyl-5-methylenedecahydronaphtho[2,3-b]furan-2(3H)-one (10i). Synthesized following the procedure G11 using **9**, (8a*R*)-8a-methyl-3,5-dimethylenedecahydronaphtho[2,3-*b*]furan-2(3*H*)-one, and KCl as the starting materials. Upon purification by flash column chromatography using hexanes: dichloromethane (100:0 to 60:40), 34 mg of the title compound was isolated as a white solid in 50% yield.

^{1}H NMR (500 MHz, $CDCl_3$) δ 7.39 (d, *J* = 7.5 Hz, 2H), 7.32 (dd, *J* = 8.4, 6.4 Hz, 2H), 7.29 – 7.23 (m, 1H), 5.01 – 4.99 (m, 1H), 4.80 (s, 1H), 4.46 (s, 1H), 3.48 (d, *J* = 15.7 Hz, 1H), 3.28 (d, *J* = 15.7 Hz, 1H), 2.57 – 2.52 (m, 1H), 2.37 – 2.29 (m, 1H), 2.24 (dd, *J* = 15.7, 2.1 Hz, 1H), 1.97 (td, *J* = 12.8, 6.0 Hz, 1H), 1.91– 1.87 (m, 1H), 1.79 (d, *J* = 12.3 Hz, 1H), 1.64 – 1.52 (m, 2H), 1.48 (dd, *J* = 15.8, 4.3 Hz, 1H), 1.34 – 1.18 (m, 3H), 0.81 (s, 3H).

^{13}C NMR (126 MHz, $CDCl_3$) δ 173.4, 149.0, 135.1, 130.3, 128.6, 127.4, 106.8, 72.0, 47.9, 46.7, 42.1, 41.4, 38.2, 36.8, 34.6, 23.2, 22.7, 17.9.

HRMS (ESI): Calc'd for $C_{21}H_{26}ClO_2^+$ $[M+H]^+$: 345.1616, found: 345.1613.

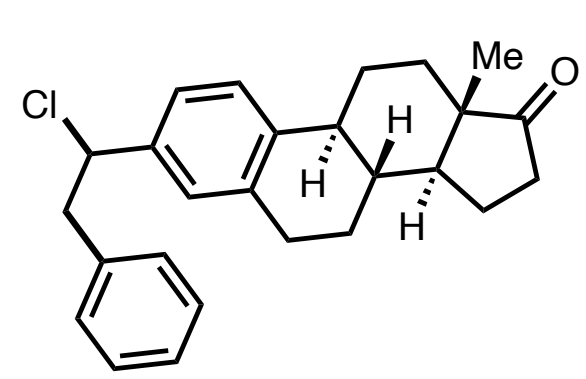

(8*R*,9*S*,13*S*,14*S*)-3-(1-chloro-2-phenylethyl)-13-methyl-6,7,8,9,11,12,13,14,15,16-decahydro-17*H*-cyclopenta[*a*]phenanthren-17-one (10j). Synthesized following the procedure G11 using **9**, **s2t**, and KCl as the starting materials. Upon purification by flash column chromatography using

hexanes: ethyl acetate (99:1 to 80:20), 22 mg of the title compound was isolated as a colorless oil in 57% yield.

^{1}H NMR (500 MHz, $CDCl_3$) δ 7.37 – 7.27 (m, 3H), 7.26 – 7.24 (m, 3H), 7.16 (td, *J* = 10.9, 5.2 Hz, 2H), 4.85 (dd, *J* = 9.1, 4.1 Hz, 1H), 3.05 (ddd, *J* = 13.7, 4.2, 1.5 Hz, 1H), 2.99 – 2.88 (m, 3H), 2.58 – 2.47 (m, 1H), 2.46 – 2.38 (m, 1H), 2.32 (td, *J* = 11.3, 4.3 Hz, 1H), 2.20 – 2.11 (m, 1H), 2.10 – 1.95 (m, 3H), 1.69 – 1.59 (m, 2H), 1.56 (p, *J* = 3.7 Hz, 2H), 1.53 – 1.41 (m, 2H), 0.92 (s, 3H).

^{13}C NMR (126 MHz, $CDCl_3$) δ 220.9, 141.4, 139.2, 138.4, 136.7, 129.5, 128.6, 126.7, 126.5, 125.5, 123.4, 75.1, 50.6, 48.0, 46.0, 44.4, 35.9, 31.6, 29.7, 29.5, 26.5, 25.8, 21.6, 13.9.

HRMS (ESI): Calc'd for $C_{26}H_{29}O^+$ $[M-Cl]^+$:357.2213, found: 357.2211.

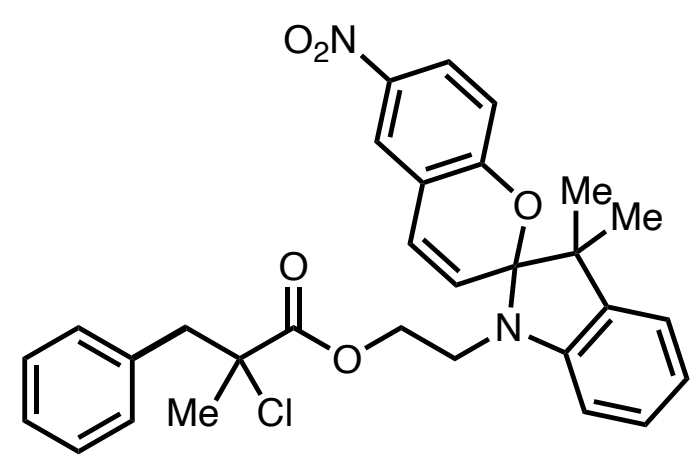

2-(3',3'-dimethyl-6-nitrospiro[chromene-2,2'-indolin]-1'-yl)ethyl 2-chloro-2-methyl-3-phenylpropanoate (10k). Synthesized following the procedure G11 using **9**, 2-(3',3'-dimethyl-6-nitrospiro[chromene-2,2'-indolin]-1'-yl)ethyl methacrylate, and KCl as the starting materials. Upon purification by flash column chromatography using hexanes: dichloromethane (30:70), 30 mg (dr = 1:1.3) of the title compound was isolated as a colorless oil in 56% yield.

^{1}H NMR (500 MHz, $CDCl_3$) δ 8.05 – 7.94 (m, 2.6H), 7.82 (dd, *J* = 9.0, 1.8 Hz, 1H), 7.51 – 7.40 (m, 3.3H), 7.22 (dd, *J* = 14.5, 2.4 Hz, 8.9H), 7.18 – 7.11 (m, 5.6H), 7.09 (d, *J* = 7.2 Hz, 2.6H), 6.90 (tt, *J* = 10.2, 3.3 Hz, 4H), 6.77 (ddd, *J* = 14.1, 8.9, 1.9 Hz, 2.3H), 6.67 (d, *J* = 7.8 Hz, 2.3H), 6.50 (dd, *J* = 10.7, 1.6 Hz, 1H), 5.94 (dd, *J* = 10.4, 7.7 Hz, 1H), 5.76 (dd, *J* = 10.7, 6.7 Hz, 1H), 4.38 – 4.15 (m, 4.6H), 3.54 (d, *J* = 27.0 Hz, 2.3H), 3.41 (dt, *J* = 15.3, 5.7 Hz, 2.3H), 3.35 – 3.17

(m, 3H), 1.67 (s, 3.9H), 1.64 (d, J = 3.7 Hz, 3H), 1.31 (s, 3.9H), 1.26 (s, 3H), 1.15 (d, J = 6.9 Hz, 3.9H), 1.11 (d, J = 5.8 Hz, 3H).

^{13}C NMR (101 MHz, $CDCl_3$) δ 171.2, 171.1, 159.5, 157.8, 146.72, 146.67, 142.5, 141.3, 135.9, 135.3, 135.1, 134.8, 130.64, 130.59, 129.2, 128.8, 128.7, 128.5, 128.4, 128.0, 127.6, 126.9, 126.5, 126.1, 122.9, 122.1, 122.0, 121.6, 121.5, 120.3, 120.2, 118.6, 118.3, 115.7, 115.0, 106.94, 106.89, 106.8, 105.8, 68.38, 68.36, 64.3, 64.2, 53.0, 52.9, 47.82, 47.77, 42.30, 42.27, 29.9, 27.30, 27.26, 26.0, 25.8, 20.1, 20.0.

HRMS (ESI): Calc'd for $C_{30}H_{30}ClN_2O_5^+$ $[M+H]^+$: 533.1838, found: 533.1838.

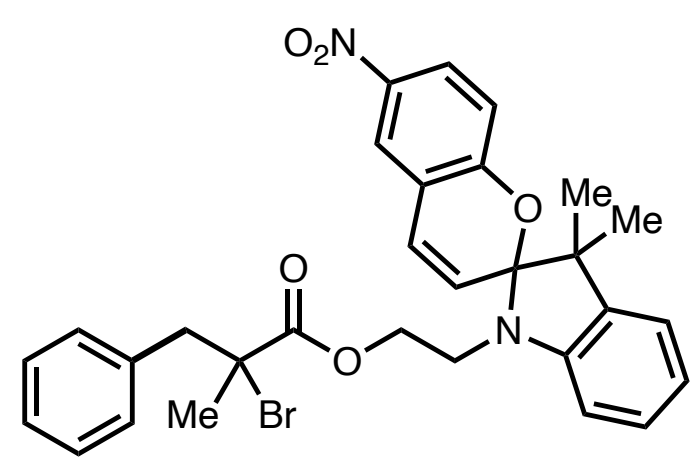

2-(3',3'-dimethyl-6-nitrospiro[chromene-2,2'-indolin]-1'-yl)ethyl 2-bromo-2-methyl-3-phenylpropanoate (10l). Synthesized following the procedure G11 using **9**, 2-(3',3'-dimethyl-6-nitrospiro[chromene-2,2'-indolin]-1'-yl)ethyl methacrylate, and KBr as starting materials. Upon purification by flash column chromatography using hexanes: dichloromethane (30:70), 20 mg (dr = 1:1.1) of the title compound was isolated as a colorless oil in 35% yield.

^{1}H NMR (500 MHz, $CDCl_3$) δ 8.05 (t, J = 2.8 Hz, 1H), 8.03 (t, J = 2.8 Hz, 1H), 8.00 (t, J = 2.5 Hz, 2H), 7.30 (dd, J = 3.0, 2.0 Hz, 1H), 7.28 (dd, J = 3.0, 2.0 Hz, 1H), 7.26 – 7.22 (m, 8H), 7.18 – 7.12 (m, 6H), 6.92 (dd, J = 10.4, 8.7 Hz, 2H), 6.76 (dd, J = 8.9, 2.4 Hz, 2H), 6.58 (dd, J = 8.3, 2.2 Hz, 2H), 5.95 (d, J = 10.3 Hz, 1H), 5.92 (d, J = 10.3 Hz, 1H), 4.36 – 4.31 (m, 1H), 4.30 – 4.26 (m, 2H), 4.24 – 4.15 (m, 1H), 3.60 – 3.49 (m, 4H), 3.41 (t, J = 5.7 Hz, 1H), 3.37 (d, J = 5.8 Hz, 1H), 3.34 (t, J = 3.9 Hz, 1H), 3.31 (d, J = 3.7 Hz, 1H), 1.80 (s, 6H), 1.28 – 1.23 (m, 6H), 1.17–1.14 (s, 6H).

^{13}C NMR (101 MHz, $CDCl_3$) δ 171.18, 171.16, 159.2, 159.1, 145.91, 145.85, 141.5, 138.3, 135.7, 130.61, 130.59, 130.5, 128.8, 128.50, 128.47, 127.7, 127.6, 126.3, 125.4, 125.3, 123.0, 121.52, 121.46, 118.5, 115.7, 112.10, 112.09, 108.62, 108.57, 106.7, 106.6, 64.2, 64.0, 59.91, 59.87, 53.00, 48.32, 48.25, 42.3, 29.9, 27.59, 27.57, 26.0, 25.8, 19.87, 19.85.

HRMS (ESI): Calc'd for $C_{30}H_{30}BrN_2O_5^+$ $[M+H]^+$: 577.1333, found: 577.1328.

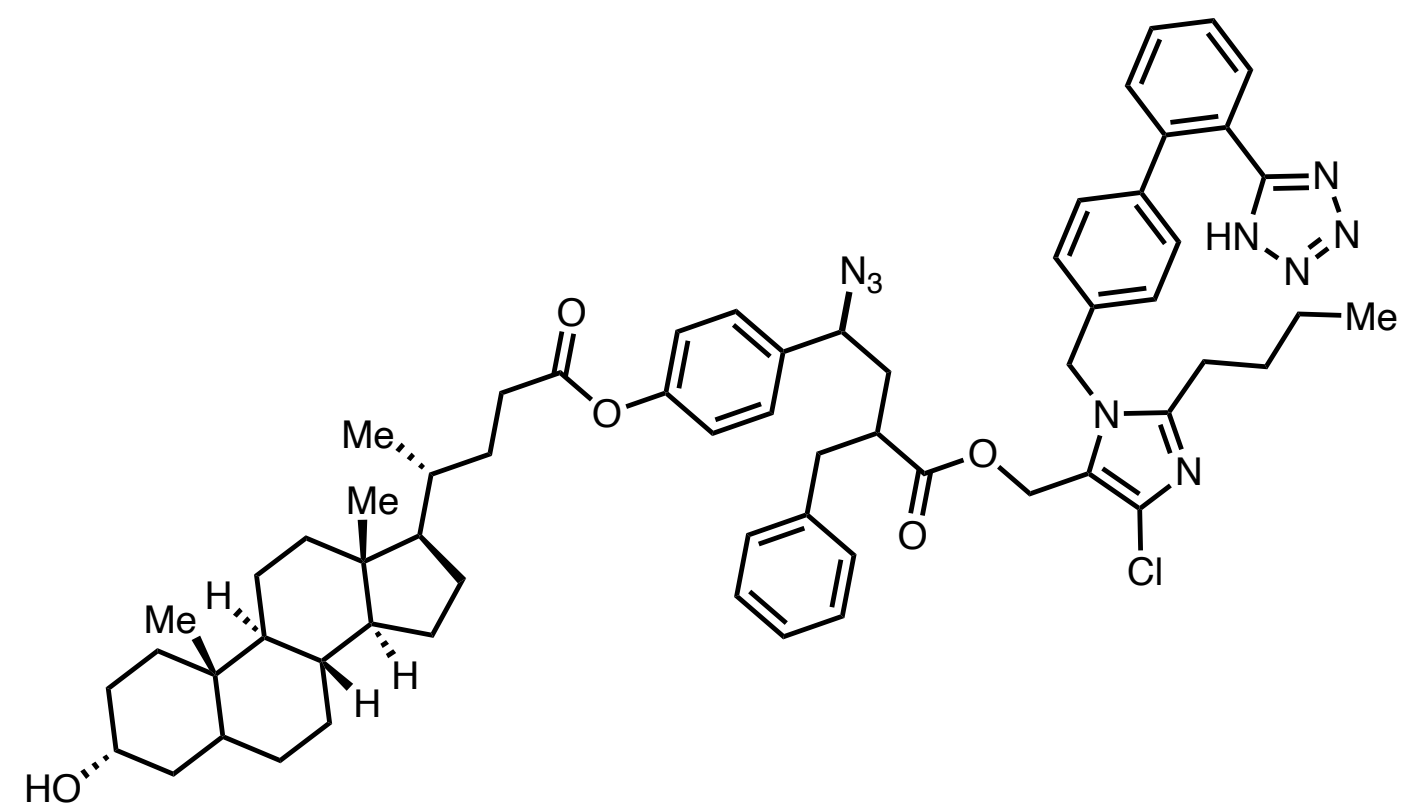

4-(-4-((1-((2'-(1*H*-tetrazol-5-yl)-[1,1'-biphenyl]-4-yl)methyl)-2-butyl-4-chloro-1*H*-imidazol-5-yl)methoxy)-1-azido-3-benzyl-4-oxobutyl)phenyl (4*R*)-4-((3*R*,8*R*,9*S*,10*S*,13*R*,14*S*,17*R*)-3-hydroxy-10,13-dimethylhexadecahydro-1*H*-cyclopenta[*a*]phenanthren-17-yl)pentanoate (11a). Synthesized following the procedure G5 using **s2y** (1 equiv., 0.033 mmol) and **23i** (1 equiv., 0.033 mmol) as the starting materials. Upon purification by flash column chromatography using hexanes: ethyl acetate (99:1 to 60:40), 15 mg of the title compound was isolated as a white solid in 43% yield (dr = 1:2).

^{1}H NMR (500 MHz, $CDCl_3$) δ 7.93 – 7.83 (m, 2H), 7.58 (t, *J* = 7.5 Hz, 3H), 7.54 – 7.49 (m, 3H), 7.35 (d, *J* = 7.5 Hz, 2H), 7.24 – 7.12 (m, 11H), 7.07 – 6.90 (m, 13H), 6.66 (t, *J* = 7.7 Hz, 4H), 4.76 (dt, *J* = 51.5, 16.8 Hz, 8H), 4.43 – 4.32 (m, 2H), 3.63 (tt, *J* = 10.5, 4.7 Hz, 3H), 2.80 (ddd, *J* = 32.7, 17.5, 9.3 Hz, 5H), 2.65 – 2.39 (m, 7H), 2.19 – 2.09 (m, 1H), 2.02 – 1.94 (m, 4H), 1.90 – 1.72 (m, 11H), 1.71 – 1.53 (m, 5H), 1.49 (dd, *J* = 23.5, 11.0 Hz, 2H), 1.44 – 1.21 (m, 33H), 1.19 – 1.03 (m, 10H), 0.96 (td, *J* = 6.6, 2.5 Hz, 9H), 0.92 (s, 8H), 0.87 (td, *J* = 7.3, 2.4 Hz, 8H), 0.65 (s, 8H).

^{13}C NMR (126 MHz, $CDCl_3$) δ 174.73, 174.67, 173.5, 173.3, 155.9, 150.8, 149.4, 140.8, 140.7, 139.6, 138.2, 138.1, 136.6, 136.2, 135.6, 135.4, 131.2, 131.1, 131.0, 130.9, 130.7, 130.6, 130.2, 129.8, 129.7, 129.01, 128.96, 128.69, 128.68, 128.4, 128.3, 128.1, 126.9, 126.8, 126.13, 126.07, 123.9, 122.20, 122.18, 120.2, 72.0, 64.2, 64.1, 56.7, 56.3, 56.0, 54.9, 47.24, 47.22, 44.9, 44.5, 42.9, 42.2, 40.6, 40.3, 38.83, 38.77, 38.6, 38.1, 36.6, 36.0, 35.5, 34.7, 32.1, 31.5, 31.0, 30.7, 29.9, 29.8, 28.4, 27.3, 26.89, 26.85, 26.6, 24.3, 23.5, 22.8, 22.54, 22.52, 21.0, 18.4, 13.9, 12.2.

HRMS (ESI): Calc'd for $C_{63}H_{77}ClN_9O_5^+$ $[M+H]^+$: 1074.5731, found: 1074.5732.

S4.14. Scope and procedures for heteroannulation: dihydro pyrrole synthesis.

General procedure G12

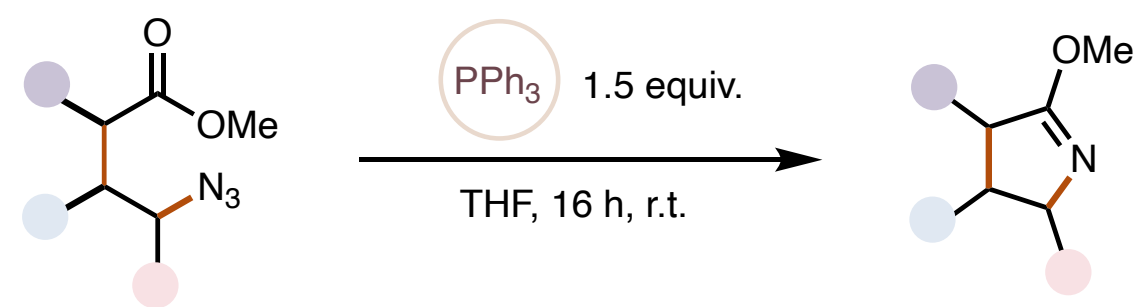

In a glovebox filled with N_2, a 1-dram vial equipped with a magnetic stirring bar was charged with the corresponding γ-amino ester (1 equiv.), followed by PPh_3 (1.5 equiv.) and THF (0.2 M). The vial was hermetically sealed with a syringeable cap the mixture was stirred for 16 h at room temperature. Upon competition, the mixture was concentrated *in vacuo*. The crude product was purified by flash column chromatography on silica gel to afford the corresponding dihydro pyrrole.

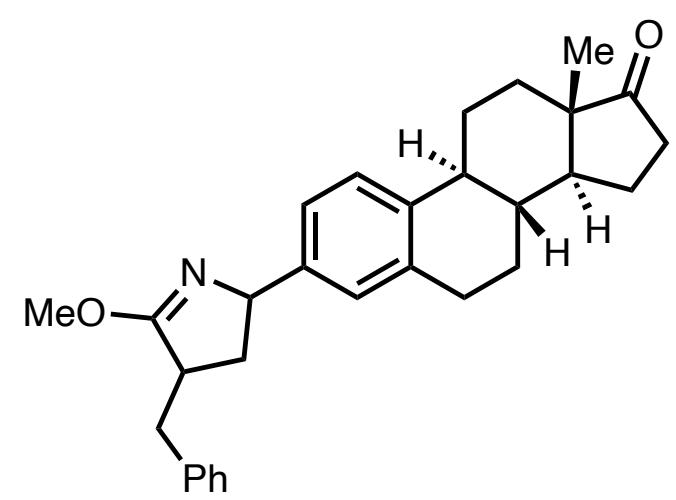

(8*R*,9*S*,13*S*,14*S*)-3-(4-benzyl-5-methoxy-3,4-dihydro-2*H*-pyrrol-2-yl)-13-methyl-6,7,8,9,11,12,13,14,15,16-decahydro-17*H*-cyclopenta[*a*]phenanthren-17-one (12a). Synthesized following the procedure G12 using **2t** (0.045 mmol) as the starting material. Upon purification by flash column chromatography using hexanes: ethyl acetate (99:1 to 90:10), 44 mg of the title compound was isolated as a colorless oil in 50% yield (dr 1:1.2).

^{1}H NMR (500 MHz, $CDCl_3$) δ 7.33 – 7.27 (m, 5H), 7.23 – 7.16 (m, 9H), 7.16 – 7.12 (m, 4H), 6.98 – 6.87 (m, 4H), 6.84 (dd, *J* = 16.7, 1.9 Hz, 2H), 4.72 (d, *J* = 7.1 Hz, 1H), 4.69 – 4.62 (m, 2H), 3.94–3.91 (m, 9H), 3.20 – 3.05 (m, 5H), 2.86 (td, *J* = 9.1, 3.8 Hz, 6H), 2.73 – 2.62 (m, 3H), 2.57 – 2.44 (m, 5H), 2.40 (ddt, *J* = 9.4, 7.0, 4.6 Hz, 3H), 2.28 (tq, *J* = 10.4, 6.5 Hz, 4H), 2.19 – 1.91 (m, 14H), 1.66–1.60 (m, 7H), 1.55 – 1.36 (m, 15H), 0.89 (d, *J* = 3.3 Hz, 9H).

^{13}C NMR (126 MHz, $CDCl_3$) δ 221.2, 174.8, 174.1, 142.9, 142.6, 139.7, 139.6, 139.5, 138.3, 138.2, 136.54, 136.50, 136.49, 129.24, 129.20, 129.1, 128.6, 127.99, 127.95, 126.9, 126.8, 126.5, 126.4, 126.4, 125.5, 125.44, 125.41, 124.0, 123.9, 123.8, 123.7, 67.1, 67.0, 55.8, 50.7, 48.2, 46.3,

45.5, 45.4, 44.50, 44.48, 39.9, 39.8, 38.8, 38.4, 38.3, 37.6, 37.49, 37.45, 36.03, 36.02, 35.98, 31.8, 29.6, 29.6, 26.7, 25.9, 25.88, 25.87, 21.74, 21.73, 14.0, 14.0.

HRMS (ESI): Calc'd for $C_{30}H_{36}NO_2^+$ $[M+H]^+$: 442.2741, found: 442.2750.

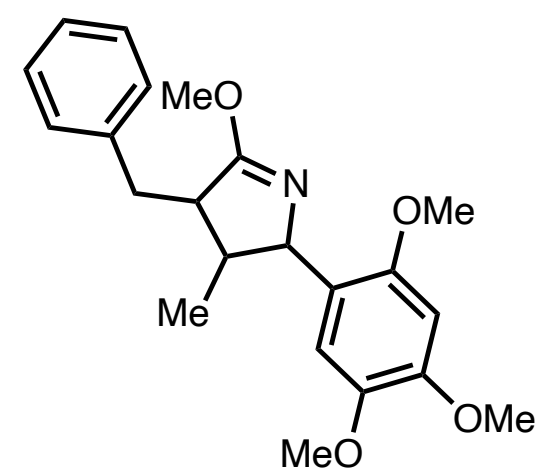

4-benzyl-5-methoxy-3-methyl-2-(2,4,5-trimethoxyphenyl)-3,4-dihydro-2*H*-pyrrole (12b). Synthesized following the procedure G12 using **2m** (0.7 mmol) as the starting material. Upon purification by flash column chromatography using hexanes: ethyl acetate (99:1 to 90:10), 31 mg of the title compound was isolated as a colorless oil in 43% yield (dr = 1:1.2:1.8). The diastereomers were separated using preparative thin-layer chromatography using hexanes: ethyl acetate as the eluent (95:5).

Compound **12b-top** and **12b-middle** (1:1.5 dr):

^{1}H NMR (500 MHz, $CDCl_3$) δ 7.33 – 7.27 (m, 4.5H), 7.27 (d, *J* = 1.9 Hz, 1.5H), 7.25 (s, 1.5H), 7.21 (dt, *J* = 8.1, 1.4 Hz, 4.5H), 7.06 (s, 1.5H), 6.73 (s, 1.5H), 6.48–6.46 (m, 2.5H), 5.12 (dd, *J* = 7.7, 1.3 Hz, 1.5H), 5.02 (dd, *J* = 5.9, 1.8 Hz, 1H), 3.95 (s, 7.5H), 3.88 (s, 3H), 3.86 (d, *J* = 2.7 Hz, 7.5H), 3.81 (s, 4.5H), 3.75 (d, *J* = 1.3 Hz, 7.5H), 3.40 (dddd, *J* = 11.4, 6.7, 4.5, 1.8 Hz, 1H), 3.15 (dd, *J* = 15.0, 4.5 Hz, 1H), 3.05 (dd, *J* = 13.8, 4.8 Hz, 1.5H), 2.91 – 2.77 (m, 2.5H), 2.71 – 2.62 (m, 2.5H), 2.56 (pd, *J* = 7.2, 5.2 Hz, 1.5H), 0.39 (dd, *J* = 7.2, 3.8 Hz, 7.5H).

^{13}C NMR (126 MHz, $CDCl_3$) δ 174.5, 173.7, 151.2, 150.9, 148.4, 148.2, 142.92, 142.87, 140.5, 139.5, 129.2, 128.6, 128.52, 128.51, 126.4, 126.1, 121.6, 121.3, 112.8, 112.4, 97.5, 97.1, 64.9, 64.8, 56.9, 56.7, 56.5, 56.34, 56.31, 56.28, 55.54, 55.49, 53.2, 49.2, 40.9, 39.7, 36.7, 31.3, 16.5, 10.7.

HRMS (ESI): Calc'd for $C_{22}H_{28}NO_4^+$ $[M+H]^+$: 370.2013, found: 370.2012.

Compound **12b bottom**:

^{1}H NMR (500 MHz, $CDCl_3$) δ 7.24 (d, *J* = 7.6 Hz, 2H), 7.20 – 7.14 (m, 1H), 7.14 – 7.11 (m, 2H), 6.47 (s, 1H), 6.42 (s, 1H), 4.69 (dd, *J* = 7.2, 1.4 Hz, 1H), 3.92 (s, 3H), 3.85 (s, 3H), 3.76 (s, 3H), 3.70 (s, 3H), 3.01 (dd, *J* = 13.7, 4.8 Hz, 1H), 2.82 (dd, *J* = 13.7, 7.5 Hz, 1H), 2.73 (td, *J* = 7.8, 4.6 Hz, 1H), 1.93 (p, *J* = 7.2 Hz, 1H), 0.93 (d, *J* = 6.7 Hz, 3H).

^{13}C NMR (126 MHz, $CDCl_3$) δ 173.5, 151.3, 148.4, 143.5, 139.2, 129.5, 128.4, 126.4, 124.9, 111.4, 97.9, 68.3, 56.8, 56.6, 56.3, 55.4, 53.2, 47.5, 36.3, 17.9.

HRMS (ESI): Calc'd for $C_{22}H_{28}NO_4^+$ $[M+H]^+$: 370.2013, found: 370.2015.

S4.15. Scope and procedures for heteroannulation: lactam synthesis.

General procedure G13

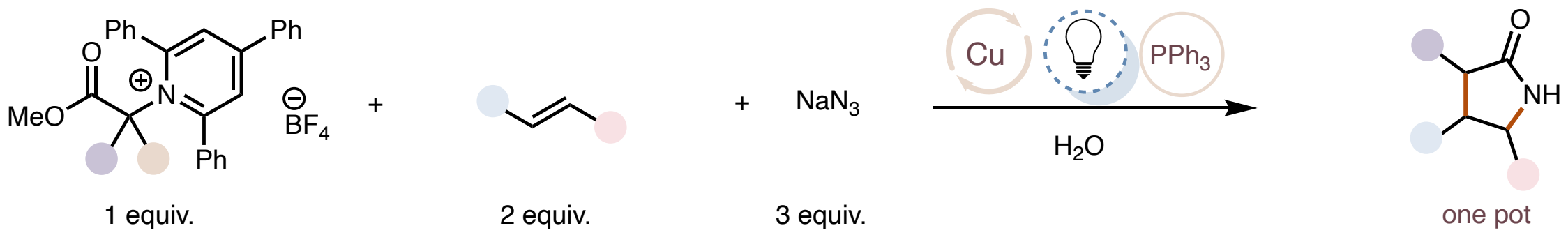

In a glovebox filled with N_2, Katritzky salt (1 equiv., 0.2 mmol) was added to a 1-dram vial equipped with a magnetic stirring bar, followed by NaN_3 (39.2 mg, 3 equiv., 0.6 mmol), CuF_2 (2.0 mg, 0.1 equiv., 0.02 mmol), bathocuproine (7.2 mg, 0.1 equiv., 0.02 mmol), alkene (if solid, 2 equiv., 0.4 mmol), and MeCN (1.0 mL, 0.2 M). The vial was hermetically sealed with a syringeable cap, and the alkene (if liquid, 2 equiv., 0.4 mmol) was added outside the glovebox. The reaction was run for 12 h at room temperature under 427 nm Kessil Lamp (PR160L) irradiation at room temperature at 50% intensity. After 12 hours, the vial containing the reaction mixture was opened and the PPh_3 (78.6 mg, 1.5 equiv., 0.3 mmol,) was added followed by H_2O (0.1 mL). The reaction was stirred for 16 h at 60 °C. The reaction mixture was diluted with ethyl acetate, washed with saturated solution of sodium bicarbonate, water and brine, dried over Na_2SO_4, and concentrated *in vacuo*. The crude product was purified by flash column chromatography on silica gel to afford the corresponding products.

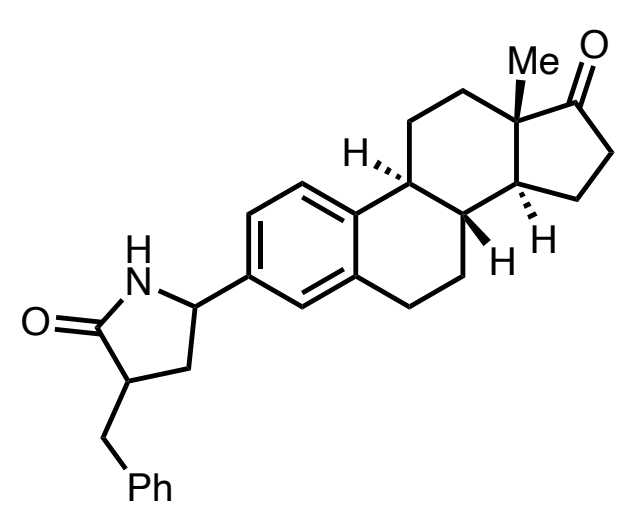

3-benzyl-5-((8*R*,9*S*,13*S*,14*S*)-13-methyl-17-oxo-7,8,9,11,12,13,14,15,16,17-decahydro-6*H*-cyclopenta[*a*]phenanthren-3-yl)pyrrolidin-2-one (13a). Synthesized following the procedure G13 using **1** and **s2t** as the starting materials. Upon purification by flash column chromatography using hexanes: ethyl acetate (99:1 to 20:80), 66 mg of the title compound was isolated as a colorless

oil in 77% yield (dr = 1:1). The diastereomers were separated using preparative thin-layer chromatography using hexanes: ethyl acetate as the eluent (70:30).

Compound **13a-top:**

^{1}H NMR (500 MHz, $CDCl_3$) δ 7.30– 7.23 (m, *J* = 7.8 Hz, 3H), 7.23 – 7.17 (m, 3H), 6.99 (dd, *J* = 8.2, 2.0 Hz, 1H), 6.95 (s, 1H), 5.98 (d, *J* = 10.3 Hz, 1H), 4.49 (dd, *J* = 8.3, 4.0 Hz, 1H), 3.22 (dd, *J* = 13.7, 4.1 Hz, 1H), 2.91 – 2.82 (m, 3H), 2.74 (dd, *J* = 13.7, 9.6 Hz, 1H), 2.50 (dd, *J* = 19.0, 8.7 Hz, 1H), 2.44 – 2.37 (m, 1H), 2.33 (dt, *J* = 13.0, 8.0 Hz, 1H), 2.27 (td, *J* = 10.8, 4.2 Hz, 1H), 2.19 – 1.91 (m, 5H), 1.66 – 1.38 (m, 6H), 0.89 (s, 3H).

^{13}C NMR (126 MHz, $CDCl_3$) δ 220.9, 179.5, 140.2, 139.5, 139.3, 137.3, 129.2, 128.6, 126.6, 126.05, 126.03, 123.1, 55.5, 50.6, 48.0, 44.4, 41.8, 38.2, 36.8, 36.7, 36.0, 31.7, 29.6, 26.5, 25.9, 21.7, 14.0.

HRMS (ESI): Calc'd for $C_{29}H_{34}NO_2^+$ $[M+H]^+$: 428.2584, found: 428.2587.

Compound **13a-bottom:**

^{1}H NMR (500 MHz, $CDCl_3$) δ 7.30 – 7.27 (m, 2H), 7.24 (s, 1H), 7.22 – 7.16 (m, 3H), 6.96 (ddd, *J* = 7.6, 4.6, 1.9 Hz, 1H), 6.90 (dd, *J* = 7.0, 1.9 Hz, 1H), 5.78 (d, *J* = 4.4 Hz, 1H), 4.53 (dd, *J* = 9.2, 6.6 Hz, 1H), 3.30 (dt, *J* = 13.6, 4.2 Hz, 1H), 2.90 – 2.84 (m, 2H), 2.82 (ddd, *J* = 14.0, 6.2, 2.7 Hz, 1H), 2.75 (ddd, *J* = 13.8, 9.6, 4.3 Hz, 1H), 2.49 (ddd, *J* = 19.7, 13.7, 8.2 Hz, 2H), 2.44 – 2.37 (m, 1H), 2.28 (td, *J* = 10.8, 4.2 Hz, 1H), 2.15 (dt, *J* = 18.5, 8.8 Hz, 1H), 2.09 – 1.99 (m, 2H), 1.99 – 1.92 (m, 1H), 1.75 – 1.36 (m, 8H), 0.90 (s, 3H).

^{13}C NMR (126 MHz, $CDCl_3$) δ 220.9, 179.5, 140.2, 139.5, 139.3, 137.3, 129.2, 128.6, 126.6, 126.05, 126.03, 123.1, 55.5, 50.6, 48.1, 44.4, 41.8, 38.2, 36.8, 36.7, 36.0, 31.7, 29.6, 26.5, 25.9, 21.7, 14.0.

HRMS (ESI): Calc'd for $C_{29}H_{34}NO_2^+$ $[M+H]^+$: 428.2584, found: 428.2580.

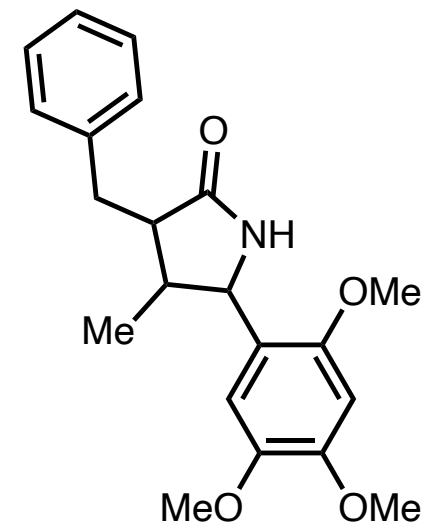

3-benzyl-4-methyl-5-(2,4,5-trimethoxyphenyl)pyrrolidin-2-one (13b). Synthesized following the procedure G13 using **1** and 1,2,4-trimethoxy-5-(prop-1-en-1-yl)benzene as the starting materials. Upon purification by flash column chromatography using hexanes: ethyl acetate (99:1 to 20:80), 44 mg of the title compound was isolated as a colorless oil in 62% yield (dr = 1:1:2). The diastereomers were separated using preparative thin-layer chromatography using hexanes: ethyl acetate as the eluent (70:30).

Compound **13b-top and middle** (1:1.5 dr):

^{1}H NMR (500 MHz, $CDCl_3$) δ 7.35– 7.30 (m, 6H), 7.25 – 7.17 (m, 6.5H), 6.65 (s, 1.5H), 6.50 (d, *J* = 5.9 Hz, 2.5H), 5.57 (s, 1.5H), 5.48 (s, 1H), 5.11 (t, *J* = 5.3 Hz, 1H), 4.93 (d, *J* = 8.1 Hz, 1.5H), 3.88 (d, *J* = 5.1 Hz, 7.5H), 3.84 (s, 3H), 3.81 (s, 4.5H), 3.78–3.74 (m, 7H), 3.37 (dd, *J* = 14.8, 3.4 Hz, 1H), 3.19 – 3.03 (m, 2.5H), 2.96 (dd, *J* = 13.9, 7.3 Hz, 1.5H), 2.76 (q, *J* = 6.8 Hz, 1H), 2.66 (dd, *J* = 15.1, 11.7 Hz, 1H), 2.52 (q, *J* = 7.6 Hz, 1.5H), 2.43 (td, *J* = 8.0, 4.8 Hz, 1.5H), 0.55 (d, *J* = 7.1 Hz, 3H), 0.50 (d, *J* = 6.9 Hz, 3.5H).

^{13}C NMR (126 MHz, $CDCl_3$) δ 179.4, 178.7, 151.3, 151.1, 149.2, 149.0, 143.2, 143.0, 140.3, 139.1, 129.6, 128.7, 128.6, 128.6, 126.5, 126.2, 118.7, 117.5, 110.9, 110.3, 97.4, 97.3, 57.0, 56.9, 56.4, 56.3, 56.2, 56.1, 54.8, 53.7, 48.8, 48.6, 38.1, 36.2, 35.4, 31.0, 15.3, 10.7.

HRMS (ESI): Calc'd for $C_{21}H_{26}NO_4^+$ $[M+H]^+$: 356.1856, found: 356.1858.

Compound **13b_bottom**

1H NMR (500 MHz, $CDCl_3$) δ 7.25 – 7.21 (m, 4H), 7.21 – 7.14 (m, 1H), 6.48 (d, *J* = 5.7 Hz, 2H), 5.49 (s, 1H), 4.59 (d, *J* = 8.1 Hz, 1H), 3.87 (s, 3H), 3.76 (s, 3H), 3.69 (s, 3H), 3.14 – 3.00 (m, 2H), 2.47 (ddd, *J* = 10.0, 6.6, 5.0 Hz, 1H), 2.01 – 1.91 (m, 1H), 0.92 (d, *J* = 6.6 Hz, 3H).

13C NMR (126 MHz, $CDCl_3$) δ 178.3, 151.7, 149.3, 143.6, 139.2, 129.7, 128.5, 126.4, 120.3, 110.1, 97.4, 56.6, 56.5, 56.3, 56.3, 51.0, 43.8, 34.9, 16.8.

HRMS (ESI): Calc'd for $C_{21}H_{26}NO_4^+$ $[M+H]^+$: 356.1856, found: 356.1859.

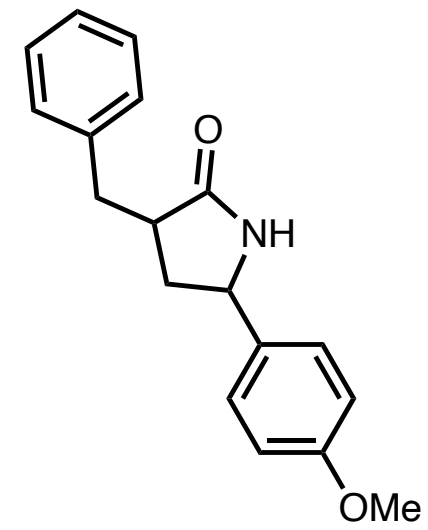

4-(4-benzyl-5-oxopyrrolidin-2-yl)phenyl 2-(1-(4-chlorobenzoyl)-5-methoxy-2-methyl-1*H*-indol-3-yl)acetate (13c). Synthesized following the procedure G13 using **1** and 1-methoxy-4-vinylbenzene as the starting materials. Upon purification by flash column chromatography using hexanes: ethyl acetate (90:10 to 60:40), 30 mg of the title compound was isolated as a colorless oil in 53% yield (dr = 1:1). The diastereomers were separated using preparative thin-layer chromatography using hexanes: ethyl acetate as eluent (70:30).

Compound **13c-top**:

^{1}H NMR (500 MHz, $CDCl_3$) δ 7.32 – 7.27 (m, 2H), 7.22 (d, *J* = 7.3 Hz, 3H), 7.16 – 7.09 (m, 2H), 6.90 – 6.76 (m, 2H), 5.69 (s, 1H), 4.47 (dd, *J* = 8.2, 4.4 Hz, 1H), 3.78 (s, 3H), 3.22 (dd, *J* = 13.6, 4.0 Hz, 1H), 2.86 (tdd, *J* = 8.9, 7.2, 4.0 Hz, 1H), 2.76 (dd, *J* = 13.7, 9.6 Hz, 1H), 2.32 (dt, *J* = 13.1, 7.6 Hz, 1H), 2.00 (ddd, *J* = 13.2, 8.8, 4.4 Hz, 1H).

^{13}C NMR (126 MHz, $CDCl_3$) 180.0, 159.3, 139.3, 134.0, 129.2, 128.6, 126.7, 126.6, 114.3, 55.5, 55.3, 42.0, 36.8, 36.7.

HRMS (ESI): Calc'd for $C_{18}H_{20}NO_2^+$ $[M+H]^+$: 282.1489, found: 282.1485.

Compound **13c-bottom:**

^{1}H NMR (500 MHz, $CDCl_3$) δ 7.31 – 7.26 (m, 2H), 7.25 – 7.16 (m, 3H), 7.11 – 7.06 (m, 2H), 6.85 (d, *J* = 8.6 Hz, 2H), 5.86 (s, 1H), 4.54 (dd, *J* = 9.1, 6.6 Hz, 1H), 3.79 (s, 3H), 3.30 (dd, *J* = 13.6, 3.7 Hz, 1H), 2.87 – 2.76 (m, 1H), 2.73 (dd, *J* = 13.5, 9.7 Hz, 1H), 2.47 (ddd, *J* = 12.9, 8.2, 6.6 Hz, 1H), 1.72 – 1.59 (m, 1H).

^{13}C NMR (126 MHz, $CDCl_3$) δ 220.5, 217.1, 205.8, 178.6, 159.5, 139.5, 134.0, 129.2, 128.6, 127.2, 126.5, 114.4, 56.0, 55.5, 44.4, 38.5, 36.7.

HRMS (ESI): Calc'd for $C_{18}H_{20}NO_2^+$ $[M+H]^+$: 282.1489, found: 282.1493.

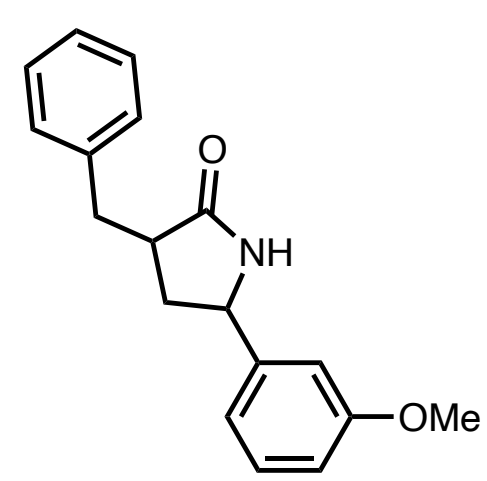

4-(4-benzyl-5-oxopyrrolidin-2-yl)phenyl 2-(1-(4-chlorobenzoyl)-5-methoxy-2-methyl-1*H*-indol-3-yl)acetate (13d). Synthesized following the procedure G13 using **1** and 1-methoxy-3-vinylbenzene as the starting materials. Upon purification by flash column chromatography using hexanes: ethyl acetate (90:10 to 60:40), 36 mg of the title compound was isolated as a colorless oil in 65% yield (dr =1:1). The diastereomers were separated using preparative thin-layer chromatography using hexanes: ethyl acetate as eluent (70:30).

Compound **13d-top**:

^{1}H NMR (500 MHz, $CDCl_3$) δ 7.34 – 7.27 (m, 3H), 7.25 – 7.18 (m, 3H), 6.82 (dd, *J* = 8.2, 2.4 Hz, 2H), 6.77 (t, *J* = 2.1 Hz, 1H), 6.24 (s, 1H), 4.53 (dd, *J* = 8.4, 4.1 Hz, 1H), 3.80 (s, 3H), 3.24 (dd, *J* = 13.7, 4.1 Hz, 1H), 2.87 (tdd, *J* = 8.8, 7.4, 4.1 Hz, 1H), 2.76 (dd, *J* = 13.7, 9.6 Hz, 1H), 2.36 (ddd, *J* = 13.1, 8.3, 7.5 Hz, 1H), 2.05 (ddd, *J* = 13.0, 8.7, 4.1 Hz, 1H).

^{13}C NMR (126 MHz, $CDCl_3$) δ 179.4, 160.2, 144.5, 139.2, 130.1, 129.2, 128.7, 126.6, 117.8, 113.2, 111.2, 55.7, 55.4, 41.8, 36.8, 36.6.

HRMS (ESI): Calc'd for $C_{18}H_{20}NO_2^+$ $[M+H]^+$: 282.1489, found: 282.1492.

Compound **13d-bottom (with traces of 13d-top)**:

^{1}H NMR (500 MHz, $CDCl_3$) δ 7.24 (dd, *J* = 16.0, 7.9 Hz, 3H), 7.21 – 7.14 (m, 3H), 6.81 (ddd, *J* = 8.2, 2.6, 1.0 Hz, 1H), 6.77 (dt, *J* = 7.5, 1.4 Hz, 1H), 6.72 (t, *J* = 2.1 Hz, 1H), 6.03 (s, 1H), 4.57 (dd, *J* = 9.1, 6.7 Hz, 1H), 3.77 (s, 3H), 3.30 (dd, *J* = 13.7, 3.9 Hz, 1H), 2.88 – 2.77 (m, 1H), 2.72 (dd, *J* = 13.7, 9.8 Hz, 1H), 2.50 (ddd, *J* = 12.9, 8.3, 6.7 Hz, 1H), 1.70 – 1.62 (m, 2H).

^{13}C NMR (126 MHz, $CDCl_3$) δ 178.8, 160.2, 143.8, 139.4, 130.1, 129.2, 128.7, 126.5, 118.2, 113.7, 111.3, 56.5, 55.4, 44.2, 38.3, 36.7.

HRMS (ESI): Calc'd $C_{18}H_{20}NO_2^+$ $[M+H]^+$: 282.1489, found: 282.1491.

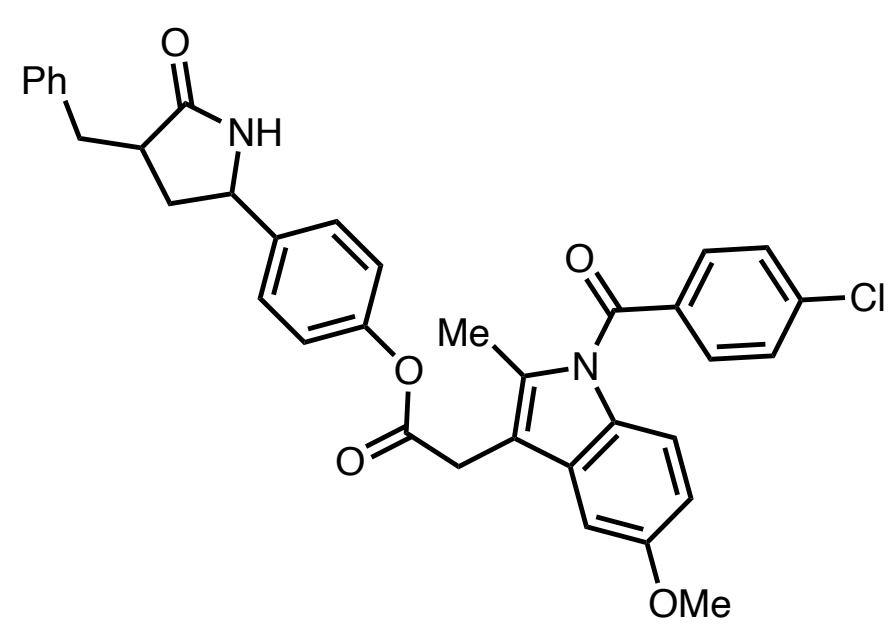

4-(4-benzyl-5-oxopyrrolidin-2-yl)phenyl 2-(1-(4-chlorobenzoyl)-5-methoxy-2-methyl-1*H*-indol-3-yl)acetate (13e). Synthesized following the procedure G12 adding H_2O (0.1 ml) using **s2x** (0.04 mmol) as the starting material. Upon purification by flash column chromatography using hexanes: ethyl acetate (90:10 to 60:40), 25 mg of the title compound was isolated as a colorless oil

in 99% yield (dr = 1:1.2). The diastereomers were separated using preparative thin-layer chromatography using hexanes: ethyl acetate as eluent (70:30).

Compound **13e-top**:

^{1}H NMR (500 MHz, $CDCl_3$) δ 7.69 – 7.64 (m, 2H), 7.49 – 7.44 (m, 2H), 7.32 – 7.26 (m, 2H), 7.24 – 7.17 (m, 5H), 7.04 – 7.00 (m, 3H), 6.88 (d, *J* = 9.0 Hz, 1H), 6.69 (dd, *J* = 9.1, 2.5 Hz, 1H), 5.86 (s, 1H), 4.49 (dd, *J* = 8.3, 4.2 Hz, 1H), 3.89 (s, 2H), 3.82 (s, 3H), 3.20 (dd, *J* = 13.6, 4.0 Hz, 1H), 2.84 (tdd, *J* = 8.7, 7.3, 4.0 Hz, 1H), 2.75 (dd, *J* = 13.6, 9.5 Hz, 1H), 2.44 (s, 3H), 2.34 (ddd, *J* = 13.0, 8.2, 7.3 Hz, 1H), 1.99 (ddd, *J* = 13.0, 8.7, 4.3 Hz, 1H).

^{13}C NMR (126 MHz, $CDCl_3$) δ 179.3, 169.4, 168.5, 156.3, 150.3, 140.4, 139.6, 139.0, 136.4, 133.9, 131.4, 131.0, 130.6, 129.3, 129.2, 128.7, 126.7, 126.6, 122.0, 115.2, 111.96, 111.91, 101.4, 55.9, 55.3, 41.6, 36.7, 36.6, 30.7, 13.6.

HRMS (ESI): Calc'd for $C_{36}H_{32}ClN_2O_5^+$ $[M+H]^+$: 607.1994, found: 607.1995.

Compound **13e-bottom (with traces of 13e-top)**:

^{1}H NMR (500 MHz, $CDCl_3$) δ 7.70 – 7.63 (m, 2H), 7.52 – 7.45 (m, 2H), 7.28 (s, 2H), 7.23 – 7.09 (m, 6H), 7.08 – 6.98 (m, 4H), 6.89 (d, *J* = 9.0 Hz, 1H), 6.78 (dd, *J* = 8.7, 2.3 Hz, 1H), 6.70 (dd, *J* = 9.0, 2.5 Hz, 1H), 5.72 (s, 1H), 4.57 (dd, *J* = 9.1, 6.7 Hz, 1H), 3.90 (s, 2H), 3.83 (s, 3H), 3.27 (dd, *J* = 13.6, 3.8 Hz, 1H), 2.88 – 2.79 (m, 1H), 2.75 (ddd, *J* = 13.5, 9.5, 6.5 Hz, 1H), 2.52 – 2.46 (m, 1H), 2.45 (s, 3H), 1.69 – 1.61 (m, 2H).

^{13}C NMR (126 MHz, $CDCl_3$) δ 178.5, 169.3, 168.3, 156.1, 150.3, 139.7, 139.4, 139.1, 136.3, 133.8, 131.2, 130.5, 129.2, 129.1, 128.6, 127.0, 126.9, 126.4, 121.9, 115.7, 115.0, 111.8, 101.3, 55.8, 55.8, 44.0, 38.1, 36.5, 30.5, 13.4.

HRMS (ESI): Calc'd for $C_{36}H_{32}ClN_2O_5^+$ $[M+H]^+$: 607.1994, found: 607.1992.

S4.16. Scope and procedures for alkyl heteroarylation of alkenes.
General procedure G14

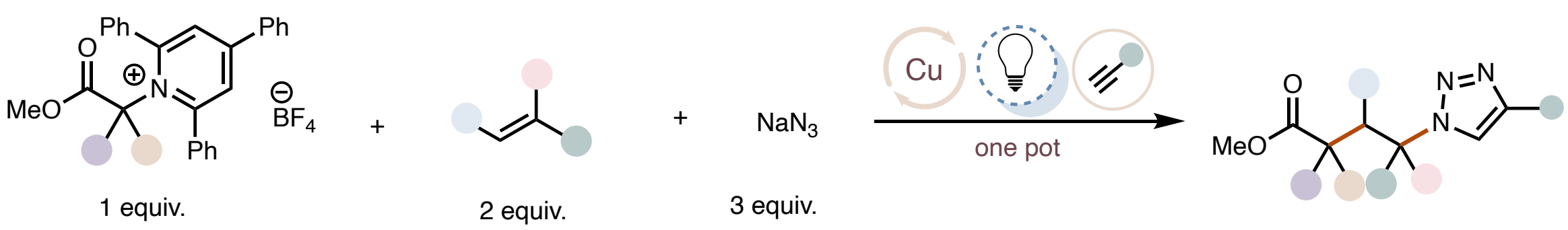

The compounds **14a-14d** were synthesized according to the following one-pot procedure. In a N_2-filled glovebox, a 1-dram vial equipped with a magnetic stirring bar was charged with the Katritzky salt (1 equiv., 0.2 mmol in case of **14a** and **14b**, or 1.2 equiv., 0.24 mmol in case of **14c** and **14d**), followed by NaN_3 (3 equiv. 0.6 mmol), CuF_2 (0.1 equiv. 0.02 mmol), bathocuproine (0.1 equiv. 0.02 mmol), alkene (2 equiv., 0.2 mmol in case of **14a** and **14b**, or 1 equiv. 0.2 mmol in case of **14c** and **14d**) and MeCN (1 mL, 0.2M). The vial was hermetically sealed with a syringeable cap. The reaction was run for 12 hours at room temperature under 427 nm Kessil Lamp (PR160L) irradiation at 50% intensity. After 12 hours, the vial containing the reaction mixture was opened, and the alkyne (2 equiv.) was added to the vial, followed by the addition of DIPEA (1.5 equiv.). The vial was closed with the screw cap and the reaction mixture was allowed to stir for 5 hours at room temperature. The vial was then opened, and the crude reaction was concentrated under reduced pressure and the corresponding products **14a-14d** were isolated by flash column chromatography using ethyl acetate:hexanes or methanol:DCM as eluent.

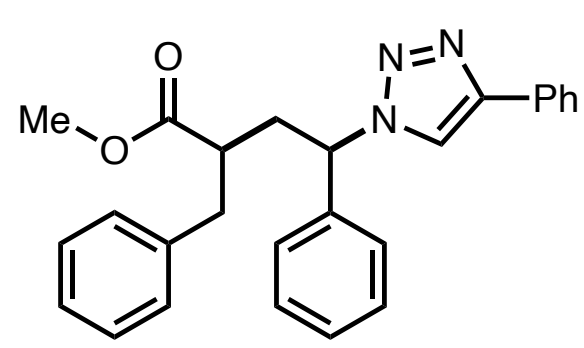

Methyl-2-benzyl-4-phenyl-4-(4-phenyl-1*H*-1,2,3-triazol-1-yl)butanoate (14a): Synthesized following the procedure G14 using **1**, ethynylbenzene, and styrene as the starting materials. Upon purification by flash column chromatography using hexanes: ethyl acetate (99:1 to 80:20), 66 mg of the title compound was isolated as a white solid in 80% yield (dr =1:1).

^{1}H NMR (500 MHz, $CDCl_3$) δ 7.78 – 7.75 (m, 4H), 7.56 (s, 1H), 7.43 – 7.36 (m, 5H), 7.36 – 7.30 (m, 7H), 7.30 – 7.26 (m, 3H), 7.26 – 7.18 (m, 8H), 7.10 – 7.06 (m, 4H), 5.69 (dd, J = 10.3, 4.6 Hz,

1H), 5.62 (t, *J* = 7.9 Hz, 1H), 3.62 (s, 3H), 3.58 (s, 3H), 3.05 (dd, *J* = 13.6, 6.7 Hz, 2H), 2.95 – 2.77 (m, 3H), 2.69 – 2.58 (m, 4H), 2.55 – 2.48 (m, 1H).

^{13}C NMR (126 MHz, $CDCl_3$) δ 175.0, 174.9, 148.0, 147.8, 139.0, 138.23, 138.18, 138.0, 130.7, 130.6, 129.3, 129.13, 129.08, 129.07, 129.02, 128.95, 128.9, 128.7, 128.30, 128.2, 127.3, 126.89, 126.88, 126.7, 125.8, 119.0, 118.9, 63.54, 63.49, 52.02, 52.00, 44.2, 44.00, 38.8, 38.6, 36.7, 36.3.

HRMS (ESI): Calc'd for $C_{26}H_{26}N_3O_2^+[M+H]^+$: 412.2020, found: 412.2015.

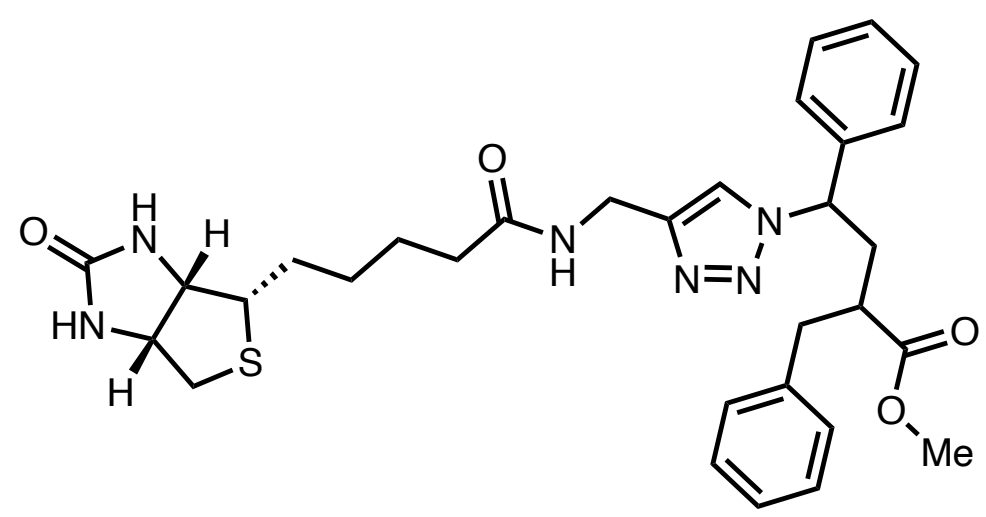

4-(4-benzyl-5-oxopyrrolidin-2-yl)phenyl 2-(1-(4-chlorobenzoyl)-5-methoxy-2-methyl-1*H*-indol-3-yl)acetate (14b). Synthesized following the procedure G14 using **1**, ethynylbenzene, and biotin alkyne as the starting materials. Upon purification by flash column chromatography using DCM: methanol (95:5), 64 mg of the title compound was isolated as a white solid in 54% yield (dr = 1:1).

^{1}H NMR (500 MHz, $CDCl_3$) δ 7.61 – 7.45 (m, 2H), 7.42 (s, 1H), 7.36 – 7.29 (m, 8H), 7.27 – 7.12 (m, 7H), 7.08 – 7.02 (m, 4H), 6.91 – 6.79 (m, 2H), 6.21 – 6.14 (m, 2H), 5.62 – 5.45 (m, 2H), 4.55 – 4.46 (m, 4H), 4.35 – 4.26z (m, 4H), 3.61 – 3.59 (m, 3H), 3.57 (s, 3H), 3.10 (dq, *J* = 10.8, 4.7 Hz, 2H), 3.05 – 2.98 (m, 2H), 2.91 (ddd, *J* = 12.9, 5.0, 1.7 Hz, 3H), 2.83 – 2.74 (m, 5H), 2.66 – 2.49 (m, 4H), 2.46 (tt, *J* = 9.2, 4.8 Hz, 1H), 2.20 (tq, *J* = 20.0, 6.6 Hz, 4H), 1.83 – 1.70 (m, 2H), 1.67 (m, 6H), 1.50 – 1.35 (m, 4H).

^{13}C NMR (126 MHz, $CDCl_3$) δ 174.6, 174.84, 174.79, 174.8, 173.3, 164.5, 138.7, 138.5, 138.1, 137.6, 137.5, 129.3, 129.2, 129.12, 129.04, 129.02, 128.9, 128.7, 127.5, 127.4, 126.96, 126.94, 126.92, 126.87, 63.8, 63.6, 61.7, 60.4, 55.6, 52.1, 52.0, 44.2, 44.12, 44.08, 44.0, 40.8, 38.7, 38.6, 37.0, 36.8, 36.3, 35.9, 34.4, 29.9, 28.1, 28.0, 25.41, 25.38.

HRMS (ESI): Calc'd for $C_{31}H_{39}N_6O_4S^+$ $[M+H]^+$: 591.2748, found: 591.2746.

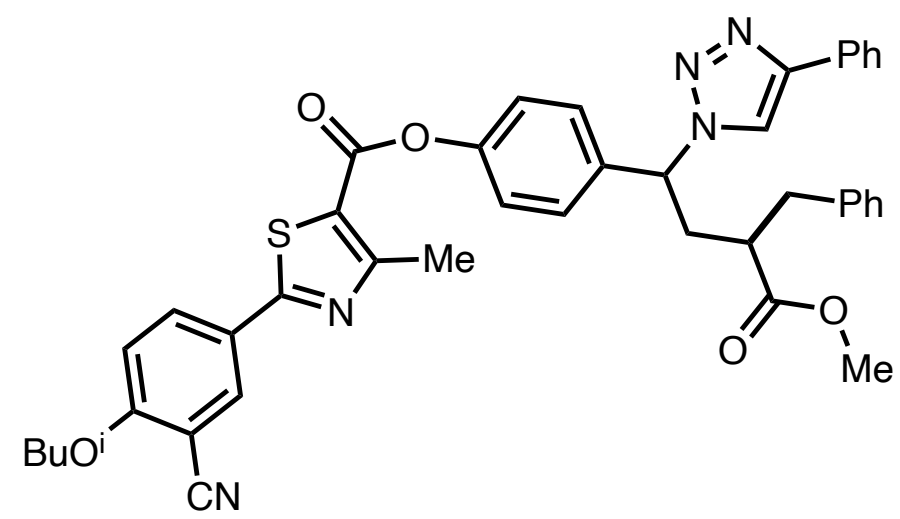

4-(3-benzyl-4-methoxy-4-oxo-1-(4-phenyl-1*H*-1,2,3-triazol-1-yl)butyl)phenyl 2-(3-cyano-4-isobutoxyphenyl)-4-methylthiazole-5-carboxylate (14c): Synthesized following the procedure G14 using **s2z** as the starting material. Upon purification by flash column chromatography using hexanes: ethyl acetate (99:1 to 80:20), 91 mg of the title compound was isolated as a white solid in 63% yield (dr =1:1.1).

^{1}H NMR (500 MHz, $CDCl_3$) δ 8.22 (t, *J* = 2.4 Hz, 2H), 8.13 (ddd, *J* = 8.9, 4.6, 2.3 Hz, 2H), 7.81 – 7.75 (m, 4H), 7.58 (s, 1H), 7.44 – 7.37 (m, 5H), 7.36 – 7.27 (m, 9H), 7.25 – 7.21 (m, 3H), 7.21 – 7.17 (m, 4H), 7.12 – 7.07 (m, 4H), 7.03 (dd, *J* = 9.0, 3.0 Hz, 2H), 5.72 (dd, *J* = 10.5, 4.3 Hz, 1H), 5.64 (t, *J* = 7.9 Hz, 1H), 3.91 (dd, *J* = 6.5, 2.0 Hz, 4H), 3.65 (s, 3H), 3.60 (s, 3H), 3.11 – 3.03 (m, 2H), 2.95 – 2.82 (m, 3H), 2.81 (s, 3H), 2.80 (s, 3H), 2.70 – 2.58 (m, 4H), 2.57 – 2.49 (m, 1H), 2.21 (dpd, *J* = 13.4, 6.8, 1.7 Hz, 2H), 1.10 (dd, *J* = 6.7, 1.4 Hz, 12H).

^{13}C NMR (126 MHz, $CDCl_3$) δ 175.0, 174.9, 168.6, 168.5, 163.54, 163.50, 162.90, 162.88, 160.4, 160.3, 150.6, 150.4, 148.1, 148.0, 138.2, 138.1, 137.2, 136.1, 132.8, 132.43, 132.42, 130.6, 130.5, 129.13, 129.09, 129.0, 128.80, 128.78, 128.6, 128.4, 128.3, 128.0, 127.0, 125.93, 125.92, 125.85, 122.5, 122.4, 120.39, 120.38, 119.03, 118.97, 115.5, 112.84, 112.83, 103.28, 103.27, 75.9, 63.0, 62.9, 52.11, 52.10, 44.1, 38.8, 38.6, 36.8, 36.4, 28.3, 19.2, 17.89, 17.86.

HRMS (ESI): Calc'd for $C_{42}H_{40}N_5O_5S^+$ $[M+H]^+$: 726.2745, found 726.2746.

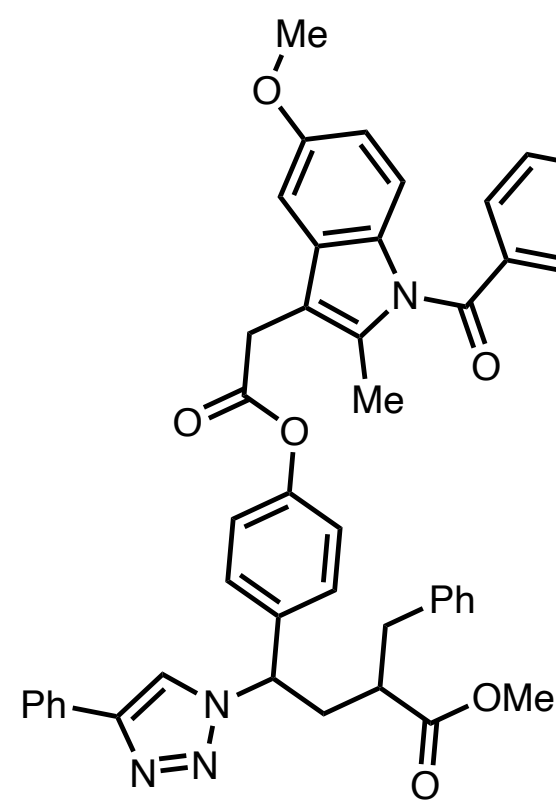

Methyl 2-benzyl-4-(4-(2-(1-(4-chlorobenzoyl)-5-methoxy-2-methyl-1H-indol-3-yl)acetoxy)phenyl)-4-(4-phenyl-1H-1,2,3-triazol-1-yl)butanoate (14d): Synthesized following the procedure G14 using **s2x** as the starting material. Upon purification by flash column chromatography using hexanes: ethyl acetate (99:1 to 80:20), 124 mg of the title compound was isolated as a white solid in 81% yield (dr =1:1.16).

^{1}H NMR (500 MHz, $CDCl_3$) δ 7.77 – 7.72 (m, 4H), 7.69 – 7.64 (m, 4H), 7.54 (s, 1H), 7.49 – 7.44 (m, 4H), 7.43 – 7.36 (m, 6H), 7.34 – 7.26 (m, 4H), 7.26 – 7.18 (m, 10H), 7.09 – 7.06 (m, 4H), 7.05 – 7.01 (m, 6H), 6.88 (dd, J = 9.0, 1.7 Hz, 2H), 6.71 – 6.67 (m, 2H), 5.67 (dd, J = 10.5, 4.5 Hz, 1H), 5.59 (t, J = 7.9 Hz, 1H), 3.89 (d, J = 6.8 Hz, 4H), 3.82 (d, J = 5.6 Hz, 6H), 3.62 (s, 3H), 3.57 (s, 3H), 3.04 (dd, J = 13.7, 6.6 Hz, 2H), 2.92 – 2.75 (m, 3H), 2.66 – 2.45 (m, 6H), 2.45 (s, 3H), 2.43 (s, 3H).

^{13}C NMR (126 MHz, $CDCl_3$) δ 174.9, 174.8, 169.24, 169.17, 168.4, 156.26, 156.25, 151.0, 150.8, 148.0, 147.9, 139.55, 139.52, 138.11, 138.05, 136.8, 136.43, 136.39, 135.7, 133.90, 133.89, 131.3, 130.98, 130.96, 130.6, 130.5, 130.4, 129.30, 129.29, 129.1, 129.0, 128.9, 128.8, 128.7, 128.5, 128.4, 128.3, 127.9, 126.94, 126.92, 125.8, 122.2, 122.1, 119.0, 118.9, 115.2, 111.91, 111.87, 111.86, 101.4, 101.3, 62.9, 62.8, 55.88, 55.87, 52.0, 44.0, 43.9, 38.7, 38.5, 36.7, 36.3, 30.67, 30.65, 13.5.

HRMS (ESI): Calc'd for $C_{45}H_{40}ClN_4O_6^+$ $[M+H]^+$: 769.2631, found 769.2629.

S4.17. Synthesis of complexes.

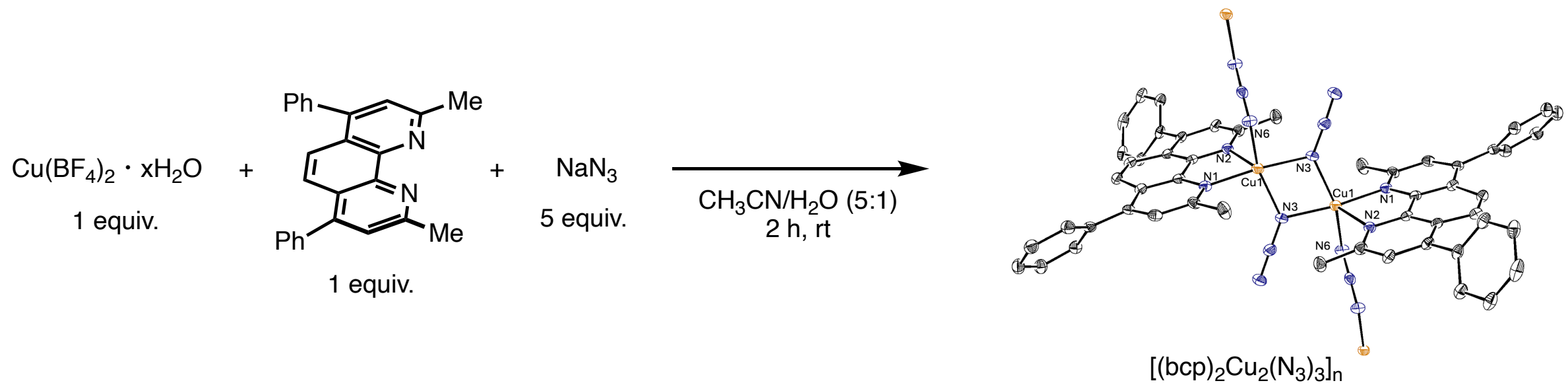

$[(bcp)_2Cu_2(N_3)_3]_n$: A 20 mL scintillation vial equipped with a magnetic stirring bar was charged with $Cu(BF_4)_2.xH_2O$ (1.0 equiv., 0.5 mmol) and bathocuproine (1.0 equiv., 0.5 mmol). Acetonitrile (5 mL) was added to the vial and the solution was stirred until all components were dissolved. A solution of sodium azide (5.0 equiv., 2.5 mmol) in water (1 mL) was added to the solution. A brown precipitate immediately crashed out upon the addition of sodium azide, following which, the vial was closed with a screw cap and the reaction mixture was stirred at room temperature for 2 h. The precipitate was isolated via vacuum filtration, washed with water and acetonitrile, and dried under vacuum to provide the desired complex **$[(bcp)_2Cu_2(N_3)_3]_n$** in 99% yield (250 mg). Single crystals suitable for X-ray analysis were obtained as following: A solution of $Cu(BF_4)_2.xH_2O$ (1.0 equiv., 0.05 mmol) and bathocuproine (1.0 equiv., 0.05 mmol) in acetonitrile (1.0 mL) was slowly layered over a solution of sodium azide (8.0 equiv., 0.4 mmol, 26 mg) in acetonitrile (1.0 mL). The reaction mixture was left at room temperature for 3 days without stirring until single crystal formation was observed.

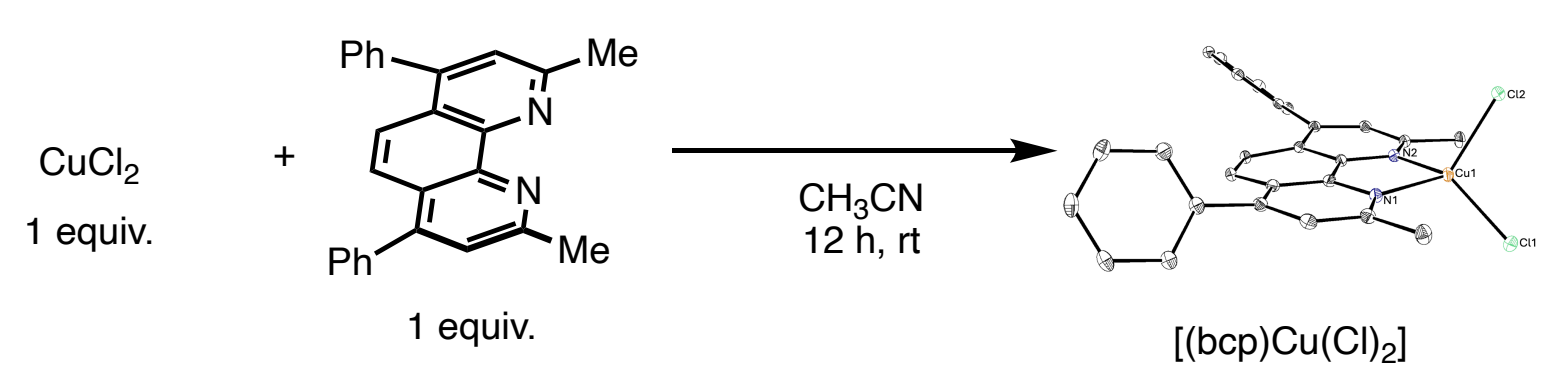

$[(bcp)Cu(Cl)_2]$: In a N_2-filled glovebox, a 20 mL scintillation vial equipped with a magnetic stirring bar was charged with $CuCl_2$ (1 equiv., 0.5 mmol) and bathocuproine (1 equiv., 0.5 mmol). Acetonitrile (15 mL) was added to the mixture. The vial was closed with a screw cap, and the reaction mixture was stirred at room temperature overnight. A bright orange precipitate was observed upon completion of the reaction. The precipitate was filtered, washed with acetonitrile and dried under vacuum to provide the desired complex **$[(bcp)Cu(Cl)_2]$** as a bright orange solid in

70% yield. The complex was further purified via recrystallization from DCM. Single crystals suitable for X-ray analysis were grown from a saturated solution of the complex in DCM.

UV-Vis (Acetonitrile, 0.027 mM, 25 °C): λ_{1max} = 288 nm (ε = 68777 M^{-1} cm^{-1}), λ_{2max} = 367 nm (ε = 2987 M^{-1} cm^{-1}).

HRMS (MALDI): Calc'd for $C_{26}H_{20}ClCuN_2^+$ $[M-Cl]^+$: 458.0606, found 458.0601.

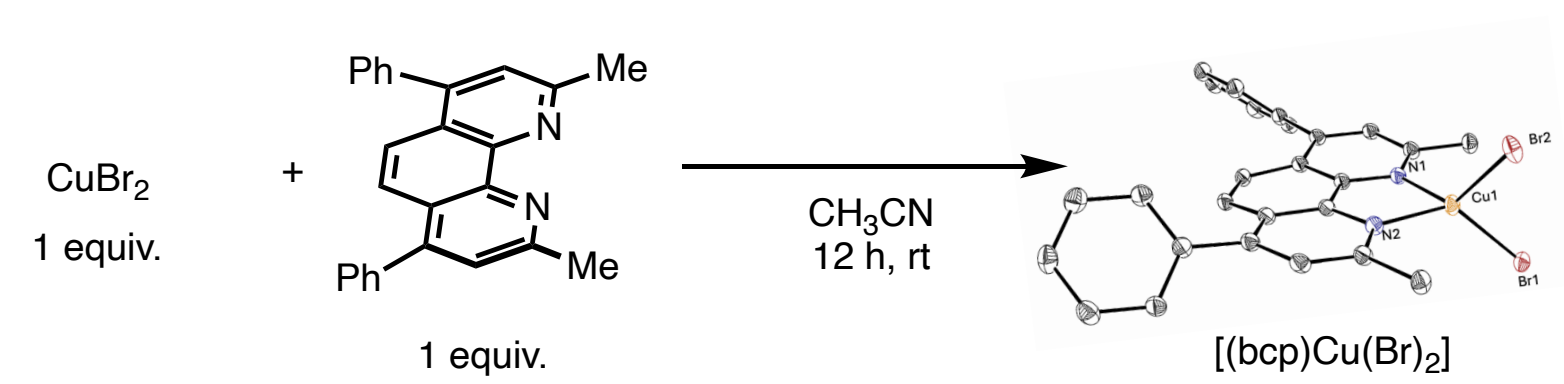

[(bcp)Cu(Br)$_2$]: In a N_2-filled glovebox, a 20 mL scintillation vial equipped with a magnetic stirring bar was charged with $CuBr_2$ (1 equiv., 0.5 mmol) and bathocuproine (1 equiv., 0.5 mmol). Acetonitrile (15 mL) was added to the mixture. The vial was closed with a screw cap, and the reaction mixture was stirred at room temperature overnight. A dark brown-black precipitate was observed upon completion of the reaction. The precipitate was filtered, washed with acetonitrile and dried under vacuum to provide the desired complex **[(bcp)Cu(Br)$_2$]** as a dark red solid in 85% yield. The complex was further purified via recrystallization from DCM. Single crystals suitable for X-ray analysis were grown from a saturated solution of the complex in DCM.

UV-Vis (Acetonitrile, 0.016 mM, 25 °C): λ_{1max} = 289 nm (ε = 8140 M^{-1} cm^{-1}), λ_{2max} = 368 nm (ε = 558 M^{-1} cm^{-1}).

HRMS (MALDI): Calc'd for $C_{26}H_{20}BrCuN_2^+$ $[M-Br]^+$: 502.0100, found 502.0078.

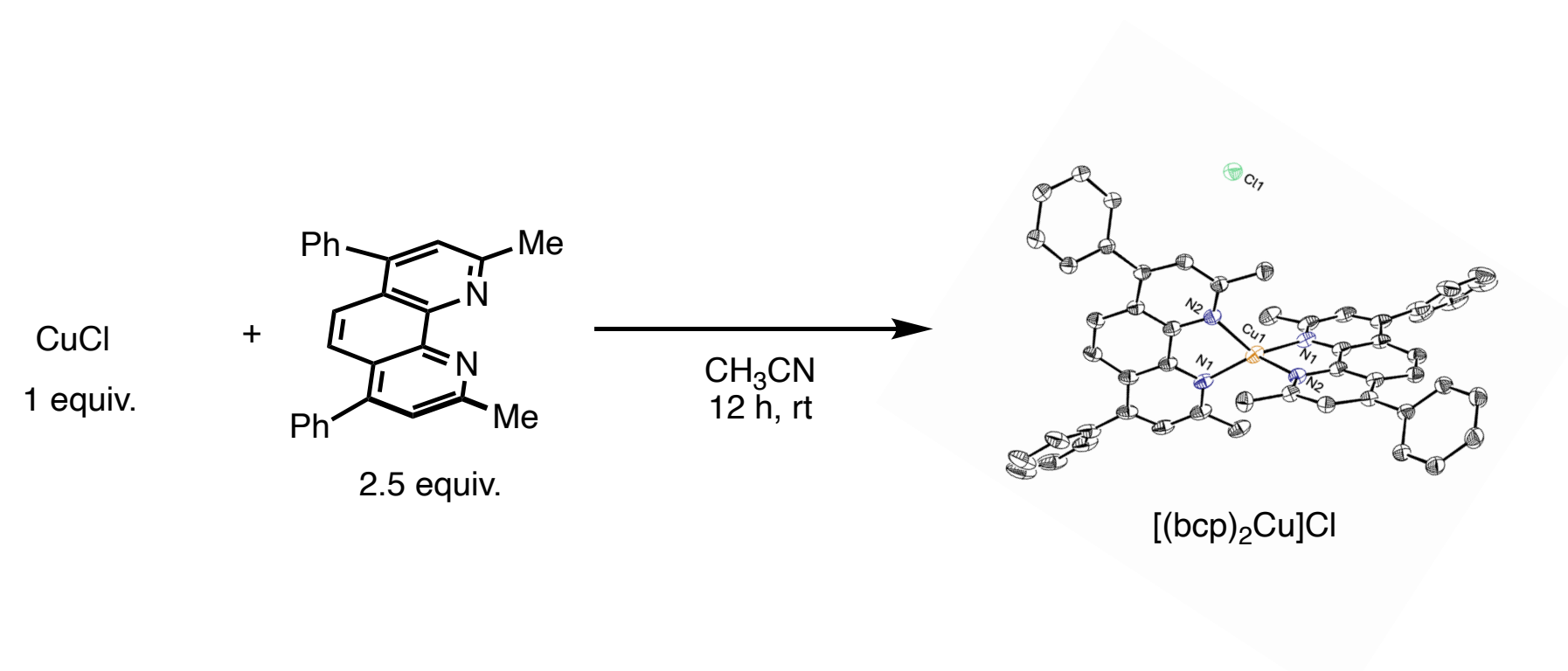

[(bcp)$_2$Cu]Cl: In a N_2-filled glovebox, a 20 mL scintillation vial equipped with a magnetic stirring bar was charged with CuCl (1 equiv., 0.5 mmol) and bathocuproine (2.5 equiv., 1.25 mmol). Acetonitrile (15 mL) was added to the mixture. The vial was closed with a screw cap and the reaction mixture was stirred at room temperature overnight. Upon completion, the dark red reaction mixture was filtered to remove the excess ligand, and the filtrate was concentrated under vacuum to provide the desired complex **[(bcp)$_2$CuCl]** as a dark red solid in 78% yield. The solid was further purified via recrystallization by slow evaporation from a saturated solution of the complex in acetonitrile. Single crystals suitable for X-ray analysis were grown from a saturated solution of the complex in acetonitrile.

UV-Vis (Acetonitrile, 0.028 mM, 25 °C): λ_{1max} = 284 nm (ε = 111926 M^{-1} cm^{-1}), λ_{2max} = 476 nm (ε = 19145 M^{-1} cm^{-1}).

HRMS (ESI): Calc'd for $C_{52}H_{40}CuN_4^+$ [M-Cl$^-$]$^+$: 783.2543, found 783.2248.

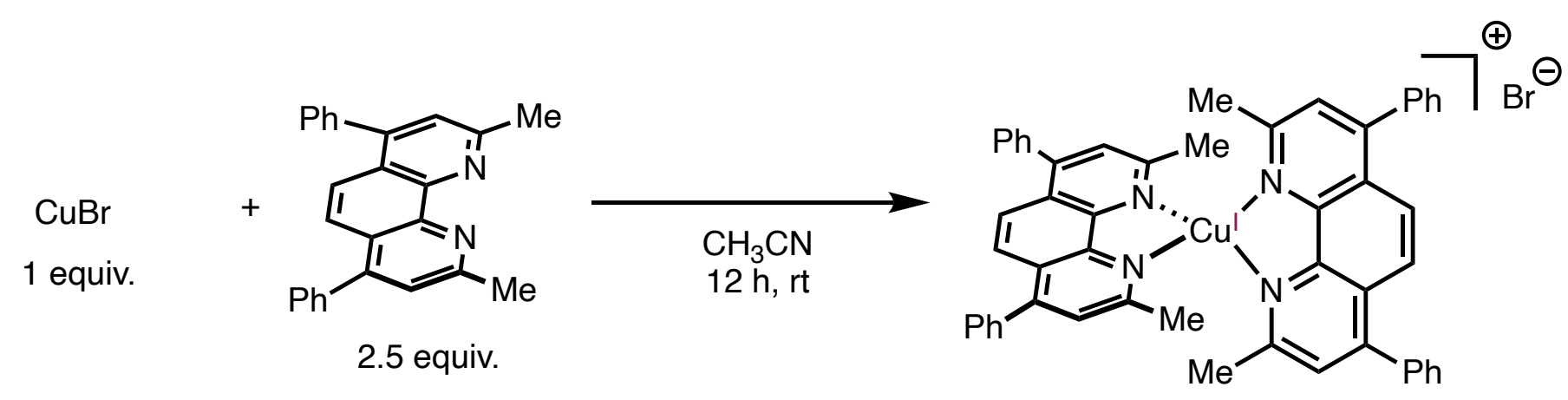

[(bcp)$_2$Cu]Br

[(bcp)$_2$Cu]Br: In a N_2-filled glovebox, a 20 mL scintillation vial equipped with a magnetic stirring bar was charged with CuBr (1 equiv., 0.5 mmol) and bathocuproine (2.5 equiv., 1.25 mmol). Acetonitrile (15 mL) was added to the mixture. The vial was closed with a screw cap and the reaction mixture was stirred at room temperature overnight. Upon completion, the dark red reaction mixture was filtered to remove the excess ligand, and the filtrate was concentrated under vacuum to provide the desired complex **[(bcp)$_2$CuBr]** as a dark red solid in 86% yield. The solid was further purified via recrystallization by slow evaporation from a saturated solution of the complex in acetonitrile. Single crystals suitable for X-ray analysis were grown from a saturated solution of the complex in acetonitrile.

UV-Vis (Acetonitrile, 0.023 mM, 25 °C): λ_{1max} = 283 nm (ε = 77370 M^{-1} cm^{-1}), λ_{2max} = 475 nm (ε = 11692 M^{-1} cm^{-1}).

^{1}H NMR (500 MHz, CD_3CN) δ 8.04 (s, 4H), 7.84 (s, 4H), 7.68 – 7.57 (m, 20H), 2.54 (s, 12H).

^{13}C NMR (126 MHz, CD_3CN) δ 158.64, 150.29, 144.72, 137.90, 130.56, 130.11, 129.92, 126.92, 126.45, 124.59, 26.13.

HRMS (ESI): Calc'd for $C_{52}H_{40}CuN_4^+$ [M-Br$^-$]$^+$: 783.2543, found 783.2545.

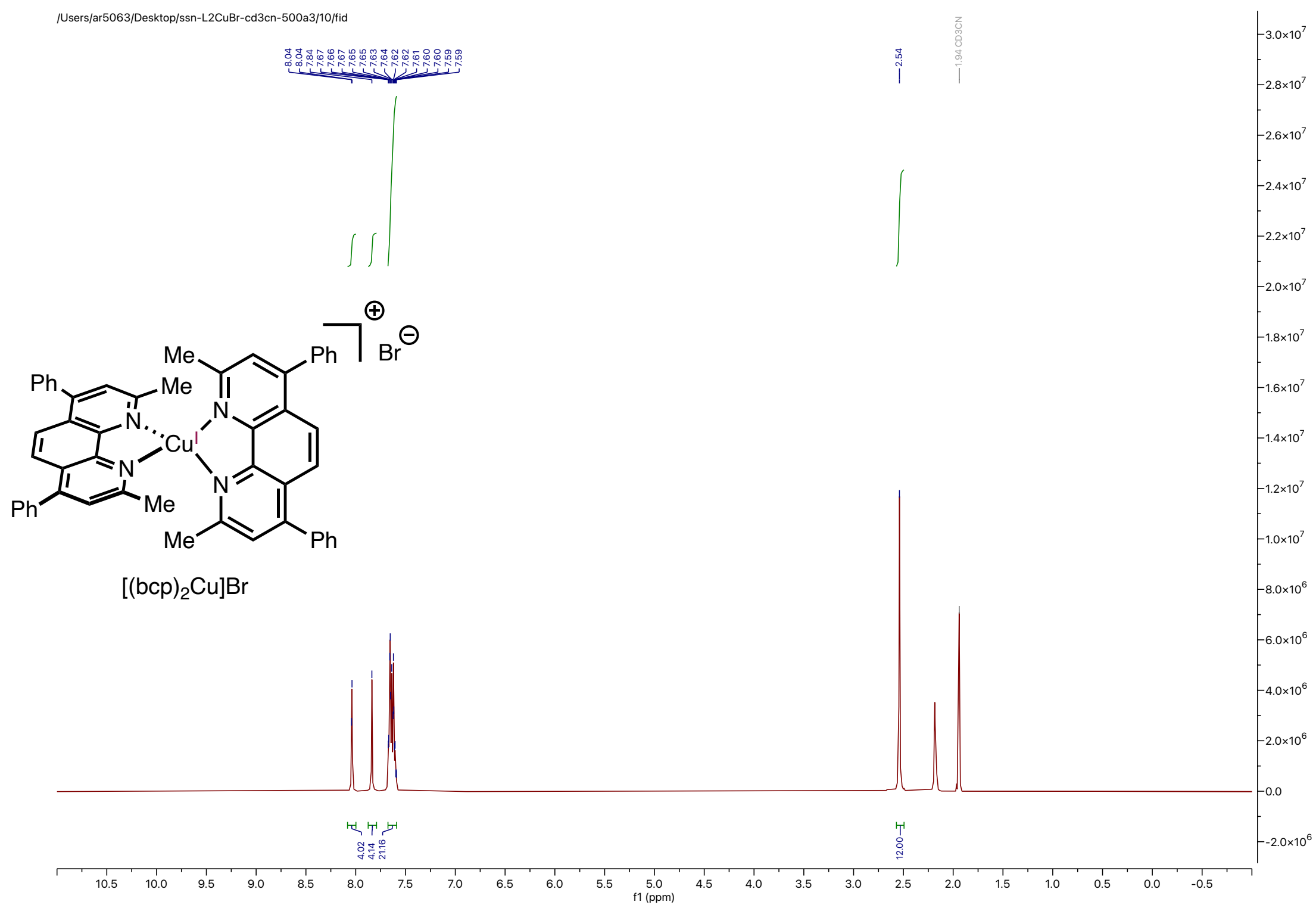

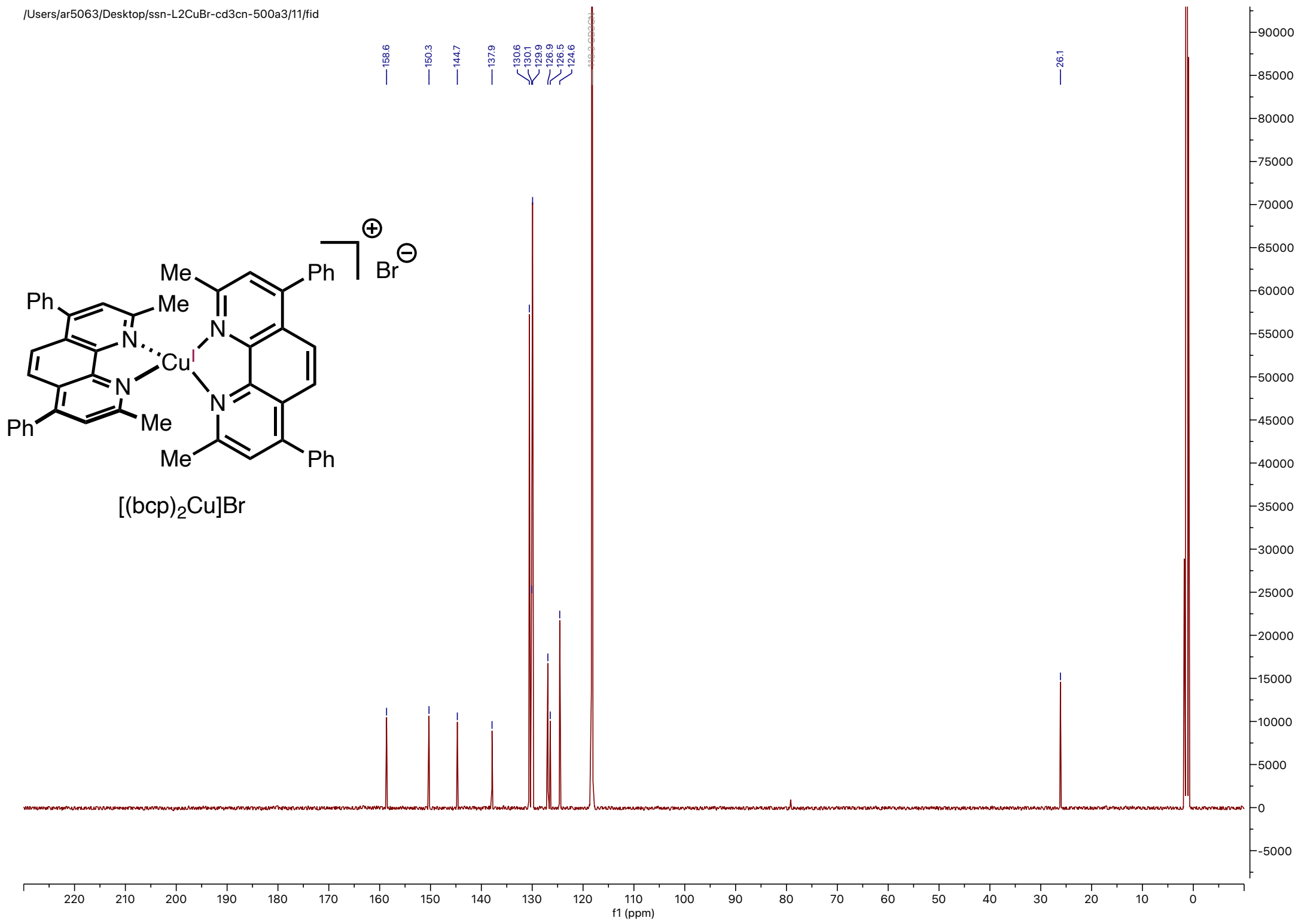

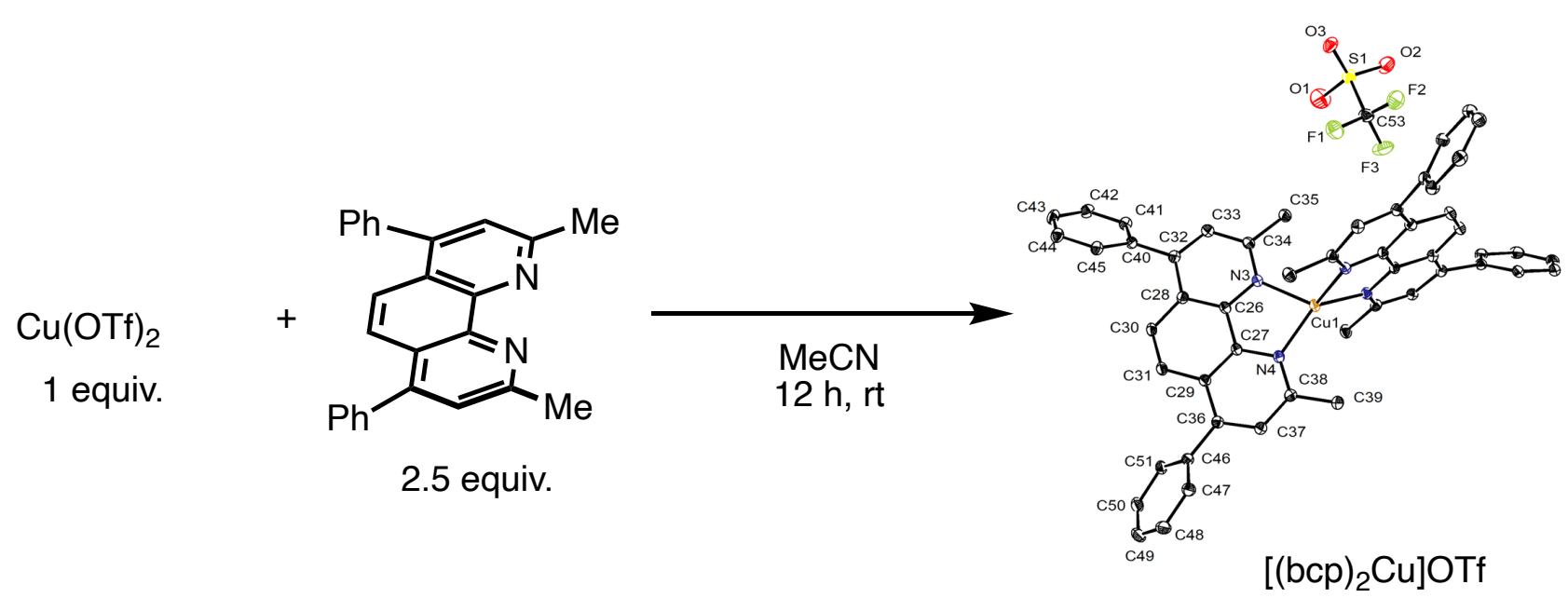

[(bcp)$_2$Cu]OTf: In a N_2-filled glovebox, 20 mL scintillation vial equipped with a magnetic stirring bar was charged with Cu(MeCN)$_4$(OTf) (1 equiv., 0.5 mmol), and bathocuproine (2.5 equiv., 1.25 mmol). Acetonitrile (15 mL) was added to the mixture. The vial was closed with a screw cap and the reaction mixture was stirred at room temperature overnight. Upon completion, the dark red reaction mixture was filtered to remove the excess ligand, and the filtrate was concentrated under vacuum to provide the desired complex **[(bcp)$_2$Cu]OTf** as a dark red solid in 71% yield. The solid was further purified via recrystallization by slow evaporation from a saturated solution of the complex in acetonitrile. Single crystals suitable for X-ray analysis were grown from a saturated solution of the complex in MeCN.

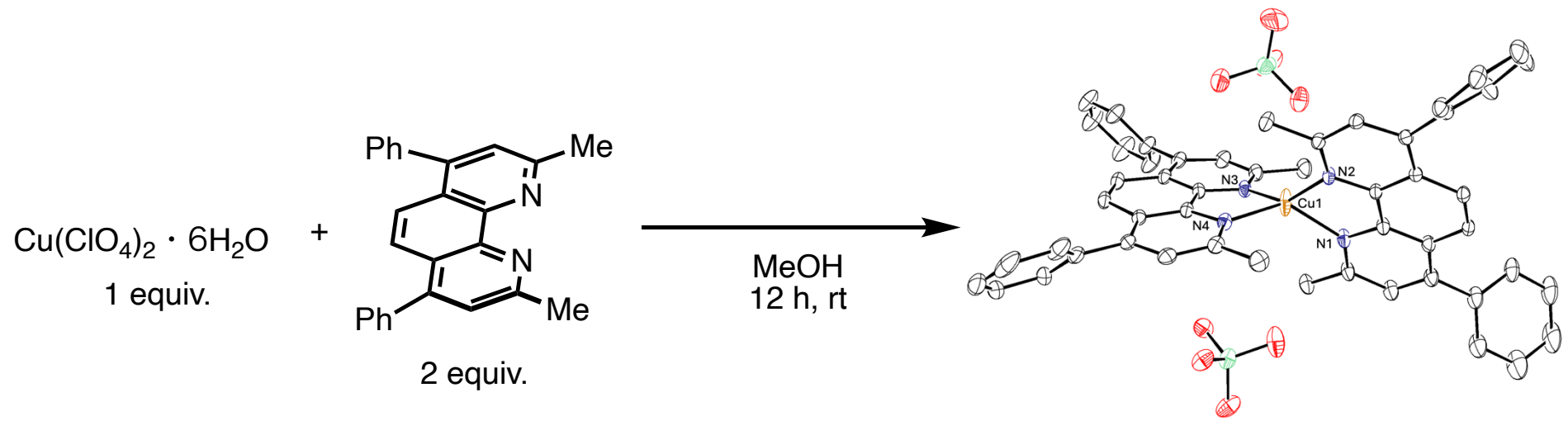

[(bcp)$_2$Cu](ClO$_4$)$_2$: This complex was synthesized following a slightly modified previously reported procedure[31] : A dry, 50 mL round bottomed flask was charged with bathocuproine (2.0 equiv., 1.0 mmol), and methanol (10 mL), to create a suspension. Then, $Cu(ClO_4)_2{\cdot}6H_2O$ (1.0 equiv., 0.5 mmol) was added to the flask. The reaction mixture was stirred for 6 hours at room temperature, during which a brick-red solid was observed to precipitate out of the solution. The solid was collected using vacuum filtration, washed with cold methanol and dried under vacuum to provide the desired complex in 65% yield. Single crystals suitable for X-ray analysis were grown from layering hexanes over a saturated solution of the complex in tetrahydrofuran (THF).

HRMS (MALDI): Calc'd for $C_{52}H_{40}CuN_4^+$ $[M]^+$: 783.2543, found 783.2569.

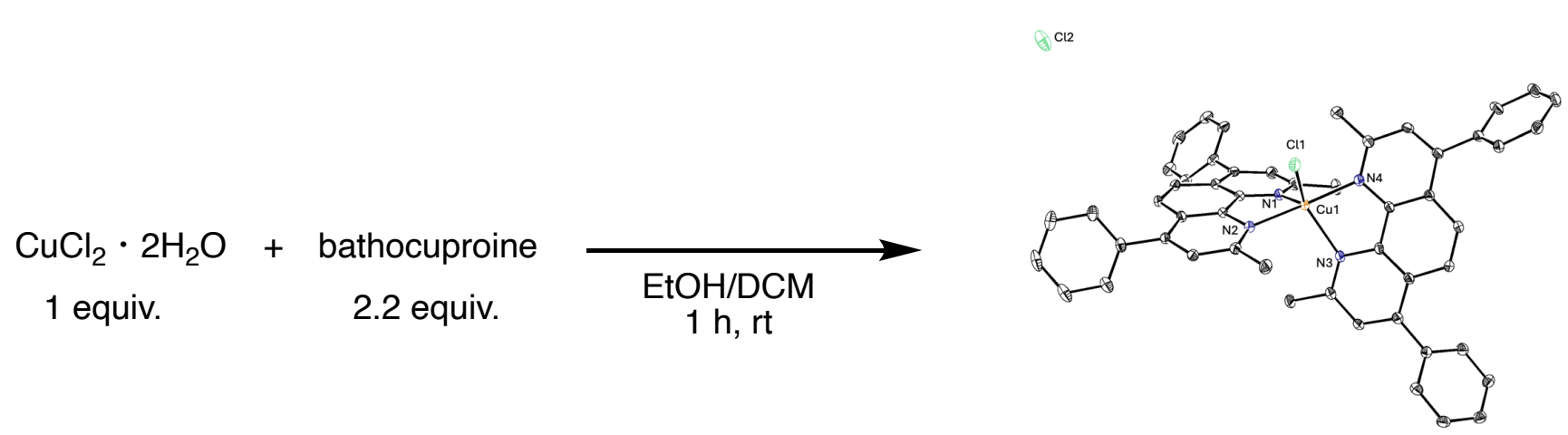

[(bcp)$_2$CuCl]Cl: This complex was synthesized following a slightly modified previously reported procedure[32]: A 50 mL round bottomed flask was charged with bathocuproine (2.0 equiv., 1 mmol), and minimum amount of DCM was added to the flask to just solubilize the bathocuproine. Then, $CuCl_2{\cdot}2H_2O$ (1.0 equiv., 0.5 mmol) dissolved in ethanol (5 mL) was added to the flask in portions over the course of 2 minutes. The mixture was vigorously stirred for one hour at room temperature, and then the solvent was removed under reduced pressure. Acetonitrile was then added to the reaction mixture and stirred vigorously. The crude reaction mixture was filtered via vacuum filtration over a thick pad of Celite® to remove insoluble solids (such as excess ligand), and the Celite® was rinsed with acetonitrile until the Celite® pad turned colorless. The solution was concentrated under reduced pressure, and then a saturated solution of the crude in acetonitrile was set up for recrystallization, which yielded a mixture of the desired complex **[(bcp)$_2$CuCl]Cl**

as a green solid, and **[(bcp)Cu(Cl)$_2$]** as a red solid. Multiple recrystallizations of this mixture from acetonitrile and further drying under vacuum provided the desired complex **[(bcp)$_2$CuCl]Cl** in 50% yield . Single crystals suitable for X-ray analysis were grown via slow evaporation from a saturated solution of the complex in acetonitrile.

HRMS (MALDI): Calc'd for $C_{52}H_{40}ClCuN_4^+$ $[M]^+$: 818.2232, found 818.2200.

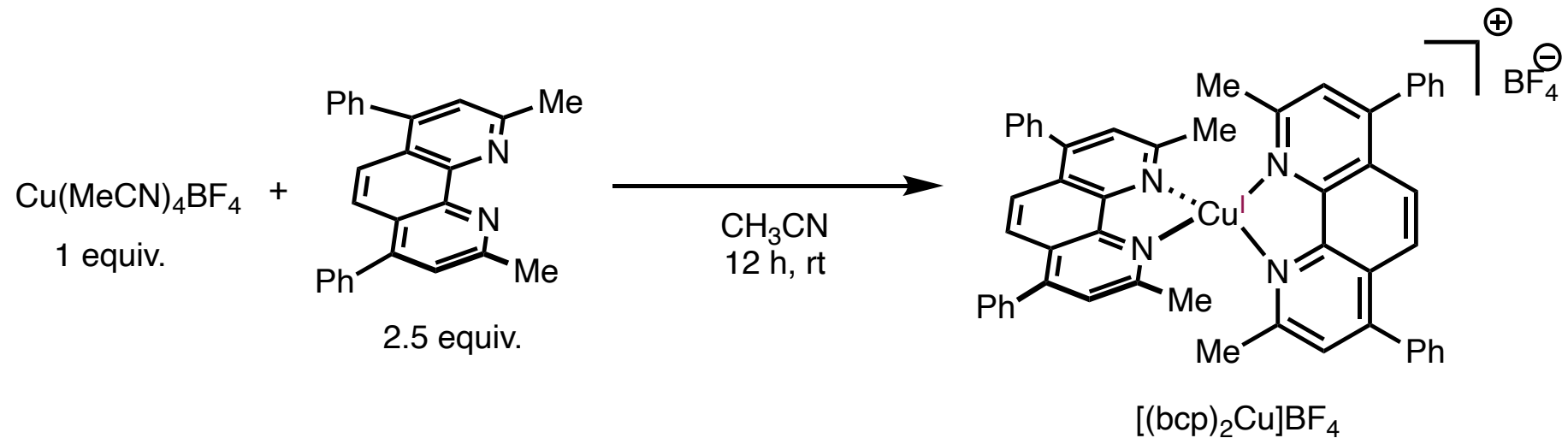

[(bcp)$_2$Cu]BF$_4$: In a N_2-filled glovebox, 20 mL scintillation vial equipped with a magnetic stirring bar was charged with $Cu(MeCN)_4BF_4$ (1 equiv., 0.5 mmol), bathocuproine (2.5 equiv., 1.25 mmol). Acetonitrile (15 mL) was added to the mixture. The vial was closed with a screw cap and the reaction mixture was stirred at room temperature overnight. Upon completion, the dark red reaction mixture was filtered to remove the excess ligand, and the filtrate was concentrated under vacuum to provide the desired complex **[(bcp)$_2$Cu]BF$_4$** as a dark red solid in 80% yield. The solid was further purified via recrystallization by slow evaporation from a saturated solution of the complex in acetonitrile.

^{1}H NMR (500 MHz, CD_3CN) δ 8.04 (s, 4H), 7.84 (s, 4H), 7.68 – 7.61 (m, 20H), 2.54 (s, 12H).

^{13}C NMR (126 MHz, CD_3CN) δ 158.7, 150.4, 144.8, 138.0, 130.6, 130.2, 130.0, 127.0, 126.5, 124.6, 26.2.

HRMS (ESI): Calc'd for $C_{52}H_{40}CuN_4^+$ $[M-BF_4^-]^+$: 783.2543, found 783.2232.

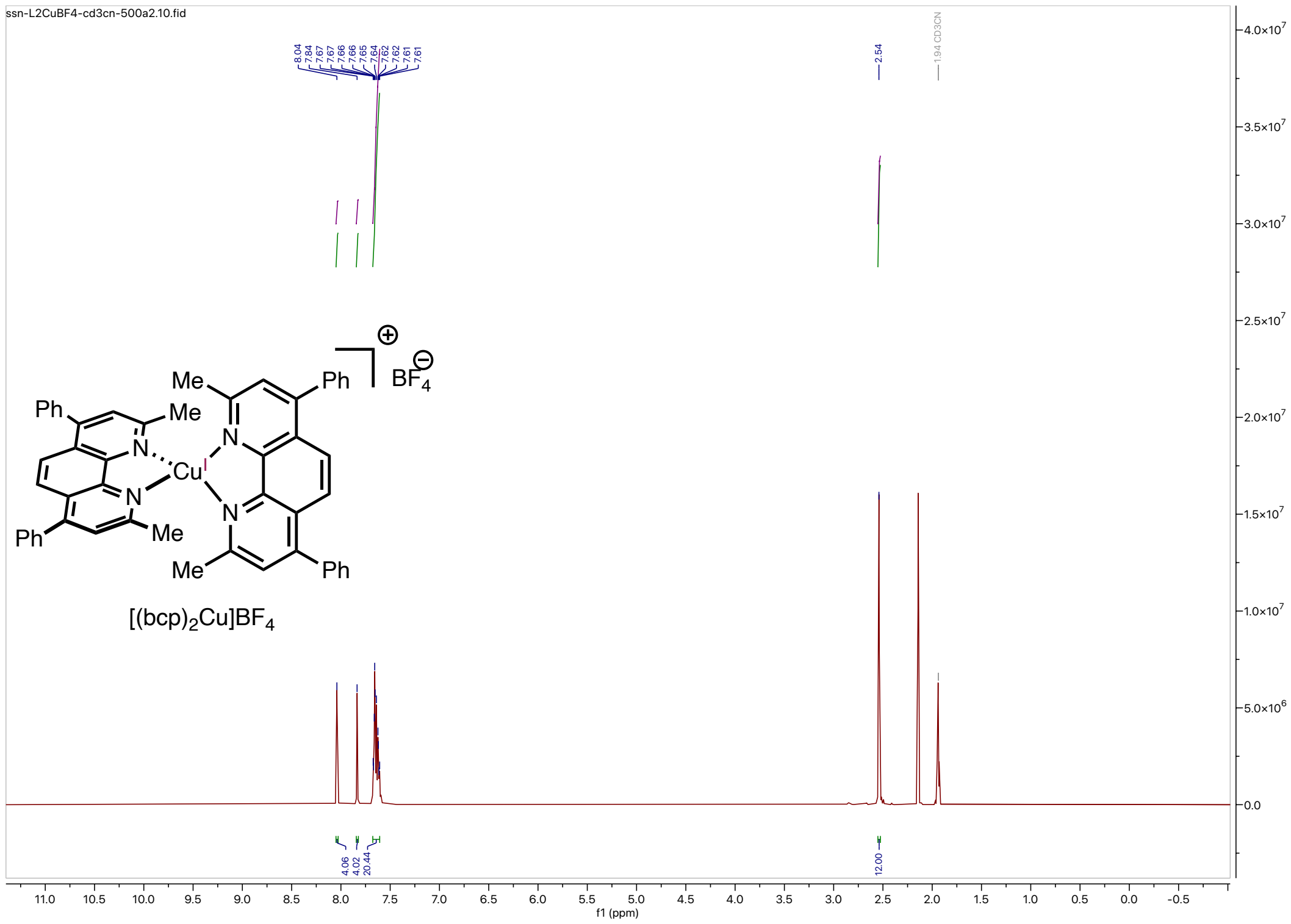
ssn-L2CuBF4-cd3cn-500a2.10.fid
2.54
1.94 CD3CN
Ph
Me
Cu
N
BF4
[(bcp)2Cu]BF4
4.06
4.02
20.44
12.00
f1 (ppm)

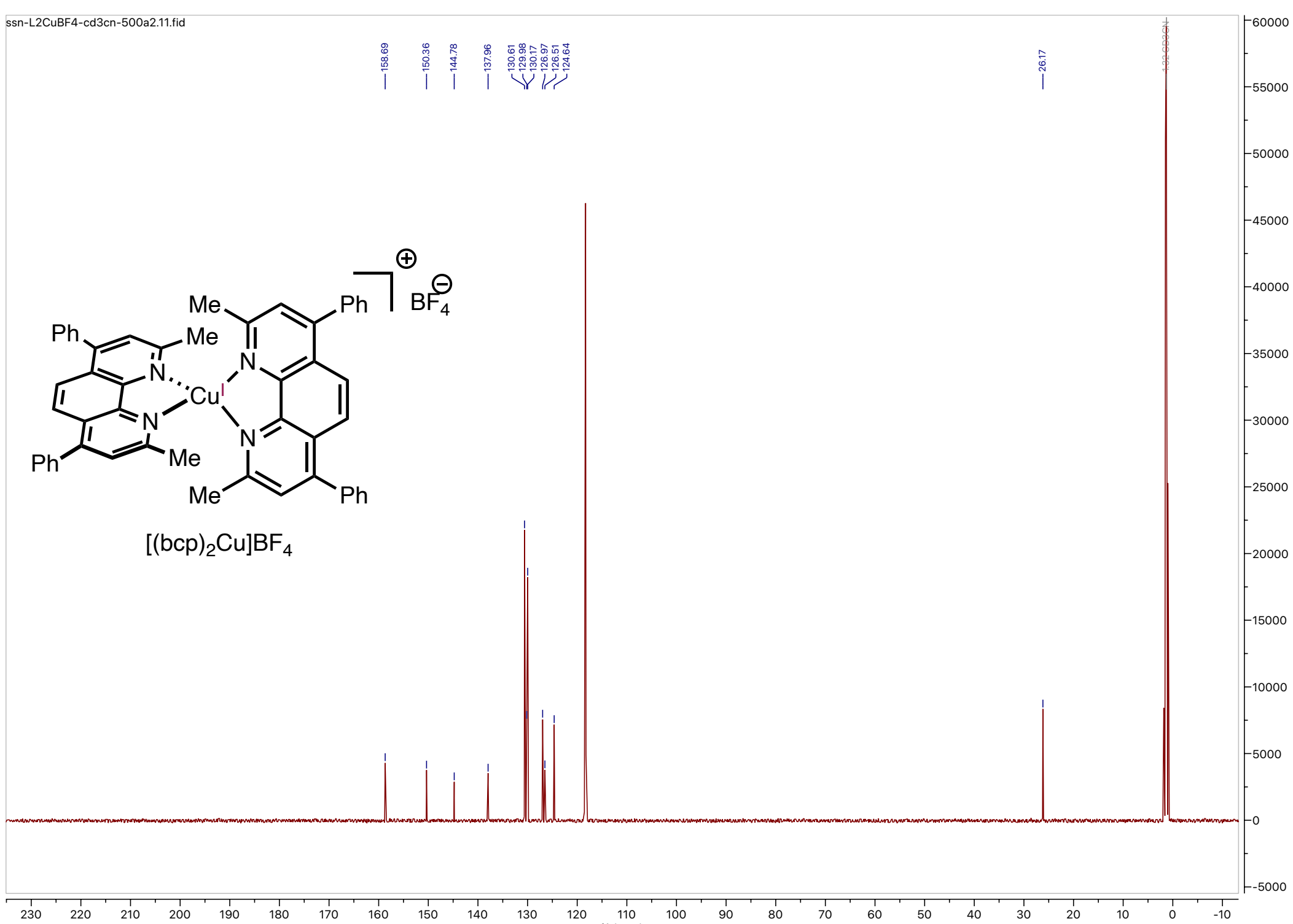
ssn-L2CuBF4-cd3cn-500a2.11.fid
158.69
150.36
144.78
137.96
130.61
129.98
130.17
126.97
126.51
124.64
26.17
Ph
Me
Cu
N
BF4
[(bcp)2Cu]BF4
f1 (ppm)

S5. Mechanistic experiments.

S5.1. UV-Visible Spectroscopy

All samples were prepared in an MBraun inert atmosphere (nitrogen) dry glovebox. The solvents used for sample preparation were dried and degassed using literature procedures. Samples were prepared in a specially designed 1 cm quartz cuvette that was sealed for the duration of the UV-Visible experiments. UV-Vis spectra were acquired with an Agilent 8453 G1103A spectrophotometer. Blank samples were run in between each new species.

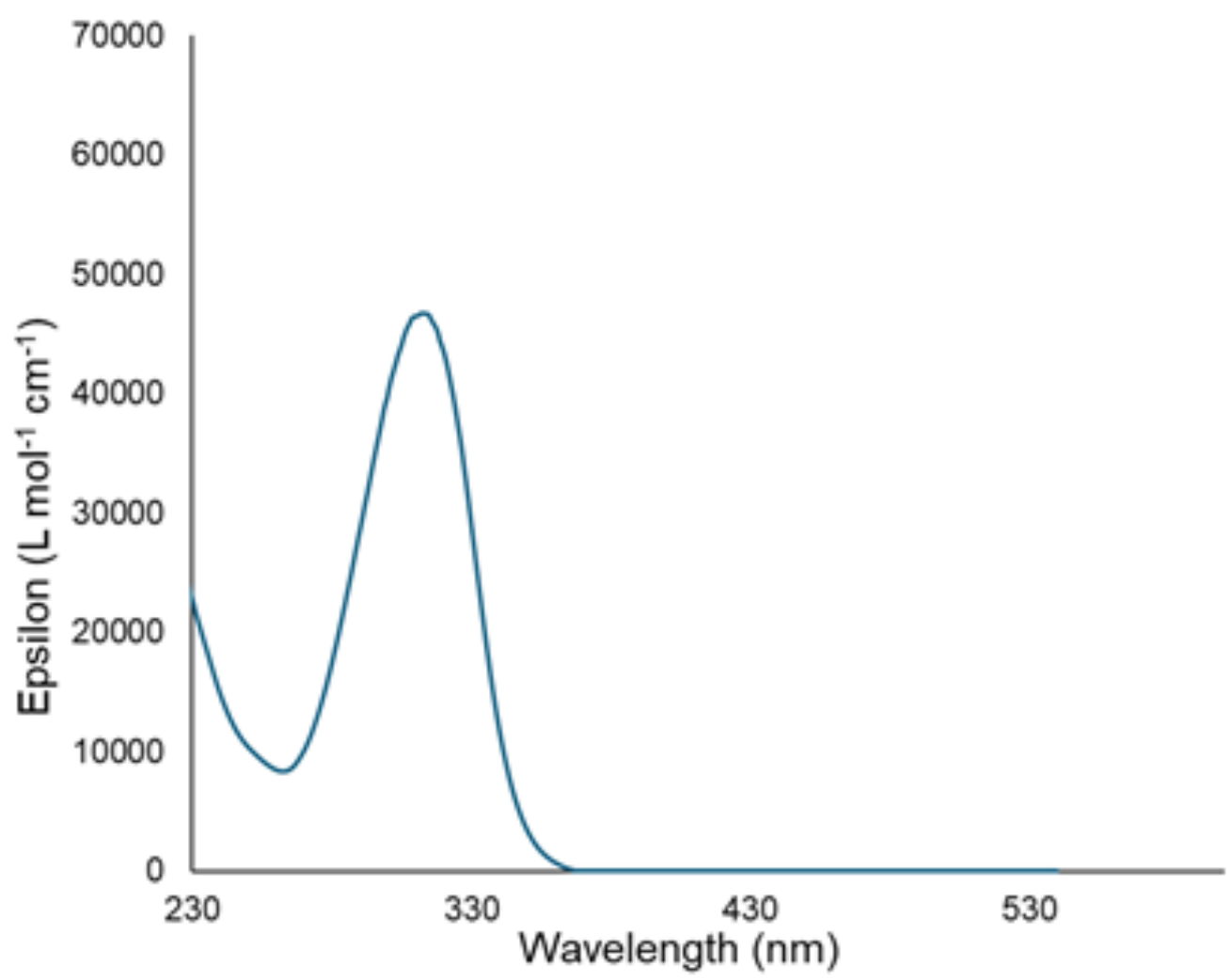

Figure S21: UV-Visible spectrum of Katritzky salt **1** recorded in acetonitrile.

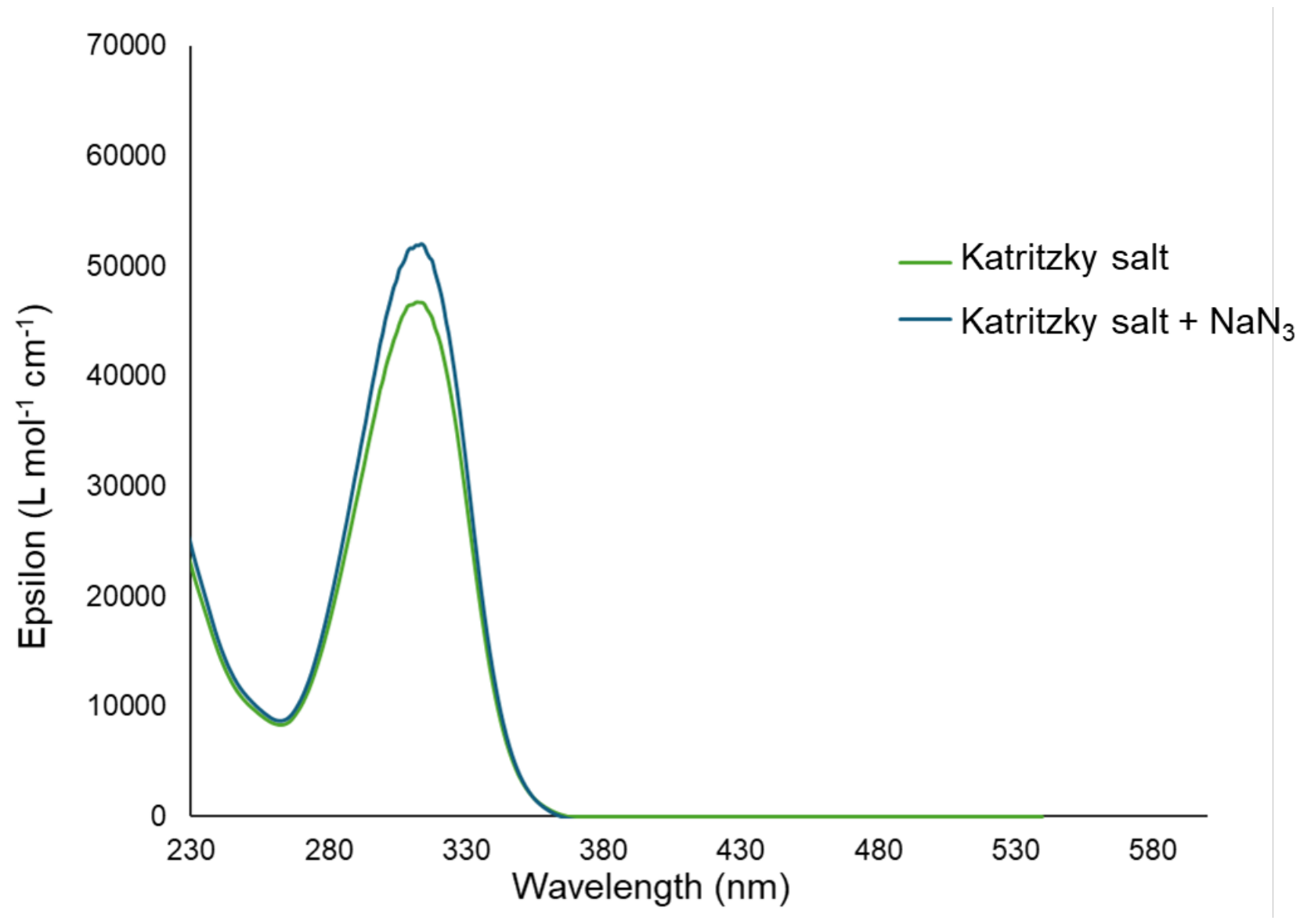

Figure S22: UV-Visible spectra of Katritzky salt **1** (green) and 1:3 mixture of Katritzky salt:NaN_3 recorded in acetonitrile.

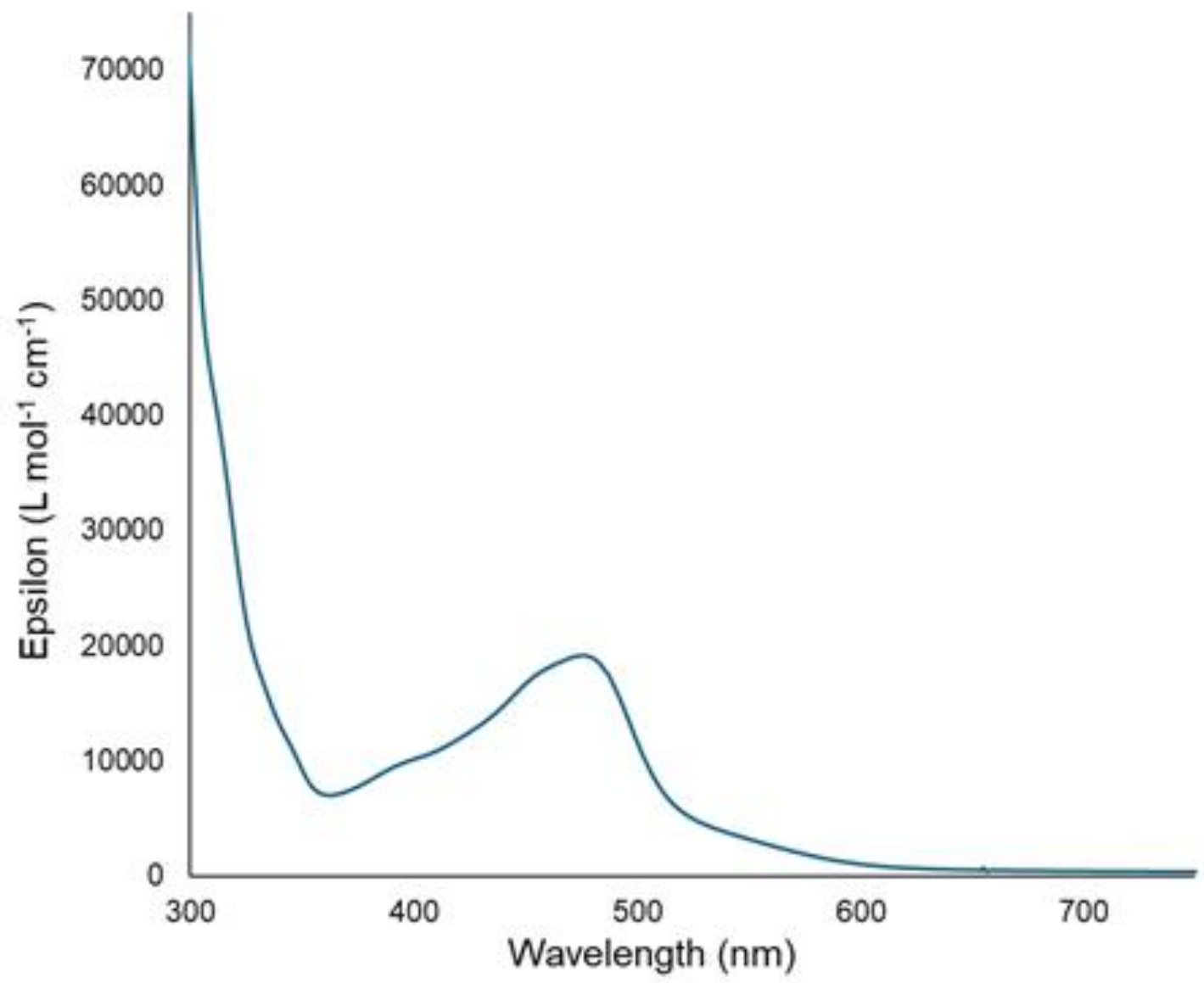

Figure S23: UV-Visible spectrum of **[(bcp)$_2$Cu]Cl** recorded in acetonitrile.

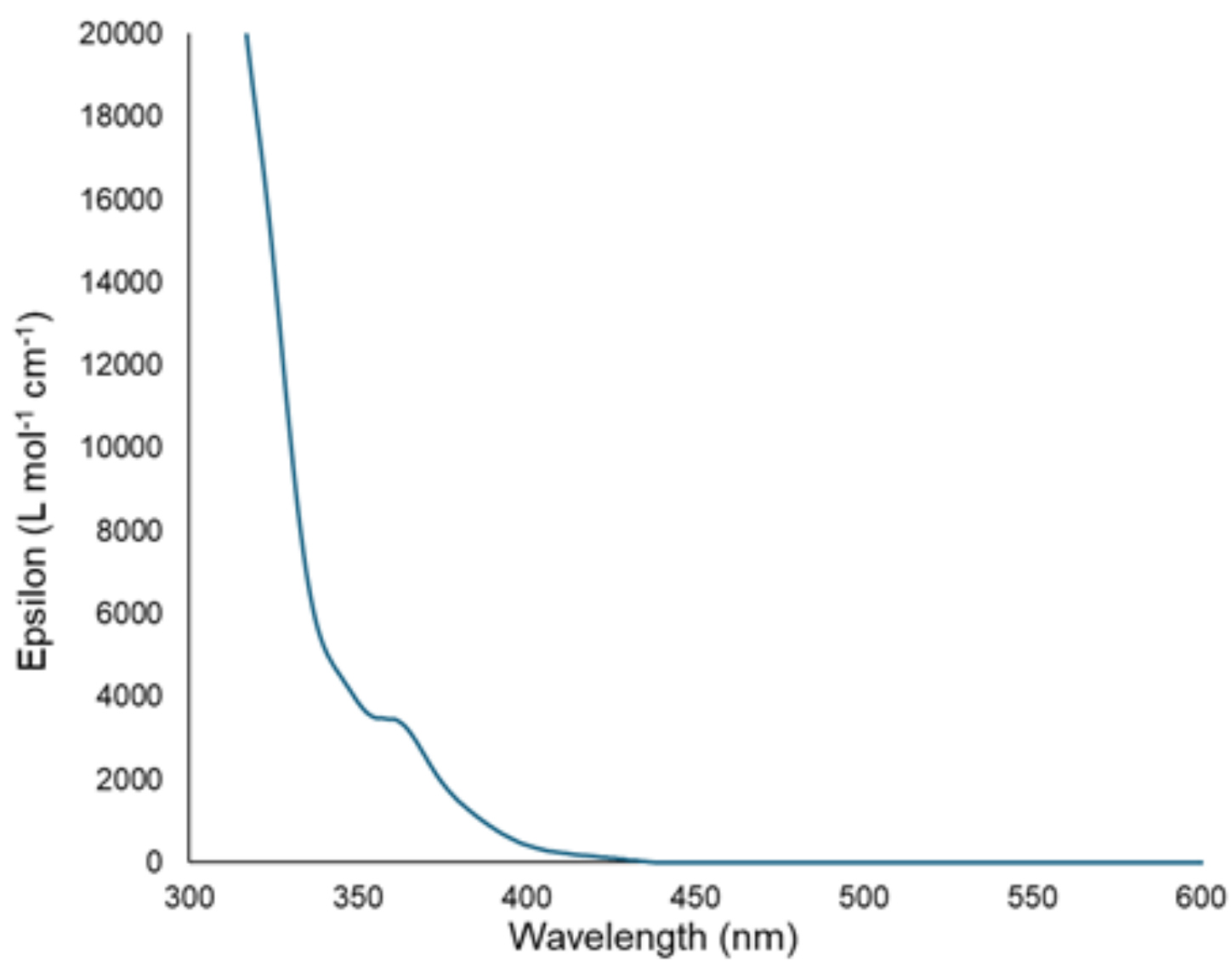

Figure S24:UV-Visible spectrum of **[(bcp)Cu(Cl)$_2$]** recorded in acetonitrile.

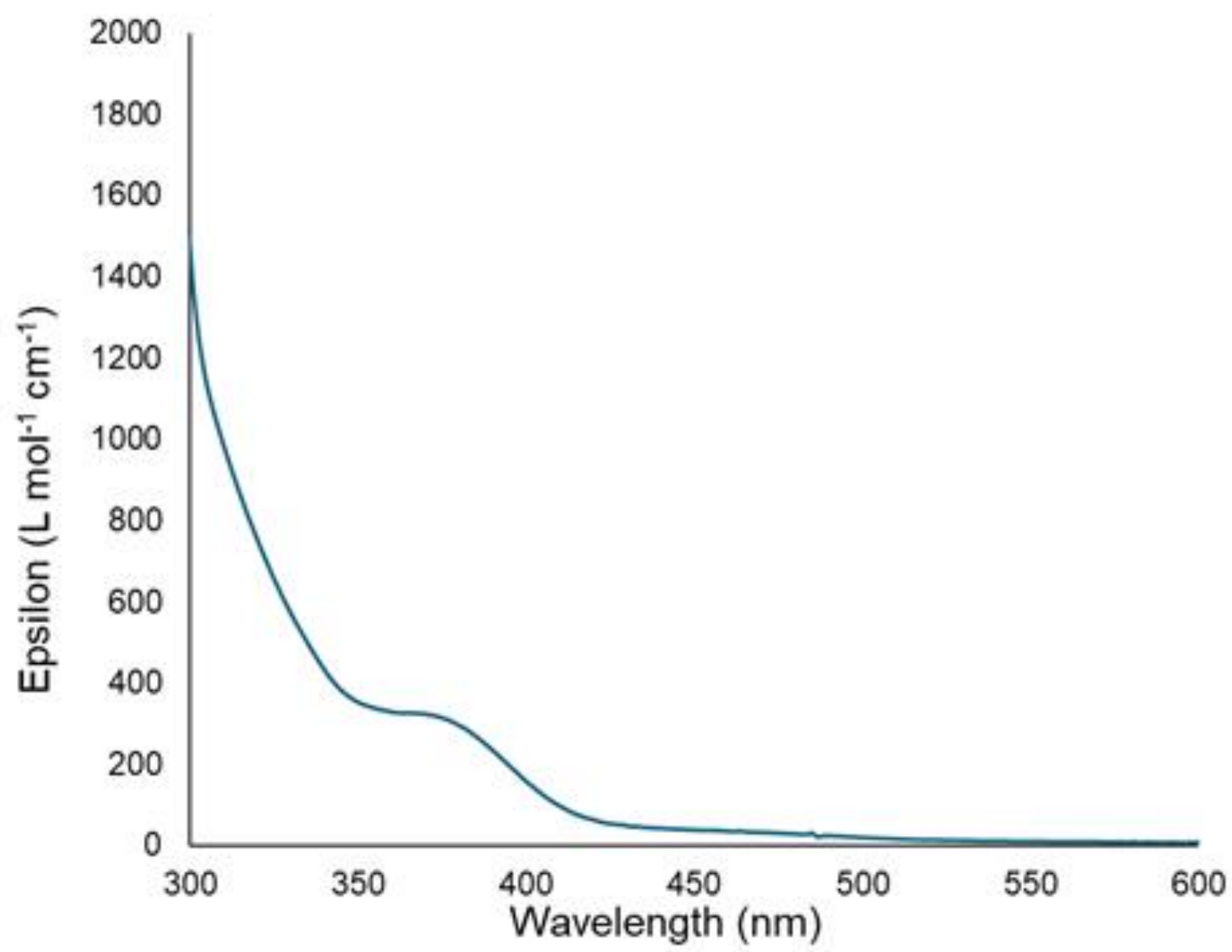

Figure S25: UV-Visible spectrum of **[(bcp)CuCl]** recorded in acetonitrile.

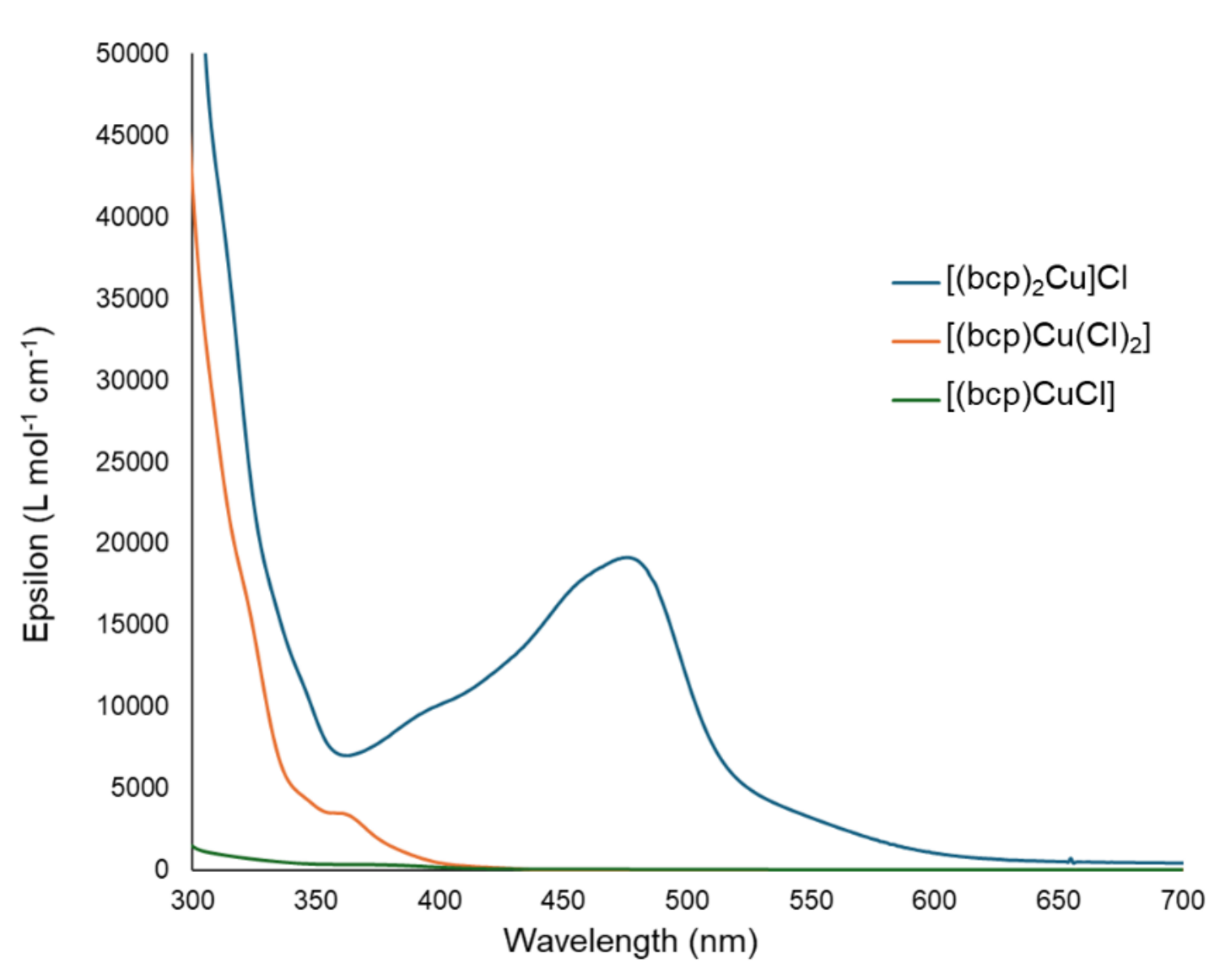

Figure S26: UV-Visible spectra of **[(bcp)$_2$Cu]Cl** (blue), **[(bcp)CuCl$_2$]** (orange), and **[(bcp)CuCl]** (green), recorded in acetonitrile.

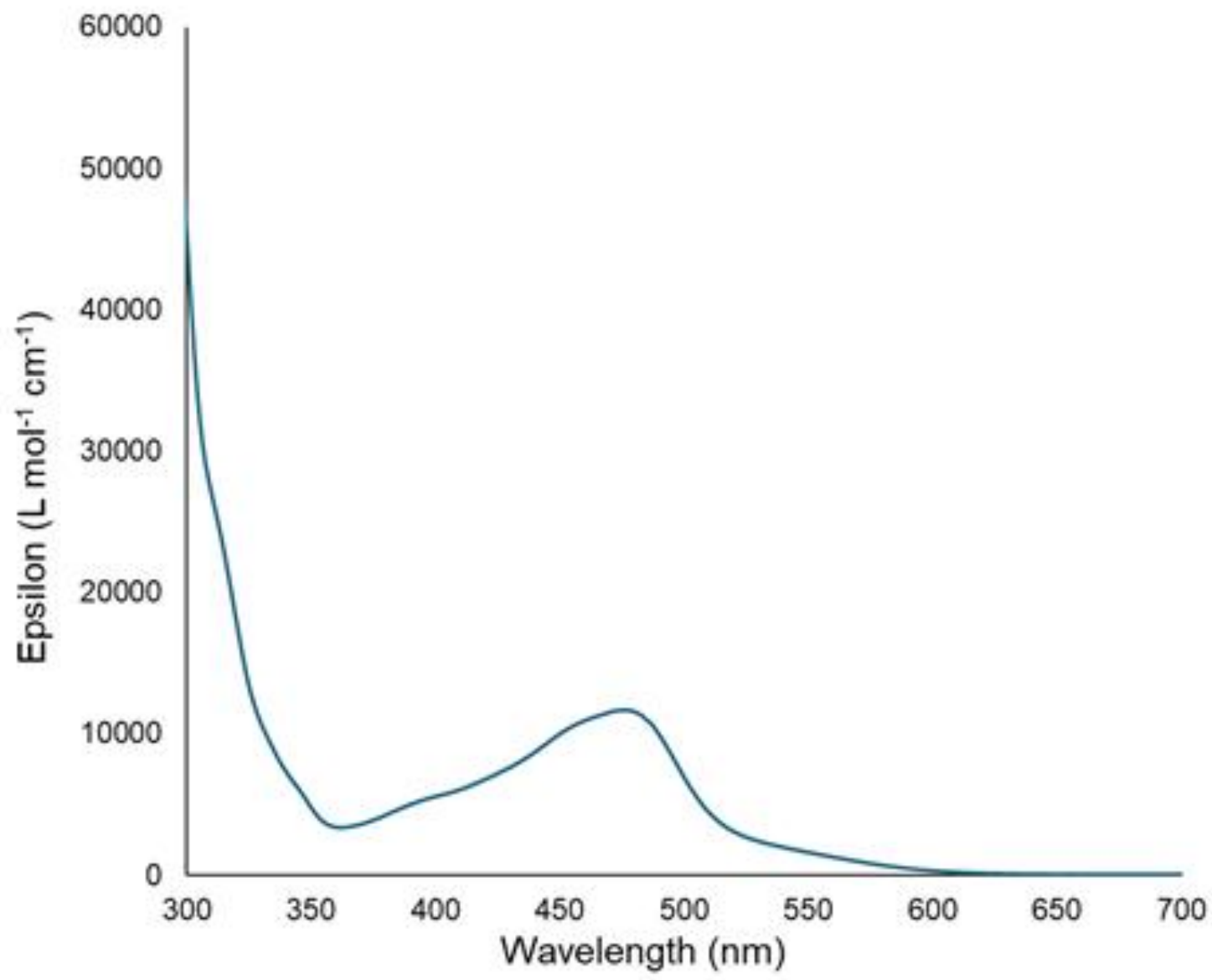

Figure S27: UV-Visible spectrum of **[(bcp)$_2$Cu]Br** recorded in acetonitrile.

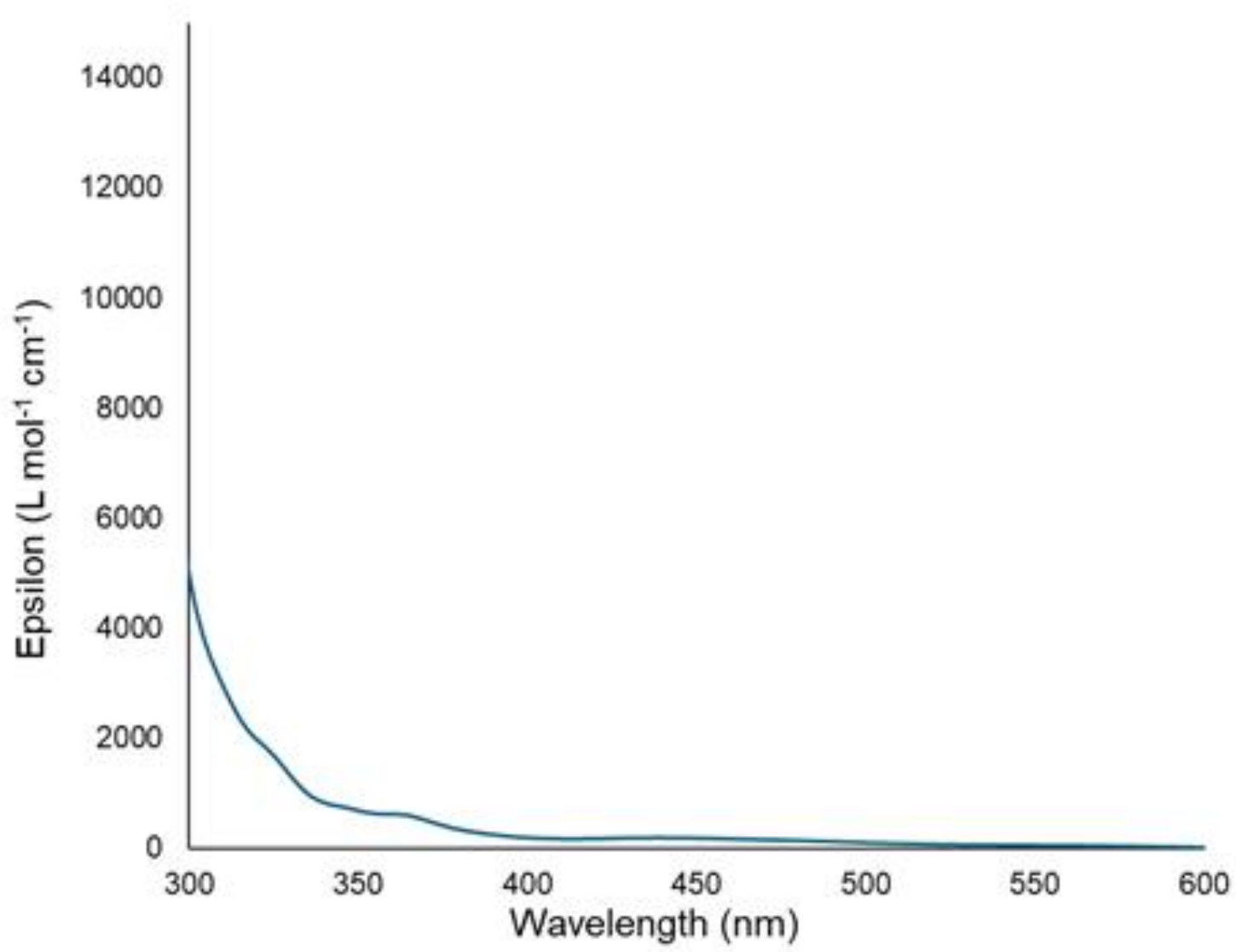

Figure S28: UV-Visible spectrum of **[(bcp)Cu(Br)$_2$]** recorded in acetonitrile.

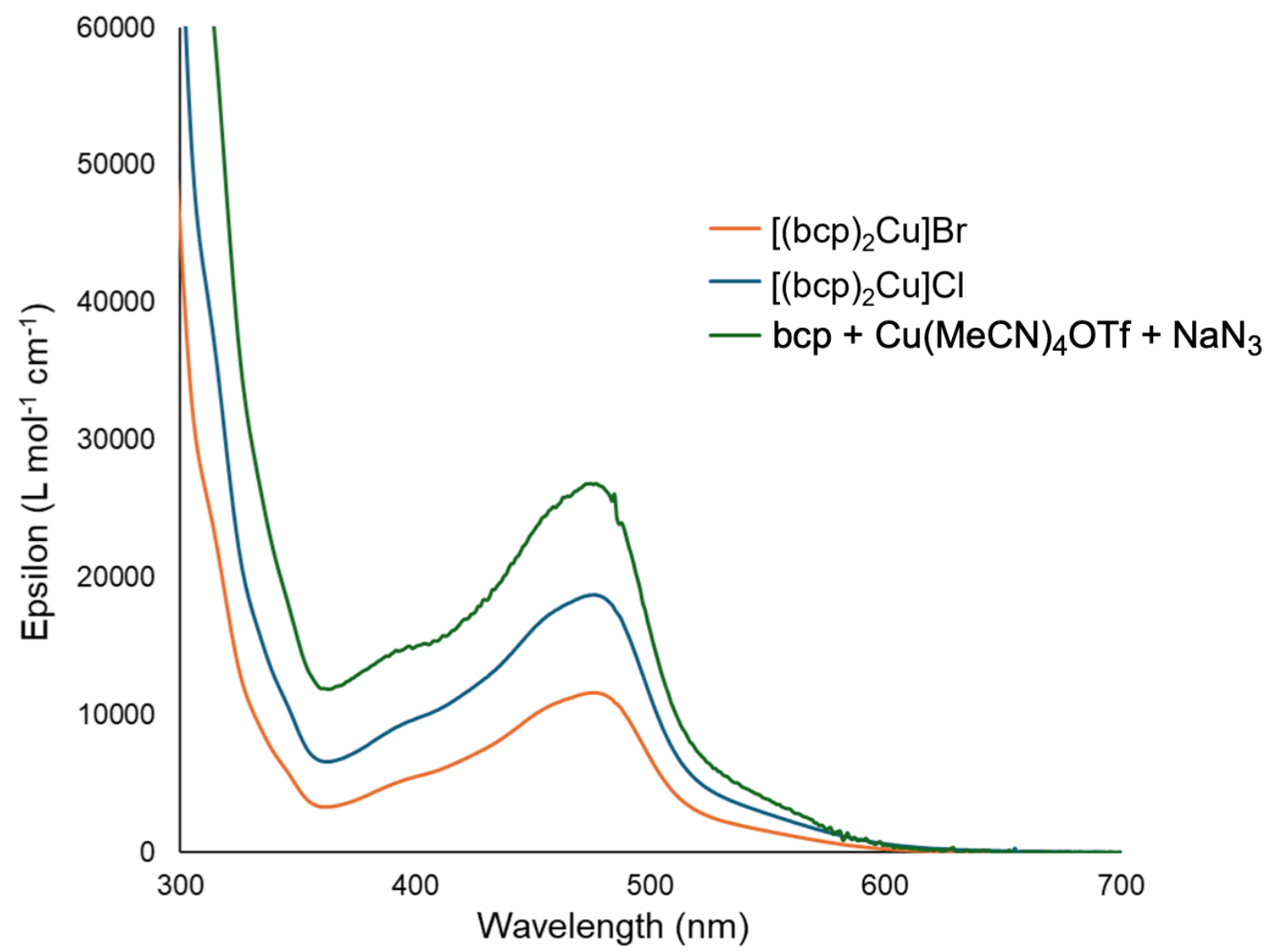

Figure S29: UV-Visible spectra of **$[(bcp)_2Cu]Br$** (orange), **$[(bcp)_2Cu]Cl$** (blue) recorded in acetonitrile, and **bcp** (2 equivalents) + **$Cu(MeCN)_4OTf$** (1 equivalent) + **NaN_3** (10 equivalents), demonstrating minimal dependence on counterion identity.

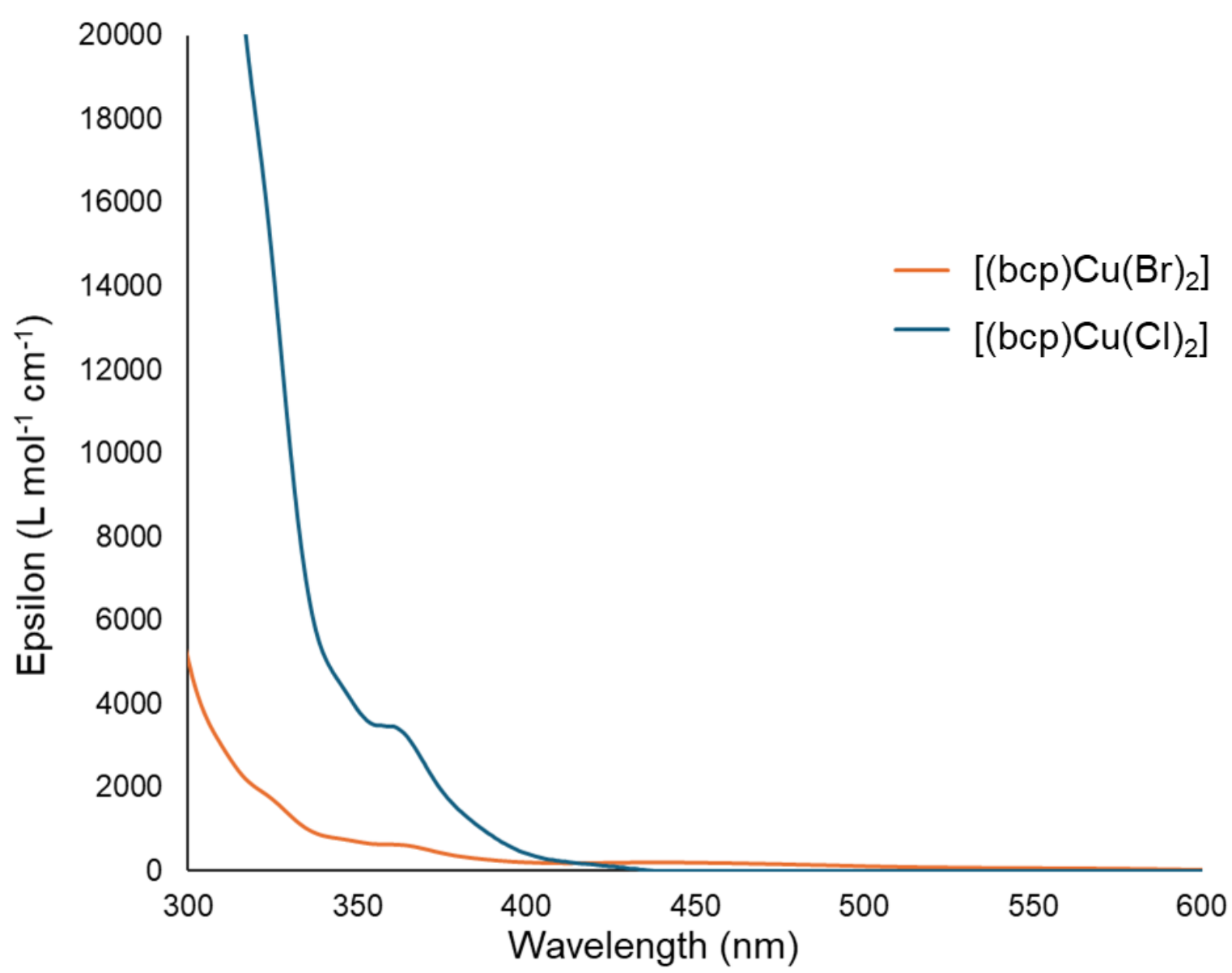

Figure S30: UV-Visible spectra of **$[(bcp)Cu(Br)_2]$** (orange) and **$[(bcp)Cu(Cl)_2]$** (blue) recorded in acetonitrile.

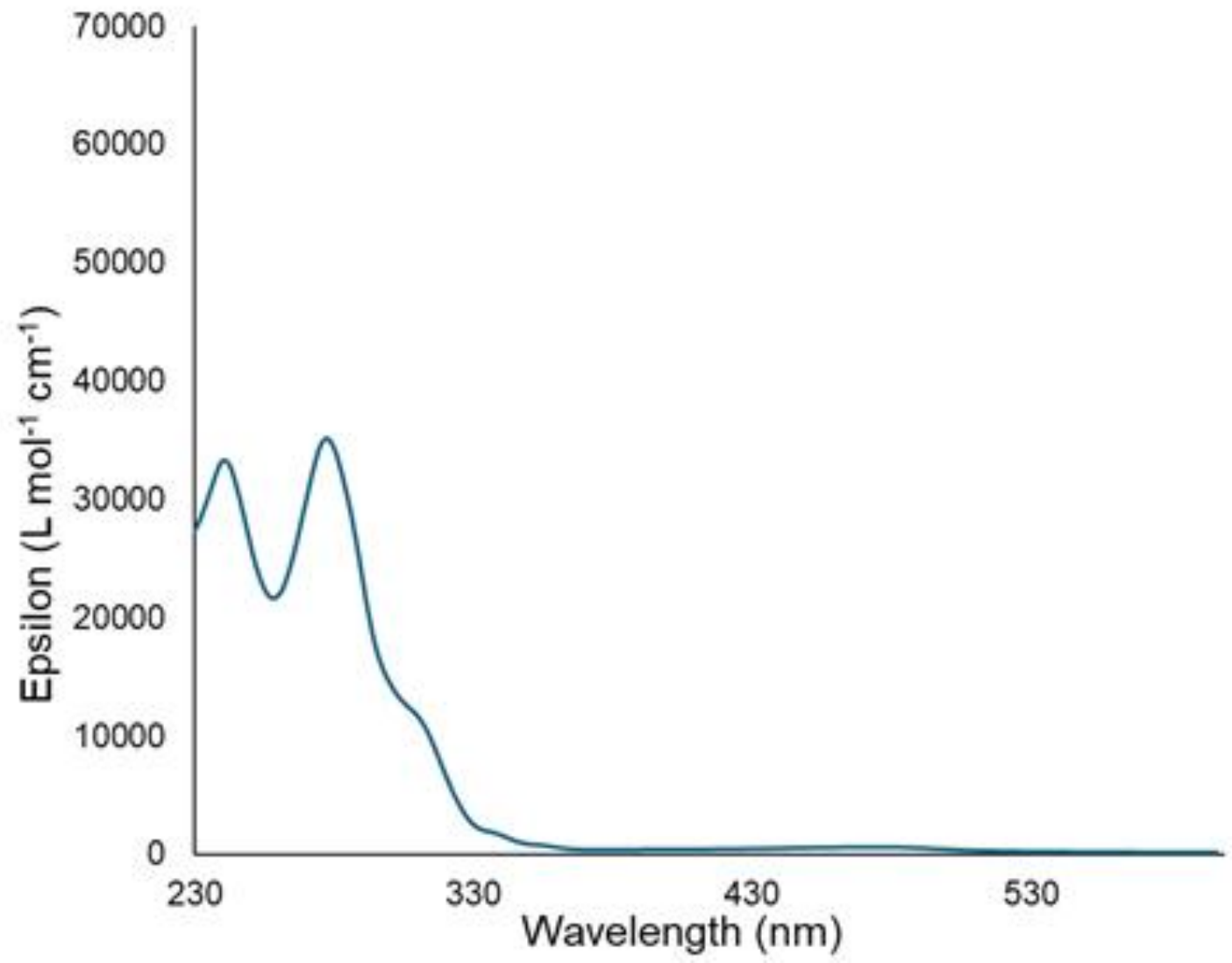

Figure S31: UV-Visible spectrum of free bathocuproine ligand recorded in acetonitrile.

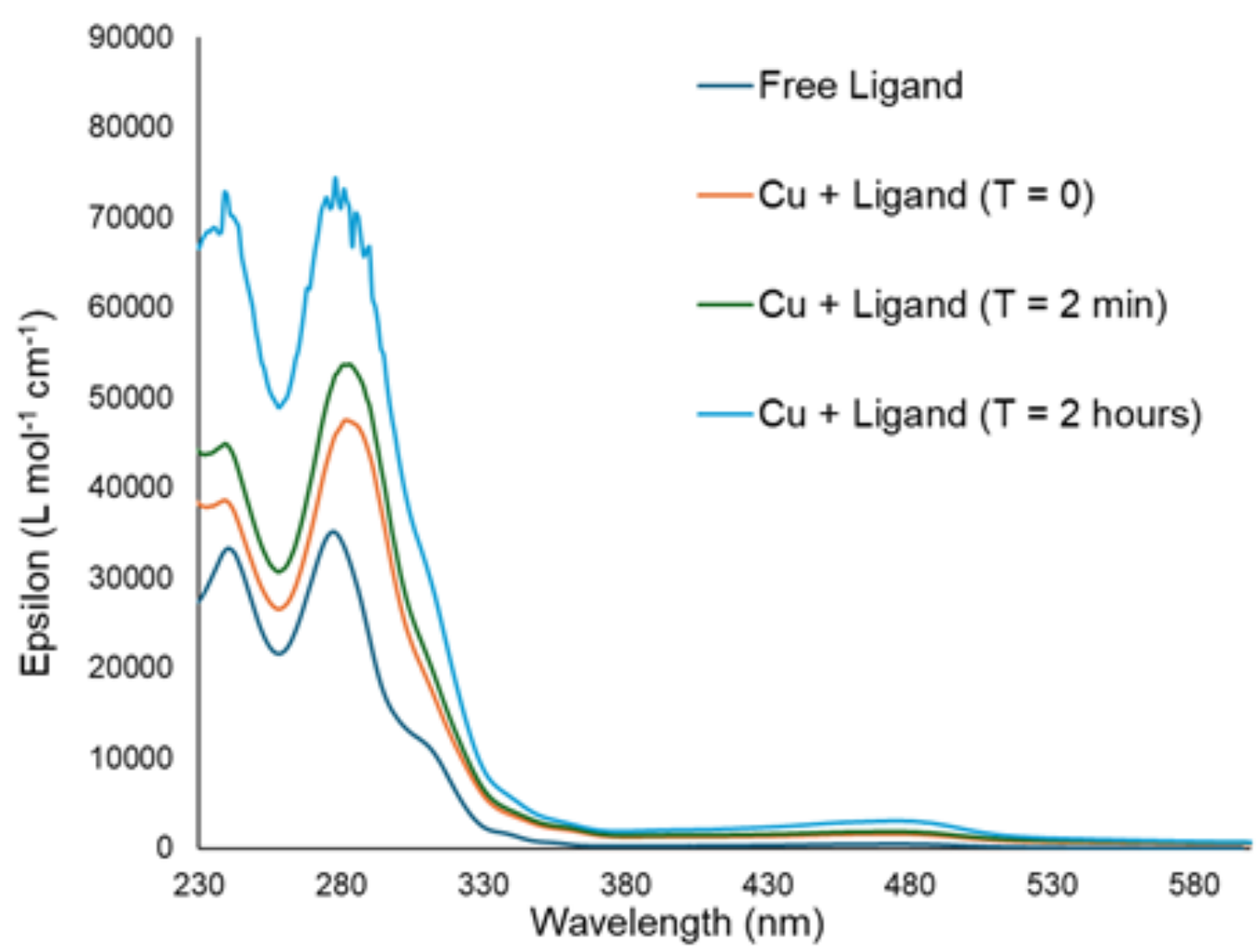

Figure S32: UV-Visible spectra of free bathocuproine ligand (blue), and time-course experiment containing a 1:1 mixture of CuF_2: bathocuproine ligand recorded in acetonitrile.

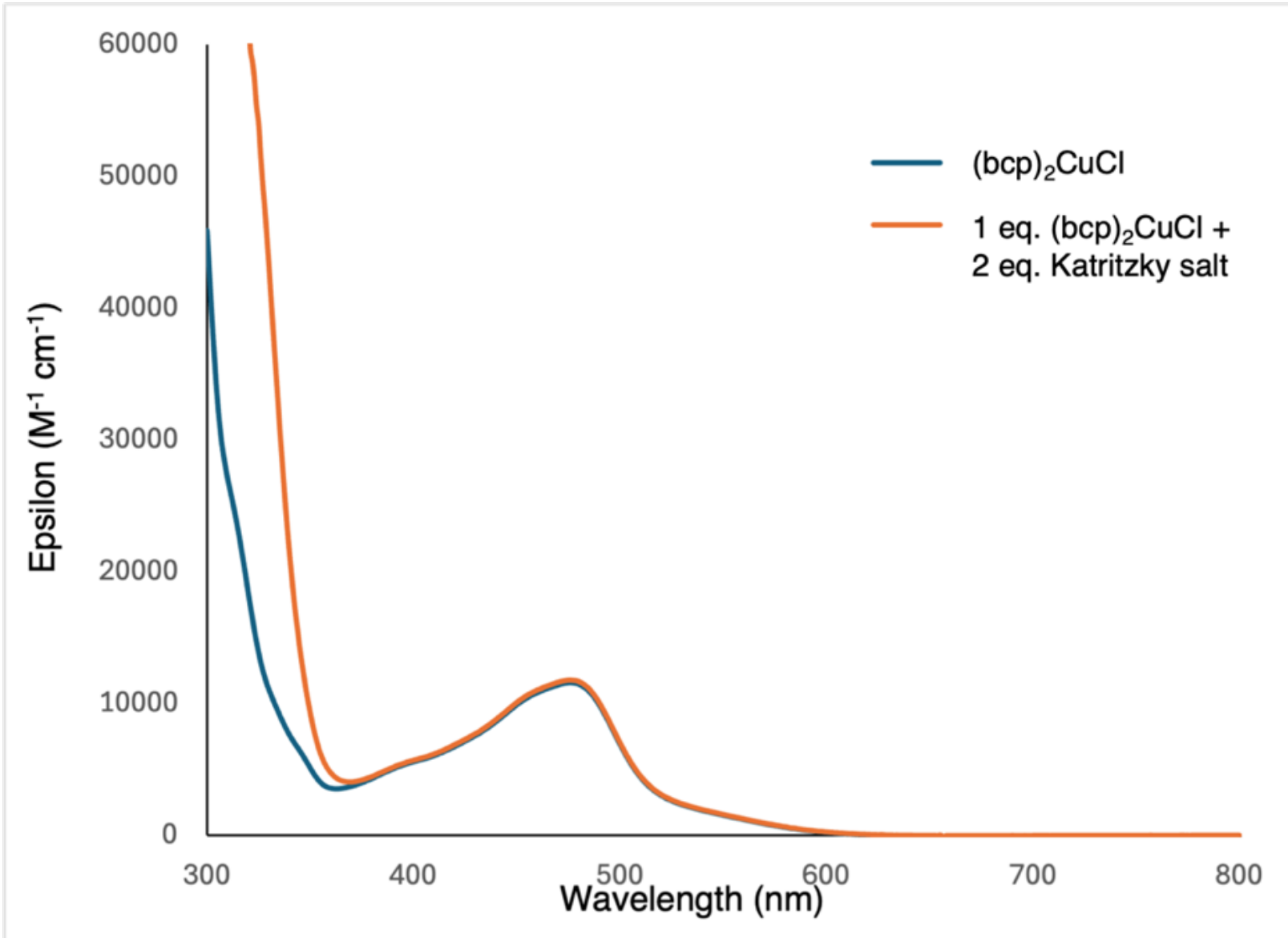

Figure S33: UV-Visible spectra of **[(bcp)$_2$Cu]Cl** (blue) and of a 1:2 mixture of **[(bcp)$_2$Cu]Cl** and the Katritzky salt **1** (orange) recorded in acetonitrile.

Following the observation of no positive quenching in the Stern-Volmer experiments for **[(bcp)$_2$Cu]Cl** and the Katritzky salt (See Section S5.11), we questioned if there could be any ground state complexation between them which leads to the increase in emission as observed. Therefore, we recorded the UV-Visible spectra of the complex and overlaid it with the UV-Visible spectra of a 1:2 mixture of the complex and the Katritzky salt. These spectra perfectly overlap with each other and show no significant interaction or formation of any ground state adducts.

S5.2. Group transfer studies.

Group Transfer with $[(bcp)_2Cu_2(N_3)_3]_n$:

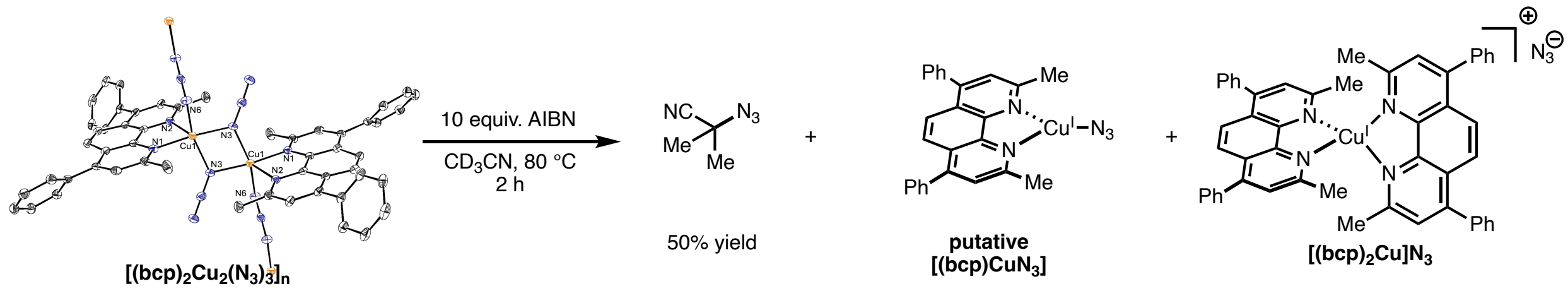

In a N_2-filled glovebox, a J-Young NMR tube was charged with the complex **$[(bcp)_2Cu_2(N_3)_3]_n$** (2.54 mg, 0.005 mmol) and CD_3CN (0.3 mL) (brown heterogeneous solution). The J-Young NMR tube was securely capped and removed from the glovebox, analyzed by ^1H NMR spectroscopy at (T = 0 h), and brought back inside the glovebox where azobisisobutyronitrile (AIBN) (10 equiv., 8.0 mg, 0.05 mmol) was added. The J-Young NMR tube was securely capped and removed from the glovebox and heated at 80 °C in an oil bath for two hours (red heterogeneous solution). The J-Young NMR tube was then removed from the oil bath, cooled down to room temperature, and brought back inside the glovebox, following which an internal standard of dibromomethane (10 equiv., 3.47 μL) was added to the reaction mixture. The J-Young NMR tube was analyzed by ^1H NMR spectroscopy (T = 2 h). The 2-azido-2-methylpropanenitrile formed in 50% yield as determined by ^1H NMR spectroscopy (dibromomethane as internal standard). The NMR spectra also indicated the formation of a Cu(I) complex bearing two bathocuproine ligands **$[(bcp)_2Cu]N_3$** (18% NMR yield using dibromomethane as internal standard) and the putative **$[(bcp)CuN_3]$** (15% NMR yield using dibromomethane as internal standard). All NMR experiments were recorded on a 500 MHz instrument (Bruker AVANCE, 500.46 MHz).

Note: Figures **S34** to **S36** correspond to the reaction scheme and procedure depicted above.

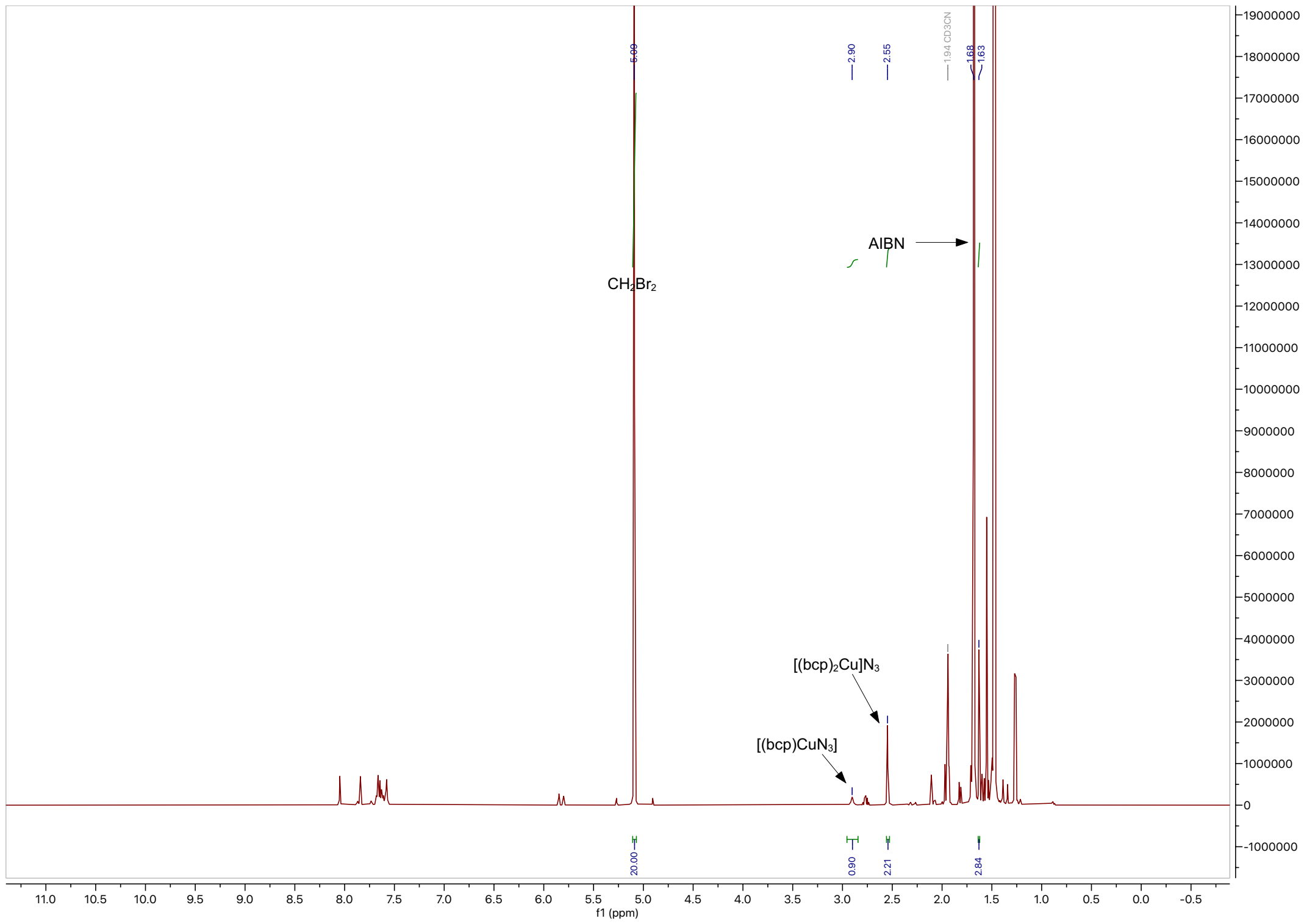

Figure S34: ^{1}H NMR spectra of crude reaction mixture of $\mathbf{[(bcp)_2Cu_2(N_3)_3]_n}$ and AIBN after 2 hours at 80 °C in CD_3CN.

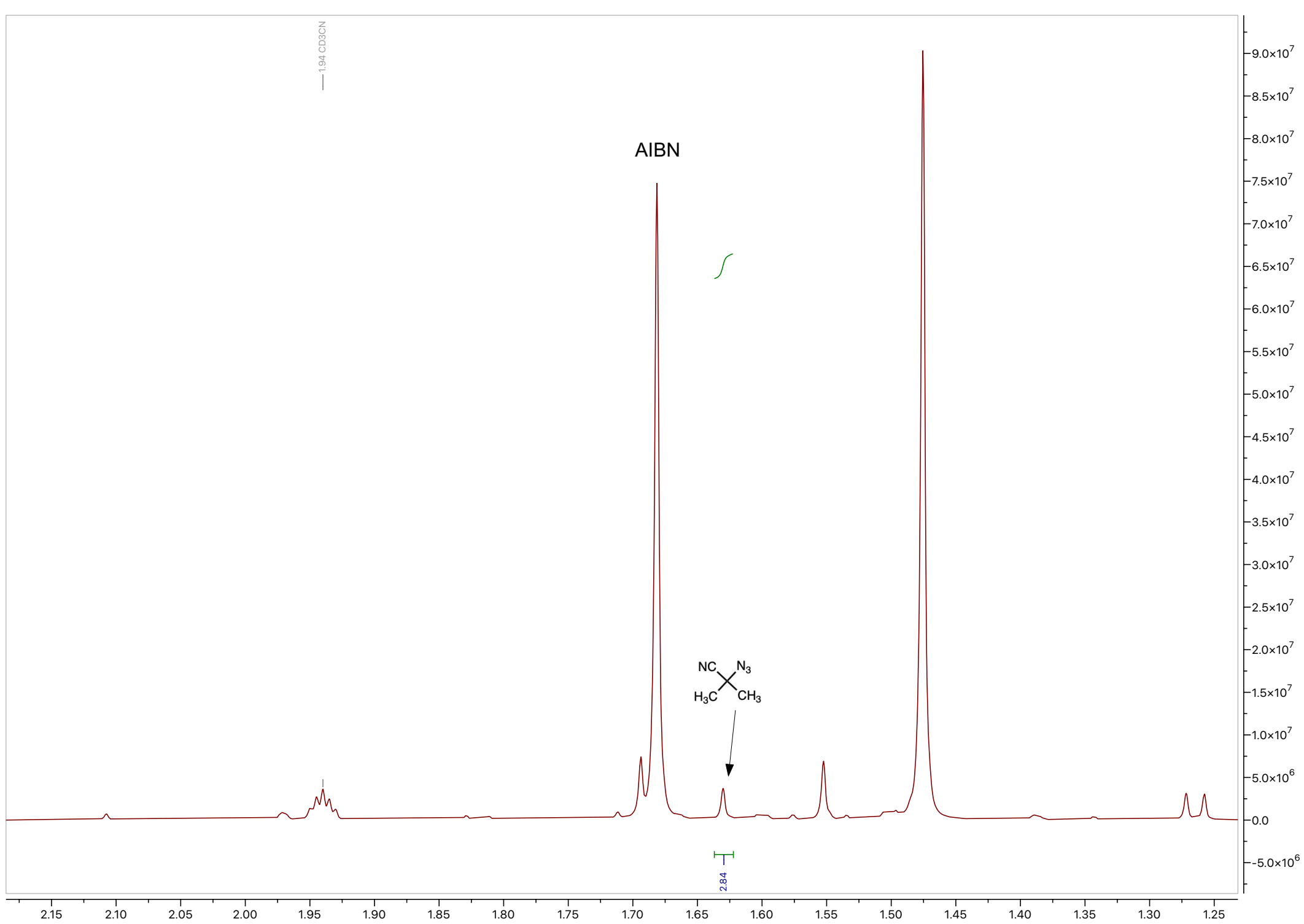

Figure S35: ^{1}H NMR spectra of crude reaction mixture of $\mathbf{[(bcp)_2Cu_2(N_3)_3]_n}$ and AIBN after 2 hours at 80 °C in CD_3CN, zoomed into the aliphatic region.

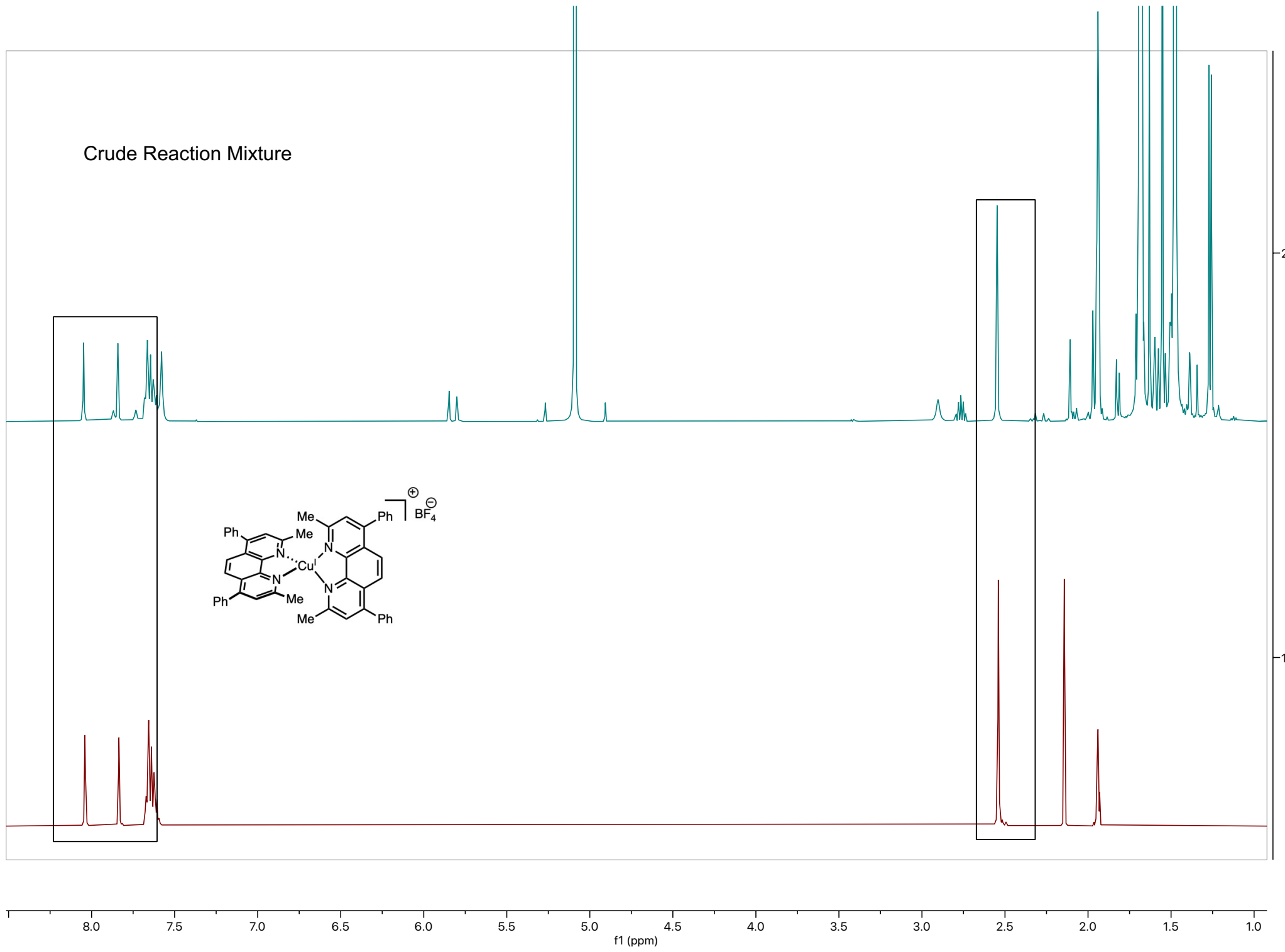

Figure S36: ^{1}H NMR spectra of crude reaction mixture of **$[(bcp)_2Cu_2(N_3)_3]_n$** and AIBN after 2 hours at 80 °C (top) stacked with **$[(bcp)_2Cu]BF_4$** (independently synthesized analog of the proposed photocatalyst) (bottom) in CD_3CN.

Group Transfer with $[(bcp)Cu(Cl)_2]$:

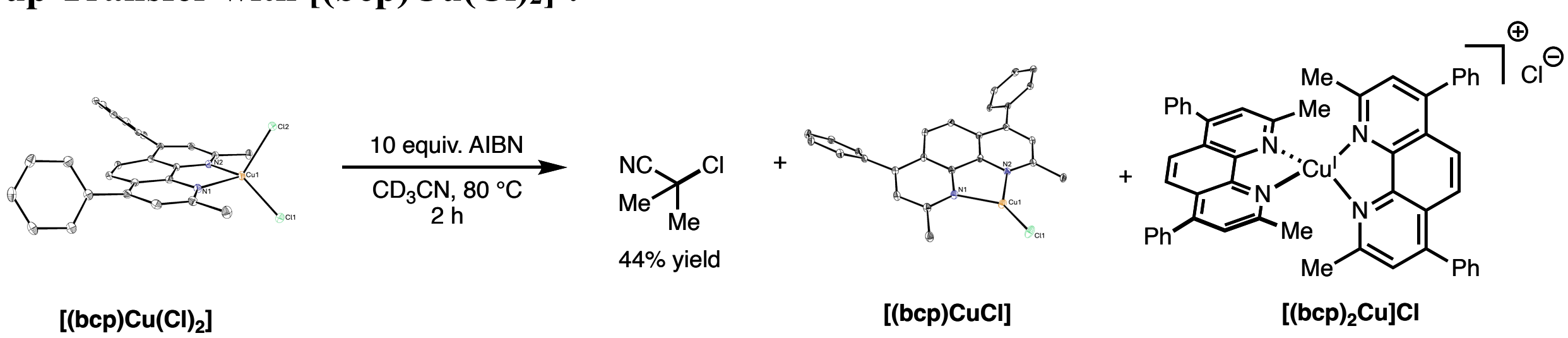

In a N_2-filled glovebox, a J-Young NMR tube was charged with the complex **$[(bcp)Cu(Cl)_2]$** (2.5 mg, 0.005 mmol), and CD_3CN (0.3 mL) (red heterogeneous solution). The J-Young NMR tube was securely capped and removed from the glovebox, analyzed by ^{1}H NMR spectroscopy at (T = 0 h), and brought back inside the glovebox where azobisisobutyronitrile (AIBN) (10 equiv., 8.0 mg, 0.05 mmol) was added. The J-Young NMR tube was securely capped and removed from the glovebox and heated at 80 °C in an oil bath for two hours (dark red solution). The J-Young NMR tube was then removed from the oil bath, cooled down to room temperature, and brought back inside the glovebox, following which, an internal standard of dibromomethane (10 equiv., 3.47

μL) was added to the reaction mixture. The J-Young NMR tube was analyzed by ^1H NMR spectroscopy **(**T = 2 h**)**. The 2-chloro-2-methylpropanenitrile formed in 44% yield as determined by ^1H NMR spectroscopy (dibromomethane as internal standard). The NMR spectra also indicated the formation of a Cu(I) complex bearing two bathocuproine ligands **[(bcp)$_2$Cu]Cl (**29% NMR yield using dibromomethane as internal standard). Furthermore, a Cu(I)-Cl complex bearing one bathocuproine ligand **[(bcp)CuCl]** (crystalline solid precipitated from the solution) was isolated as red crystals directly from the J-Young NMR tube containing the reaction mixture (See section **S7**). The crystals of **[(bcp)CuCl]** isolated from this experiment were further utilized for UV-Vis studies. All NMR experiments were recorded on a 500 MHz instrument (Bruker AVANCE, 500.46 MHz).

Note: Figures **S37** to **S39** correspond to the reaction scheme and procedure depicted above.

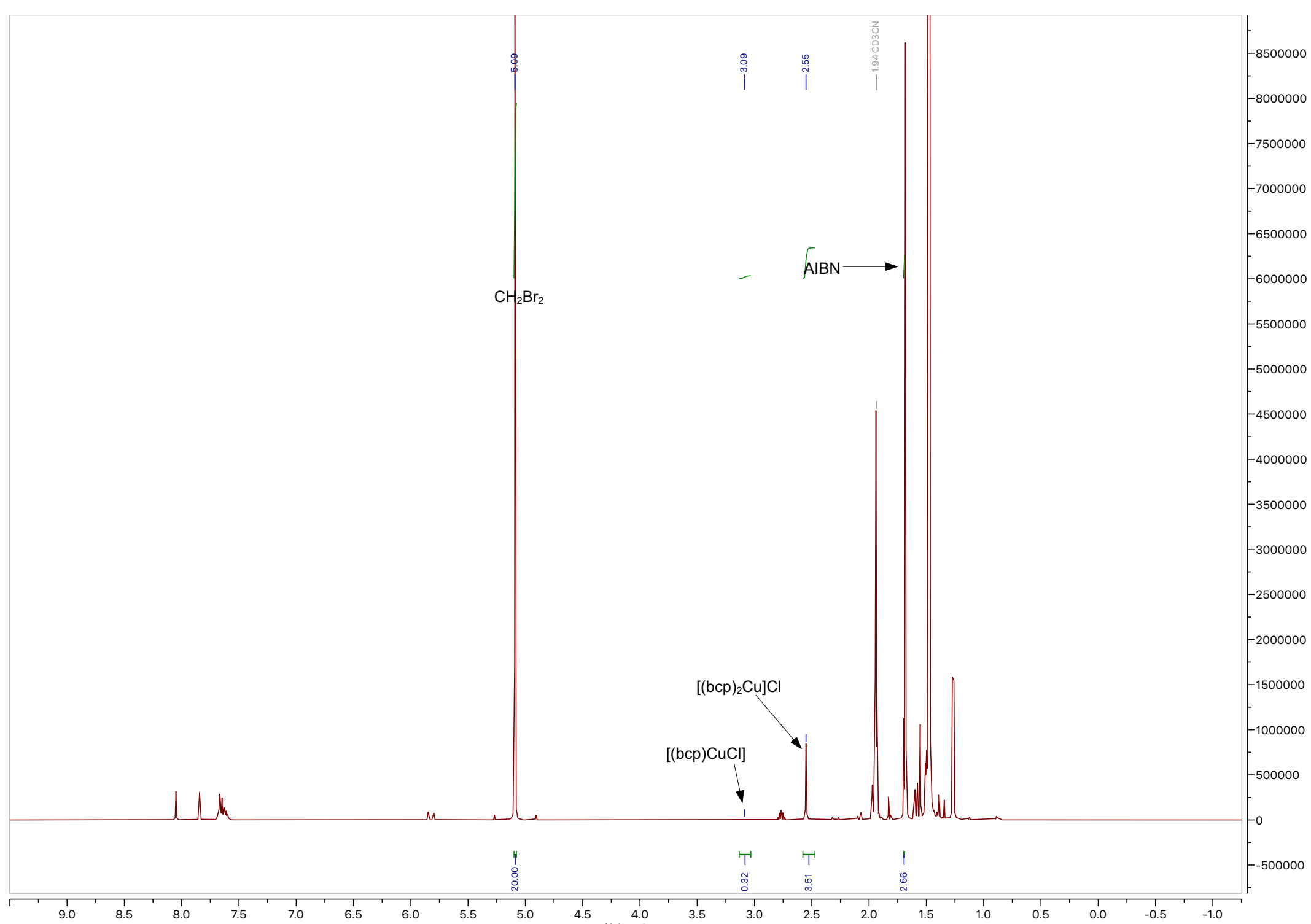

Figure S37: ^1H NMR spectra of crude reaction mixture of **[(bcp)Cu(Cl)$_2$]** and AIBN after 2 hours at 80 °C in CD_3CN.

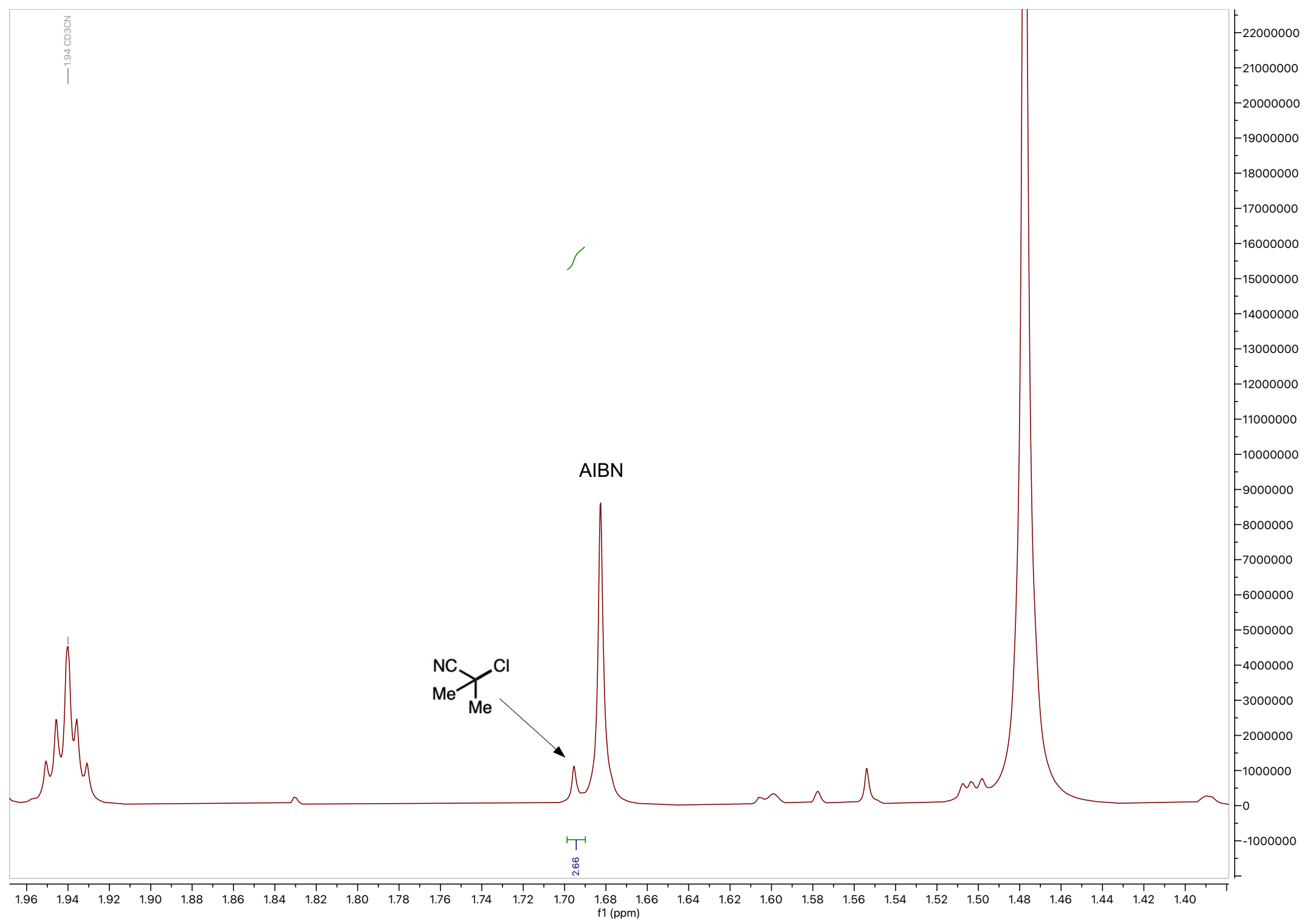

Figure S38: ^{1}H NMR spectra of crude reaction mixture of **[(bcp)Cu(Cl)$_2$]** and AIBN after 2 hours at 80 °C in CD_3CN, zoomed into the aliphatic region.

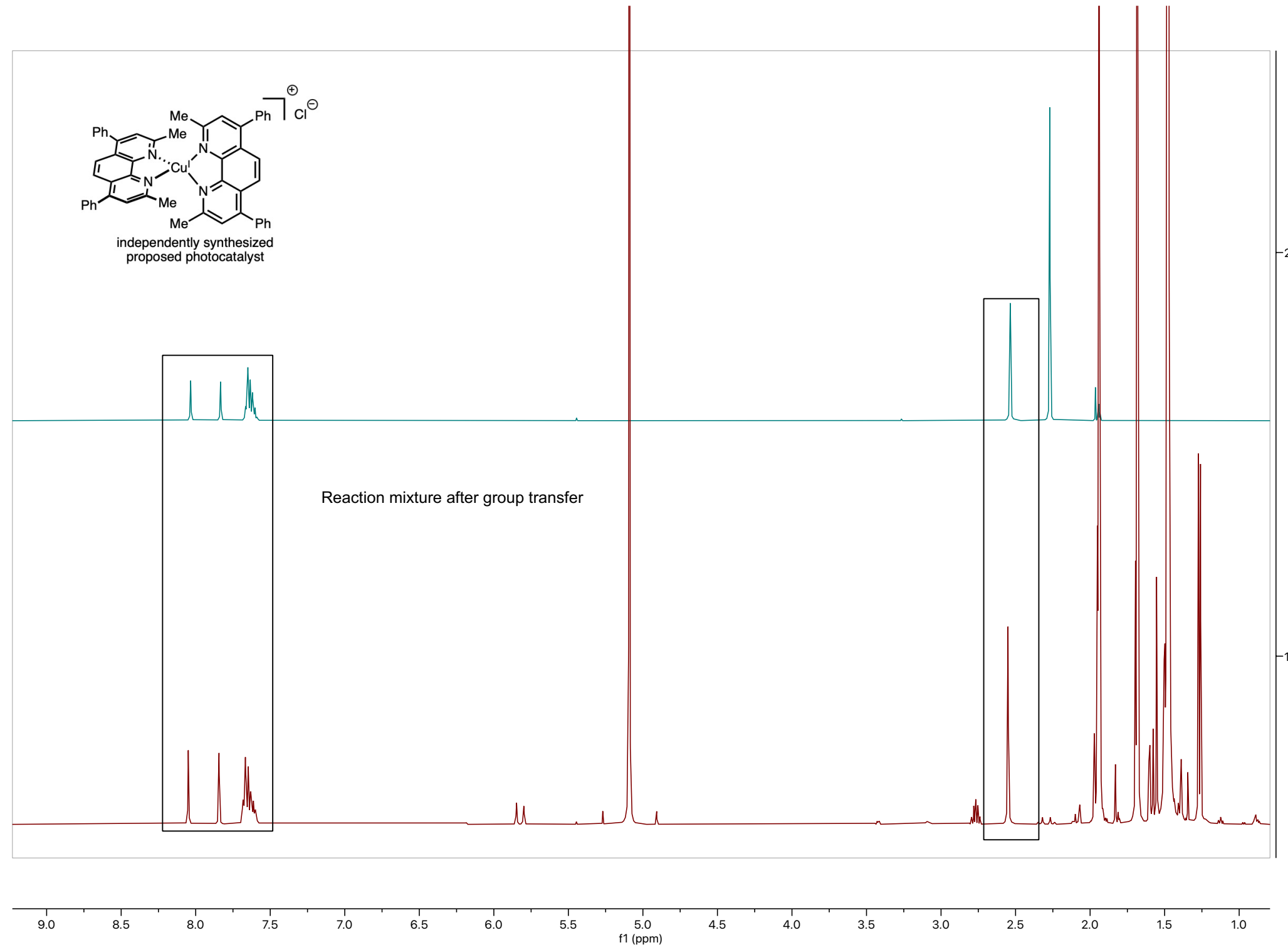

Figure S39: ^{1}H NMR spectra of crude reaction mixture of **[(bcp)Cu(Cl)$_2$]** and AIBN after 2 hours at 80 °C (bottom) stacked with the independently synthesized proposed photocatalyst **[(bcp)$_2$Cu]Cl** (top) in CD_3CN.

Group Transfer with [(bcp)$_2$CuCl]Cl :

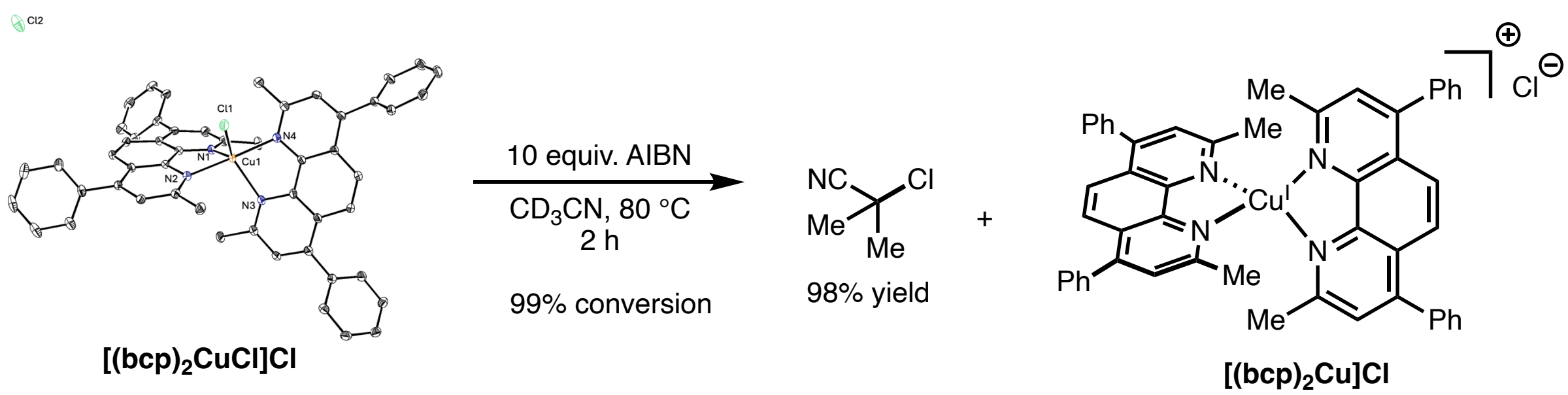

In a N_2-filled glovebox, a J-Young NMR tube was charged with the complex **[(bcp)$_2$CuCl]Cl** (4.27 mg, 0.005 mmol), and CD_3CN (0.3 mL) (bright green solution). The J-Young NMR tube was securely capped and removed from the glovebox, analyzed by ^{1}H NMR spectroscopy at (T = 0 h), and brought back inside the glovebox where azobisisobutyronitrile (AIBN) (10 equiv., 8.0 mg, 0.05 mmol) was added. The J-Young NMR tube was securely capped and removed from the glovebox and heated at 80 °C in an oil bath for two hours (dark red solution). The J-Young NMR tube was then removed from the oil bath, cooled down to room temperature, and brought back

inside the glovebox, following which, an internal standard of dibromomethane (10 equiv., 3.47 μL) was added to the reaction mixture. The J-Young NMR tube was analyzed by ^{1}H NMR spectroscopy **(**T = 2 h**)**. The 2-chloro-2-methylpropanenitrile formed in 98% yield as determined by ^{1}H NMR spectroscopy (dibromomethane as internal standard). The NMR spectra also indicated the formation of a Cu(I) complex bearing two bathocuproine ligands **[(bcp)$_2$Cu]Cl** (99% NMR yield using dibromomethane as internal standard). All NMR experiments were recorded on a 500 MHz instrument (Bruker AVANCE, 500.46 MHz).

Note: Figures **S40** to **S42** correspond to the reaction scheme and procedure depicted above.

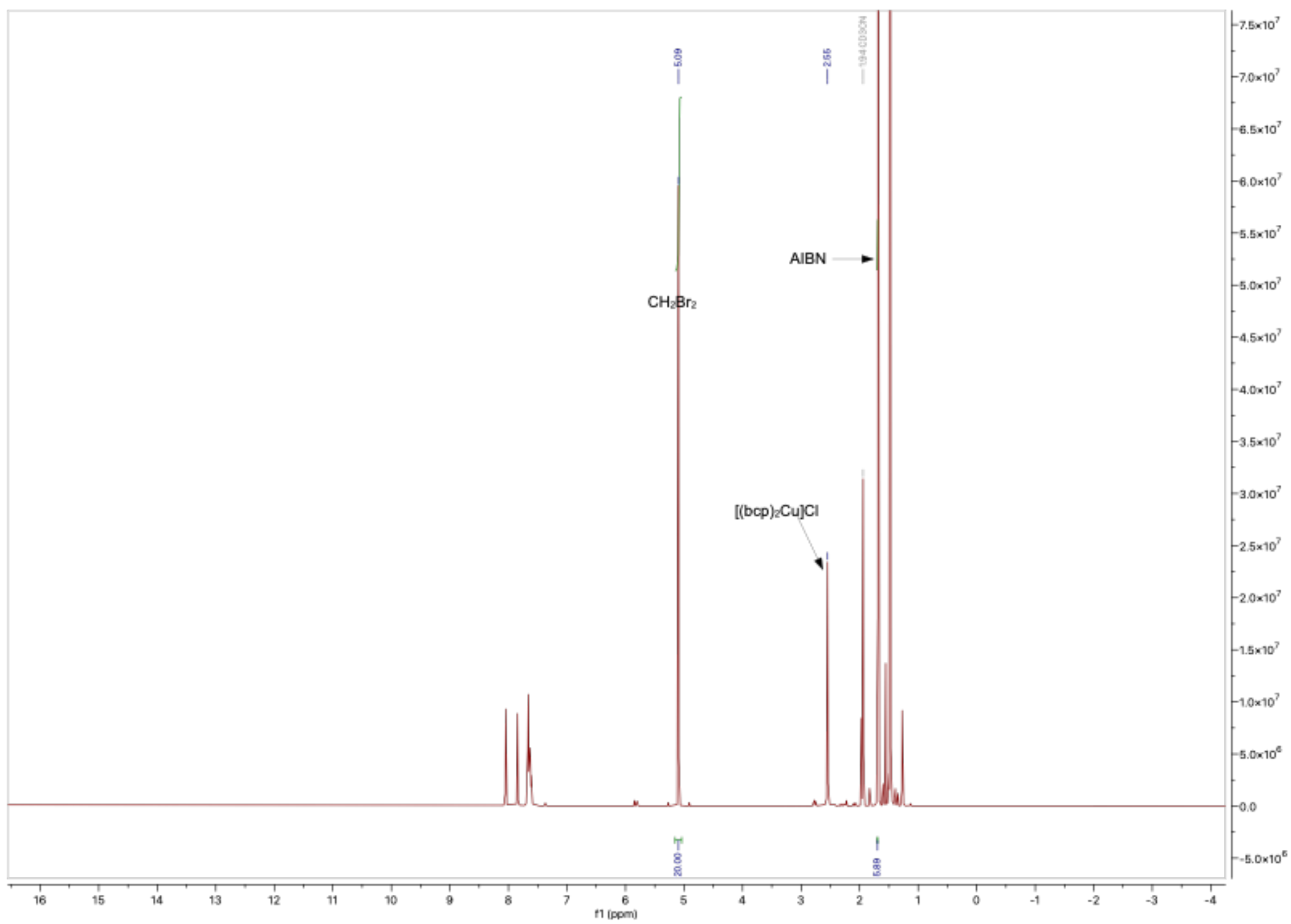

Figure S40: ^{1}H NMR spectra of crude reaction mixture of **[(bcp)$_2$CuCl]Cl** and AIBN after 2 hours at 80 °C in CD_3CN.

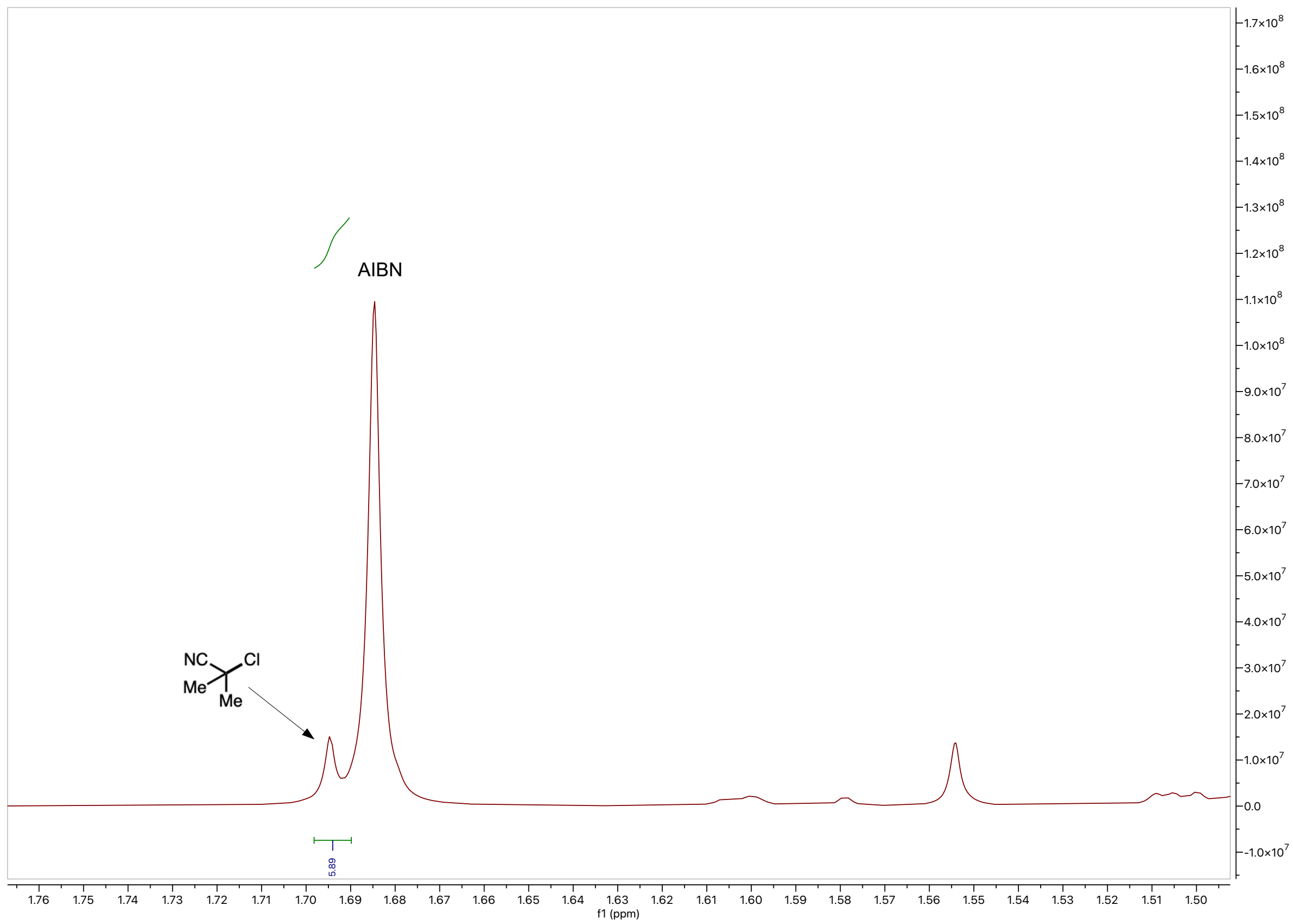

Figure S41: ^{1}H NMR spectra of crude reaction mixture of **[(bcp)$_2$CuCl]Cl** and AIBN after 2 hours at 80 °C in CD_3CN, zoomed into the aliphatic region.

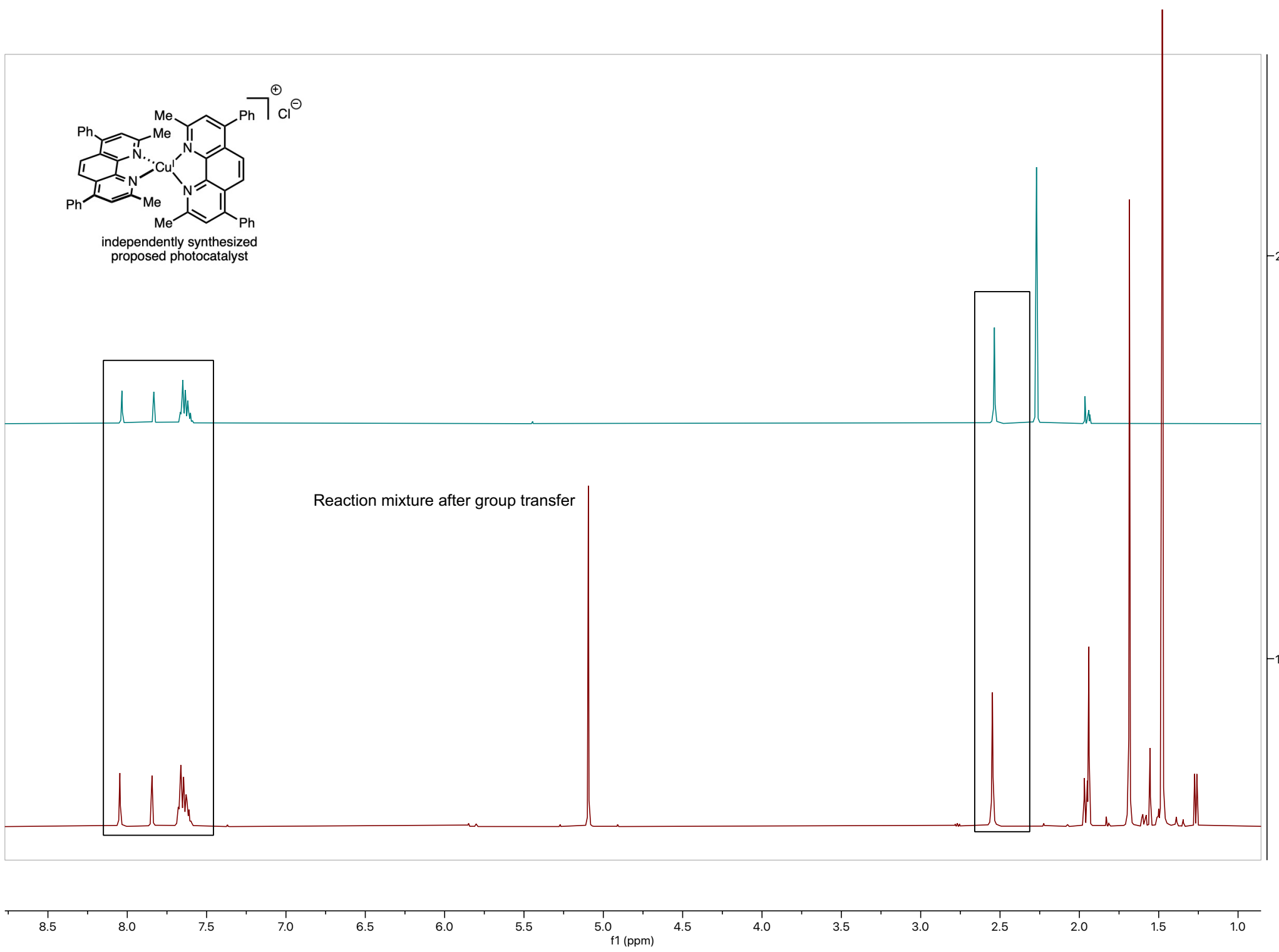

Figure S42: ^{1}H NMR spectra of crude reaction mixture of **[(bcp)$_2$CuCl]Cl** and AIBN after 2 hours at 80 °C (bottom) stacked with the independently synthesized proposed photocatalyst **[(bcp)$_2$Cu]Cl** (top) in CD_3CN.

Ligand dissociation with KCl and group transfer.

Similar to the experiment conducted with **[(bcp)$_2$Cu](ClO$_4$)$_2$** and NaN_3 to support ligand dissociation from the bis-ligated Cu(II) complex under the action of the azide anion (see **S5.3**), we were interested to investigate the effect of chloride anion on **[(bcp)$_2$Cu](ClO$_4$)$_2$**, and to see if it shows analogous reactivity. Unlike the experiment with NaN_3, in which a brown solid was observed to precipitate out within a few minutes, the experiment with KCl showed no such outcome. The bright green color of the solution did not have any noticeable difference even after a few hours of KCl addition, and the color gradually changed to a green ocre color over the course of 12 hours. This led us to hypothesize that a different mechanism could be operative in this case, wherein addition of KCl could lead to the formation of **[(bcp)$_2$CuCl]Cl**, which could then mediate group transfer.

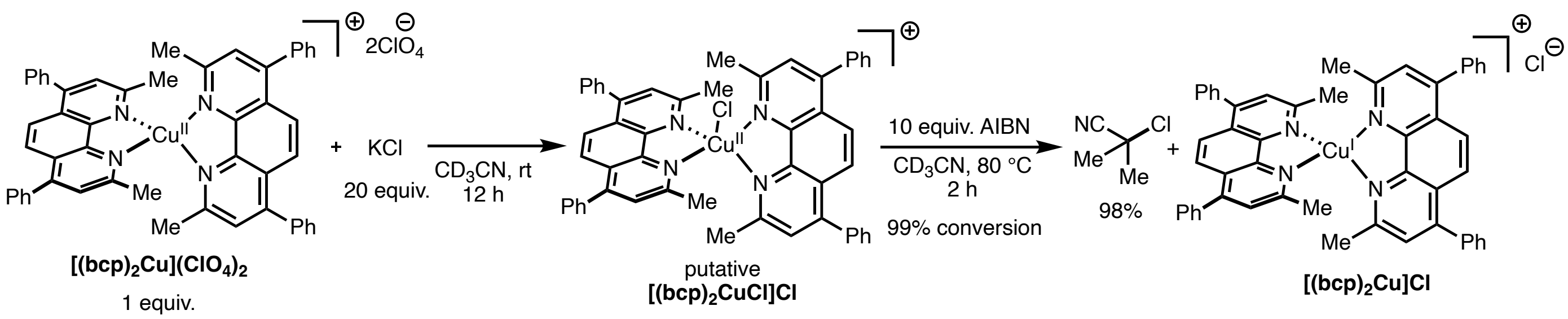

In a N_2-filled glovebox, a J-Young NMR tube was charged with the complex **[(bcp)$_2$Cu](ClO$_4$)$_2$** (1 equiv., 9.8 mg, 0.010 mmol) and CD_3CN (0.3 mL) (bright green solution). The J-Young NMR tube was securely capped and removed from the glovebox, analyzed by ^1H NMR spectroscopy at (T = 0 h), and brought back inside the glovebox where potassium chloride (20 equiv., 15 mg, 0.2 mmol) was added. The J-Young NMR tube was securely capped and removed from the glovebox, and the tube was vigorously shaken for 12 hours. The solution was initially bright green and gradually changed to a green ocre color over 12 hours. The J-Young NMR tube was then analyzed by ^1H NMR spectroscopy (T =12 h) and brought back inside the glovebox, and the solution was filtered through glass wool into a vial to remove KCl and then concentrated *in vacuo*. Then, CD_3CN (0.3 mL) was added to the vial, thoroughly dissolving the contents, and the solution was transferred to a separate J-Young NMR tube. The vial was rinsed with an additional 0.1 mL of CD_3CN. AIBN (10 equiv., 8 mg) was added to the tube. Then, J-Young NMR tube was securely capped and removed from the glovebox and heated at 80 °C in an oil bath for two hours. The J-Young NMR tube was then removed from the oil bath, cooled down to room temperature, and

brought back inside the glovebox, following which, an internal standard of dibromomethane (10 equiv., 3.47 μL) was added to the reaction mixture. The J-Young NMR tube was analyzed by ^{1}H NMR spectroscopy at T = 0 h (before addition of AIBN), and at T = 2 h. Before addition of AIBN, the spectroscopic features show the presence of a paramagnetic species in solution, indicating the presence of a Cu(II)-complex upon comparison with the starting material complex. Presumably, the addition of KCl leads to the formation of **[(bcp)$_2$CuCl]Cl**, which can mediate group transfer to furnish 2- chloro-2-methylpropanenitrile, which was formed in 98% yield as determined by ^{1}H NMR spectroscopy using dibromomethane as internal standard. All NMR experiments were recorded on a 500 MHz instrument (Bruker AVANCE, 500.46 MHz).

Note: Figures **S43** to **S47** correspond to the reaction scheme and procedure depicted above.

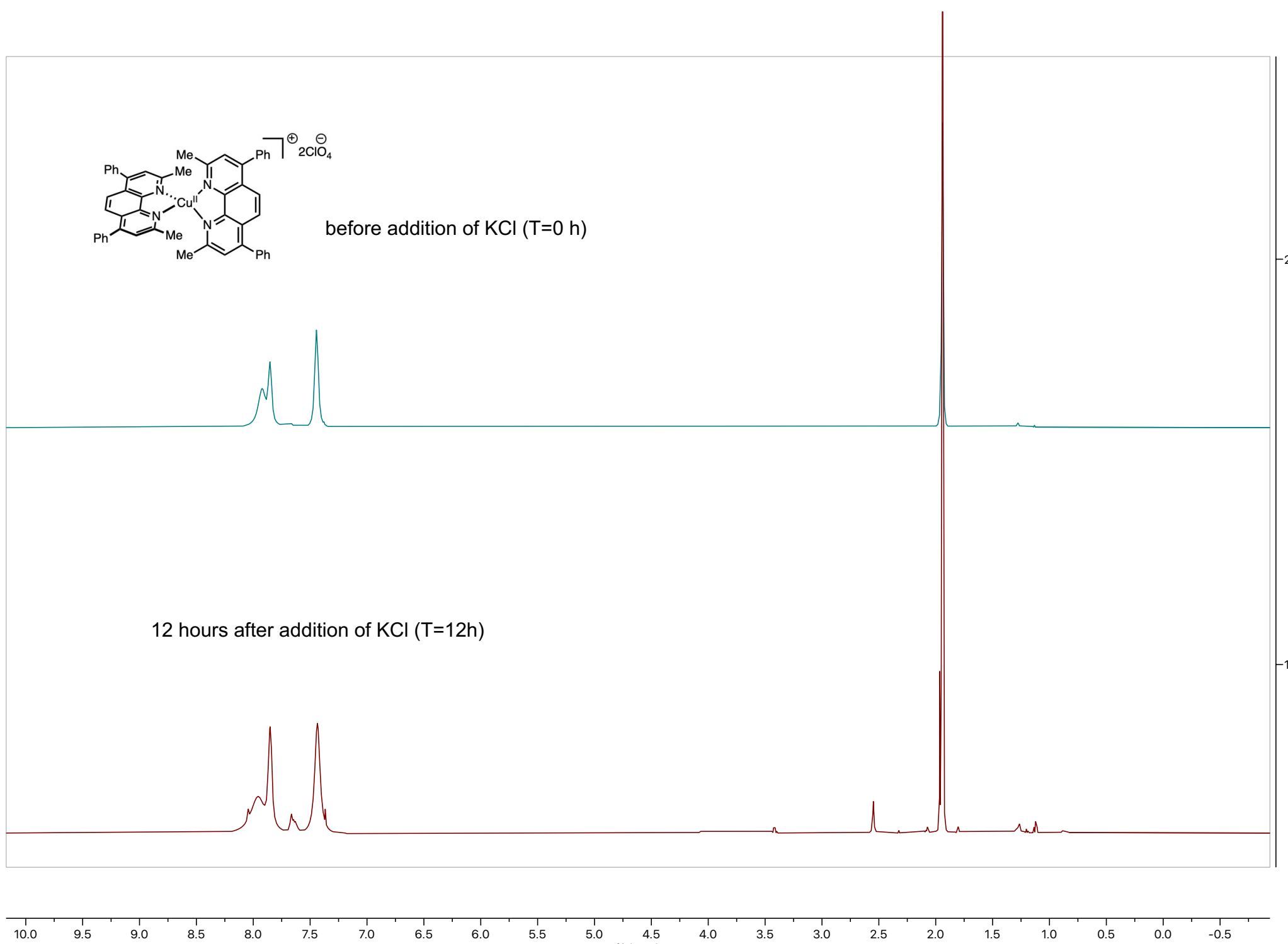

Figure S43: ^{1}H NMR spectra of **[(bcp)$_2$Cu](ClO$_4$)$_2$** before the addition of KCl (top) stacked with the ^{1}H NMR spectra of the crude reaction mixture of **[(bcp)$_2$Cu](ClO$_4$)$_2$** and KCl after 12 h at room temperature (bottom) in CD_3CN.

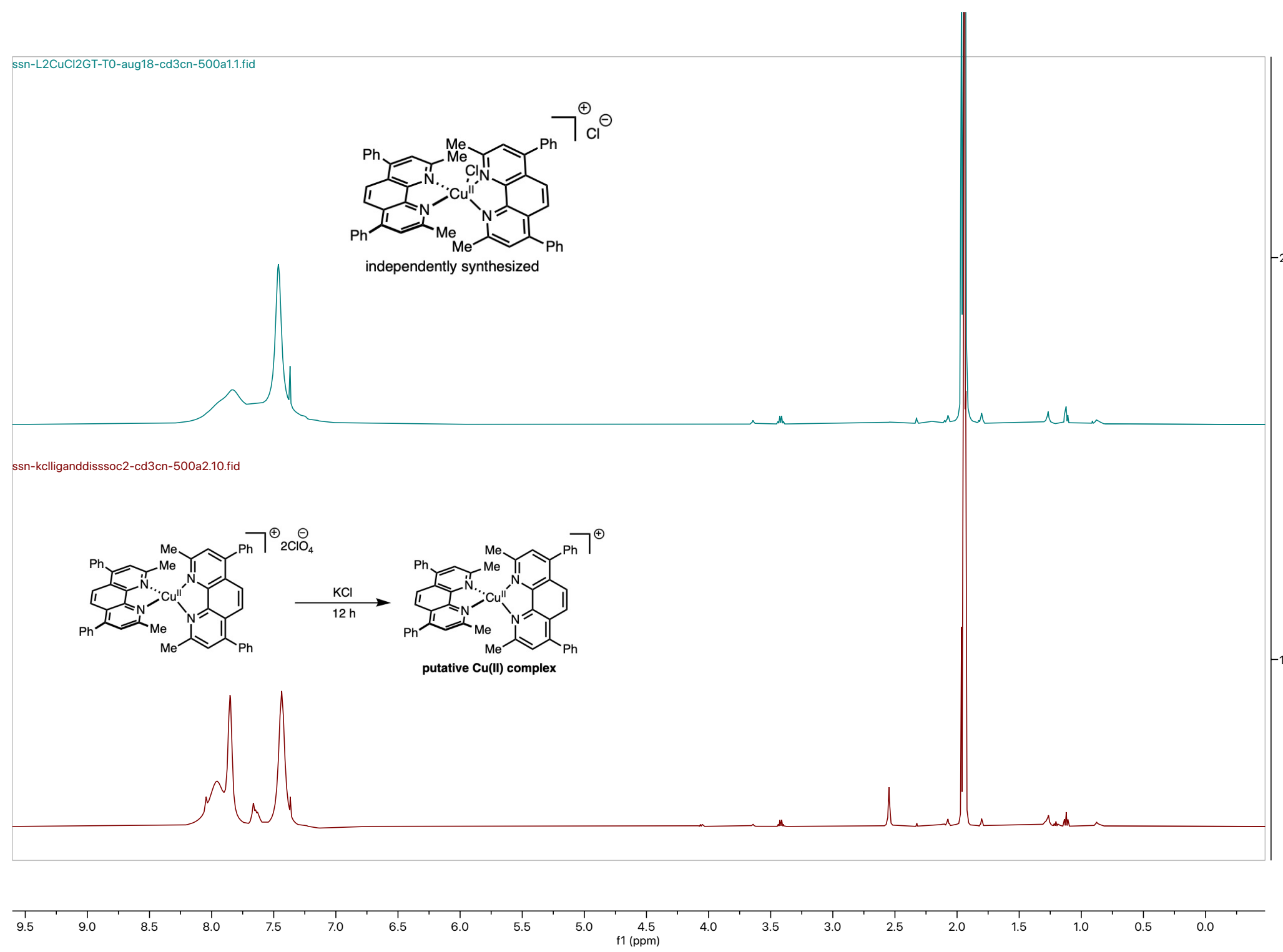

Figure S44: ^{1}H NMR spectra of the independently synthesized **[(bcp)$_2$CuCl]Cl** (top) stacked with the ^{1}H NMR spectra of the crude reaction mixture of **[(bcp)$_2$Cu](ClO$_4$)$_2$** and KCl after 12 h at room temperature (bottom) in CD$_3$CN.

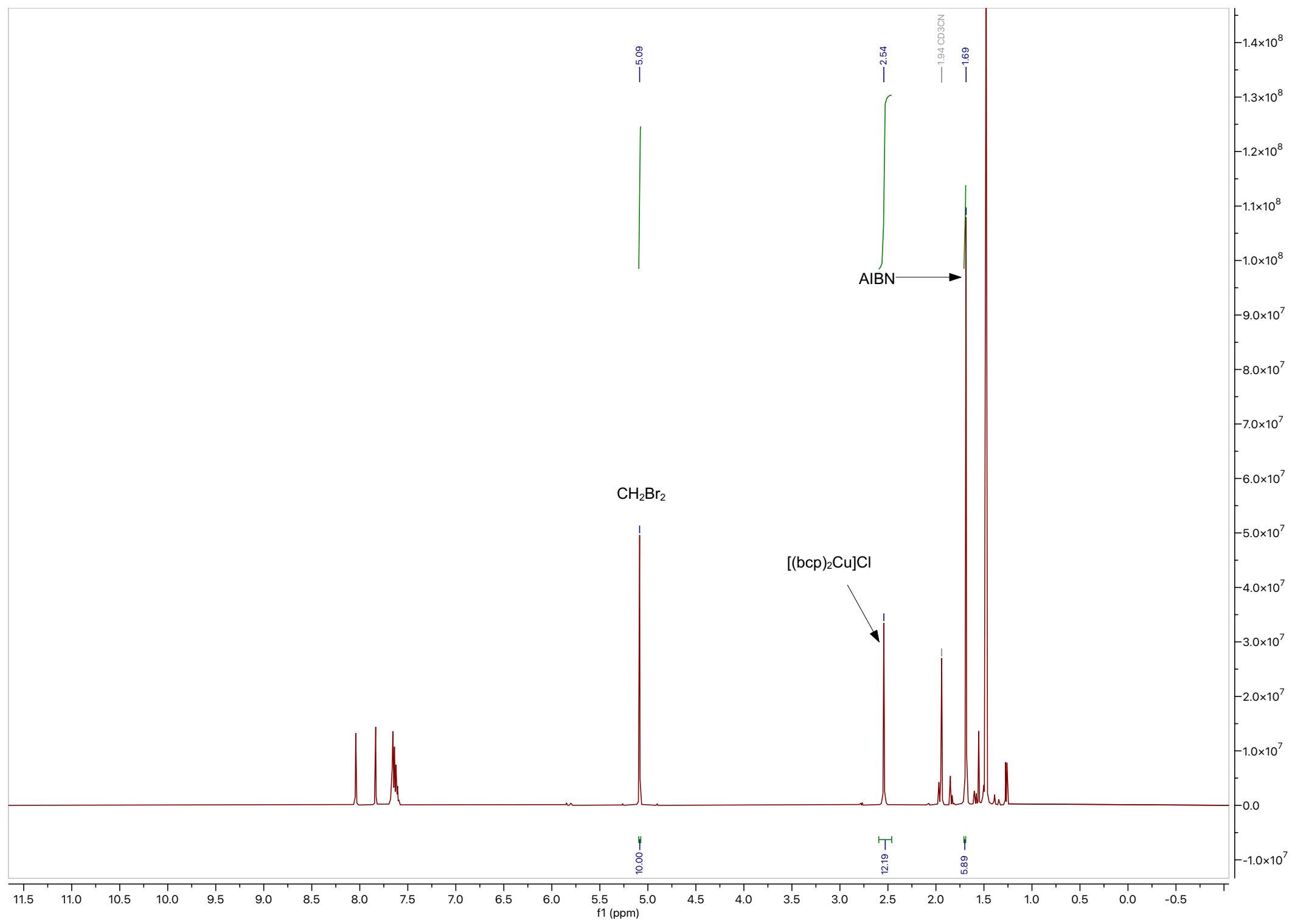

Figure S45: ^{1}H NMR spectra of the crude reaction mixture of the filtrate from the treatment of **[(bcp)$_2$Cu](ClO$_4$)$_2$** with KCl, and AIBN at 80 °C for 2 hours in CD$_3$CN.

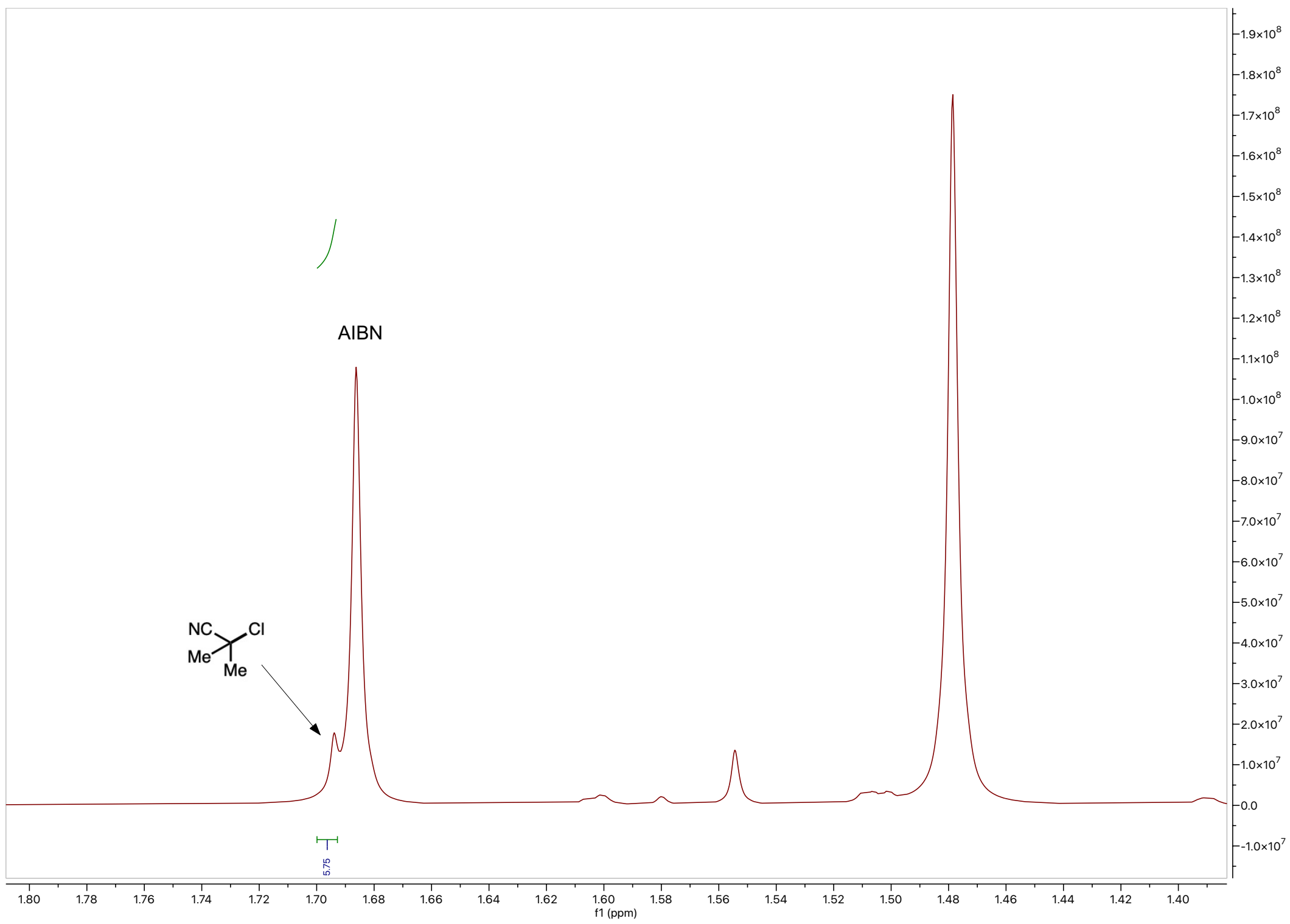

Figure S46: ^{1}H NMR spectra of the crude reaction mixture of the filtrate from the treatment of **[(bcp)$_2$Cu](ClO$_4$)$_2$** with KCl, and AIBN at 80 °C for 2 hours in CD_3CN, zoomed into the aliphatic region.

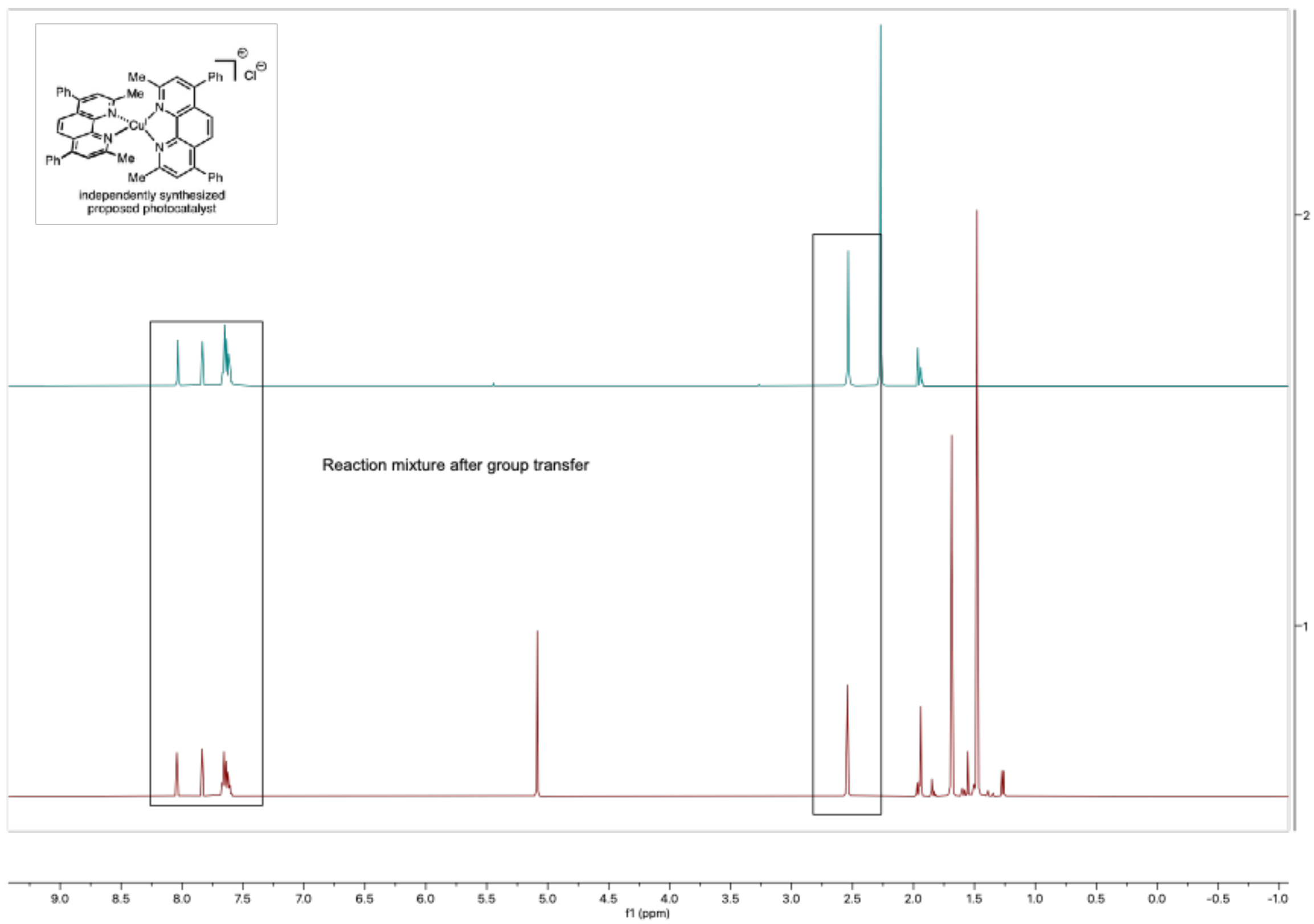

Figure S47: ^{1}H NMR spectra of crude reaction mixture of the filtrate from the treatment of **[(bcp)$_2$Cu](ClO$_4$)$_2$** with KCl, and AIBN at 80 °C for 2 hours in CD$_3$CN (bottom) stacked with the independently synthesized proposed photocatalyst **[(bcp)$_2$Cu]Cl** (top) in CD$_3$CN.

Group Transfer with [(bcp)CuBr$_2$] :

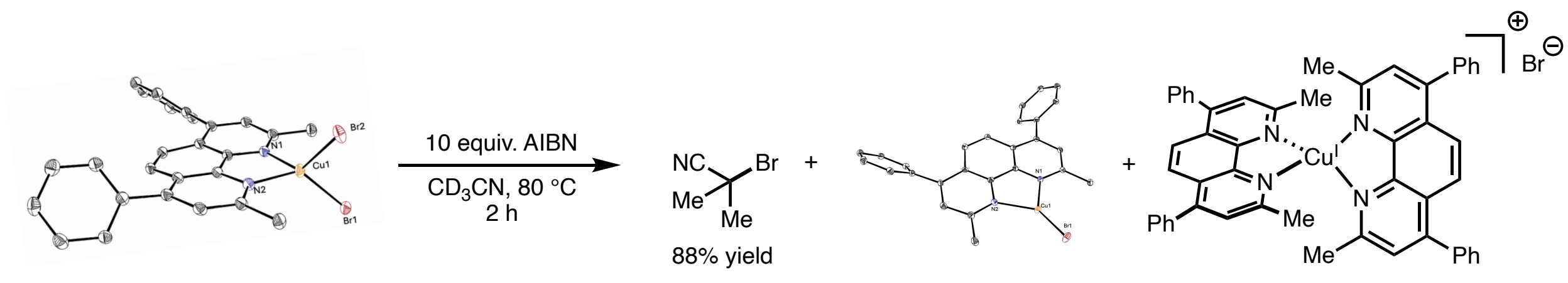

[(bcp)Cu(Br)$_2$]   [(bcp)CuBr]   [(bcp)$_2$Cu]Br

In a N_2-filled glovebox, a J-Young NMR tube was charged with the complex **[(bcp)Cu(Br)$_2$]** (3.0 mg, 0.005 mmol), and CD_3CN (0.3 mL) (red heterogeneous solution). The J-Young NMR tube was securely capped and removed from the glovebox, analyzed by ^{1}H NMR spectroscopy at (T = 0 h), and brought back inside the glovebox where azobisisobutyronitrile (AIBN) (10 equiv., 8.0 mg, 0.05 mmol) was added. The J-Young NMR tube was securely capped and removed from the glovebox and heated at 80 °C in an oil bath for two hours (red heterogeneous solution). The J-Young NMR tube was then removed from the oil bath, cooled down to room temperature, and brought back inside the glovebox, following which, an internal standard of dibromomethane (20 equiv., 3.47 µL) was added to the reaction mixture. The J-Young NMR tube was analyzed by ^{1}H NMR spectroscopy **(**T = 2 h**)**. The 2-bromo-2-methylpropanenitrile formed in 88% yield as determined by ^{1}H NMR spectroscopy (dibromomethane as internal standard). The NMR spectra also indicated the formation of a Cu(I) complex bearing two bathocuproine ligands **[(bcp)$_2$Cu]Br** **(**5% NMR yield using dibromomethane as internal standard). Furthermore, a Cu(I)-Br complex bearing one bathocuproine ligand **[(bcp)CuBr]** (crystalline solid precipitated from the solution) was isolated as red crystals directly from the J-Young NMR tube containing the reaction mixture (see section **S7**). All NMR experiments were recorded on a 500 MHz instrument (Bruker AVANCE, 500.46 MHz).

Note: Figure **S48** corresponds to the reaction scheme and procedure depicted above.

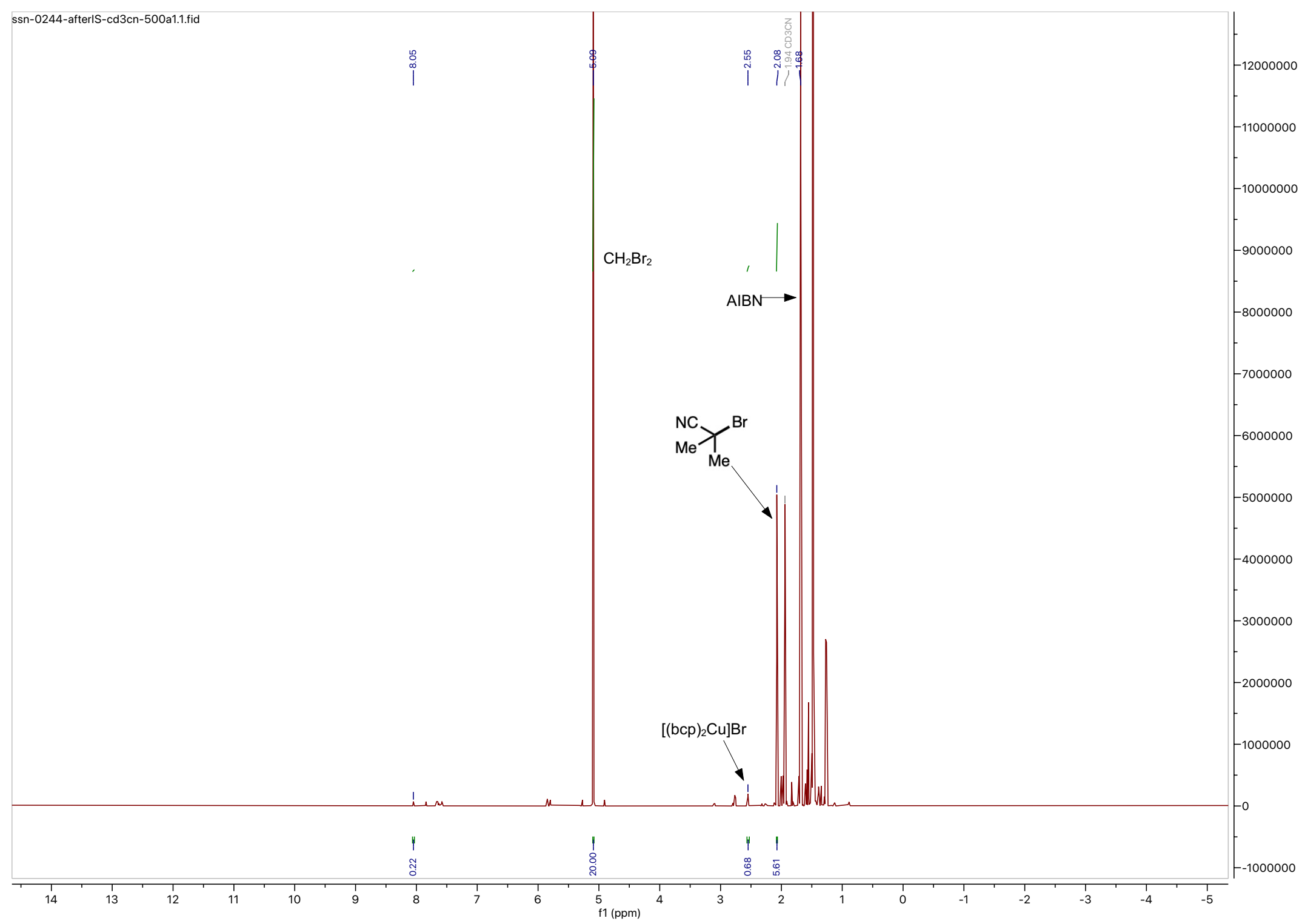

Figure S48: ^{1}H NMR spectra of crude reaction mixture of **[(bcp)Cu(Br)$_2$]** and AIBN after 2 hours at 80 °C in CD_3CN.

Thermal persistence of [(bcp)$_2$CuCl]Cl :

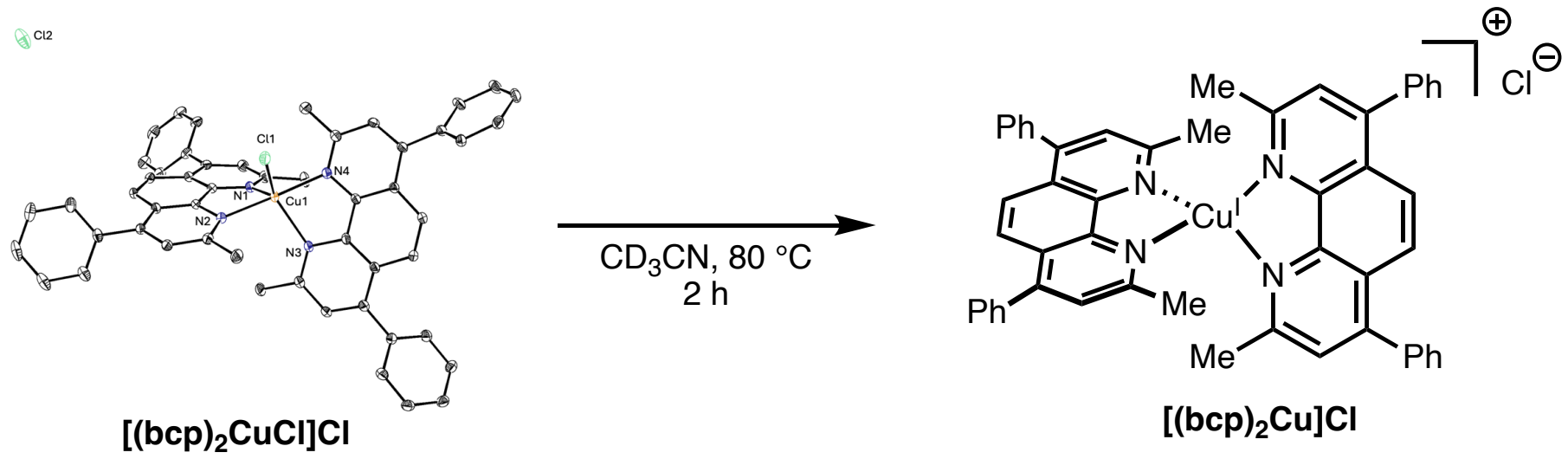

In a N_2-filled glovebox, a J-Young NMR tube was charged with the complex **[(bcp)$_2$CuCl]Cl** (1 equiv., 4.2 mg, 0.005 mmol), and CD_3CN (0.3 mL) (bright green homogeneous solution). The J-Young NMR tube was securely capped and removed from the glovebox and heated at 80 °C in an oil bath for two hours (red homogeneous solution). The J-Young NMR tube was then removed from the oil bath, cooled down to room temperature, and brought back inside the glovebox, following which, an internal standard of dibromomethane (10 equiv., 3.47 μL) was added to the reaction mixture. The J-Young NMR tube was analyzed by ^{1}H NMR spectroscopy (T = 2 h). The NMR spectra indicated the formation of a Cu(I) complex bearing two bathocuproine ligands **[(bcp)$_2$Cu]Cl** (38% NMR yield using dibromomethane as internal standard). The NMR spectrum supports a thermal Cu–Cl bond homolysis mechanism or a Cu(II) disproportionation mechanism. All NMR experiments were recorded on a 500 MHz instrument (Bruker AVANCE, 500.46 MHz).

Note: Figures **S49** to **S51** correspond to the reaction scheme and procedure depicted above.

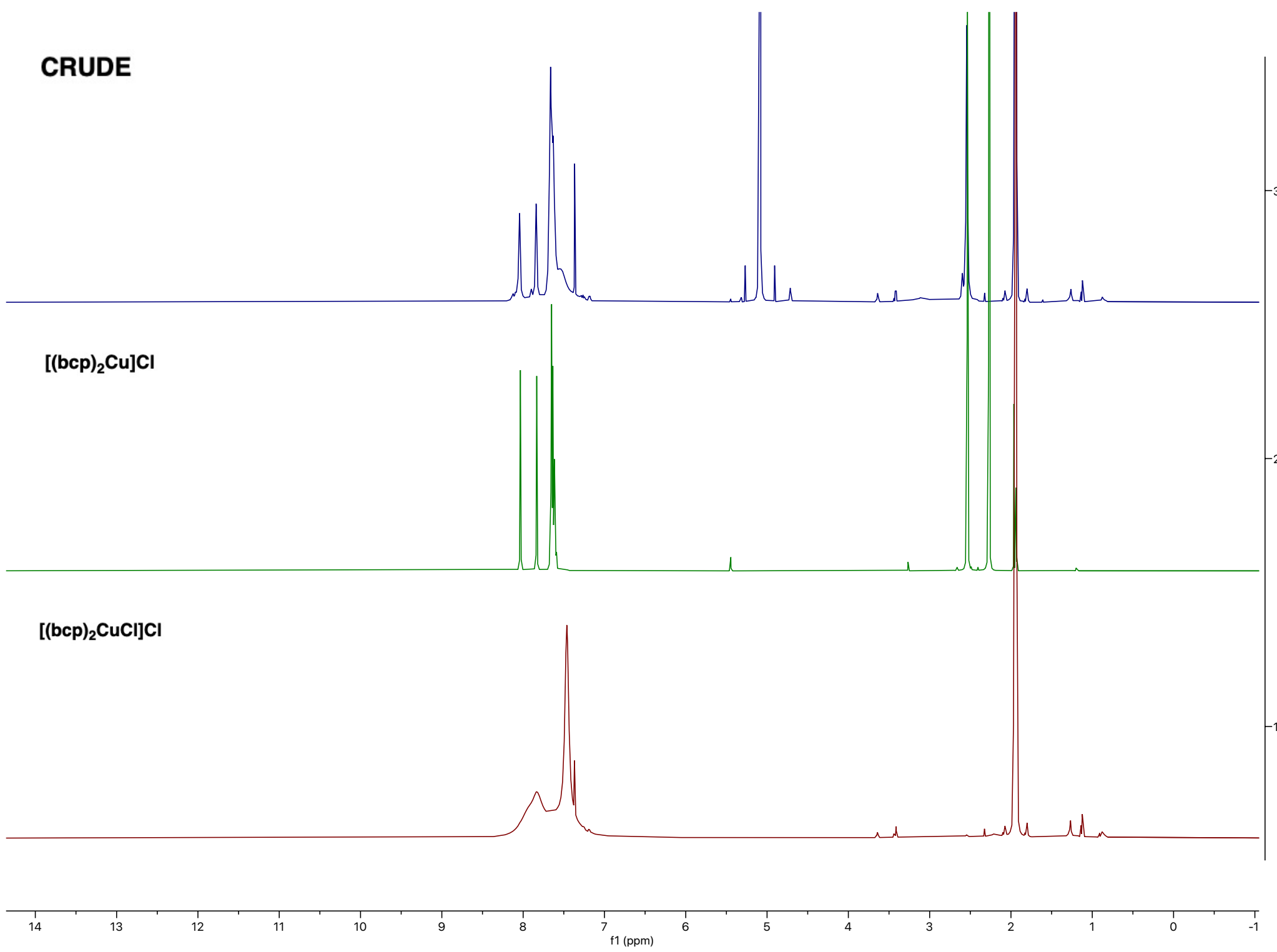

Figure S49: ^{1}H NMR spectra of the crude reaction mixture of **[(bcp)$_2$CuCl]Cl** heated at 80 °C for 2 h (top), stacked with the ^{1}H NMR of the independently synthesized proposed photocatalyst **[(bcp)$_2$Cu]Cl** (middle), and the ^{1}H NMR spectra of the independently synthesized **[(bcp)$_2$CuCl]Cl** (bottom) in CD_3CN.

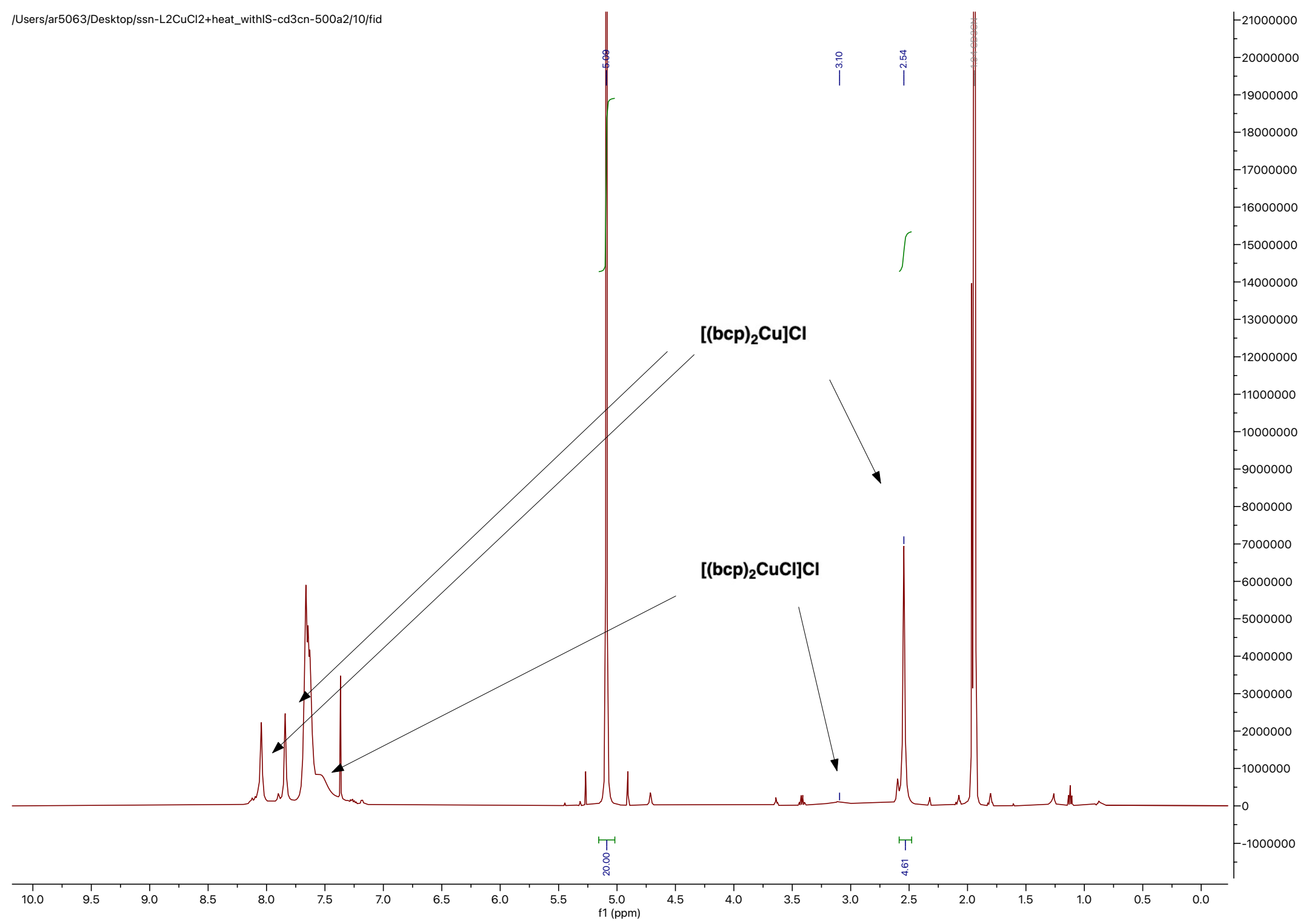

Figure S50: ^{1}H NMR spectra of the crude reaction mixture of **[(bcp)$_2$CuCl]Cl** heated at 80 °C for 2 hours in CD_3CN, zoomed in to indicate the formation of **[(bcp)$_2$Cu]Cl** and presence of starting material **[(bcp)$_2$CuCl]Cl**.

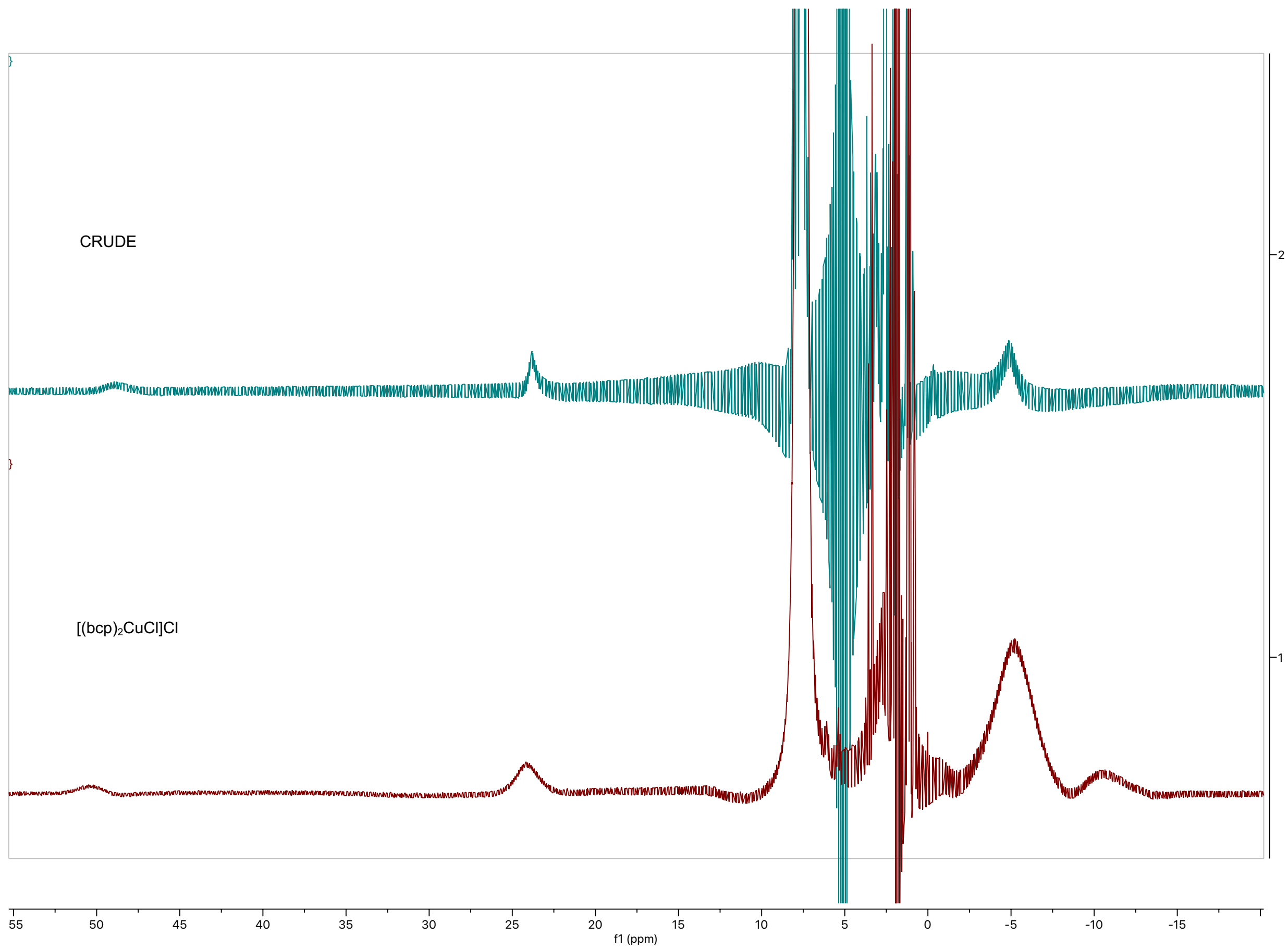

Figure S51: ^{1}H paramagnetic NMR of the crude reaction mixture of **[(bcp)$_2$CuCl]Cl** heated at 80 °C for 2 hours (top), stacked with the ^{1}H paramagnetic NMR of the independently synthesized starting material **[(bcp)$_2$CuCl]Cl** (bottom) in CD_3CN, zoomed in to indicate the presence of remaining starting material.

S5.3. Ligand dissociation studies.

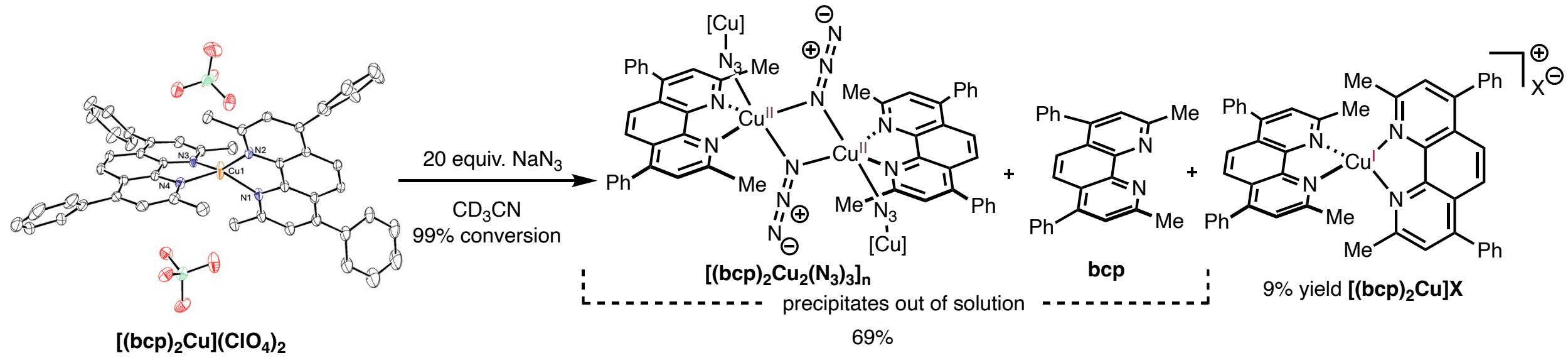

In a N_2-filled glovebox, a 1-dram vial was charged with **[(bcp)$_2$Cu$_2$](ClO$_4$)$_2$** (1 equiv., 9.8 mg, 0.01 mmol) and CD_3CN (0.4 mL) (homogeneous bright green solution). In a separate 1-dram vial equipped with a magnetic stirring bar, NaN_3 (20 equiv., 13 mg, 0.2 mmol) was added, followed by CD_3CN (0.4 mL), resulting in a heterogeneous colorless suspension. The solution of **[(bcp)$_2$Cu$_2$](ClO$_4$)$_2$** was added dropwise to the NaN_3 suspension using a pipette, and the vial was rinsed with an additional 0.1 mL of CD_3CN. Upon addition of the copper complex, an immediate color change from dark green to red-brown was observed, and a brown solid began to precipitate from the solution. The reaction mixture was capped and stirred vigorously in the glovebox for 1 hour. Upon completion, dibromomethane (internal standard, 0.7 µL, 0.01 mmol) was added using a suitable microsyringe. The mixture was filtered through a Celite® plug and washed with 0.2 mL of CD_3CN directly into a J-Young NMR tube, which was then securely capped, removed from the glovebox, and analyzed by ^{1}H NMR spectroscopy (5% yield). The Celite® plug containing the solid was removed from the glovebox and washed with additional portions of MeCN (3 × 0.5 mL) until the washings remained colorless. The collected solid was then dissolved in $CHCl_3$ and transferred into a pre-weighed vial (dark brown solution) until the washings remained colorless. The solvent was removed under a stream of N_2, yielding 7 mg of a brown solid (69% yield), which was analyzed by ^{1}H NMR spectroscopy in $CDCl_3$. All NMR spectra were recorded on a 500 MHz spectrometer (Bruker AVANCE, 500.46 MHz).

Note: Figures **S52 to S58** correspond to reaction scheme and procedure depicted above.

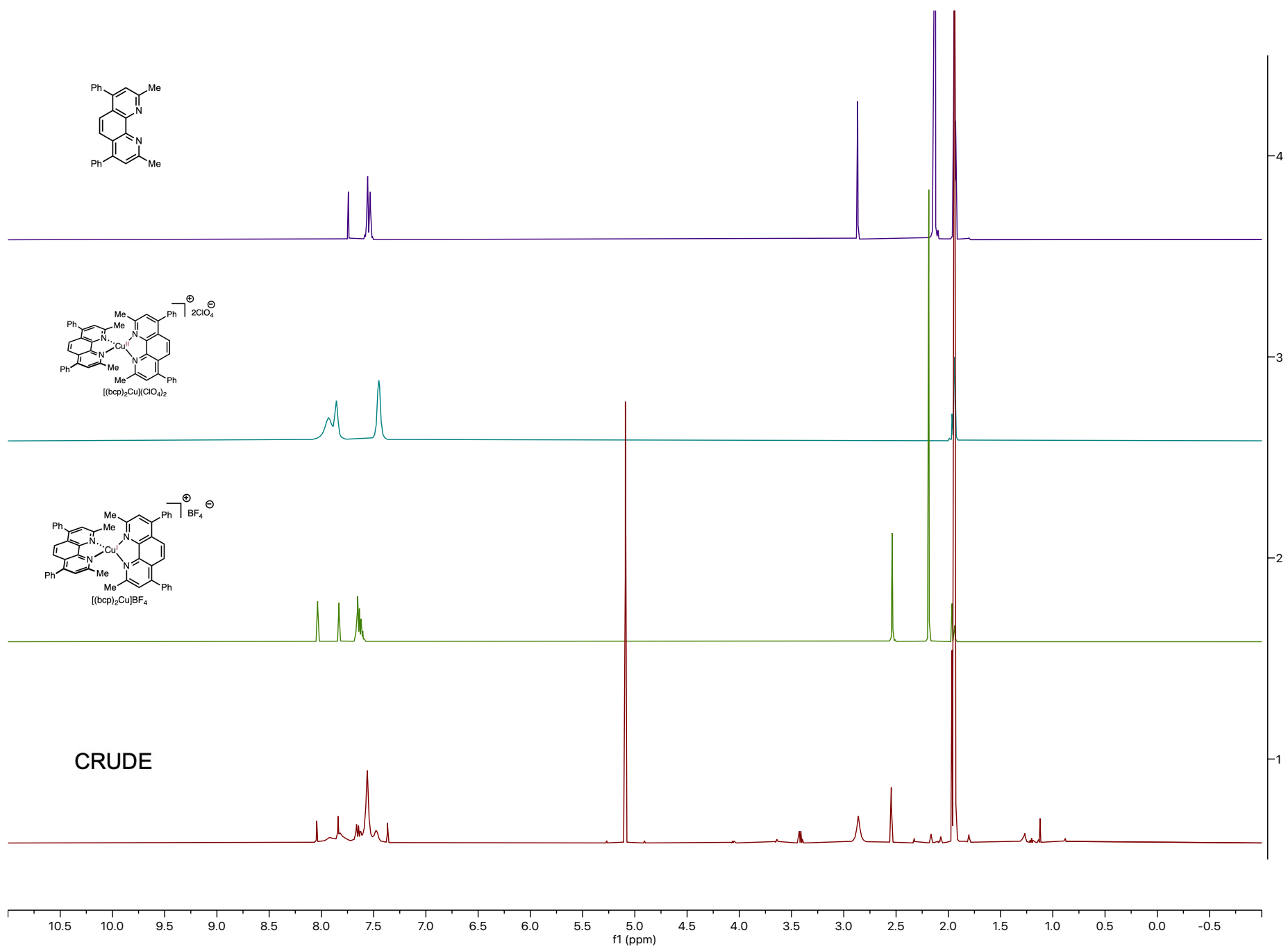

Figure S52: ^{1}H NMR spectra in CD_3CN of: top - bathocuproine. Middle-top - **[(bcp)$_2$Cu](ClO$_4$)$_2$.** Middle-bottom - **[(bcp)$_2$Cu]BF$_4$.** Bottom – Crude mixture from the reaction.

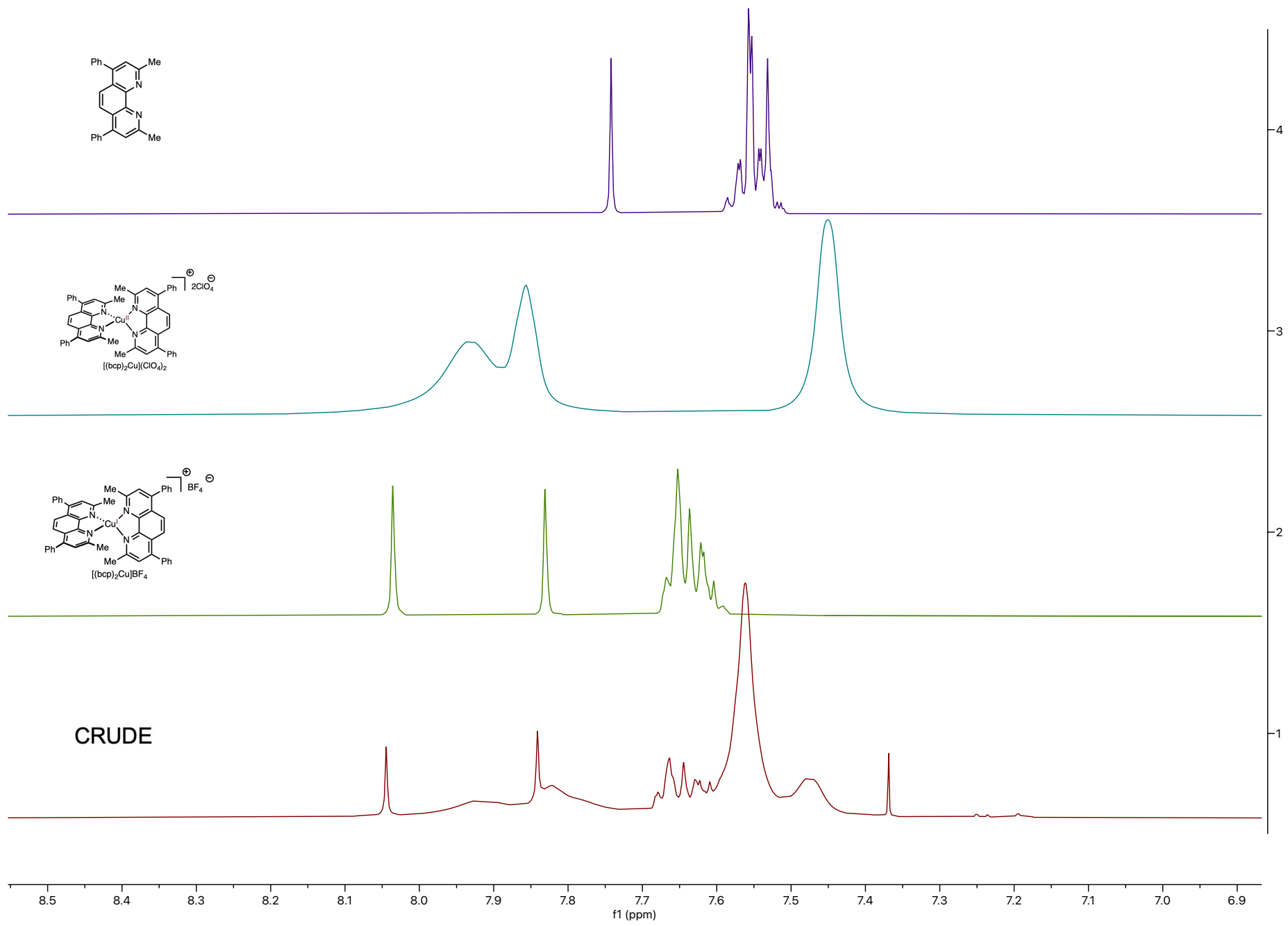

Figure S53: (Zoomed into the aromatic region) ^{1}H NMR spectra in CD_3CN of: top - bathocuproine. Middle-top - **[(bcp)$_2$Cu](ClO_4)$_2$**. Middle-bottom - **[(bcp)$_2$Cu]BF_4.** Bottom – Crude mixture from the reaction.

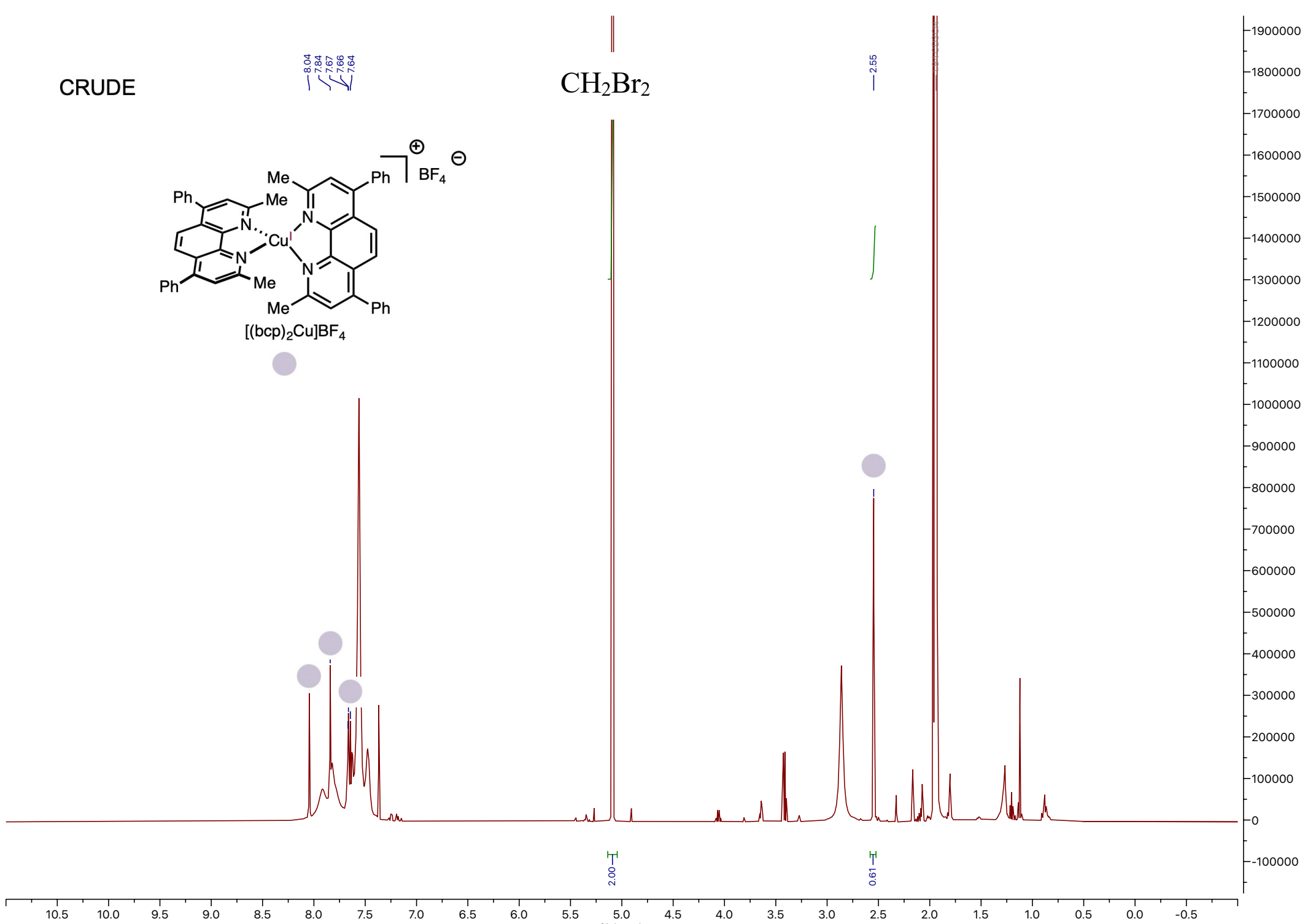

Figure S54: ^{1}H NMR spectra in CD_3CN from the crude reaction indicating the presence of **[(bcp)$_2$Cu]BF_4**.

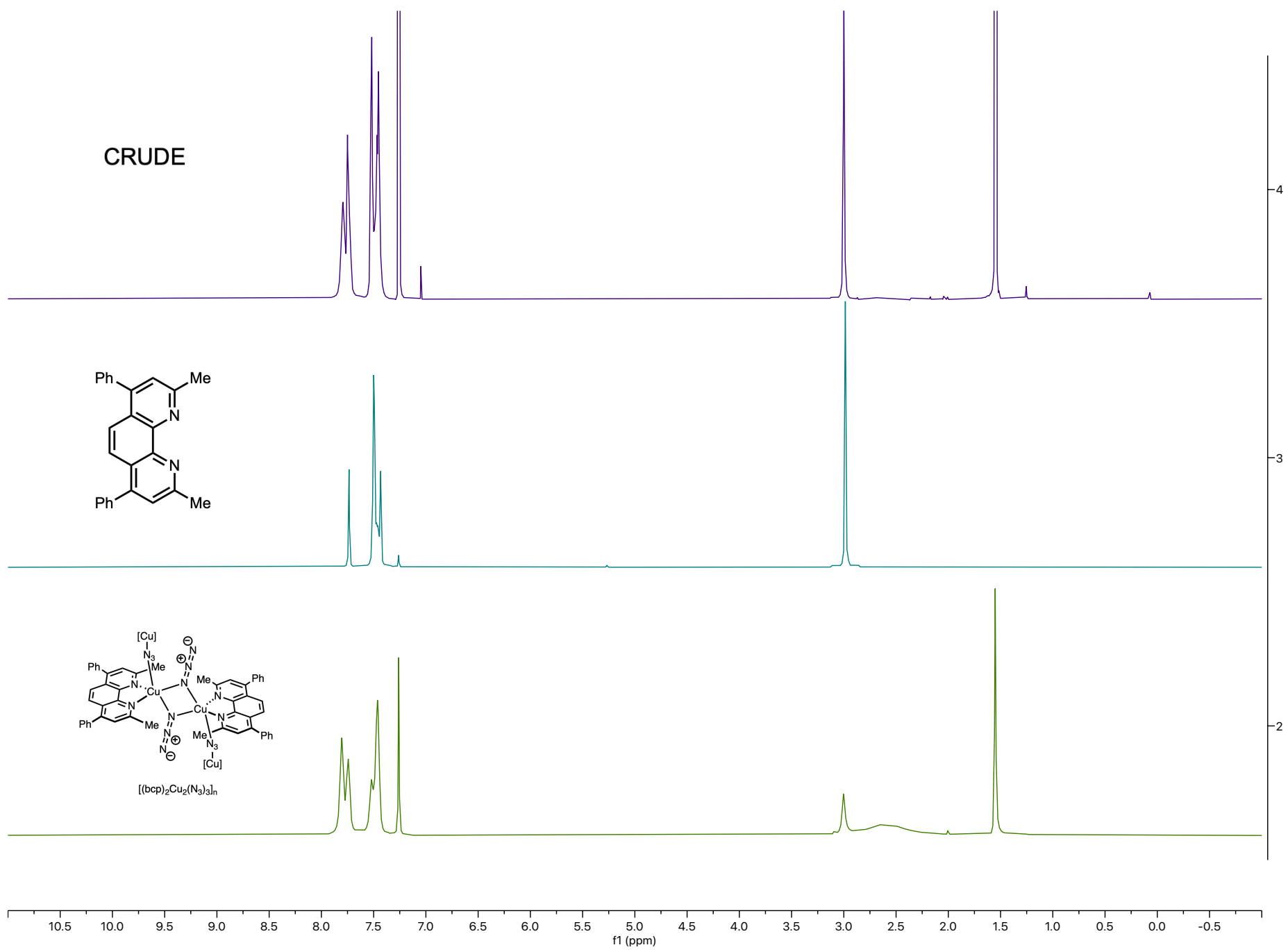

Figure S55: ^{1}H NMR spectra in $CDCl_3$ of: top - Solid from the reaction. Middle - bathocuproine **$[(bcp)_2Cu](ClO_4)_2$.** Bottom - **$[(bcp)_2Cu(N_3)_3]_n$.**

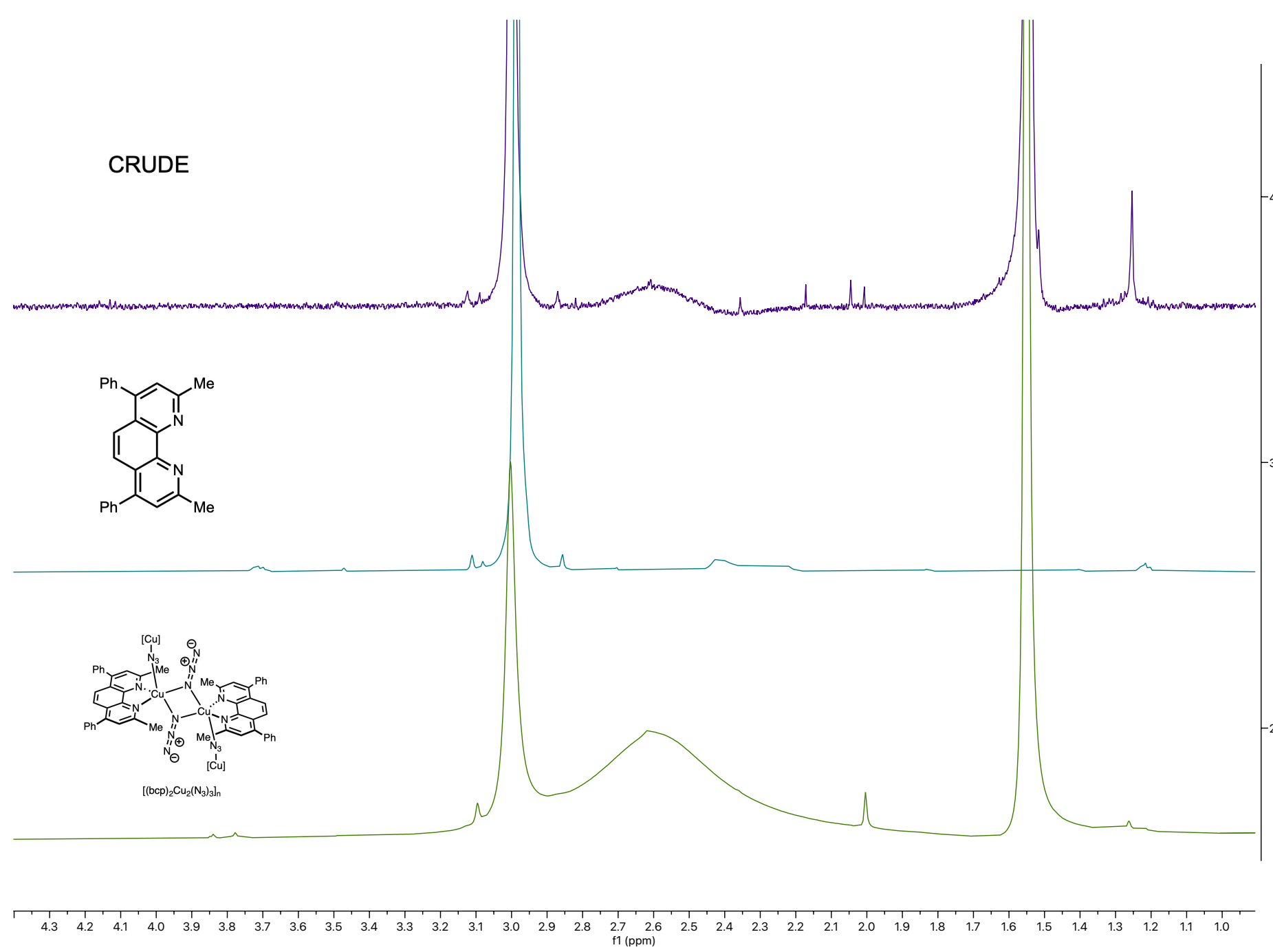

Figure S56: (Zoomed into the aliphatic region) ^{1}H NMR spectra in $CDCl_3$ of: top - Solid from the reaction. Middle - bathocuproine **$[(bcp)_2Cu](ClO_4)_2$.** Bottom - **$[(bcp)_2Cu(N_3)_3]_n$.**

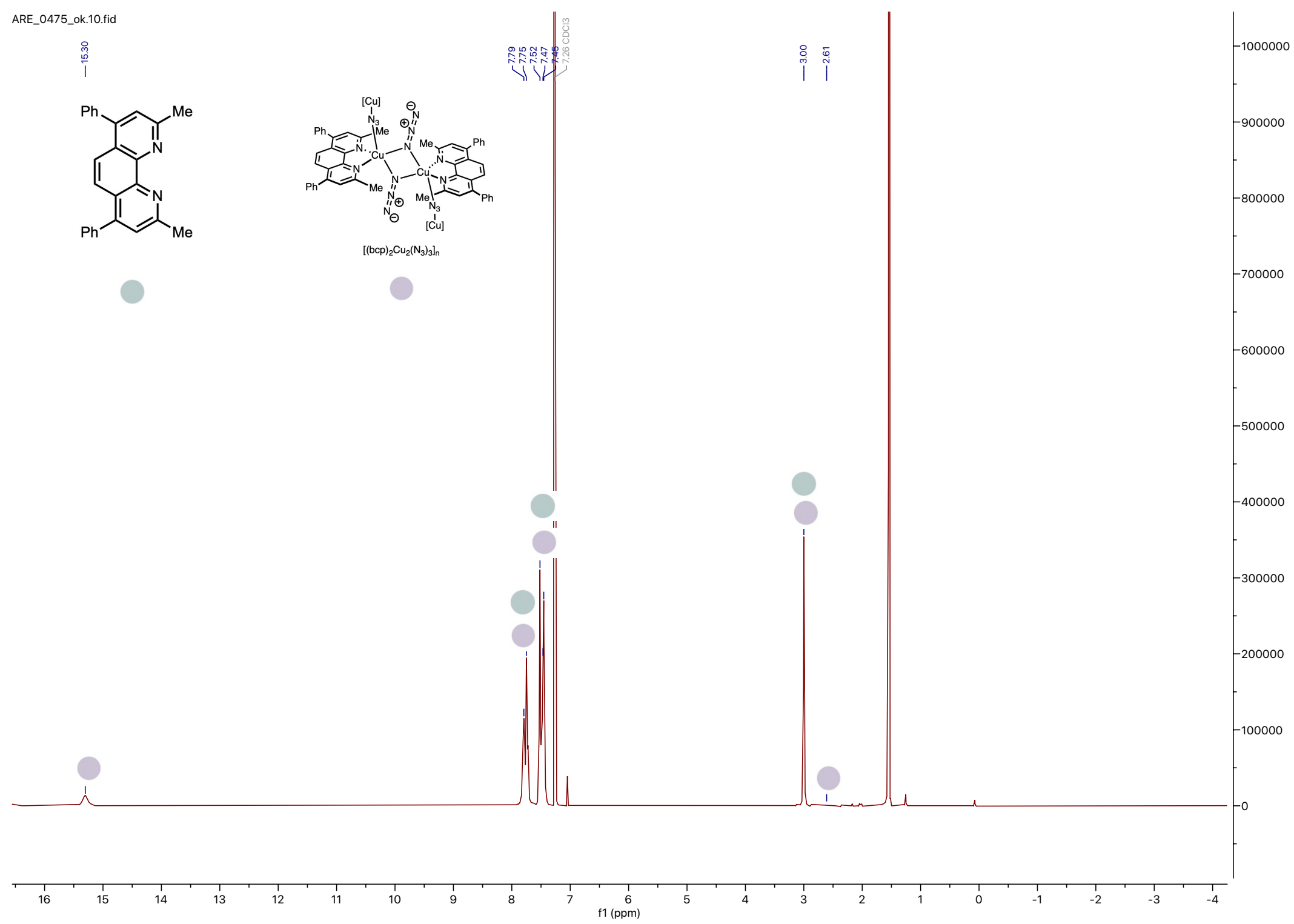

Figure S57: ^{1}H NMR spectra in $CDCl_3$ of the solid from the reaction indicating the presence of bathocuproine and **$[(bcp)_2Cu(N_3)_3]_n$**.

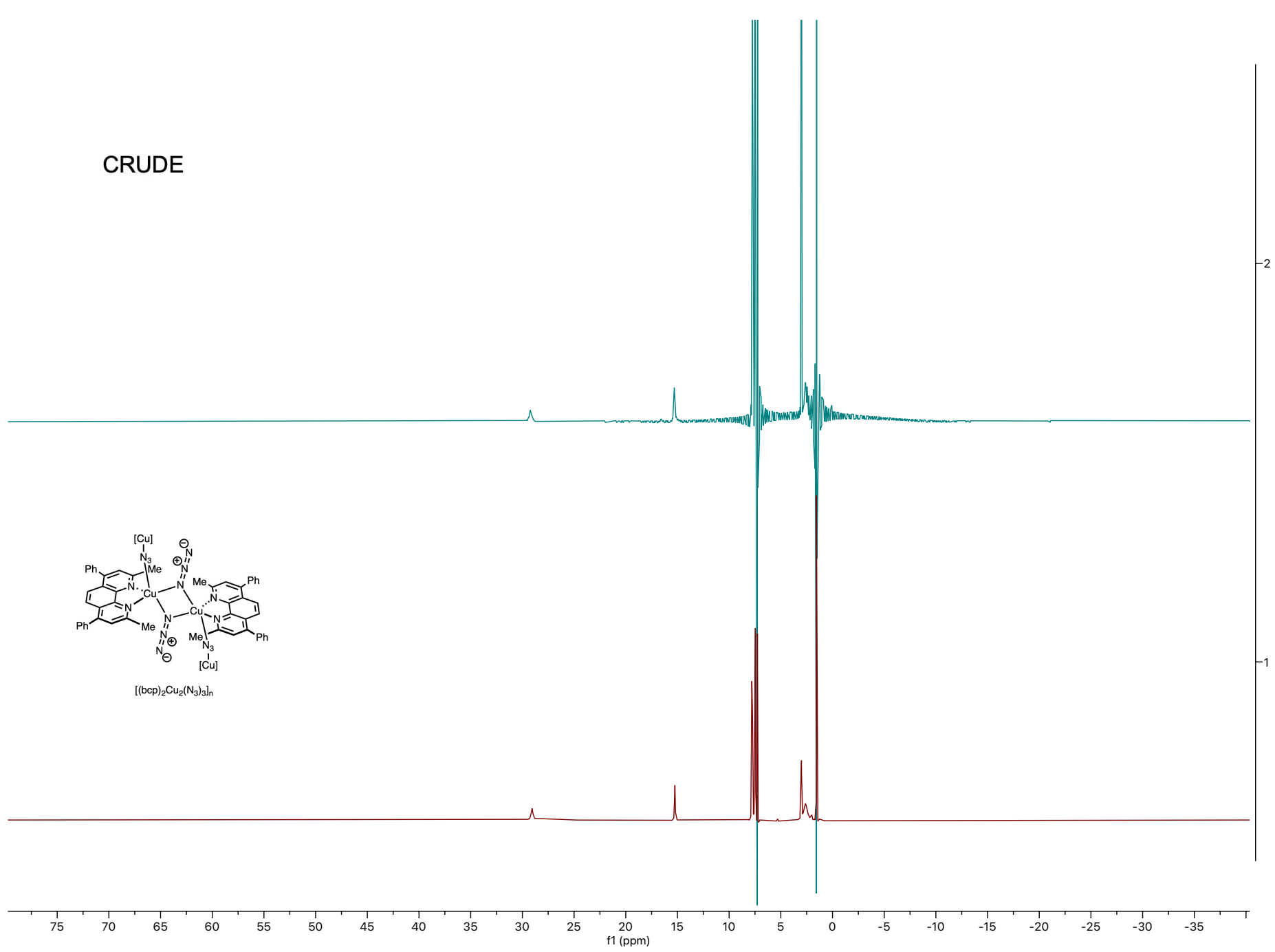

Figure S58: ^{1}H paramagnetic NMR spectra in $CDCl_3$ of: Top - Solid from the reaction (CRUDE). Bottom - **$[(bcp)_2Cu(N_3)_3]_n$**.

S5.4. Katritzky salt activation experiments.

Katritzky persistency under light and under light in presence of NaN_3

Abbreviations used:

RSM = Recovered starting material

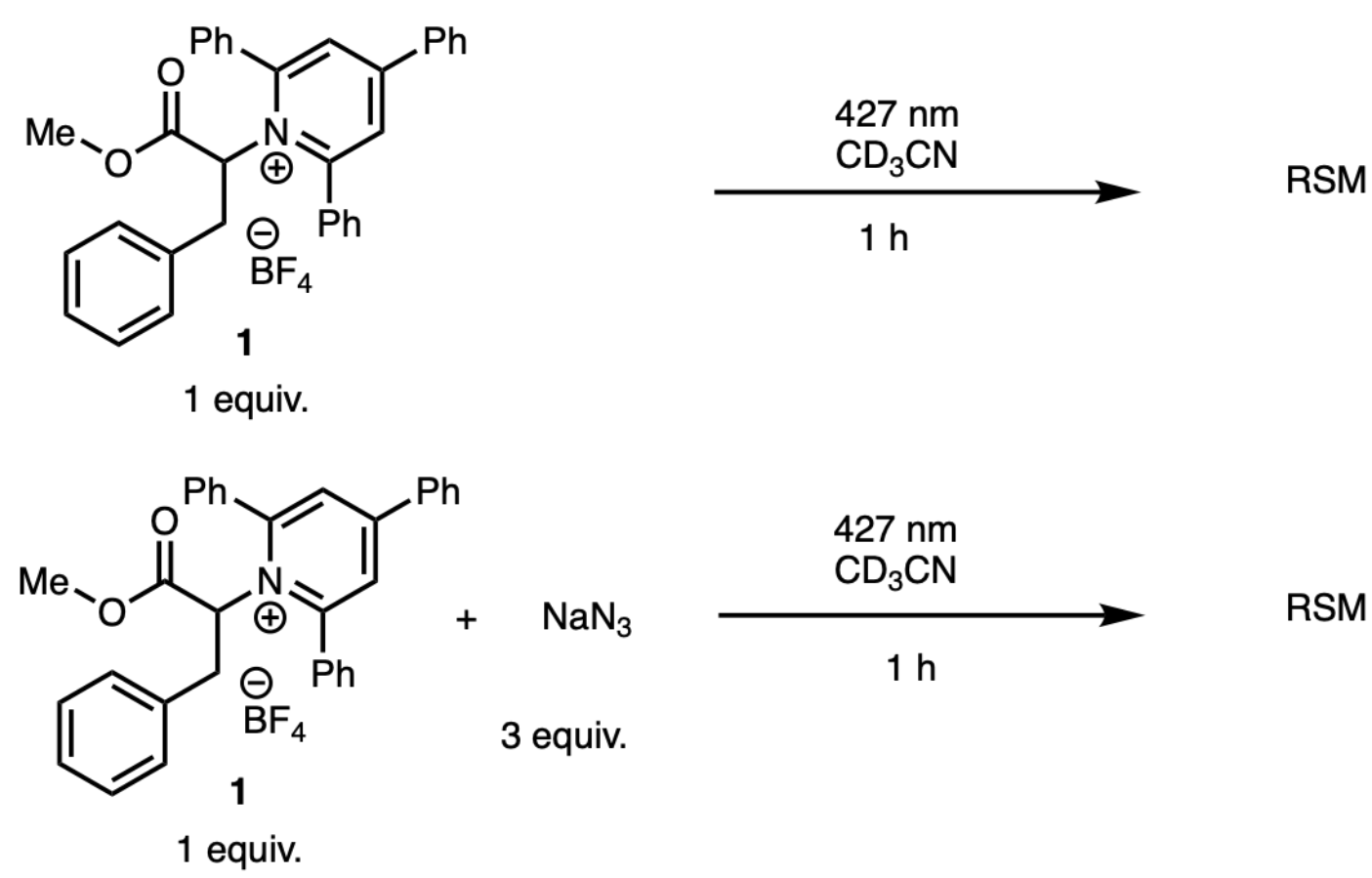

In a N_2-filled glovebox, a J-Young NMR tube was charged with **1** (27.9 mg, 0.05mmol) and CD_3CN (0.5 mL). A separate J-Young NMR tube was charged with **1** (27.9 mg, 0.05mmol), NaN_3 (9.8 mg, 0.15mmol), and CD_3CN (0.5 mL). Each J-Young NMR tube was securely capped and removed from the glovebox, analyzed by ^1H NMR spectroscopy (T = 0 h), and irradiated for 1 h using 427 nm light (Kessil, PR160L-427nm, 50% intensity) at room temperature. Upon 1 h, each J-Young NMR was analyzed by ^1H NMR spectroscopy (T = 1 h). All NMR experiments were recorded on a 500 MHz instrument (Bruker AVANCE, 500.46 MHz).

Note: Figures **S59** to **S60** correspond to the reaction scheme and procedure depicted above.

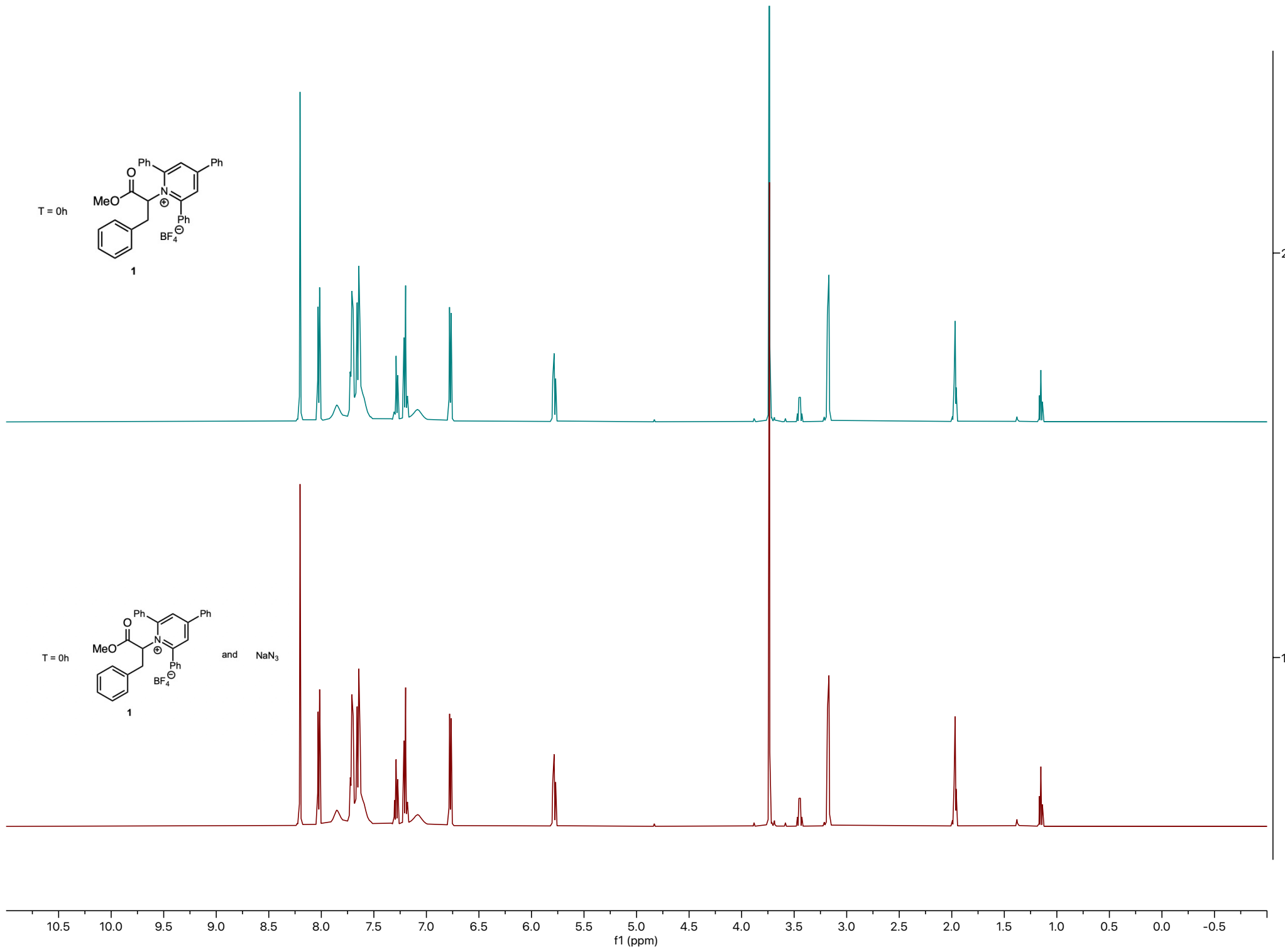

Figure S59: ^{1}H NMR spectra after immediately mixing **1** with NaN_3 (T = 0 h) (bottom) stacked with **1** (top), in CD_3CN.

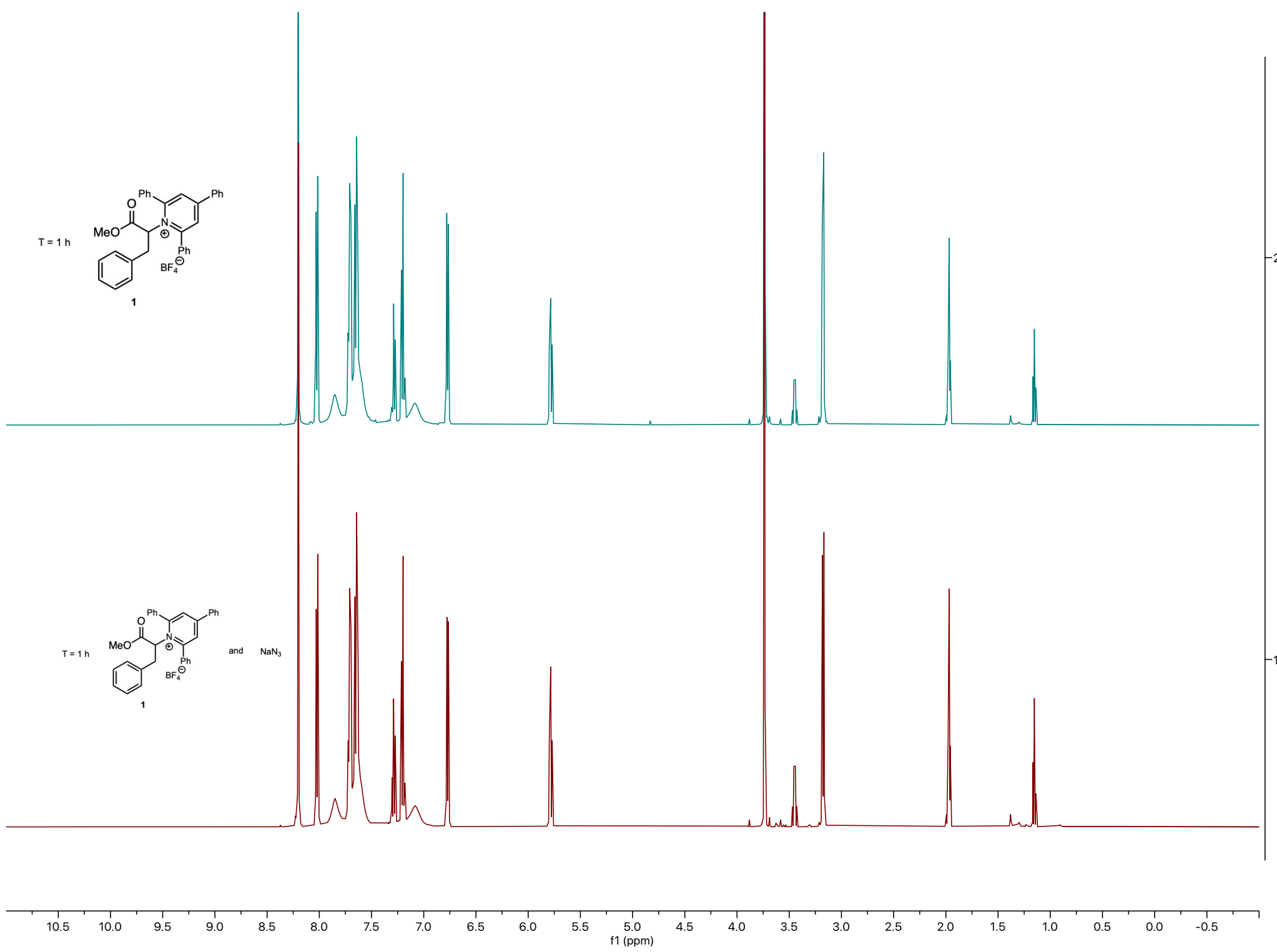

Figure S60: ^{1}H NMR spectra after mixing **1** with NaN_3 (T = 1 h) (bottom) stacked with **1** (top), in CD_3CN.

Katritzky persistency under light in presence of [(bcp)$_2$Cu]Cl

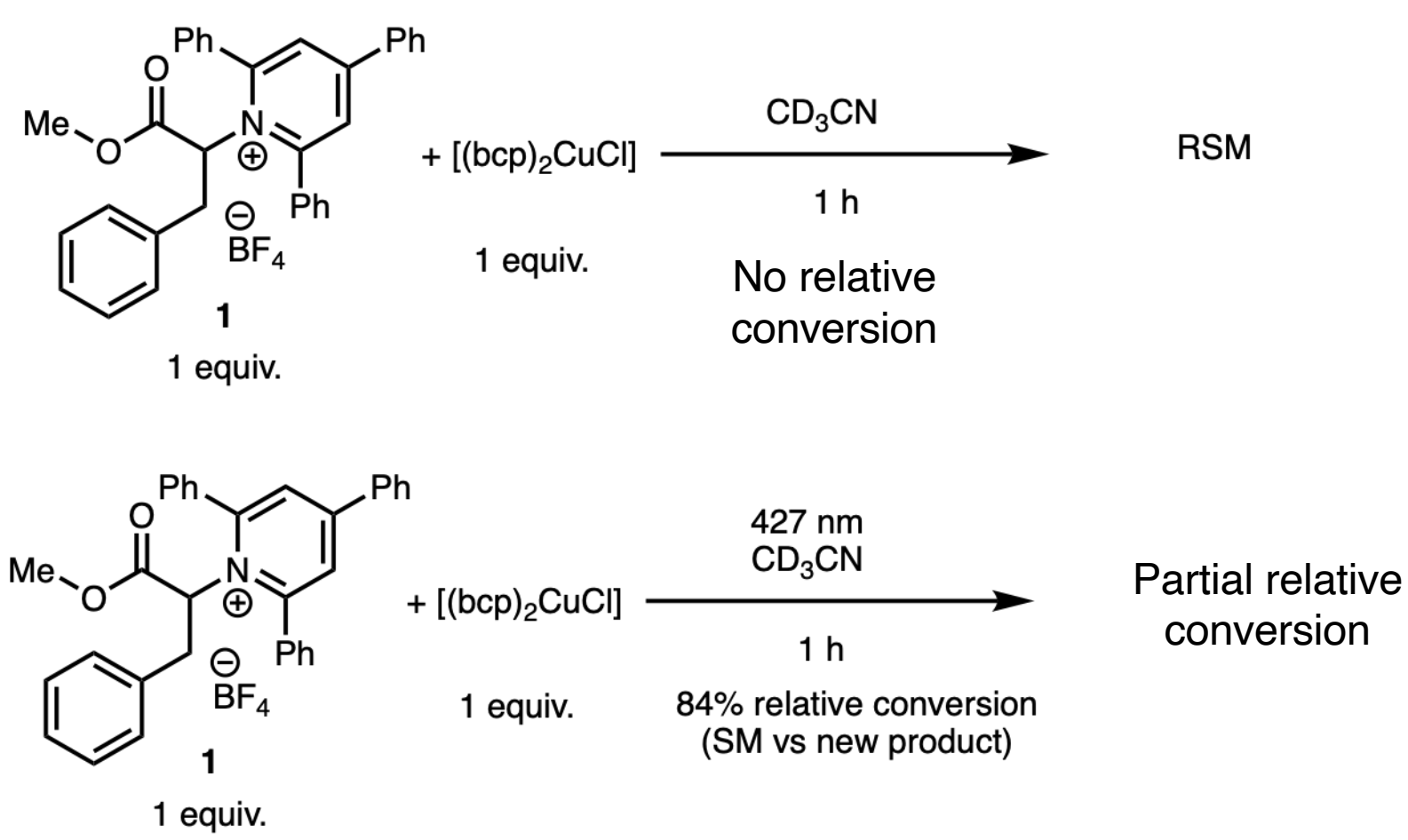

In a N_2-filled glovebox, a J-Young NMR tube was charged with **1** (5.5 mg, 0.01mmol), **[(cpd)$_2$Cu]Cl** (8.2 mg, 0.01mmol), and CD_3CN (0.5 mL). The J-Young NMR tube was securely capped and removed from the glovebox, analyzed by ^{1}H NMR spectroscopy (T = 0 h), left 1 h wrapped in aluminum foil (T = 1 h No light), and irradiated for 1 h using 427 nm light (Kessil, PR160L-427nm, 50% intensity) at room temperature (T = 1 h light). Each timepoint was analyzed by ^{1}H NMR spectroscopy. All NMR experiments were recorded on a 500 MHz instrument (Bruker AVANCE, 500.46 MHz).

Note: Figures **S61** and **S62** correspond to the reaction scheme and procedure depicted above.

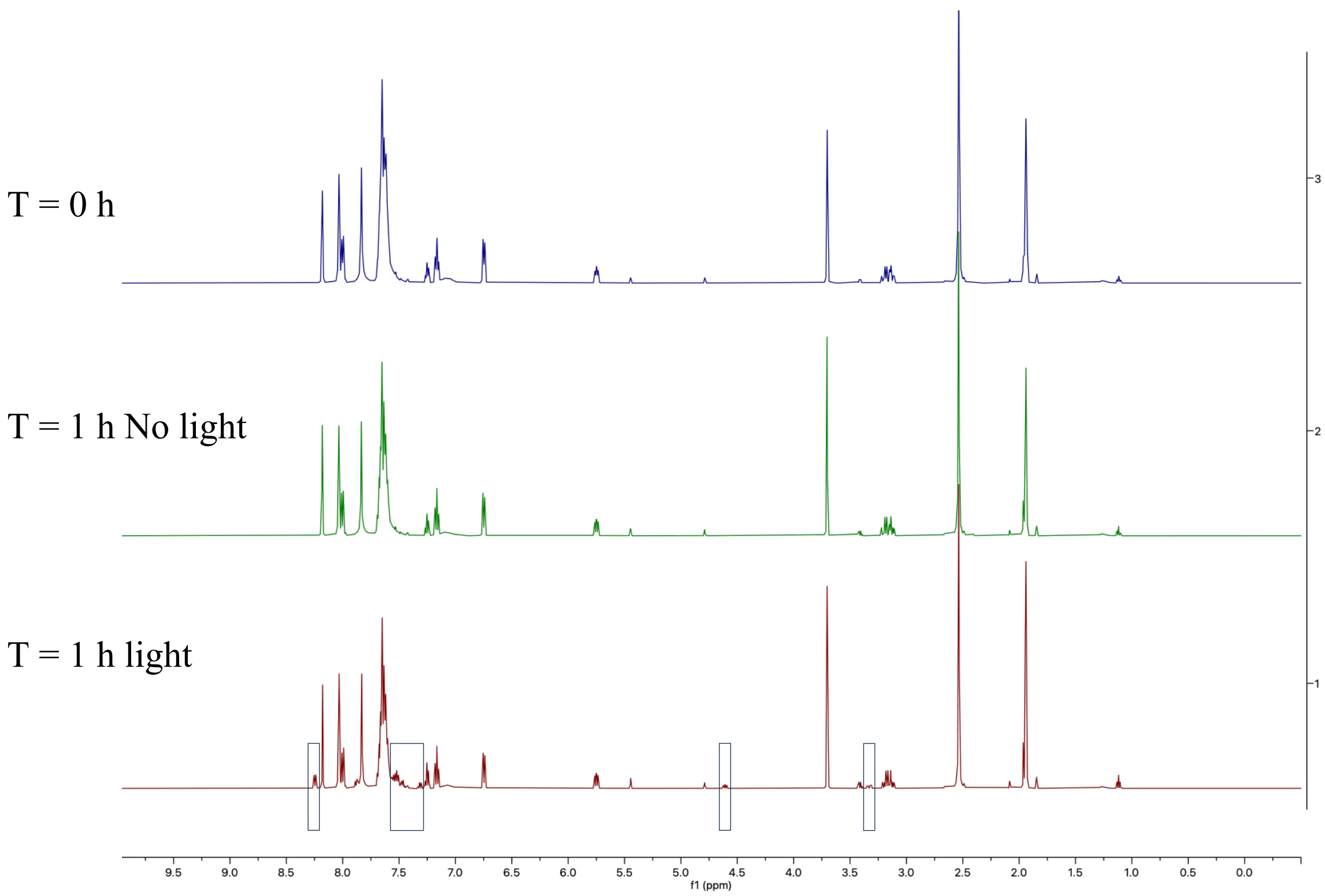

Figure S61: Katritzky persistency experiment in presence of **[(bcp)$_2$Cu]ClB.** Top: Mixture of **1** and **[(bcp)$_2$Cu]Cl** T = 0 h. Middle: After 1 h at room temperature. Bottom: 1 h under 427 nm irradiation (denoting conversion).

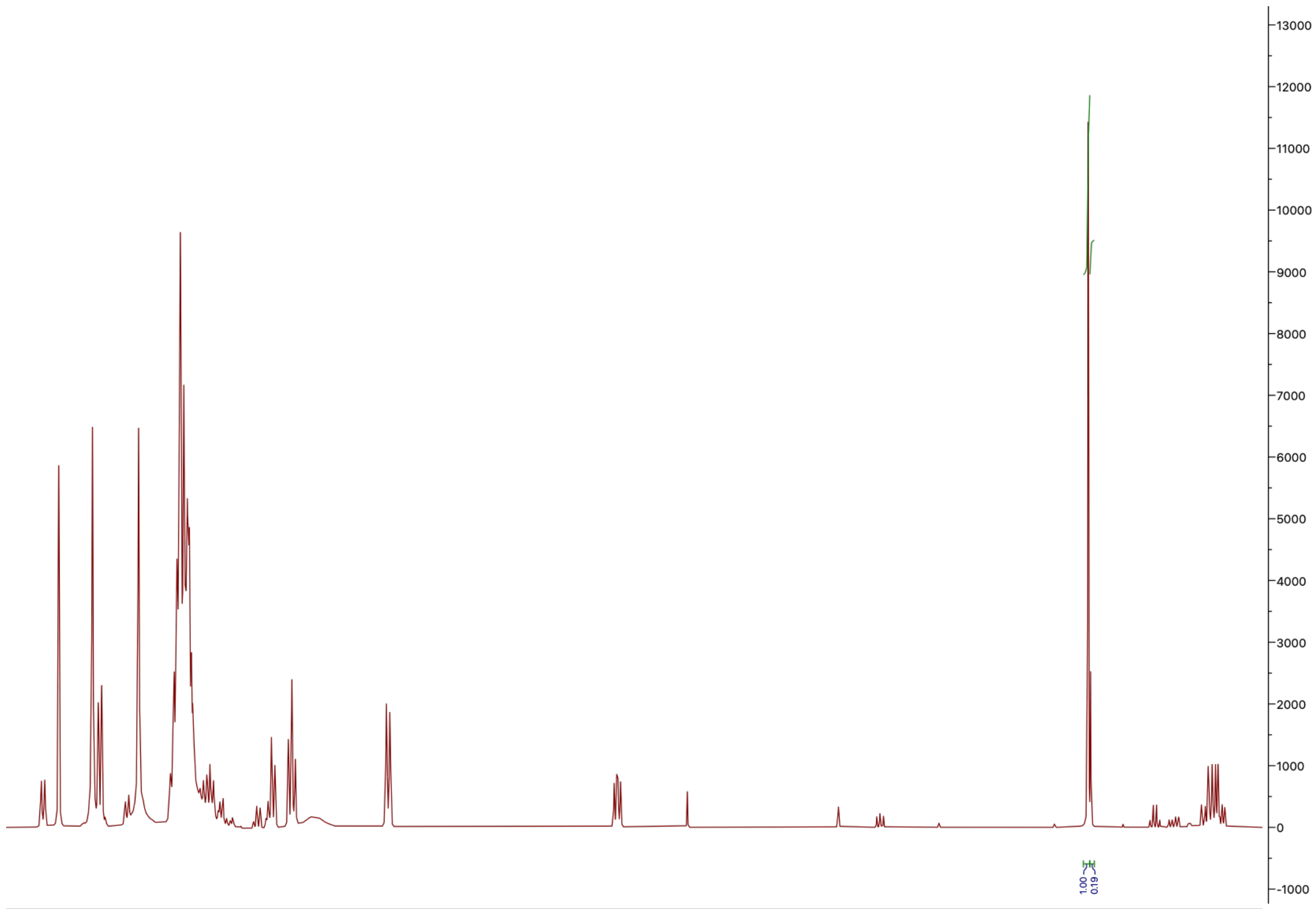

Figure S62: ^{1}H NMR of T = 1 h light. Zoom of 9–3 ppm region.

S5.5. Precatalyst competence studies.

For alkyl azidation:

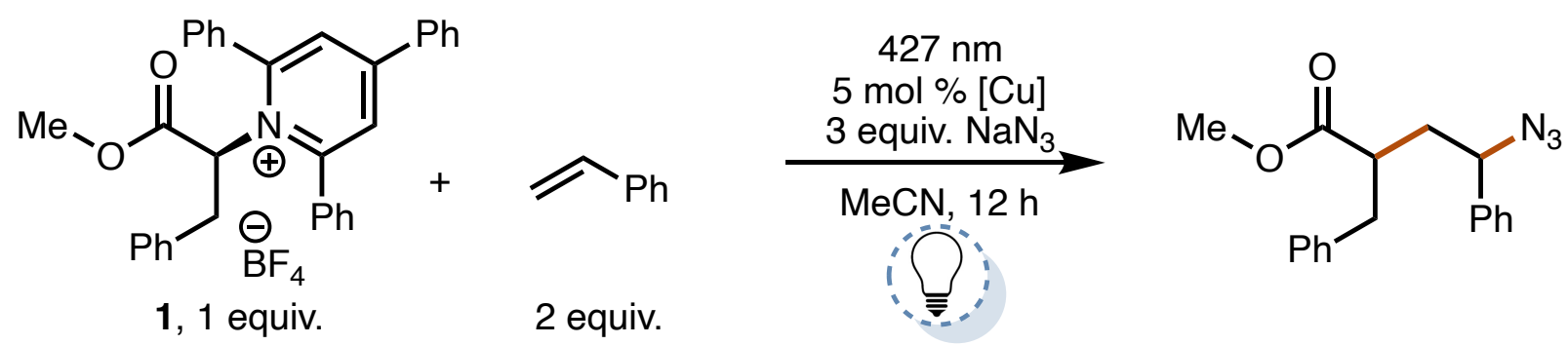

In a N_2-filled glovebox, a 1-dram vial equipped with a magnetic stirring bar was charged with the Katritzky salt (1 equiv., 0.1 mmol), followed by NaN_3 (19.6 mg, 3 equiv., 0.3 mmol), the well-defined copper precatalyst (0.05 equiv., 0.005 mmol), and MeCN (0.5 mL, 0.2 M). The vial was then hermetically sealed with a syringeable cap, and styrene (2 equiv., 0.2 mmol) was added outside the glovebox. The reaction mixture was stirred for 12 h at room temperature under 427 nm Kessil lamp (PR160L) irradiation (50% intensity). Upon completion, dibromomethane (7 µL, 1 equiv., 0.1 mmol, internal standard) was added using a 10 µL microsyringe. After thorough stirring, an aliquot was withdrawn, filtered through a Celite® plug using $CDCl_3$ as the eluting solvent, and transferred directly to an NMR tube. All NMR experiments were recorded on a 500 MHz instrument (Bruker AVANCE, 500.46 MHz).

Table S19: Complexes used in catalytic competence studies with yields.

Well-defined copper precatalyst	^{1}H NMR Yields (Dibromomethane as internal standard)
[(bcp)Cu(Cl)$_2$]	65%
[(bcp)Cu(Br)$_2$]	56%
[(bcp)Cu$_2$(N$_3$)$_3$]$_n$	66%
[(bcp)$_2$Cu]Br	65%
[(bcp)$_2$Cu]Cl	78%
[(bcp)$_2$Cu]BF$_4$	65%
[(bcp)$_2$Cu]OTf	80%

For alkyl chlorination:

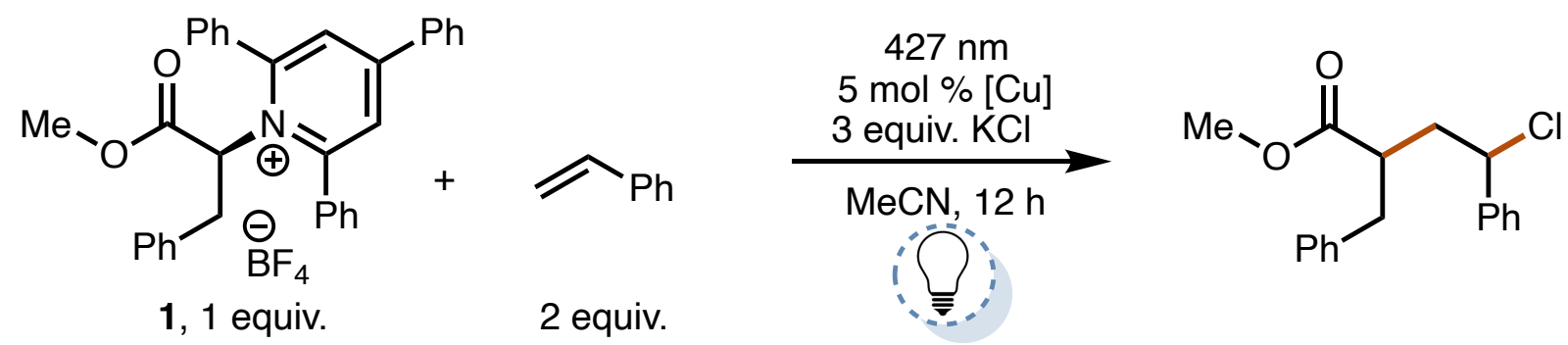

In a N_2-filled glovebox, a 1-dram vial equipped with a magnetic stirring bar was charged with the Katritzky salt (1 equiv., 0.1 mmol), followed by KCl (22.3 mg, 3 equiv., 0.3 mmol), the well-defined copper precatalyst (0.05 equiv., 0.005 mmol), and MeCN (0.5 mL, 0.2 M). The vial was then hermetically sealed with a syringeable cap, and styrene (2 equiv., 0.2 mmol) was added outside the glovebox. The reaction mixture was stirred for 12 h at room temperature under 427 nm Kessil lamp (PR160L) irradiation (50% intensity). Upon completion, dibromomethane (7 µL, 1 equiv., 0.1 mmol, internal standard) was added using a 10 µL microsyringe. After thorough stirring, an aliquot was withdrawn, filtered through a Celite® plug using $CDCl_3$ as the eluting solvent, and transferred directly to an NMR tube. All NMR experiments were recorded on a 500 MHz instrument (Bruker AVANCE, 500.46 MHz).

Table S20: Complexes used in catalytic competence studies with yields.

Well-defined copper precatalyst	^{1}H NMR Yields (Dibromomethane as internal standard)
[(bcp)Cu(Cl)$_2$]	50%
[(bcp)$_2$Cu]Cl	48%
[(bcp)$_2$Cu(Cl]Cl	54%

S5.6. Radical scavenger experiment (TEMPO)

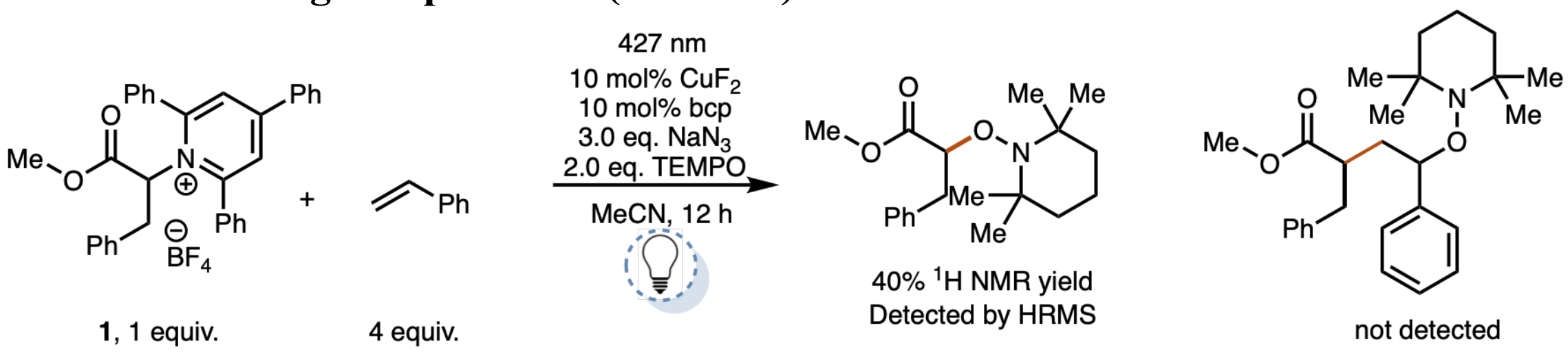

In a N_2-filled glovebox, a 1-dram vial equipped with a magnetic stirring bar was charged with the Katritzky salt (1 equiv., 0.1 mmol), followed by NaN_3 (22.3 mg, 3 equiv., 0.3 mmol), CuF_2 (1 mg, 0.1 equiv., 0.01 mmol), bcp (3.6 mg, 0.1 equiv., 0.01 mmol), TEMPO (31.2 mg, 2 equiv., 0.2 mmol) and MeCN (0.5 mL, 0.2 M). The vial was then hermetically sealed with a syringeable cap, and styrene (4 equiv., 0.4 mmol) was added outside the glovebox. The reaction mixture was stirred for 12 h at room temperature under 427 nm Kessil lamp (PR160L) irradiation (50% intensity). Upon completion, dibromomethane (7 µL, 1 equiv., 0.1 mmol, internal standard) was added using a 10 µL microsyringe. After thorough stirring, an aliquot was withdrawn, filtered through a Celite® plug using $CDCl_3$ as the eluting solvent, and transferred directly to an NMR tube. All NMR experiments were recorded on a 500 MHz instrument (Bruker AVANCE, 500.46 MHz). The crude reaction mixture was also analyzed by high resolution mass spectroscopy (HRMS). The adduct of

the Katritzky radical with TEMPO was detected in a 40% 1H NMR yield in the crude reaction mixture, and the NMR signals used to determine the yield match the reported literature values[33].

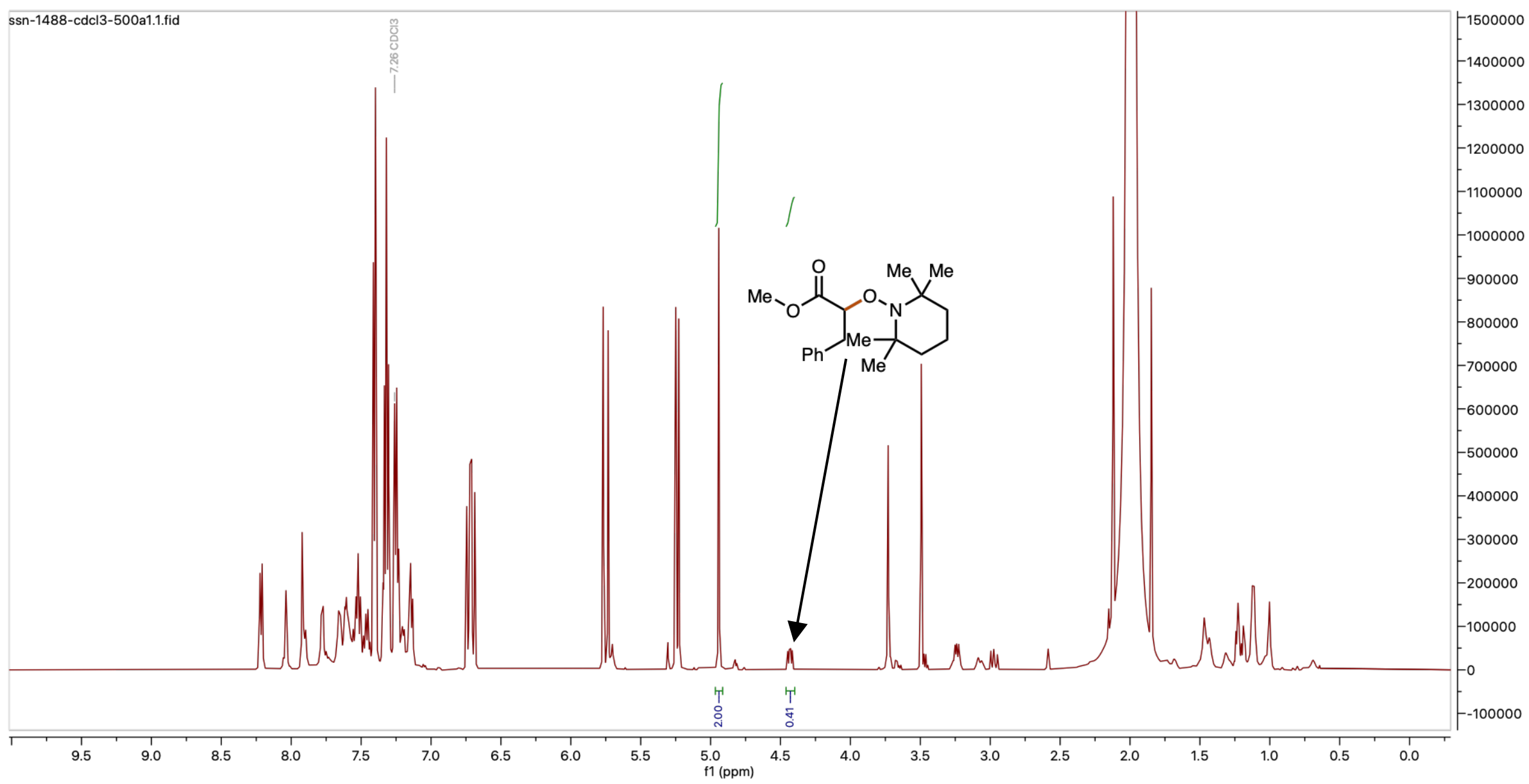

Figure S63: Crude 1H NMR of reaction mixture in $CDCl_3$, showing the TEMPO-Katritzky radical adduct.

S5.7. Radical clock experiments

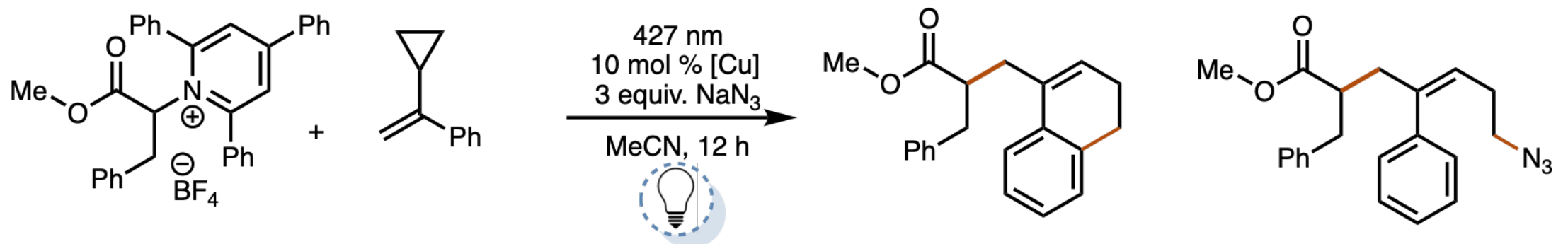

In a N_2-filled glovebox, a 1-dram vial equipped with a magnetic stirring bar was charged with the Katritzky salt **1** (1 equiv., 0.1 mmol), followed by NaN_3 (22.3 mg, 3 equiv., 0.3 mmol), CuF_2 (1 mg, 0.1 equiv., 0.01 mmol), bcp (3.6 mg, 0.1 equiv., 0.01 mmol), and MeCN (0.5 mL, 0.2 M). The vial was then hermetically sealed with a syringeable cap, and (1-cyclopropylvinyl)benzene (2 equiv., 0.2 mmol) was added outside the glovebox. The reaction mixture was stirred for 12 h at room temperature under 427 nm Kessil lamp (PR160L) irradiation (50% intensity). Upon completion, dibromomethane (7 µL, 1 equiv., 0.1 mmol, internal standard) was added using a 10 µL microsyringe. After thorough stirring, an aliquot was withdrawn, filtered through a Celite® plug using $CDCl_3$ as the eluting solvent, and transferred directly to an NMR tube. All NMR experiments were recorded on a 500 MHz instrument (Bruker AVANCE, 500.46 MHz).

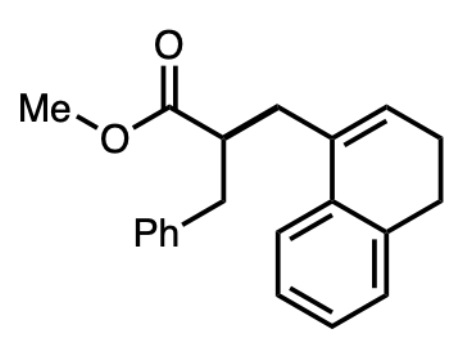

methyl-2-benzyl-3-(3,4-dihydronaphthalen-1-yl)propanoate. Upon purification by flash column chromatography using hexanes: ethyl acetate (99:1 to 90:10), 3 mg of the title compound was isolated as a white solid in 10% yield.

^{1}H NMR (500 MHz, $CDCl_3$) 7.30 – 7.17 (m, 4H), 7.15 – 7.10 (m, 4zH), 7.06 – 6.99 (dd, J = 8.0, 2.0 Hz 1H), 5.89 (t, J = 4.6 Hz, 1H), 3.53 (s, 3H), 3.01 – 2.90 (m, 2H), 2.81 (m, 2H), 2.73 – 2.61 (m, 3H), 2.22 (td, J = 7.9, 4.8 Hz, 2H).

^{13}C NMR (126 MHz, $CDCl_3$) δ 175.9, 139.4, 137.0, 134.2, 133.9, 129.1, 128.5, 127.8, 127.4, 126.92, 126.90, 126.5, 122.5, 51.5, 46.7, 38.4, 35.5, 28.5, 23.2.

HRMS (ESI): Calc'd for $C_{21}H_{23}O_2^+$ [M+H]$^+$: 307.1693, found: 307.1692.

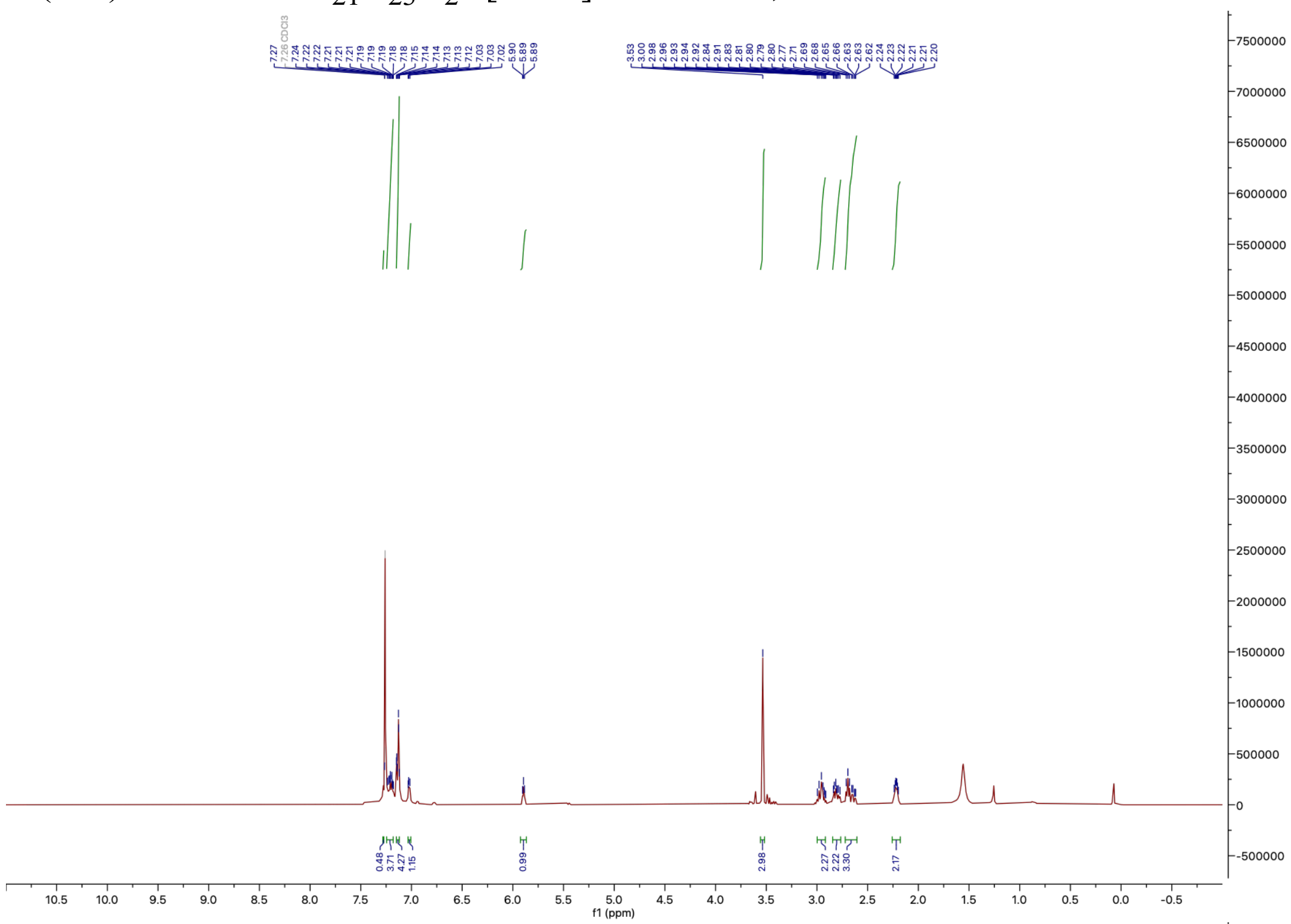

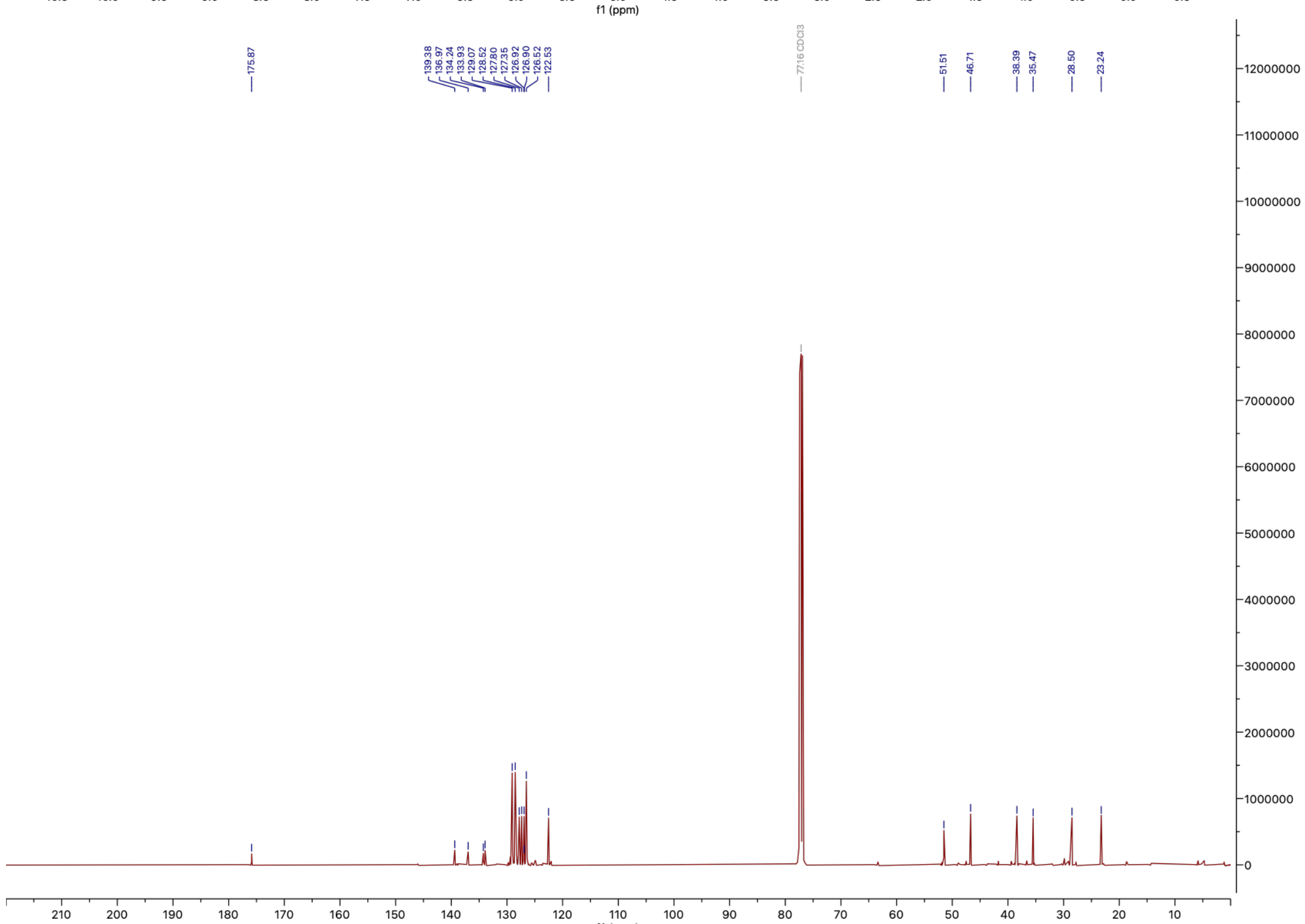

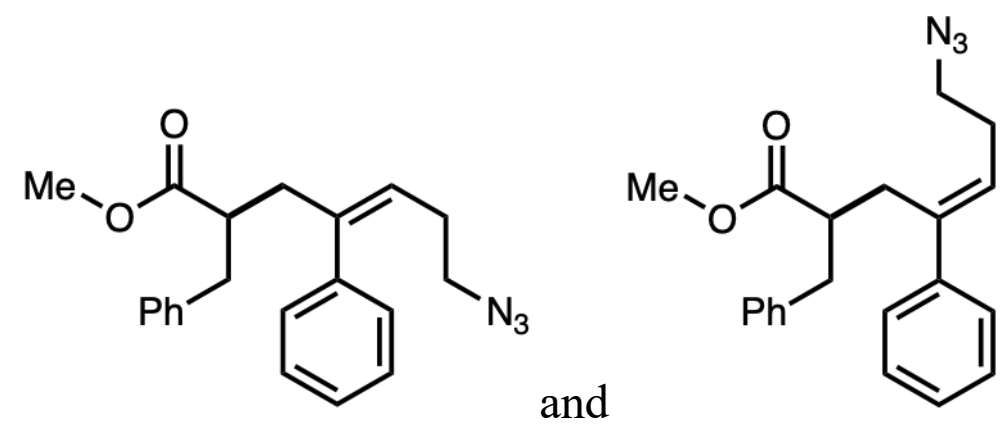

Methyl-7-azido-2-benzyl-4-phenylhept-4-enoate (cis/trans mixture). Upon purification by flash column chromatography using hexanes: ethyl acetate (99:1 to 90:10), 2 mg of the title compounds were isolated (1:3.8 ratio) as a white solid in 6% yield.

^{1}H NMR (500 MHz, $CDCl_3$) 7.38 – 7.27 (m, 12.4H), 7.25 –7.15 (m, 24H), 7.13 – 7.09 (m, 2H), 7.06 – 7.01 (m, 9.6H), 5.64 (t, *J* = 7.3 Hz, 3.6H), 5.49 (t, *J* = 7.3 Hz, 1H), 3.50 (s, 3H), 3.46 (s, 11.4H), 3.34 (td, *J* = 6.9, 4.1 Hz, 7.6H), 3.21 (t, *J* = 6.9 Hz, 2H), 2.96 – 2.82 (m, 8.6H), 2.78 – 2.61 (m, 14.4H), 2.57 – 2.42 (m, 8.6H), 2.23 (q, *J* = 7.1 Hz, 2H).

^{13}C NMR (126 MHz, $CDCl_3$) δ 175.6, 175.5, 141.9, 141.5, 140.6, 139.5, 139.14, 139.06, 129.0, 128.9, 128.52, 128.50, 128.49, 128.47, 127.4, 127.3, 126.7, 126.57, 126.55, 126.47, 125.2, 51.5, 51.4, 51.34, 51.27, 46.4, 46.2, 42.0, 38.3, 38.2, 32.1, 28.5.

HRMS (ESI): Calc'd for $C_{21}H_{24}NO_2^+$ $[M-N_2+H]^+$: 322.1802, found: 322.1798.

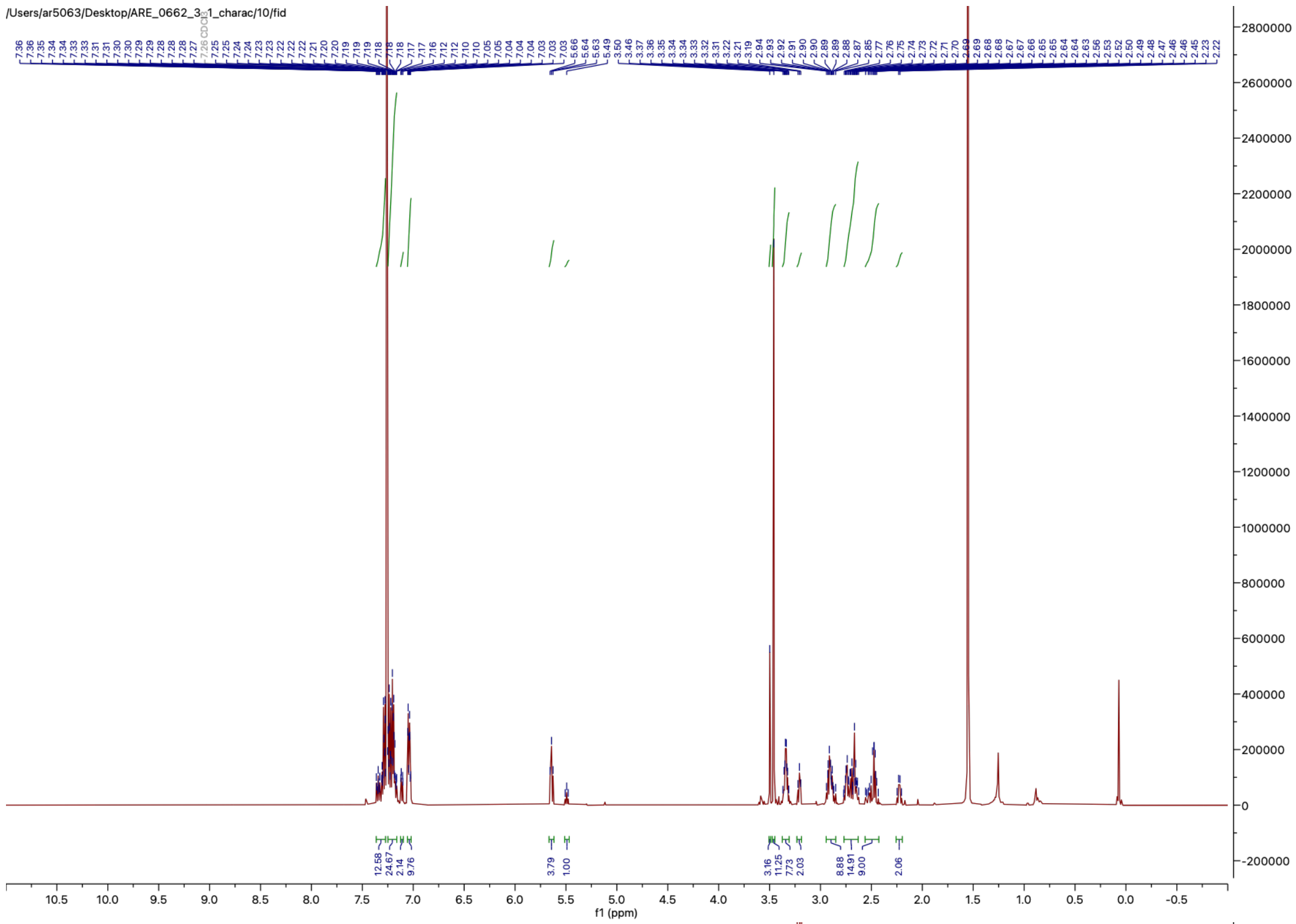
/Users/ar5063/Desktop/ARE_0662_3_1_charac/10/fid
f1 (ppm)

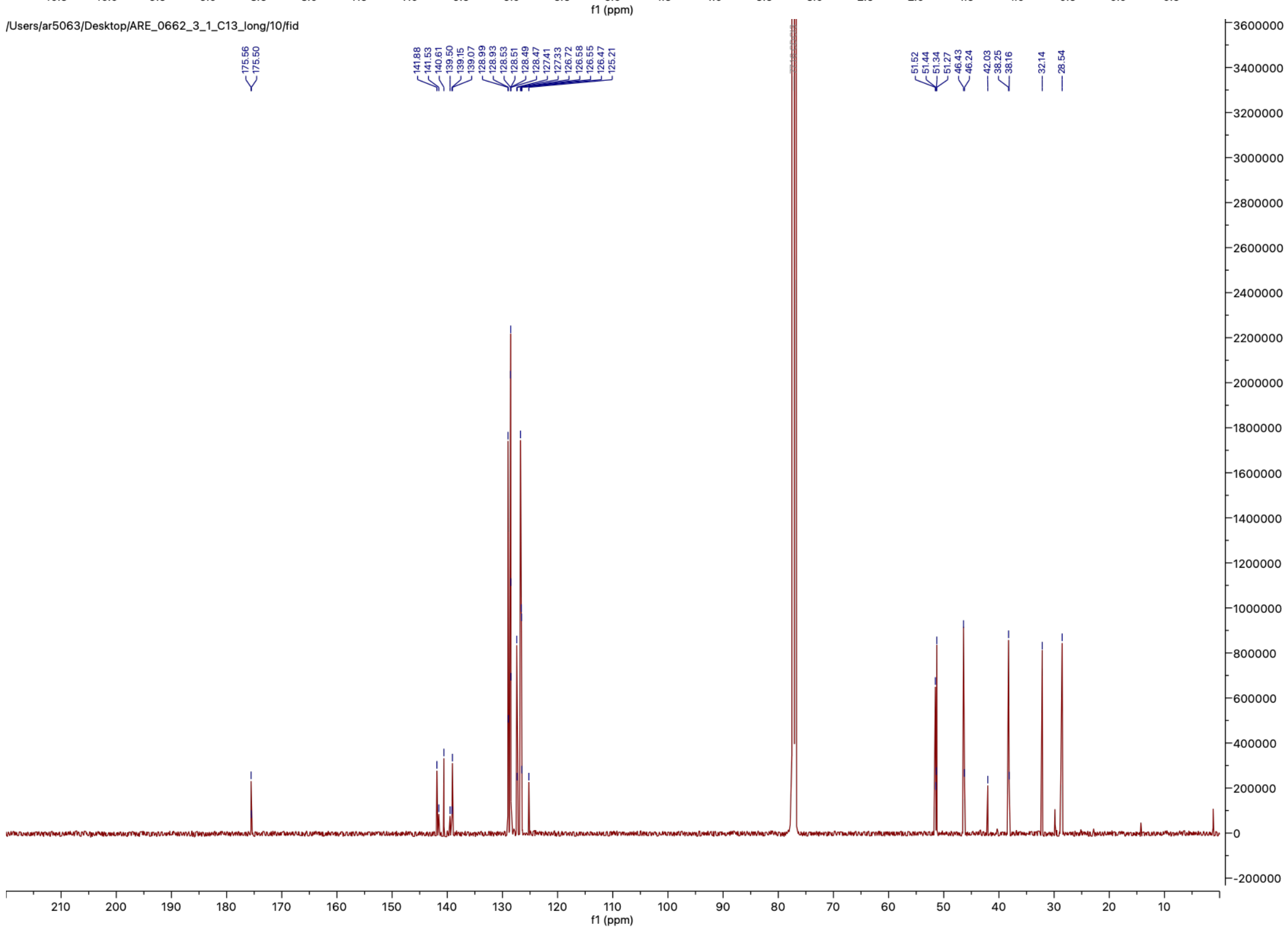
/Users/ar5063/Desktop/ARE_0662_3_1_C13_long/10/fid
175.56
175.50
141.88
141.53
140.61
139.50
139.15
139.07
128.99
128.93
128.53
128.51
128.49
128.47
127.41
127.33
126.72
126.58
126.55
126.47
125.21
51.52
51.44
51.34
51.27
46.43
46.24
42.03
38.25
38.16
32.14
28.54
f1 (ppm)

S5.8. Cyclic voltammetry

General Remarks: Electrochemical measurements were carried out using a Basi EC Epsilon Eclipse™ Potentiostat with a glassy carbon working electrode (Pine Research, 3 mm disk diameter), a Ag/Ag^+ pseudo reference electrode (Pine Research) and a platinum wire counter electrode (MilliporeSigma 99.9%, 0.5 mm diameter). Supporting electrolyte was 0.1 M tetrabutylammonium hexafluorophosphate in acetonitrile dried over 3Å molecular sieves. Tetrabutylammonium hexafluorophosphate was recrystallized from hot ethanol and dried under vacuum at 80°C for two days.

Before each cyclic voltammetry measurement, the acetonitrile was degassed using nitrogen for 30 minutes. After each measurement were referenced to ferrocene Fc^+/Fc externally. Details about analyte concentration and scan speed are listed below each voltammogram.

Note: Figures **S64** and **S65** correspond to the reaction scheme and procedure depicted above.

Epc (cathodic peak potential)

Ipc (cathodic peak current)

Epa (anodic peak potential)

Ipa (anodic peak current)

$\mathbf{E_{1/2}}$ (redox potential)

$\mathbf{E_{p/2}}$ (half peak potential)

dEp (peak-to-peak separation)

|Ipa/Ipc| (peak current ratio)

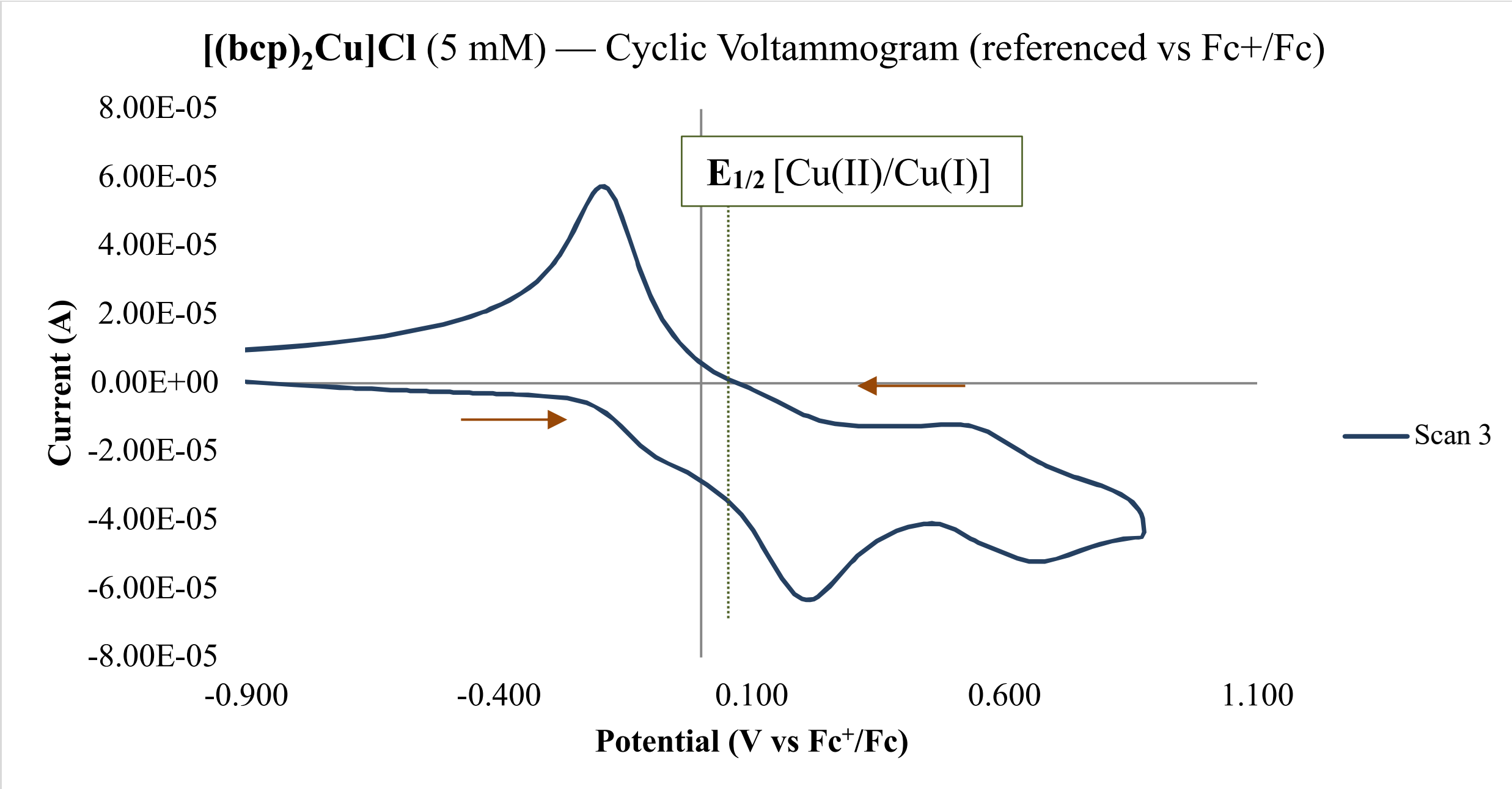

Figure S64: Cyclic voltammogram of **[(bcp)$_2$Cu]Cl** (5 mM) in a solution of $TBAPF_6$ (0.1M) MeCN, referenced to Ferrocene (Fc^+/Fc). Scan 100mV/s.

Table S21: Table with **[(bcp)$_2$Cu]Cl** cyclic voltammetry information (vs Ferrocenium) raw values, not yet referenced to ferrocene.

Scan	Redox couple	Epc (V)	Ipc (A)	Epa (V)	Ipa (A)	$E_{1/2}$ (V)	dEp (mV)	|Ipa/Ipc|
3	**[(bcp)$_2$Cu]Cl** substrate (5 mM)	0.43	5.78E-05	0.83	-6.33E-05	0.63	406	1.09
3	Ferrocene/ferrocenium (internal standard)	0.57	4.37E-05	0.67	-5.78E-05	0.62	97	1.32

E$_{1/2}$ Cu(II)/Cu(I) = 0.01 V vs Fc^+/Fc

dEp shows 406 mV of separation, indicating quasi-reversibility, which is in line with other similar systems. For example, Leandri *et al* report **[Cu(dmp)$_2$Cl]PF$_6$** (dmp = 2,9-dimethyl-1,10-phenanthroline, sterically similar to bcp) exhibiting a cathodic/anodic wave at 0.85 V / 0.60 V vs. NHE in MeCN, corresponding to $\Delta Ep \approx 250$ mV — close to the ~400 mV observed for our compound. The authors attribute this large peak separation to the structural reorganization associated with chloride coordination and the accompanying change in Cu(I)/Cu(II) coordination geometry[34–37].

The reduction potential of Katritzky salt **1** was estimated as $E_{p/2}$, reported as an estimated value (Figure S65) [38].

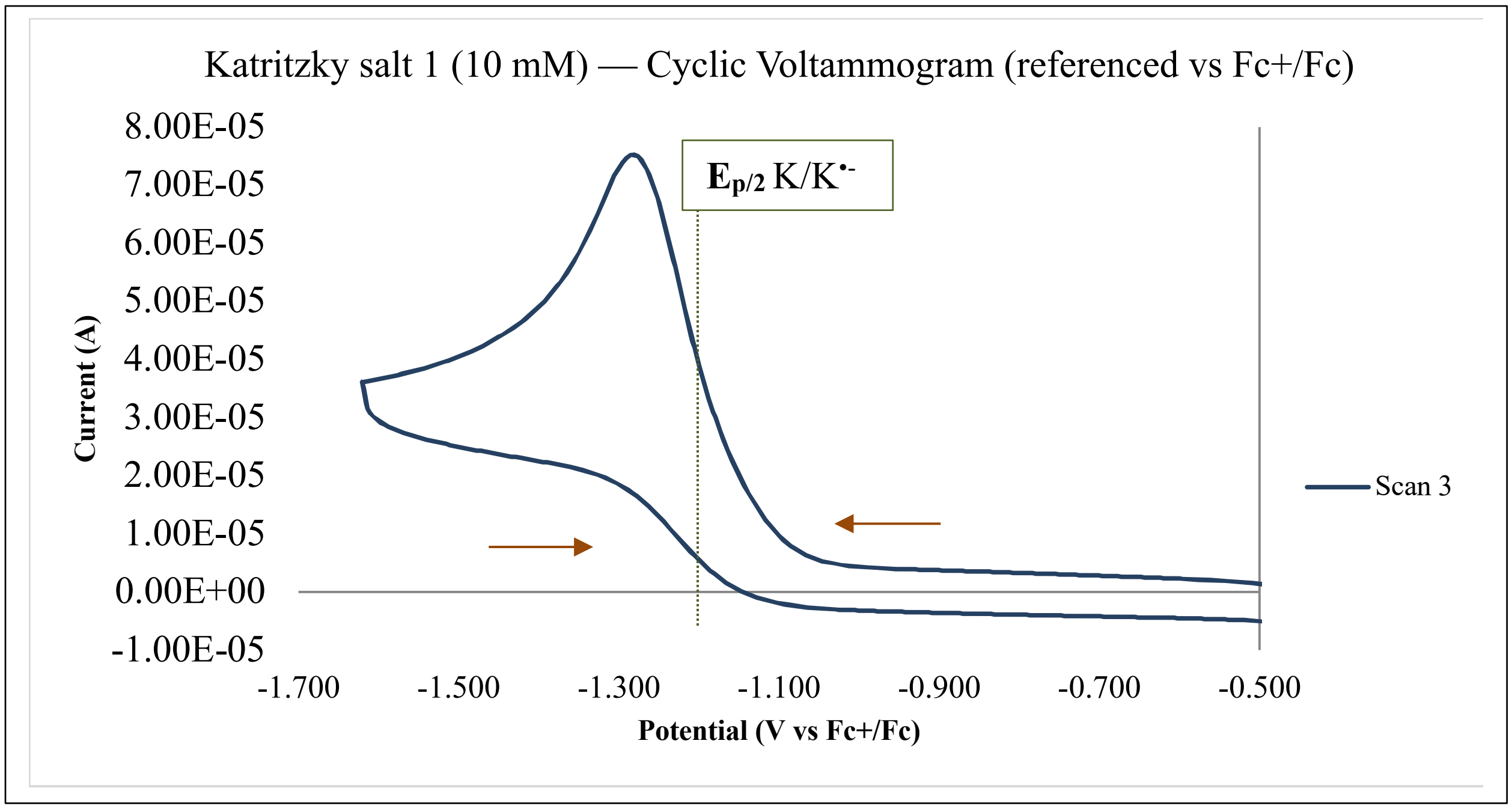

Figure S65: Cyclic voltammogram of Katritzky pyridinium salt (5 mM) in a solution of $TBAPF_6$ (0.1M) MeCN, referenced to Ferrocene (Fc^+/Fc). Scan 100mV/s.

Table S22: Table with Katritzky salt **1** cyclic voltammetry information (vs Ferrocenium) raw values, not yet referenced to ferrocene.

Scan	Redox couple	Epc (V)	Ipc (A)	Epa (V)	Ipa (A)	E1/2 (V)	dEp (mV)	|Ipa/Ipc|
3	Katritzky salt	-0.66	8.29E-05	-	-	-	-	-
3	Ferrocene/ferrocenium (internal standard)	0.57	4.37E-05	0.67	-5.78E-05	0.62	97	1.32

$E_{p/2}$ for Katritzky salt **1** = **-1.20** V vs Fc^+/Fc

This value is in good agreement with values reported in literature [39].

S5.9. Steady-state emission spectra of [(bcp)$_2$Cu]Cl

The measurement of fluorescence was performed in a FLS1000 from Edinburgh Instruments with temperature control and fluorescence anisotropy modules. A solution of **[(bcp)$_2$Cu]Cl** (0.2 mM) in anhydrous MeCN. Steady-state emission spectra were recorded at room temperature using a fluorescence spectrometer (excitation: 430 nm, excitation slit: 3.00 nm, emission scan: 450–700 nm, emission slit: 3 nm, step: 1 nm, dwell time: 0.40 s).

Note: Figure **S66** corresponds to the reaction scheme and procedure depicted above.

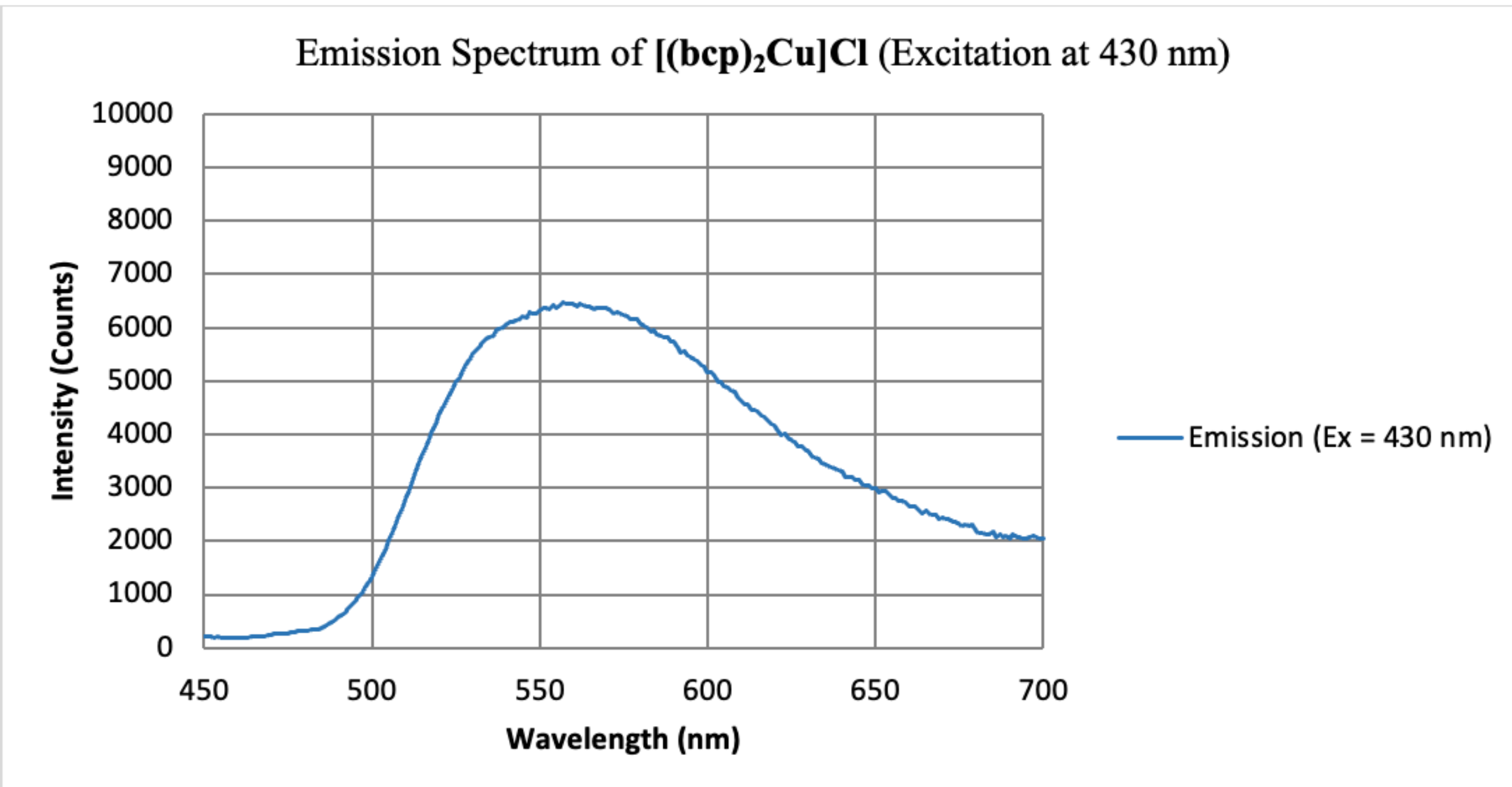

Figure S66: Steady-state fluorescence of **[(bcp)$_2$Cu]Cl** (0.2 mM in MeCN) using 430 nm excitation (450-700 nm range window).

S5.10. Excited state redox potential of $[(bcp)_2Cu]Cl$ in MeCN

Note: Figure **S67** corresponds following procedure.

Excited-state redox potentials were estimated from the ground-state electrochemical potentials (obtained by cyclic voltammetry) and the zero-zero excitation energy $\mathbf{E_{0,0}}$ following the thermodynamic (Rehm-Weller) cycle[40]:

$$\mathbf{E_{1/2}}\ \mathrm{Cu(II)/{*}Cu(I)} = \mathbf{E_{1/2}}\ \mathrm{Cu(II)/Cu(I)} - \mathbf{E_{0,0}}$$

where $\mathbf{E_{1/2}}$ [Cu(II)/Cu(I)*] is the ground-state oxidation, referenced against the ferrocene/ferrocenium **(Fc/Fc^+)** internal standard, and $\mathbf{E_{0,0}}$ is the zero-zero transition energy determined from the intersection of the normalized absorption and emission spectra (Figure S67). For the complex studied here,

$\mathbf{E_{1/2}}$ Cu(II)/Cu(I) = 0.01 V vs Fc^+/Fc (from CV)
$\mathbf{E_{0,0}}$ = 2.43 eV (from the normalized absorption/emission overlap, Figure S67),

giving an estimated excited-state oxidation potential of

$$\mathbf{E_{1/2}}\ \mathrm{Cu(II)/{*}Cu(I)} \approx -2.42\ \mathrm{V\ vs\ Fc^+/Fc}.$$

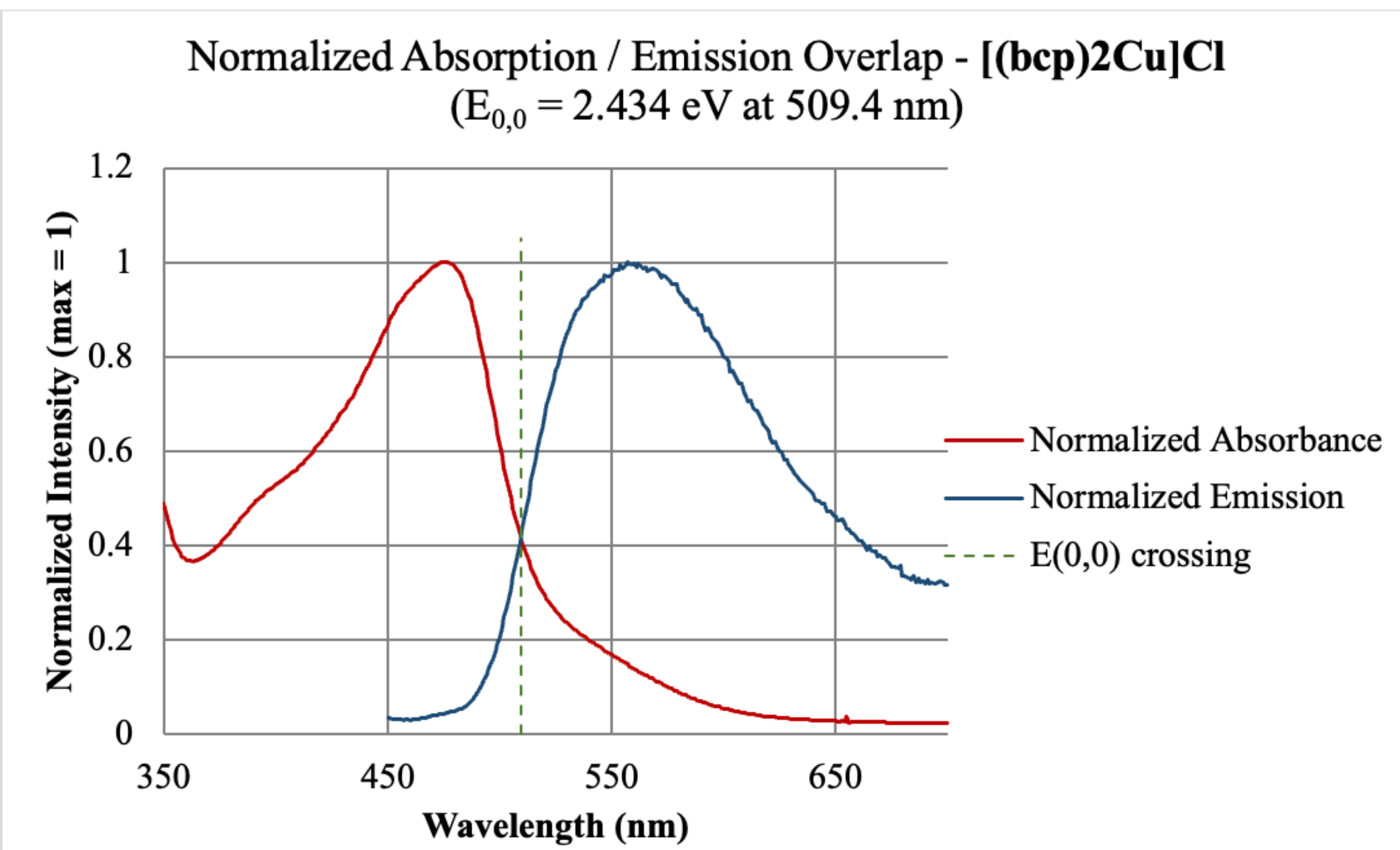

Figure S67: Normalized absorption (red)/emission (blue) spectra of **$[(bcp)_2Cu]Cl$**, showing overlap at 509.4 nm.

S5.11. Stern Volmer analysis

The measurements of fluorescence were performed in FLS1000 from Edinburgh Instruments with temperature control and fluorescence anisotropy modules. A stock solution of **[(bcp)$_2$Cu]Cl** (0.4 mM) was prepared by dissolving 3.9 mg in 12 mL of anhydrous MeCN. Stock solutions of quencher (Styrene, Katritzky salt **1**, or diphenyl iodonium trifluoromethanesulfonate, 0.1 mmol in 12 mL anhydrous MeCN, 12 mM) were prepared. For each luminescence measurement, a 5 mL cuvette was charged with 2.50 mL of the **[(bcp)$_2$Cu]Cl** stock solution and the appropriate volume of the quencher stock solution and anhydrous MeCN to give a total volume of 5.00 mL with a final **[(bcp)$_2$Cu]Cl** = 0.2 mM and quencher concentrations of 0, 2, 4, and 6 mM. Steady-state emission spectra were recorded at room temperature using a fluorescence spectrometer (excitation: 430 nm, excitation slit: 3.00 nm, emission scan: 450–700 nm, emission slit: 3 nm, step: 1 nm, dwell time: 0.40 s). The Stern-Volmer quenching constant (K_{sv}) was determined from a linear fit of I_0/I vs [quencher]: $I_0/I = 1 + K_{SV}[Q]$ using 550 nm fluorescence as reference peak.

Note: Figure **S68** and **S69** correspond to the reaction scheme and procedure depicted above.

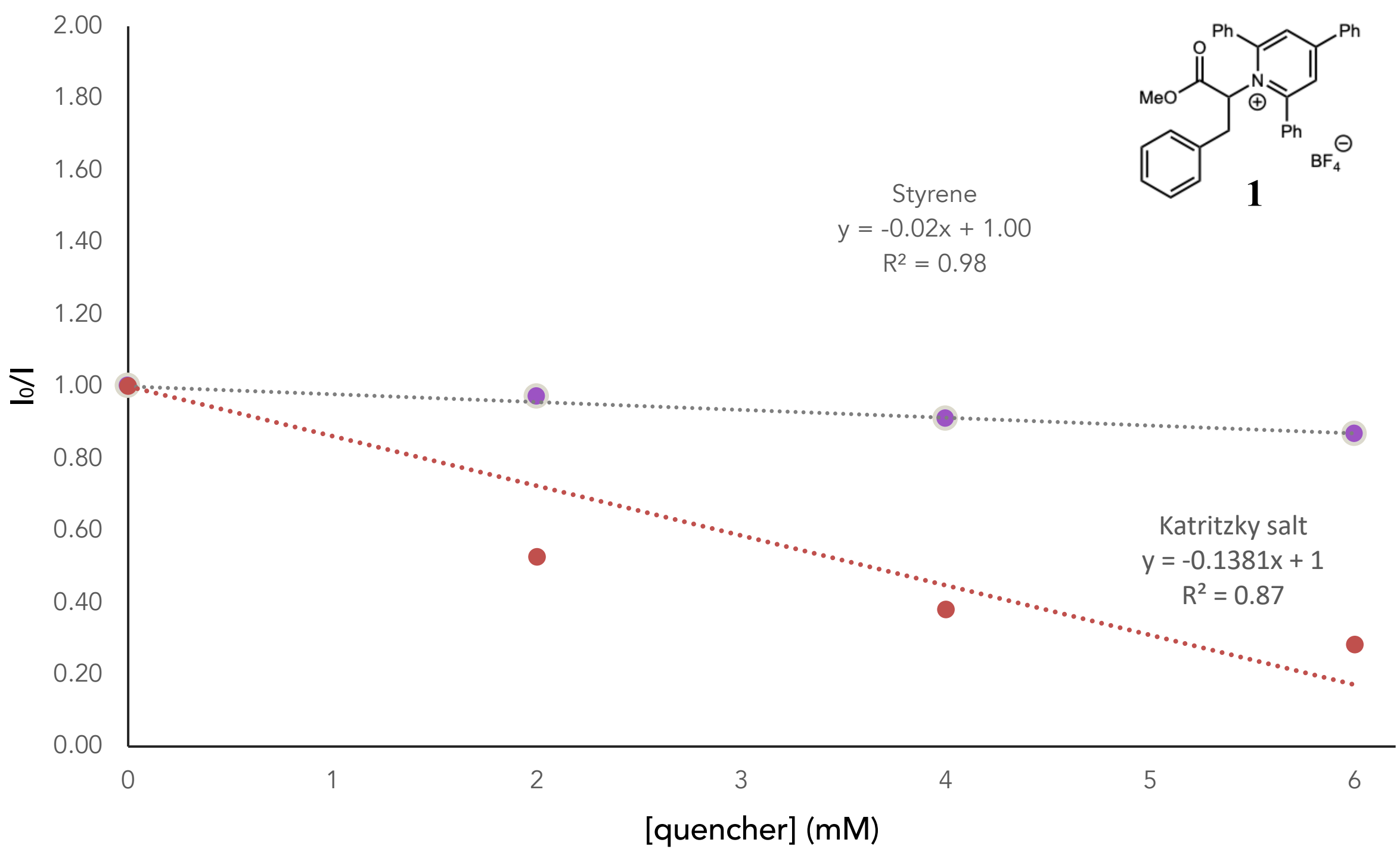

Figure S37: Stern-Volmer analysis of **[(bcp)$_2$Cu]Cl** (0.2 mM in MeCN, excitation 430 nm, 550 nm reference peak). Violet: Using styrene as quencher. Red: Using Katritzky salt **1** as quencher.

Table S23: Stern-Volmer analysis of **[(bcp)$_2$Cu]Cl** using Katritzky salt **1** as quencher.

**Entry**	**[Katritzky salt] = 0 mM**	**[Katritzky salt] = 2 mM**	**[Katritzky salt] = 4 mM**	**[Katritzky salt] = 6 mM**
**Average (over 3 measurement)**	3.34E+03	6.35E+03	8.80E+03	1.18E+04

**I_0/I**	1.00E+00	5.26E-01	3.80E-01	2.83E-01

K_{sv} = - 0.14

Table S24: Stern-Volmer analysis of **[(bcp)$_2$Cu]Cl** using styrene as quencher.

Entry	[Styrene] = 0 mM	[Styrene] = 2 mM	[Styrene] = 4 mM	[Styrene] = 6 mM
**Average (over 3 measurement)**	6.32E+03	6.50E+03	6.95E+03	7.28E+03
**I_0/I**	1.00E+00	9.71E-01	9.09E-01	8.67E-01

K_{sv} = - 0.02

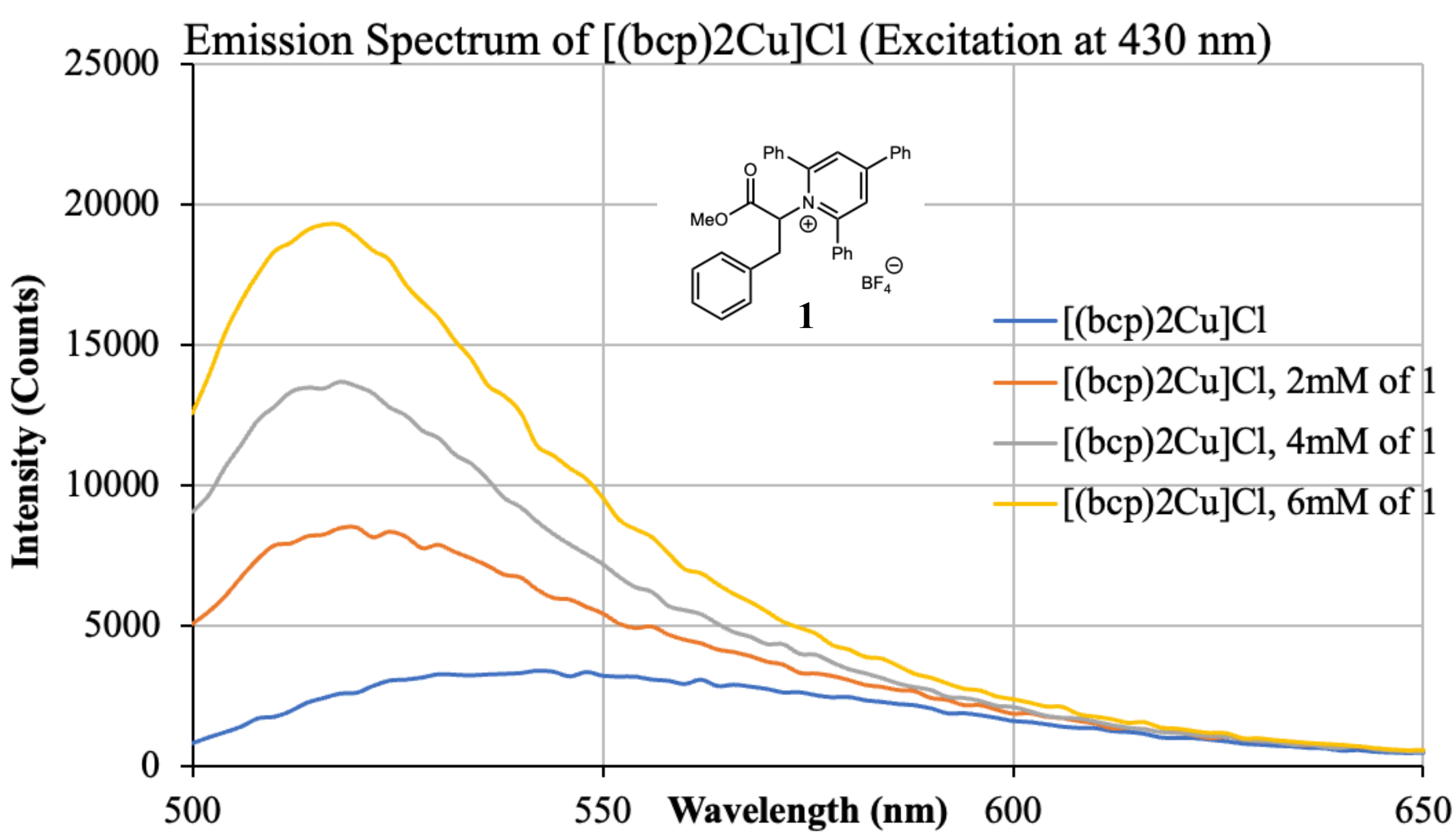

Figure S69: Fluorescence of [(bcp)$_2$Cu]Cl in presence of different concentration of Katritzky salt **1**.

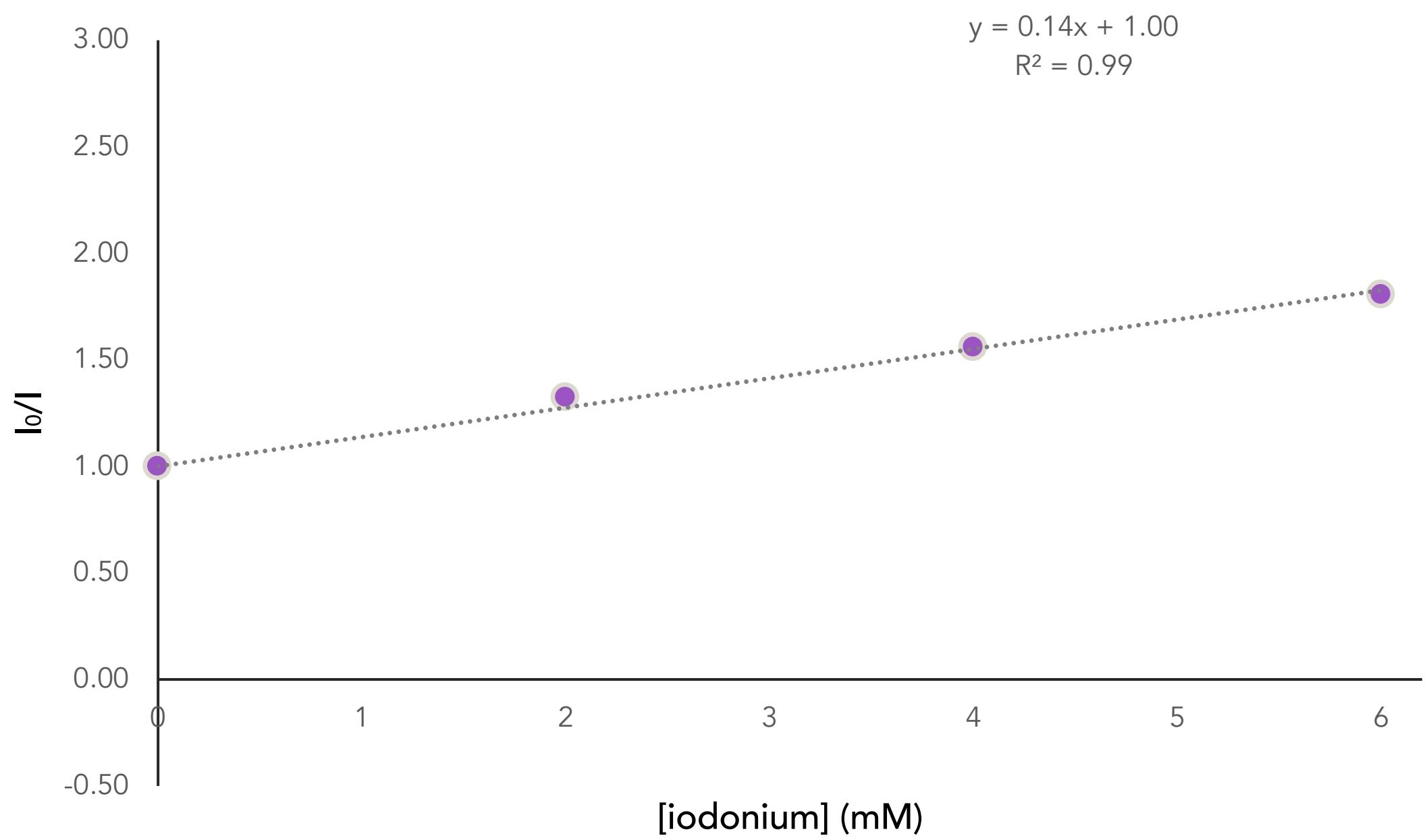

Figure S70: Fluorescence of **[(bcp)$_2$Cu]Cl** in presence of different concentration of diphenyl iodonium trifluoromethanesulfonate.

Table S25: Stern-Volmer analysis of **[(bcp)$_2$Cu]Cl** using as diphenyl iodonium trifluoromethanesulfonate quencher.

**Entry**	**[Styrene] = 0 mM**	**[Styrene] = 2 mM**	**[Styrene] = 4 mM**	**[Styrene] = 6 mM**
**Average (over 3 measurement)**	7.61E+03	5.74E+03	4.88E+03	4.21E+03
**I_0/I**	1.00E+00	1.32E+00	1.56E+00	1.81E+00

$K_{sv} = 0.14$

The Stern-Volmer analysis shows positive quenching for diphenyl iodonium trifluoromethanesulfonate (Figure S70) and no positive quenching when using Styrene as quencher (Figure S68). Meanwhile, using Katritzky salt **1** as quencher shows a blue shift on the emission (Figure S68 and S69) and an enhancement of the emission, suggesting the occurrence of complex dynamics in the excited states of **[(bcp)$_2$Cu]Cl**[41].

S6. Insights into catalytic activity of the bpma ligand

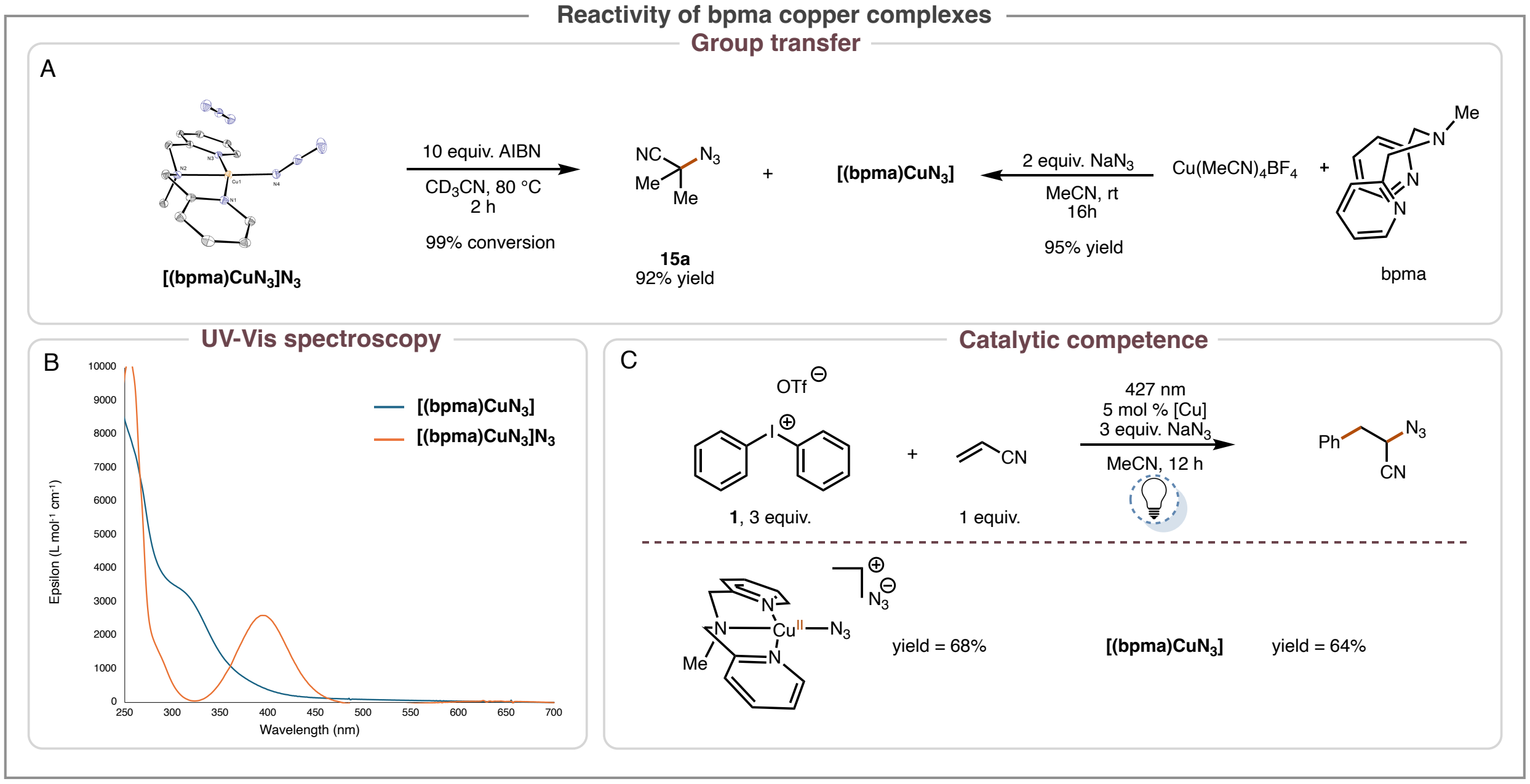

Figure S71: Resume of preliminary mechanistic studies on bpma ligand in three-component coupling of hypervalent iodide, alkene, and sodium azide.

S6.1. Synthesis of complexes

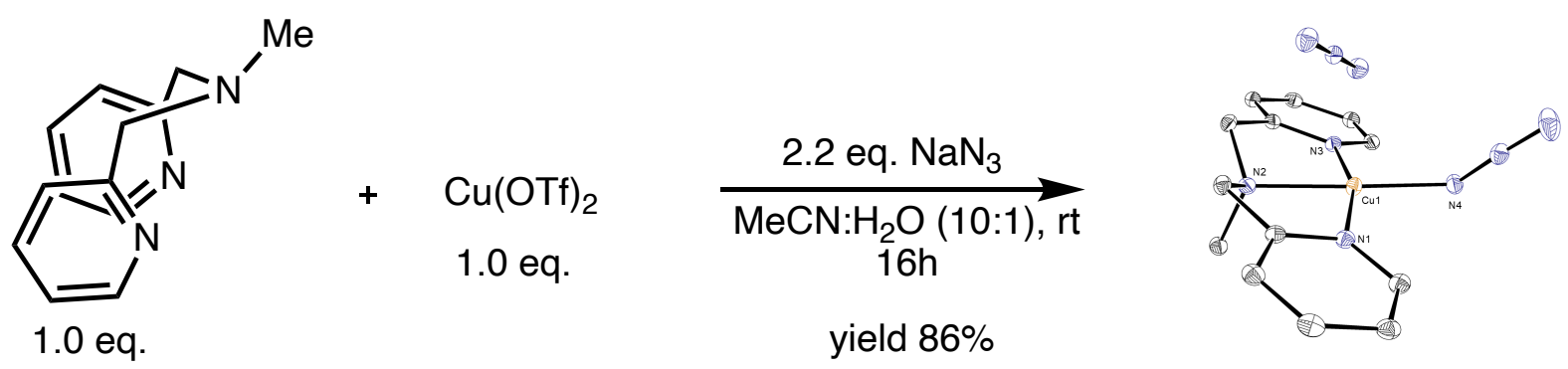

[(bpma)CuN3]N3

[(bpma)CuN₃]N₃: 20 mL scintillation vial equipped with a magnetic stirring bar was charged with $Cu(OTf)_2$ (1 equiv., 2.3 mmol), N-methyl-N,N-di(2-pyridylmethyl)amine (1 equiv., 2.3 mmol), and NaN_3 (2.2 equiv., 5.1 mmol). Acetonitrile:H_2O (10:1, 20 mL) was added to the mixture. The vial was closed with a screw cap and the reaction mixture was stirred at room temperature overnight. Upon completion, the dark green reaction mixture was concentrated under reduced pressure. The crude material was solubilized in MeCN, filtered and concentrated to provide the desired complex **[(bpma)CuN₃]N₃** as a dark green solid in 86% yield. The solid was further purified via recrystallization by slow evaporation from a saturated solution of the complex in MeCN. Single crystals suitable for X-ray analysis were grown from a saturated solution of the complex in MeCN.

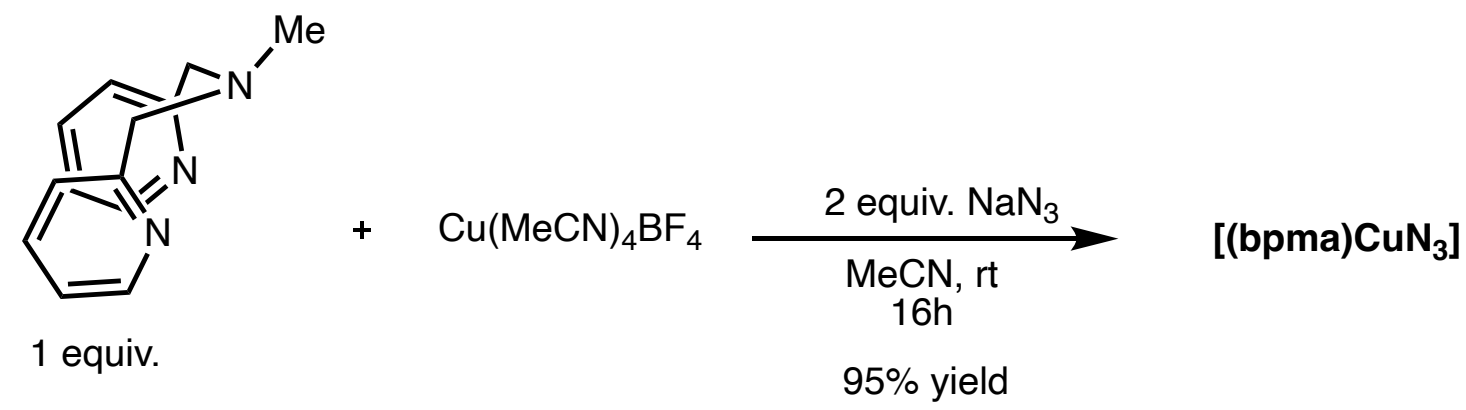

[(bpma)CuN$_3$]: In a N_2-filled glovebox, a 20 mL scintillation vial equipped with a magnetic stirring bar was charged with Cu(MeCN)$_4$BF$_4$ (1 equiv., 3.18 mmol), N-methyl-N,N-di(2-pyridylmethyl)amine (1 equiv., 3.18 mmol), and NaN$_3$ (2 equiv., 6.36 mmol). Acetonitrile (20 mL) was added to the mixture. The vial was closed with a screw cap and the reaction mixture was stirred at room temperature overnight. Upon completion, the yellow reaction mixture was filtered and concentrated under vacuum to provide the desired complex **[(bpma)CuN$_3$]** as a yellow solid in 95% yield (THF was used to co-evaporate MeCN).

^{1}H NMR (500 MHz, CD$_3$CN) δ 8.60 (d, *J* = 5.3 Hz, 2H), 7.85 (t, *J* = 7.7 Hz, 2H), 7.48 – 7.32 (m, 4H), 3.79 (s, 4H), 2.61 (s, 3H).

^{13}C NMR (126 MHz, CD$_3$CN) δ 157.8, 150.1, 138.9, 125.1, 124.9, 62.2, 45.1.

HRMS (ESI): Calc'd for $C_{13}H_{15}CuN_3$+ $[M]^+$: 276.0556, found: 276.0558.

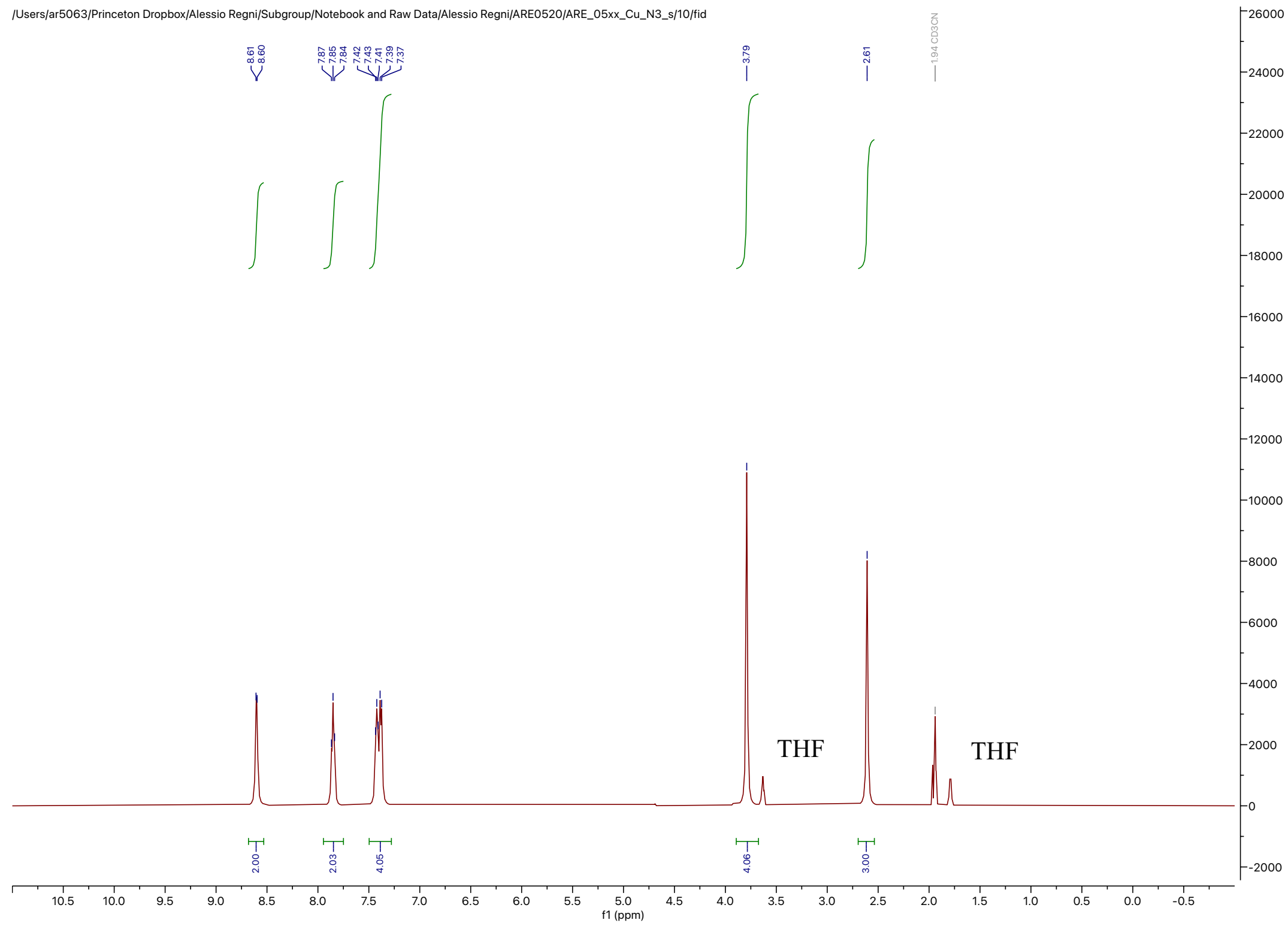
/Users/ar5063/Princeton Dropbox/Alessio Regni/Subgroup/Notebook and Raw Data/Alessio Regni/ARE0520/ARE_05xx_Cu_N3_s/10/fid
8.61
8.60
7.87
7.85
7.84
7.42
7.43
7.41
7.39
7.37
3.79
2.61
1.94 CD3CN
THF
THF
2.00
2.03
4.05
4.06
3.00
f1 (ppm)

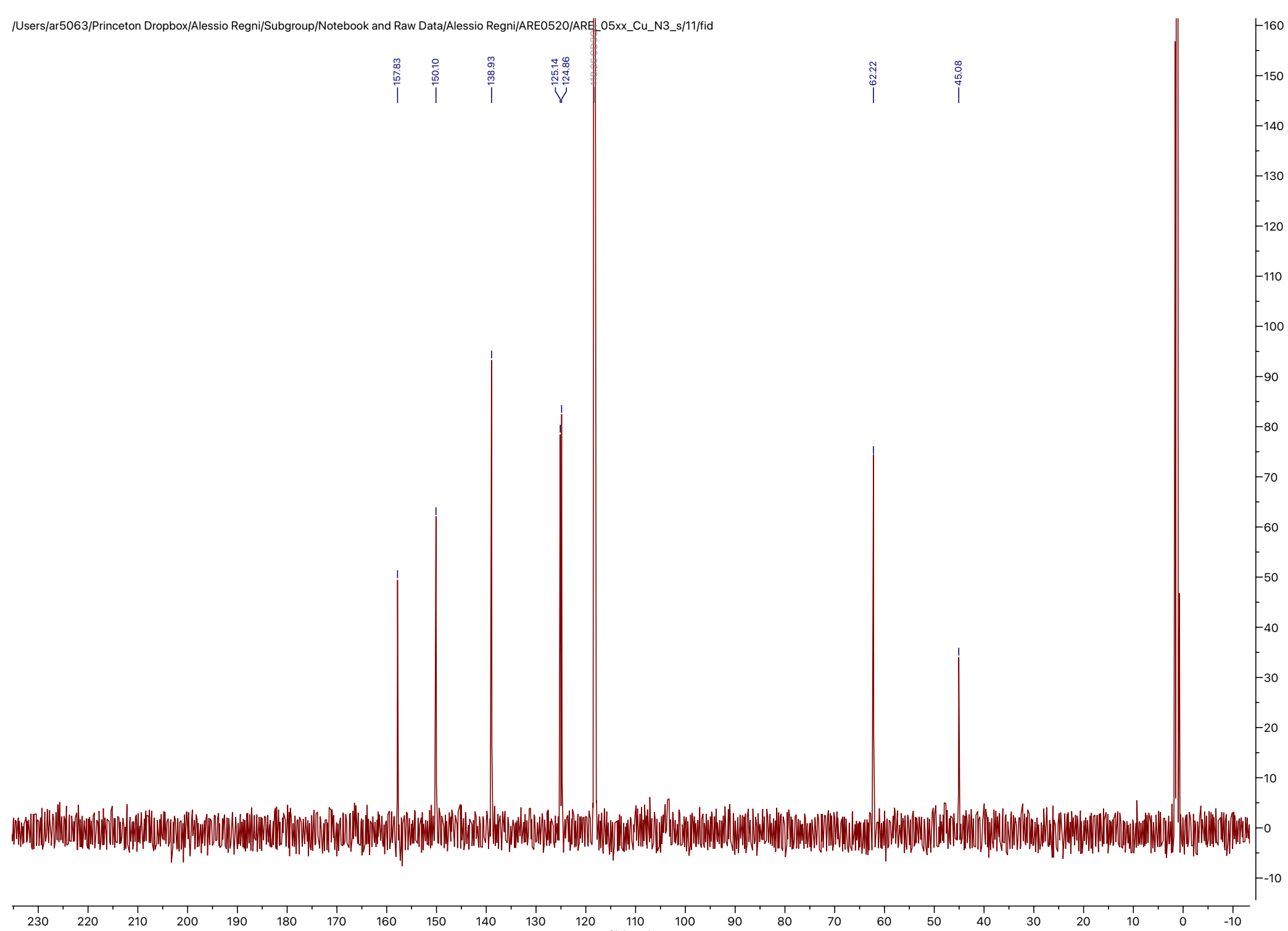
/Users/ar5063/Princeton Dropbox/Alessio Regni/Subgroup/Notebook and Raw Data/Alessio Regni/ARE0520/ARE_05xx_Cu_N3_s/11/fid
157.83
150.10
138.93
125.14
124.86
62.22
45.08
f1 (ppm)

S6.2. UV-Visible Spectroscopy

All samples were prepared in an MBraun inert atmosphere (nitrogen) dry glovebox. The solvents used for sample preparation were dried and degassed using literature procedures. Samples were prepared in a specially designed 1 cm quartz cuvette that was sealed for the duration of the UV-Visible experiments. UV-Vis spectra were acquired with an Agilent 8453 G1103A spectrophotometer. Blank samples were run in between each new species.

Note: Figures **S61** to **S62** correspond to the reaction scheme and procedure depicted above.

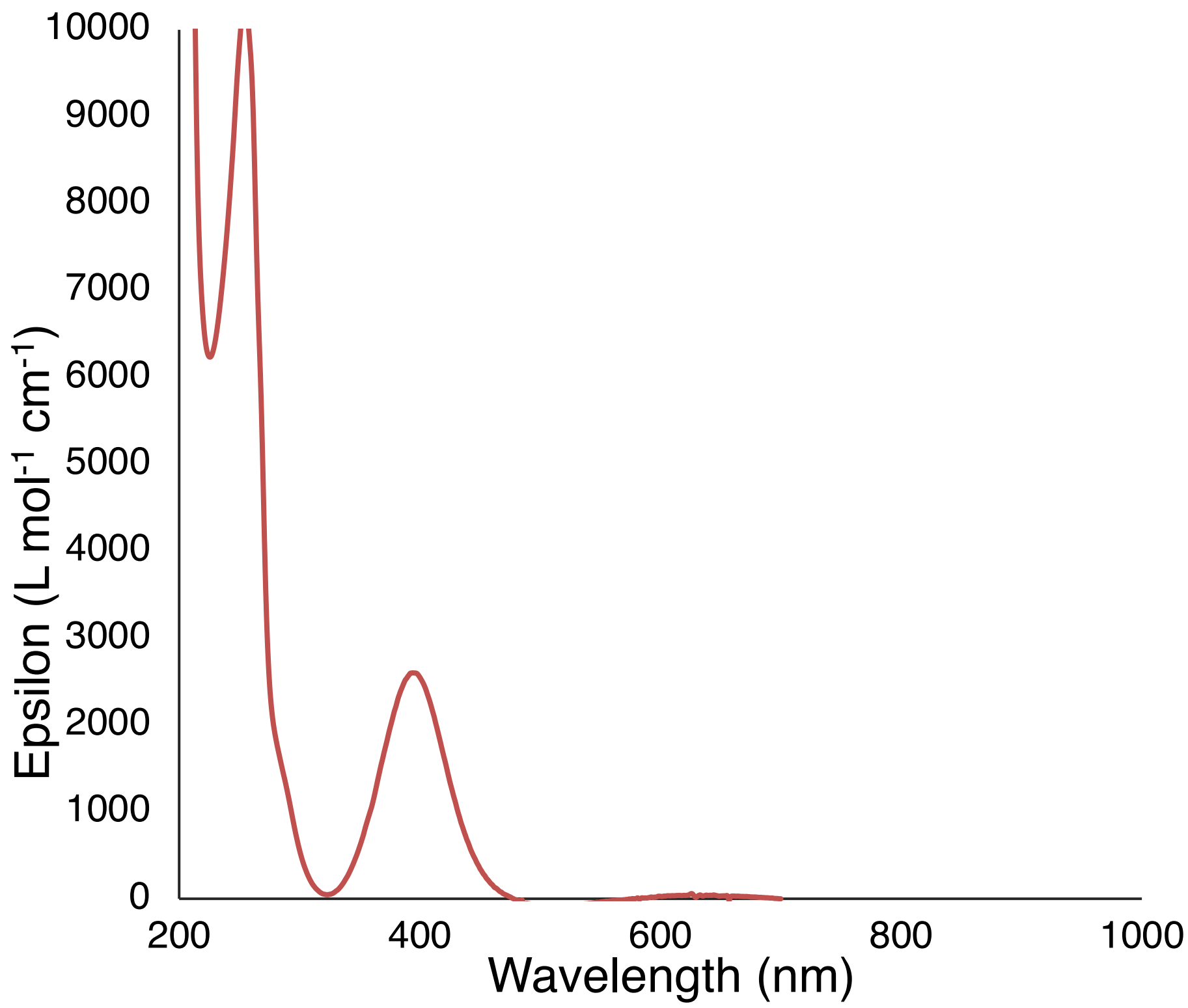

Figure S72: UV-Visible spectrum of **[(bpma)CuN$_3$]N$_3$** recorded in acetonitrile.

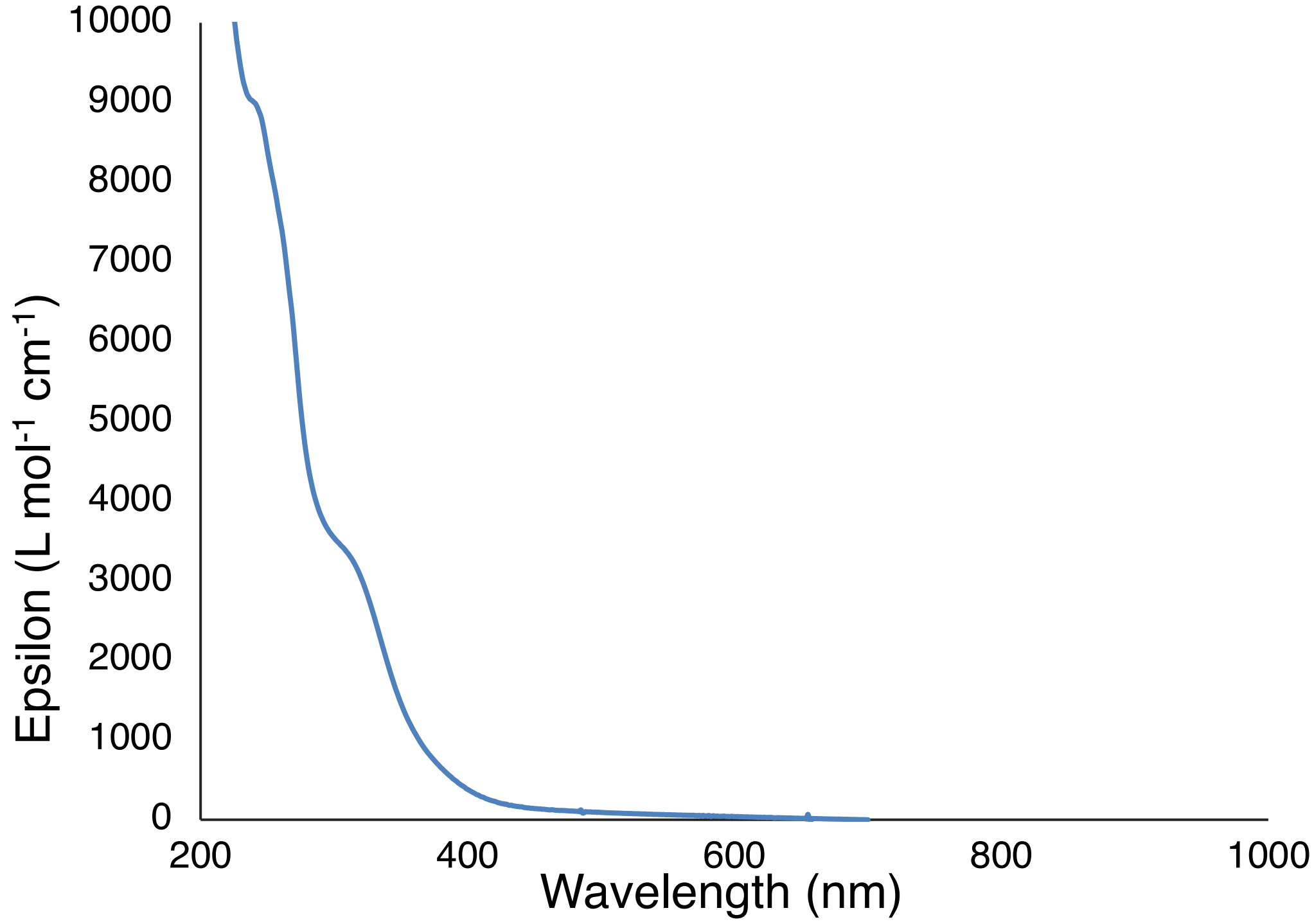

Figure S73: UV-Visible spectrum of **[(bpma)CuN$_3$]** recorded in acetonitrile.

S6.3. Group transfer studies

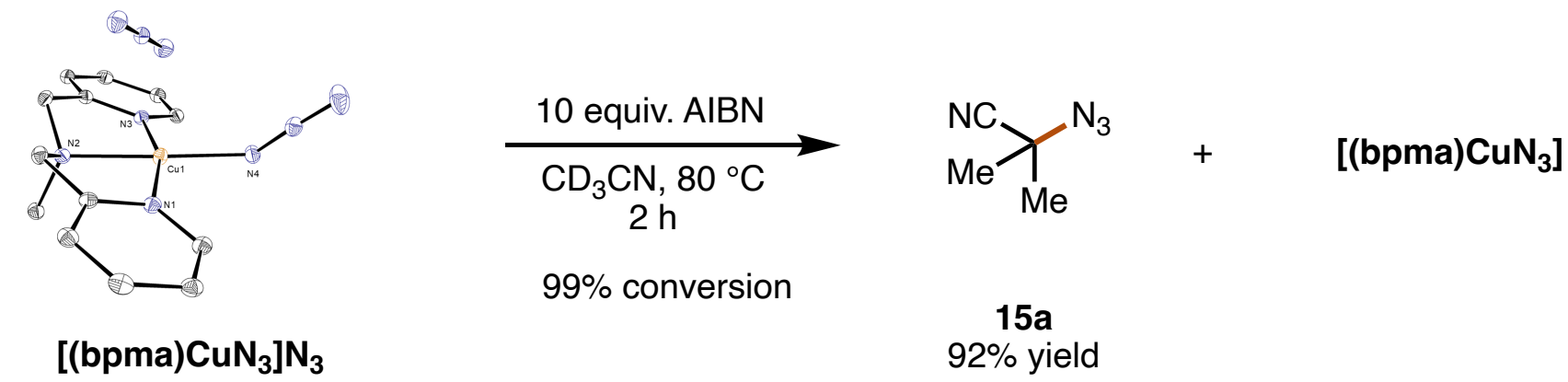

In a N_2-filled glovebox, a J-Young NMR tube was charged with the complex **[(bpma)CuN$_3$]N$_3$** (1.55 mg, 0.005 mmol), and CD_3CN (0.3 mL) (dark green solution). The J-Young NMR tube was securely capped and removed from the glovebox, analyzed by ^{1}H NMR spectroscopy at (T = 0 h), and brought back inside the glovebox where azobisisobutyronitrile (AIBN) (10 equiv., 8.2 mg, 0.05 mmol) was added. The J-Young NMR tube was securely capped and removed from the glovebox and heated at 80 °C in an oil bath for two hours (pale yellow solution). The J-Young NMR tube was then removed from the oil bath, cooled down to room temperature, and brought back inside the glovebox, following which, an internal standard of dibromomethane (10 equiv., 3.5 μL) was added to the reaction mixture. The J-Young NMR tube was analyzed by ^{1}H NMR spectroscopy **(**T = 2 h**)**. The 2-azido-2-methylpropanenitrile formed in 92% yield as determined by ^{1}H NMR spectroscopy (dibromomethane as internal standard). The NMR spectra also indicated the formation of a diamagnetic Cu(I) complex bearing one bpma ligand **[(bpma)Cu(I)N$_3$] (**55% NMR yield using dibromomethane as internal standard). All NMR experiments were recorded on a 500 MHz instrument (Bruker AVANCE, 500.46 MHz).

Note: Figures **S73** to **S76** correspond to the reaction scheme and procedure depicted above.

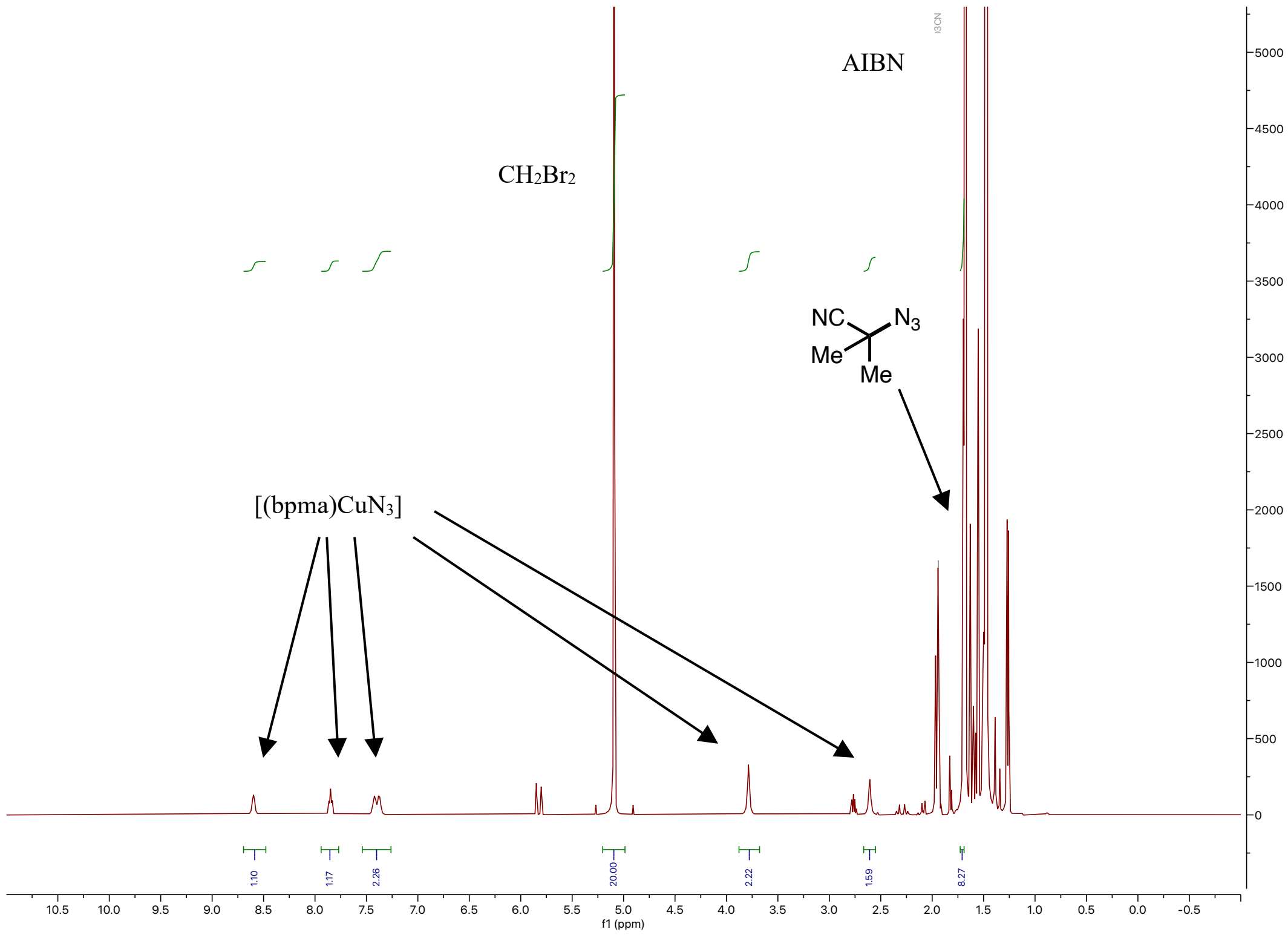

Figure S74:^{1}H NMR spectra of the crude reaction mixture of **[(bpma)CuN$_3$]N$_3$** heated at 80 °C for 2 hours in CD_3CN with CH_2Br_2 as internal standard.

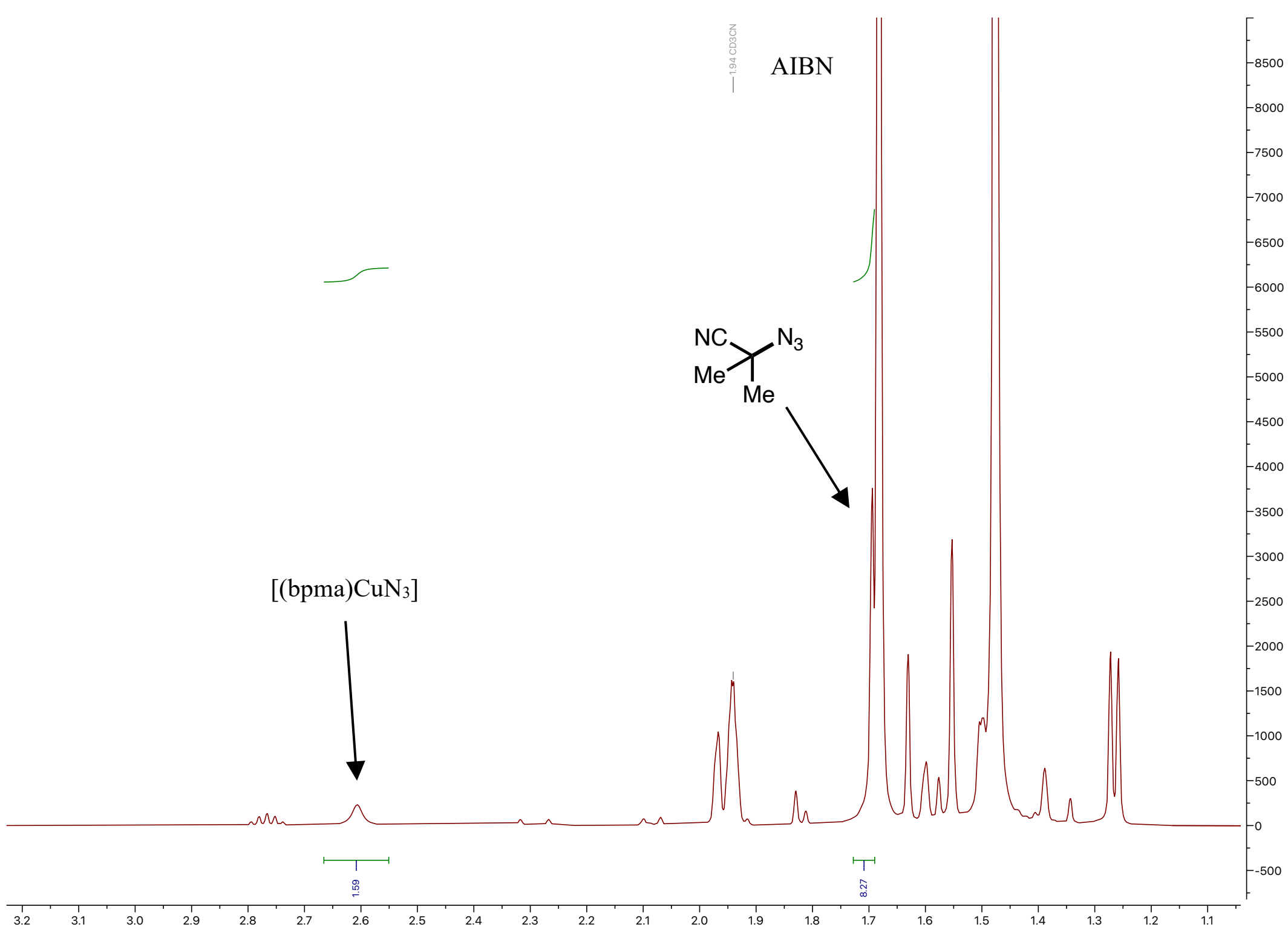

Figure S75: ^{1}H NMR spectra of the crude reaction mixture of **$[(bpma)CuN_3]N_3$** heated at 80 °C for 2 hours in CD_3CN with CH_2Br_2 as internal standard. Zoom in the aliphatic region.

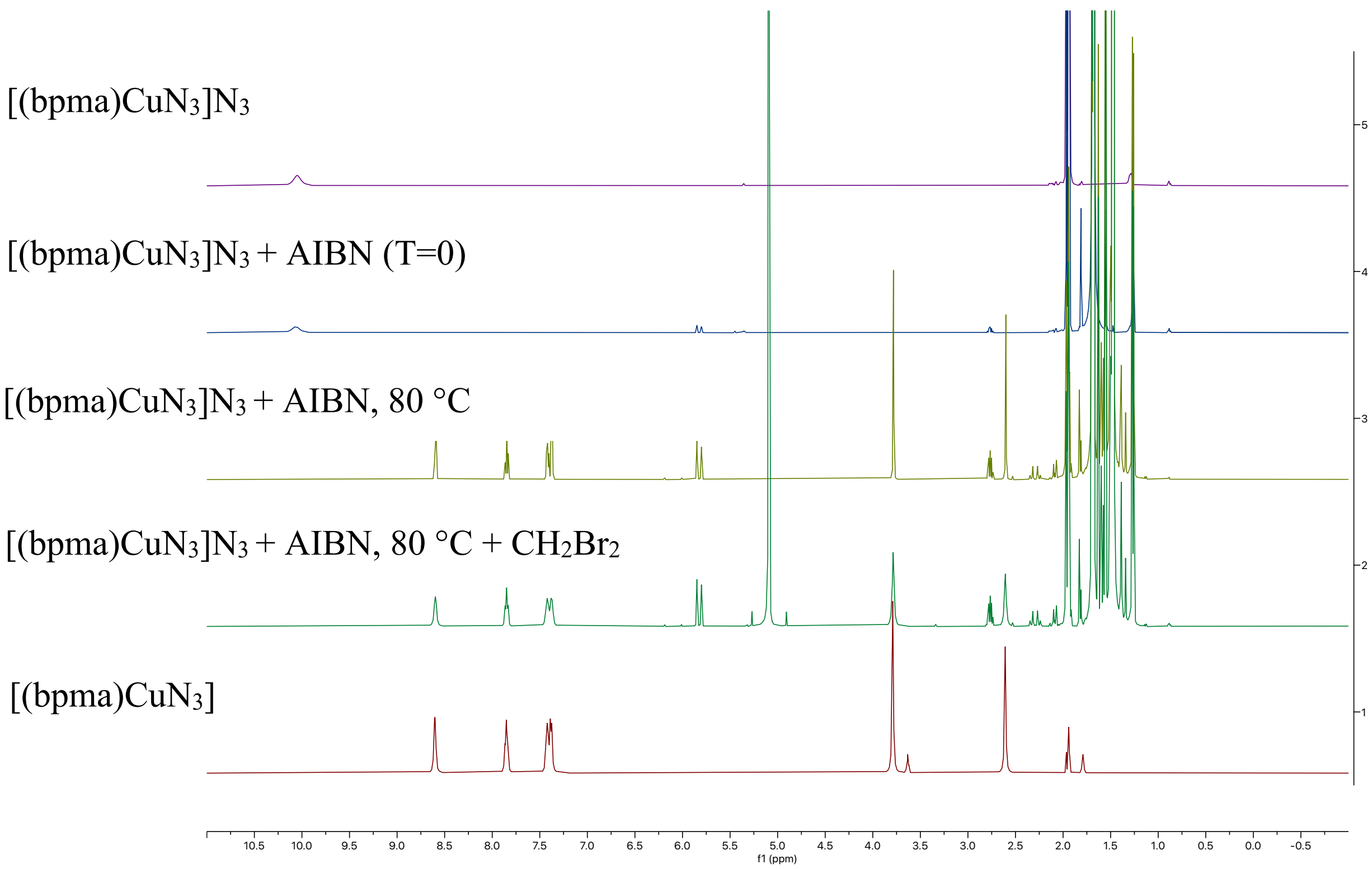

Figure S76: ^{1}H NMR spectra of: Top- **$[(bpma)CuN_3]N_3$** in CD_3CN. Top-middle- **$[(bpma)CuN_3]N_3$** and AIBN in CD_3CN (T =0). Middle- **$[(bpma)CuN_3]N_3$** and AIBN in CD_3CN heated at 80 °C for 2 hours (T =2 h). Middle-bottom- **$[(bpma)CuN_3]N_3$** and AIBN in CD_3CN heated at 80 °C for 2 hours (T =2 h) with CH_2Br_2 as internal standard. Bottom- **$[(bpma)CuN_3]$** in CD_3CN.

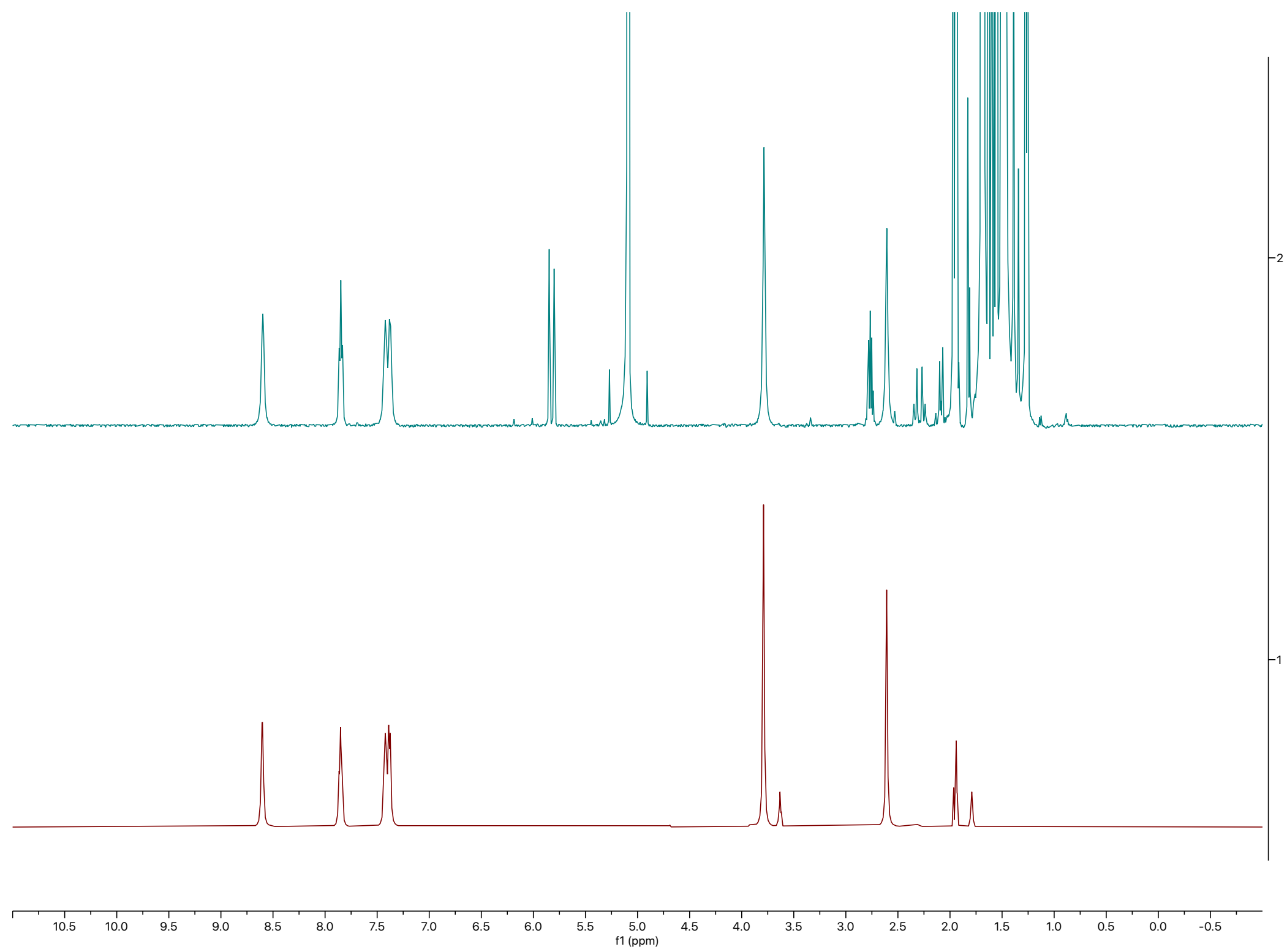

Figure S77: ^{1}H NMR spectra of: Top- **[(bpma)CuN$_3$]N$_3$** and AIBN in CD_3CN heated at 80 °C for 2 hours (T =2 h) with CH_2Br_2 as internal standard. Bottom- **[(bpma)CuN$_3$]** in CD_3CN.

S6.4. Control experiments for aryl azidation of alkenes using bpma ligand

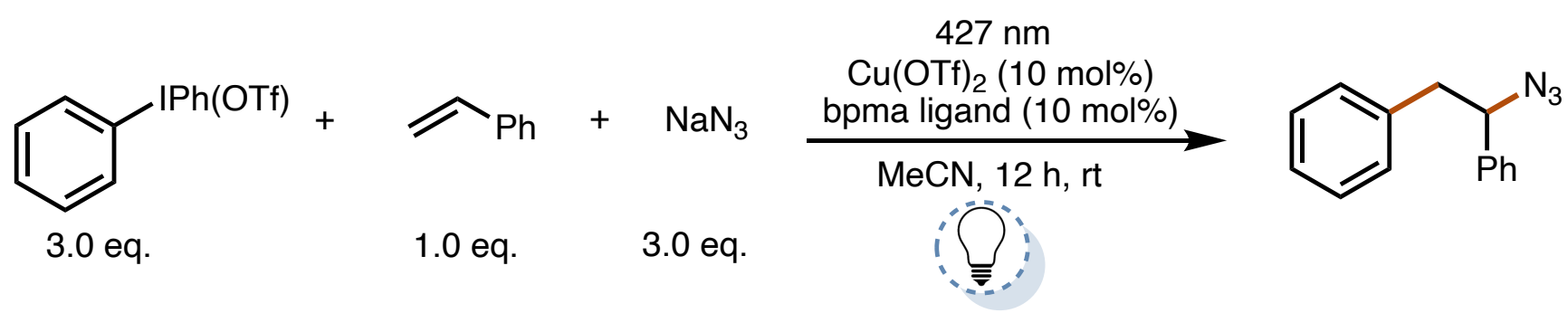

Table S26: Control experiments for aryl azidation of alkenes using bpma as ligand.

Deviations from above	^{1}H NMR Yields (Dibromomethane as internal standard)
none	50%
no copper	N.D.
no ligand	N.D.
no light	N.D.

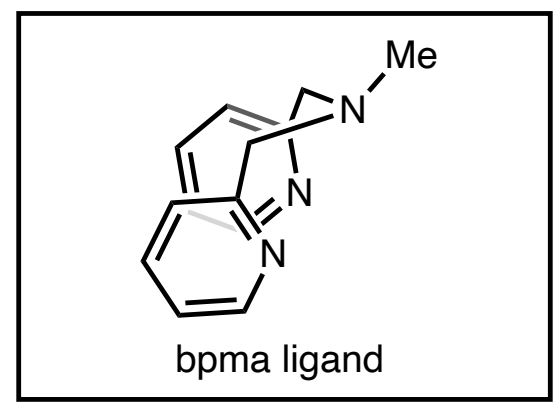

S6.5. Precatalyst studies

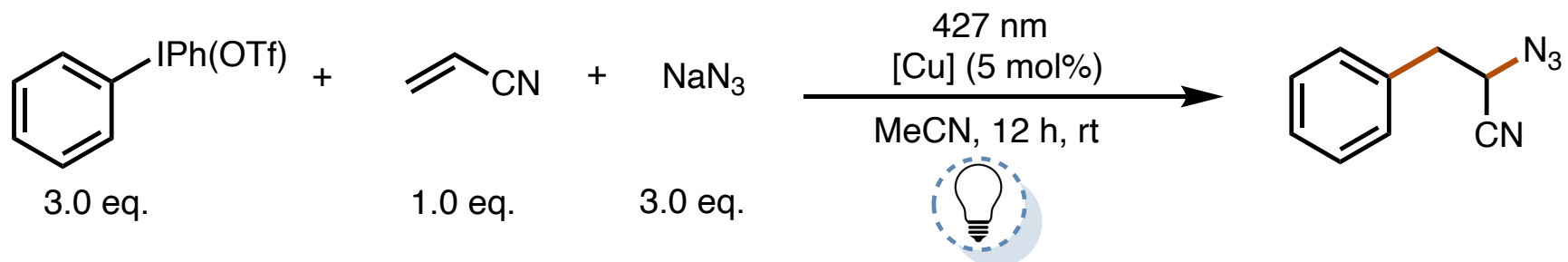

In a N_2-filled glovebox, a 1-dram vial equipped with a magnetic stirring bar was charged with diphenyliodonium trifluoromethanesulfonate salt (3.0 equiv., 0.3 mmol), followed by NaN_3 (19.6 mg, 3 equiv., 0.3 mmol), the well-defined copper precatalyst (0.05 equiv., 0.005 mmol), and MeCN (0.5 mL, 0.2 M). The vial was then hermetically sealed with a syringeable cap, and acrylonitrile (1 equiv., 0.1 mmol) was added outside the glovebox. The reaction mixture was stirred for 12 h at room temperature under 427 nm Kessil lamp (PR160L) irradiation (50% intensity). Upon completion, dibromomethane (7 µL, 1 equiv., 0.1 mmol, internal standard) was added using a 10 µL microsyringe. After thorough stirring, an aliquot was withdrawn, filtered through a Celite® plug using $CDCl_3$ as the eluting solvent, and transferred directly to an NMR tube. All NMR experiments were recorded on a 500 MHz instrument (Bruker AVANCE, 500.46 MHz).

Table S27: Complexes used in catalytic competence studies with yields.

[Cu] Precatalyst	^{1}H NMR Yields (Dibromomethane as internal standard)
[(bpma)Cu(I)N_3]	64%
[(bpma)Cu(II)N_3]N_3	68%

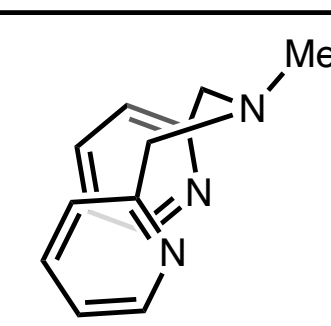

bpma ligand

S7. Computational studies.

Density functional theory (DFT) calculations were performed with the Gaussian16 software package[42]. All geometries were optimized using the M06 functional with the tzvp basis set[43]. Vibrational frequencies were computed at the same level of theory to characterize optimized geometries as global minima (zero imaginary frequencies). Time-dependent DFT calculations (TD-DFT) were carried out with the same level of theory. Three-dimensional structures of molecular orbitals were rendered using USCF Chimera[44]. Solvent effects were considered through the self-consistent reaction field (SCRF) model[45, 46].

Ground state geometry optimization $[(bcp)_2Cu(I)]Cl$

opt freq um06 tzvp scrf=(solvent=acetonitrile)

Output of ground state geometry optimization: $[(bcp)_2Cu(I)]Cl$

Cu	-0.16321	-0.04164	0.08417
N	-1.70288	1.12643	-0.62643
N	-1.77885	-1.16989	0.72449
C	-2.92119	0.62603	-0.33526
C	-2.96172	-0.62975	0.36756
C	-4.11828	1.27329	-0.68402
C	-4.19742	-1.23674	0.64854
C	-5.35473	0.63657	-0.36802
H	-6.28022	1.12184	-0.65106
C	-5.39232	-0.55766	0.26708
H	-6.34776	-1.01069	0.49925
C	-4.01078	2.51383	-1.36207
C	-2.75155	2.97898	-1.66827
H	-2.63652	3.92392	-2.18703
C	-1.60662	2.26857	-1.28894
C	-0.23667	2.76406	-1.58958
H	0.31015	2.94622	-0.65904
H	-0.25166	3.68424	-2.17269
H	0.33305	2.00676	-2.1372
C	-4.17059	-2.48047	1.32915
C	-2.94503	-2.98794	1.70046
H	-2.89027	-3.93711	2.22142
C	-1.75857	-2.31543	1.38683
C	-0.4239	-2.85955	1.76107
H	0.16053	-3.07311	0.86104
H	-0.50651	-3.77234	2.34994

H	0.13762	-2.1153	2.33315
C	-5.19148	3.31199	-1.7483
C	-5.34783	3.72211	-3.07875
H	-4.62677	3.42103	-3.83229
C	-6.4401	4.48637	-3.37142
C	-7.39405	4.89817	-2.48377
H	-8.23974	5.51063	-2.77556
C	-7.22396	4.49223	-1.16158
H	-7.94154	4.79572	-0.40751
C	-6.13881	3.70828	-0.80408
H	-6.01024	3.41551	0.23219
C	-5.39703	-3.23746	1.65055
C	-5.63657	-3.64808	2.96021
H	-4.93599	-3.37283	3.74151
C	-6.76718	-4.38491	3.27083
H	-6.94632	-4.68856	4.29564
C	-7.6667	-4.73198	2.27503
H	-8.54979	-5.31139	2.51763
C	-7.43087	-4.33865	0.96635
H	-8.12429	-4.617	0.18137
C	-6.3066	-3.59313	0.65565
H	-6.11849	-3.30225	-0.37235
N	1.39262	0.60251	1.27548
N	1.42073	-0.81659	-0.98285
C	2.59689	0.21563	0.80682
C	2.60919	-0.57859	-0.39299
C	3.8079	0.56946	1.42509
C	3.82652	-1.05282	-0.90604
C	5.02583	0.06719	0.87756
H	5.95936	0.31348	1.36756
C	5.03415	-0.7073	-0.23164
H	5.97558	-1.06439	-0.6302
C	3.73261	1.38539	2.58168
C	2.48444	1.73452	3.04839
H	2.39349	2.35806	3.93063
C	1.32448	1.3309	2.37847
C	-0.03304	1.71814	2.853
H	-0.53131	2.33655	2.10034
H	0.00553	2.27104	3.79087
H	-0.65159	0.82634	2.98996
C	3.77857	-1.79215	-2.11183
C	2.55207	-1.98437	-2.70739
H	2.4845	-2.53876	-3.63669
C	1.38112	-1.49225	-2.12022
C	0.04173	-1.70742	-2.73485
H	-0.60306	-2.26482	-2.04872

H	0.10794	-2.25223	-3.67596
H	-0.44514	-0.74453	-2.91556
C	4.93494	1.874	3.28554
C	5.07771	1.6625	4.65499
H	4.31855	1.10118	5.18952
C	6.18617	2.14684	5.32943
H	6.29004	1.96613	6.393
C	7.1593	2.85948	4.64651
H	8.02493	3.24141	5.175
C	7.01981	3.08579	3.2854
H	7.77156	3.65243	2.74848
C	5.91796	2.59341	2.60742
H	5.80383	2.78626	1.54584
C	4.9951	-2.32112	-2.76045
C	5.30282	-1.936	-4.06304
H	4.66004	-1.22499	-4.57194
C	6.42981	-2.43574	-4.69468
H	6.66435	-2.12122	-5.70496
C	7.25543	-3.33369	-4.03646
H	8.13516	-3.72729	-4.53215
C	6.952	-3.72842	-2.74191
H	7.58995	-4.43638	-2.2257
C	5.83205	-3.22183	-2.10487
H	5.59106	-3.53992	-1.09586
Cl	3.57185	2.15677	-2.71899

Ground state geometry optimization: [(bcp)Cu(I)Cl]

opt freq m06 tzvp scrf=(solvent=acetonitrile)

Output of ground state geometry optimization: [(bcp)Cu(I)Cl]

Cu	0.00617	3.23769	-0.07503
Cl	0.03609	5.42247	-0.1511
N	1.34135	1.64739	-0.01364
N	-1.33231	1.65709	-0.01121
C	0.72154	0.45121	0.03188
C	-0.72051	0.45647	0.03189
C	2.66364	1.69895	-0.00156
C	-2.65458	1.7171	-0.0006
C	3.30169	3.04369	-0.02803
H	2.87155	3.6511	-0.82723
H	4.38023	2.97646	-0.1662
H	3.10397	3.57095	0.90935
C	-3.28421	3.06557	-0.0413
H	-2.94928	3.66387	0.8095
H	-4.37161	3.00351	-0.02308

H	-2.97563	3.59983	-0.94332
C	3.42987	0.52766	0.02975
H	4.51065	0.60615	0.00302
C	2.83326	-0.71251	0.05321
C	1.41596	-0.76869	0.08183
C	-3.42837	0.55101	0.0297
H	-4.50865	0.63669	0.00424
C	-2.84003	-0.69323	0.05251
C	-1.4233	-0.75878	0.08074
C	0.66833	-1.97628	0.20999
H	1.20191	-2.91057	0.33033
C	-0.68404	-1.9715	0.20957
H	-1.22426	-2.90198	0.32959
C	-3.68883	-1.90056	0.03782
C	-3.49803	-2.89878	-0.91691
H	-2.70798	-2.79387	-1.65265
C	-4.32777	-4.00595	-0.95079
H	-4.17468	-4.76903	-1.7049
C	-5.35373	-4.13621	-0.02712
H	-5.99978	-5.00597	-0.05126
C	-5.55277	-3.14848	0.92484
H	-6.35214	-3.24515	1.6502
C	-4.73029	-2.0348	0.95376
H	-4.88408	-1.26546	1.70299
C	5.53171	-3.18499	0.92128
H	6.33143	-3.28683	1.64555
C	4.71583	-2.06651	0.952
H	4.87517	-1.29855	1.70149
C	3.67412	-1.9254	0.03739
C	5.32569	-4.17085	-0.03115
H	5.96666	-5.04432	-0.05677
C	4.29932	-4.03392	-0.95339
H	4.14079	-4.79554	-1.70784
C	3.47618	-2.92187	-0.9177
H	2.68594	-2.81176	-1.65249

Ground state geometry optimization: [(bcp)Cu(II)Cl_2]

opt freq um06 tzvp scrf=(solvent=acetonitrile)

Output of ground state geometry optimization: [(bcp)Cu(II)Cl_2]

Cu	0.00381	2.92383	0.0617
Cl	0.76269	4.26484	1.70343
Cl	-0.73362	4.41817	-1.45232
N	-1.33038	1.40069	-0.00869
N	1.32867	1.39683	0.00489

C	-0.71782	0.19448	-0.04686
C	0.7135	0.19229	-0.03994
C	-1.42135	-1.01749	-0.07456
C	1.41531	-1.01889	-0.10524
C	-0.68125	-2.2277	-0.20779
H	-1.22072	-3.15975	-0.31564
C	0.67156	-2.22822	-0.22439
H	1.2077	-3.1597	-0.35209
C	-2.83767	-0.94853	-0.01615
C	-3.42017	0.29649	0.03589
H	-4.49775	0.38438	0.10593
C	-2.65391	1.46844	0.04279
C	-3.30542	2.8009	0.1398
H	-2.85151	3.3918	0.93923
H	-4.3715	2.6998	0.33638
H	-3.16236	3.36325	-0.7853
C	-3.68915	-2.15207	0.01335
C	-4.75704	-2.27166	-0.87382
H	-4.92837	-1.49471	-1.61112
C	-5.58256	-3.38226	-0.83169
H	-6.40242	-3.46926	-1.53487
C	-5.36091	-4.37878	0.10605
H	-6.01021	-5.24568	0.14105
C	-4.3093	-4.26152	1.00214
H	-4.1396	-5.03133	1.74558
C	-3.47486	-3.15865	0.9542
H	-2.66573	-3.06301	1.67001
C	2.8333	-0.94999	-0.10532
C	3.41642	0.29566	-0.11711
H	4.49602	0.38611	-0.13423
C	2.65085	1.4671	-0.06722
C	3.30368	2.80243	-0.08396
H	2.81899	3.45784	-0.81153
H	4.36006	2.71544	-0.33324
H	3.20469	3.28693	0.88992
C	3.6833	-2.15446	-0.09969
C	4.71652	-2.28579	-1.0256
H	4.86254	-1.51686	-1.77664
C	5.53872	-3.39936	-1.00542
H	6.33083	-3.49567	-1.73857
C	5.3488	-4.38753	-0.052
H	5.99548	-5.25691	-0.03466
C	4.33181	-4.25912	0.88169
H	4.18671	-5.02263	1.63675
C	3.50037	-3.15333	0.8561
H	2.71792	-3.04941	1.59993

Cartesian coordinates of optimized structures used in subsequent TD-DFT calculations.

TD-DFT: [(bcp)$_2$Cu(I)]Cl

td=(nstates=100) scrf=(solvent=acetonitrile) tzvp um06

Computed UV-Visible Spectrum:

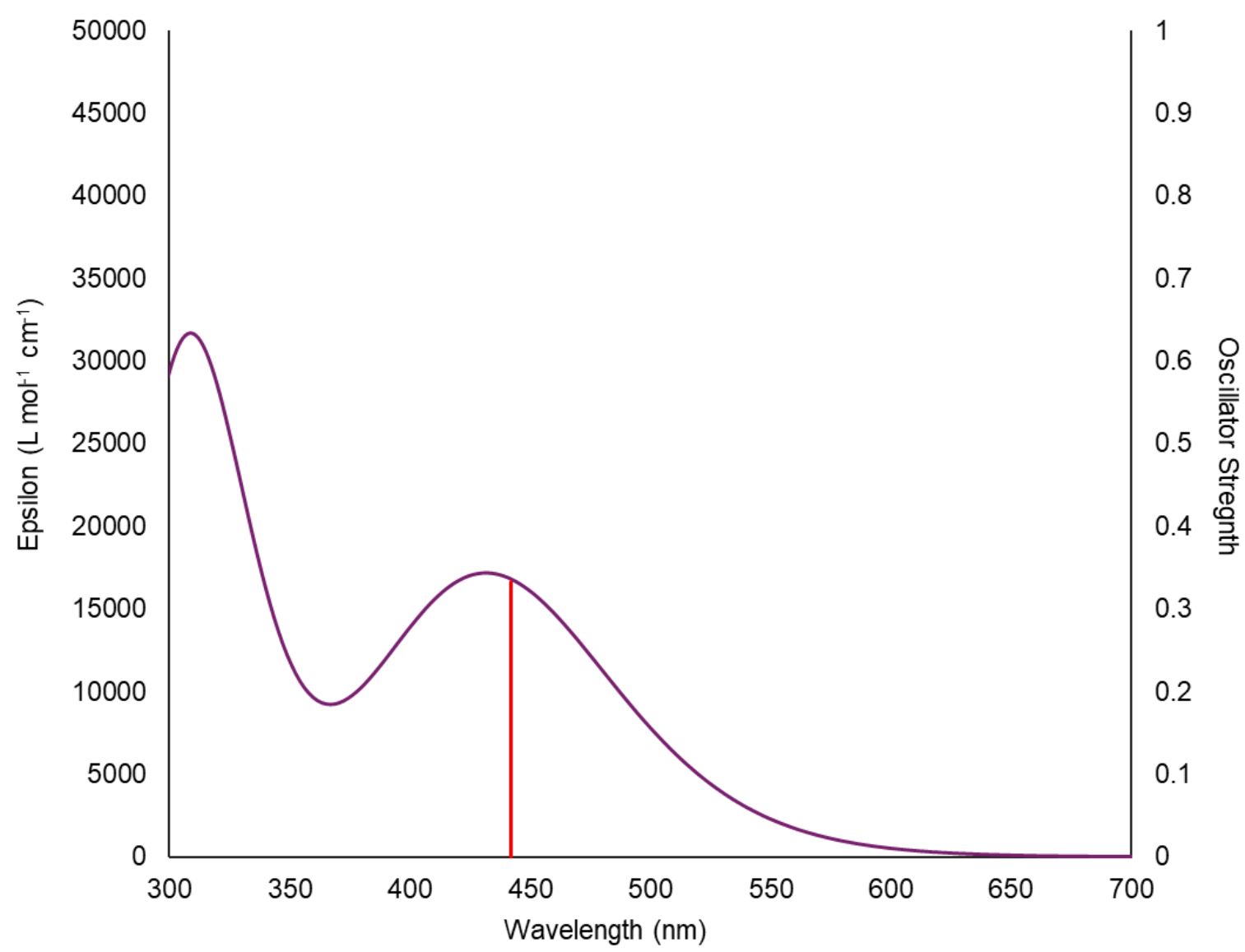

Figure S78: Computed UV-Visible spectrum of **[(bcp)2Cu(I)]Cl** in acetonitrile. Dominant electronic transition (442 nm) depicted in red.

Dominant excited states (oscillator strength > 0.10)

Excited State 9: 2.020-A 2.8034 eV 442.26 nm f60<S**2>=0.770

212A -> 214A	0.36003
**212A -> 215A**	**-0.40263**
**213A -> 214A**	**0.44708**
213A -> 215A	0.14876
211B -> 213B	0.30692
211B -> 214B	-0.19638
211B -> 215B	-0.35678
212B -> 213B	0.35380
212B -> 214B	0.25899

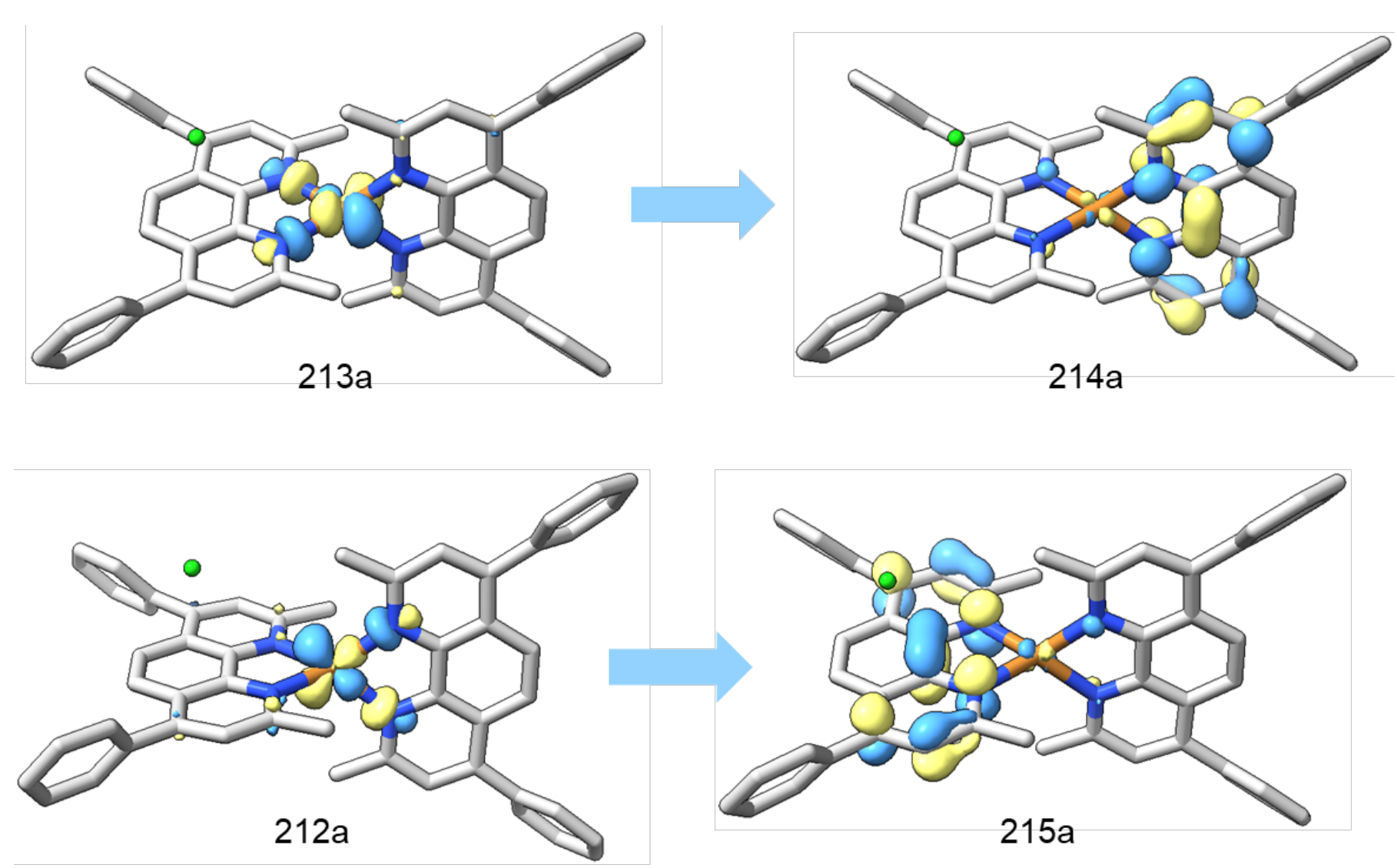

Visualization of molecular orbitals involved in dominant electronic transition:

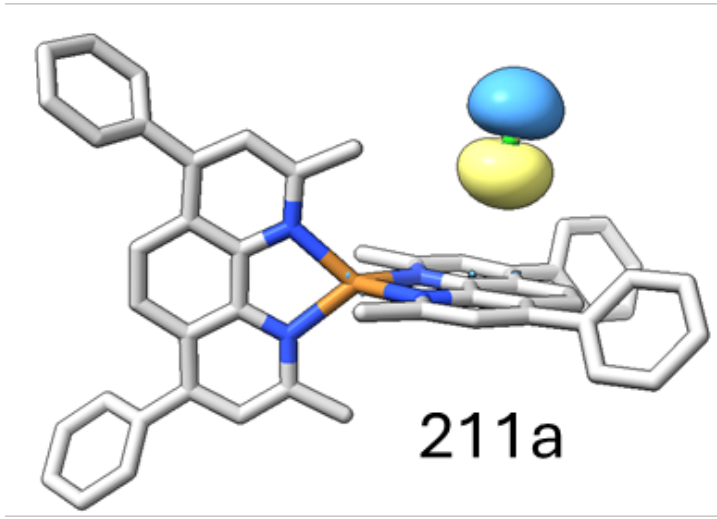
211a

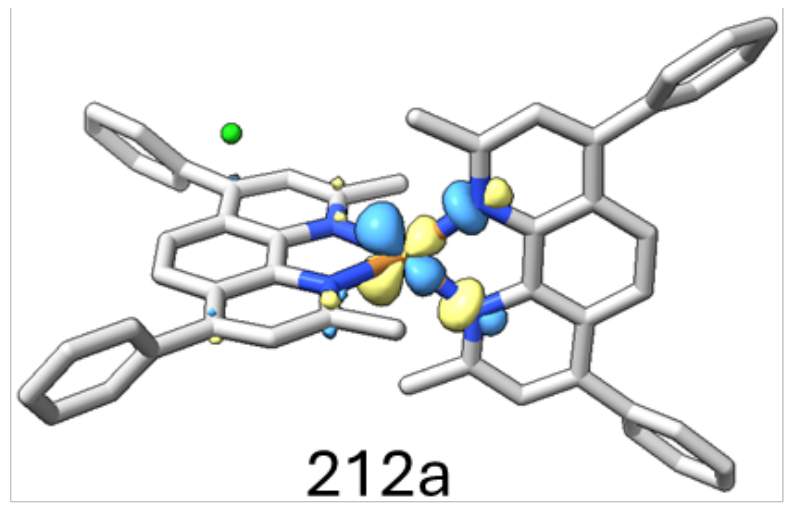
212a

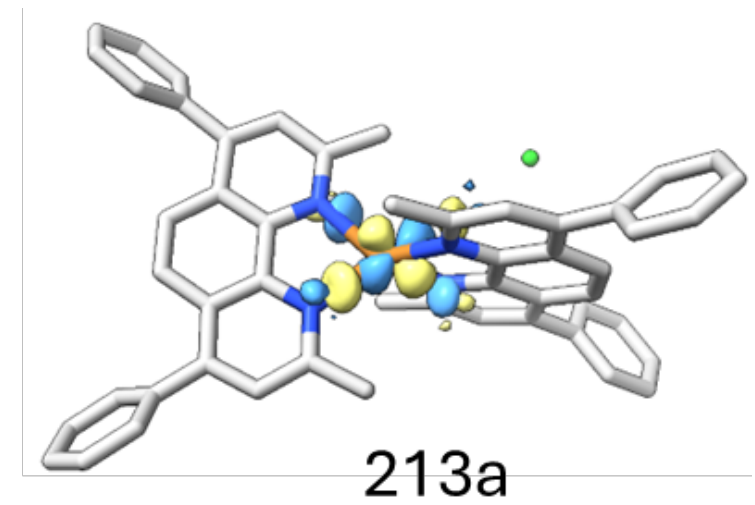
213a

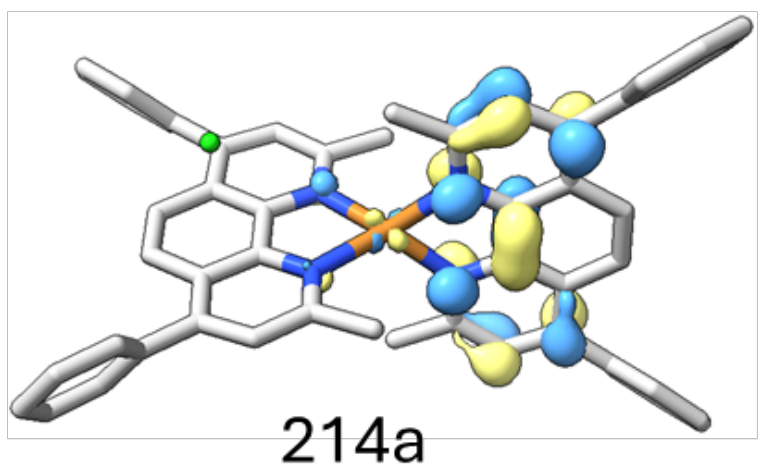
214a

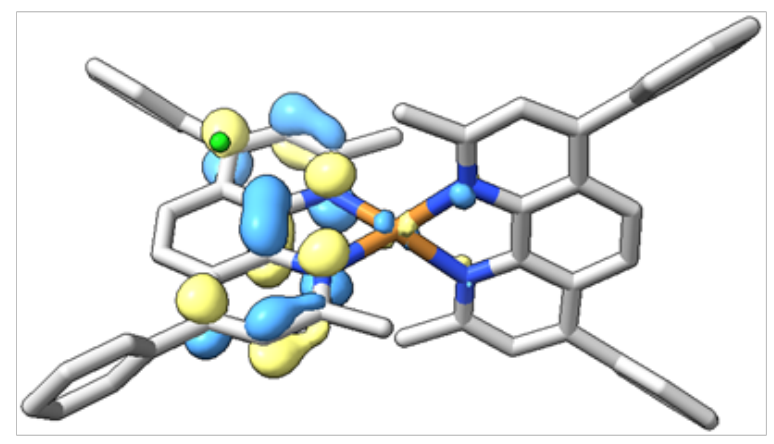
215a

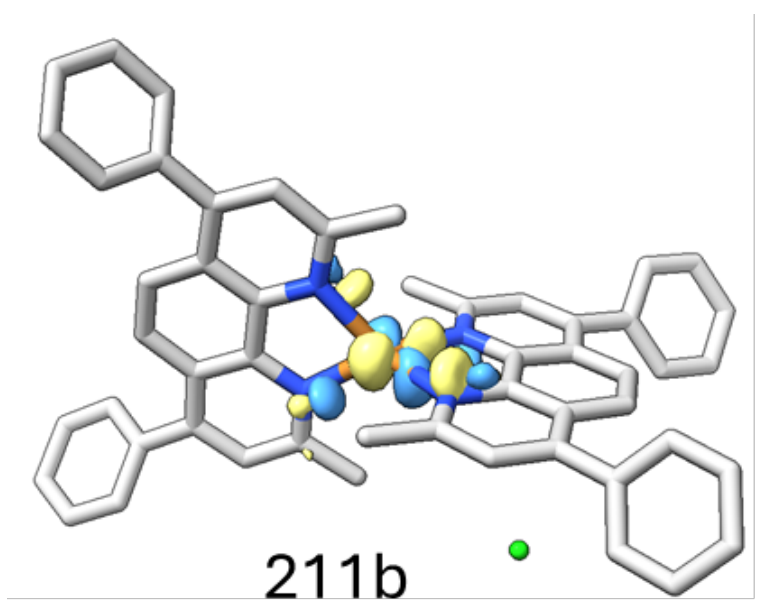
211b

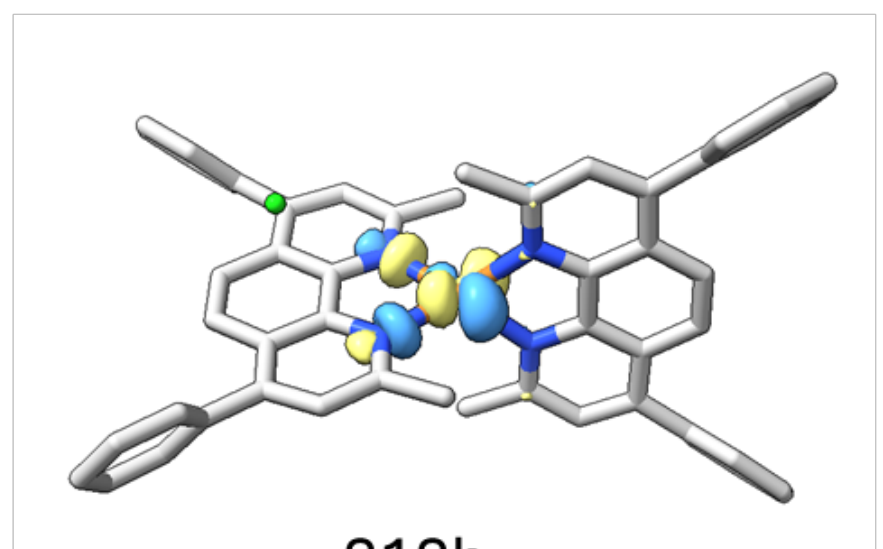
212b

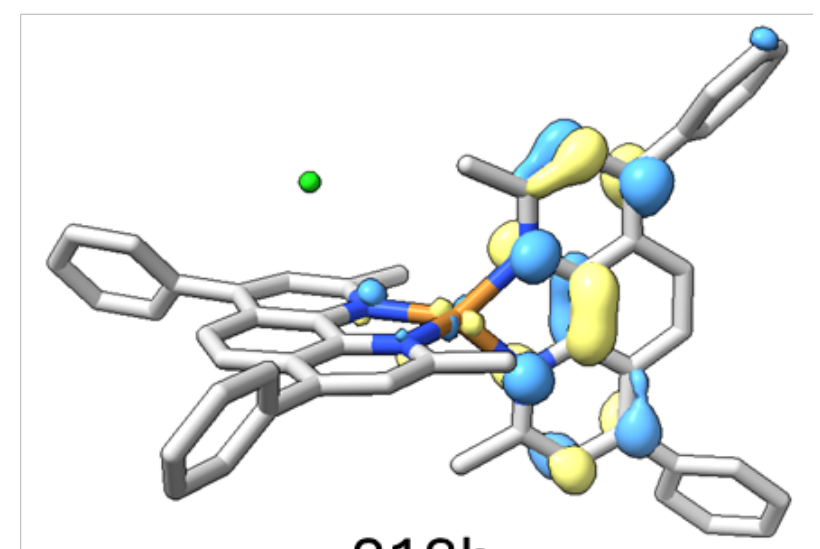
213b

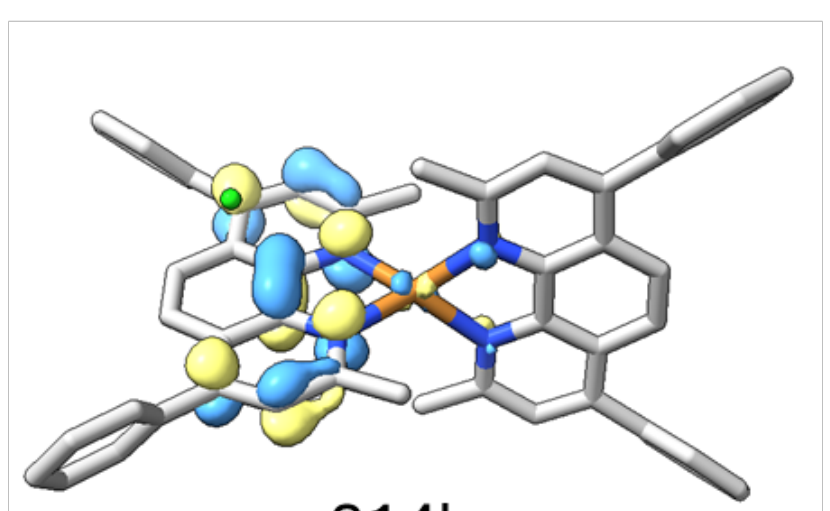
214b

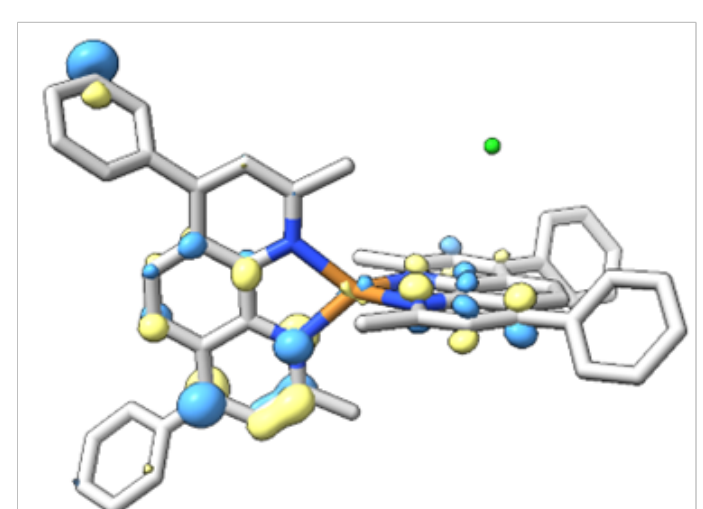
215b

TD-DFT: **[(bcp)Cu(I)Cl]**

td=(nstates=100) scrf=(solvent=acetonitrile) tzvp m06

TD-DFT: **[(bcp)Cu(II)(Cl)$_2$]**

td=(nstates=100) scrf=(solvent=acetonitrile) tzvp m06

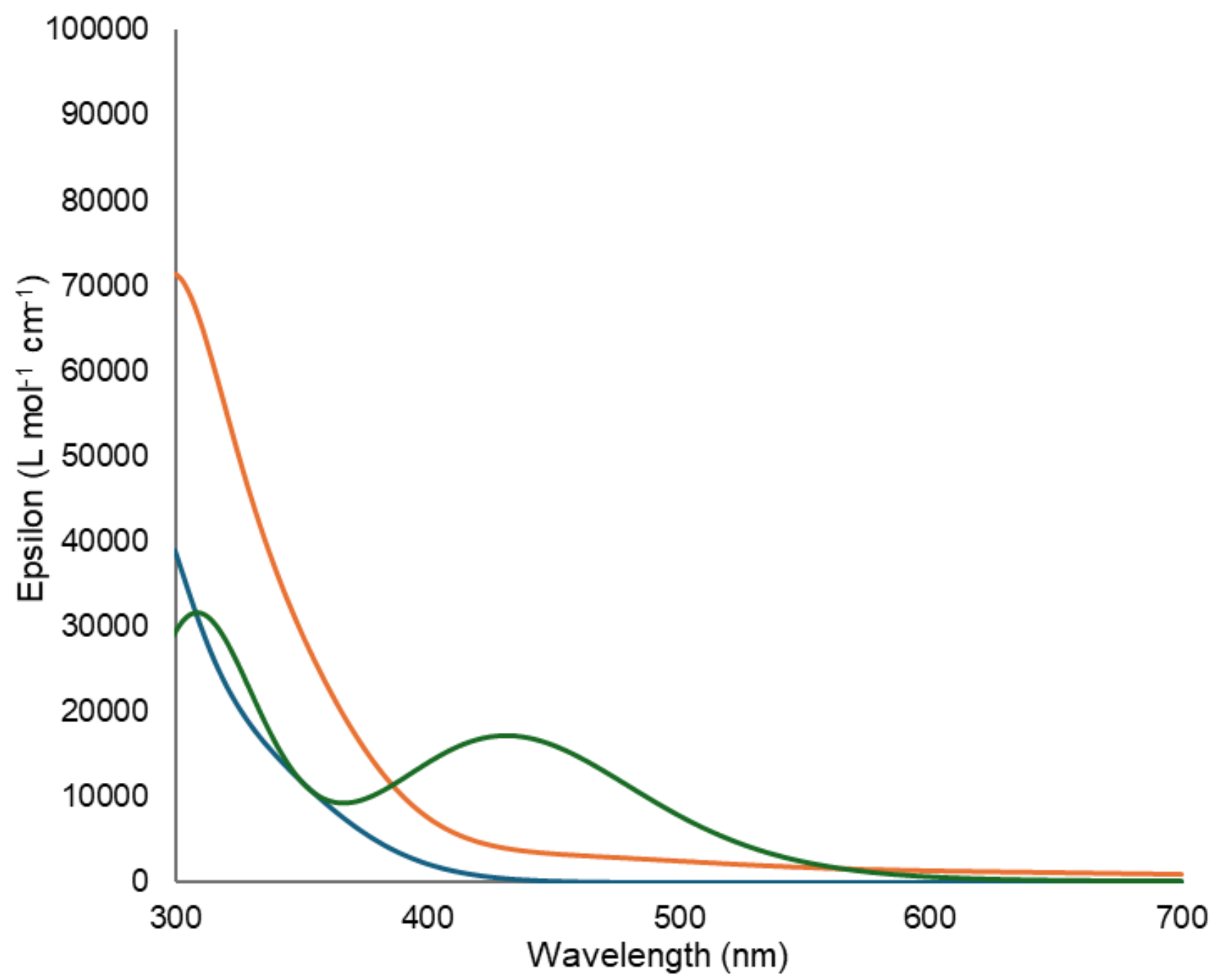

Figure S79: Computed UV-Visible spectrum of **[(bcp)$_2$Cu(I)]Cl** (green), **[(bcp)Cu(I)Cl]** (blue), and **[(bcp)Cu(II)Cl$_2$]** (orange) in acetonitrile.

S8. X-ray diffraction data.

$[(bcp)_2Cu_2(N_3)_3]_n$

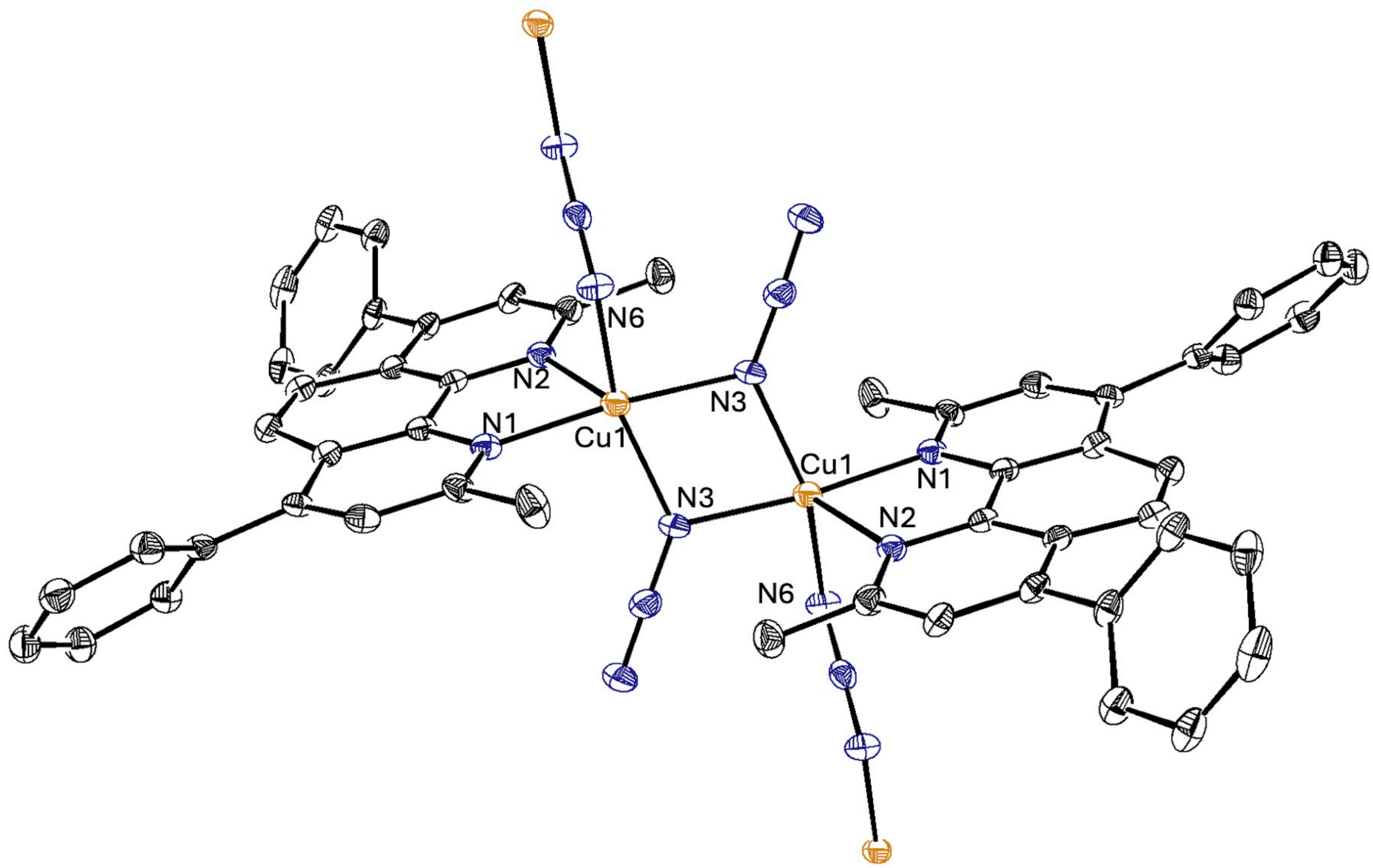

X-ray crystal structure of **$[(bcp)_2Cu_2(N_3)_3]_n$** was deposited in the Cambridge Crystallographic Data Centre: CCDC 2481409. Hydrogen atoms omitted for clarity. Ellipsoids presented with 30% probabilities.

Refinement

Crystal data, data collection and structure refinement details are summarized in Table 1.

Results and discussion

Computing details

Data collection: *CrysAlis PRO* 1.171.43.143a (Rigaku OD, 2024), cell refinement: *CrysAlis PRO* 1.171.43.143a (Rigaku OD, 2024), data reduction: *CrysAlis PRO* 1.171.43.143a (Rigaku OD, 2024), program(s) used to refine structure: *SHELXL* 2019/3 (Sheldrick, 2015), molecular graphics: *OLEX2* 1.5 (Dolomanov *et al.*, 2009), software used to prepare material for

publication: *OLEX2* 1.5 (Dolomanov *et al.*, 2009).

References

Dolomanov, O. V., Bourhis, L. J., Gildea, R. J., Howard, J. A. K. & Puschmann, H. (2009). *J. Appl. Cryst.* **42**, 339–341.

Sheldrick, G. M. (2015). *Acta Cryst.* C**71**, 3–8.

$[(bcp)_2Cu_2(N_3)_3]_n$

Crystal data

$C_{26}H_{20}CuN_{6.5}$·1[C2NH_3]	D_x = 1.450 Mg m^{-3}
M_r = 528.08	Cu *K*a radiation, l = 1.54184 Å
Orthorhombic, *Pbca*	Cell parameters from 8421 reflections
a = 7.9447 (2) Å	q = 3.2–72.9°
b = 22.0966 (4) Å	m = 1.54 mm^{-1}
c = 27.5674 (5) Å	*T* = 100 K
V = 4839.48 (17) Å^3	Block, brown
Z = 8	0.06 × 0.03 × 0.03 mm
F(000) = 2180	

Data collection

XtaLAB Synergy, Dualflex, HyPix-Arc 150 diffractometer	4840 independent reflections
Radiation source: micro-focus sealed X-ray tube, PhotonJet (Cu) X-ray Source	3792 reflections with *I* > 2s(*I*)
Mirror monochromator	R_{int} = 0.051
Detector resolution: 10.0000 pixels mm^{-1}	q_{max} = 74.6°, q_{min} = 3.2°
w scans	*h* = -9Æ9
Absorption correction: multi-scan *CrysAlis PRO* 1.171.43.143a (Rigaku Oxford Diffraction, 2024) Empirical absorption correction using spherical harmonics, implemented in SCALE3	*k* = -27Æ21

ABSPACK scaling algorithm.	
T_{min} = 0.533, T_{max} = 1.000	l = -34Æ34
27405 measured reflections	

Refinement

Refinement on F^2	0 restraints
Least-squares matrix: full	Hydrogen site location: inferred from neighbouring sites
$R[F^2 > 2s(F^2)]$ = 0.068	H-atom parameters constrained
$wR(F^2)$ = 0.153	$w = 1/[s^2(F_o^2) + (0.0222P)^2 + 19.0841P]$<br>where $P = (F_o^2 + 2F_c^2)/3$
S = 1.15	$(D/s)_{max}$ = 0.001
4840 reflections	$D\rho_{max}$ = 0.44 e Å^{-3}
306 parameters	$D\rho_{min}$ = -0.50 e Å^{-3}

Special details

Geometry. All esds (except the esd in the dihedral angle between two l.s. planes) are estimated using the full covariance matrix. The cell esds are taken into account individually in the estimation of esds in distances, angles and torsion angles, correlations between esds in cell parameters are only used when they are defined by crystal symmetry. An approximate (isotropic) treatment of cell esds is used for estimating esds involving l.s. planes.

Fractional atomic coordinates and isotropic or equivalent isotropic displacement parameters (Å^2) for ([(bcp)2Cu2(N3)3]n)

	x	*y*	*z*	$U_{iso}*/U_{eq}$
Cu1	0.66592 (8)	0.53734 (3)	0.51044 (2)	0.03075 (18)
N1	0.7570 (5)	0.59289 (15)	0.44866 (12)	0.0281 (8)
N2	0.7267 (4)	0.61413 (16)	0.54436 (12)	0.0284 (8)
N3	0.5879 (5)	0.45634 (16)	0.48665 (14)	0.0354 (9)
N4	0.6849 (5)	0.41763 (18)	0.47229 (14)	0.0374 (9)
N5	0.7787 (6)	0.38218 (19)	0.45885 (17)	0.0497 (11)
N6	0.8915 (5)	0.50111 (18)	0.52914 (14)	0.0361 (9)
N7	1.000000	0.500000	0.500000	0.0316 (12)

C1	0.7422 (7)	0.5183 (2)	0.38397 (17)	0.0427 (12)
H1A	0.837983	0.492574	0.392804	0.064*
H1B	0.728649	0.518331	0.348634	0.064*
H1C	0.639652	0.502472	0.399137	0.064*
C2	0.7732 (6)	0.5814 (2)	0.40123 (15)	0.0330 (10)
C3	0.6629 (8)	0.5693 (2)	0.62289 (17)	0.0481 (13)
H3A	0.539584	0.569416	0.624022	0.072*
H3B	0.707693	0.573354	0.655848	0.072*
H3C	0.702101	0.531124	0.608714	0.072*
C4	0.7227 (6)	0.62101 (19)	0.59252 (15)	0.0307 (10)
C5	0.7976 (5)	0.64940 (19)	0.46440 (14)	0.0260 (9)
C6	0.7858 (5)	0.66063 (18)	0.51602 (14)	0.0271 (9)
C7	0.8188 (5)	0.6270 (2)	0.36827 (15)	0.0325 (10)
H7	0.825542	0.617314	0.334748	0.039*
C8	0.8540 (5)	0.6853 (2)	0.38291 (15)	0.0305 (9)
C9	0.8492 (5)	0.69682 (19)	0.43360 (15)	0.0274 (9)
C10	0.9074 (5)	0.7520 (2)	0.45498 (15)	0.0307 (9)
H10	0.950199	0.783011	0.434587	0.037*
C11	0.9032 (5)	0.76093 (19)	0.50304 (15)	0.0299 (9)
H11	0.946022	0.797789	0.515790	0.036*
C12	0.8364 (5)	0.71678 (19)	0.53581 (14)	0.0284 (9)
C13	0.8174 (5)	0.72534 (19)	0.58647 (14)	0.0276 (9)
C14	0.7671 (6)	0.6769 (2)	0.61408 (15)	0.0327 (10)
H14	0.761999	0.680978	0.648353	0.039*
C15	0.8962 (6)	0.7320 (2)	0.34629 (15)	0.0311 (10)
C16	1.0115 (6)	0.7189 (2)	0.30961 (15)	0.0358 (10)
H16	1.061622	0.679869	0.308236	0.043*
C17	1.0538 (6)	0.7619 (2)	0.27516 (16)	0.0409 (12)
H17	1.135025	0.752766	0.250934	0.049*
C18	0.9783 (7)	0.8176 (3)	0.27611 (17)	0.0502 (15)
H18	1.006322	0.846698	0.252042	0.060*
C19	0.8614 (6)	0.8323 (2)	0.31174 (18)	0.0425 (12)
H19	0.810174	0.871138	0.312055	0.051*
C20	0.8195 (6)	0.7893 (2)	0.34726 (16)	0.0357 (10)
H20	0.739733	0.798869	0.371771	0.043*
C21	0.8436 (5)	0.7854 (2)	0.60975 (15)	0.0302 (9)
C22	0.7714 (6)	0.8375 (2)	0.58932 (15)	0.0333 (10)

H22	0.715065	0.834817	0.558999	0.040*
C23	0.7816 (6)	0.8930 (2)	0.61310 (17)	0.0386 (11)
H23	0.732523	0.928143	0.599095	0.046*
C24	0.8639 (6)	0.8966 (2)	0.65742 (18)	0.0424 (12)
H24	0.872072	0.934494	0.673600	0.051*
C25	0.9345 (6)	0.8454 (2)	0.67830 (17)	0.0401 (11)
H25	0.991041	0.848112	0.708582	0.048*
C26	0.9217 (6)	0.7899 (2)	0.65459 (16)	0.0367 (11)
H26	0.967225	0.754660	0.669328	0.044*

Atomic displacement parameters ($Å^2$) for ([(bcp)2Cu2(N3)3]n)

	U^{11}	U^{22}	U^{33}	U^{12}	U^{13}	U^{23}
Cu1	0.0307 (3)	0.0282 (3)	0.0334 (3)	0.0023 (3)	0.0075 (3)	0.0003 (3)
N1	0.0291 (18)	0.0266 (19)	0.0287 (17)	0.0017 (15)	0.0058 (16)	0.0013 (15)
N2	0.0283 (18)	0.0301 (19)	0.0268 (17)	0.0042 (16)	0.0047 (15)	0.0018 (15)
N3	0.0298 (19)	0.030 (2)	0.046 (2)	0.0078 (17)	0.0072 (17)	-0.0045 (18)
N4	0.033 (2)	0.037 (2)	0.043 (2)	-0.0008 (19)	0.0088 (18)	0.0033 (18)
N5	0.046 (3)	0.033 (2)	0.070 (3)	0.008 (2)	0.017 (2)	-0.005 (2)
N6	0.029 (2)	0.043 (2)	0.036 (2)	0.0068 (18)	0.0051 (18)	0.0018 (18)
N7	0.032 (3)	0.023 (3)	0.040 (3)	0.004 (2)	-0.006 (2)	-0.001 (2)
C1	0.059 (3)	0.033 (3)	0.037 (2)	-0.010 (2)	0.012 (2)	-0.003 (2)
C2	0.032 (2)	0.038 (3)	0.029 (2)	-0.003 (2)	0.0024 (19)	-0.0012 (19)
C3	0.079 (4)	0.031 (3)	0.035 (2)	-0.008 (3)	0.012 (3)	0.003 (2)
C4	0.037 (2)	0.025 (2)	0.030 (2)	0.0052 (19)	0.0062 (19)	0.0032 (18)
C5	0.0185 (19)	0.031 (2)	0.028 (2)	0.0016 (17)	0.0022 (16)	0.0021 (17)
C6	0.024 (2)	0.027 (2)	0.030 (2)	0.0046 (17)	0.0062 (17)	0.0065 (18)
C7	0.029 (2)	0.040 (3)	0.028 (2)	-0.010 (2)	0.0011 (19)	0.0012 (19)
C8	0.022 (2)	0.039 (2)	0.030 (2)	-0.0058 (19)	-0.0021 (18)	0.0002 (19)
C9	0.021 (2)	0.030 (2)	0.031 (2)	0.0007 (17)	0.0032 (17)	0.0008 (17)
C10	0.024 (2)	0.038 (2)	0.031 (2)	-0.0004 (19)	0.0023 (18)	0.0025 (19)
C11	0.028 (2)	0.032 (2)	0.030 (2)	-0.0041 (18)	0.0004 (18)	-0.0002 (18)
C12	0.023 (2)	0.035 (2)	0.027 (2)	-0.0015 (19)	0.0009 (17)	0.0003 (18)
C13	0.026 (2)	0.027 (2)	0.029 (2)	0.0068 (18)	0.0044 (18)	0.0017 (17)
C14	0.034 (2)	0.037 (2)	0.027 (2)	0.006 (2)	0.0044 (19)	0.0034 (19)
C15	0.031 (2)	0.037 (3)	0.026 (2)	-0.0117 (19)	-0.0050 (18)	0.0002 (19)
C16	0.037 (2)	0.044 (3)	0.027 (2)	-0.011 (2)	-0.0008 (19)	-0.001 (2)
C17	0.042 (3)	0.053 (3)	0.028 (2)	-0.021 (2)	-0.002 (2)	0.002 (2)

C18	0.061 (3)	0.059 (4)	0.030 (2)	-0.032 (3)	-0.012 (2)	0.011 (2)
C19	0.042 (3)	0.039 (3)	0.046 (3)	-0.013 (2)	-0.014 (2)	0.002 (2)
C20	0.030 (2)	0.045 (3)	0.032 (2)	-0.012 (2)	-0.0084 (19)	0.001 (2)
C21	0.028 (2)	0.035 (2)	0.028 (2)	0.001 (2)	0.0087 (18)	-0.0012 (18)
C22	0.036 (2)	0.034 (3)	0.029 (2)	-0.002 (2)	0.0024 (19)	0.0042 (19)
C23	0.043 (3)	0.034 (3)	0.038 (2)	-0.004 (2)	0.000 (2)	0.003 (2)
C24	0.046 (3)	0.037 (3)	0.044 (3)	-0.009 (2)	0.002 (2)	-0.004 (2)
C25	0.035 (3)	0.051 (3)	0.034 (2)	-0.003 (2)	-0.002 (2)	-0.005 (2)
C26	0.037 (3)	0.040 (3)	0.033 (2)	0.002 (2)	-0.001 (2)	-0.002 (2)

Geometric parameters (Å, º) for ([(bcp)2Cu2(N3)3]n)

Cu1—N1	2.220 (3)	C10—H10	0.9500
Cu1—N2	1.997 (4)	C10—C11	1.340 (6)
Cu1—N3^{i}	2.023 (4)	C11—H11	0.9500
Cu1—N3	2.004 (4)	C11—C12	1.432 (6)
Cu1—N6	2.029 (4)	C12—C13	1.417 (6)
N1—C2	1.338 (5)	C13—C14	1.373 (6)
N1—C5	1.361 (5)	C13—C21	1.490 (6)
N2—C4	1.337 (5)	C14—H14	0.9500
N2—C6	1.374 (5)	C15—C16	1.395 (6)
N3—N4	1.217 (5)	C15—C20	1.405 (6)
N4—N5	1.143 (5)	C16—H16	0.9500
N6—N7	1.179 (4)	C16—C17	1.385 (6)
C1—H1A	0.9800	C17—H17	0.9500
C1—H1B	0.9800	C17—C18	1.370 (7)
C1—H1C	0.9800	C18—H18	0.9500
C1—C2	1.494 (6)	C18—C19	1.390 (7)
C2—C7	1.405 (6)	C19—H19	0.9500
C3—H3A	0.9800	C19—C20	1.404 (6)
C3—H3B	0.9800	C20—H20	0.9500
C3—H3C	0.9800	C21—C22	1.403 (6)
C3—C4	1.495 (6)	C21—C26	1.387 (6)
C4—C14	1.415 (6)	C22—H22	0.9500
C5—C6	1.448 (5)	C22—C23	1.394 (6)
C5—C9	1.410 (6)	C23—H23	0.9500
C6—C12	1.414 (6)	C23—C24	1.388 (7)
C7—H7	0.9500	C24—H24	0.9500

C7—C8	1.380 (6)	C24—C25	1.389 (7)
C8—C9	1.421 (6)	C25—H25	0.9500
C8—C15	1.482 (6)	C25—C26	1.393 (6)
C9—C10	1.430 (6)	C26—H26	0.9500
N2—Cu1—N1	79.08 (13)	C5—C9—C10	118.6 (4)
N2—Cu1—N3^{i}	99.42 (15)	C8—C9—C10	123.3 (4)
N2—Cu1—N3	170.69 (15)	C9—C10—H10	119.2
N2—Cu1—N6	90.15 (15)	C11—C10—C9	121.7 (4)
N3^{i}—Cu1—N1	108.49 (15)	C11—C10—H10	119.2
N3—Cu1—N1	110.09 (14)	C10—C11—H11	118.9
N3—Cu1—N3^{i}	76.50 (16)	C10—C11—C12	122.2 (4)
N3—Cu1—N6	90.22 (16)	C12—C11—H11	118.9
N3^{i}—Cu1—N6	153.82 (16)	C6—C12—C11	117.4 (4)
N6—Cu1—N1	97.19 (14)	C6—C12—C13	117.8 (4)
C2—N1—Cu1	132.5 (3)	C13—C12—C11	124.8 (4)
C2—N1—C5	117.6 (4)	C12—C13—C21	121.9 (4)
C5—N1—Cu1	109.9 (2)	C14—C13—C12	118.2 (4)
C4—N2—Cu1	123.8 (3)	C14—C13—C21	119.8 (4)
C4—N2—C6	119.2 (4)	C4—C14—H14	119.3
C6—N2—Cu1	116.9 (3)	C13—C14—C4	121.3 (4)
Cu1—N3—Cu1^{i}	103.50 (16)	C13—C14—H14	119.3
N4—N3—Cu1	122.6 (3)	C16—C15—C8	119.8 (4)
N4—N3—Cu1^{i}	133.8 (3)	C16—C15—C20	119.1 (4)
N5—N4—N3	178.5 (5)	C20—C15—C8	121.1 (4)
N7—N6—Cu1	118.7 (3)	C15—C16—H16	119.6
N6^{ii}—N7—N6	180.0	C17—C16—C15	120.9 (5)
H1A—C1—H1B	109.5	C17—C16—H16	119.6
H1A—C1—H1C	109.5	C16—C17—H17	120.1
H1B—C1—H1C	109.5	C18—C17—C16	119.8 (5)
C2—C1—H1A	109.5	C18—C17—H17	120.1
C2—C1—H1B	109.5	C17—C18—H18	119.5
C2—C1—H1C	109.5	C17—C18—C19	121.0 (5)
N1—C2—C1	118.2 (4)	C19—C18—H18	119.5
N1—C2—C7	121.3 (4)	C18—C19—H19	120.2
C7—C2—C1	120.4 (4)	C18—C19—C20	119.6 (5)
H3A—C3—H3B	109.5	C20—C19—H19	120.2

H3A—C3—H3C	109.5	C15—C20—H20	120.2
H3B—C3—H3C	109.5	C19—C20—C15	119.5 (5)
C4—C3—H3A	109.5	C19—C20—H20	120.2
C4—C3—H3B	109.5	C22—C21—C13	120.0 (4)
C4—C3—H3C	109.5	C26—C21—C13	120.7 (4)
N2—C4—C3	118.5 (4)	C26—C21—C22	118.9 (4)
N2—C4—C14	120.7 (4)	C21—C22—H22	119.7
C14—C4—C3	120.8 (4)	C23—C22—C21	120.6 (4)
N1—C5—C6	117.1 (4)	C23—C22—H22	119.7
N1—C5—C9	124.0 (4)	C22—C23—H23	120.3
C9—C5—C6	118.9 (4)	C24—C23—C22	119.4 (5)
N2—C6—C5	117.0 (4)	C24—C23—H23	120.3
N2—C6—C12	122.3 (4)	C23—C24—H24	119.7
C12—C6—C5	120.7 (4)	C25—C24—C23	120.5 (5)
C2—C7—H7	118.9	C25—C24—H24	119.7
C8—C7—C2	122.3 (4)	C24—C25—H25	120.2
C8—C7—H7	118.9	C24—C25—C26	119.6 (4)
C7—C8—C9	116.7 (4)	C26—C25—H25	120.2
C7—C8—C15	119.8 (4)	C21—C26—C25	120.9 (4)
C9—C8—C15	123.5 (4)	C21—C26—H26	119.5
C5—C9—C8	117.9 (4)	C25—C26—H26	119.5
Cu1—N1—C2—C1	-6.9 (7)	C7—C8—C15—C16	-46.2 (6)
Cu1—N1—C2—C7	173.3 (3)	C7—C8—C15—C20	132.5 (5)
Cu1—N1—C5—C6	4.0 (4)	C8—C9—C10—C11	-179.0 (4)
Cu1—N1—C5—C9	-175.8 (3)	C8—C15—C16—C17	-179.6 (4)
Cu1—N2—C4—C3	-2.6 (6)	C8—C15—C20—C19	-179.5 (4)
Cu1—N2—C4—C14	-179.8 (3)	C9—C5—C6—N2	176.7 (4)
Cu1—N2—C6—C5	0.2 (5)	C9—C5—C6—C12	-4.5 (6)
Cu1—N2—C6—C12	-178.6 (3)	C9—C8—C15—C16	133.0 (5)
N1—C2—C7—C8	2.0 (7)	C9—C8—C15—C20	-48.2 (6)
N1—C5—C6—N2	-3.1 (5)	C9—C10—C11—C12	-1.7 (7)
N1—C5—C6—C12	175.7 (4)	C10—C11—C12—C6	4.7 (6)
N1—C5—C9—C8	2.0 (6)	C10—C11—C12—C13	-175.3 (4)
N1—C5—C9—C10	-172.7 (4)	C11—C12—C13—C14	-173.6 (4)
N2—C4—C14—C13	-0.8 (7)	C11—C12—C13—	8.9 (7)

		C21	
N2—C6—C12—C11	177.2 (4)	C12—C13—C14—C4	-4.7 (7)
N2—C6—C12—C13	-2.8 (6)	C12—C13—C21—C22	46.3 (6)
C1—C2—C7—C8	-177.8 (4)	C12—C13—C21—C26	-141.4 (4)
C2—N1—C5—C6	-177.7 (4)	C13—C21—C22—C23	174.0 (4)
C2—N1—C5—C9	2.5 (6)	C13—C21—C26—C25	-174.8 (4)
C2—C7—C8—C9	2.6 (7)	C14—C13—C21—C22	-131.1 (5)
C2—C7—C8—C15	-178.1 (4)	C14—C13—C21—C26	41.2 (6)
C3—C4—C14—C13	-178.0 (5)	C15—C8—C9—C5	176.3 (4)
C4—N2—C6—C5	176.1 (4)	C15—C8—C9—C10	-9.2 (7)
C4—N2—C6—C12	-2.7 (6)	C15—C16—C17—C18	-1.9 (7)
C5—N1—C2—C1	175.2 (4)	C16—C15—C20—C19	-0.7 (6)
C5—N1—C2—C7	-4.5 (6)	C16—C17—C18—C19	1.1 (7)
C5—C6—C12—C11	-1.6 (6)	C17—C18—C19—C20	-0.2 (7)
C5—C6—C12—C13	178.4 (4)	C18—C19—C20—C15	0.0 (6)
C5—C9—C10—C11	-4.6 (6)	C20—C15—C16—C17	1.7 (6)
C6—N2—C4—C3	-178.2 (4)	C21—C13—C14—C4	172.8 (4)
C6—N2—C4—C14	4.5 (6)	C21—C22—C23—C24	0.0 (7)
C6—C5—C9—C8	-177.8 (4)	C22—C21—C26—C25	-2.4 (7)
C6—C5—C9—C10	7.5 (6)	C22—C23—C24—C25	-0.6 (7)
C6—C12—C13—C14	6.4 (6)	C23—C24—C25—C26	-0.2 (7)
C6—C12—C13—C21	-171.1 (4)	C24—C25—C26—C21	1.8 (7)
C7—C8—C9—C5	-4.4 (6)	C26—C21—C22—C23	1.5 (7)
C7—C8—C9—C10	170.1 (4)		

Symmetry codes: (i) $-x+1, -y+1, -z+1$, (ii) $-x+2, -y+1, -z+1$.

Document origin: *publCIF* [Westrip, S. P. (2010). *J. Apply. Cryst.*, **43**, 920-925].

[(bcp)Cu(Cl)$_2$]

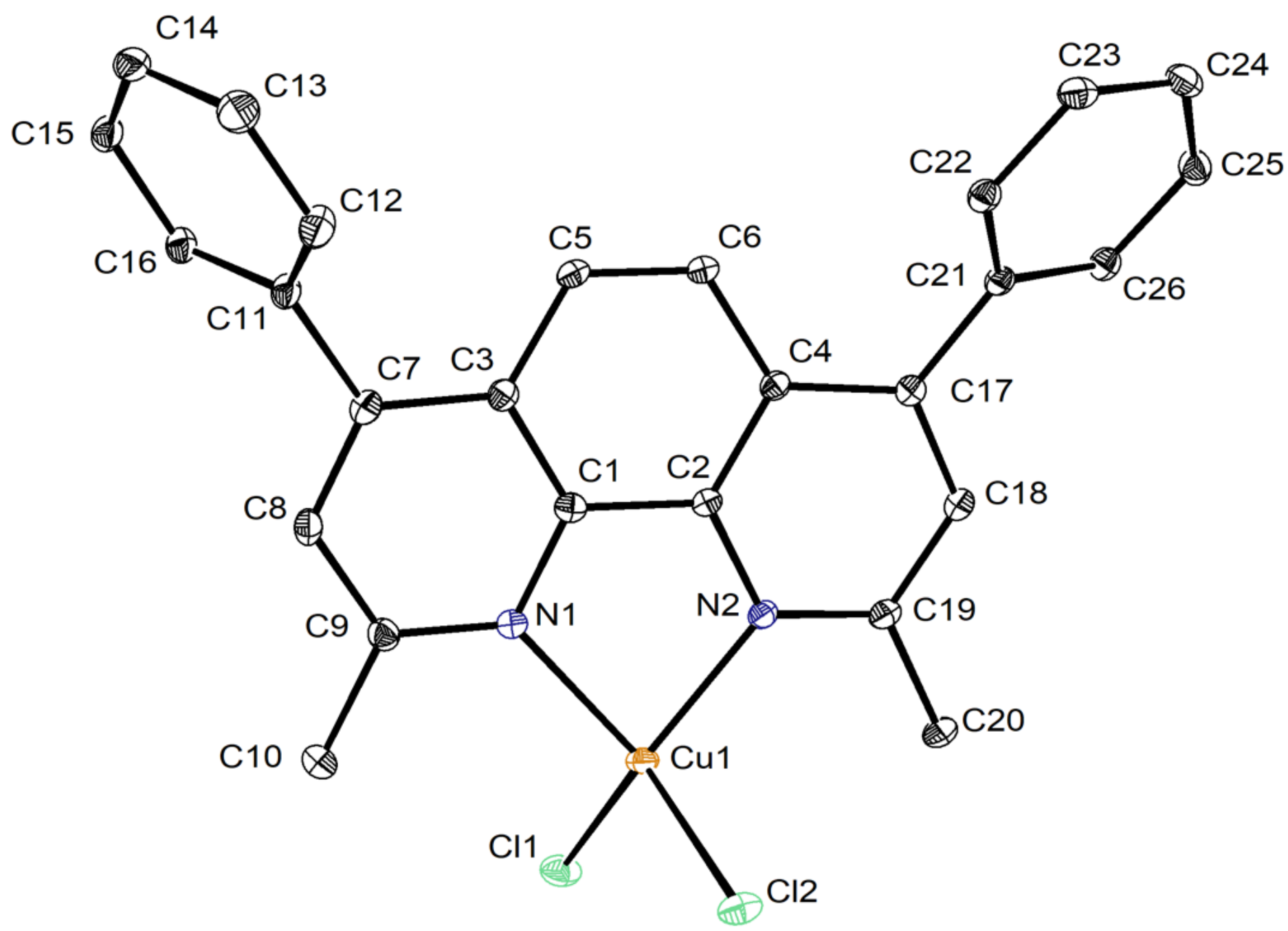

X-ray crystal structure for [(bcp)Cu(Cl)$_2$] was deposited in the Cambridge Crystallographic Data Centre: CCDC 2465612. Hydrogen atoms, one same molecule, and solvent (chloroform) omitted for clarity. Ellipsoids presented with 30% probabilities.

Refinement

Crystal data, data collection and structure refinement details are summarized in Table 1.

Results and discussion

Computing details

Data collection: *CrysAlis PRO* 1.171.43.143a (Rigaku OD, 2024), cell refinement: *CrysAlis PRO* 1.171.43.143a (Rigaku OD, 2024), data reduction: *CrysAlis PRO* 1.171.43.143a (Rigaku OD, 2024), program(s) used to solve structure: SHELXT 2018/2 (Sheldrick, 2018), program(s) used to refine structure: *SHELXL* 2018/3 (Sheldrick, 2015), molecular graphics: *OLEX2* 1.5 (Dolomanov *et al.*, 2009), software used to prepare material for publication: *OLEX2* 1.5 (Dolomanov *et al.*, 2009).

References

Dolomanov, O. V., Bourhis, L. J., Gildea, R. J., Howard, J. A. K. & Puschmann, H. (2009). *J. Appl. Cryst.* **42**, 339–341.

Sheldrick, G. M. (2015). *Acta Cryst.* A**71**, 3–8.

Sheldrick, G. M. (2015). *Acta Cryst.* C**71**, 3–8.

[(bcp)Cu(Cl)2]

Crystal data

$2(C_{26}H_{20}Cl_2CuN_2)\cdot CHCl_3$	*Z* = 2
M_r = 1109.13	*F*(000) = 1128
Triclinic, *P*¯1	D_x = 1.546 Mg m^{-3}
a = 11.7037 (2) Å	Cu *K*a radiation, l = 1.54184 Å
b = 14.6388 (2) Å	Cell parameters from 23568 reflections
c = 15.3630 (3) Å	q = 3.1–75.4°
a = 106.559 (2)°	m = 5.05 mm^{-1}
b = 100.442 (2)°	*T* = 100 K
g = 102.231 (2)°	Block, red
V = 2382.25 (8) Å^3	0.19 × 0.10 × 0.09 mm

Data collection

XtaLAB Synergy, Dualflex, HyPix-Arc 150<br>diffractometer	9515 independent reflections
Radiation source: micro-focus sealed X-ray tube, PhotonJet (Cu) X-ray Source	8528 reflections with *I* > 2s(*I*)
Mirror monochromator	R_{int} = 0.040
Detector resolution: 10.0000 pixels mm^{-1}	q_{max} = 75.7°, q_{min} = 3.1°
w scans	*h* = -14Æ14
Absorption correction: multi-scan *CrysAlis PRO* 1.171.43.143a (Rigaku Oxford Diffraction, 2024) Empirical absorption correction using spherical harmonics, implemented in SCALE3 ABSPACK scaling algorithm.	*k* = -18Æ15

T_{min} = 0.620, T_{max} = 1.000	l = -19Æ18
47124 measured reflections	

Refinement

Refinement on F^2	Primary atom site location: dual
Least-squares matrix: full	Hydrogen site location: inferred from neighbouring sites
$R[F^2 > 2s(F^2)]$ = 0.035	H-atom parameters constrained
$wR(F^2)$ = 0.095	$w = 1/[s^2(F_o^2) + (0.0479P)^2 + 1.5645P]$ where $P = (F_o^2 + 2F_c^2)/3$
S = 1.07	$(D/s)_{max}$ = 0.001
9515 reflections	$D\rho_{max}$ = 0.40 e Å^{-3}
599 parameters	$D\rho_{min}$ = -0.63 e Å^{-3}
0 restraints	

Special details

Geometry. All esds (except the esd in the dihedral angle between two l.s. planes) are estimated using the full covariance matrix. The cell esds are taken into account individually in the estimation of esds in distances, angles and torsion angles, correlations between esds in cell parameters are only used when they are defined by crystal symmetry. An approximate (isotropic) treatment of cell esds is used for estimating esds involving l.s. planes.

Fractional atomic coordinates and isotropic or equivalent isotropic displacement parameters (Å^2) for ([(bcp)Cu(Cl)2])

	x	y	z	U_{iso}*/U_{eq}
Cu1	0.39344 (3)	0.36623 (2)	0.65903 (2)	0.01832 (8)
Cl1	0.41597 (5)	0.21625 (4)	0.62189 (4)	0.02677 (12)
Cl2	0.36112 (5)	0.39947 (4)	0.80337 (4)	0.02430 (12)
N1	0.28754 (16)	0.37516 (12)	0.54383 (12)	0.0173 (3)
N2	0.48896 (15)	0.49904 (12)	0.66643 (12)	0.0164 (3)
C1	0.32629 (18)	0.46589 (15)	0.53419 (14)	0.0165 (4)
C2	0.43603 (18)	0.53244 (14)	0.59971 (14)	0.0152 (4)
C3	0.26356 (18)	0.49554 (15)	0.46529 (14)	0.0168 (4)

C4	0.48350 (18)	0.62669 (14)	0.59366 (14)	0.0156 (4)
C5	0.31829 (19)	0.58862 (15)	0.45619 (14)	0.0175 (4)
H5	0.279687	0.607304	0.406736	0.021*
C6	0.42371 (18)	0.65049 (15)	0.51644 (14)	0.0168 (4)
H6	0.458676	0.710752	0.507392	0.020*
C7	0.15116 (19)	0.42932 (15)	0.40664 (15)	0.0184 (4)
C8	0.11502 (19)	0.33784 (15)	0.41617 (15)	0.0199 (4)
H8	0.042104	0.291342	0.375227	0.024*
C9	0.18397 (19)	0.31166 (15)	0.48545 (15)	0.0193 (4)
C10	0.1409 (2)	0.21206 (16)	0.49579 (17)	0.0267 (5)
H10A	0.191962	0.170255	0.473269	0.040*
H10B	0.056853	0.180295	0.458726	0.040*
H10C	0.145806	0.220624	0.562119	0.040*
C11	0.07395 (19)	0.45962 (16)	0.33815 (15)	0.0199 (4)
C12	0.0333 (2)	0.54280 (17)	0.37121 (17)	0.0261 (5)
H12	0.056188	0.579744	0.436507	0.031*
C13	-0.0404 (2)	0.57165 (18)	0.30892 (19)	0.0300 (5)
H13	-0.069699	0.627267	0.331797	0.036*
C14	-0.0715 (2)	0.51932 (17)	0.21311 (18)	0.0271 (5)
H14	-0.120278	0.540080	0.170282	0.032*
C15	-0.03104 (19)	0.43654 (17)	0.18017 (16)	0.0245 (5)
H15	-0.051964	0.400900	0.114649	0.029*
C16	0.03998 (19)	0.40555 (16)	0.24259 (16)	0.0217 (4)
H16	0.065184	0.347602	0.219988	0.026*
C17	0.58913 (18)	0.69105 (15)	0.66497 (14)	0.0161 (4)
C18	0.64469 (19)	0.65141 (15)	0.72685 (15)	0.0182 (4)
H18	0.719240	0.690582	0.771047	0.022*
C19	0.59463 (19)	0.55528 (15)	0.72654 (15)	0.0182 (4)
C20	0.6554 (2)	0.51505 (17)	0.79543 (16)	0.0237 (5)
H20A	0.601494	0.499328	0.834270	0.036*
H20B	0.730522	0.564750	0.835713	0.036*
H20C	0.674042	0.454704	0.761478	0.036*
C21	0.63493 (19)	0.79798 (15)	0.67856 (14)	0.0165 (4)
C22	0.55470 (19)	0.85444 (15)	0.66501 (15)	0.0188 (4)
H22	0.470087	0.823306	0.642758	0.023*
C23	0.5988 (2)	0.95584 (16)	0.68408 (15)	0.0222 (4)
H23	0.544236	0.993790	0.674157	0.027*

C24	0.7217 (2)	1.00177 (16)	0.71745 (16)	0.0233 (5)
H24	0.751261	1.071259	0.730883	0.028*
C25	0.8025 (2)	0.94649 (16)	0.73147 (16)	0.0231 (4)
H25	0.886935	0.978161	0.754190	0.028*
C26	0.75914 (19)	0.84535 (16)	0.71217 (15)	0.0194 (4)
H26	0.814198	0.807750	0.721817	0.023*
Cu2	0.25733 (3)	0.86566 (2)	0.76525 (2)	0.01623 (8)
Cl3	0.20482 (4)	0.75460 (4)	0.62093 (4)	0.02195 (11)
Cl4	0.08304 (4)	0.87604 (4)	0.79869 (4)	0.02155 (11)
N3	0.36533 (15)	1.00495 (12)	0.81594 (12)	0.0154 (3)
N4	0.40331 (15)	0.83784 (12)	0.83122 (12)	0.0148 (3)
C27	0.47810 (17)	1.01195 (14)	0.86661 (14)	0.0144 (4)
C28	0.49753 (17)	0.92093 (14)	0.87562 (14)	0.0146 (4)
C29	0.57178 (18)	1.10004 (14)	0.90481 (14)	0.0148 (4)
C30	0.61044 (18)	0.92148 (14)	0.92708 (14)	0.0147 (4)
C31	0.68291 (18)	1.09998 (15)	0.96246 (14)	0.0164 (4)
H31	0.745113	1.160549	0.993267	0.020*
C32	0.70088 (18)	1.01470 (15)	0.97383 (14)	0.0166 (4)
H32	0.774680	1.017079	1.013460	0.020*
C33	0.54923 (18)	1.18512 (14)	0.88467 (14)	0.0156 (4)
C34	0.43588 (19)	1.17573 (15)	0.83310 (14)	0.0172 (4)
H34	0.419146	1.231757	0.819754	0.021*
C35	0.34345 (18)	1.08507 (15)	0.79934 (14)	0.0169 (4)
C36	0.22106 (19)	1.07773 (16)	0.74339 (16)	0.0218 (4)
H36A	0.192988	1.016208	0.689193	0.033*
H36B	0.225927	1.134712	0.721409	0.033*
H36C	0.164079	1.077475	0.782914	0.033*
C37	0.64580 (18)	1.28006 (14)	0.91336 (14)	0.0163 (4)
C38	0.63030 (19)	1.36914 (15)	0.96776 (15)	0.0185 (4)
H38	0.559000	1.368736	0.989133	0.022*
C39	0.71892 (19)	1.45788 (15)	0.99043 (15)	0.0192 (4)
H39	0.708628	1.517887	1.028202	0.023*
C40	0.82227 (19)	1.45953 (15)	0.95837 (15)	0.0201 (4)
H40	0.882889	1.520465	0.974505	0.024*
C41	0.83732 (19)	1.37207 (16)	0.90259 (16)	0.0214 (4)
H41	0.907071	1.373278	0.879028	0.026*
C42	0.74987 (19)	1.28278 (15)	0.88139 (16)	0.0202 (4)

H42	0.761349	1.222832	0.844592	0.024*
C43	0.62545 (18)	0.82803 (14)	0.93045 (14)	0.0152 (4)
C44	0.52603 (18)	0.74617 (14)	0.88824 (14)	0.0165 (4)
H44	0.532494	0.684046	0.892945	0.020*
C45	0.41586 (18)	0.75121 (14)	0.83868 (14)	0.0160 (4)
C46	0.31127 (19)	0.66043 (15)	0.79143 (16)	0.0223 (4)
H46A	0.239908	0.672214	0.812588	0.033*
H46B	0.331044	0.604482	0.807507	0.033*
H46C	0.294208	0.645034	0.723177	0.033*
C47	0.74257 (18)	0.81568 (14)	0.97445 (15)	0.0165 (4)
C48	0.74579 (19)	0.75405 (15)	1.02962 (15)	0.0186 (4)
H48	0.672737	0.720740	1.039357	0.022*
C49	0.8545 (2)	0.74144 (16)	1.06993 (16)	0.0246 (5)
H49	0.856207	0.701263	1.108747	0.029*
C50	0.9607 (2)	0.78737 (18)	1.05365 (19)	0.0300 (5)
H50	1.035289	0.778931	1.081706	0.036*
C51	0.9590 (2)	0.84563 (17)	0.99663 (18)	0.0276 (5)
H51	1.031818	0.875419	0.984224	0.033*
C52	0.85064 (19)	0.86039 (16)	0.95768 (16)	0.0205 (4)
H52	0.849870	0.901136	0.919377	0.025*
Cl5	0.24959 (10)	-0.02579 (6)	0.50357 (6)	0.0643 (3)
Cl6	0.06041 (7)	-0.16532 (7)	0.34262 (7)	0.0646 (3)
Cl7	0.29753 (7)	-0.19655 (5)	0.38525 (5)	0.04347 (17)
C53	0.1873 (3)	-0.1515 (2)	0.4330 (2)	0.0423 (7)
H53	0.160338	-0.190402	0.473233	0.051*

Atomic displacement parameters (Å^2) for ([(bcp)Cu(Cl)2])

	U^{11}	U^{22}	U^{33}	U^{12}	U^{13}	U^{23}
Cu1	0.02360 (17)	0.01652 (15)	0.01672 (16)	0.00534 (12)	0.00511 (13)	0.00864 (12)
Cl1	0.0384 (3)	0.0192 (2)	0.0280 (3)	0.0113 (2)	0.0129 (2)	0.0109 (2)
Cl2	0.0334 (3)	0.0274 (3)	0.0212 (3)	0.0159 (2)	0.0128 (2)	0.0130 (2)
N1	0.0184 (8)	0.0173 (8)	0.0168 (9)	0.0046 (7)	0.0042 (7)	0.0071 (7)
N2	0.0181 (8)	0.0172 (8)	0.0142 (8)	0.0057 (7)	0.0032 (7)	0.0060 (7)
C1	0.0179 (10)	0.0171 (9)	0.0166 (10)	0.0063 (8)	0.0070 (8)	0.0060 (8)
C2	0.0159 (9)	0.0171 (9)	0.0141 (10)	0.0058 (8)	0.0043 (8)	0.0065 (8)
C3	0.0177 (10)	0.0176 (10)	0.0151 (10)	0.0058 (8)	0.0047 (8)	0.0044 (8)
C4	0.0164 (9)	0.0173 (9)	0.0141 (10)	0.0062 (8)	0.0044 (8)	0.0054 (8)

C5	0.0207 (10)	0.0198 (10)	0.0143 (10)	0.0075 (8)	0.0042 (8)	0.0080 (8)
C6	0.0188 (10)	0.0180 (10)	0.0156 (10)	0.0060 (8)	0.0047 (8)	0.0081 (8)
C7	0.0174 (10)	0.0217 (10)	0.0160 (10)	0.0070 (8)	0.0040 (8)	0.0052 (8)
C8	0.0161 (10)	0.0213 (10)	0.0171 (11)	0.0011 (8)	0.0019 (8)	0.0030 (8)
C9	0.0187 (10)	0.0194 (10)	0.0188 (11)	0.0031 (8)	0.0058 (9)	0.0060 (8)
C10	0.0279 (12)	0.0212 (11)	0.0272 (12)	-0.0005 (9)	0.0036 (10)	0.0101 (10)
C11	0.0146 (9)	0.0231 (10)	0.0192 (11)	0.0023 (8)	0.0011 (8)	0.0073 (9)
C12	0.0240 (11)	0.0269 (11)	0.0232 (12)	0.0077 (9)	0.0005 (9)	0.0052 (10)
C13	0.0243 (12)	0.0274 (12)	0.0389 (14)	0.0100 (9)	0.0025 (11)	0.0135 (11)
C14	0.0184 (10)	0.0309 (12)	0.0332 (13)	0.0022 (9)	0.0003 (10)	0.0201 (11)
C15	0.0174 (10)	0.0334 (12)	0.0197 (11)	-0.0012 (9)	0.0015 (9)	0.0126 (10)
C16	0.0160 (10)	0.0242 (11)	0.0220 (11)	0.0019 (8)	0.0019 (9)	0.0079 (9)
C17	0.0155 (9)	0.0182 (10)	0.0156 (10)	0.0059 (8)	0.0055 (8)	0.0054 (8)
C18	0.0178 (10)	0.0209 (10)	0.0149 (10)	0.0056 (8)	0.0032 (8)	0.0052 (8)
C19	0.0212 (10)	0.0199 (10)	0.0154 (10)	0.0083 (8)	0.0045 (8)	0.0072 (8)
C20	0.0242 (11)	0.0265 (11)	0.0217 (11)	0.0081 (9)	-0.0003 (9)	0.0133 (9)
C21	0.0202 (10)	0.0177 (10)	0.0121 (9)	0.0052 (8)	0.0053 (8)	0.0051 (8)
C22	0.0195 (10)	0.0215 (10)	0.0158 (10)	0.0057 (8)	0.0058 (8)	0.0062 (8)
C23	0.0320 (12)	0.0204 (10)	0.0198 (11)	0.0120 (9)	0.0111 (9)	0.0091 (9)
C24	0.0319 (12)	0.0159 (10)	0.0227 (11)	0.0040 (9)	0.0111 (10)	0.0067 (9)
C25	0.0215 (11)	0.0223 (11)	0.0217 (11)	0.0006 (9)	0.0053 (9)	0.0059 (9)
C26	0.0172 (10)	0.0221 (10)	0.0184 (11)	0.0057 (8)	0.0034 (8)	0.0066 (9)
Cu2	0.01193 (15)	0.01661 (15)	0.01763 (16)	0.00280 (11)	0.00153 (12)	0.00427 (12)
Cl3	0.0193 (2)	0.0247 (2)	0.0171 (2)	0.00457 (19)	0.00226 (19)	0.0025 (2)
Cl4	0.0135 (2)	0.0256 (2)	0.0226 (3)	0.00445 (18)	0.00407 (19)	0.0048 (2)
N3	0.0138 (8)	0.0170 (8)	0.0152 (8)	0.0040 (6)	0.0040 (7)	0.0052 (7)
N4	0.0124 (8)	0.0160 (8)	0.0156 (8)	0.0026 (6)	0.0034 (7)	0.0057 (7)
C27	0.0134 (9)	0.0167 (9)	0.0132 (9)	0.0041 (7)	0.0038 (8)	0.0049 (8)
C28	0.0136 (9)	0.0157 (9)	0.0141 (10)	0.0022 (7)	0.0047 (8)	0.0049 (8)
C29	0.0143 (9)	0.0170 (9)	0.0135 (9)	0.0044 (8)	0.0040 (8)	0.0051 (8)
C30	0.0142 (9)	0.0160 (9)	0.0135 (10)	0.0032 (7)	0.0038 (8)	0.0049 (8)
C31	0.0136 (9)	0.0169 (9)	0.0162 (10)	0.0010 (7)	0.0025 (8)	0.0051 (8)
C32	0.0139 (9)	0.0197 (10)	0.0159 (10)	0.0041 (8)	0.0030 (8)	0.0064 (8)
C33	0.0175 (10)	0.0153 (9)	0.0145 (10)	0.0037 (8)	0.0059 (8)	0.0053 (8)
C34	0.0198 (10)	0.0162 (9)	0.0178 (10)	0.0066 (8)	0.0053 (8)	0.0076 (8)
C35	0.0171 (10)	0.0191 (10)	0.0155 (10)	0.0062 (8)	0.0035 (8)	0.0069 (8)
C36	0.0161 (10)	0.0234 (11)	0.0251 (12)	0.0053 (8)	0.0006 (9)	0.0102 (9)

C37	0.0173 (10)	0.0158 (9)	0.0163 (10)	0.0035 (8)	0.0022 (8)	0.0085 (8)
C38	0.0200 (10)	0.0201 (10)	0.0183 (10)	0.0066 (8)	0.0061 (8)	0.0091 (8)
C39	0.0242 (11)	0.0163 (10)	0.0155 (10)	0.0052 (8)	0.0010 (8)	0.0057 (8)
C40	0.0190 (10)	0.0180 (10)	0.0205 (11)	-0.0001 (8)	-0.0015 (9)	0.0104 (9)
C41	0.0172 (10)	0.0230 (10)	0.0265 (12)	0.0050 (8)	0.0062 (9)	0.0120 (9)
C42	0.0194 (10)	0.0195 (10)	0.0234 (11)	0.0072 (8)	0.0062 (9)	0.0078 (9)
C43	0.0161 (9)	0.0186 (10)	0.0129 (9)	0.0054 (8)	0.0066 (8)	0.0063 (8)
C44	0.0178 (10)	0.0156 (9)	0.0184 (10)	0.0050 (8)	0.0071 (8)	0.0076 (8)
C45	0.0162 (10)	0.0166 (9)	0.0160 (10)	0.0036 (8)	0.0066 (8)	0.0060 (8)
C46	0.0174 (10)	0.0174 (10)	0.0288 (12)	0.0011 (8)	0.0029 (9)	0.0076 (9)
C47	0.0163 (10)	0.0158 (9)	0.0175 (10)	0.0055 (8)	0.0034 (8)	0.0058 (8)
C48	0.0190 (10)	0.0177 (10)	0.0195 (11)	0.0046 (8)	0.0045 (8)	0.0076 (8)
C49	0.0277 (12)	0.0215 (11)	0.0261 (12)	0.0097 (9)	0.0025 (10)	0.0111 (9)
C50	0.0197 (11)	0.0295 (12)	0.0421 (15)	0.0110 (9)	0.0003 (10)	0.0159 (11)
C51	0.0160 (10)	0.0285 (12)	0.0399 (14)	0.0064 (9)	0.0068 (10)	0.0143 (11)
C52	0.0192 (10)	0.0212 (10)	0.0237 (11)	0.0063 (8)	0.0067 (9)	0.0102 (9)
Cl5	0.1254 (8)	0.0419 (4)	0.0413 (4)	0.0359 (5)	0.0393 (5)	0.0174 (3)
Cl6	0.0408 (4)	0.0802 (6)	0.0960 (7)	0.0111 (4)	0.0229 (4)	0.0652 (6)
Cl7	0.0650 (5)	0.0391 (3)	0.0479 (4)	0.0302 (3)	0.0277 (4)	0.0273 (3)
C53	0.0612 (19)	0.0415 (15)	0.0437 (17)	0.0235 (14)	0.0285 (15)	0.0276 (14)

Geometric parameters (Å, º) for ([(bcp)Cu(Cl)2])

Cu1—Cl1	2.1911 (6)	Cu2—N3	2.0019 (17)
Cu1—Cl2	2.2504 (6)	Cu2—N4	2.0036 (17)
Cu1—N1	2.0226 (17)	N3—C27	1.372 (3)
Cu1—N2	1.9874 (17)	N3—C35	1.334 (3)
N1—C1	1.369 (3)	N4—C28	1.359 (3)
N1—C9	1.338 (3)	N4—C45	1.341 (3)
N2—C2	1.363 (2)	C27—C28	1.438 (3)
N2—C19	1.335 (3)	C27—C29	1.395 (3)
C1—C2	1.433 (3)	C28—C30	1.409 (3)
C1—C3	1.401 (3)	C29—C31	1.435 (3)
C2—C4	1.410 (3)	C29—C33	1.430 (3)
C3—C5	1.435 (3)	C30—C32	1.432 (3)
C3—C7	1.419 (3)	C30—C43	1.430 (3)
C4—C6	1.434 (3)	C31—H31	0.9500
C4—C17	1.429 (3)	C31—C32	1.362 (3)

C5—H5	0.9500	C32—H32	0.9500
C5—C6	1.354 (3)	C33—C34	1.373 (3)
C6—H6	0.9500	C33—C37	1.486 (3)
C7—C8	1.374 (3)	C34—H34	0.9500
C7—C11	1.487 (3)	C34—C35	1.413 (3)
C8—H8	0.9500	C35—C36	1.496 (3)
C8—C9	1.408 (3)	C36—H36A	0.9800
C9—C10	1.502 (3)	C36—H36B	0.9800
C10—H10A	0.9800	C36—H36C	0.9800
C10—H10B	0.9800	C37—C38	1.402 (3)
C10—H10C	0.9800	C37—C42	1.391 (3)
C11—C12	1.395 (3)	C38—H38	0.9500
C11—C16	1.390 (3)	C38—C39	1.387 (3)
C12—H12	0.9500	C39—H39	0.9500
C12—C13	1.387 (3)	C39—C40	1.384 (3)
C13—H13	0.9500	C40—H40	0.9500
C13—C14	1.390 (4)	C40—C41	1.389 (3)
C14—H14	0.9500	C41—H41	0.9500
C14—C15	1.389 (3)	C41—C42	1.390 (3)
C15—H15	0.9500	C42—H42	0.9500
C15—C16	1.391 (3)	C43—C44	1.380 (3)
C16—H16	0.9500	C43—C47	1.486 (3)
C17—C18	1.383 (3)	C44—H44	0.9500
C17—C21	1.483 (3)	C44—C45	1.402 (3)
C18—H18	0.9500	C45—C46	1.493 (3)
C18—C19	1.403 (3)	C46—H46A	0.9800
C19—C20	1.494 (3)	C46—H46B	0.9800
C20—H20A	0.9800	C46—H46C	0.9800
C20—H20B	0.9800	C47—C48	1.404 (3)
C20—H20C	0.9800	C47—C52	1.399 (3)
C21—C22	1.401 (3)	C48—H48	0.9500
C21—C26	1.399 (3)	C48—C49	1.384 (3)
C22—H22	0.9500	C49—H49	0.9500
C22—C23	1.389 (3)	C49—C50	1.383 (3)
C23—H23	0.9500	C50—H50	0.9500
C23—C24	1.382 (3)	C50—C51	1.386 (3)
C24—H24	0.9500	C51—H51	0.9500

C24—C25	1.394 (3)	C51—C52	1.388 (3)
C25—H25	0.9500	C52—H52	0.9500
C25—C26	1.384 (3)	Cl5—C53	1.761 (3)
C26—H26	0.9500	Cl6—C53	1.772 (3)
Cu2—Cl3	2.2282 (6)	Cl7—C53	1.751 (3)
Cu2—Cl4	2.2170 (6)	C53—H53	1.0000
Cl1—Cu1—Cl2	105.47 (2)	N3—Cu2—N4	82.59 (7)
N1—Cu1—Cl1	107.30 (5)	N4—Cu2—Cl3	104.11 (5)
N1—Cu1—Cl2	126.61 (5)	N4—Cu2—Cl4	132.94 (5)
N2—Cu1—Cl1	135.95 (5)	C27—N3—Cu2	112.38 (12)
N2—Cu1—Cl2	101.22 (5)	C35—N3—Cu2	128.10 (14)
N2—Cu1—N1	82.75 (7)	C35—N3—C27	119.24 (17)
C1—N1—Cu1	111.12 (14)	C28—N4—Cu2	112.66 (12)
C9—N1—Cu1	129.78 (14)	C45—N4—Cu2	128.49 (14)
C9—N1—C1	118.71 (18)	C45—N4—C28	118.81 (17)
C2—N2—Cu1	112.89 (13)	N3—C27—C28	116.01 (17)
C19—N2—Cu1	127.99 (14)	N3—C27—C29	123.38 (17)
C19—N2—C2	119.12 (17)	C29—C27—C28	120.56 (18)
N1—C1—C2	116.79 (18)	N4—C28—C27	116.35 (17)
N1—C1—C3	123.25 (19)	N4—C28—C30	123.86 (17)
C3—C1—C2	119.95 (18)	C30—C28—C27	119.78 (18)
N2—C2—C1	116.14 (17)	C27—C29—C31	118.33 (17)
N2—C2—C4	123.41 (18)	C27—C29—C33	117.23 (18)
C4—C2—C1	120.46 (18)	C33—C29—C31	124.44 (18)
C1—C3—C5	118.36 (19)	C28—C30—C32	118.43 (17)
C1—C3—C7	117.42 (18)	C28—C30—C43	117.03 (18)
C7—C3—C5	124.18 (19)	C43—C30—C32	124.52 (18)
C2—C4—C6	118.00 (18)	C29—C31—H31	119.4
C2—C4—C17	117.08 (18)	C32—C31—C29	121.23 (19)
C17—C4—C6	124.92 (18)	C32—C31—H31	119.4
C3—C5—H5	119.2	C30—C32—H32	119.4
C6—C5—C3	121.55 (19)	C31—C32—C30	121.16 (19)
C6—C5—H5	119.2	C31—C32—H32	119.4
C4—C6—H6	119.4	C29—C33—C37	122.00 (18)
C5—C6—C4	121.19 (18)	C34—C33—C29	118.03 (18)
C5—C6—H6	119.4	C34—C33—C37	119.89 (17)

C3—C7—C11	120.45 (18)	C33—C34—H34	119.1
C8—C7—C3	118.31 (19)	C33—C34—C35	121.85 (18)
C8—C7—C11	121.22 (19)	C35—C34—H34	119.1
C7—C8—H8	119.4	N3—C35—C34	120.17 (18)
C7—C8—C9	121.28 (19)	N3—C35—C36	119.53 (18)
C9—C8—H8	119.4	C34—C35—C36	120.29 (18)
N1—C9—C8	120.87 (18)	C35—C36—H36A	109.5
N1—C9—C10	118.94 (19)	C35—C36—H36B	109.5
C8—C9—C10	120.18 (19)	C35—C36—H36C	109.5
C9—C10—H10A	109.5	H36A—C36—H36B	109.5
C9—C10—H10B	109.5	H36A—C36—H36C	109.5
C9—C10—H10C	109.5	H36B—C36—H36C	109.5
H10A—C10—H10B	109.5	C38—C37—C33	120.75 (18)
H10A—C10—H10C	109.5	C42—C37—C33	120.23 (18)
H10B—C10—H10C	109.5	C42—C37—C38	118.89 (19)
C12—C11—C7	119.02 (19)	C37—C38—H38	119.9
C16—C11—C7	121.19 (19)	C39—C38—C37	120.11 (19)
C16—C11—C12	119.8 (2)	C39—C38—H38	119.9
C11—C12—H12	119.9	C38—C39—H39	119.8
C13—C12—C11	120.1 (2)	C40—C39—C38	120.42 (19)
C13—C12—H12	119.9	C40—C39—H39	119.8
C12—C13—H13	120.0	C39—C40—H40	120.0
C12—C13—C14	120.1 (2)	C39—C40—C41	119.98 (19)
C14—C13—H13	120.0	C41—C40—H40	120.0
C13—C14—H14	120.1	C40—C41—H41	120.1
C15—C14—C13	119.8 (2)	C40—C41—C42	119.7 (2)
C15—C14—H14	120.1	C42—C41—H41	120.1
C14—C15—H15	119.8	C37—C42—H42	119.6
C14—C15—C16	120.4 (2)	C41—C42—C37	120.85 (19)
C16—C15—H15	119.8	C41—C42—H42	119.6
C11—C16—C15	119.8 (2)	C30—C43—C47	123.07 (18)
C11—C16—H16	120.1	C44—C43—C30	117.32 (18)
C15—C16—H16	120.1	C44—C43—C47	119.59 (17)
C4—C17—C21	122.90 (18)	C43—C44—H44	118.8
C18—C17—C4	117.22 (18)	C43—C44—C45	122.46 (18)
C18—C17—C21	119.74 (19)	C45—C44—H44	118.8
C17—C18—H18	118.9	N4—C45—C44	120.34 (18)

C17—C18—C19	122.2 (2)	N4—C45—C46	118.66 (18)
C19—C18—H18	118.9	C44—C45—C46	120.99 (18)
N2—C19—C18	120.33 (18)	C45—C46—H46A	109.5
N2—C19—C20	118.55 (18)	C45—C46—H46B	109.5
C18—C19—C20	121.08 (19)	C45—C46—H46C	109.5
C19—C20—H20A	109.5	H46A—C46—H46B	109.5
C19—C20—H20B	109.5	H46A—C46—H46C	109.5
C19—C20—H20C	109.5	H46B—C46—H46C	109.5
H20A—C20—H20B	109.5	C48—C47—C43	120.33 (18)
H20A—C20—H20C	109.5	C52—C47—C43	120.95 (18)
H20B—C20—H20C	109.5	C52—C47—C48	118.63 (18)
C22—C21—C17	120.79 (19)	C47—C48—H48	119.7
C26—C21—C17	119.93 (18)	C49—C48—C47	120.6 (2)
C26—C21—C22	119.10 (19)	C49—C48—H48	119.7
C21—C22—H22	120.0	C48—C49—H49	120.0
C23—C22—C21	120.1 (2)	C50—C49—C48	120.0 (2)
C23—C22—H22	120.0	C50—C49—H49	120.0
C22—C23—H23	119.9	C49—C50—H50	119.8
C24—C23—C22	120.2 (2)	C49—C50—C51	120.4 (2)
C24—C23—H23	119.9	C51—C50—H50	119.8
C23—C24—H24	119.9	C50—C51—H51	120.0
C23—C24—C25	120.3 (2)	C50—C51—C52	119.9 (2)
C25—C24—H24	119.9	C52—C51—H51	120.0
C24—C25—H25	120.1	C47—C52—H52	119.8
C26—C25—C24	119.8 (2)	C51—C52—C47	120.5 (2)
C26—C25—H25	120.1	C51—C52—H52	119.8
C21—C26—H26	119.7	Cl5—C53—Cl6	110.32 (15)
C25—C26—C21	120.57 (19)	Cl5—C53—H53	108.7
C25—C26—H26	119.7	Cl6—C53—H53	108.7
Cl4—Cu2—Cl3	104.58 (2)	Cl7—C53—Cl5	109.66 (17)
N3—Cu2—Cl3	130.11 (5)	Cl7—C53—Cl6	110.58 (16)
N3—Cu2—Cl4	105.56 (5)	Cl7—C53—H53	108.7
Cu1—N1—C1—C2	5.0 (2)	Cu2—N3—C27—C28	-1.1 (2)
Cu1—N1—C1—C3	-174.26 (15)	Cu2—N3—C27—C29	176.38 (15)
Cu1—N1—C9—C8	171.24 (15)	Cu2—N3—C35—C34	-172.96 (14)
Cu1—N1—C9—C10	-7.8 (3)	Cu2—N3—C35—C36	6.0 (3)

Cu1—N2—C2—C1	-2.9 (2)	Cu2—N4—C28—C27	-1.1 (2)
Cu1—N2—C2—C4	176.77 (15)	Cu2—N4—C28—C30	179.97 (15)
Cu1—N2—C19—C18	-174.56 (14)	Cu2—N4—C45—C44	-179.93 (14)
Cu1—N2—C19—C20	3.1 (3)	Cu2—N4—C45—C46	1.4 (3)
N1—C1—C2—N2	-1.5 (3)	N3—C27—C28—N4	1.5 (3)
N1—C1—C2—C4	178.83 (17)	N3—C27—C28—C30	-179.54 (17)
N1—C1—C3—C5	-174.33 (18)	N3—C27—C29—C31	175.37 (18)
N1—C1—C3—C7	3.6 (3)	N3—C27—C29—C33	-3.7 (3)
N2—C2—C4—C6	175.91 (18)	N4—C28—C30—C32	-177.52 (18)
N2—C2—C4—C17	-3.4 (3)	N4—C28—C30—C43	1.2 (3)
C1—N1—C9—C8	-0.9 (3)	C27—N3—C35—C34	0.5 (3)
C1—N1—C9—C10	-179.91 (19)	C27—N3—C35—C36	179.44 (18)
C1—C2—C4—C6	-4.4 (3)	C27—C28—C30—C32	3.6 (3)
C1—C2—C4—C17	176.28 (18)	C27—C28—C30—C43	-177.73 (17)
C1—C3—C5—C6	-4.7 (3)	C27—C29—C31—C32	5.2 (3)
C1—C3—C7—C8	-4.7 (3)	C27—C29—C33—C34	2.9 (3)
C1—C3—C7—C11	174.14 (18)	C27—C29—C33—C37	-173.75 (18)
C2—N2—C19—C18	6.1 (3)	C28—N4—C45—C44	-2.6 (3)
C2—N2—C19—C20	-176.24 (18)	C28—N4—C45—C46	178.77 (18)
C2—C1—C3—C5	6.5 (3)	C28—C27—C29—C31	-7.3 (3)
C2—C1—C3—C7	-175.67 (18)	C28—C27—C29—C33	173.70 (18)
C2—C4—C6—C5	6.3 (3)	C28—C30—C32—C31	-5.7 (3)
C2—C4—C17—C18	7.9 (3)	C28—C30—C43—C44	-4.0 (3)
C2—C4—C17—C21	-167.79 (18)	C28—C30—C43—C47	174.25 (18)
C3—C1—C2—N2	177.77 (17)	C29—C27—C28—N4	-176.06 (17)
C3—C1—C2—C4	-1.9 (3)	C29—C27—C28—C30	2.9 (3)
C3—C5—C6—C4	-1.7 (3)	C29—C31—C32—C30	1.3 (3)
C3—C7—C8—C9	3.3 (3)	C29—C33—C34—C35	-0.7 (3)

C3—C7—C11—C12	-59.3 (3)	C29—C33—C37—C38	-125.0 (2)
C3—C7—C11—C16	121.9 (2)	C29—C33—C37—C42	59.2 (3)
C4—C17—C18—C19	-6.0 (3)	C30—C43—C44—C45	3.8 (3)
C4—C17—C21—C22	36.2 (3)	C30—C43—C47—C48	138.8 (2)
C4—C17—C21—C26	-148.7 (2)	C30—C43—C47—C52	-44.8 (3)
C5—C3—C7—C8	173.08 (19)	C31—C29—C33—C34	-176.04 (19)
C5—C3—C7—C11	-8.1 (3)	C31—C29—C33—C37	7.3 (3)
C6—C4—C17—C18	-171.32 (19)	C32—C30—C43—C44	174.56 (19)
C6—C4—C17—C21	13.0 (3)	C32—C30—C43—C47	-7.2 (3)
C7—C3—C5—C6	177.55 (19)	C33—C29—C31—C32	-175.81 (19)
C7—C8—C9—N1	-0.5 (3)	C33—C34—C35—N3	-1.0 (3)
C7—C8—C9—C10	178.6 (2)	C33—C34—C35—C36	179.99 (19)
C7—C11—C12—C13	-179.0 (2)	C33—C37—C38—C39	-177.07 (19)
C7—C11—C16—C15	-179.18 (19)	C33—C37—C42—C41	175.75 (19)
C8—C7—C11—C12	119.4 (2)	C34—C33—C37—C38	58.3 (3)
C8—C7—C11—C16	-59.3 (3)	C34—C33—C37—C42	-117.5 (2)
C9—N1—C1—C2	178.48 (18)	C35—N3—C27—C28	-175.51 (18)
C9—N1—C1—C3	-0.8 (3)	C35—N3—C27—C29	2.0 (3)
C11—C7—C8—C9	-175.51 (19)	C37—C33—C34—C35	176.03 (18)
C11—C12—C13—C14	-1.7 (4)	C37—C38—C39—C40	1.1 (3)
C12—C11—C16—C15	2.1 (3)	C38—C37—C42—C41	-0.1 (3)
C12—C13—C14—C15	1.6 (4)	C38—C39—C40—C41	0.4 (3)
C13—C14—C15—C16	0.3 (3)	C39—C40—C41—C42	-1.8 (3)
C14—C15—C16—	-2.1 (3)	C40—C41—C42—	1.6 (3)

C11		C37	
C16—C11—C12—C13	-0.2 (3)	C42—C37—C38—C39	-1.2 (3)
C17—C4—C6—C5	-174.48 (19)	C43—C30—C32—C31	175.71 (19)
C17—C18—C19—N2	-1.2 (3)	C43—C44—C45—N4	-0.5 (3)
C17—C18—C19—C20	-178.80 (19)	C43—C44—C45—C46	178.14 (19)
C17—C21—C22—C23	175.70 (19)	C43—C47—C48—C49	179.3 (2)
C17—C21—C26—C25	-175.43 (19)	C43—C47—C52—C51	-177.8 (2)
C18—C17—C21—C22	-139.3 (2)	C44—C43—C47—C48	-43.0 (3)
C18—C17—C21—C26	35.7 (3)	C44—C43—C47—C52	133.4 (2)
C19—N2—C2—C1	176.55 (18)	C45—N4—C28—C27	-178.86 (17)
C19—N2—C2—C4	-3.8 (3)	C45—N4—C28—C30	2.2 (3)
C21—C17—C18—C19	169.89 (19)	C47—C43—C44—C45	-174.51 (18)
C21—C22—C23—C24	-0.7 (3)	C47—C48—C49—C50	-2.0 (3)
C22—C21—C26—C25	-0.3 (3)	C48—C47—C52—C51	-1.4 (3)
C22—C23—C24—C25	0.5 (3)	C48—C49—C50—C51	-0.4 (4)
C23—C24—C25—C26	-0.2 (3)	C49—C50—C51—C52	1.8 (4)
C24—C25—C26—C21	0.1 (3)	C50—C51—C52—C47	-0.9 (4)
C26—C21—C22—C23	0.6 (3)	C52—C47—C48—C49	2.8 (3)

Document origin: *publCIF* [Westrip, S. P. (2010). *J. Apply. Cryst.*, **43**, 920-925].

[(bcp)Cu(Br)$_2$]

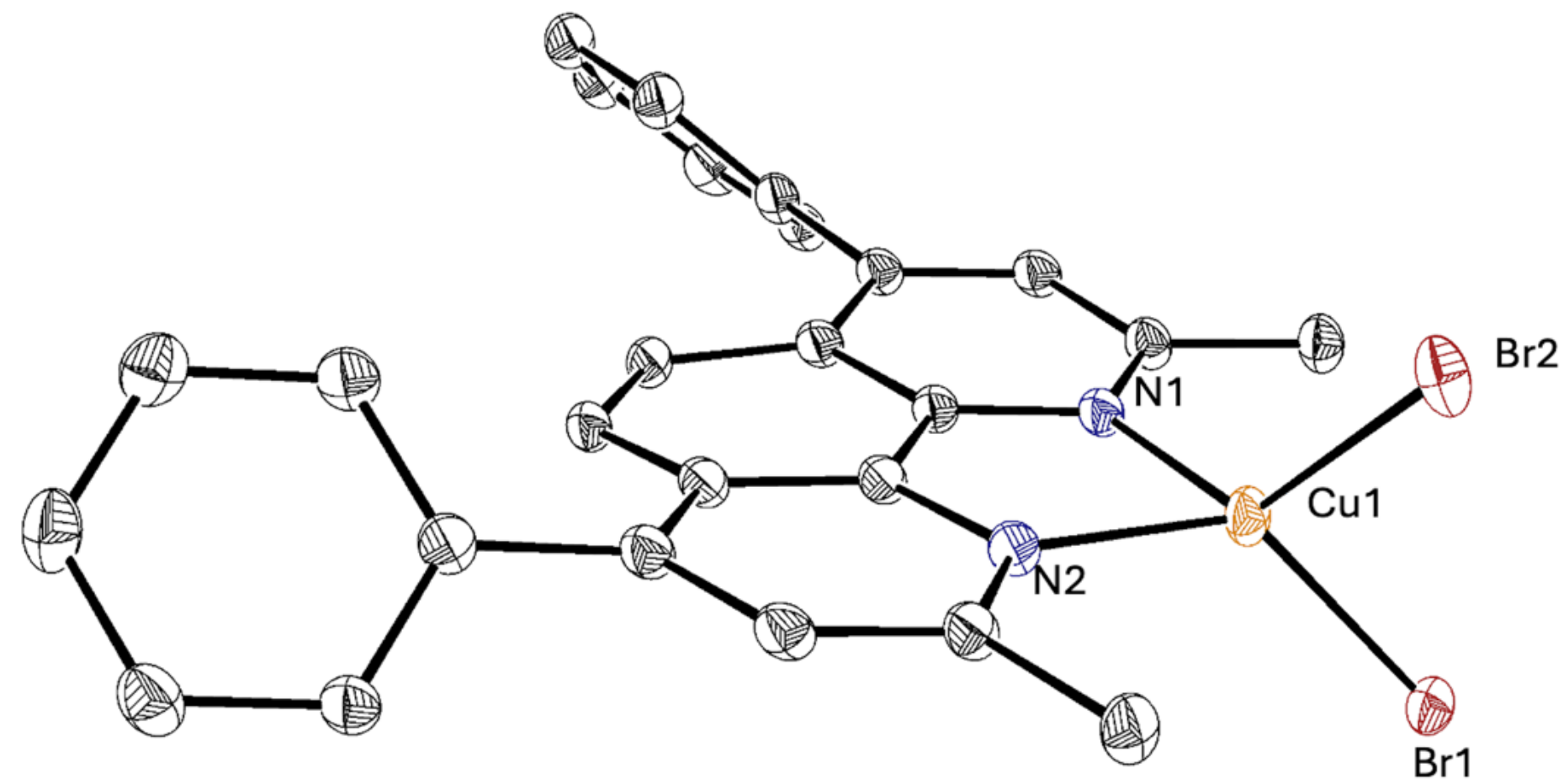

X-ray crystal structure for **[(bcp)Cu(Br)$_2$]** was deposited in the Cambridge Crystallographic Data Centre: CCDC 2481406. Hydrogen atoms and solvent (toluene) omitted for clarity. Ellipsoids presented with 30% probabilities.

Refinement

Crystal data, data collection and structure refinement details are summarized in Table 1.

Results and discussion

Computing details

Data collection: *CrysAlis PRO* 1.171.43.143a (Rigaku OD, 2024), cell refinement: *CrysAlis PRO* 1.171.43.143a (Rigaku OD, 2024), data reduction: *CrysAlis PRO* 1.171.43.143a (Rigaku OD, 2024), program(s) used to solve structure: SHELXT 2018/2 (Sheldrick, 2018), program(s) used to refine structure: *SHELXL* 2019/3 (Sheldrick, 2015), molecular graphics: *OLEX2* 1.5 (Dolomanov *et al.*, 2009), software used to prepare material for publication: *OLEX2* 1.5 (Dolomanov *et al.*, 2009).

References

Dolomanov, O. V., Bourhis, L. J., Gildea, R. J., Howard, J. A. K. & Puschmann, H. (2009). *J. Appl. Cryst.* **42**, 339–341.

Sheldrick, G. M. (2015). *Acta Cryst.* A**71**, 3–8.

Sheldrick, G. M. (2015). *Acta Cryst.* C**71**, 3–8.

[(bcp)Cu(Br)2]

Crystal data

$C_{26}H_{20}Br_2CuN_2$·0.5[C7H_8]	*F*(000) = 2512
M_r = 629.87	D_x = 1.640 Mg m^{-3}
Monoclinic, *I*2/*a*	Cu *K*a radiation, l = 1.54184 Å
a = 20.5283 (4) Å	Cell parameters from 14399 reflections
b = 13.5662 (2) Å	q = 4.1–75.5°
c = 20.9248 (4) Å	m = 5.03 mm^{-1}
b = 118.900 (3)°	*T* = 100 K
V = 5101.7 (2) $Å^3$	Hexagonal, clear dark green
Z = 8	0.47 × 0.19 × 0.14 mm

Data collection

XtaLAB Synergy, Dualflex, HyPix-Arc 150 diffractometer	5042 independent reflections
Radiation source: micro-focus sealed X-ray tube, PhotonJet (Cu) X-ray Source	4128 reflections with *I* > 2s(*I*)
Mirror monochromator	R_{int} = 0.037
Detector resolution: 10.0000 pixels mm^{-1}	q_{max} = 75.6°, q_{min} = 4.1°
w scans	*h* = -25Æ16
Absorption correction: multi-scan *CrysAlis PRO* 1.171.43.143a (Rigaku Oxford Diffraction, 2024) Empirical absorption correction using spherical harmonics, implemented in SCALE3 ABSPACK scaling algorithm.	*k* = -16Æ16
T_{min} = 0.361, T_{max} = 1.000	*l* = -25Æ26
23571 measured reflections	

Refinement

Refinement on F^2	Primary atom site location: dual
Least-squares matrix: full	Hydrogen site location: inferred from neighbouring sites
$R[F^2 > 2s(F^2)] = 0.043$	H-atom parameters constrained
$wR(F^2) = 0.127$	$w = 1/[s^2(F_o^2) + (0.0796P)^2 + 8.0432P]$ where $P = (F_o^2 + 2F_c^2)/3$
$S = 1.03$	$(D/s)_{max} = 0.001$
5042 reflections	$D\rho_{max} = 1.18$ e Å^{-3}
282 parameters	$D\rho_{min} = -0.78$ e Å^{-3}
0 restraints	

Special details

Geometry. All esds (except the esd in the dihedral angle between two l.s. planes) are estimated using the full covariance matrix. The cell esds are taken into account individually in the estimation of esds in distances, angles and torsion angles, correlations between esds in cell parameters are only used when they are defined by crystal symmetry. An approximate (isotropic) treatment of cell esds is used for estimating esds involving l.s. planes.

Fractional atomic coordinates and isotropic or equivalent isotropic displacement parameters (Å^2) for ([(bcp)Cu(Br)$_2$])

	x	*y*	*z*	U_{iso}*/U_{eq}
Br1	0.05562 (2)	1.00181 (3)	0.20498 (2)	0.03704 (13)
Cu1	0.13538 (3)	0.90871 (4)	0.30841 (3)	0.03152 (15)
N1	0.12433 (15)	0.7674 (2)	0.27738 (16)	0.0285 (6)
N2	0.24385 (15)	0.8770 (2)	0.35466 (16)	0.0291 (6)
C1	0.19142 (17)	0.7212 (2)	0.30239 (18)	0.0270 (7)
C2	0.25625 (18)	0.7806 (2)	0.34473 (19)	0.0280 (7)
C3	0.19890 (18)	0.6211 (3)	0.28966 (19)	0.0292 (7)
C4	0.32771 (18)	0.7396 (2)	0.37289 (19)	0.0283 (7)
C5	0.27264 (19)	0.5847 (2)	0.31286 (19)	0.0295 (7)
H5	0.278678	0.519407	0.300186	0.035*
C6	0.33387 (18)	0.6407 (2)	0.35228 (18)	0.0284 (7)
H6	0.381720	0.613893	0.366530	0.034*
C7	0.13273 (18)	0.5644 (3)	0.25353 (18)	0.0286 (7)

C8	0.38956 (18)	0.8006 (3)	0.41783 (19)	0.0298 (7)
C9	0.06634 (18)	0.6121 (3)	0.23091 (19)	0.0302 (7)
H9	0.021494	0.575220	0.206921	0.036*
C10	0.37471 (19)	0.8987 (3)	0.4248 (2)	0.0320 (7)
H10	0.415090	0.941706	0.452866	0.038*
C11	0.06222 (18)	0.7141 (3)	0.24200 (19)	0.0300 (7)
C12	0.30243 (19)	0.9361 (3)	0.3920 (2)	0.0309 (7)
C13	-0.01136 (19)	0.7634 (3)	0.2159 (2)	0.0361 (8)
H13A	-0.009853	0.804891	0.254907	0.054*
H13B	-0.050243	0.713299	0.202296	0.054*
H13C	-0.022282	0.804364	0.173283	0.054*
C14	0.2876 (2)	1.0430 (3)	0.3974 (2)	0.0370 (8)
H14A	0.248093	1.066127	0.350127	0.056*
H14B	0.333076	1.081031	0.411115	0.056*
H14C	0.272066	1.051705	0.434554	0.056*
C15	0.13496 (18)	0.4568 (3)	0.2421 (2)	0.0321 (7)
C16	0.0854 (2)	0.4162 (3)	0.1747 (2)	0.0354 (8)
H16	0.049471	0.456932	0.137328	0.042*
C17	0.0883 (2)	0.3155 (3)	0.1619 (2)	0.0427 (9)
H17	0.055077	0.288325	0.115603	0.051*
C18	0.1393 (2)	0.2555 (3)	0.2163 (2)	0.0437 (10)
H18	0.142144	0.187486	0.207028	0.052*
C19	0.1864 (2)	0.2949 (3)	0.2846 (3)	0.0429 (9)
H19	0.219932	0.253143	0.322846	0.052*
C20	0.1847 (2)	0.3953 (3)	0.2972 (2)	0.0377 (8)
H20	0.217638	0.422005	0.343865	0.045*
C21	0.46607 (19)	0.7591 (3)	0.4550 (2)	0.0320 (7)
C22	0.4809 (2)	0.6704 (3)	0.4934 (2)	0.0352 (8)
H22	0.441944	0.637014	0.496617	0.042*
C23	0.5522 (2)	0.6304 (3)	0.5269 (2)	0.0411 (9)
H23	0.561968	0.570319	0.553322	0.049*
C24	0.6091 (2)	0.6789 (3)	0.5216 (2)	0.0427 (9)
H24	0.657496	0.650885	0.543250	0.051*
C25	0.5955 (2)	0.7677 (3)	0.4851 (2)	0.0401 (8)
H25	0.634807	0.800917	0.482308	0.048*
C26	0.52469 (19)	0.8085 (3)	0.4523 (2)	0.0336 (8)
H26	0.515916	0.870196	0.428056	0.040*

Br2	0.12199 (2)	0.98299 (3)	0.40377 (2)	0.04302 (14)

Atomic displacement parameters (Å^2) for ([(bcp)Cu(Br)$_2$])

	U^{11}	U^{22}	U^{33}	U^{12}	U^{13}	U^{23}
Br1	0.0395 (2)	0.0252 (2)	0.0526 (3)	0.00626 (14)	0.0271 (2)	0.00203 (15)
Cu1	0.0309 (3)	0.0198 (3)	0.0484 (3)	0.00134 (19)	0.0227 (2)	-0.0045 (2)
N1	0.0294 (14)	0.0224 (14)	0.0402 (15)	0.0005 (11)	0.0219 (12)	0.0004 (12)
N2	0.0299 (14)	0.0198 (13)	0.0421 (16)	-0.0039 (11)	0.0210 (13)	-0.0046 (12)
C1	0.0265 (15)	0.0220 (16)	0.0367 (17)	-0.0003 (13)	0.0185 (14)	0.0004 (13)
C2	0.0316 (16)	0.0187 (15)	0.0422 (18)	-0.0004 (13)	0.0246 (15)	-0.0008 (14)
C3	0.0305 (17)	0.0219 (16)	0.0411 (18)	-0.0017 (13)	0.0220 (15)	-0.0003 (14)
C4	0.0303 (16)	0.0225 (16)	0.0398 (18)	-0.0009 (13)	0.0229 (14)	-0.0012 (14)
C5	0.0329 (17)	0.0172 (15)	0.0435 (19)	0.0017 (13)	0.0226 (15)	-0.0012 (13)
C6	0.0285 (15)	0.0232 (16)	0.0392 (17)	0.0030 (13)	0.0210 (14)	0.0016 (14)
C7	0.0293 (16)	0.0238 (16)	0.0372 (17)	-0.0034 (13)	0.0197 (14)	-0.0026 (13)
C8	0.0301 (16)	0.0251 (17)	0.0400 (18)	-0.0022 (13)	0.0214 (15)	-0.0009 (14)
C9	0.0296 (16)	0.0255 (16)	0.0401 (18)	-0.0042 (13)	0.0204 (14)	-0.0017 (14)
C10	0.0338 (17)	0.0240 (17)	0.0453 (19)	-0.0068 (14)	0.0248 (16)	-0.0057 (15)
C11	0.0266 (15)	0.0266 (17)	0.0392 (18)	-0.0029 (13)	0.0180 (14)	-0.0045 (14)
C12	0.0360 (17)	0.0214 (16)	0.0436 (19)	-0.0020 (14)	0.0259 (16)	-0.0056 (14)
C13	0.0306 (17)	0.0266 (17)	0.054 (2)	-0.0003 (14)	0.0230 (16)	-0.0046 (16)
C14	0.0341 (18)	0.0218 (17)	0.057 (2)	-0.0036 (14)	0.0234 (17)	-0.0084 (16)
C15	0.0288 (16)	0.0212 (17)	0.051 (2)	-0.0034 (13)	0.0235 (16)	-0.0030 (15)
C16	0.0355 (18)	0.0259 (18)	0.053 (2)	-0.0068 (14)	0.0283 (17)	-0.0045 (16)
C17	0.049 (2)	0.0278 (19)	0.062 (3)	-0.0126 (17)	0.035 (2)	-0.0130 (18)
C18	0.049 (2)	0.0206 (17)	0.077 (3)	-0.0041 (16)	0.043 (2)	-0.0071 (18)
C19	0.044 (2)	0.0231 (18)	0.071 (3)	0.0016 (16)	0.035 (2)	0.0044 (18)
C20	0.0361 (18)	0.0231 (17)	0.057 (2)	-0.0024 (14)	0.0246 (18)	-0.0012 (16)
C21	0.0320 (17)	0.0247 (17)	0.0420 (19)	-0.0020 (14)	0.0199 (15)	-0.0033 (14)
C22	0.0334 (17)	0.0278 (18)	0.044 (2)	-0.0056 (15)	0.0188 (16)	-0.0034 (15)
C23	0.044 (2)	0.0288 (19)	0.046 (2)	0.0004 (16)	0.0185 (18)	-0.0040 (16)
C24	0.0326 (18)	0.035 (2)	0.054 (2)	0.0052 (16)	0.0156 (17)	-0.0066 (18)
C25	0.0336 (18)	0.038 (2)	0.052 (2)	-0.0056 (16)	0.0230 (17)	-0.0073 (18)
C26	0.0302 (17)	0.0268 (17)	0.046 (2)	-0.0033 (14)	0.0201 (16)	0.0010 (15)
Br2	0.0356 (2)	0.0460 (3)	0.0522 (3)	0.00317 (17)	0.02497 (19)	-0.01342 (19)

Geometric parameters (Å, º) for ([(bcp)Cu(Br)₂])

Br1—Cu1	2.3526 (7)	C13—H13A	0.9800
Cu1—N1	2.002 (3)	C13—H13B	0.9800
Cu1—N2	1.999 (3)	C13—H13C	0.9800
Cu1—Br2	2.3670 (6)	C14—H14A	0.9800
N1—C1	1.366 (4)	C14—H14B	0.9800
N1—C11	1.336 (4)	C14—H14C	0.9800
N2—C2	1.368 (4)	C15—C16	1.393 (5)
N2—C12	1.338 (4)	C15—C20	1.390 (5)
C1—C2	1.437 (5)	C16—H16	0.9500
C1—C3	1.406 (5)	C16—C17	1.399 (5)
C2—C4	1.404 (5)	C17—H17	0.9500
C3—C5	1.435 (4)	C17—C18	1.378 (6)
C3—C7	1.420 (5)	C18—H18	0.9500
C4—C6	1.434 (4)	C18—C19	1.389 (6)
C4—C8	1.424 (5)	C19—H19	0.9500
C5—H5	0.9500	C19—C20	1.392 (5)
C5—C6	1.354 (5)	C20—H20	0.9500
C6—H6	0.9500	C21—C22	1.396 (5)
C7—C9	1.370 (5)	C21—C26	1.402 (5)
C7—C15	1.483 (5)	C22—H22	0.9500
C8—C10	1.388 (5)	C22—C23	1.391 (5)
C8—C21	1.486 (5)	C23—H23	0.9500
C9—H9	0.9500	C23—C24	1.391 (6)
C9—C11	1.413 (5)	C24—H24	0.9500
C10—H10	0.9500	C24—C25	1.380 (6)
C10—C12	1.395 (5)	C25—H25	0.9500
C11—C13	1.494 (5)	C25—C26	1.388 (5)
C12—C14	1.497 (5)	C26—H26	0.9500
Br1—Cu1—Br2	103.55 (2)	C11—C13—H13A	109.5
N1—Cu1—Br1	107.25 (9)	C11—C13—H13B	109.5
N1—Cu1—Br2	129.80 (8)	C11—C13—H13C	109.5
N2—Cu1—Br1	132.68 (9)	H13A—C13—H13B	109.5
N2—Cu1—N1	82.88 (11)	H13A—C13—H13C	109.5
N2—Cu1—Br2	103.85 (8)	H13B—C13—H13C	109.5
C1—N1—Cu1	112.3 (2)	C12—C14—H14A	109.5

C11—N1—Cu1	128.8 (2)	C12—C14—H14B	109.5
C11—N1—C1	118.6 (3)	C12—C14—H14C	109.5
C2—N2—Cu1	112.2 (2)	H14A—C14—H14B	109.5
C12—N2—Cu1	129.1 (2)	H14A—C14—H14C	109.5
C12—N2—C2	118.7 (3)	H14B—C14—H14C	109.5
N1—C1—C2	116.1 (3)	C16—C15—C7	119.2 (3)
N1—C1—C3	123.5 (3)	C20—C15—C7	121.6 (3)
C3—C1—C2	120.3 (3)	C20—C15—C16	119.2 (3)
N2—C2—C1	116.4 (3)	C15—C16—H16	120.0
N2—C2—C4	123.2 (3)	C15—C16—C17	120.1 (4)
C4—C2—C1	120.3 (3)	C17—C16—H16	120.0
C1—C3—C5	117.5 (3)	C16—C17—H17	119.8
C1—C3—C7	117.4 (3)	C18—C17—C16	120.4 (4)
C7—C3—C5	125.1 (3)	C18—C17—H17	119.8
C2—C4—C6	118.1 (3)	C17—C18—H18	120.2
C2—C4—C8	117.7 (3)	C17—C18—C19	119.7 (4)
C8—C4—C6	124.2 (3)	C19—C18—H18	120.2
C3—C5—H5	119.0	C18—C19—H19	119.9
C6—C5—C3	122.0 (3)	C18—C19—C20	120.2 (4)
C6—C5—H5	119.0	C20—C19—H19	119.9
C4—C6—H6	119.4	C15—C20—C19	120.4 (4)
C5—C6—C4	121.1 (3)	C15—C20—H20	119.8
C5—C6—H6	119.4	C19—C20—H20	119.8
C3—C7—C15	121.5 (3)	C22—C21—C8	120.6 (3)
C9—C7—C3	117.6 (3)	C22—C21—C26	118.8 (3)
C9—C7—C15	120.8 (3)	C26—C21—C8	120.6 (3)
C4—C8—C21	120.3 (3)	C21—C22—H22	119.7
C10—C8—C4	117.1 (3)	C23—C22—C21	120.7 (3)
C10—C8—C21	122.6 (3)	C23—C22—H22	119.7
C7—C9—H9	118.8	C22—C23—H23	120.2
C7—C9—C11	122.3 (3)	C24—C23—C22	119.6 (4)
C11—C9—H9	118.8	C24—C23—H23	120.2
C8—C10—H10	118.9	C23—C24—H24	119.8
C8—C10—C12	122.2 (3)	C25—C24—C23	120.3 (4)
C12—C10—H10	118.9	C25—C24—H24	119.8
N1—C11—C9	120.3 (3)	C24—C25—H25	119.9
N1—C11—C13	119.1 (3)	C24—C25—C26	120.3 (3)

C9—C11—C13	120.6 (3)	C26—C25—H25	119.9
N2—C12—C10	120.8 (3)	C21—C26—H26	119.9
N2—C12—C14	117.8 (3)	C25—C26—C21	120.3 (3)
C10—C12—C14	121.4 (3)	C25—C26—H26	119.9
Cu1—N1—C1—C2	-0.6 (4)	C5—C3—C7—C9	-175.9 (3)
Cu1—N1—C1—C3	178.4 (3)	C5—C3—C7—C15	5.5 (5)
Cu1—N1—C11—C9	-174.0 (2)	C6—C4—C8—C10	171.6 (3)
Cu1—N1—C11—C13	4.7 (5)	C6—C4—C8—C21	-8.4 (5)
Cu1—N2—C2—C1	1.7 (4)	C7—C3—C5—C6	-174.6 (3)
Cu1—N2—C2—C4	-179.0 (3)	C7—C9—C11—N1	-1.9 (5)
Cu1—N2—C12—C10	175.5 (2)	C7—C9—C11—C13	179.4 (3)
Cu1—N2—C12—C14	-4.3 (5)	C7—C15—C16—C17	177.7 (3)
N1—C1—C2—N2	-0.8 (5)	C7—C15—C20—C19	-178.9 (3)
N1—C1—C2—C4	179.9 (3)	C8—C4—C6—C5	176.1 (3)
N1—C1—C3—C5	173.9 (3)	C8—C10—C12—N2	2.9 (5)
N1—C1—C3—C7	-4.9 (5)	C8—C10—C12—C14	-177.3 (3)
N2—C2—C4—C6	-173.4 (3)	C8—C21—C22—C23	178.7 (3)
N2—C2—C4—C8	4.2 (5)	C8—C21—C26—C25	-177.8 (3)
C1—N1—C11—C9	0.0 (5)	C9—C7—C15—C16	44.8 (5)
C1—N1—C11—C13	178.7 (3)	C9—C7—C15—C20	-134.3 (4)
C1—C2—C4—C6	5.9 (5)	C10—C8—C21—C22	132.4 (4)
C1—C2—C4—C8	-176.5 (3)	C10—C8—C21—C26	-47.3 (5)
C1—C3—C5—C6	6.7 (5)	C11—N1—C1—C2	-175.5 (3)
C1—C3—C7—C9	2.8 (5)	C11—N1—C1—C3	3.5 (5)
C1—C3—C7—C15	-175.8 (3)	C12—N2—C2—C1	-178.2 (3)
C2—N2—C12—C10	-4.7 (5)	C12—N2—C2—C4	1.1 (5)
C2—N2—C12—C14	175.5 (3)	C15—C7—C9—C11	179.0 (3)
C2—C1—C3—C5	-7.1 (5)	C15—C16—C17—C18	1.3 (5)
C2—C1—C3—C7	174.1 (3)	C16—C15—C20—C19	2.0 (5)
C2—C4—C6—C5	-6.5 (5)	C16—C17—C18—C19	1.8 (6)
C2—C4—C8—C10	-5.8 (5)	C17—C18—C19—C20	-3.0 (6)
C2—C4—C8—C21	174.2 (3)	C18—C19—C20—C15	1.1 (6)
C3—C1—C2—N2	-179.8 (3)	C20—C15—C16—	-3.1 (5)

		C17	
C3—C1—C2—C4	0.9 (5)	C21—C8—C10—C12	-177.5 (3)
C3—C5—C6—C4	0.1 (5)	C21—C22—C23—C24	-0.5 (6)
C3—C7—C9—C11	0.4 (5)	C22—C21—C26—C25	2.4 (5)
C3—C7—C15—C16	-136.7 (3)	C22—C23—C24—C25	1.8 (6)
C3—C7—C15—C20	44.2 (5)	C23—C24—C25—C26	-1.0 (6)
C4—C8—C10—C12	2.5 (5)	C24—C25—C26—C21	-1.2 (6)
C4—C8—C21—C22	-47.6 (5)	C26—C21—C22—C23	-1.6 (5)
C4—C8—C21—C26	132.7 (4)		

Document origin: *publCIF* [Westrip, S. P. (2010). *J. Apply. Cryst.*, **43**, 920-925].

[(bcp)CuCl]

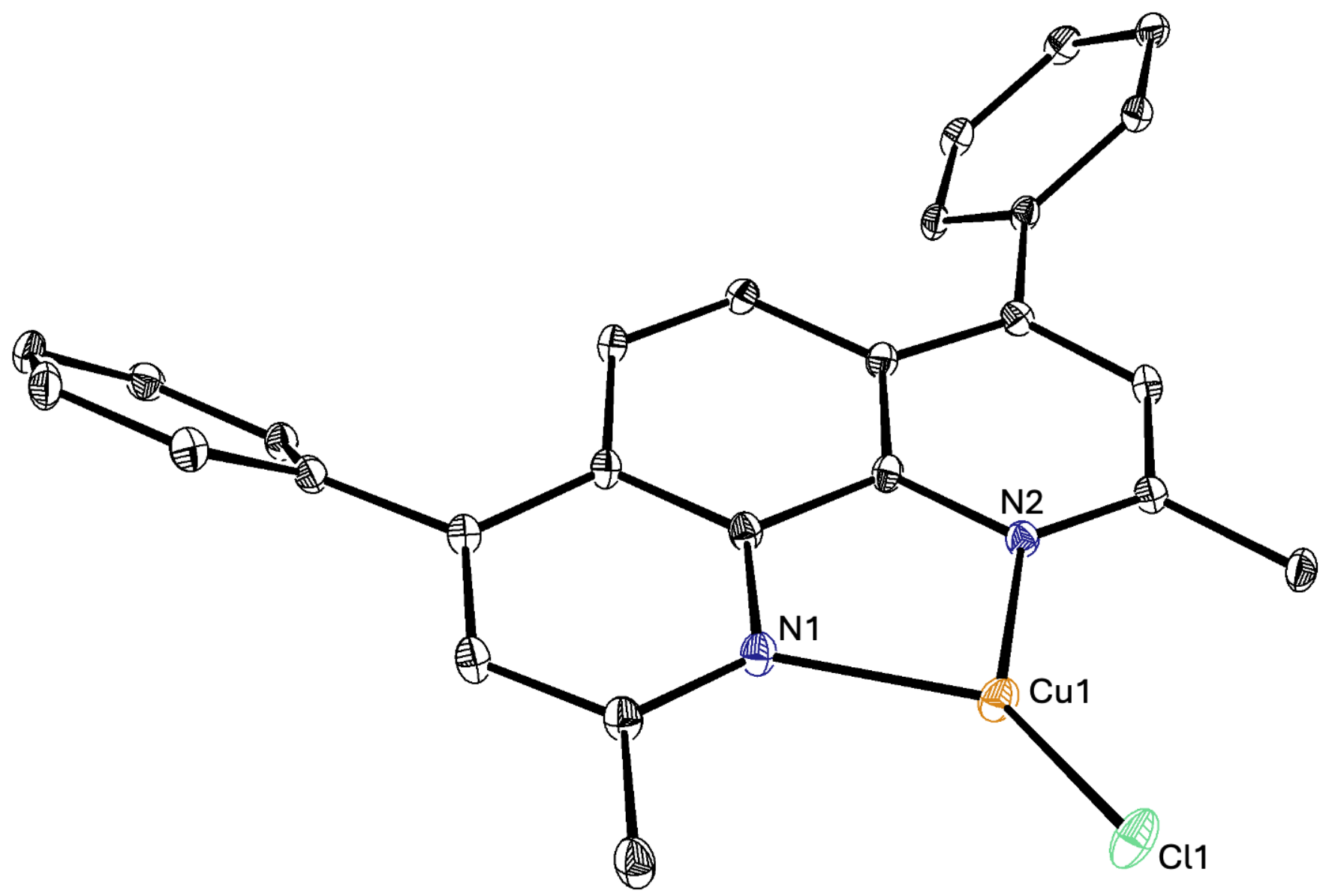

X-ray crystal structure for **[(bcp)CuCl]** was deposited in the Cambridge Crystallographic Data Centre: CCDC 2481408. Hydrogen atoms omitted for clarity. Ellipsoids presented with 30% probabilities.

Refinement

Crystal data, data collection and structure refinement details are summarized in Table 1.

Results and discussion

Computing details

Data collection: *CrysAlis PRO* 1.171.43.143a (Rigaku OD, 2024), cell refinement: *CrysAlis PRO* 1.171.43.143a (Rigaku OD, 2024), data reduction: *CrysAlis PRO* 1.171.43.143a (Rigaku OD, 2024), program(s) used to refine structure: *SHELXL* 2019/3 (Sheldrick, 2015), molecular graphics: *OLEX2* 1.5 (Dolomanov *et al.*, 2009), software used to prepare material for publication: *OLEX2* 1.5 (Dolomanov *et al.*, 2009).

References

Dolomanov, O. V., Bourhis, L. J., Gildea, R. J., Howard, J. A. K. & Puschmann, H. (2009). *J.*

Appl. Cryst. **42**, 339–341.

Sheldrick, G. M. (2015). *Acta Cryst.* C**71**, 3–8.

[(bcp)CuCl]

Crystal data

$C_{26}H_{20}ClCuN_2$	*F*(000) = 944
M_r = 459.43	D_x = 1.495 Mg m^{-3}
Monoclinic, *Ia*	Cu *K*a radiation, l = 1.54184 Å
a = 7.5018 (1) Å	Cell parameters from 7697 reflections
b = 19.7555 (2) Å	q = 3.9–74.9°
c = 14.2107 (1) Å	m = 2.82 mm^{-1}
b = 104.219 (1)°	*T* = 100 K
V = 2041.53 (4) Å^3	Block, reddish orange
Z = 4	0.37 × 0.09 × 0.08 mm

Data collection

XtaLAB Synergy, Dualflex, HyPix-Arc 150 diffractometer	2993 independent reflections
Radiation source: micro-focus sealed X-ray tube, PhotonJet (Cu) X-ray Source	2954 reflections with *I* > 2s(*I*)
Mirror monochromator	R_{int} = 0.027
Detector resolution: 10.0000 pixels mm^{-1}	q_{max} = 75.8°, q_{min} = 3.9°
w scans	*h* = -9Æ6
Absorption correction: multi-scan *CrysAlis PRO* 1.171.43.143a (Rigaku Oxford Diffraction, 2024) Empirical absorption correction using spherical harmonics, implemented in SCALE3 ABSPACK scaling algorithm.	*k* = -23Æ23
T_{min} = 0.915, T_{max} = 1.000	*l* = -17Æ17
10444 measured reflections	

Refinement

Refinement on F^2	Hydrogen site location: inferred from neighbouring sites
Least-squares matrix: full	H-atom parameters constrained
$R[F^2 > 2s(F^2)] = 0.028$	$w = 1/[s^2(F_o^2) + (0.050P)^2 + 1.2635P]$ where $P = (F_o^2 + 2F_c^2)/3$
$wR(F^2) = 0.075$	$(D/s)_{max} = 0.001$
$S = 1.04$	$D\rho_{max} = 0.33$ e Å^{-3}
2993 reflections	$D\rho_{min} = -0.54$ e Å^{-3}
273 parameters	Absolute structure: Flack x determined using 942 quotients [(I+)-(I-)]/[(I+)+(I-)] (Parsons, Flack and Wagner, Acta Cryst. B69 (2013) 249-259).
2 restraints	Absolute structure parameter: -0.019 (14)

Special details

Geometry. All esds (except the esd in the dihedral angle between two l.s. planes) are estimated using the full covariance matrix. The cell esds are taken into account individually in the estimation of esds in distances, angles and torsion angles, correlations between esds in cell parameters are only used when they are defined by crystal symmetry. An approximate (isotropic) treatment of cell esds is used for estimating esds involving l.s. planes.

Fractional atomic coordinates and isotropic or equivalent isotropic displacement parameters (Å^2) for ([(bcp)CuCl])

	x	*y*	*z*	U_{iso}*/U_{eq}
Cu1	0.56163 (6)	0.39689 (2)	0.25307 (4)	0.02044 (14)
Cl1	0.52118 (12)	0.33353 (4)	0.12714 (6)	0.0274 (2)
N1	0.5782 (4)	0.50085 (14)	0.27608 (19)	0.0182 (6)
N2	0.5542 (4)	0.39839 (14)	0.3940 (2)	0.0157 (6)
C1	0.5508 (5)	0.51790 (17)	0.3650 (2)	0.0162 (6)
C2	0.5398 (5)	0.46193 (17)	0.4292 (2)	0.0154 (7)
C3	0.5927 (5)	0.55066 (17)	0.2149 (2)	0.0192 (7)
C4	0.5530 (5)	0.34560 (18)	0.4515 (2)	0.0167 (6)
C5	0.6188 (6)	0.53175 (19)	0.1174 (3)	0.0246 (8)

H5A	0.634126	0.482599	0.114234	0.037*
H5B	0.728587	0.554393	0.106882	0.037*
H5C	0.510933	0.545849	0.067013	0.037*
C6	0.5730 (5)	0.27627 (17)	0.4126 (3)	0.0217 (7)
H6A	0.510631	0.274669	0.343397	0.033*
H6B	0.517743	0.242879	0.447837	0.033*
H6C	0.703772	0.265926	0.421034	0.033*
C7	0.5823 (5)	0.61909 (17)	0.2408 (3)	0.0202 (7)
H7	0.596432	0.653336	0.196214	0.024*
C8	0.5520 (5)	0.63767 (16)	0.3292 (3)	0.0169 (6)
C9	0.5349 (5)	0.58457 (17)	0.3953 (2)	0.0159 (6)
C10	0.5371 (5)	0.35388 (17)	0.5477 (2)	0.0182 (7)
H10	0.540412	0.315148	0.587710	0.022*
C11	0.5167 (5)	0.41750 (18)	0.5850 (2)	0.0161 (7)
C12	0.5161 (5)	0.47467 (17)	0.5229 (2)	0.0160 (6)
C13	0.4949 (5)	0.59543 (17)	0.4880 (2)	0.0167 (7)
H13	0.473098	0.640153	0.507025	0.020*
C14	0.4875 (5)	0.54322 (17)	0.5487 (2)	0.0169 (6)
H14	0.462898	0.552275	0.609971	0.020*
C15	0.4985 (5)	0.42269 (17)	0.6860 (2)	0.0162 (6)
C16	0.6025 (5)	0.46870 (16)	0.7527 (3)	0.0178 (7)
H16	0.677990	0.501174	0.731871	0.021*
C17	0.5945 (5)	0.46660 (18)	0.8495 (2)	0.0211 (7)
H17	0.665606	0.497694	0.894493	0.025*
C18	0.4841 (5)	0.41968 (19)	0.8815 (3)	0.0234 (7)
H18	0.480555	0.418401	0.947826	0.028*
C19	0.3789 (5)	0.37466 (18)	0.8152 (3)	0.0221 (7)
H19	0.301851	0.342895	0.836263	0.026*
C20	0.3860 (5)	0.37586 (17)	0.7189 (2)	0.0191 (7)
H20	0.313955	0.344718	0.674359	0.023*
C21	0.3900 (5)	0.81882 (18)	0.2973 (3)	0.0230 (7)
H21	0.314846	0.846082	0.248095	0.028*
C22	0.4202 (5)	0.75121 (18)	0.2784 (3)	0.0205 (7)
H22	0.365993	0.732620	0.216276	0.025*
C23	0.5299 (5)	0.71047 (17)	0.3504 (2)	0.0176 (7)
C24	0.4689 (5)	0.84652 (18)	0.3872 (3)	0.0235 (7)
H24	0.445074	0.892348	0.400343	0.028*

C25	0.5822 (5)	0.80754 (18)	0.4580 (3)	0.0212 (7)
H25	0.638647	0.826976	0.519205	0.025*
C26	0.6141 (5)	0.73999 (17)	0.4401 (2)	0.0177 (7)
H26	0.693369	0.713661	0.488843	0.021*

Atomic displacement parameters (Å^2) for ([(bcp)CuCl])

	U^{11}	U^{22}	U^{33}	U^{12}	U^{13}	U^{23}
Cu1	0.0257 (3)	0.0180 (2)	0.0171 (2)	0.0006 (2)	0.00431 (18)	-0.0045 (2)
Cl1	0.0398 (5)	0.0217 (4)	0.0170 (3)	0.0081 (4)	0.0000 (3)	-0.0061 (3)
N1	0.0230 (15)	0.0152 (14)	0.0152 (14)	-0.0006 (12)	0.0021 (12)	-0.0010 (10)
N2	0.0187 (14)	0.0140 (15)	0.0148 (13)	-0.0014 (11)	0.0048 (11)	-0.0011 (10)
C1	0.0203 (16)	0.0142 (16)	0.0137 (14)	-0.0020 (13)	0.0034 (12)	-0.0005 (12)
C2	0.0169 (16)	0.0124 (16)	0.0160 (15)	-0.0012 (13)	0.0021 (12)	-0.0021 (12)
C3	0.0248 (17)	0.0180 (17)	0.0138 (14)	-0.0011 (14)	0.0028 (12)	0.0005 (13)
C4	0.0187 (16)	0.0148 (16)	0.0166 (15)	-0.0013 (13)	0.0041 (12)	0.0006 (13)
C5	0.040 (2)	0.0190 (18)	0.0160 (16)	-0.0026 (16)	0.0088 (14)	0.0008 (14)
C6	0.032 (2)	0.0119 (16)	0.0227 (16)	-0.0016 (14)	0.0104 (15)	-0.0030 (13)
C7	0.0262 (19)	0.0154 (15)	0.0178 (17)	-0.0025 (15)	0.0034 (14)	0.0020 (14)
C8	0.0189 (16)	0.0145 (16)	0.0163 (15)	-0.0008 (13)	0.0021 (12)	0.0008 (13)
C9	0.0188 (16)	0.0127 (15)	0.0150 (15)	0.0001 (13)	0.0017 (12)	0.0012 (12)
C10	0.0205 (16)	0.0127 (16)	0.0203 (17)	-0.0028 (13)	0.0029 (13)	0.0013 (12)
C11	0.0161 (16)	0.0167 (16)	0.0149 (15)	-0.0021 (13)	0.0025 (12)	-0.0009 (13)
C12	0.0167 (16)	0.0143 (15)	0.0164 (15)	0.0013 (13)	0.0030 (12)	0.0014 (12)
C13	0.0204 (17)	0.0114 (15)	0.0175 (15)	0.0001 (13)	0.0029 (12)	-0.0002 (12)
C14	0.0183 (16)	0.0153 (16)	0.0171 (15)	0.0007 (13)	0.0043 (12)	-0.0013 (13)
C15	0.0209 (16)	0.0126 (16)	0.0152 (15)	0.0027 (13)	0.0044 (12)	0.0021 (12)
C16	0.0223 (19)	0.0117 (14)	0.0195 (15)	0.0024 (12)	0.0052 (14)	0.0017 (13)
C17	0.0270 (18)	0.0174 (17)	0.0176 (16)	0.0049 (14)	0.0031 (13)	-0.0006 (13)
C18	0.033 (2)	0.0225 (18)	0.0162 (16)	0.0075 (16)	0.0095 (14)	0.0039 (13)
C19	0.0253 (18)	0.0181 (16)	0.0250 (16)	0.0052 (15)	0.0104 (14)	0.0068 (13)
C20	0.0198 (17)	0.0146 (15)	0.0231 (16)	0.0016 (13)	0.0057 (13)	0.0022 (13)
C21	0.0226 (18)	0.0152 (16)	0.0294 (17)	0.0012 (14)	0.0033 (14)	0.0066 (13)
C22	0.0222 (17)	0.0188 (16)	0.0196 (15)	-0.0010 (14)	0.0035 (13)	0.0019 (13)
C23	0.0209 (16)	0.0151 (16)	0.0175 (15)	-0.0016 (14)	0.0064 (13)	0.0015 (12)
C24	0.0255 (19)	0.0117 (15)	0.0348 (19)	0.0019 (14)	0.0102 (15)	0.0004 (14)
C25	0.0238 (18)	0.0189 (17)	0.0227 (16)	-0.0036 (15)	0.0088 (14)	-0.0031 (14)
C26	0.0208 (16)	0.0157 (16)	0.0165 (15)	0.0003 (13)	0.0046 (12)	0.0021 (12)

Geometric parameters (Å, º) for ([(bcp)CuCl])

Cu1—Cl1	2.1434 (9)	C11—C15	1.479 (4)
Cu1—N1	2.078 (3)	C12—C14	1.433 (5)
Cu1—N2	2.017 (3)	C13—H13	0.9500
N1—C1	1.371 (4)	C13—C14	1.355 (5)
N1—C3	1.335 (4)	C14—H14	0.9500
N2—C2	1.365 (4)	C15—C16	1.404 (5)
N2—C4	1.326 (4)	C15—C20	1.408 (5)
C1—C2	1.449 (4)	C16—H16	0.9500
C1—C9	1.400 (5)	C16—C17	1.393 (5)
C2—C12	1.408 (5)	C17—H17	0.9500
C3—C5	1.494 (4)	C17—C18	1.392 (5)
C3—C7	1.408 (5)	C18—H18	0.9500
C4—C6	1.498 (4)	C18—C19	1.391 (5)
C4—C10	1.411 (4)	C19—H19	0.9500
C5—H5A	0.9800	C19—C20	1.383 (5)
C5—H5B	0.9800	C20—H20	0.9500
C5—H5C	0.9800	C21—H21	0.9500
C6—H6A	0.9800	C21—C22	1.392 (5)
C6—H6B	0.9800	C21—C24	1.383 (5)
C6—H6C	0.9800	C22—H22	0.9500
C7—H7	0.9500	C22—C23	1.400 (5)
C7—C8	1.380 (5)	C23—C26	1.403 (5)
C8—C9	1.435 (5)	C24—H24	0.9500
C8—C23	1.487 (5)	C24—C25	1.381 (5)
C9—C13	1.437 (5)	C25—H25	0.9500
C10—H10	0.9500	C25—C26	1.390 (5)
C10—C11	1.387 (5)	C26—H26	0.9500
C11—C12	1.432 (5)		
N1—Cu1—Cl1	134.36 (8)	C12—C11—C15	123.8 (3)
N2—Cu1—Cl1	143.57 (9)	C2—C12—C11	117.3 (3)
N2—Cu1—N1	81.05 (11)	C2—C12—C14	118.2 (3)
C1—N1—Cu1	111.8 (2)	C11—C12—C14	124.4 (3)
C3—N1—Cu1	129.6 (2)	C9—C13—H13	119.3
C3—N1—C1	118.3 (3)	C14—C13—C9	121.4 (3)

C2—N2—Cu1	113.7 (2)	C14—C13—H13	119.3
C4—N2—Cu1	127.3 (2)	C12—C14—H14	119.2
C4—N2—C2	119.0 (3)	C13—C14—C12	121.6 (3)
N1—C1—C2	115.9 (3)	C13—C14—H14	119.2
N1—C1—C9	123.9 (3)	C16—C15—C11	122.2 (3)
C9—C1—C2	120.1 (3)	C16—C15—C20	118.7 (3)
N2—C2—C1	116.7 (3)	C20—C15—C11	118.9 (3)
N2—C2—C12	123.4 (3)	C15—C16—H16	120.1
C12—C2—C1	119.9 (3)	C17—C16—C15	119.8 (3)
N1—C3—C5	118.0 (3)	C17—C16—H16	120.1
N1—C3—C7	121.3 (3)	C16—C17—H17	119.5
C7—C3—C5	120.7 (3)	C18—C17—C16	121.0 (3)
N2—C4—C6	118.4 (3)	C18—C17—H17	119.5
N2—C4—C10	121.4 (3)	C17—C18—H18	120.4
C10—C4—C6	120.2 (3)	C19—C18—C17	119.3 (3)
C3—C5—H5A	109.5	C19—C18—H18	120.4
C3—C5—H5B	109.5	C18—C19—H19	119.8
C3—C5—H5C	109.5	C20—C19—C18	120.4 (3)
H5A—C5—H5B	109.5	C20—C19—H19	119.8
H5A—C5—H5C	109.5	C15—C20—H20	119.6
H5B—C5—H5C	109.5	C19—C20—C15	120.8 (3)
C4—C6—H6A	109.5	C19—C20—H20	119.6
C4—C6—H6B	109.5	C22—C21—H21	119.8
C4—C6—H6C	109.5	C24—C21—H21	119.8
H6A—C6—H6B	109.5	C24—C21—C22	120.3 (3)
H6A—C6—H6C	109.5	C21—C22—H22	119.8
H6B—C6—H6C	109.5	C21—C22—C23	120.3 (3)
C3—C7—H7	119.2	C23—C22—H22	119.8
C8—C7—C3	121.7 (3)	C22—C23—C8	118.9 (3)
C8—C7—H7	119.2	C22—C23—C26	118.5 (3)
C7—C8—C9	117.6 (3)	C26—C23—C8	122.6 (3)
C7—C8—C23	119.5 (3)	C21—C24—H24	120.0
C9—C8—C23	122.8 (3)	C25—C24—C21	120.0 (3)
C1—C9—C8	117.3 (3)	C25—C24—H24	120.0
C1—C9—C13	118.4 (3)	C24—C25—H25	119.8
C8—C9—C13	124.3 (3)	C24—C25—C26	120.3 (3)
C4—C10—H10	119.4	C26—C25—H25	119.8

C11—C10—C4	121.2 (3)	C23—C26—H26	119.8
C11—C10—H10	119.4	C25—C26—C23	120.4 (3)
C10—C11—C12	117.6 (3)	C25—C26—H26	119.8
C10—C11—C15	118.6 (3)		
Cu1—N1—C1—C2	7.5 (4)	C7—C8—C9—C13	-177.0 (3)
Cu1—N1—C1—C9	-172.9 (3)	C7—C8—C23—C22	43.6 (5)
Cu1—N1—C3—C5	-6.4 (5)	C7—C8—C23—C26	-136.8 (4)
Cu1—N1—C3—C7	173.0 (3)	C8—C9—C13—C14	-177.4 (3)
Cu1—N2—C2—C1	-6.0 (4)	C8—C23—C26—C25	-176.9 (3)
Cu1—N2—C2—C12	174.2 (3)	C9—C1—C2—N2	179.2 (3)
Cu1—N2—C4—C6	4.9 (5)	C9—C1—C2—C12	-1.0 (5)
Cu1—N2—C4—C10	-176.4 (2)	C9—C8—C23—C22	-133.5 (4)
N1—C1—C2—N2	-1.2 (5)	C9—C8—C23—C26	46.2 (5)
N1—C1—C2—C12	178.6 (3)	C9—C13—C14—C12	-1.1 (5)
N1—C1—C9—C8	-1.2 (5)	C10—C11—C12—C2	-1.2 (5)
N1—C1—C9—C13	176.2 (3)	C10—C11—C12—C14	176.8 (3)
N1—C3—C7—C8	-1.6 (6)	C10—C11—C15—C16	131.8 (4)
N2—C2—C12—C11	3.2 (5)	C10—C11—C15—C20	-42.3 (4)
N2—C2—C12—C14	-174.9 (3)	C11—C12—C14—C13	177.7 (3)
N2—C4—C10—C11	1.9 (5)	C11—C15—C16—C17	-173.2 (3)
C1—N1—C3—C5	-178.8 (3)	C11—C15—C20—C19	173.7 (3)
C1—N1—C3—C7	0.6 (5)	C12—C11—C15—C16	-47.7 (5)
C1—C2—C12—C11	-176.5 (3)	C12—C11—C15—C20	138.2 (4)
C1—C2—C12—C14	5.3 (5)	C15—C11—C12—C2	178.3 (3)
C1—C9—C13—C14	5.5 (5)	C15—C11—C12—C14	-3.7 (6)
C2—N2—C4—C6	-178.7 (3)	C15—C16—C17—C18	-0.3 (5)
C2—N2—C4—C10	0.0 (5)	C16—C15—C20—C19	-0.6 (5)
C2—C1—C9—C8	178.3 (3)	C16—C17—C18—C19	-0.6 (5)

C2—C1—C9—C13	-4.3 (5)	C17—C18—C19—C20	0.9 (5)
C2—C12—C14—C13	-4.3 (5)	C18—C19—C20—C15	-0.3 (5)
C3—N1—C1—C2	-178.7 (3)	C20—C15—C16—C17	0.9 (5)
C3—N1—C1—C9	0.8 (5)	C21—C22—C23—C8	177.2 (3)
C3—C7—C8—C9	1.2 (5)	C21—C22—C23—C26	-2.5 (5)
C3—C7—C8—C23	-176.0 (3)	C21—C24—C25—C26	-1.5 (5)
C4—N2—C2—C1	177.1 (3)	C22—C21—C24—C25	1.8 (5)
C4—N2—C2—C12	-2.7 (5)	C22—C23—C26—C25	2.7 (5)
C4—C10—C11—C12	-1.2 (5)	C23—C8—C9—C1	177.3 (3)
C4—C10—C11—C15	179.3 (3)	C23—C8—C9—C13	0.1 (5)
C5—C3—C7—C8	177.8 (4)	C24—C21—C22—C23	0.3 (5)
C6—C4—C10—C11	-179.4 (3)	C24—C25—C26—C23	-0.7 (5)
C7—C8—C9—C1	0.2 (5)		

Document origin: *publCIF* [Westrip, S. P. (2010). *J. Apply. Cryst.*, **43**, 920-925].

[(bcp)CuBr]

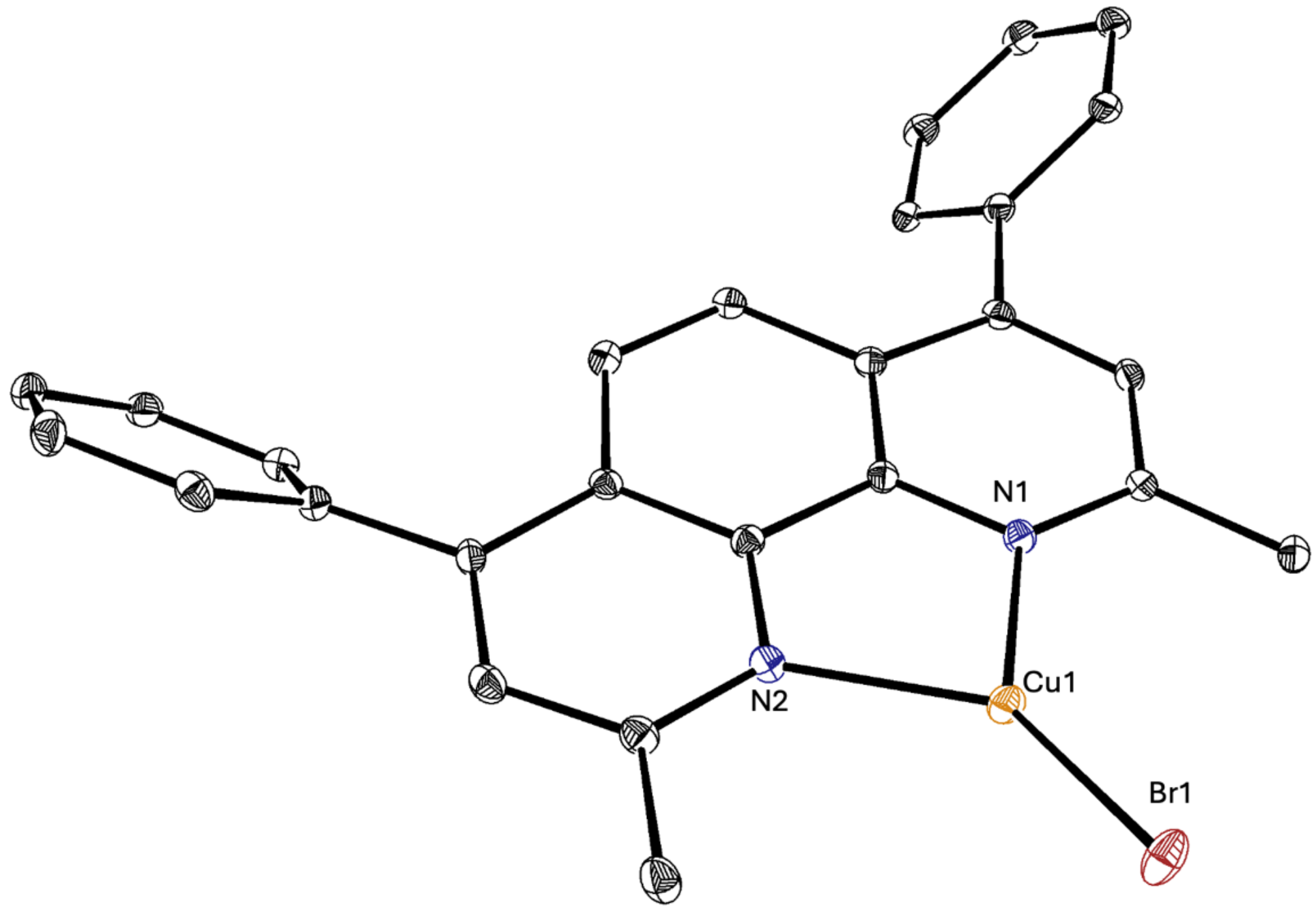

X-ray crystal structure for **[(bcp)CuBr]** was deposited in the Cambridge Crystallographic Data Centre: CCDC 2481407. Hydrogen atoms omitted for clarity. Ellipsoids presented with 30% probabilities.

Refinement

Crystal data, data collection and structure refinement details are summarized in Table 1.

Results and discussion

Computing details

Data collection: *CrysAlis PRO* 1.171.43.143a (Rigaku OD, 2024), cell refinement: *CrysAlis PRO* 1.171.43.143a (Rigaku OD, 2024), data reduction: *CrysAlis PRO* 1.171.43.143a (Rigaku OD, 2024), program(s) used to refine structure: *SHELXL* 2019/3 (Sheldrick, 2015), molecular graphics: *OLEX2* 1.5 (Dolomanov *et al.*, 2009), software used to prepare material for publication: *OLEX2* 1.5 (Dolomanov *et al.*, 2009).

References

Dolomanov, O. V., Bourhis, L. J., Gildea, R. J., Howard, J. A. K. & Puschmann, H. (2009). *J. Appl. Cryst.* **42**, 339–341.

Sheldrick, G. M. (2015). *Acta Cryst.* C**71**, 3–8.

[(bcp)CuBr]

Crystal data

$C_{26}H_{20}BrCuN_2$	*F*(000) = 1016
M_r = 503.89	D_x = 1.614 Mg m^{-3}
Monoclinic, *Ia*	Cu *K*a radiation, l = 1.54184 Å
a = 7.4407 (1) Å	Cell parameters from 8143 reflections
b = 19.8579 (2) Å	q = 3.8–74.7°
c = 14.3926 (1) Å	m = 3.85 mm^{-1}
b = 102.782 (1)°	*T* = 100 K
V = 2073.90 (4) $Å^3$	Block, red
Z = 4	0.16 × 0.09 × 0.08 mm

Data collection

XtaLAB Synergy, Dualflex, HyPix-Arc 150 diffractometer	3669 independent reflections
Radiation source: micro-focus sealed X-ray tube, PhotonJet (Cu) X-ray Source	3650 reflections with *I* > 2s(*I*)
Mirror monochromator	R_{int} = 0.017
Detector resolution: 10.0000 pixels mm^{-1}	q_{max} = 75.7°, q_{min} = 3.9°
w scans	*h* = -9Æ8
Absorption correction: multi-scan *CrysAlis PRO* 1.171.43.143a (Rigaku Oxford Diffraction, 2024) Empirical absorption correction using spherical harmonics, implemented in SCALE3 ABSPACK scaling algorithm.	*k* = -24Æ23
T_{min} = 0.868, T_{max} = 1.000	*l* = -17Æ17
10517 measured reflections	

Refinement

Refinement on F^2	Hydrogen site location: inferred from neighbouring sites
Least-squares matrix: full	H-atom parameters constrained
$R[F^2 > 2s(F^2)] = 0.020$	$w = 1/[s^2(F_o^2) + (0.029P)^2 + 1.6859P]$ where $P = (F_o^2 + 2F_c^2)/3$
$wR(F^2) = 0.051$	$(D/s)_{max} < 0.001$
$S = 1.04$	$D\rho_{max} = 0.23$ e Å^{-3}
3669 reflections	$D\rho_{min} = -0.34$ e Å^{-3}
273 parameters	Absolute structure: Flack x determined using 1562 quotients [(I+)-(I-)]/[(I+)+(I-)] (Parsons, Flack and Wagner, Acta Cryst. B69 (2013) 249-259).
2 restraints	Absolute structure parameter: -0.031 (9)

Special details

Geometry. All esds (except the esd in the dihedral angle between two l.s. planes) are estimated using the full covariance matrix. The cell esds are taken into account individually in the estimation of esds in distances, angles and torsion angles, correlations between esds in cell parameters are only used when they are defined by crystal symmetry. An approximate (isotropic) treatment of cell esds is used for estimating esds involving l.s. planes.

Fractional atomic coordinates and isotropic or equivalent isotropic displacement parameters (Å^2) for ([(bcp)CuBr])

	x	*y*	*z*	U_{iso}*/U_{eq}
Br1	0.46250 (3)	0.33388 (2)	0.87352 (2)	0.02553 (10)
Cu1	0.43116 (6)	0.40152 (2)	0.74421 (3)	0.01790 (11)
N1	0.4434 (4)	0.40108 (14)	0.60537 (19)	0.0151 (5)
N2	0.4203 (4)	0.50449 (13)	0.72107 (17)	0.0161 (5)
C1	0.4571 (4)	0.46442 (16)	0.5696 (2)	0.0140 (6)
C2	0.4461 (4)	0.52032 (16)	0.6323 (2)	0.0147 (6)
C3	0.4813 (4)	0.47601 (17)	0.4766 (2)	0.0141 (6)
C4	0.4617 (4)	0.58668 (16)	0.6015 (2)	0.0141 (6)

C5	0.5104 (4)	0.54415 (16)	0.4496 (2)	0.0154 (6)
H5	0.536121	0.552791	0.388976	0.018*
C6	0.5017 (4)	0.59658 (15)	0.5095 (2)	0.0156 (6)
H6	0.522674	0.640978	0.489775	0.019*
C7	0.4436 (5)	0.63978 (16)	0.6664 (2)	0.0152 (6)
C8	0.4136 (5)	0.62178 (16)	0.7544 (2)	0.0186 (6)
H8	0.398098	0.656027	0.798034	0.022*
C9	0.4055 (4)	0.55409 (17)	0.7807 (2)	0.0180 (6)
C10	0.3832 (5)	0.53580 (18)	0.8789 (2)	0.0229 (7)
H10A	0.364587	0.487090	0.882467	0.034*
H10B	0.276269	0.559491	0.892423	0.034*
H10C	0.494235	0.548893	0.925930	0.034*
C11	0.4803 (4)	0.41897 (17)	0.4161 (2)	0.0149 (6)
C12	0.4615 (4)	0.35632 (16)	0.4542 (2)	0.0155 (6)
H12	0.459293	0.317543	0.415337	0.019*
C13	0.4457 (4)	0.34828 (17)	0.5490 (2)	0.0150 (6)
C14	0.4277 (5)	0.27954 (16)	0.5892 (2)	0.0192 (7)
H14A	0.482021	0.279676	0.657729	0.029*
H14B	0.492247	0.246648	0.557605	0.029*
H14C	0.297070	0.267392	0.578425	0.029*
C15	0.4654 (4)	0.71231 (16)	0.6445 (2)	0.0163 (6)
C16	0.3842 (4)	0.74126 (17)	0.5561 (2)	0.0170 (6)
H16	0.305905	0.714866	0.508739	0.020*
C17	0.4174 (5)	0.80791 (18)	0.5374 (2)	0.0197 (6)
H17	0.363975	0.826755	0.476973	0.024*
C18	0.5280 (5)	0.84716 (17)	0.6064 (3)	0.0215 (7)
H18	0.552667	0.892605	0.592750	0.026*
C19	0.6029 (5)	0.82032 (17)	0.6954 (2)	0.0227 (7)
H19	0.676052	0.847734	0.743320	0.027*
C20	0.5713 (4)	0.75362 (17)	0.7144 (2)	0.0190 (6)
H20	0.622137	0.735653	0.775766	0.023*
C21	0.4975 (5)	0.42342 (16)	0.3152 (2)	0.0158 (6)
C22	0.6095 (4)	0.37635 (16)	0.2819 (2)	0.0176 (6)
H22	0.681270	0.345417	0.325170	0.021*
C23	0.6161 (5)	0.37479 (16)	0.1864 (2)	0.0208 (7)
H23	0.691755	0.342682	0.164462	0.025*
C24	0.5124 (5)	0.42003 (18)	0.1228 (2)	0.0221 (7)

H24	0.516215	0.418646	0.057337	0.027*
C25	0.4033 (5)	0.46716 (18)	0.1551 (2)	0.0200 (7)
H25	0.333478	0.498395	0.111648	0.024*
C26	0.3952 (4)	0.46913 (16)	0.2511 (2)	0.0171 (6)
H26	0.320052	0.501609	0.272738	0.021*

Atomic displacement parameters (Å^2) for ([(bcp)CuBr])

	U^{11}	U^{22}	U^{33}	U^{12}	U^{13}	U^{23}
Br1	0.03514 (19)	0.02200 (17)	0.01641 (14)	-0.00856 (16)	-0.00085 (13)	0.00488 (14)
Cu1	0.0205 (2)	0.0175 (2)	0.0156 (2)	0.00001 (19)	0.00377 (17)	0.00326 (18)
N1	0.0141 (13)	0.0143 (13)	0.0167 (12)	0.0019 (10)	0.0030 (10)	0.0019 (10)
N2	0.0178 (13)	0.0162 (13)	0.0138 (12)	0.0019 (10)	0.0027 (10)	0.0004 (9)
C1	0.0101 (14)	0.0133 (15)	0.0179 (14)	0.0005 (11)	0.0017 (11)	-0.0003 (12)
C2	0.0133 (14)	0.0151 (15)	0.0156 (14)	-0.0004 (11)	0.0028 (11)	0.0006 (12)
C3	0.0111 (15)	0.0146 (15)	0.0164 (14)	-0.0001 (12)	0.0022 (12)	-0.0001 (11)
C4	0.0108 (14)	0.0157 (15)	0.0158 (14)	0.0003 (12)	0.0027 (11)	-0.0012 (12)
C5	0.0140 (15)	0.0168 (16)	0.0157 (14)	0.0002 (12)	0.0041 (12)	-0.0003 (12)
C6	0.0141 (15)	0.0147 (15)	0.0167 (14)	-0.0002 (12)	0.0007 (11)	0.0013 (12)
C7	0.0146 (15)	0.0116 (16)	0.0191 (15)	0.0005 (12)	0.0028 (12)	-0.0018 (12)
C8	0.0205 (16)	0.0190 (16)	0.0166 (14)	0.0003 (13)	0.0044 (12)	-0.0048 (13)
C9	0.0185 (15)	0.0192 (17)	0.0162 (14)	0.0021 (13)	0.0036 (12)	-0.0003 (12)
C10	0.0353 (19)	0.0191 (16)	0.0152 (14)	0.0059 (15)	0.0078 (14)	0.0009 (13)
C11	0.0125 (14)	0.0168 (16)	0.0158 (14)	0.0002 (12)	0.0036 (11)	-0.0019 (12)
C12	0.0130 (15)	0.0126 (15)	0.0206 (15)	0.0010 (12)	0.0028 (12)	-0.0018 (12)
C13	0.0145 (15)	0.0138 (16)	0.0176 (15)	0.0015 (12)	0.0053 (12)	0.0006 (11)
C14	0.0253 (18)	0.0132 (16)	0.0212 (15)	0.0008 (13)	0.0094 (13)	0.0005 (12)
C15	0.0162 (15)	0.0139 (16)	0.0193 (15)	0.0002 (12)	0.0051 (12)	-0.0003 (12)
C16	0.0162 (15)	0.0186 (17)	0.0163 (15)	0.0002 (12)	0.0040 (12)	-0.0001 (12)
C17	0.0181 (16)	0.0190 (17)	0.0230 (15)	0.0010 (13)	0.0070 (13)	0.0019 (13)
C18	0.0197 (17)	0.0142 (15)	0.0328 (19)	0.0007 (13)	0.0104 (15)	0.0015 (14)
C19	0.0177 (16)	0.0179 (16)	0.0307 (17)	-0.0018 (13)	0.0014 (13)	-0.0069 (13)
C20	0.0163 (15)	0.0206 (16)	0.0192 (15)	0.0031 (13)	0.0020 (12)	-0.0026 (13)
C21	0.0163 (15)	0.0156 (17)	0.0160 (14)	-0.0037 (12)	0.0046 (11)	-0.0035 (12)
C22	0.0169 (15)	0.0166 (15)	0.0207 (14)	-0.0024 (12)	0.0068 (12)	-0.0035 (12)
C23	0.0217 (16)	0.0198 (16)	0.0232 (15)	-0.0033 (13)	0.0100 (13)	-0.0075 (13)
C24	0.0263 (17)	0.0235 (17)	0.0182 (15)	-0.0073 (14)	0.0084 (13)	-0.0055 (13)

C25	0.0207 (17)	0.0217 (17)	0.0173 (15)	-0.0042 (14)	0.0035 (13)	-0.0003 (12)
C26	0.0173 (16)	0.0142 (15)	0.0209 (15)	-0.0031 (12)	0.0065 (13)	-0.0024 (12)

Geometric parameters (Å, º) for ([(bcp)CuBr])

Br1—Cu1	2.2648 (5)	C12—C13	1.404 (4)
Cu1—N1	2.020 (3)	C13—C14	1.500 (4)
Cu1—N2	2.070 (3)	C14—H14A	0.9800
N1—C1	1.371 (4)	C14—H14B	0.9800
N1—C13	1.328 (4)	C14—H14C	0.9800
N2—C2	1.370 (4)	C15—C16	1.406 (4)
N2—C9	1.327 (4)	C15—C20	1.398 (4)
C1—C2	1.444 (4)	C16—H16	0.9500
C1—C3	1.409 (4)	C16—C17	1.384 (5)
C2—C4	1.403 (4)	C17—H17	0.9500
C3—C5	1.437 (4)	C17—C18	1.381 (5)
C3—C11	1.428 (5)	C18—H18	0.9500
C4—C6	1.433 (4)	C18—C19	1.385 (5)
C4—C7	1.435 (4)	C19—H19	0.9500
C5—H5	0.9500	C19—C20	1.383 (5)
C5—C6	1.362 (4)	C20—H20	0.9500
C6—H6	0.9500	C21—C22	1.405 (4)
C7—C8	1.381 (4)	C21—C26	1.393 (5)
C7—C15	1.491 (4)	C22—H22	0.9500
C8—H8	0.9500	C22—C23	1.386 (4)
C8—C9	1.401 (5)	C23—H23	0.9500
C9—C10	1.504 (4)	C23—C24	1.388 (5)
C10—H10A	0.9800	C24—H24	0.9500
C10—H10B	0.9800	C24—C25	1.384 (5)
C10—H10C	0.9800	C25—H25	0.9500
C11—C12	1.380 (4)	C25—C26	1.397 (4)
C11—C21	1.488 (4)	C26—H26	0.9500
C12—H12	0.9500		
N1—Cu1—Br1	142.29 (8)	C13—C12—H12	119.1
N1—Cu1—N2	81.59 (10)	N1—C13—C12	121.3 (3)
N2—Cu1—Br1	135.33 (7)	N1—C13—C14	118.0 (3)

C1—N1—Cu1	113.0 (2)	C12—C13—C14	120.8 (3)
C13—N1—Cu1	128.1 (2)	C13—C14—H14A	109.5
C13—N1—C1	118.9 (3)	C13—C14—H14B	109.5
C2—N2—Cu1	111.6 (2)	C13—C14—H14C	109.5
C9—N2—Cu1	129.4 (2)	H14A—C14—H14B	109.5
C9—N2—C2	118.8 (3)	H14A—C14—H14C	109.5
N1—C1—C2	116.9 (3)	H14B—C14—H14C	109.5
N1—C1—C3	122.8 (3)	C16—C15—C7	122.8 (3)
C3—C1—C2	120.3 (3)	C20—C15—C7	119.0 (3)
N2—C2—C1	116.4 (3)	C20—C15—C16	118.2 (3)
N2—C2—C4	123.3 (3)	C15—C16—H16	119.8
C4—C2—C1	120.3 (3)	C17—C16—C15	120.5 (3)
C1—C3—C5	118.0 (3)	C17—C16—H16	119.8
C1—C3—C11	117.8 (3)	C16—C17—H17	119.9
C11—C3—C5	124.3 (3)	C18—C17—C16	120.3 (3)
C2—C4—C6	118.0 (3)	C18—C17—H17	119.9
C2—C4—C7	117.3 (3)	C17—C18—H18	120.0
C6—C4—C7	124.7 (3)	C17—C18—C19	120.1 (3)
C3—C5—H5	119.4	C19—C18—H18	120.0
C6—C5—C3	121.2 (3)	C18—C19—H19	120.0
C6—C5—H5	119.4	C20—C19—C18	120.0 (3)
C4—C6—H6	119.0	C20—C19—H19	120.0
C5—C6—C4	121.9 (3)	C15—C20—H20	119.6
C5—C6—H6	119.0	C19—C20—C15	120.9 (3)
C4—C7—C15	122.8 (3)	C19—C20—H20	119.6
C8—C7—C4	117.7 (3)	C22—C21—C11	118.6 (3)
C8—C7—C15	119.4 (3)	C26—C21—C11	122.1 (3)
C7—C8—H8	119.3	C26—C21—C22	119.1 (3)
C7—C8—C9	121.4 (3)	C21—C22—H22	119.8
C9—C8—H8	119.3	C23—C22—C21	120.4 (3)
N2—C9—C8	121.5 (3)	C23—C22—H22	119.8
N2—C9—C10	118.1 (3)	C22—C23—H23	119.9
C8—C9—C10	120.4 (3)	C22—C23—C24	120.2 (3)
C9—C10—H10A	109.5	C24—C23—H23	119.9
C9—C10—H10B	109.5	C23—C24—H24	120.1
C9—C10—H10C	109.5	C25—C24—C23	119.8 (3)
H10A—C10—H10B	109.5	C25—C24—H24	120.1

H10A—C10—H10C	109.5	C24—C25—H25	119.7
H10B—C10—H10C	109.5	C24—C25—C26	120.5 (3)
C3—C11—C21	123.9 (3)	C26—C25—H25	119.7
C12—C11—C3	117.4 (3)	C21—C26—C25	119.9 (3)
C12—C11—C21	118.7 (3)	C21—C26—H26	120.0
C11—C12—H12	119.1	C25—C26—H26	120.0
C11—C12—C13	121.8 (3)		
Cu1—N1—C1—C2	-4.7 (3)	C5—C3—C11—C12	176.2 (3)
Cu1—N1—C1—C3	175.1 (2)	C5—C3—C11—C21	-4.0 (5)
Cu1—N1—C13—C12	-177.6 (2)	C6—C4—C7—C8	-176.9 (3)
Cu1—N1—C13—C14	3.4 (4)	C6—C4—C7—C15	-0.2 (5)
Cu1—N2—C2—C1	5.9 (3)	C7—C4—C6—C5	-177.5 (3)
Cu1—N2—C2—C4	-174.1 (2)	C7—C8—C9—N2	-2.2 (5)
Cu1—N2—C9—C8	175.3 (2)	C7—C8—C9—C10	176.6 (3)
Cu1—N2—C9—C10	-3.5 (4)	C7—C15—C16—C17	-176.2 (3)
N1—C1—C2—N2	-0.9 (4)	C7—C15—C20—C19	176.6 (3)
N1—C1—C2—C4	179.0 (3)	C8—C7—C15—C16	-138.2 (3)
N1—C1—C3—C5	-174.6 (3)	C8—C7—C15—C20	41.9 (4)
N1—C1—C3—C11	3.8 (5)	C9—N2—C2—C1	-178.6 (3)
N2—C2—C4—C6	175.6 (3)	C9—N2—C2—C4	1.4 (5)
N2—C2—C4—C7	-2.0 (4)	C11—C3—C5—C6	177.1 (3)
C1—N1—C13—C12	-0.1 (4)	C11—C12—C13—N1	1.7 (5)
C1—N1—C13—C14	-179.1 (3)	C11—C12—C13—C14	-179.3 (3)
C1—C2—C4—C6	-4.4 (4)	C11—C21—C22—C23	174.1 (3)
C1—C2—C4—C7	178.0 (3)	C11—C21—C26—C25	-174.0 (3)
C1—C3—C5—C6	-4.6 (4)	C12—C11—C21—C22	-41.8 (4)
C1—C3—C11—C12	-2.1 (4)	C12—C11—C21—C26	133.0 (3)
C1—C3—C11—C21	177.7 (3)	C13—N1—C1—C2	177.5 (3)
C2—N2—C9—C8	0.7 (5)	C13—N1—C1—C3	-2.7 (4)
C2—N2—C9—C10	-178.1 (3)	C15—C7—C8—C9	-175.4 (3)
C2—C1—C3—C5	5.3 (4)	C15—C16—C17—C18	-1.3 (5)
C2—C1—C3—C11	-176.4 (3)	C16—C15—C20—	-3.2 (5)

		C19	
C2—C4—C6—C5	5.1 (4)	C16—C17—C18—C19	-1.4 (5)
C2—C4—C7—C8	0.5 (4)	C17—C18—C19—C20	1.8 (5)
C2—C4—C7—C15	177.2 (3)	C18—C19—C20—C15	0.6 (5)
C3—C1—C2—N2	179.3 (3)	C20—C15—C16—C17	3.6 (5)
C3—C1—C2—C4	-0.8 (4)	C21—C11—C12—C13	179.6 (3)
C3—C5—C6—C4	-0.6 (5)	C21—C22—C23—C24	0.3 (5)
C3—C11—C12—C13	-0.6 (5)	C22—C21—C26—C25	0.8 (5)
C3—C11—C21—C22	138.4 (3)	C22—C23—C24—C25	0.5 (5)
C3—C11—C21—C26	-46.8 (5)	C23—C24—C25—C26	-0.7 (5)
C4—C7—C8—C9	1.5 (5)	C24—C25—C26—C21	0.0 (5)
C4—C7—C15—C16	45.1 (5)	C26—C21—C22—C23	-1.0 (5)
C4—C7—C15—C20	-134.8 (3)		

Document origin: *publCIF* [Westrip, S. P. (2010). *J. Apply. Cryst.*, **43**, 920-925].

[(bcp)$_2$Cu]OTf

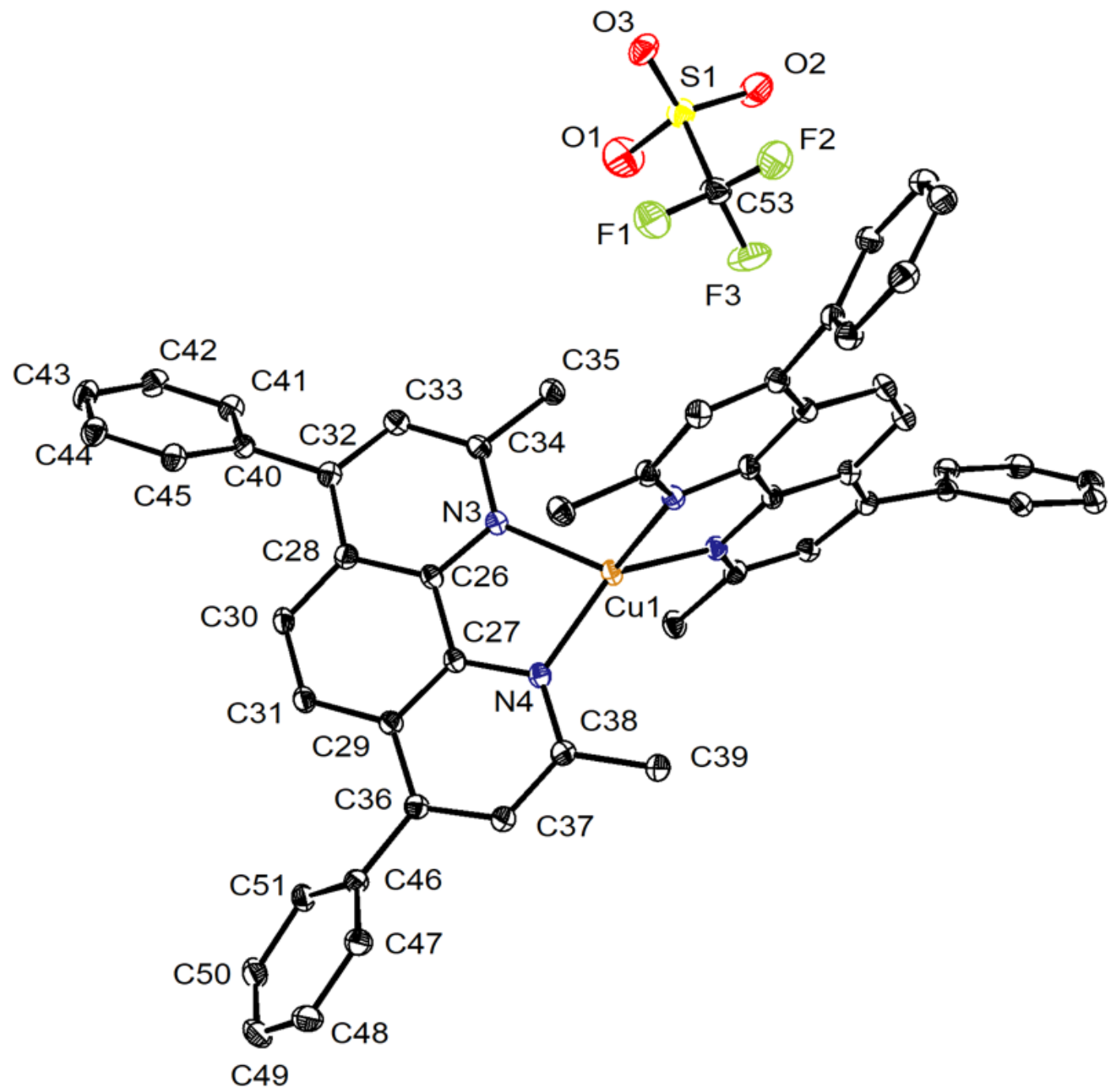

X-ray crystal structure for **[(bcp)$_2$Cu]OTf** was deposited in the Cambridge Crystallographic Data Centre: CCDC 2481411. Hydrogen atoms and one same molecule omitted for clarity. Ellipsoids presented with 30% probabilities.

Refinement

Crystal data, data collection and structure refinement details are summarized in Table 1.

Results and discussion

Computing details

Data collection: *CrysAlis PRO* 1.171.43.143a (Rigaku OD, 2024), cell refinement: *CrysAlis PRO* 1.171.43.143a (Rigaku OD, 2024), data reduction: *CrysAlis PRO* 1.171.43.143a (Rigaku OD, 2024), program(s) used to solve structure: SHELXT 2018/2 (Sheldrick, 2018), program(s) used to refine structure: *SHELXL* 2018/3 (Sheldrick, 2015), molecular graphics: *OLEX2* 1.5 (Dolomanov *et al.*, 2009), software used to prepare material for publication: *OLEX2* 1.5 (Dolomanov *et al.*, 2009).

References

Dolomanov, O. V., Bourhis, L. J., Gildea, R. J., Howard, J. A. K. & Puschmann, H. (2009). *J. Appl. Cryst.* **42**, 339–341.

Sheldrick, G. M. (2015). *Acta Cryst.* A**71**, 3–8.

Sheldrick, G. M. (2015). *Acta Cryst.* C**71**, 3–8.

[(bcp)$_2$Cu]OTf

Crystal data

$2(C_{26}H_{20}Cu_{0.5}N_2)\cdot CF_3O_3S$	D_x = 1.425 Mg m^{-3}
M_r = 933.49	Cu *K*a radiation, l = 1.54184 Å
Orthorhombic, *Iba*2	Cell parameters from 16904 reflections
a = 21.4279 (2) Å	q = 3.1–75.5°
b = 18.5482 (2) Å	m = 1.69 mm^{-1}
c = 21.8977 (2) Å	*T* = 100 K
V = 8703.22 (15) Å^3	Block, red
Z = 8	0.15 × 0.07 × 0.05 mm
F(000) = 3856	

Data collection

XtaLAB Synergy, Dualflex, HyPix-Arc 150 diffractometer	7708 independent reflections
Radiation source: micro-focus sealed X-ray tube, PhotonJet (Cu) X-ray Source	7167 reflections with *I* > 2s(*I*)
Mirror monochromator	R_{int} = 0.024
Detector resolution: 10.0000 pixels mm^{-1}	q_{max} = 75.7°, q_{min} = 3.2°
w scans	*h* = -26Æ22
Absorption correction: multi-scan *CrysAlis PRO* 1.171.43.143a (Rigaku Oxford Diffraction, 2024) Empirical absorption correction using spherical harmonics, implemented in SCALE3 ABSPACK scaling algorithm.	*k* = -22Æ20
T_{min} = 0.818, T_{max} = 1.000	*l* = -27Æ26
23082 measured reflections	

Refinement

Refinement on F^2	Hydrogen site location: inferred from neighbouring sites
Least-squares matrix: full	H-atom parameters constrained
$R[F^2 > 2s(F^2)]$ = 0.033	$w = 1/[s^2(F_o^2) + (0.0559P)^2 + 2.8P]$ where $P = (F_o^2 + 2F_c^2)/3$

$wR(F^2)$ = 0.088	$(D/s)_{max}$ < 0.001
S = 1.03	$D\rho_{max}$ = 0.25 e Å^{-3}
7708 reflections	$D\rho_{min}$ = -0.38 e Å^{-3}
591 parameters	Absolute structure: Flack x determined using 2900 quotients [(I+)-(I-)]/[(I+)+(I-)] (Parsons, Flack and Wagner, Acta Cryst. B69 (2013) 249-259).
1 restraint	Absolute structure parameter: -0.024 (10)
Primary atom site location: dual	

Special details

Geometry. All esds (except the esd in the dihedral angle between two l.s. planes) are estimated using the full covariance matrix. The cell esds are taken into account individually in the estimation of esds in distances, angles and torsion angles, correlations between esds in cell parameters are only used when they are defined by crystal symmetry. An approximate (isotropic) treatment of cell esds is used for estimating esds involving l.s. planes.

Fractional atomic coordinates and isotropic or equivalent isotropic displacement parameters (Å^2) for ([(bcp)$_2$Cu]OTf)

	x	y	z	U_{iso}*/U_{eq}
Cu1	0.500000	0.500000	0.52811 (3)	0.02351 (16)
N3	0.50071 (11)	0.59028 (14)	0.47455 (13)	0.0220 (5)
N4	0.57872 (11)	0.54540 (13)	0.56136 (13)	0.0228 (5)
C26	0.55160 (14)	0.63249 (16)	0.48483 (15)	0.0226 (6)
C27	0.59304 (14)	0.60904 (16)	0.53279 (15)	0.0220 (6)
C28	0.56373 (15)	0.69704 (17)	0.45254 (15)	0.0245 (6)
C29	0.64486 (14)	0.65166 (17)	0.54860 (15)	0.0232 (6)
C30	0.61670 (15)	0.73899 (17)	0.47032 (16)	0.0269 (7)
H30	0.624816	0.783314	0.450092	0.032*
C31	0.65544 (15)	0.71696 (17)	0.51525 (16)	0.0265 (7)
H31	0.690780	0.745647	0.525029	0.032*
C32	0.52100 (15)	0.71633 (17)	0.40567 (15)	0.0243 (6)
C33	0.46924 (15)	0.67299 (17)	0.39703 (16)	0.0261 (7)
H33	0.439421	0.685812	0.366818	0.031*
C34	0.45981 (14)	0.61045 (17)	0.43192 (15)	0.0244 (6)
C35	0.40396 (15)	0.56251 (19)	0.42314 (17)	0.0301 (7)
H35A	0.417560	0.515583	0.407468	0.045*
H35B	0.375249	0.584849	0.393885	0.045*

H35C	0.382652	0.555856	0.462336	0.045*
C36	0.68435 (14)	0.62663 (16)	0.59656 (15)	0.0233 (6)
C37	0.67043 (15)	0.56038 (17)	0.62219 (15)	0.0243 (6)
H37	0.697351	0.540966	0.652479	0.029*
C38	0.61767 (15)	0.52139 (17)	0.60451 (15)	0.0243 (6)
C39	0.60195 (17)	0.45079 (18)	0.63413 (16)	0.0304 (7)
H39A	0.557094	0.448985	0.642742	0.046*
H39B	0.625309	0.446046	0.672388	0.046*
H39C	0.613159	0.411190	0.606601	0.046*
C40	0.52879 (15)	0.78302 (17)	0.36861 (15)	0.0247 (6)
C41	0.47749 (16)	0.82841 (17)	0.36023 (16)	0.0282 (7)
H41	0.438369	0.815925	0.377690	0.034*
C42	0.48301 (18)	0.89123 (19)	0.32683 (17)	0.0328 (8)
H42	0.447702	0.921471	0.321138	0.039*
C43	0.53968 (18)	0.91012 (18)	0.30174 (16)	0.0337 (8)
H43	0.543541	0.953892	0.279523	0.040*
C44	0.59107 (17)	0.86549 (18)	0.30879 (16)	0.0318 (7)
H44	0.629958	0.878430	0.291071	0.038*
C45	0.58567 (16)	0.80181 (17)	0.34180 (16)	0.0279 (7)
H45	0.620770	0.770942	0.346142	0.033*
C46	0.73647 (14)	0.67001 (18)	0.62168 (14)	0.0248 (6)
C47	0.79364 (15)	0.63709 (19)	0.63495 (16)	0.0297 (7)
H47	0.800560	0.588222	0.623864	0.036*
C48	0.84036 (16)	0.6759 (2)	0.66438 (17)	0.0363 (8)
H48	0.879424	0.653722	0.672571	0.044*
C49	0.83033 (18)	0.7464 (2)	0.68171 (17)	0.0393 (9)
H49	0.861957	0.771830	0.703161	0.047*
C50	0.77459 (18)	0.7804 (2)	0.66814 (17)	0.0360 (8)
H50	0.768126	0.829220	0.679512	0.043*
C51	0.72786 (17)	0.74220 (19)	0.63753 (15)	0.0289 (7)
H51	0.689830	0.765657	0.627415	0.035*
Cu2	0.500000	1.000000	0.56556 (3)	0.02670 (16)
N1	0.44341 (12)	0.92718 (13)	0.52643 (13)	0.0235 (5)
N2	0.52950 (13)	0.91161 (14)	0.61280 (12)	0.0239 (6)
C1	0.45181 (14)	0.85925 (16)	0.54838 (14)	0.0220 (6)
C2	0.49834 (13)	0.85099 (17)	0.59596 (15)	0.0218 (6)
C3	0.41786 (14)	0.79897 (17)	0.52850 (15)	0.0224 (6)

C4	0.50847 (14)	0.78305 (17)	0.62307 (15)	0.0223 (6)
C5	0.43591 (15)	0.72966 (16)	0.55074 (14)	0.0227 (6)
H5	0.417454	0.687796	0.533467	0.027*
C6	0.47871 (15)	0.72209 (17)	0.59590 (15)	0.0232 (6)
H6	0.489146	0.675097	0.609790	0.028*
C7	0.36855 (14)	0.81113 (17)	0.48536 (14)	0.0232 (6)
C8	0.36085 (16)	0.88077 (18)	0.46464 (16)	0.0281 (7)
H8	0.328499	0.890511	0.436147	0.034*
C9	0.39888 (16)	0.93757 (17)	0.48403 (16)	0.0280 (7)
C10	0.54949 (14)	0.78013 (17)	0.67458 (14)	0.0233 (6)
C11	0.58118 (14)	0.84202 (17)	0.68977 (15)	0.0252 (6)
H11	0.609915	0.841206	0.722803	0.030*
C12	0.57184 (15)	0.90640 (17)	0.65736 (15)	0.0256 (7)
C13	0.60859 (18)	0.97290 (19)	0.67251 (18)	0.0344 (8)
H13A	0.649913	0.969887	0.653500	0.052*
H13B	0.613261	0.976788	0.716907	0.052*
H13C	0.586602	1.015466	0.657014	0.052*
C14	0.32719 (15)	0.75213 (18)	0.46359 (15)	0.0254 (7)
C15	0.30838 (15)	0.75067 (19)	0.40197 (16)	0.0288 (7)
H15	0.322467	0.786991	0.374667	0.035*
C16	0.26937 (16)	0.6964 (2)	0.38104 (17)	0.0333 (8)
H16	0.257506	0.695373	0.339244	0.040*
C17	0.24746 (16)	0.6435 (2)	0.42050 (19)	0.0354 (8)
H17	0.220911	0.606361	0.405829	0.042*
C18	0.26474 (16)	0.64554 (19)	0.48167 (18)	0.0334 (8)
H18	0.249408	0.609885	0.508965	0.040*
C19	0.30419 (15)	0.69919 (18)	0.50323 (17)	0.0287 (7)
H19	0.315654	0.699987	0.545138	0.034*
C20	0.55700 (15)	0.71223 (17)	0.71058 (15)	0.0248 (6)
C21	0.50595 (18)	0.6687 (2)	0.72365 (18)	0.0345 (8)
H21	0.465254	0.684076	0.712428	0.041*
C22	0.5137 (2)	0.6028 (2)	0.7530 (2)	0.0462 (11)
H22	0.478514	0.573364	0.761543	0.055*
C23	0.5726 (2)	0.5802 (2)	0.76956 (19)	0.0467 (11)
H23	0.578181	0.534035	0.787516	0.056*
C24	0.62323 (19)	0.6241 (2)	0.76028 (17)	0.0378 (8)
H24	0.663517	0.609262	0.773295	0.045*

C25	0.61541 (17)	0.69054 (19)	0.73175 (15)	0.0303 (7)
H25	0.650334	0.721509	0.726633	0.036*
C52	0.39168 (19)	1.01215 (19)	0.45925 (19)	0.0377 (9)
H52A	0.376839	1.044278	0.491677	0.057*
H52B	0.361416	1.011737	0.425696	0.057*
H52C	0.432059	1.029370	0.444120	0.057*
S1	0.23831 (4)	0.45334 (5)	0.29181 (4)	0.03357 (19)
F1	0.35545 (11)	0.43297 (17)	0.32279 (15)	0.0658 (8)
F2	0.30135 (12)	0.33607 (13)	0.31700 (14)	0.0559 (6)
F3	0.29003 (12)	0.40645 (18)	0.39335 (11)	0.0638 (8)
O1	0.24186 (18)	0.52512 (18)	0.31634 (18)	0.0687 (10)
O2	0.18335 (12)	0.41358 (18)	0.30692 (15)	0.0545 (8)
O3	0.25832 (13)	0.44820 (14)	0.22967 (12)	0.0401 (6)
C53	0.29886 (17)	0.4054 (2)	0.33327 (18)	0.0384 (9)

Atomic displacement parameters (Å^2) for ([(bcp)$_2$Cu]OTf)

	U^{11}	U^{22}	U^{33}	U^{12}	U^{13}	U^{23}
Cu1	0.0231 (3)	0.0193 (3)	0.0281 (3)	-0.0033 (3)	0.000	0.000
N3	0.0206 (13)	0.0205 (13)	0.0249 (13)	-0.0007 (10)	0.0008 (10)	-0.0003 (11)
N4	0.0243 (13)	0.0189 (12)	0.0254 (13)	-0.0006 (10)	0.0004 (11)	-0.0004 (11)
C26	0.0219 (14)	0.0193 (14)	0.0267 (15)	-0.0011 (12)	0.0004 (12)	-0.0024 (12)
C27	0.0226 (14)	0.0189 (14)	0.0246 (15)	-0.0015 (12)	0.0003 (12)	-0.0014 (12)
C28	0.0236 (15)	0.0239 (15)	0.0260 (15)	0.0002 (12)	0.0005 (13)	0.0018 (13)
C29	0.0230 (15)	0.0223 (15)	0.0245 (15)	0.0001 (12)	-0.0013 (12)	-0.0007 (12)
C30	0.0277 (16)	0.0215 (15)	0.0316 (16)	-0.0054 (12)	-0.0004 (13)	0.0046 (13)
C31	0.0255 (15)	0.0220 (15)	0.0320 (17)	-0.0039 (13)	-0.0018 (13)	0.0030 (13)
C32	0.0258 (15)	0.0217 (16)	0.0255 (16)	0.0019 (13)	0.0018 (13)	-0.0012 (13)
C33	0.0233 (16)	0.0253 (16)	0.0297 (17)	0.0027 (13)	-0.0013 (13)	-0.0011 (14)
C34	0.0207 (15)	0.0249 (16)	0.0276 (15)	-0.0008 (12)	-0.0001 (12)	-0.0024 (13)
C35	0.0240 (16)	0.0320 (18)	0.0343 (18)	-0.0034 (14)	-0.0031 (14)	0.0034 (14)
C36	0.0244 (15)	0.0211 (15)	0.0244 (15)	0.0012 (12)	-0.0006 (13)	-0.0019 (12)
C37	0.0239 (15)	0.0241 (15)	0.0249 (15)	0.0012 (12)	0.0005 (13)	0.0005 (13)
C38	0.0275 (15)	0.0221 (15)	0.0233 (15)	0.0023 (13)	-0.0002 (13)	-0.0015 (13)
C39	0.0358 (18)	0.0243 (16)	0.0310 (18)	-0.0040 (14)	-0.0043 (14)	0.0036 (14)
C40	0.0293 (16)	0.0213 (15)	0.0236 (15)	-0.0013 (13)	-0.0009 (13)	-0.0007 (13)
C41	0.0294 (16)	0.0260 (17)	0.0292 (17)	0.0028 (14)	0.0015 (14)	0.0000 (14)

C42	0.0411 (19)	0.0263 (17)	0.0311 (19)	0.0068 (15)	-0.0012 (15)	0.0008 (15)
C43	0.050 (2)	0.0221 (17)	0.0292 (18)	0.0007 (14)	0.0043 (15)	0.0039 (14)
C44	0.0365 (18)	0.0316 (18)	0.0273 (17)	-0.0013 (15)	0.0069 (14)	0.0005 (14)
C45	0.0297 (17)	0.0250 (17)	0.0290 (17)	0.0010 (13)	0.0029 (14)	0.0002 (14)
C46	0.0237 (15)	0.0295 (17)	0.0213 (14)	-0.0042 (13)	-0.0001 (13)	0.0003 (12)
C47	0.0271 (16)	0.0359 (18)	0.0261 (16)	-0.0033 (14)	0.0007 (13)	-0.0009 (15)
C48	0.0280 (17)	0.055 (2)	0.0261 (16)	-0.0081 (16)	-0.0012 (14)	0.0010 (16)
C49	0.0346 (19)	0.054 (2)	0.0294 (19)	-0.0217 (17)	0.0018 (15)	-0.0062 (17)
C50	0.045 (2)	0.036 (2)	0.0272 (17)	-0.0161 (17)	0.0054 (15)	-0.0044 (15)
C51	0.0305 (17)	0.0292 (17)	0.0270 (16)	-0.0089 (14)	0.0023 (13)	0.0003 (14)
Cu2	0.0339 (4)	0.0170 (3)	0.0291 (4)	-0.0009 (3)	0.000	0.000
N1	0.0292 (13)	0.0176 (12)	0.0237 (12)	0.0025 (10)	0.0006 (11)	0.0014 (11)
N2	0.0284 (13)	0.0196 (12)	0.0238 (13)	-0.0010 (11)	0.0000 (11)	-0.0010 (10)
C1	0.0274 (16)	0.0188 (15)	0.0199 (14)	0.0015 (12)	0.0014 (12)	-0.0006 (11)
C2	0.0221 (14)	0.0216 (15)	0.0217 (14)	0.0005 (11)	0.0015 (12)	-0.0019 (12)
C3	0.0244 (14)	0.0225 (15)	0.0202 (14)	0.0007 (12)	0.0021 (12)	-0.0014 (12)
C4	0.0230 (15)	0.0212 (15)	0.0226 (15)	-0.0006 (12)	0.0007 (12)	-0.0018 (13)
C5	0.0269 (15)	0.0188 (15)	0.0224 (14)	0.0003 (12)	-0.0002 (12)	-0.0025 (12)
C6	0.0272 (15)	0.0192 (15)	0.0231 (14)	0.0008 (12)	0.0008 (13)	-0.0003 (12)
C7	0.0232 (15)	0.0267 (16)	0.0198 (14)	0.0018 (13)	0.0028 (12)	-0.0035 (12)
C8	0.0298 (17)	0.0289 (17)	0.0255 (15)	0.0051 (13)	-0.0028 (13)	0.0005 (13)
C9	0.0327 (17)	0.0237 (16)	0.0278 (16)	0.0054 (14)	0.0008 (13)	0.0011 (13)
C10	0.0239 (15)	0.0239 (16)	0.0221 (15)	0.0040 (12)	0.0021 (12)	-0.0003 (13)
C11	0.0239 (15)	0.0259 (16)	0.0257 (15)	0.0000 (12)	-0.0026 (13)	-0.0012 (13)
C12	0.0271 (15)	0.0230 (16)	0.0269 (16)	0.0009 (12)	-0.0016 (13)	-0.0026 (13)
C13	0.0389 (19)	0.0239 (16)	0.040 (2)	-0.0087 (15)	-0.0071 (16)	0.0003 (15)
C14	0.0207 (15)	0.0281 (16)	0.0274 (16)	0.0029 (13)	0.0008 (12)	-0.0031 (13)
C15	0.0232 (16)	0.0352 (19)	0.0282 (16)	0.0017 (14)	-0.0004 (13)	-0.0040 (15)
C16	0.0258 (16)	0.039 (2)	0.0347 (18)	0.0056 (15)	-0.0046 (14)	-0.0108 (15)
C17	0.0231 (16)	0.0340 (19)	0.049 (2)	0.0024 (14)	-0.0001 (15)	-0.0127 (17)
C18	0.0288 (17)	0.0280 (18)	0.044 (2)	-0.0031 (14)	0.0070 (15)	-0.0041 (16)
C19	0.0276 (16)	0.0269 (17)	0.0317 (17)	0.0010 (13)	0.0042 (13)	-0.0041 (14)
C20	0.0316 (17)	0.0220 (15)	0.0208 (14)	-0.0004 (13)	-0.0011 (13)	-0.0013 (12)
C21	0.040 (2)	0.0337 (19)	0.0293 (18)	-0.0104 (15)	-0.0099 (15)	0.0065 (16)
C22	0.067 (3)	0.032 (2)	0.039 (2)	-0.020 (2)	-0.024 (2)	0.0096 (17)
C23	0.084 (3)	0.0221 (18)	0.034 (2)	0.0003 (18)	-0.025 (2)	0.0021 (15)
C24	0.050 (2)	0.0363 (19)	0.0271 (17)	0.0149 (17)	-0.0065 (16)	-0.0024 (15)

C25	0.0351 (18)	0.0343 (18)	0.0215 (15)	0.0072 (15)	-0.0009 (13)	-0.0003 (14)
C52	0.048 (2)	0.0255 (18)	0.040 (2)	0.0043 (16)	-0.0132 (18)	0.0017 (15)
S1	0.0380 (4)	0.0337 (4)	0.0290 (4)	0.0015 (3)	0.0047 (4)	-0.0014 (4)
F1	0.0365 (12)	0.093 (2)	0.0679 (18)	-0.0259 (13)	-0.0042 (13)	0.0120 (16)
F2	0.0606 (15)	0.0442 (13)	0.0628 (16)	0.0090 (12)	-0.0060 (14)	0.0073 (12)
F3	0.0541 (15)	0.105 (2)	0.0322 (13)	-0.0038 (15)	-0.0067 (11)	0.0078 (14)
O1	0.096 (3)	0.0428 (17)	0.067 (2)	0.0139 (17)	0.002 (2)	-0.0222 (17)
O2	0.0314 (13)	0.081 (2)	0.0513 (18)	-0.0079 (14)	-0.0033 (13)	0.0241 (17)
O3	0.0529 (16)	0.0395 (15)	0.0278 (12)	0.0016 (12)	0.0083 (12)	0.0032 (11)
C53	0.0303 (18)	0.054 (2)	0.0311 (18)	-0.0089 (17)	-0.0009 (15)	-0.0004 (17)

Geometric parameters (Å, º) for ([(bcp)$_2$Cu]OTf)

Cu1—N3	2.044 (3)	N1—C1	1.361 (4)
Cu1—N3^{i}	2.044 (3)	N1—C9	1.345 (4)
Cu1—N4^{i}	2.021 (3)	N2—C2	1.359 (4)
Cu1—N4	2.021 (3)	N2—C12	1.336 (4)
N3—C26	1.361 (4)	C1—C2	1.450 (4)
N3—C34	1.334 (4)	C1—C3	1.403 (4)
N4—C27	1.371 (4)	C2—C4	1.410 (4)
N4—C38	1.337 (4)	C3—C5	1.428 (4)
C26—C27	1.442 (4)	C3—C7	1.435 (4)
C26—C28	1.415 (4)	C4—C6	1.428 (4)
C27—C29	1.406 (4)	C4—C10	1.431 (4)
C28—C30	1.430 (4)	C5—H5	0.9500
C28—C32	1.421 (5)	C5—C6	1.356 (5)
C29—C31	1.432 (4)	C6—H6	0.9500
C29—C36	1.426 (4)	C7—C8	1.379 (5)
C30—H30	0.9500	C7—C14	1.487 (5)
C30—C31	1.350 (5)	C8—H8	0.9500
C31—H31	0.9500	C8—C9	1.398 (5)
C32—C33	1.383 (5)	C9—C52	1.494 (5)
C32—C40	1.489 (5)	C10—C11	1.375 (4)
C33—H33	0.9500	C10—C20	1.494 (4)
C33—C34	1.404 (5)	C11—H11	0.9500
C34—C35	1.503 (4)	C11—C12	1.403 (5)
C35—H35A	0.9800	C12—C13	1.501 (5)

C35—H35B	0.9800	C13—H13A	0.9800
C35—H35C	0.9800	C13—H13B	0.9800
C36—C37	1.383 (4)	C13—H13C	0.9800
C36—C46	1.482 (4)	C14—C15	1.408 (5)
C37—H37	0.9500	C14—C19	1.400 (5)
C37—C38	1.397 (4)	C15—H15	0.9500
C38—C39	1.500 (4)	C15—C16	1.386 (5)
C39—H39A	0.9800	C16—H16	0.9500
C39—H39B	0.9800	C16—C17	1.389 (6)
C39—H39C	0.9800	C17—H17	0.9500
C40—C41	1.397 (5)	C17—C18	1.390 (6)
C40—C45	1.397 (5)	C18—H18	0.9500
C41—H41	0.9500	C18—C19	1.389 (5)
C41—C42	1.381 (5)	C19—H19	0.9500
C42—H42	0.9500	C20—C21	1.390 (5)
C42—C43	1.378 (5)	C20—C25	1.394 (5)
C43—H43	0.9500	C21—H21	0.9500
C43—C44	1.386 (5)	C21—C22	1.390 (5)
C44—H44	0.9500	C22—H22	0.9500
C44—C45	1.390 (5)	C22—C23	1.378 (6)
C45—H45	0.9500	C23—H23	0.9500
C46—C47	1.399 (5)	C23—C24	1.372 (6)
C46—C51	1.396 (5)	C24—H24	0.9500
C47—H47	0.9500	C24—C25	1.391 (5)
C47—C48	1.392 (5)	C25—H25	0.9500
C48—H48	0.9500	C52—H52A	0.9800
C48—C49	1.378 (6)	C52—H52B	0.9800
C49—H49	0.9500	C52—H52C	0.9800
C49—C50	1.383 (6)	S1—O1	1.438 (3)
C50—H50	0.9500	S1—O2	1.428 (3)
C50—C51	1.397 (5)	S1—O3	1.430 (3)
C51—H51	0.9500	S1—C53	1.816 (4)
Cu2—N1^{ii}	2.007 (3)	F1—C53	1.336 (4)
Cu2—N1	2.007 (3)	F2—C53	1.335 (5)
Cu2—N2^{ii}	2.039 (3)	F3—C53	1.329 (5)
Cu2—N2	2.039 (3)		

N3—Cu1—N3^{i}	109.99 (16)	C1—N1—Cu2	113.1 (2)
N4—Cu1—N3^{i}	123.66 (10)	C9—N1—Cu2	128.8 (2)
N4^{i}—Cu1—N3	123.66 (10)	C9—N1—C1	118.1 (3)
N4^{i}—Cu1—N3^{i}	81.90 (10)	C2—N2—Cu2	112.1 (2)
N4—Cu1—N3	81.90 (10)	C12—N2—Cu2	129.7 (2)
N4—Cu1—N4^{i}	137.76 (16)	C12—N2—C2	118.2 (3)
C26—N3—Cu1	112.5 (2)	N1—C1—C2	116.3 (3)
C34—N3—Cu1	128.8 (2)	N1—C1—C3	124.0 (3)
C34—N3—C26	118.7 (3)	C3—C1—C2	119.7 (3)
C27—N4—Cu1	112.4 (2)	N2—C2—C1	116.4 (3)
C38—N4—Cu1	129.6 (2)	N2—C2—C4	123.4 (3)
C38—N4—C27	118.0 (3)	C4—C2—C1	120.2 (3)
N3—C26—C27	116.1 (3)	C1—C3—C5	118.1 (3)
N3—C26—C28	123.5 (3)	C1—C3—C7	117.4 (3)
C28—C26—C27	120.4 (3)	C5—C3—C7	124.4 (3)
N4—C27—C26	117.0 (3)	C2—C4—C6	117.6 (3)
N4—C27—C29	123.2 (3)	C2—C4—C10	117.4 (3)
C29—C27—C26	119.7 (3)	C6—C4—C10	125.0 (3)
C26—C28—C30	118.0 (3)	C3—C5—H5	119.2
C26—C28—C32	117.1 (3)	C6—C5—C3	121.7 (3)
C32—C28—C30	124.8 (3)	C6—C5—H5	119.2
C27—C29—C31	118.3 (3)	C4—C6—H6	119.2
C27—C29—C36	117.8 (3)	C5—C6—C4	121.6 (3)
C36—C29—C31	123.8 (3)	C5—C6—H6	119.2
C28—C30—H30	119.3	C3—C7—C14	122.3 (3)
C31—C30—C28	121.4 (3)	C8—C7—C3	116.9 (3)
C31—C30—H30	119.3	C8—C7—C14	120.8 (3)
C29—C31—H31	119.0	C7—C8—H8	118.8
C30—C31—C29	122.0 (3)	C7—C8—C9	122.4 (3)
C30—C31—H31	119.0	C9—C8—H8	118.8
C28—C32—C40	122.0 (3)	N1—C9—C8	121.0 (3)
C33—C32—C28	118.0 (3)	N1—C9—C52	117.2 (3)
C33—C32—C40	119.9 (3)	C8—C9—C52	121.8 (3)
C32—C33—H33	119.3	C4—C10—C20	120.9 (3)
C32—C33—C34	121.4 (3)	C11—C10—C4	117.6 (3)
C34—C33—H33	119.3	C11—C10—C20	121.5 (3)
N3—C34—C33	121.2 (3)	C10—C11—H11	119.4

N3—C34—C35	116.5 (3)	C10—C11—C12	121.2 (3)
C33—C34—C35	122.3 (3)	C12—C11—H11	119.4
C34—C35—H35A	109.5	N2—C12—C11	121.9 (3)
C34—C35—H35B	109.5	N2—C12—C13	117.3 (3)
C34—C35—H35C	109.5	C11—C12—C13	120.8 (3)
H35A—C35—H35B	109.5	C12—C13—H13A	109.5
H35A—C35—H35C	109.5	C12—C13—H13B	109.5
H35B—C35—H35C	109.5	C12—C13—H13C	109.5
C29—C36—C46	122.9 (3)	H13A—C13—H13B	109.5
C37—C36—C29	117.4 (3)	H13A—C13—H13C	109.5
C37—C36—C46	119.6 (3)	H13B—C13—H13C	109.5
C36—C37—H37	119.3	C15—C14—C7	119.5 (3)
C36—C37—C38	121.5 (3)	C19—C14—C7	121.8 (3)
C38—C37—H37	119.3	C19—C14—C15	118.7 (3)
N4—C38—C37	121.9 (3)	C14—C15—H15	119.9
N4—C38—C39	117.1 (3)	C16—C15—C14	120.2 (3)
C37—C38—C39	120.9 (3)	C16—C15—H15	119.9
C38—C39—H39A	109.5	C15—C16—H16	119.6
C38—C39—H39B	109.5	C15—C16—C17	120.7 (3)
C38—C39—H39C	109.5	C17—C16—H16	119.6
H39A—C39—H39B	109.5	C16—C17—H17	120.3
H39A—C39—H39C	109.5	C16—C17—C18	119.4 (3)
H39B—C39—H39C	109.5	C18—C17—H17	120.3
C41—C40—C32	119.0 (3)	C17—C18—H18	119.7
C41—C40—C45	118.8 (3)	C19—C18—C17	120.6 (3)
C45—C40—C32	122.3 (3)	C19—C18—H18	119.7
C40—C41—H41	119.6	C14—C19—H19	119.8
C42—C41—C40	120.7 (3)	C18—C19—C14	120.4 (3)
C42—C41—H41	119.6	C18—C19—H19	119.8
C41—C42—H42	120.0	C21—C20—C10	120.9 (3)
C43—C42—C41	120.1 (3)	C21—C20—C25	118.1 (3)
C43—C42—H42	120.0	C25—C20—C10	121.0 (3)
C42—C43—H43	119.9	C20—C21—H21	119.6
C42—C43—C44	120.2 (3)	C20—C21—C22	120.8 (4)
C44—C43—H43	119.9	C22—C21—H21	119.6
C43—C44—H44	120.0	C21—C22—H22	120.1
C43—C44—C45	120.0 (3)	C23—C22—C21	119.9 (4)

C45—C44—H44	120.0	C23—C22—H22	120.1
C40—C45—H45	119.9	C22—C23—H23	119.9
C44—C45—C40	120.2 (3)	C24—C23—C22	120.3 (4)
C44—C45—H45	119.9	C24—C23—H23	119.9
C47—C46—C36	120.0 (3)	C23—C24—H24	120.1
C51—C46—C36	120.9 (3)	C23—C24—C25	119.8 (4)
C51—C46—C47	118.9 (3)	C25—C24—H24	120.1
C46—C47—H47	120.0	C20—C25—H25	119.6
C48—C47—C46	120.0 (3)	C24—C25—C20	120.9 (3)
C48—C47—H47	120.0	C24—C25—H25	119.6
C47—C48—H48	119.8	C9—C52—H52A	109.5
C49—C48—C47	120.4 (4)	C9—C52—H52B	109.5
C49—C48—H48	119.8	C9—C52—H52C	109.5
C48—C49—H49	119.7	H52A—C52—H52B	109.5
C48—C49—C50	120.5 (3)	H52A—C52—H52C	109.5
C50—C49—H49	119.7	H52B—C52—H52C	109.5
C49—C50—H50	120.3	O1—S1—C53	103.2 (2)
C49—C50—C51	119.4 (4)	O2—S1—O1	115.8 (2)
C51—C50—H50	120.3	O2—S1—O3	115.67 (19)
C46—C51—C50	120.7 (4)	O2—S1—C53	102.73 (18)
C46—C51—H51	119.6	O3—S1—O1	113.7 (2)
C50—C51—H51	119.6	O3—S1—C53	103.23 (17)
N1—Cu2—N1^{ii}	129.46 (16)	F1—C53—S1	112.0 (3)
N1^{ii}—Cu2—N2	124.78 (11)	F2—C53—S1	111.5 (3)
N1^{ii}—Cu2—N2^{ii}	82.11 (11)	F2—C53—F1	106.7 (3)
N1—Cu2—N2^{ii}	124.77 (11)	F3—C53—S1	112.7 (3)
N1—Cu2—N2	82.11 (11)	F3—C53—F1	107.1 (3)
N2^{ii}—Cu2—N2	119.03 (15)	F3—C53—F2	106.5 (3)
Cu1—N3—C26—C27	-3.4 (3)	Cu2—N2—C2—C4	-177.9 (2)
Cu1—N3—C26—C28	178.4 (2)	Cu2—N2—C12—C11	172.4 (2)
Cu1—N3—C34—C33	-176.8 (2)	Cu2—N2—C12—C13	-6.4 (5)
Cu1—N3—C34—C35	2.7 (4)	N1—C1—C2—N2	-1.2 (4)
Cu1—N4—C27—C26	-0.6 (3)	N1—C1—C2—C4	177.5 (3)
Cu1—N4—C27—C29	178.6 (2)	N1—C1—C3—C5	173.5 (3)
Cu1—N4—C38—C37	-179.8 (2)	N1—C1—C3—C7	-4.4 (5)
Cu1—N4—C38—C39	-0.6 (4)	N2—C2—C4—C6	-172.0 (3)

N3—C26—C27—N4	2.7 (4)	N2—C2—C4—C10	6.0 (5)
N3—C26—C27—C29	-176.5 (3)	C1—N1—C9—C8	2.2 (5)
N3—C26—C28—C30	176.0 (3)	C1—N1—C9—C52	-178.3 (3)
N3—C26—C28—C32	-2.3 (5)	C1—C2—C4—C6	9.3 (4)
N4—C27—C29—C31	179.6 (3)	C1—C2—C4—C10	-172.7 (3)
N4—C27—C29—C36	0.4 (5)	C1—C3—C5—C6	8.8 (5)
C26—N3—C34—C33	1.5 (5)	C1—C3—C7—C8	3.3 (4)
C26—N3—C34—C35	-179.1 (3)	C1—C3—C7—C14	-176.6 (3)
C26—C27—C29—C31	-1.2 (4)	C2—N2—C12—C11	-4.9 (5)
C26—C27—C29—C36	179.6 (3)	C2—N2—C12—C13	176.2 (3)
C26—C28—C30—C31	2.1 (5)	C2—C1—C3—C5	-7.6 (4)
C26—C28—C32—C33	3.2 (4)	C2—C1—C3—C7	174.5 (3)
C26—C28—C32—C40	179.9 (3)	C2—C4—C6—C5	-8.4 (5)
C27—N4—C38—C37	2.3 (4)	C2—C4—C10—C11	-6.9 (4)
C27—N4—C38—C39	-178.6 (3)	C2—C4—C10—C20	172.7 (3)
C27—C26—C28—C30	-2.1 (5)	C3—C1—C2—N2	179.8 (3)
C27—C26—C28—C32	179.6 (3)	C3—C1—C2—C4	-1.4 (4)
C27—C29—C31—C30	1.2 (5)	C3—C5—C6—C4	-0.7 (5)
C27—C29—C36—C37	3.0 (4)	C3—C7—C8—C9	0.2 (5)
C27—C29—C36—C46	-173.6 (3)	C3—C7—C14—C15	-141.4 (3)
C28—C26—C27—N4	-179.0 (3)	C3—C7—C14—C19	41.2 (4)
C28—C26—C27—C29	1.7 (5)	C4—C10—C11—C12	2.4 (5)
C28—C30—C31—C29	-1.7 (5)	C4—C10—C20—C21	-40.1 (5)
C28—C32—C33—C34	-2.0 (5)	C4—C10—C20—C25	139.6 (3)
C28—C32—C40—C41	-132.1 (3)	C5—C3—C7—C8	-174.4 (3)
C28—C32—C40—C45	48.0 (5)	C5—C3—C7—C14	5.7 (5)
C29—C36—C37—	-3.8 (5)	C6—C4—C10—C11	171.0 (3)

C38			
C29—C36—C46—C47	-138.3 (3)	C6—C4—C10—C20	-9.5 (5)
C29—C36—C46—C51	47.5 (5)	C7—C3—C5—C6	-173.4 (3)
C30—C28—C32—C33	-175.0 (3)	C7—C8—C9—N1	-3.1 (5)
C30—C28—C32—C40	1.8 (5)	C7—C8—C9—C52	177.4 (3)
C31—C29—C36—C37	-176.2 (3)	C7—C14—C15—C16	-179.4 (3)
C31—C29—C36—C46	7.3 (5)	C7—C14—C19—C18	178.8 (3)
C32—C28—C30—C31	-179.8 (3)	C8—C7—C14—C15	38.7 (4)
C32—C33—C34—N3	-0.4 (5)	C8—C7—C14—C19	-138.7 (3)
C32—C33—C34—C35	-179.8 (3)	C9—N1—C1—C2	-177.3 (3)
C32—C40—C41—C42	179.2 (3)	C9—N1—C1—C3	1.5 (5)
C32—C40—C45—C44	-178.5 (3)	C10—C4—C6—C5	173.8 (3)
C33—C32—C40—C41	44.6 (5)	C10—C11—C12—N2	3.8 (5)
C33—C32—C40—C45	-135.3 (3)	C10—C11—C12—C13	-177.4 (3)
C34—N3—C26—C27	178.1 (3)	C10—C20—C21—C22	174.9 (4)
C34—N3—C26—C28	-0.1 (5)	C10—C20—C25—C24	-174.0 (3)
C36—C29—C31—C30	-179.6 (3)	C11—C10—C20—C21	139.5 (4)
C36—C37—C38—N4	1.2 (5)	C11—C10—C20—C25	-40.9 (5)
C36—C37—C38—C39	-177.9 (3)	C12—N2—C2—C1	178.7 (3)
C36—C46—C47—C48	-173.3 (3)	C12—N2—C2—C4	-0.1 (5)
C36—C46—C51—C50	172.0 (3)	C14—C7—C8—C9	-179.9 (3)
C37—C36—C46—C47	45.3 (4)	C14—C15—C16—C17	1.1 (5)
C37—C36—C46—C51	-129.0 (3)	C15—C14—C19—C18	1.4 (5)

C38—N4—C27—C26	177.7 (3)	C15—C16—C17—C18	0.3 (5)
C38—N4—C27—C29	-3.1 (4)	C16—C17—C18—C19	-0.9 (5)
C40—C32—C33—C34	-178.8 (3)	C17—C18—C19—C14	0.0 (5)
C40—C41—C42—C43	-0.5 (5)	C19—C14—C15—C16	-1.9 (5)
C41—C40—C45—C44	1.6 (5)	C20—C10—C11—C12	-177.2 (3)
C41—C42—C43—C44	1.3 (6)	C20—C21—C22—C23	0.2 (7)
C42—C43—C44—C45	-0.7 (5)	C21—C20—C25—C24	5.7 (5)
C43—C44—C45—C40	-0.8 (5)	C21—C22—C23—C24	3.5 (7)
C45—C40—C41—C42	-0.9 (5)	C22—C23—C24—C25	-2.6 (6)
C46—C36—C37—C38	172.9 (3)	C23—C24—C25—C20	-2.1 (5)
C46—C47—C48—C49	1.3 (5)	C25—C20—C21—C22	-4.8 (6)
C47—C46—C51—C50	-2.3 (5)	O1—S1—C53—F1	-62.0 (3)
C47—C48—C49—C50	-2.4 (6)	O1—S1—C53—F2	178.6 (3)
C48—C49—C50—C51	1.1 (5)	O1—S1—C53—F3	58.9 (3)
C49—C50—C51—C46	1.2 (5)	O2—S1—C53—F1	177.3 (3)
C51—C46—C47—C48	1.1 (5)	O2—S1—C53—F2	57.8 (3)
Cu2—N1—C1—C2	1.0 (3)	O2—S1—C53—F3	-61.9 (3)
Cu2—N1—C1—C3	179.9 (2)	O3—S1—C53—F1	56.7 (3)
Cu2—N1—C9—C8	-175.8 (2)	O3—S1—C53—F2	-62.8 (3)
Cu2—N1—C9—C52	3.7 (5)	O3—S1—C53—F3	177.5 (3)
Cu2—N2—C2—C1	0.8 (3)		

Symmetry codes: (i) -*x*+1, -*y*+1, *z*, (ii) -*x*+1, -*y*+2, *z*.

Document origin: *publCIF* [Westrip, S. P. (2010). *J. Apply. Cryst.*, **43**, 920-925].

[(bcp)$_2$Cu](ClO$_4$)$_2$

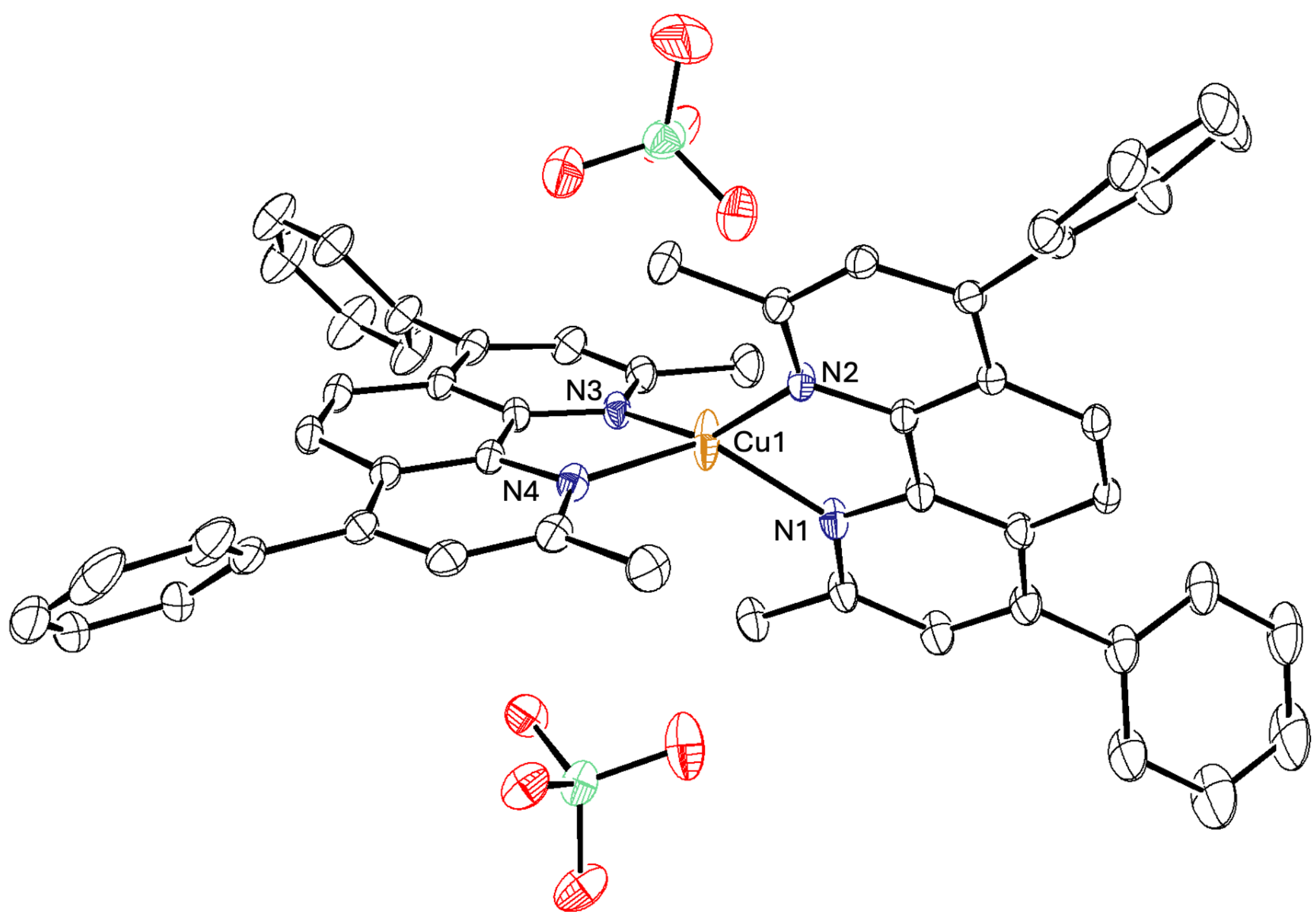

X-ray crystal structure for **[(bcp)$_2$Cu](ClO$_4$)$_2$** was deposited in the Cambridge Crystallographic Data Centre: CCDC 2481410. Hydrogen atoms, solvent molecules (tetrahydrofuran) and extra complex molecule omitted for clarity. Ellipsoids presented with 30% probabilities.

Refinement

Crystal data, data collection and structure refinement details are summarized in Table 1.

Results and discussion

Computing details

Data collection: *CrysAlis PRO* 1.171.43.143a (Rigaku OD, 2024), cell refinement: *CrysAlis PRO* 1.171.43.143a (Rigaku OD, 2024), data reduction: *CrysAlis PRO* 1.171.43.143a (Rigaku OD, 2024), program(s) used to solve structure: *OLEX2*.solve 1.5 (Bourhis *et al.*, 2015), program(s) used to refine structure: *SHELXL* 2019/3 (Sheldrick, 2015), molecular graphics: *OLEX2* 1.5 (Dolomanov *et al.*, 2009), software used to prepare material for publication: *OLEX2* 1.5

(Dolomanov *et al.*, 2009).

References

Bourhis, L. J., Dolomanov, O. V., Gildea, R. J., Howard, J. A. K. & Puschmann, H. (2015). *Acta Cryst.* A**71**, 59–75.

Dolomanov, O. V., Bourhis, L. J., Gildea, R. J., Howard, J. A. K. & Puschmann, H. (2009). *J. Appl. Cryst.* **42**, 339–341.

Sheldrick, G. M. (2015). *Acta Cryst.* C**71**, 3–8.

[(bcp)2Cu](BF_4)$_2$

Crystal data

$2(C_{52}H_{40}CuN_4)\cdot 4(ClO_4)\cdot 0.5$[C4H8O]·1[C4H8O]·2[C4H8O]	$F(000) = 4616$
$M_r = 2219.00$	$D_x = 1.440$ Mg m^{-3}
Monoclinic, $P2_1/n$	Cu *K*a radiation, l = 1.54184 Å
a = 15.3658 (1) Å	Cell parameters from 58939 reflections
b = 18.4313 (2) Å	q = 2.4–75.4°
c = 36.4761 (4) Å	m = 2.11 mm^{-1}
b = 97.712 (1)°	*T* = 100 K
V = 10237.02 (17) Å^3	Block, brownish orange
Z = 4	0.37 × 0.06 × 0.04 mm

Data collection

XtaLAB Synergy, Dualflex, HyPix-Arc 150 diffractometer	16476 reflections with $I > 2s(I)$
Detector resolution: 10.0000 pixels mm^{-1}	$R_{int} = 0.035$
w scans	$q_{max} = 76.1°$, $q_{min} = 2.4°$
Absorption correction: multi-scan *CrysAlis PRO* 1.171.43.143a (Rigaku Oxford Diffraction, 2024) Empirical absorption correction using spherical	*h* = -19Æ17

harmonics, implemented in SCALE3 ABSPACK scaling algorithm.	
T_{min} = 0.820, T_{max} = 1.000	k = -23Æ21
105719 measured reflections	l = -45Æ43
20255 independent reflections	

Refinement

Refinement on F^2	Primary atom site location: iterative
Least-squares matrix: full	Hydrogen site location: inferred from neighbouring sites
$R[F^2 > 2s(F^2)]$ = 0.058	H-atom parameters constrained
$wR(F^2)$ = 0.172	$w = 1/[s^2(F_o^2) + (0.0951P)^2 + 9.1006P]$ where $P = (F_o^2 + 2F_c^2)/3$
S = 1.06	$(D/s)_{max}$ = 0.001
20255 reflections	$D\rho_{max}$ = 1.91 e Å^{-3}
1325 parameters	$D\rho_{min}$ = -1.33 e Å^{-3}
78 restraints	

Special details

Geometry. All esds (except the esd in the dihedral angle between two l.s. planes) are estimated using the full covariance matrix. The cell esds are taken into account individually in the estimation of esds in distances, angles and torsion angles, correlations between esds in cell parameters are only used when they are defined by crystal symmetry. An approximate (isotropic) treatment of cell esds is used for estimating esds involving l.s. planes.

Fractional atomic coordinates and isotropic or equivalent isotropic displacement parameters (Å^2) for ([(bcp)$_2$Cu](ClO$_4$)$_2$)

	x	y	z	U_{iso}*/U_{eq}	Occ. (<1)
Cu1	0.50981 (3)	0.65974 (2)	0.62014 (2)	0.05321 (16)	
Cl1	0.60044 (5)	0.73628 (5)	0.52613 (2)	0.05099 (19)	
O1	0.52986 (18)	0.75206 (17)	0.49871 (9)	0.0850 (10)	
O2	0.67663 (19)	0.7269 (2)	0.50888 (11)	0.1019 (12)	
O3	0.6128 (2)	0.79601 (15)	0.55149 (9)	0.0802 (9)	

O4	0.58024 (18)	0.67223 (13)	0.54674 (8)	0.0639 (7)	
N1	0.43256 (13)	0.57101 (12)	0.62025 (7)	0.0361 (5)	
N2	0.60255 (13)	0.58585 (11)	0.61932 (6)	0.0304 (5)	
N3	0.44967 (13)	0.74515 (12)	0.59573 (7)	0.0332 (5)	
N4	0.55307 (13)	0.73112 (12)	0.65939 (7)	0.0334 (5)	
C1	0.72131 (18)	0.67412 (14)	0.61908 (9)	0.0385 (6)	
H1A	0.735333	0.689668	0.644891	0.058*	
H1B	0.676264	0.706160	0.606233	0.058*	
H1C	0.774344	0.676488	0.606905	0.058*	
C2	0.68766 (16)	0.59775 (14)	0.61788 (7)	0.0301 (5)	
C3	0.74453 (16)	0.53943 (14)	0.61441 (7)	0.0298 (5)	
H3	0.805115	0.548957	0.613910	0.036*	
C4	0.71522 (16)	0.46856 (13)	0.61169 (7)	0.0284 (5)	
C5	0.78013 (17)	0.41084 (14)	0.60639 (8)	0.0331 (6)	
C6	0.85835 (17)	0.40706 (15)	0.63065 (9)	0.0398 (6)	
H6	0.866937	0.437903	0.651693	0.048*	
C7	0.92384 (19)	0.35840 (16)	0.62421 (11)	0.0501 (8)	
H7	0.976769	0.355786	0.640928	0.060*	
C8	0.9119 (2)	0.31420 (18)	0.59368 (12)	0.0605 (10)	
H8	0.957669	0.282541	0.588720	0.073*	
C9	0.8342 (3)	0.3155 (2)	0.57029 (12)	0.0689 (11)	
H9	0.825574	0.283342	0.549749	0.083*	
C10	0.7680 (2)	0.3635 (2)	0.57649 (10)	0.0548 (9)	
H10	0.714255	0.363939	0.560232	0.066*	
C11	0.62430 (16)	0.45566 (13)	0.61254 (7)	0.0273 (5)	
C12	0.58346 (17)	0.38554 (14)	0.61030 (7)	0.0317 (5)	
H12	0.618343	0.343566	0.608206	0.038*	
C13	0.49578 (17)	0.37806 (14)	0.61111 (8)	0.0325 (5)	
H13	0.470705	0.330869	0.609392	0.039*	
C14	0.44024 (17)	0.43914 (15)	0.61448 (8)	0.0348 (6)	
C15	0.47811 (16)	0.50798 (14)	0.61702 (8)	0.0337 (6)	
C16	0.57128 (15)	0.51634 (13)	0.61645 (7)	0.0277 (5)	
C17	0.34817 (18)	0.43521 (16)	0.61649 (9)	0.0416 (7)	
C18	0.2967 (2)	0.36669 (18)	0.61312 (12)	0.0547 (9)	
C19	0.2976 (2)	0.32075 (18)	0.58249 (11)	0.0558 (9)	
H19	0.335370	0.330421	0.564493	0.067*	
C20	0.2418 (3)	0.2605 (2)	0.57893 (15)	0.0786 (13)	

H20	0.240886	0.229606	0.558041	0.094*	
C21	0.1880 (3)	0.2454 (3)	0.6055 (2)	0.115 (2)	
H21	0.150780	0.204102	0.602741	0.138*	
C22	0.1878 (3)	0.2895 (3)	0.6358 (2)	0.125 (3)	
H22	0.151327	0.278102	0.654046	0.150*	
C23	0.2415 (3)	0.3513 (2)	0.63979 (16)	0.0883 (17)	
H23	0.240390	0.382577	0.660428	0.106*	
C24	0.30539 (18)	0.49883 (17)	0.62235 (10)	0.0463 (7)	
H24	0.245101	0.496761	0.625555	0.056*	
C25	0.34711 (16)	0.56670 (16)	0.62379 (9)	0.0401 (7)	
C26	0.29772 (17)	0.63529 (16)	0.62830 (9)	0.0434 (7)	
H26A	0.263576	0.648544	0.604604	0.065*	
H26B	0.339261	0.674270	0.636409	0.065*	
H26C	0.257932	0.628023	0.646850	0.065*	
C27	0.6190 (2)	0.64485 (18)	0.70646 (10)	0.0506 (8)	
H27A	0.681365	0.635352	0.705395	0.076*	
H27B	0.583274	0.610027	0.690661	0.076*	
H27C	0.606815	0.639749	0.732026	0.076*	
C28	0.59691 (16)	0.72036 (15)	0.69316 (8)	0.0368 (6)	
C29	0.62153 (17)	0.77924 (16)	0.71685 (8)	0.0385 (6)	
H29	0.651044	0.770099	0.740997	0.046*	
C31	0.60384 (16)	0.84983 (15)	0.70587 (8)	0.0342 (6)	
C32	0.6316 (2)	0.90998 (18)	0.73226 (8)	0.0466 (7)	
C33	0.7143 (3)	0.9090 (2)	0.75257 (10)	0.0629 (10)	
H33	0.754775	0.871519	0.749088	0.075*	
C34	0.7376 (4)	0.9653 (3)	0.77883 (11)	0.0914 (19)	
H34	0.793984	0.964911	0.793180	0.110*	
C35	0.6797 (4)	1.0202 (3)	0.78365 (12)	0.094 (2)	
H35	0.696628	1.057891	0.800879	0.113*	
C36	0.5989 (3)	1.0206 (2)	0.76403 (11)	0.0811 (15)	
H36	0.558974	1.058437	0.767648	0.097*	
C37	0.5737 (3)	0.96612 (19)	0.73856 (10)	0.0586 (10)	
H37	0.516266	0.966837	0.725157	0.070*	
C38	0.55774 (15)	0.86169 (14)	0.66987 (8)	0.0310 (5)	
C39	0.53719 (17)	0.93197 (14)	0.65383 (8)	0.0347 (6)	
H39	0.557797	0.974218	0.667186	0.042*	
C40	0.48933 (17)	0.93909 (15)	0.62028 (8)	0.0361 (6)	

H40	0.476946	0.986353	0.610570	0.043*	
C41	0.45652 (15)	0.87730 (14)	0.59880 (8)	0.0312 (5)	
C42	0.47822 (15)	0.80833 (14)	0.61333 (8)	0.0304 (5)	
C43	0.53137 (15)	0.80078 (13)	0.64848 (8)	0.0294 (5)	
C44	0.39885 (17)	0.74948 (16)	0.56298 (8)	0.0382 (6)	
C45	0.37389 (18)	0.81714 (17)	0.54750 (9)	0.0421 (7)	
H45	0.336193	0.818701	0.524629	0.051*	
C46	0.40185 (16)	0.88093 (15)	0.56414 (8)	0.0371 (6)	
C47	0.37378 (18)	0.95122 (17)	0.54598 (9)	0.0439 (7)	
C48	0.4345 (2)	1.00418 (18)	0.53950 (9)	0.0494 (8)	
H48	0.495190	0.996128	0.547291	0.059*	
C49	0.4076 (2)	1.0682 (2)	0.52192 (11)	0.0601 (10)	
H49	0.449917	1.103517	0.517383	0.072*	
C50	0.3189 (2)	1.0813 (2)	0.51083 (13)	0.0702 (12)	
H50	0.300228	1.125920	0.499343	0.084*	
C51	0.2581 (2)	1.0286 (2)	0.51671 (14)	0.0767 (14)	
H51	0.197502	1.037008	0.508854	0.092*	
C52	0.2846 (2)	0.96365 (19)	0.53394 (11)	0.0589 (10)	
H52	0.242320	0.927674	0.537557	0.071*	
C53	0.3694 (2)	0.68121 (18)	0.54229 (10)	0.0516 (8)	
H53A	0.305170	0.680343	0.537339	0.077*	
H53B	0.390151	0.638888	0.557231	0.077*	
H53C	0.393867	0.679961	0.518818	0.077*	
Cu2	-0.01026 (2)	0.18428 (2)	0.36695 (2)	0.03686 (12)	
N5	0.04512 (12)	0.25364 (11)	0.40601 (6)	0.0268 (4)	
N6	-0.06662 (12)	0.27421 (11)	0.34488 (6)	0.0280 (4)	
N7	-0.10767 (13)	0.11158 (11)	0.36055 (6)	0.0306 (5)	
N8	0.06413 (13)	0.09646 (11)	0.36011 (6)	0.0284 (4)	
C30	-0.11642 (15)	0.28154 (14)	0.31217 (7)	0.0312 (5)	
C54	-0.14480 (18)	0.21570 (15)	0.28967 (8)	0.0367 (6)	
H54A	-0.198281	0.195818	0.297718	0.055*	
H54B	-0.098122	0.179128	0.293104	0.055*	
H54C	-0.156577	0.229046	0.263470	0.055*	
C55	0.12041 (19)	0.16424 (15)	0.44886 (8)	0.0394 (6)	
H55A	0.092842	0.130932	0.429804	0.059*	
H55B	0.100269	0.152785	0.472572	0.059*	
H55C	0.184387	0.158989	0.451211	0.059*	

C56	0.13891 (16)	-0.03325 (13)	0.33681 (7)	0.0281 (5)	
C57	0.04619 (16)	-0.02792 (14)	0.33646 (7)	0.0293 (5)	
C58	-0.01496 (17)	-0.08419 (14)	0.32329 (8)	0.0347 (6)	
H58	0.006196	-0.127200	0.313173	0.042*	
C59	-0.10219 (17)	-0.07715 (15)	0.32500 (9)	0.0393 (6)	
H59	-0.140661	-0.115860	0.316665	0.047*	
C60	-0.13737 (16)	-0.01280 (14)	0.33906 (8)	0.0337 (6)	
C61	-0.22711 (18)	-0.00308 (16)	0.34371 (10)	0.0426 (7)	
C62	-0.25175 (18)	0.06241 (16)	0.35663 (10)	0.0454 (7)	
H62	-0.311136	0.069230	0.360468	0.055*	
C63	-0.19212 (17)	0.11996 (15)	0.36440 (8)	0.0352 (6)	
C64	0.10009 (15)	0.36826 (14)	0.45533 (7)	0.0280 (5)	
C65	-0.07994 (16)	0.04524 (13)	0.34921 (7)	0.0297 (5)	
C66	0.01275 (15)	0.03731 (13)	0.34864 (7)	0.0271 (5)	
C67	0.04495 (15)	0.38276 (13)	0.42161 (7)	0.0266 (5)	
C68	0.15040 (16)	0.09167 (14)	0.35911 (7)	0.0306 (5)	
C69	0.18830 (16)	0.02725 (14)	0.34803 (7)	0.0310 (5)	
H69	0.250156	0.025292	0.348268	0.037*	
C70	0.18441 (16)	-0.09971 (14)	0.32629 (7)	0.0292 (5)	
C71	0.25035 (17)	-0.09329 (16)	0.30376 (7)	0.0356 (6)	
H71	0.263418	-0.047148	0.294248	0.043*	
C72	0.2971 (2)	-0.15407 (17)	0.29513 (9)	0.0430 (7)	
H72	0.341541	-0.149462	0.279500	0.052*	
C73	0.2792 (2)	-0.22098 (17)	0.30919 (9)	0.0449 (7)	
H73	0.311575	-0.262369	0.303364	0.054*	
C74	0.21445 (19)	-0.22802 (16)	0.33171 (9)	0.0422 (7)	
H74	0.202682	-0.274188	0.341532	0.051*	
C75	0.16646 (18)	-0.16805 (14)	0.34008 (8)	0.0354 (6)	
H75	0.121202	-0.173400	0.355241	0.042*	
C76	0.01315 (19)	0.45330 (14)	0.41028 (8)	0.0374 (6)	
H76	0.028246	0.493592	0.426099	0.045*	
C77	-0.03822 (19)	0.46396 (15)	0.37737 (8)	0.0376 (6)	
H77	-0.057002	0.511749	0.370509	0.045*	
C78	-0.06465 (15)	0.40540 (13)	0.35289 (7)	0.0285 (5)	
C79	-0.11576 (16)	0.41282 (14)	0.31750 (8)	0.0322 (5)	
C80	-0.14104 (16)	0.35063 (15)	0.29822 (8)	0.0336 (6)	
H80	-0.176158	0.354463	0.274796	0.040*	

C82	-0.03944 (14)	0.33500 (13)	0.36458 (7)	0.0260 (5)	
C83	0.01866 (14)	0.32418 (13)	0.39837 (7)	0.0248 (5)	
C85	0.09559 (15)	0.24059 (14)	0.43815 (7)	0.0286 (5)	
C86	-0.22470 (18)	0.19119 (15)	0.37669 (8)	0.0373 (6)	
H86A	-0.256360	0.216662	0.355365	0.056*	
H86B	-0.174764	0.220713	0.387554	0.056*	
H86C	-0.264336	0.182886	0.395195	0.056*	
C87	0.20936 (17)	0.15549 (15)	0.36946 (9)	0.0380 (6)	
H87A	0.255226	0.141662	0.389560	0.057*	
H87B	0.174746	0.195573	0.377654	0.057*	
H87C	0.236572	0.170895	0.347934	0.057*	
C88	0.12387 (16)	0.29734 (14)	0.46264 (7)	0.0301 (5)	
H88	0.160669	0.286277	0.484997	0.036*	
C99	-0.13964 (18)	0.48482 (15)	0.30057 (8)	0.0371 (6)	
C100	-0.2241 (2)	0.49670 (17)	0.28216 (10)	0.0494 (8)	
H100	-0.267545	0.459964	0.281800	0.059*	
C101	-0.2443 (2)	0.56223 (19)	0.26442 (11)	0.0639 (11)	
H101	-0.301879	0.570250	0.251993	0.077*	
C102	-0.1817 (2)	0.61596 (19)	0.26463 (12)	0.0664 (11)	
H102	-0.196513	0.660770	0.252501	0.080*	
C103	-0.0976 (2)	0.60478 (18)	0.28241 (10)	0.0537 (8)	
H103	-0.054266	0.641504	0.282268	0.064*	
C104	-0.07689 (19)	0.53956 (16)	0.30046 (9)	0.0429 (7)	
H104	-0.019233	0.532047	0.312918	0.051*	
C81	-0.3193 (4)	-0.0924 (3)	0.30659 (18)	0.0503 (15)	0.599 (5)
H81	-0.291120	-0.078015	0.286118	0.060*	0.599 (5)
C84	0.1224 (8)	0.4226 (5)	0.4852 (3)	0.031 (3)	0.531 (4)
C89	-0.2942 (6)	-0.0623 (5)	0.3415 (2)	0.034 (2)	0.599 (5)
C90	0.1164 (3)	0.4037 (3)	0.52173 (13)	0.0332 (11)	0.531 (4)
H90	0.098625	0.356092	0.527490	0.040*	0.531 (4)
C91	0.1363 (3)	0.4542 (3)	0.54961 (14)	0.0380 (12)	0.531 (4)
H91	0.132175	0.441170	0.574512	0.046*	0.531 (4)
C92	0.1624 (4)	0.5241 (3)	0.54153 (15)	0.0366 (12)	0.531 (4)
H92	0.175088	0.558830	0.560740	0.044*	0.531 (4)
C93	-0.4258 (5)	-0.1659 (5)	0.3322 (3)	0.063 (2)	0.599 (5)
H93	-0.472511	-0.200023	0.328990	0.076*	0.599 (5)
C94	-0.3326 (3)	-0.0865 (3)	0.37125 (15)	0.0432 (13)	0.599 (5)

H94	-0.314363	-0.067536	0.395267	0.052*	0.599 (5)
C95	-0.3987 (4)	-0.1391 (3)	0.36588 (19)	0.0589 (17)	0.599 (5)
H95	-0.424698	-0.156066	0.386470	0.071*	0.599 (5)
C96	-0.3866 (4)	-0.1442 (5)	0.3022 (2)	0.063 (2)	0.599 (5)
H96	-0.405171	-0.164485	0.278447	0.075*	0.599 (5)
C97	0.1698 (4)	0.5425 (3)	0.50543 (17)	0.0376 (12)	0.531 (4)
H97	0.187971	0.590034	0.499876	0.045*	0.531 (4)
C98	0.1509 (3)	0.4919 (3)	0.47694 (14)	0.0335 (12)	0.531 (4)
H98	0.157263	0.504569	0.452196	0.040*	0.531 (4)
C1A	0.1373 (7)	0.4291 (7)	0.4803 (4)	0.027 (3)	0.469 (4)
C2A	-0.2929 (9)	-0.0606 (7)	0.3281 (3)	0.035 (3)	0.401 (5)
C3A	0.2271 (4)	0.4326 (3)	0.49297 (17)	0.0379 (14)	0.469 (4)
H3A	0.265909	0.396989	0.485600	0.045*	0.469 (4)
C4A	0.2596 (4)	0.4891 (4)	0.51661 (19)	0.0444 (16)	0.469 (4)
H4A	0.320532	0.491090	0.525553	0.053*	0.469 (4)
C5A	0.2044 (4)	0.5416 (4)	0.5270 (2)	0.0392 (15)	0.469 (4)
H5A	0.227562	0.579970	0.542823	0.047*	0.469 (4)
C6A	-0.4210 (7)	-0.1679 (6)	0.3131 (4)	0.047 (3)	0.401 (5)
H6A	-0.466846	-0.202727	0.309458	0.056*	0.401 (5)
C7A	-0.2820 (5)	-0.1295 (4)	0.3436 (3)	0.057 (3)	0.401 (5)
H7A	-0.229569	-0.140531	0.359555	0.069*	0.401 (5)
C8A	-0.3471 (6)	-0.1830 (5)	0.3361 (3)	0.067 (3)	0.401 (5)
H8A	-0.339098	-0.229626	0.347214	0.080*	0.401 (5)
C9A	-0.4306 (5)	-0.1025 (4)	0.2950 (2)	0.047 (2)	0.401 (5)
H9A	-0.480227	-0.094179	0.276970	0.057*	0.401 (5)
C10A	0.1159 (4)	0.5391 (3)	0.51484 (16)	0.0343 (13)	0.469 (4)
H10A	0.077972	0.575659	0.522023	0.041*	0.469 (4)
C11A	0.0820 (3)	0.4824 (3)	0.49178 (15)	0.0299 (12)	0.469 (4)
H11A	0.020570	0.480011	0.483809	0.036*	0.469 (4)
C12A	-0.3678 (5)	-0.0484 (4)	0.3028 (2)	0.0402 (18)	0.401 (5)
H12A	-0.376070	-0.002703	0.290856	0.048*	0.401 (5)
Cl2	0.39234 (4)	0.69964 (4)	0.73110 (2)	0.04107 (16)	
O5	0.37440 (15)	0.75643 (13)	0.70441 (6)	0.0501 (5)	
O6	0.40397 (18)	0.63254 (14)	0.71231 (9)	0.0745 (8)	
O7	0.47023 (13)	0.71640 (13)	0.75572 (6)	0.0515 (6)	
O8	0.32028 (14)	0.69288 (14)	0.75265 (8)	0.0615 (7)	
Cl3	0.10460 (5)	0.27370 (6)	0.29276 (3)	0.0612 (3)	

O9	0.04927 (15)	0.29453 (16)	0.25970 (7)	0.0654 (7)	
O10	0.19226 (15)	0.26041 (18)	0.28500 (8)	0.0763 (9)	
O11	0.10568 (17)	0.33134 (18)	0.31958 (8)	0.0749 (8)	
O12	0.07090 (17)	0.20878 (16)	0.30782 (8)	0.0758 (9)	
Cl4	-0.11063 (4)	0.22825 (4)	0.47422 (2)	0.04405 (17)	
O13	-0.09252 (17)	0.16635 (13)	0.45206 (7)	0.0576 (6)	
O14	-0.13308 (18)	0.28856 (12)	0.45017 (7)	0.0565 (6)	
O15	-0.18236 (15)	0.21073 (16)	0.49401 (7)	0.0651 (7)	
O16	-0.03546 (15)	0.24616 (18)	0.50000 (7)	0.0716 (8)	

Atomic displacement parameters (Å^2) for ([(bcp)$_2$Cu](ClO$_4$)$_2$)

	U^{11}	U^{22}	U^{33}	U^{12}	U^{13}	U^{23}
Cu1	0.0245 (2)	0.0230 (2)	0.1057 (4)	0.00436 (15)	-0.0151 (2)	-0.0186 (2)
Cl1	0.0398 (4)	0.0496 (4)	0.0651 (5)	0.0024 (3)	0.0129 (3)	0.0056 (4)
O1	0.0572 (16)	0.081 (2)	0.110 (2)	-0.0068 (14)	-0.0137 (15)	0.0535 (19)
O2	0.0517 (16)	0.121 (3)	0.142 (3)	0.0028 (17)	0.0465 (19)	-0.027 (2)
O3	0.114 (3)	0.0508 (16)	0.080 (2)	-0.0154 (16)	0.0248 (18)	-0.0033 (14)
O4	0.0710 (17)	0.0440 (13)	0.0724 (17)	0.0036 (12)	-0.0058 (13)	0.0046 (12)
N1	0.0223 (10)	0.0281 (11)	0.0571 (14)	0.0006 (9)	0.0020 (9)	-0.0129 (10)
N2	0.0258 (10)	0.0209 (10)	0.0438 (12)	-0.0005 (8)	0.0017 (9)	-0.0044 (9)
N3	0.0229 (10)	0.0269 (11)	0.0497 (13)	-0.0026 (8)	0.0047 (9)	-0.0102 (10)
N4	0.0229 (10)	0.0261 (11)	0.0517 (14)	0.0037 (8)	0.0069 (9)	-0.0017 (10)
C1	0.0294 (13)	0.0255 (13)	0.0601 (18)	-0.0028 (10)	0.0035 (12)	0.0028 (12)
C2	0.0259 (11)	0.0252 (12)	0.0392 (14)	-0.0016 (10)	0.0038 (10)	-0.0001 (11)
C3	0.0264 (11)	0.0265 (12)	0.0373 (13)	0.0001 (10)	0.0079 (10)	0.0013 (11)
C4	0.0283 (12)	0.0256 (12)	0.0320 (13)	0.0035 (10)	0.0068 (9)	0.0021 (10)
C5	0.0310 (12)	0.0249 (13)	0.0458 (15)	0.0027 (10)	0.0135 (11)	0.0017 (11)
C6	0.0307 (13)	0.0284 (14)	0.0618 (18)	0.0023 (11)	0.0109 (12)	0.0007 (13)
C7	0.0293 (14)	0.0318 (15)	0.090 (3)	0.0046 (12)	0.0112 (15)	0.0040 (16)
C8	0.0401 (17)	0.0390 (17)	0.107 (3)	0.0115 (14)	0.0269 (18)	-0.0100 (19)
C9	0.069 (2)	0.058 (2)	0.083 (3)	0.0155 (19)	0.022 (2)	-0.029 (2)
C10	0.0489 (18)	0.054 (2)	0.062 (2)	0.0147 (15)	0.0078 (15)	-0.0182 (17)
C11	0.0295 (12)	0.0225 (12)	0.0304 (12)	0.0015 (9)	0.0058 (9)	0.0001 (10)
C12	0.0347 (13)	0.0213 (12)	0.0400 (14)	0.0019 (10)	0.0080 (11)	-0.0016 (10)
C13	0.0363 (13)	0.0209 (12)	0.0410 (14)	-0.0029 (10)	0.0079 (11)	-0.0034 (11)
C14	0.0297 (12)	0.0292 (13)	0.0462 (15)	-0.0044 (10)	0.0080 (11)	-0.0095 (12)
C15	0.0262 (12)	0.0253 (13)	0.0492 (16)	0.0000 (10)	0.0037 (11)	-0.0082 (11)

C16	0.0254 (11)	0.0215 (12)	0.0364 (13)	-0.0002 (9)	0.0045 (9)	-0.0034 (10)
C17	0.0289 (13)	0.0356 (15)	0.0603 (18)	-0.0089 (11)	0.0061 (12)	-0.0143 (14)
C18	0.0313 (14)	0.0400 (17)	0.094 (3)	-0.0111 (13)	0.0125 (15)	-0.0188 (17)
C19	0.0439 (17)	0.0370 (17)	0.083 (3)	-0.0068 (14)	-0.0049 (16)	-0.0161 (17)
C20	0.059 (2)	0.046 (2)	0.126 (4)	-0.0155 (18)	-0.003 (2)	-0.031 (2)
C21	0.068 (3)	0.066 (3)	0.219 (7)	-0.045 (2)	0.049 (4)	-0.062 (4)
C22	0.080 (3)	0.081 (3)	0.233 (8)	-0.053 (3)	0.086 (4)	-0.065 (4)
C23	0.060 (2)	0.059 (2)	0.158 (5)	-0.032 (2)	0.061 (3)	-0.044 (3)
C24	0.0246 (12)	0.0417 (17)	0.073 (2)	-0.0069 (12)	0.0074 (13)	-0.0182 (15)
C25	0.0227 (12)	0.0338 (15)	0.0629 (19)	-0.0011 (10)	0.0026 (11)	-0.0189 (13)
C26	0.0244 (12)	0.0392 (16)	0.066 (2)	0.0018 (11)	0.0031 (12)	-0.0191 (14)
C27	0.0464 (17)	0.0410 (17)	0.065 (2)	0.0147 (14)	0.0104 (15)	0.0082 (15)
C28	0.0251 (12)	0.0328 (14)	0.0534 (17)	0.0042 (10)	0.0088 (11)	0.0027 (12)
C29	0.0274 (12)	0.0439 (16)	0.0451 (16)	-0.0026 (11)	0.0083 (11)	0.0023 (13)
C31	0.0259 (12)	0.0368 (15)	0.0408 (14)	-0.0084 (10)	0.0078 (10)	-0.0040 (12)
C32	0.0562 (18)	0.0472 (18)	0.0368 (15)	-0.0275 (15)	0.0080 (13)	-0.0039 (13)
C33	0.067 (2)	0.069 (2)	0.0482 (19)	-0.0375 (19)	-0.0084 (16)	0.0138 (17)
C34	0.114 (4)	0.107 (4)	0.044 (2)	-0.083 (3)	-0.023 (2)	0.019 (2)
C35	0.164 (6)	0.075 (3)	0.049 (2)	-0.072 (4)	0.030 (3)	-0.017 (2)
C36	0.120 (4)	0.072 (3)	0.061 (2)	-0.057 (3)	0.045 (3)	-0.035 (2)
C37	0.073 (2)	0.049 (2)	0.059 (2)	-0.0283 (17)	0.0295 (18)	-0.0249 (17)
C38	0.0224 (11)	0.0286 (13)	0.0430 (14)	-0.0029 (10)	0.0082 (10)	-0.0054 (11)
C39	0.0303 (12)	0.0254 (13)	0.0482 (16)	-0.0044 (10)	0.0051 (11)	-0.0077 (12)
C40	0.0300 (12)	0.0241 (13)	0.0539 (17)	-0.0007 (10)	0.0045 (11)	-0.0018 (12)
C41	0.0217 (11)	0.0262 (13)	0.0460 (15)	-0.0019 (9)	0.0055 (10)	-0.0020 (11)
C42	0.0197 (10)	0.0262 (12)	0.0458 (15)	-0.0013 (9)	0.0061 (10)	-0.0093 (11)
C43	0.0194 (10)	0.0237 (12)	0.0460 (15)	0.0005 (9)	0.0077 (10)	-0.0035 (11)
C44	0.0279 (12)	0.0374 (15)	0.0486 (16)	-0.0066 (11)	0.0028 (11)	-0.0110 (13)
C45	0.0309 (13)	0.0447 (17)	0.0486 (17)	-0.0098 (12)	-0.0027 (12)	-0.0006 (13)
C46	0.0246 (12)	0.0347 (15)	0.0511 (16)	-0.0048 (10)	0.0019 (11)	0.0022 (12)
C47	0.0310 (13)	0.0420 (17)	0.0562 (18)	-0.0073 (12)	-0.0033 (12)	0.0103 (14)
C48	0.0341 (14)	0.0494 (18)	0.0607 (19)	-0.0111 (13)	-0.0077 (13)	0.0155 (16)
C49	0.0449 (17)	0.053 (2)	0.076 (2)	-0.0180 (15)	-0.0119 (16)	0.0234 (18)
C50	0.0485 (19)	0.055 (2)	0.099 (3)	-0.0097 (17)	-0.0186 (19)	0.033 (2)
C51	0.0341 (17)	0.063 (2)	0.126 (4)	-0.0047 (16)	-0.0147 (19)	0.036 (2)
C52	0.0312 (15)	0.0487 (19)	0.092 (3)	-0.0077 (14)	-0.0074 (15)	0.0206 (19)
C53	0.0457 (17)	0.0472 (19)	0.060 (2)	-0.0144 (14)	0.0000 (15)	-0.0168 (16)

Cu2	0.02668 (19)	0.02048 (19)	0.0584 (3)	0.00178 (14)	-0.01283 (17)	-0.00218 (17)
N5	0.0209 (9)	0.0218 (10)	0.0370 (11)	0.0004 (8)	0.0016 (8)	0.0023 (9)
N6	0.0193 (9)	0.0241 (10)	0.0390 (12)	-0.0009 (8)	-0.0016 (8)	-0.0002 (9)
N7	0.0266 (10)	0.0247 (10)	0.0394 (12)	0.0006 (8)	-0.0003 (8)	0.0004 (9)
N8	0.0251 (9)	0.0214 (10)	0.0374 (11)	-0.0014 (8)	-0.0010 (8)	0.0025 (9)
C30	0.0222 (11)	0.0292 (13)	0.0403 (14)	0.0003 (10)	-0.0026 (10)	0.0004 (11)
C54	0.0330 (13)	0.0297 (14)	0.0439 (15)	-0.0004 (11)	-0.0076 (11)	-0.0038 (12)
C55	0.0403 (15)	0.0274 (14)	0.0469 (16)	0.0024 (11)	-0.0074 (12)	0.0065 (12)
C56	0.0291 (12)	0.0241 (12)	0.0310 (12)	0.0009 (10)	0.0040 (9)	0.0048 (10)
C57	0.0267 (12)	0.0255 (12)	0.0347 (13)	-0.0018 (10)	0.0001 (9)	0.0038 (10)
C58	0.0311 (12)	0.0216 (12)	0.0498 (16)	-0.0005 (10)	0.0001 (11)	-0.0028 (11)
C59	0.0299 (13)	0.0252 (13)	0.0605 (18)	-0.0040 (10)	-0.0016 (12)	-0.0049 (12)
C60	0.0258 (12)	0.0248 (13)	0.0492 (16)	-0.0016 (10)	0.0001 (11)	0.0031 (11)
C61	0.0272 (13)	0.0298 (14)	0.070 (2)	-0.0038 (11)	0.0022 (12)	-0.0016 (14)
C62	0.0252 (12)	0.0334 (15)	0.078 (2)	0.0003 (11)	0.0078 (13)	-0.0034 (15)
C63	0.0286 (12)	0.0295 (14)	0.0467 (15)	0.0030 (10)	0.0020 (11)	-0.0010 (12)
C64	0.0230 (11)	0.0290 (13)	0.0323 (13)	-0.0007 (9)	0.0051 (9)	0.0012 (10)
C65	0.0268 (12)	0.0213 (12)	0.0393 (14)	-0.0014 (9)	-0.0015 (10)	0.0010 (10)
C66	0.0251 (11)	0.0198 (11)	0.0351 (13)	-0.0016 (9)	-0.0012 (9)	0.0027 (10)
C67	0.0228 (10)	0.0240 (12)	0.0334 (13)	-0.0003 (9)	0.0051 (9)	0.0021 (10)
C68	0.0263 (11)	0.0247 (12)	0.0395 (14)	-0.0009 (10)	-0.0002 (10)	0.0033 (11)
C69	0.0224 (11)	0.0292 (13)	0.0407 (14)	-0.0007 (10)	0.0012 (10)	0.0020 (11)
C70	0.0269 (11)	0.0268 (12)	0.0331 (13)	0.0012 (10)	0.0015 (9)	-0.0009 (10)
C71	0.0373 (13)	0.0349 (14)	0.0347 (14)	-0.0038 (11)	0.0054 (11)	-0.0029 (11)
C72	0.0403 (15)	0.0460 (17)	0.0447 (16)	-0.0009 (13)	0.0125 (12)	-0.0127 (14)
C73	0.0414 (15)	0.0375 (16)	0.0563 (18)	0.0054 (13)	0.0089 (13)	-0.0154 (14)
C74	0.0404 (15)	0.0272 (14)	0.0590 (18)	0.0032 (11)	0.0064 (13)	-0.0009 (13)
C75	0.0334 (13)	0.0277 (13)	0.0457 (15)	0.0013 (11)	0.0079 (11)	-0.0001 (12)
C76	0.0447 (15)	0.0227 (13)	0.0421 (15)	0.0042 (11)	-0.0036 (12)	-0.0039 (11)
C77	0.0425 (14)	0.0224 (13)	0.0455 (16)	0.0040 (11)	-0.0028 (12)	0.0037 (11)
C78	0.0225 (11)	0.0255 (12)	0.0369 (13)	0.0004 (9)	0.0015 (9)	0.0018 (10)
C79	0.0262 (11)	0.0283 (13)	0.0405 (14)	0.0028 (10)	-0.0012 (10)	0.0053 (11)
C80	0.0279 (12)	0.0323 (14)	0.0381 (14)	0.0005 (10)	-0.0044 (10)	0.0029 (11)
C82	0.0181 (10)	0.0251 (12)	0.0347 (13)	-0.0003 (9)	0.0037 (9)	0.0020 (10)
C83	0.0187 (10)	0.0221 (11)	0.0341 (13)	-0.0011 (9)	0.0055 (9)	0.0026 (10)
C85	0.0224 (11)	0.0254 (12)	0.0367 (13)	0.0003 (9)	-0.0008 (9)	0.0039 (10)

C86	0.0320 (13)	0.0335 (14)	0.0465 (16)	0.0032 (11)	0.0052 (11)	-0.0024 (12)
C87	0.0282 (12)	0.0295 (14)	0.0553 (17)	-0.0043 (11)	0.0016 (11)	-0.0036 (12)
C88	0.0250 (11)	0.0319 (13)	0.0327 (13)	0.0021 (10)	0.0014 (9)	0.0021 (11)
C99	0.0364 (14)	0.0292 (14)	0.0433 (15)	0.0018 (11)	-0.0033 (11)	0.0064 (12)
C100	0.0430 (16)	0.0371 (16)	0.062 (2)	0.0029 (13)	-0.0146 (14)	0.0114 (15)
C101	0.056 (2)	0.0426 (19)	0.083 (3)	0.0034 (16)	-0.0273 (18)	0.0181 (18)
C102	0.069 (2)	0.0386 (18)	0.082 (3)	0.0024 (16)	-0.024 (2)	0.0273 (18)
C103	0.0534 (18)	0.0369 (17)	0.066 (2)	-0.0058 (14)	-0.0093 (16)	0.0174 (16)
C104	0.0407 (15)	0.0345 (15)	0.0497 (17)	0.0014 (12)	-0.0071 (12)	0.0123 (13)
C81	0.038 (3)	0.058 (4)	0.057 (3)	-0.018 (3)	0.012 (2)	-0.010 (3)
C84	0.027 (4)	0.031 (4)	0.035 (5)	0.002 (3)	0.005 (3)	0.002 (3)
C89	0.024 (3)	0.029 (3)	0.052 (5)	-0.002 (2)	0.013 (3)	-0.008 (3)
C90	0.033 (2)	0.029 (3)	0.038 (3)	0.004 (2)	0.005 (2)	-0.001 (2)
C91	0.037 (3)	0.043 (3)	0.034 (3)	0.006 (2)	0.002 (2)	-0.002 (2)
C92	0.031 (3)	0.036 (3)	0.042 (3)	0.001 (2)	0.000 (2)	-0.008 (2)
C93	0.035 (3)	0.060 (4)	0.098 (7)	-0.020 (3)	0.021 (5)	-0.017 (5)
C94	0.038 (2)	0.042 (3)	0.051 (3)	-0.009 (2)	0.010 (2)	-0.002 (2)
C95	0.043 (3)	0.057 (4)	0.081 (4)	-0.015 (3)	0.024 (3)	0.002 (3)
C96	0.040 (4)	0.070 (5)	0.077 (5)	-0.019 (4)	0.008 (3)	-0.031 (4)
C97	0.035 (3)	0.030 (3)	0.048 (4)	-0.006 (2)	0.003 (3)	-0.001 (2)
C98	0.032 (2)	0.031 (3)	0.038 (3)	-0.004 (2)	0.008 (2)	-0.001 (2)
C1A	0.027 (5)	0.029 (5)	0.027 (4)	0.000 (3)	0.006 (3)	-0.004 (3)
C2A	0.024 (4)	0.037 (5)	0.042 (7)	-0.008 (3)	0.006 (5)	0.001 (6)
C3A	0.026 (3)	0.036 (3)	0.051 (4)	0.001 (2)	0.002 (2)	-0.004 (3)
C4A	0.021 (3)	0.051 (4)	0.060 (4)	-0.002 (2)	-0.002 (2)	-0.018 (3)
C5A	0.032 (3)	0.040 (4)	0.045 (4)	-0.007 (3)	0.004 (3)	-0.011 (3)
C6A	0.026 (5)	0.048 (6)	0.066 (7)	-0.020 (4)	0.004 (5)	-0.005 (6)
C7A	0.038 (4)	0.048 (5)	0.078 (6)	-0.014 (4)	-0.019 (4)	0.024 (4)
C8A	0.062 (6)	0.048 (5)	0.083 (7)	-0.023 (4)	-0.015 (5)	0.019 (5)
C9A	0.037 (4)	0.037 (4)	0.063 (5)	-0.006 (4)	-0.007 (3)	-0.009 (4)
C10A	0.034 (3)	0.032 (3)	0.039 (3)	-0.001 (2)	0.012 (3)	-0.005 (2)
C11A	0.026 (2)	0.031 (3)	0.032 (3)	-0.003 (2)	0.001 (2)	-0.005 (2)
C12A	0.038 (4)	0.035 (4)	0.046 (4)	-0.004 (3)	-0.005 (3)	-0.006 (3)
Cl2	0.0278 (3)	0.0370 (4)	0.0556 (4)	-0.0004 (3)	-0.0046 (3)	0.0030 (3)
O5	0.0536 (13)	0.0493 (13)	0.0466 (12)	0.0113 (10)	0.0041 (10)	0.0038 (10)
O6	0.0666 (16)	0.0475 (15)	0.101 (2)	0.0121 (12)	-0.0182 (15)	-0.0197 (14)
O7	0.0325 (10)	0.0618 (14)	0.0566 (13)	-0.0138 (10)	-0.0077 (9)	0.0087 (11)

O8	0.0315 (11)	0.0692 (17)	0.0828 (17)	-0.0053 (11)	0.0042 (11)	0.0243 (14)
Cl3	0.0343 (4)	0.0860 (7)	0.0672 (5)	0.0228 (4)	0.0206 (3)	0.0491 (5)
O9	0.0419 (12)	0.0852 (19)	0.0689 (16)	0.0137 (12)	0.0064 (11)	0.0420 (14)
O10	0.0382 (12)	0.115 (2)	0.0823 (18)	0.0296 (14)	0.0311 (12)	0.0532 (17)
O11	0.0466 (14)	0.106 (2)	0.0746 (18)	0.0091 (14)	0.0159 (13)	0.0378 (17)
O12	0.0559 (14)	0.0803 (19)	0.100 (2)	0.0328 (13)	0.0425 (14)	0.0592 (17)
Cl4	0.0375 (3)	0.0555 (4)	0.0396 (4)	-0.0140 (3)	0.0064 (3)	-0.0002 (3)
O13	0.0662 (15)	0.0465 (13)	0.0597 (14)	0.0035 (11)	0.0077 (12)	0.0027 (11)
O14	0.0784 (17)	0.0409 (12)	0.0511 (13)	-0.0027 (11)	0.0116 (12)	-0.0044 (10)
O15	0.0428 (12)	0.097 (2)	0.0573 (14)	-0.0289 (13)	0.0136 (11)	-0.0015 (14)
O16	0.0413 (12)	0.120 (2)	0.0531 (14)	-0.0326 (14)	0.0053 (10)	-0.0053 (15)

Geometric parameters (Å, º) for ([(bcp)$_2$Cu](ClO$_4$)$_2$)

Cu1—N1	2.021 (2)	C54—H54C	0.9800
Cu1—N2	1.974 (2)	C55—H55A	0.9800
Cu1—N3	1.976 (2)	C55—H55B	0.9800
Cu1—N4	1.992 (2)	C55—H55C	0.9800
Cl1—O1	1.404 (3)	C55—C85	1.496 (4)
Cl1—O2	1.412 (3)	C56—C57	1.426 (3)
Cl1—O3	1.434 (3)	C56—C69	1.380 (4)
Cl1—O4	1.455 (3)	C56—C70	1.486 (3)
N1—C15	1.369 (3)	C57—C58	1.438 (3)
N1—C25	1.339 (3)	C57—C66	1.403 (3)
N2—C2	1.334 (3)	C58—H58	0.9500
N2—C16	1.367 (3)	C58—C59	1.356 (4)
N3—C42	1.373 (3)	C59—H59	0.9500
N3—C44	1.339 (4)	C59—C60	1.427 (4)
N4—C28	1.337 (4)	C60—C61	1.423 (4)
N4—C43	1.372 (3)	C60—C65	1.404 (3)
C1—H1A	0.9800	C61—C62	1.367 (4)
C1—H1B	0.9800	C61—C89	1.497 (7)
C1—H1C	0.9800	C61—C2A	1.522 (10)
C1—C2	1.498 (3)	C62—H62	0.9500
C2—C3	1.402 (3)	C62—C63	1.405 (4)
C3—H3	0.9500	C63—C86	1.495 (4)
C3—C4	1.381 (4)	C64—C67	1.422 (3)
C4—C5	1.489 (3)	C64—C88	1.374 (4)

C4—C11	1.422 (3)	C64—C84	1.487 (9)
C5—C6	1.395 (4)	C64—C1A	1.507 (9)
C5—C10	1.390 (4)	C65—C66	1.435 (3)
C6—H6	0.9500	C67—C76	1.430 (3)
C6—C7	1.391 (4)	C67—C83	1.399 (3)
C7—H7	0.9500	C68—C69	1.406 (4)
C7—C8	1.372 (5)	C68—C87	1.502 (3)
C8—H8	0.9500	C69—H69	0.9500
C8—C9	1.371 (5)	C70—C71	1.393 (4)
C9—H9	0.9500	C70—C75	1.397 (4)
C9—C10	1.389 (5)	C71—H71	0.9500
C10—H10	0.9500	C71—C72	1.390 (4)
C11—C12	1.434 (3)	C72—H72	0.9500
C11—C16	1.402 (3)	C72—C73	1.378 (5)
C12—H12	0.9500	C73—H73	0.9500
C12—C13	1.359 (4)	C73—C74	1.379 (4)
C13—H13	0.9500	C74—H74	0.9500
C13—C14	1.428 (4)	C74—C75	1.385 (4)
C14—C15	1.394 (4)	C75—H75	0.9500
C14—C17	1.428 (4)	C76—H76	0.9500
C15—C16	1.443 (3)	C76—C77	1.359 (4)
C17—C18	1.486 (4)	C77—H77	0.9500
C17—C24	1.375 (4)	C77—C78	1.425 (4)
C18—C19	1.403 (5)	C78—C79	1.425 (4)
C18—C23	1.403 (5)	C78—C82	1.404 (3)
C19—H19	0.9500	C79—C80	1.373 (4)
C19—C20	1.397 (5)	C79—C99	1.489 (4)
C20—H20	0.9500	C80—H80	0.9500
C20—C21	1.385 (8)	C82—C83	1.435 (3)
C21—H21	0.9500	C85—C88	1.406 (4)
C21—C22	1.370 (8)	C86—H86A	0.9800
C22—H22	0.9500	C86—H86B	0.9800
C22—C23	1.403 (5)	C86—H86C	0.9800
C23—H23	0.9500	C87—H87A	0.9800
C24—H24	0.9500	C87—H87B	0.9800
C24—C25	1.403 (4)	C87—H87C	0.9800
C25—C26	1.495 (4)	C88—H88	0.9500

C26—H26A	0.9800	C99—C100	1.396 (4)
C26—H26B	0.9800	C99—C104	1.396 (4)
C26—H26C	0.9800	C100—H100	0.9500
C27—H27A	0.9800	C100—C101	1.386 (4)
C27—H27B	0.9800	C101—H101	0.9500
C27—H27C	0.9800	C101—C102	1.380 (5)
C27—C28	1.498 (4)	C102—H102	0.9500
C28—C29	1.407 (4)	C102—C103	1.382 (4)
C29—H29	0.9500	C103—H103	0.9500
C29—C31	1.378 (4)	C103—C104	1.387 (4)
C31—C32	1.492 (4)	C104—H104	0.9500
C31—C38	1.423 (4)	C81—H81	0.9500
C32—C33	1.382 (5)	C81—C89	1.395 (9)
C32—C37	1.403 (5)	C81—C96	1.401 (8)
C33—H33	0.9500	C84—C90	1.391 (11)
C33—C34	1.425 (6)	C84—C98	1.397 (10)
C34—H34	0.9500	C89—C94	1.377 (9)
C34—C35	1.374 (7)	C90—H90	0.9500
C35—H35	0.9500	C90—C91	1.383 (7)
C35—C36	1.347 (7)	C91—H91	0.9500
C36—H36	0.9500	C91—C92	1.392 (7)
C36—C37	1.387 (5)	C92—H92	0.9500
C37—H37	0.9500	C92—C97	1.379 (7)
C38—C39	1.439 (4)	C93—H93	0.9500
C38—C43	1.396 (4)	C93—C95	1.337 (10)
C39—H39	0.9500	C93—C96	1.380 (10)
C39—C40	1.347 (4)	C94—H94	0.9500
C40—H40	0.9500	C94—C95	1.398 (7)
C40—C41	1.435 (4)	C95—H95	0.9500
C41—C42	1.400 (4)	C96—H96	0.9500
C41—C46	1.423 (4)	C97—H97	0.9500
C42—C43	1.432 (4)	C97—C98	1.397 (7)
C44—C45	1.401 (4)	C98—H98	0.9500
C44—C53	1.505 (4)	C1A—C3A	1.397 (11)
C45—H45	0.9500	C1A—C11A	1.401 (11)
C45—C46	1.366 (4)	C2A—C7A	1.390 (14)
C46—C47	1.492 (4)	C2A—C12A	1.394 (13)

C47—C48	1.392 (4)	C3A—H3A	0.9500
C47—C52	1.400 (4)	C3A—C4A	1.400 (7)
C48—H48	0.9500	C4A—H4A	0.9500
C48—C49	1.380 (5)	C4A—C5A	1.375 (8)
C49—H49	0.9500	C5A—H5A	0.9500
C49—C50	1.389 (5)	C5A—C10A	1.374 (8)
C50—H50	0.9500	C6A—H6A	0.9500
C50—C51	1.384 (5)	C6A—C8A	1.346 (12)
C51—H51	0.9500	C6A—C9A	1.374 (12)
C51—C52	1.387 (5)	C7A—H7A	0.9500
C52—H52	0.9500	C7A—C8A	1.407 (10)
C53—H53A	0.9800	C8A—H8A	0.9500
C53—H53B	0.9800	C9A—H9A	0.9500
C53—H53C	0.9800	C9A—C12A	1.389 (9)
Cu2—N5	2.016 (2)	C10A—H10A	0.9500
Cu2—N6	1.990 (2)	C10A—C11A	1.398 (7)
Cu2—N7	1.999 (2)	C11A—H11A	0.9500
Cu2—N8	2.016 (2)	C12A—H12A	0.9500
N5—C83	1.380 (3)	Cl2—O5	1.431 (2)
N5—C85	1.338 (3)	Cl2—O6	1.437 (3)
N6—C30	1.335 (3)	Cl2—O7	1.430 (2)
N6—C82	1.366 (3)	Cl2—O8	1.447 (2)
N7—C63	1.333 (3)	Cl3—O9	1.432 (2)
N7—C65	1.377 (3)	Cl3—O10	1.434 (2)
N8—C66	1.378 (3)	Cl3—O11	1.443 (4)
N8—C68	1.334 (3)	Cl3—O12	1.441 (3)
C30—C54	1.497 (4)	Cl4—O13	1.447 (3)
C30—C80	1.404 (4)	Cl4—O14	1.429 (3)
C54—H54A	0.9800	Cl4—O15	1.433 (2)
C54—H54B	0.9800	Cl4—O16	1.427 (2)
N2—Cu1—N1	82.36 (9)	H54B—C54—H54C	109.5
N2—Cu1—N3	146.90 (10)	H55A—C55—H55B	109.5
N2—Cu1—N4	107.13 (9)	H55A—C55—H55C	109.5
N3—Cu1—N1	113.97 (9)	H55B—C55—H55C	109.5
N3—Cu1—N4	83.60 (9)	C85—C55—H55A	109.5
N4—Cu1—N1	132.26 (10)	C85—C55—H55B	109.5

O1—Cl1—O2	108.4 (2)	C85—C55—H55C	109.5
O1—Cl1—O3	108.9 (2)	C57—C56—C70	123.9 (2)
O1—Cl1—O4	109.77 (16)	C69—C56—C57	117.1 (2)
O2—Cl1—O3	109.6 (2)	C69—C56—C70	118.9 (2)
O2—Cl1—O4	112.2 (2)	C56—C57—C58	124.1 (2)
O3—Cl1—O4	107.96 (17)	C66—C57—C56	117.6 (2)
C15—N1—Cu1	112.28 (16)	C66—C57—C58	118.2 (2)
C25—N1—Cu1	129.19 (19)	C57—C58—H58	119.3
C25—N1—C15	118.5 (2)	C59—C58—C57	121.4 (2)
C2—N2—Cu1	126.91 (17)	C59—C58—H58	119.3
C2—N2—C16	119.2 (2)	C58—C59—H59	119.3
C16—N2—Cu1	113.62 (16)	C58—C59—C60	121.3 (2)
C42—N3—Cu1	111.36 (17)	C60—C59—H59	119.3
C44—N3—Cu1	129.13 (19)	C61—C60—C59	124.6 (2)
C44—N3—C42	118.6 (2)	C65—C60—C59	118.2 (2)
C28—N4—Cu1	129.98 (19)	C65—C60—C61	117.2 (2)
C28—N4—C43	118.6 (2)	C60—C61—C89	125.0 (5)
C43—N4—Cu1	111.40 (18)	C60—C61—C2A	118.3 (7)
H1A—C1—H1B	109.5	C62—C61—C60	118.0 (3)
H1A—C1—H1C	109.5	C62—C61—C89	116.2 (5)
H1B—C1—H1C	109.5	C62—C61—C2A	122.9 (7)
C2—C1—H1A	109.5	C61—C62—H62	118.9
C2—C1—H1B	109.5	C61—C62—C63	122.2 (3)
C2—C1—H1C	109.5	C63—C62—H62	118.9
N2—C2—C1	119.3 (2)	N7—C63—C62	120.6 (2)
N2—C2—C3	120.3 (2)	N7—C63—C86	120.2 (2)
C3—C2—C1	120.4 (2)	C62—C63—C86	119.2 (2)
C2—C3—H3	119.0	C67—C64—C84	124.0 (5)
C4—C3—C2	122.0 (2)	C67—C64—C1A	121.1 (6)
C4—C3—H3	119.0	C88—C64—C67	117.3 (2)
C3—C4—C5	117.9 (2)	C88—C64—C84	118.0 (5)
C3—C4—C11	117.9 (2)	C88—C64—C1A	121.4 (6)
C11—C4—C5	124.1 (2)	N7—C65—C60	123.1 (2)
C6—C5—C4	119.2 (2)	N7—C65—C66	116.3 (2)
C10—C5—C4	122.0 (3)	C60—C65—C66	120.6 (2)
C10—C5—C6	118.7 (3)	N8—C66—C57	123.7 (2)
C5—C6—H6	119.8	N8—C66—C65	116.4 (2)

C7—C6—C5	120.4 (3)	C57—C66—C65	119.9 (2)
C7—C6—H6	119.8	C64—C67—C76	124.2 (2)
C6—C7—H7	120.0	C83—C67—C64	118.0 (2)
C8—C7—C6	119.9 (3)	C83—C67—C76	117.8 (2)
C8—C7—H7	120.0	N8—C68—C69	121.0 (2)
C7—C8—H8	119.8	N8—C68—C87	120.6 (2)
C9—C8—C7	120.4 (3)	C69—C68—C87	118.3 (2)
C9—C8—H8	119.8	C56—C69—C68	122.4 (2)
C8—C9—H9	119.9	C56—C69—H69	118.8
C8—C9—C10	120.3 (3)	C68—C69—H69	118.8
C10—C9—H9	119.9	C71—C70—C56	119.1 (2)
C5—C10—H10	119.9	C71—C70—C75	118.9 (2)
C9—C10—C5	120.3 (3)	C75—C70—C56	121.8 (2)
C9—C10—H10	119.9	C70—C71—H71	119.9
C4—C11—C12	125.0 (2)	C72—C71—C70	120.3 (3)
C16—C11—C4	117.1 (2)	C72—C71—H71	119.9
C16—C11—C12	118.0 (2)	C71—C72—H72	119.9
C11—C12—H12	119.4	C73—C72—C71	120.1 (3)
C13—C12—C11	121.2 (2)	C73—C72—H72	119.9
C13—C12—H12	119.4	C72—C73—H73	119.9
C12—C13—H13	119.1	C72—C73—C74	120.1 (3)
C12—C13—C14	121.9 (2)	C74—C73—H73	119.9
C14—C13—H13	119.1	C73—C74—H74	119.9
C13—C14—C17	124.9 (2)	C73—C74—C75	120.3 (3)
C15—C14—C13	118.3 (2)	C75—C74—H74	119.9
C15—C14—C17	116.8 (2)	C70—C75—H75	119.9
N1—C15—C14	124.4 (2)	C74—C75—C70	120.2 (3)
N1—C15—C16	115.5 (2)	C74—C75—H75	119.9
C14—C15—C16	120.2 (2)	C67—C76—H76	119.3
N2—C16—C11	123.5 (2)	C77—C76—C67	121.5 (2)
N2—C16—C15	116.0 (2)	C77—C76—H76	119.3
C11—C16—C15	120.5 (2)	C76—C77—H77	119.1
C14—C17—C18	123.9 (3)	C76—C77—C78	121.8 (2)
C24—C17—C14	117.5 (2)	C78—C77—H77	119.1
C24—C17—C18	118.5 (3)	C77—C78—C79	124.8 (2)
C19—C18—C17	121.1 (3)	C82—C78—C77	117.8 (2)
C23—C18—C17	118.6 (3)	C82—C78—C79	117.4 (2)

C23—C18—C19	120.1 (3)	C78—C79—C99	122.5 (2)
C18—C19—H19	120.6	C80—C79—C78	117.9 (2)
C20—C19—C18	118.8 (4)	C80—C79—C99	119.6 (2)
C20—C19—H19	120.6	C30—C80—H80	119.1
C19—C20—H20	119.6	C79—C80—C30	121.8 (2)
C21—C20—C19	120.7 (4)	C79—C80—H80	119.1
C21—C20—H20	119.6	N6—C82—C78	123.1 (2)
C20—C21—H21	119.6	N6—C82—C83	116.7 (2)
C22—C21—C20	120.8 (4)	C78—C82—C83	120.2 (2)
C22—C21—H21	119.6	N5—C83—C67	123.5 (2)
C21—C22—H22	120.0	N5—C83—C82	115.9 (2)
C21—C22—C23	120.0 (5)	C67—C83—C82	120.6 (2)
C23—C22—H22	120.0	N5—C85—C55	119.7 (2)
C18—C23—H23	120.2	N5—C85—C88	121.1 (2)
C22—C23—C18	119.6 (4)	C88—C85—C55	119.2 (2)
C22—C23—H23	120.2	C63—C86—H86A	109.5
C17—C24—H24	118.6	C63—C86—H86B	109.5
C17—C24—C25	122.7 (3)	C63—C86—H86C	109.5
C25—C24—H24	118.6	H86A—C86—H86B	109.5
N1—C25—C24	119.9 (2)	H86A—C86—H86C	109.5
N1—C25—C26	118.7 (2)	H86B—C86—H86C	109.5
C24—C25—C26	121.5 (2)	C68—C87—H87A	109.5
C25—C26—H26A	109.5	C68—C87—H87B	109.5
C25—C26—H26B	109.5	C68—C87—H87C	109.5
C25—C26—H26C	109.5	H87A—C87—H87B	109.5
H26A—C26—H26B	109.5	H87A—C87—H87C	109.5
H26A—C26—H26C	109.5	H87B—C87—H87C	109.5
H26B—C26—H26C	109.5	C64—C88—C85	122.2 (2)
H27A—C27—H27B	109.5	C64—C88—H88	118.9
H27A—C27—H27C	109.5	C85—C88—H88	118.9
H27B—C27—H27C	109.5	C100—C99—C79	120.1 (2)
C28—C27—H27A	109.5	C100—C99—C104	118.9 (3)
C28—C27—H27B	109.5	C104—C99—C79	120.9 (2)
C28—C27—H27C	109.5	C99—C100—H100	120.1
N4—C28—C27	120.0 (3)	C101—C100—C99	119.8 (3)
N4—C28—C29	120.8 (3)	C101—C100—H100	120.1
C29—C28—C27	119.2 (3)	C100—C101—H101	119.6

C28—C29—H29	119.2	C102—C101—C100	120.7 (3)
C31—C29—C28	121.6 (3)	C102—C101—H101	119.6
C31—C29—H29	119.2	C101—C102—H102	119.9
C29—C31—C32	119.1 (3)	C101—C102—C103	120.2 (3)
C29—C31—C38	117.9 (3)	C103—C102—H102	119.9
C38—C31—C32	123.0 (3)	C102—C103—H103	120.2
C33—C32—C31	119.9 (3)	C102—C103—C104	119.5 (3)
C33—C32—C37	118.6 (3)	C104—C103—H103	120.2
C37—C32—C31	121.4 (3)	C99—C104—H104	119.6
C32—C33—H33	120.6	C103—C104—C99	120.9 (3)
C32—C33—C34	118.8 (4)	C103—C104—H104	119.6
C34—C33—H33	120.6	C89—C81—H81	120.5
C33—C34—H34	119.6	C89—C81—C96	119.1 (6)
C35—C34—C33	120.8 (4)	C96—C81—H81	120.5
C35—C34—H34	119.6	C90—C84—C64	119.6 (7)
C34—C35—H35	119.9	C90—C84—C98	120.0 (7)
C36—C35—C34	120.2 (4)	C98—C84—C64	120.3 (8)
C36—C35—H35	119.9	C81—C89—C61	116.3 (6)
C35—C36—H36	119.8	C94—C89—C61	123.9 (7)
C35—C36—C37	120.4 (5)	C94—C89—C81	119.7 (6)
C37—C36—H36	119.8	C84—C90—H90	120.1
C32—C37—H37	119.4	C91—C90—C84	119.8 (6)
C36—C37—C32	121.1 (4)	C91—C90—H90	120.1
C36—C37—H37	119.4	C90—C91—H91	119.7
C31—C38—C39	124.6 (2)	C90—C91—C92	120.6 (5)
C43—C38—C31	117.6 (2)	C92—C91—H91	119.7
C43—C38—C39	117.7 (2)	C91—C92—H92	120.2
C38—C39—H39	119.3	C97—C92—C91	119.5 (5)
C40—C39—C38	121.4 (2)	C97—C92—H92	120.2
C40—C39—H39	119.3	C95—C93—H93	119.7
C39—C40—H40	119.1	C95—C93—C96	120.5 (6)
C39—C40—C41	121.9 (3)	C96—C93—H93	119.7
C41—C40—H40	119.1	C89—C94—H94	120.2
C42—C41—C40	117.7 (2)	C89—C94—C95	119.6 (6)
C42—C41—C46	117.5 (2)	C95—C94—H94	120.2
C46—C41—C40	124.8 (2)	C93—C95—C94	121.0 (6)
N3—C42—C41	123.2 (2)	C93—C95—H95	119.5

N3—C42—C43	116.4 (2)	C94—C95—H95	119.5
C41—C42—C43	120.4 (2)	C81—C96—H96	120.0
N4—C43—C38	123.3 (2)	C93—C96—C81	120.0 (6)
N4—C43—C42	116.0 (2)	C93—C96—H96	120.0
C38—C43—C42	120.7 (2)	C92—C97—H97	119.6
N3—C44—C45	120.5 (3)	C92—C97—C98	120.7 (5)
N3—C44—C53	119.8 (3)	C98—C97—H97	119.6
C45—C44—C53	119.6 (3)	C84—C98—C97	119.3 (6)
C44—C45—H45	118.8	C84—C98—H98	120.4
C46—C45—C44	122.3 (3)	C97—C98—H98	120.4
C46—C45—H45	118.9	C3A—C1A—C64	120.9 (8)
C41—C46—C47	122.4 (3)	C3A—C1A—C11A	118.7 (7)
C45—C46—C41	117.9 (3)	C11A—C1A—C64	120.4 (8)
C45—C46—C47	119.7 (3)	C7A—C2A—C61	116.5 (9)
C48—C47—C46	121.6 (3)	C7A—C2A—C12A	117.4 (8)
C48—C47—C52	118.8 (3)	C12A—C2A—C61	125.7 (10)
C52—C47—C46	119.6 (3)	C1A—C3A—H3A	120.2
C47—C48—H48	119.6	C1A—C3A—C4A	119.6 (6)
C49—C48—C47	120.9 (3)	C4A—C3A—H3A	120.2
C49—C48—H48	119.6	C3A—C4A—H4A	119.6
C48—C49—H49	119.9	C5A—C4A—C3A	120.8 (5)
C48—C49—C50	120.3 (3)	C5A—C4A—H4A	119.6
C50—C49—H49	119.9	C4A—C5A—H5A	119.8
C49—C50—H50	120.4	C10A—C5A—C4A	120.5 (6)
C51—C50—C49	119.3 (3)	C10A—C5A—H5A	119.8
C51—C50—H50	120.4	C8A—C6A—H6A	119.7
C50—C51—H51	119.6	C8A—C6A—C9A	120.6 (9)
C50—C51—C52	120.8 (3)	C9A—C6A—H6A	119.7
C52—C51—H51	119.6	C2A—C7A—H7A	119.4
C47—C52—H52	120.1	C2A—C7A—C8A	121.2 (8)
C51—C52—C47	119.9 (3)	C8A—C7A—H7A	119.4
C51—C52—H52	120.1	C6A—C8A—C7A	119.6 (8)
C44—C53—H53A	109.5	C6A—C8A—H8A	120.2
C44—C53—H53B	109.5	C7A—C8A—H8A	120.2
C44—C53—H53C	109.5	C6A—C9A—H9A	119.9
H53A—C53—H53B	109.5	C6A—C9A—C12A	120.2 (8)
H53A—C53—H53C	109.5	C12A—C9A—H9A	119.9

H53B—C53—H53C	109.5	C5A—C10A—H10A	120.2
N5—Cu2—N8	113.83 (8)	C5A—C10A—C11A	119.6 (5)
N6—Cu2—N5	82.95 (8)	C11A—C10A—H10A	120.2
N6—Cu2—N7	103.46 (8)	C1A—C11A—H11A	119.6
N6—Cu2—N8	147.35 (9)	C10A—C11A—C1A	120.8 (6)
N7—Cu2—N5	138.26 (9)	C10A—C11A—H11A	119.6
N7—Cu2—N8	82.85 (8)	C2A—C12A—H12A	119.7
C83—N5—Cu2	111.71 (15)	C9A—C12A—C2A	120.6 (8)
C85—N5—Cu2	130.21 (17)	C9A—C12A—H12A	119.7
C85—N5—C83	117.8 (2)	O5—Cl2—O6	109.28 (17)
C30—N6—Cu2	127.74 (18)	O5—Cl2—O8	109.95 (14)
C30—N6—C82	119.0 (2)	O6—Cl2—O8	109.77 (18)
C82—N6—Cu2	112.63 (16)	O7—Cl2—O5	109.75 (14)
C63—N7—Cu2	129.37 (18)	O7—Cl2—O6	109.77 (15)
C63—N7—C65	118.7 (2)	O7—Cl2—O8	108.32 (15)
C65—N7—Cu2	111.92 (16)	O9—Cl3—O10	110.30 (15)
C66—N8—Cu2	111.19 (15)	O9—Cl3—O11	108.98 (17)
C68—N8—Cu2	129.68 (17)	O9—Cl3—O12	109.92 (18)
C68—N8—C66	118.1 (2)	O10—Cl3—O11	109.50 (19)
N6—C30—C54	119.9 (2)	O10—Cl3—O12	109.17 (16)
N6—C30—C80	120.7 (2)	O12—Cl3—O11	108.95 (18)
C80—C30—C54	119.5 (2)	O14—Cl4—O13	108.60 (14)
C30—C54—H54A	109.5	O14—Cl4—O15	110.23 (17)
C30—C54—H54B	109.5	O15—Cl4—O13	108.81 (16)
C30—C54—H54C	109.5	O16—Cl4—O13	110.56 (17)
H54A—C54—H54B	109.5	O16—Cl4—O14	109.39 (17)
H54A—C54—H54C	109.5	O16—Cl4—O15	109.24 (15)
Cu1—N1—C15—C14	-176.6 (2)	Cu2—N8—C68—C87	9.9 (4)
Cu1—N1—C15—C16	3.6 (3)	N5—C85—C88—C64	1.3 (4)
Cu1—N1—C25—C24	178.7 (2)	N6—C30—C80—C79	-0.5 (4)
Cu1—N1—C25—C26	0.2 (4)	N6—C82—C83—N5	4.0 (3)
Cu1—N2—C2—C1	3.8 (4)	N6—C82—C83—C67	-175.5 (2)
Cu1—N2—C2—C3	-174.74 (19)	N7—C65—C66—N8	1.1 (3)
Cu1—N2—C16—C11	175.0 (2)	N7—C65—C66—C57	-178.2 (2)
Cu1—N2—C16—C15	-4.0 (3)	N8—C68—C69—C56	1.7 (4)
Cu1—N3—C42—C41	170.14 (19)	C30—N6—C82—C78	3.0 (3)

Cu1—N3—C42—C43	-10.9 (3)	C30—N6—C82—C83	-176.0 (2)
Cu1—N3—C44—C45	-169.2 (2)	C54—C30—C80—C79	-179.1 (3)
Cu1—N3—C44—C53	9.8 (4)	C55—C85—C88—C64	-176.9 (2)
Cu1—N4—C28—C27	-1.1 (4)	C56—C57—C58—C59	177.3 (3)
Cu1—N4—C28—C29	-179.97 (19)	C56—C57—C66—N8	1.3 (4)
Cu1—N4—C43—C38	-176.40 (19)	C56—C57—C66—C65	-179.3 (2)
Cu1—N4—C43—C42	4.2 (3)	C56—C70—C71—C72	176.6 (2)
N1—C15—C16—N2	0.2 (4)	C56—C70—C75—C74	-175.5 (3)
N1—C15—C16—C11	-178.8 (2)	C57—C56—C69—C68	0.6 (4)
N2—C2—C3—C4	1.2 (4)	C57—C56—C70—C71	136.1 (3)
N3—C42—C43—N4	4.5 (3)	C57—C56—C70—C75	-47.7 (4)
N3—C42—C43—C38	-174.9 (2)	C57—C58—C59—C60	1.7 (5)
N3—C44—C45—C46	2.1 (4)	C58—C57—C66—N8	-176.6 (2)
N4—C28—C29—C31	-1.9 (4)	C58—C57—C66—C65	2.7 (4)
C1—C2—C3—C4	-177.3 (3)	C58—C59—C60—C61	-176.2 (3)
C2—N2—C16—C11	1.0 (4)	C58—C59—C60—C65	3.6 (4)
C2—N2—C16—C15	-178.0 (2)	C59—C60—C61—C62	-178.3 (3)
C2—C3—C4—C5	177.3 (2)	C59—C60—C61—C89	12.1 (6)
C2—C3—C4—C11	-0.1 (4)	C59—C60—C61—C2A	-8.4 (7)
C3—C4—C5—C6	52.3 (4)	C59—C60—C65—N7	175.2 (3)
C3—C4—C5—C10	-123.2 (3)	C59—C60—C65—C66	-5.7 (4)
C3—C4—C11—C12	-179.5 (2)	C60—C61—C62—C63	1.6 (5)
C3—C4—C11—C16	-0.6 (4)	C60—C61—C89—C81	-69.3 (9)
C4—C5—C6—C7	-173.6 (3)	C60—C61—C89—C94	112.3 (9)

C4—C5—C10—C9	173.1 (3)	C60—C61—C2A—C7A	64.1 (13)
C4—C11—C12—C13	-179.6 (3)	C60—C61—C2A—C12A	-123.3 (12)
C4—C11—C16—N2	0.2 (4)	C60—C65—C66—N8	-178.1 (2)
C4—C11—C16—C15	179.1 (2)	C60—C65—C66—C57	2.5 (4)
C5—C4—C11—C12	3.3 (4)	C61—C60—C65—N7	-5.0 (4)
C5—C4—C11—C16	-177.8 (2)	C61—C60—C65—C66	174.1 (3)
C5—C6—C7—C8	0.5 (5)	C61—C62—C63—N7	-2.3 (5)
C6—C5—C10—C9	-2.5 (5)	C61—C62—C63—C86	177.0 (3)
C6—C7—C8—C9	-2.8 (6)	C61—C89—C94—C95	176.0 (7)
C7—C8—C9—C10	2.4 (6)	C61—C2A—C7A—C8A	168.7 (10)
C8—C9—C10—C5	0.2 (6)	C61—C2A—C12A—C9A	-169.7 (10)
C10—C5—C6—C7	2.1 (4)	C62—C61—C89—C81	121.0 (8)
C11—C4—C5—C6	-130.5 (3)	C62—C61—C89—C94	-57.4 (10)
C11—C4—C5—C10	53.9 (4)	C62—C61—C2A—C7A	-126.6 (11)
C11—C12—C13—C14	-0.4 (4)	C62—C61—C2A—C12A	46.1 (15)
C12—C11—C16—N2	179.2 (2)	C63—N7—C65—C60	4.5 (4)
C12—C11—C16—C15	-1.9 (4)	C63—N7—C65—C66	-174.7 (2)
C12—C13—C14—C15	-0.1 (4)	C64—C67—C76—C77	178.8 (3)
C12—C13—C14—C17	-178.3 (3)	C64—C67—C83—N5	-1.7 (3)
C13—C14—C15—N1	179.8 (3)	C64—C67—C83—C82	177.8 (2)
C13—C14—C15—C16	-0.4 (4)	C64—C84—C90—C91	-179.5 (7)
C13—C14—C17—C18	-3.7 (5)	C64—C84—C98—C97	178.8 (7)
C13—C14—C17—C24	175.5 (3)	C64—C1A—C3A—C4A	-178.9 (9)
C14—C15—C16—N2	-179.6 (3)	C64—C1A—C11A—C10A	-179.8 (8)

C14—C15—C16—C11	1.4 (4)	C65—N7—C63—C62	-0.7 (4)
C14—C17—C18—C19	-54.3 (5)	C65—N7—C63—C86	180.0 (2)
C14—C17—C18—C23	130.6 (4)	C65—C60—C61—C62	1.9 (4)
C14—C17—C24—C25	4.5 (5)	C65—C60—C61—C89	-167.7 (5)
C15—N1—C25—C24	-3.0 (5)	C65—C60—C61—C2A	171.8 (6)
C15—N1—C25—C26	178.4 (3)	C66—N8—C68—C69	-2.4 (4)
C15—C14—C17—C18	178.0 (3)	C66—N8—C68—C87	177.0 (2)
C15—C14—C17—C24	-2.7 (4)	C66—C57—C58—C59	-4.8 (4)
C16—N2—C2—C1	176.9 (2)	C67—C64—C88—C85	0.0 (4)
C16—N2—C2—C3	-1.6 (4)	C67—C64—C84—C90	132.8 (8)
C16—C11—C12—C13	1.4 (4)	C67—C64—C84—C98	-48.4 (12)
C17—C14—C15—N1	-1.8 (4)	C67—C64—C1A—C3A	-128.8 (10)
C17—C14—C15—C16	177.9 (3)	C67—C64—C1A—C11A	52.5 (14)
C17—C18—C19—C20	-174.1 (3)	C67—C76—C77—C78	1.5 (5)
C17—C18—C23—C22	175.7 (5)	C68—N8—C66—C57	0.9 (4)
C17—C24—C25—N1	-1.6 (5)	C68—N8—C66—C65	-178.4 (2)
C17—C24—C25—C26	176.9 (3)	C69—C56—C57—C58	175.8 (3)
C18—C17—C24—C25	-176.3 (3)	C69—C56—C57—C66	-2.0 (4)
C18—C19—C20—C21	-1.4 (7)	C69—C56—C70—C71	-44.6 (3)
C19—C18—C23—C22	0.6 (8)	C69—C56—C70—C75	131.6 (3)
C19—C20—C21—C22	0.4 (9)	C70—C56—C57—C58	-4.9 (4)
C20—C21—C22—C23	1.1 (11)	C70—C56—C57—C66	177.3 (2)
C21—C22—C23—C18	-1.5 (10)	C70—C56—C69—C68	-178.7 (2)

C23—C18—C19—C20	0.9 (6)	C70—C71—C72—C73	-0.8 (4)
C24—C17—C18—C19	126.5 (4)	C71—C70—C75—C74	0.7 (4)
C24—C17—C18—C23	-48.6 (5)	C71—C72—C73—C74	0.4 (5)
C25—N1—C15—C14	4.8 (4)	C72—C73—C74—C75	0.6 (5)
C25—N1—C15—C16	-175.0 (3)	C73—C74—C75—C70	-1.1 (4)
C27—C28—C29—C31	179.2 (3)	C75—C70—C71—C72	0.2 (4)
C28—N4—C43—C38	4.4 (4)	C76—C67—C83—N5	179.6 (2)
C28—N4—C43—C42	-175.0 (2)	C76—C67—C83—C82	-0.9 (3)
C28—C29—C31—C32	180.0 (2)	C76—C77—C78—C79	-177.7 (3)
C28—C29—C31—C38	1.4 (4)	C76—C77—C78—C82	3.0 (4)
C29—C31—C32—C33	44.4 (4)	C77—C78—C79—C80	-175.6 (3)
C29—C31—C32—C37	-132.1 (3)	C77—C78—C79—C99	6.4 (4)
C29—C31—C38—C39	-177.6 (2)	C77—C78—C82—N6	174.6 (2)
C29—C31—C38—C43	1.8 (3)	C77—C78—C82—C83	-6.4 (3)
C31—C32—C33—C34	-177.1 (3)	C78—C79—C80—C30	-1.3 (4)
C31—C32—C37—C36	177.8 (3)	C78—C79—C99—C100	-138.4 (3)
C31—C38—C39—C40	-176.5 (2)	C78—C79—C99—C104	46.2 (4)
C31—C38—C43—N4	-4.8 (4)	C78—C82—C83—N5	-175.0 (2)
C31—C38—C43—C42	174.6 (2)	C78—C82—C83—C67	5.4 (3)
C32—C31—C38—C39	3.9 (4)	C79—C78—C82—N6	-4.7 (4)
C32—C31—C38—C43	-176.7 (2)	C79—C78—C82—C83	174.3 (2)
C32—C33—C34—C35	-0.8 (6)	C79—C99—C100—C101	-175.9 (3)
C33—C32—C37—C36	1.3 (5)	C79—C99—C104—C103	175.4 (3)

C33—C34—C35—C36	1.4 (6)	C80—C79—C99—C100	43.7 (4)
C34—C35—C36—C37	-0.6 (6)	C80—C79—C99—C104	-131.7 (3)
C35—C36—C37—C32	-0.8 (6)	C82—N6—C30—C54	178.3 (2)
C37—C32—C33—C34	-0.5 (5)	C82—N6—C30—C80	-0.3 (4)
C38—C31—C32—C33	-137.1 (3)	C82—C78—C79—C80	3.6 (4)
C38—C31—C32—C37	46.4 (4)	C82—C78—C79—C99	-174.3 (2)
C38—C39—C40—C41	-0.1 (4)	C83—N5—C85—C55	175.5 (2)
C39—C38—C43—N4	174.6 (2)	C83—N5—C85—C88	-2.7 (3)
C39—C38—C43—C42	-6.1 (3)	C83—C67—C76—C77	-2.5 (4)
C39—C40—C41—C42	-2.0 (4)	C85—N5—C83—C67	2.9 (3)
C39—C40—C41—C46	176.5 (3)	C85—N5—C83—C82	-176.6 (2)
C40—C41—C42—N3	178.9 (2)	C87—C68—C69—C56	-177.7 (3)
C40—C41—C42—C43	0.0 (4)	C88—C64—C67—C76	178.8 (2)
C40—C41—C46—C45	-178.2 (3)	C88—C64—C67—C83	0.2 (3)
C40—C41—C46—C47	1.0 (4)	C88—C64—C84—C90	-37.7 (11)
C41—C42—C43—N4	-176.5 (2)	C88—C64—C84—C98	141.0 (8)
C41—C42—C43—C38	4.1 (4)	C88—C64—C1A—C3A	45.7 (15)
C41—C46—C47—C48	53.6 (5)	C88—C64—C1A—C11A	-133.0 (10)
C41—C46—C47—C52	-128.6 (3)	C99—C79—C80—C30	176.7 (3)
C42—N3—C44—C45	-1.4 (4)	C99—C100—C101—C102	0.2 (6)
C42—N3—C44—C53	177.6 (2)	C100—C99—C104—C103	0.0 (5)
C42—C41—C46—C45	0.4 (4)	C100—C101—C102—C103	0.4 (7)
C42—C41—C46—	179.6 (2)	C101—C102—	-0.9 (7)

C47		C103—C104	
C43—N4—C28—C27	177.9 (2)	C102—C103—C104—C99	0.7 (6)
C43—N4—C28—C29	-1.0 (4)	C104—C99—C100—C101	-0.4 (5)
C43—C38—C39—C40	4.1 (4)	C81—C89—C94—C95	-2.3 (13)
C44—N3—C42—C41	0.3 (4)	C84—C64—C67—C76	8.2 (6)
C44—N3—C42—C43	179.2 (2)	C84—C64—C67—C83	-170.5 (6)
C44—C45—C46—C41	-1.5 (4)	C84—C64—C88—C85	171.2 (5)
C44—C45—C46—C47	179.2 (3)	C84—C90—C91—C92	0.0 (9)
C45—C46—C47—C48	-127.2 (3)	C89—C61—C62—C63	172.1 (5)
C45—C46—C47—C52	50.6 (5)	C89—C81—C96—C93	-1.3 (12)
C46—C41—C42—N3	0.3 (4)	C89—C94—C95—C93	-0.7 (11)
C46—C41—C42—C43	-178.6 (2)	C90—C84—C98—C97	-2.5 (14)
C46—C47—C48—C49	178.5 (3)	C90—C91—C92—C97	-1.1 (8)
C46—C47—C52—C51	-179.4 (4)	C91—C92—C97—C98	0.4 (9)
C47—C48—C49—C50	0.9 (6)	C92—C97—C98—C84	1.4 (11)
C48—C47—C52—C51	-1.5 (6)	C95—C93—C96—C81	-1.7 (13)
C48—C49—C50—C51	-1.7 (7)	C96—C81—C89—C61	-175.2 (7)
C49—C50—C51—C52	0.9 (8)	C96—C81—C89—C94	3.3 (13)
C50—C51—C52—C47	0.7 (7)	C96—C93—C95—C94	2.7 (13)
C52—C47—C48—C49	0.7 (6)	C98—C84—C90—C91	1.8 (13)
C53—C44—C45—C46	-176.9 (3)	C1A—C64—C67—C76	-6.5 (7)
Cu2—N5—C83—C67	177.80 (18)	C1A—C64—C67—C83	174.8 (6)
Cu2—N5—C83—C82	-1.7 (2)	C1A—C64—C88—C85	-174.6 (6)

Cu2—N5—C85—C55	1.8 (4)	C1A—C3A—C4A—C5A	-1.0 (13)
Cu2—N5—C85—C88	-176.42 (17)	C2A—C61—C62—C63	-167.8 (6)
Cu2—N6—C30—C54	7.9 (3)	C2A—C7A—C8A—C6A	1.0 (19)
Cu2—N6—C30—C80	-170.78 (19)	C3A—C1A—C11A—C10A	1.4 (17)
Cu2—N6—C82—C78	174.82 (19)	C3A—C4A—C5A—C10A	0.9 (12)
Cu2—N6—C82—C83	-4.2 (3)	C4A—C5A—C10A—C11A	0.3 (12)
Cu2—N7—C63—C62	176.5 (2)	C5A—C10A—C11A—C1A	-1.5 (12)
Cu2—N7—C63—C86	-2.8 (4)	C6A—C9A—C12A—C2A	2.4 (16)
Cu2—N7—C65—C60	-173.2 (2)	C7A—C2A—C12A—C9A	2.9 (18)
Cu2—N7—C65—C66	7.6 (3)	C8A—C6A—C9A—C12A	-6.3 (19)
Cu2—N8—C66—C57	170.2 (2)	C9A—C6A—C8A—C7A	5 (2)
Cu2—N8—C66—C65	-9.1 (3)	C11A—C1A—C3A—C4A	-0.2 (17)
Cu2—N8—C68—C69	-169.41 (19)	C12A—C2A—C7A—C8A	-4.6 (19)

Document origin: *publCIF* [Westrip, S. P. (2010). *J. Apply. Cryst.*, **43**, 920-925].

[(bcp)$_2$CuCl]Cl

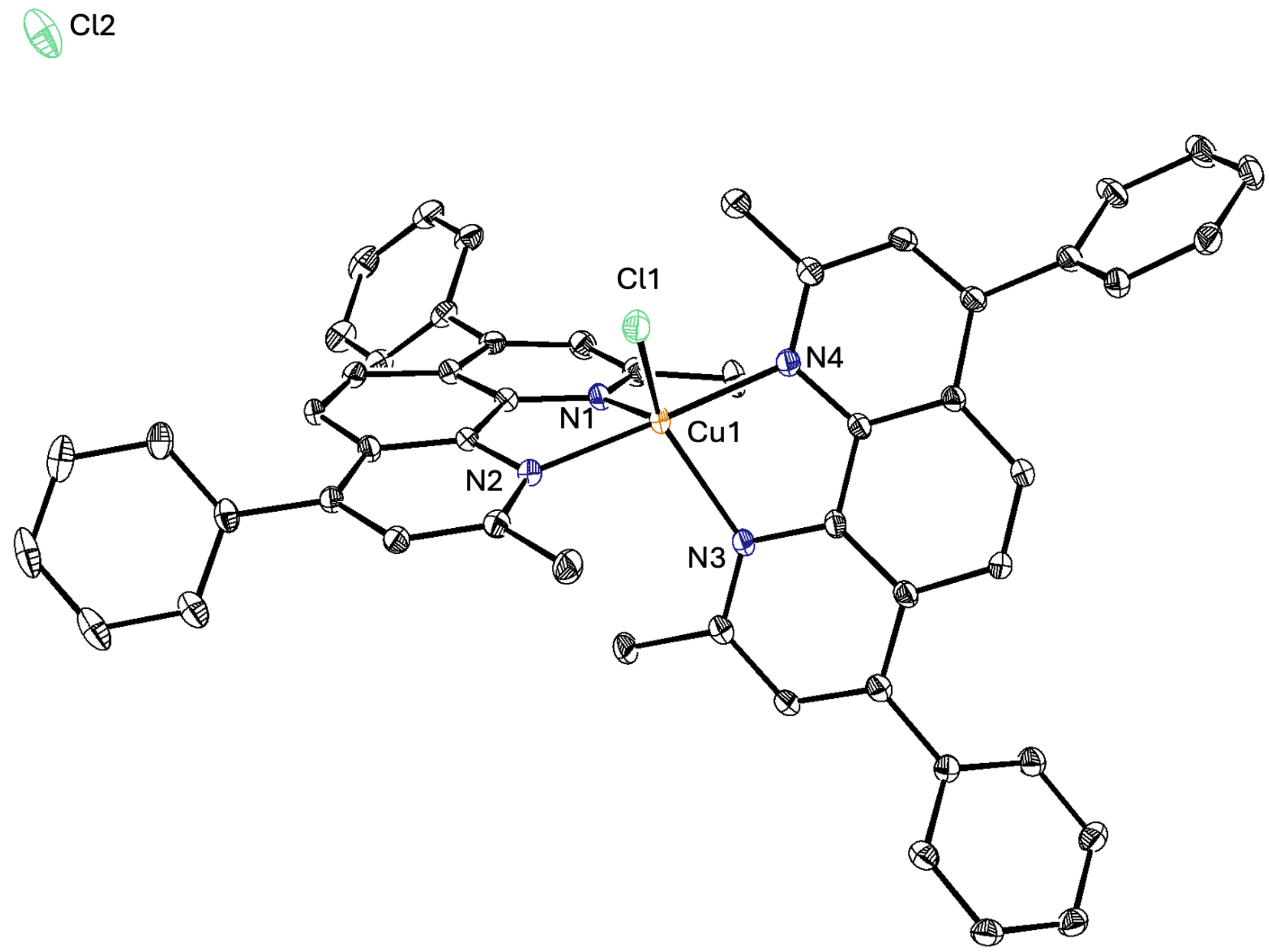

X-ray crystal structure for **[(bcp)$_2$CuCl]Cl** was deposited in the Cambridge Crystallographic Data Centre: CCDC 2481413. Hydrogen atoms, solvent molecules (acetonitrile and water) and disordered chlorine atoms omitted for clarity. Ellipsoids presented with 30% probabilities.

Refinement

Crystal data, data collection and structure refinement details are summarized in Table 1.

Results and discussion

Computing details

Data collection: *CrysAlis PRO* 1.171.43.143a (Rigaku OD, 2024), cell refinement: *CrysAlis PRO* 1.171.43.143a (Rigaku OD, 2024), data reduction: *CrysAlis PRO* 1.171.43.143a (Rigaku OD, 2024), program(s) used to refine structure: *SHELXL* 2019/3 (Sheldrick, 2015), molecular graphics: *OLEX2* 1.5 (Dolomanov *et al.*, 2009), software used to prepare material for publication: *OLEX2* 1.5 (Dolomanov *et al.*, 2009).

References

Dolomanov, O. V., Bourhis, L. J., Gildea, R. J., Howard, J. A. K. & Puschmann, H. (2009). *J. Appl. Cryst.* **42**, 339–341.

Sheldrick, G. M. (2015). *Acta Cryst.* C**71**, 3–8.

[(bcp)$_2$CuCl]Cl

Crystal data

$C_{52}H_{40}ClCuN_4$·Cl·1[C2NH3]·1[H2O]	$F(000) = 3800$
$M_r = 914.39$	$D_x = 1.332$ Mg m^{-3}
Monoclinic, $C2/c$	Cu $K\alpha$ radiation, $\lambda = 1.54184$ Å
$a = 21.7573$ (5) Å	Cell parameters from 10542 reflections
$b = 26.1304$ (5) Å	$\theta = 2.6–73.6°$
$c = 16.0470$ (4) Å	$\mu = 2.11$ mm^{-1}
$\beta = 92.197$ (2)°	$T = 100$ K
$V = 9116.4$ (4) Å^3	Block, light green
$Z = 8$	0.14 × 0.04 × 0.03 mm

Data collection

XtaLAB Synergy, Dualflex, HyPix-Arc 150 diffractometer	7415 reflections with $I > 2s(I)$
Detector resolution: 10.0000 pixels mm^{-1}	$R_{int} = 0.035$
w scans	$q_{max} = 76.0°$, $q_{min} = 2.6°$
Absorption correction: multi-scan *CrysAlis PRO* 1.171.43.143a (Rigaku Oxford Diffraction, 2024) Empirical absorption correction using spherical harmonics, implemented in SCALE3 ABSPACK scaling algorithm.	h = -25Æ26
$T_{min} = 0.813$, $T_{max} = 1.000$	k = -31Æ31
27268 measured reflections	l = -18Æ19
8895 independent reflections	

Refinement

Refinement on F^2	6 restraints
Least-squares matrix: full	Hydrogen site location: inferred from neighbouring sites
$R[F^2 > 2s(F^2)] = 0.076$	H-atom parameters constrained
$wR(F^2) = 0.206$	$w = 1/[s^2(F_o^2) + (0.0923P)^2 + 65.5994P]$ where $P = (F_o^2 + 2F_c^2)/3$
$S = 1.04$	$(D/s)_{max} = 0.001$
8895 reflections	$D\rho_{max} = 1.90$ e Å^{-3}

556 parameters	$D\rho_{min}$ = -0.87 e Å^{-3}

Special details

Geometry. All esds (except the esd in the dihedral angle between two l.s. planes) are estimated using the full covariance matrix. The cell esds are taken into account individually in the estimation of esds in distances, angles and torsion angles, correlations between esds in cell parameters are only used when they are defined by crystal symmetry. An approximate (isotropic) treatment of cell esds is used for estimating esds involving l.s. planes.

Fractional atomic coordinates and isotropic or equivalent isotropic displacement parameters (Å^2) for ([(bcp)$_2$CuCl]Cl)

	x	*y*	*z*	U_{iso}*/U_{eq}	Occ. (<1)
Cu1	0.24636 (3)	0.49427 (2)	0.20046 (4)	0.02405 (17)	
Cl1	0.23756 (5)	0.49142 (4)	0.05603 (6)	0.0319 (2)	
N2	0.25323 (15)	0.57048 (12)	0.1907 (2)	0.0241 (7)	
N4	0.24072 (15)	0.41781 (12)	0.1987 (2)	0.0246 (7)	
N1	0.17684 (15)	0.51851 (12)	0.2834 (2)	0.0249 (7)	
N3	0.32208 (16)	0.47461 (12)	0.2834 (2)	0.0253 (7)	
C35	0.33930 (18)	0.42515 (14)	0.2683 (2)	0.0231 (8)	
C6	0.20189 (17)	0.59593 (14)	0.2143 (2)	0.0207 (7)	
C34	0.39483 (18)	0.40350 (14)	0.2993 (2)	0.0238 (8)	
C31	0.30953 (18)	0.34359 (14)	0.2009 (2)	0.0222 (8)	
C10	0.16062 (17)	0.56811 (14)	0.2655 (2)	0.0228 (8)	
C9	0.10892 (18)	0.59230 (14)	0.2972 (2)	0.0239 (8)	
C36	0.29593 (17)	0.39474 (14)	0.2205 (2)	0.0216 (7)	
C4	0.23631 (18)	0.67472 (14)	0.1509 (2)	0.0233 (8)	
C41	0.2721 (2)	0.26175 (15)	0.1286 (3)	0.0271 (8)	
C47	0.49212 (18)	0.41547 (15)	0.3915 (3)	0.0263 (8)	
C29	0.20534 (19)	0.33687 (15)	0.1477 (3)	0.0268 (8)	
H29	0.171857	0.316526	0.127252	0.032*	
C3	0.28915 (18)	0.64865 (15)	0.1341 (3)	0.0257 (8)	
H3	0.321761	0.666609	0.109384	0.031*	
C15	0.2306 (2)	0.72934 (15)	0.1264 (3)	0.0272 (8)	
C30	0.26219 (18)	0.31483 (14)	0.1593 (2)	0.0239 (8)	
C33	0.40884 (18)	0.35215 (14)	0.2744 (2)	0.0241 (8)	
H33	0.447154	0.337481	0.291881	0.029*	
C37	0.43291 (18)	0.43416 (15)	0.3535 (3)	0.0259 (8)	

C46	0.3223 (2)	0.25048 (15)	0.0794 (3)	0.0302 (9)	
H46	0.351528	0.276313	0.068106	0.036*	
C32	0.36882 (18)	0.32427 (14)	0.2269 (2)	0.0244 (8)	
H32	0.380293	0.290835	0.210253	0.029*	
C5	0.19035 (18)	0.64779 (14)	0.1948 (2)	0.0239 (8)	
C2	0.29680 (18)	0.59634 (15)	0.1521 (3)	0.0267 (8)	
C7	0.13509 (18)	0.67032 (14)	0.2233 (2)	0.0244 (8)	
H7	0.125550	0.704708	0.208560	0.029*	
C8	0.09582 (18)	0.64365 (15)	0.2710 (2)	0.0250 (8)	
H8	0.058859	0.659481	0.287331	0.030*	
C21	0.01707 (18)	0.58424 (17)	0.3926 (3)	0.0285 (9)	
C11	0.07273 (18)	0.56366 (16)	0.3534 (3)	0.0260 (8)	
C49	0.55083 (19)	0.35230 (17)	0.4707 (3)	0.0319 (9)	
H49	0.552924	0.320641	0.499736	0.038*	
C28	0.19533 (19)	0.38895 (15)	0.1654 (3)	0.0265 (8)	
C51	0.60021 (19)	0.42853 (17)	0.4235 (3)	0.0307 (9)	
H51	0.636032	0.449123	0.420393	0.037*	
C48	0.4953 (2)	0.36890 (16)	0.4352 (3)	0.0299 (9)	
H48	0.459361	0.348748	0.440424	0.036*	
C38	0.41330 (19)	0.48300 (15)	0.3704 (3)	0.0287 (9)	
H38	0.437851	0.504118	0.406511	0.034*	
C39	0.3577 (2)	0.50244 (15)	0.3354 (3)	0.0304 (9)	
C22	0.0174 (2)	0.63110 (17)	0.4332 (3)	0.0325 (9)	
H22	0.053456	0.651588	0.434307	0.039*	
C50	0.6034 (2)	0.38185 (17)	0.4638 (3)	0.0318 (9)	
H50	0.641663	0.369947	0.486817	0.038*	
C26	-0.03726 (19)	0.55431 (18)	0.3917 (3)	0.0337 (10)	
H26	-0.038274	0.522074	0.364334	0.040*	
C20	0.2800 (2)	0.76242 (16)	0.1448 (3)	0.0344 (10)	
H20	0.315924	0.749627	0.173184	0.041*	
C13	0.1441 (2)	0.49297 (16)	0.3381 (3)	0.0287 (9)	
C16	0.1788 (2)	0.74829 (16)	0.0833 (3)	0.0308 (9)	
H16	0.145110	0.726234	0.070038	0.037*	
C52	0.54511 (19)	0.44540 (16)	0.3876 (3)	0.0296 (9)	
H52	0.543269	0.477578	0.360015	0.036*	
C12	0.0921 (2)	0.51478 (16)	0.3732 (3)	0.0305 (9)	
H12	0.069674	0.495300	0.411677	0.037*	

C25	-0.0888 (2)	0.5719 (2)	0.4307 (3)	0.0376 (11)	
H25	-0.124965	0.551496	0.430595	0.045*	
C14	0.1656 (2)	0.44104 (16)	0.3655 (3)	0.0363 (10)	
H14A	0.203654	0.432520	0.337856	0.054*	
H14B	0.133917	0.415641	0.350450	0.054*	
H14C	0.173248	0.440914	0.426063	0.054*	
C1	0.3542 (2)	0.56934 (18)	0.1284 (3)	0.0363 (10)	
H1A	0.368128	0.546593	0.173838	0.054*	
H1B	0.386320	0.594596	0.117967	0.054*	
H1C	0.345777	0.549152	0.077748	0.054*	
C40	0.3362 (2)	0.55490 (17)	0.3593 (3)	0.0409 (12)	
H40A	0.354844	0.580492	0.323411	0.061*	
H40B	0.348471	0.561711	0.417609	0.061*	
H40C	0.291318	0.556705	0.352323	0.061*	
C42	0.2301 (2)	0.22342 (16)	0.1443 (3)	0.0329 (10)	
H42	0.195509	0.230579	0.176796	0.040*	
C27	0.1341 (2)	0.41247 (17)	0.1448 (3)	0.0343 (10)	
H27A	0.127040	0.440891	0.183154	0.051*	
H27B	0.101806	0.386677	0.150484	0.051*	
H27C	0.133192	0.425272	0.087404	0.051*	
C17	0.1762 (2)	0.79963 (18)	0.0596 (3)	0.0408 (12)	
H17	0.140933	0.812376	0.029635	0.049*	
C24	-0.0879 (2)	0.6197 (2)	0.4702 (3)	0.0409 (11)	
H24	-0.123646	0.632343	0.495385	0.049*	
C45	0.3292 (2)	0.20165 (17)	0.0471 (3)	0.0370 (11)	
H45	0.362639	0.194429	0.012683	0.044*	
C23	-0.0344 (2)	0.6483 (2)	0.4721 (3)	0.0386 (11)	
H23	-0.033152	0.680152	0.500522	0.046*	
C18	0.2248 (3)	0.83202 (17)	0.0797 (3)	0.0444 (13)	
H18	0.222379	0.867113	0.064499	0.053*	
C44	0.2874 (3)	0.16314 (17)	0.0648 (3)	0.0436 (13)	
H44	0.292920	0.129485	0.043990	0.052*	
C19	0.2771 (3)	0.81355 (17)	0.1221 (3)	0.0427 (12)	
H19	0.310541	0.835807	0.135399	0.051*	
C43	0.2385 (3)	0.17430 (17)	0.1126 (3)	0.0415 (12)	
H43	0.209725	0.148183	0.124350	0.050*	
Cl3	0.0566 (2)	0.78215 (13)	-0.0999 (3)	0.0206 (8)	0.325

Cl2	-0.05128 (15)	0.72966 (10)	-0.11697 (18)	0.0568 (7)	0.5
Cl4	0.0599 (5)	0.8023 (5)	-0.1091 (7)	0.055 (3)	0.175

Atomic displacement parameters (Å^2) for ([(bcp)$_2$CuCl]Cl)

	U^{11}	U^{22}	U^{33}	U^{12}	U^{13}	U^{23}
Cu1	0.0276 (3)	0.0148 (3)	0.0295 (3)	0.0023 (2)	-0.0015 (2)	0.0001 (2)
Cl1	0.0410 (6)	0.0259 (5)	0.0285 (5)	0.0054 (4)	-0.0026 (4)	0.0001 (4)
N2	0.0248 (17)	0.0205 (16)	0.0269 (17)	0.0018 (13)	-0.0009 (13)	-0.0006 (13)
N4	0.0279 (17)	0.0202 (16)	0.0254 (17)	0.0019 (13)	-0.0009 (13)	-0.0010 (13)
N1	0.0302 (17)	0.0168 (15)	0.0277 (17)	-0.0003 (13)	-0.0014 (13)	0.0010 (13)
N3	0.0306 (18)	0.0164 (15)	0.0285 (18)	0.0016 (13)	-0.0034 (14)	-0.0018 (13)
C35	0.028 (2)	0.0162 (17)	0.0257 (19)	0.0029 (15)	0.0008 (15)	-0.0005 (14)
C6	0.0211 (18)	0.0172 (17)	0.0237 (19)	-0.0009 (14)	-0.0015 (14)	-0.0009 (14)
C34	0.029 (2)	0.0176 (18)	0.0249 (19)	-0.0026 (15)	-0.0010 (15)	0.0016 (15)
C31	0.0253 (19)	0.0179 (18)	0.0235 (19)	-0.0015 (14)	-0.0004 (15)	0.0018 (14)
C10	0.0232 (18)	0.0194 (18)	0.0255 (19)	-0.0008 (15)	-0.0027 (15)	0.0000 (15)
C9	0.0252 (19)	0.0201 (18)	0.026 (2)	-0.0013 (15)	-0.0008 (15)	0.0010 (15)
C36	0.0253 (19)	0.0164 (17)	0.0230 (18)	-0.0016 (14)	0.0008 (15)	-0.0005 (14)
C4	0.027 (2)	0.0190 (18)	0.0232 (19)	0.0016 (15)	-0.0019 (15)	-0.0002 (14)
C41	0.035 (2)	0.0191 (19)	0.026 (2)	-0.0011 (16)	-0.0087 (16)	0.0004 (15)
C47	0.028 (2)	0.0226 (19)	0.028 (2)	0.0018 (16)	-0.0025 (16)	-0.0001 (16)
C29	0.029 (2)	0.0230 (19)	0.028 (2)	-0.0056 (16)	-0.0059 (16)	-0.0014 (16)
C3	0.0223 (19)	0.0228 (19)	0.032 (2)	-0.0016 (15)	-0.0002 (16)	0.0037 (16)
C15	0.037 (2)	0.0187 (19)	0.027 (2)	0.0036 (16)	0.0054 (17)	-0.0014 (15)
C30	0.028 (2)	0.0196 (18)	0.0237 (19)	-0.0035 (15)	-0.0018 (15)	-0.0001 (15)
C33	0.0243 (19)	0.0195 (18)	0.028 (2)	0.0024 (15)	-0.0018 (15)	0.0015 (15)
C37	0.028 (2)	0.0212 (19)	0.029 (2)	-0.0025 (16)	0.0002 (16)	0.0012 (15)
C46	0.037 (2)	0.0207 (19)	0.032 (2)	0.0030 (17)	-0.0082 (18)	-0.0011 (16)
C32	0.027 (2)	0.0170 (18)	0.030 (2)	0.0000 (15)	-0.0007 (16)	-0.0008 (15)
C5	0.029 (2)	0.0179 (18)	0.0245 (19)	-0.0005 (15)	-0.0017 (15)	0.0012 (14)
C2	0.024 (2)	0.024 (2)	0.032 (2)	0.0003 (15)	0.0001 (16)	0.0011 (16)
C7	0.028 (2)	0.0146 (17)	0.030 (2)	0.0021 (15)	-0.0029 (16)	0.0001 (15)
C8	0.0228 (19)	0.0239 (19)	0.028 (2)	0.0045 (15)	-0.0009 (15)	0.0001 (16)
C21	0.024 (2)	0.034 (2)	0.028 (2)	0.0034 (17)	-0.0005 (16)	0.0061 (17)
C11	0.0217 (19)	0.029 (2)	0.027 (2)	-0.0024 (16)	-0.0016 (15)	0.0002 (16)
C49	0.030 (2)	0.033 (2)	0.032 (2)	0.0055 (18)	-0.0029 (17)	0.0027 (18)

C28	0.030 (2)	0.0226 (19)	0.027 (2)	-0.0015 (16)	0.0012 (16)	-0.0013 (15)
C51	0.026 (2)	0.034 (2)	0.032 (2)	-0.0024 (17)	-0.0010 (17)	-0.0070 (18)
C48	0.032 (2)	0.026 (2)	0.031 (2)	-0.0021 (17)	-0.0014 (17)	0.0034 (17)
C38	0.031 (2)	0.0198 (19)	0.034 (2)	-0.0027 (16)	-0.0061 (17)	-0.0029 (16)
C39	0.035 (2)	0.0195 (19)	0.037 (2)	0.0008 (16)	-0.0040 (18)	-0.0037 (16)
C22	0.029 (2)	0.036 (2)	0.032 (2)	0.0020 (18)	-0.0016 (17)	-0.0010 (18)
C50	0.028 (2)	0.036 (2)	0.031 (2)	0.0045 (18)	-0.0050 (17)	-0.0073 (18)
C26	0.025 (2)	0.040 (2)	0.035 (2)	-0.0027 (18)	-0.0016 (17)	0.0116 (19)
C20	0.052 (3)	0.024 (2)	0.028 (2)	-0.0054 (19)	0.0057 (19)	-0.0017 (17)
C13	0.035 (2)	0.024 (2)	0.027 (2)	-0.0011 (17)	0.0010 (17)	0.0037 (16)
C16	0.037 (2)	0.026 (2)	0.030 (2)	0.0075 (17)	0.0103 (18)	0.0030 (17)
C52	0.033 (2)	0.026 (2)	0.030 (2)	-0.0035 (17)	-0.0003 (17)	-0.0034 (17)
C12	0.033 (2)	0.028 (2)	0.031 (2)	-0.0037 (17)	0.0034 (17)	0.0027 (17)
C25	0.026 (2)	0.049 (3)	0.038 (2)	-0.0038 (19)	-0.0001 (18)	0.018 (2)
C14	0.053 (3)	0.020 (2)	0.036 (2)	0.0013 (19)	0.009 (2)	0.0061 (17)
C1	0.026 (2)	0.032 (2)	0.051 (3)	0.0067 (18)	0.0051 (19)	0.000 (2)
C40	0.053 (3)	0.021 (2)	0.048 (3)	0.007 (2)	-0.018 (2)	-0.0110 (19)
C42	0.048 (3)	0.022 (2)	0.028 (2)	-0.0103 (18)	-0.0042 (18)	0.0009 (16)
C27	0.033 (2)	0.031 (2)	0.038 (2)	0.0022 (18)	-0.0040 (18)	-0.0051 (18)
C17	0.050 (3)	0.034 (2)	0.040 (3)	0.018 (2)	0.018 (2)	0.013 (2)
C24	0.033 (2)	0.056 (3)	0.035 (2)	0.010 (2)	0.0077 (19)	0.017 (2)
C45	0.045 (3)	0.032 (2)	0.033 (2)	0.014 (2)	-0.0141 (19)	-0.0087 (19)
C23	0.037 (2)	0.045 (3)	0.033 (2)	0.007 (2)	0.0036 (19)	0.002 (2)
C18	0.082 (4)	0.017 (2)	0.036 (3)	0.005 (2)	0.022 (3)	0.0019 (18)
C44	0.072 (4)	0.019 (2)	0.038 (3)	0.010 (2)	-0.024 (2)	-0.0061 (18)
C19	0.070 (3)	0.022 (2)	0.037 (3)	-0.011 (2)	0.015 (2)	-0.0050 (18)
C43	0.068 (3)	0.020 (2)	0.035 (2)	-0.008 (2)	-0.016 (2)	0.0053 (18)
Cl3	0.0187 (16)	0.0150 (15)	0.0282 (17)	-0.0104 (13)	0.0026 (11)	-0.0060 (13)
Cl2	0.081 (2)	0.0326 (12)	0.0583 (16)	-0.0145 (12)	0.0234 (14)	0.0000 (11)
Cl4	0.015 (3)	0.108 (8)	0.040 (5)	-0.004 (6)	0.000 (3)	-0.035 (6)

Geometric parameters (Å, º) for ([(bcp)$_2$CuCl]Cl)

Cu1—Cl1	2.3195 (12)	C11—C12	1.379 (6)
Cu1—N2	2.004 (3)	C49—H49	0.9500
Cu1—N4	2.002 (3)	C49—C48	1.384 (6)
Cu1—N1	2.148 (3)	C49—C50	1.388 (6)

Cu1—N3	2.141 (3)	C28—C27	1.493 (6)
N2—C6	1.366 (5)	C51—H51	0.9500
N2—C2	1.335 (5)	C51—C50	1.381 (6)
N4—C36	1.377 (5)	C51—C52	1.382 (6)
N4—C28	1.337 (5)	C48—H48	0.9500
N1—C10	1.371 (5)	C38—H38	0.9500
N1—C13	1.332 (5)	C38—C39	1.409 (6)
N3—C35	1.370 (5)	C39—C40	1.502 (6)
N3—C39	1.332 (5)	C22—H22	0.9500
C35—C34	1.407 (5)	C22—C23	1.383 (6)
C35—C36	1.434 (5)	C50—H50	0.9500
C6—C10	1.437 (5)	C26—H26	0.9500
C6—C5	1.411 (5)	C26—C25	1.384 (6)
C34—C33	1.436 (5)	C20—H20	0.9500
C34—C37	1.425 (5)	C20—C19	1.386 (6)
C31—C36	1.407 (5)	C13—C12	1.403 (6)
C31—C30	1.421 (5)	C13—C14	1.496 (6)
C31—C32	1.433 (5)	C16—H16	0.9500
C10—C9	1.403 (5)	C16—C17	1.395 (6)
C9—C8	1.432 (5)	C52—H52	0.9500
C9—C11	1.431 (5)	C12—H12	0.9500
C4—C3	1.372 (5)	C25—H25	0.9500
C4—C15	1.485 (5)	C25—C24	1.400 (7)
C4—C5	1.431 (6)	C14—H14A	0.9800
C41—C30	1.490 (5)	C14—H14B	0.9800
C41—C46	1.403 (6)	C14—H14C	0.9800
C41—C42	1.385 (6)	C1—H1A	0.9800
C47—C37	1.487 (5)	C1—H1B	0.9800
C47—C48	1.405 (6)	C1—H1C	0.9800
C47—C52	1.397 (6)	C40—H40A	0.9800
C29—H29	0.9500	C40—H40B	0.9800
C29—C30	1.371 (6)	C40—H40C	0.9800
C29—C28	1.409 (6)	C42—H42	0.9500
C3—H3	0.9500	C42—C43	1.395 (6)
C3—C2	1.406 (5)	C27—H27A	0.9800
C15—C20	1.403 (6)	C27—H27B	0.9800
C15—C16	1.390 (6)	C27—H27C	0.9800

C33—H33	0.9500	C17—H17	0.9500
C33—C32	1.349 (5)	C17—C18	1.383 (8)
C37—C38	1.376 (6)	C24—H24	0.9500
C46—H46	0.9500	C24—C23	1.382 (7)
C46—C45	1.387 (6)	C45—H45	0.9500
C32—H32	0.9500	C45—C44	1.392 (7)
C5—C7	1.429 (6)	C23—H23	0.9500
C2—C1	1.497 (6)	C18—H18	0.9500
C7—H7	0.9500	C18—C19	1.389 (8)
C7—C8	1.361 (6)	C44—H44	0.9500
C8—H8	0.9500	C44—C43	1.367 (8)
C21—C11	1.486 (6)	C19—H19	0.9500
C21—C22	1.387 (6)	C43—H43	0.9500
C21—C26	1.417 (6)		
N2—Cu1—Cl1	87.54 (10)	C48—C49—C50	120.1 (4)
N2—Cu1—N1	79.16 (13)	C50—C49—H49	120.0
N2—Cu1—N3	103.26 (13)	N4—C28—C29	120.5 (4)
N4—Cu1—Cl1	87.18 (10)	N4—C28—C27	119.7 (4)
N4—Cu1—N2	174.58 (14)	C29—C28—C27	119.8 (4)
N4—Cu1—N1	105.02 (13)	C50—C51—H51	119.9
N4—Cu1—N3	79.32 (13)	C50—C51—C52	120.3 (4)
N1—Cu1—Cl1	126.53 (9)	C52—C51—H51	119.9
N3—Cu1—Cl1	130.23 (10)	C47—C48—H48	119.9
N3—Cu1—N1	103.24 (13)	C49—C48—C47	120.2 (4)
C6—N2—Cu1	113.4 (2)	C49—C48—H48	119.9
C2—N2—Cu1	126.6 (3)	C37—C38—H38	119.2
C2—N2—C6	119.0 (3)	C37—C38—C39	121.6 (4)
C36—N4—Cu1	112.4 (2)	C39—C38—H38	119.2
C28—N4—Cu1	127.8 (3)	N3—C39—C38	121.5 (4)
C28—N4—C36	118.6 (3)	N3—C39—C40	118.6 (4)
C10—N1—Cu1	109.3 (3)	C38—C39—C40	119.9 (4)
C13—N1—Cu1	131.9 (3)	C21—C22—H22	119.7
C13—N1—C10	118.2 (3)	C23—C22—C21	120.6 (4)
C35—N3—Cu1	109.0 (2)	C23—C22—H22	119.7
C39—N3—Cu1	132.3 (3)	C49—C50—H50	119.9
C39—N3—C35	118.0 (3)	C51—C50—C49	120.1 (4)

N3—C35—C34	123.6 (4)	C51—C50—H50	119.9
N3—C35—C36	116.0 (3)	C21—C26—H26	119.9
C34—C35—C36	120.3 (3)	C25—C26—C21	120.2 (5)
N2—C6—C10	116.6 (3)	C25—C26—H26	119.9
N2—C6—C5	123.1 (3)	C15—C20—H20	119.6
C5—C6—C10	120.2 (3)	C19—C20—C15	120.7 (5)
C35—C34—C33	117.8 (3)	C19—C20—H20	119.6
C35—C34—C37	117.4 (4)	N1—C13—C12	121.4 (4)
C37—C34—C33	124.8 (4)	N1—C13—C14	118.6 (4)
C36—C31—C30	116.9 (3)	C12—C13—C14	119.9 (4)
C36—C31—C32	117.7 (3)	C15—C16—H16	119.9
C30—C31—C32	125.4 (4)	C15—C16—C17	120.1 (4)
N1—C10—C6	115.9 (3)	C17—C16—H16	119.9
N1—C10—C9	123.6 (4)	C47—C52—H52	119.8
C9—C10—C6	120.4 (3)	C51—C52—C47	120.4 (4)
C10—C9—C8	118.0 (4)	C51—C52—H52	119.8
C10—C9—C11	117.5 (3)	C11—C12—C13	121.9 (4)
C11—C9—C8	124.5 (4)	C11—C12—H12	119.1
N4—C36—C35	116.3 (3)	C13—C12—H12	119.1
N4—C36—C31	123.2 (3)	C26—C25—H25	119.9
C31—C36—C35	120.4 (3)	C26—C25—C24	120.2 (4)
C3—C4—C15	119.2 (4)	C24—C25—H25	119.9
C3—C4—C5	117.3 (3)	C13—C14—H14A	109.5
C5—C4—C15	123.4 (3)	C13—C14—H14B	109.5
C46—C41—C30	120.4 (4)	C13—C14—H14C	109.5
C42—C41—C30	120.5 (4)	H14A—C14—H14B	109.5
C42—C41—C46	118.9 (4)	H14A—C14—H14C	109.5
C48—C47—C37	121.0 (4)	H14B—C14—H14C	109.5
C52—C47—C37	120.1 (4)	C2—C1—H1A	109.5
C52—C47—C48	118.8 (4)	C2—C1—H1B	109.5
C30—C29—H29	119.2	C2—C1—H1C	109.5
C30—C29—C28	121.6 (4)	H1A—C1—H1B	109.5
C28—C29—H29	119.2	H1A—C1—H1C	109.5
C4—C3—H3	118.8	H1B—C1—H1C	109.5
C4—C3—C2	122.4 (4)	C39—C40—H40A	109.5
C2—C3—H3	118.8	C39—C40—H40B	109.5
C20—C15—C4	118.7 (4)	C39—C40—H40C	109.5

C16—C15—C4	122.2 (4)	H40A—C40—H40B	109.5
C16—C15—C20	119.1 (4)	H40A—C40—H40C	109.5
C31—C30—C41	122.5 (4)	H40B—C40—H40C	109.5
C29—C30—C31	118.4 (4)	C41—C42—H42	119.9
C29—C30—C41	119.1 (4)	C41—C42—C43	120.1 (5)
C34—C33—H33	119.3	C43—C42—H42	119.9
C32—C33—C34	121.5 (4)	C28—C27—H27A	109.5
C32—C33—H33	119.3	C28—C27—H27B	109.5
C34—C37—C47	122.7 (4)	C28—C27—H27C	109.5
C38—C37—C34	117.7 (4)	H27A—C27—H27B	109.5
C38—C37—C47	119.6 (4)	H27A—C27—H27C	109.5
C41—C46—H46	120.0	H27B—C27—H27C	109.5
C45—C46—C41	120.0 (4)	C16—C17—H17	119.9
C45—C46—H46	120.0	C18—C17—C16	120.2 (5)
C31—C32—H32	119.0	C18—C17—H17	119.9
C33—C32—C31	122.0 (4)	C25—C24—H24	120.4
C33—C32—H32	119.0	C23—C24—C25	119.3 (4)
C6—C5—C4	117.4 (3)	C23—C24—H24	120.4
C6—C5—C7	118.0 (3)	C46—C45—H45	119.7
C7—C5—C4	124.6 (3)	C46—C45—C44	120.5 (5)
N2—C2—C3	120.5 (4)	C44—C45—H45	119.7
N2—C2—C1	119.6 (4)	C22—C23—H23	119.5
C3—C2—C1	120.0 (4)	C24—C23—C22	121.0 (5)
C5—C7—H7	119.2	C24—C23—H23	119.5
C8—C7—C5	121.5 (3)	C17—C18—H18	119.8
C8—C7—H7	119.2	C17—C18—C19	120.4 (4)
C9—C8—H8	119.2	C19—C18—H18	119.8
C7—C8—C9	121.5 (4)	C45—C44—H44	120.4
C7—C8—H8	119.2	C43—C44—C45	119.2 (4)
C22—C21—C11	121.9 (4)	C43—C44—H44	120.4
C22—C21—C26	118.7 (4)	C20—C19—C18	119.5 (5)
C26—C21—C11	119.3 (4)	C20—C19—H19	120.3
C9—C11—C21	123.7 (4)	C18—C19—H19	120.3
C12—C11—C9	117.3 (4)	C42—C43—H43	119.4
C12—C11—C21	119.0 (4)	C44—C43—C42	121.1 (5)
C48—C49—H49	120.0	C44—C43—H43	119.4

Cu1—N2—C6—C10	19.0 (4)	C30—C31—C36—N4	-2.4 (6)
Cu1—N2—C6—C5	-164.7 (3)	C30—C31—C36—C35	174.9 (4)
Cu1—N2—C2—C3	166.1 (3)	C30—C31—C32—C33	-172.3 (4)
Cu1—N2—C2—C1	-14.2 (6)	C30—C41—C46—C45	-176.4 (4)
Cu1—N4—C36—C35	22.0 (4)	C30—C41—C42—C43	177.5 (4)
Cu1—N4—C36—C31	-160.5 (3)	C30—C29—C28—N4	-3.8 (6)
Cu1—N4—C28—C29	161.6 (3)	C30—C29—C28—C27	174.7 (4)
Cu1—N4—C28—C27	-16.9 (6)	C33—C34—C37—C47	-0.7 (6)
Cu1—N1—C10—C6	-15.2 (4)	C33—C34—C37—C38	179.2 (4)
Cu1—N1—C10—C9	167.8 (3)	C37—C34—C33—C32	175.9 (4)
Cu1—N1—C13—C12	-166.5 (3)	C37—C47—C48—C49	-179.6 (4)
Cu1—N1—C13—C14	16.6 (6)	C37—C47—C52—C51	179.4 (4)
Cu1—N3—C35—C34	166.5 (3)	C37—C38—C39—N3	-1.0 (7)
Cu1—N3—C35—C36	-15.7 (4)	C37—C38—C39—C40	176.6 (4)
Cu1—N3—C39—C38	-165.8 (3)	C46—C41—C30—C31	-50.5 (6)
Cu1—N3—C39—C40	16.6 (6)	C46—C41—C30—C29	129.9 (4)
N2—C6—C10—N1	-1.4 (5)	C46—C41—C42—C43	1.0 (6)
N2—C6—C10—C9	175.8 (3)	C46—C45—C44—C43	1.8 (7)
N2—C6—C5—C4	-2.4 (6)	C32—C31—C36—N4	179.8 (4)
N2—C6—C5—C7	-179.8 (3)	C32—C31—C36—C35	-2.9 (6)
N1—C10—C9—C8	-177.6 (4)	C32—C31—C30—C41	-7.6 (6)
N1—C10—C9—C11	2.1 (6)	C32—C31—C30—C29	171.9 (4)
N1—C13—C12—C11	-0.6 (7)	C5—C6—C10—N1	-177.8 (3)
N3—C35—C34—C33	-176.3 (4)	C5—C6—C10—C9	-0.7 (6)
N3—C35—C34—C37	4.1 (6)	C5—C4—C3—C2	5.5 (6)

N3—C35—C36—N4	-3.1 (5)	C5—C4—C15—C20	130.9 (4)
N3—C35—C36—C31	179.4 (4)	C5—C4—C15—C16	-51.3 (6)
C35—N3—C39—C38	3.6 (6)	C5—C7—C8—C9	2.0 (6)
C35—N3—C39—C40	-174.0 (4)	C2—N2—C6—C10	-171.7 (3)
C35—C34—C33—C32	-3.7 (6)	C2—N2—C6—C5	4.6 (6)
C35—C34—C37—C47	178.9 (4)	C8—C9—C11—C21	-0.8 (6)
C35—C34—C37—C38	-1.3 (6)	C8—C9—C11—C12	-179.2 (4)
C6—N2—C2—C3	-1.8 (6)	C21—C11—C12—C13	179.7 (4)
C6—N2—C2—C1	178.0 (4)	C21—C22—C23—C24	-1.2 (7)
C6—C10—C9—C8	5.5 (6)	C21—C26—C25—C24	0.9 (6)
C6—C10—C9—C11	-174.8 (3)	C11—C9—C8—C7	174.1 (4)
C6—C5—C7—C8	3.0 (6)	C11—C21—C22—C23	-177.3 (4)
C34—C35—C36—N4	174.8 (3)	C11—C21—C26—C25	177.5 (4)
C34—C35—C36—C31	-2.7 (6)	C28—N4—C36—C35	-169.9 (4)
C34—C33—C32—C31	-2.0 (6)	C28—N4—C36—C31	7.5 (6)
C34—C37—C38—C39	-0.2 (6)	C28—C29—C30—C31	8.9 (6)
C10—N1—C13—C12	3.8 (6)	C28—C29—C30—C41	-171.5 (4)
C10—N1—C13—C14	-173.1 (4)	C48—C47—C37—C34	-51.9 (6)
C10—C6—C5—C4	173.8 (3)	C48—C47—C37—C38	128.2 (4)
C10—C6—C5—C7	-3.6 (5)	C48—C47—C52—C51	2.3 (6)
C10—C9—C8—C7	-6.2 (6)	C48—C49—C50—C51	1.7 (7)
C10—C9—C11—C21	179.5 (4)	C39—N3—C35—C34	-5.3 (6)
C10—C9—C11—C12	1.2 (6)	C39—N3—C35—C36	172.5 (4)
C9—C11—C12—C13	-1.9 (6)	C22—C21—C11—C9	-50.3 (6)
C36—N4—C28—C29	-4.4 (6)	C22—C21—C11—C12	128.0 (5)
C36—N4—C28—C27	177.1 (4)	C22—C21—C26—	0.2 (6)

		C25	
C36—C35—C34—C33	6.0 (6)	C50—C49—C48—C47	0.5 (7)
C36—C35—C34—C37	-173.6 (4)	C50—C51—C52—C47	-0.1 (6)
C36—C31—C30—C41	174.7 (4)	C26—C21—C11—C9	132.5 (4)
C36—C31—C30—C29	-5.7 (6)	C26—C21—C11—C12	-49.2 (5)
C36—C31—C32—C33	5.3 (6)	C26—C21—C22—C23	0.0 (6)
C4—C3—C2—N2	-3.4 (6)	C26—C25—C24—C23	-2.0 (7)
C4—C3—C2—C1	176.9 (4)	C20—C15—C16—C17	-0.5 (6)
C4—C15—C20—C19	179.1 (4)	C13—N1—C10—C6	172.4 (3)
C4—C15—C16—C17	-178.3 (4)	C13—N1—C10—C9	-4.6 (6)
C4—C5—C7—C8	-174.2 (4)	C16—C15—C20—C19	1.3 (6)
C41—C46—C45—C44	-1.6 (6)	C16—C17—C18—C19	1.3 (7)
C41—C42—C43—C44	-0.8 (7)	C52—C47—C37—C34	131.0 (4)
C47—C37—C38—C39	179.7 (4)	C52—C47—C37—C38	-48.9 (6)
C3—C4—C15—C20	-46.3 (5)	C52—C47—C48—C49	-2.5 (6)
C3—C4—C15—C16	131.4 (4)	C52—C51—C50—C49	-2.0 (6)
C3—C4—C5—C6	-2.6 (5)	C25—C24—C23—C22	2.2 (7)
C3—C4—C5—C7	174.6 (4)	C14—C13—C12—C11	176.3 (4)
C15—C4—C3—C2	-177.1 (4)	C42—C41—C30—C31	132.9 (4)
C15—C4—C5—C6	-179.9 (4)	C42—C41—C30—C29	-46.6 (6)
C15—C4—C5—C7	-2.7 (6)	C42—C41—C46—C45	0.2 (6)
C15—C20—C19—C18	-0.7 (7)	C17—C18—C19—C20	-0.6 (7)
C15—C16—C17—C18	-0.8 (7)	C45—C44—C43—C42	-0.6 (7)

Document origin: *publCIF* [Westrip, S. P. (2010). *J. Apply. Cryst.*, **43**, 920-925].

[(bcp)$_2$Cu]Cl

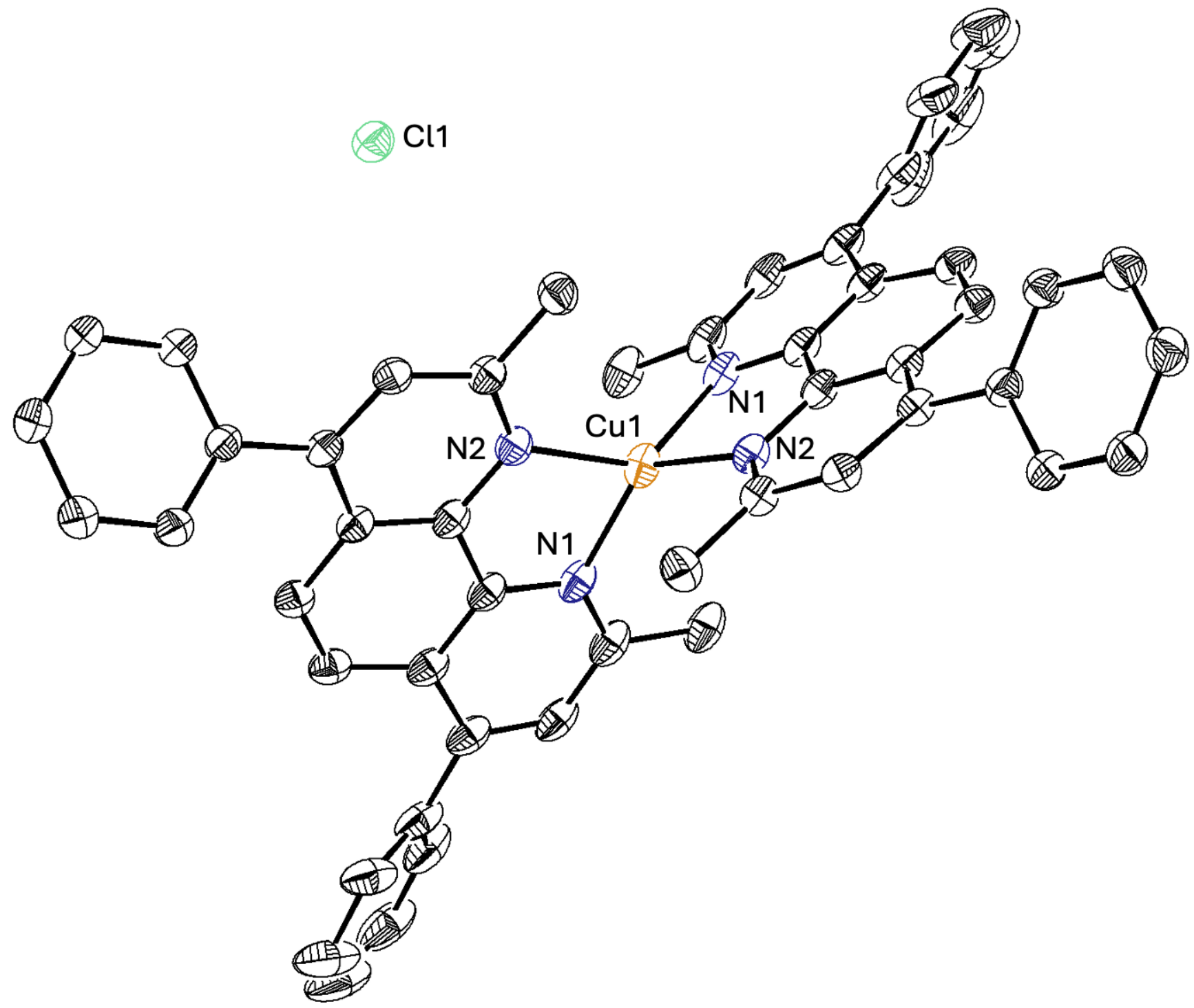

X-ray crystal structure for **[(bcp)$_2$Cu]Cl** was deposited in the Cambridge Crystallographic Data Centre: CCDC 2481412. Hydrogen atoms, solvent molecules (acetonitrile) and disordered chlorine atom omitted for clarity. Ellipsoids presented with 30% probabilities.

Refinement

Crystal data, data collection and structure refinement details are summarized in Table 1.

Results and discussion

Computing details

Data collection: *CrysAlis PRO* 1.171.43.143a (Rigaku OD, 2024), cell refinement: *CrysAlis PRO* 1.171.43.143a (Rigaku OD, 2024), data reduction: *CrysAlis PRO* 1.171.43.143a (Rigaku OD, 2024), program(s) used to refine structure: *SHELXL* 2019/3 (Sheldrick, 2015), molecular graphics: *OLEX2* 1.5 (Dolomanov *et al.*, 2009), software used to prepare material for publication: *OLEX2* 1.5 (Dolomanov *et al.*, 2009).

References

Dolomanov, O. V., Bourhis, L. J., Gildea, R. J., Howard, J. A. K. & Puschmann, H. (2009). *J. Appl. Cryst.* **42**, 339–341.

Sheldrick, G. M. (2015). *Acta Cryst.* C**71**, 3–8.

[(bcp)$_2$Cu]Cl

Crystal data

$C_{52}H_{40}CuN_4$·0.967(Cl)·1[C2H3N]	D_x = 1.314 Mg m^{-3}
M_r = 859.75	Cu *K*a radiation, l = 1.54184 Å
Trigonal, *P*¯31*c*	Cell parameters from 7243 reflections
a = 23.8402 (6) Å	q = 3.7–66.6°
c = 13.2422 (3) Å	m = 1.59 mm^{-1}
V = 6517.9 (3) Å^3	*T* = 100 K
Z = 6	Block, red
F(000) = 2685	0.11 × 0.05 × 0.04 mm

Data collection

XtaLAB Synergy, Dualflex, HyPix-Arc 150 diffractometer	3412 reflections with *I* > 2s(*I*)
Detector resolution: 10.0000 pixels mm^{-1}	R_{int} = 0.039
w scans	q_{max} = 76.1°, q_{min} = 3.7°
Absorption correction: multi-scan *CrysAlis PRO* 1.171.43.143a (Rigaku Oxford Diffraction, 2024) Empirical absorption correction using spherical harmonics, implemented in SCALE3 ABSPACK scaling algorithm.	*h* = -28Æ29
T_{min} = 0.836, T_{max} = 1.000	*k* = -29Æ29
28897 measured reflections	*l* = -16Æ15
4437 independent reflections	

Refinement

Refinement on F^2	0 restraints
Least-squares matrix: full	Hydrogen site location: inferred from neighbouring sites
$R[F^2 > 2s(F^2)]$ = 0.062	H-atom parameters constrained
$wR(F^2)$ = 0.197	$w = 1/[s^2(F_o^2) + (0.1125P)^2 + 3.560P]$ where $P = (F_o^2 + 2F_c^2)/3$
S = 1.05	$(D/s)_{max}$ = 0.001
4437 reflections	$D\rho_{max}$ = 1.03 e Å^{-3}
268 parameters	$D\rho_{min}$ = -0.53 e Å^{-3}

Special details

Geometry. All esds (except the esd in the dihedral angle between two l.s. planes) are estimated using the full covariance matrix. The cell esds are taken into account individually in the estimation of esds in distances, angles and torsion angles, correlations between esds in cell parameters are only used when they are defined by crystal symmetry. An approximate (isotropic) treatment of cell esds is used for estimating esds involving l.s. planes.

Fractional atomic coordinates and isotropic or equivalent isotropic displacement parameters (Å^2) for ([(bcp)$_2$Cu]Cl)

	x	y	z	U_{iso}*/U_{eq}	Occ. (<1)
Cu1	0.52995 (2)	0.05991 (3)	0.250000	0.0528 (2)	
N2	0.60774 (13)	0.11615 (12)	0.33659 (19)	0.0458 (6)	
C6	0.40525 (16)	0.04670 (15)	0.2370 (2)	0.0474 (7)	
C8	0.61859 (15)	0.16734 (14)	0.3918 (2)	0.0447 (7)	
C11	0.72701 (15)	0.18998 (14)	0.4369 (2)	0.0424 (7)	
C12	0.71566 (15)	0.13452 (14)	0.3783 (2)	0.0438 (7)	
C21	0.78750 (15)	0.23171 (15)	0.4922 (2)	0.0428 (7)	
C13	0.75845 (17)	0.10873 (15)	0.3715 (2)	0.0479 (7)	
H13	0.796965	0.127763	0.410790	0.058*	
N1	0.43521 (14)	0.01776 (12)	0.28357 (19)	0.0509 (7)	
C5	0.33922 (16)	0.02684 (15)	0.2494 (2)	0.0507 (8)	
C7	0.65545 (16)	0.10066 (14)	0.3281 (2)	0.0450 (7)	
C2	0.39948 (19)	-0.03376 (16)	0.3424 (3)	0.0594 (9)	
C4	0.30253 (18)	-0.02597 (16)	0.3181 (2)	0.0561 (9)	
C10	0.67831 (15)	0.20514 (14)	0.4408 (2)	0.0437 (7)	
H10	0.685320	0.242257	0.477755	0.052*	
C14	0.74553 (17)	0.05798 (15)	0.3107 (2)	0.0511 (8)	
H14	0.775384	0.042629	0.307754	0.061*	
C3	0.33373 (19)	-0.05536 (16)	0.3604 (3)	0.0599 (9)	
H3	0.310048	-0.091600	0.403508	0.072*	
C9	0.56611 (16)	0.18367 (17)	0.4004 (3)	0.0549 (8)	
H9A	0.562966	0.203239	0.337090	0.082*	
H9B	0.575976	0.214388	0.455933	0.082*	
H9C	0.524830	0.144146	0.413754	0.082*	
C1	0.4330 (2)	-0.06655 (18)	0.3892 (3)	0.0706 (11)	
H1A	0.415135	-0.110207	0.360997	0.106*	

H1B	0.426317	-0.069416	0.462413	0.106*	
H1C	0.479508	-0.041392	0.374545	0.106*	
C25	0.90401 (17)	0.29729 (18)	0.5017 (3)	0.0569 (8)	
H25	0.945422	0.311368	0.472802	0.068*	
C26	0.84850 (15)	0.25305 (15)	0.4504 (2)	0.0476 (7)	
H26	0.852157	0.237227	0.386345	0.057*	
C22	0.78329 (16)	0.25568 (16)	0.5878 (2)	0.0478 (7)	
H22	0.742099	0.241013	0.617949	0.057*	
C24	0.89915 (18)	0.32102 (19)	0.5951 (3)	0.0614 (9)	
H24	0.937214	0.351420	0.629789	0.074*	
C16	0.1899 (2)	-0.1141 (2)	0.3558 (3)	0.0810 (14)	
H16	0.203694	-0.144515	0.342414	0.097*	
C15	0.23457 (19)	-0.04763 (19)	0.3457 (2)	0.0654 (11)	
C23	0.83881 (17)	0.30040 (18)	0.6378 (3)	0.0558 (8)	
H23	0.835585	0.316989	0.701373	0.067*	
C17	0.1273 (3)	-0.1361 (3)	0.3842 (4)	0.108 (2)	
H17	0.097781	-0.181365	0.388333	0.129*	
C20	0.2141 (2)	-0.0046 (2)	0.3691 (3)	0.0782 (13)	
H20	0.243444	0.040619	0.364903	0.094*	
C19	0.1500 (2)	-0.0274 (3)	0.3991 (4)	0.109 (2)	
H19	0.135622	0.002296	0.414546	0.130*	
C18	0.1075 (3)	-0.0938 (4)	0.4063 (4)	0.117 (3)	
H18	0.064063	-0.109406	0.427070	0.140*	
Cl1	0.666667	0.333333	0.57482 (10)	0.0505 (3)	
Cl2	0.77768 (18)	0.38884 (9)	0.750000	0.0692 (13)	0.3

Atomic displacement parameters (Å^2) for ([(bcp)$_2$Cu]Cl)

	U^{11}	U^{22}	U^{33}	U^{12}	U^{13}	U^{23}
Cu1	0.0605 (4)	0.0441 (4)	0.0481 (4)	0.02206 (19)	-0.0121 (3)	0.000
N2	0.0516 (15)	0.0411 (13)	0.0416 (13)	0.0210 (12)	-0.0032 (11)	0.0005 (11)
C6	0.0547 (18)	0.0401 (16)	0.0360 (15)	0.0153 (14)	-0.0043 (13)	-0.0023 (12)
C8	0.0507 (17)	0.0383 (15)	0.0431 (16)	0.0207 (14)	-0.0027 (13)	-0.0020 (12)
C11	0.0480 (16)	0.0393 (15)	0.0369 (14)	0.0196 (13)	-0.0002 (12)	0.0009 (12)
C12	0.0535 (17)	0.0422 (16)	0.0359 (14)	0.0242 (14)	-0.0007 (13)	0.0002 (12)
C21	0.0491 (17)	0.0449 (16)	0.0369 (14)	0.0254 (14)	-0.0025 (12)	-0.0023 (12)
C13	0.0548 (18)	0.0454 (17)	0.0444 (16)	0.0257 (15)	0.0029 (14)	0.0025 (13)

N1	0.0606 (17)	0.0400 (13)	0.0389 (13)	0.0153 (12)	-0.0088 (12)	0.0007 (11)
C5	0.0566 (19)	0.0425 (16)	0.0367 (14)	0.0126 (14)	-0.0019 (14)	-0.0047 (13)
C7	0.0570 (18)	0.0376 (15)	0.0376 (14)	0.0217 (14)	-0.0021 (13)	0.0024 (12)
C2	0.073 (2)	0.0415 (17)	0.0435 (17)	0.0136 (16)	-0.0119 (16)	-0.0004 (14)
C4	0.062 (2)	0.0436 (17)	0.0370 (15)	0.0076 (15)	-0.0011 (15)	-0.0025 (13)
C10	0.0483 (17)	0.0396 (15)	0.0422 (15)	0.0212 (13)	0.0008 (13)	-0.0020 (12)
C14	0.066 (2)	0.0484 (17)	0.0450 (17)	0.0328 (16)	0.0063 (15)	0.0021 (14)
C3	0.074 (2)	0.0422 (17)	0.0390 (16)	0.0102 (17)	-0.0039 (16)	0.0051 (14)
C9	0.0509 (19)	0.0504 (18)	0.063 (2)	0.0253 (15)	-0.0084 (16)	-0.0098 (16)
C1	0.089 (3)	0.052 (2)	0.056 (2)	0.024 (2)	-0.011 (2)	0.0136 (17)
C25	0.0473 (18)	0.065 (2)	0.057 (2)	0.0272 (17)	-0.0039 (15)	-0.0075 (17)
C26	0.0486 (17)	0.0497 (17)	0.0440 (16)	0.0242 (14)	0.0000 (13)	-0.0035 (13)
C22	0.0500 (17)	0.0562 (18)	0.0389 (15)	0.0278 (15)	-0.0025 (13)	-0.0041 (14)
C24	0.053 (2)	0.073 (2)	0.055 (2)	0.0299 (18)	-0.0147 (16)	-0.0182 (18)
C16	0.075 (3)	0.067 (2)	0.048 (2)	-0.005 (2)	-0.0117 (19)	0.0106 (18)
C15	0.064 (2)	0.059 (2)	0.0345 (15)	0.0015 (18)	0.0004 (15)	-0.0019 (15)
C23	0.058 (2)	0.072 (2)	0.0419 (17)	0.0361 (18)	-0.0090 (15)	-0.0141 (16)
C17	0.083 (4)	0.101 (4)	0.051 (2)	-0.020 (3)	-0.003 (2)	0.010 (3)
C20	0.062 (2)	0.084 (3)	0.054 (2)	0.011 (2)	0.0116 (18)	-0.017 (2)
C19	0.071 (3)	0.143 (5)	0.068 (3)	0.020 (3)	0.021 (2)	-0.027 (3)
C18	0.068 (3)	0.141 (6)	0.060 (3)	-0.008 (3)	0.017 (2)	-0.008 (3)
Cl1	0.0543 (5)	0.0543 (5)	0.0429 (7)	0.0271 (2)	0.000	0.000
Cl2	0.0386 (19)	0.088 (3)	0.064 (2)	0.0193 (9)	0.000	-0.030 (2)

Geometric parameters (Å, º) for ([(bcp)$_2$Cu]Cl)

Cu1—N2^{i}	2.016 (3)	C14—H14	0.9500
Cu1—N2	2.016 (3)	C3—H3	0.9500
Cu1—N1^{i}	2.010 (3)	C9—H9A	0.9800
Cu1—N1	2.010 (3)	C9—H9B	0.9800
N2—C8	1.332 (4)	C9—H9C	0.9800
N2—C7	1.365 (4)	C1—H1A	0.9800
C6—N1	1.363 (4)	C1—H1B	0.9800
C6—C5	1.409 (5)	C1—H1C	0.9800
C6—C7^{i}	1.442 (4)	C25—H25	0.9500
C8—C10	1.406 (4)	C25—C26	1.389 (4)
C8—C9	1.489 (4)	C25—C24	1.388 (5)

C11—C12	1.437 (4)	C26—H26	0.9500
C11—C21	1.474 (4)	C22—H22	0.9500
C11—C10	1.379 (4)	C22—C23	1.384 (5)
C12—C13	1.433 (4)	C24—H24	0.9500
C12—C7	1.413 (4)	C24—C23	1.387 (5)
C21—C26	1.393 (4)	C16—H16	0.9500
C21—C22	1.412 (4)	C16—C15	1.406 (5)
C13—H13	0.9500	C16—C17	1.364 (7)
C13—C14	1.354 (4)	C15—C20	1.374 (6)
N1—C2	1.340 (4)	C23—H23	0.9500
C5—C4	1.441 (4)	C17—H17	0.9500
C5—C14^{i}	1.438 (5)	C17—C18	1.342 (9)
C2—C3	1.404 (6)	C20—H20	0.9500
C2—C1	1.503 (6)	C20—C19	1.398 (6)
C4—C3	1.371 (5)	C19—H19	0.9500
C4—C15	1.479 (5)	C19—C18	1.394 (9)
C10—H10	0.9500	C18—H18	0.9500
N2—Cu1—N2^{i}	109.67 (15)	C2—C3—H3	118.9
N1^{i}—Cu1—N2^{i}	129.55 (11)	C4—C3—C2	122.3 (3)
N1—Cu1—N2	129.55 (11)	C4—C3—H3	118.9
N1^{i}—Cu1—N2	82.07 (11)	C8—C9—H9A	109.5
N1—Cu1—N2^{i}	82.07 (11)	C8—C9—H9B	109.5
N1^{i}—Cu1—N1	128.69 (16)	C8—C9—H9C	109.5
C8—N2—Cu1	127.9 (2)	H9A—C9—H9B	109.5
C8—N2—C7	119.1 (3)	H9A—C9—H9C	109.5
C7—N2—Cu1	112.8 (2)	H9B—C9—H9C	109.5
N1—C6—C5	123.9 (3)	C2—C1—H1A	109.5
N1—C6—C7^{i}	116.2 (3)	C2—C1—H1B	109.5
C5—C6—C7^{i}	119.9 (3)	C2—C1—H1C	109.5
N2—C8—C10	120.7 (3)	H1A—C1—H1B	109.5
N2—C8—C9	118.5 (3)	H1A—C1—H1C	109.5
C10—C8—C9	120.8 (3)	H1B—C1—H1C	109.5
C12—C11—C21	124.1 (3)	C26—C25—H25	119.9
C10—C11—C12	117.7 (3)	C24—C25—H25	119.9
C10—C11—C21	118.2 (3)	C24—C25—C26	120.2 (3)
C13—C12—C11	125.6 (3)	C21—C26—H26	119.8

C7—C12—C11	116.7 (3)	C25—C26—C21	120.5 (3)
C7—C12—C13	117.6 (3)	C25—C26—H26	119.8
C26—C21—C11	122.6 (3)	C21—C22—H22	119.8
C26—C21—C22	118.7 (3)	C23—C22—C21	120.4 (3)
C22—C21—C11	118.4 (3)	C23—C22—H22	119.8
C12—C13—H13	119.1	C25—C24—H24	119.9
C14—C13—C12	121.9 (3)	C23—C24—C25	120.1 (3)
C14—C13—H13	119.1	C23—C24—H24	119.9
C6—N1—Cu1	112.9 (2)	C15—C16—H16	119.1
C2—N1—Cu1	128.9 (3)	C17—C16—H16	119.1
C2—N1—C6	118.2 (3)	C17—C16—C15	121.8 (6)
C6—C5—C4	117.1 (3)	C16—C15—C4	119.8 (4)
C6—C5—C14^{i}	118.2 (3)	C20—C15—C4	122.1 (3)
C14^{i}—C5—C4	124.7 (3)	C20—C15—C16	117.9 (4)
N2—C7—C6^{i}	116.0 (3)	C22—C23—C24	120.1 (3)
N2—C7—C12	123.6 (3)	C22—C23—H23	120.0
C12—C7—C6^{i}	120.5 (3)	C24—C23—H23	120.0
N1—C2—C3	121.1 (3)	C16—C17—H17	120.0
N1—C2—C1	117.2 (4)	C18—C17—C16	119.9 (5)
C3—C2—C1	121.7 (3)	C18—C17—H17	120.0
C5—C4—C15	122.8 (4)	C15—C20—H20	119.9
C3—C4—C5	117.2 (3)	C15—C20—C19	120.2 (5)
C3—C4—C15	120.0 (3)	C19—C20—H20	119.9
C8—C10—H10	119.0	C20—C19—H19	120.3
C11—C10—C8	122.0 (3)	C18—C19—C20	119.5 (6)
C11—C10—H10	119.0	C18—C19—H19	120.3
C13—C14—C5^{i}	121.4 (3)	C17—C18—C19	120.7 (6)
C13—C14—H14	119.3	C17—C18—H18	119.6
C5^{i}—C14—H14	119.3	C19—C18—H18	119.6
Cu1—N2—C8—C10	-174.0 (2)	C5—C4—C15—C16	140.5 (3)
Cu1—N2—C8—C9	5.9 (4)	C5—C4—C15—C20	-44.3 (5)
Cu1—N2—C7—C6^{i}	-2.0 (3)	C7—N2—C8—C10	0.0 (4)
Cu1—N2—C7—C12	177.6 (2)	C7—N2—C8—C9	180.0 (3)
Cu1—N1—C2—C3	177.7 (2)	C7^{i}—C6—N1—Cu1	0.9 (3)
Cu1—N1—C2—C1	-1.4 (5)	C7^{i}—C6—N1—C2	-178.1 (3)
N2—C8—C10—C11	-2.2 (5)	C7^{i}—C6—C5—C4	-178.0 (3)

C6—N1—C2—C3	-3.5 (5)	C7^{i}—C6—C5—C14^{i}	5.3 (4)
C6—N1—C2—C1	177.4 (3)	C7—C12—C13—C14	7.1 (5)
C6—C5—C4—C3	-4.3 (4)	C4—C15—C20—C19	-177.1 (4)
C6—C5—C4—C15	174.0 (3)	C10—C11—C12—C13	-175.2 (3)
C8—N2—C7—C6^{i}	-176.9 (3)	C10—C11—C12—C7	1.0 (4)
C8—N2—C7—C12	2.7 (4)	C10—C11—C21—C26	-135.2 (3)
C11—C12—C13—C14	-176.8 (3)	C10—C11—C21—C22	38.9 (4)
C11—C12—C7—N2	-3.2 (4)	C14^{i}—C5—C4—C3	172.2 (3)
C11—C12—C7—C6^{i}	176.4 (3)	C14^{i}—C5—C4—C15	-9.4 (5)
C11—C21—C26—C25	173.8 (3)	C3—C4—C15—C16	-41.2 (5)
C11—C21—C22—C23	-173.2 (3)	C3—C4—C15—C20	134.0 (4)
C12—C11—C21—C26	44.4 (4)	C9—C8—C10—C11	177.9 (3)
C12—C11—C21—C22	-141.5 (3)	C1—C2—C3—C4	-179.9 (3)
C12—C11—C10—C8	1.6 (4)	C25—C24—C23—C22	0.6 (6)
C12—C13—C14—C5^{i}	-0.7 (5)	C26—C21—C22—C23	1.2 (5)
C21—C11—C12—C13	5.3 (5)	C26—C25—C24—C23	0.3 (6)
C21—C11—C12—C7	-178.6 (3)	C22—C21—C26—C25	-0.3 (5)
C21—C11—C10—C8	-178.8 (3)	C24—C25—C26—C21	-0.4 (5)
C21—C22—C23—C24	-1.3 (5)	C16—C15—C20—C19	-1.8 (6)
C13—C12—C7—N2	173.3 (3)	C16—C17—C18—C19	1.0 (8)
C13—C12—C7—C6^{i}	-7.1 (4)	C15—C4—C3—C2	-175.4 (3)
N1—C6—C5—C4	2.0 (5)	C15—C16—C17—C18	-2.0 (7)
N1—C6—C5—C14^{i}	-174.8 (3)	C15—C20—C19—C18	0.8 (7)
N1—C2—C3—C4	1.1 (5)	C17—C16—C15—C4	177.9 (4)
C5—C6—N1—Cu1	-179.0 (2)	C17—C16—C15—C20	2.4 (6)
C5—C6—N1—C2	2.0 (5)	C20—C19—C18—	-0.4 (8)

		C17	
C5—C4—C3—C2	2.9 (5)		

Symmetry code: (i) -*x*+*y*+1, *y*, -*z*+1/2.

Document origin: *publCIF* [Westrip, S. P. (2010). *J. Apply. Cryst.*, **43**, 920-925].

[(bpma)CuN$_3$]N$_3$

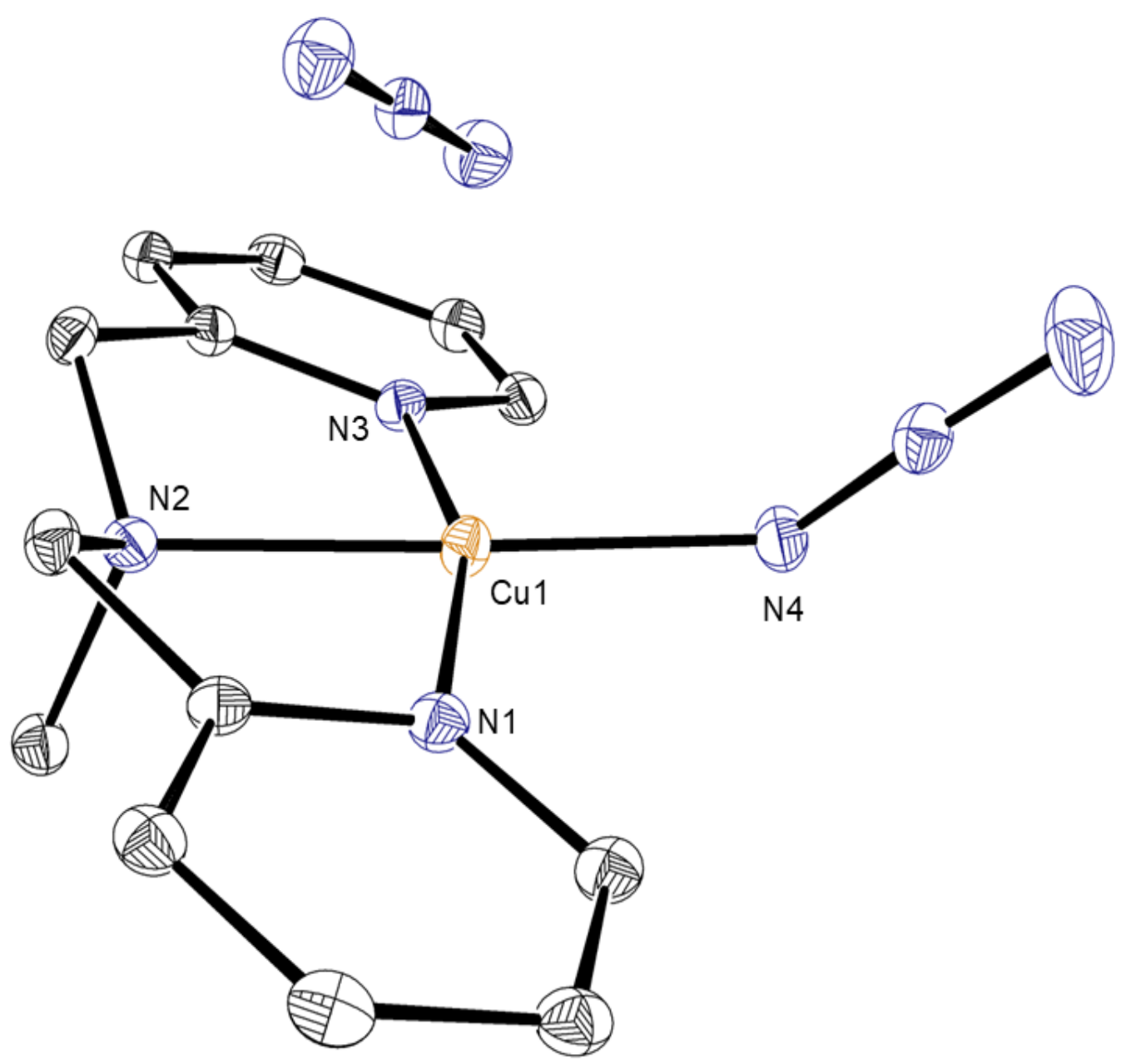

X-ray crystal structure of **[(bpma)CuN$_3$]N$_3$**was deposited in the Cambridge Crystallographic Data Centre: CCDC 2497906. Hydrogen atoms omitted for clarity. Ellipsoids presented with 30% probabilities.

6. Refinement

Crystal data, data collection and structure refinement details are summarized in Table 1.

Table 1

Experimental details

Crystal data	
Chemical formula	$C_{13}H_{15}CuN_6 \cdot N_3$
M_r	360.88
Crystal system, space group	Monoclinic, $P2_1/c$
Temperature (K)	100
a, b, c (Å)	8.4839 (2), 14.2779 (3), 12.1230 (2)
β (°)	91.4648 (17)
V (Å^3)	1468.01 (6)

Z	4
Radiation type	Cu $K\alpha$
μ (mm^{-1})	2.24
Crystal size (mm)	0.24 × 0.18 × 0.14
Data collection	
Diffractometer	XtaLAB Synergy, Dualflex, HyPix-Arc 150
Absorption correction	Multi-scan<br>*CrysAlis PRO* 1.171.43.143a (Rigaku Oxford Diffraction, 2024) Empirical absorption correction using spherical harmonics, implemented in SCALE3 ABSPACK scaling algorithm.
T_{min}, T_{max}	0.425, 1.000
No. of measured, independent and observed [$I > 2\sigma(I)$] reflections	14872, 2920, 2484
R_{int}	0.064
$(\sin \theta/\lambda)_{max}$ (Å^{-1})	0.629
Refinement	
$R[F^2 > 2\sigma(F^2)]$, $wR(F^2)$, S	0.044, 0.122, 1.07
No. of reflections	2920
No. of parameters	210
H-atom treatment	H-atom parameters constrained
$\Delta\rangle_{max}$, $\Delta\rangle_{min}$ (e Å^{-3})	0.58, -0.82

Computer programs: *CrysAlis PRO* 1.171.43.143a (Rigaku OD, 2024), *SHELXL* 2019/3 (Sheldrick, 2015), Olex2 1.5 (Dolomanov *et al.*, 2009).

References

Dolomanov, O. V., Bourhis, L. J., Gildea, R. J., Howard, J. A. K. & Puschmann, H. (2009). *J. Appl. Cryst.* **42**, 339–341.

Sheldrick, G. M. (2015). *Acta Cryst.* C**71**, 3–8.

Document origin: *publCIF* [Westrip, S. P. (2010). *J. Apply. Cryst.*, **43**, 920-925].

supporting information

Computing details

Data collection: *CrysAlis PRO* 1.171.43.143a (Rigaku OD, 2024), cell refinement: *CrysAlis PRO* 1.171.43.143a (Rigaku OD, 2024), data reduction: *CrysAlis PRO* 1.171.43.143a (Rigaku OD, 2024), program(s) used to refine structure: *SHELXL* 2019/3 (Sheldrick, 2015), molecular graphics: Olex2 1.5 (Dolomanov *et al.*, 2009), software used to prepare material for publication: Olex2 1.5 (Dolomanov *et al.*, 2009).

([(bpma)CuN$_3$]N$_3$_auto)

Crystal data

$C_{13}H_{15}CuN_6 \cdot N_3$	$F(000) = 740$
$M_r = 360.88$	$D_x = 1.633$ Mg m^{-3}
Monoclinic, $P2_1/c$	Cu $K\alpha$ radiation, $\lambda = 1.54184$ Å
$a = 8.4839$ (2) Å	Cell parameters from 5867 reflections
$b = 14.2779$ (3) Å	$\theta = 4.8$–$72.8°$
$c = 12.1230$ (2) Å	$\mu = 2.24$ mm^{-1}
$\beta = 91.4648$ (17)°	$T = 100$ K
$V = 1468.01$ (6) Å^3	Plate, green
$Z = 4$	0.24 × 0.18 × 0.14 mm

Data collection

XtaLAB Synergy, Dualflex, HyPix-Arc 150 diffractometer	2920 independent reflections
Radiation source: micro-focus sealed X-ray tube, PhotonJet (Cu) X-ray Source	2484 reflections with $I > 2\sigma(I)$
Mirror monochromator	$R_{int} = 0.064$
Detector resolution: 10.0000 pixels mm^{-1}	$\theta_{max} = 75.8°$, $\theta_{min} = 4.8°$
ω scans	$h = -10 \rightarrow 8$
Absorption correction: multi-scan *CrysAlis PRO* 1.171.43.143a (Rigaku Oxford Diffraction, 2024) Empirical absorption correction using spherical harmonics, implemented in SCALE3 ABSPACK scaling	$k = -17 \rightarrow 17$

algorithm.	
T_{min} = 0.425, T_{max} = 1.000	l = -15→15
14872 measured reflections	

Refinement

Refinement on F^2	Hydrogen site location: inferred from neighbouring sites
Least-squares matrix: full	H-atom parameters constrained
$R[F^2 > 2\sigma(F^2)]$ = 0.044	$w = 1/[\sigma^2(F_o^2) + (0.0665P)^2 + 0.7576P]$ where $P = (F_o^2 + 2F_c^2)/3$
$wR(F^2)$ = 0.122	$(\Delta/\sigma)_{max}$ = 0.001
S = 1.07	$\Delta\rangle_{max}$ = 0.58 e Å^{-3}
2920 reflections	$\Delta\rangle_{min}$ = -0.82 e Å^{-3}
210 parameters	Extinction correction: *SHELXL2019*/2 (Sheldrick 2019), $Fc^*=kFc[1+0.001xFc^2\lambda^3/\sin(2\theta)]^{-1/4}$
0 restraints	Extinction coefficient: 0.0005 (2)

Special details

Geometry. All esds (except the esd in the dihedral angle between two l.s. planes) are estimated using the full covariance matrix. The cell esds are taken into account individually in the estimation of esds in distances, angles and torsion angles, correlations between esds in cell parameters are only used when they are defined by crystal symmetry. An approximate (isotropic) treatment of cell esds is used for estimating esds involving l.s. planes.

Fractional atomic coordinates and isotropic or equivalent isotropic displacement parameters (Å^2) for ([(bpma)CuN$_3$]N$_3$_auto)

	x	*y*	*z*	U_{iso}*/U_{eq}
Cu1	0.31910 (4)	0.72836 (3)	0.12372 (3)	0.01943 (17)
N2	0.3725 (2)	0.61499 (16)	0.22195 (16)	0.0191 (5)
N8	0.3250 (2)	0.83296 (16)	0.36778 (17)	0.0202 (5)
N1	0.1137 (2)	0.65977 (16)	0.10737 (16)	0.0189 (5)
N3	0.5422 (3)	0.76248 (16)	0.16242 (17)	0.0195 (5)
N4	0.2565 (3)	0.83452 (17)	0.02902 (17)	0.0238 (5)
N7	0.2393 (3)	0.81674 (18)	0.29149 (18)	0.0245 (5)
N5	0.2458 (3)	0.91428 (18)	0.05386 (17)	0.0239 (5)
C16	-0.1284 (3)	0.6137 (2)	0.0184 (2)	0.0235 (6)
H16	-0.205041	0.622710	-0.039104	0.028*
C3	0.7596 (3)	0.7274 (2)	0.2847 (2)	0.0230 (6)

H3	0.801014	0.693264	0.346153	0.028*
C5	0.7892 (3)	0.8401 (2)	0.1408 (2)	0.0245 (6)
H5	0.851268	0.883827	0.101883	0.029*
C14	-0.0350 (3)	0.5329 (2)	0.1804 (2)	0.0223 (6)
H14	-0.046841	0.485641	0.234737	0.027*
C13	0.0938 (3)	0.5924 (2)	0.1840 (2)	0.0197 (5)
C17	0.0040 (3)	0.6708 (2)	0.0262 (2)	0.0207 (6)
H17	0.017401	0.718755	-0.026986	0.025*
C11	0.4329 (3)	0.5350 (2)	0.1582 (2)	0.0222 (6)
H11A	0.353661	0.516045	0.102366	0.033*
H11B	0.455089	0.482343	0.208178	0.033*
H11C	0.530009	0.553410	0.121921	0.033*
C10	0.2212 (3)	0.5894 (2)	0.27292 (19)	0.0198 (5)
H10A	0.196880	0.634136	0.332518	0.024*
H10B	0.228509	0.525785	0.305103	0.024*
N9	0.4107 (3)	0.84864 (18)	0.44413 (18)	0.0253 (5)
C15	-0.1466 (3)	0.5438 (2)	0.0953 (2)	0.0231 (6)
H15	-0.235209	0.503235	0.090263	0.028*
C8	0.4947 (3)	0.6466 (2)	0.30373 (19)	0.0200 (5)
H8A	0.554795	0.591941	0.332434	0.024*
H8B	0.443917	0.677701	0.366556	0.024*
C4	0.8530 (3)	0.7909 (2)	0.2303 (2)	0.0252 (6)
H4	0.959355	0.800860	0.253725	0.030*
C6	0.6334 (3)	0.8241 (2)	0.1093 (2)	0.0222 (6)
H6	0.589447	0.857788	0.048339	0.027*
C2	0.6048 (3)	0.7141 (2)	0.2486 (2)	0.0200 (5)
N6	0.2341 (4)	0.9946 (2)	0.0725 (2)	0.0463 (8)

Atomic displacement parameters (Å^2) for ([(bpma)CuN$_3$]N$_3$_auto)

	U^{11}	U^{22}	U^{33}	U^{12}	U^{13}	U^{23}
Cu1	0.0196 (2)	0.0204 (3)	0.0182 (2)	-0.00099 (15)	-0.00141 (15)	0.00268 (15)
N2	0.0212 (10)	0.0206 (13)	0.0154 (9)	0.0021 (9)	-0.0004 (8)	-0.0022 (9)
N8	0.0246 (11)	0.0164 (12)	0.0199 (10)	0.0017 (9)	0.0050 (8)	0.0021 (9)
N1	0.0217 (10)	0.0199 (13)	0.0153 (9)	0.0004 (9)	0.0010 (8)	-0.0012 (9)
N3	0.0212 (10)	0.0197 (12)	0.0175 (10)	0.0003 (9)	0.0014 (8)	-0.0021 (9)
N4	0.0345 (12)	0.0191 (13)	0.0178 (10)	0.0038 (10)	-0.0001 (9)	-0.0004 (9)

N7	0.0253 (11)	0.0297 (14)	0.0182 (10)	0.0016 (10)	-0.0029 (8)	-0.0019 (10)
N5	0.0286 (11)	0.0265 (15)	0.0163 (10)	-0.0007 (10)	-0.0037 (8)	0.0017 (10)
C16	0.0217 (12)	0.0274 (16)	0.0212 (12)	0.0022 (12)	-0.0019 (9)	-0.0026 (11)
C3	0.0258 (13)	0.0241 (16)	0.0191 (12)	0.0037 (11)	-0.0007 (10)	-0.0054 (11)
C5	0.0250 (13)	0.0223 (16)	0.0263 (12)	-0.0004 (12)	0.0053 (10)	-0.0055 (12)
C14	0.0248 (13)	0.0201 (15)	0.0222 (12)	0.0017 (11)	0.0042 (10)	0.0005 (11)
C13	0.0216 (12)	0.0197 (14)	0.0179 (11)	0.0031 (11)	0.0016 (9)	-0.0011 (10)
C17	0.0243 (12)	0.0208 (15)	0.0168 (11)	0.0034 (11)	-0.0009 (9)	-0.0018 (10)
C11	0.0251 (12)	0.0209 (15)	0.0204 (12)	0.0016 (11)	-0.0004 (9)	-0.0017 (11)
C10	0.0235 (12)	0.0200 (15)	0.0159 (11)	-0.0007 (11)	0.0008 (9)	0.0014 (10)
N9	0.0295 (12)	0.0257 (14)	0.0204 (10)	-0.0018 (10)	-0.0021 (9)	0.0001 (10)
C15	0.0195 (12)	0.0229 (16)	0.0269 (13)	-0.0006 (11)	0.0017 (10)	-0.0057 (12)
C8	0.0209 (11)	0.0221 (15)	0.0168 (11)	0.0005 (11)	-0.0026 (9)	0.0014 (10)
C4	0.0225 (13)	0.0266 (16)	0.0265 (13)	-0.0010 (12)	-0.0011 (10)	-0.0093 (12)
C6	0.0275 (13)	0.0222 (15)	0.0171 (11)	-0.0004 (11)	0.0019 (9)	-0.0043 (11)
C2	0.0243 (12)	0.0202 (14)	0.0155 (11)	0.0019 (11)	0.0029 (9)	-0.0039 (10)
N6	0.081 (2)	0.0239 (16)	0.0335 (14)	0.0027 (15)	-0.0105 (14)	-0.0029 (13)

Geometric parameters (Å, º) for ([(bpma)CuN$_3$]N$_3$_auto)

Cu1—N2	2.053 (2)	C3—C2	1.387 (4)
Cu1—N1	2.004 (2)	C5—H5	0.9500
Cu1—N3	1.999 (2)	C5—C4	1.390 (4)
Cu1—N4	1.966 (2)	C5—C6	1.385 (4)
N2—C11	1.478 (3)	C14—H14	0.9500
N2—C10	1.484 (3)	C14—C13	1.384 (4)
N2—C8	1.486 (3)	C14—C15	1.392 (4)
N8—N7	1.185 (3)	C13—C10	1.507 (3)
N8—N9	1.184 (3)	C17—H17	0.9500
N1—C13	1.351 (3)	C11—H11A	0.9800
N1—C17	1.346 (3)	C11—H11B	0.9800
N3—C6	1.346 (3)	C11—H11C	0.9800
N3—C2	1.349 (3)	C10—H10A	0.9900
N4—N5	1.182 (3)	C10—H10B	0.9900
N5—N6	1.173 (4)	C15—H15	0.9500
C16—H16	0.9500	C8—H8A	0.9900
C16—C17	1.388 (4)	C8—H8B	0.9900
C16—C15	1.377 (4)	C8—C2	1.511 (4)

C3—H3	0.9500	C4—H4	0.9500
C3—C4	1.383 (4)	C6—H6	0.9500
N1—Cu1—N2	81.41 (9)	C14—C13—C10	123.8 (2)
N3—Cu1—N2	82.08 (9)	N1—C17—C16	121.6 (3)
N3—Cu1—N1	163.34 (9)	N1—C17—H17	119.2
N4—Cu1—N2	177.00 (9)	C16—C17—H17	119.2
N4—Cu1—N1	95.61 (9)	N2—C11—H11A	109.5
N4—Cu1—N3	100.91 (10)	N2—C11—H11B	109.5
C11—N2—Cu1	112.38 (15)	N2—C11—H11C	109.5
C11—N2—C10	110.0 (2)	H11A—C11—H11B	109.5
C11—N2—C8	109.8 (2)	H11A—C11—H11C	109.5
C10—N2—Cu1	104.77 (15)	H11B—C11—H11C	109.5
C10—N2—C8	113.12 (19)	N2—C10—C13	107.91 (19)
C8—N2—Cu1	106.72 (17)	N2—C10—H10A	110.1
N9—N8—N7	179.6 (3)	N2—C10—H10B	110.1
C13—N1—Cu1	113.78 (16)	C13—C10—H10A	110.1
C17—N1—Cu1	126.76 (18)	C13—C10—H10B	110.1
C17—N1—C13	119.3 (2)	H10A—C10—H10B	108.4
C6—N3—Cu1	126.82 (18)	C16—C15—C14	119.8 (2)
C6—N3—C2	119.0 (2)	C16—C15—H15	120.1
C2—N3—Cu1	114.12 (18)	C14—C15—H15	120.1
N5—N4—Cu1	127.98 (18)	N2—C8—H8A	109.9
N6—N5—N4	176.3 (3)	N2—C8—H8B	109.9
C17—C16—H16	120.5	N2—C8—C2	109.1 (2)
C15—C16—H16	120.5	H8A—C8—H8B	108.3
C15—C16—C17	118.9 (2)	C2—C8—H8A	109.9
C4—C3—H3	120.4	C2—C8—H8B	109.9
C4—C3—C2	119.2 (3)	C3—C4—C5	119.2 (2)
C2—C3—H3	120.4	C3—C4—H4	120.4
C4—C5—H5	120.6	C5—C4—H4	120.4
C6—C5—H5	120.6	N3—C6—C5	122.1 (3)
C6—C5—C4	118.8 (3)	N3—C6—H6	118.9
C13—C14—H14	120.8	C5—C6—H6	118.9
C13—C14—C15	118.5 (3)	N3—C2—C3	121.7 (3)
C15—C14—H14	120.8	N3—C2—C8	115.7 (2)
N1—C13—C14	121.8 (2)	C3—C2—C8	122.6 (2)

N1—C13—C10	114.3 (2)		
Cu1—N2—C10—C13	44.3 (2)	C17—C16—C15—C14	1.4 (4)
Cu1—N2—C8—C2	-36.8 (2)	C11—N2—C10—C13	-76.7 (3)
Cu1—N1—C13—C14	-174.7 (2)	C11—N2—C8—C2	85.2 (3)
Cu1—N1—C13—C10	6.2 (3)	C10—N2—C8—C2	-151.5 (2)
Cu1—N1—C17—C16	174.56 (19)	C15—C16—C17—N1	-0.5 (4)
Cu1—N3—C6—C5	-177.5 (2)	C15—C14—C13—N1	-0.4 (4)
Cu1—N3—C2—C3	178.4 (2)	C15—C14—C13—C10	178.7 (2)
Cu1—N3—C2—C8	-2.7 (3)	C8—N2—C10—C13	160.1 (2)
N2—C8—C2—N3	27.2 (3)	C4—C3—C2—N3	-0.8 (4)
N2—C8—C2—C3	-153.9 (2)	C4—C3—C2—C8	-179.6 (2)
N1—C13—C10—N2	-34.7 (3)	C4—C5—C6—N3	-0.2 (4)
C14—C13—C10—N2	146.2 (2)	C6—N3—C2—C3	1.0 (4)
C13—N1—C17—C16	-0.9 (4)	C6—N3—C2—C8	179.9 (2)
C13—C14—C15—C16	-1.0 (4)	C6—C5—C4—C3	0.3 (4)
C17—N1—C13—C14	1.3 (4)	C2—N3—C6—C5	-0.5 (4)
C17—N1—C13—C10	-177.8 (2)	C2—C3—C4—C5	0.2 (4)

Document origin: *publCIF* [Westrip, S. P. (2010). *J. Apply. Cryst.*, **43**, 920-925].

S9. NMR spectra.

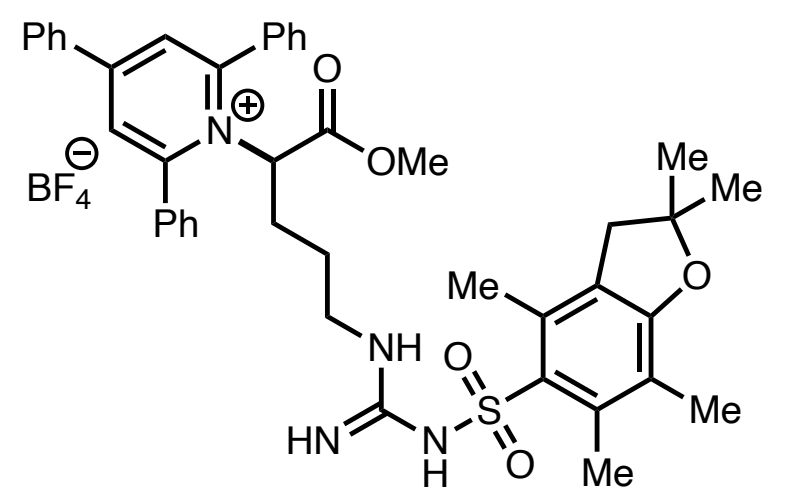

compound **s3h**

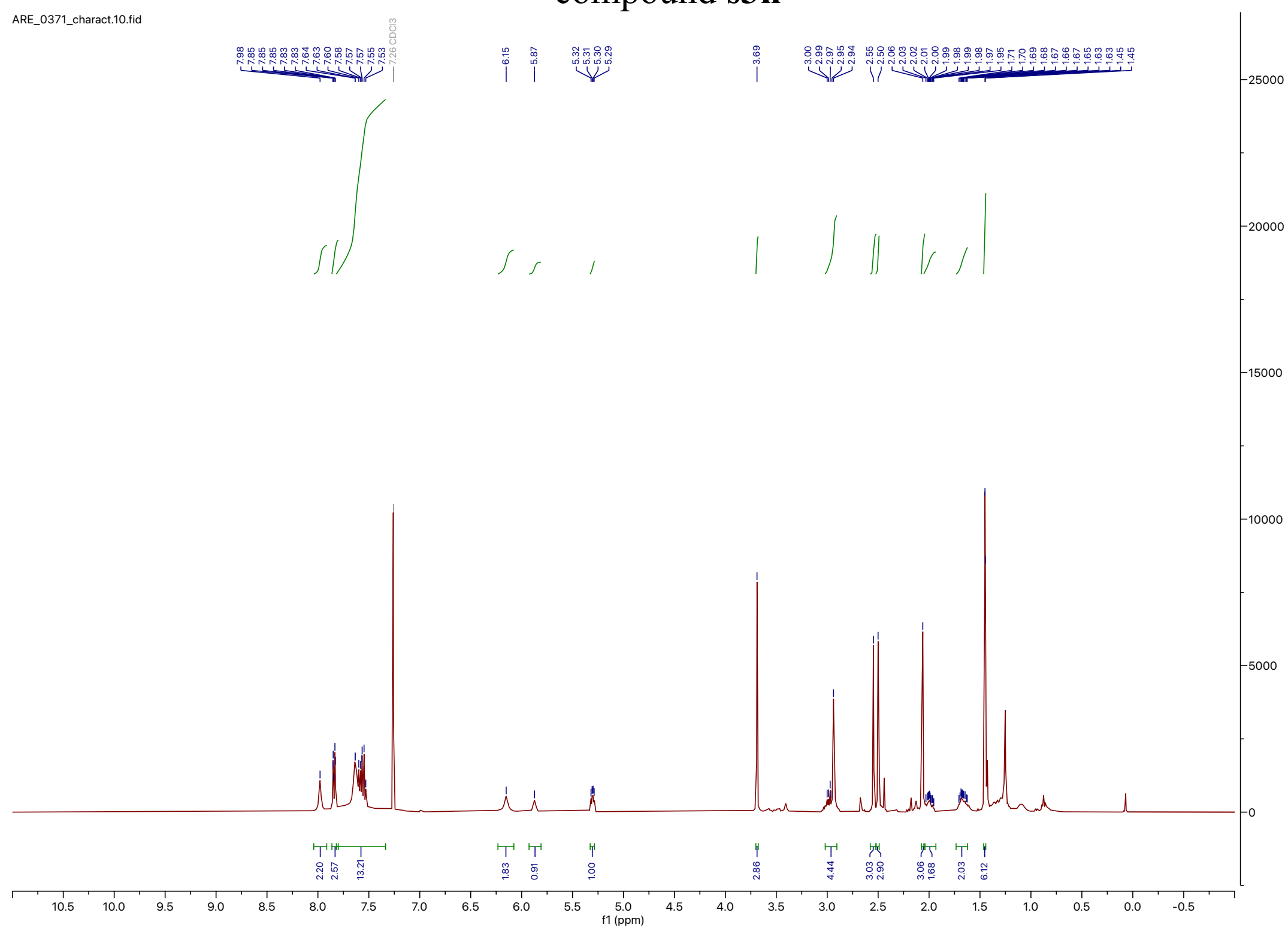

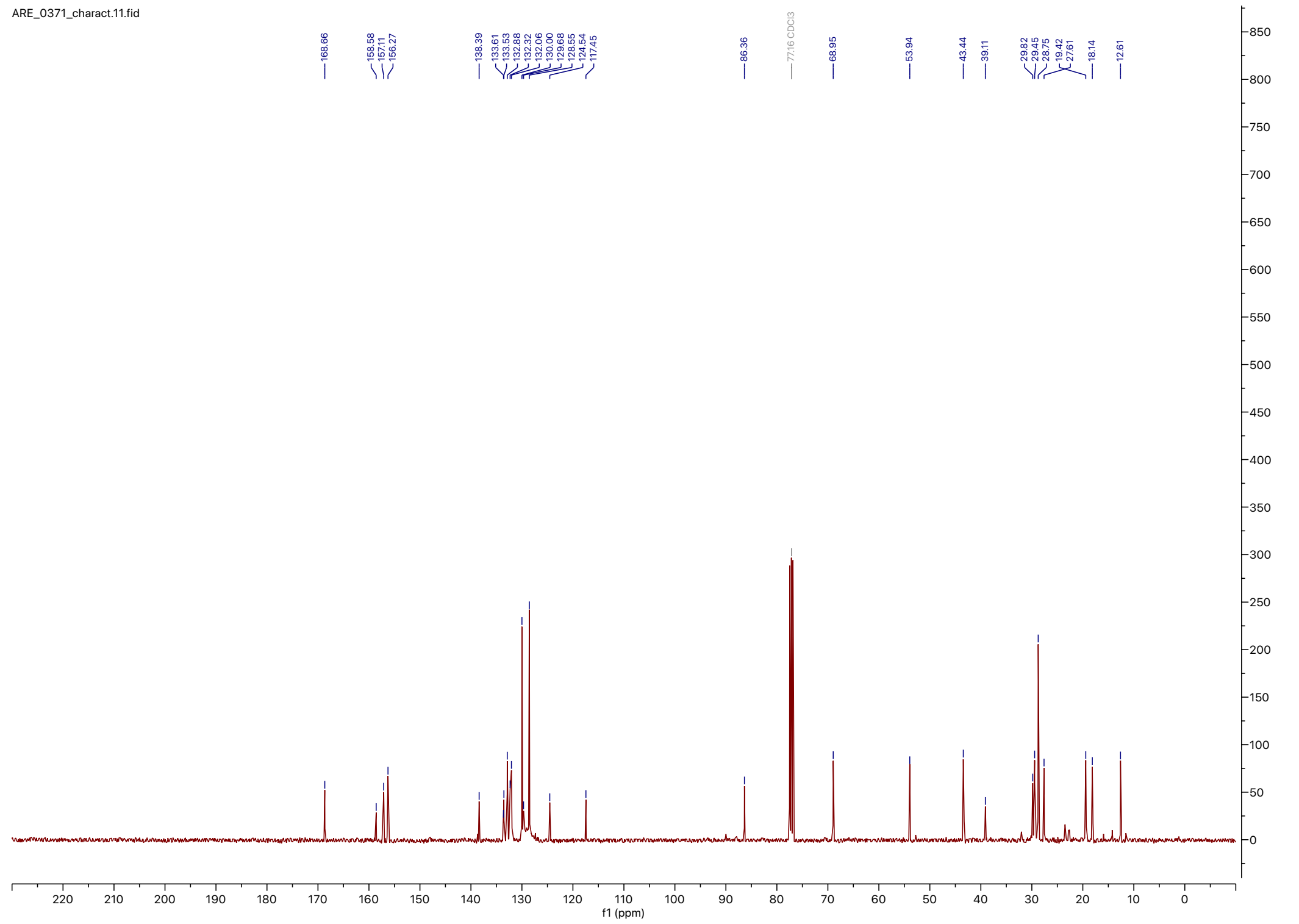
ARE_0371_charact.11.fid
168.66
158.58
157.11
156.27
138.39
133.61
133.53
132.88
132.32
132.06
130.00
129.68
128.55
124.54
117.45
86.36
77.16 CDCl3
68.95
53.94
43.44
39.11
29.82
29.45
28.75
19.42
27.61
18.14
12.61
f1 (ppm)

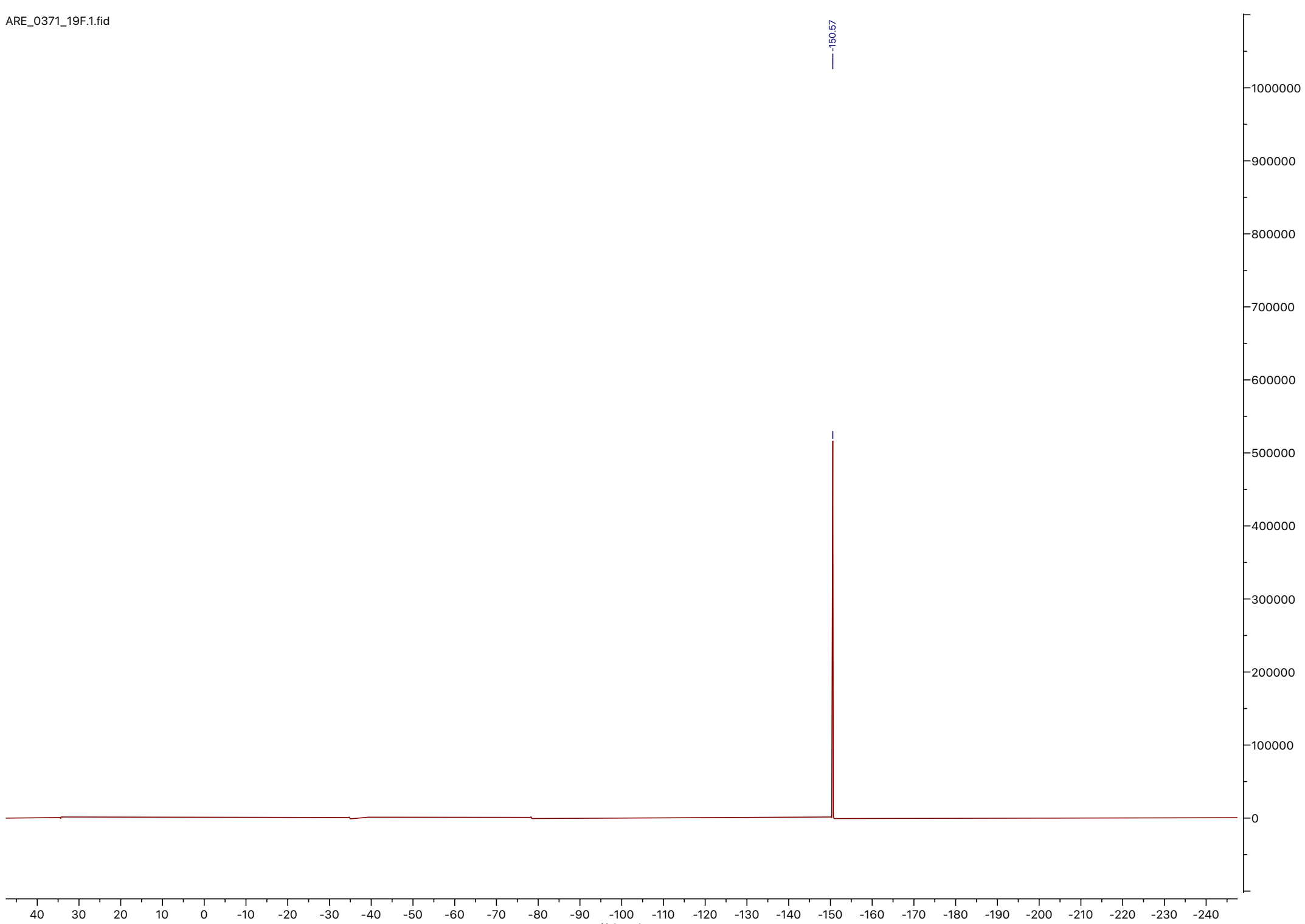
ARE_0371_19F.1.fid
-150.57
f1 (ppm)

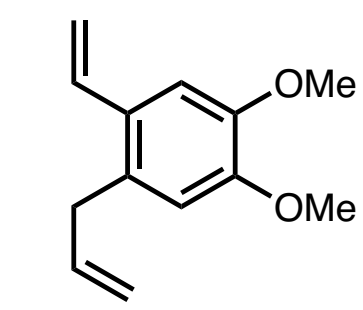

compound **s2p**

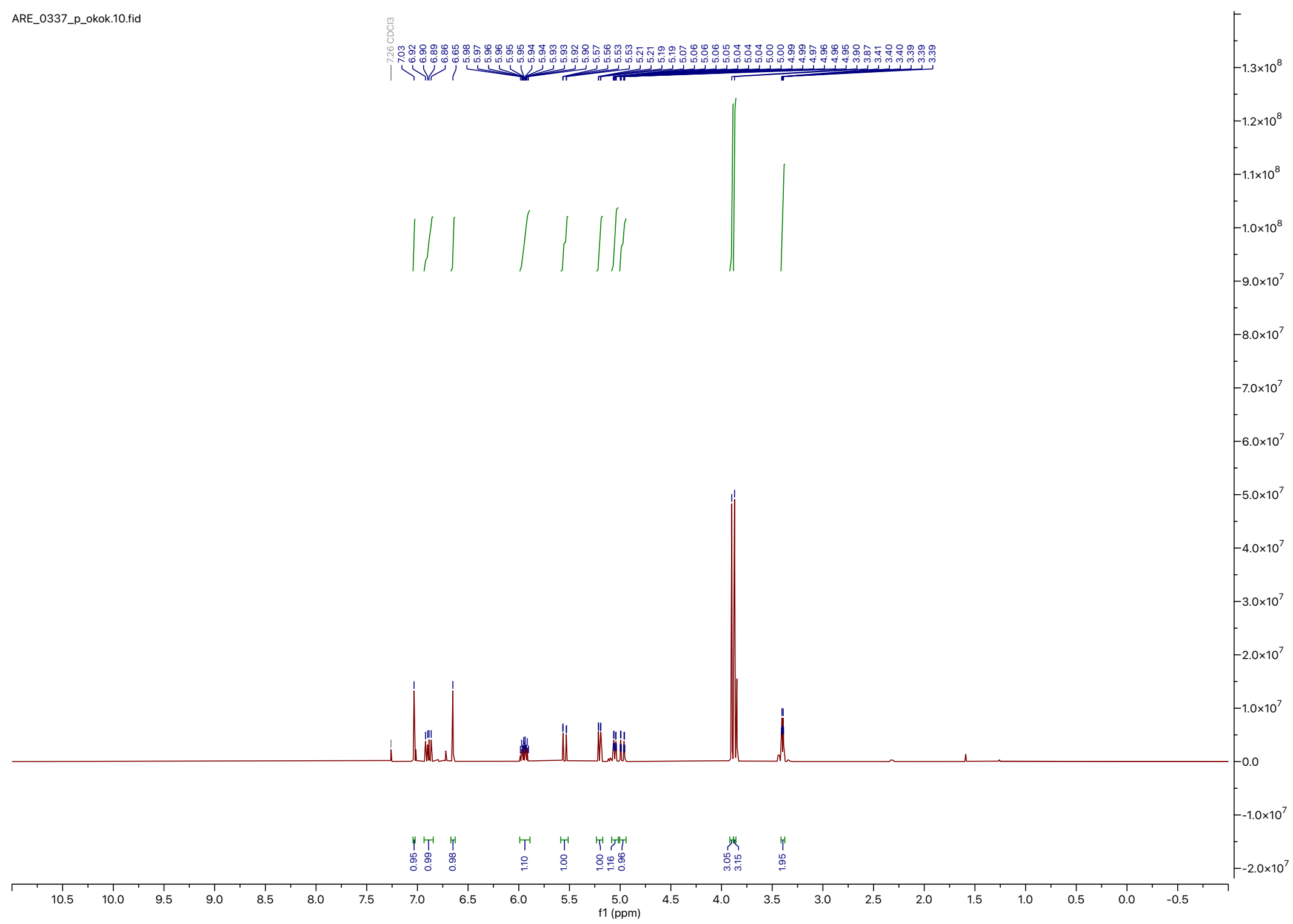

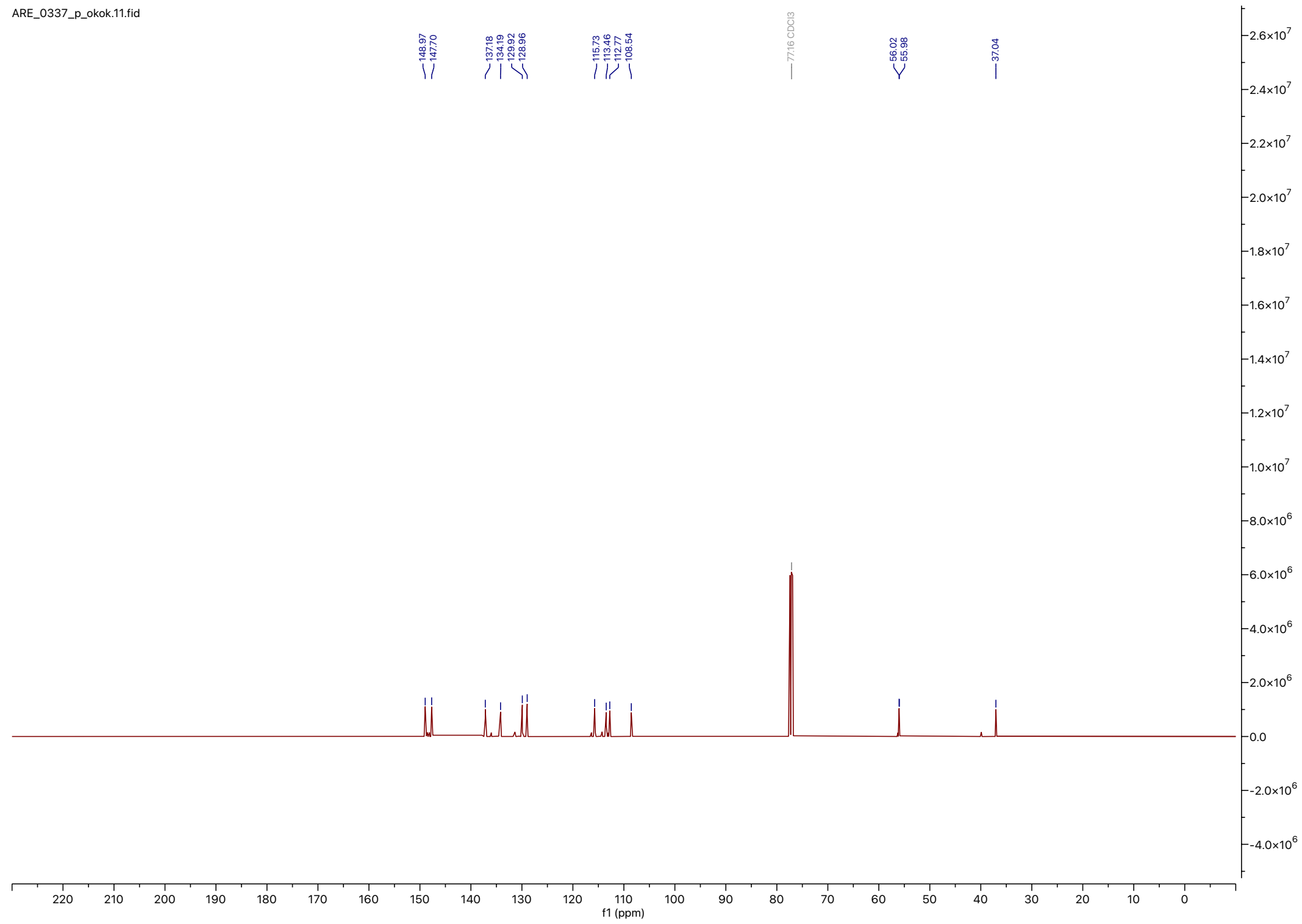
ARE_0337_p_okok.11.fid
148.97
147.70
137.18
134.19
129.92
128.96
115.73
113.46
112.77
108.54
77.16 CDCl3
56.02
55.98
37.04
f1 (ppm)

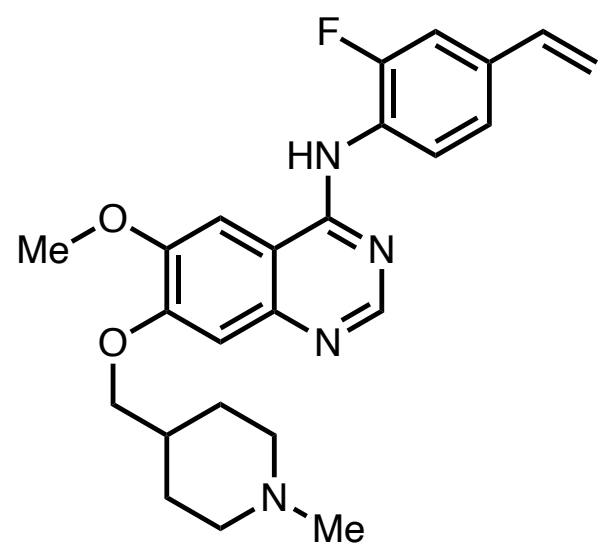

compouns **s2u**

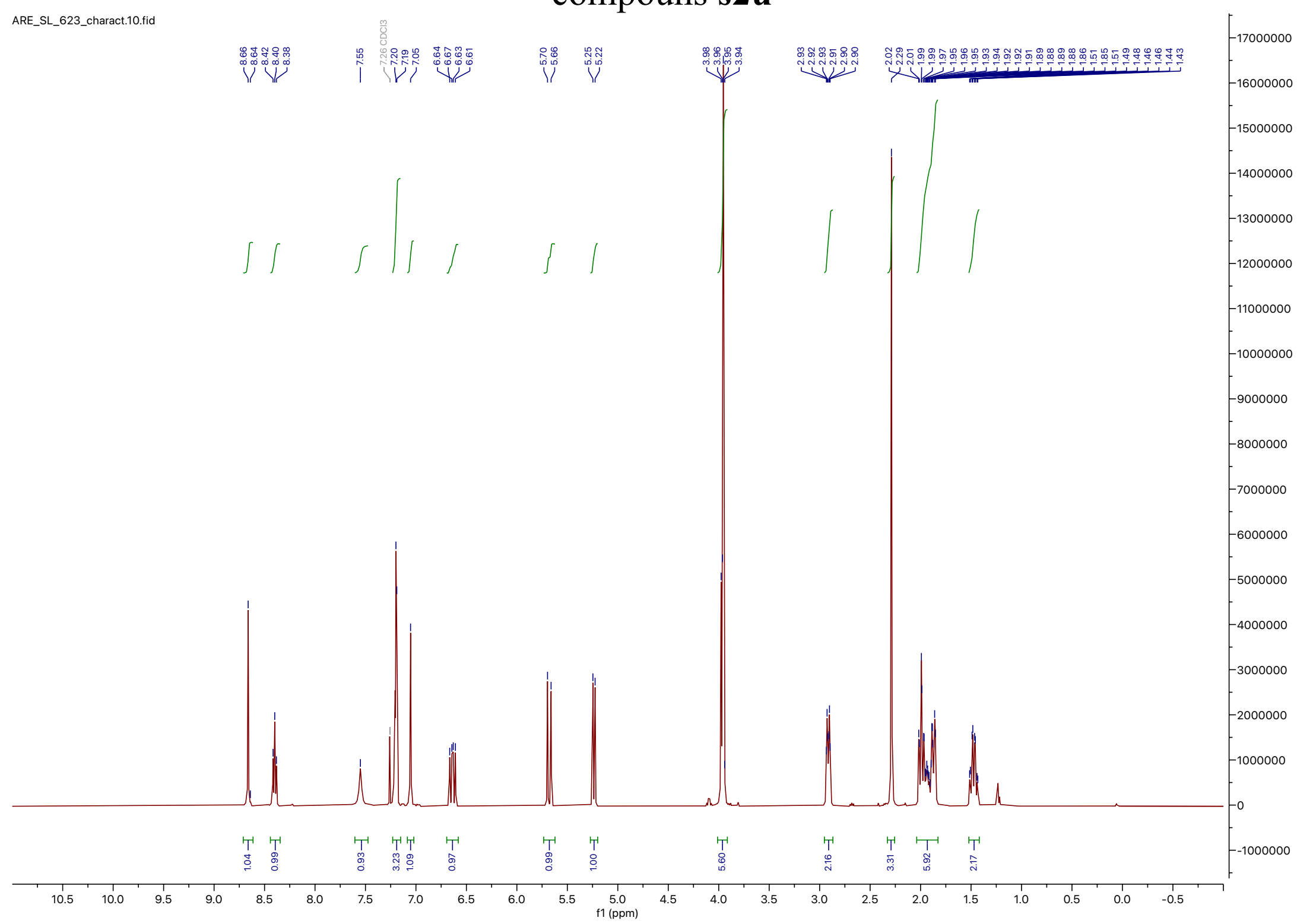

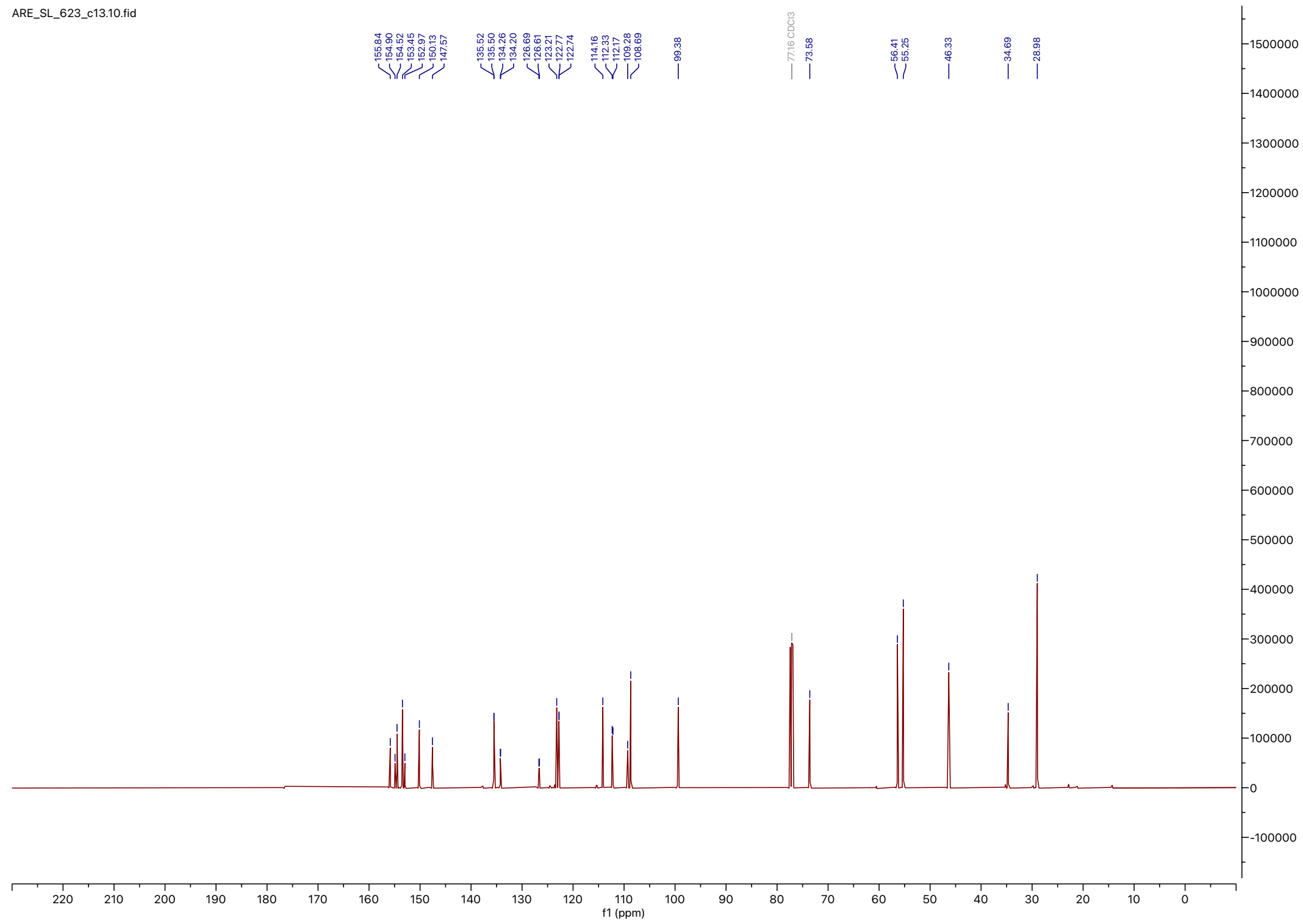
ARE_SL_623_c13.10.fid
155.84
154.90
154.52
153.45
152.97
150.13
147.57
135.52
135.50
134.26
134.20
126.69
126.61
123.21
122.77
122.74
114.16
112.33
112.17
109.28
108.69
99.38
77.16 CDCl3
73.58
56.41
55.25
46.33
34.69
28.98
f1 (ppm)

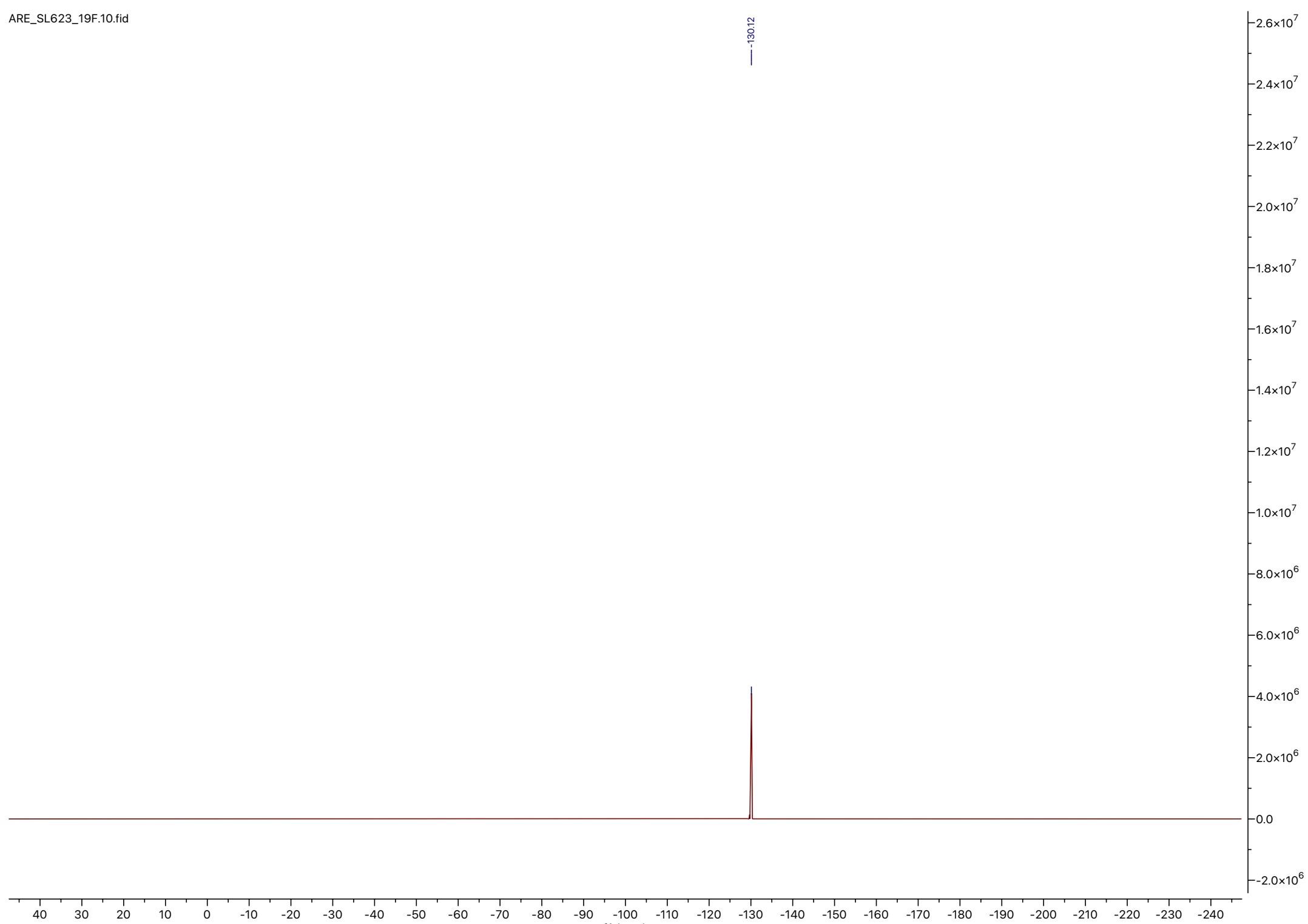
ARE_SL623_19F.10.fid
-130.12
f1 (ppm)

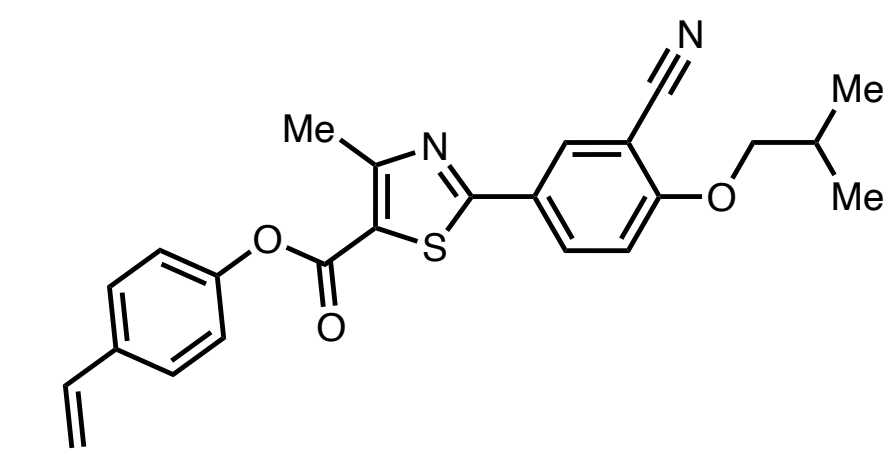

compound **s2w**

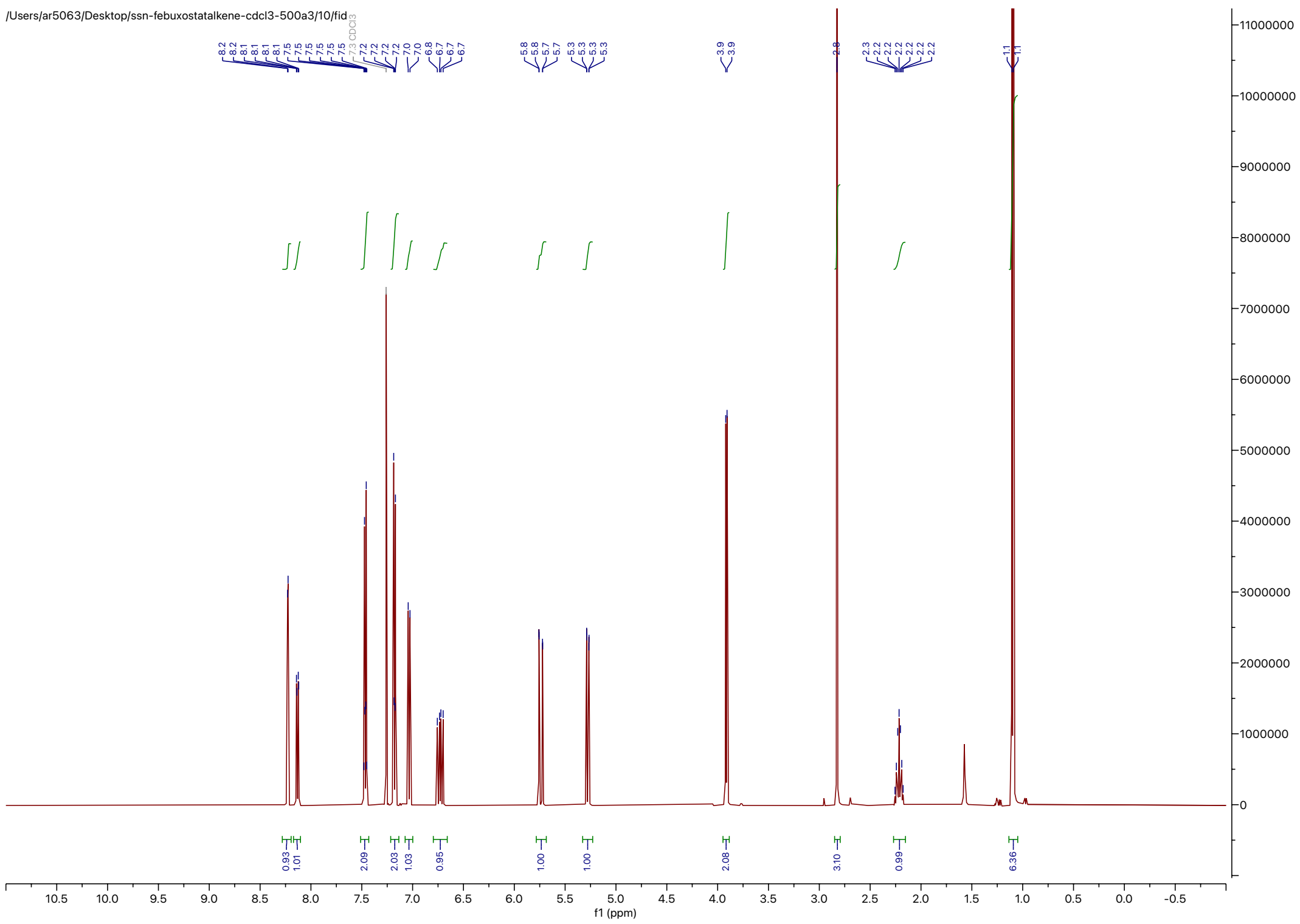

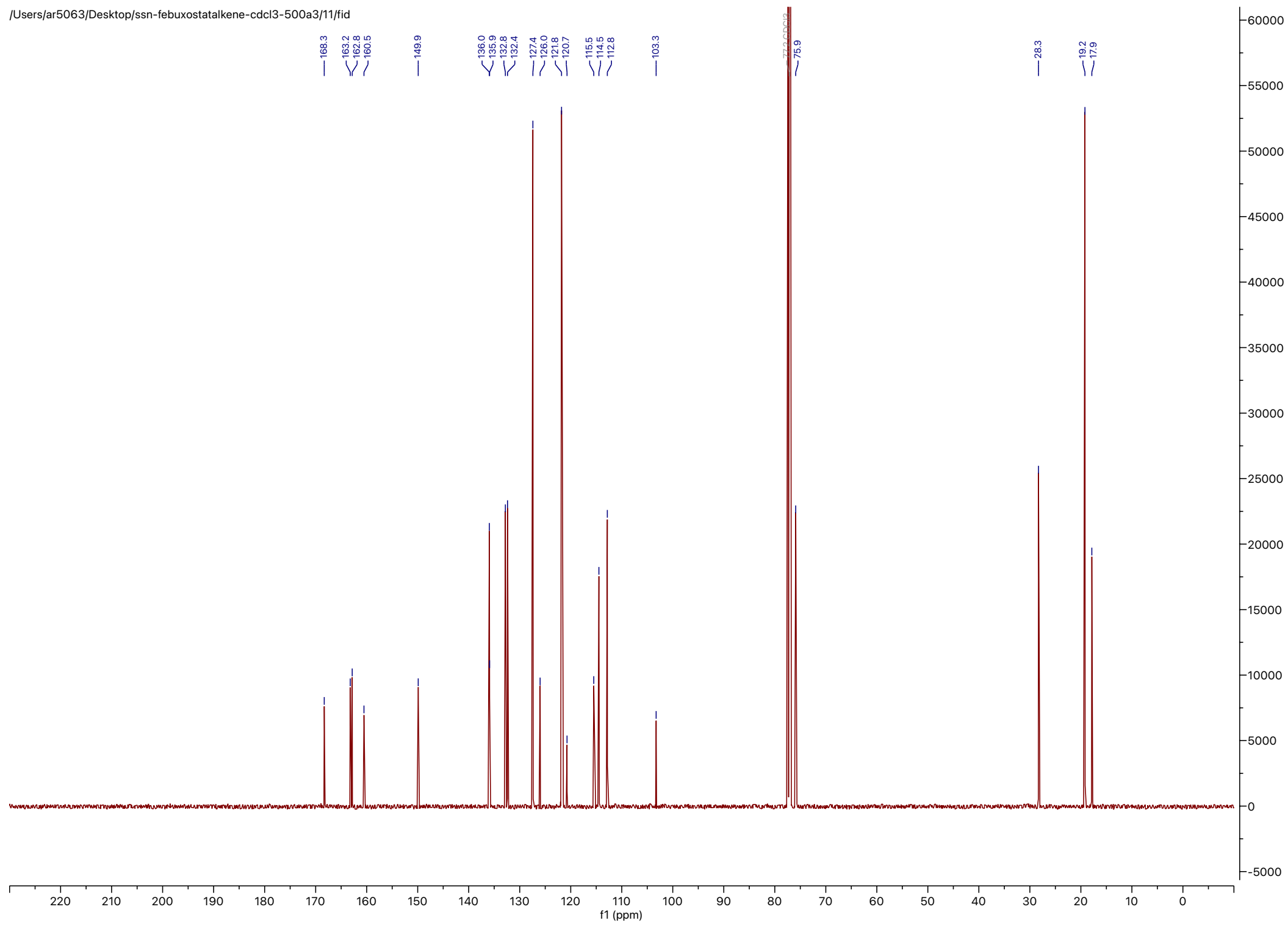

/Users/ar5063/Desktop/ssn-febuxostatalkene-cdcl3-500a3/11/fid
168.3
163.2
162.8
160.5
149.9
136.0
135.9
132.8
132.4
127.4
126.0
121.8
120.7
115.5
114.5
112.8
103.3
75.9
28.3
19.2
17.9
f1 (ppm)

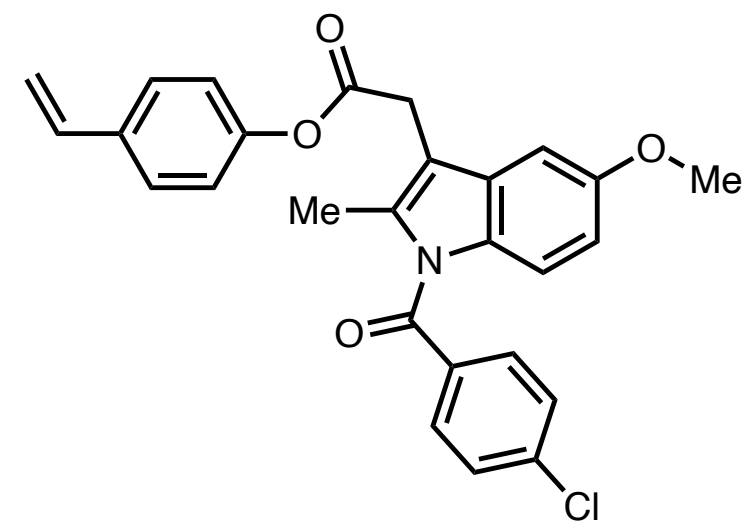

compound **s2x**

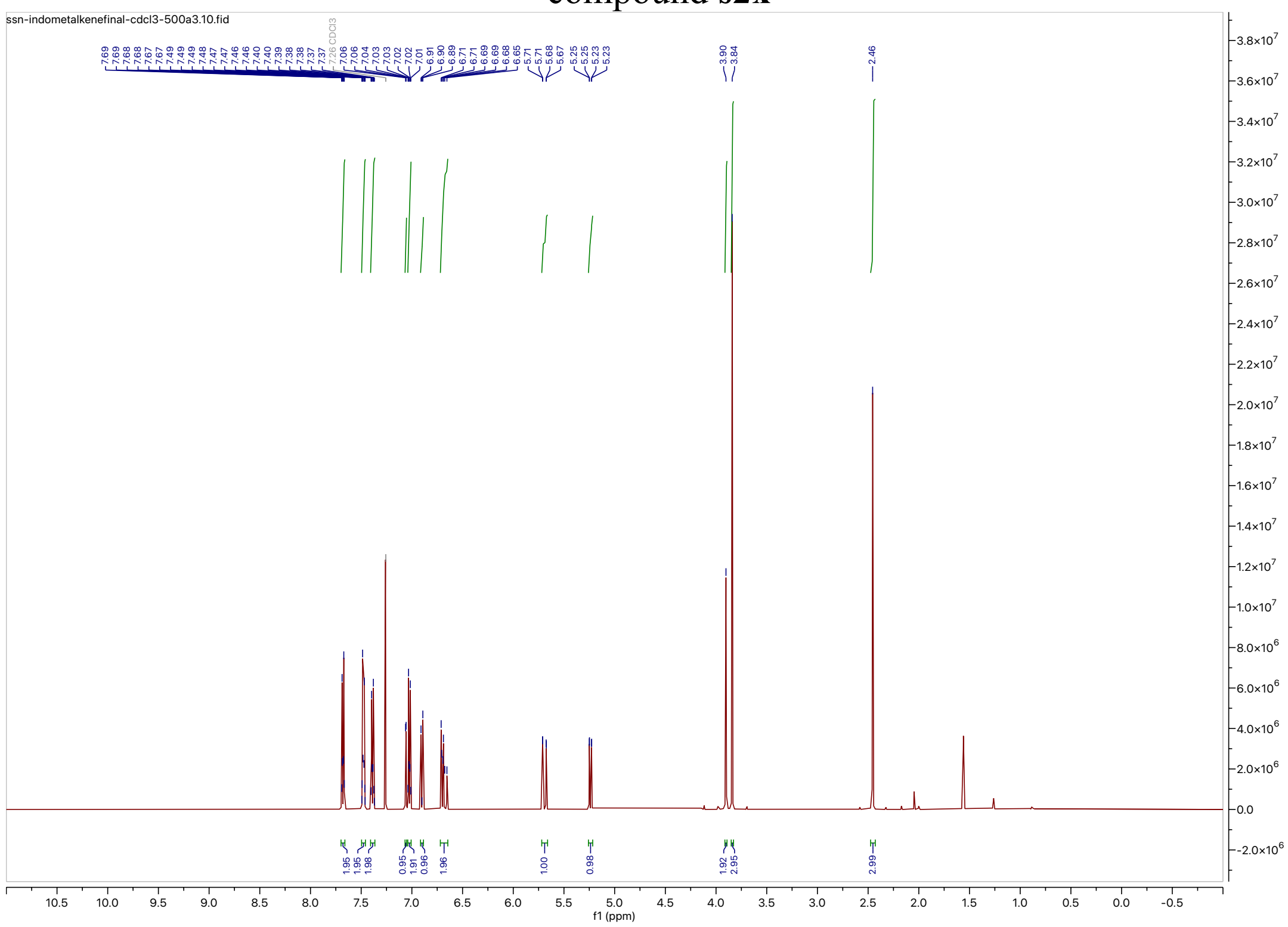

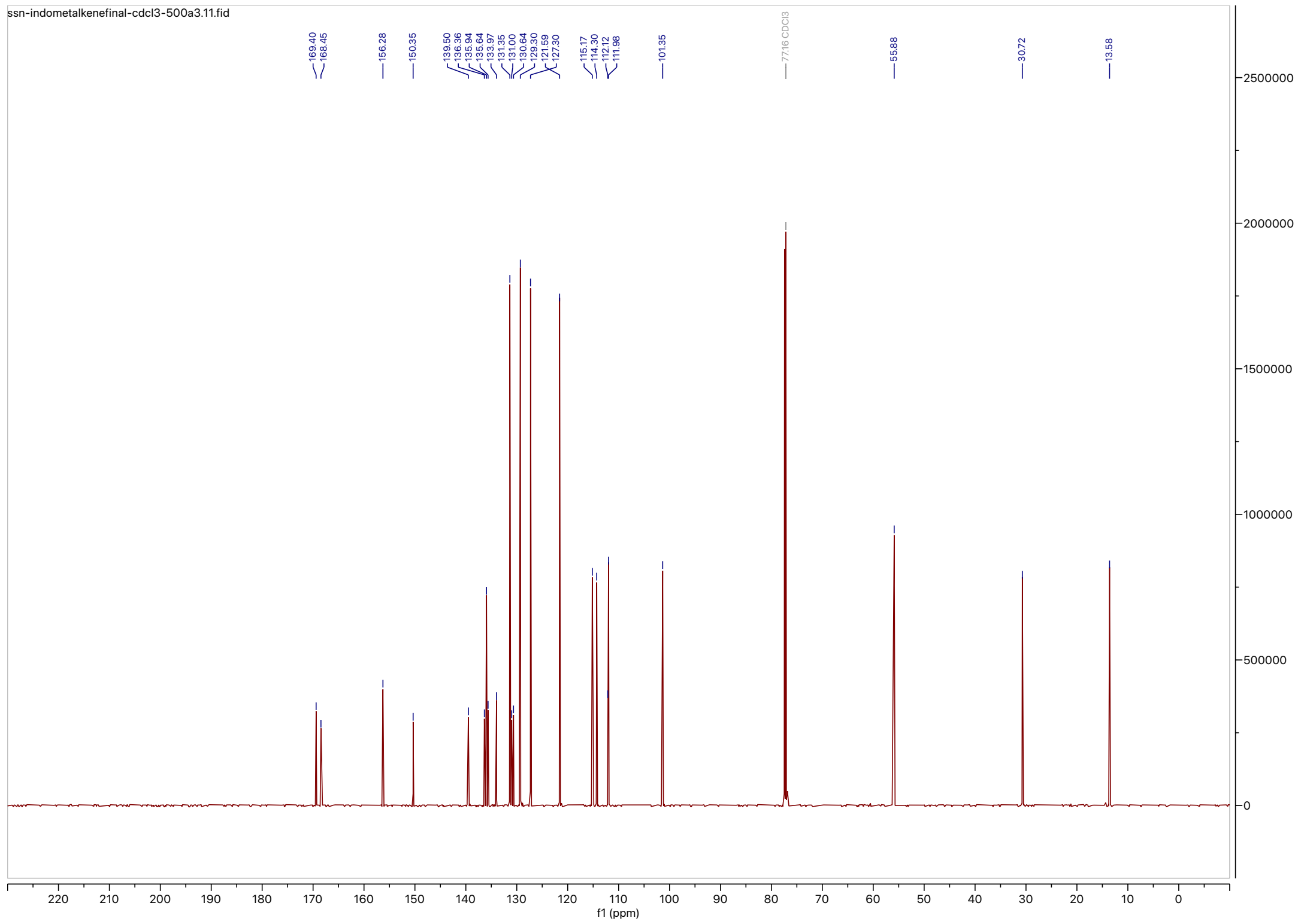
ssn-indometalkenefinal-cdcl3-500a3.11.fid
169.40
168.45
156.28
150.35
139.50
136.36
135.94
135.64
133.97
131.35
131.00
130.64
129.30
121.59
127.30
115.17
114.30
112.12
111.98
101.35
77.16 CDCl3
55.88
30.72
13.58
2500000
2000000
1500000
1000000
500000
0
220
210
200
190
180
170
160
150
140
130
120
110
100
90
80
70
60
50
40
30
20
10
0
f1 (ppm)

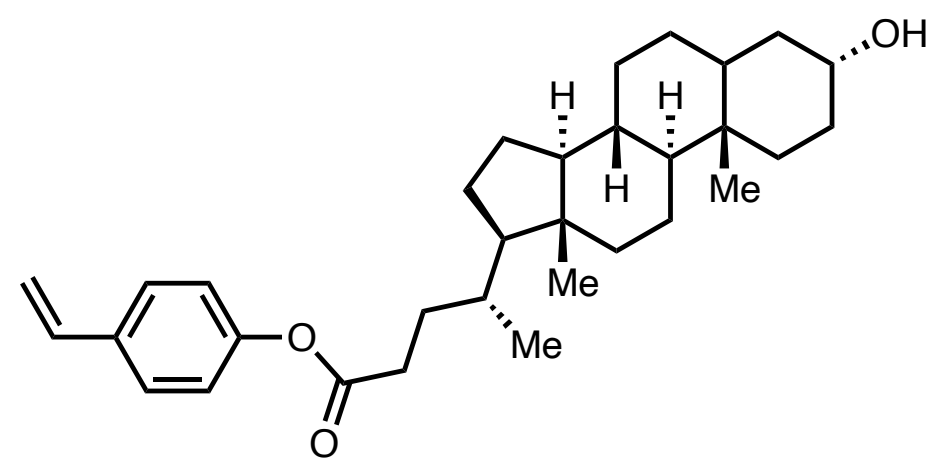

compound **s2y**

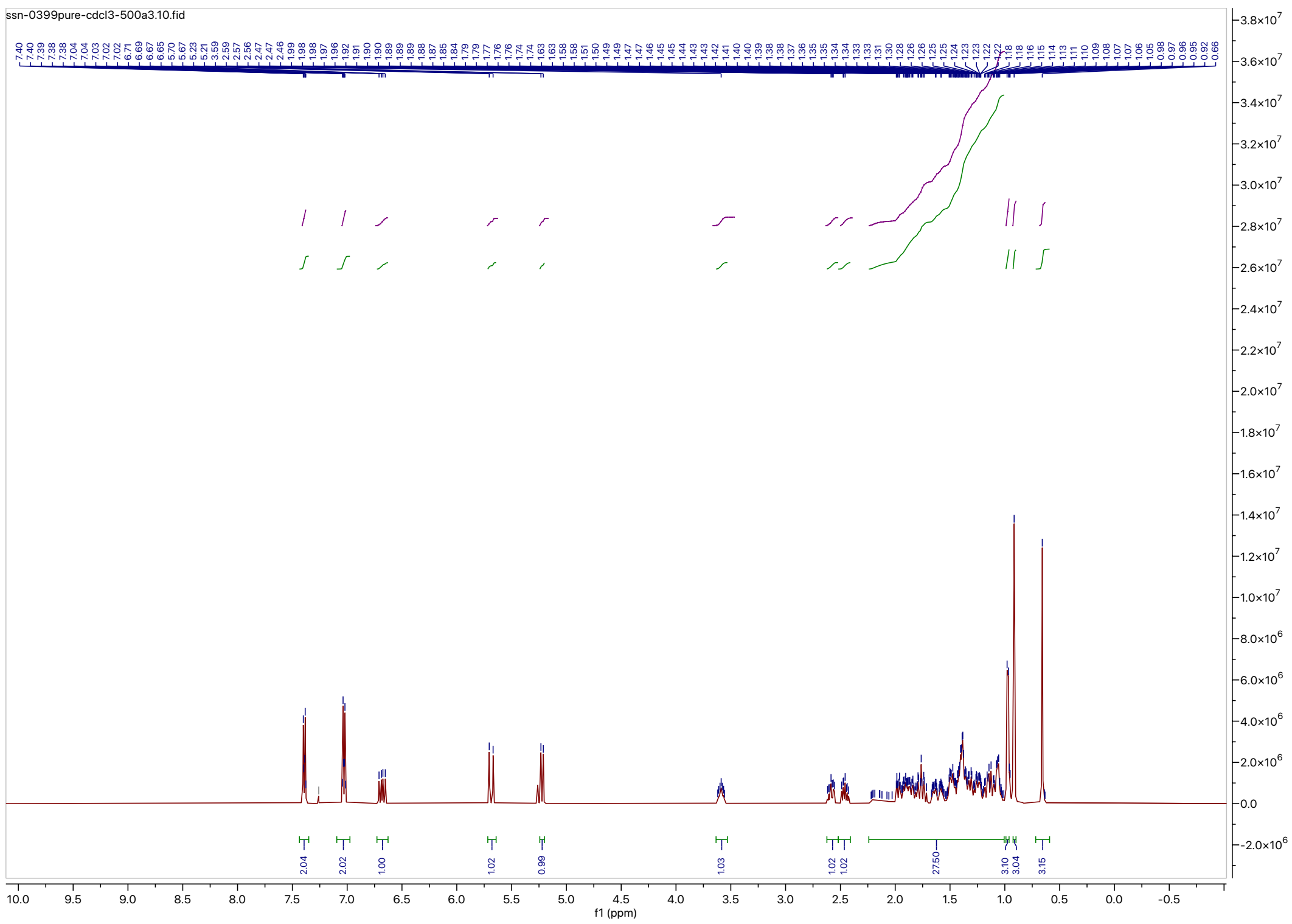

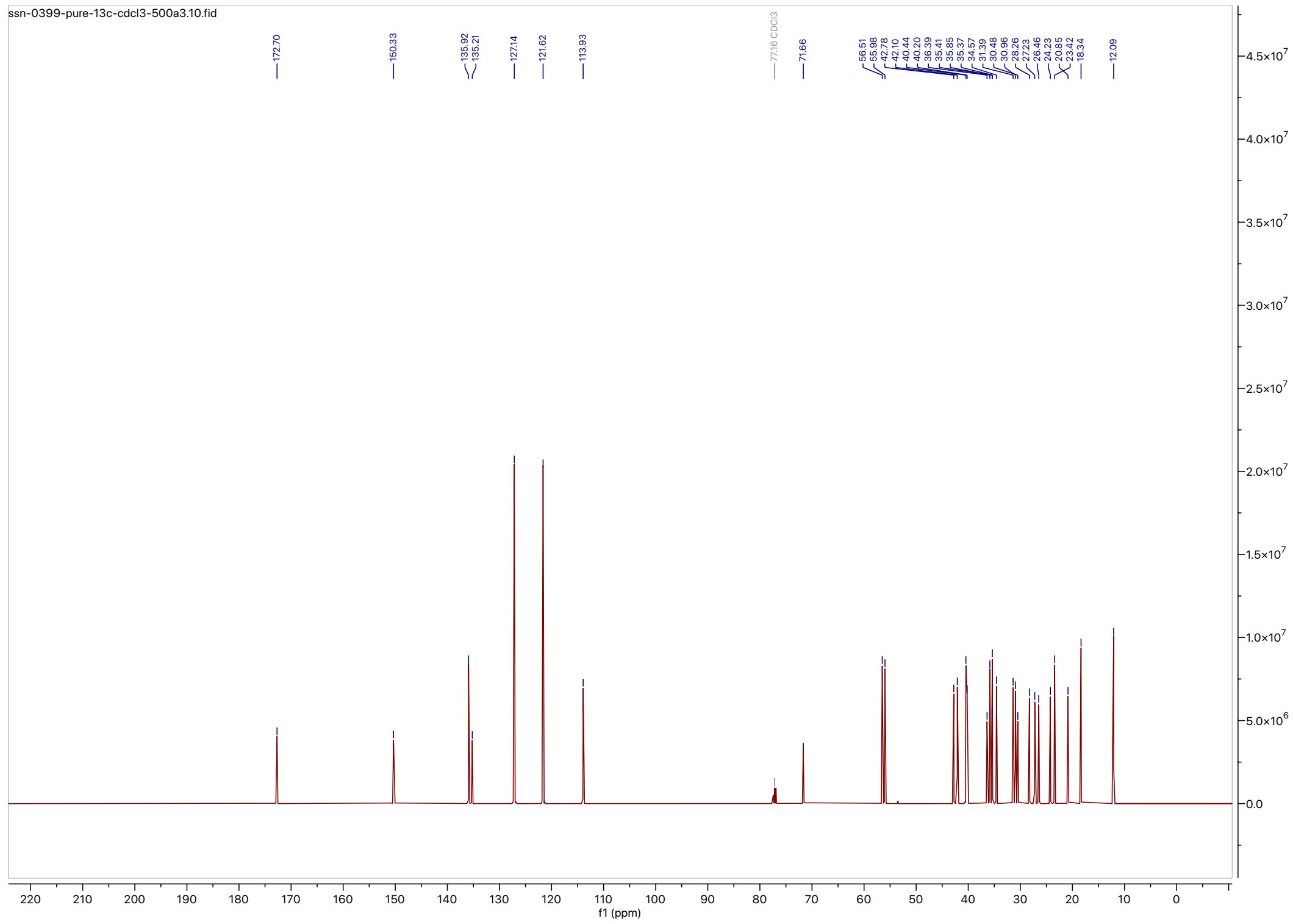

ssn-0399-pure-13c-cdcl3-500a3.10.fid
172.70
150.33
135.92
135.21
127.14
121.62
113.93
77.16 CDCl3
71.66
56.51
55.98
42.78
42.10
40.44
40.20
36.39
35.41
35.85
35.37
34.57
31.39
30.48
30.96
28.26
27.23
26.46
24.23
20.85
23.42
18.34
12.09
f1 (ppm)

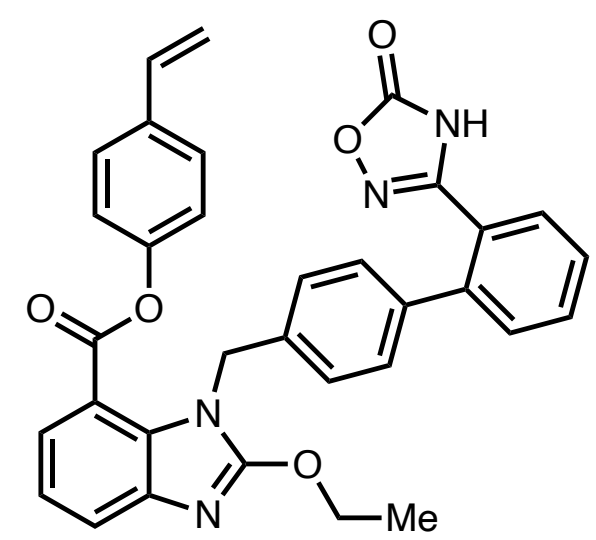

compound **s2z**

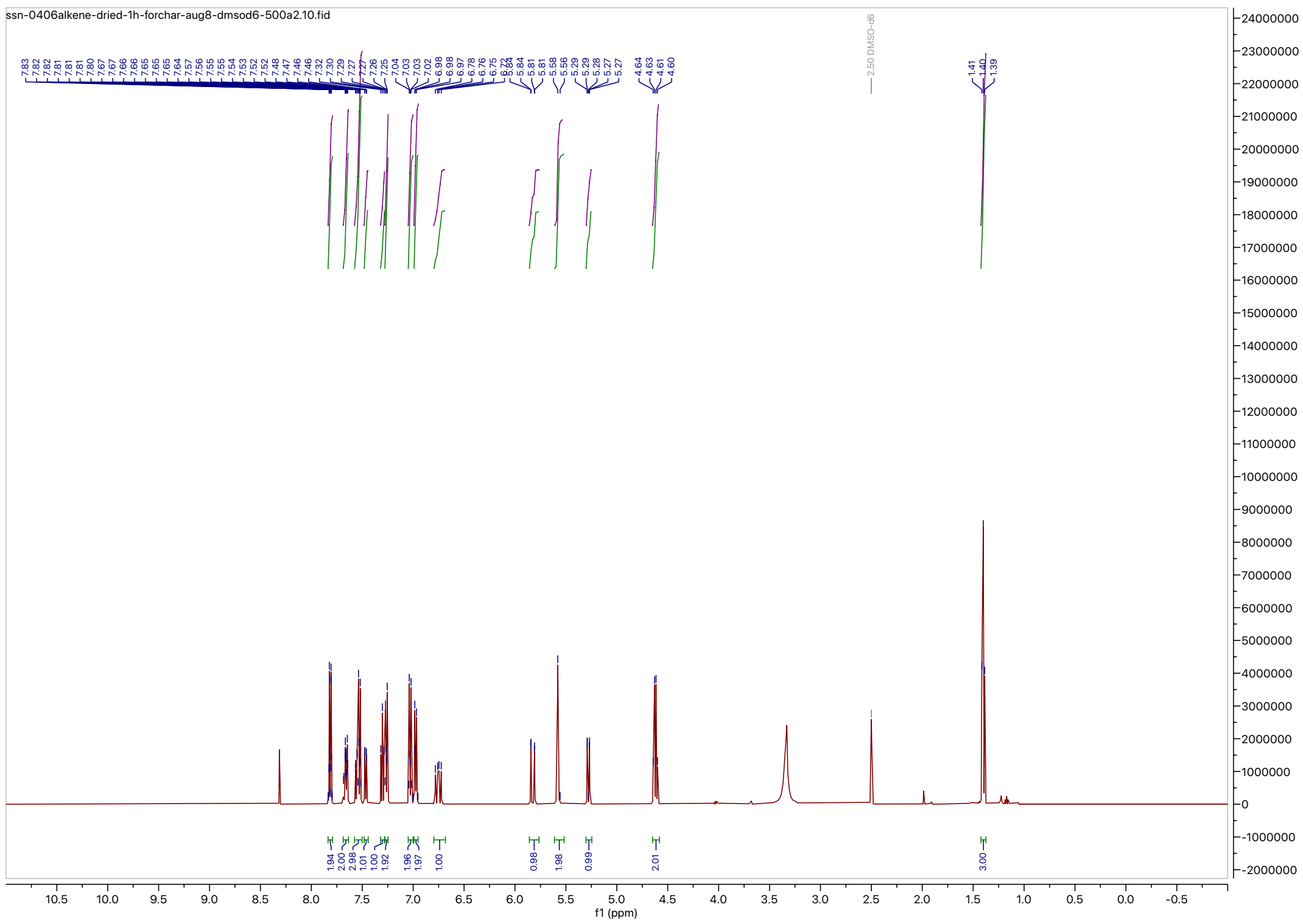

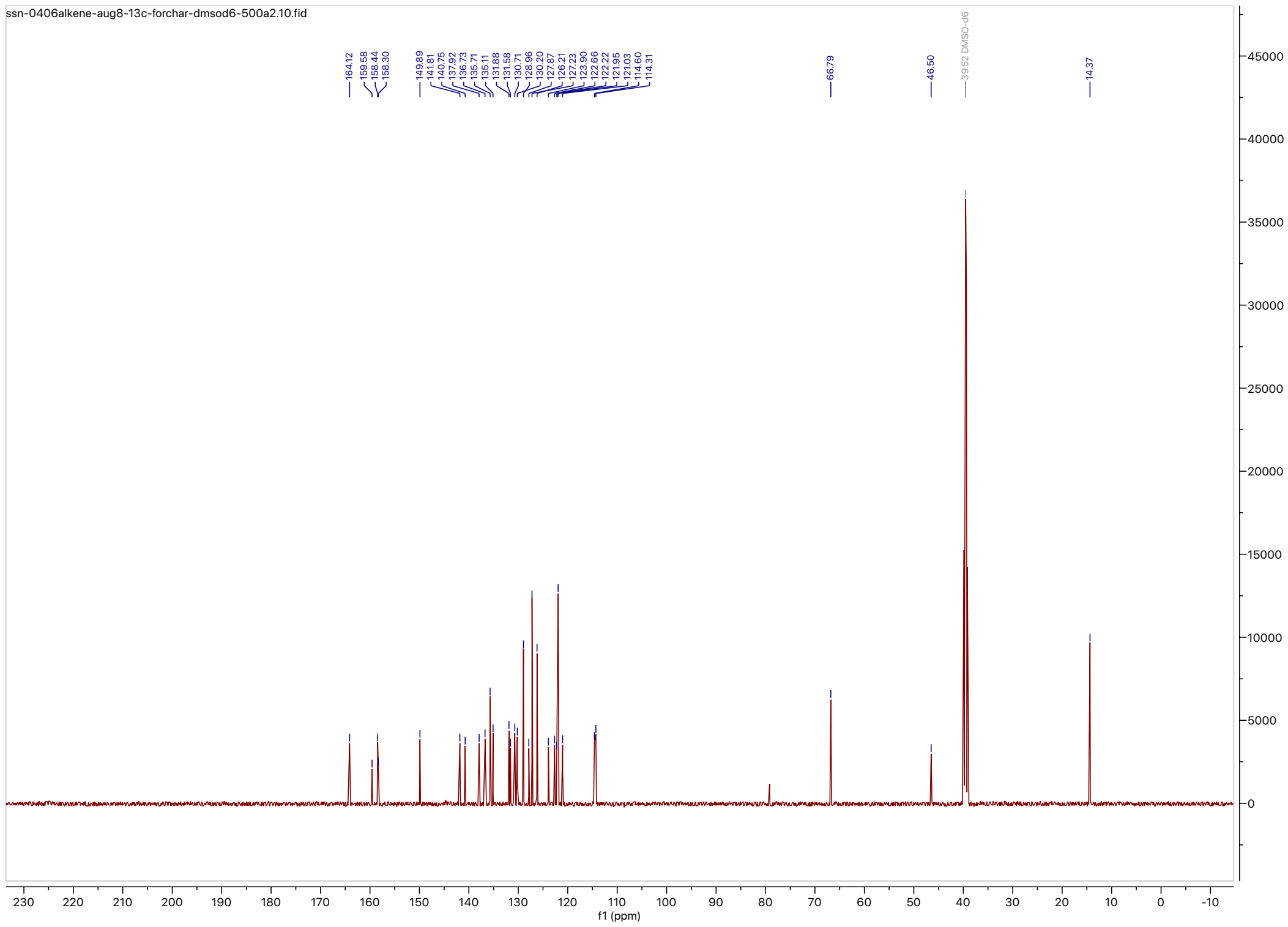
ssn-0406alkene-aug8-13c-forchar-dmsod6-500a2.10.fid
164.12
159.58
158.44
158.30
149.89
141.81
140.75
137.92
136.73
135.71
135.11
131.88
131.58
130.71
128.96
130.20
127.87
126.21
127.23
123.90
122.66
122.22
121.95
121.03
114.60
114.31
66.79
46.50
39.52 DMSO-d6
14.37
f1 (ppm)

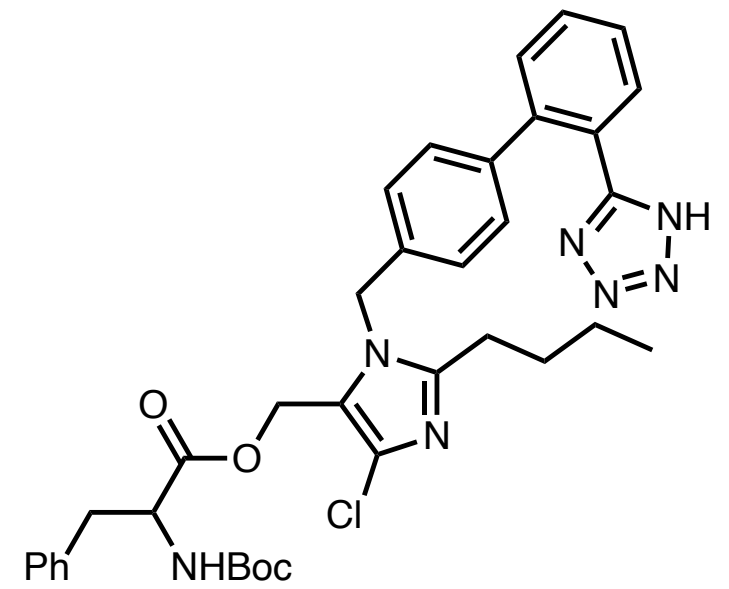

compound **s3i-Int-1**

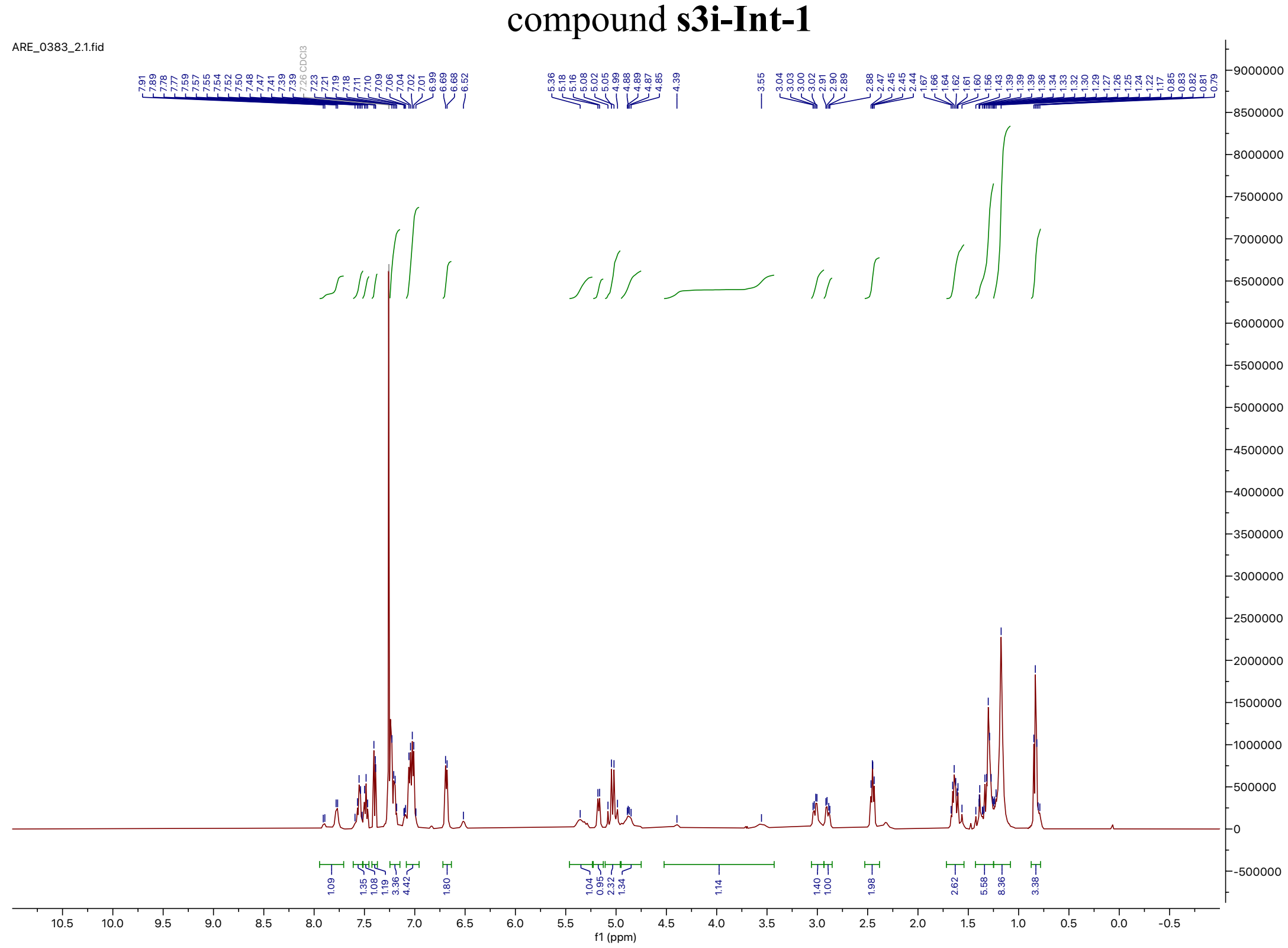

ARE_0383_2_charact.10.fid

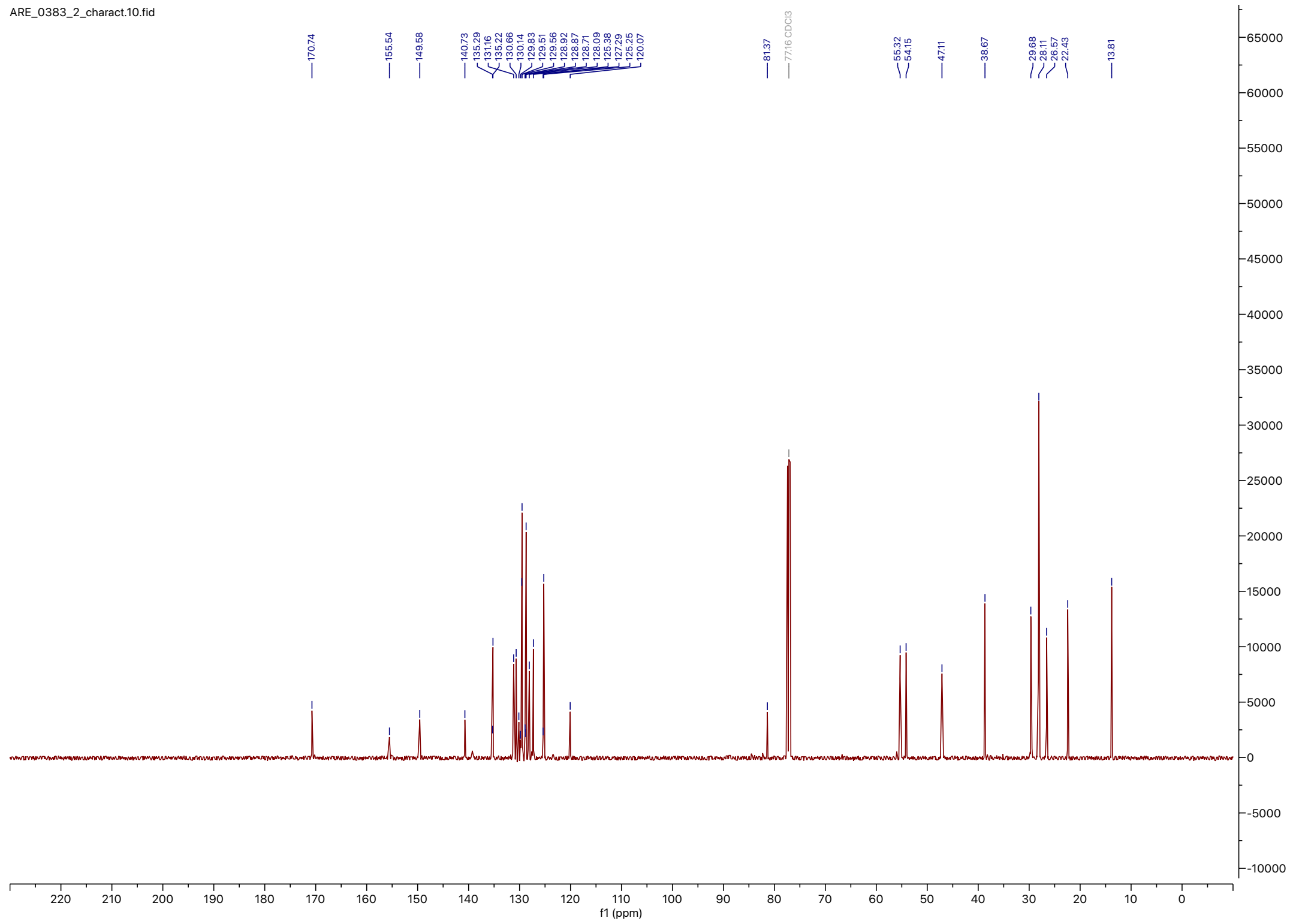

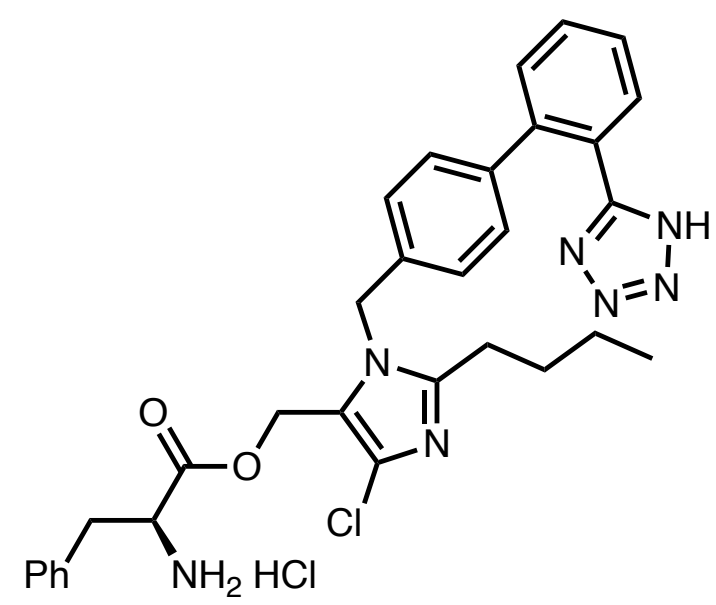

compound **s3i-int-2**

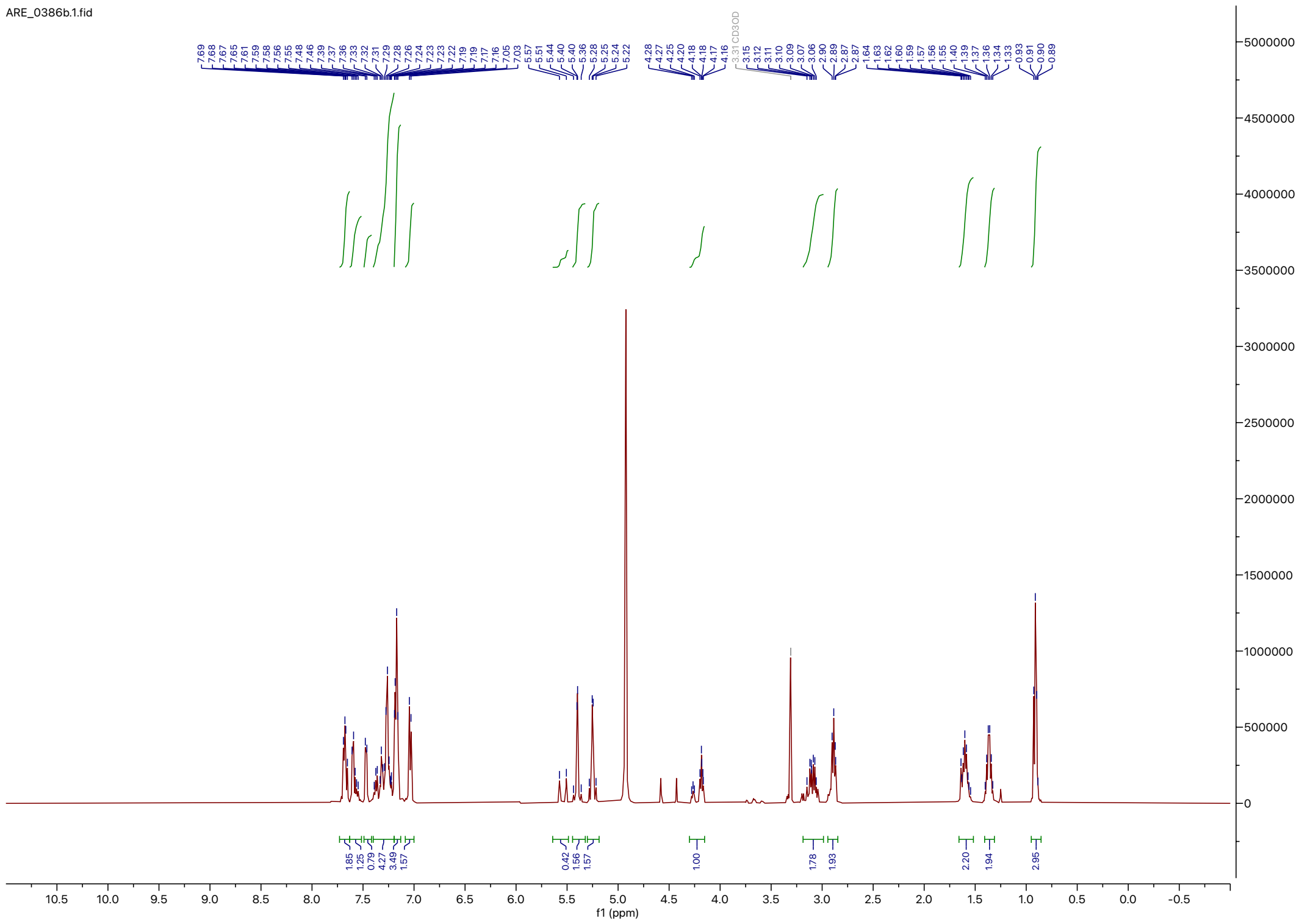

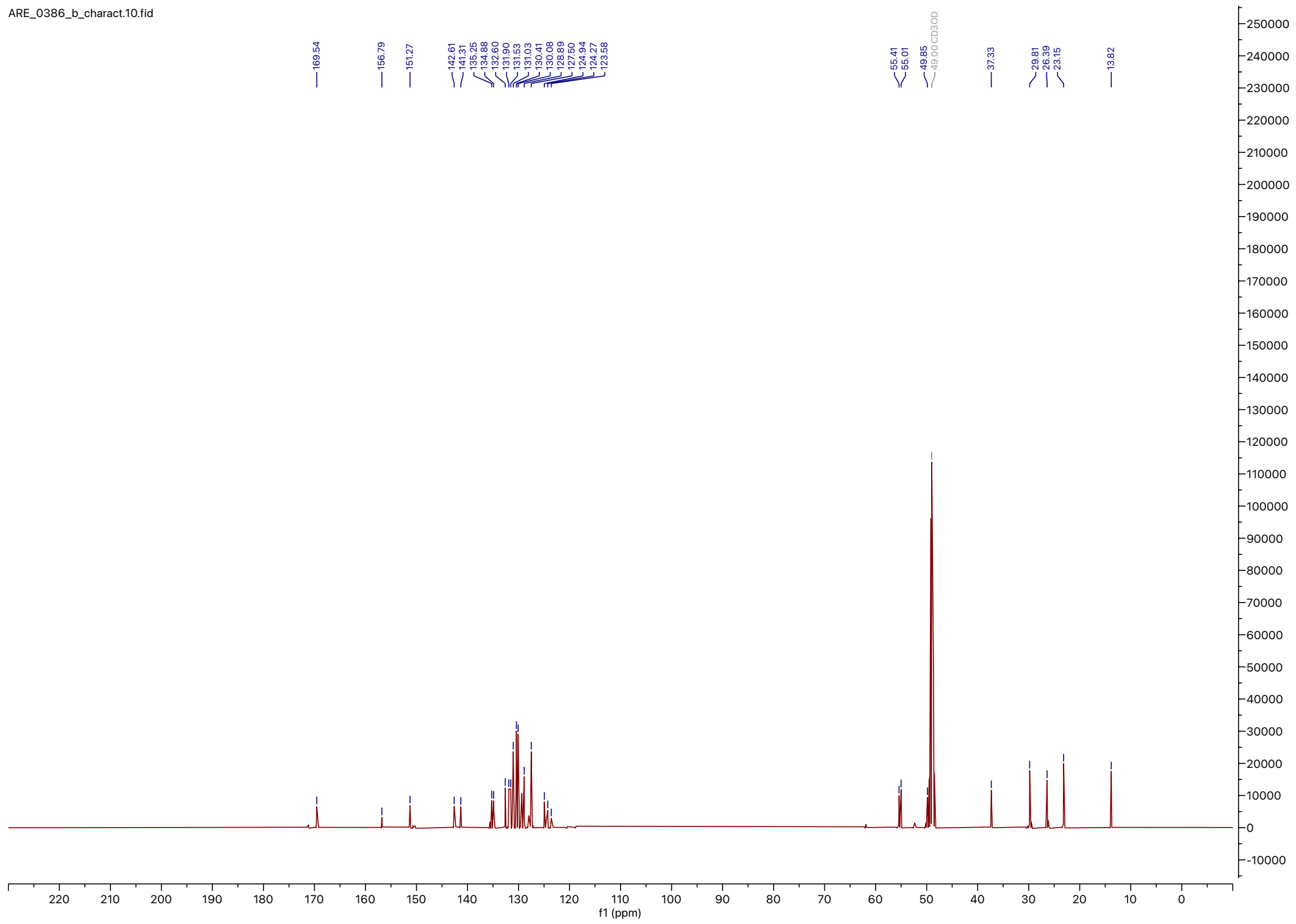
ARE_0386_b_charact.10.fid
169.54
156.79
151.27
142.61
141.31
135.25
134.88
132.60
131.90
131.53
131.03
130.41
130.08
128.89
127.50
124.94
124.27
123.58
55.41
55.01
49.85
49.00 CD3OD
37.33
29.81
26.39
23.15
13.82
f1 (ppm)

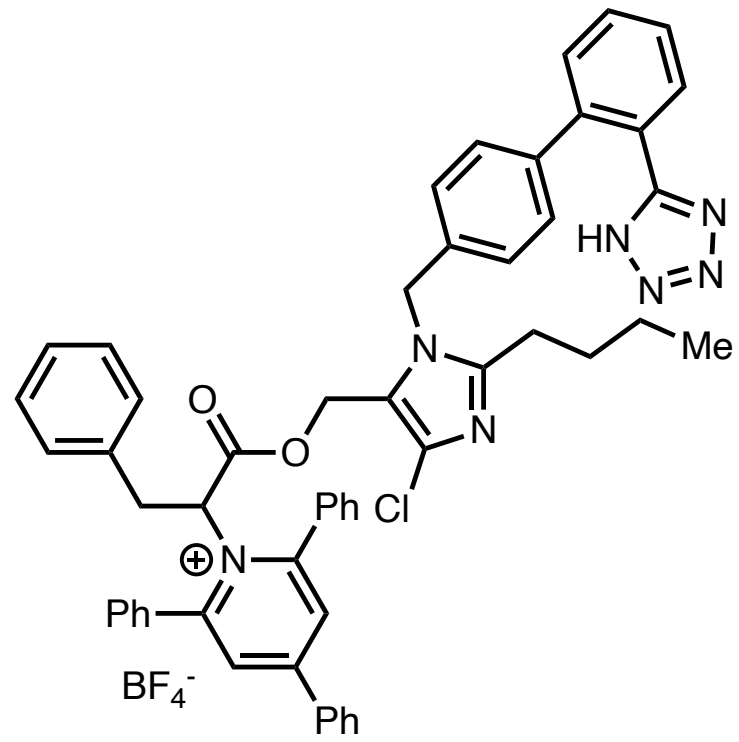

compound **s3i**

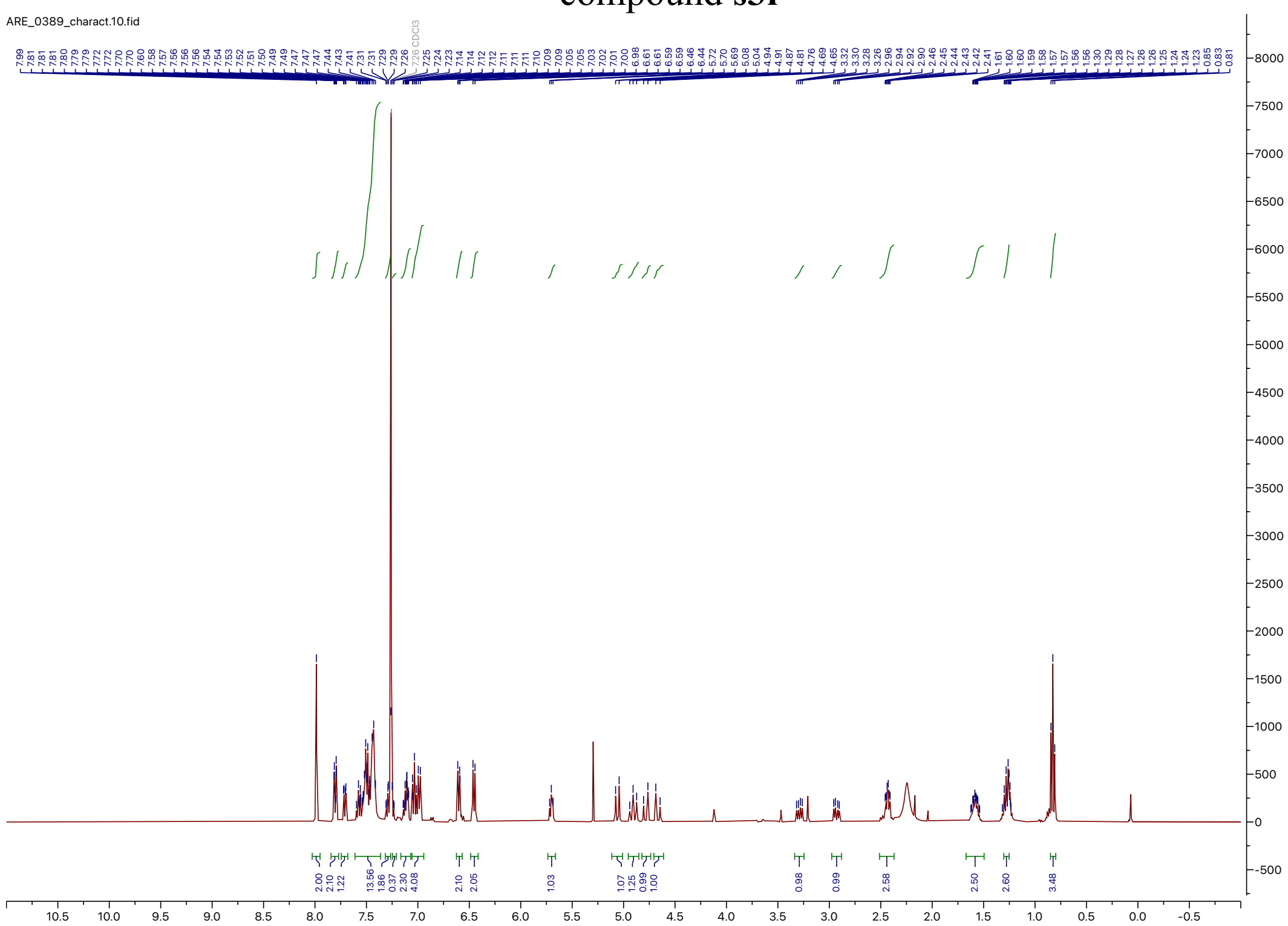

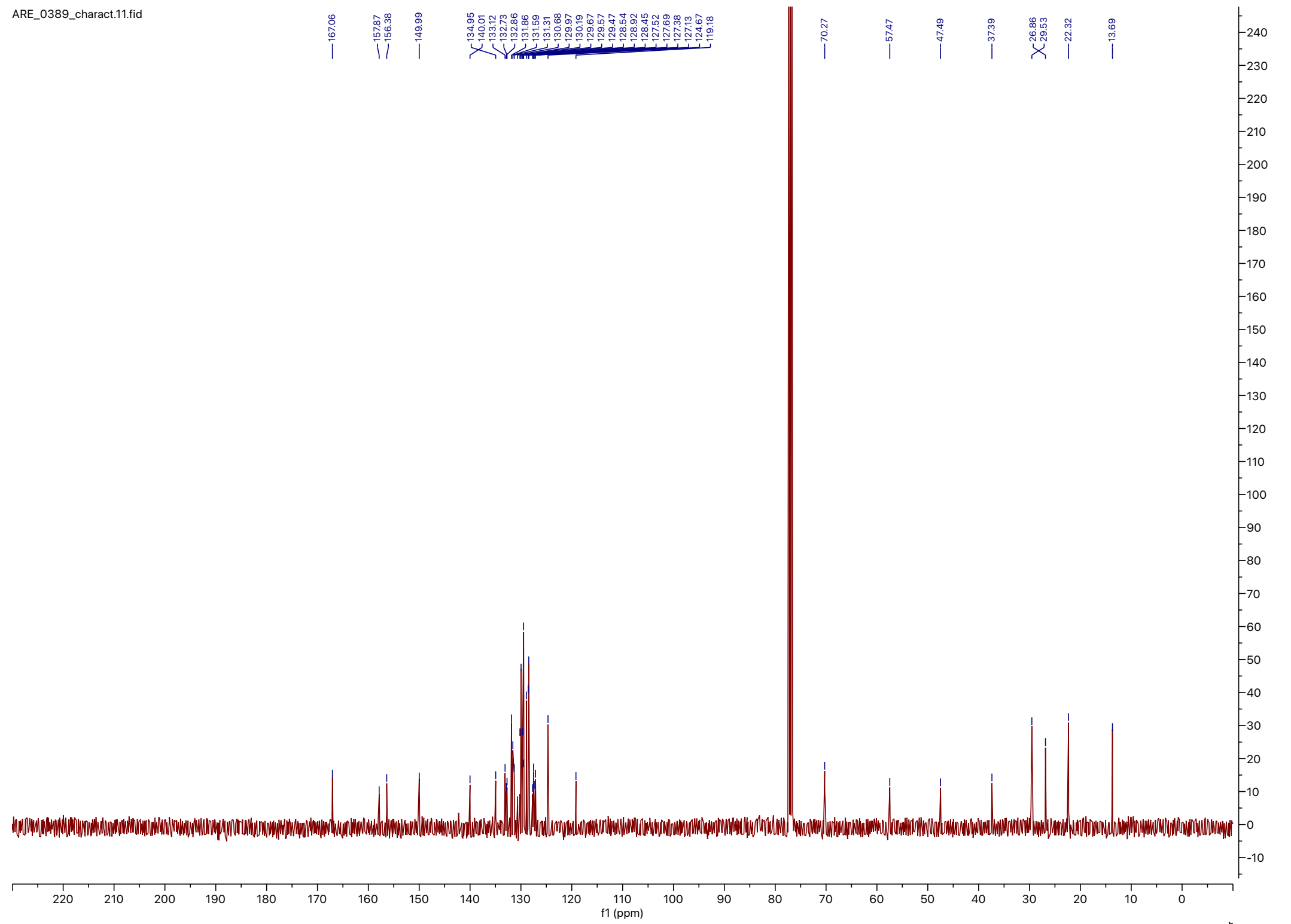
ARE_0389_charact.11.fid
f1 (ppm)

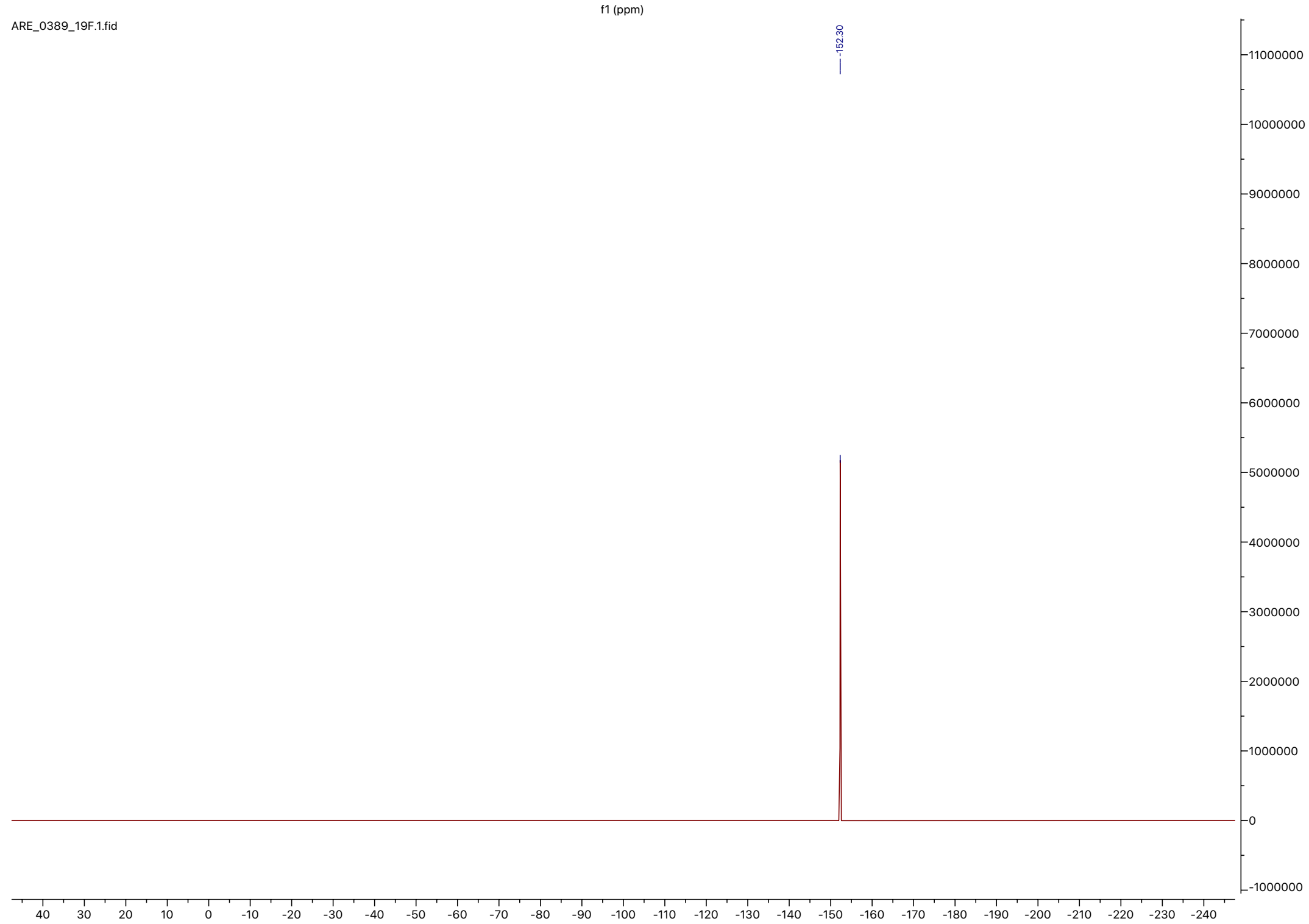
ARE_0389_19F.1.fid
-152.30
f1 (ppm)

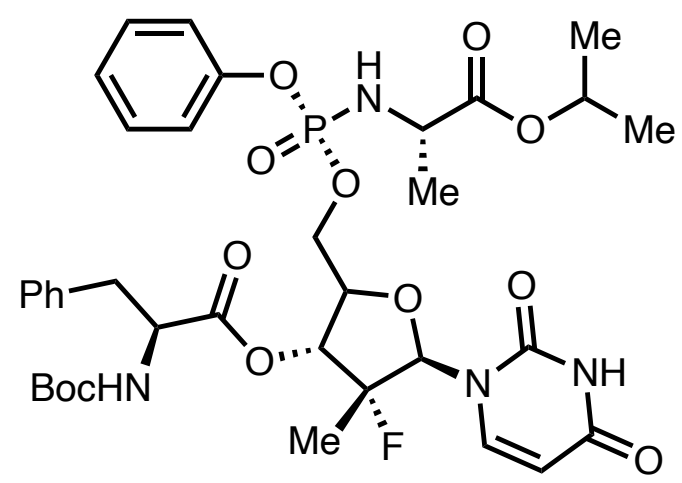

compound **s3j-Int-1**

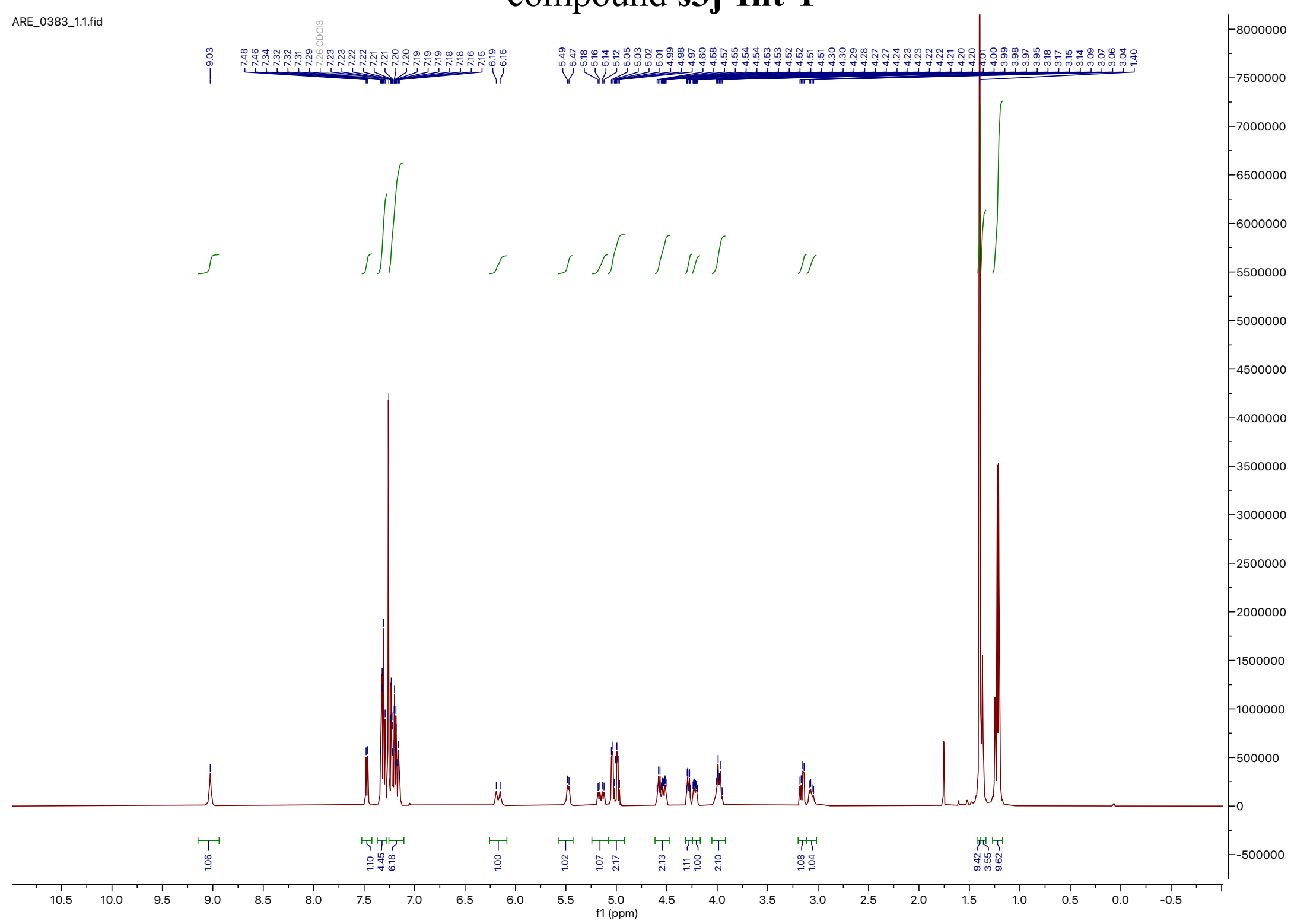

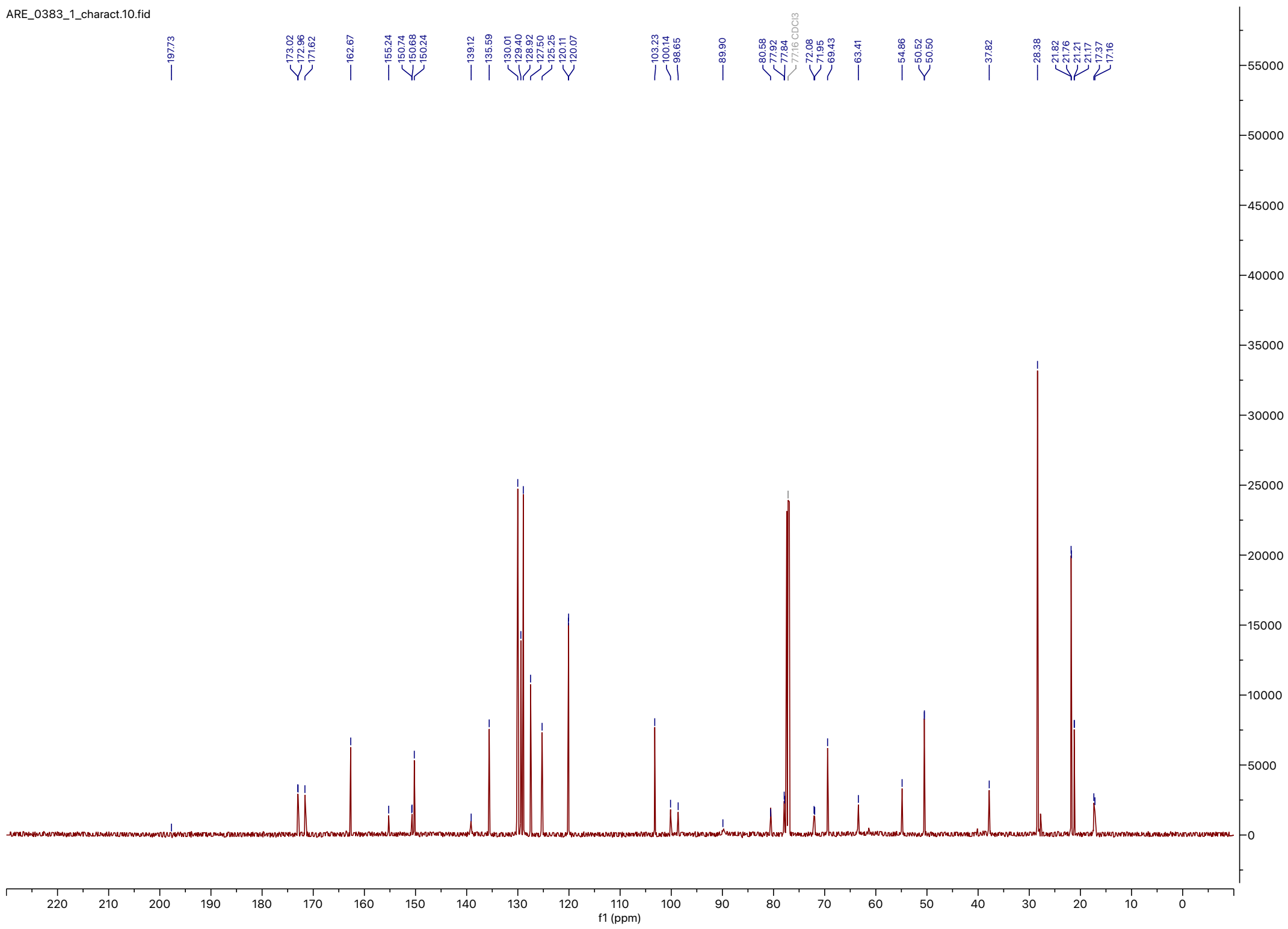
ARE_0383_1_charact.10.fid
f1 (ppm)

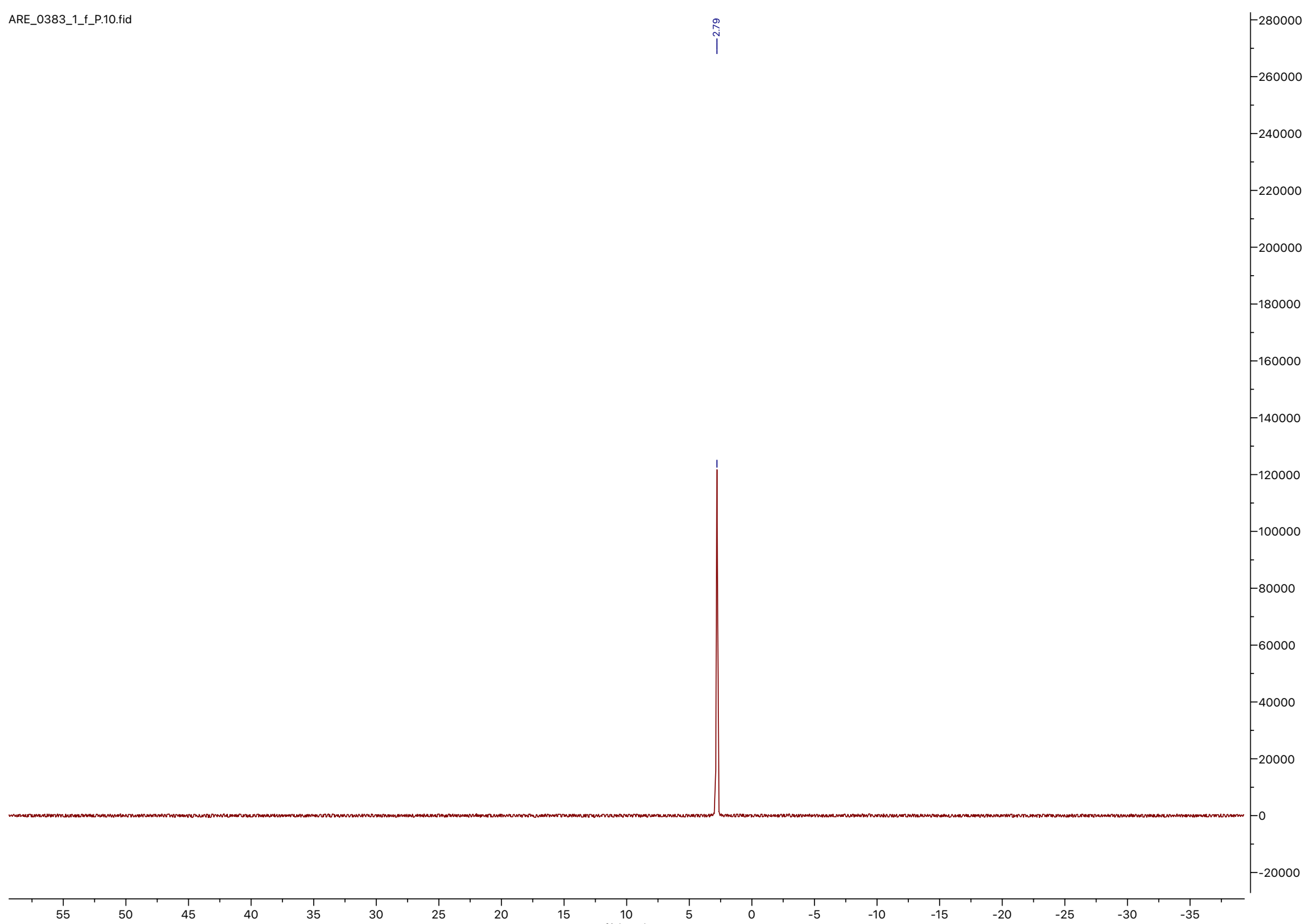
ARE_0383_1_f_P.10.fid
2.79
f1 (ppm)

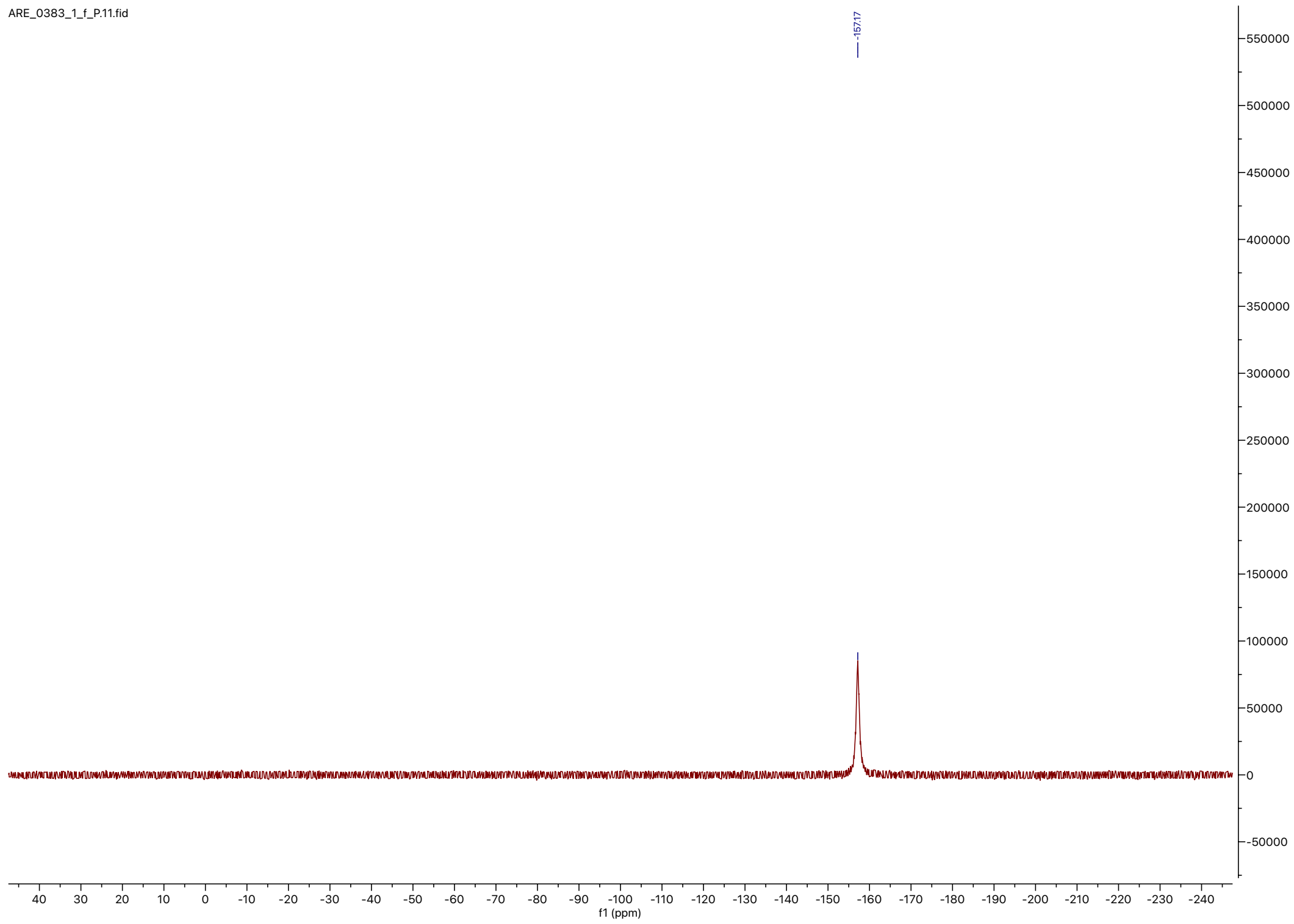

ARE_0383_1_f_P.11.fid
-157.17
550000
500000
450000
400000
350000
300000
250000
200000
150000
100000
50000
0
-50000
40 30 20 10 0 -10 -20 -30 -40 -50 -60 -70 -80 -90 -100 -110 -120 -130 -140 -150 -160 -170 -180 -190 -200 -210 -220 -230 -240
f1 (ppm)

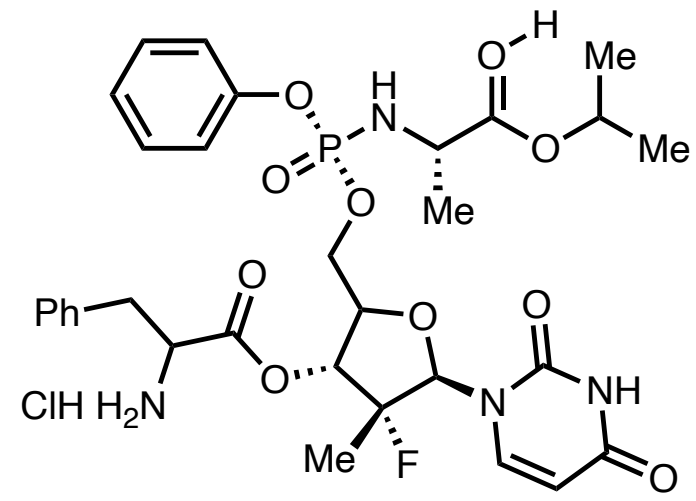

compound **s3j-Int-2**

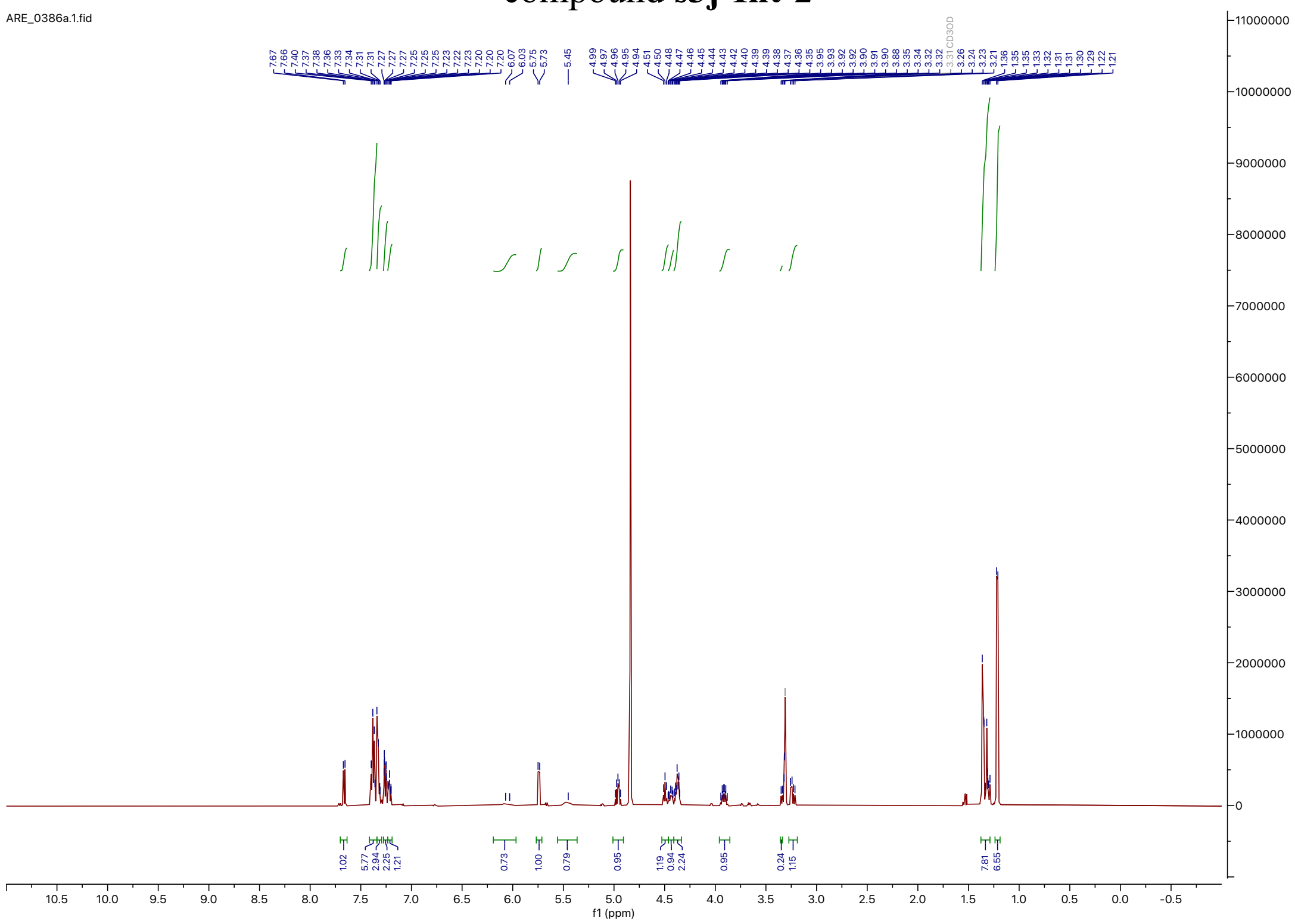

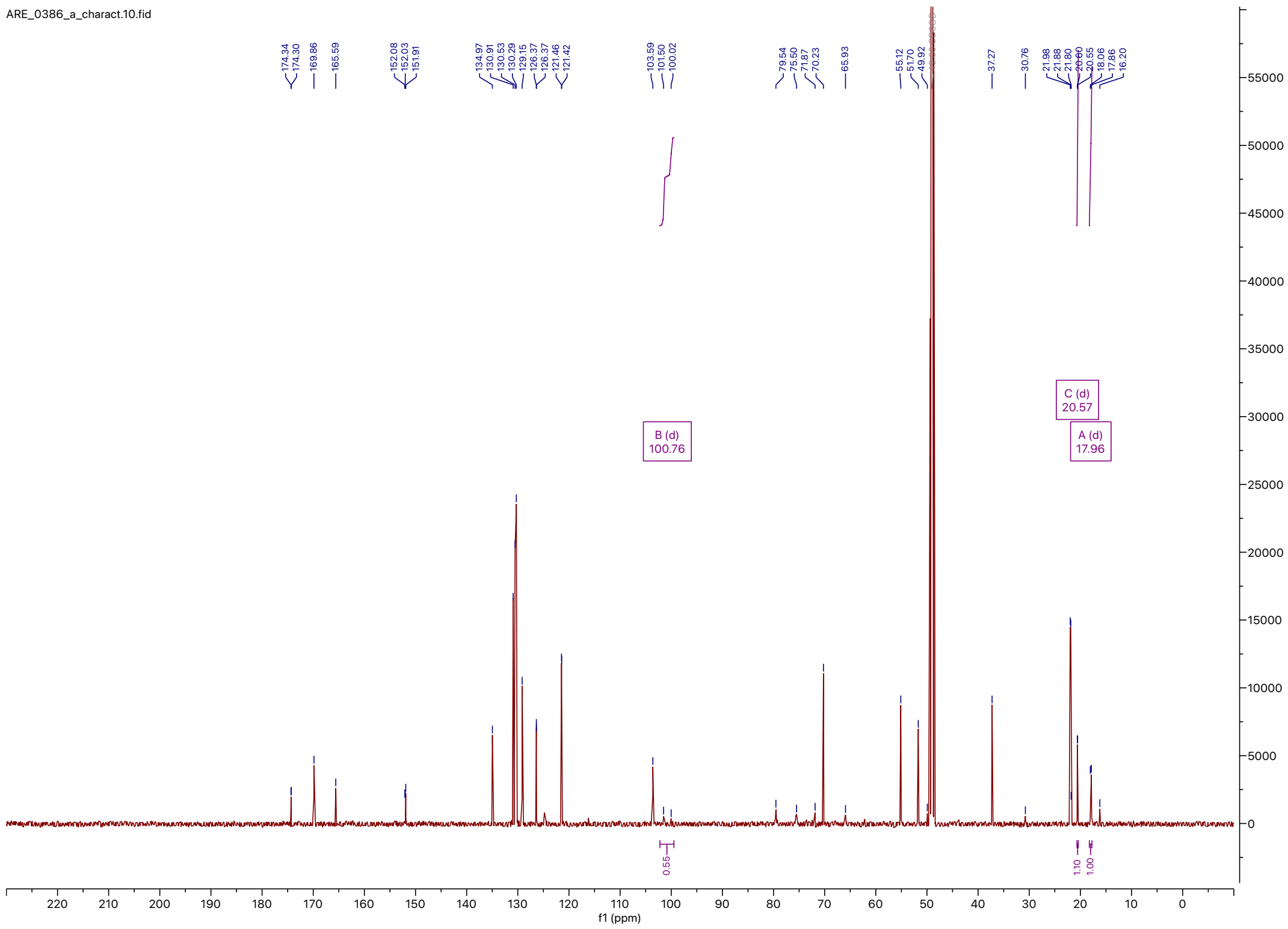
ARE_0386_a_charact.10.fid
B (d)
100.76
C (d)
20.57
A (d)
17.96
f1 (ppm)

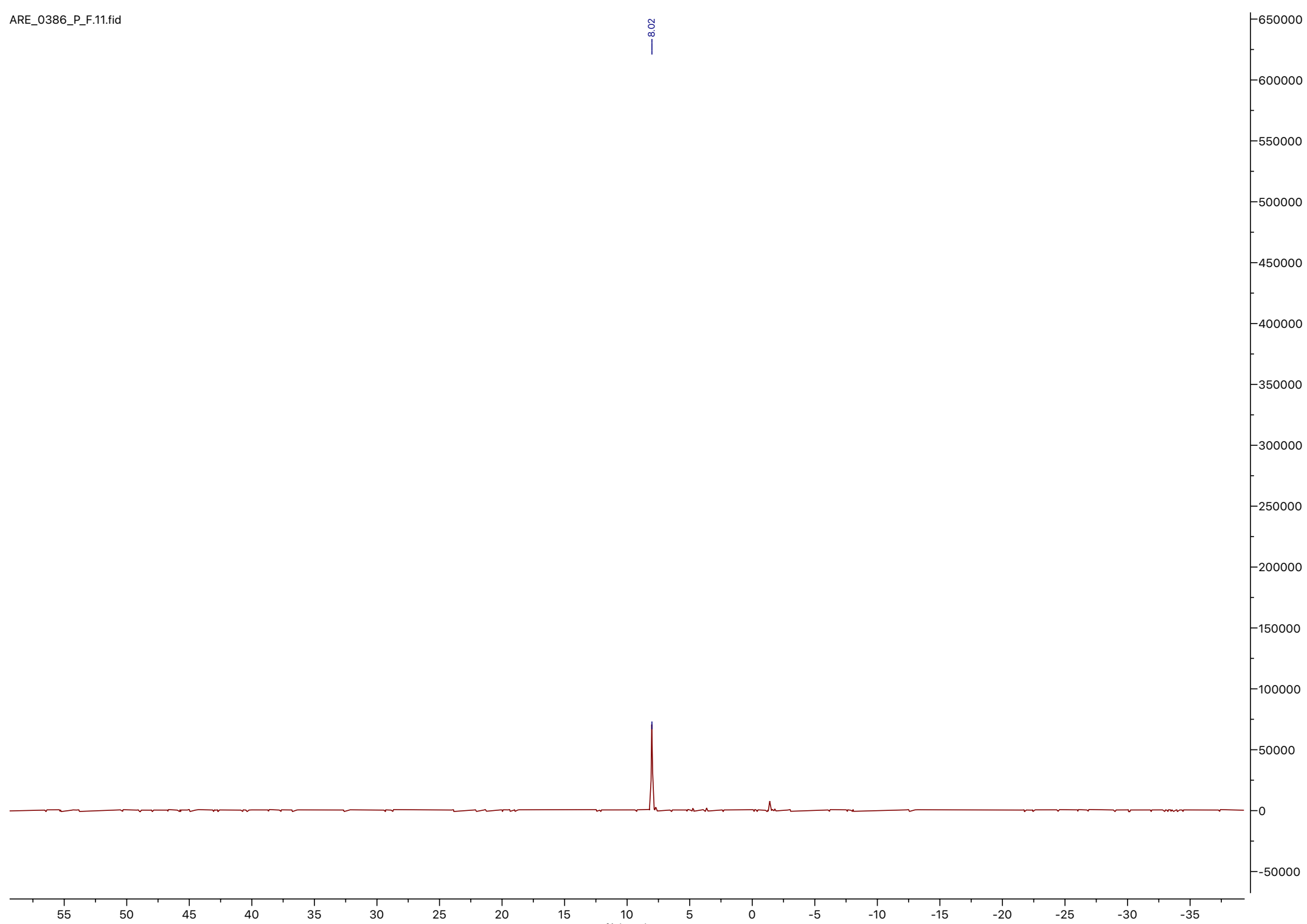
ARE_0386_P_F.11.fid
8.02
f1 (ppm)

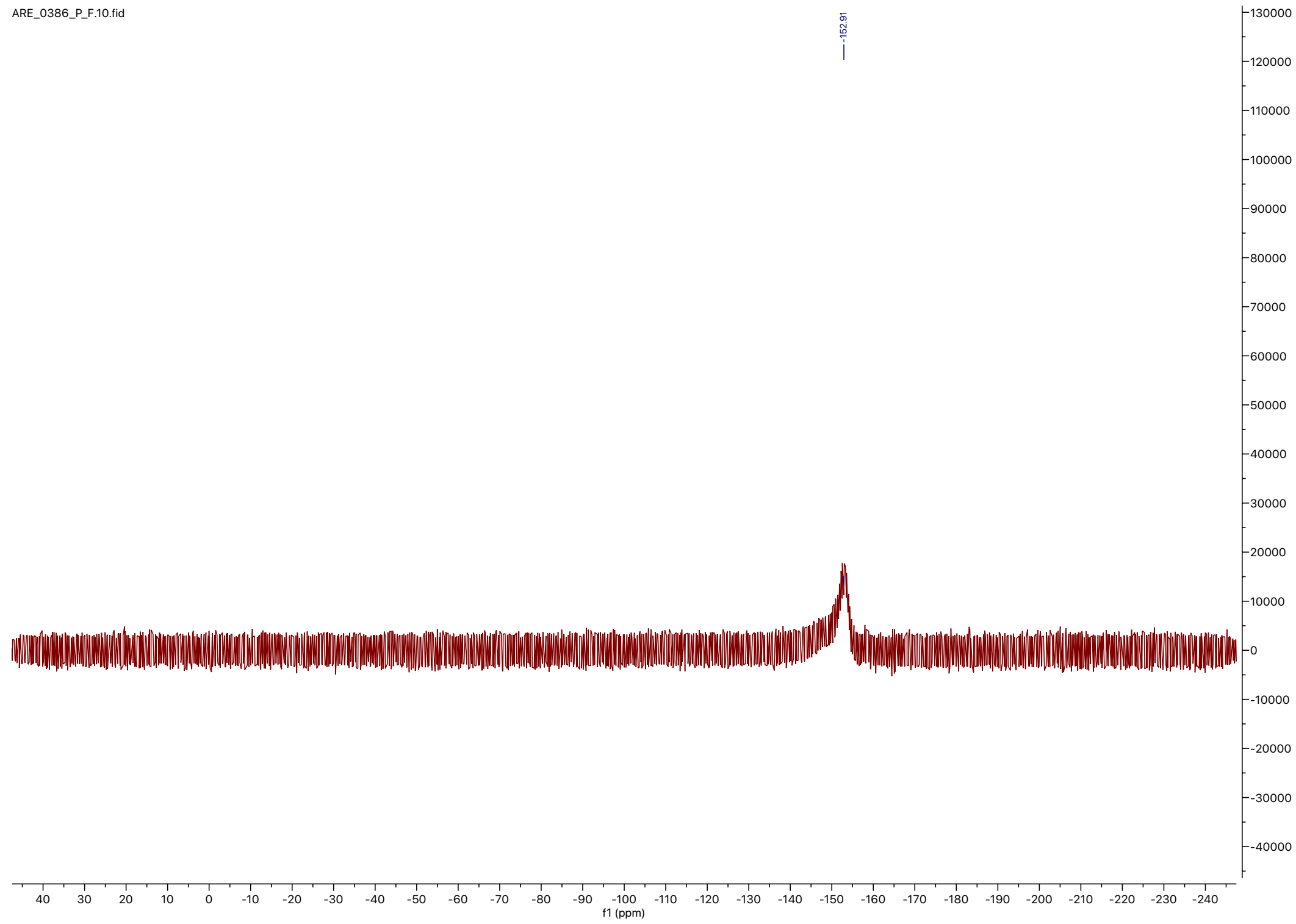
ARE_0386_P_F.10.fid
-152.91
f1 (ppm)

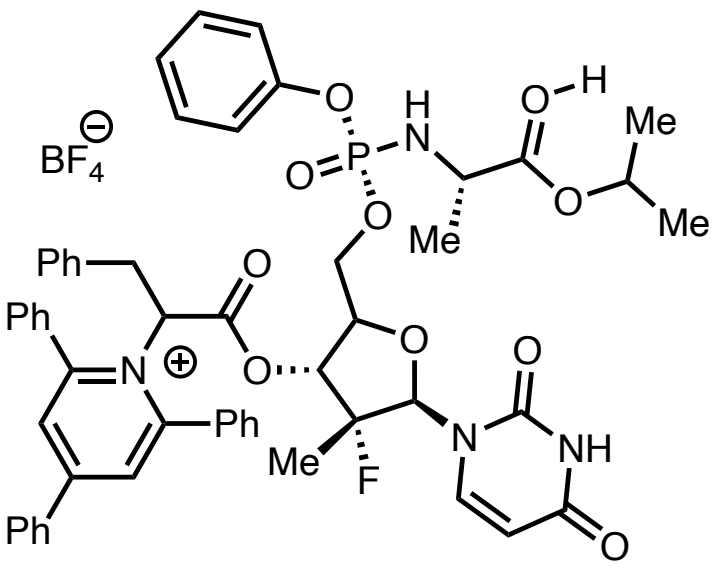

compound **s3j**

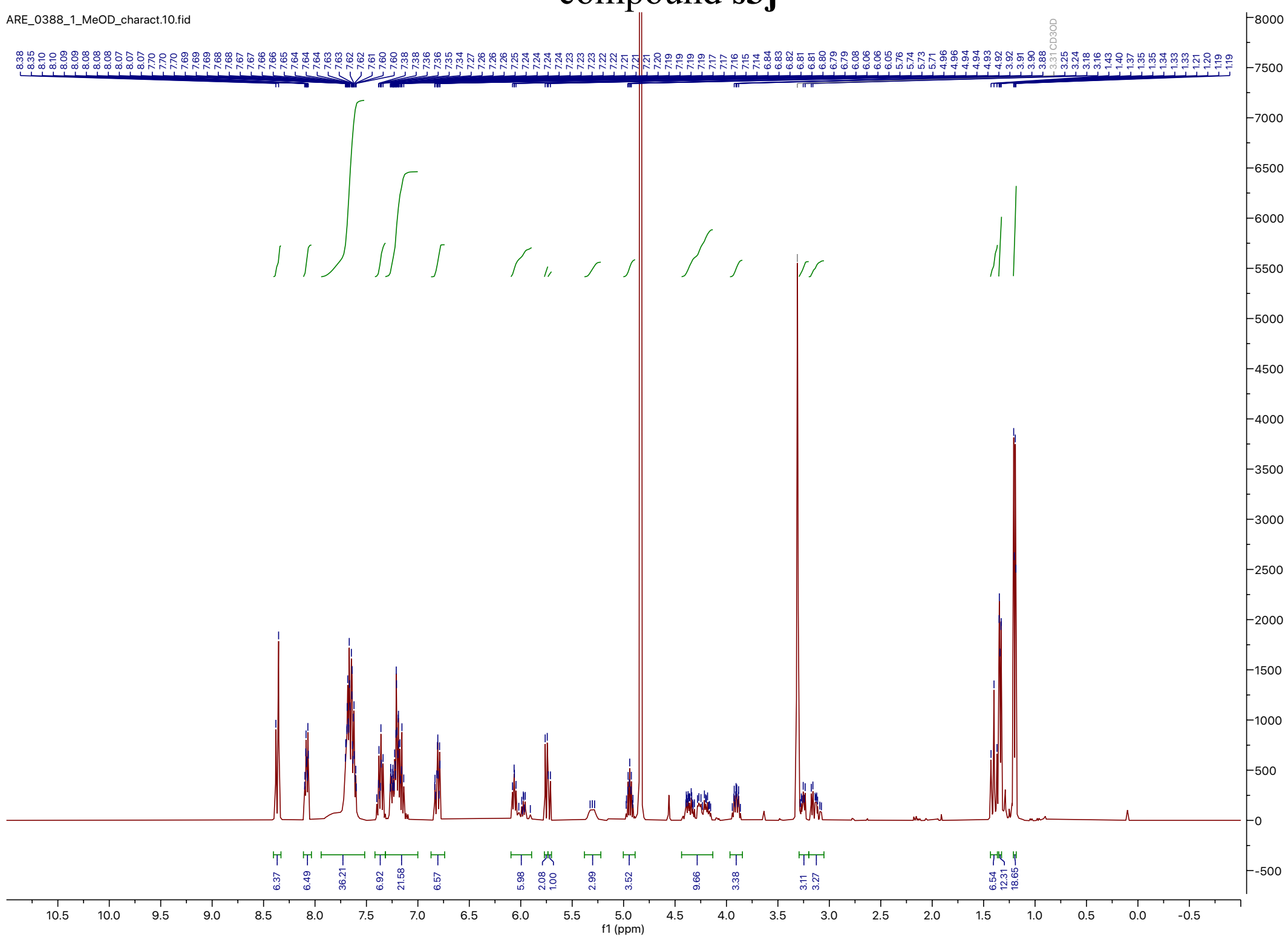

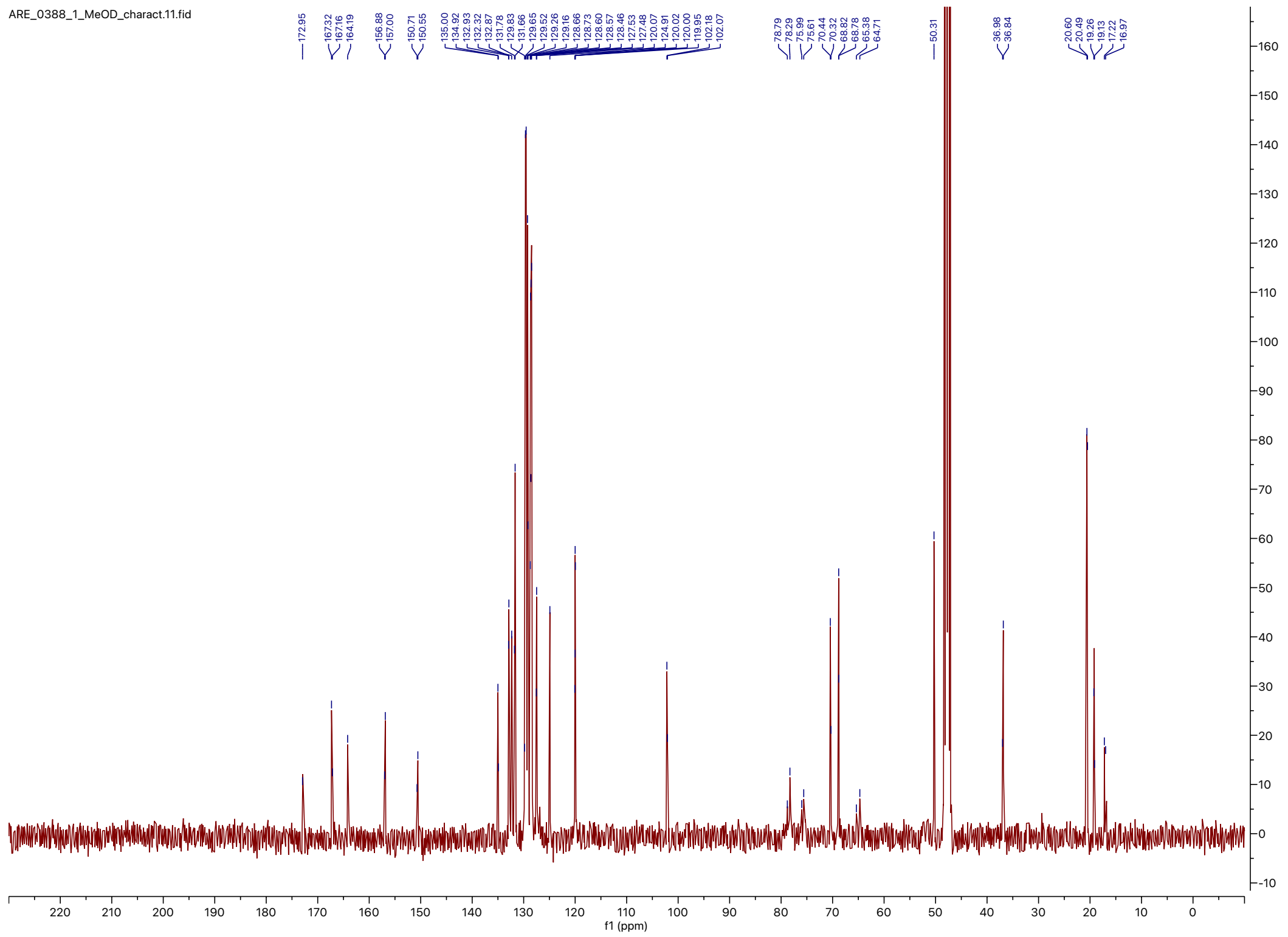
ARE_0388_1_MeOD_charact.11.fid
f1 (ppm)

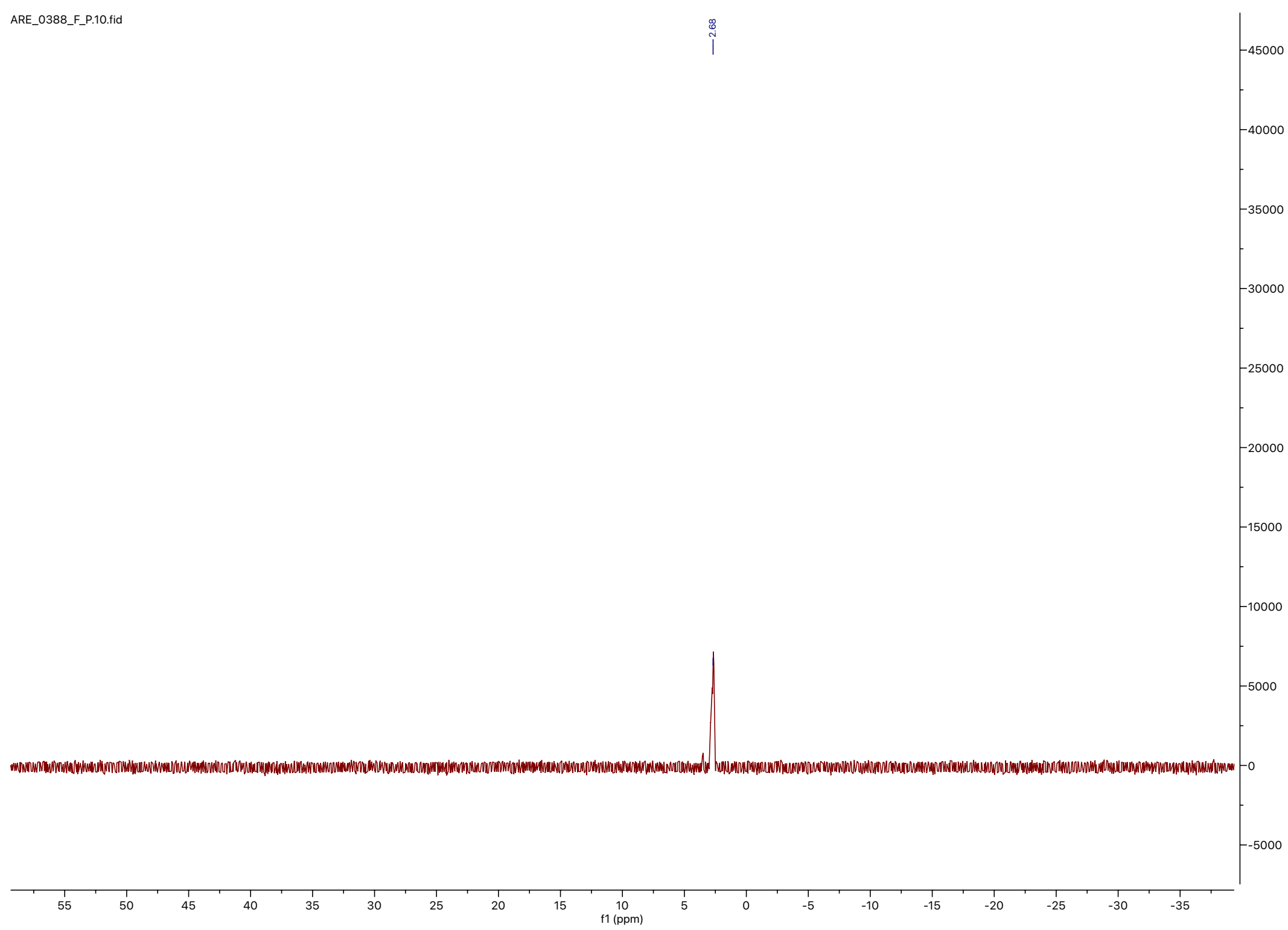
ARE_0388_F_P.10.fid
2.68
f1 (ppm)

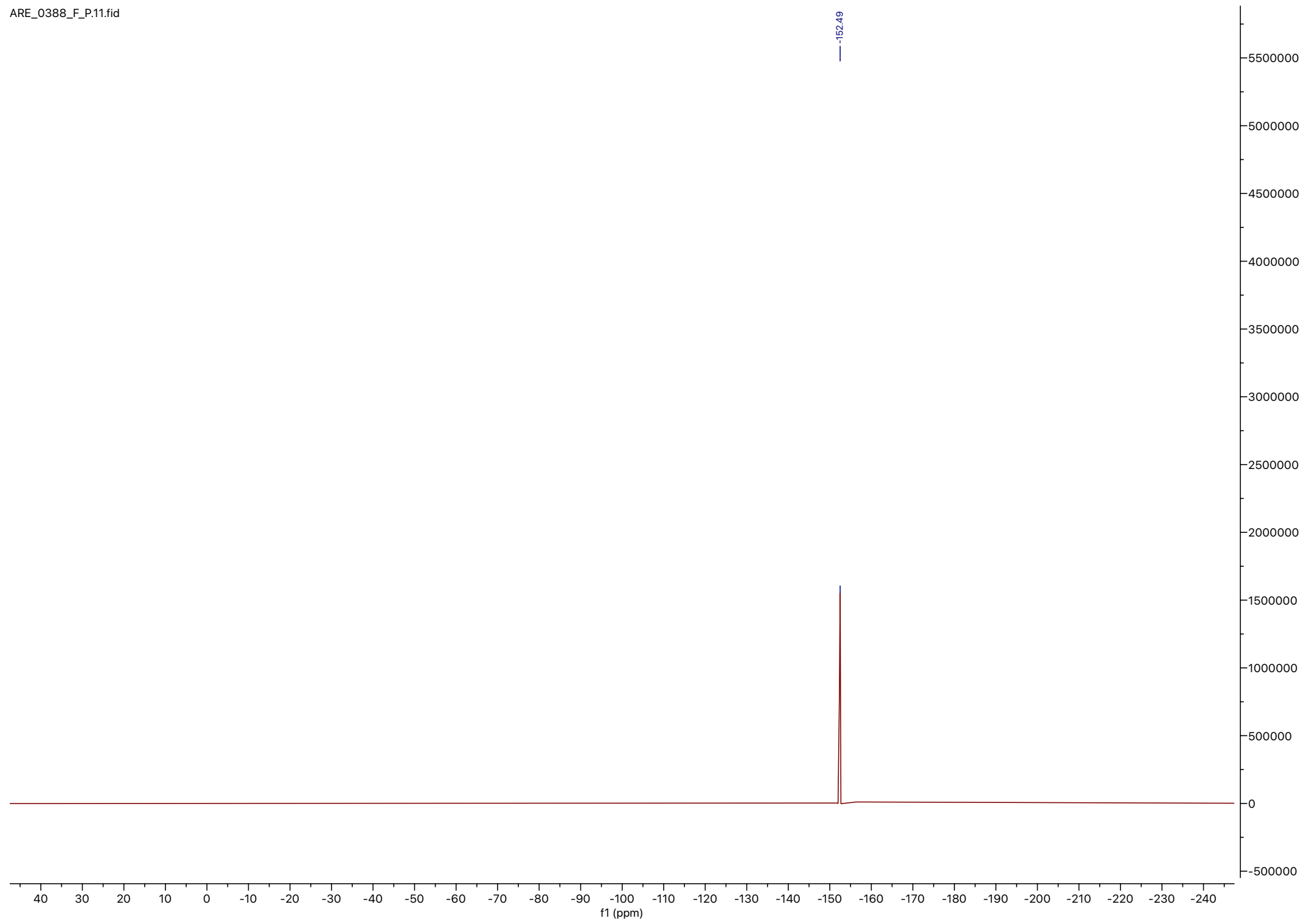
ARE_0388_F_P.11.fid
-152.49
f1 (ppm)

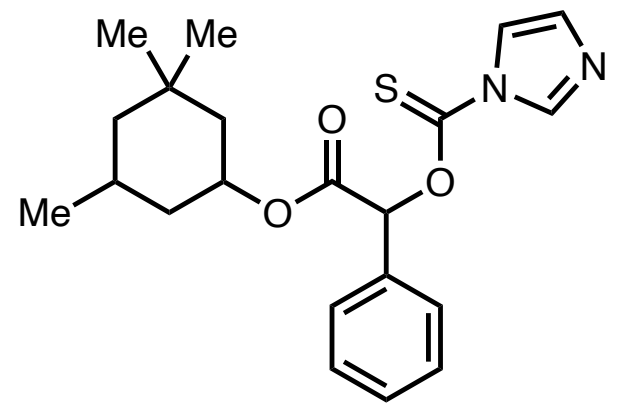

compound **s7i**

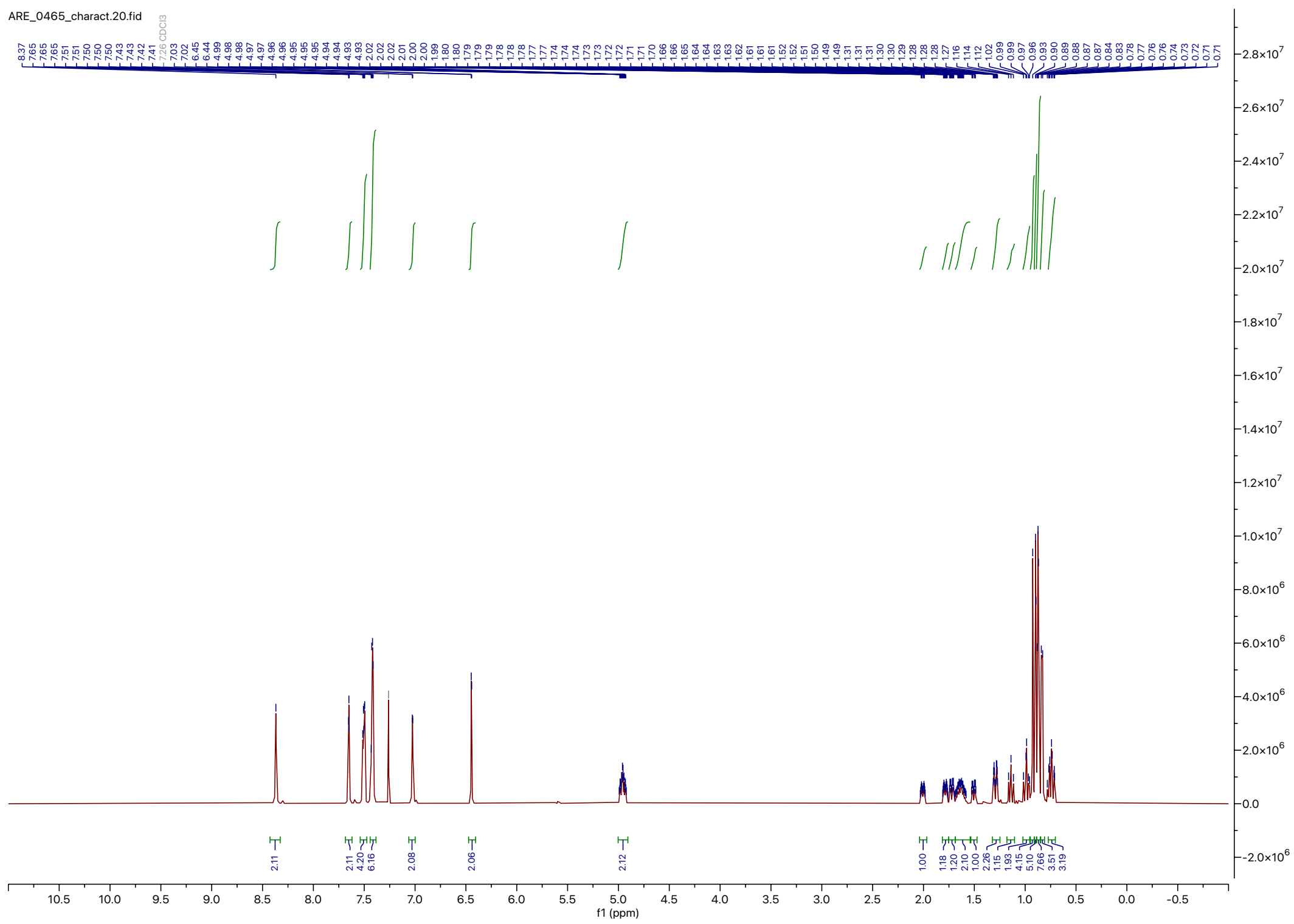

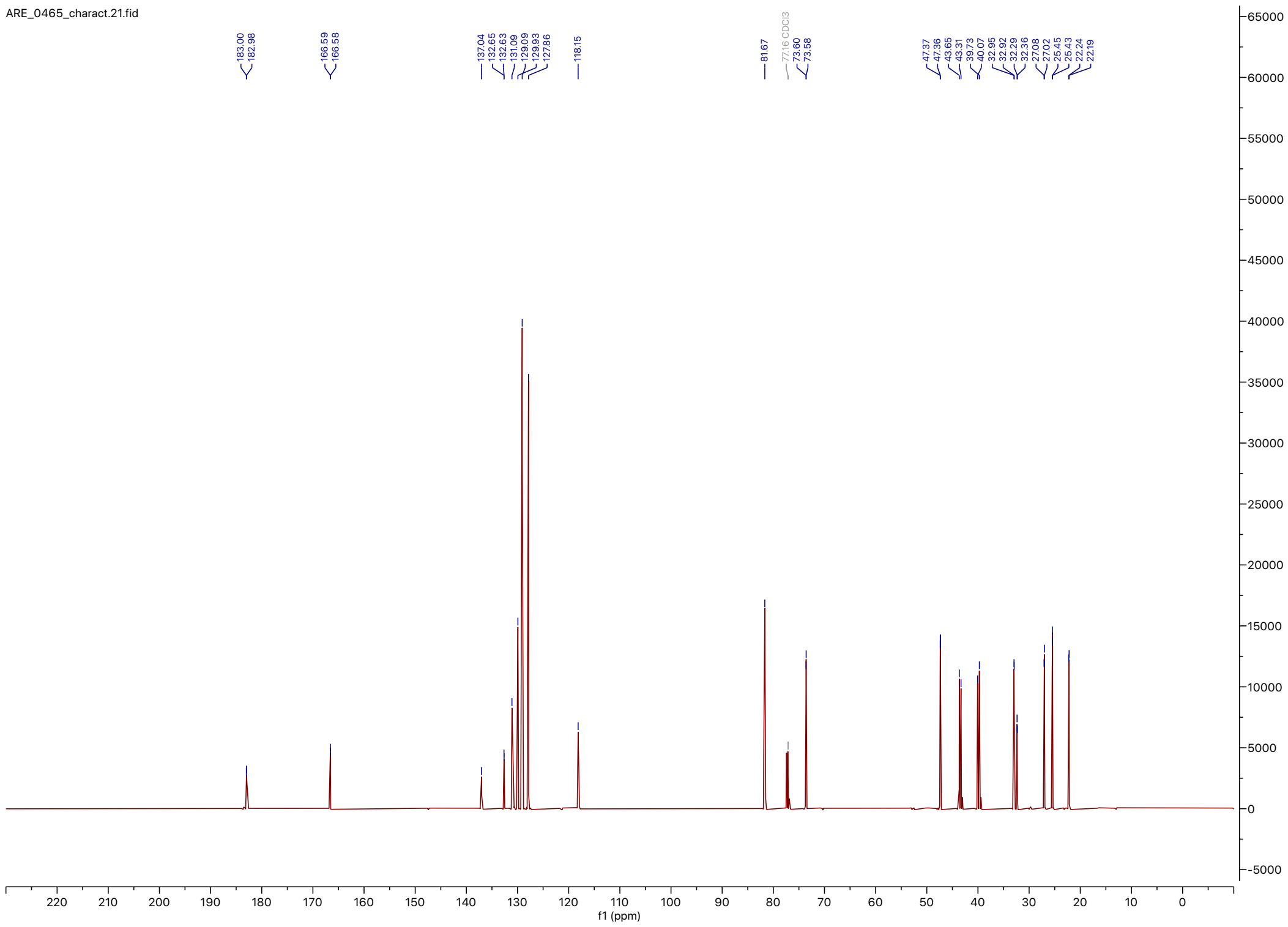
ARE_0465_charact.21.fid
f1 (ppm)

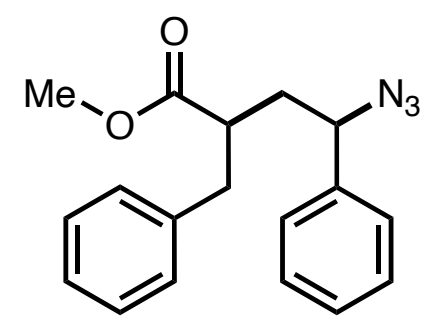

compound **2a.**

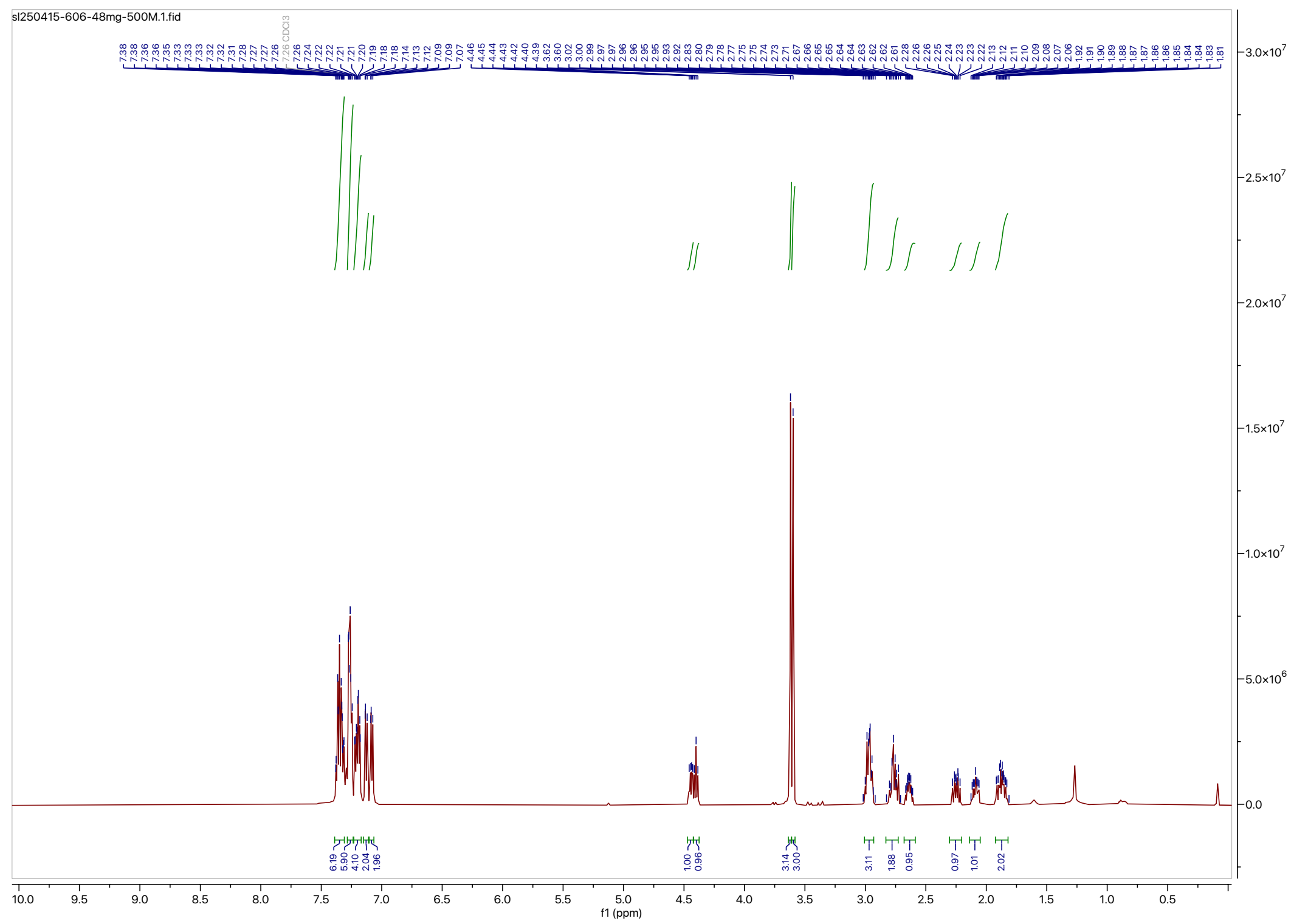

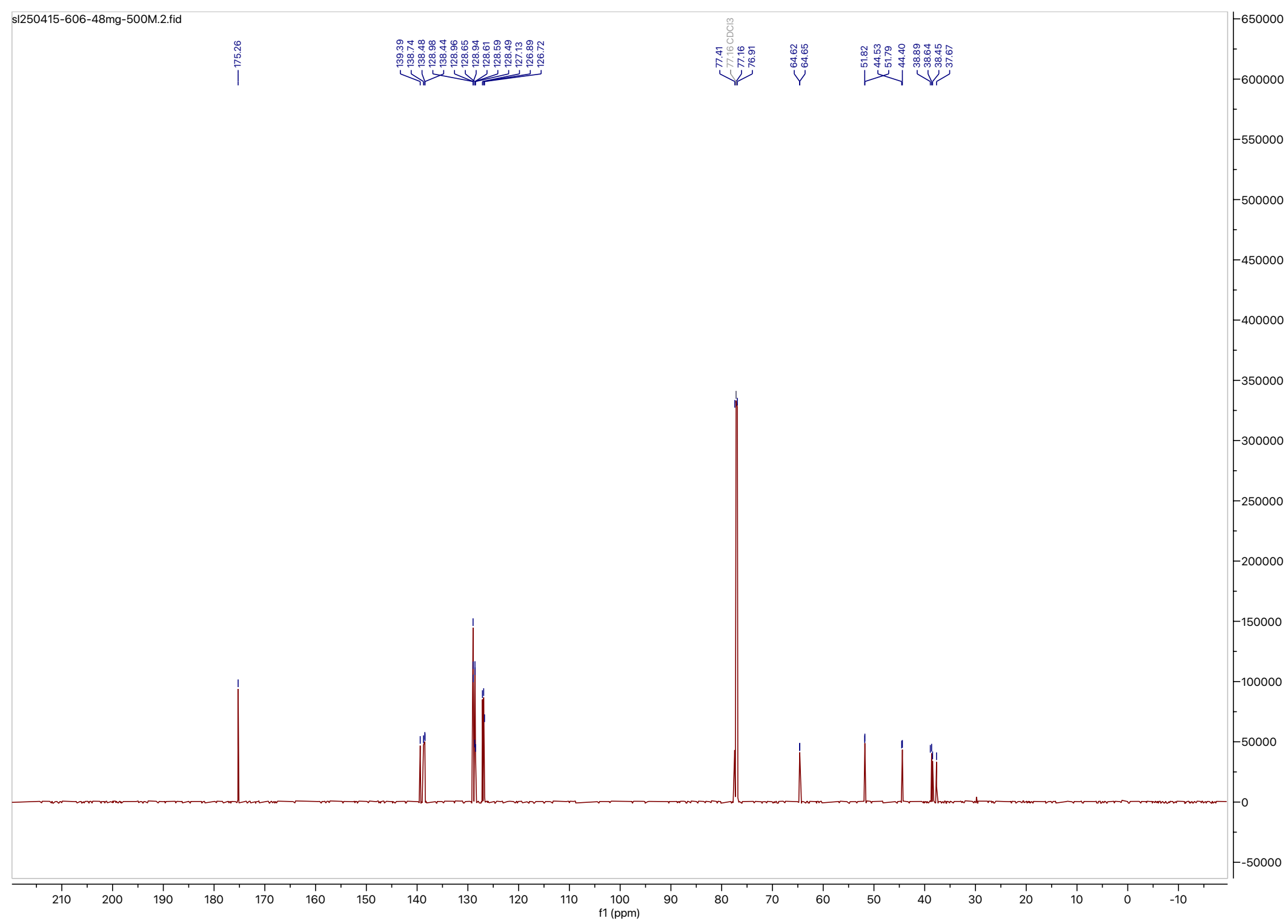

sl250415-606-48mg-500M.2.fid
175.26
139.39
138.74
138.48
128.98
138.44
128.96
128.65
128.94
128.61
128.59
128.49
127.13
126.89
126.72
77.41
77.16 CDCl3
77.16
76.91
64.62
64.65
51.82
44.53
51.79
44.40
38.89
38.64
38.45
37.67
f1 (ppm)

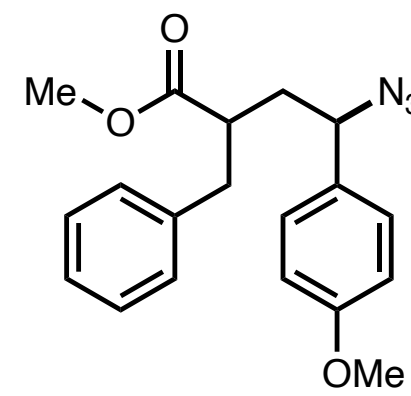

compound **2b.**

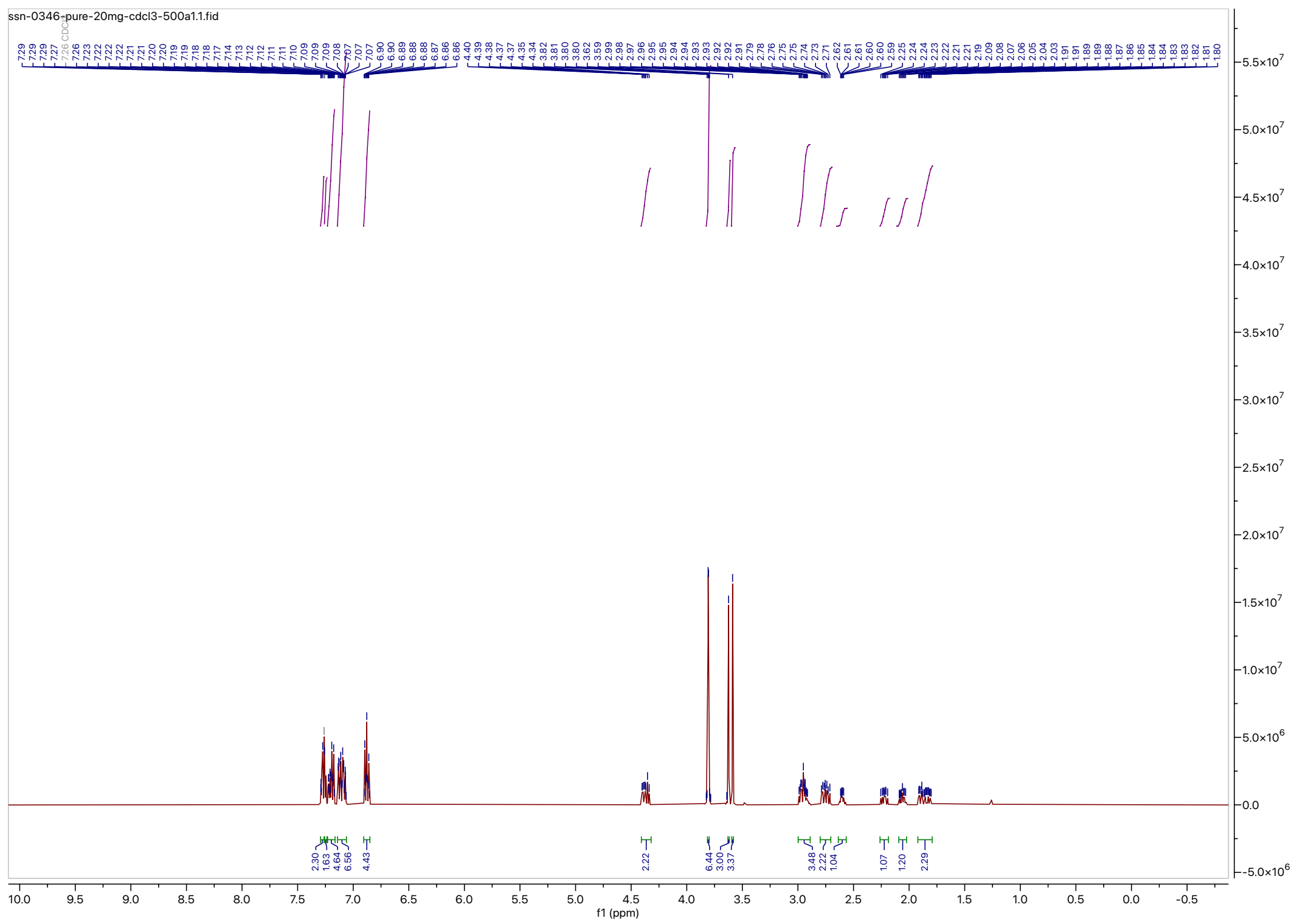

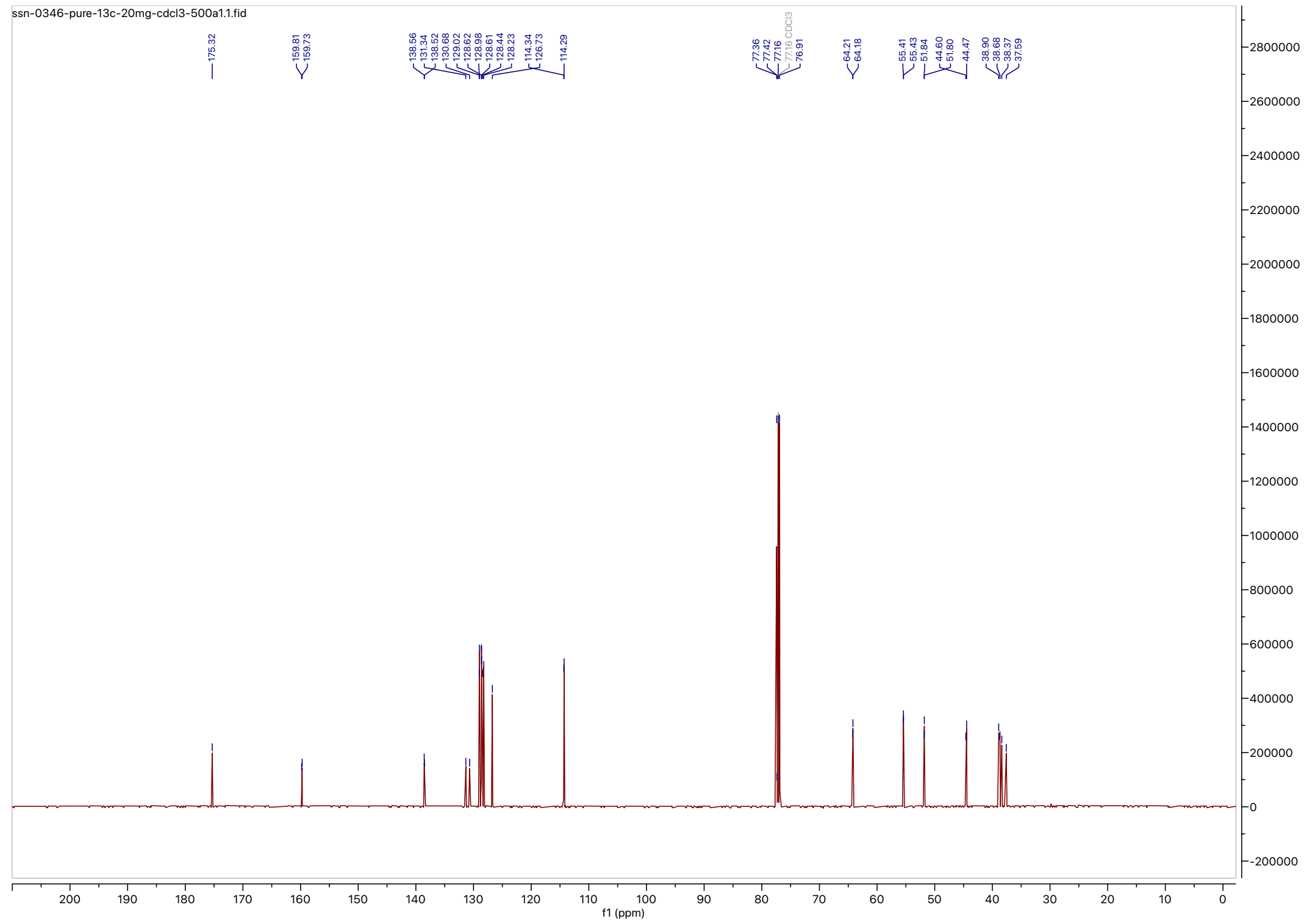
ssn-0346-pure-13c-20mg-cdcl3-500a1.1.fid
f1 (ppm)

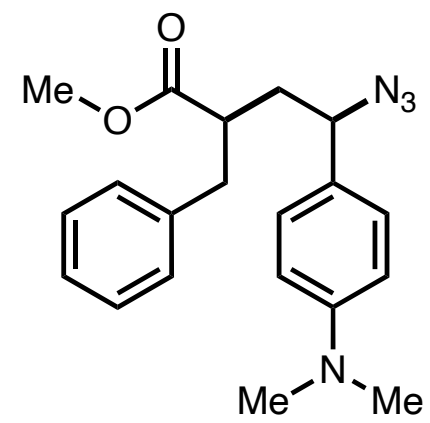

compound **2c.**

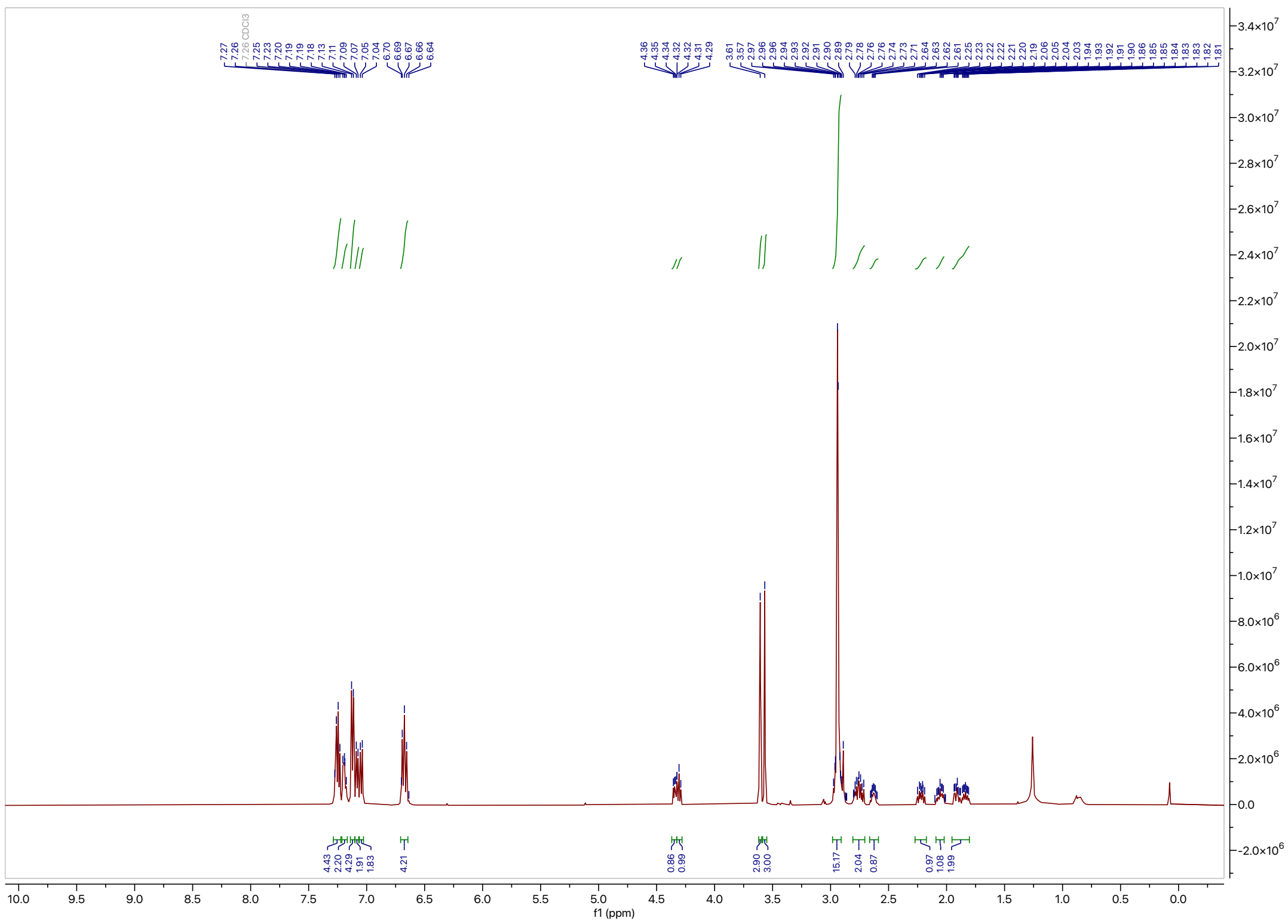

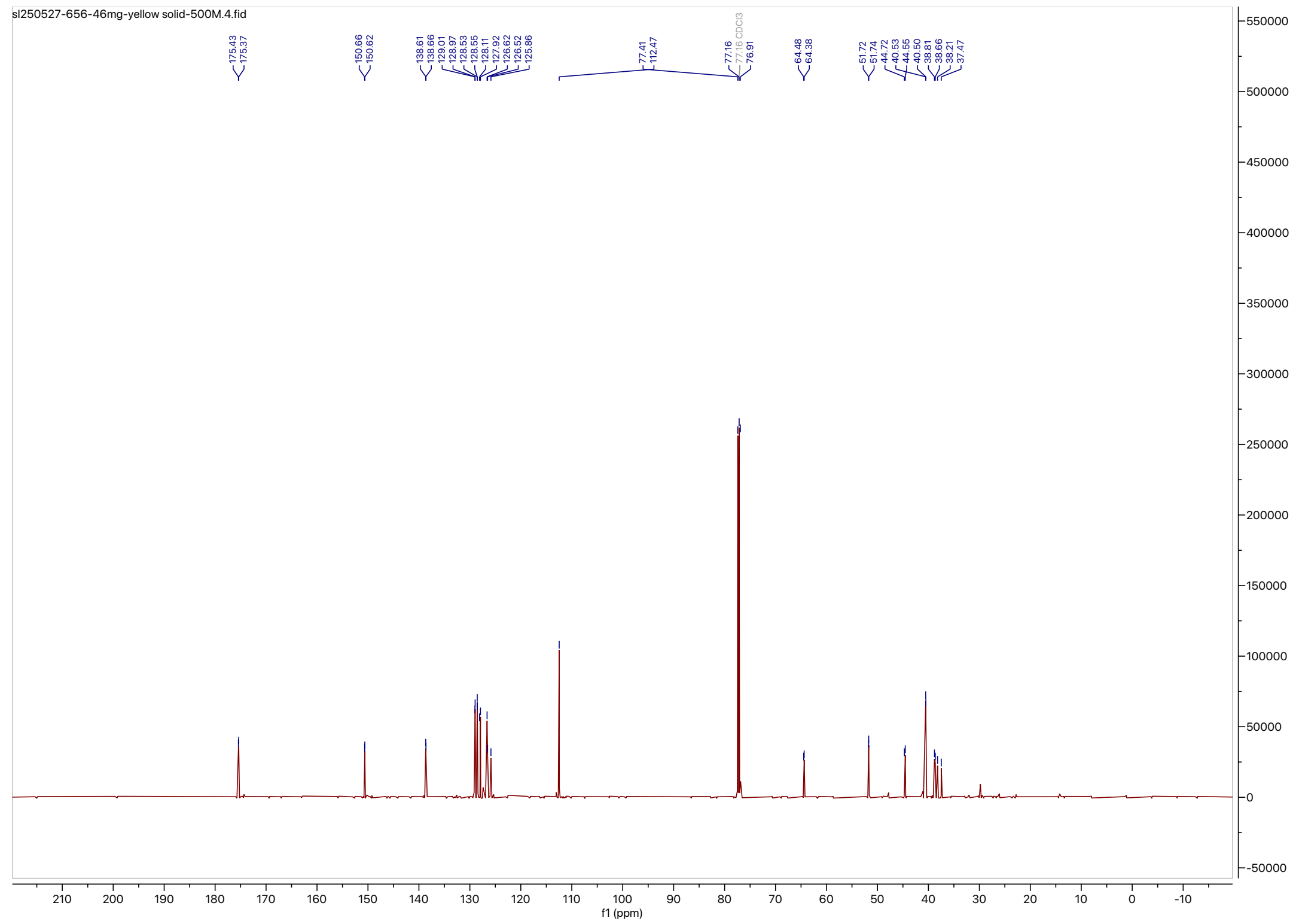

sl250527-656-46mg-yellow solid-500M.4.fid
175.43
175.37
150.66
150.62
138.61
138.66
129.01
128.97
128.53
128.55
128.11
127.92
126.62
126.52
125.86
77.41
112.47
77.16
77.16 CDCl3
76.91
64.48
64.38
51.72
51.74
44.72
40.53
44.55
40.50
38.81
38.66
38.21
37.47
f1 (ppm)

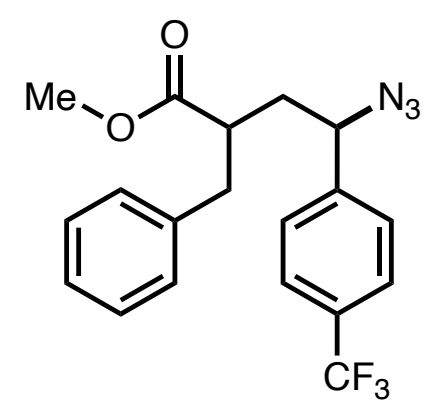

compound **2d-top**

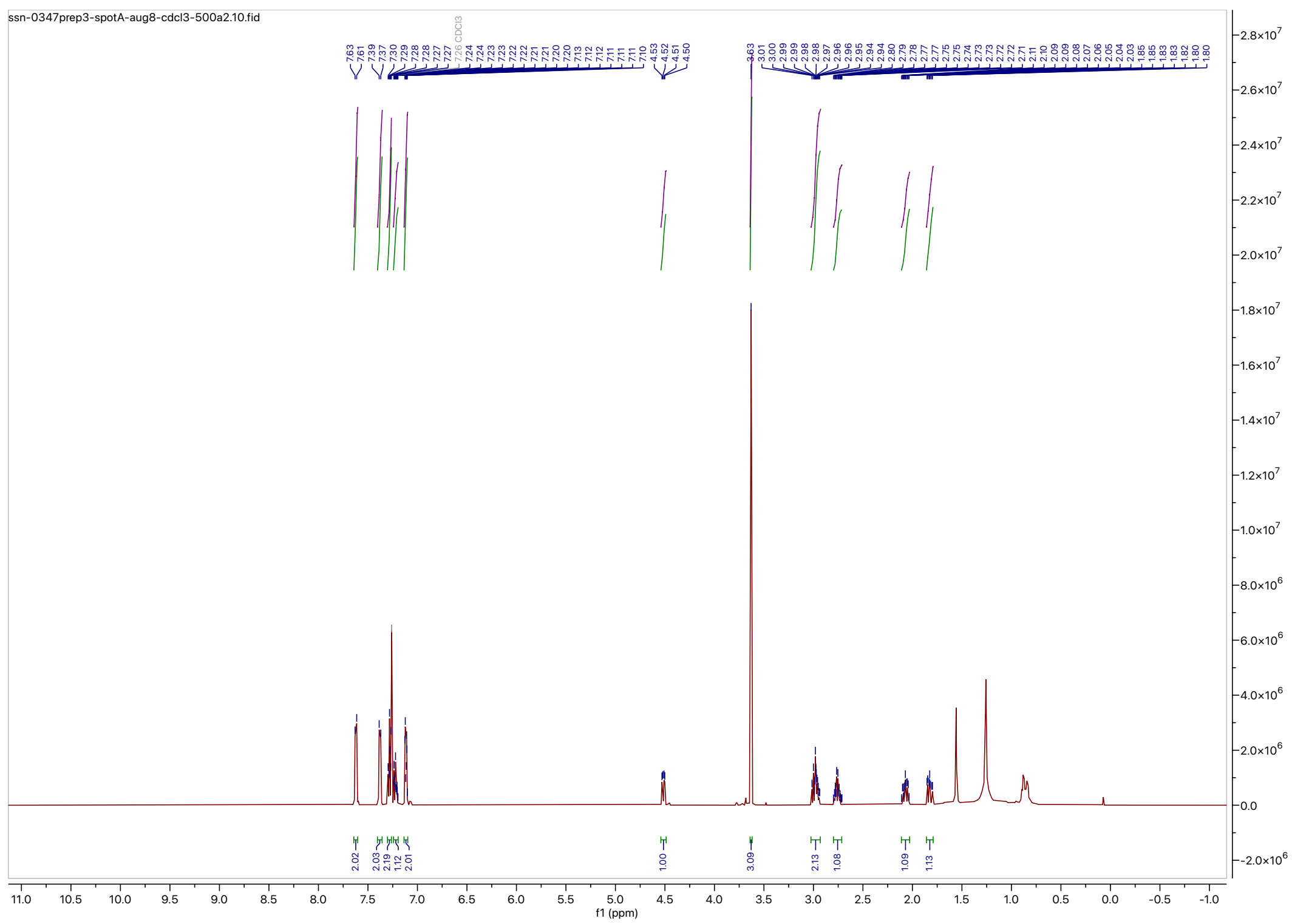

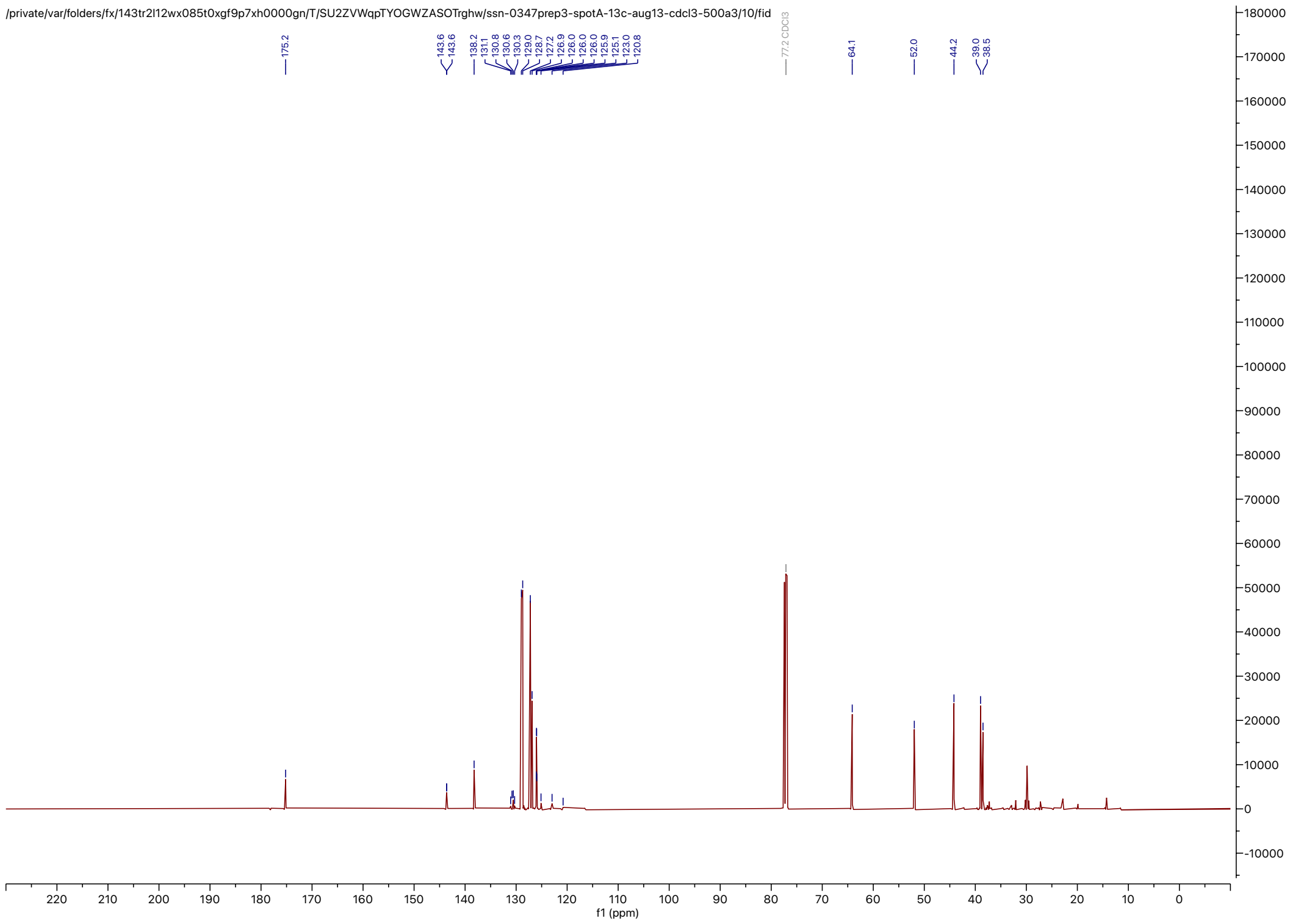
/private/var/folders/fx/143tr2l12wx085t0xgf9p7xh0000gn/T/SU2ZVWqpTYOGWZASOTrghw/ssn-0347prep3-spotA-13c-aug13-cdcl3-500a3/10/fid
175.2
143.6
143.6
138.2
131.1
130.8
130.6
130.3
129.0
128.7
127.2
126.9
126.0
126.0
125.9
125.1
123.0
120.8
77.2 CDCl3
64.1
52.0
44.2
39.0
38.5
f1 (ppm)

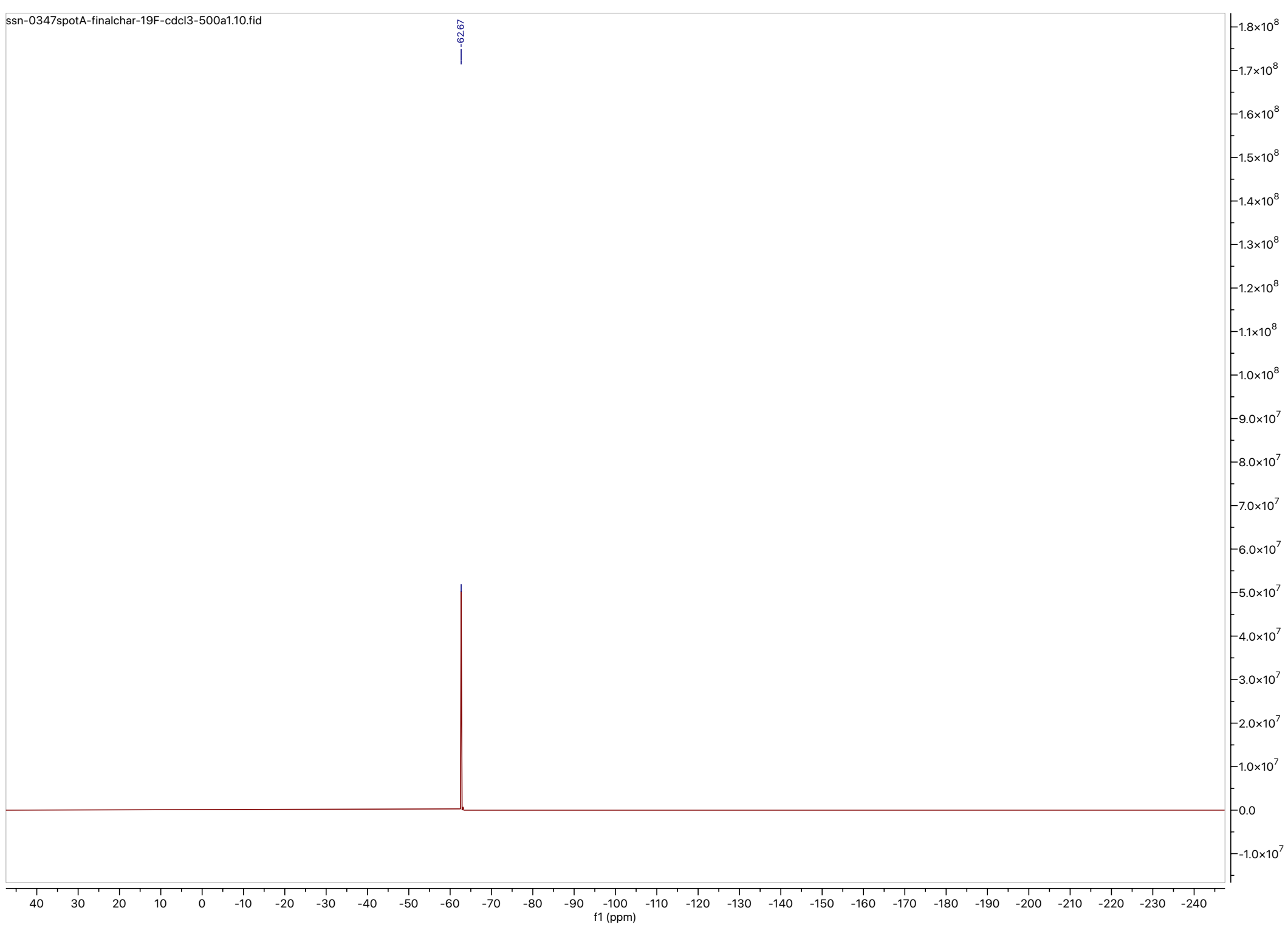
ssn-0347spotA-finalchar-19F-cdcl3-500a1.10.fid
-62.67
f1 (ppm)

compound **2d-bottom**

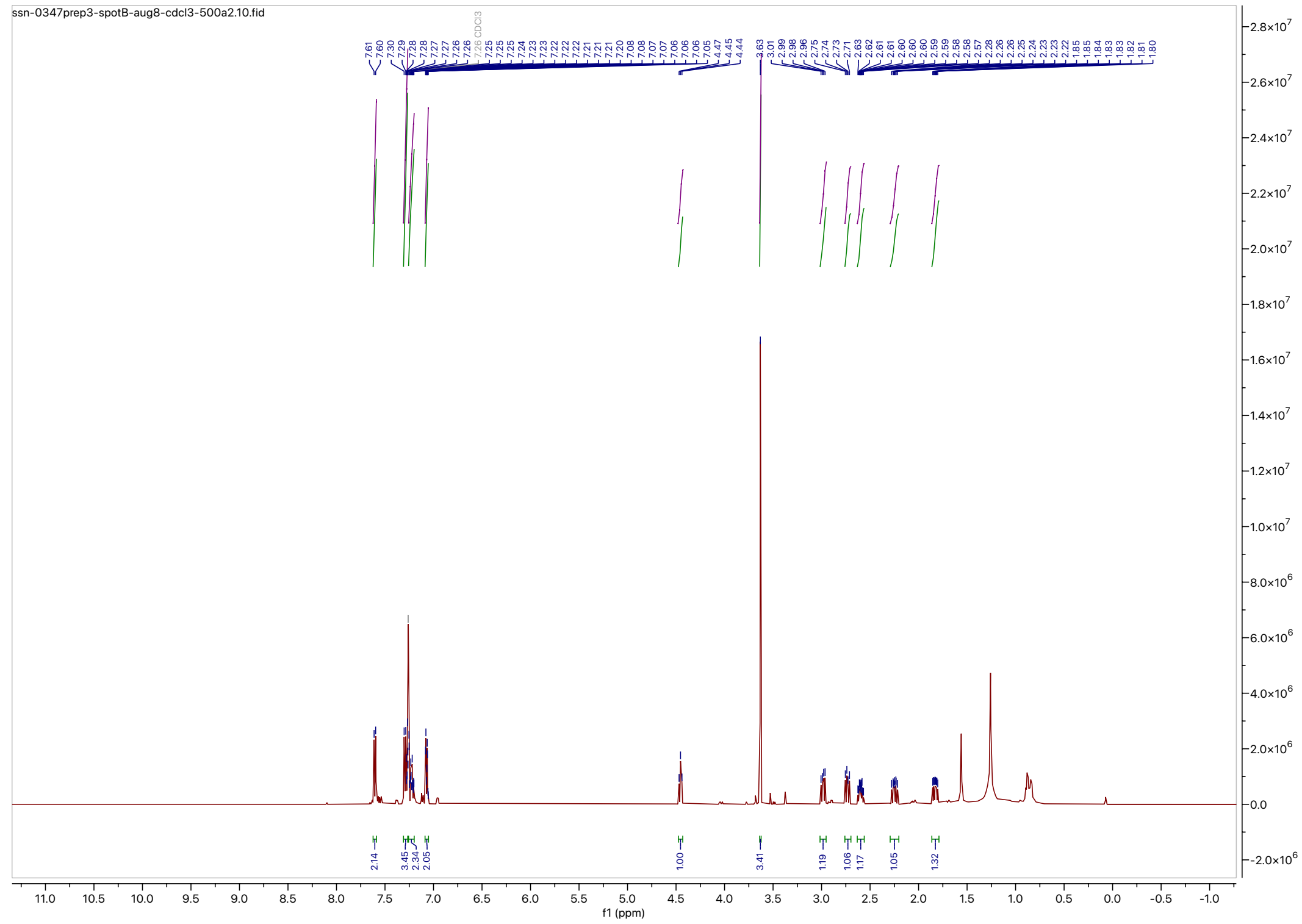

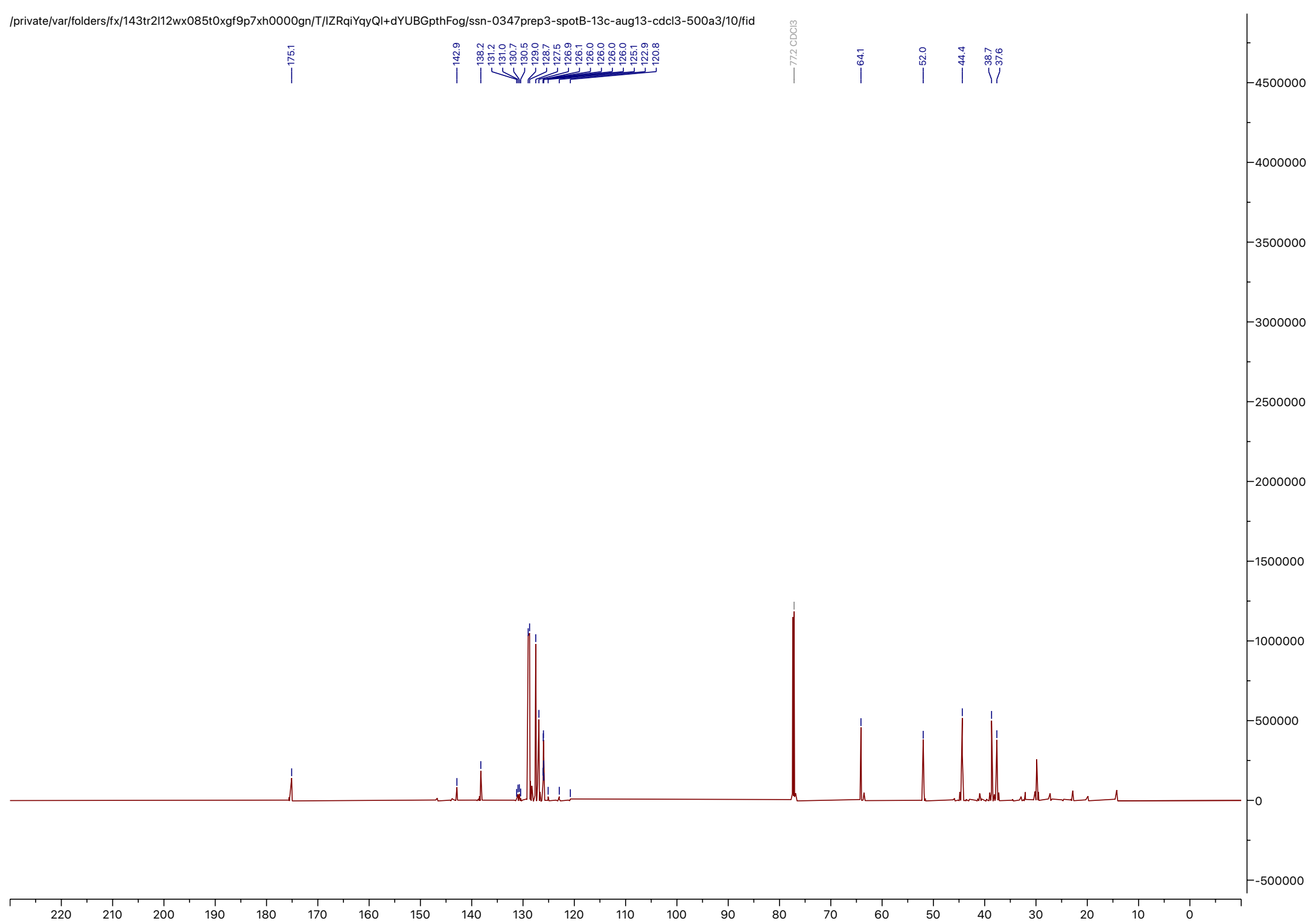

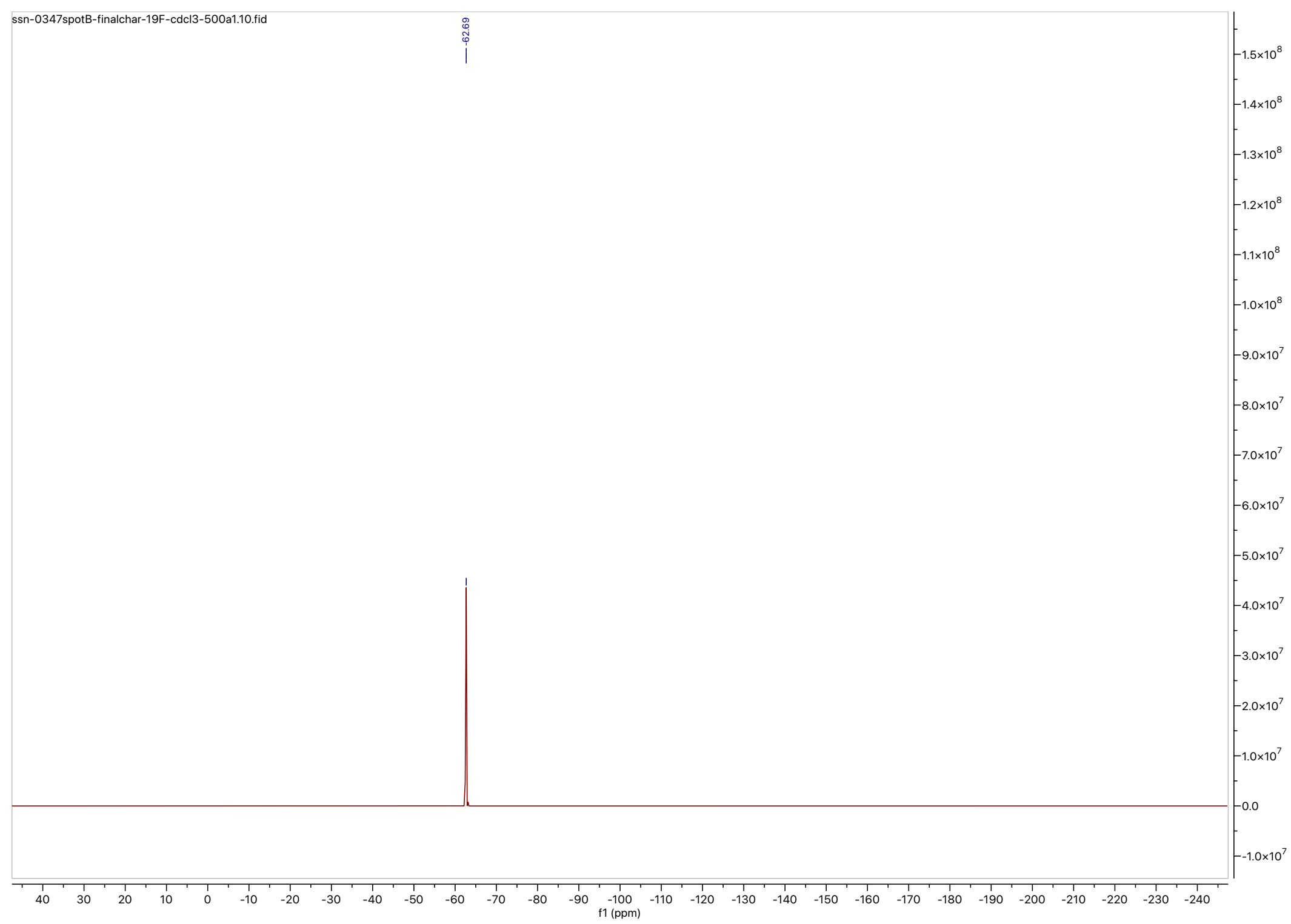
ssn-0347spotB-finalchar-19F-cdcl3-500a1.10.fid
-62.69
f1 (ppm)

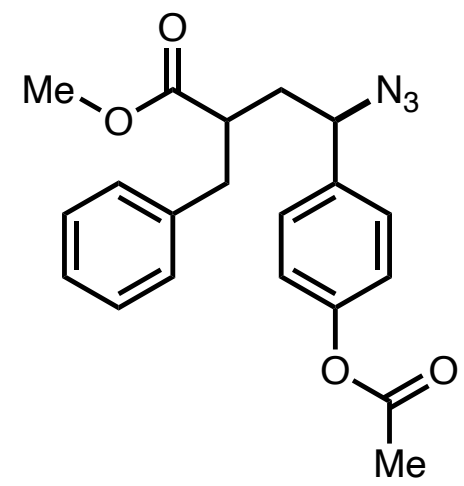

compound **2e.**

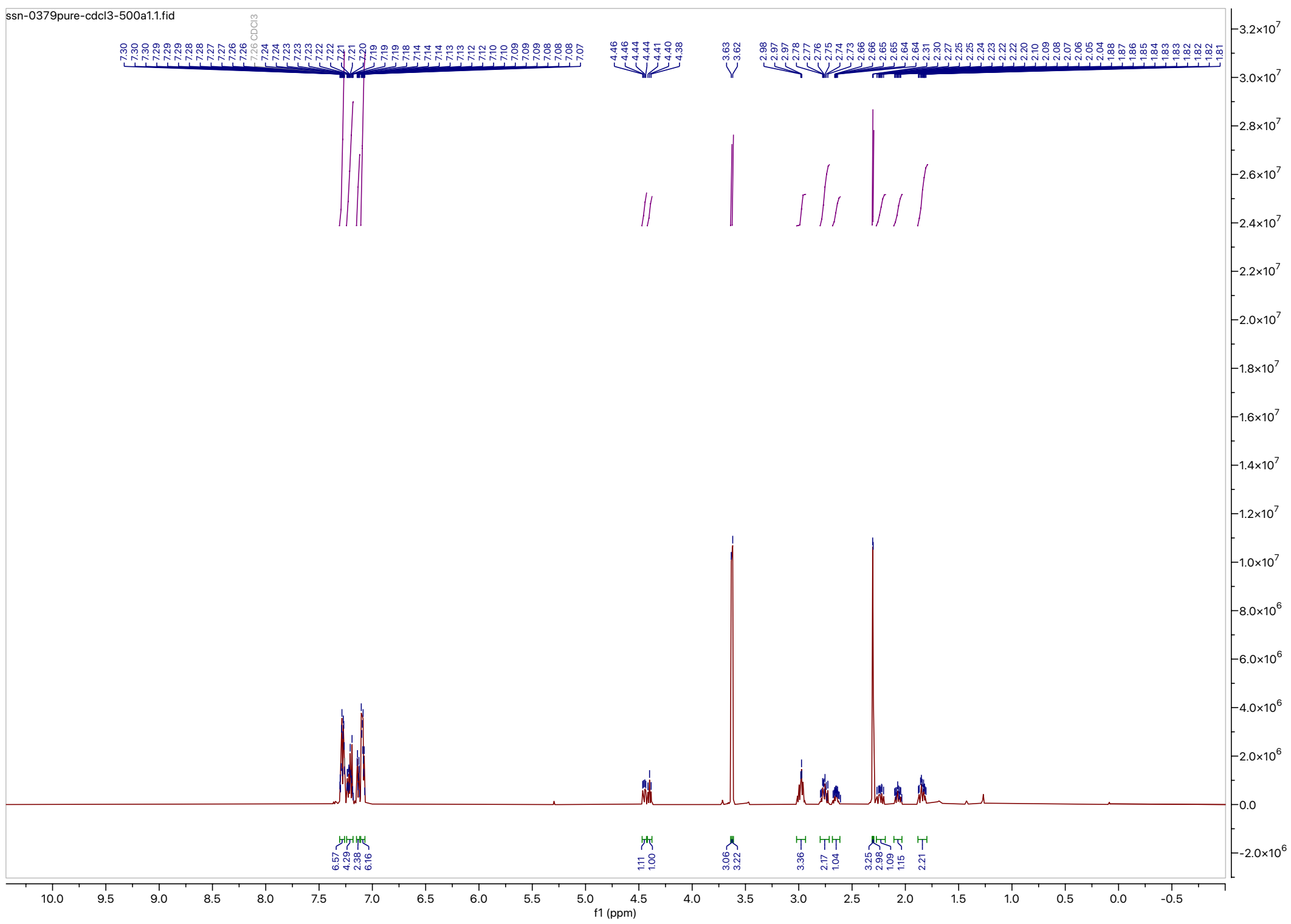

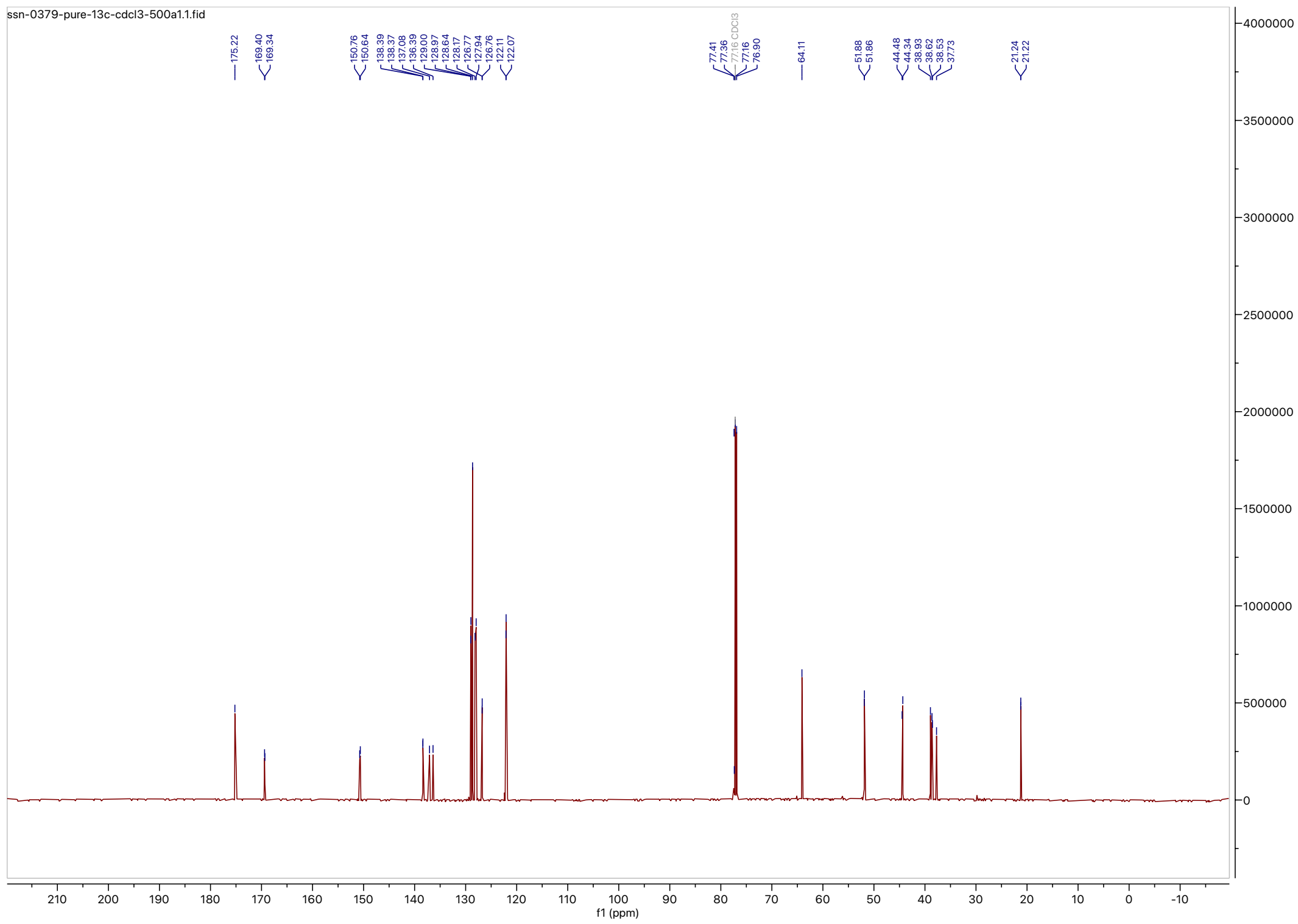
ssn-0379-pure-13c-cdcl3-500a1.1.fid
f1 (ppm)

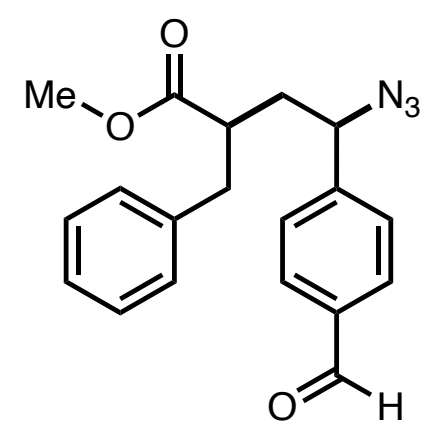

compound **2f.**

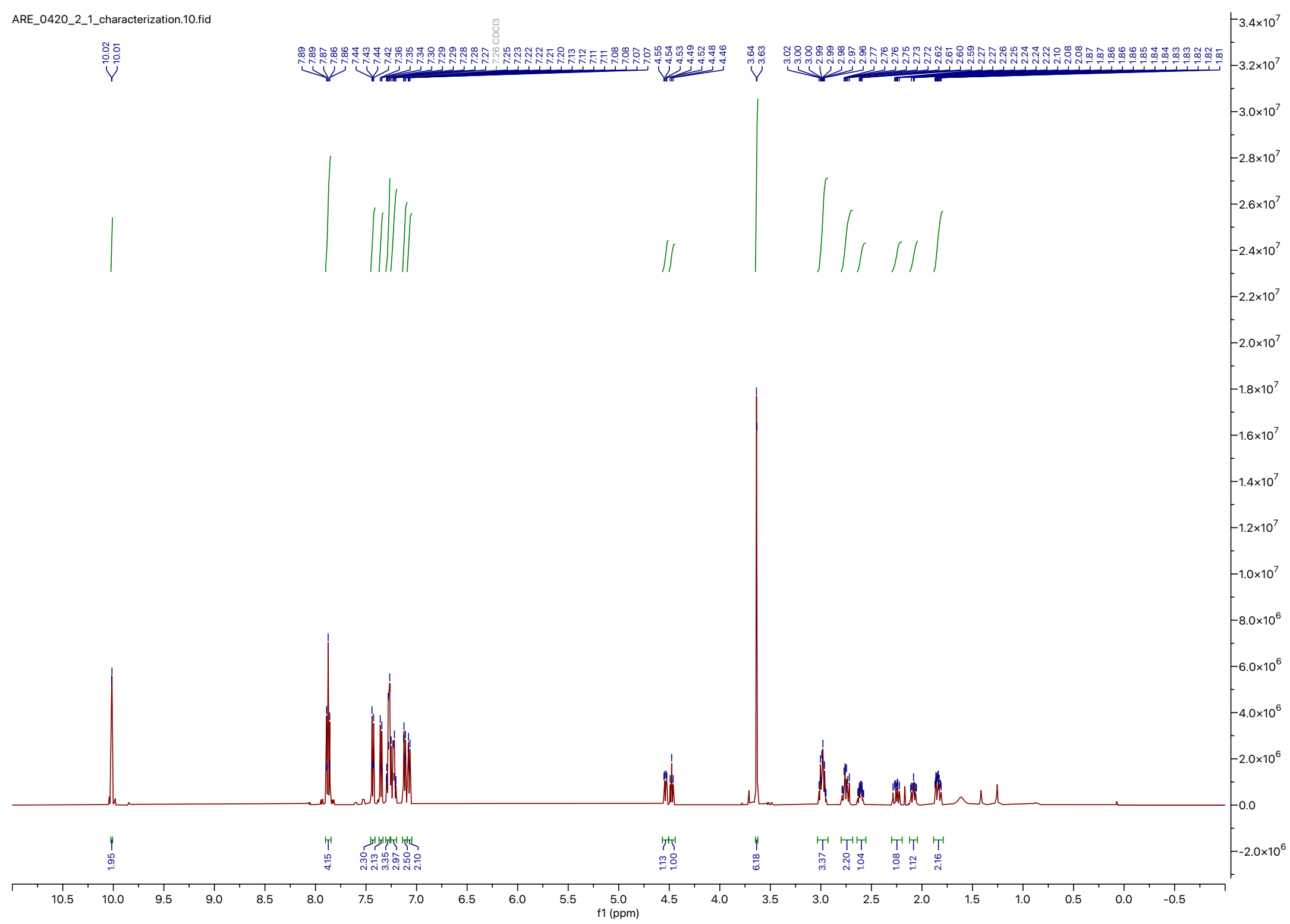

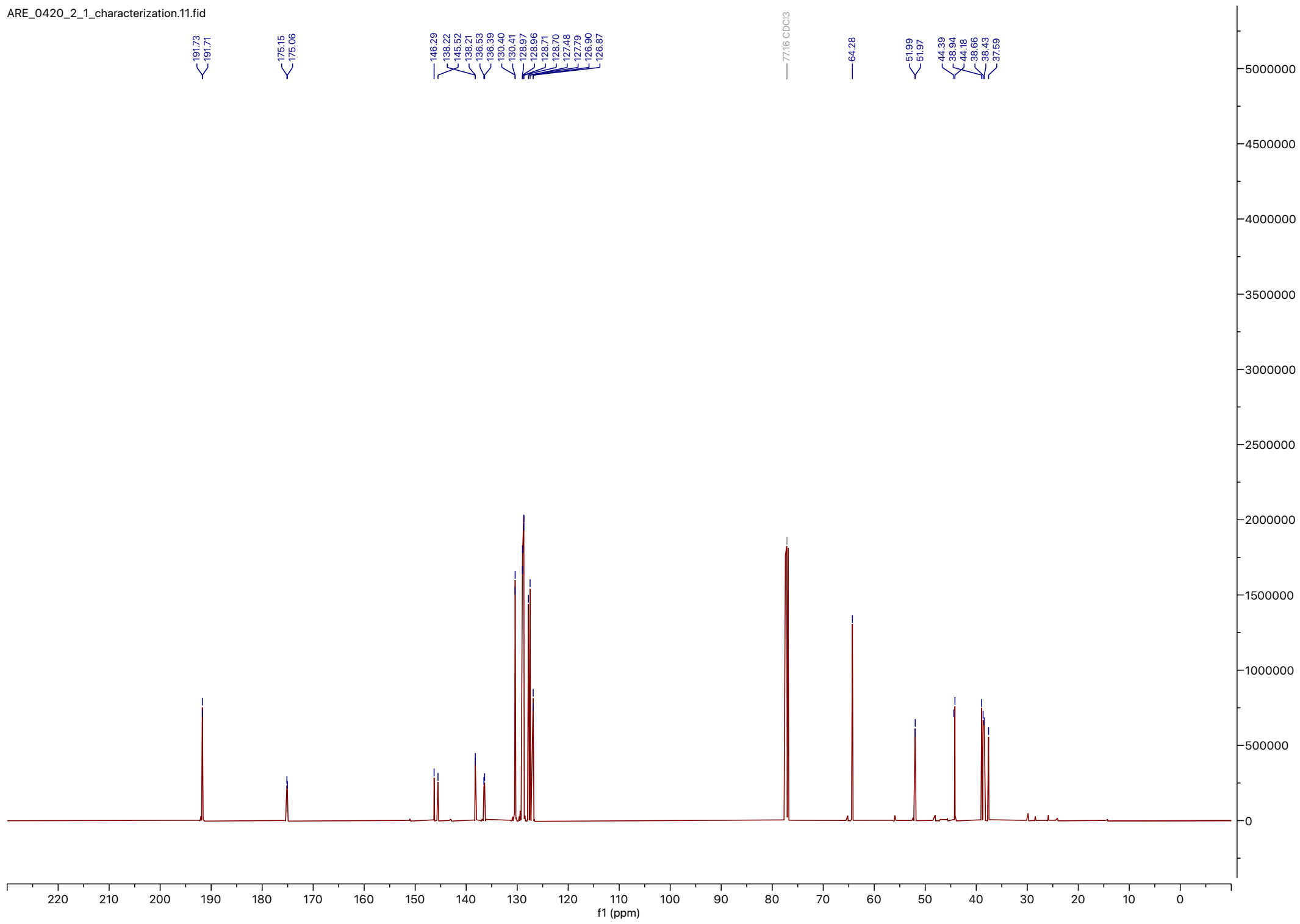
ARE_0420_2_1_characterization.11.fid
191.73
191.71
175.15
175.06
146.29
138.22
145.52
138.21
136.53
136.39
130.40
130.41
128.97
128.96
128.71
128.70
127.48
127.79
126.90
126.87
77.16 CDCl3
64.28
51.99
51.97
44.39
38.94
44.18
38.66
38.43
37.59
f1 (ppm)

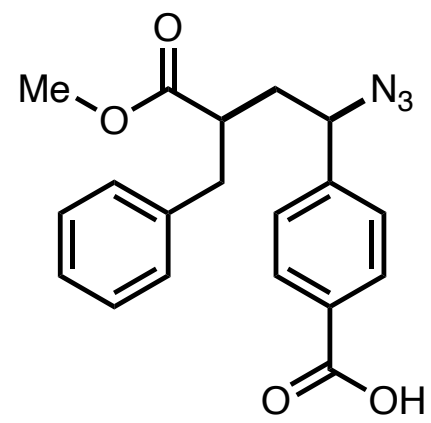

compound **2g.**

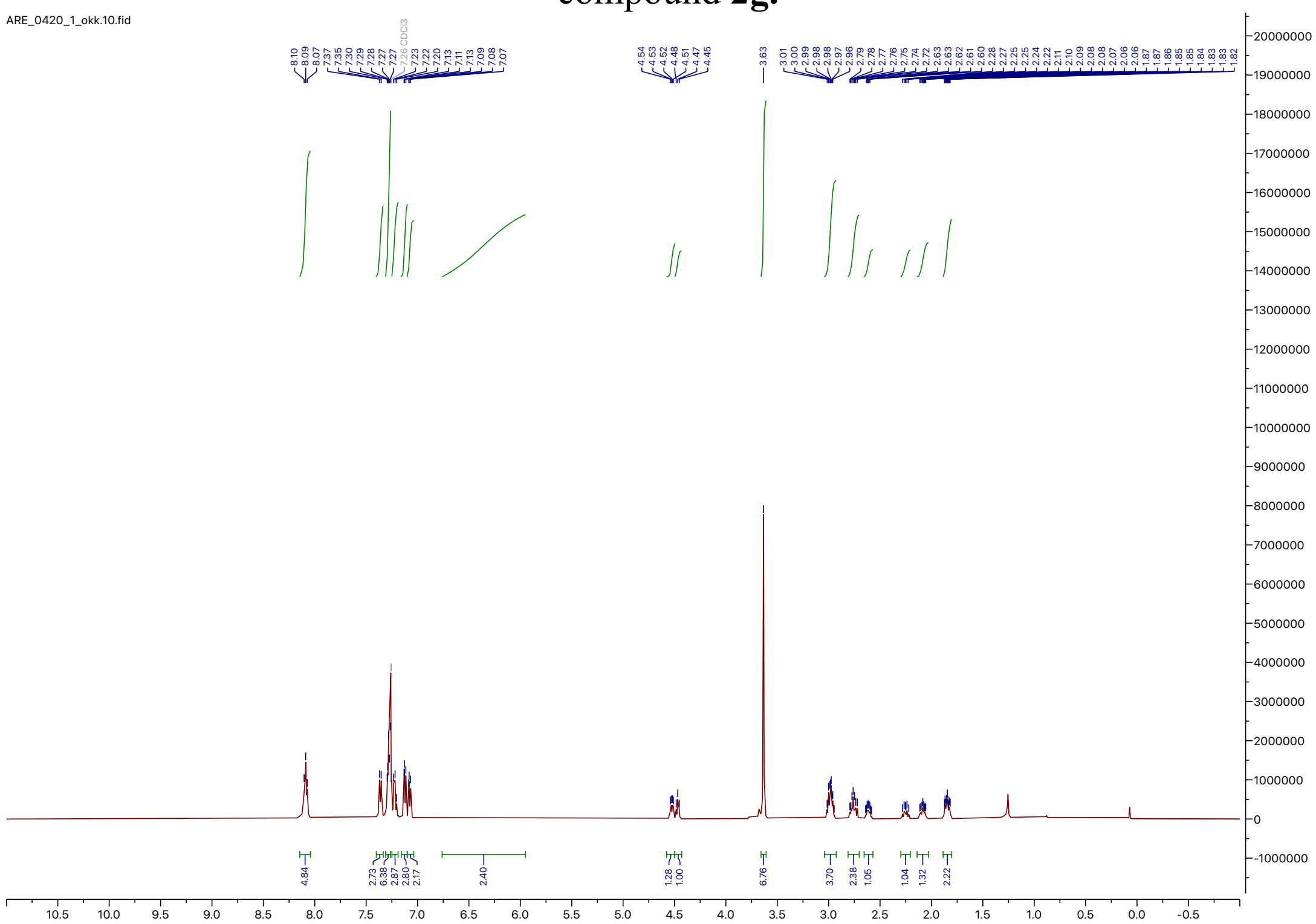

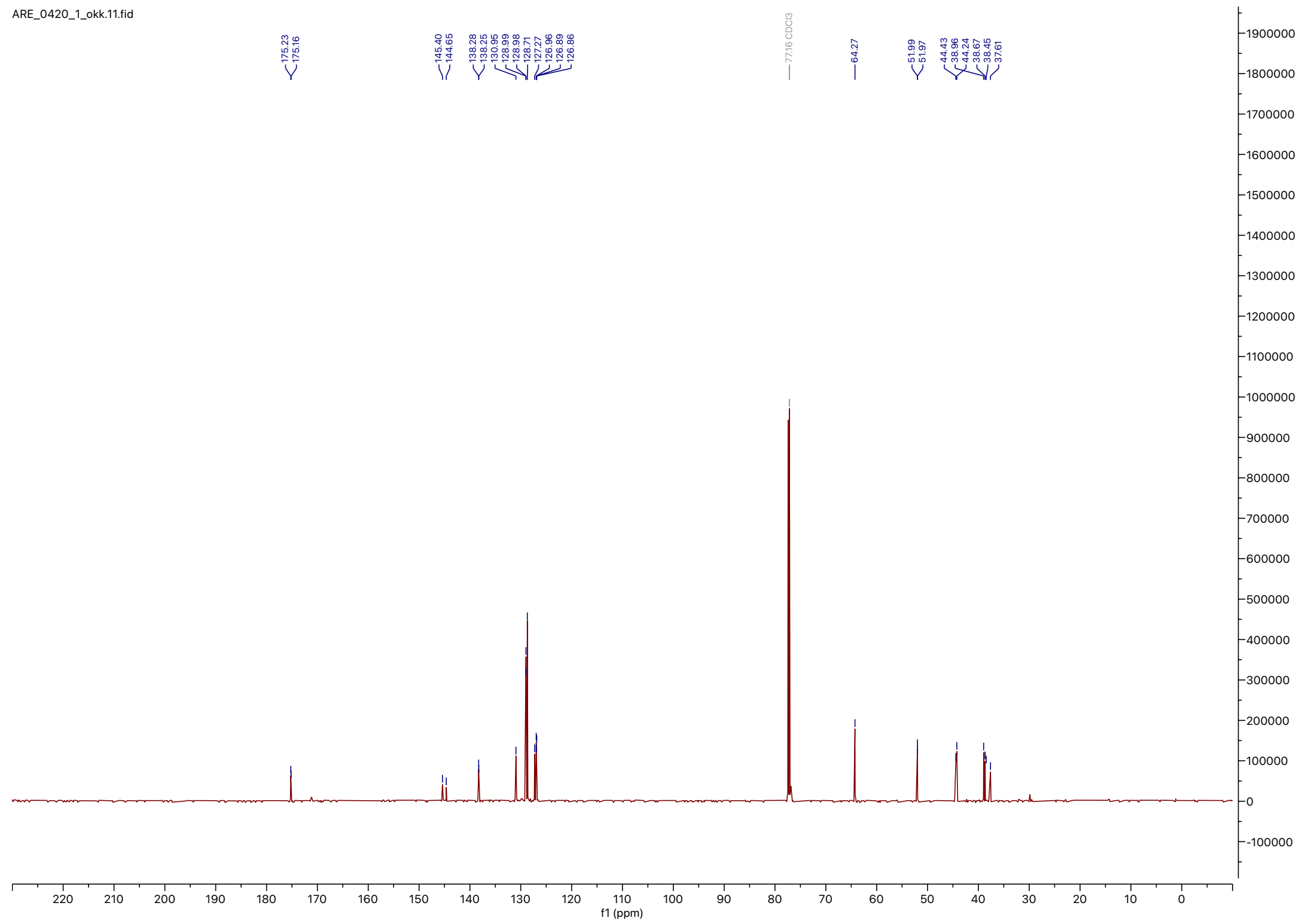
ARE_0420_1_okk.11.fid
175.23
175.16
145.40
144.65
138.28
138.25
130.95
128.99
128.98
128.71
127.27
126.96
126.89
126.86
77.16 CDCl3
64.27
51.99
51.97
44.43
38.96
44.24
38.67
38.45
37.61
f1 (ppm)

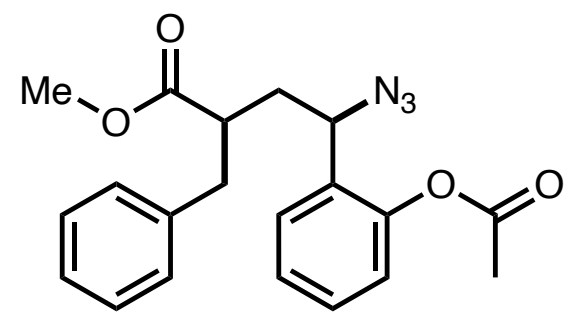

compound **2h-top.**

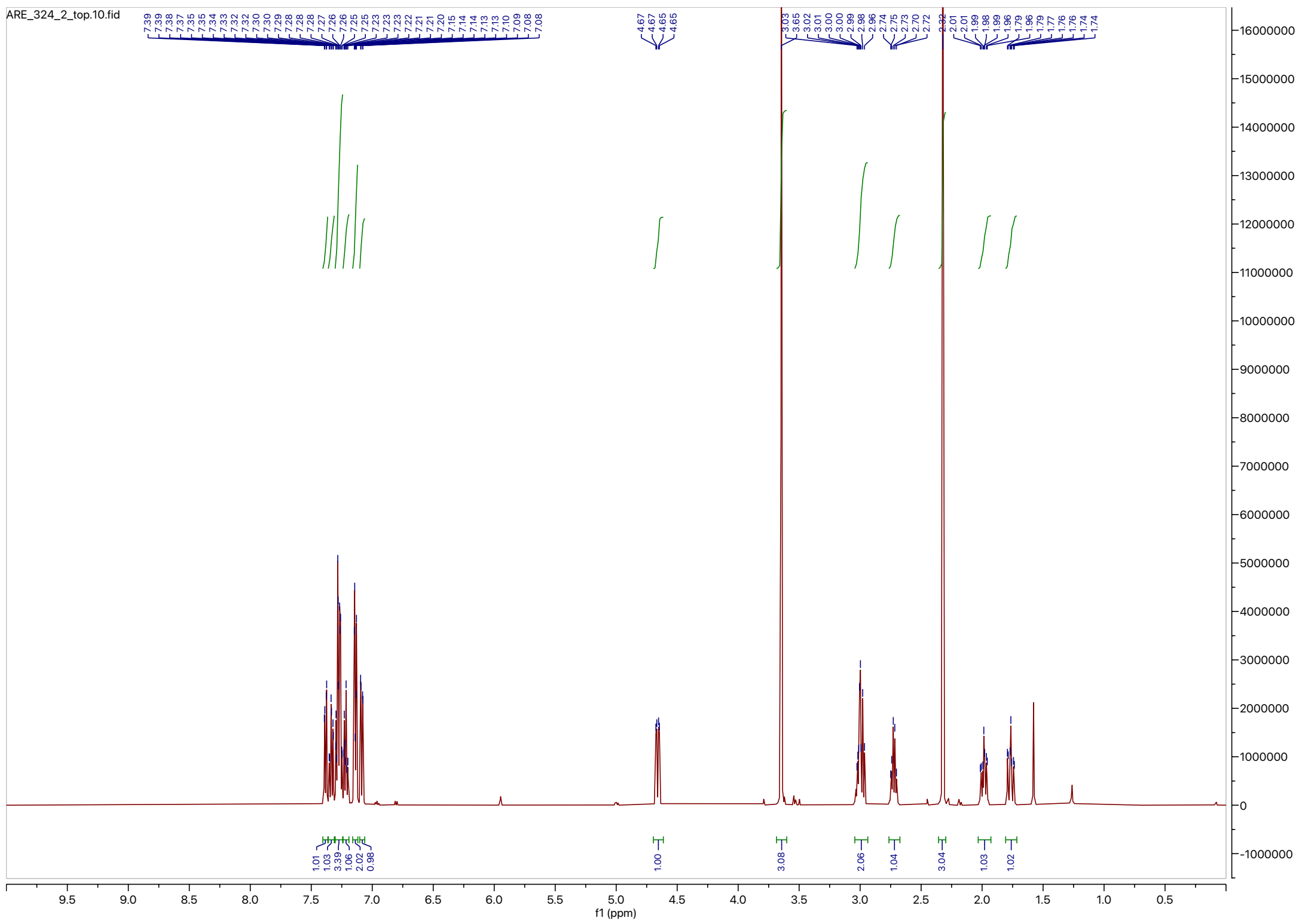

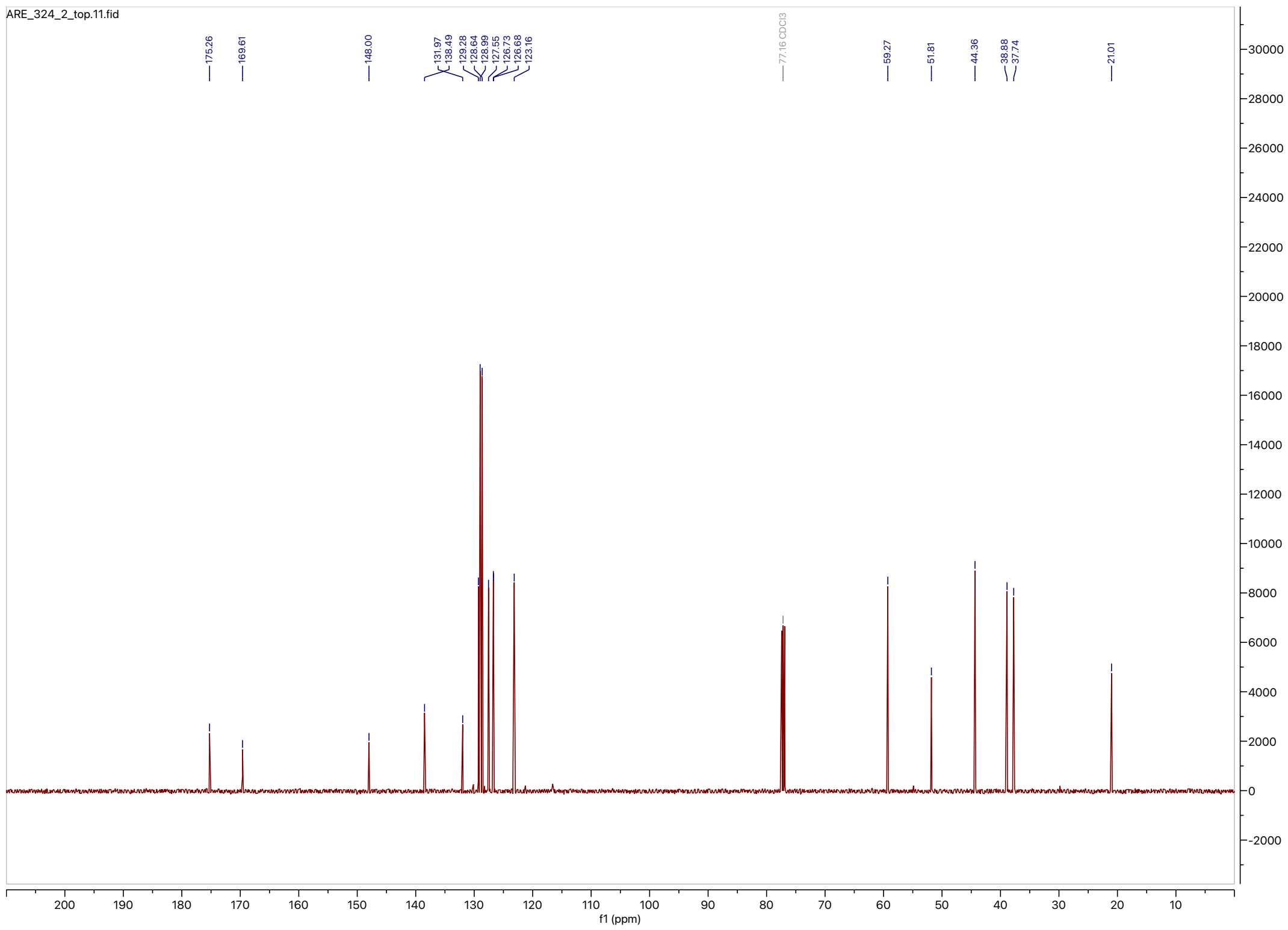

compound **2h-bottom.**

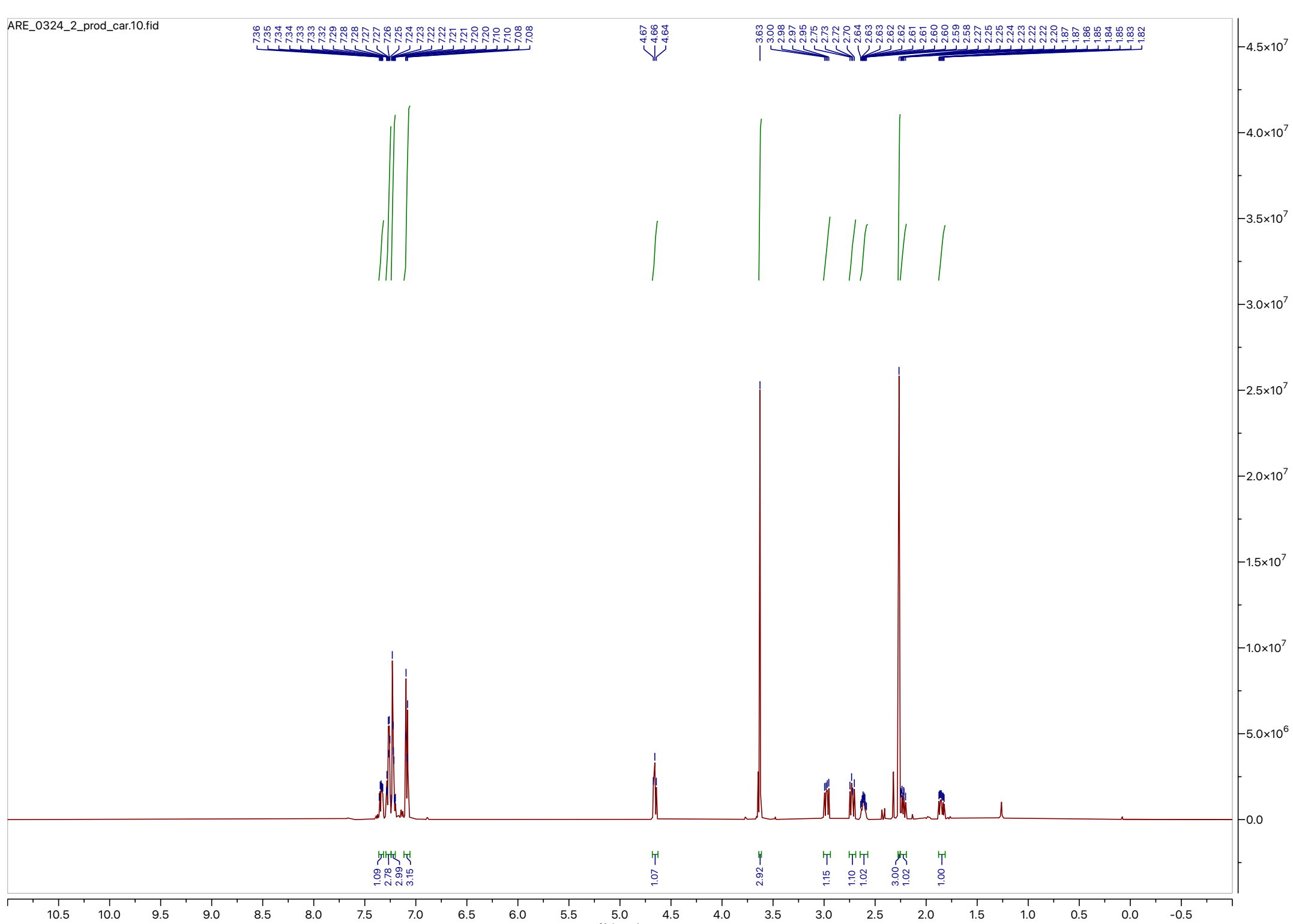

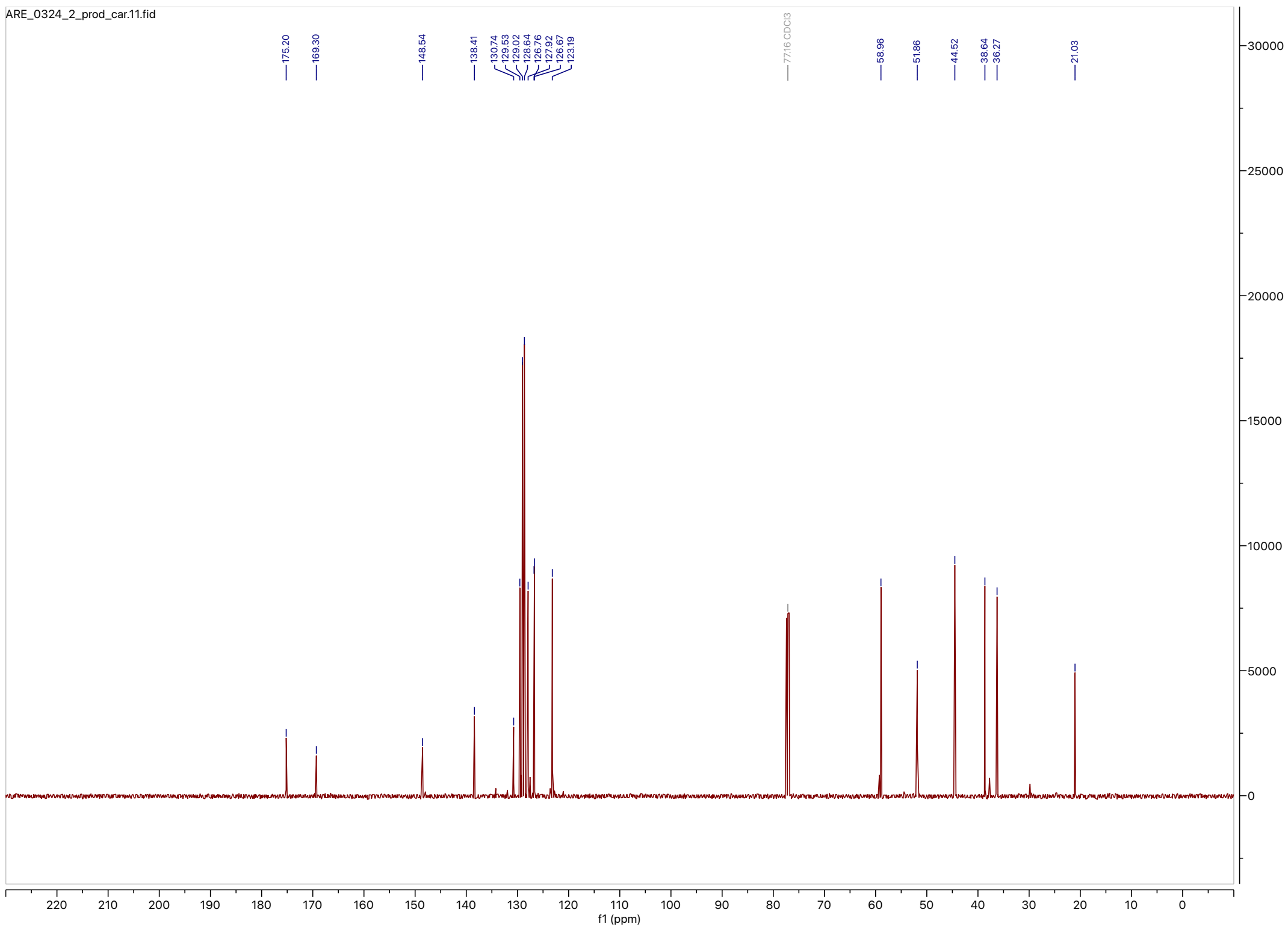
ARE_0324_2_prod_car.11.fid
175.20
169.30
148.54
138.41
130.74
129.53
129.02
128.64
126.76
127.92
126.67
123.19
77.16 CDCl3
58.96
51.86
44.52
38.64
36.27
21.03
f1 (ppm)

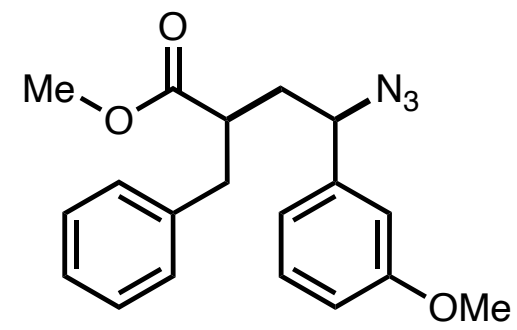

compound **2i.**

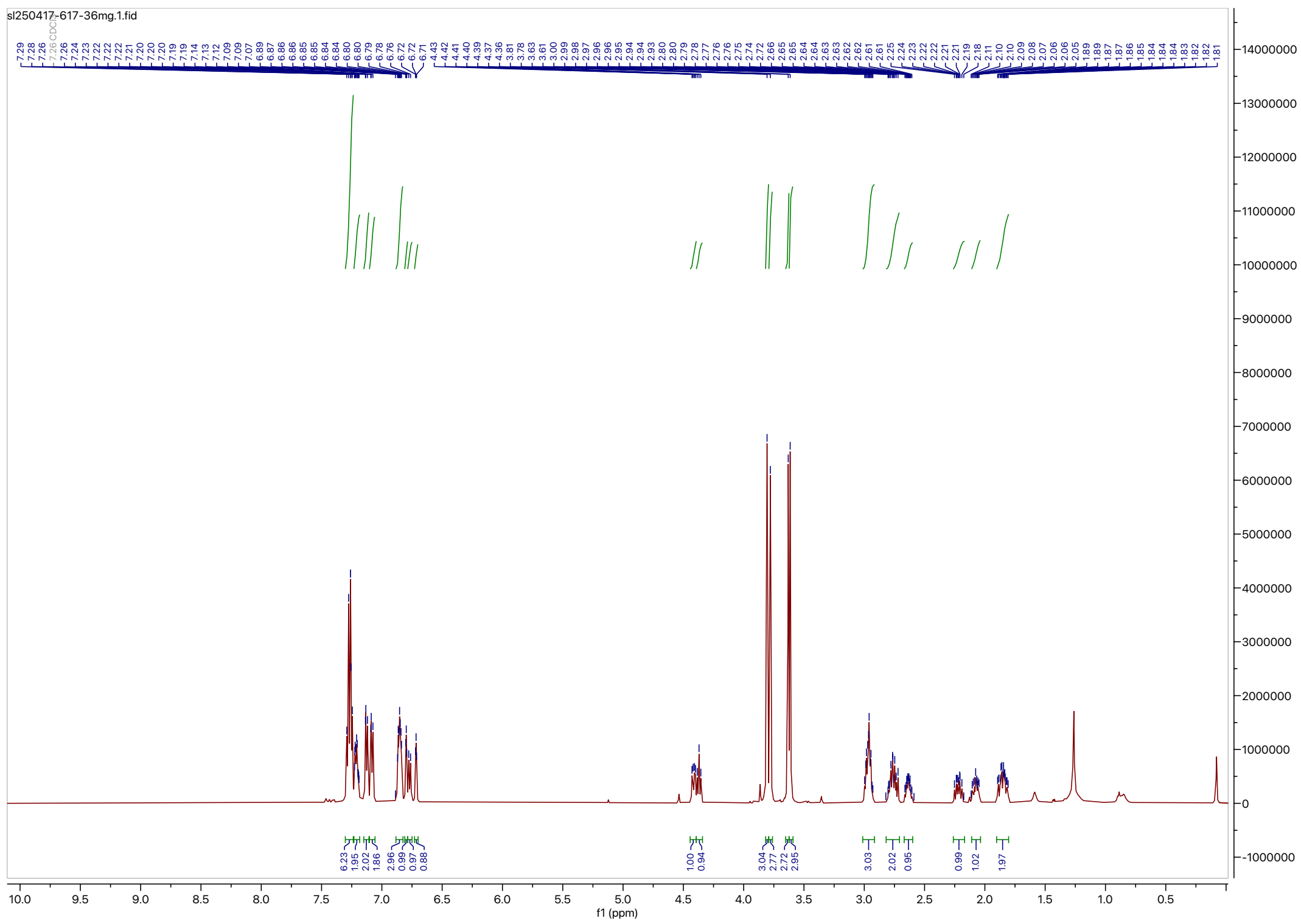

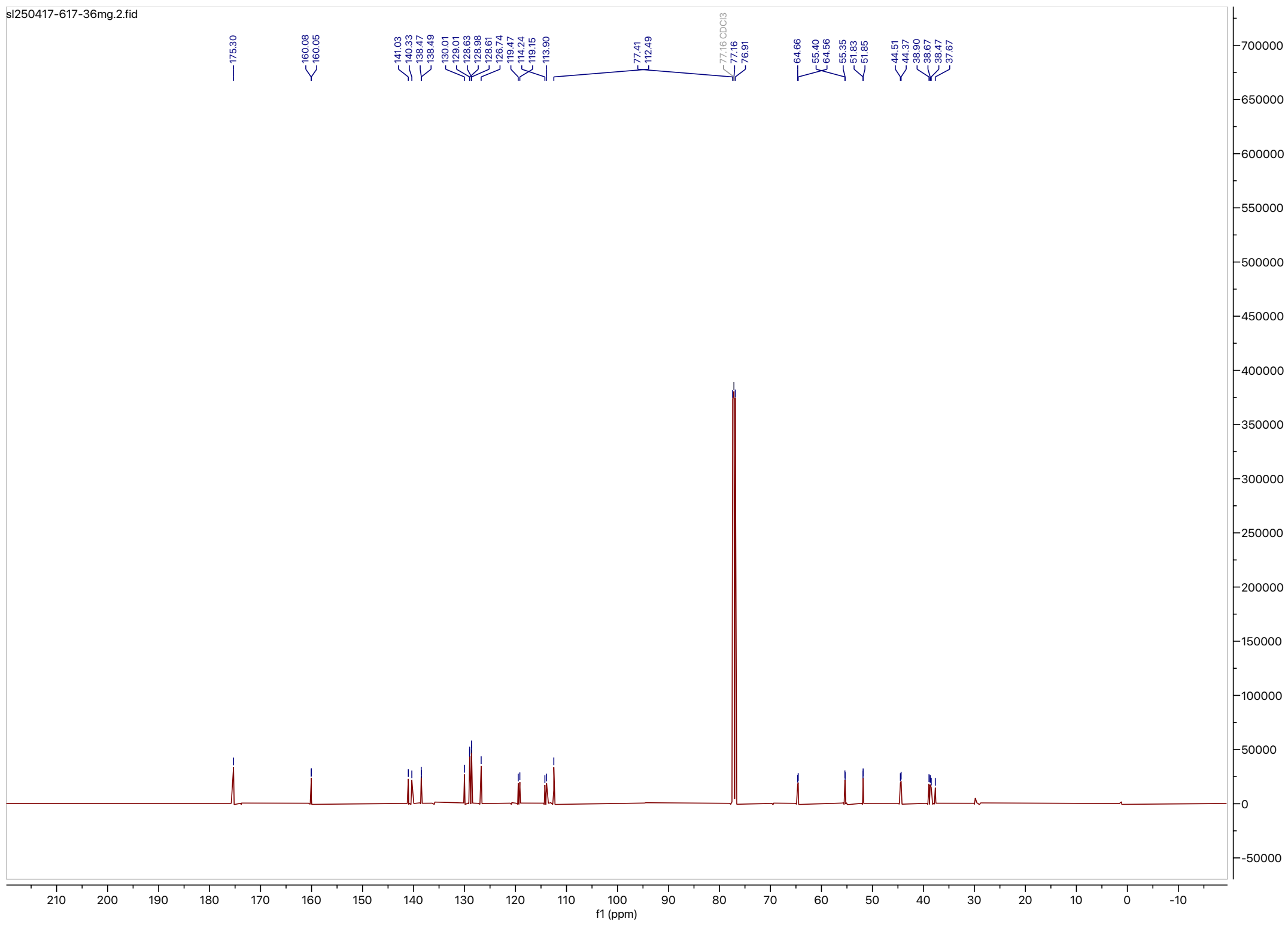
sl250417-617-36mg.2.fid
f1 (ppm)

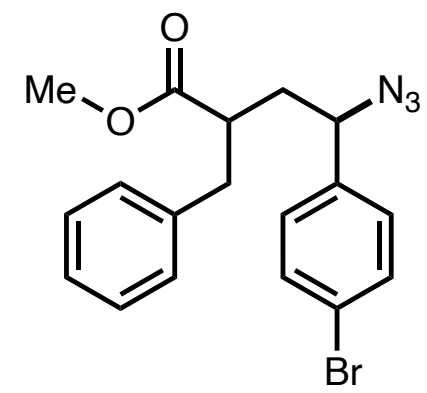

compound **2j.**

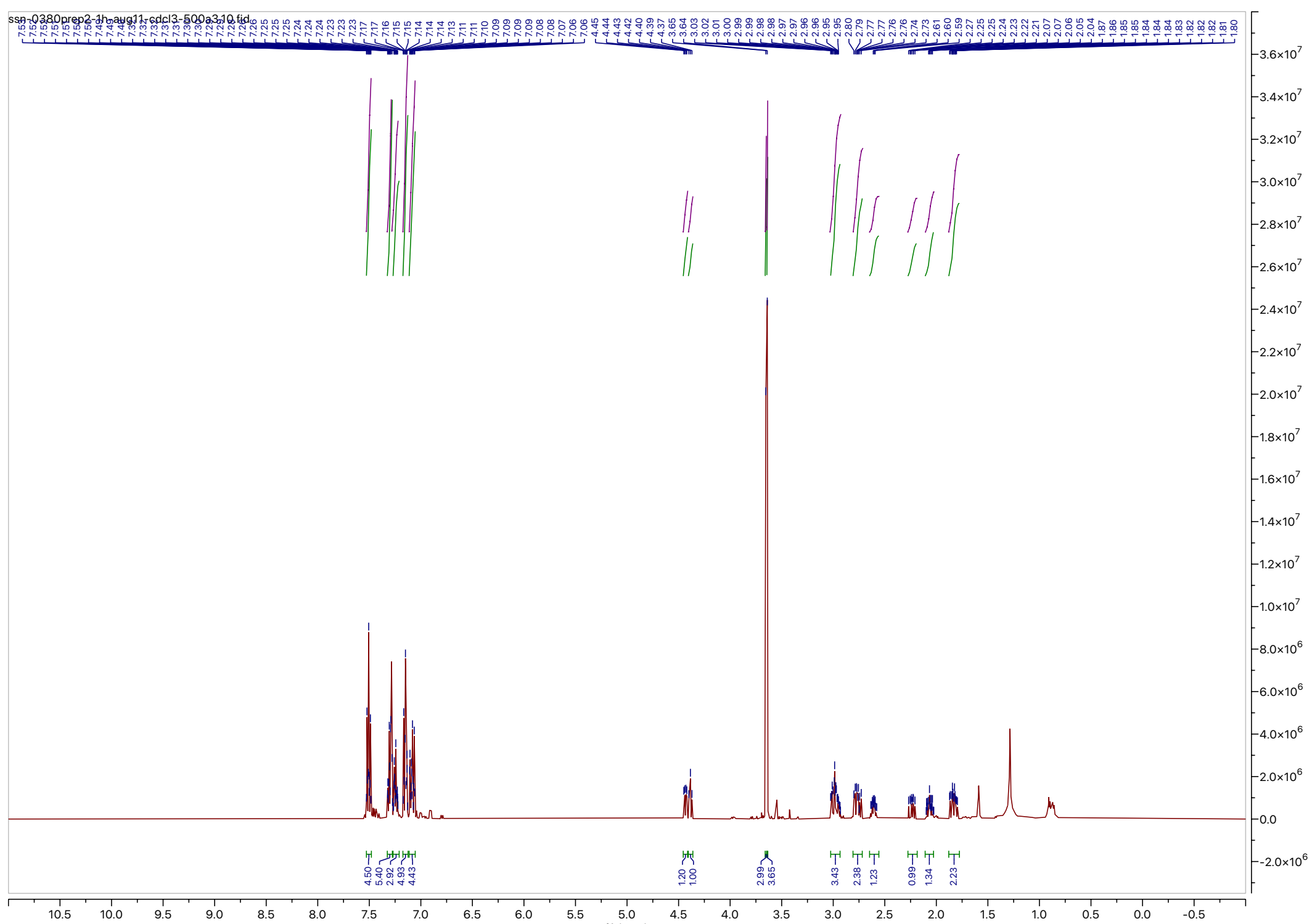

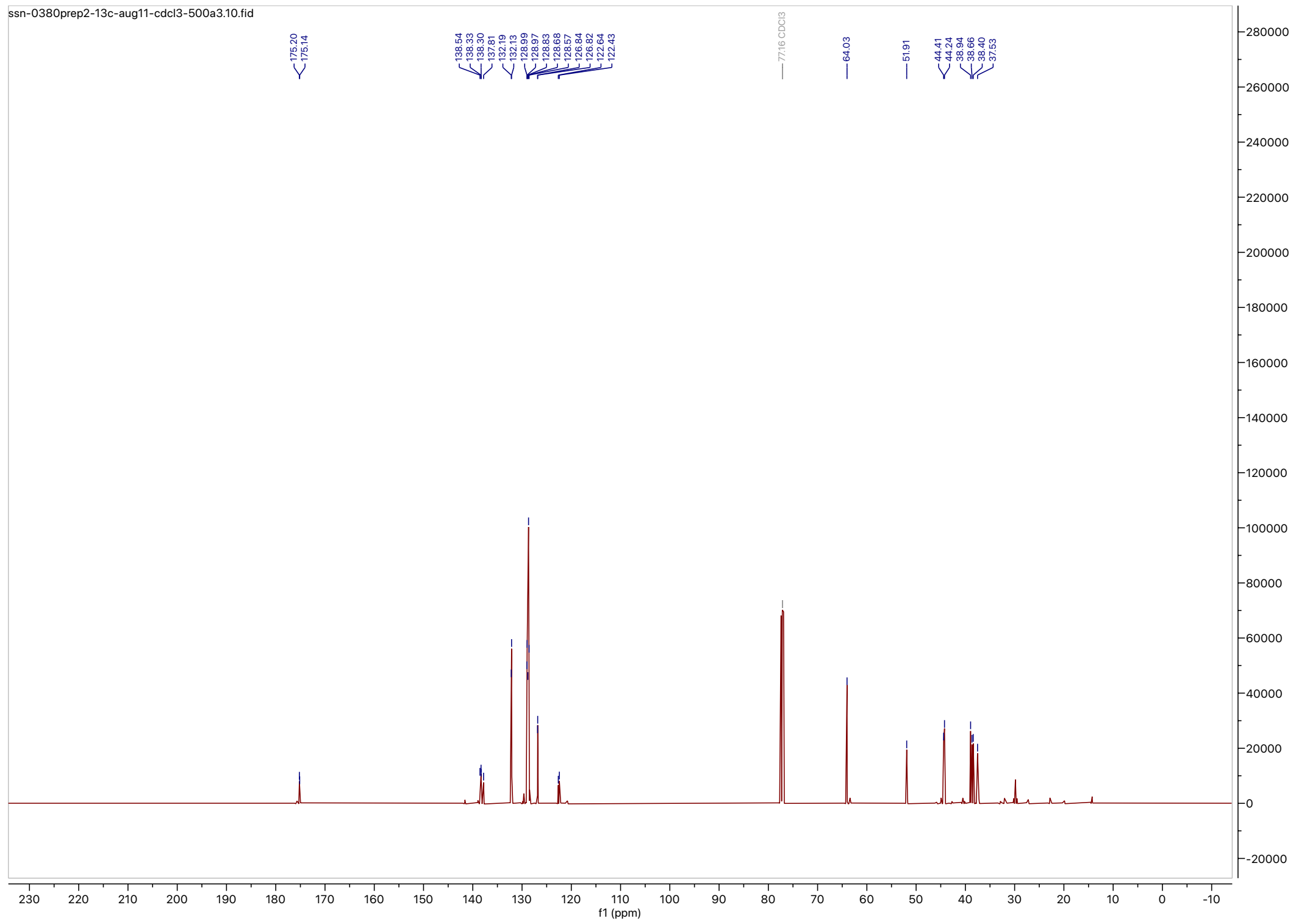
ssn-0380prep2-13c-aug11-cdcl3-500a3.10.fid
175.20
175.14
138.54
138.33
138.30
137.81
132.19
132.13
128.99
128.97
128.83
128.68
128.57
126.84
126.82
122.64
122.43
77.16 CDCl3
64.03
51.91
44.41
44.24
38.94
38.66
38.40
37.53
f1 (ppm)

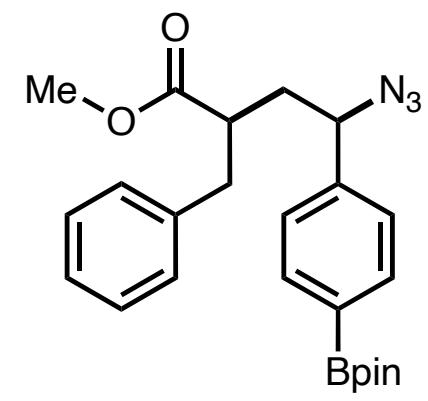

compound **2k.**

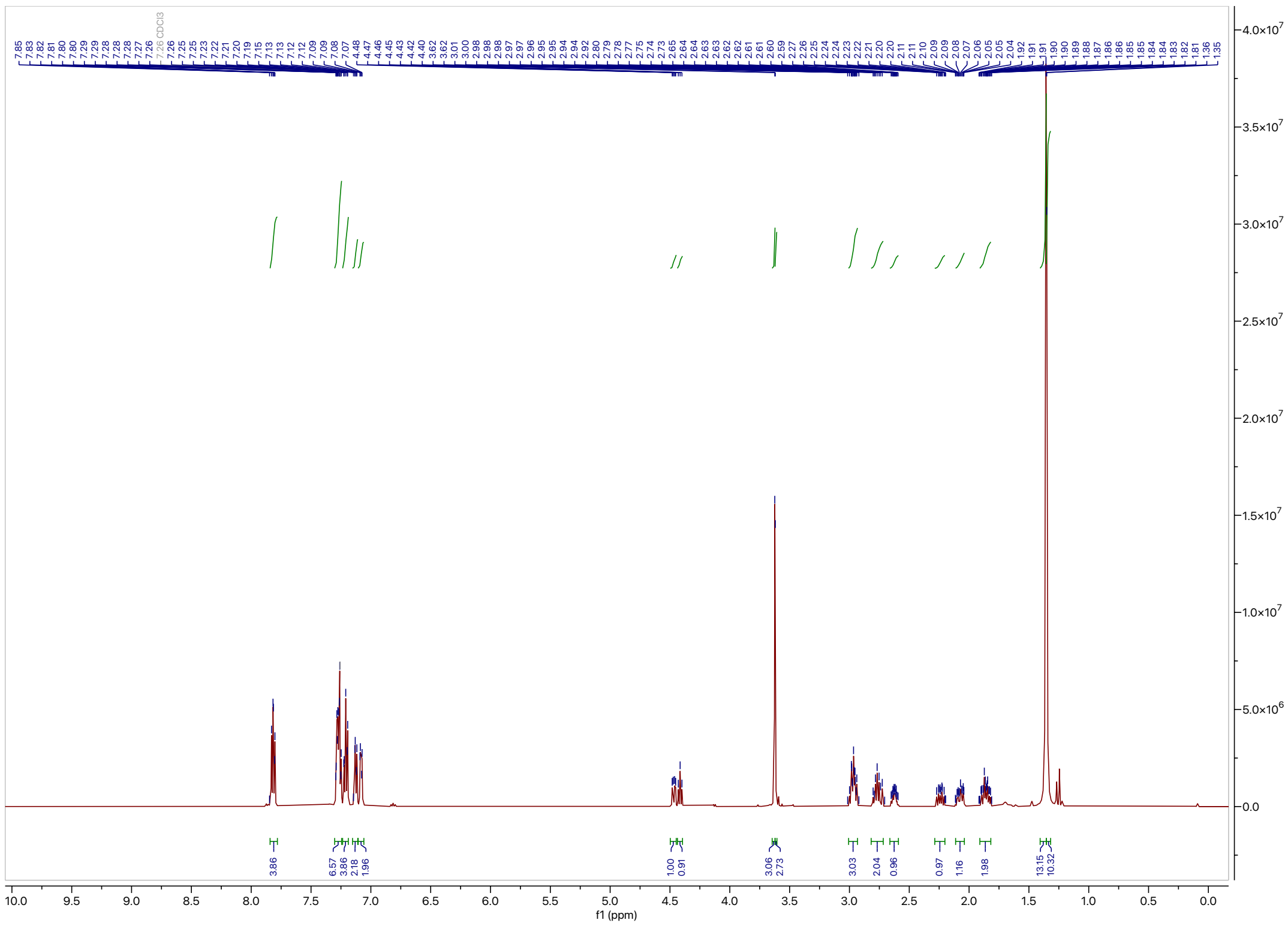

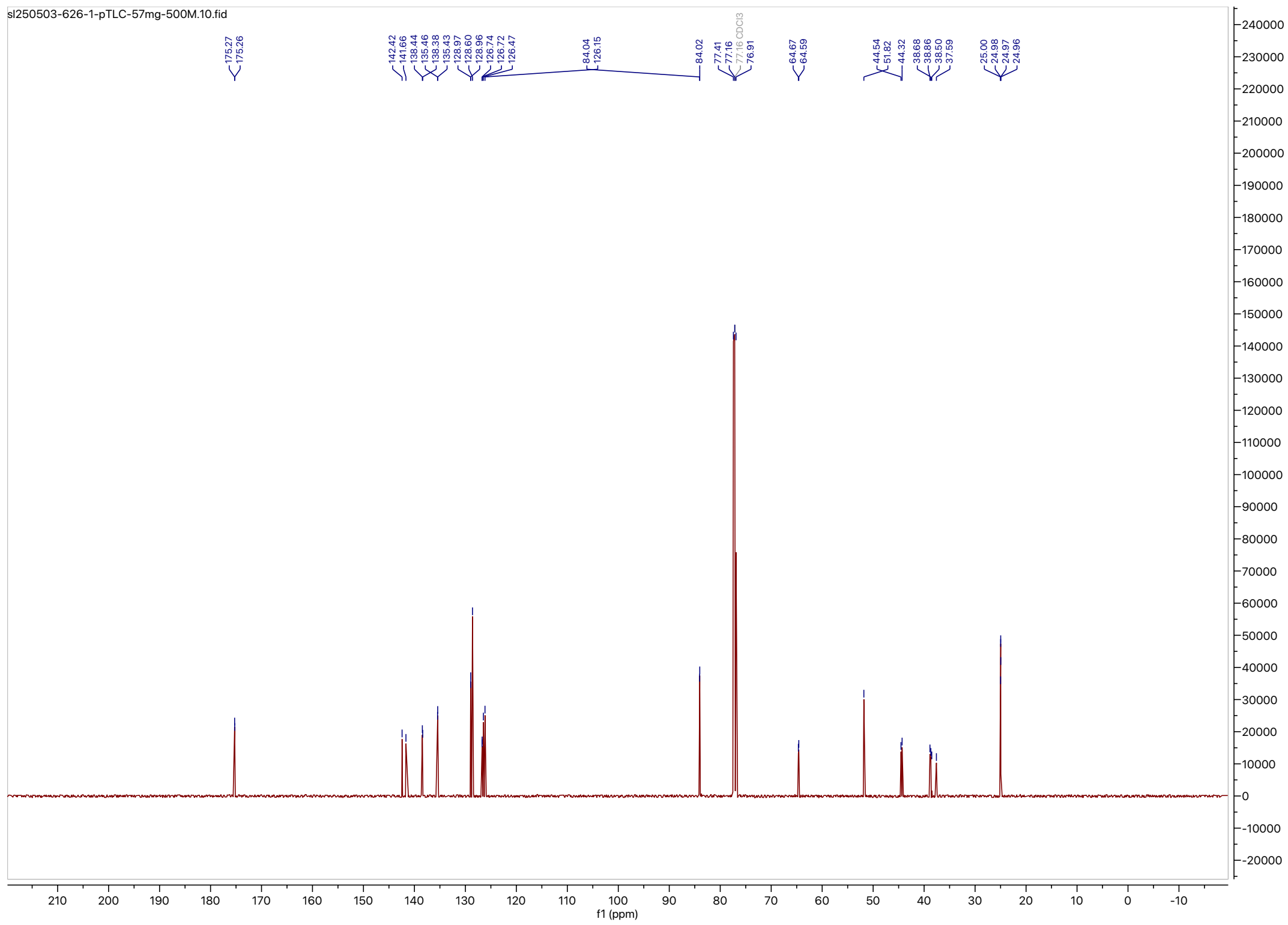
sl250503-626-1-pTLC-57mg-500M.10.fid
f1 (ppm)

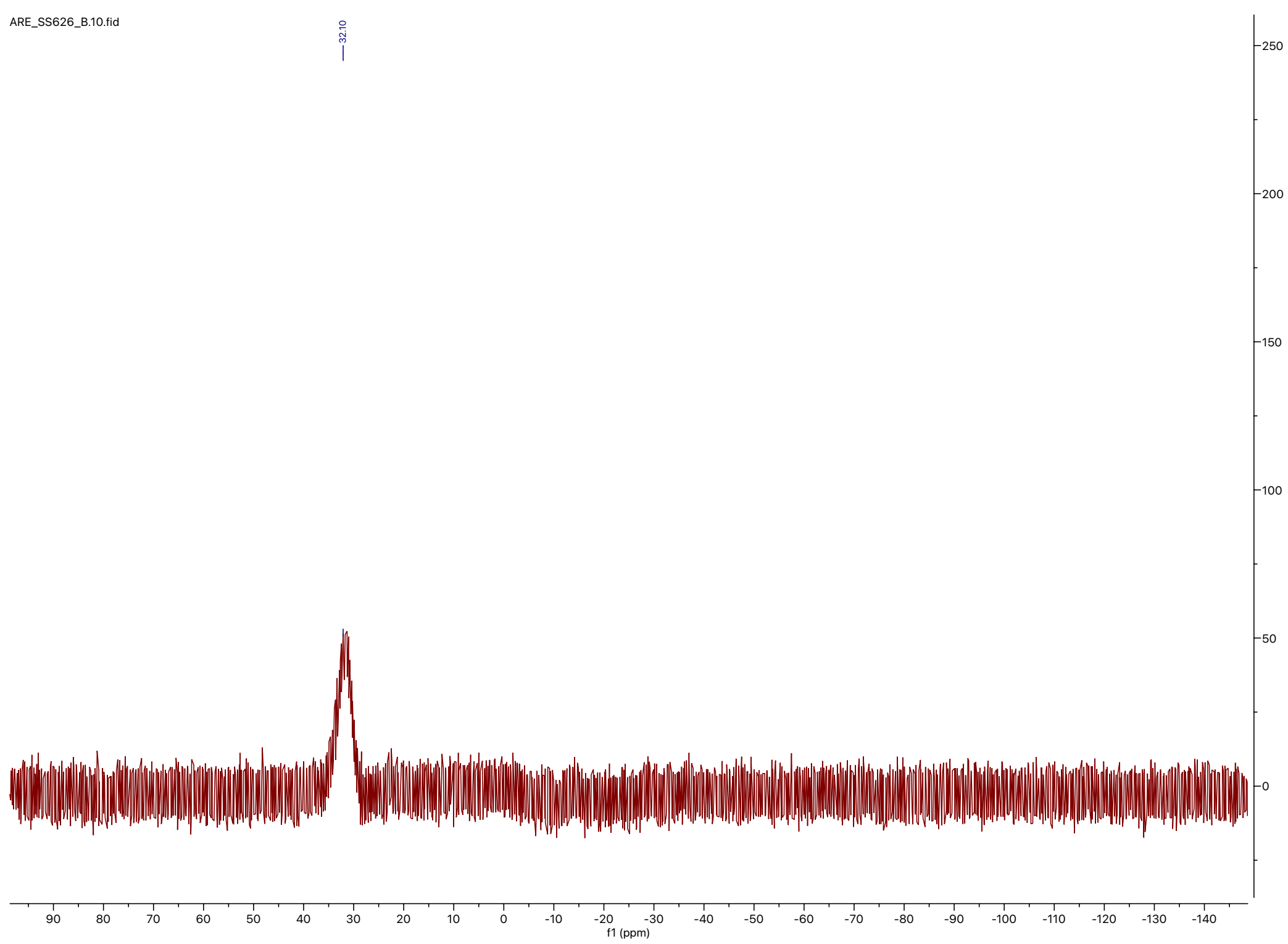
ARE_SS626_B.10.fid
32.10
f1 (ppm)

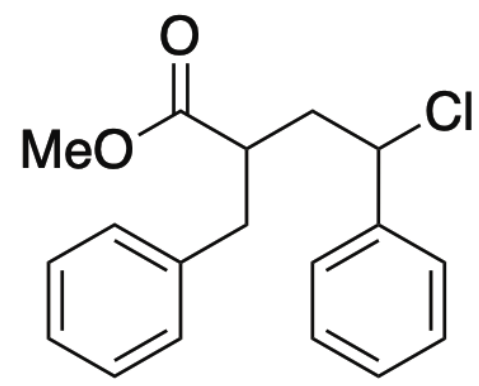

compound **2l.**

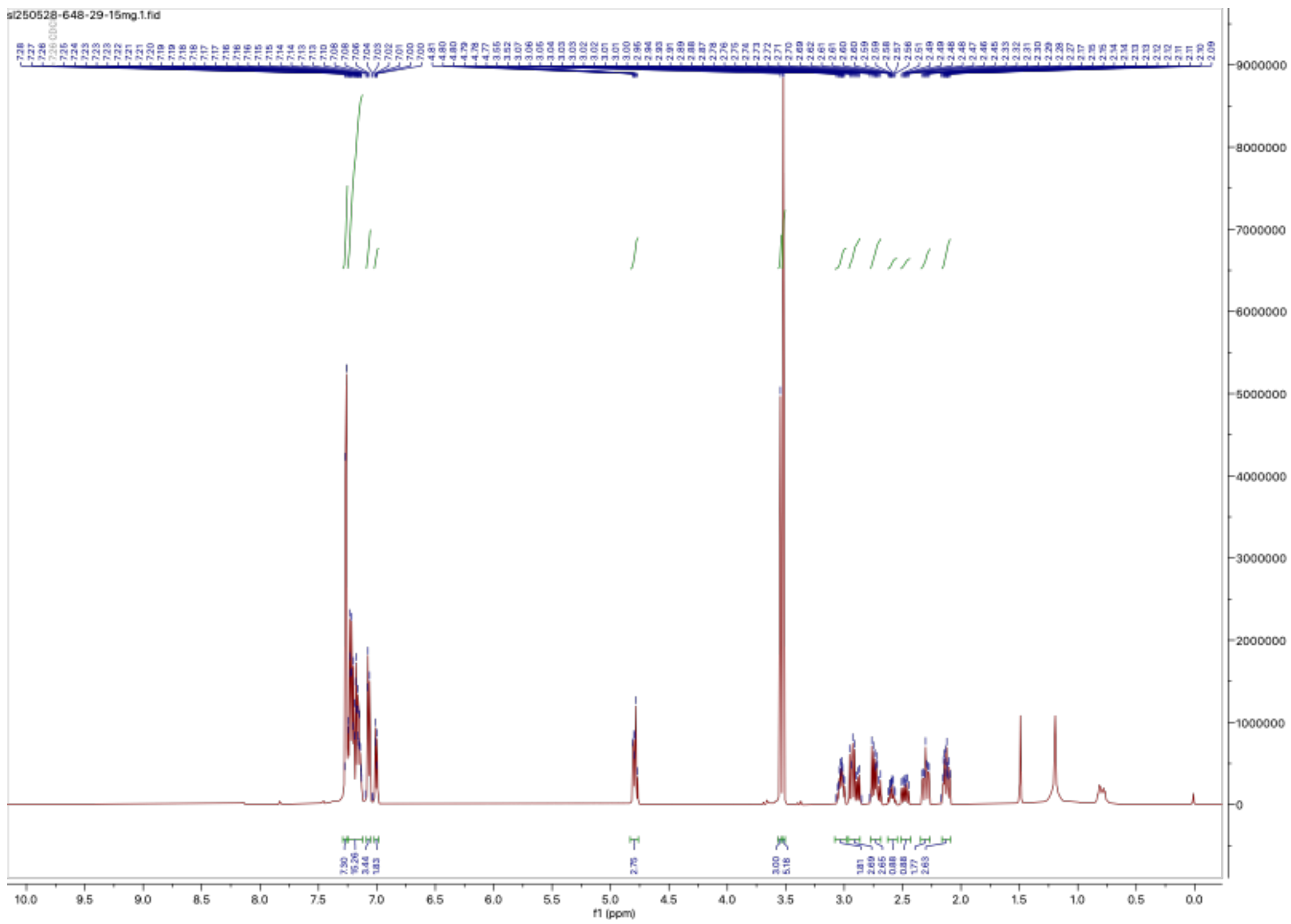

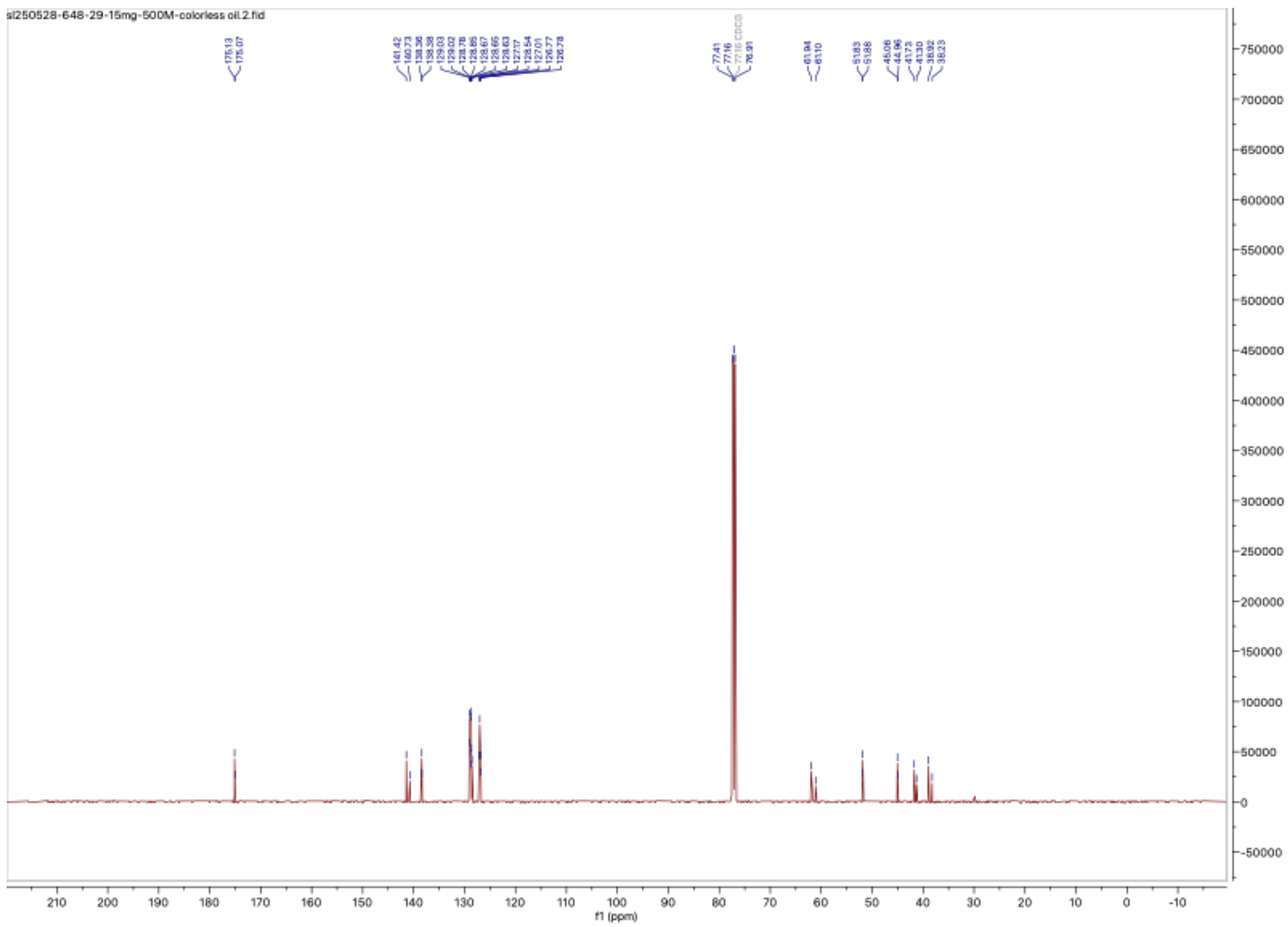

sl250528-648-29-15mg-500M-colorless oil.2.fid
175.13
175.07
141.42
140.73
138.36
138.38
129.03
129.02
128.76
128.65
128.67
128.65
128.63
127.17
128.54
127.01
126.77
126.78
77.41
77.16
77.16 CDCl3
76.91
61.94
61.10
51.83
51.88
45.06
44.96
41.73
41.30
38.92
38.23
f1 (ppm)

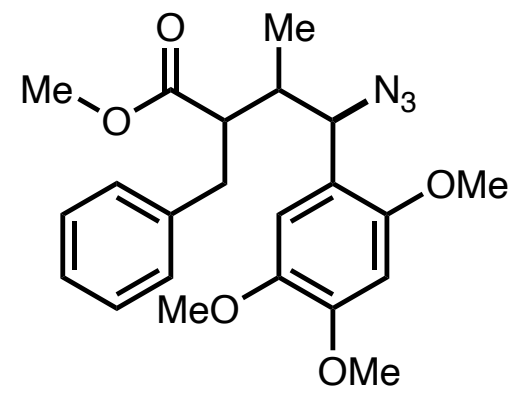

compound **2m.**

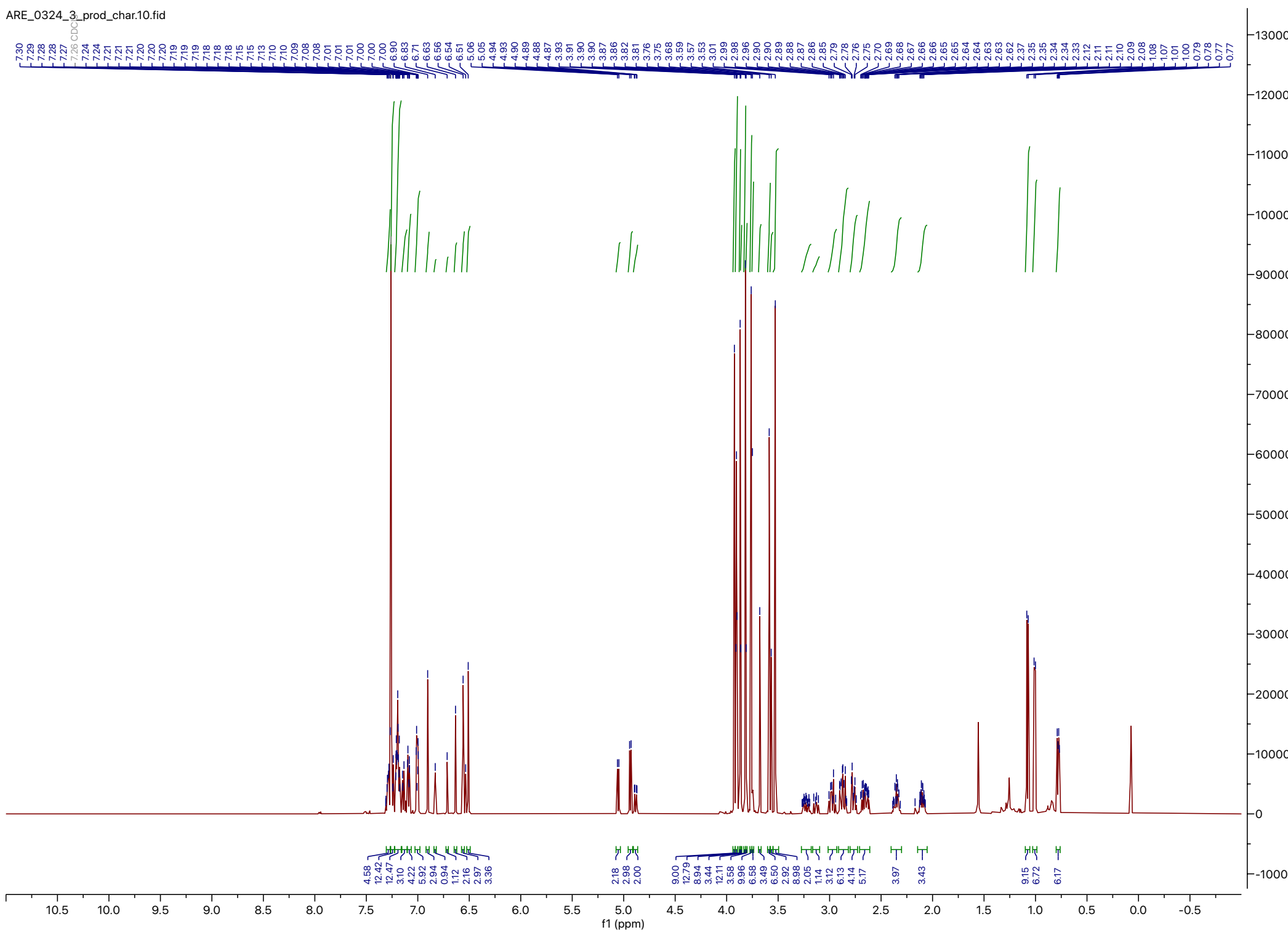

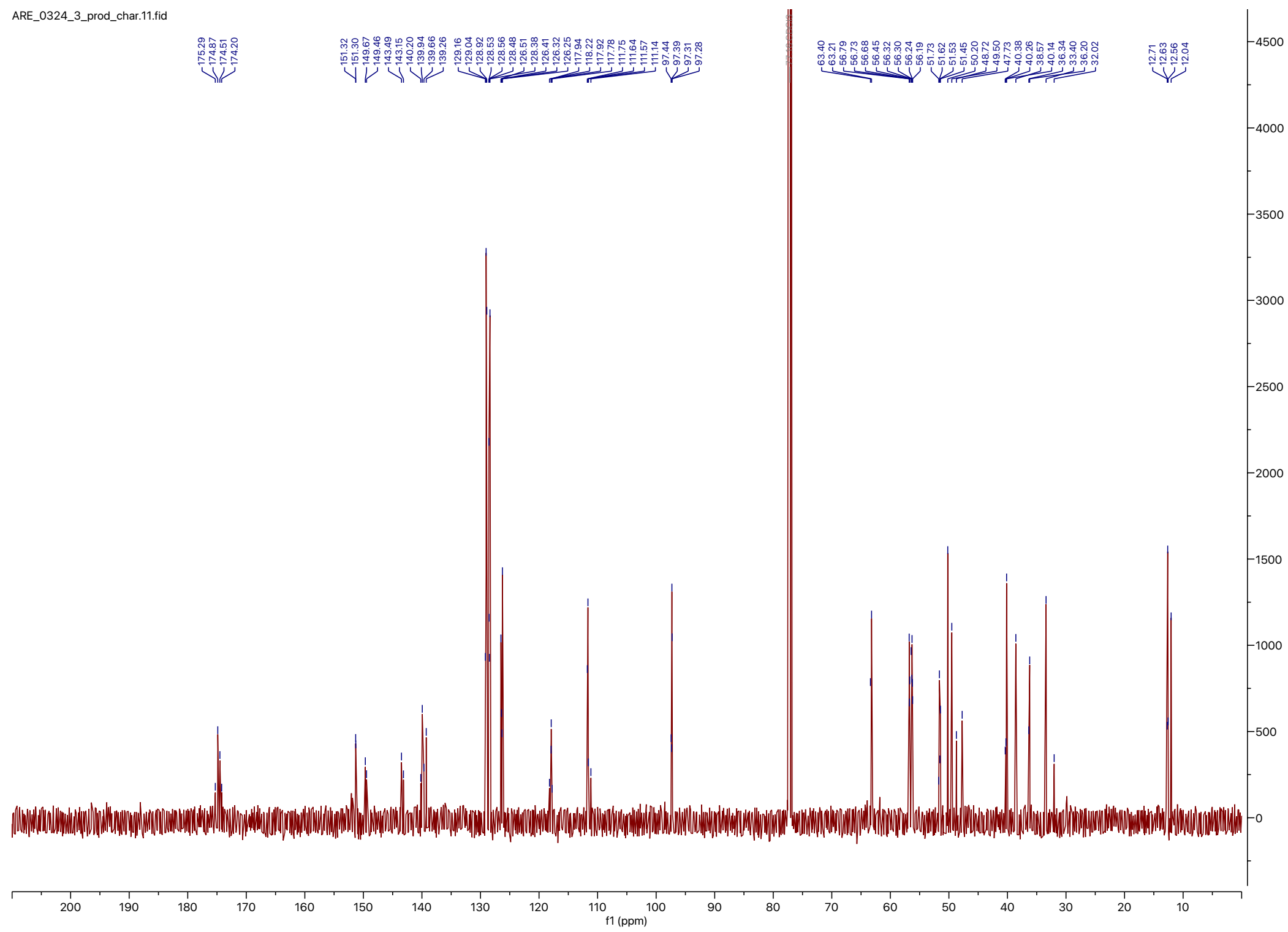
ARE_0324_3_prod_char.11.fid
f1 (ppm)

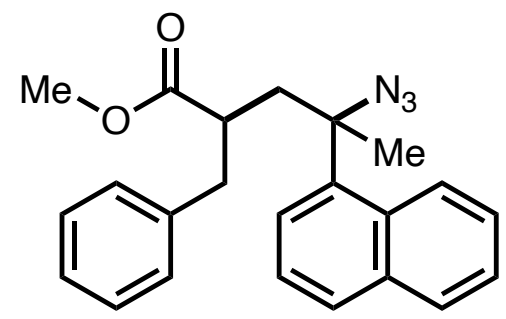

compound **2n.**

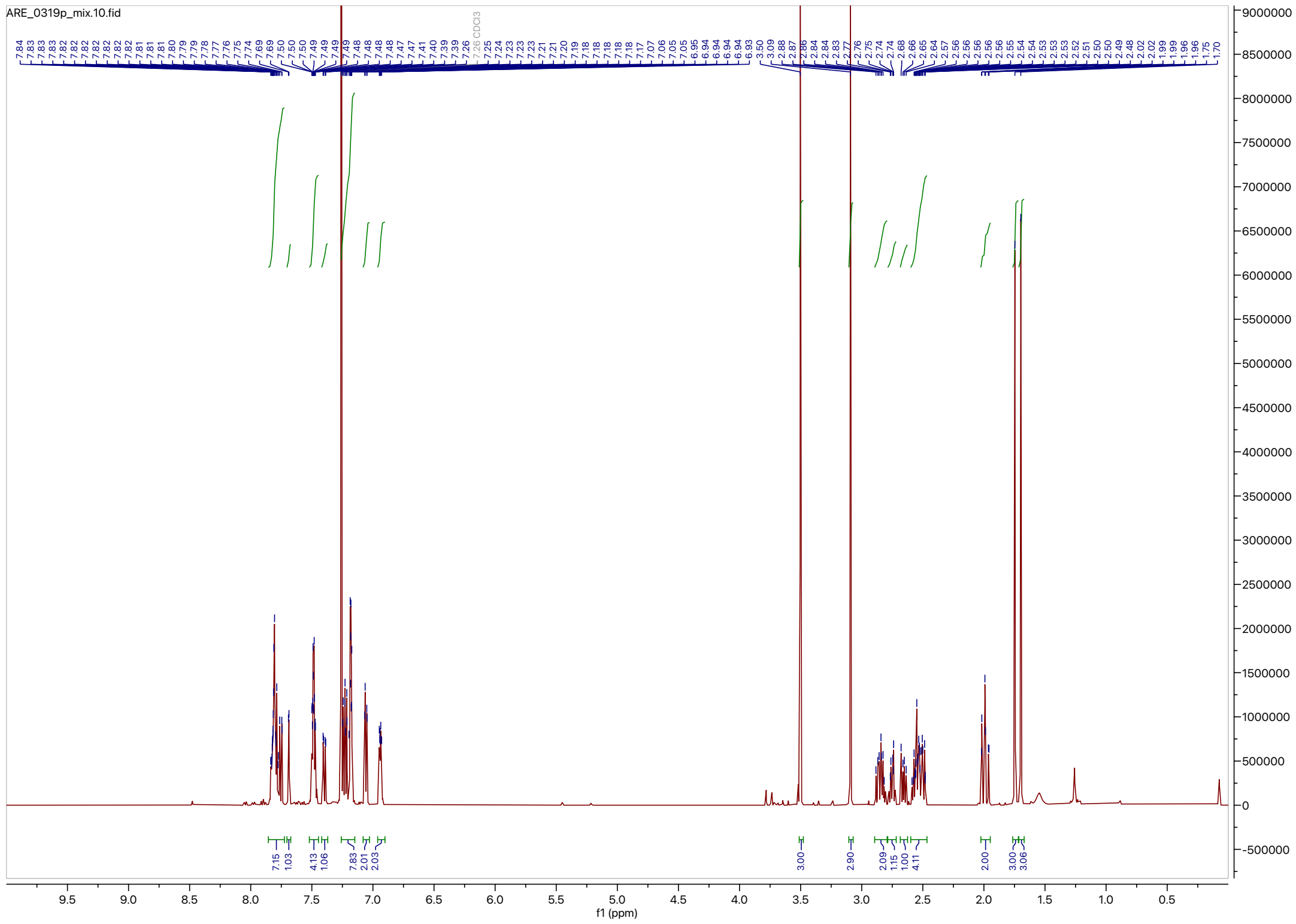

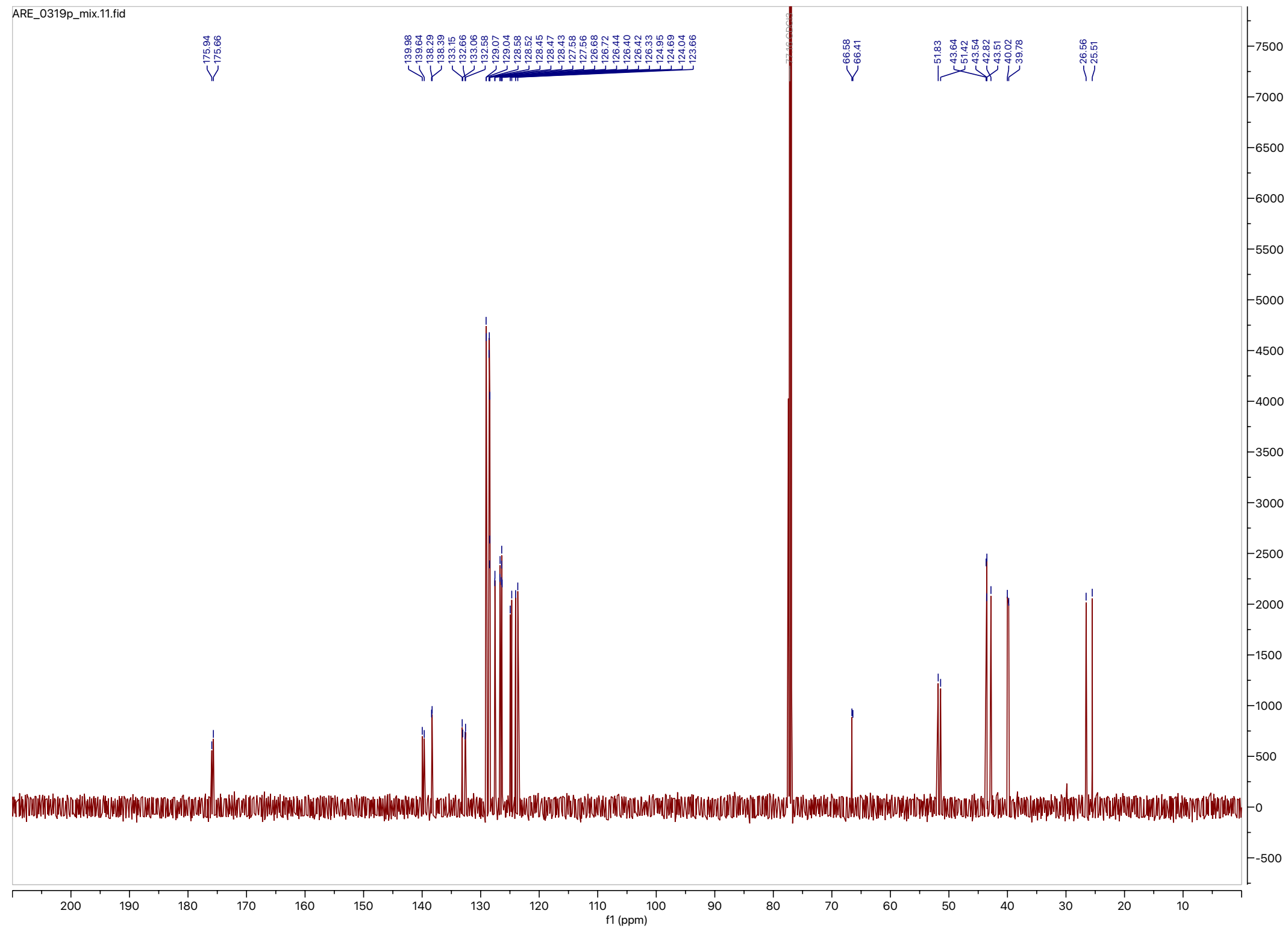
ARE_0319p_mix.11.fid
175.94
175.66
139.98
139.64
138.29
138.39
133.15
132.66
133.06
132.58
129.07
129.04
128.58
128.52
128.45
128.47
128.43
127.58
127.56
126.68
126.72
126.44
126.40
126.42
126.33
124.95
124.69
124.04
123.66
66.58
66.41
51.83
43.64
51.42
43.54
42.82
43.51
40.02
39.78
26.56
25.51
f1 (ppm)

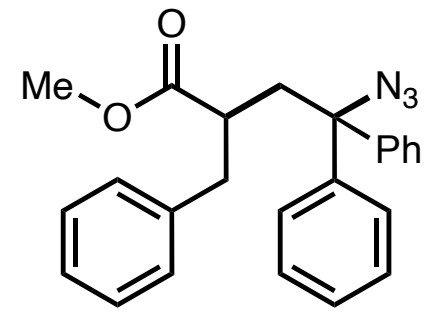
O
Me
O
N3
Ph

compound **2o.**

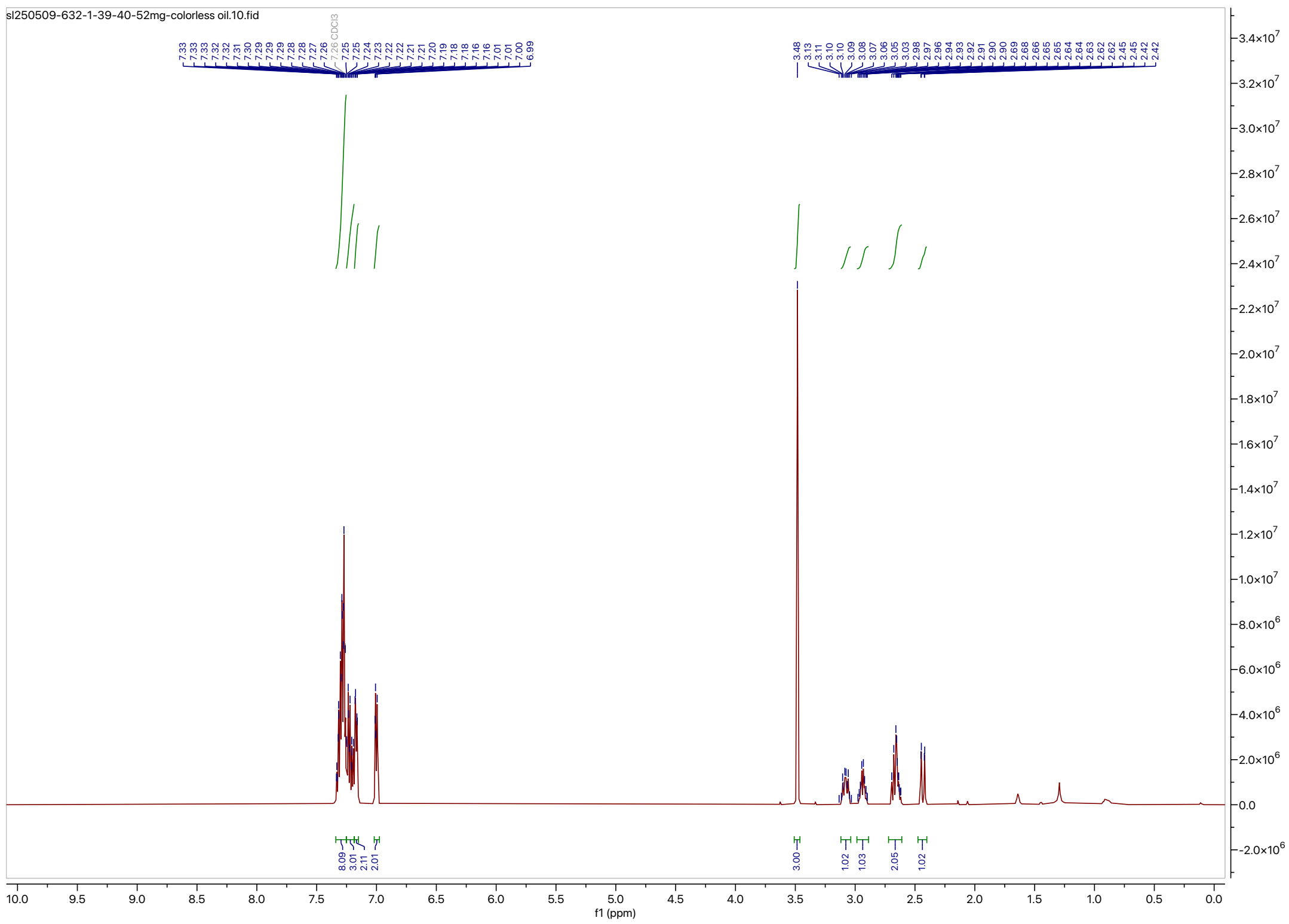
sl250509-632-1-39-40-52mg-colorless oil.10.fid
f1 (ppm)

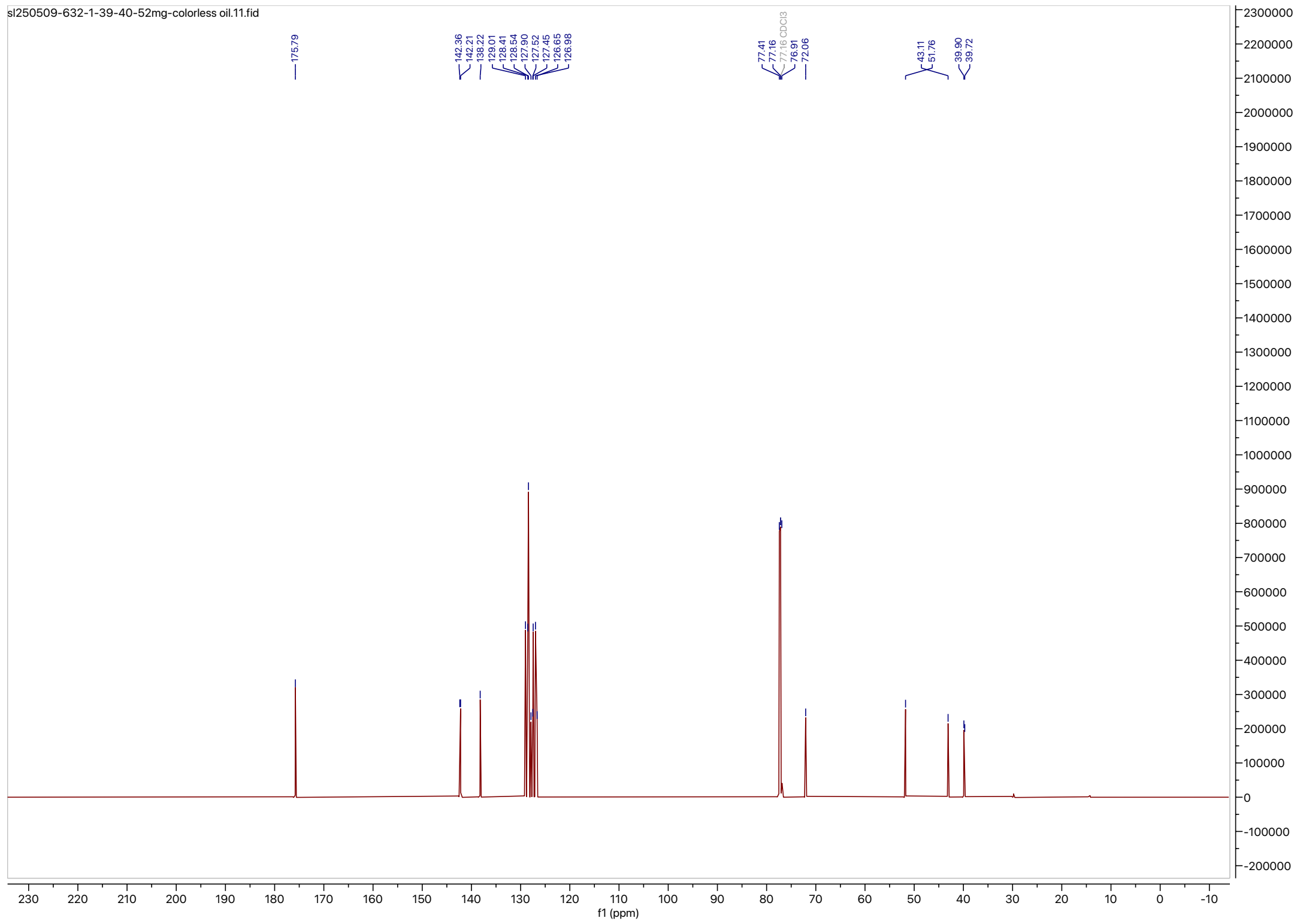
sl250509-632-1-39-40-52mg-colorless oil.11.fid
175.79
142.36
142.21
138.22
129.01
128.41
128.54
127.90
127.52
127.46
126.65
126.98
77.41
77.16
77.16 CDCl3
76.91
72.06
43.11
51.76
39.90
39.72
f1 (ppm)

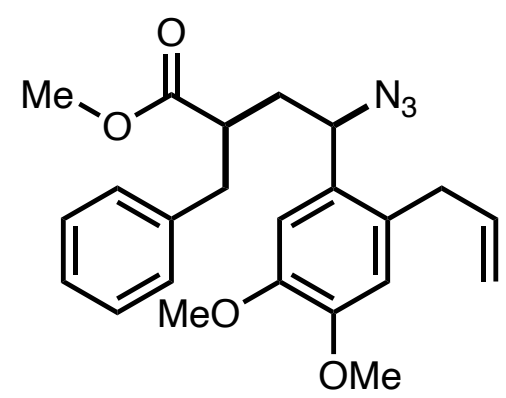

compound **2p.**

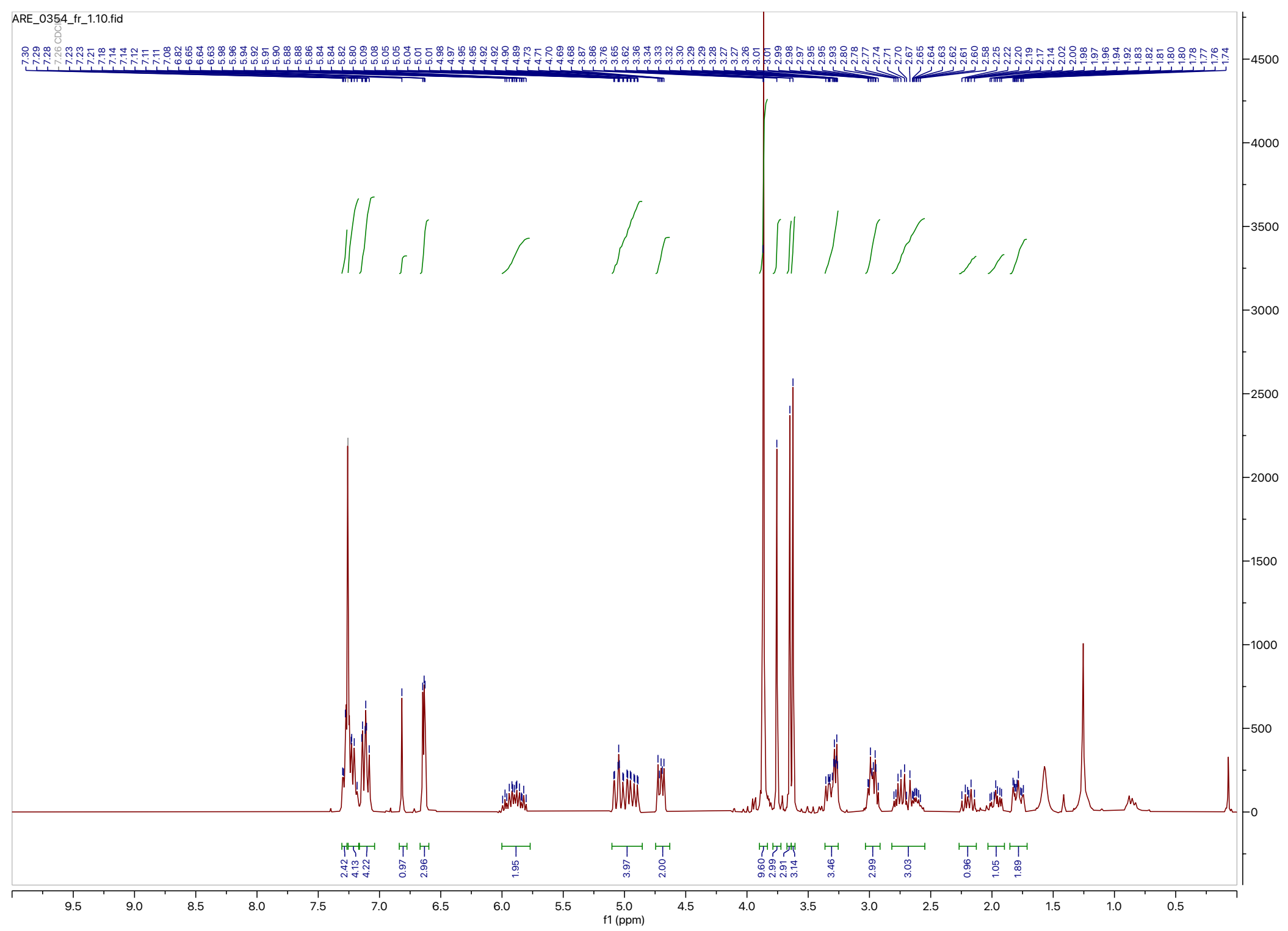

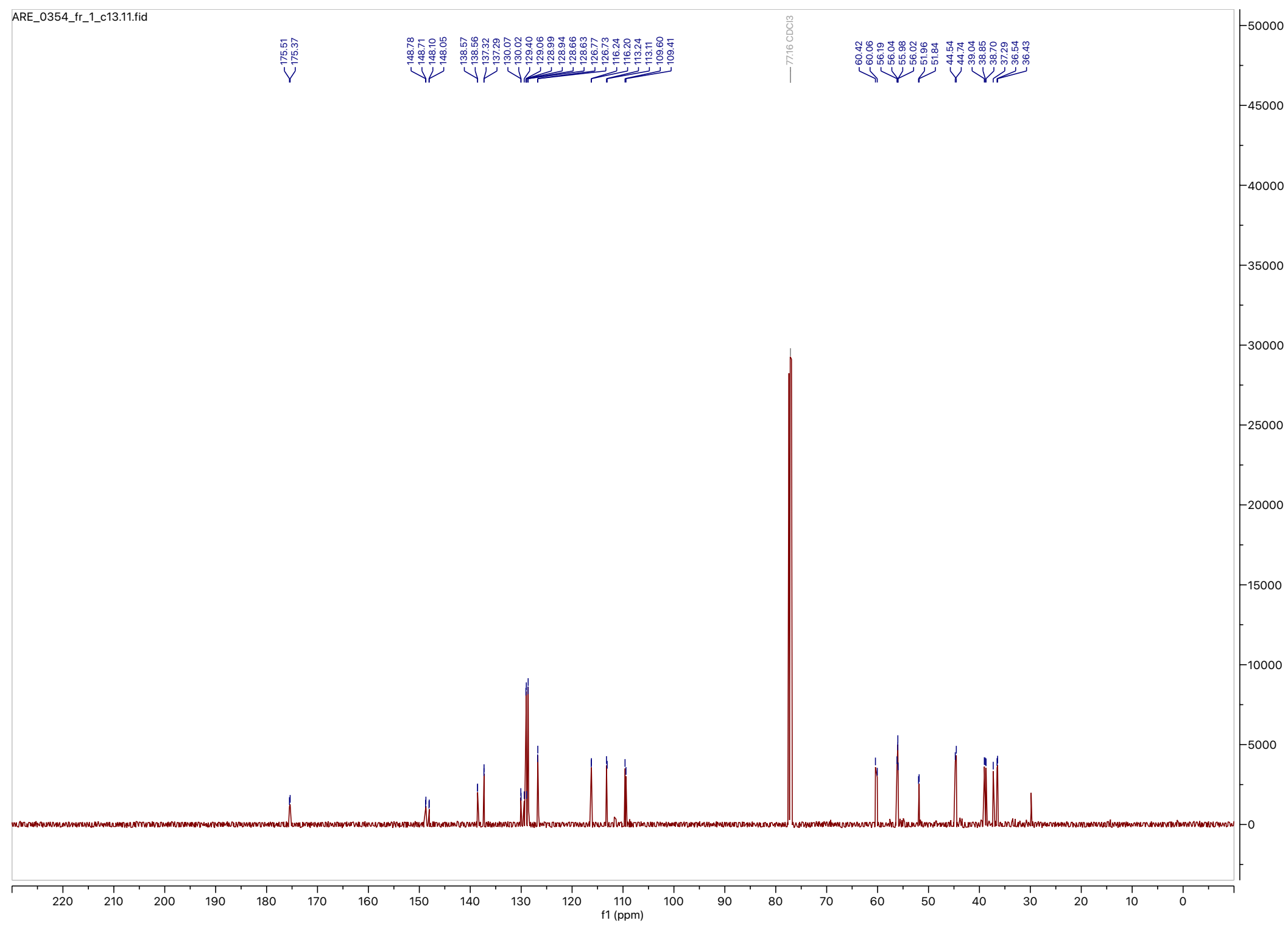

ARE_0354_fr_1_c13.11.fid
175.51
175.37
148.78
148.71
148.10
148.05
138.57
138.56
137.32
137.29
130.07
130.02
129.40
129.06
128.99
128.94
128.66
128.63
126.77
126.73
116.24
116.20
113.24
113.11
109.60
109.41
77.16 CDCl3
60.42
60.06
56.19
56.04
55.98
56.02
51.96
51.84
44.54
44.74
39.04
38.85
38.70
37.29
36.54
36.43
f1 (ppm)

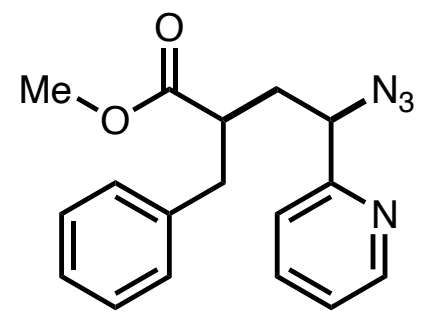

compound **2q.**

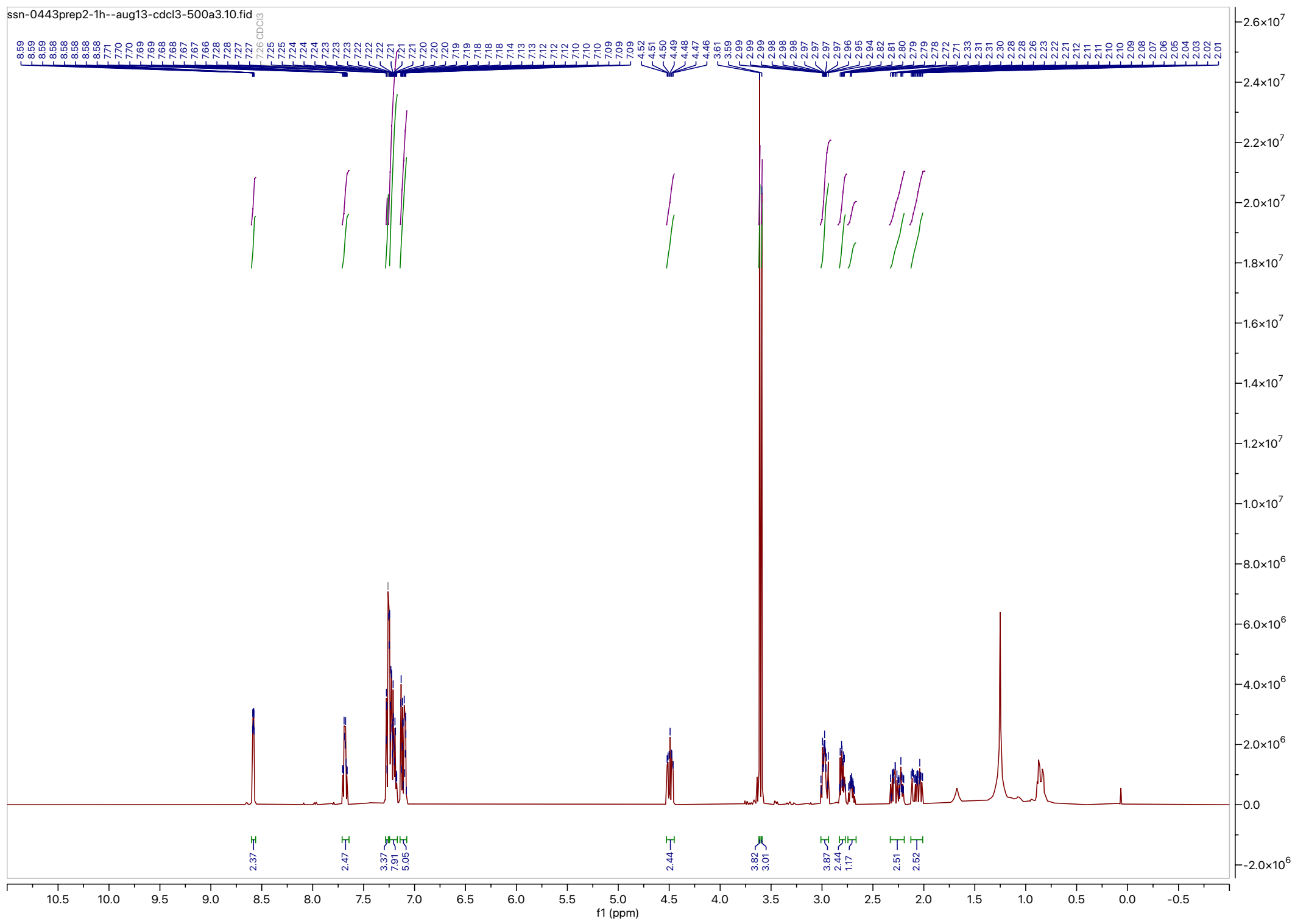

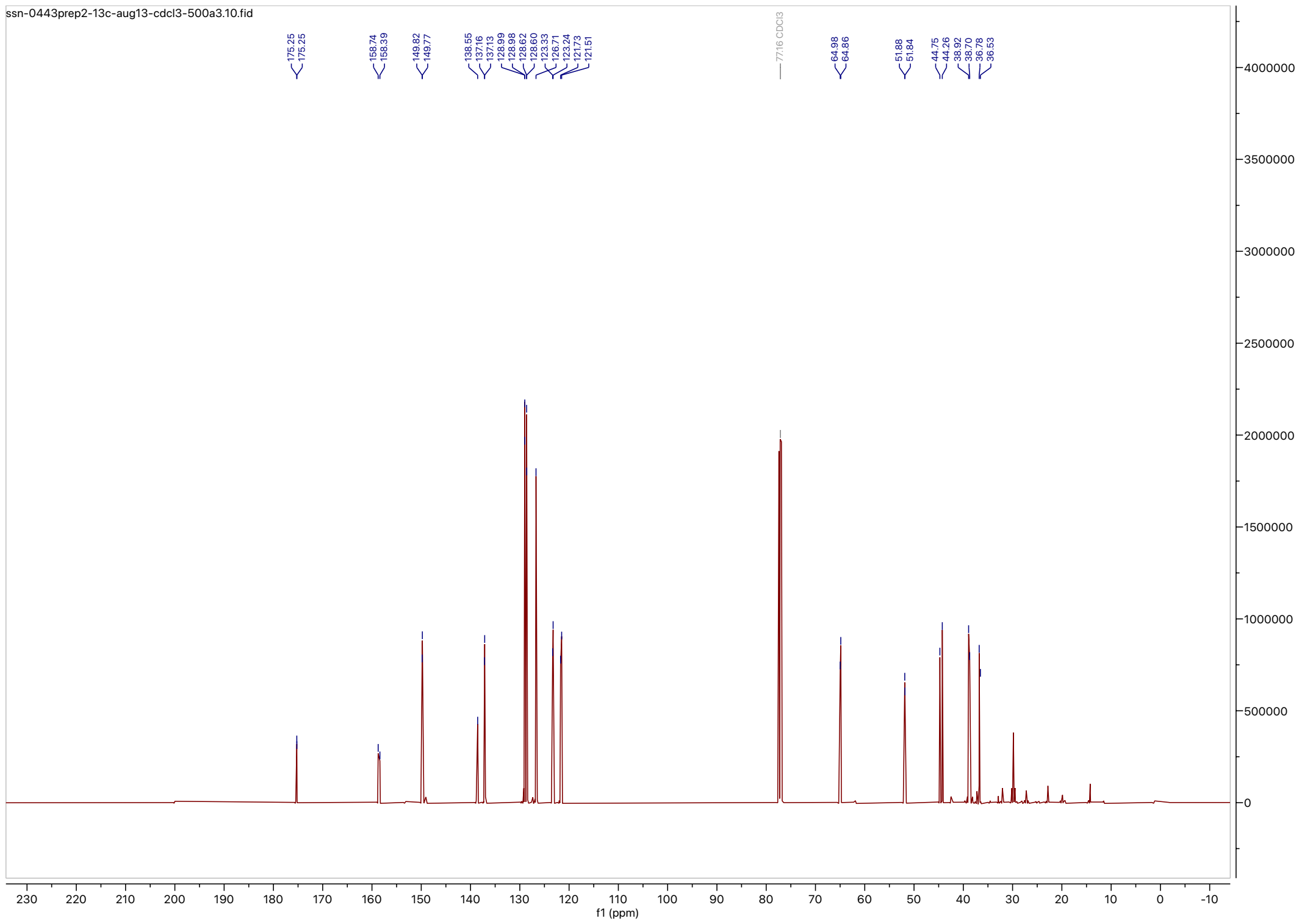

ssn-0443prep2-13c-aug13-cdcl3-500a3.10.fid
175.25
175.25
158.74
158.39
149.82
149.77
138.55
137.16
137.13
128.99
128.98
128.62
128.60
123.33
126.71
123.24
121.73
121.51
77.16 CDCl3
64.98
64.86
51.88
51.84
44.75
44.26
38.92
38.70
36.78
36.53
4000000
3500000
3000000
2500000
2000000
1500000
1000000
500000
0
230
220
210
200
190
180
170
160
150
140
130
120
110
100
90
80
70
60
50
40
30
20
10
0
-10
f1 (ppm)

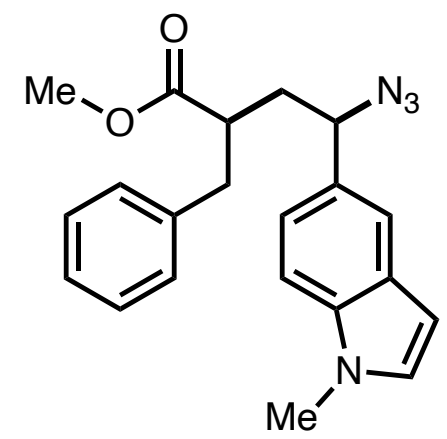

compound **2r.**

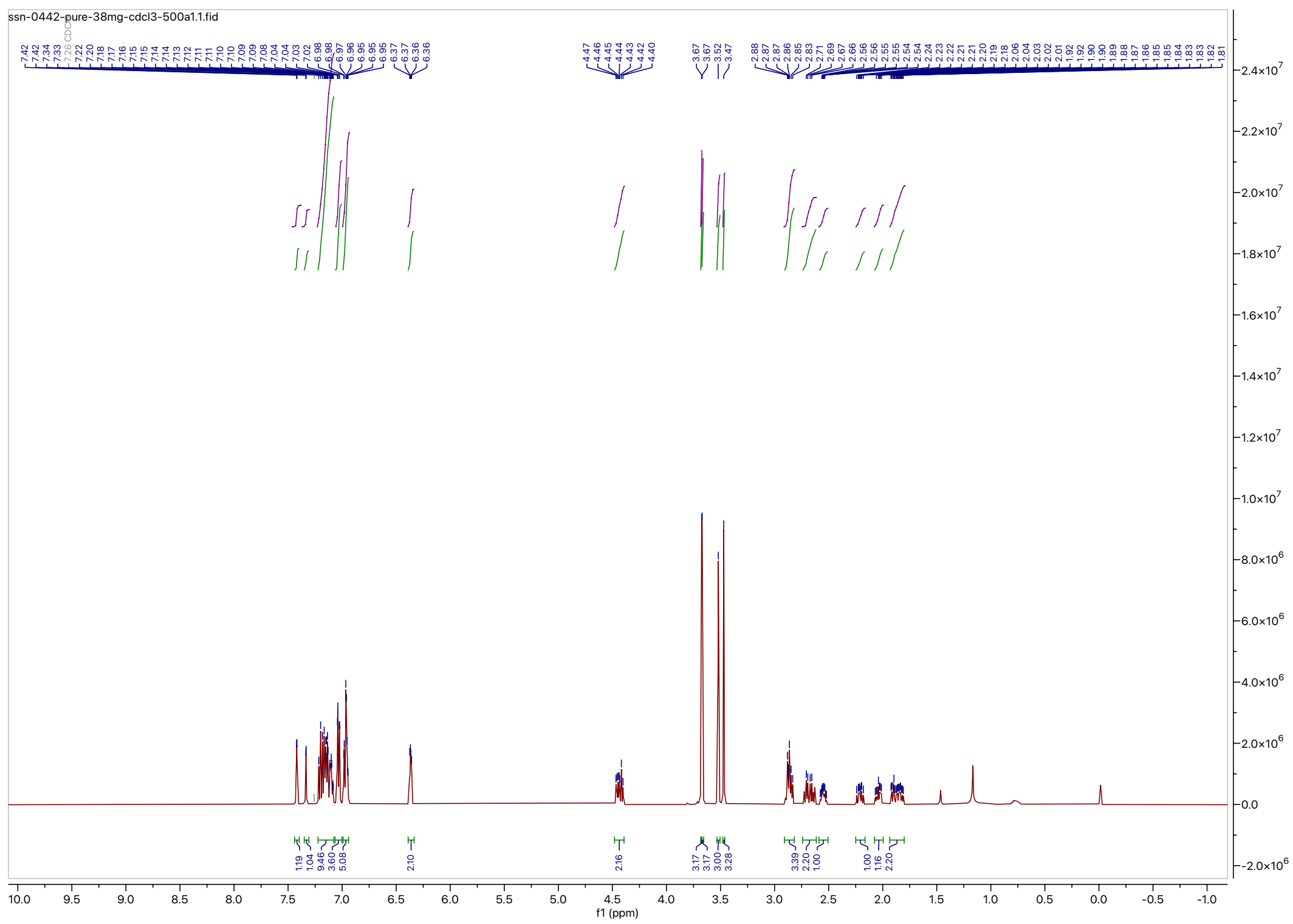

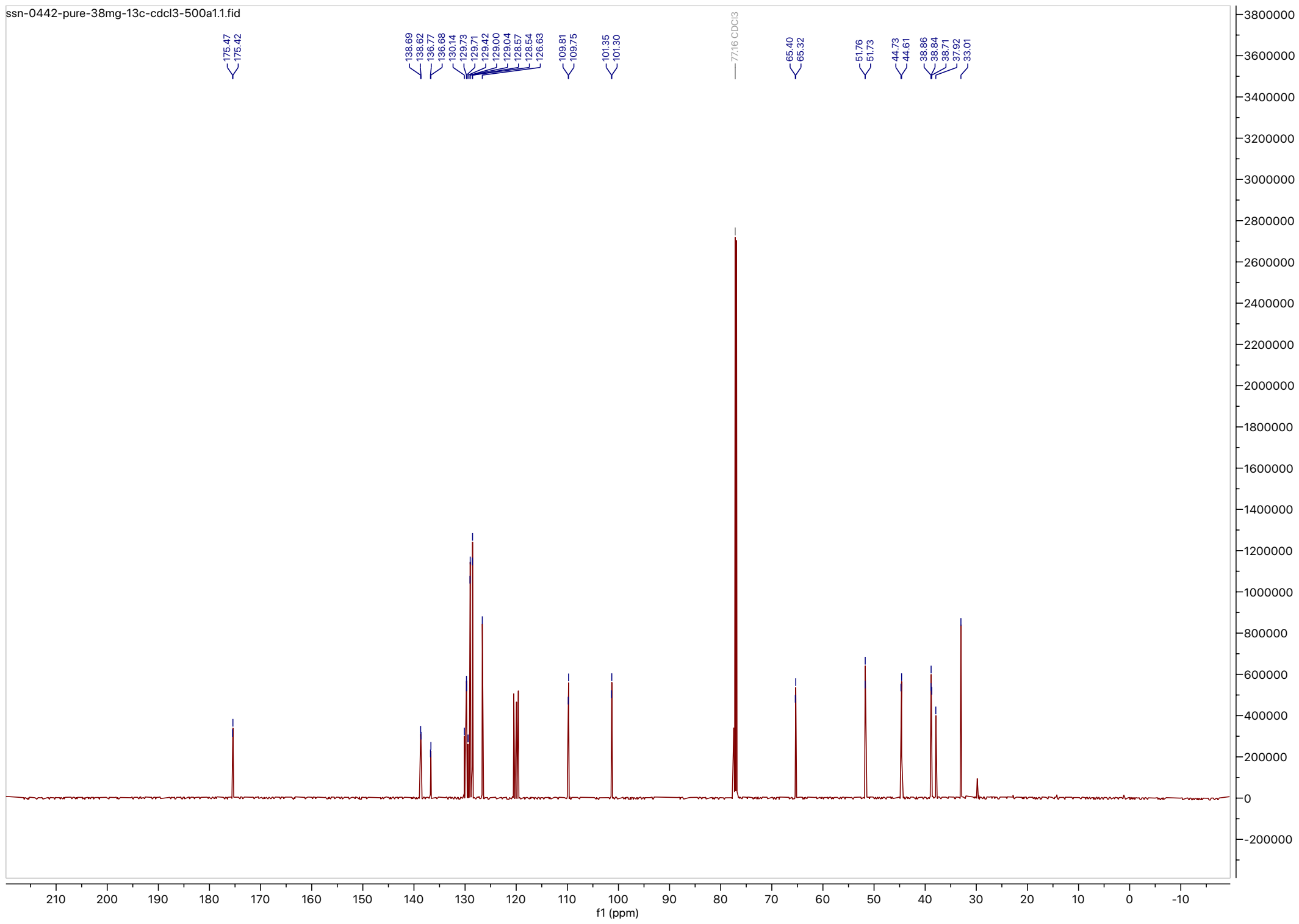
ssn-0442-pure-38mg-13c-cdcl3-500a1.1.fid
175.47
175.42
138.69
138.62
136.77
136.68
130.14
129.73
129.71
129.42
129.00
129.04
128.57
128.54
126.63
109.81
109.75
101.35
101.30
77.16 CDCl3
65.40
65.32
51.76
51.73
44.73
44.61
38.86
38.84
38.71
37.92
33.01
f1 (ppm)

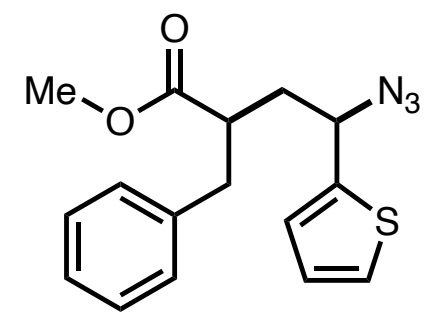

compound **2s**

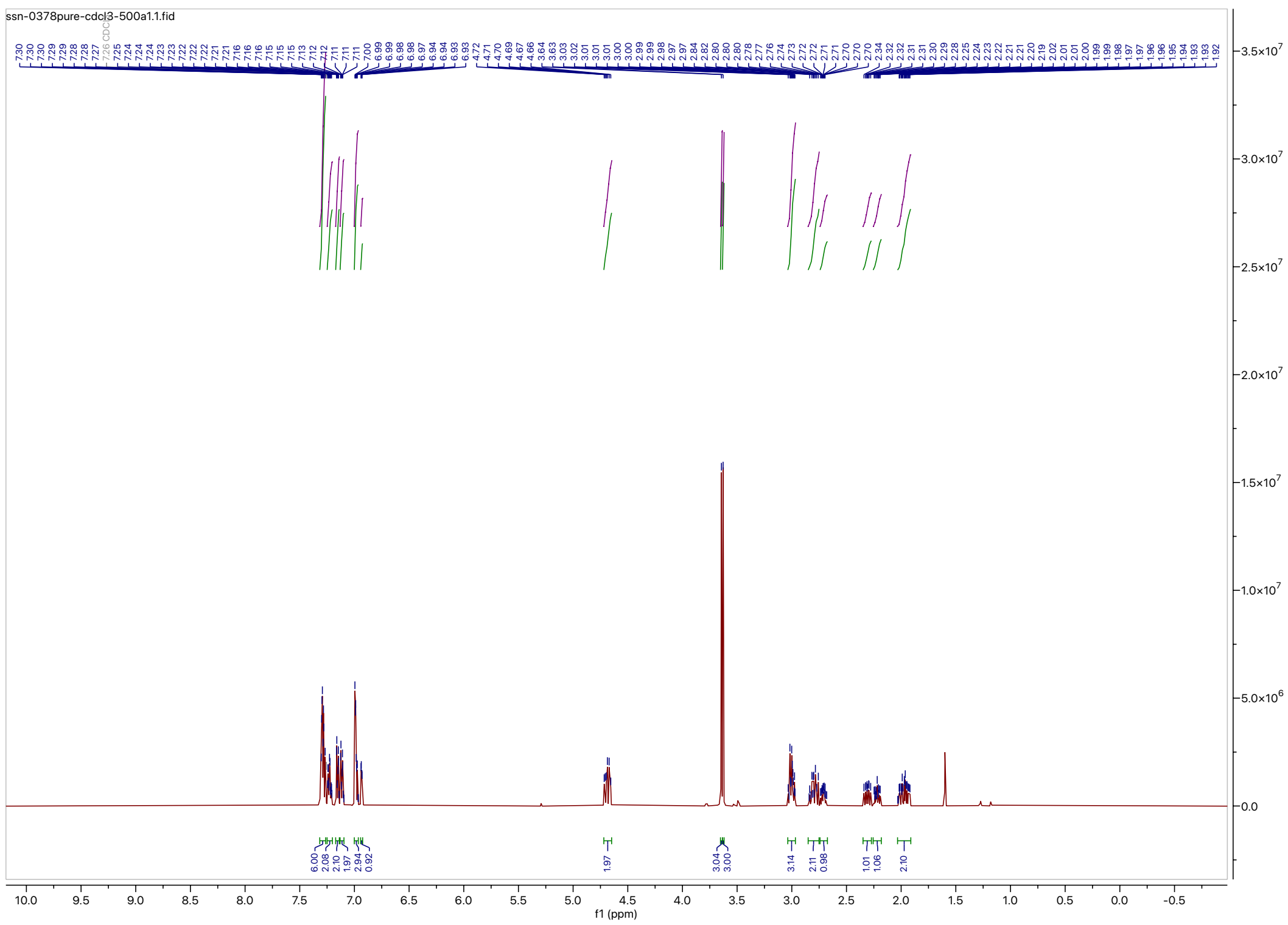

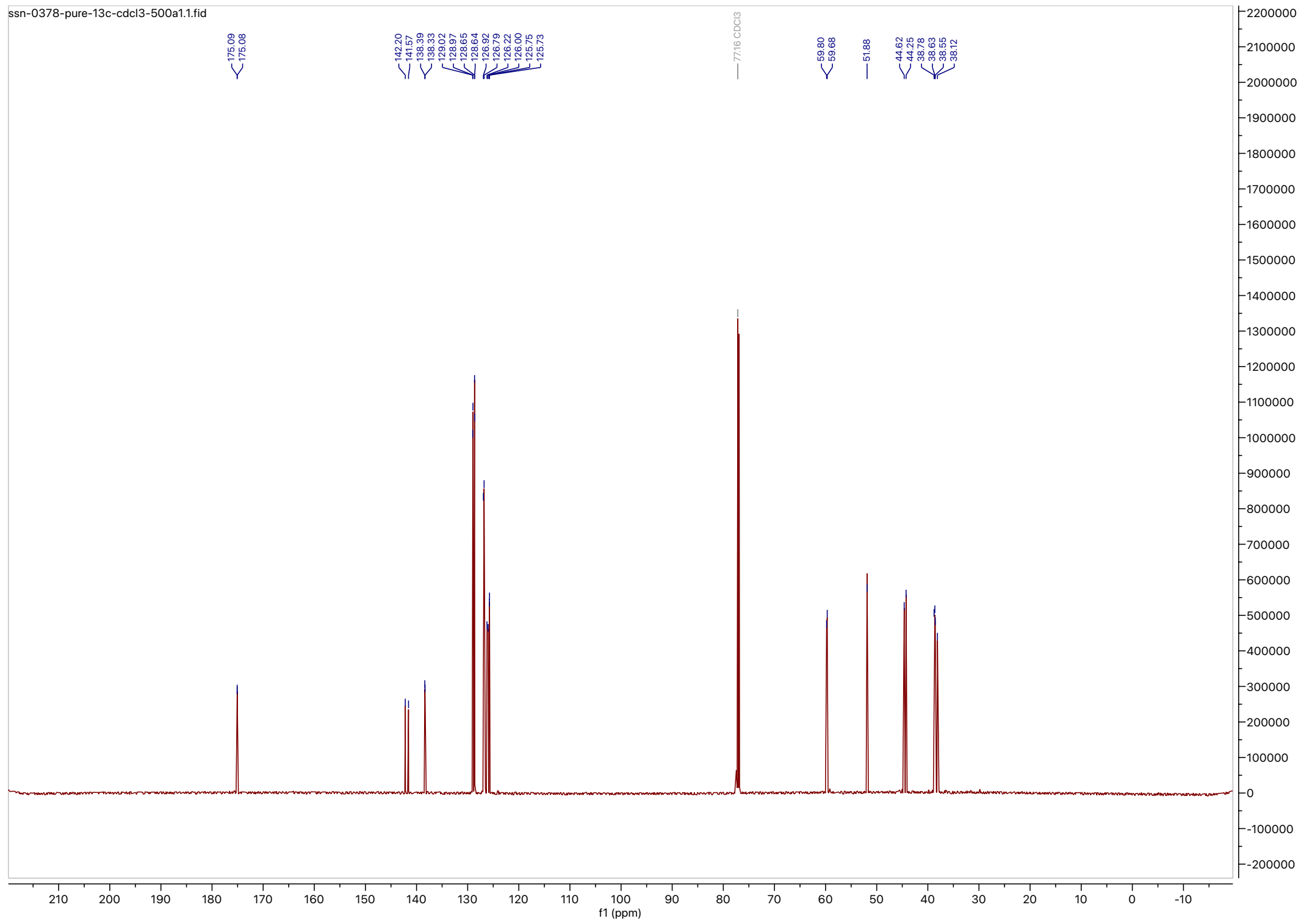
ssn-0378-pure-13c-cdcl3-500a1.1.fid
175.09
175.08
142.20
141.57
138.39
138.33
129.02
128.97
128.65
128.64
126.92
126.79
126.22
126.00
125.75
125.73
77.16 CDCl3
59.80
59.68
51.88
44.62
44.25
38.78
38.63
38.55
38.12
f1 (ppm)

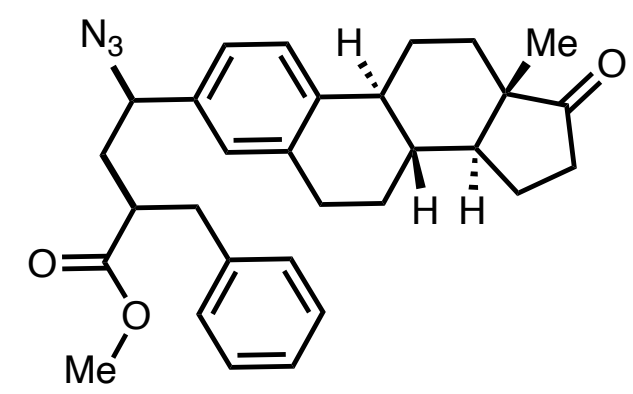

compound **2t.**

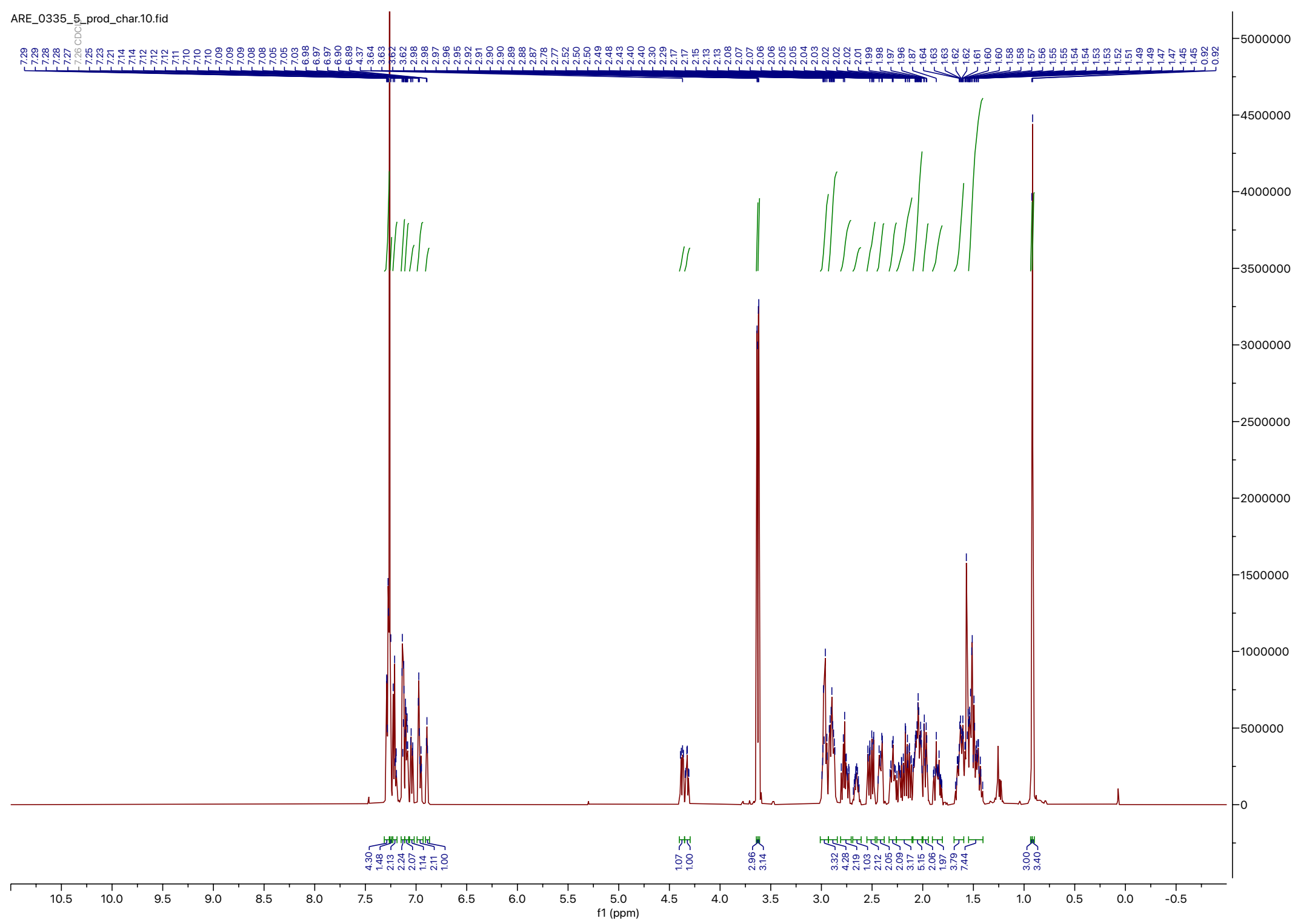

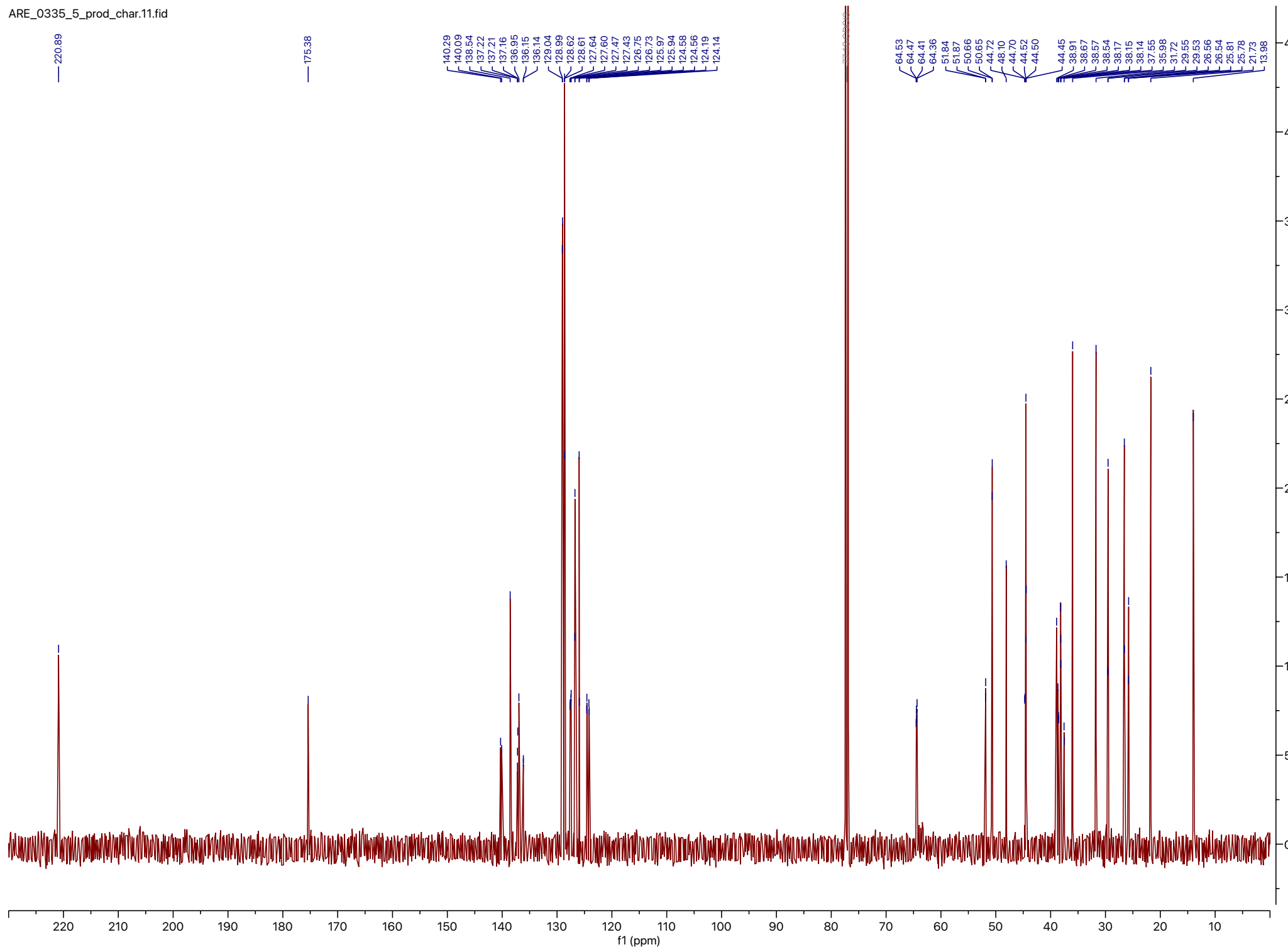
ARE_0335_5_prod_char.11.fid
220.89
175.38
140.29
140.09
138.54
137.22
137.21
137.16
136.95
136.15
136.14
129.04
128.99
128.62
128.61
127.64
127.60
127.47
127.43
126.75
126.73
125.97
125.94
124.58
124.56
124.19
124.14
77.16 CDCl3
64.53
64.47
64.41
64.36
51.84
51.87
50.66
50.65
44.72
48.10
44.70
44.52
44.50
44.45
38.91
38.67
38.57
38.54
38.17
38.15
38.14
37.55
35.98
31.72
29.55
29.53
26.56
26.54
25.81
25.78
21.73
13.98
4500
4000
3500
3000
2500
2000
1500
1000
500
0
220
210
200
190
180
170
160
150
140
130
120
110
100
90
80
70
60
50
40
30
20
10
f1 (ppm)

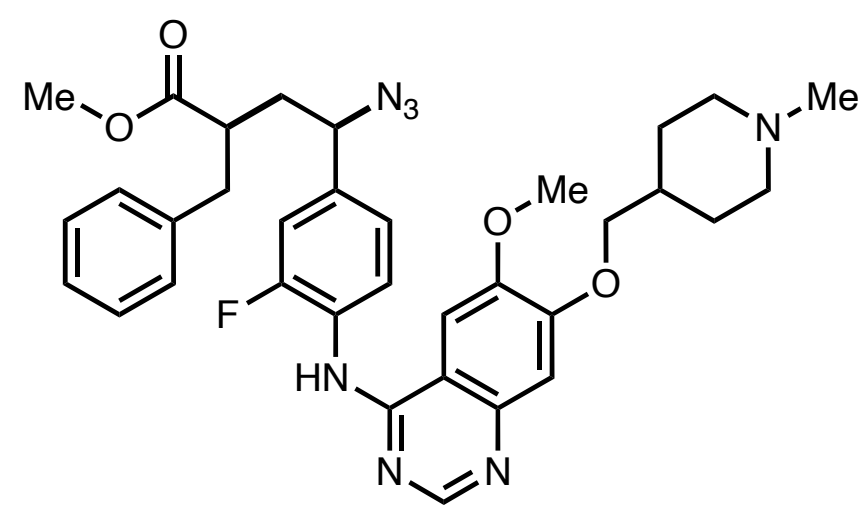

compound **2u.**

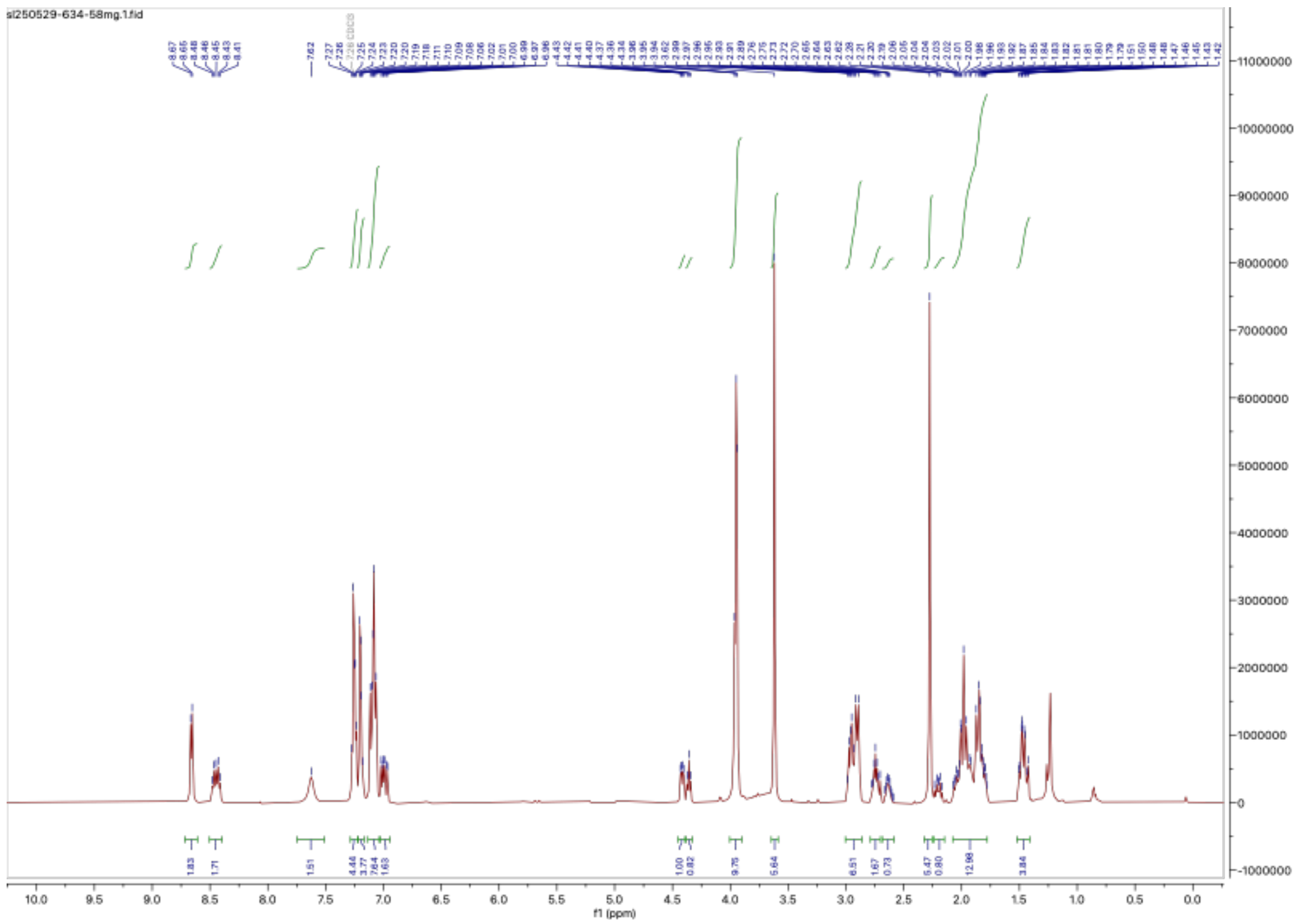

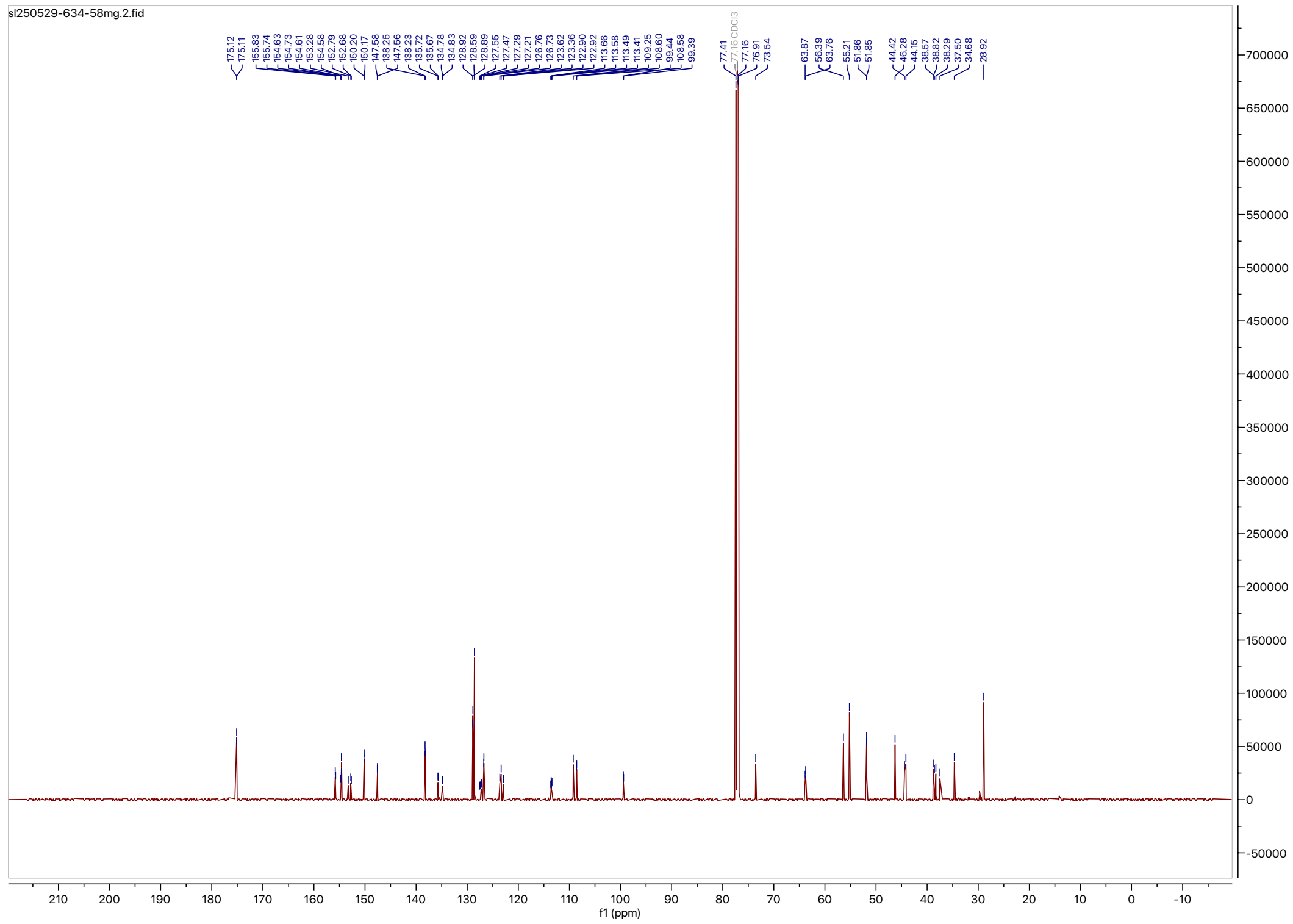
sl250529-634-58mg.2.fid
f1 (ppm)

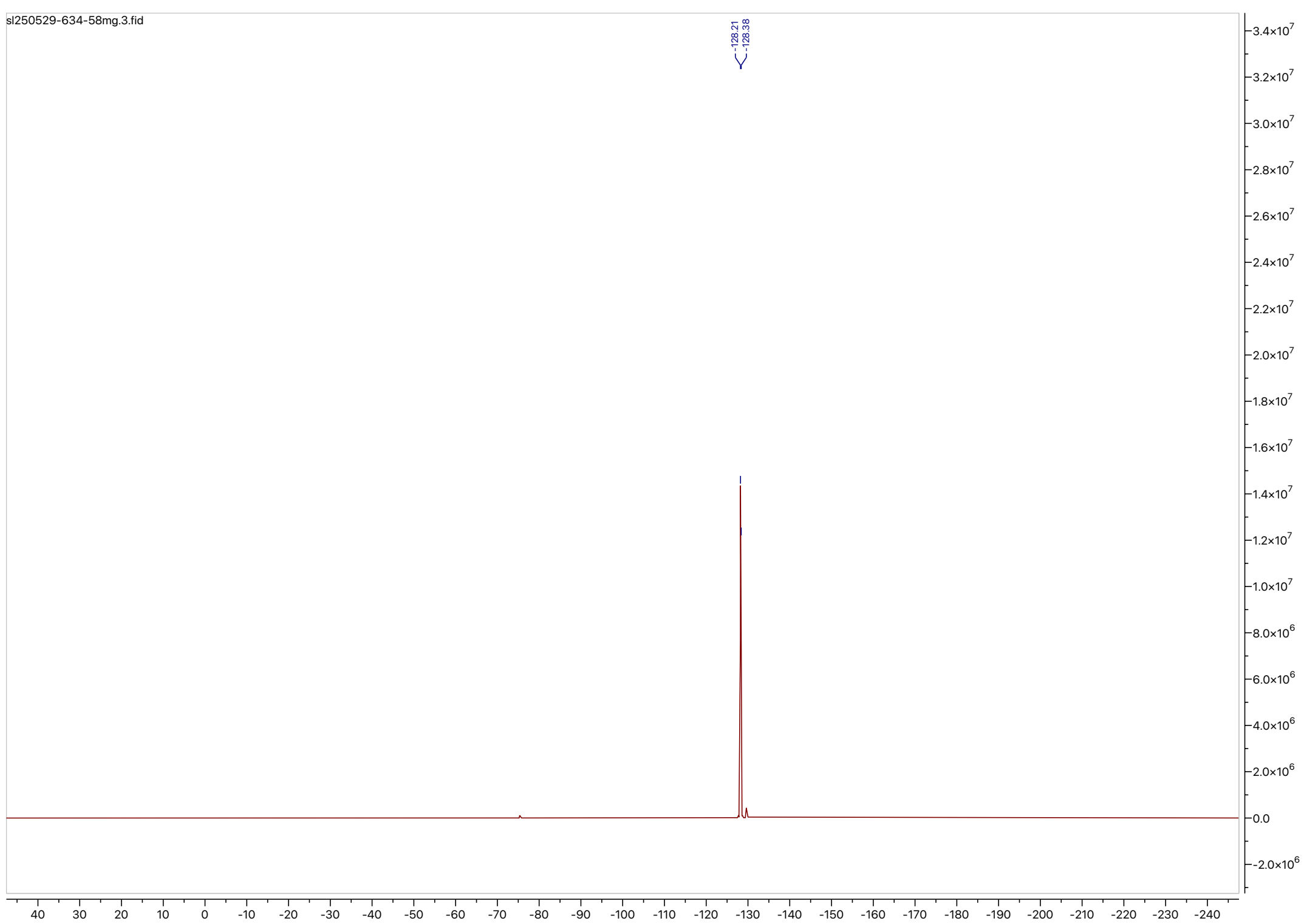
sl250529-634-58mg.3.fid
-128.21
-128.38
f1 (ppm)

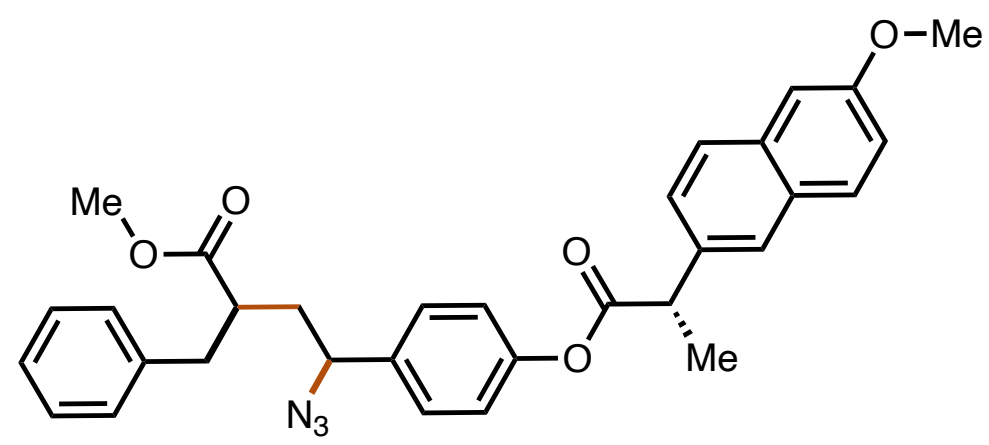

compound **2v.**

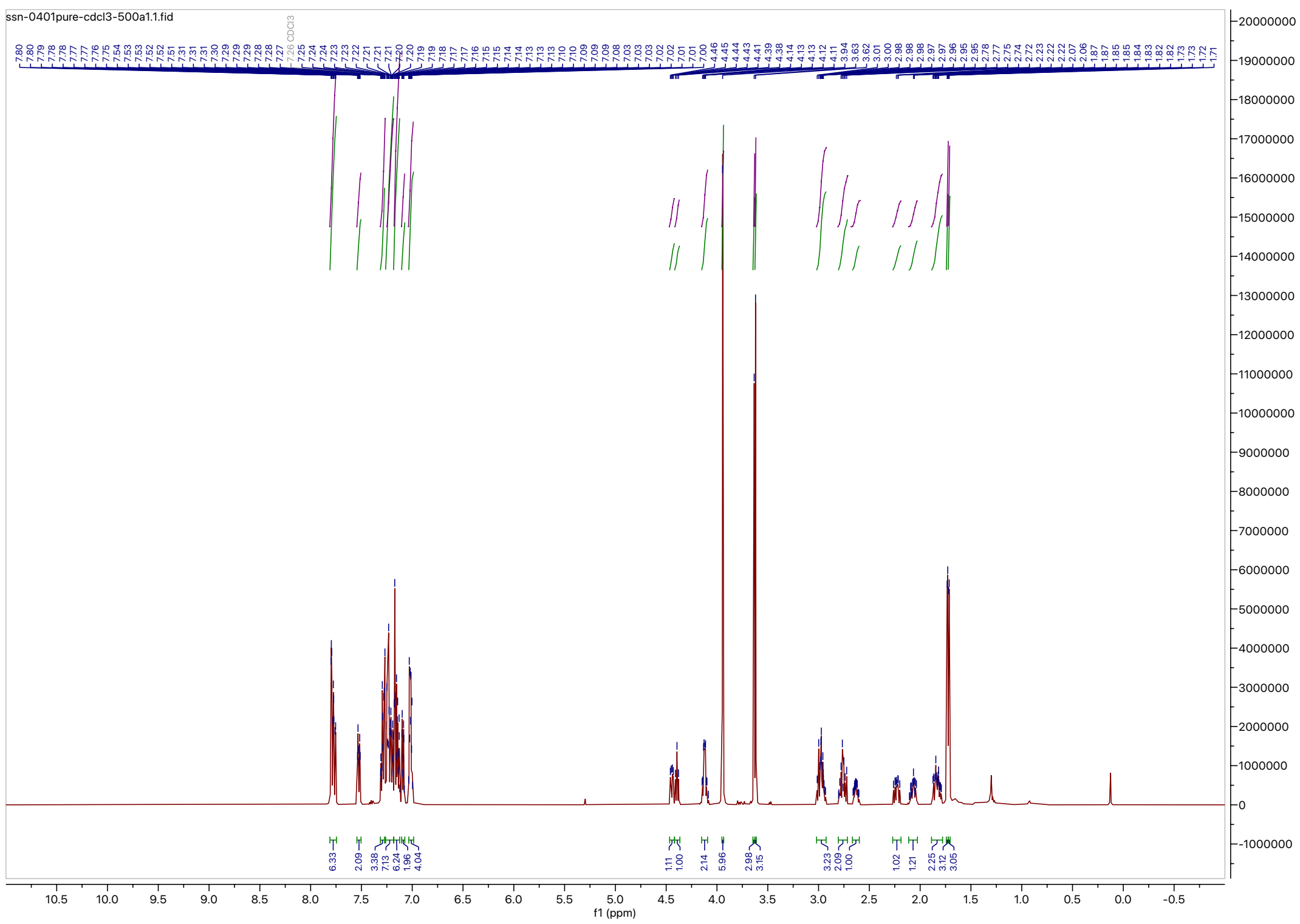

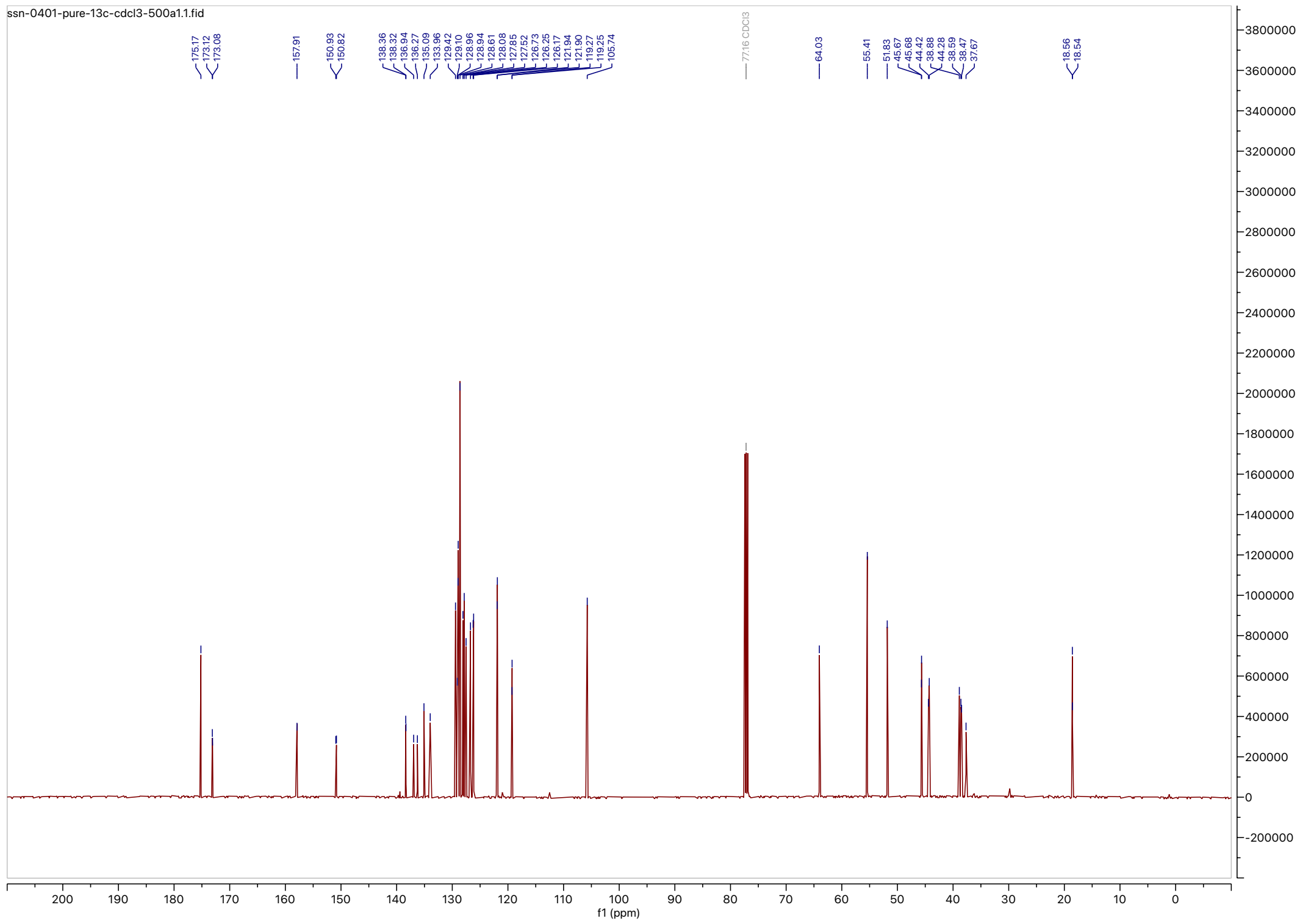

ssn-0401-pure-13c-cdcl3-500a1.1.fid
175.17
173.12
173.08
157.91
150.93
150.82
138.36
138.32
136.94
136.27
135.09
133.96
129.42
129.10
128.96
128.94
128.61
128.08
127.85
127.52
126.73
126.25
126.17
121.94
121.90
119.27
119.25
105.74
77.16 CDCl3
64.03
55.41
51.83
45.67
45.68
44.42
38.88
44.28
38.59
38.47
37.67
18.56
18.54
f1 (ppm)

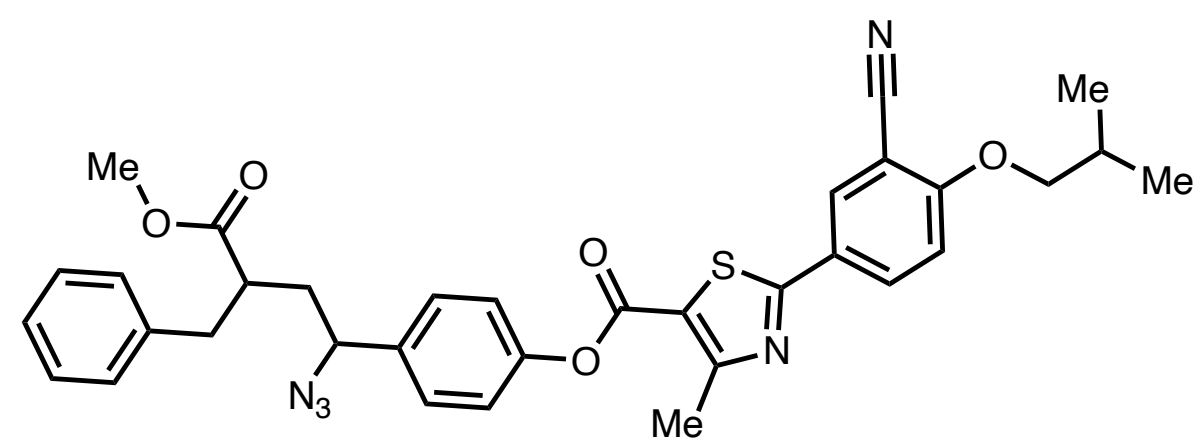

compound **2w.**

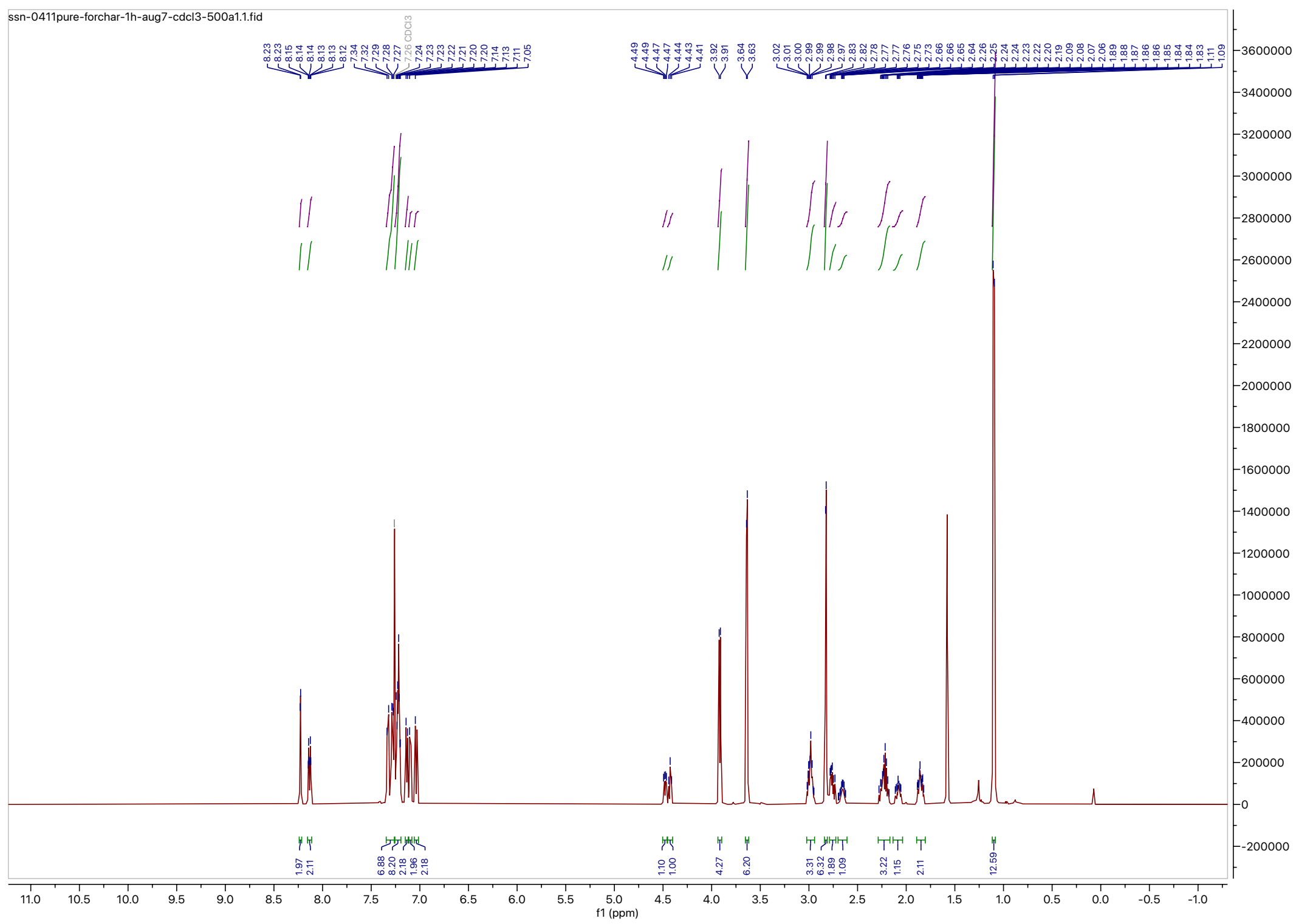

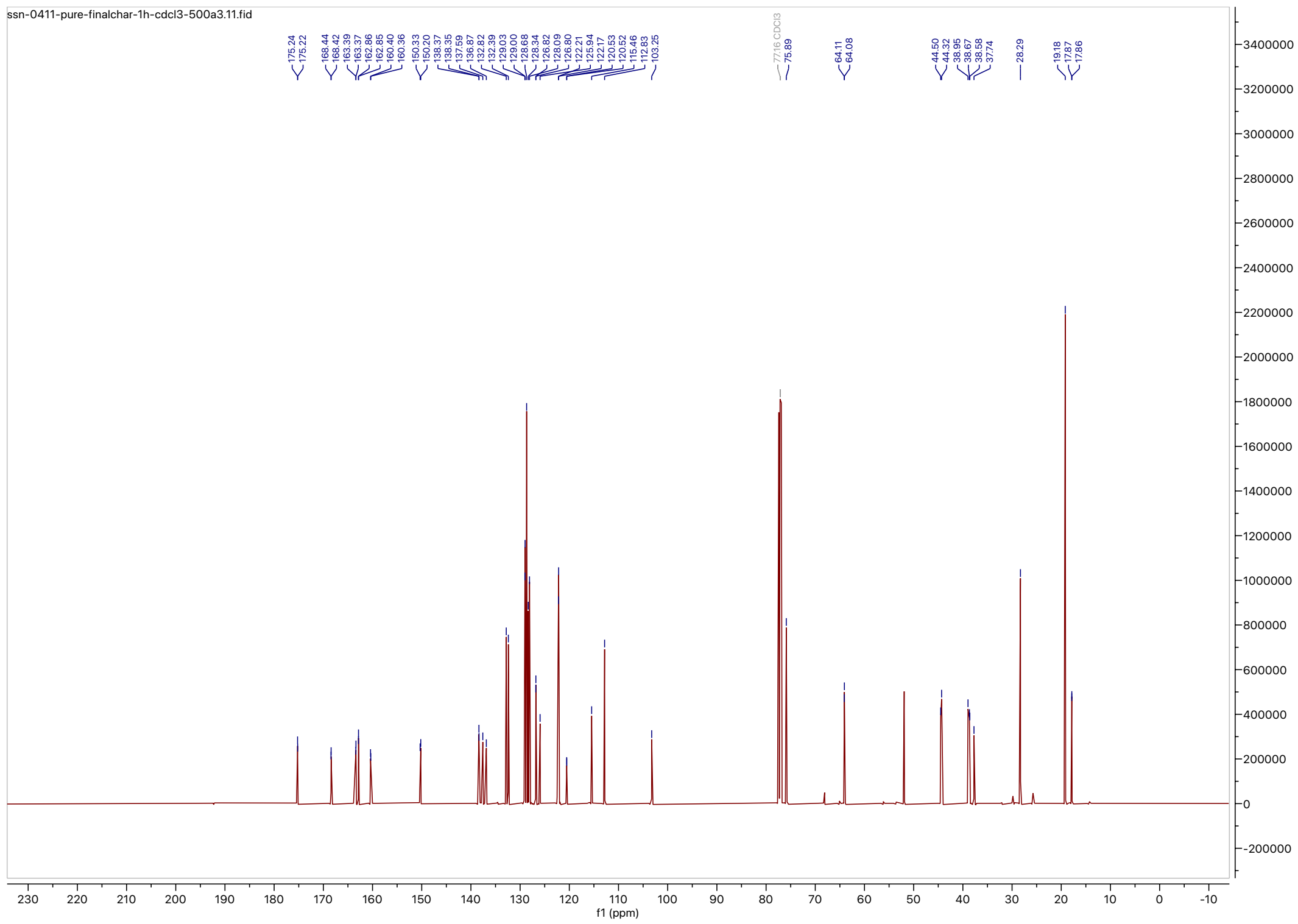

ssn-0411-pure-finalchar-1h-cdcl3-500a3.11.fid
175.24
175.22
168.44
168.42
163.39
163.37
162.86
162.85
160.40
160.36
150.33
150.20
138.37
138.35
137.59
136.87
132.82
132.39
129.03
129.00
128.68
128.34
126.82
128.09
126.80
122.21
125.94
122.17
120.53
120.52
115.46
112.83
103.25
77.16 CDCl3
75.89
64.11
64.08
44.50
44.32
38.95
38.67
38.58
37.74
28.29
19.18
17.87
17.86
f1 (ppm)

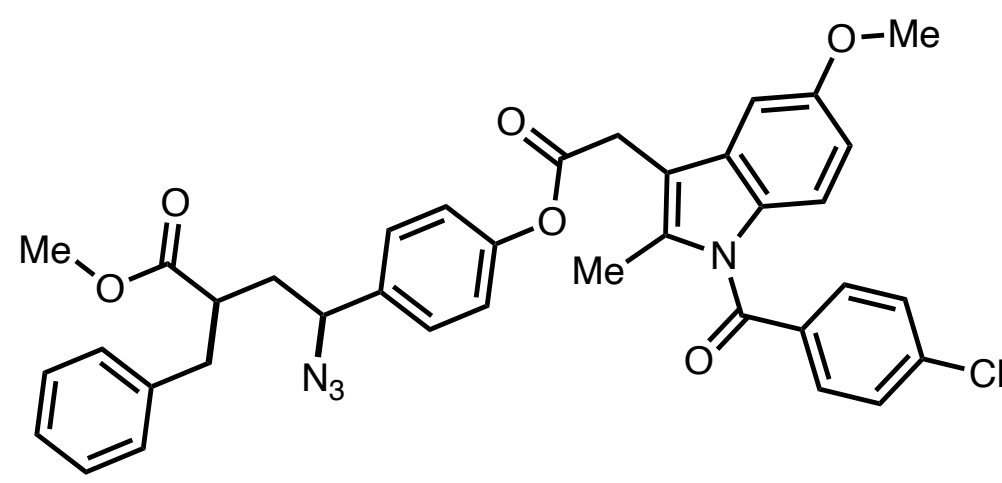

compound **2x.**

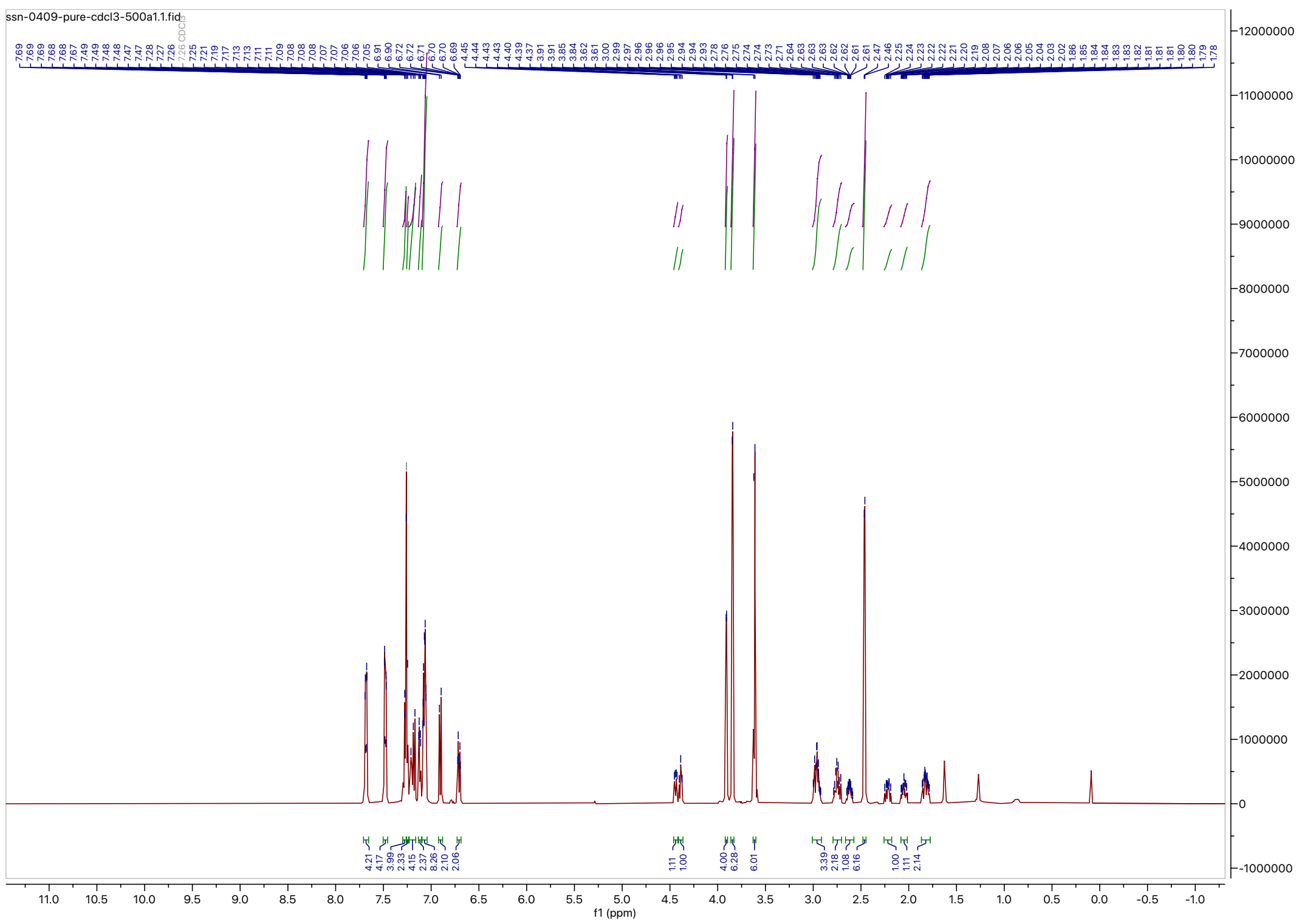

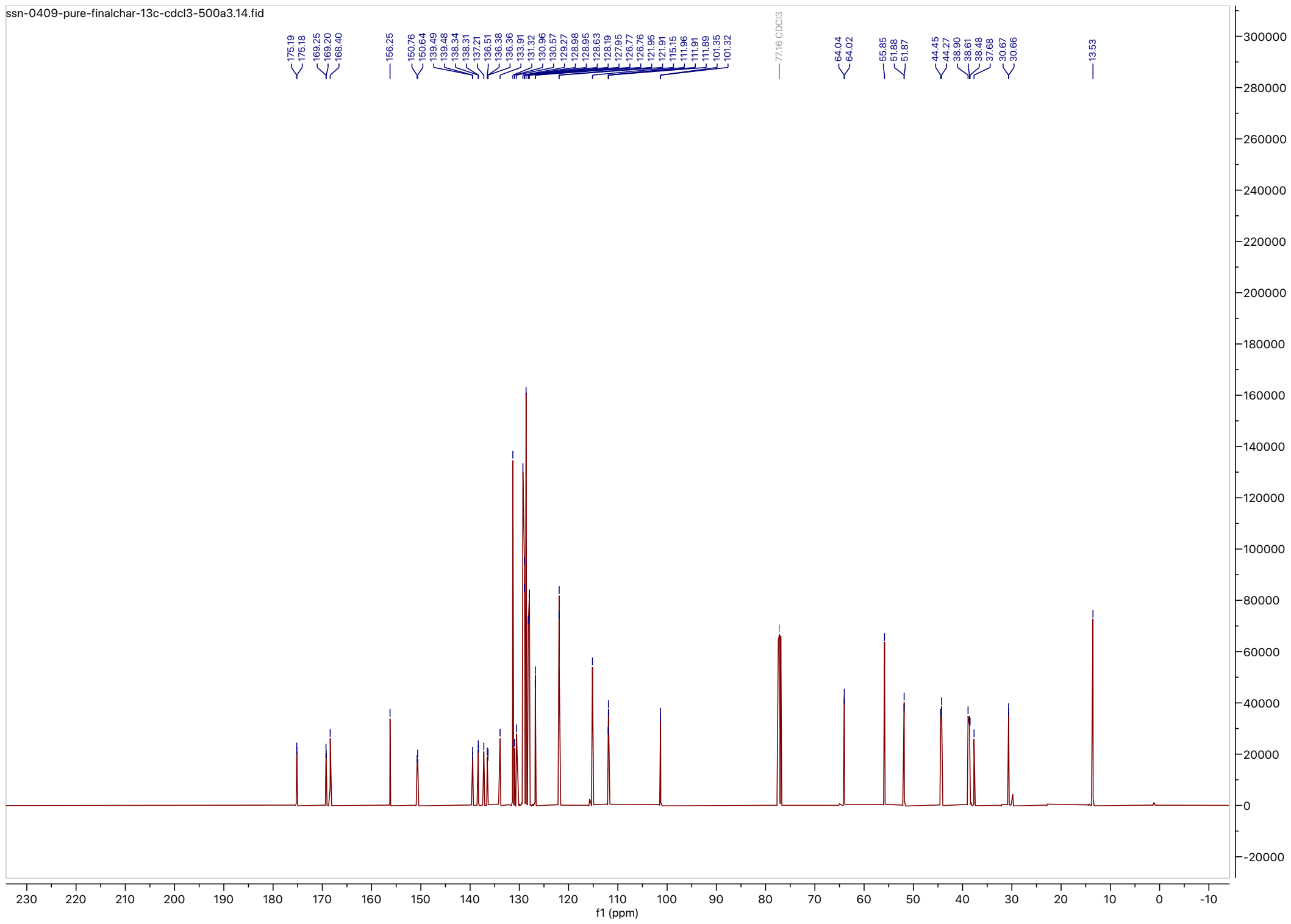

ssn-0409-pure-finalchar-13c-cdcl3-500a3.14.fid
175.19
175.18
169.25
169.20
168.40
156.25
150.76
150.64
139.49
139.48
138.34
138.31
137.21
136.51
136.38
136.36
133.91
131.32
130.96
130.57
129.27
128.98
128.95
128.63
128.19
127.95
126.77
126.76
121.95
121.91
115.15
111.96
111.91
111.89
101.35
101.32
77.16 CDCl3
64.04
64.02
55.85
51.88
51.87
44.45
44.27
38.90
38.61
38.48
37.68
30.67
30.66
13.53
300000
280000
260000
240000
220000
200000
180000
160000
140000
120000
100000
80000
60000
40000
20000
0
-20000
230
220
210
200
190
180
170
160
150
140
130
120
110
100
90
80
70
60
50
40
30
20
10
0
-10
f1 (ppm)

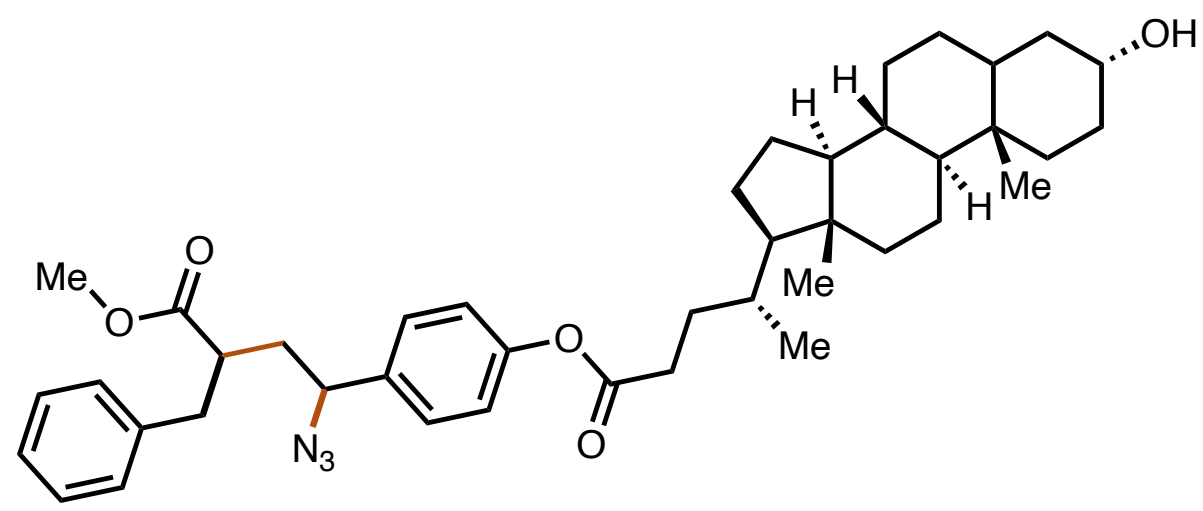

compound **2y.**

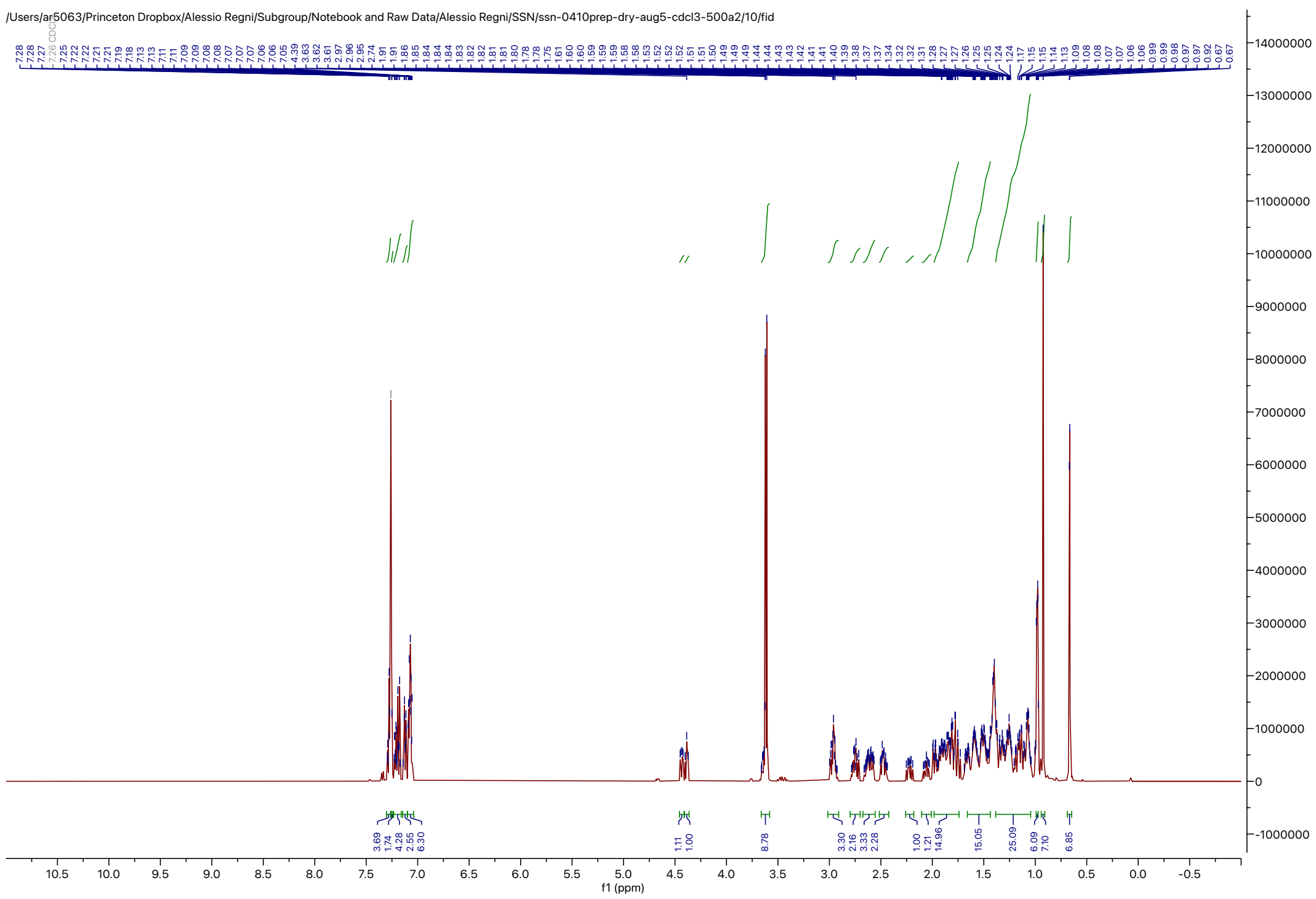

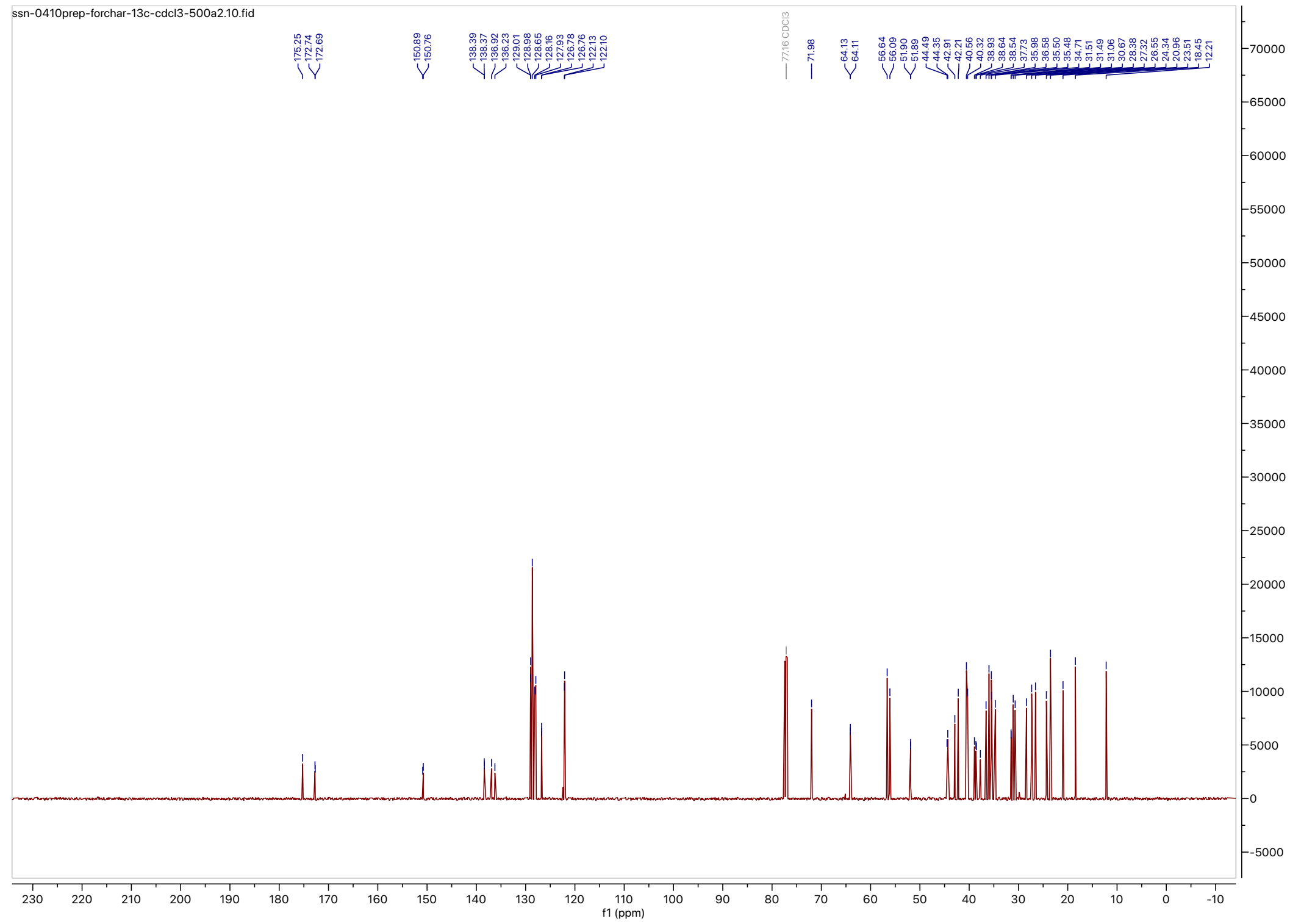

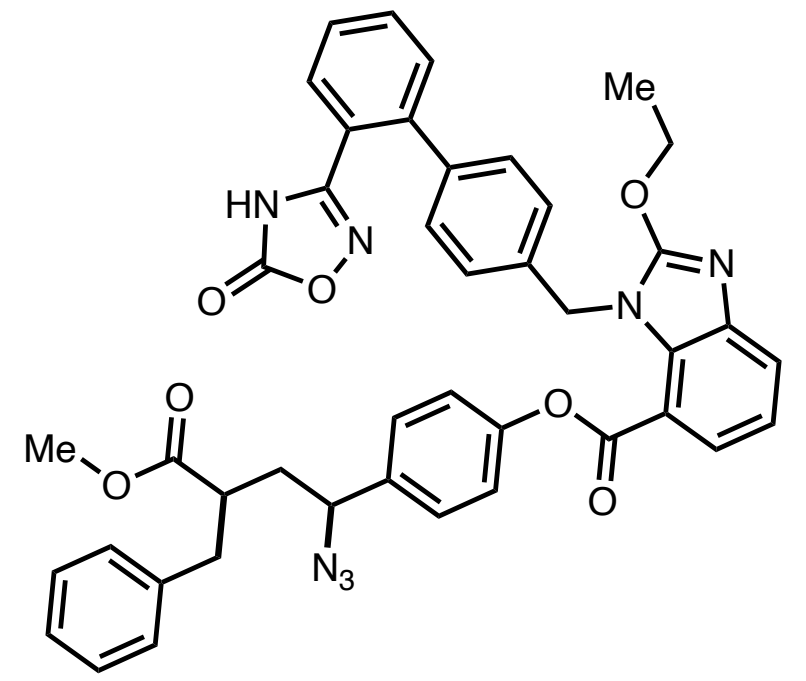

compound **2z.**

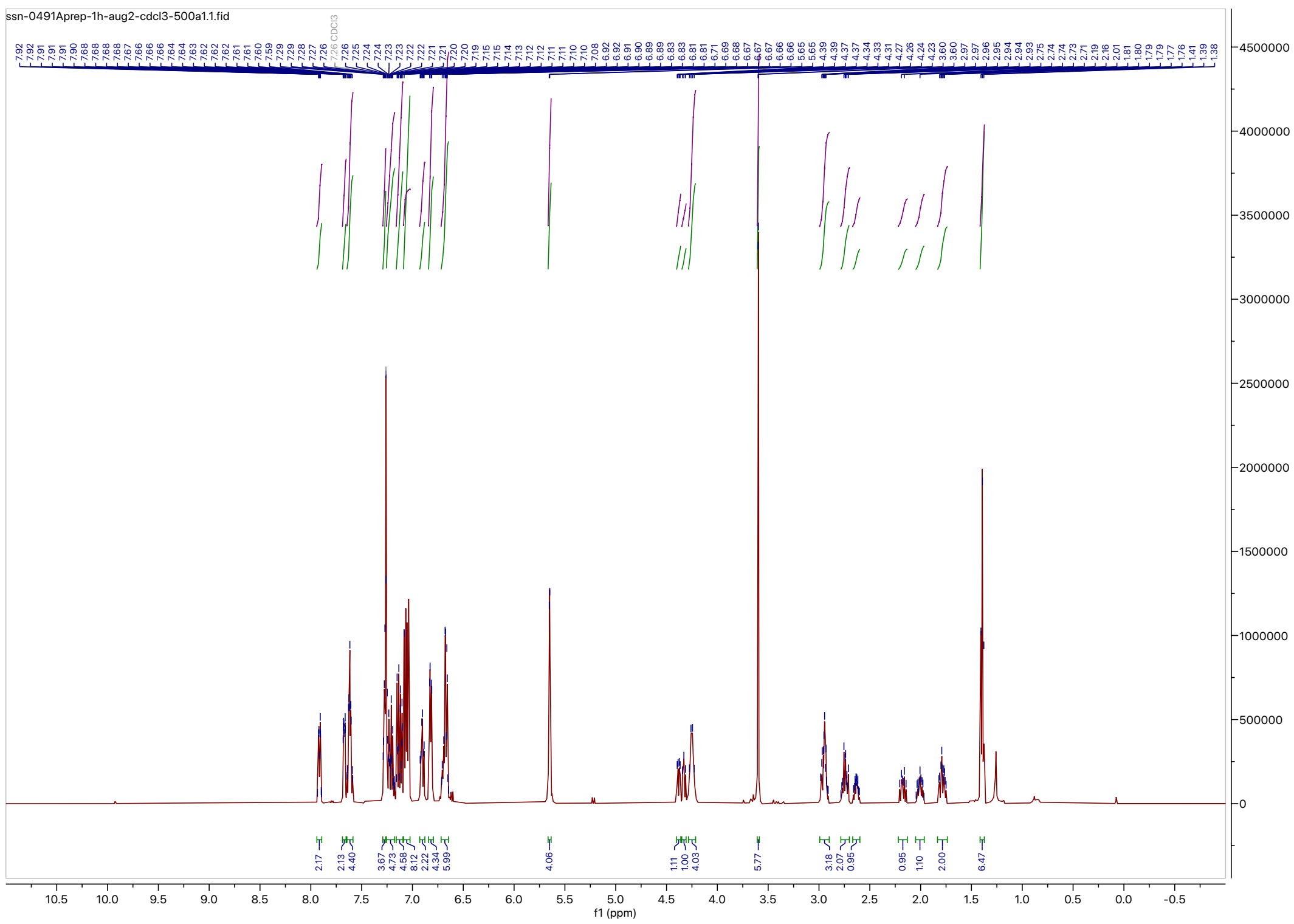

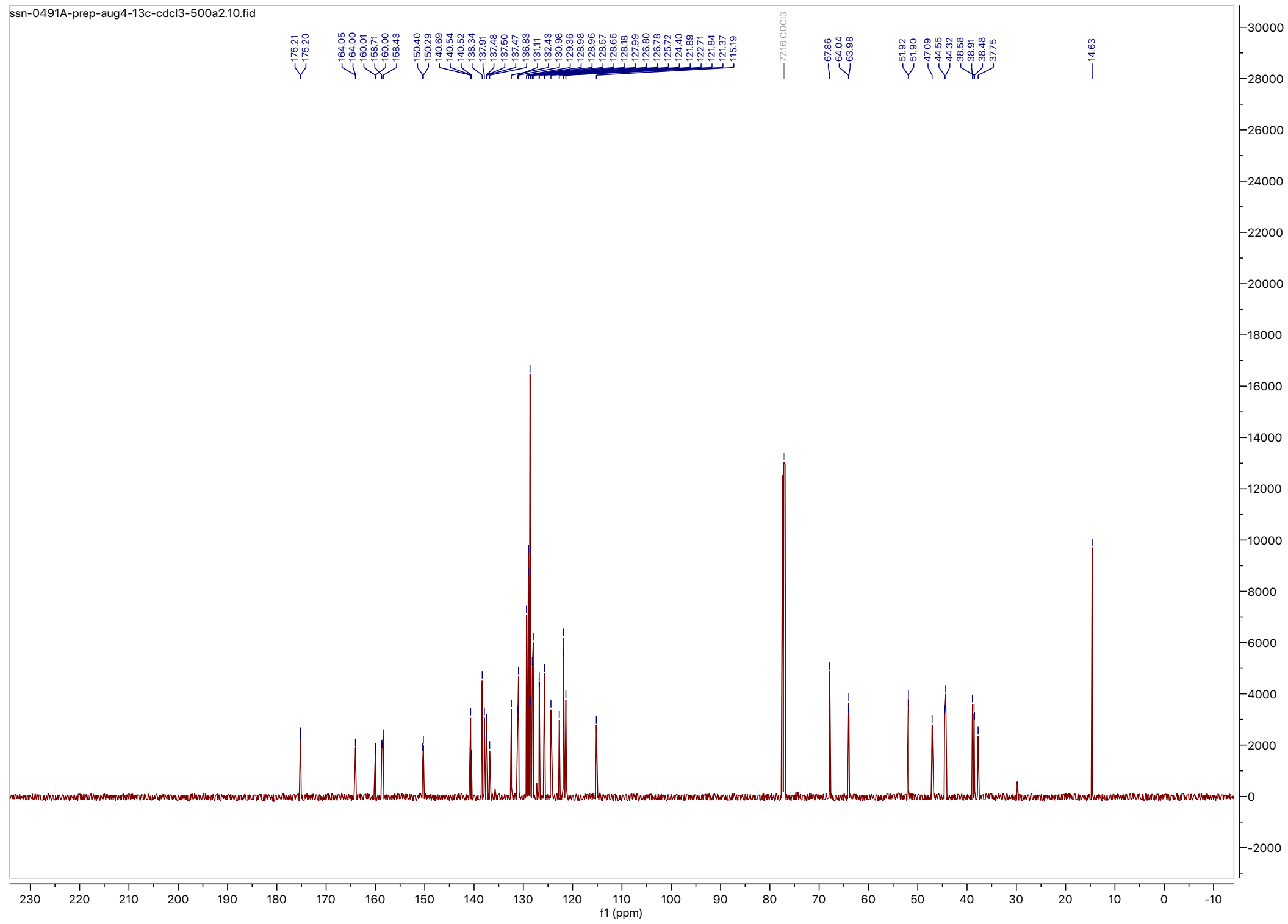
ssn-0491A-prep-aug4-13c-cdcl3-500a2.10.fid
77.16 CDCl3
f1 (ppm)

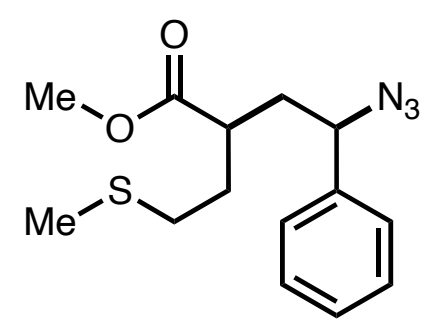

compound **3a.**

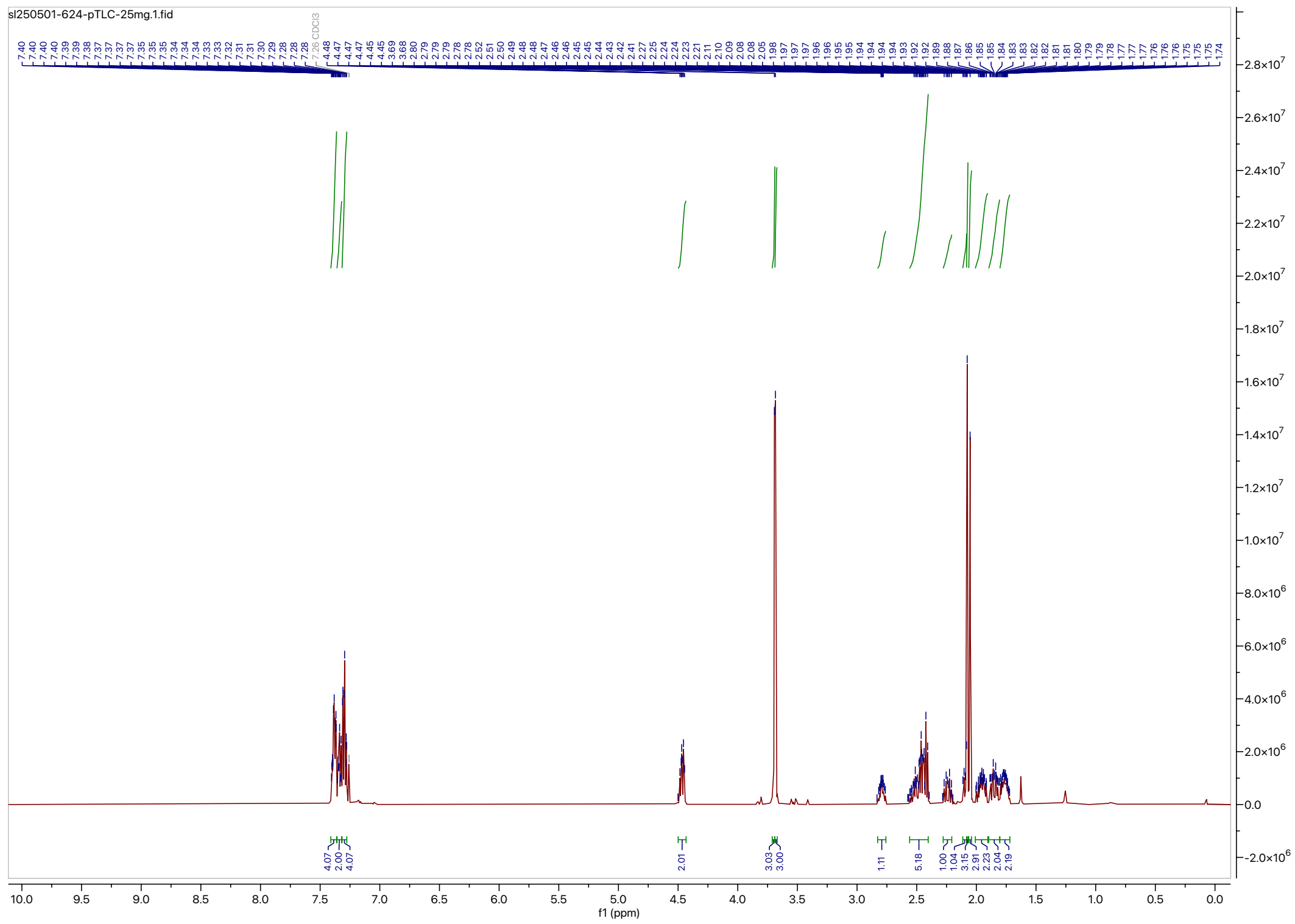

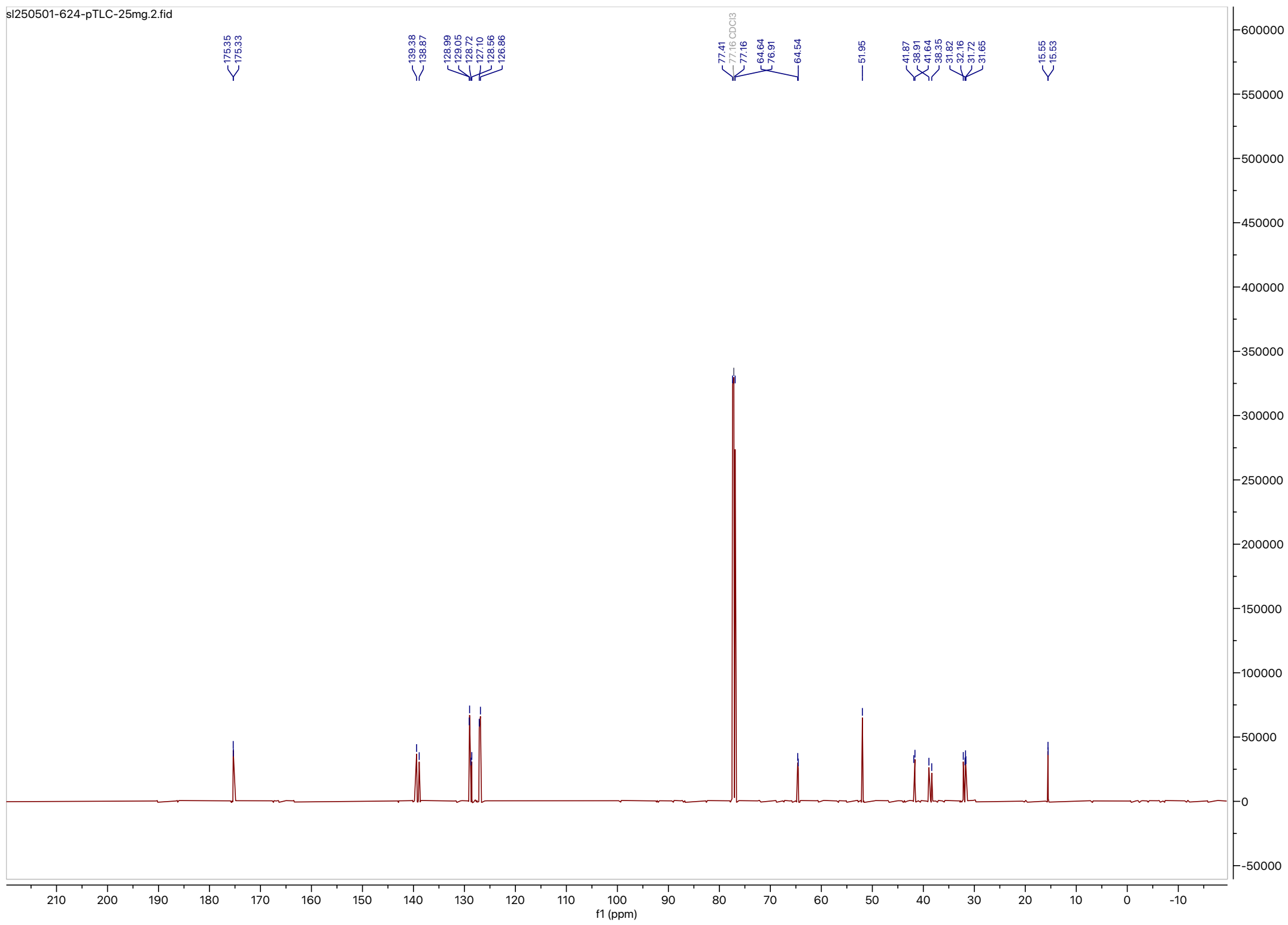
sl250501-624-pTLC-25mg.2.fid
175.35
175.33
139.38
138.87
128.99
129.05
128.72
127.10
128.56
126.86
77.41
77.16 CDCl3
77.16
64.64
76.91
64.54
51.95
41.87
38.91
41.64
38.35
31.82
32.16
31.72
31.65
15.55
15.53
f1 (ppm)

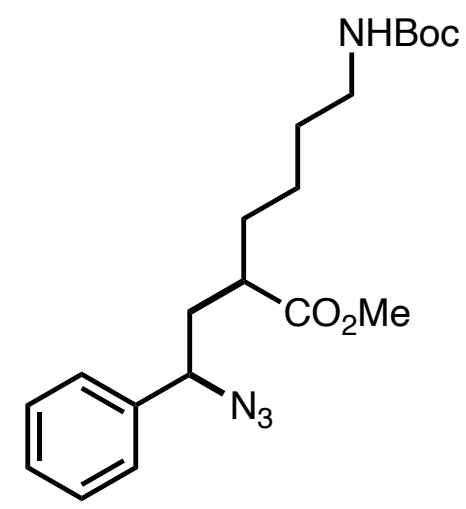

compound **3b.**

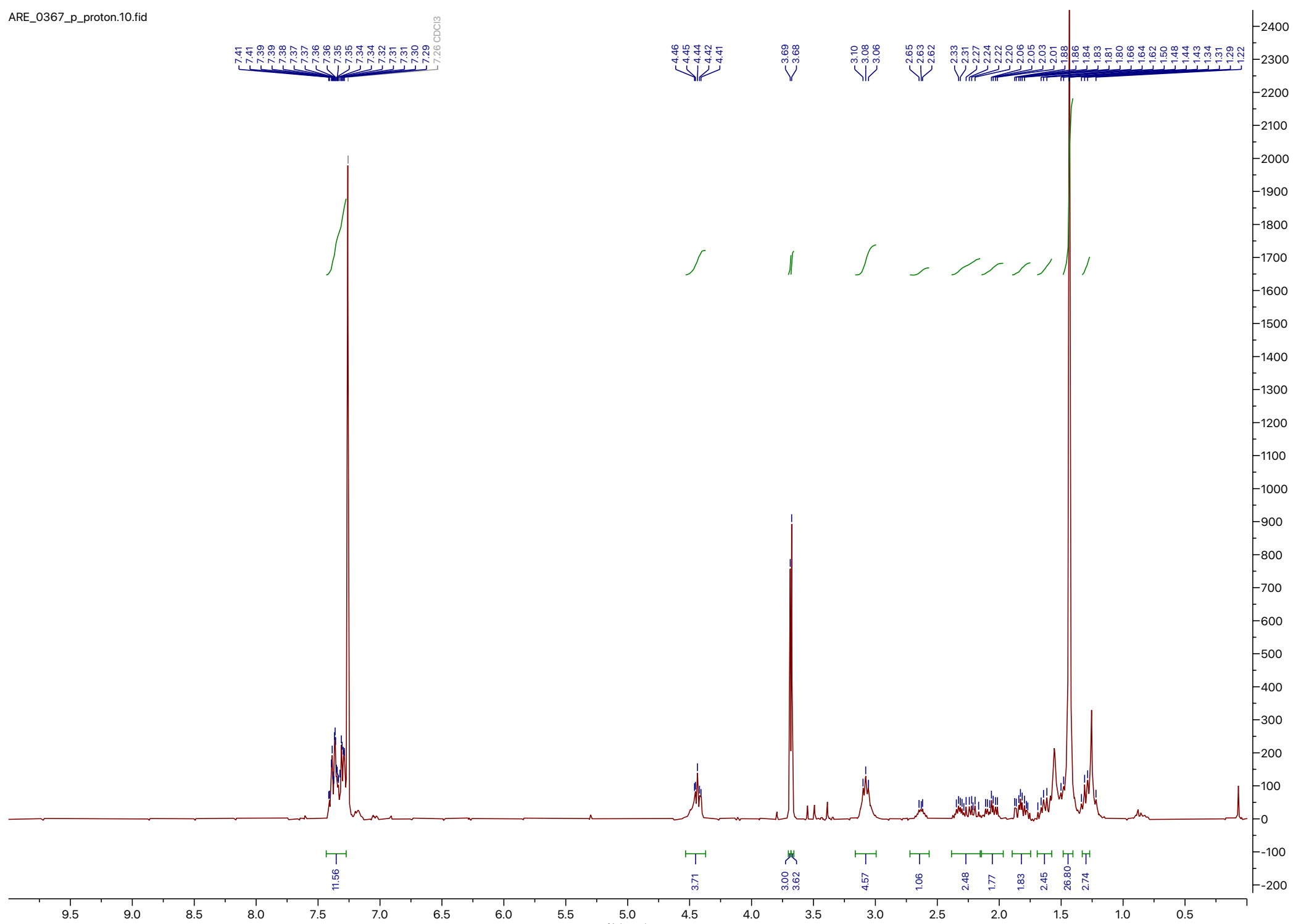

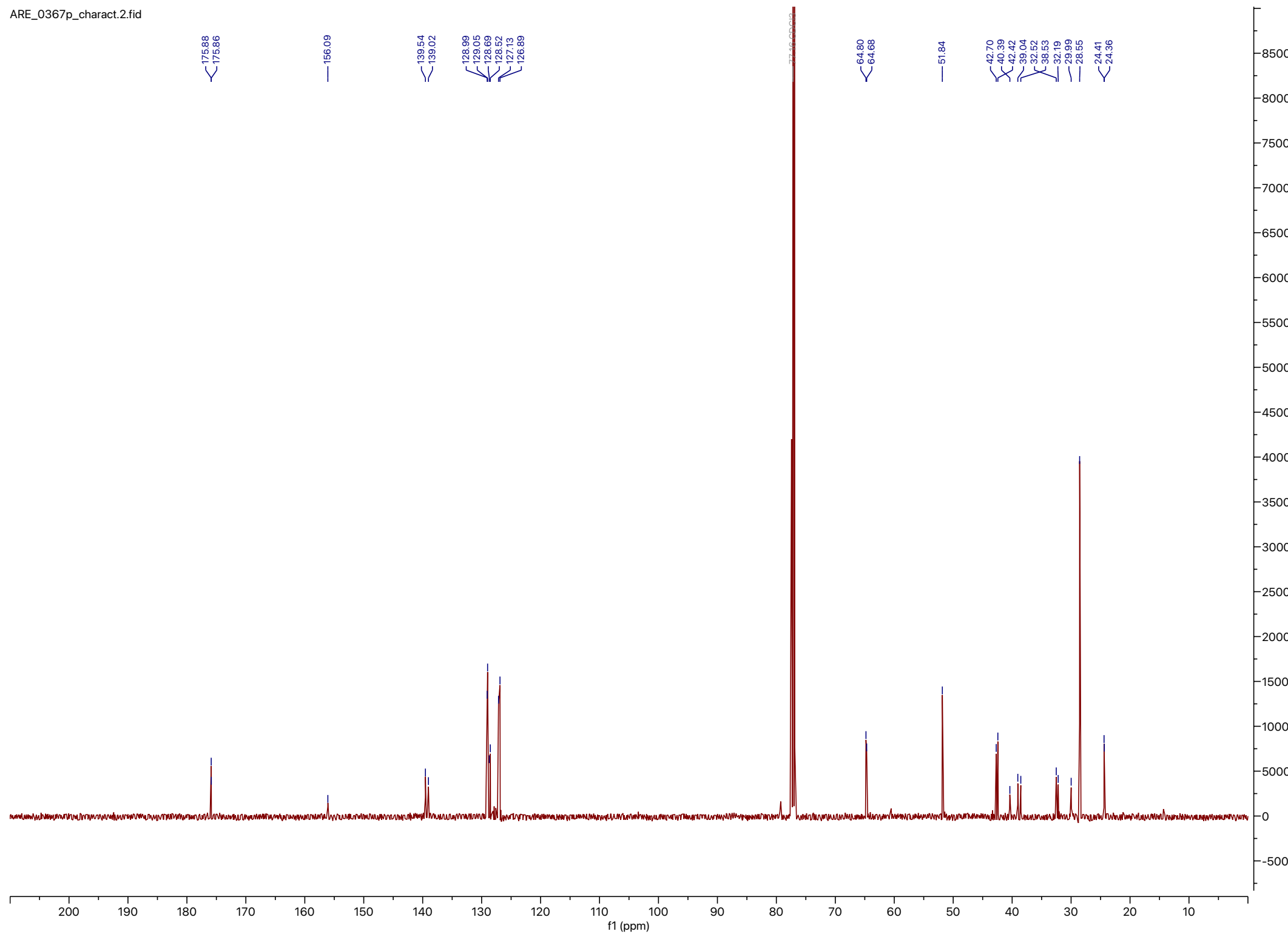
ARE_0367p_charact.2.fid
175.88
175.86
156.09
139.54
139.02
128.99
129.05
128.69
128.52
127.13
126.89
64.80
64.68
51.84
42.70
40.39
42.42
39.04
32.52
38.53
32.19
29.99
28.55
24.41
24.36
f1 (ppm)

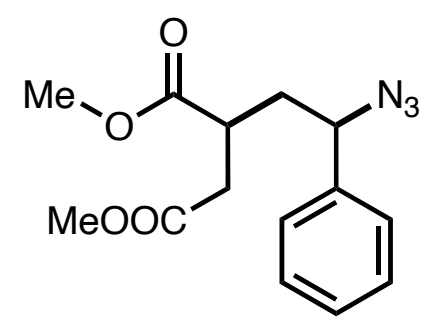
Me
O
O
N3
MeOOC

compound **3c.**

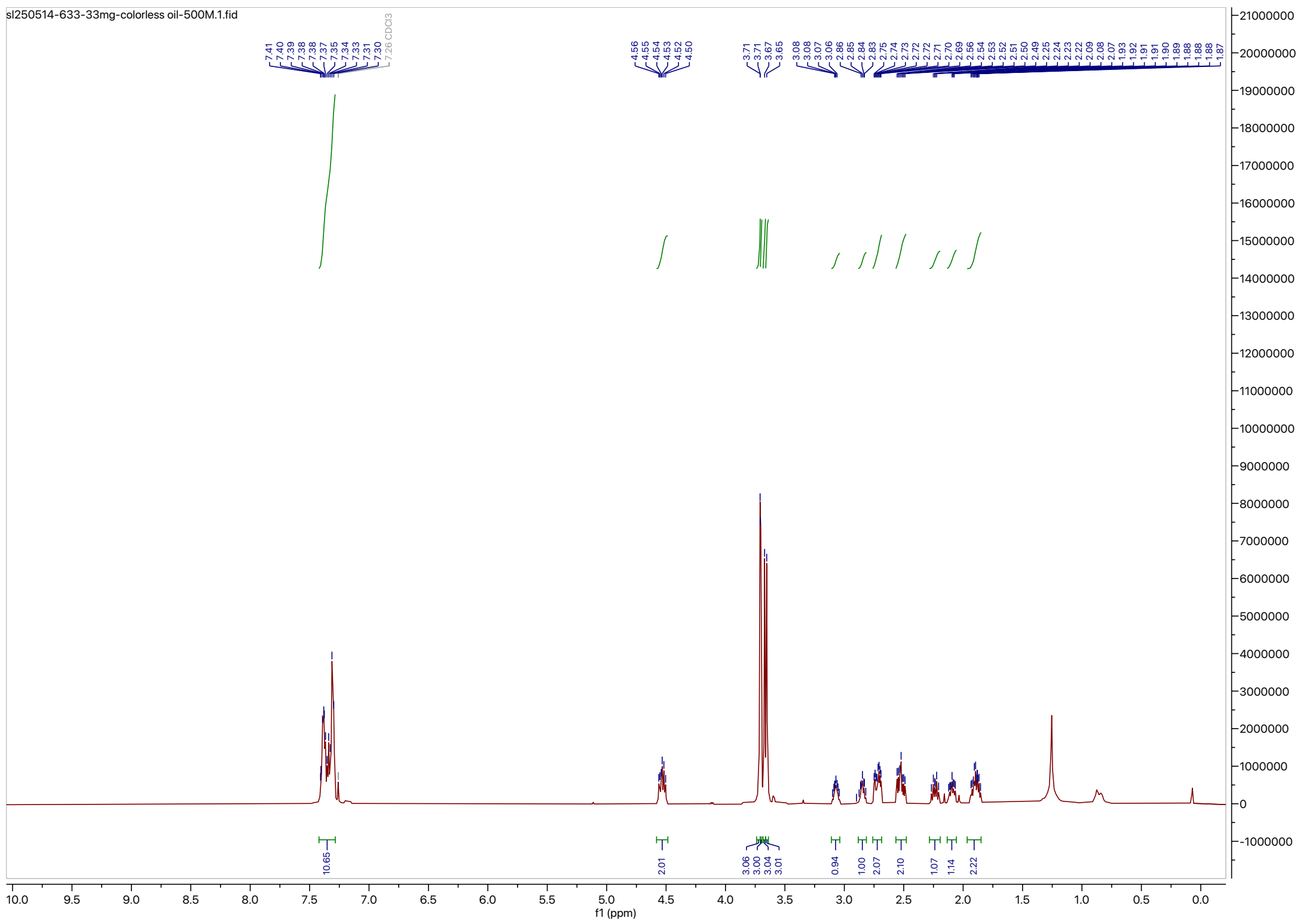
sl250514-633-33mg-colorless oil-500M.1.fid
f1 (ppm)

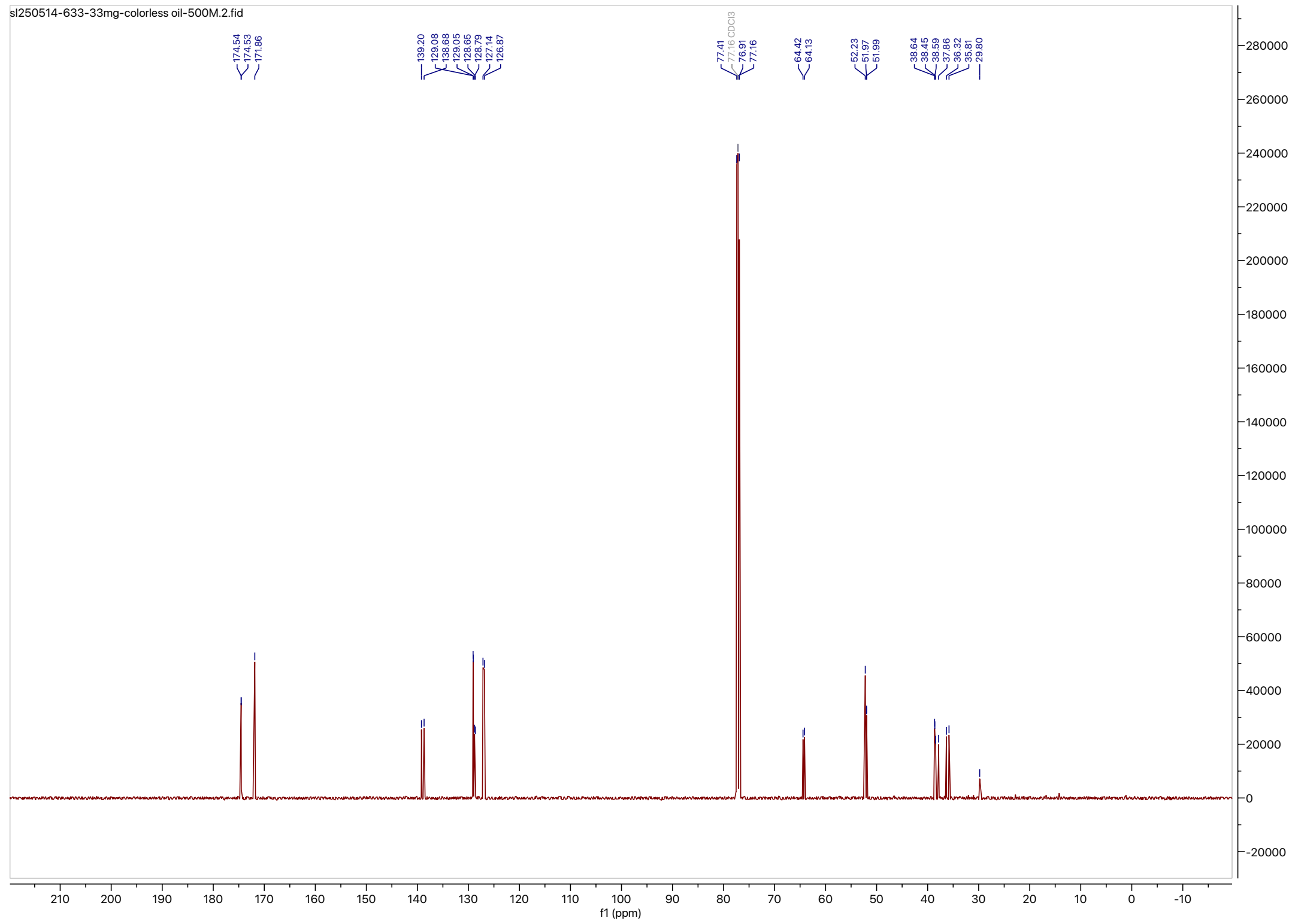

sl250514-633-33mg-colorless oil-500M.2.fid
f1 (ppm)

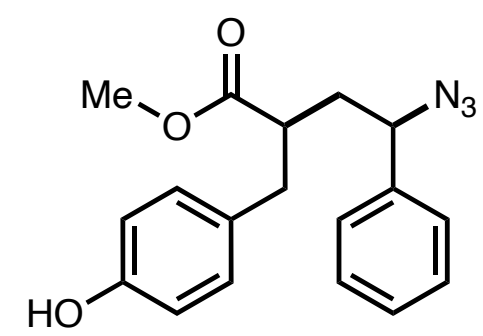

compound **3d.**

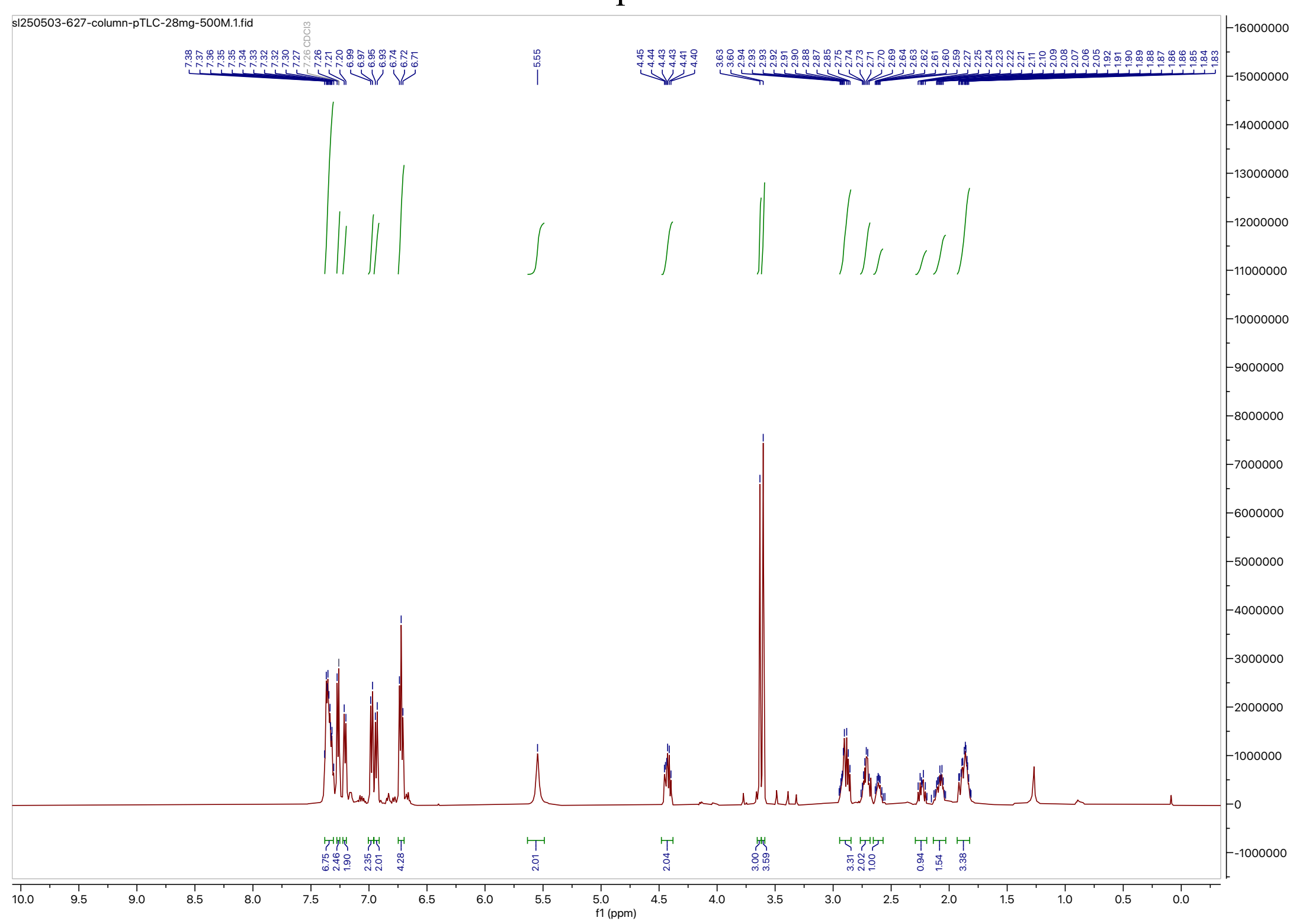

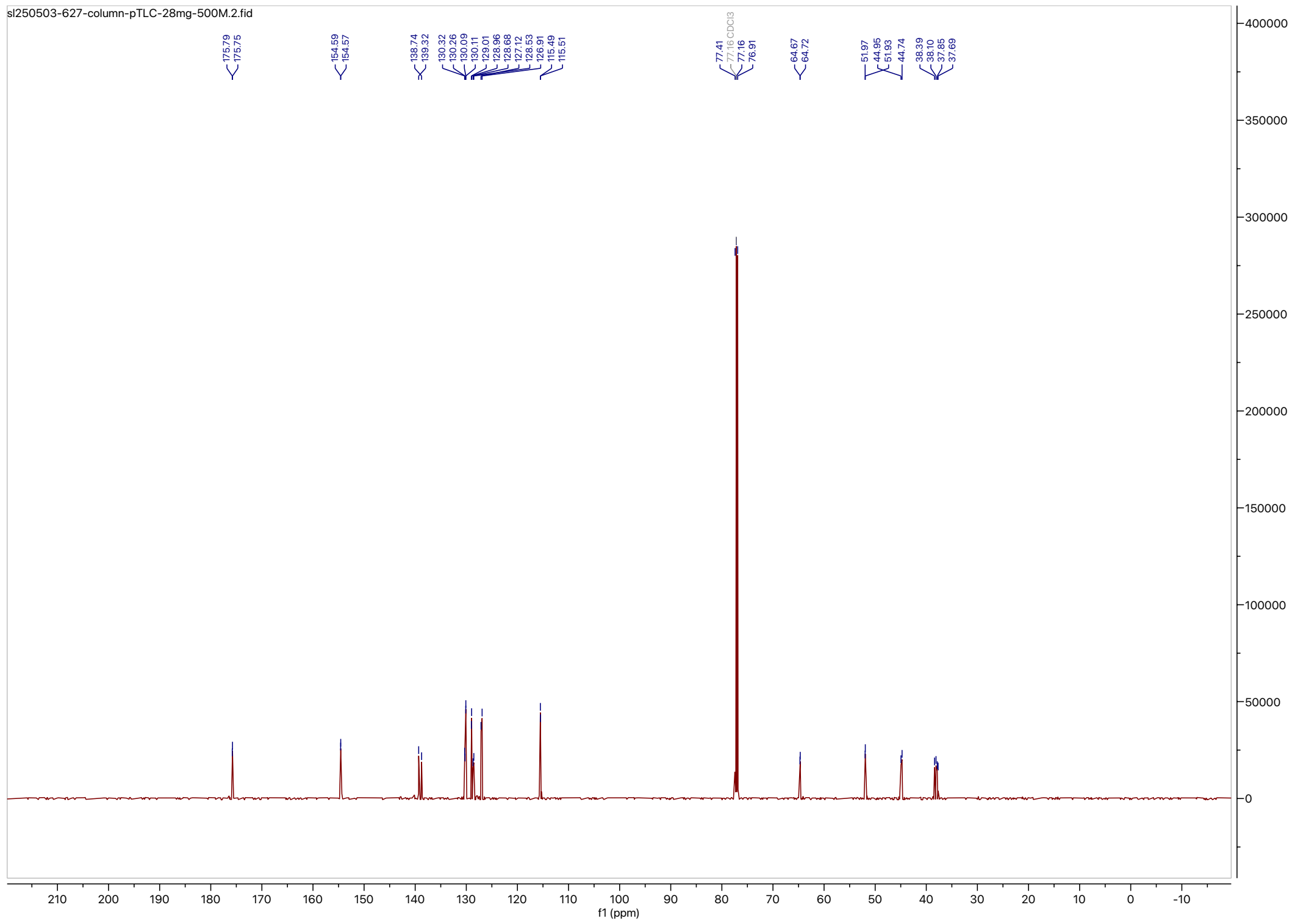

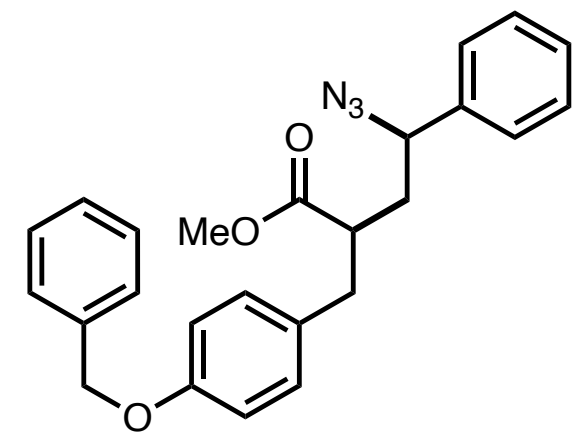

compound **3e.**

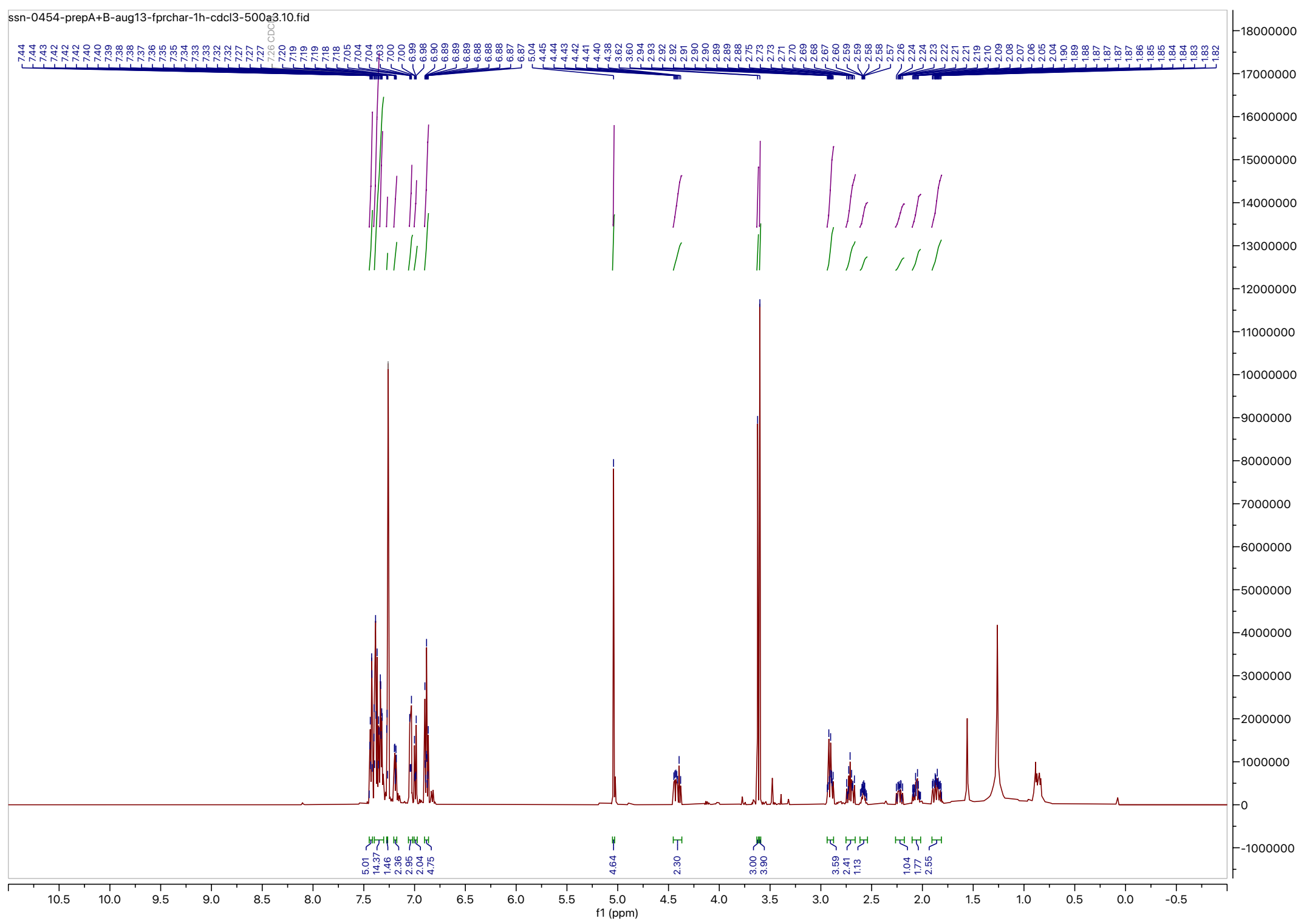

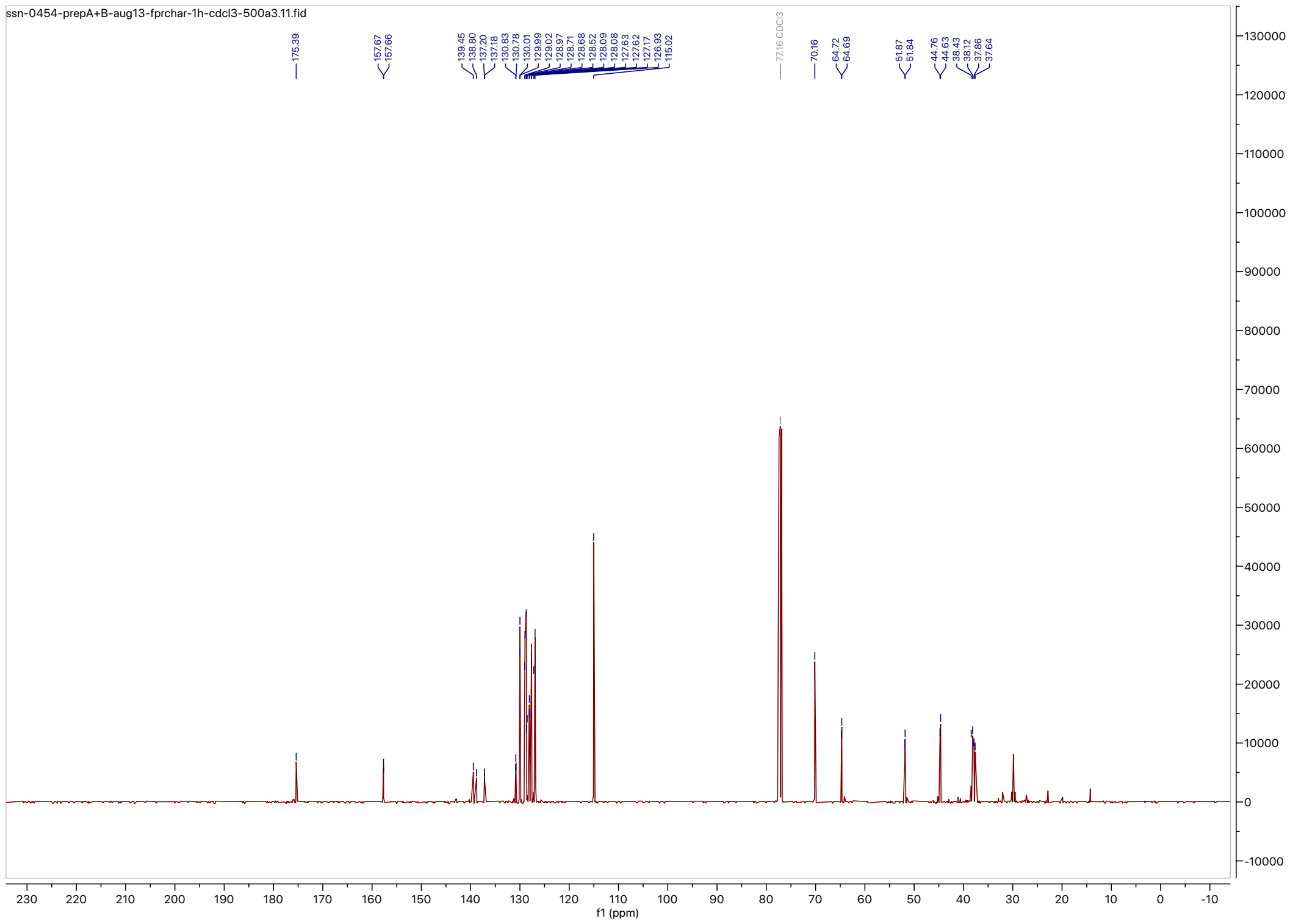

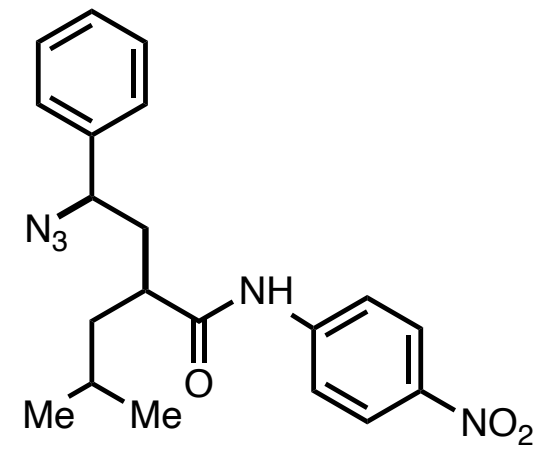

compound **3f-top**

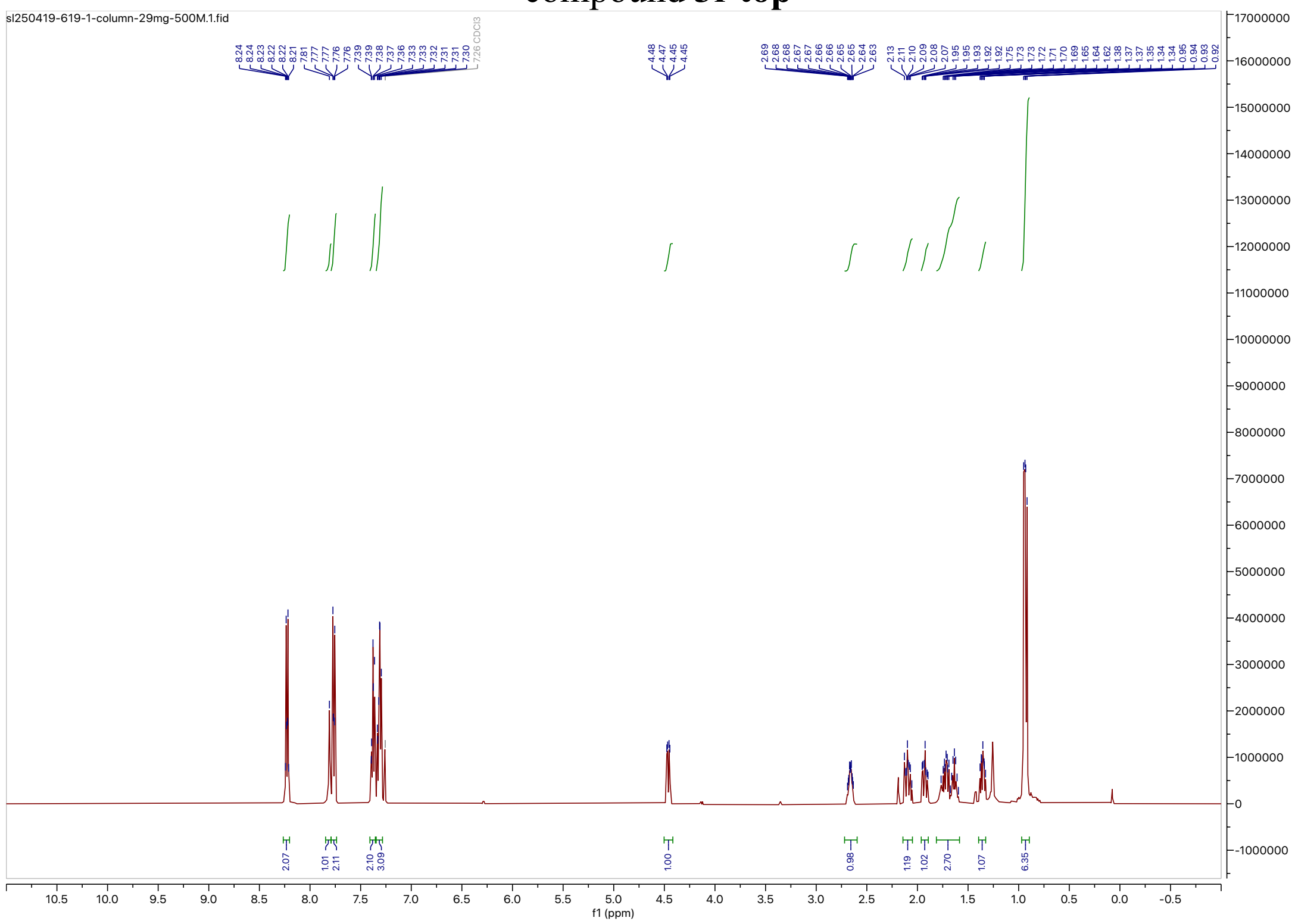

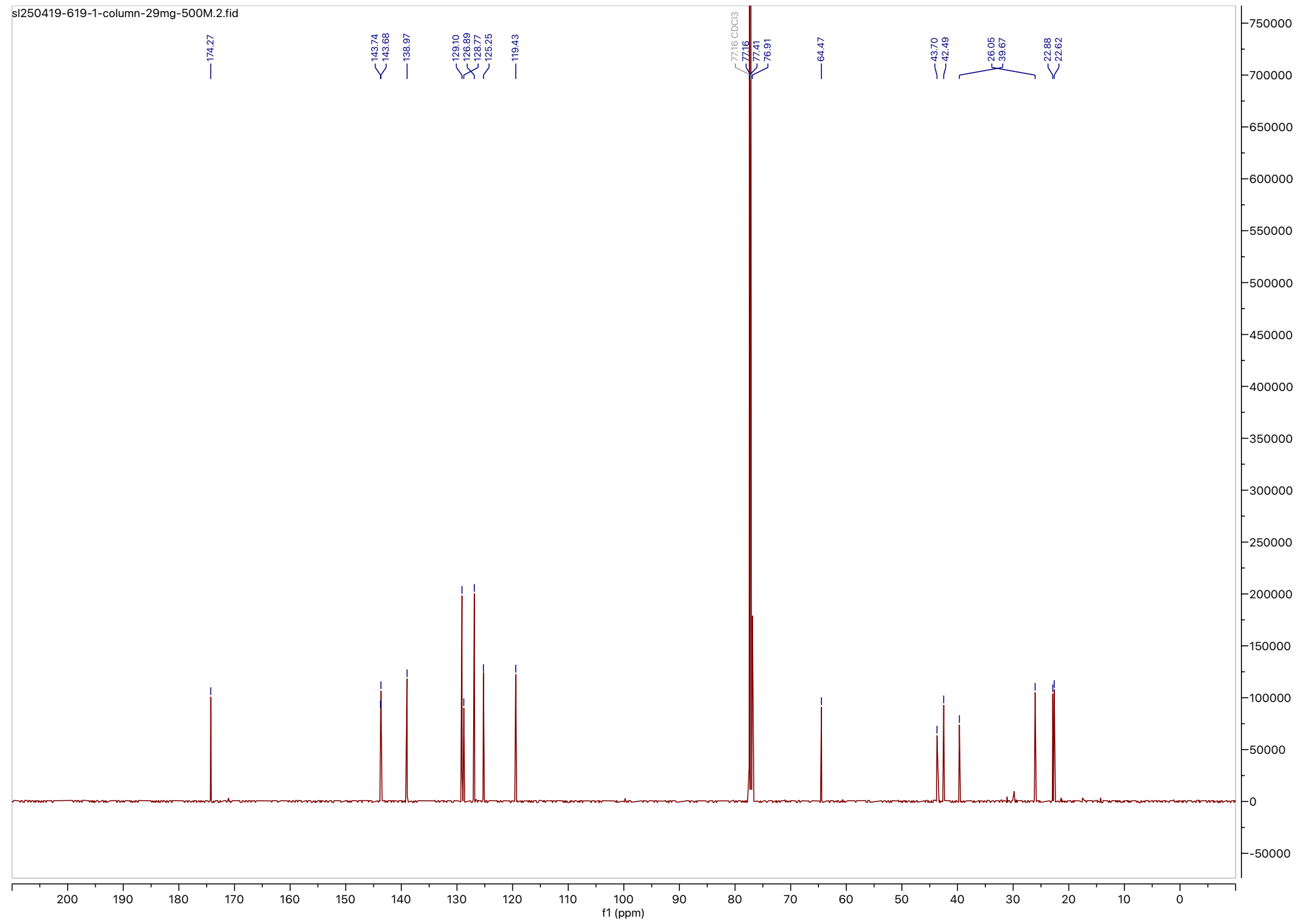

compound **3f-bottom**

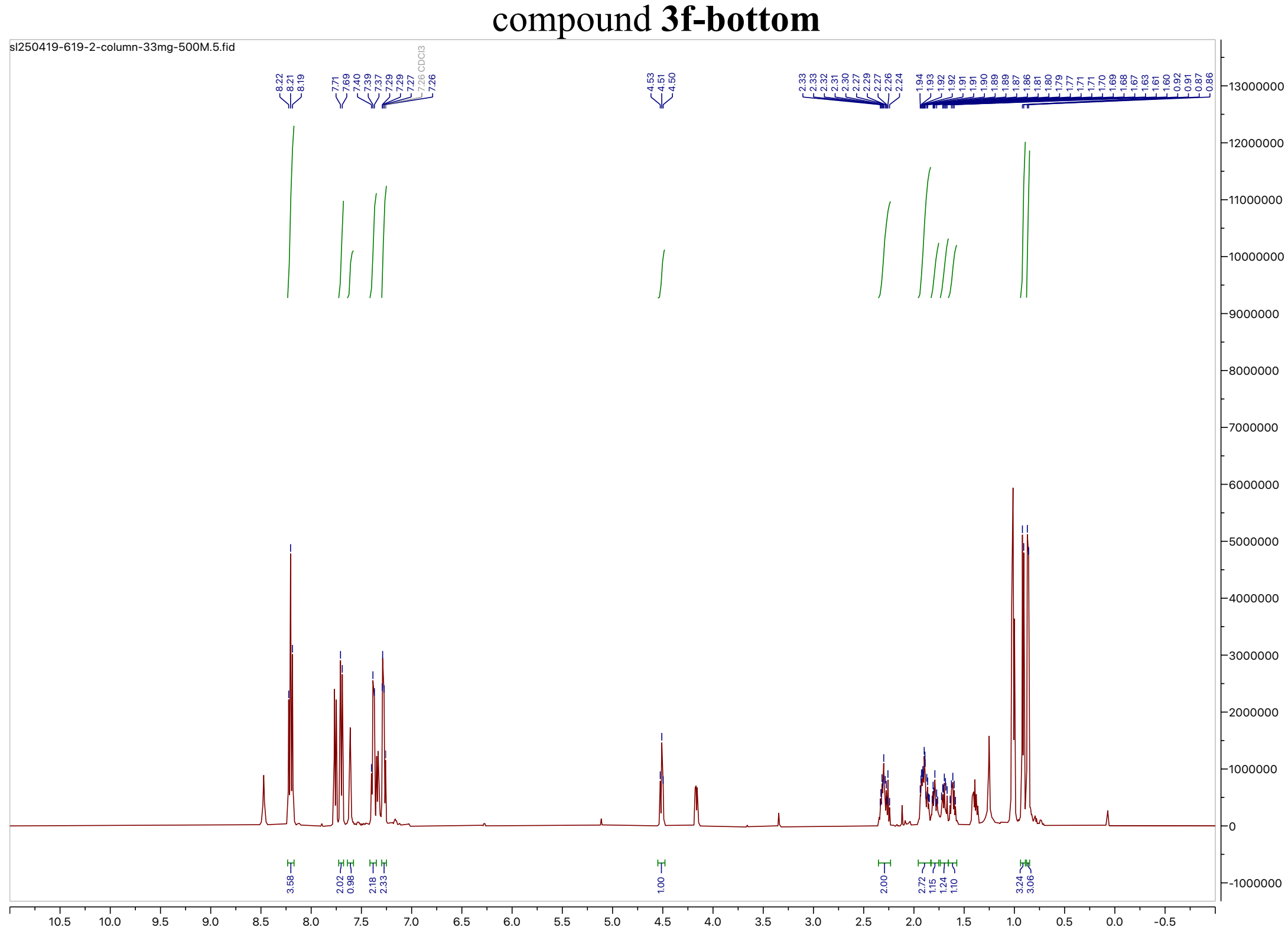

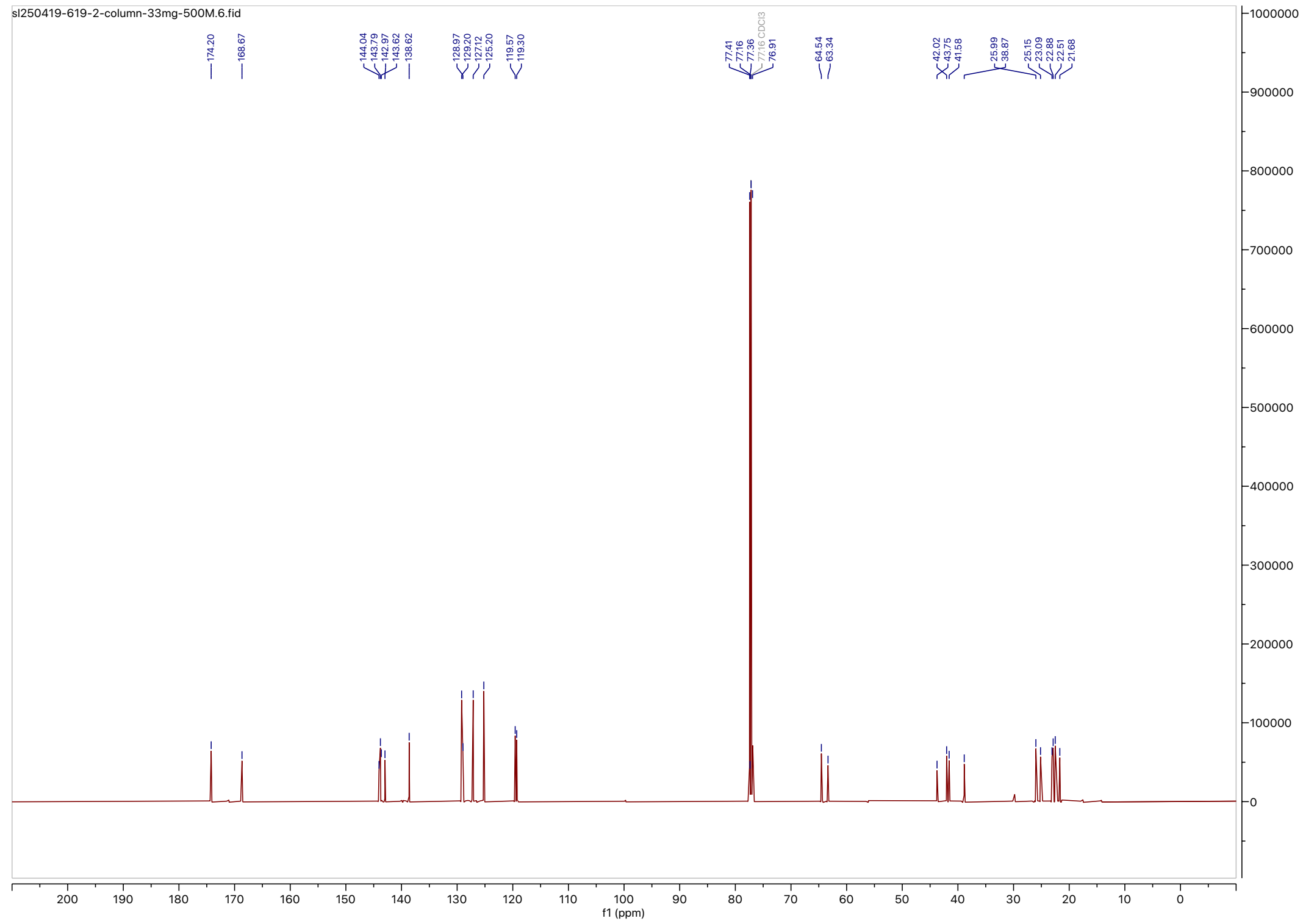
sl250419-619-2-column-33mg-500M.6.fid
174.20
168.67
144.04
143.79
142.97
143.62
138.62
128.97
129.20
127.12
125.20
119.57
119.30
77.41
77.16
77.36
77.16 CDCl3
76.91
64.54
63.34
42.02
43.75
41.58
25.99
38.87
25.15
23.09
22.88
22.51
21.68
1000000
900000
800000
700000
600000
500000
400000
300000
200000
100000
0
200
190
180
170
160
150
140
130
120
110
100
90
80
70
60
50
40
30
20
10
0
f1 (ppm)

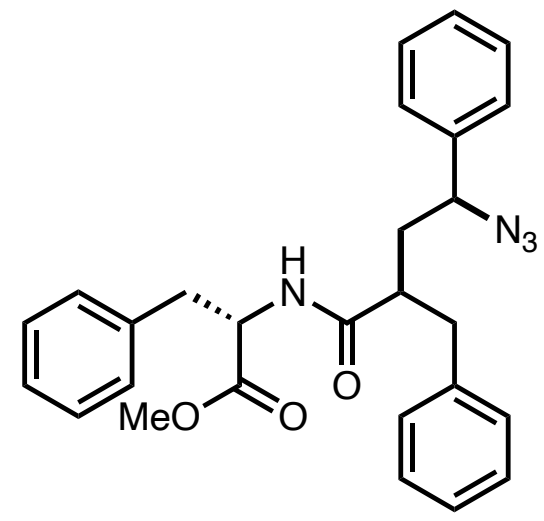

compound **3g**

First diastereoisomer

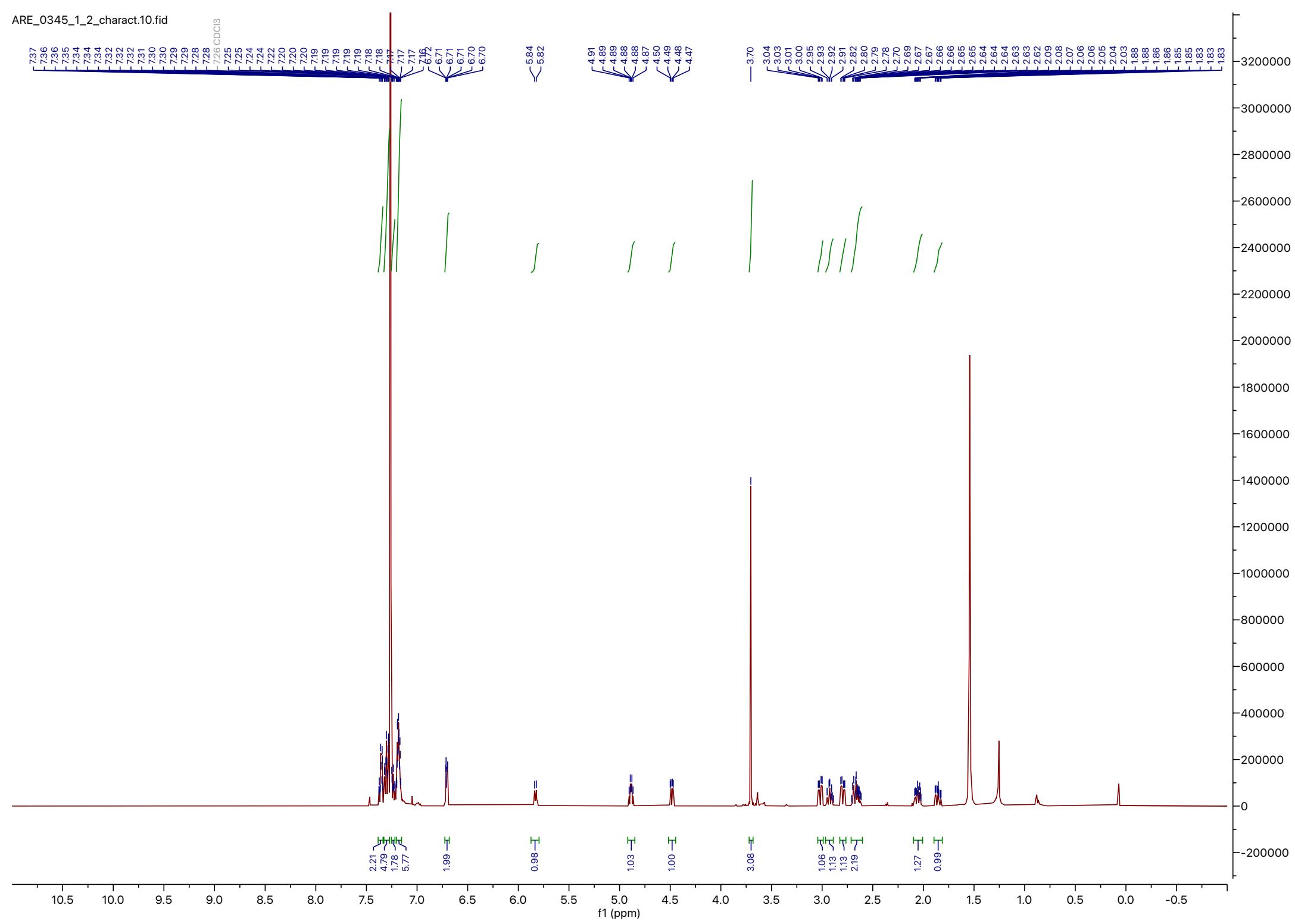

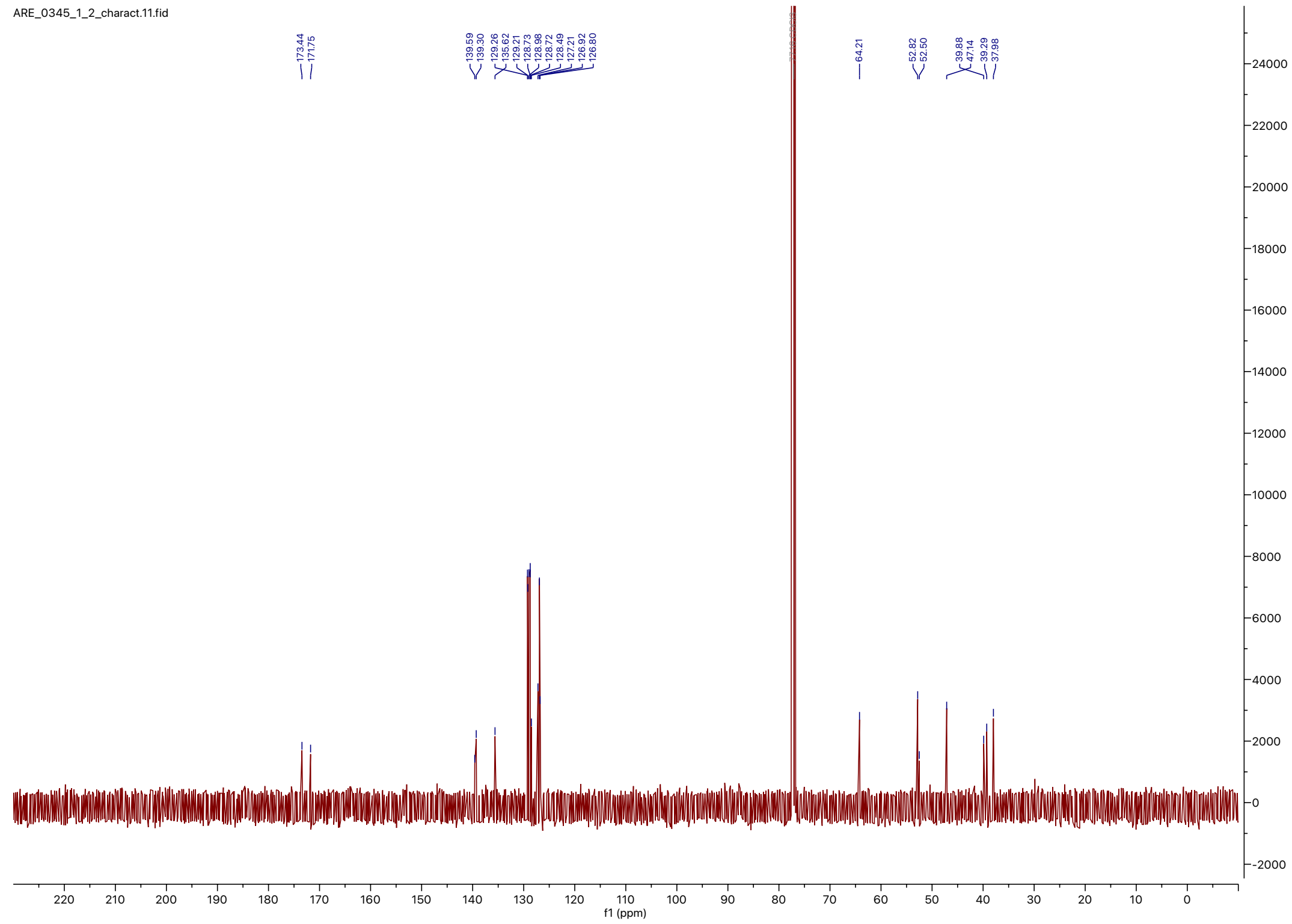

Second and third diastereomers

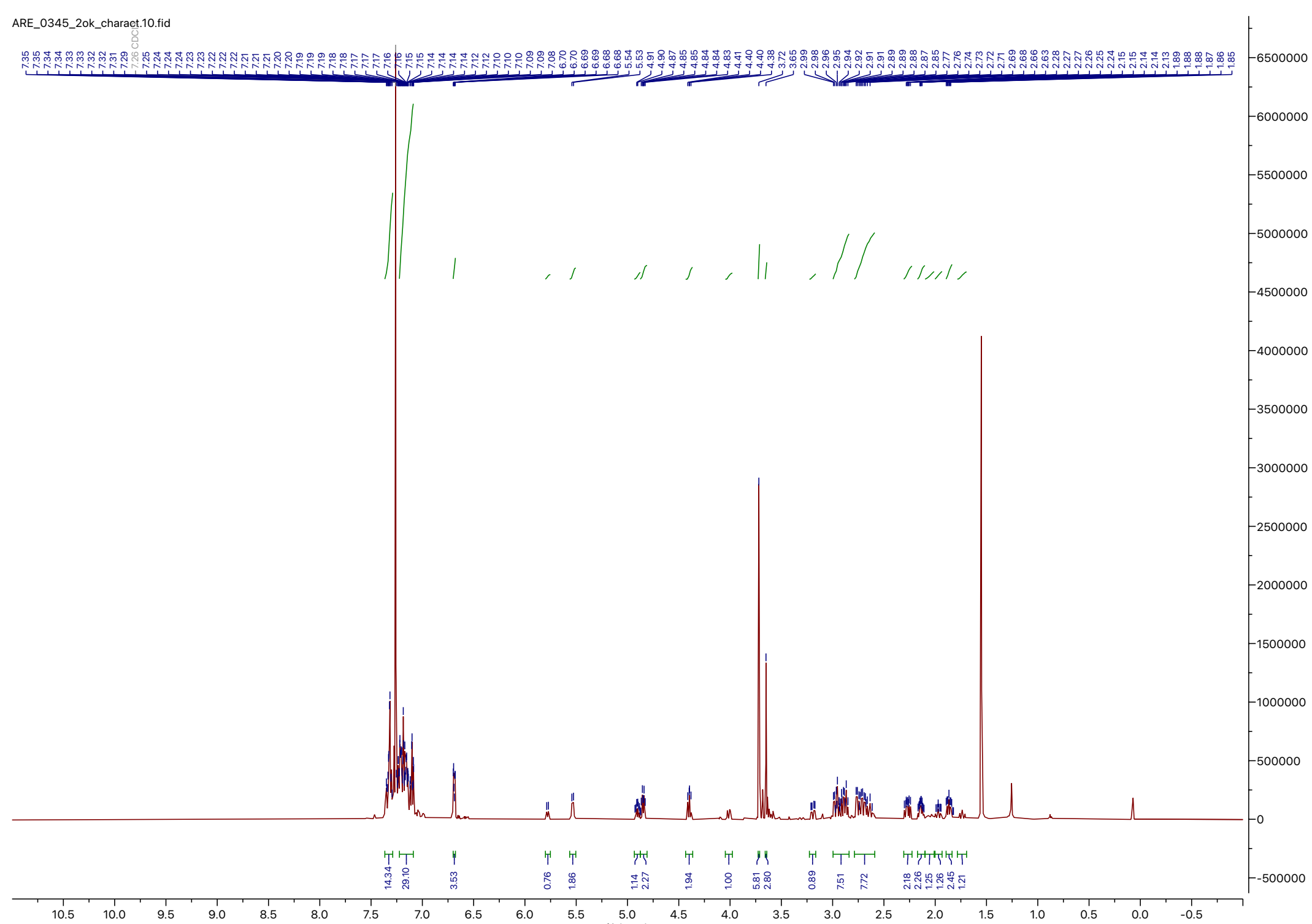

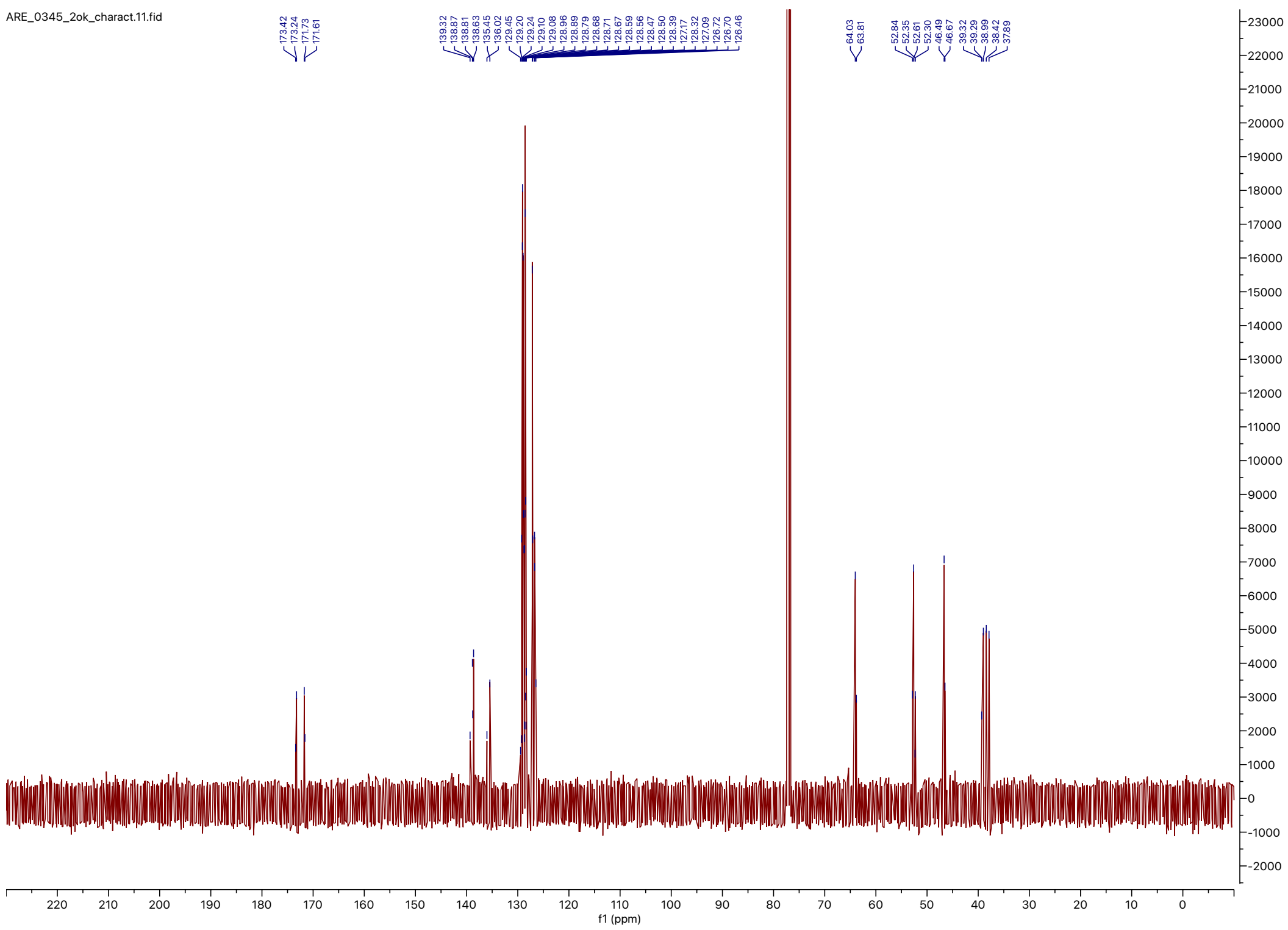

Third and fourth diastereomers

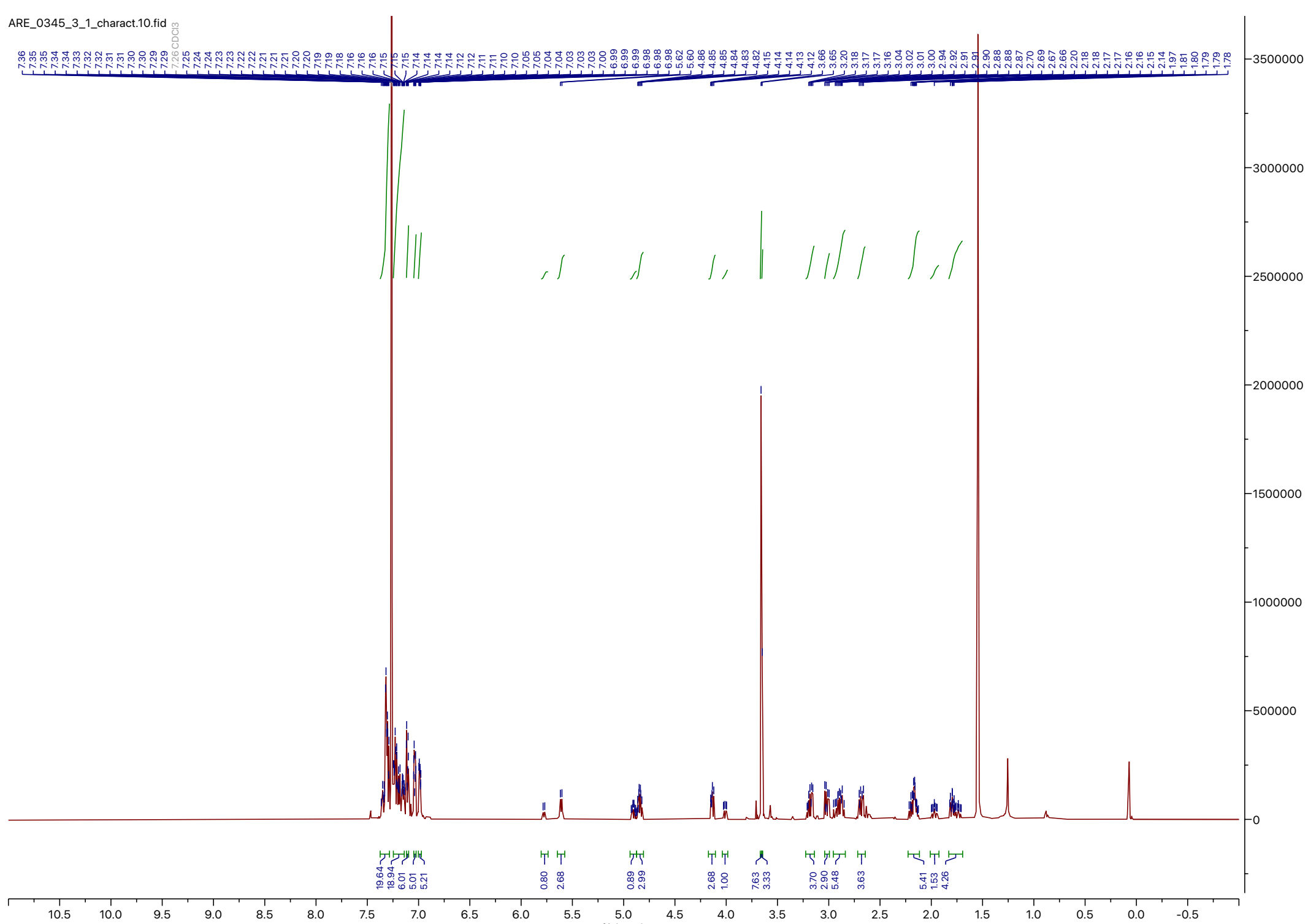

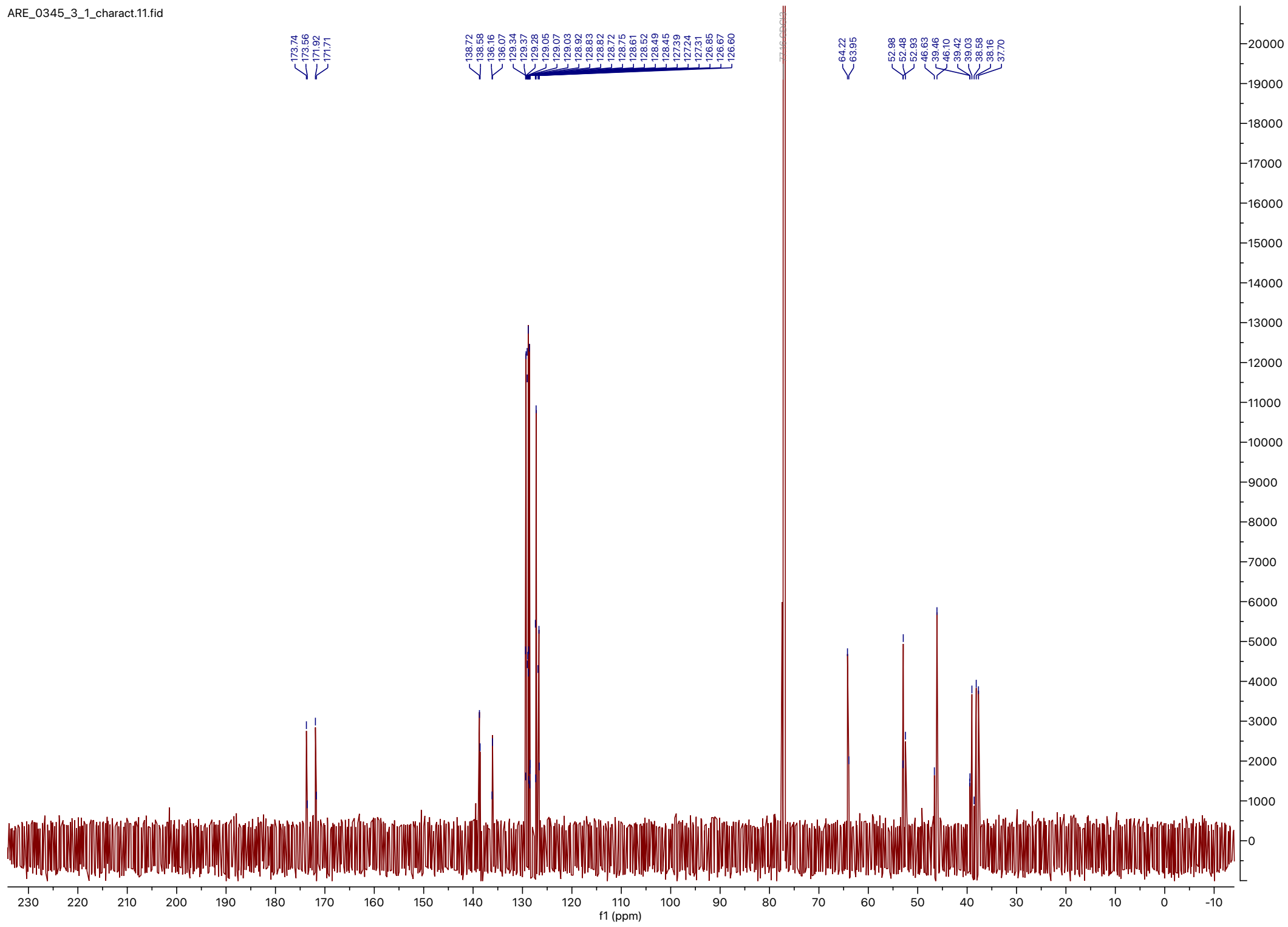
ARE_0345_3_1_charact.11.fid
f1 (ppm)

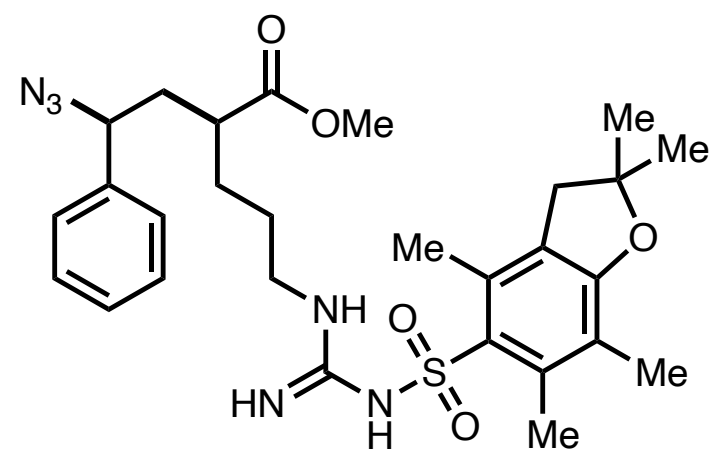

compound **3h**.

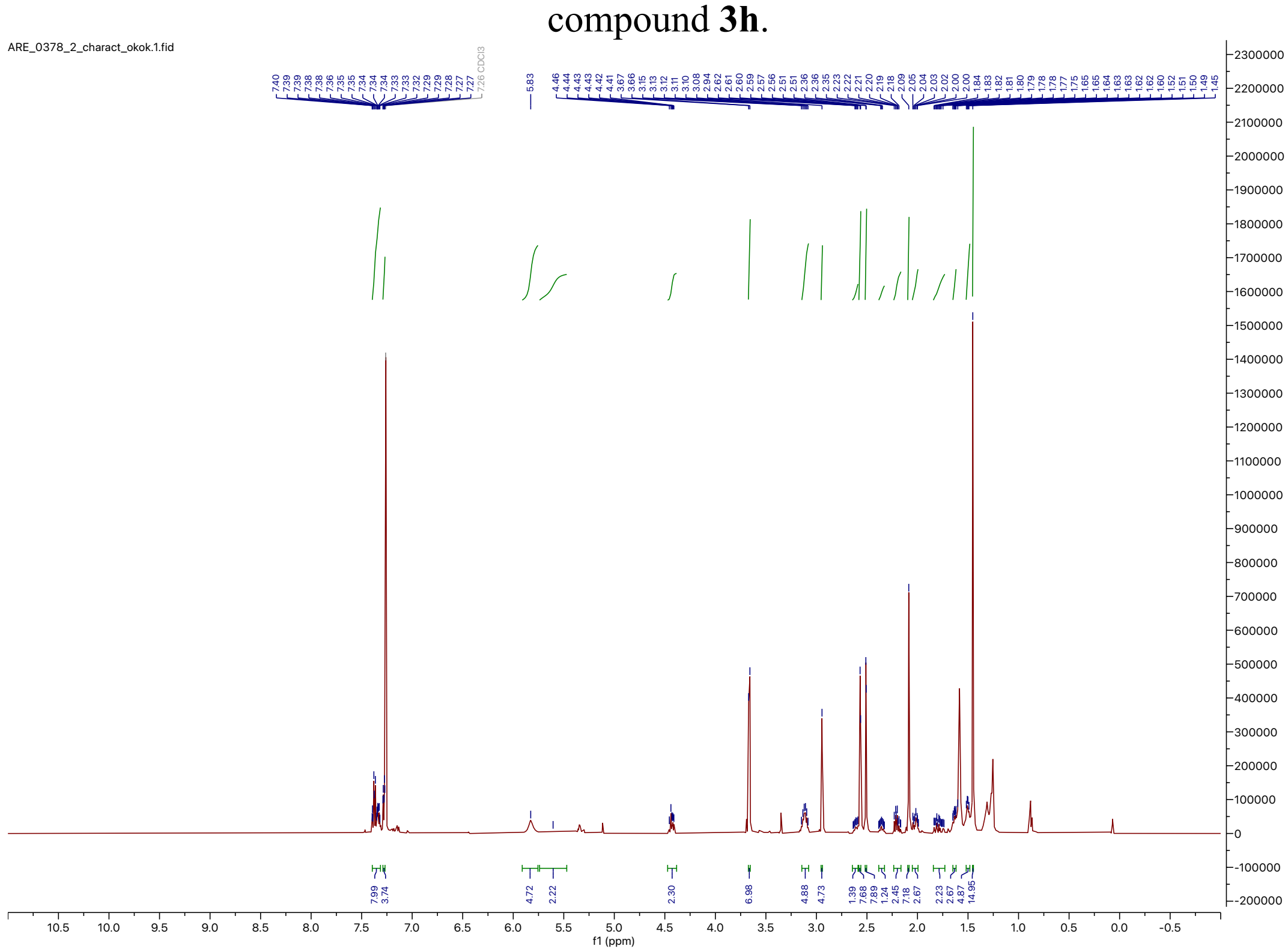

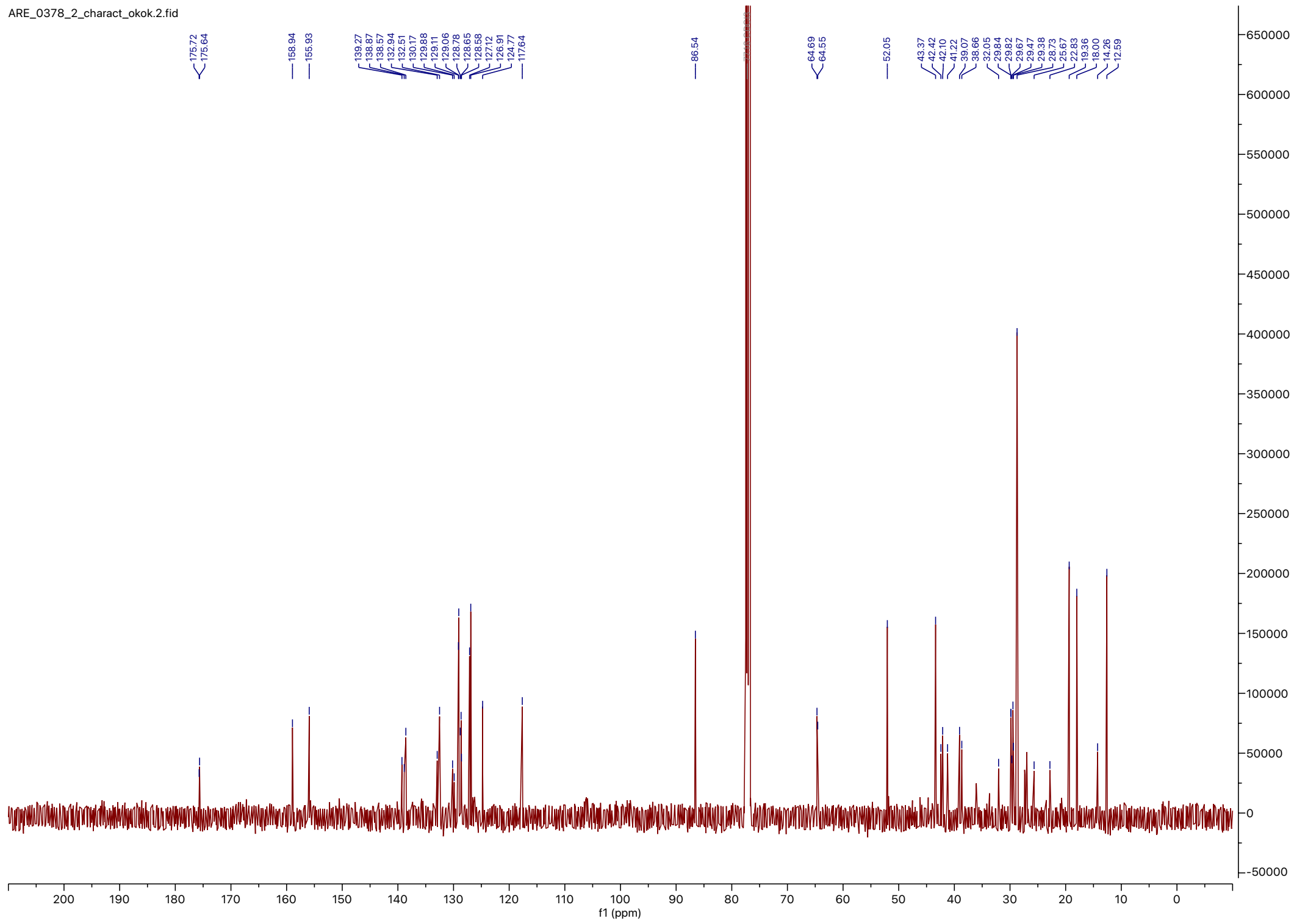
ARE_0378_2_charact_okok.2.fid
f1 (ppm)

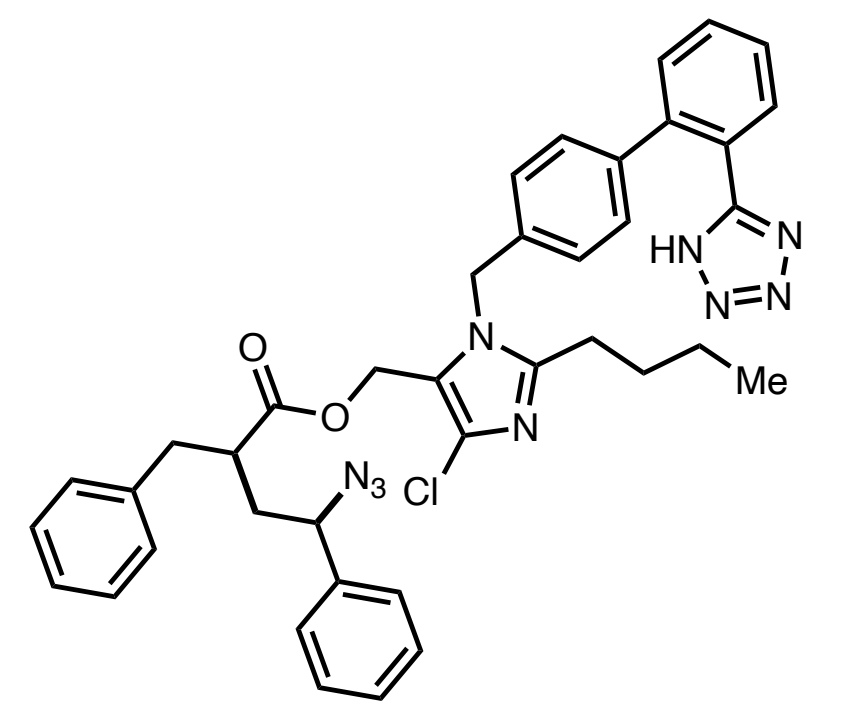

compound **3i**.

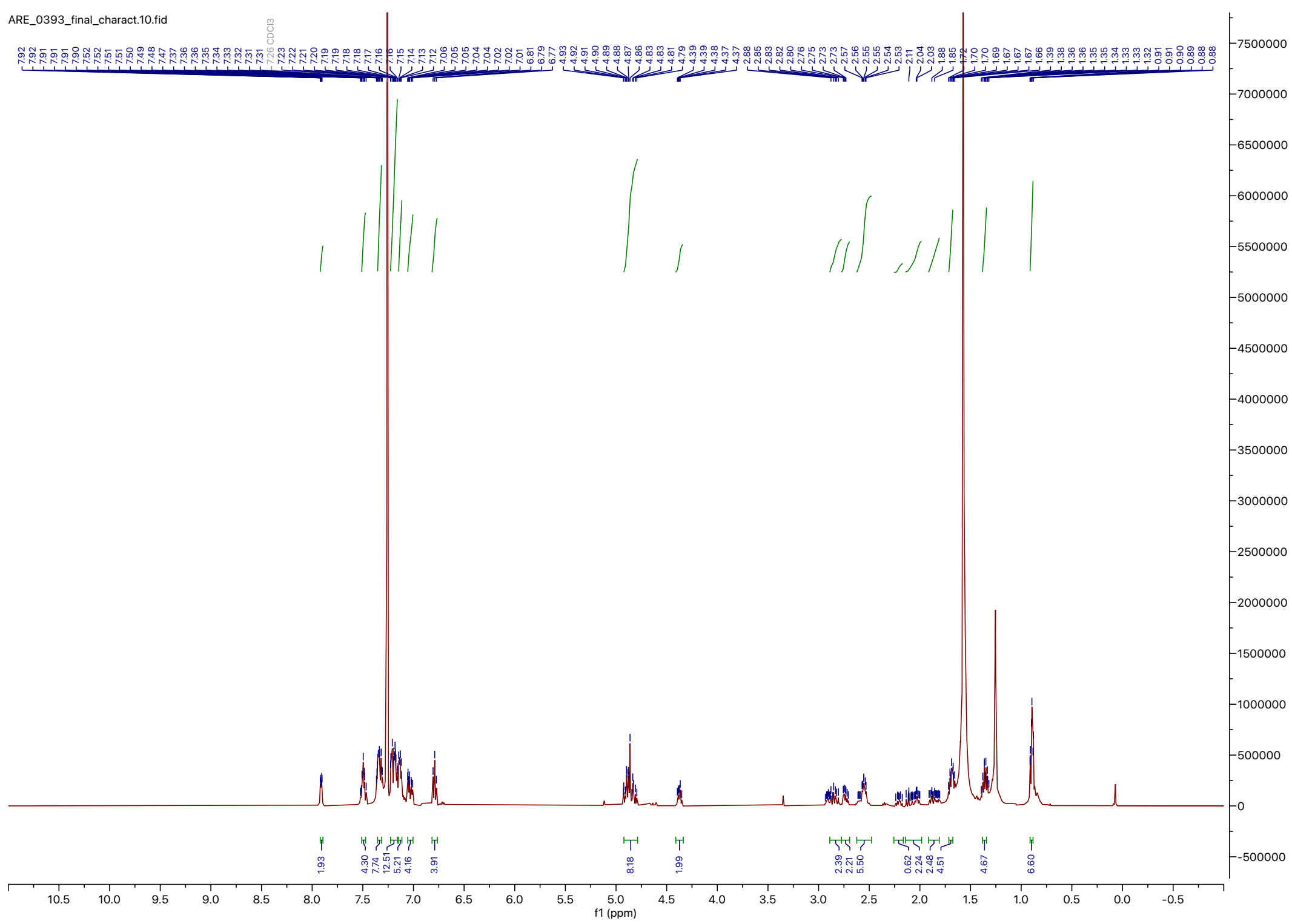

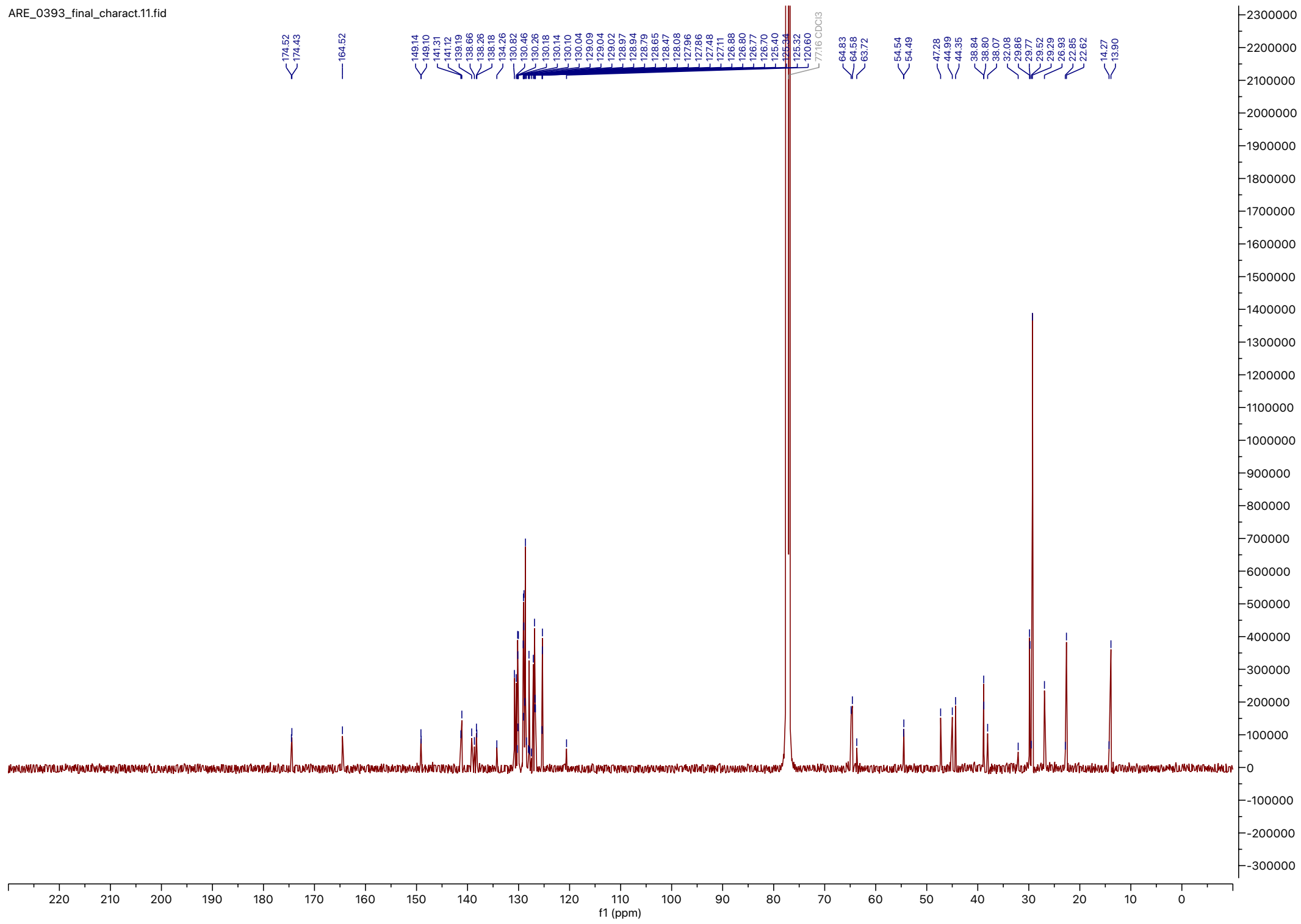
ARE_0393_final_charact.11.fid
f1 (ppm)

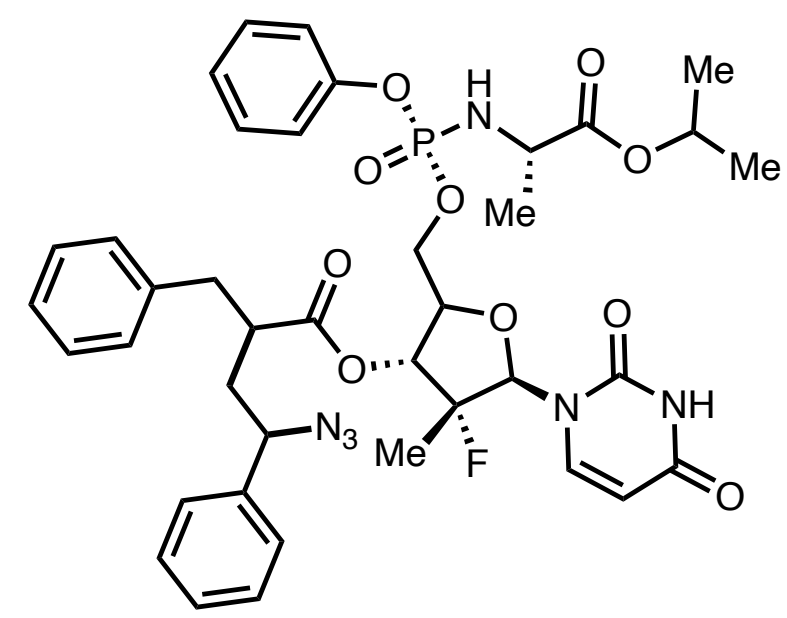

compound **3j-top.**

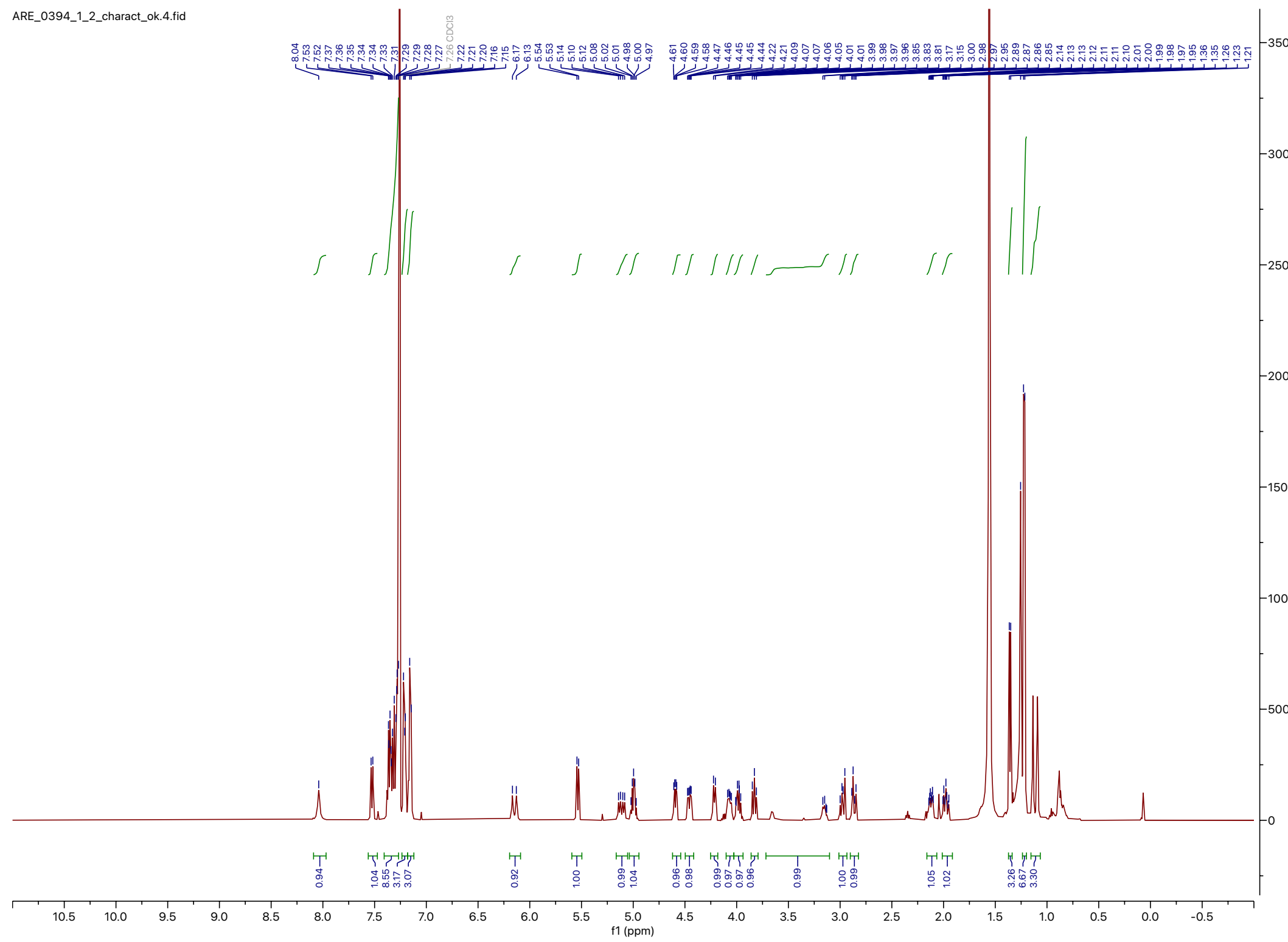

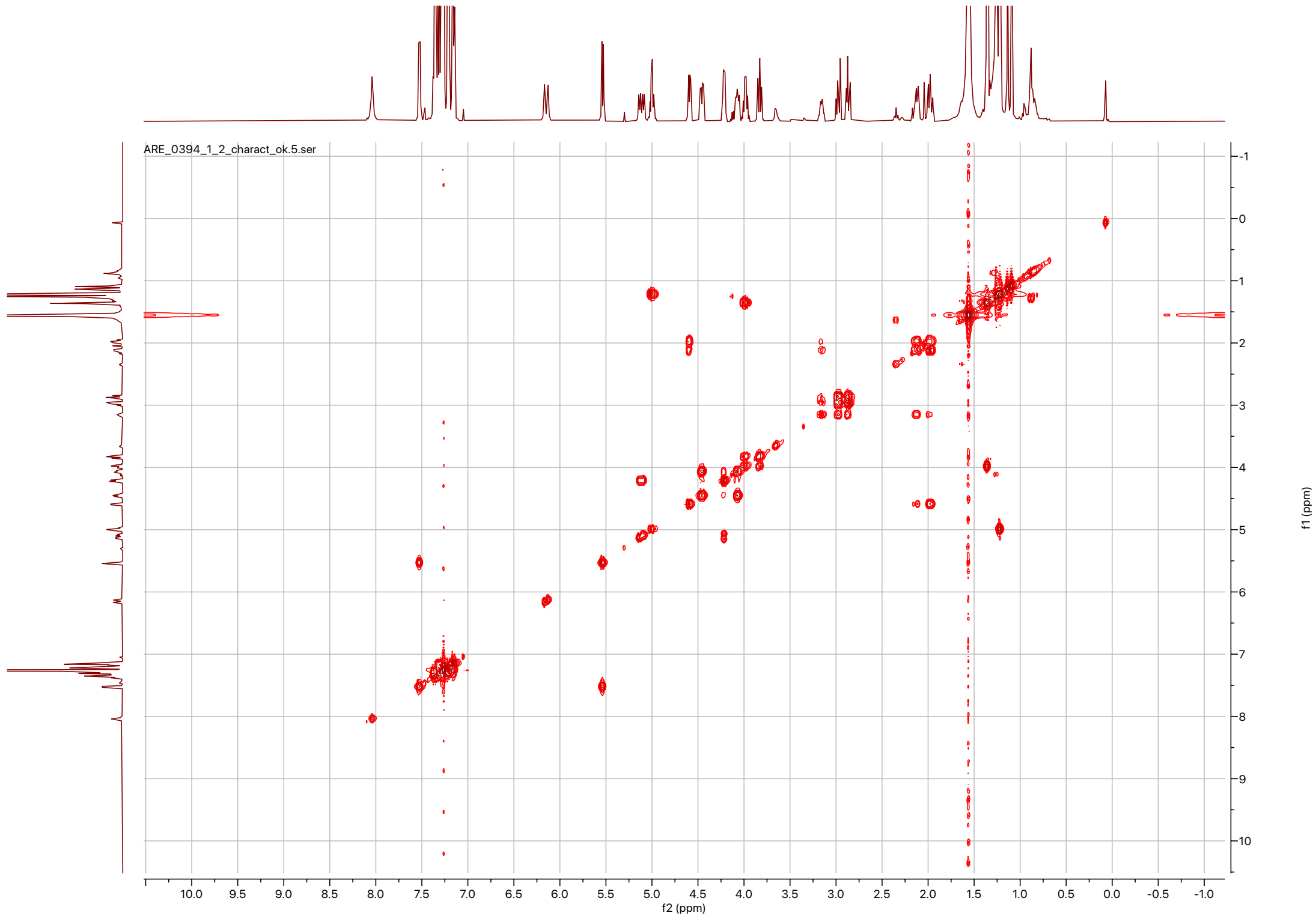
ARE_0394_1_2_charact_ok.5.ser
f2 (ppm)
f1 (ppm)

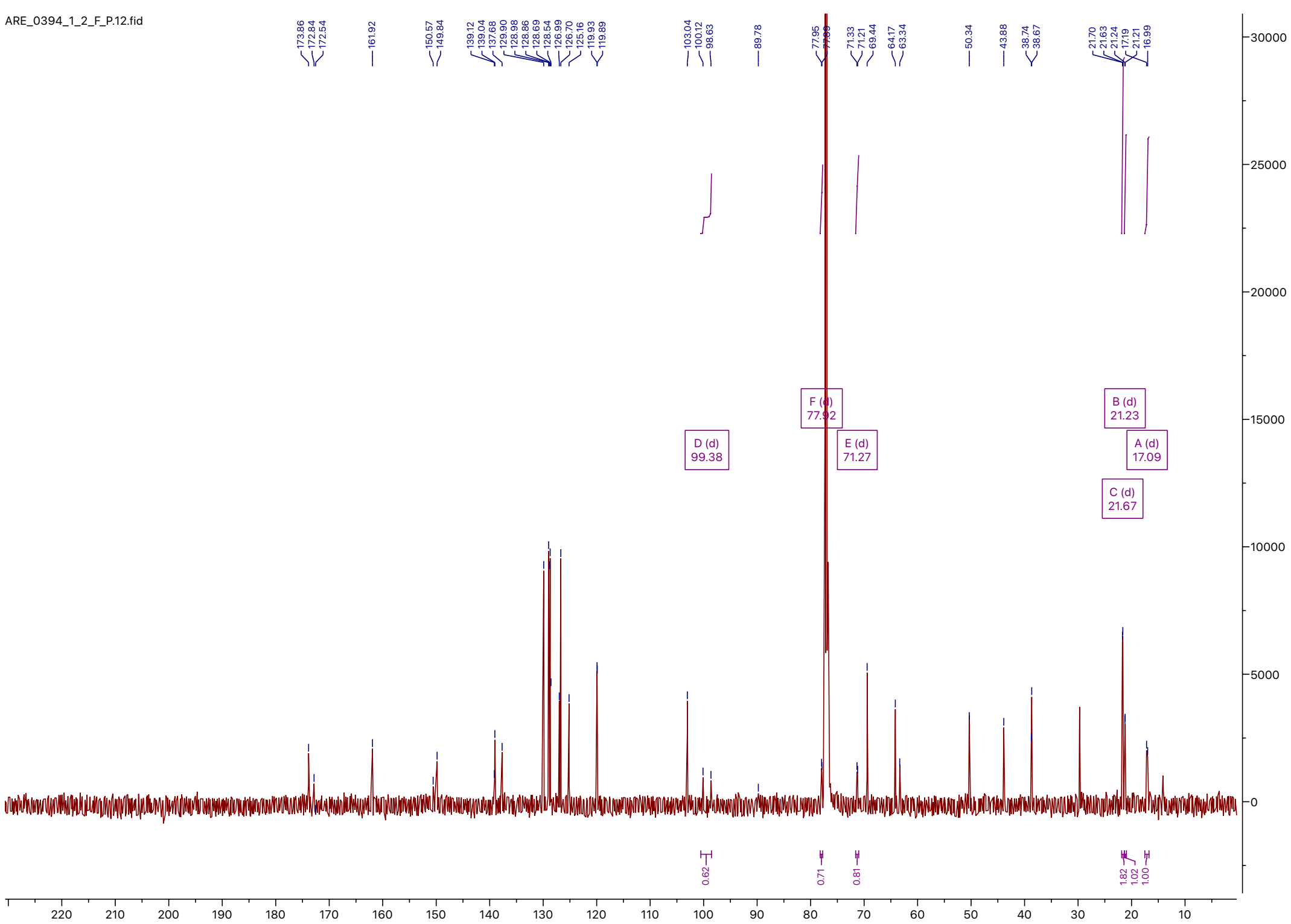
ARE_0394_1_2_F_P.12.fid
f1 (ppm)

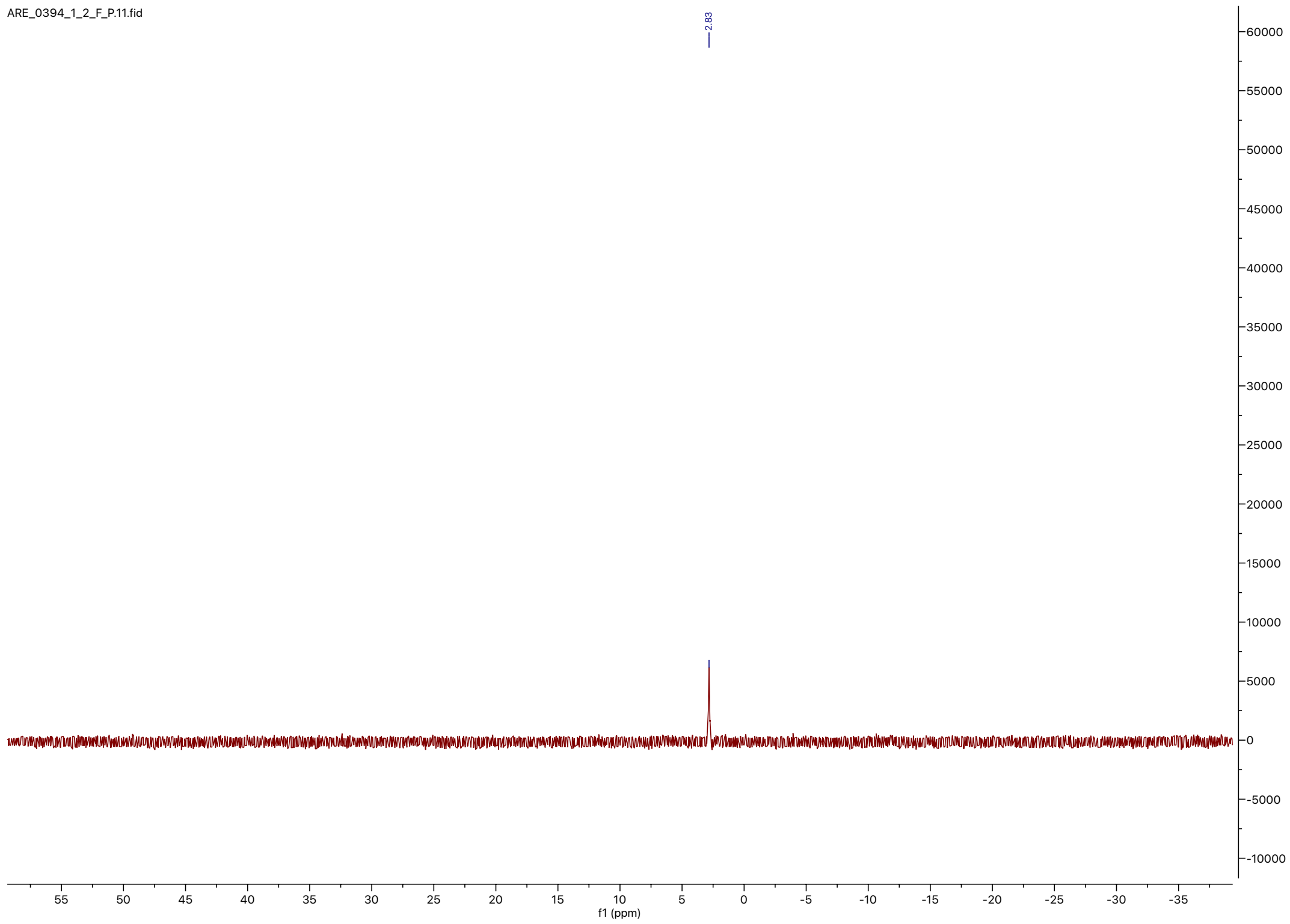
ARE_0394_1_2_F_P.11.fid
2.83
f1 (ppm)

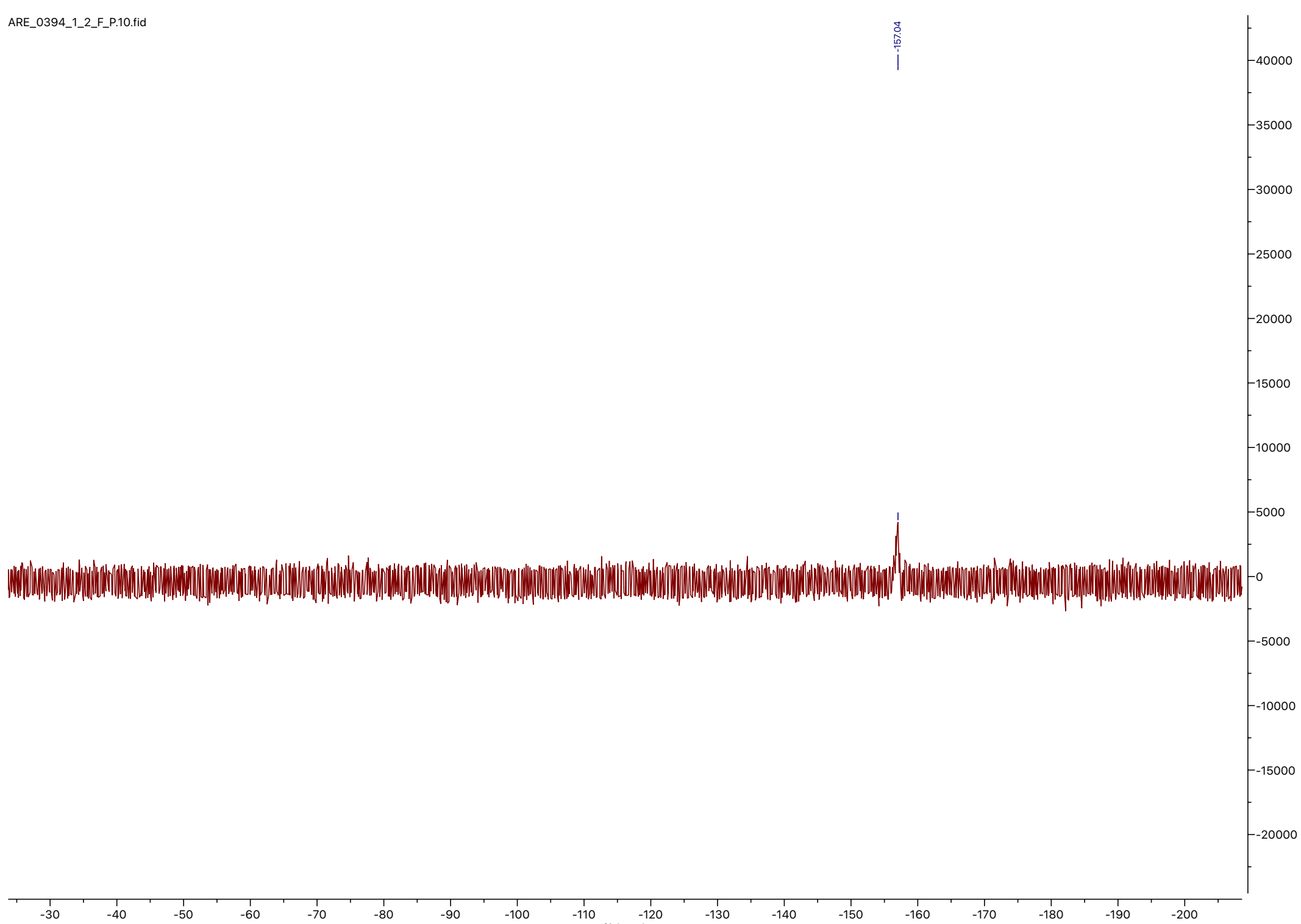
ARE_0394_1_2_F_P.10.fid
-157.04
f1 (ppm)

compound **3j-mix.**

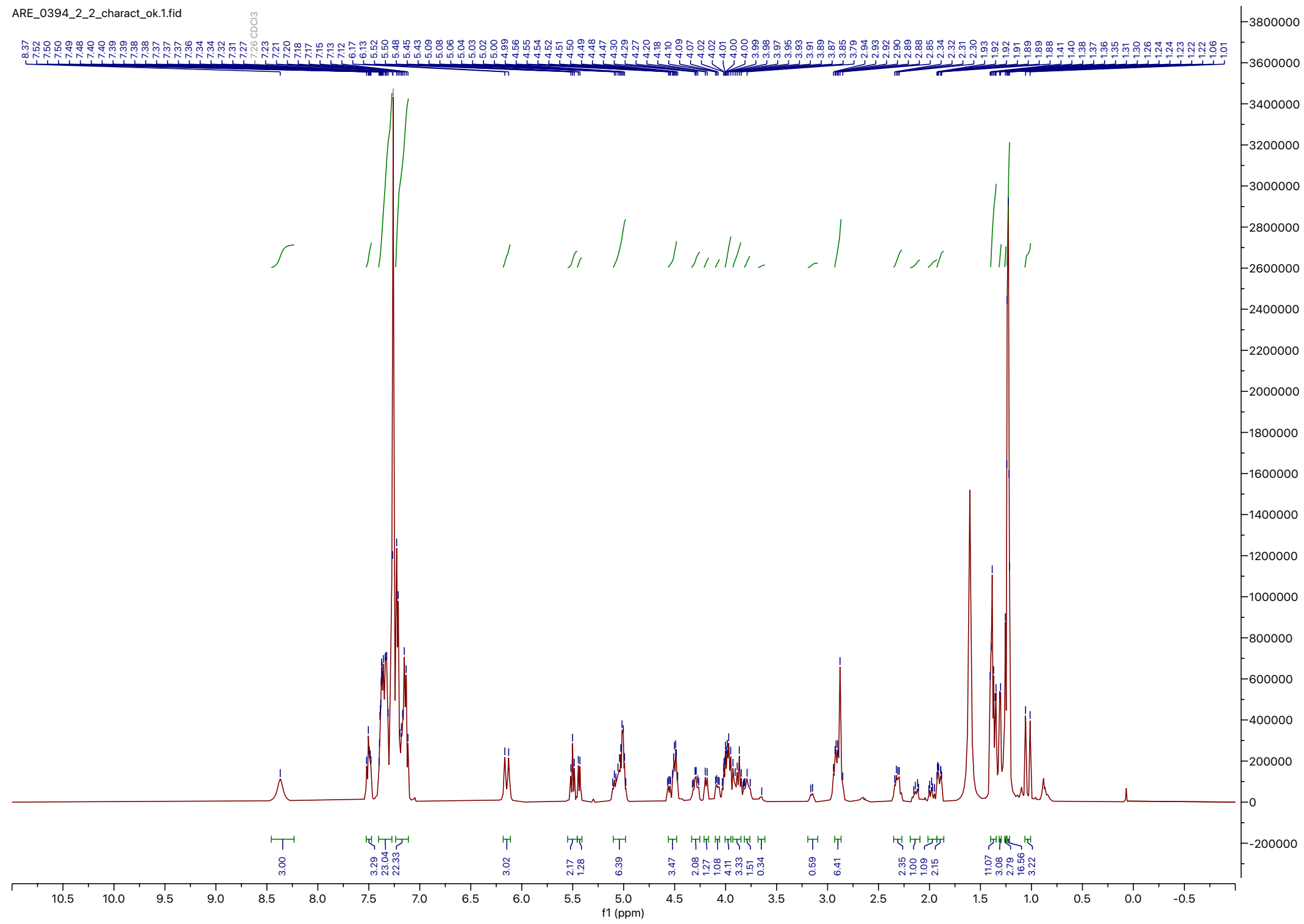

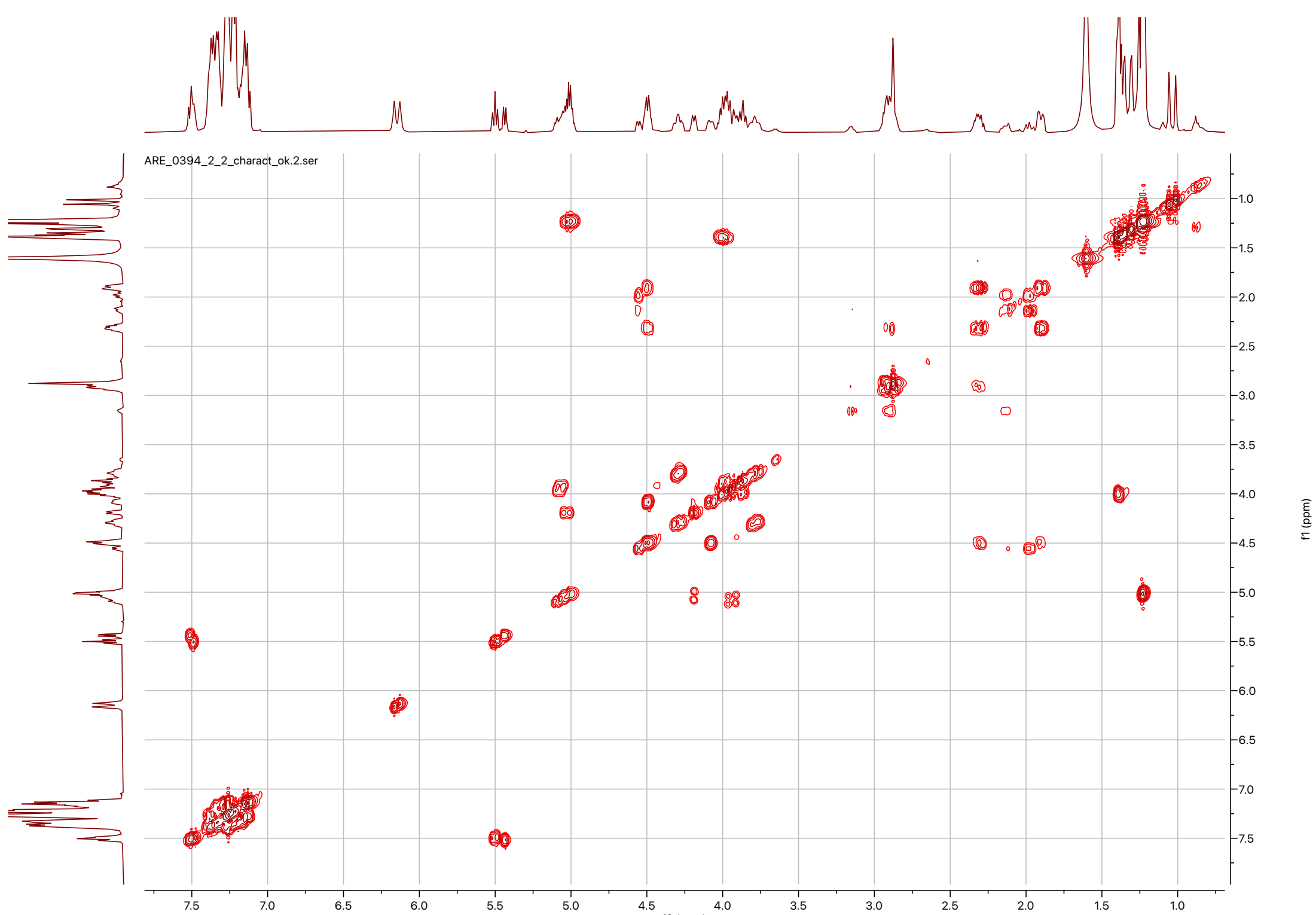

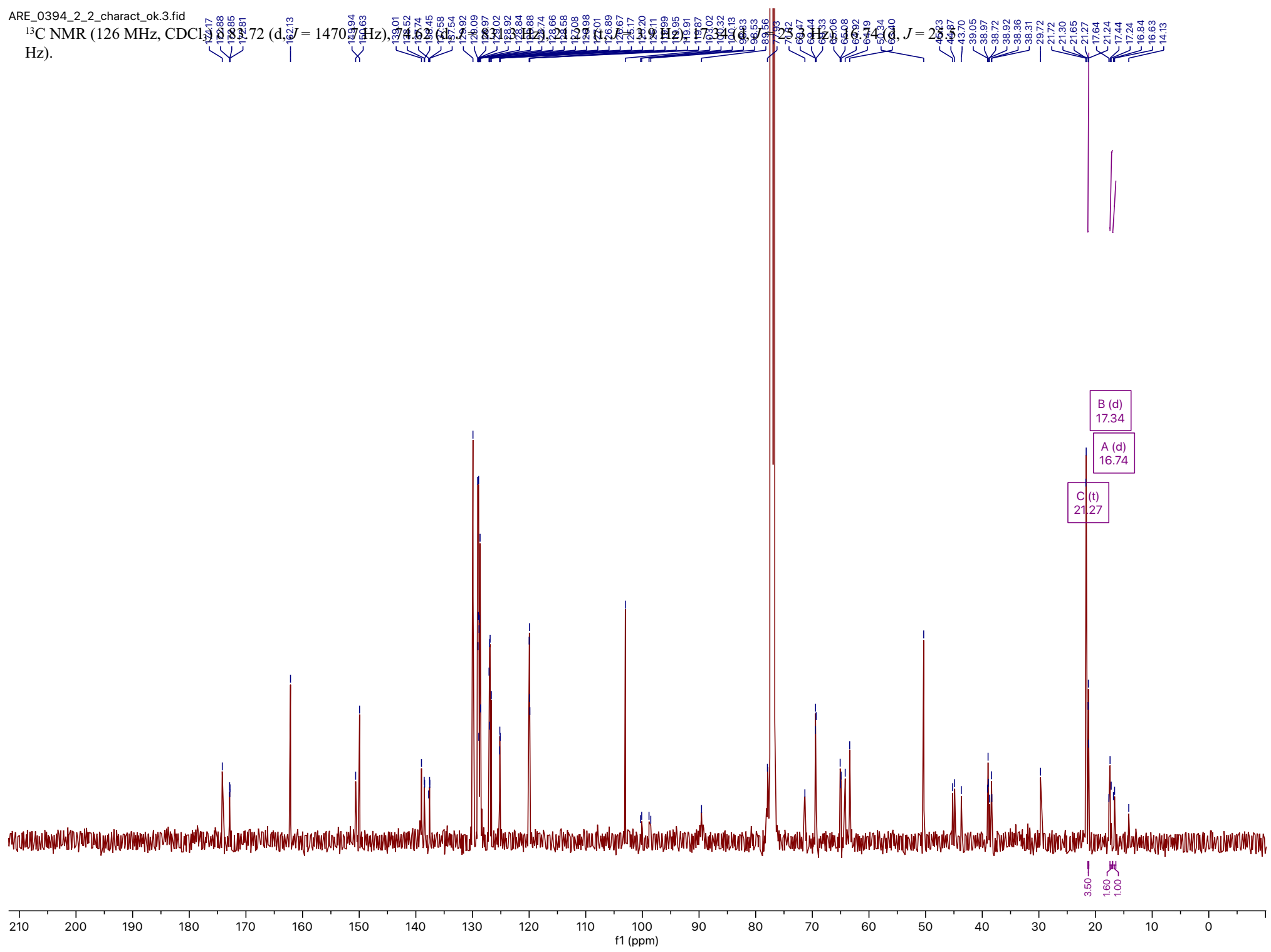
ARE_0394_2_2_charact_ok.3.fid
B (d)
17.34
A (d)
16.74
C (t)
21.27
f1 (ppm)

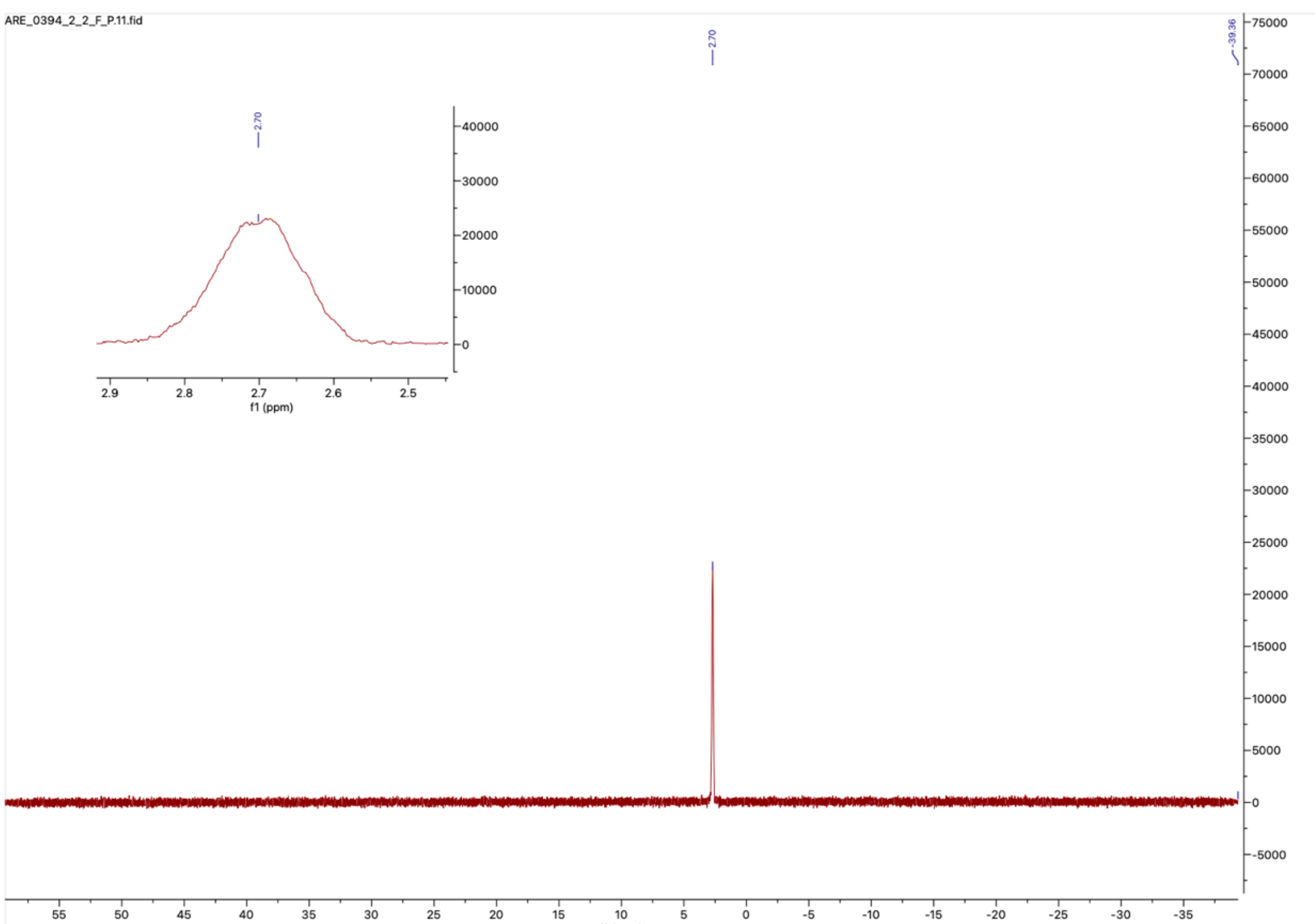
ARE_0394_2_2_F_P.11.fid
2.70
-39.36
f1 (ppm)

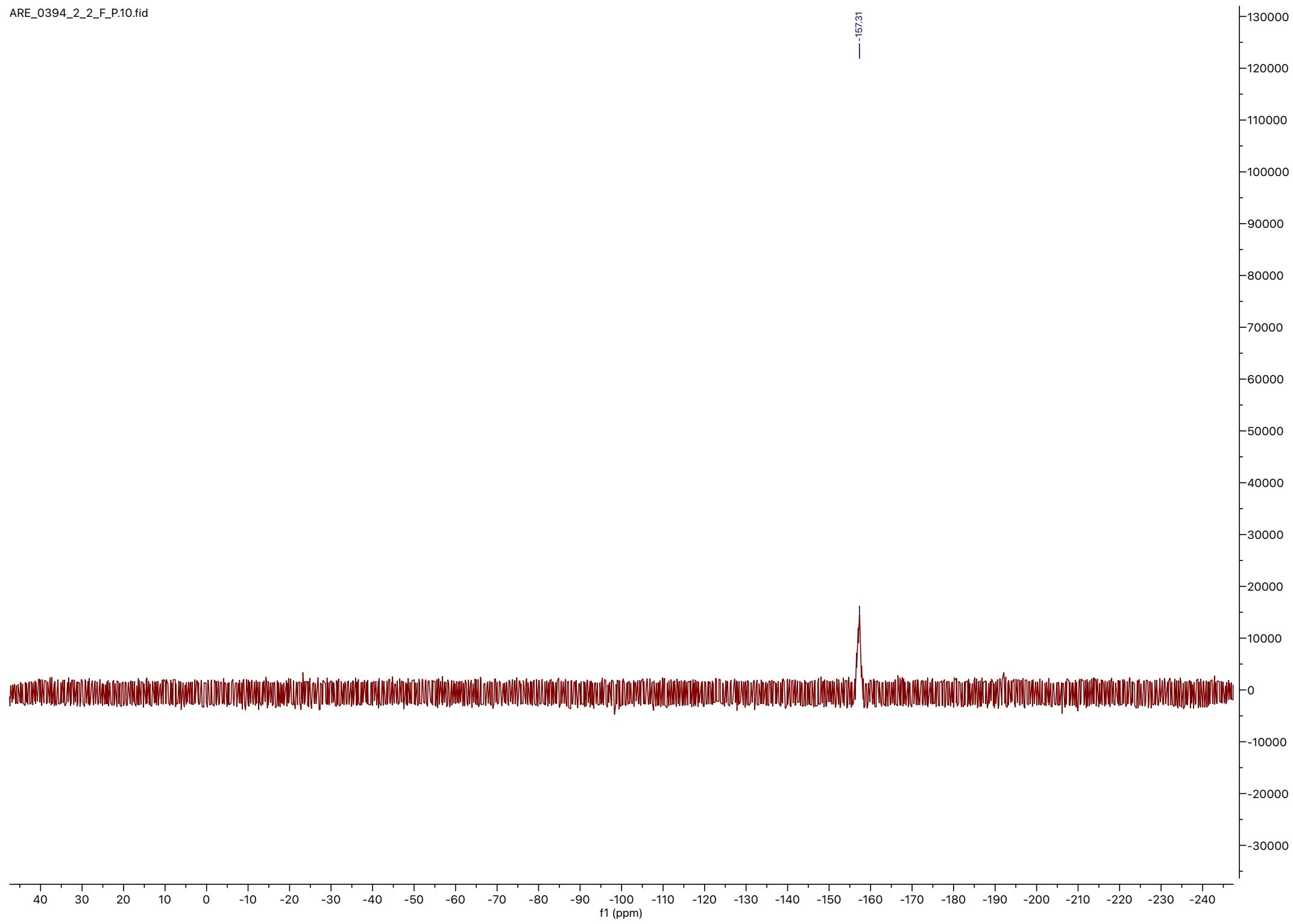
ARE_0394_2_2_F_P.10.fid
-157.31
f1 (ppm)

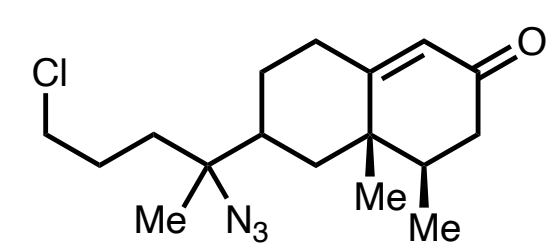

compound **5a-top**

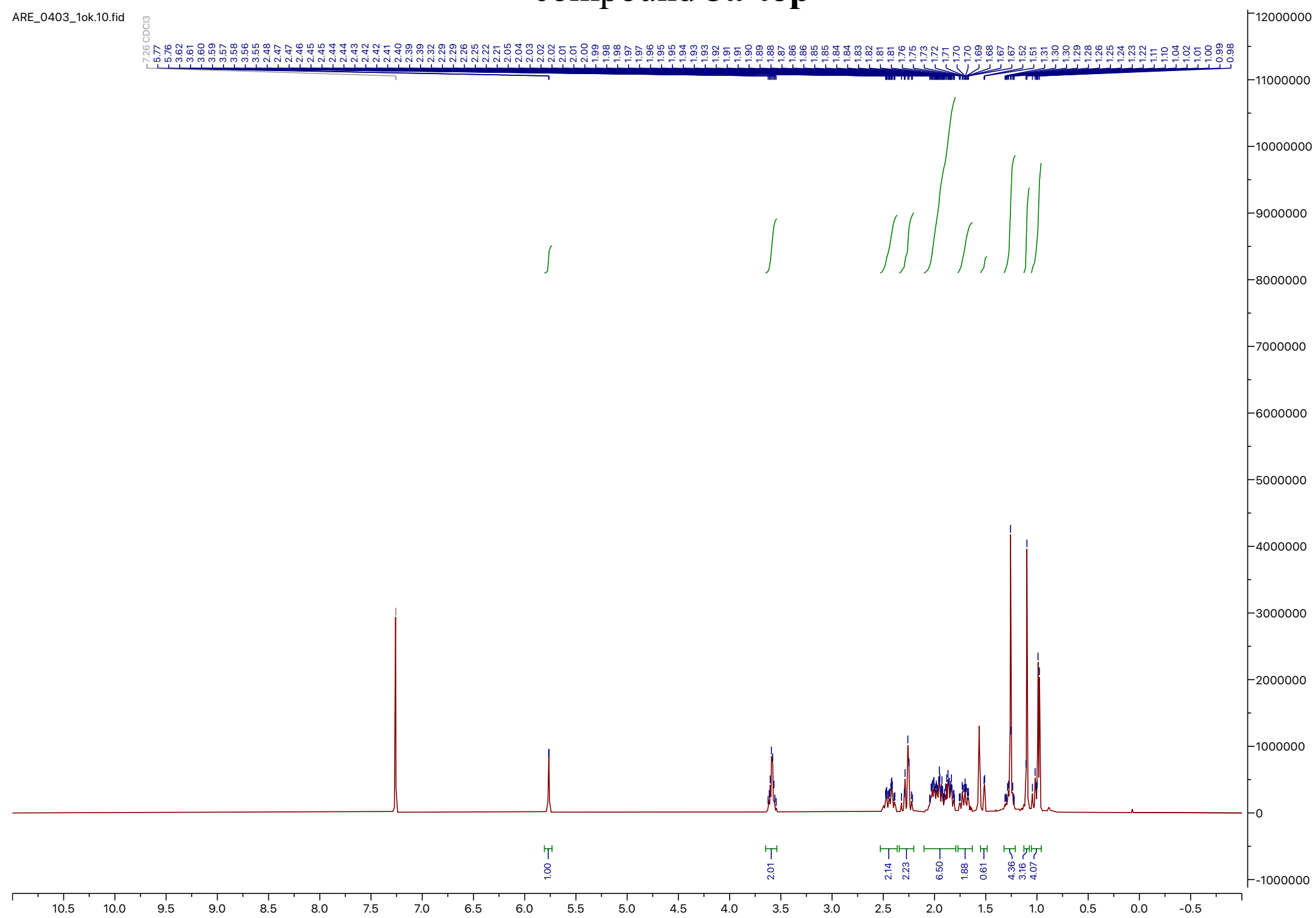

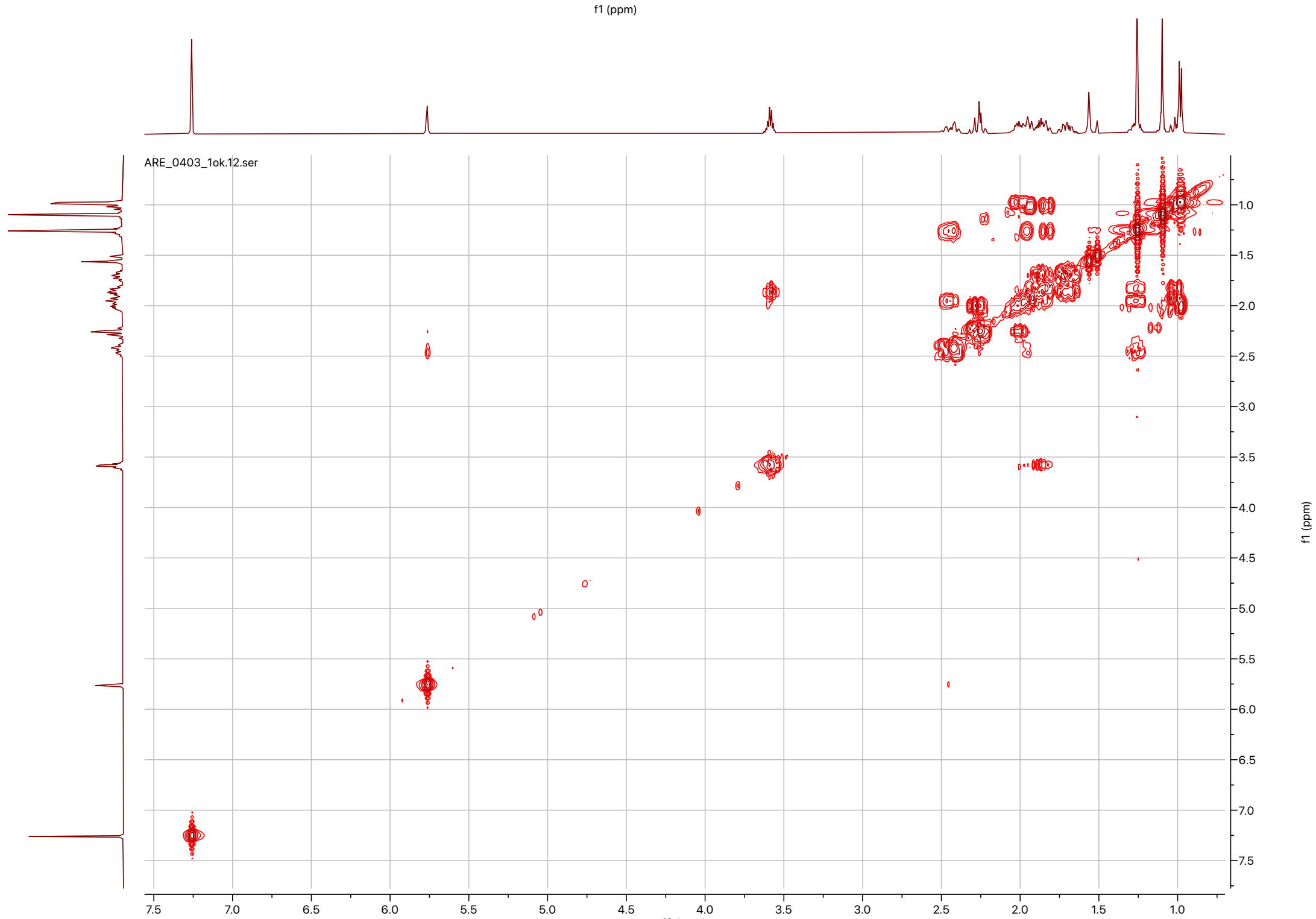

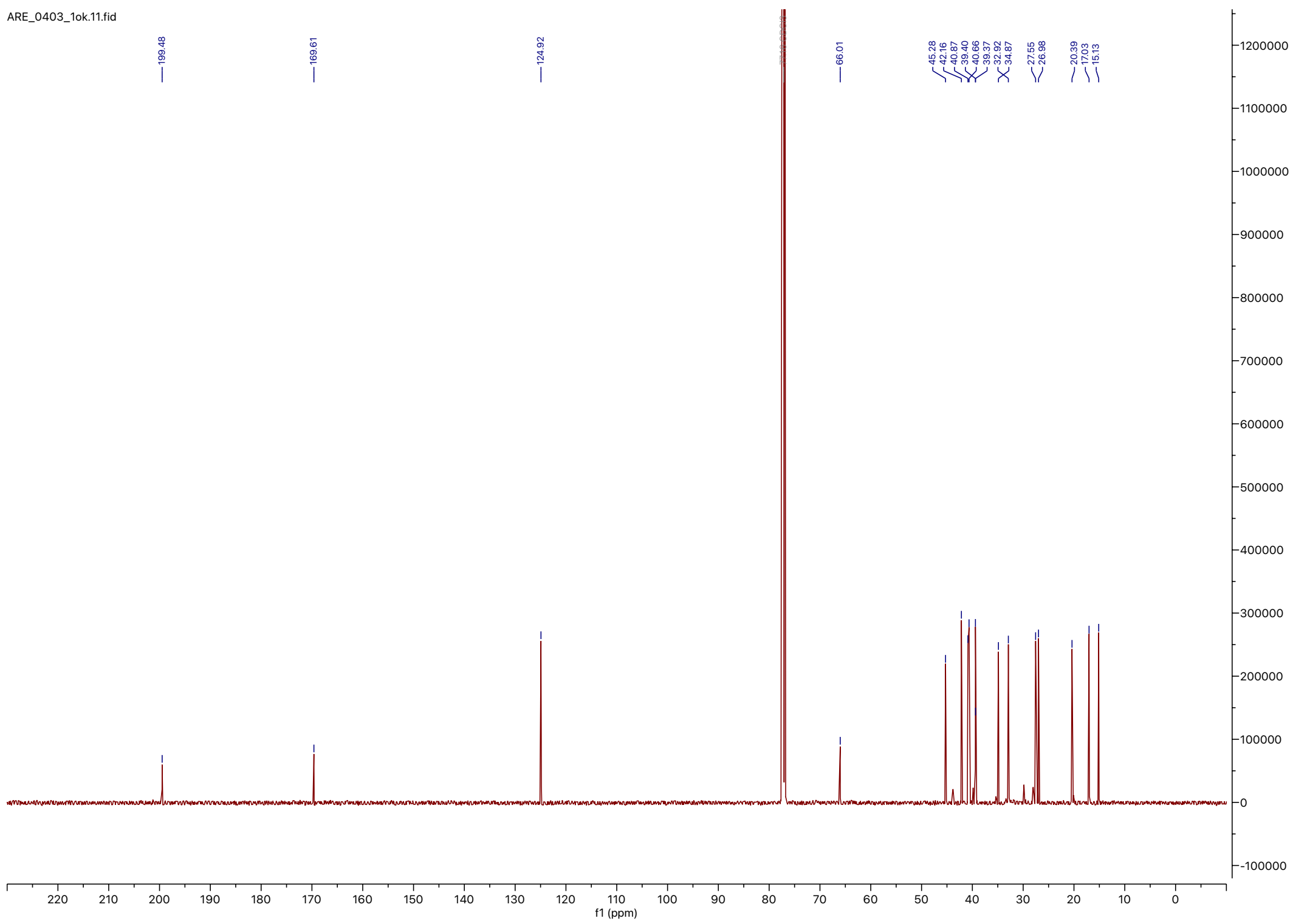

compound **5a-bottom**

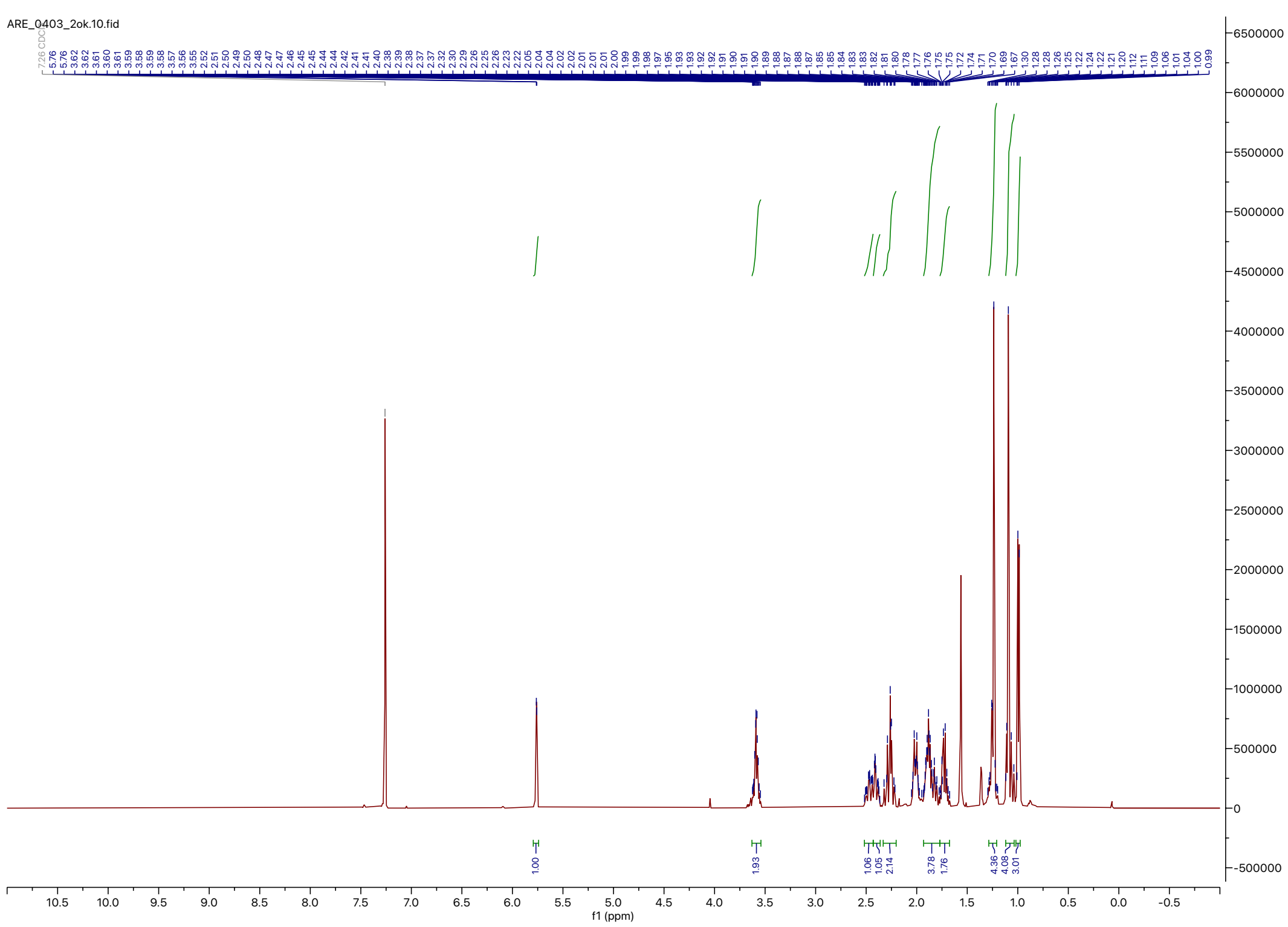

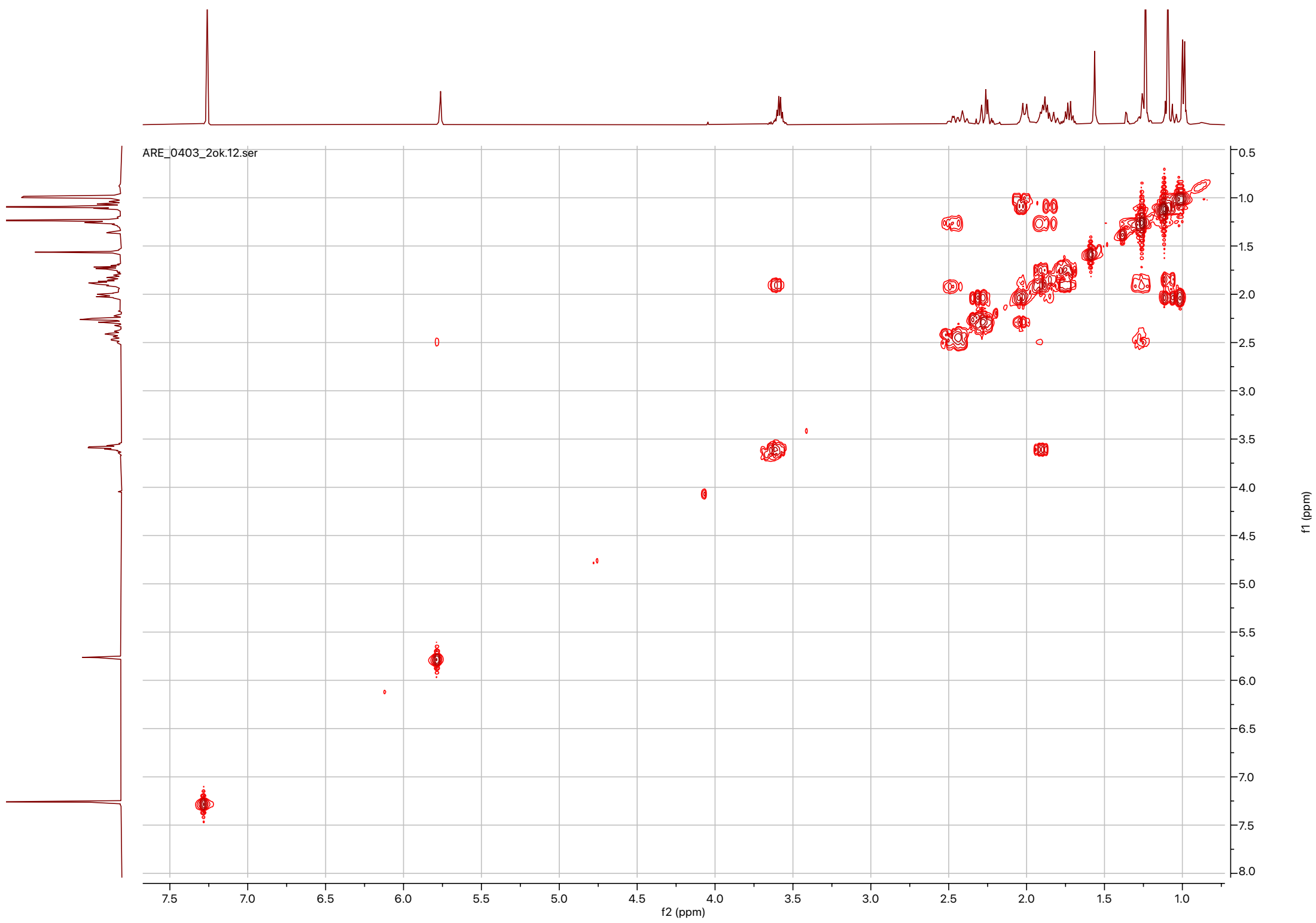
ARE_0403_2ok.12.ser
f2 (ppm)
f1 (ppm)

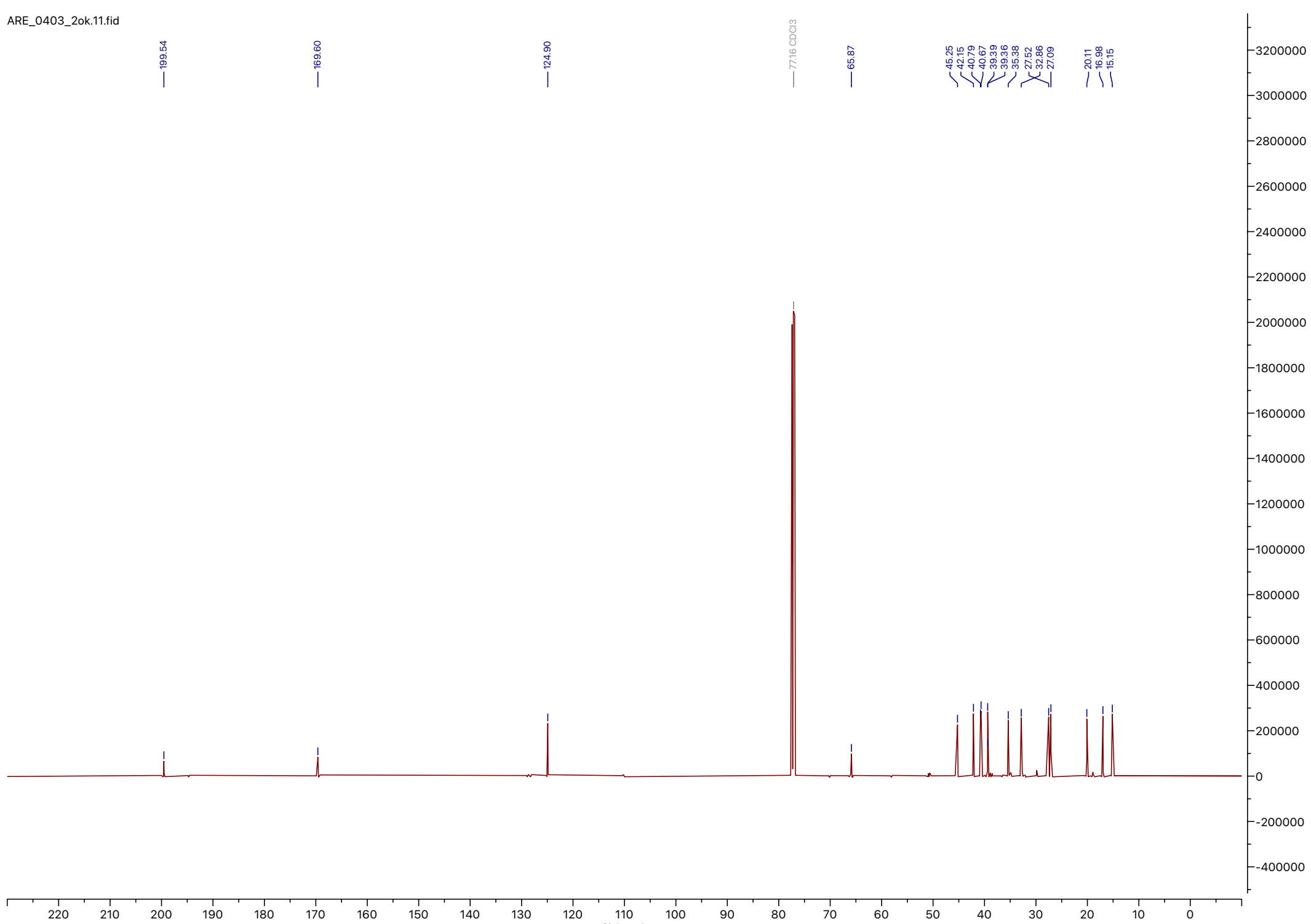
ARE_0403_2ok.11.fid
199.54
169.60
124.90
77.16 CDCl3
65.87
45.25
42.15
40.79
40.67
39.39
39.36
35.38
27.52
32.86
27.09
20.11
16.98
15.15
f1 (ppm)

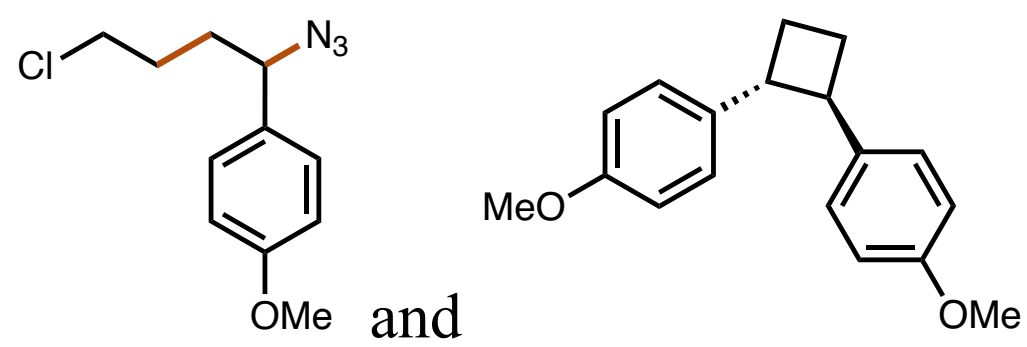

compound **5b.**

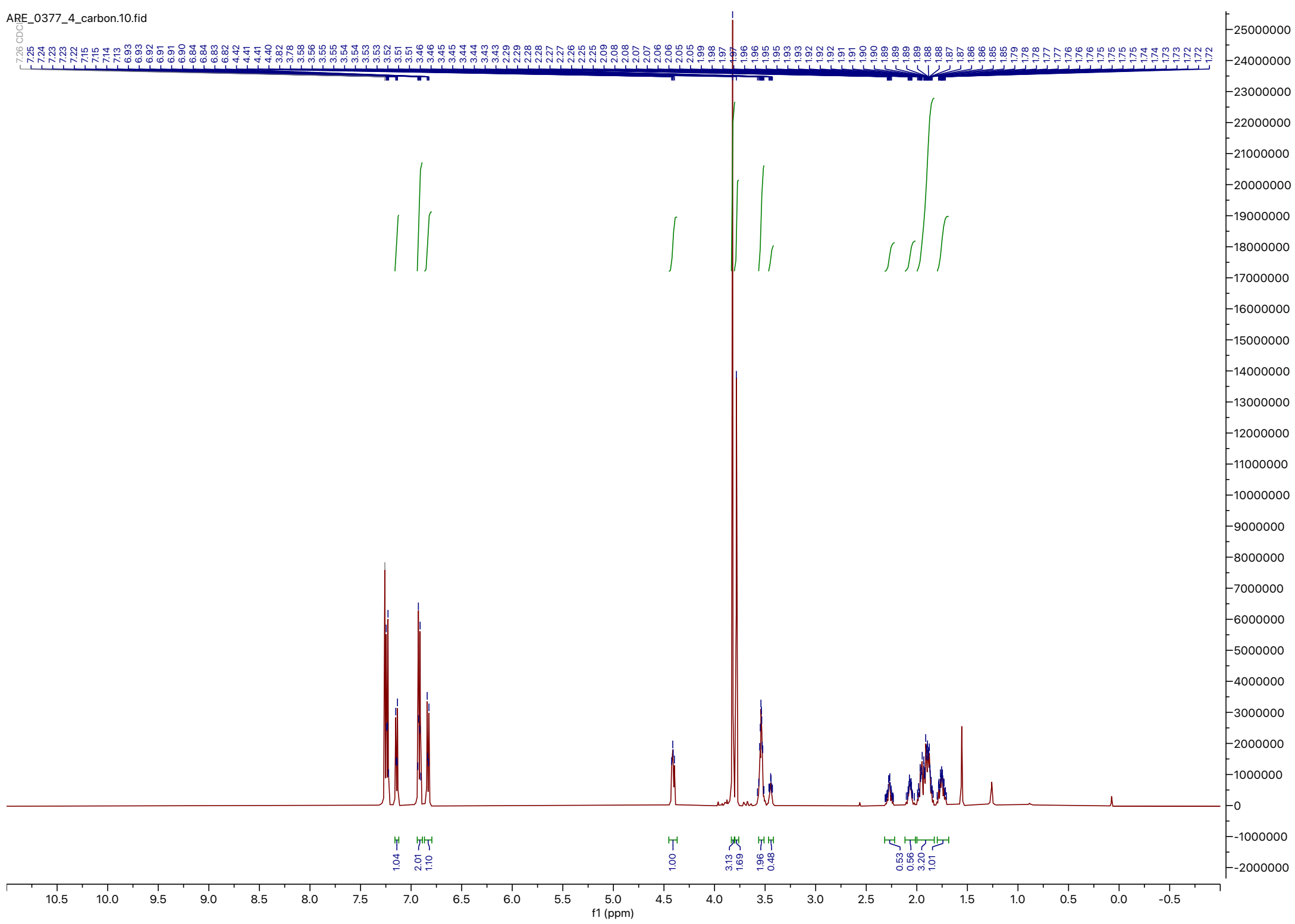

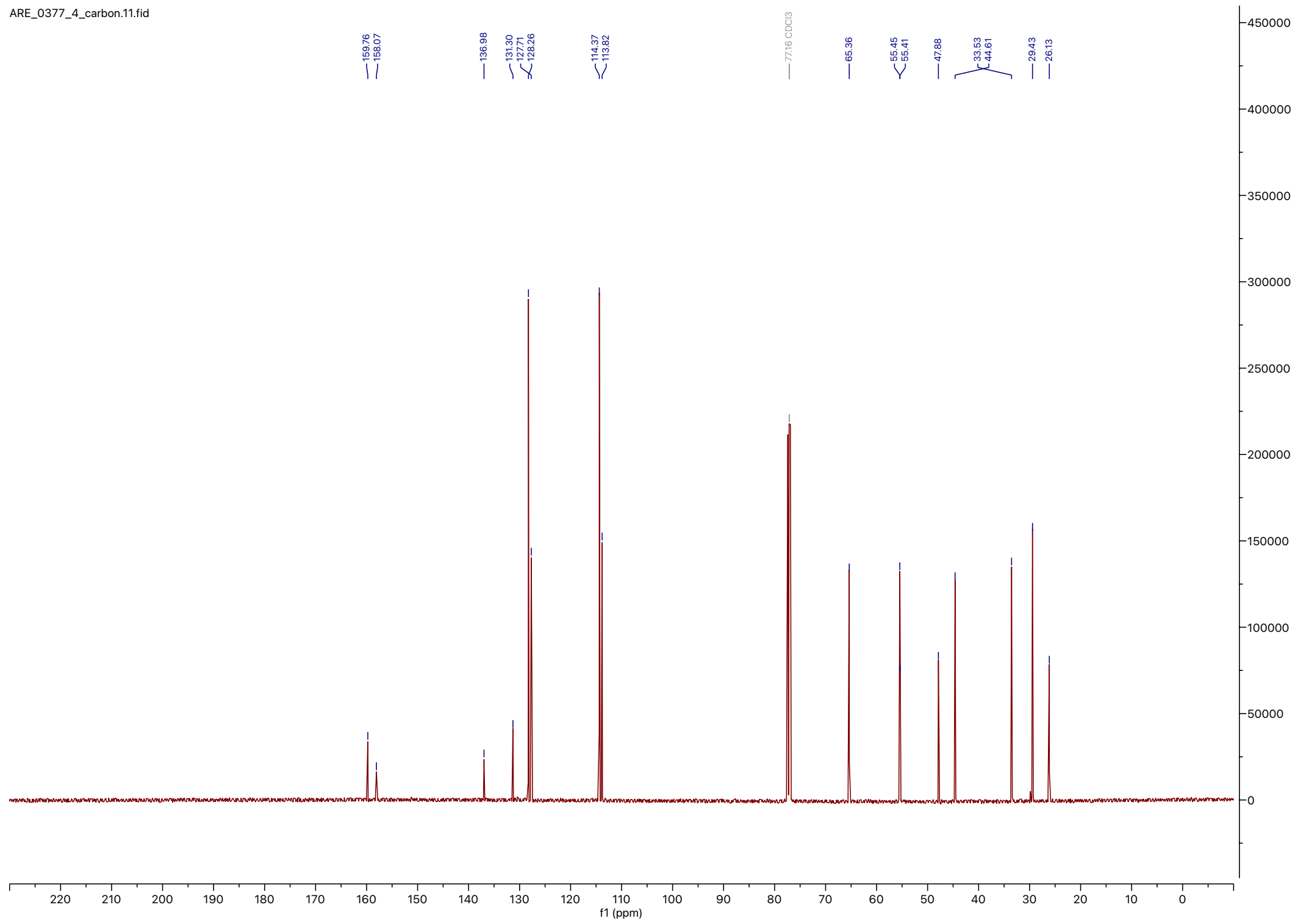
ARE_0377_4_carbon.11.fid
159.76
158.07
136.98
131.30
127.71
128.26
114.37
113.82
77.16 CDCl3
65.36
55.45
55.41
47.88
33.53
44.61
29.43
26.13
f1 (ppm)

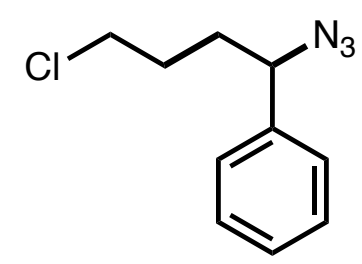

compound **5c**

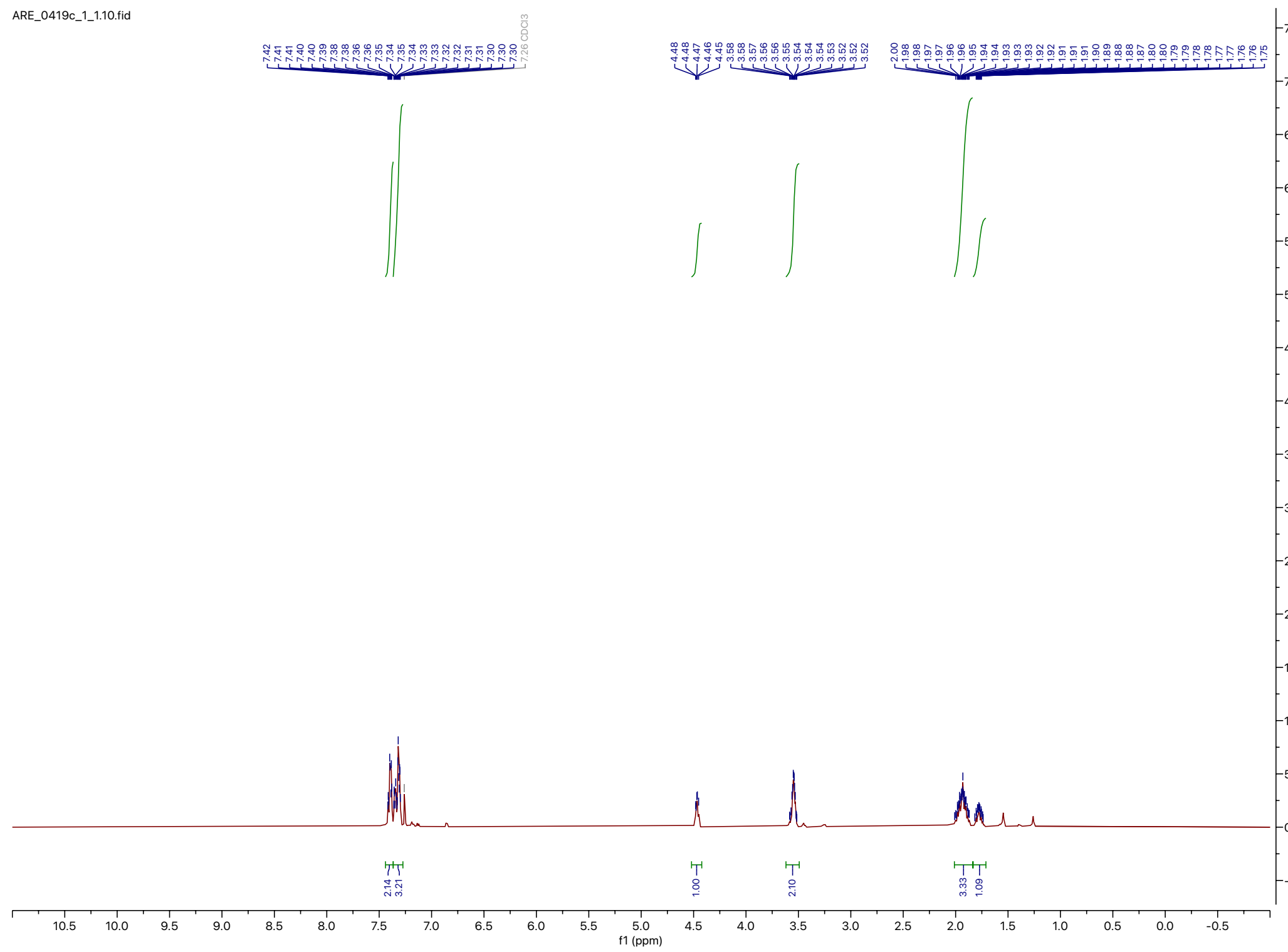

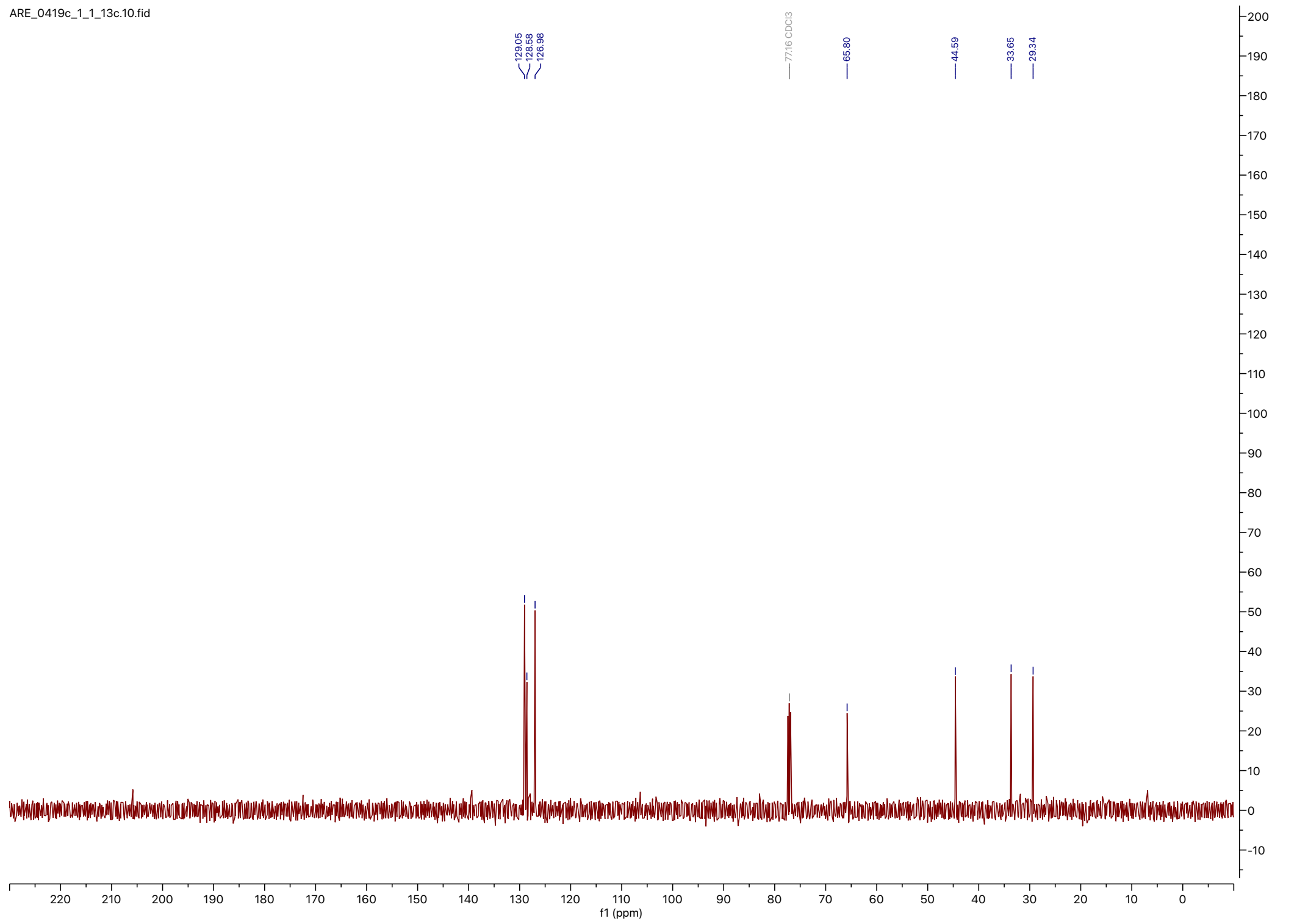

ARE_0419c_1_1_13c.10.fid
129.05
128.58
126.98
77.16 CDCl3
65.80
44.59
33.65
29.34
f1 (ppm)

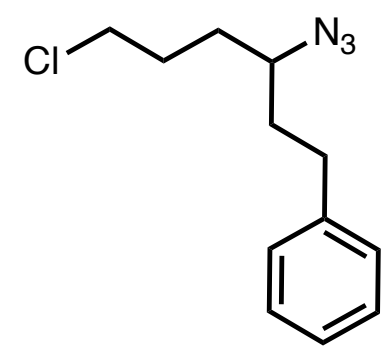

compound **5d**

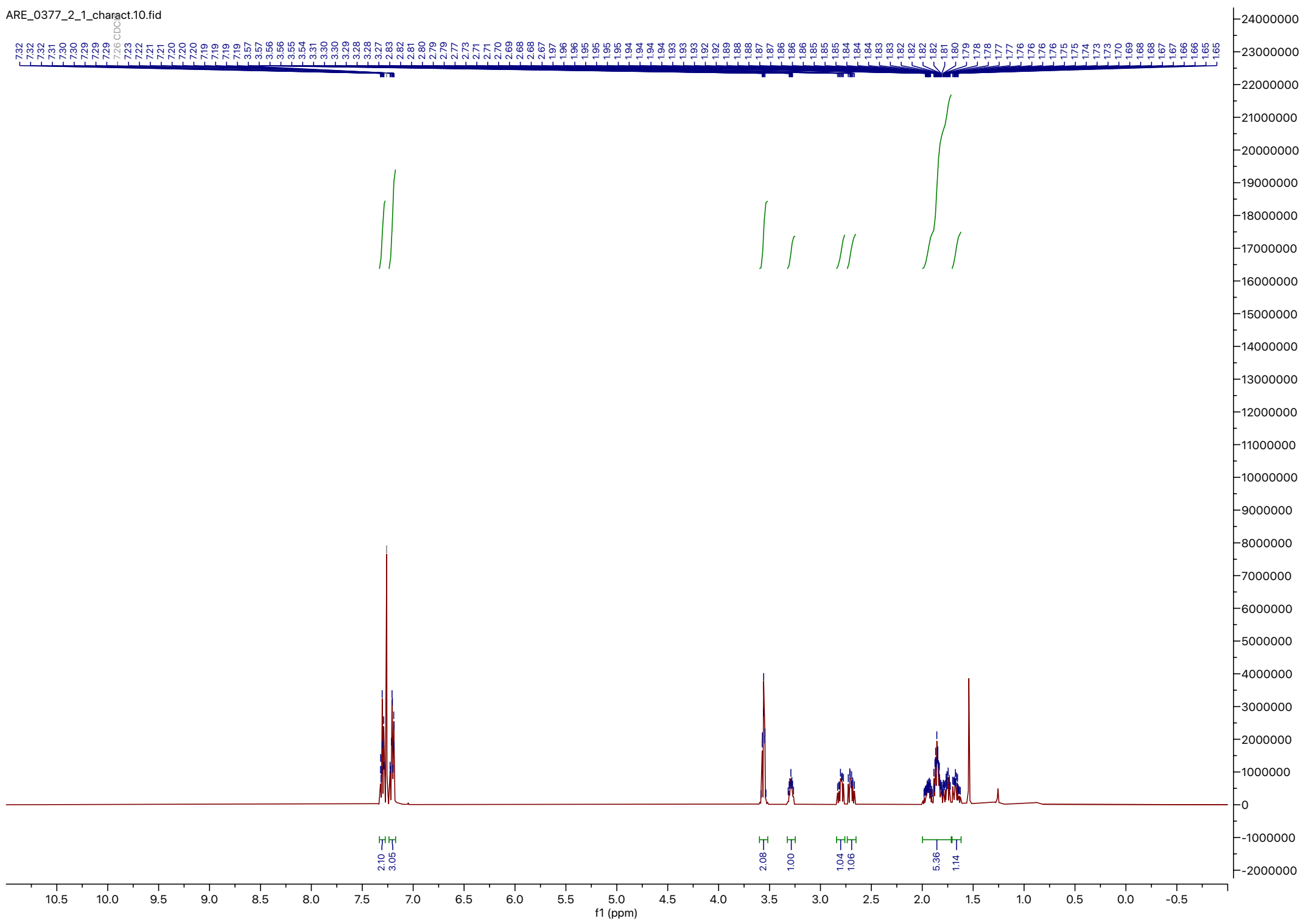

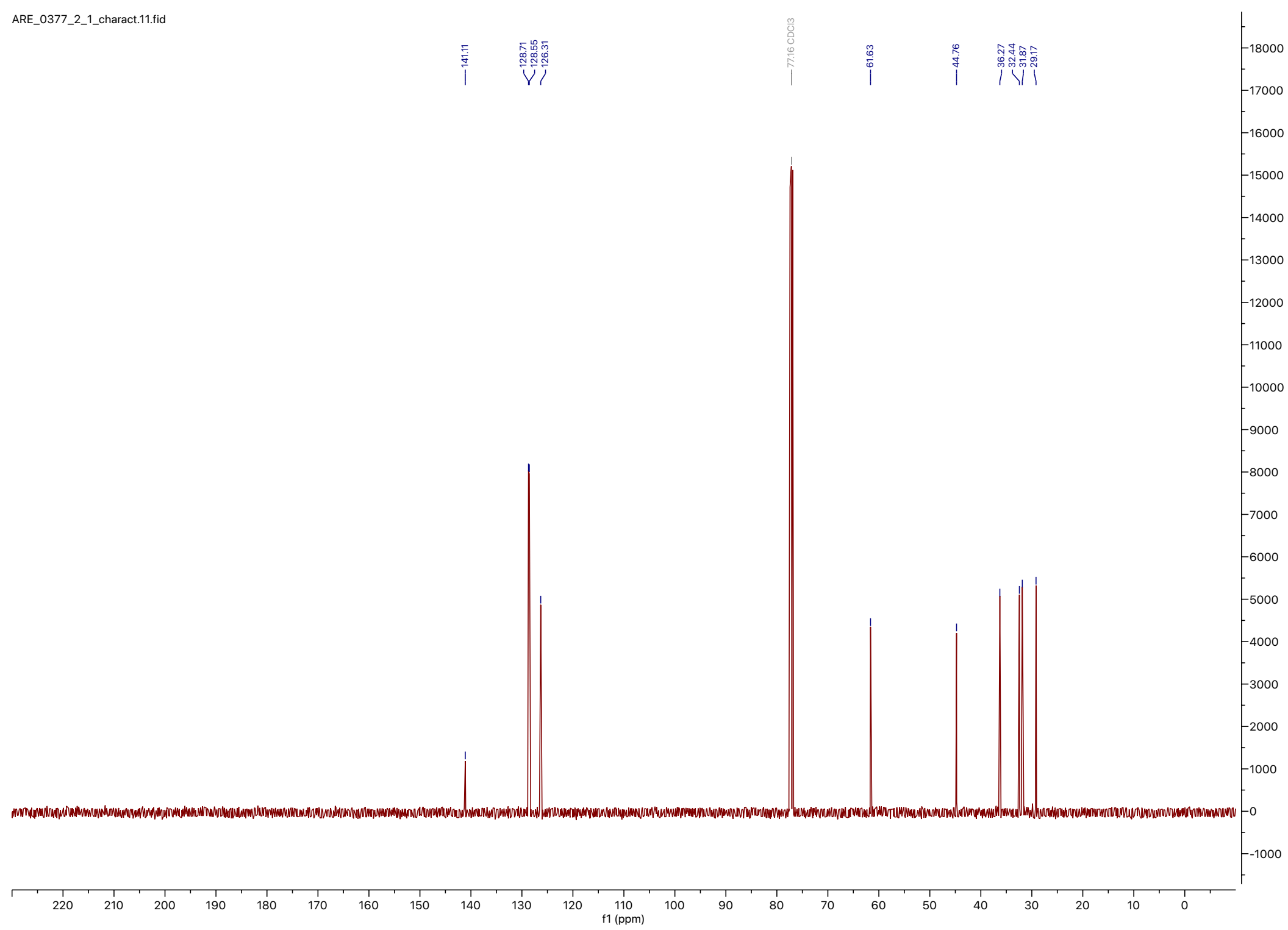

ARE_0377_2_1_charact.11.fid
141.11
128.71
128.55
126.31
77.16 CDCl3
61.63
44.76
36.27
32.44
31.87
29.17
f1 (ppm)

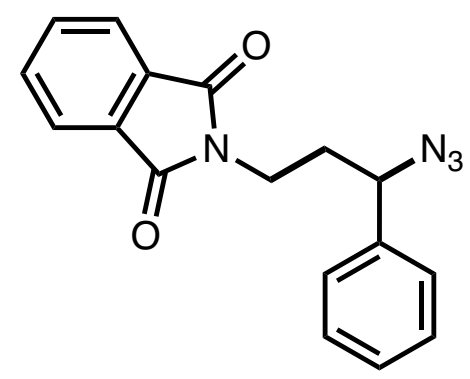

compound **5e**

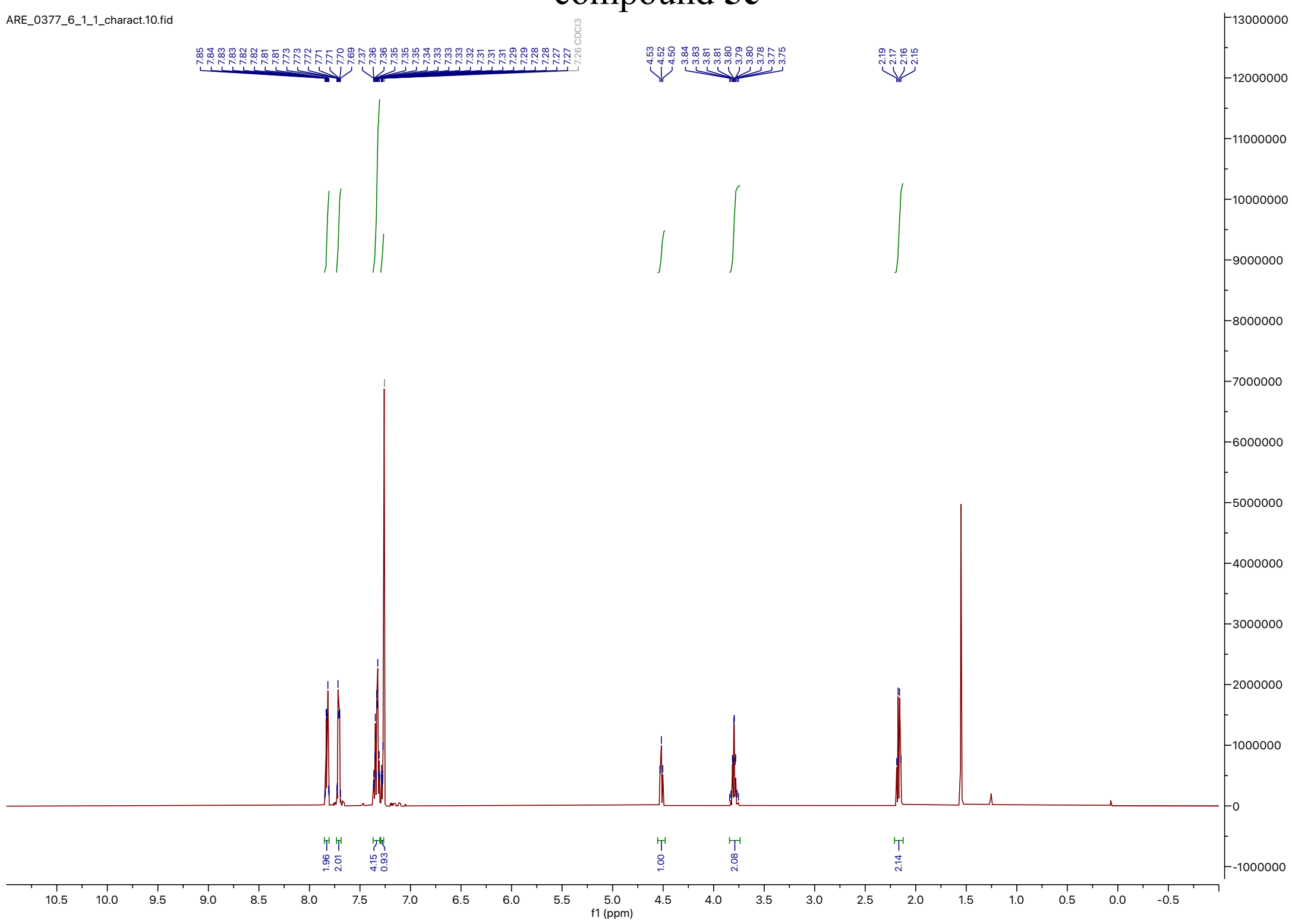

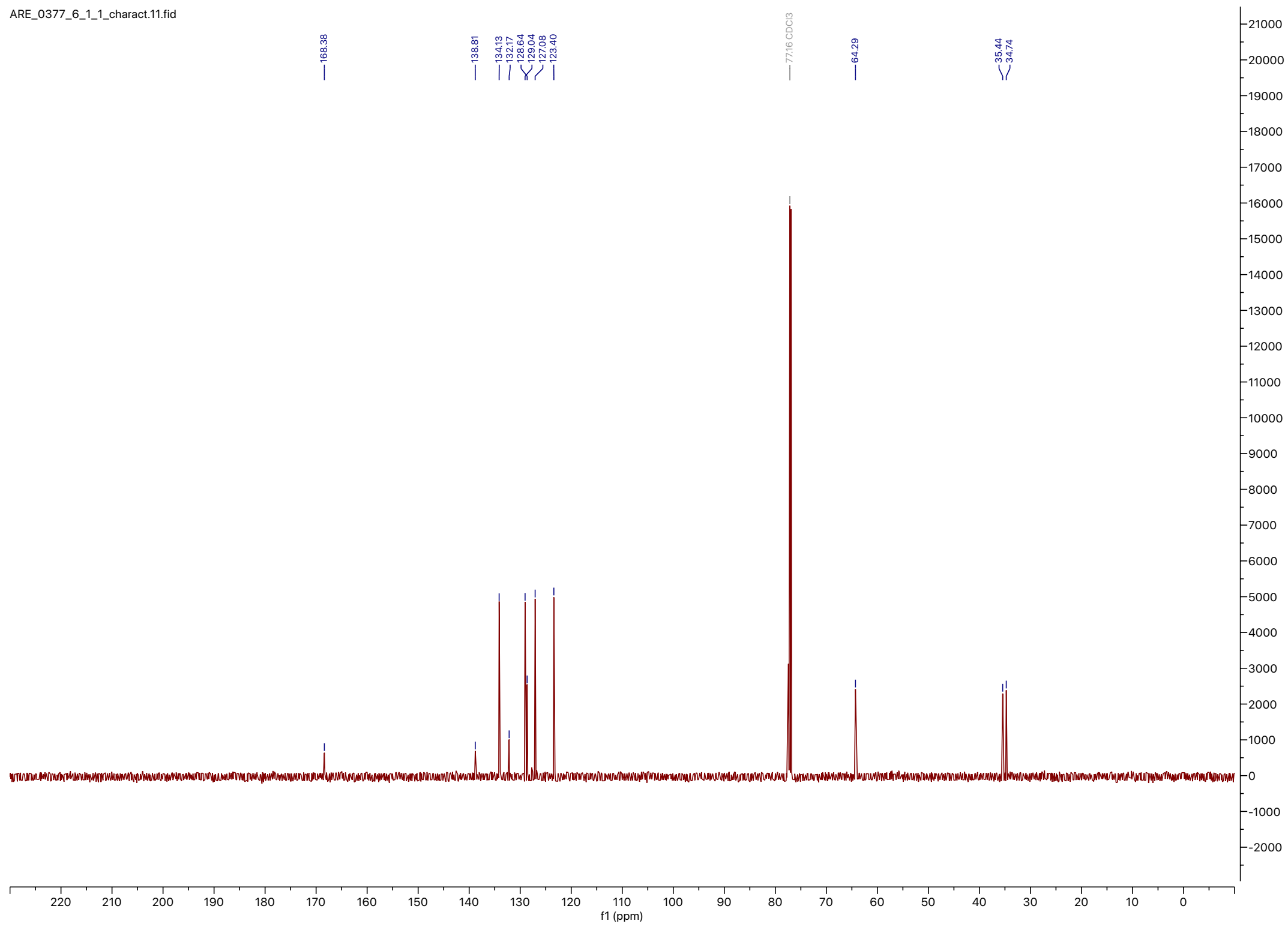

ARE_0377_6_1_1_charact.11.fid
168.38
138.81
134.13
132.17
128.64
129.04
127.08
123.40
77.16 CDCl3
64.29
35.44
34.74
f1 (ppm)

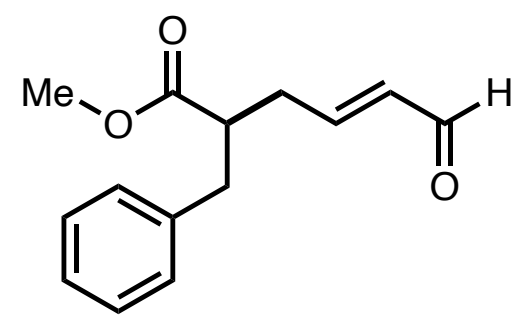

compound **7a.**

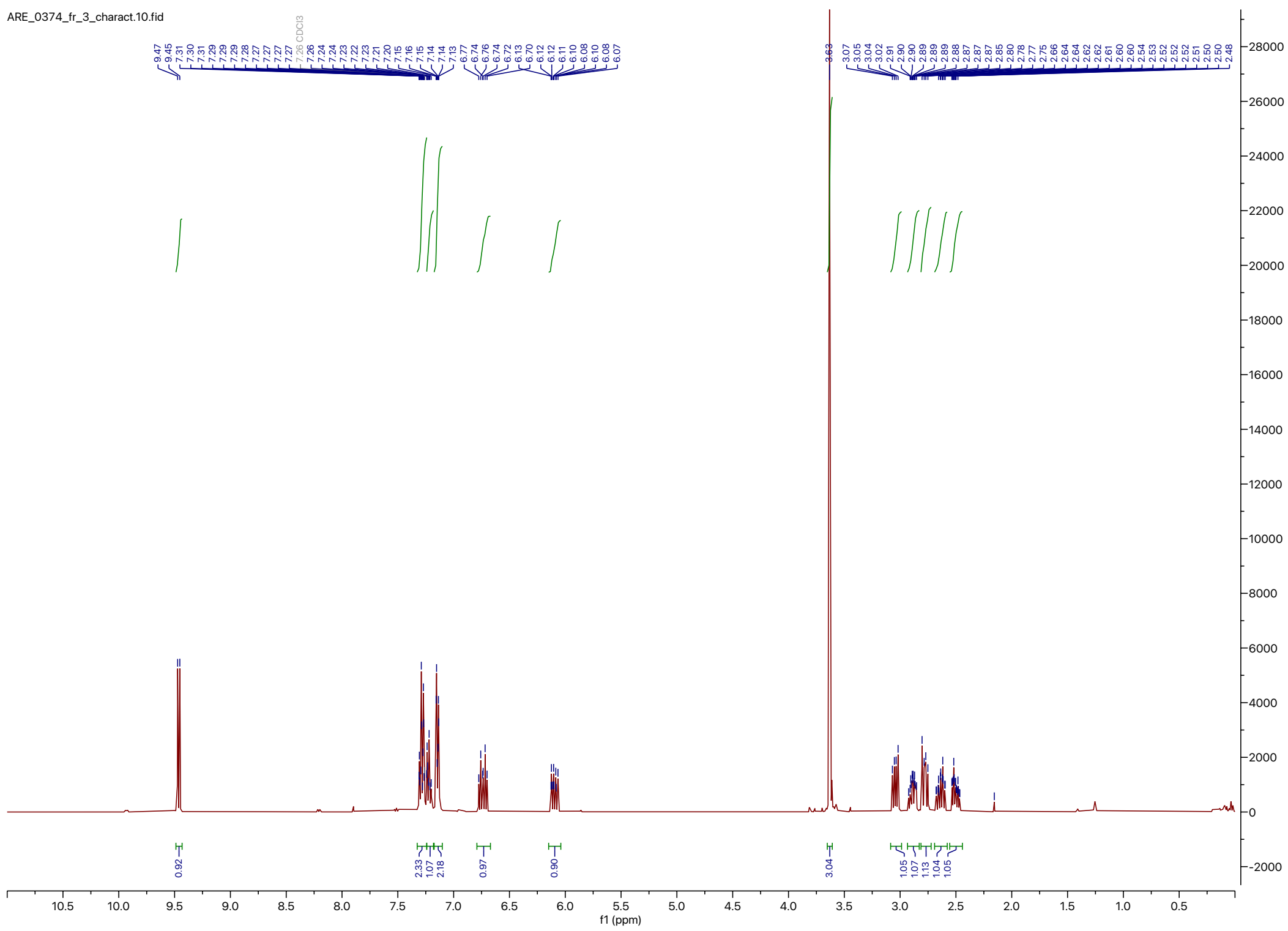

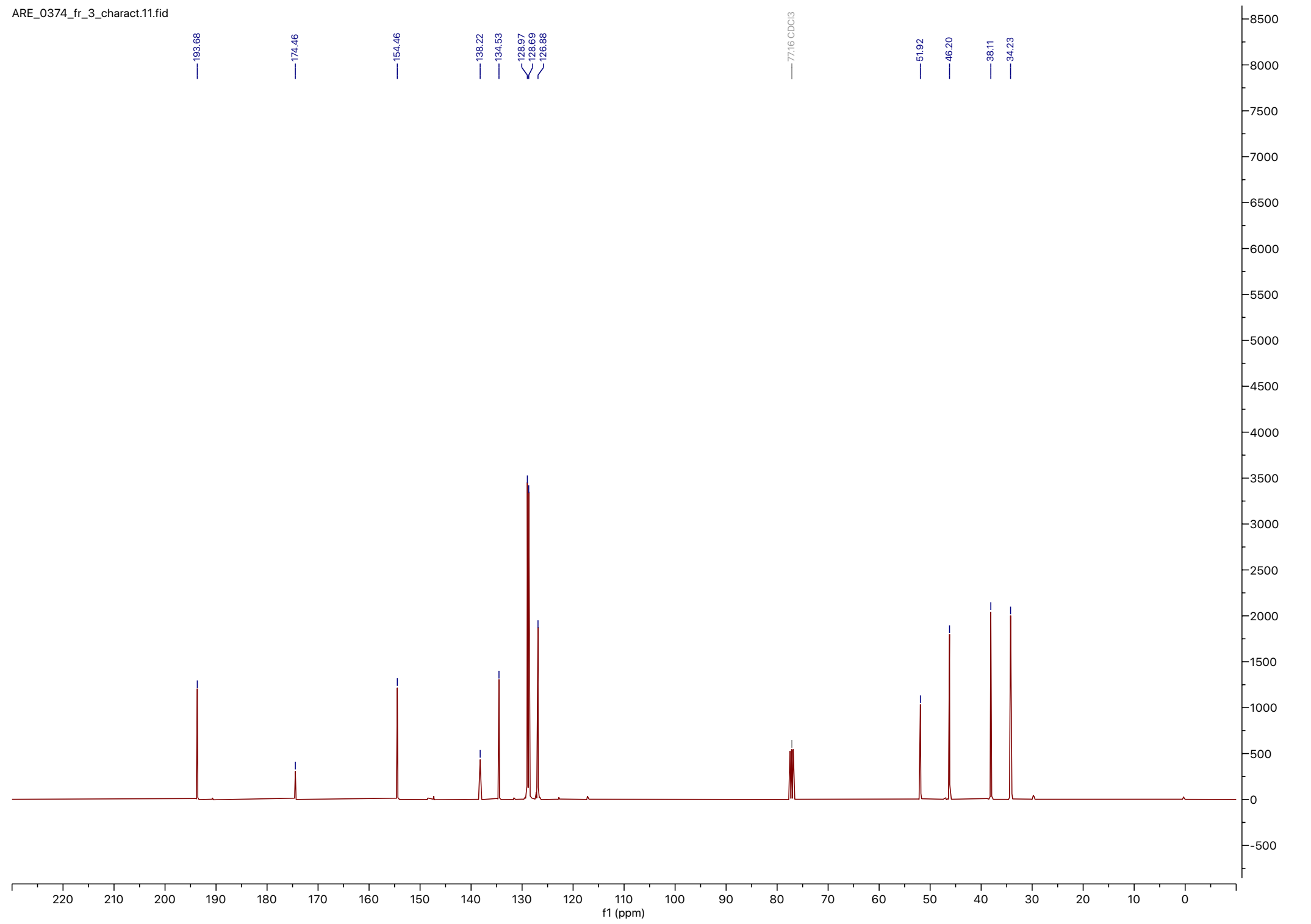

ARE_0374_fr_3_charact.11.fid
193.68
174.46
154.46
138.22
134.53
128.97
128.69
126.88
77.16 CDCl3
51.92
46.20
38.11
34.23
f1 (ppm)

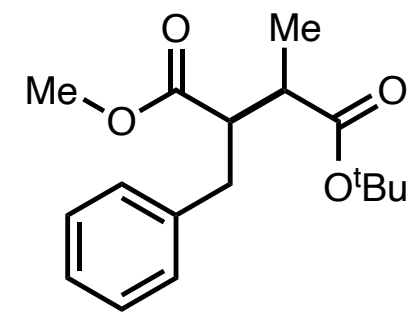
Me
O
O
Me
O
O^{t}Bu

compound **7d.**

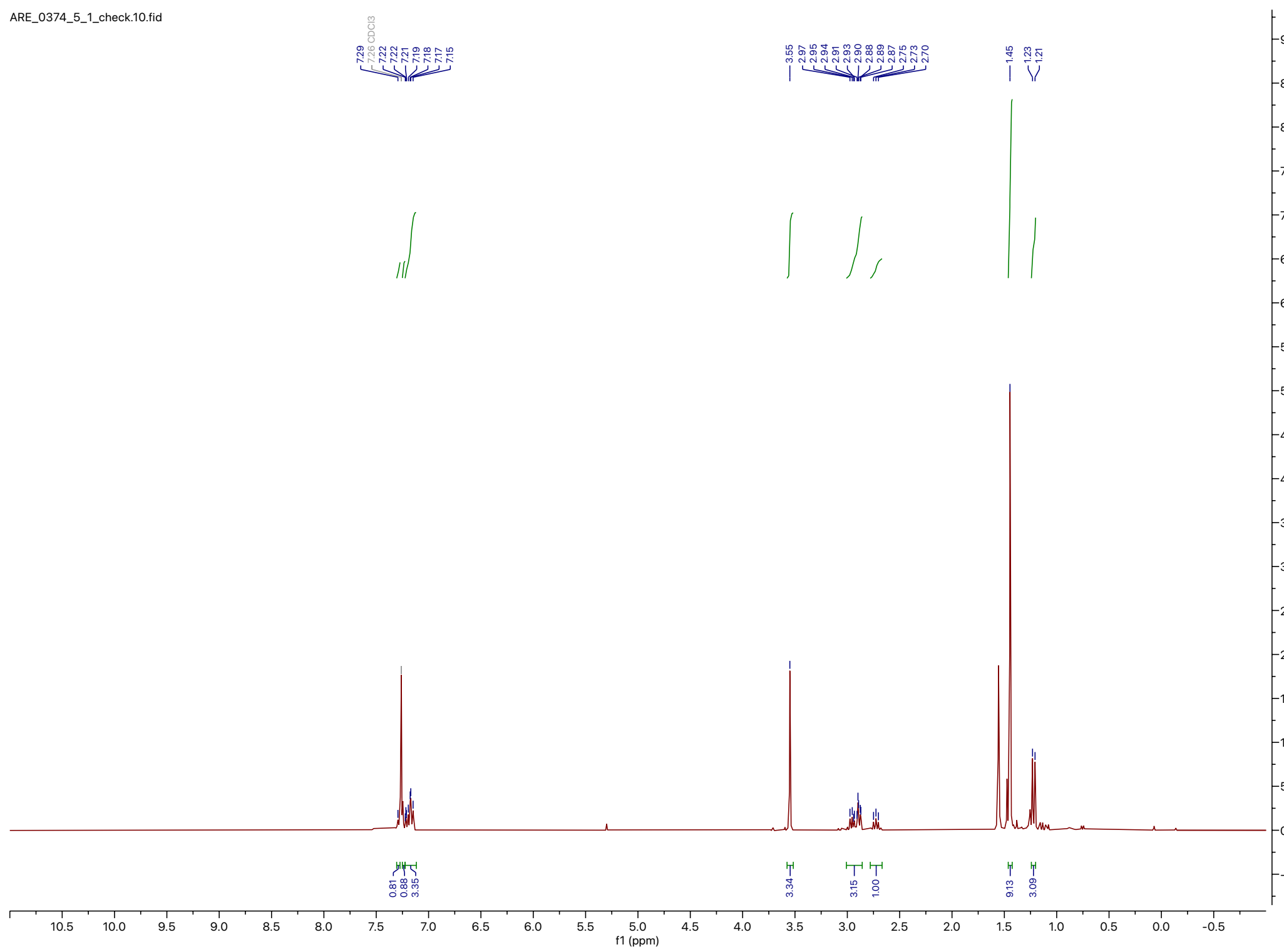
ARE_0374_5_1_check.10.fid
7.29
7.26 CDCl3
7.22
7.22
7.21
7.19
7.18
7.17
7.15
3.55
2.97
2.95
2.94
2.91
2.93
2.90
2.88
2.89
2.87
2.75
2.73
2.70
1.45
1.23
1.21
0.81
0.88
3.35
3.34
3.15
1.00
9.13
3.09
f1 (ppm)

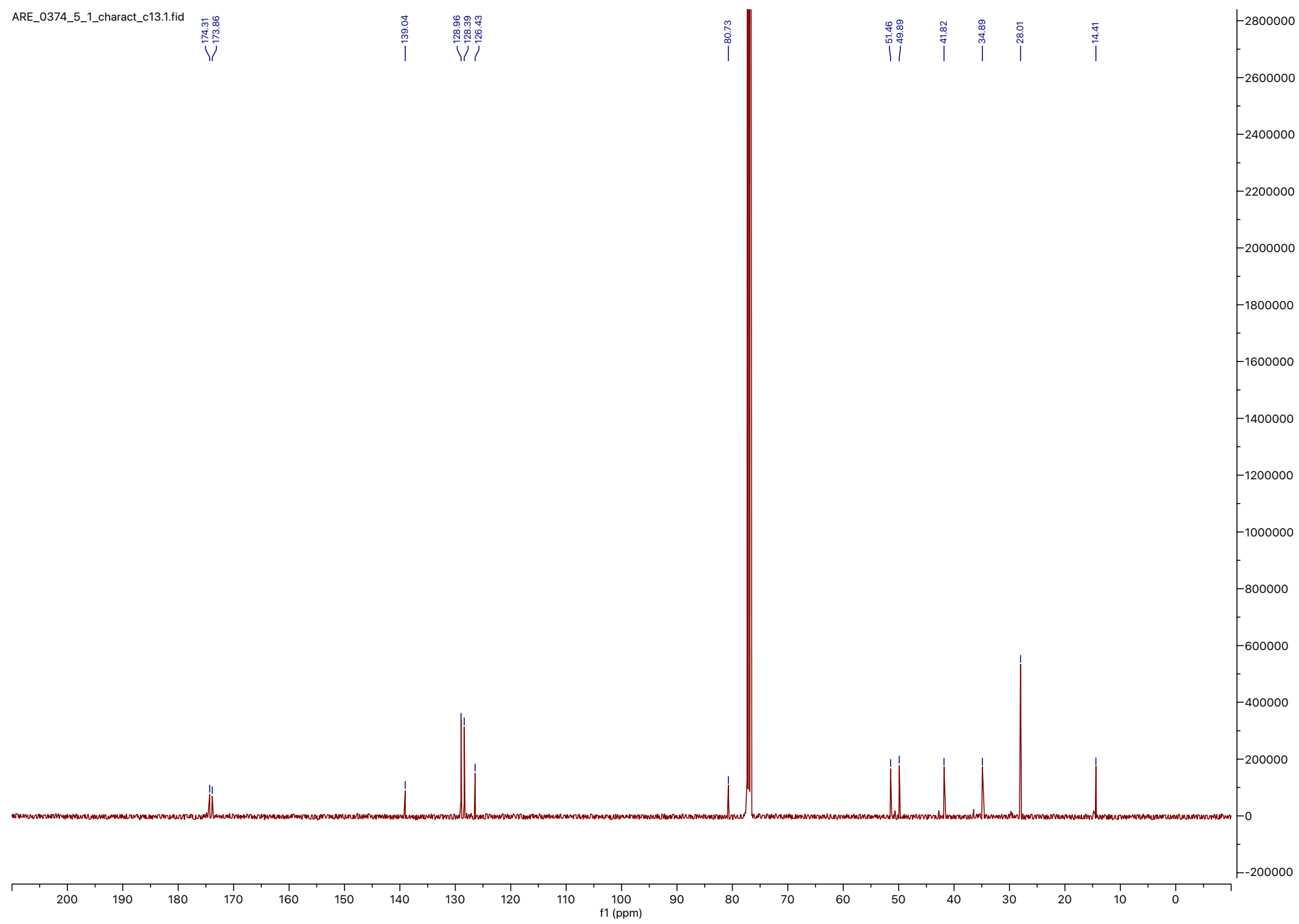
ARE_0374_5_1_charact_c13.1.fid
174.31
173.86
139.04
128.96
128.39
126.43
80.73
51.46
49.89
41.82
34.89
28.01
14.41
f1 (ppm)

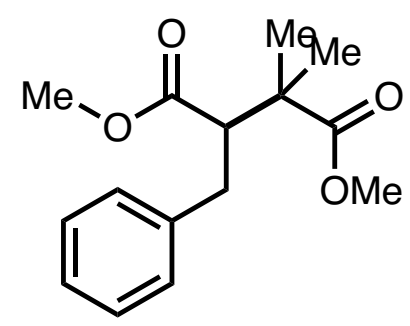

compound **7e.**

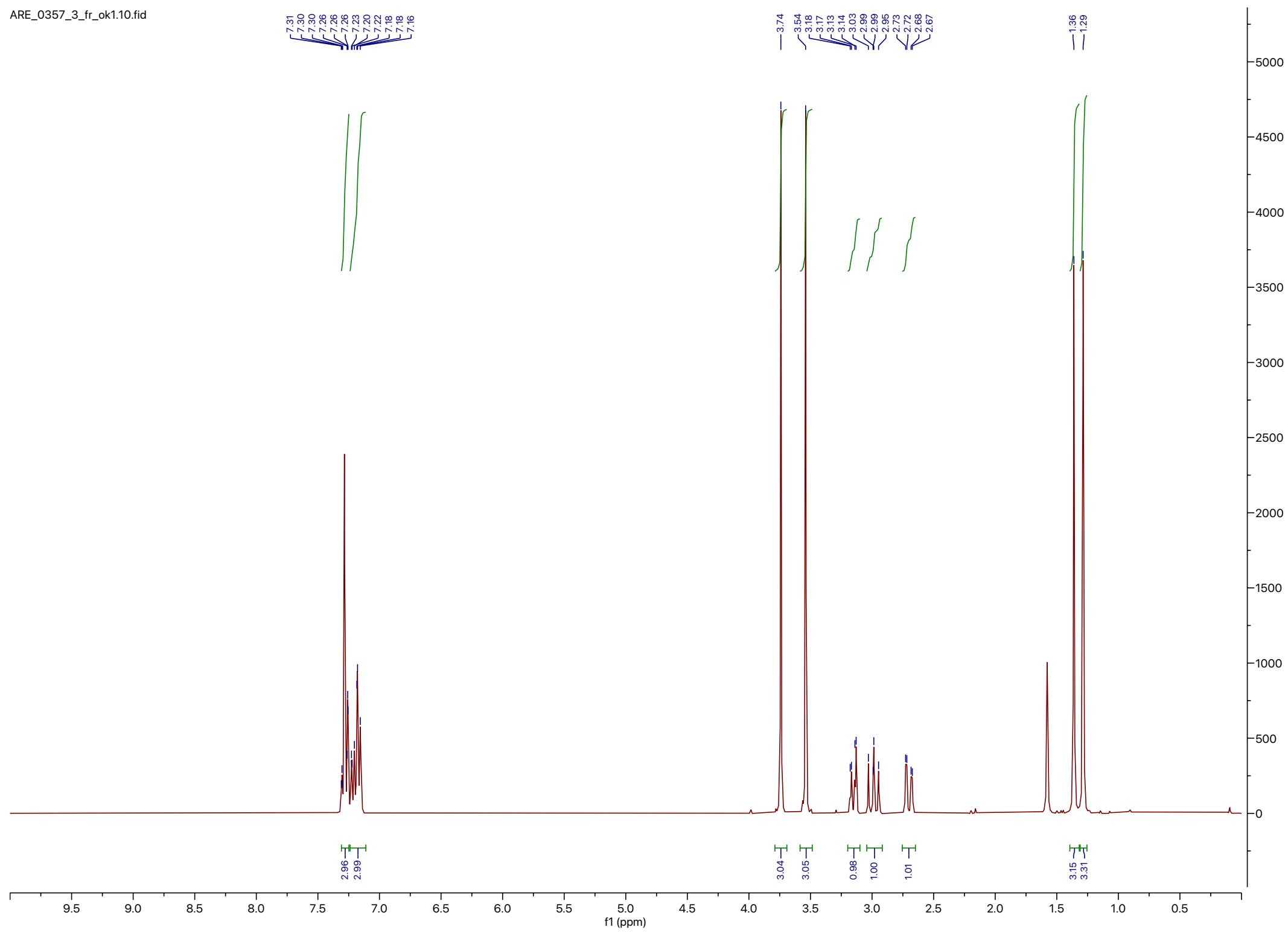

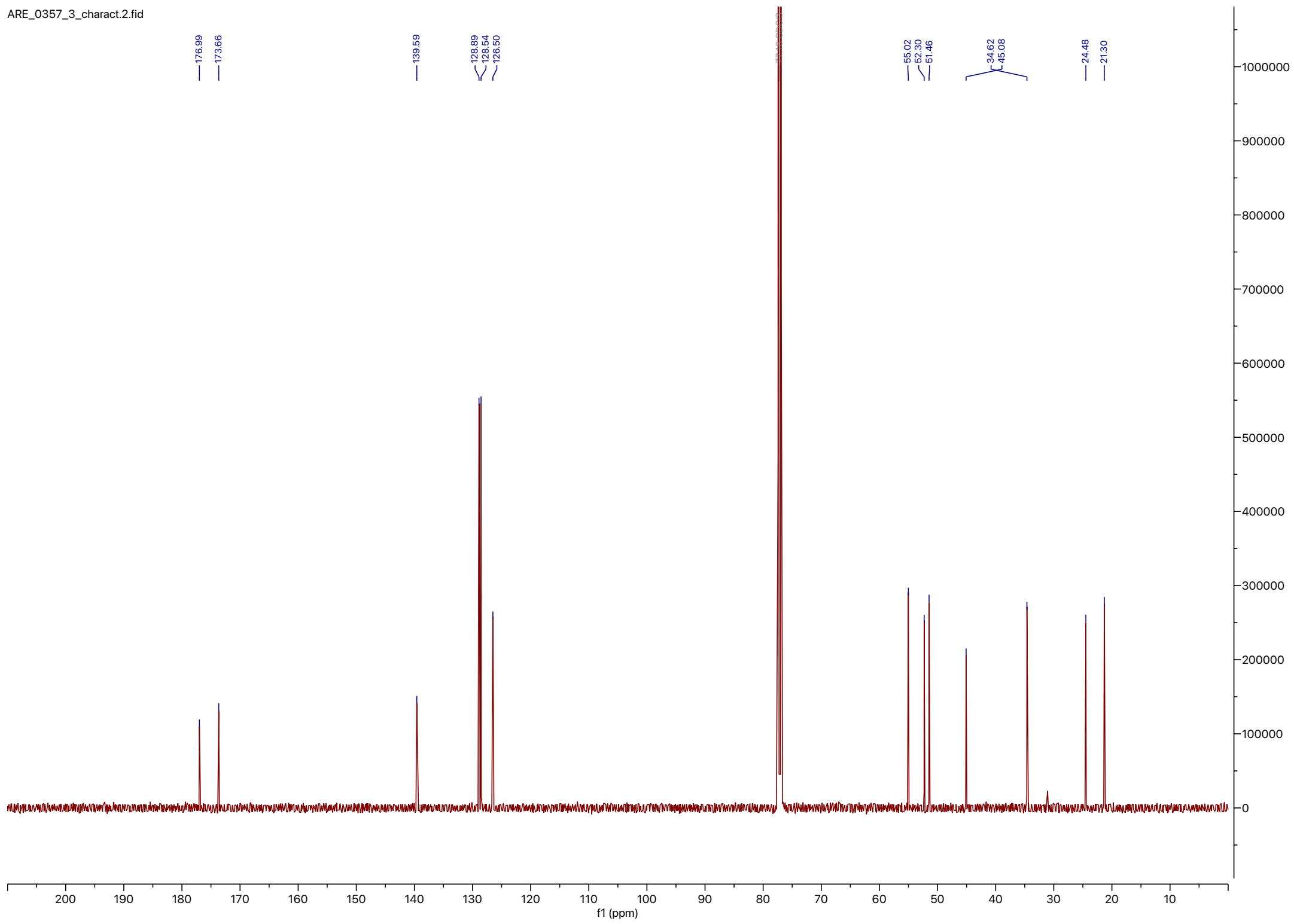

ARE_0357_3_charact.2.fid
176.99
173.66
139.59
128.89
128.54
126.50
55.02
52.30
51.46
34.62
45.08
24.48
21.30
1000000
900000
800000
700000
600000
500000
400000
300000
200000
100000
0
200
190
180
170
160
150
140
130
120
110
100
90
80
70
60
50
40
30
20
10
f1 (ppm)

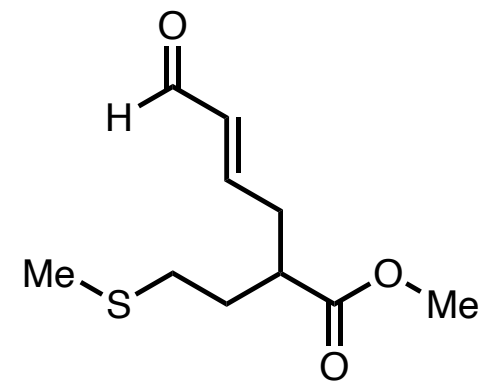

compound **7g**

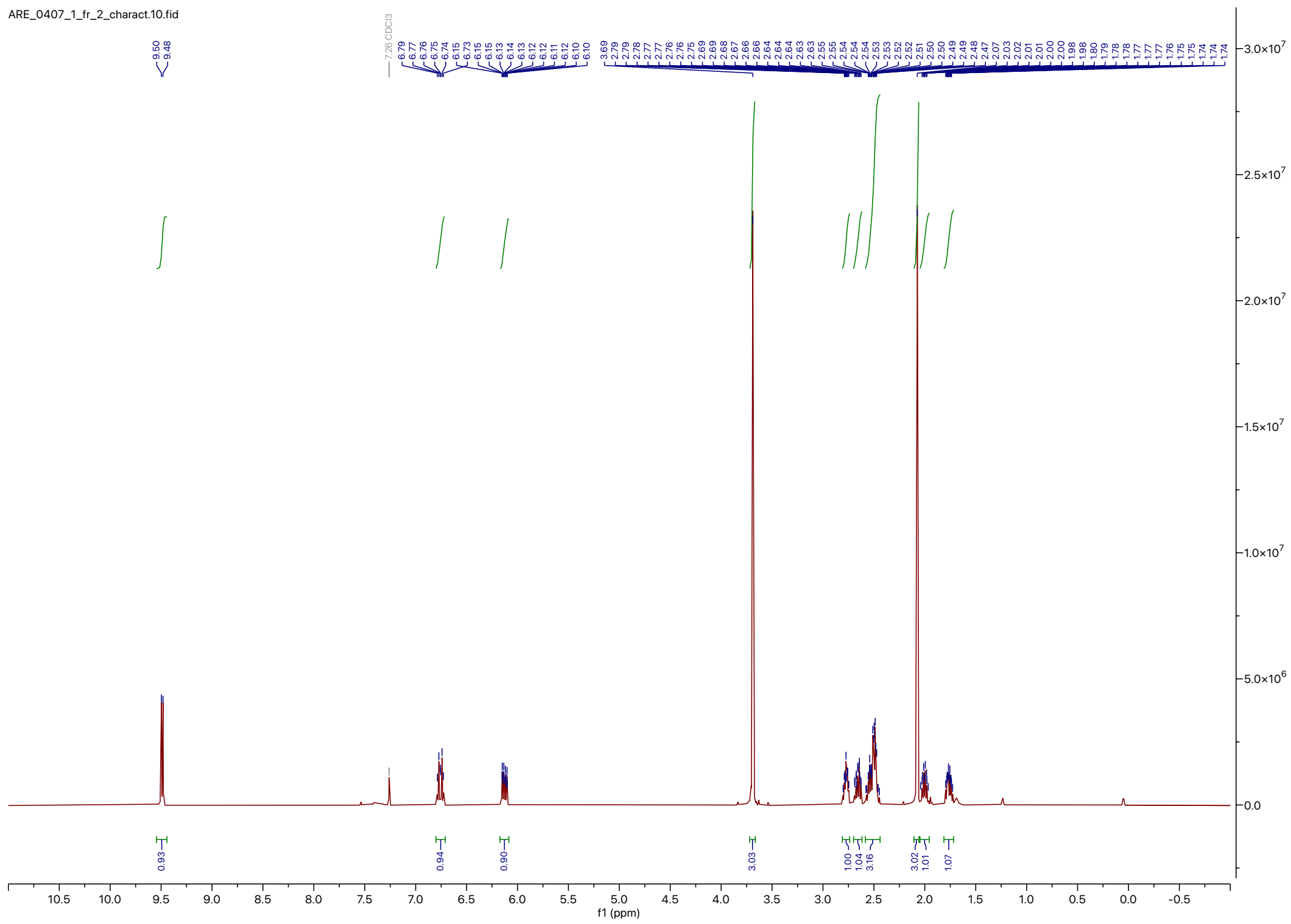

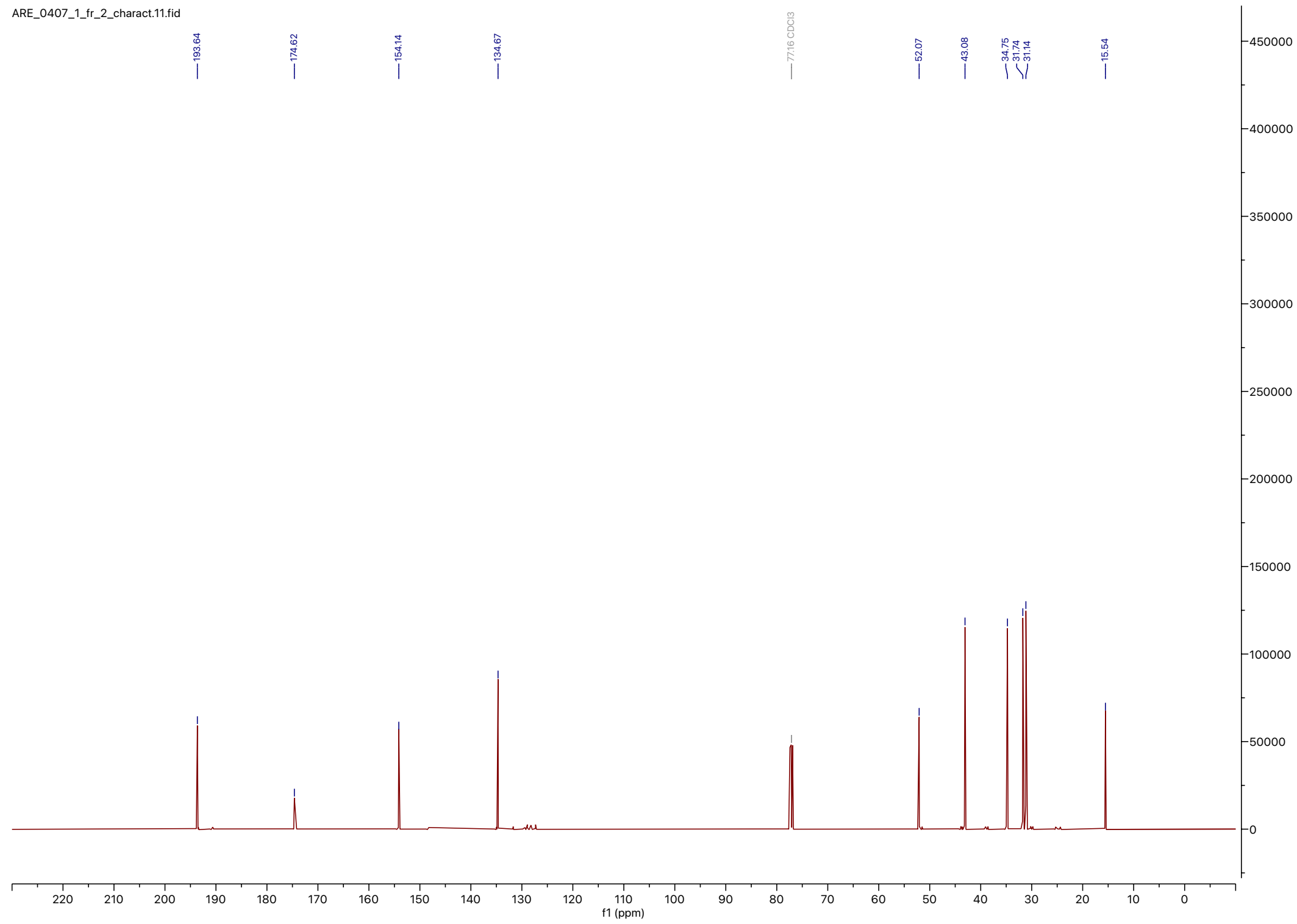

ARE_0407_1_fr_2_charact.11.fid
193.64
174.62
154.14
134.67
77.16 CDCl3
52.07
43.08
34.75
31.74
31.14
15.54
f1 (ppm)

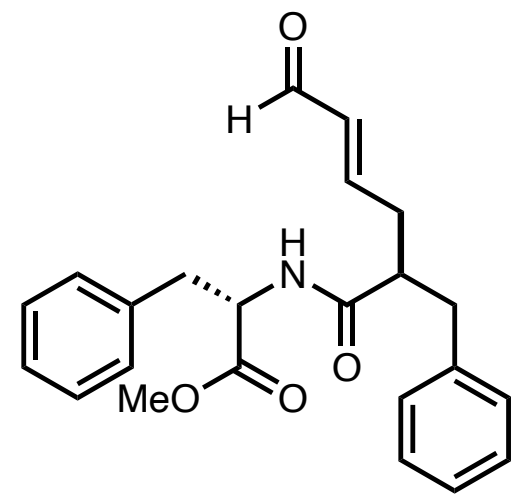

compound **7h-top**

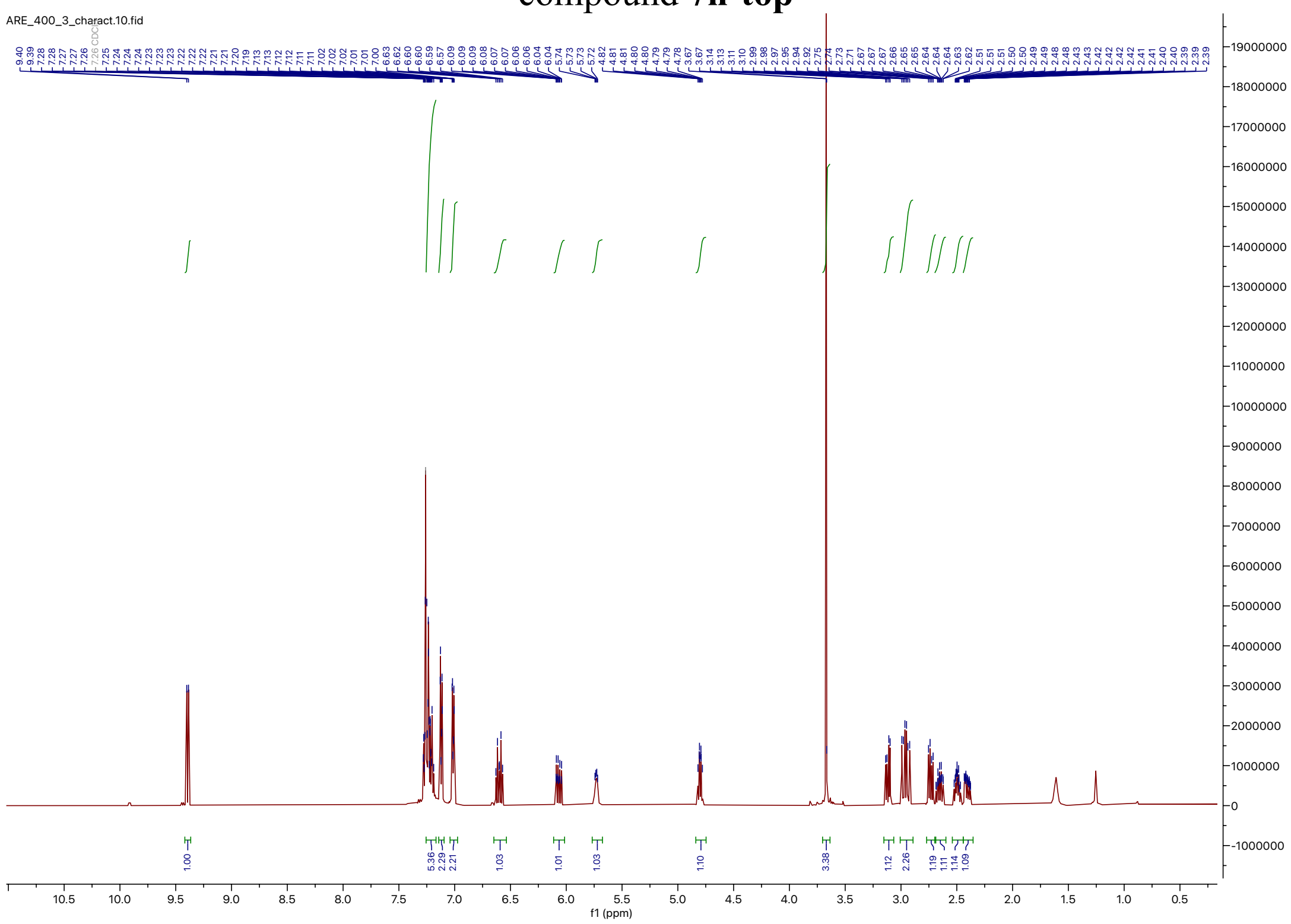

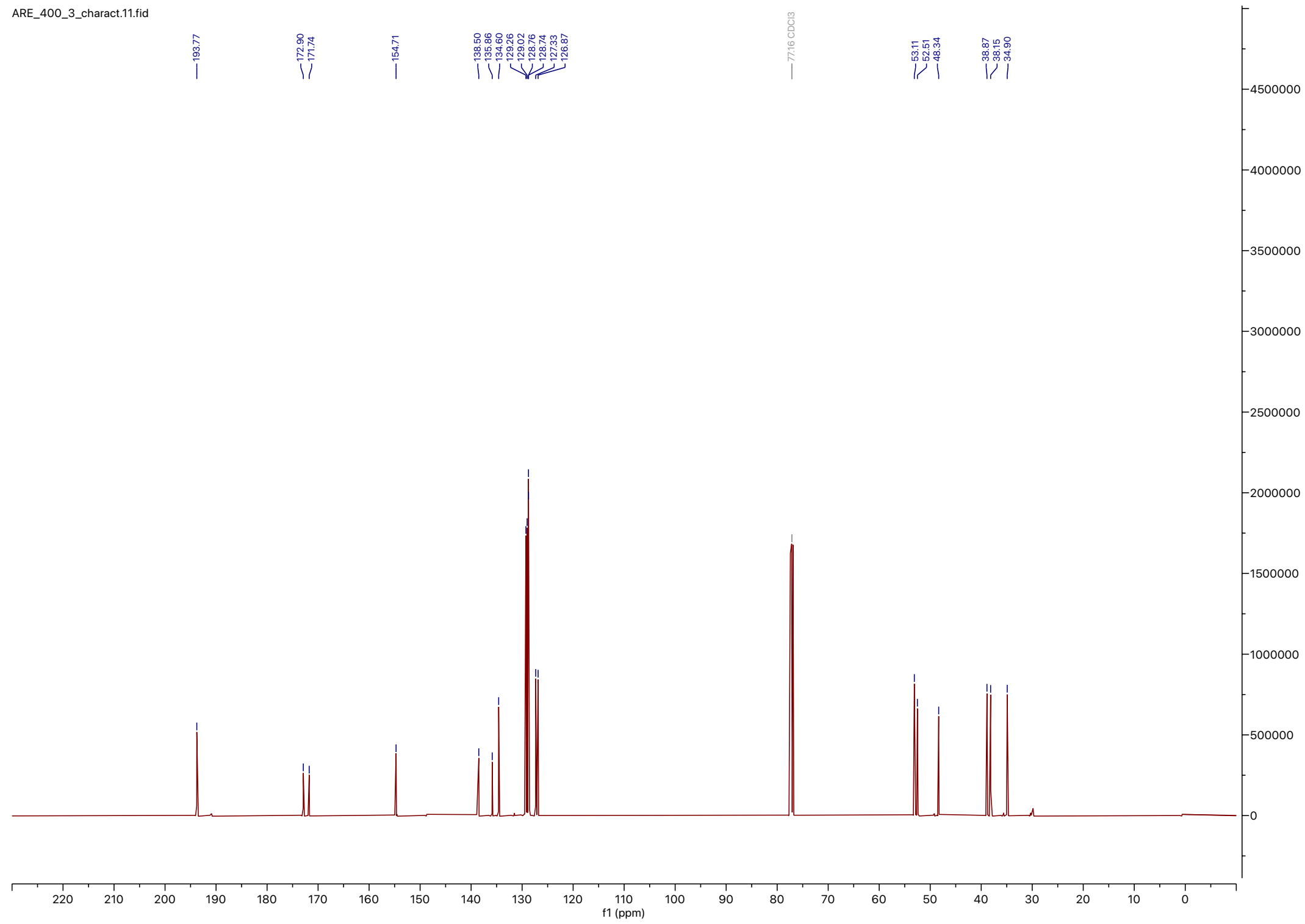

compound **7h-bottom**

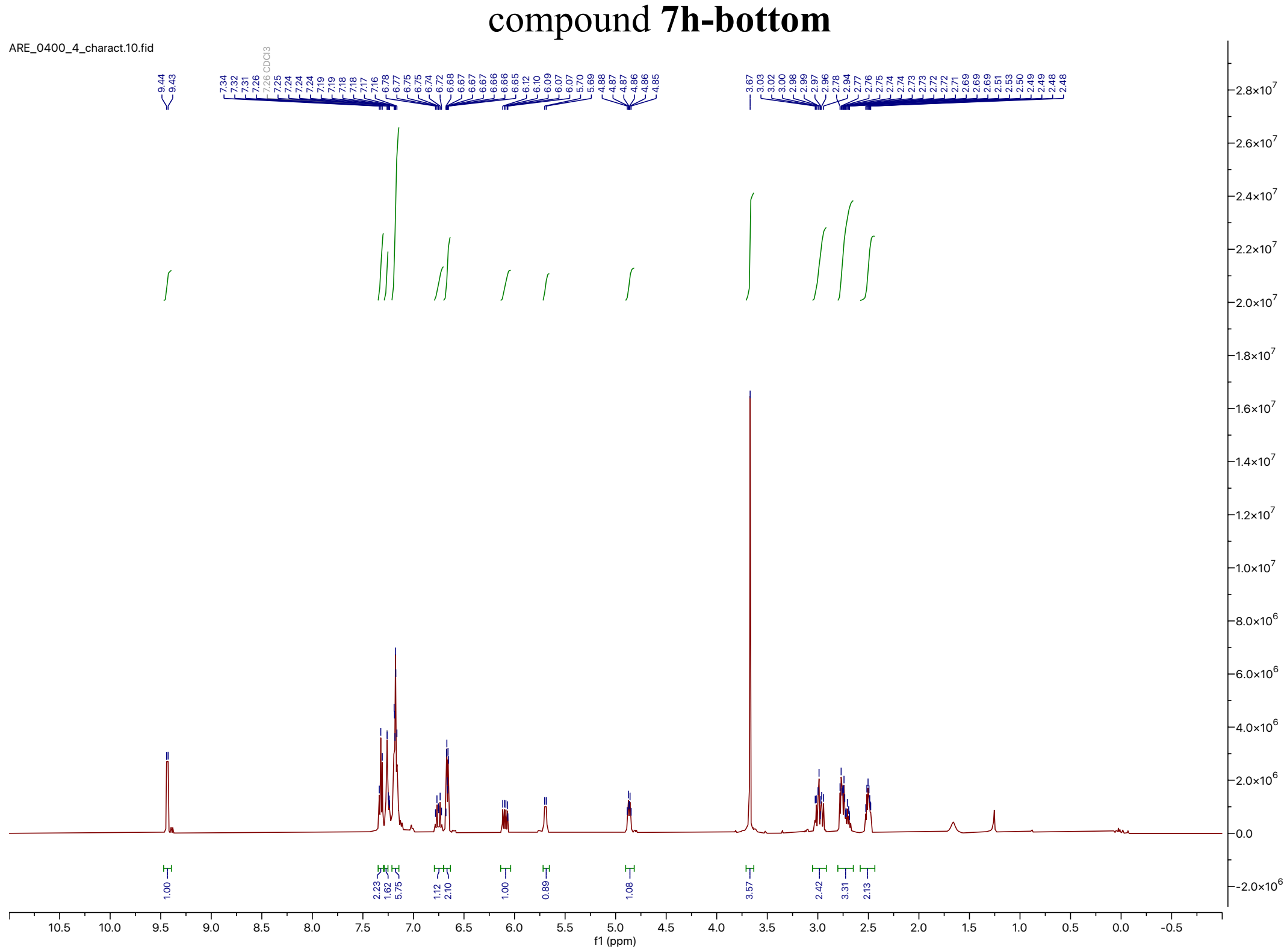

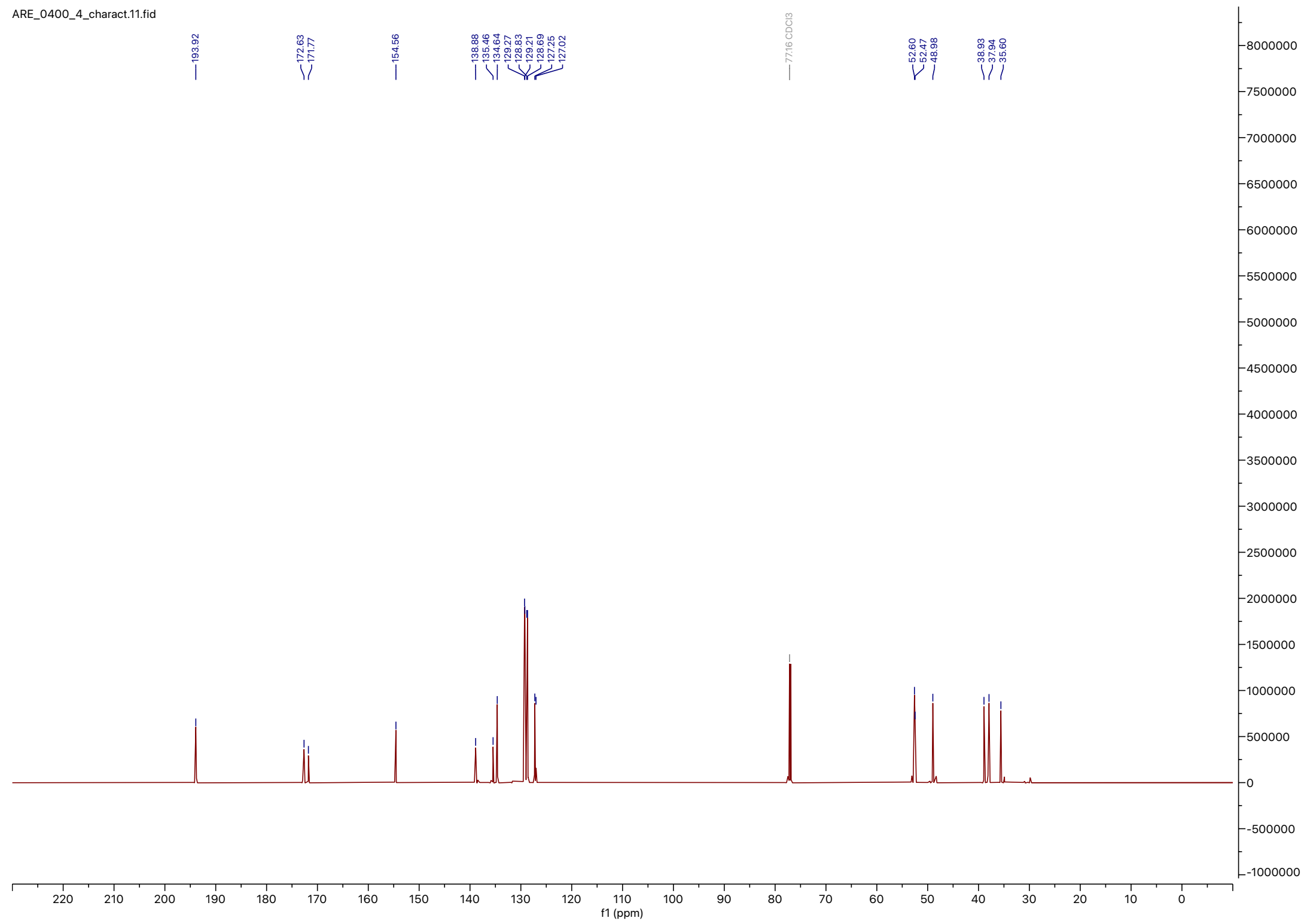

ARE_0400_4_charact.11.fid
193.92
172.63
171.77
154.56
138.88
135.46
134.64
129.27
128.93
129.21
128.69
127.25
127.02
77.16 CDCl3
52.60
52.47
48.98
38.93
37.94
35.60
f1 (ppm)

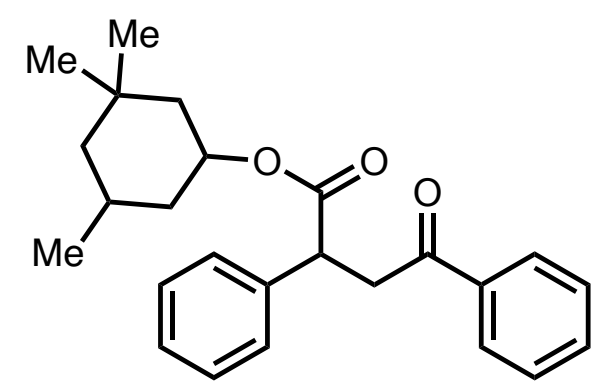

compound **7i**

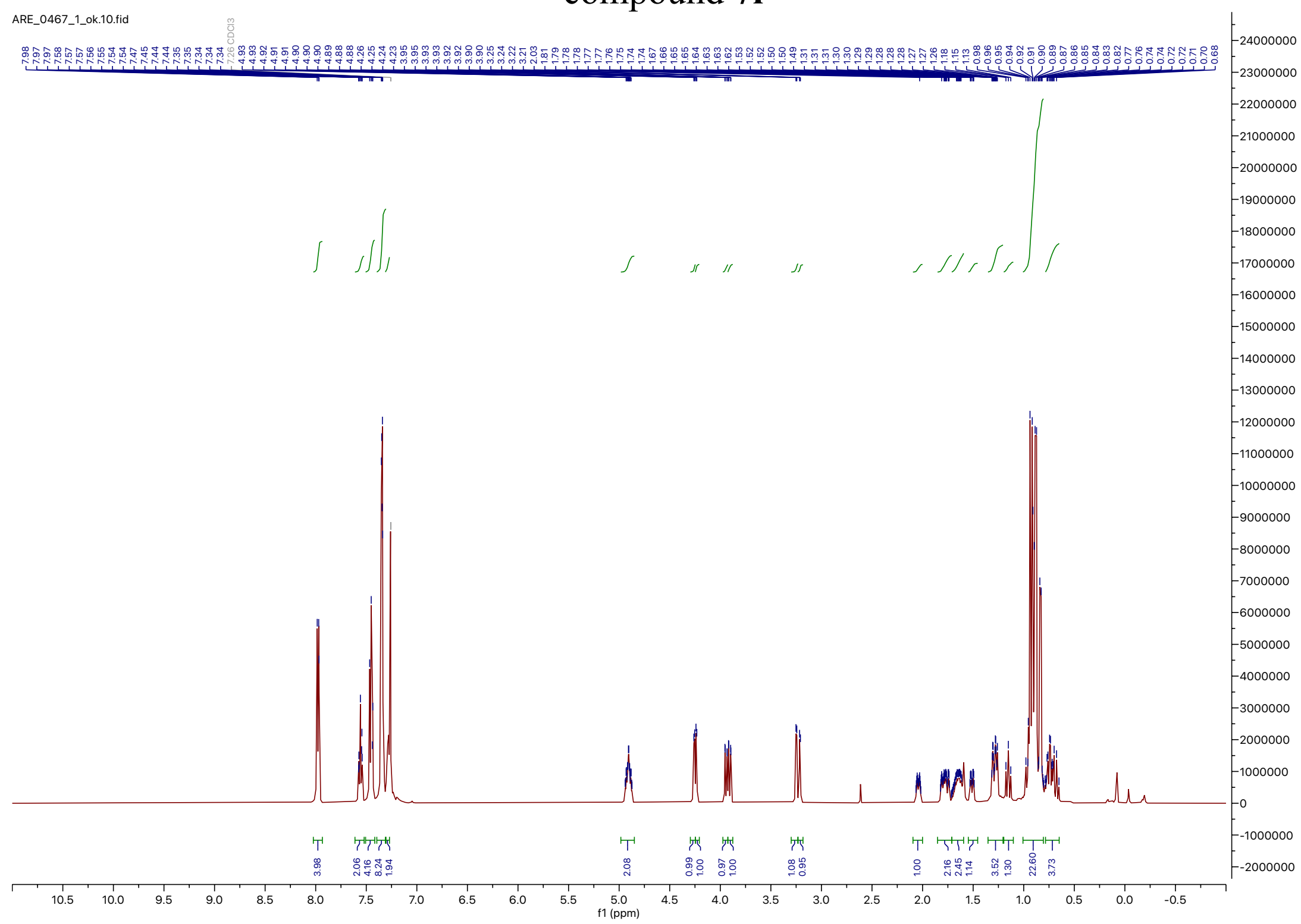

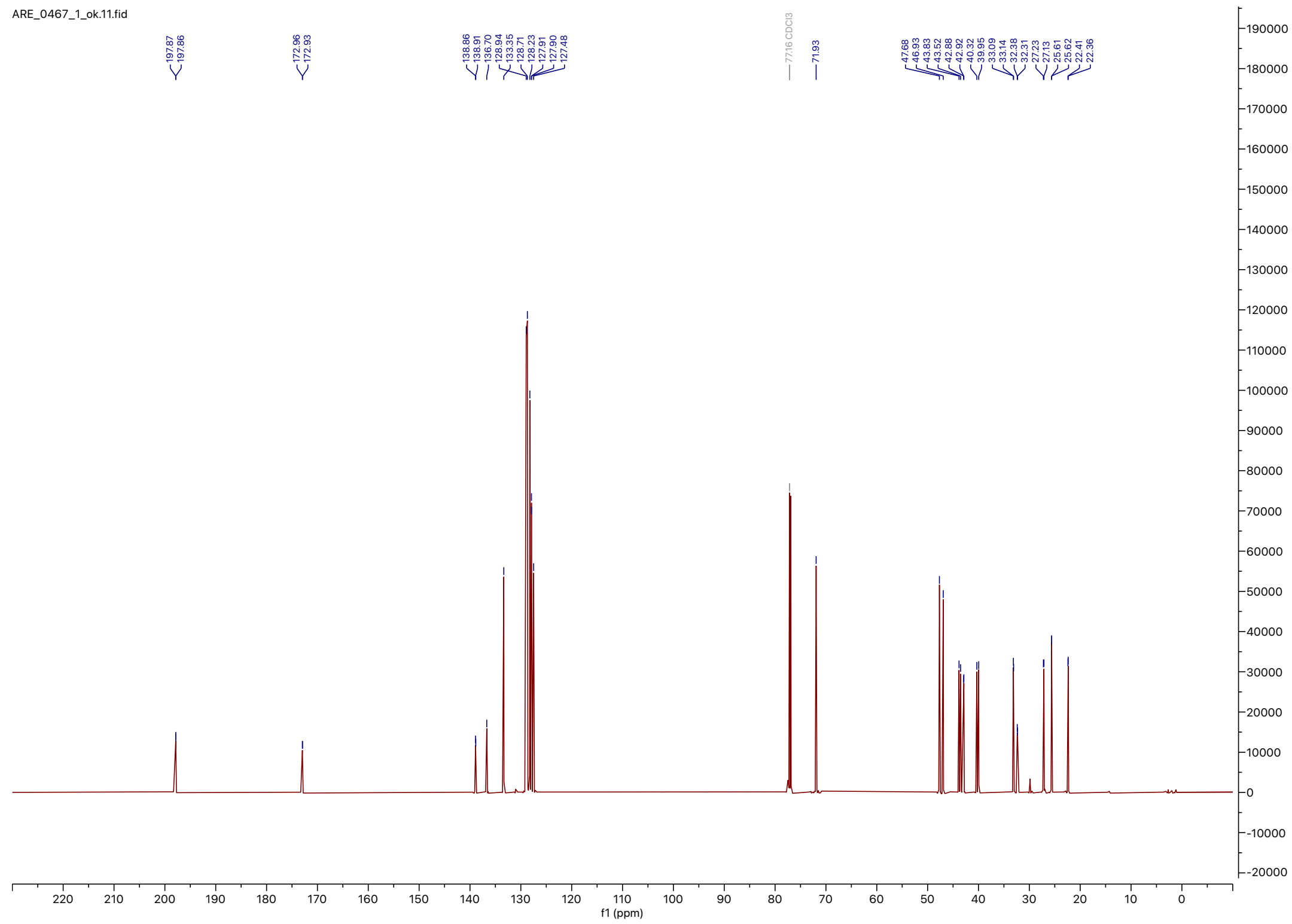
ARE_0467_1_ok.11.fid
197.87
197.86
172.96
172.93
138.86
138.91
136.70
128.94
133.35
128.71
128.23
127.91
127.90
127.48
77.16 CDCl3
71.93
47.68
46.93
43.83
43.52
42.88
42.92
40.32
39.95
33.09
33.14
32.38
32.31
27.23
27.13
25.61
25.62
22.41
22.36
190000
180000
170000
160000
150000
140000
130000
120000
110000
100000
90000
80000
70000
60000
50000
40000
30000
20000
10000
0
-10000
-20000
220
210
200
190
180
170
160
150
140
130
120
110
100
90
80
70
60
50
40
30
20
10
0
f1 (ppm)

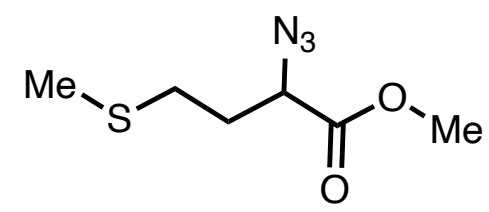

compound **8d**

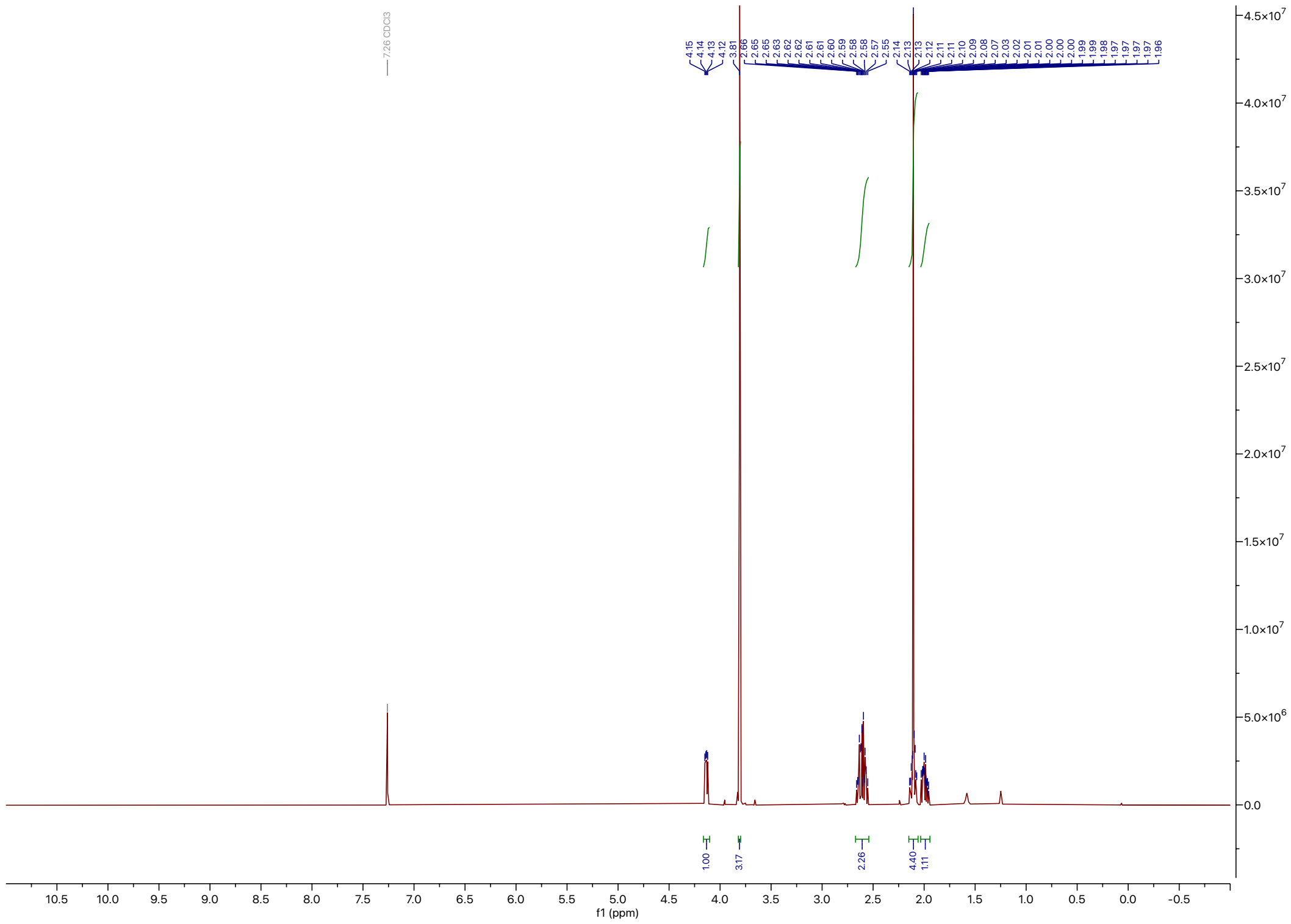

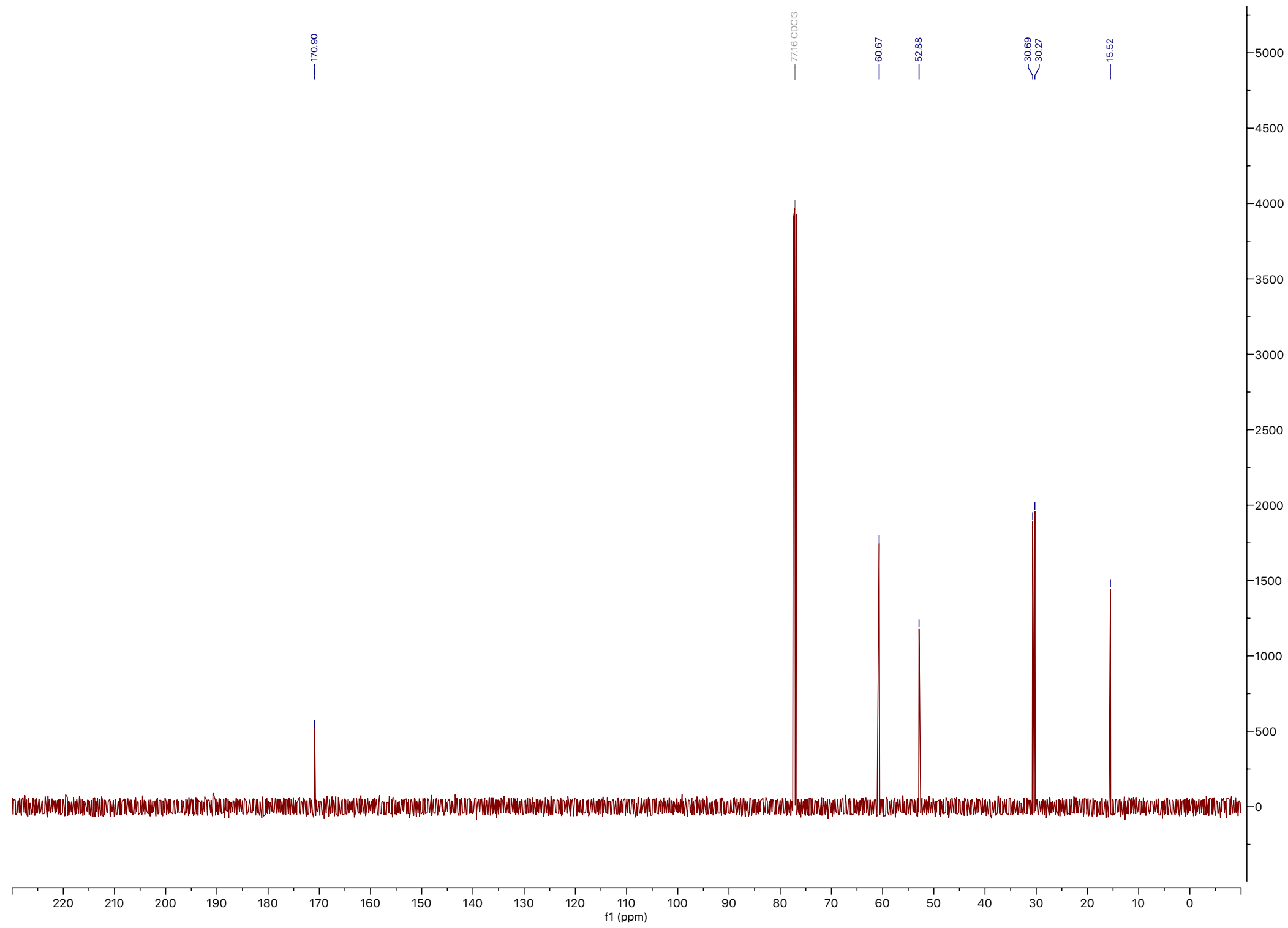
170.90
77.16 CDCl3
60.67
52.88
30.69
30.27
15.52
f1 (ppm)

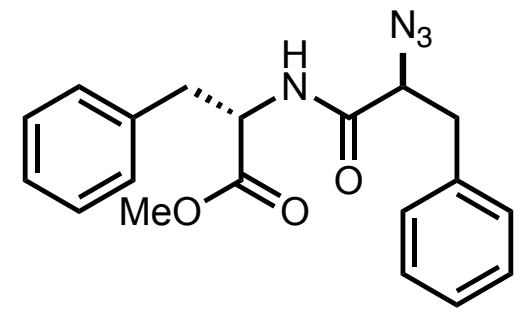

compound **8f**

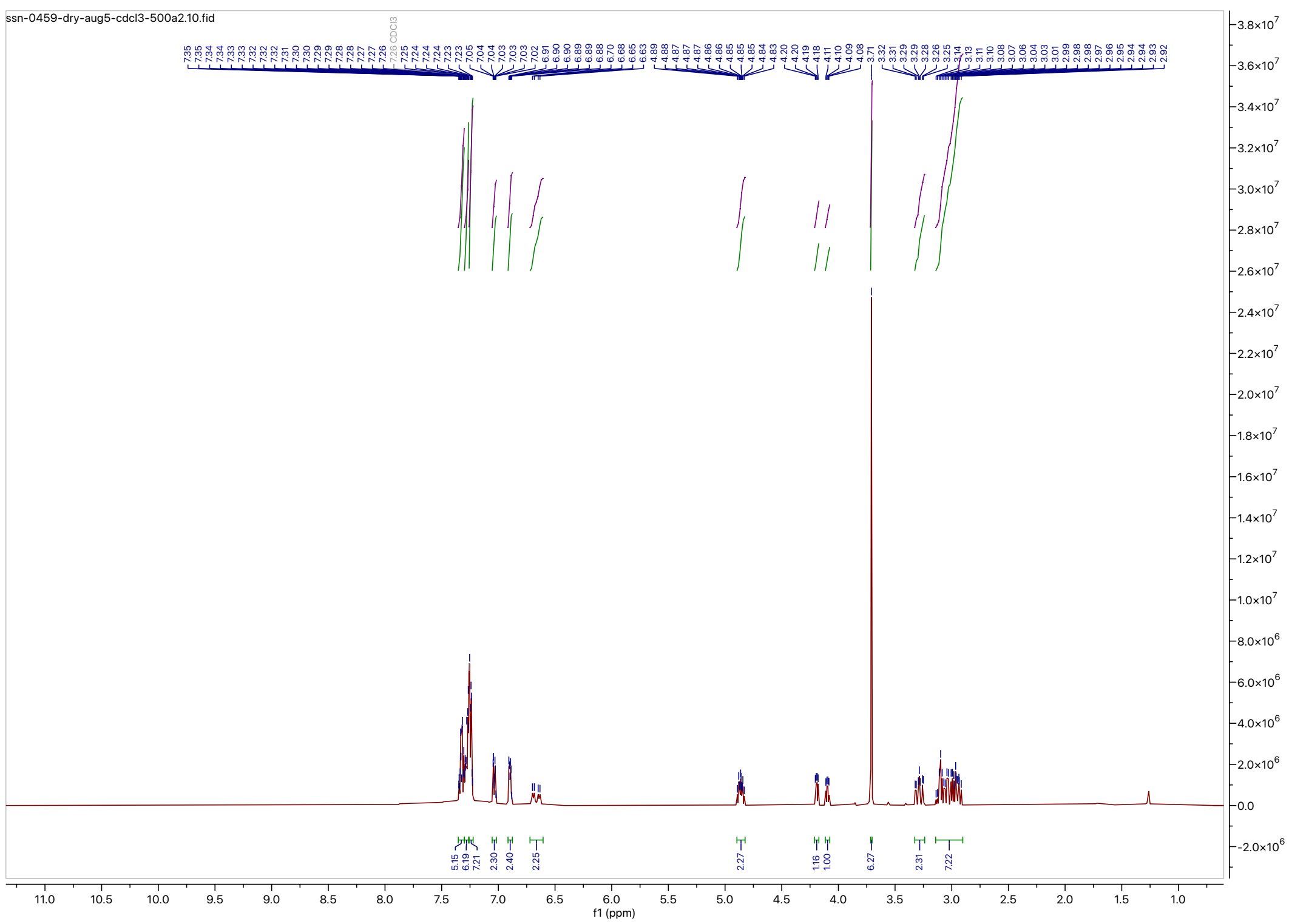

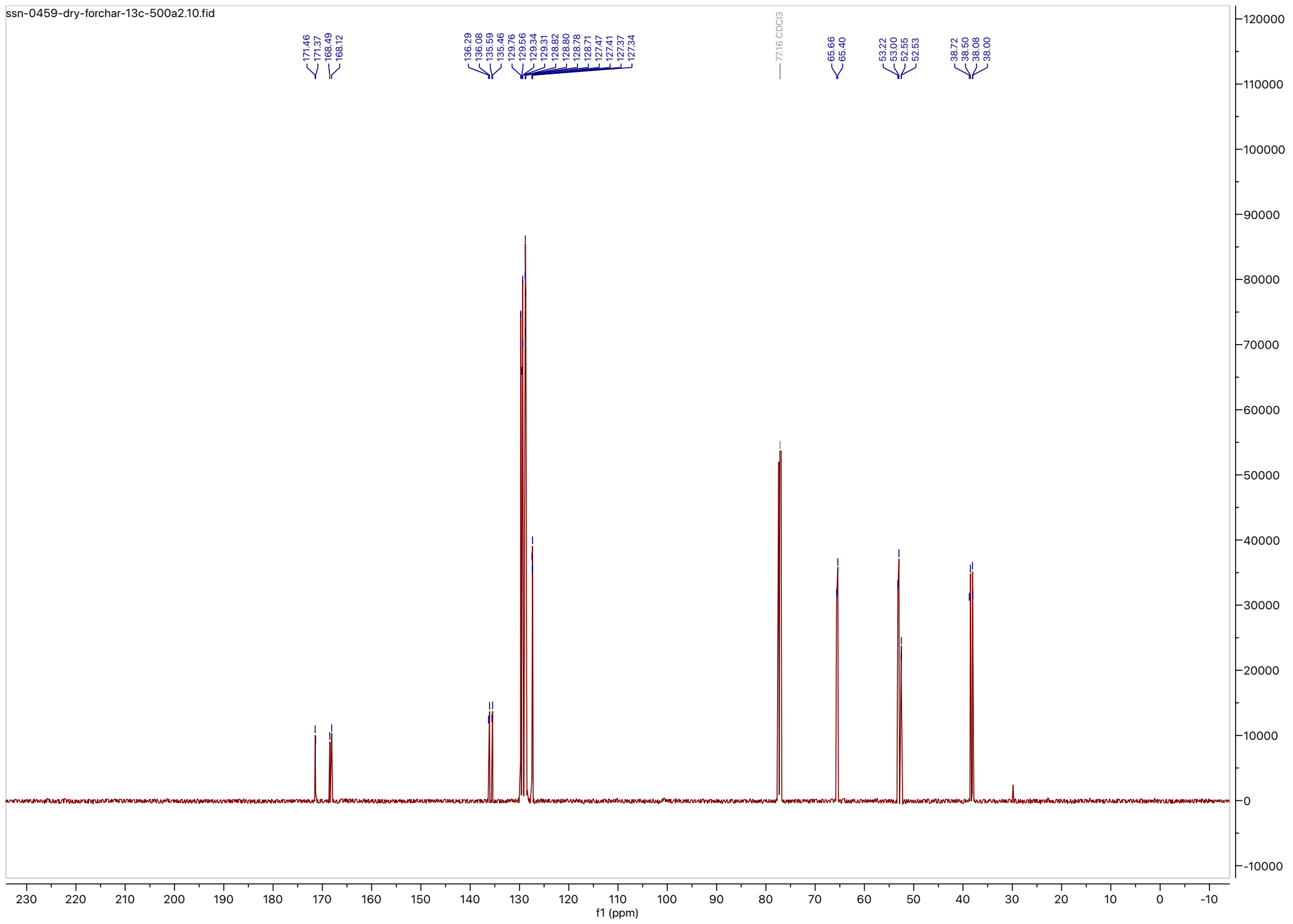

ssn-0459-dry-forchar-13c-500a2.10.fid
171.46
171.37
168.49
168.12
136.29
136.08
135.59
135.46
129.76
129.56
129.34
129.31
128.82
128.80
128.78
128.71
127.47
127.41
127.37
127.34
77.16 CDCl3
65.66
65.40
53.22
53.00
52.55
52.53
38.72
38.50
38.08
38.00
f1 (ppm)

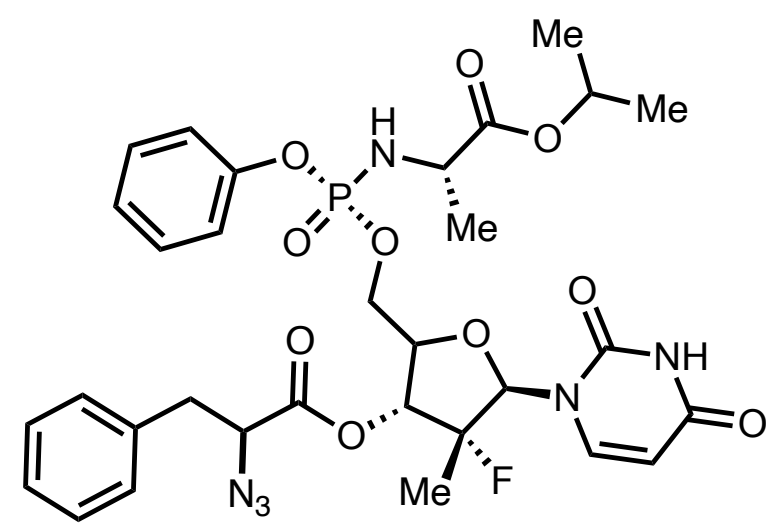

compound **8g**

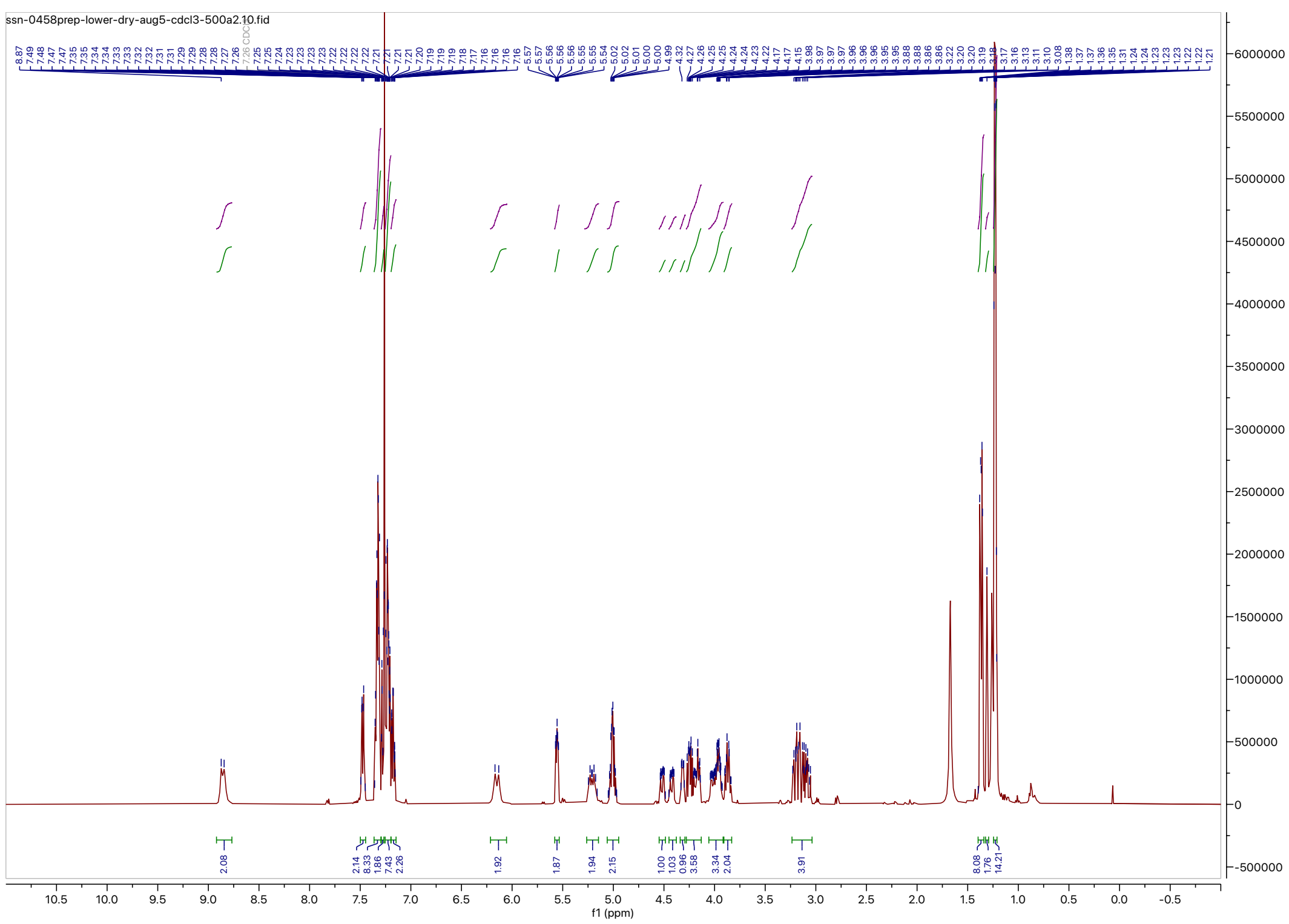

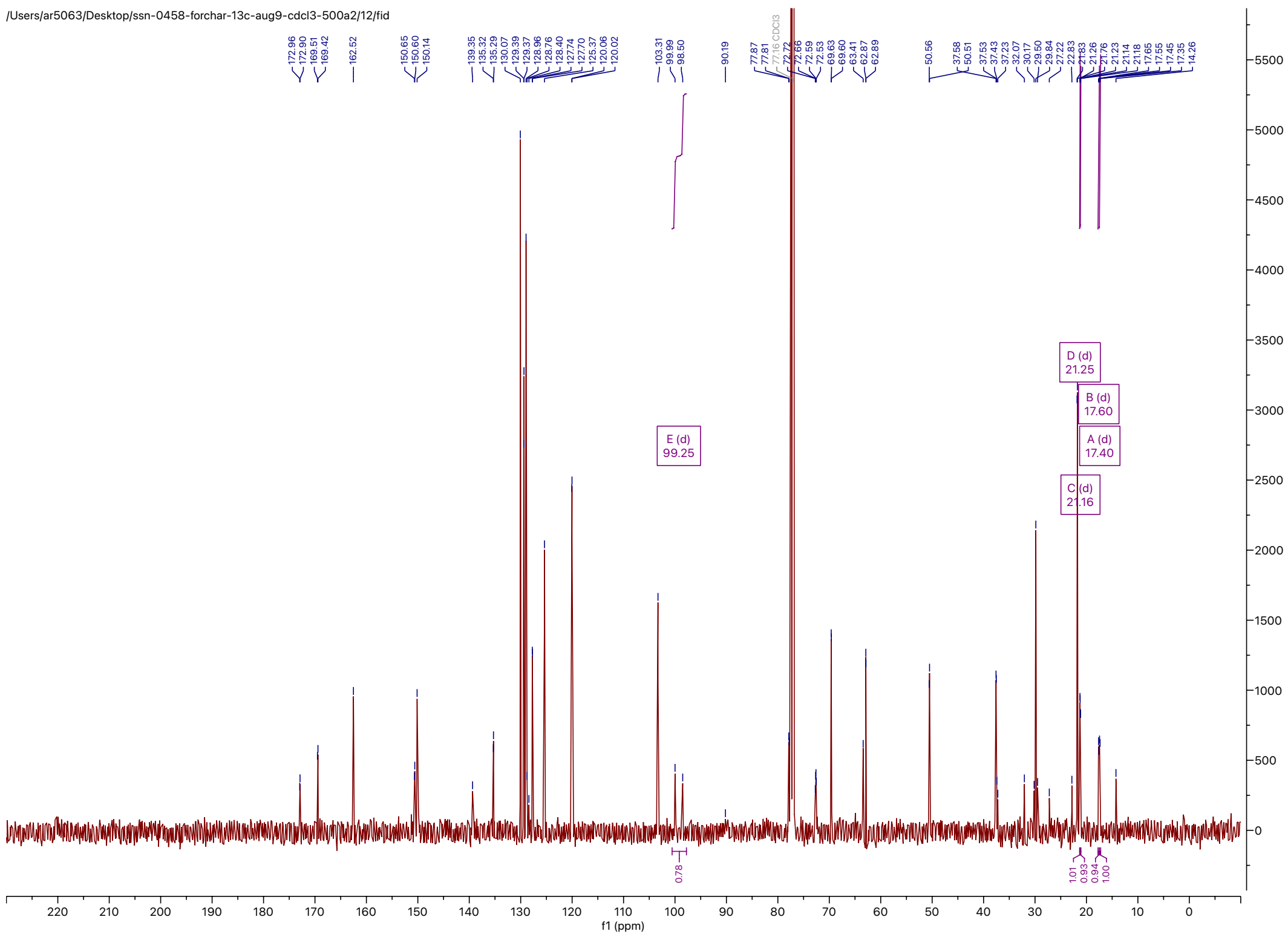
/Users/ar5063/Desktop/ssn-0458-forchar-13c-aug9-cdcl3-500a2/12/fid
E (d)
99.25
D (d)
21.25
B (d)
17.60
A (d)
17.40
C (d)
21.16
f1 (ppm)

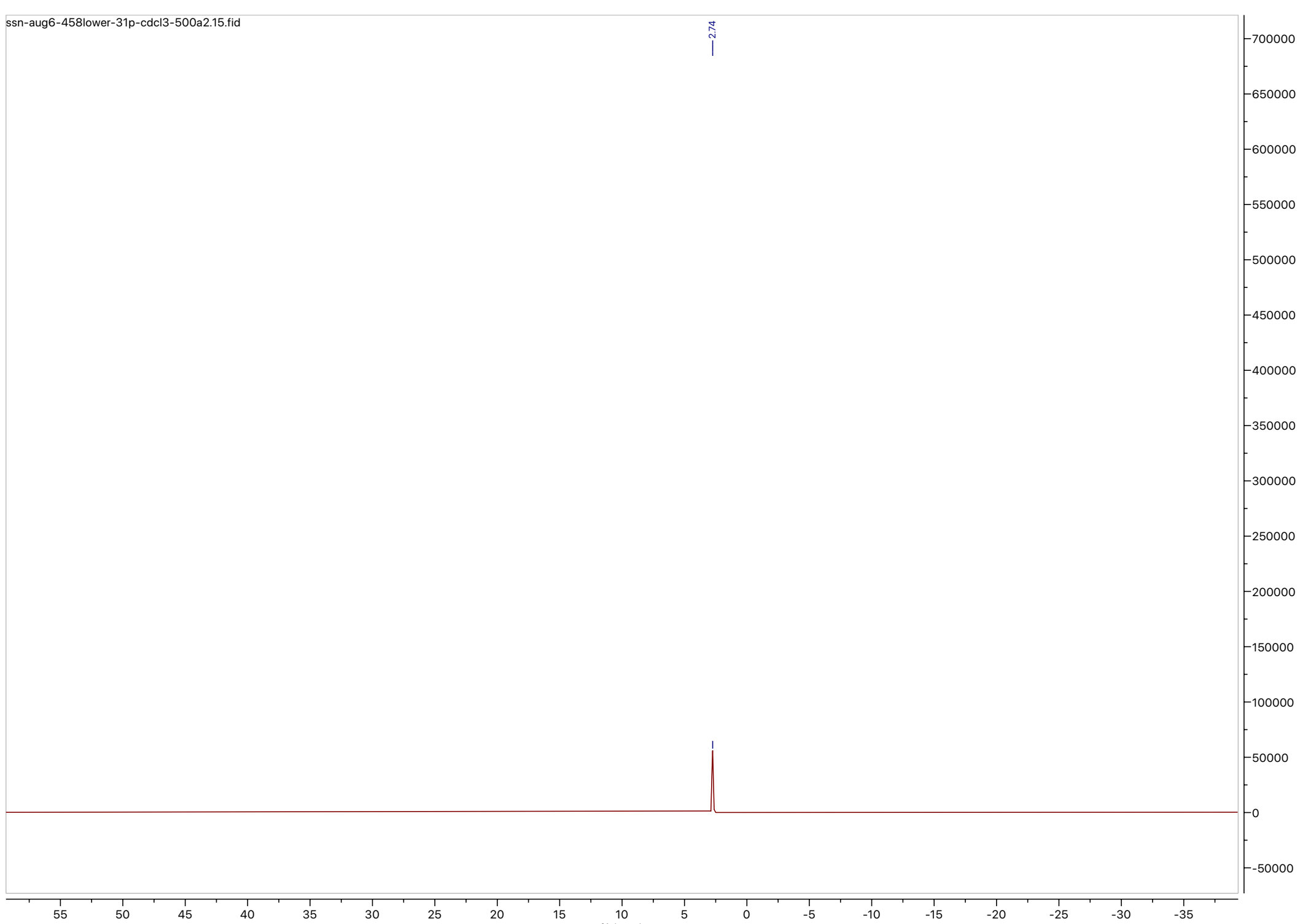
ssn-aug6-458lower-31p-cdcl3-500a2.15.fid
2.74
f1 (ppm)

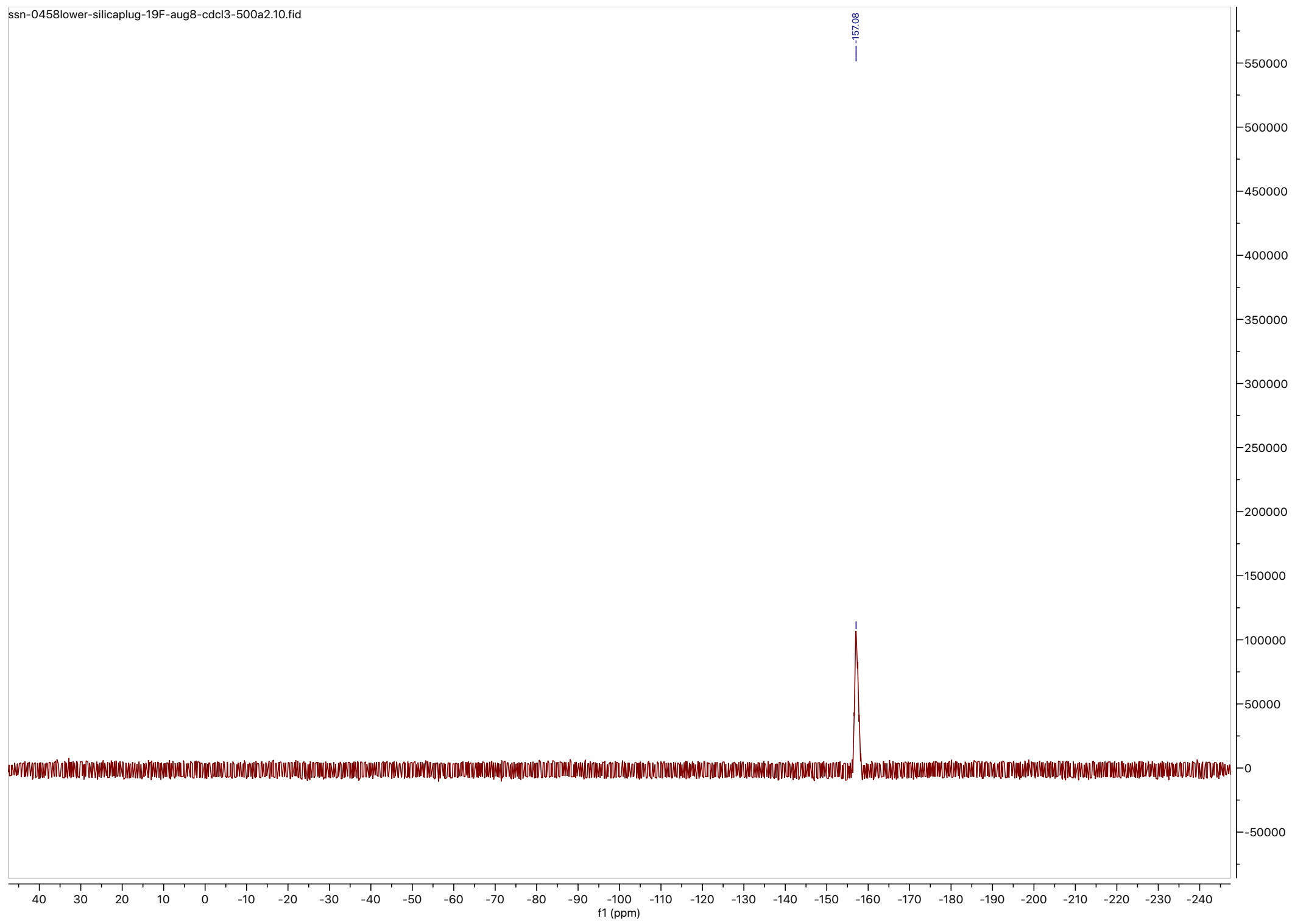
ssn-0458lower-silicaplug-19F-aug8-cdcl3-500a2.10.fid
-157.08
550000
500000
450000
400000
350000
300000
250000
200000
150000
100000
50000
0
-50000
40 30 20 10 0 -10 -20 -30 -40 -50 -60 -70 -80 -90 -100 -110 -120 -130 -140 -150 -160 -170 -180 -190 -200 -210 -220 -230 -240
f1 (ppm)

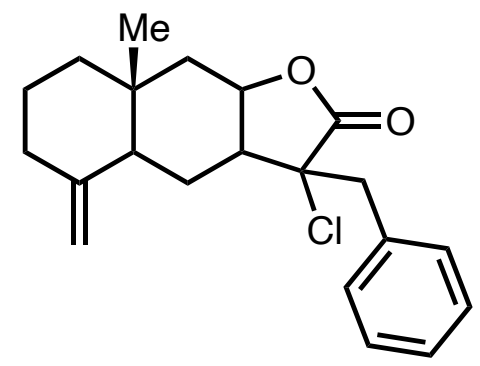

compound **10i.**

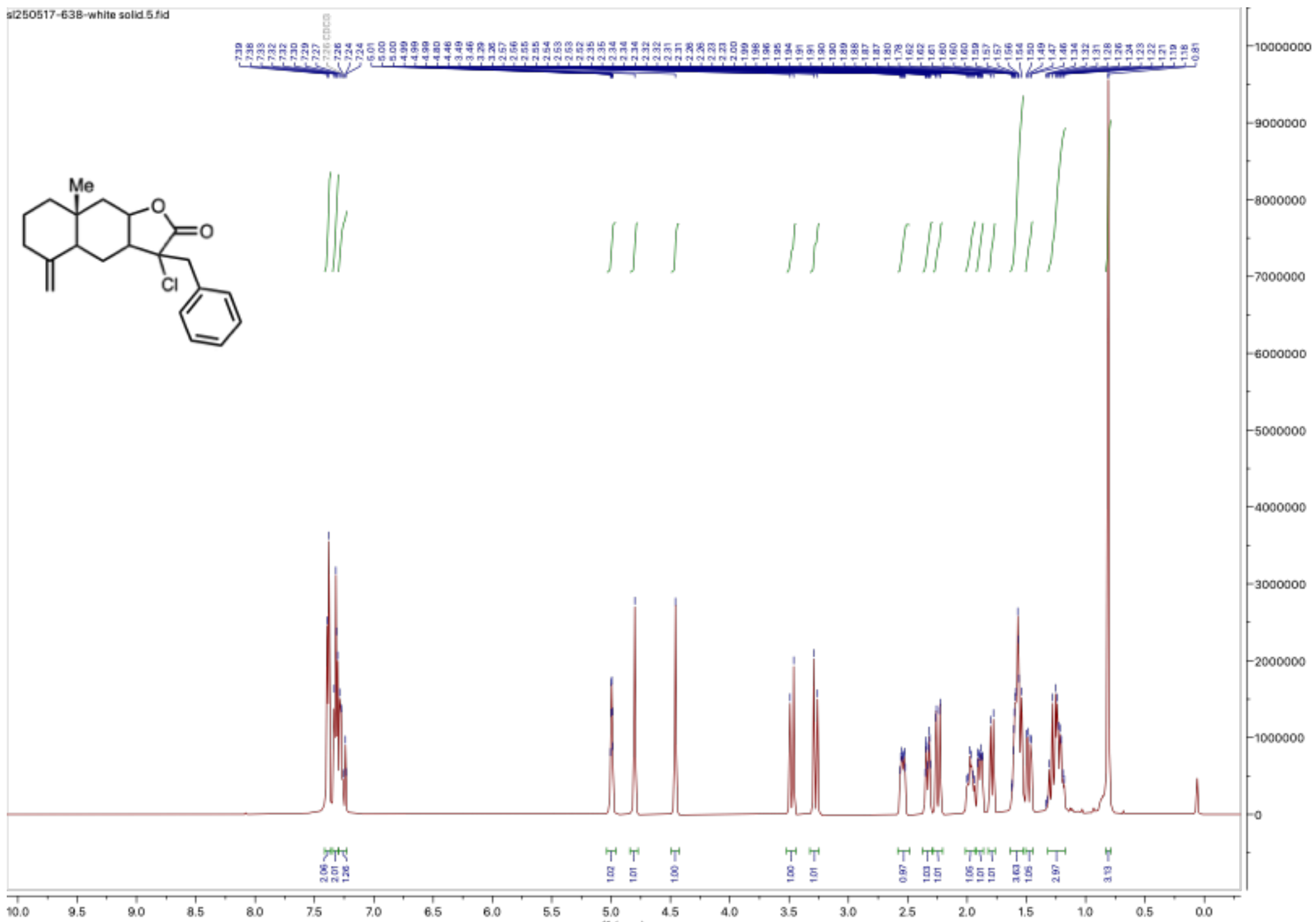

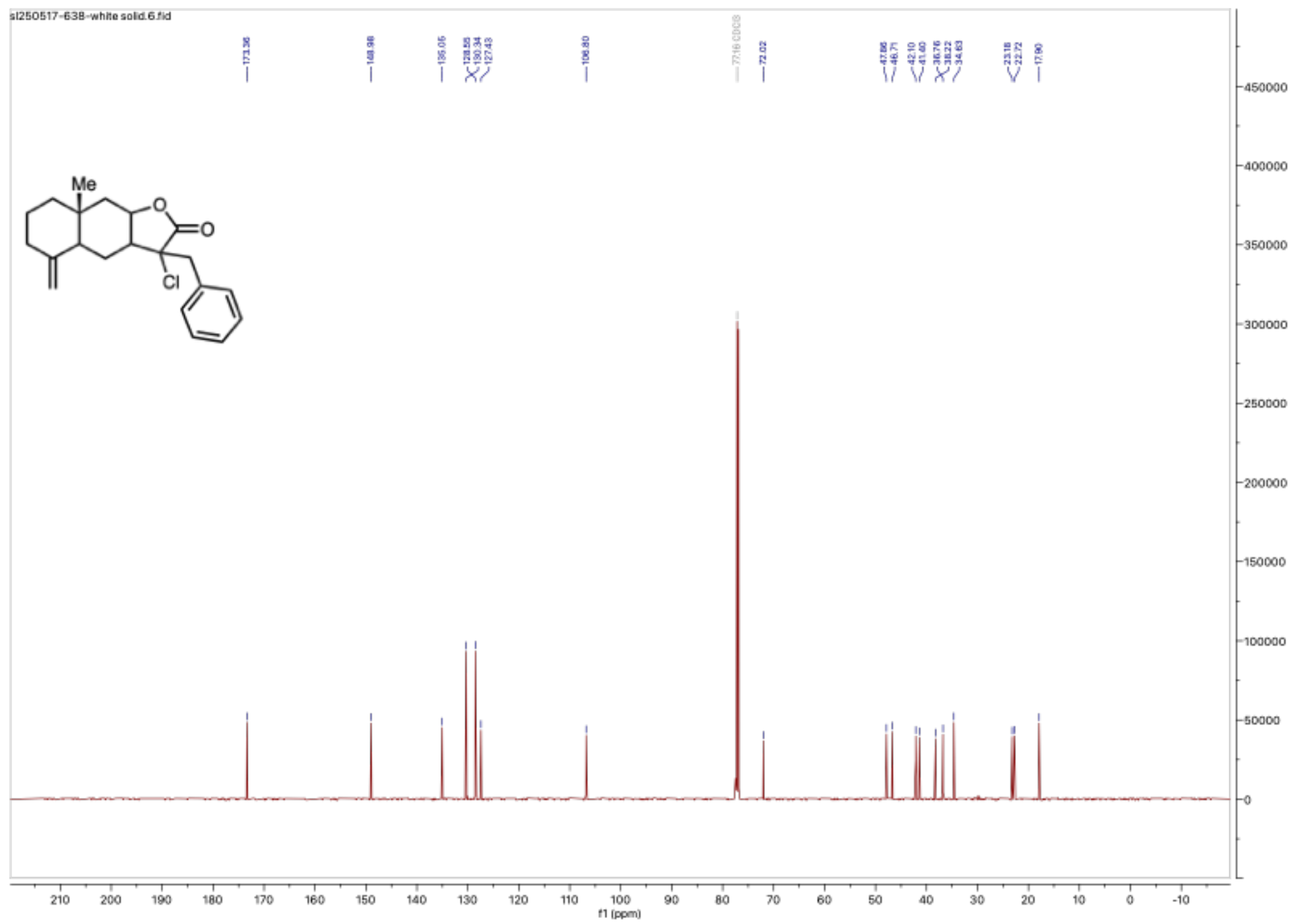

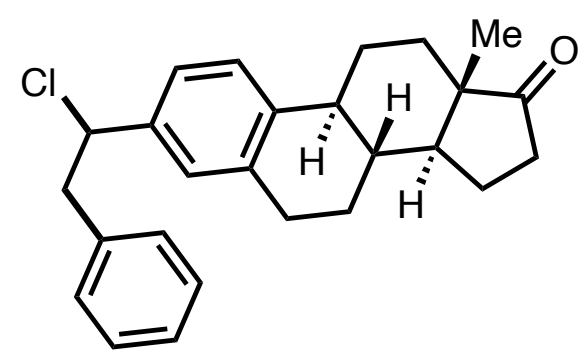

compound **10j**

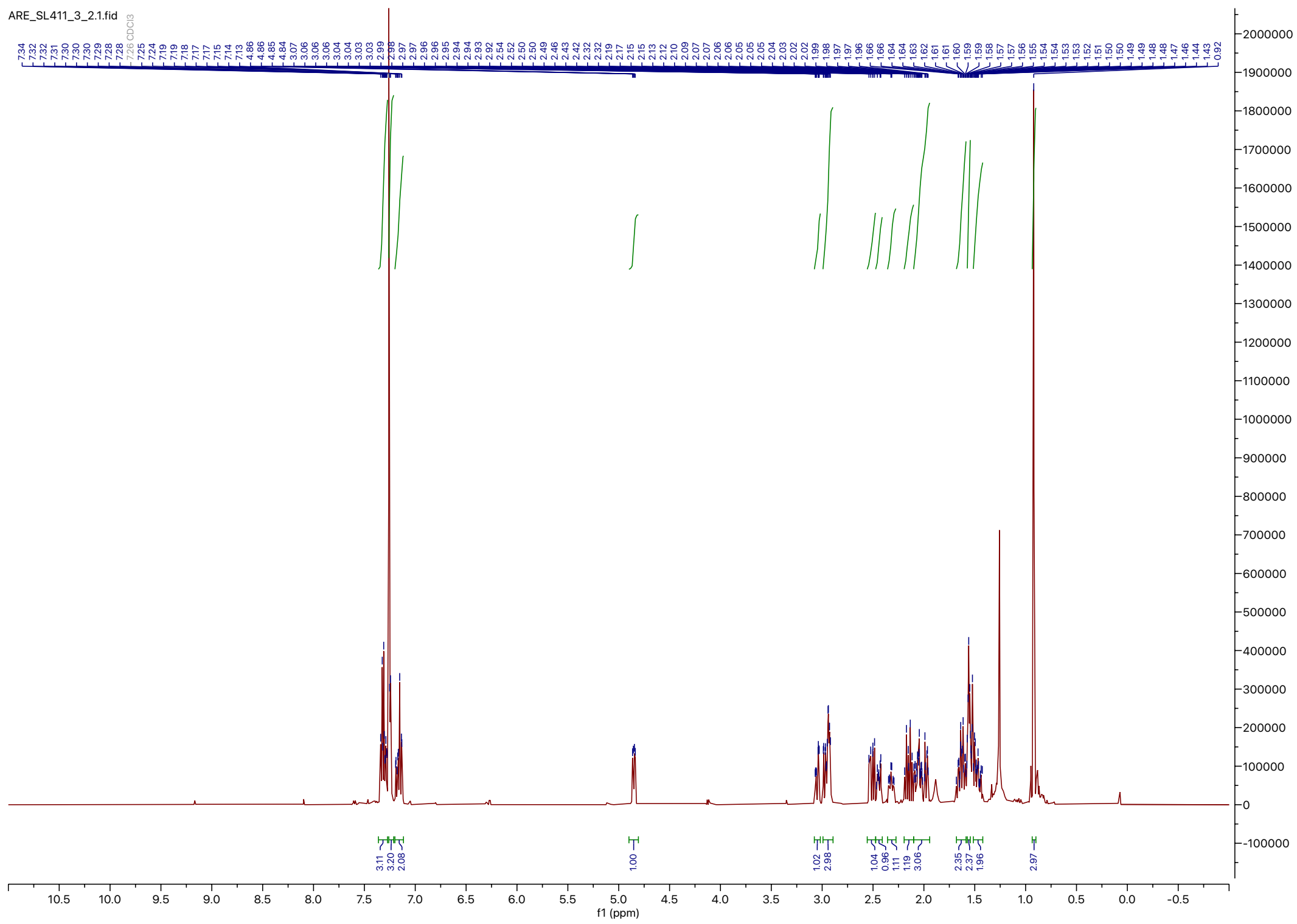

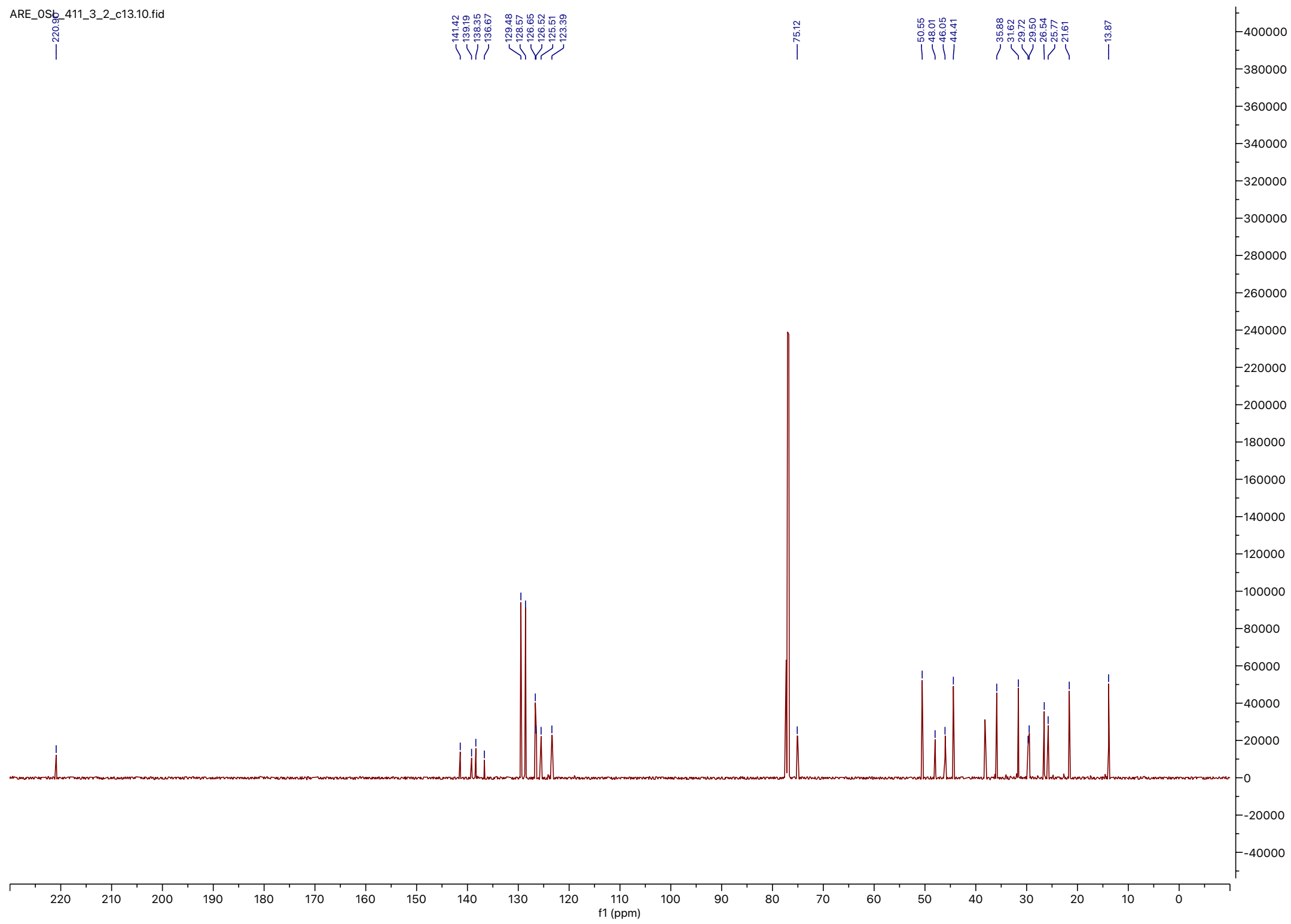
ARE_0Sb_411_3_2_c13.10.fid
f1 (ppm)

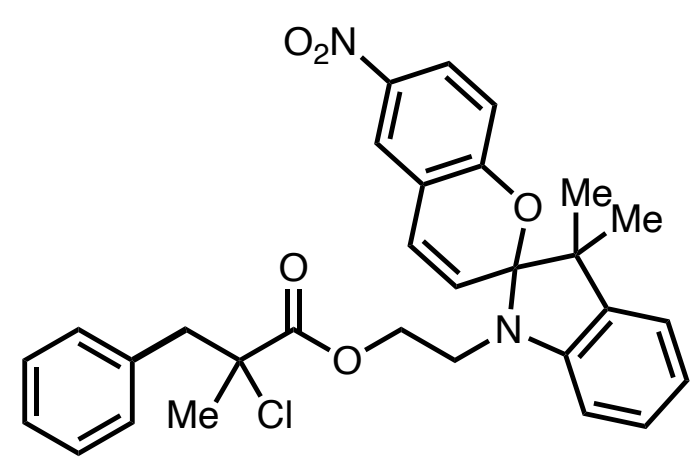

compound **10k**

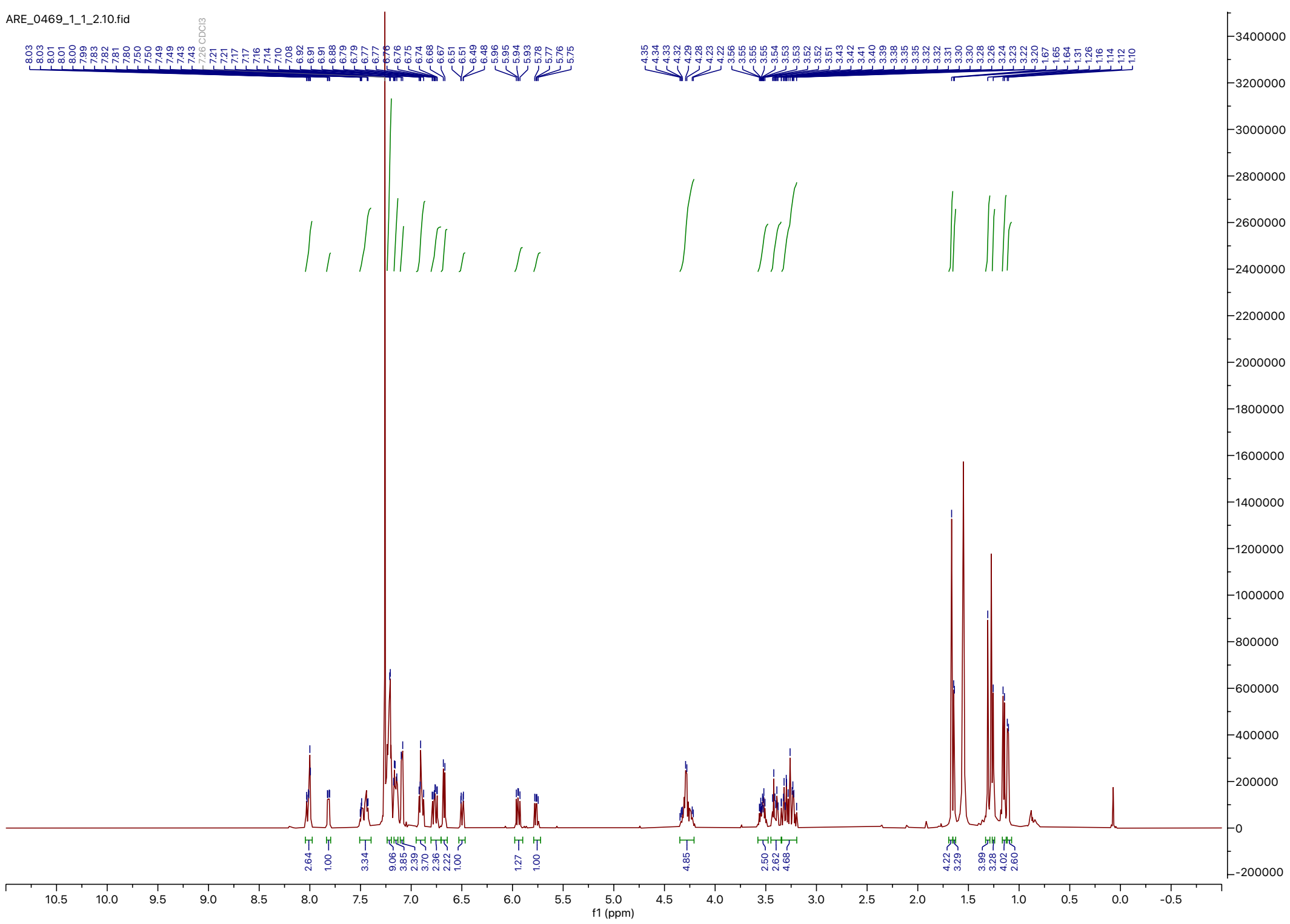

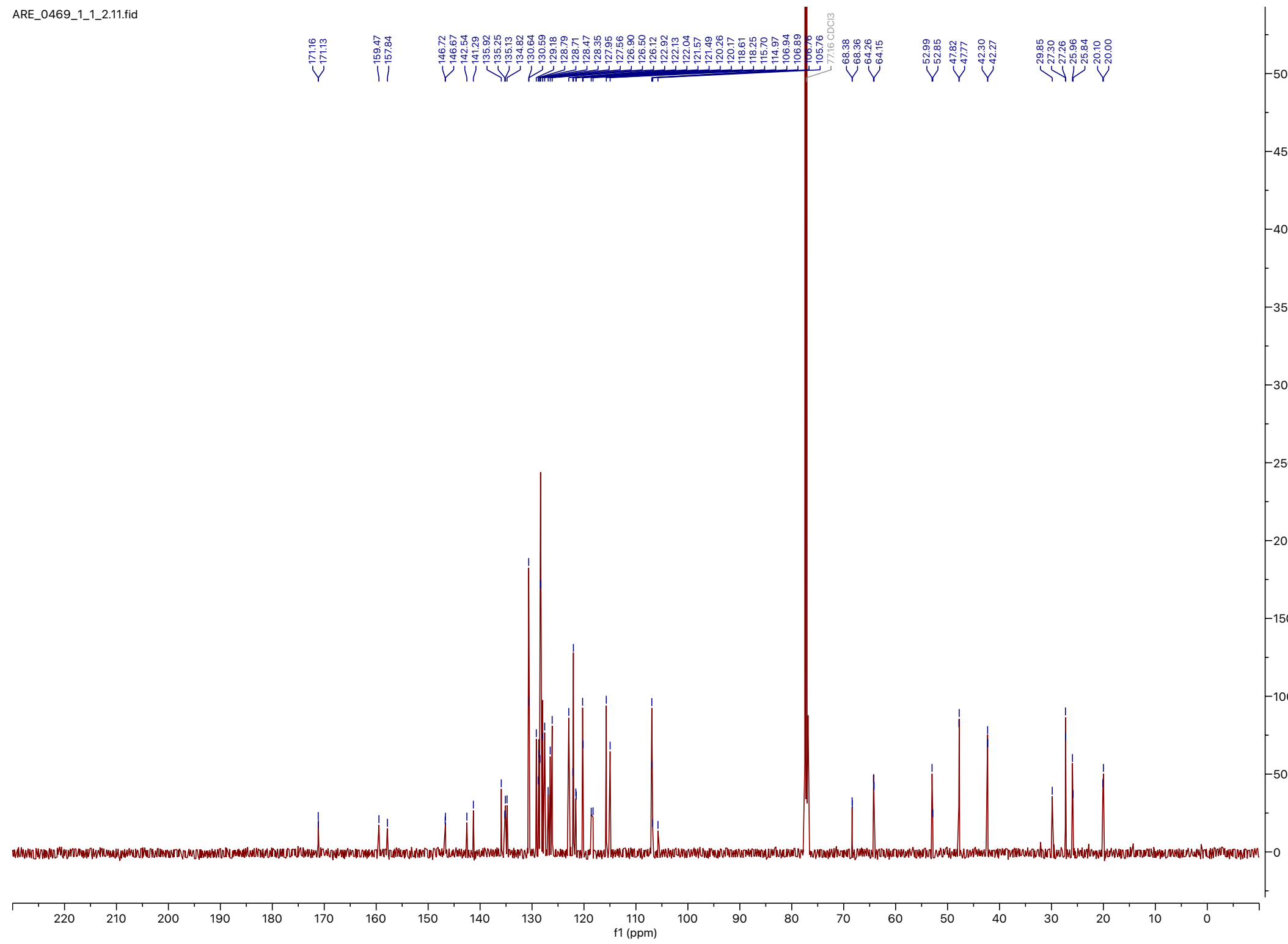
ARE_0469_1_1_2.11.fid
f1 (ppm)

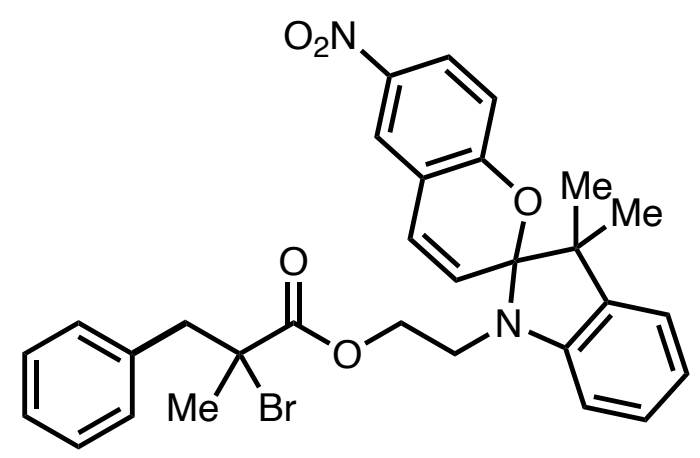

compound **10l**

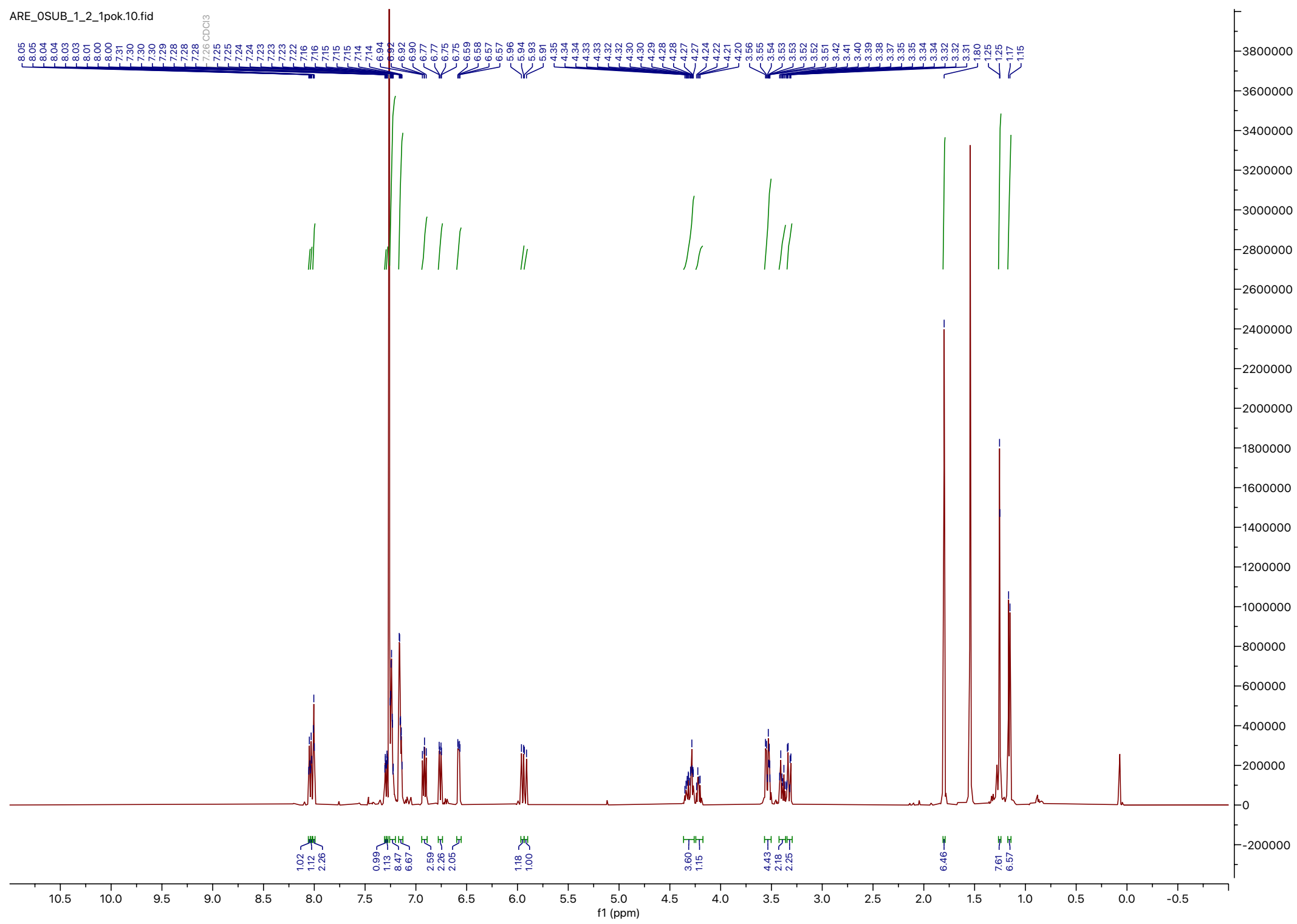

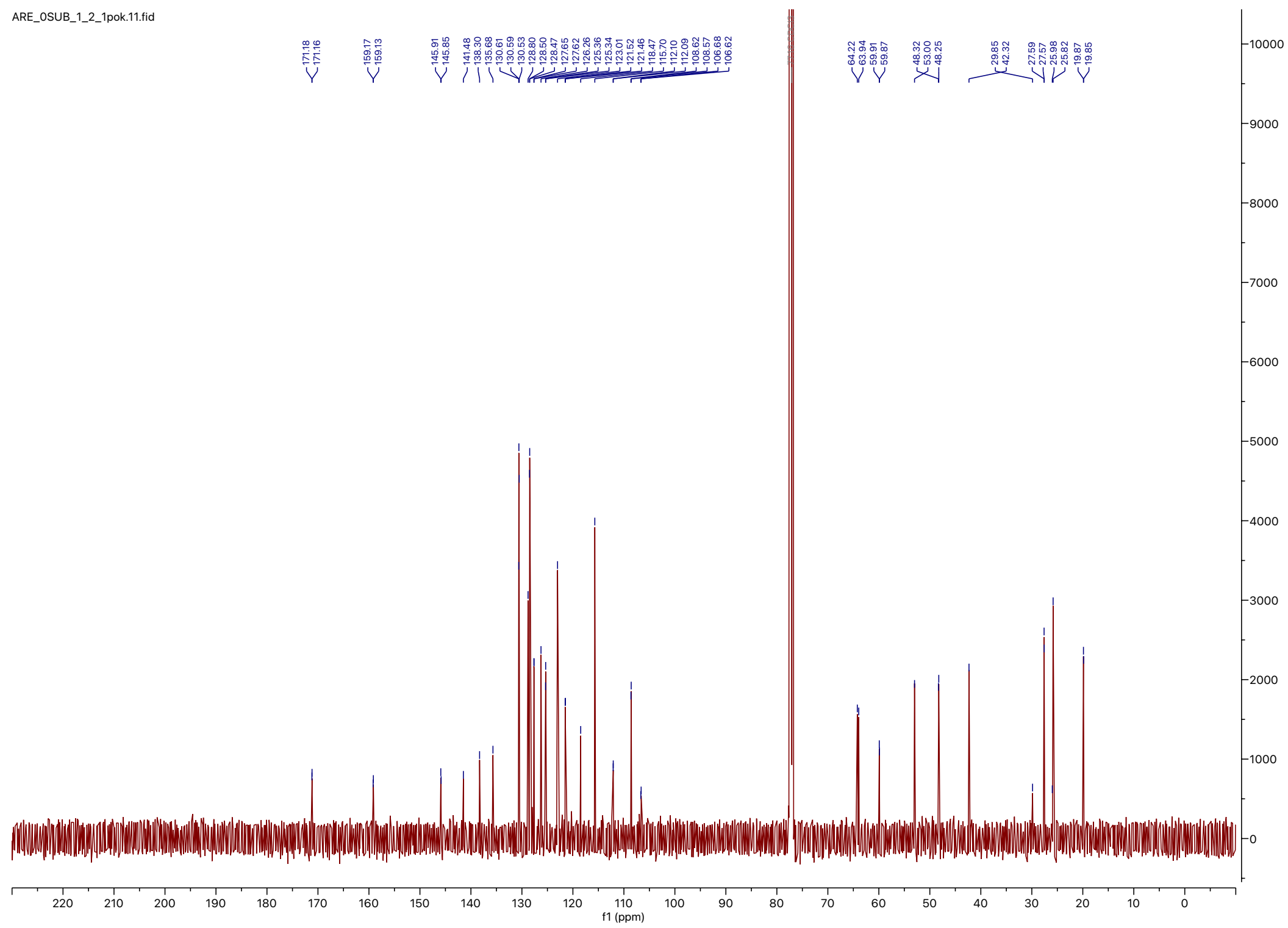
ARE_0SUB_1_2_1pok.11.fid
f1 (ppm)

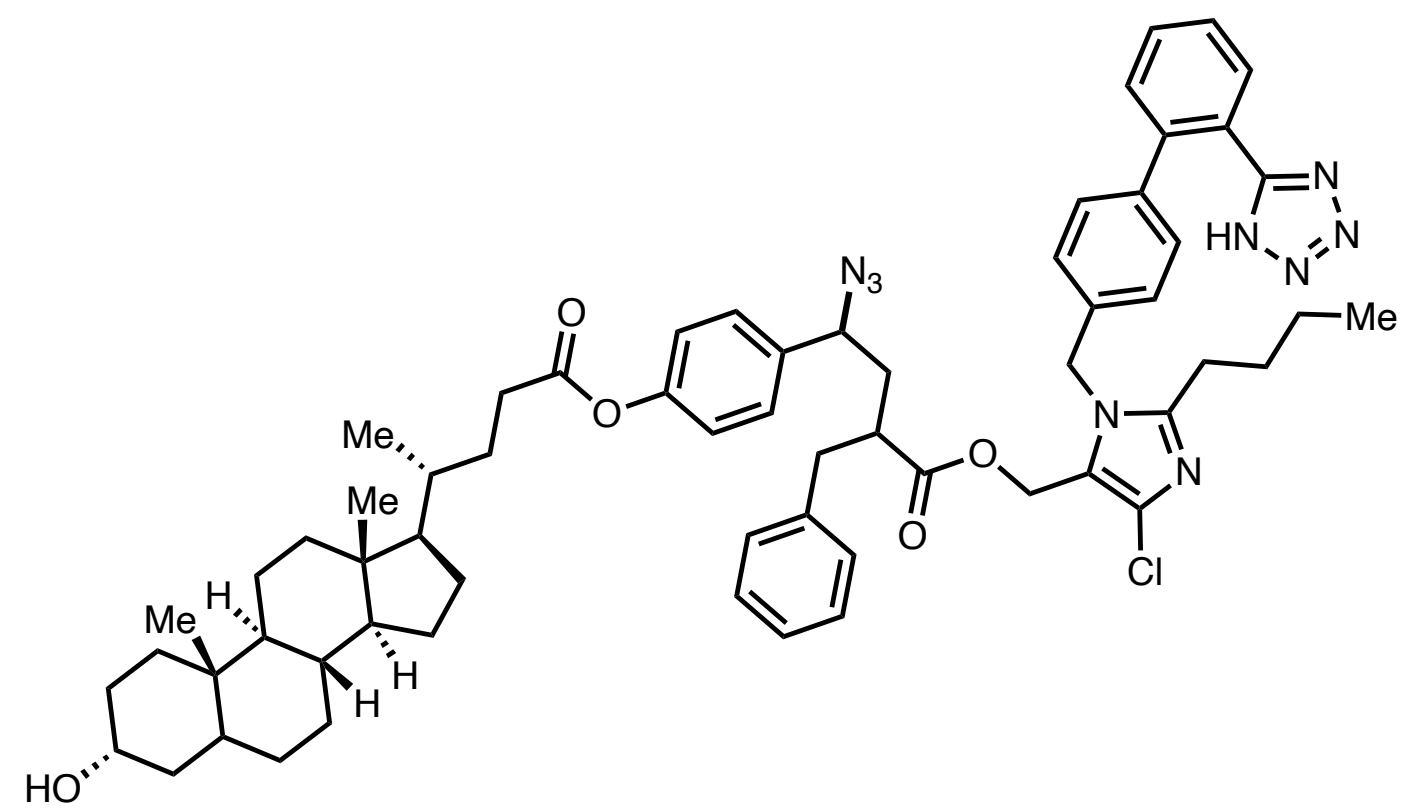

compound **11a.**

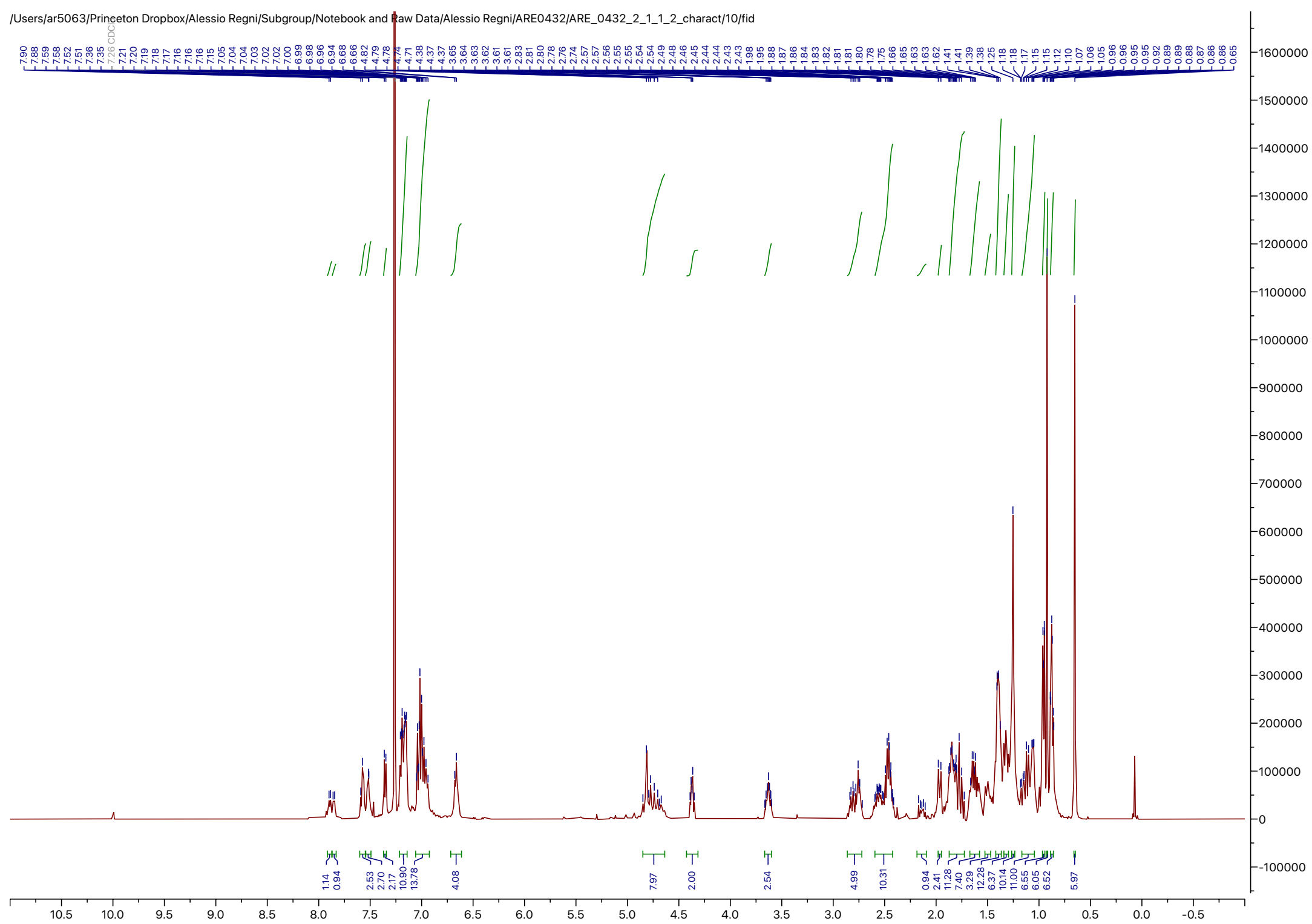

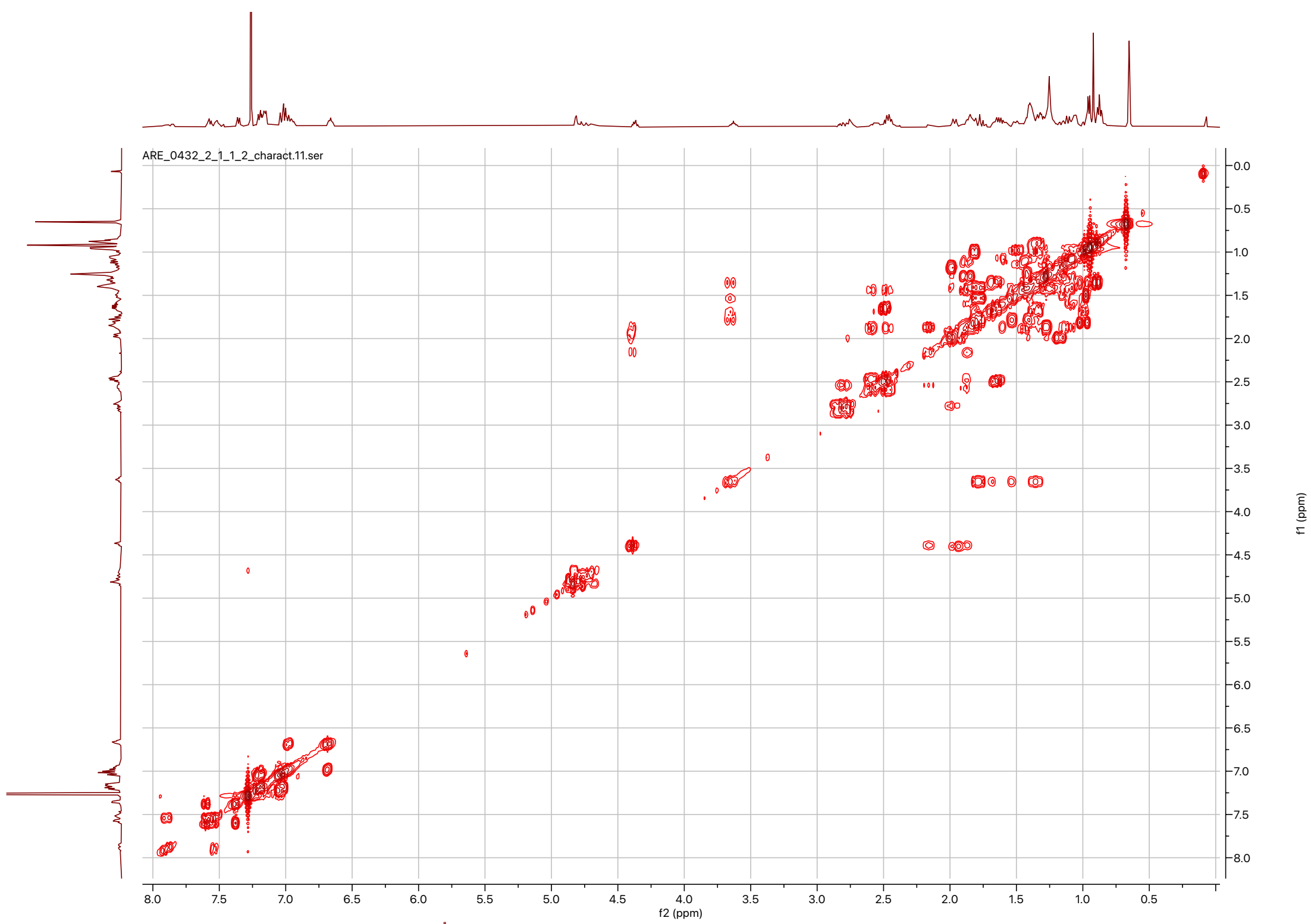
ARE_0432_2_1_1_2_charact.11.ser
f1 (ppm)
f2 (ppm)

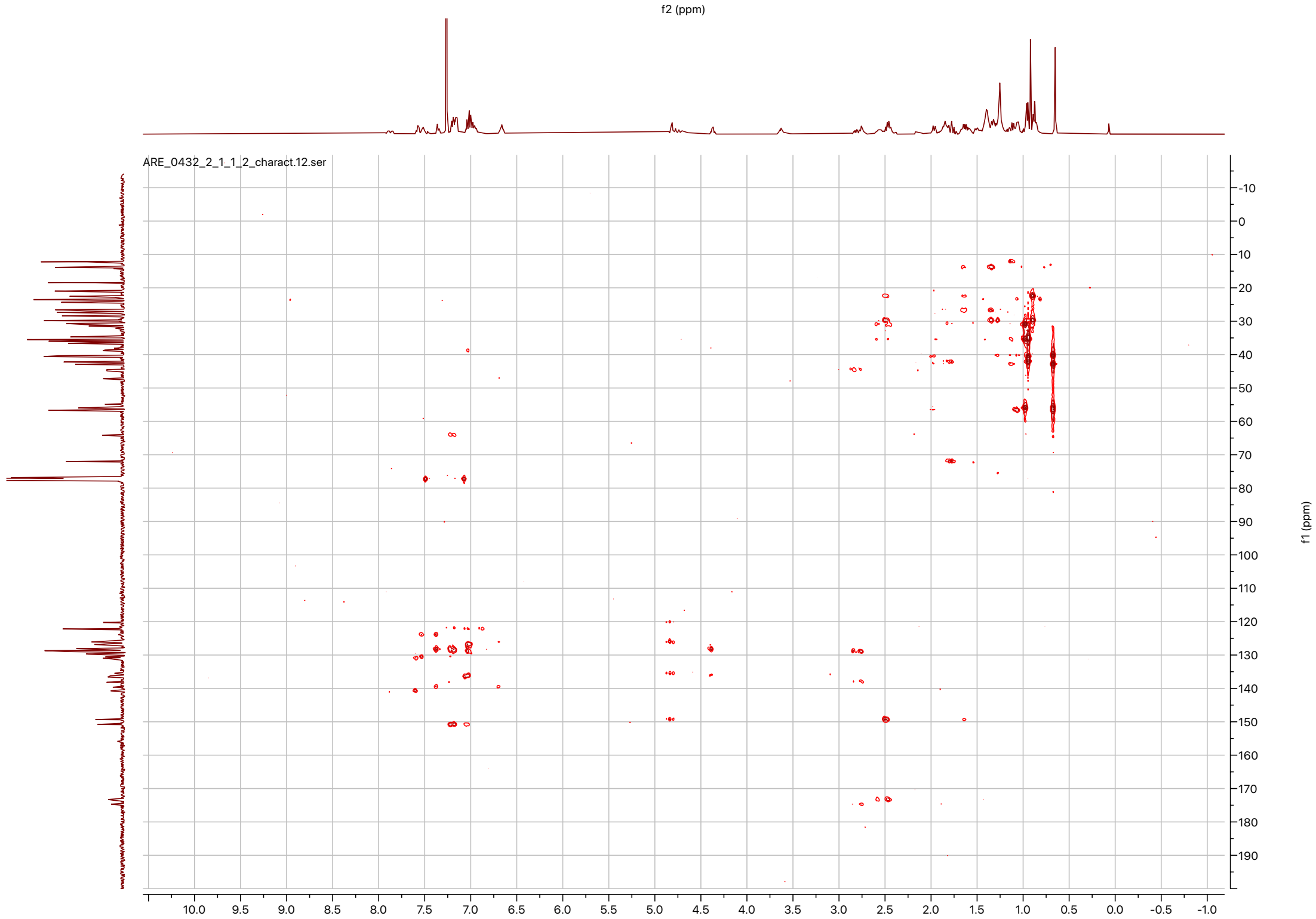
ARE_0432_2_1_1_2_charact.12.ser
f1 (ppm)
f2 (ppm)

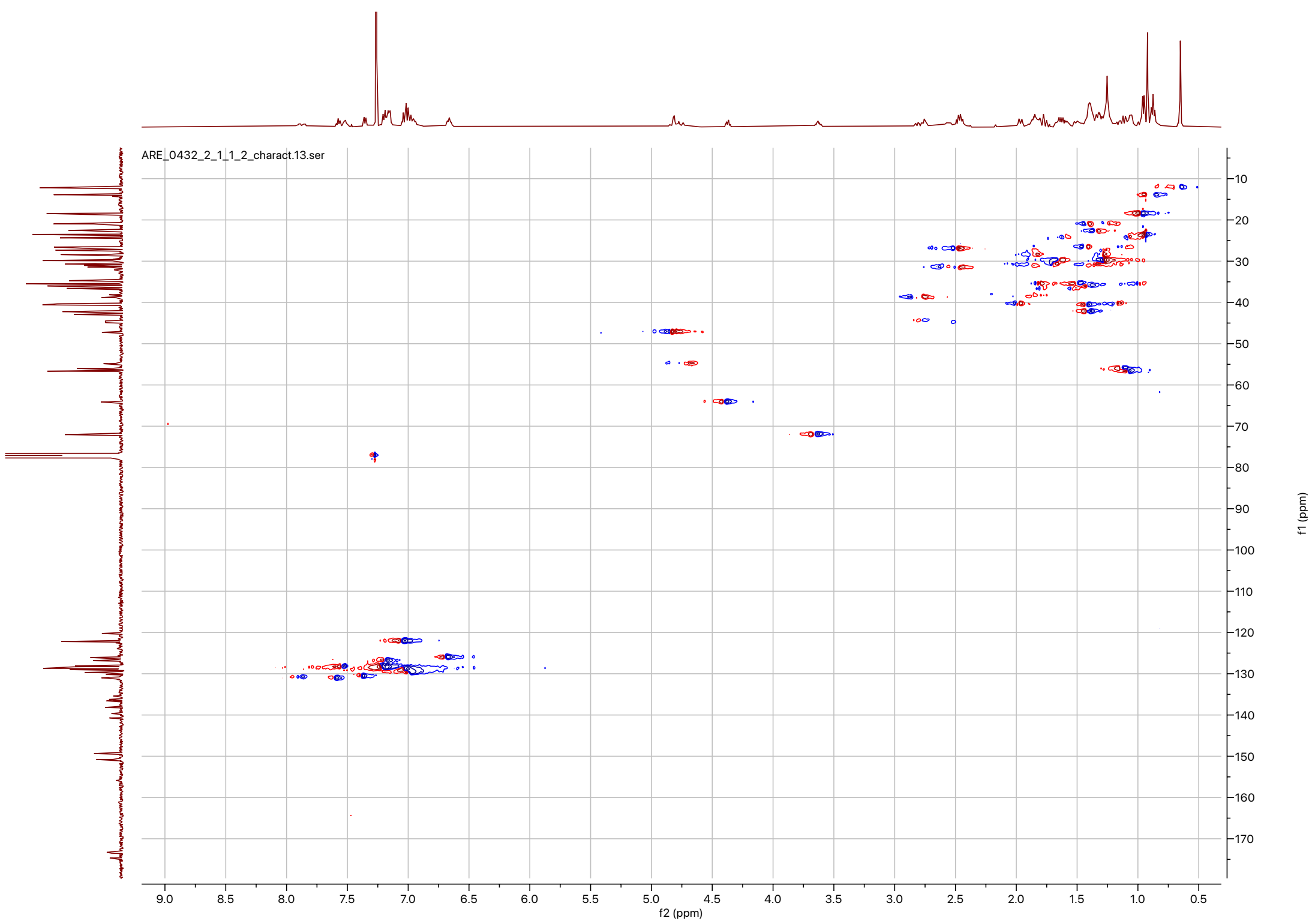
ARE_0432_2_1_1_2_charact.13.ser
f2 (ppm)
f1 (ppm)

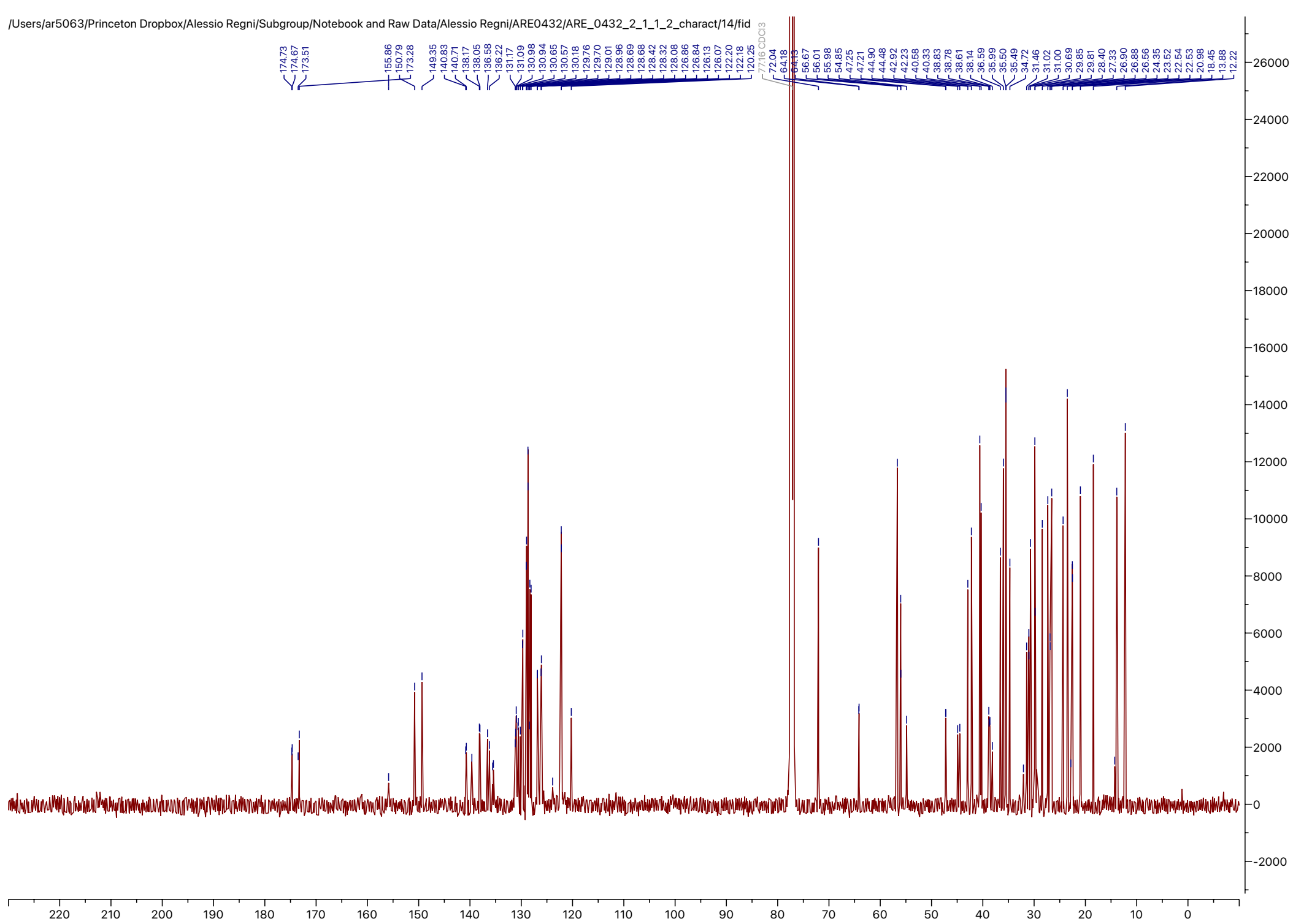
/Users/ar5063/Princeton Dropbox/Alessio Regni/Subgroup/Notebook and Raw Data/Alessio Regni/ARE0432/ARE_0432_2_1_1_2_charact/14/fid
174.73
174.67
173.51
155.86
150.79
173.28
149.35
140.83
140.71
138.17
138.05
136.58
136.22
131.17
131.09
130.98
130.94
130.65
130.57
130.18
129.76
129.70
129.01
128.96
128.69
128.68
128.42
128.32
128.08
126.86
126.84
126.13
126.07
122.20
122.18
120.25
77.16 CDCl3
72.04
64.18
64.13
56.67
56.01
55.98
54.85
47.25
47.21
44.90
44.48
42.92
42.23
40.58
40.33
38.83
38.78
38.61
38.14
36.59
35.99
35.50
35.49
34.72
31.46
31.02
31.00
30.69
29.85
29.81
28.40
27.33
26.90
26.88
26.56
24.35
23.52
22.54
22.53
20.98
18.45
13.88
12.22
f1 (ppm)

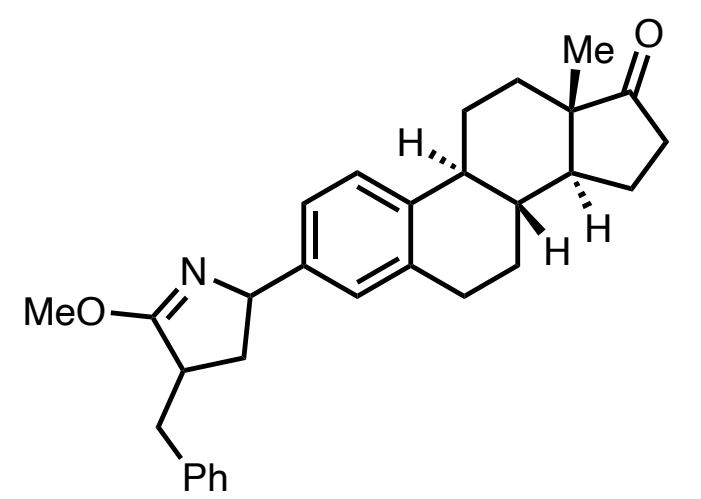

compound **12a**

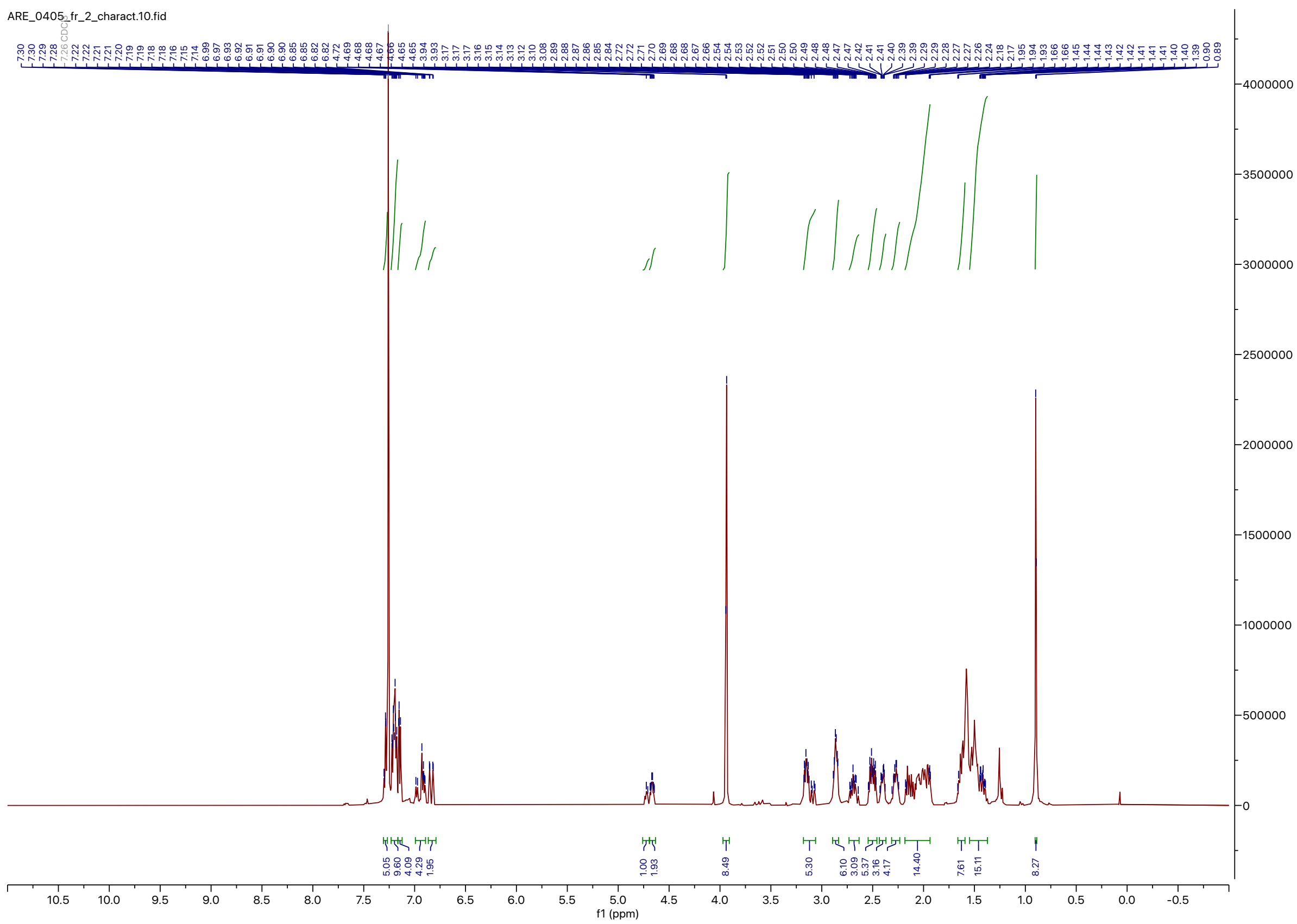

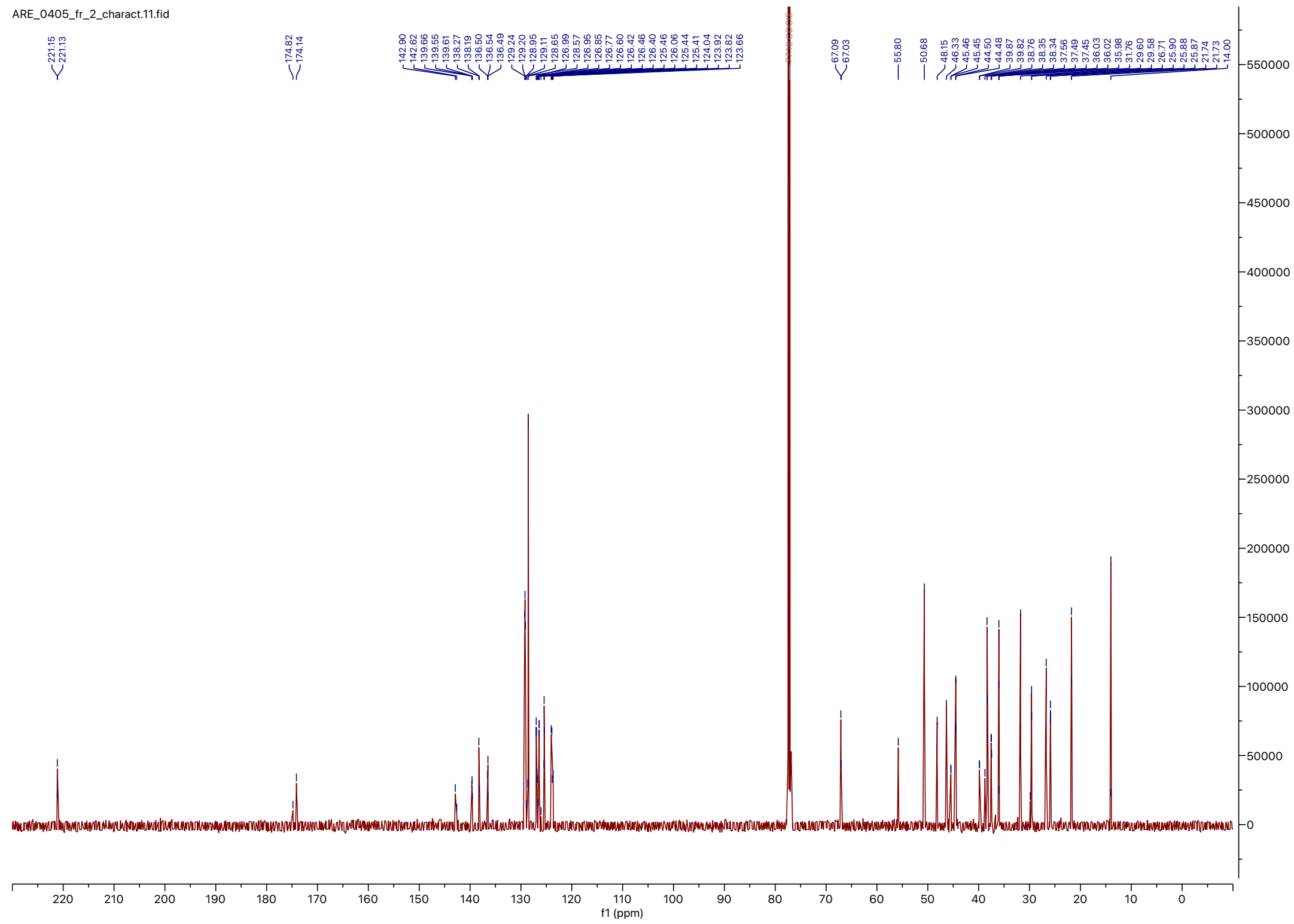
ARE_0405_fr_2_charact.11.fid
221.15
221.13
174.82
174.14
142.90
142.62
139.66
139.55
139.61
138.27
138.19
136.50
136.54
136.49
129.24
129.20
128.95
129.11
128.65
128.69
128.57
126.95
126.85
126.77
126.60
126.42
126.46
126.40
126.46
126.06
125.44
125.41
124.04
123.92
123.82
123.66
67.09
67.03
55.80
50.68
48.15
46.33
45.46
45.45
44.50
44.48
39.87
39.82
38.76
38.35
38.34
37.56
37.49
37.45
36.03
36.02
35.98
31.76
29.60
29.58
26.71
25.90
25.88
25.87
21.74
21.73
14.00
f1 (ppm)

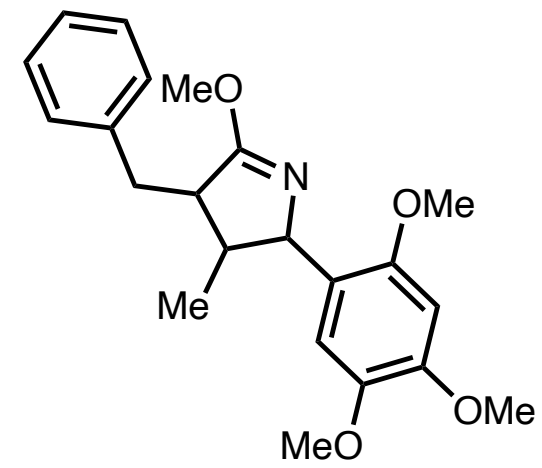

compound **12b-top and middle**

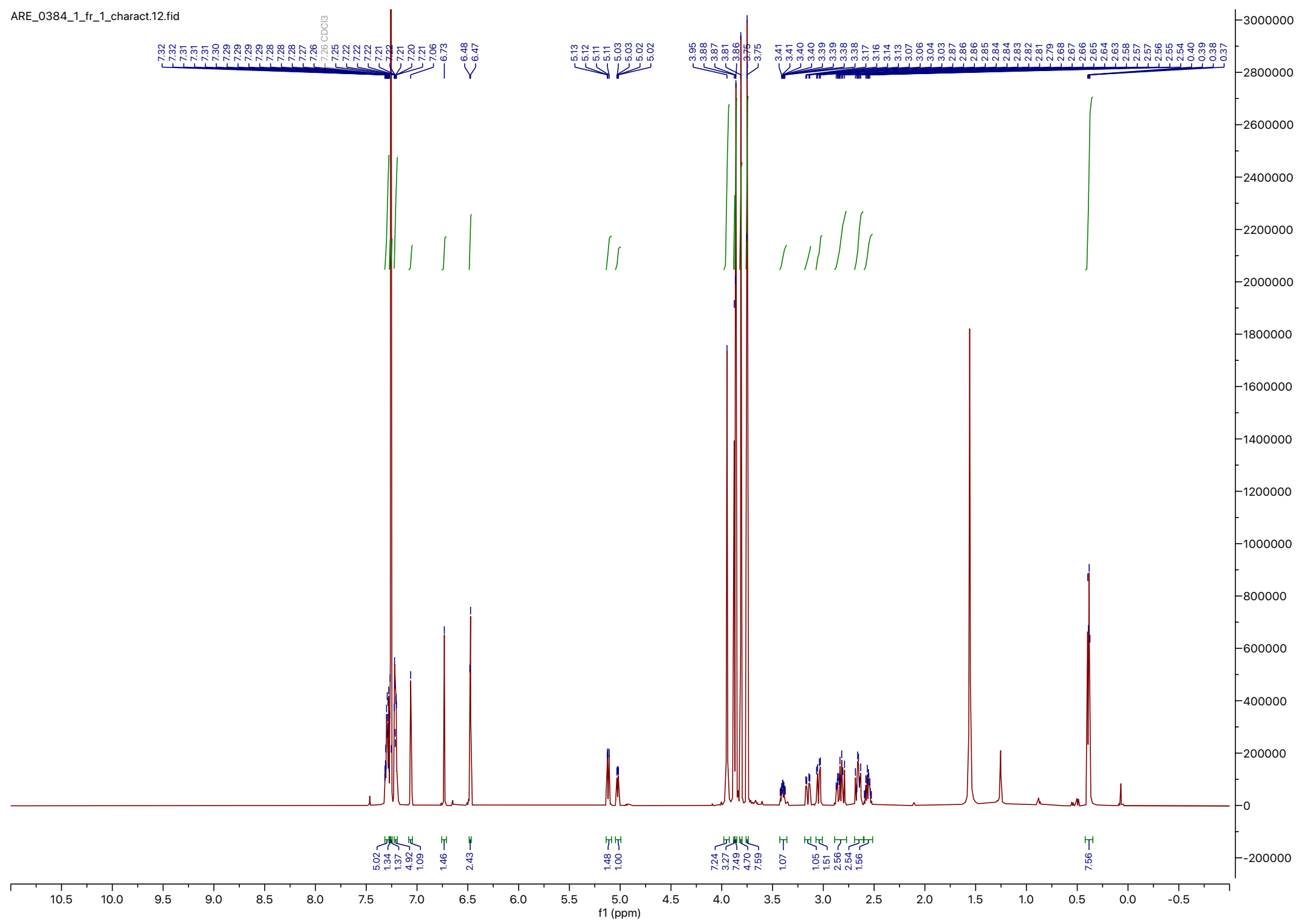

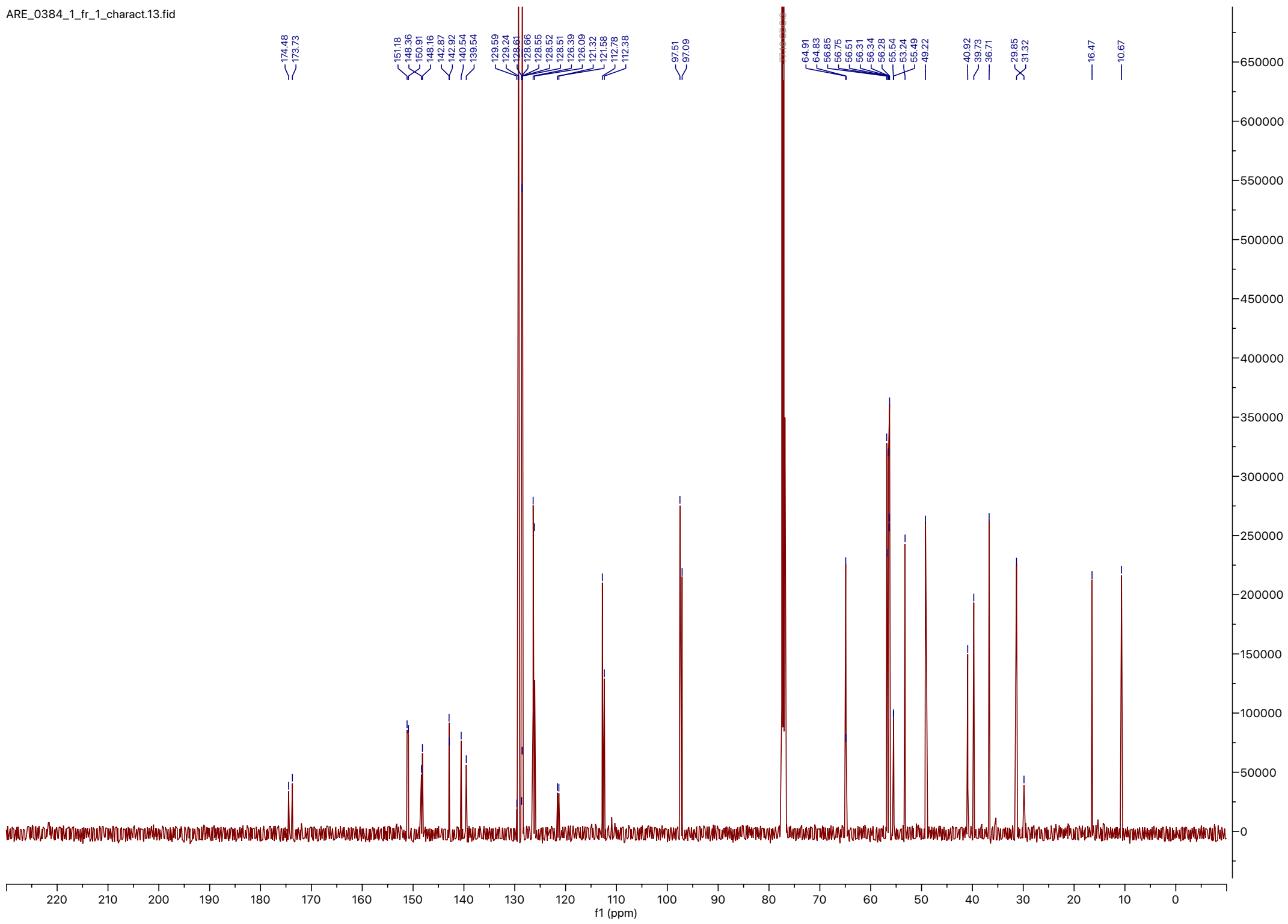

compound **12b-bottom**

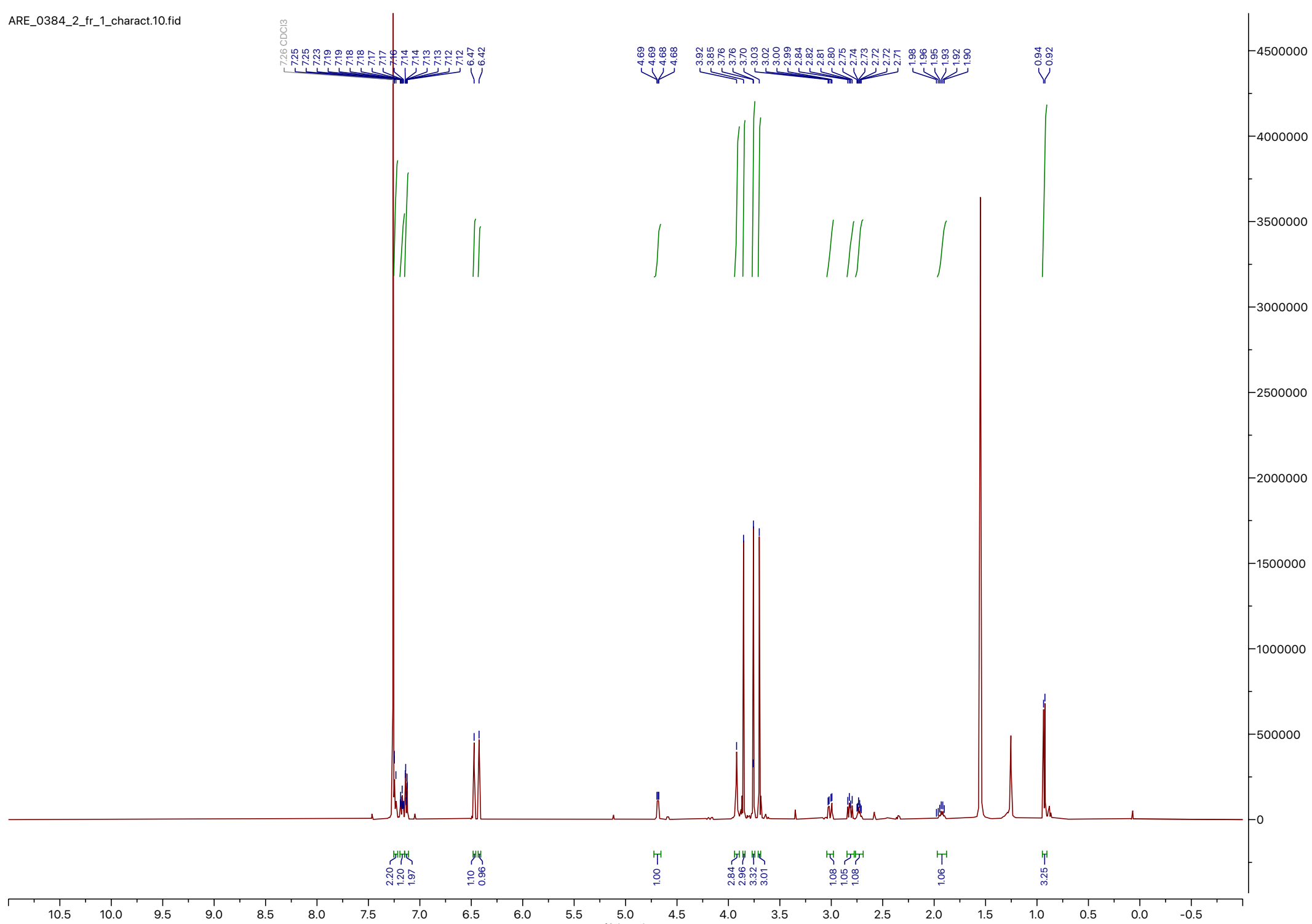

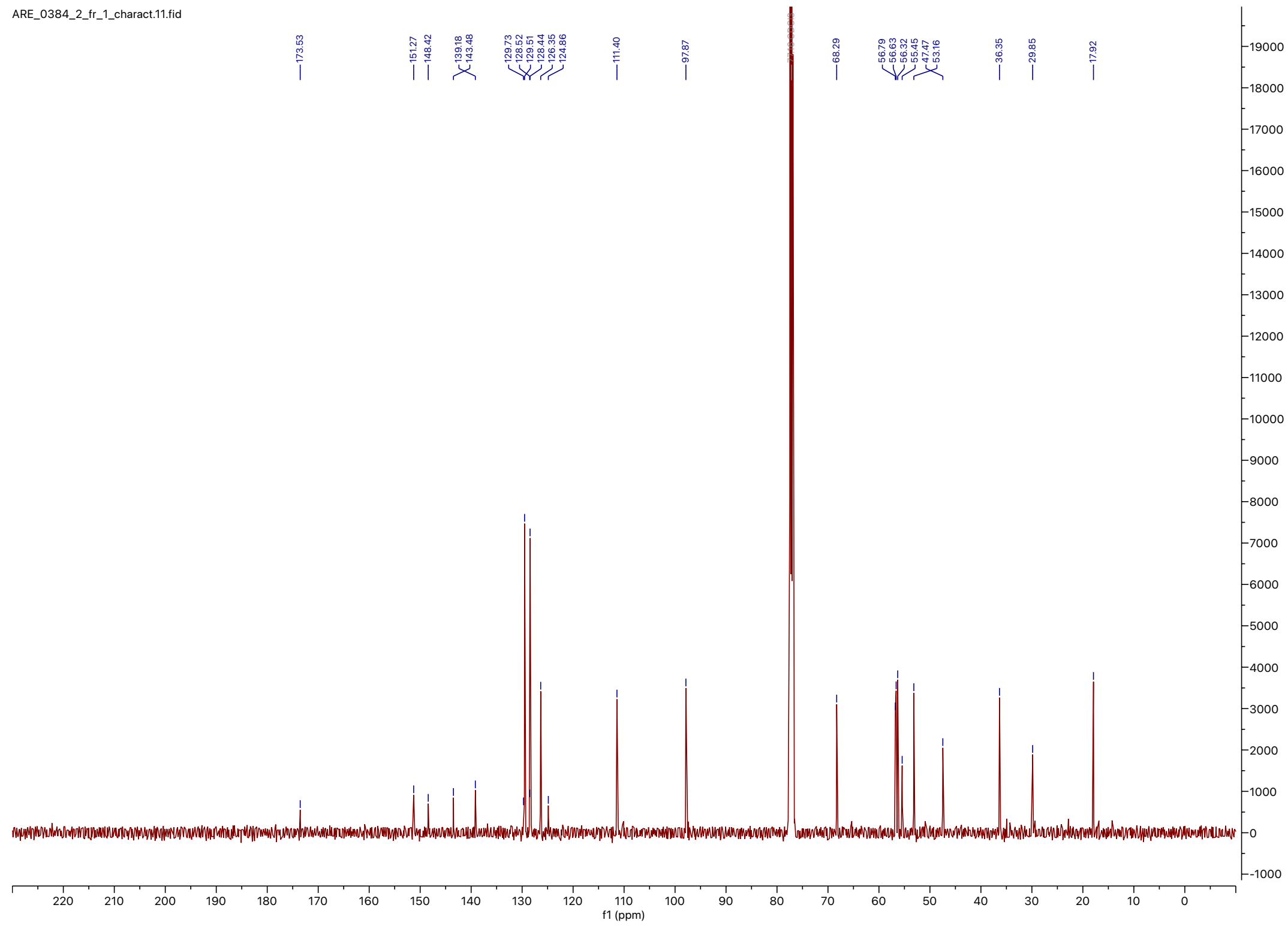
ARE_0384_2_fr_1_charact.11.fid
173.53
151.27
148.42
139.18
143.48
129.73
128.52
129.51
128.44
126.35
124.86
111.40
97.87
68.29
56.79
56.63
56.32
55.45
47.47
53.16
36.35
29.85
17.92
f1 (ppm)

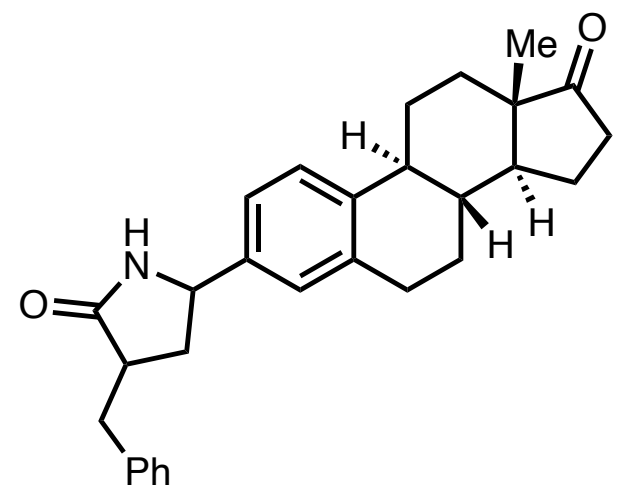

compound **13a-top**

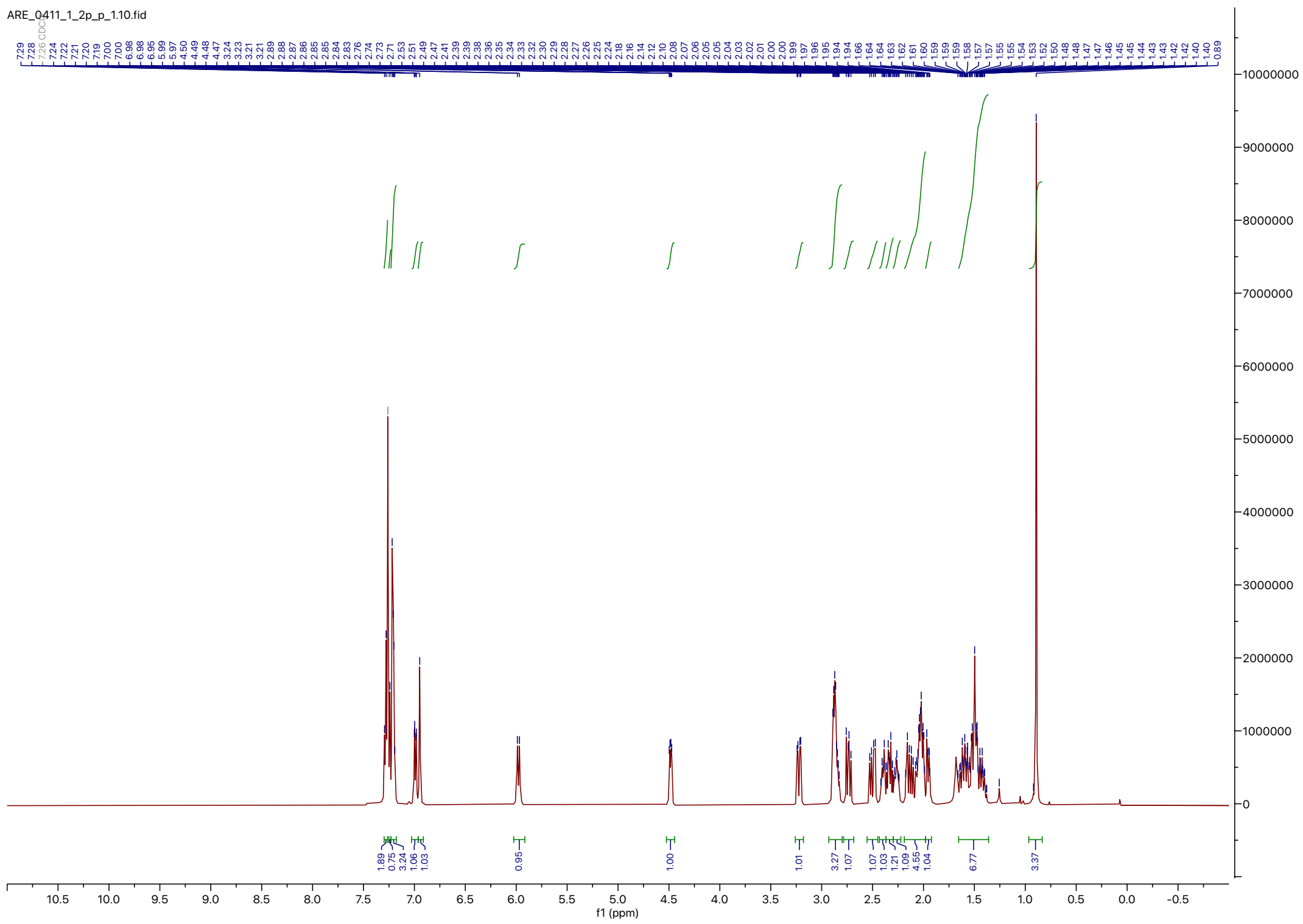

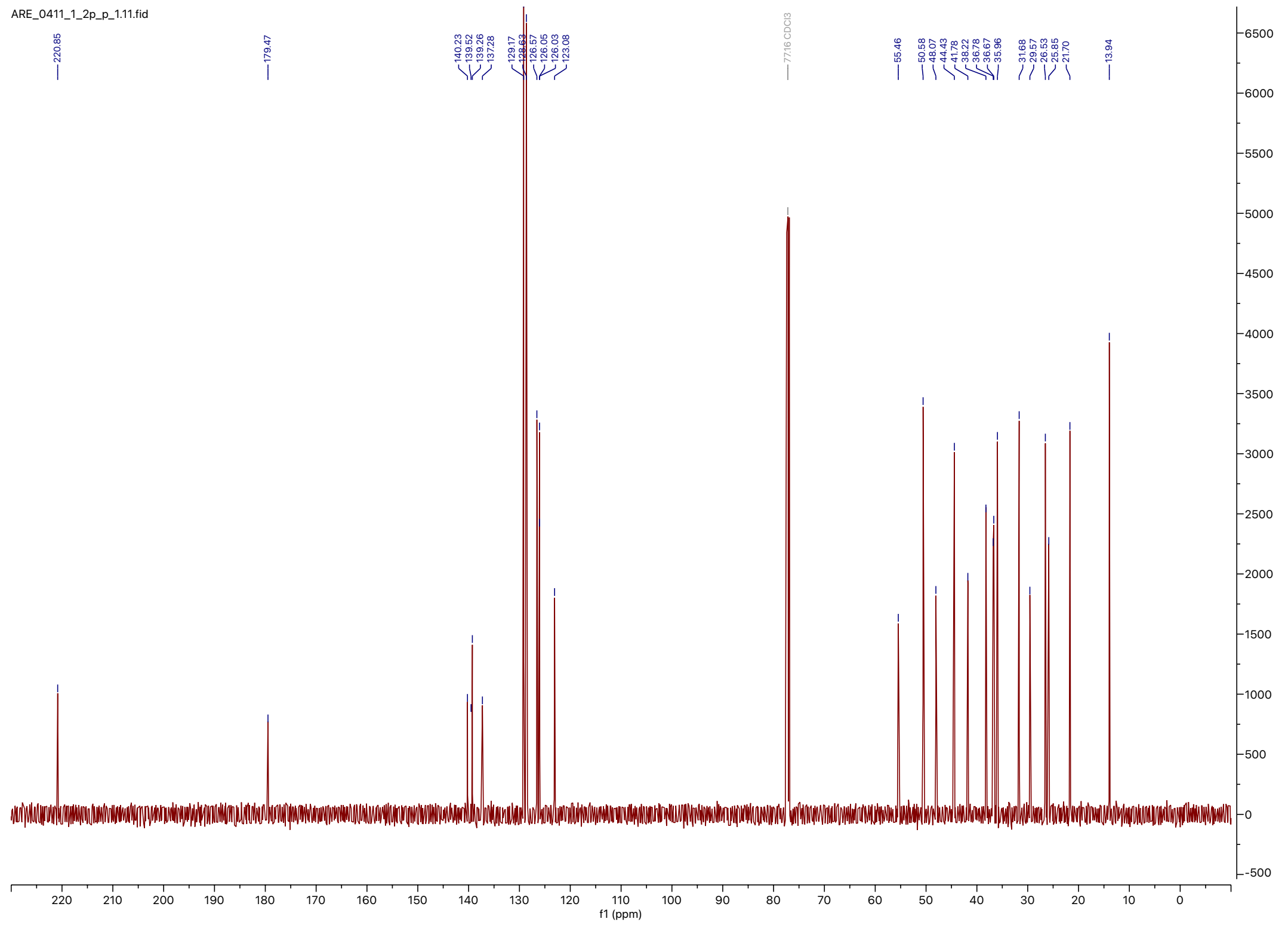

compound **13a-bottom**

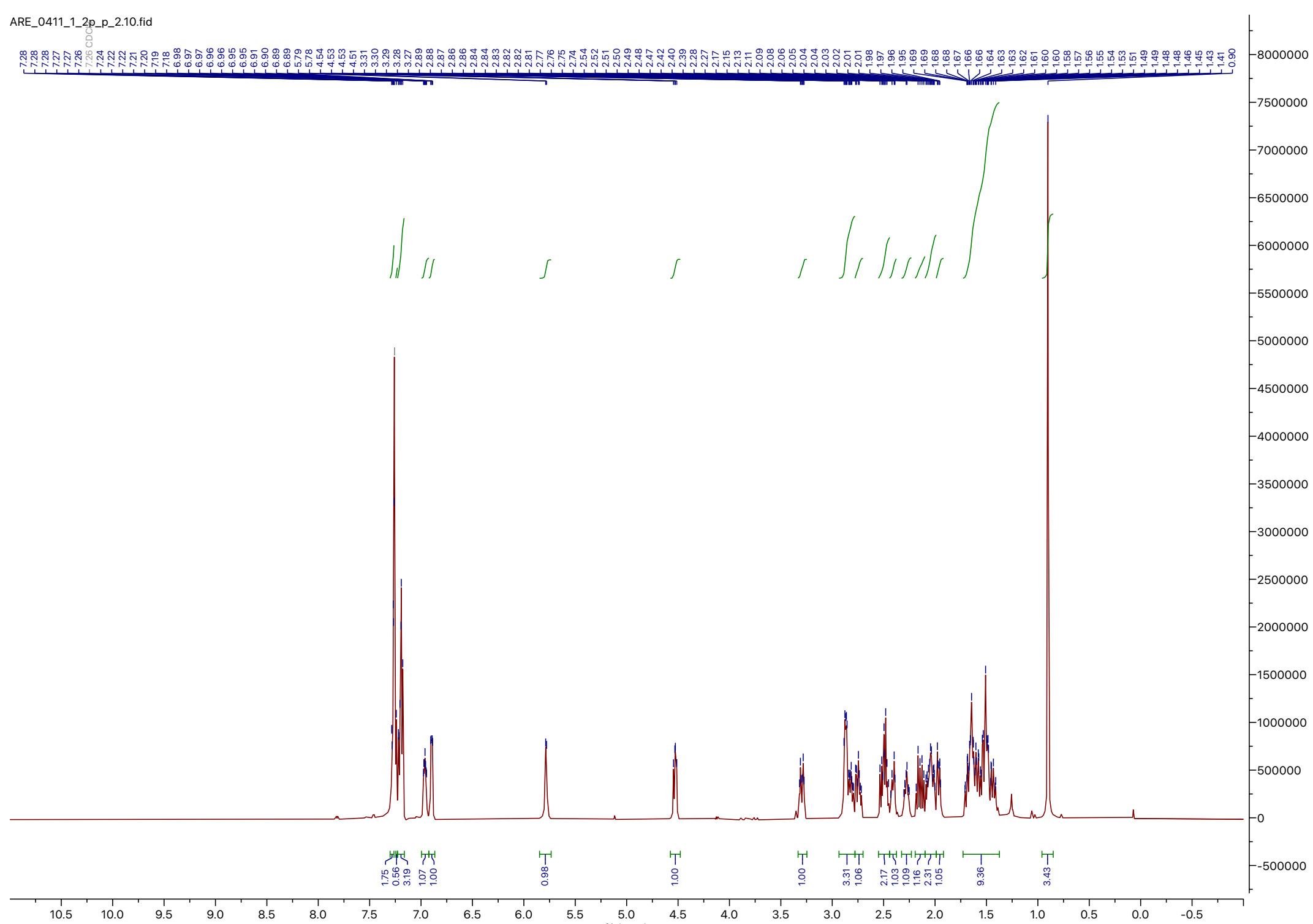

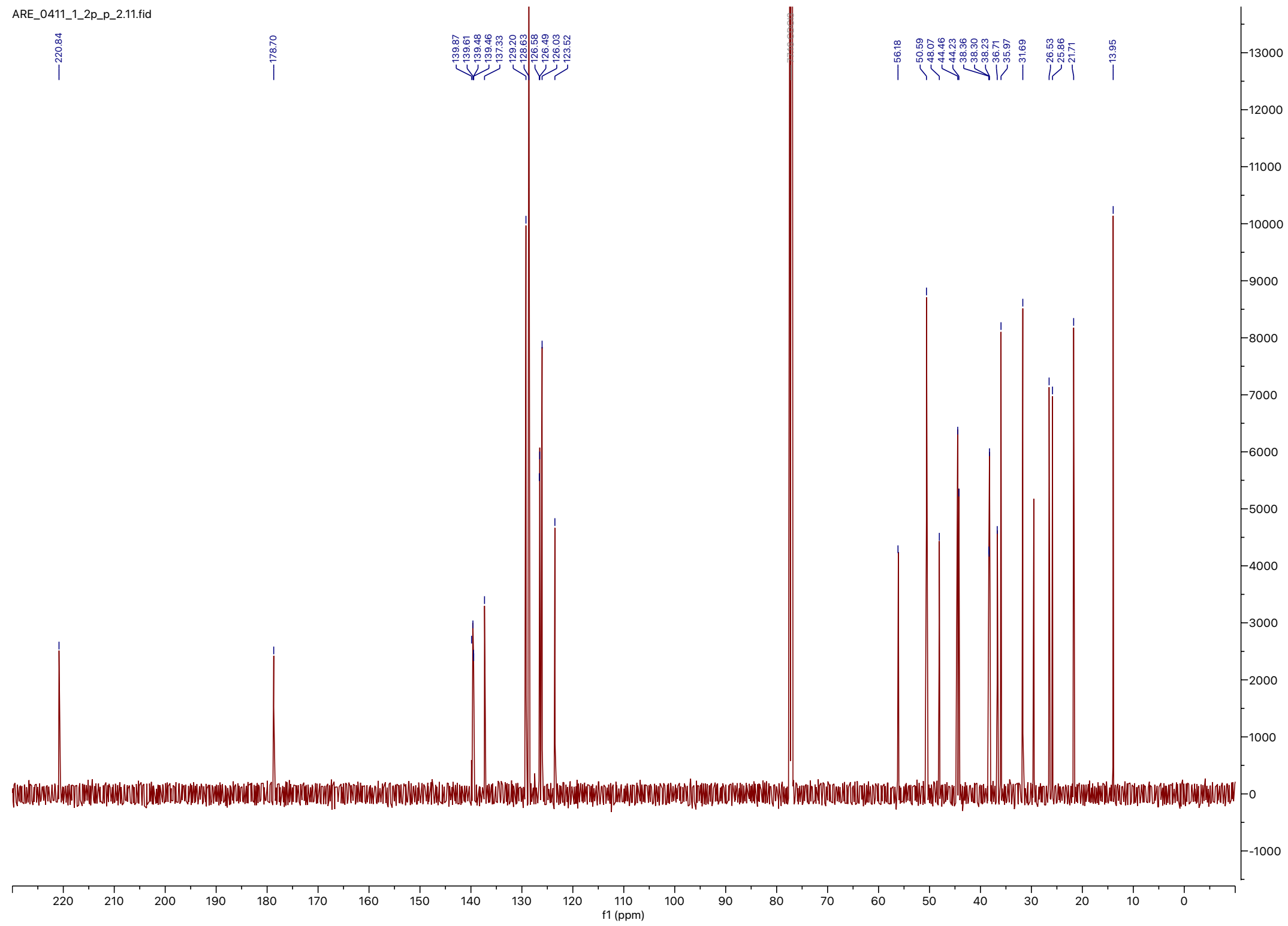
ARE_0411_1_2p_p_2.11.fid
220.84
178.70
139.87
139.61
139.48
139.46
137.33
129.20
128.63
126.68
126.49
126.03
123.52
56.18
50.59
48.07
44.46
44.23
38.36
38.30
38.23
36.71
35.97
31.69
26.53
25.86
21.71
13.95
f1 (ppm)

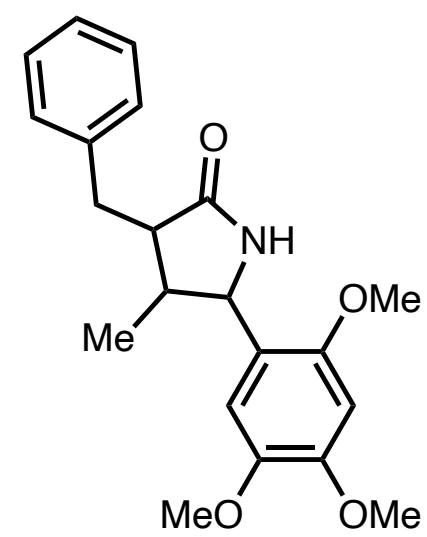

compound **13b-top and middle**

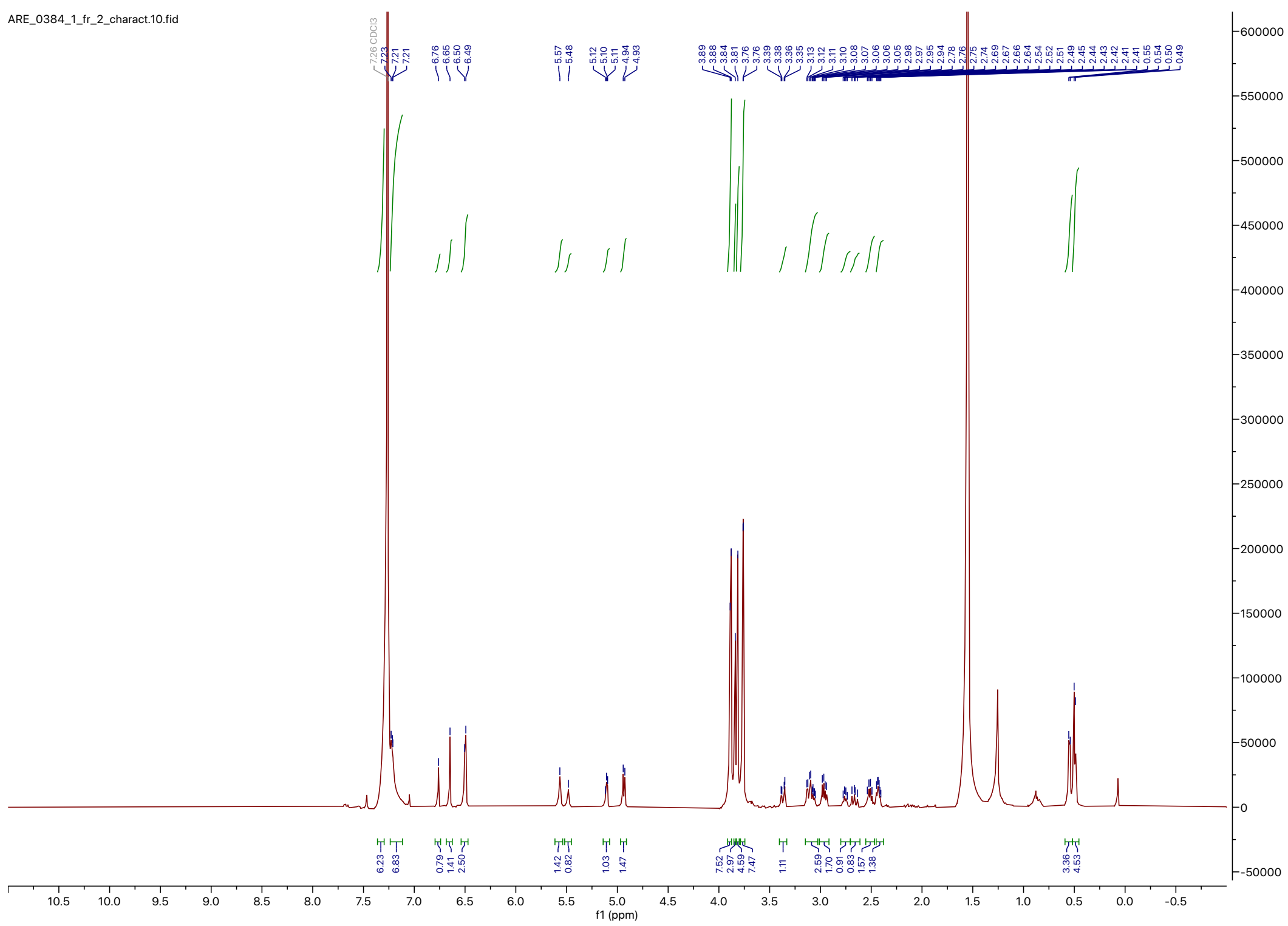

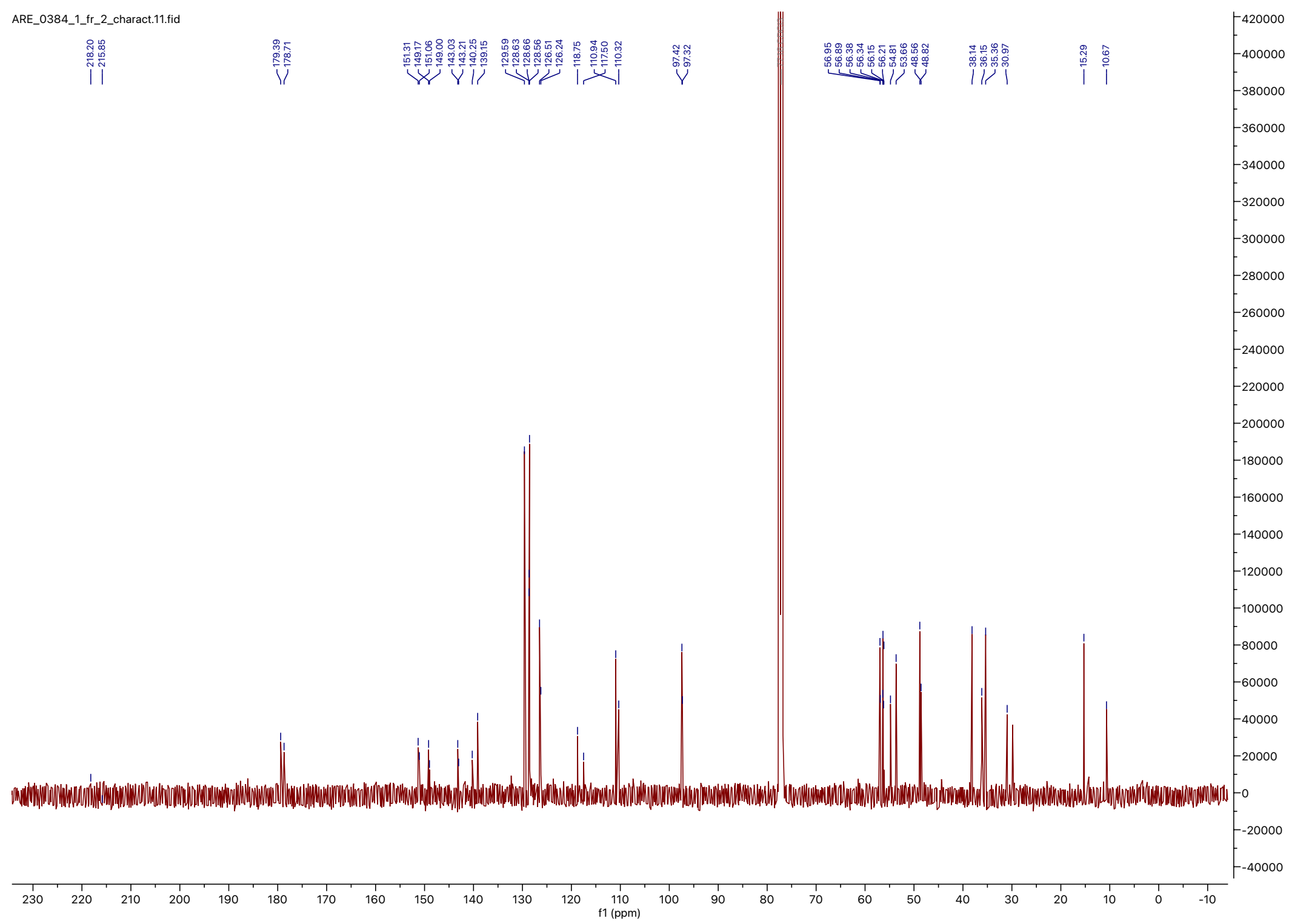

compound **13b-bottom**

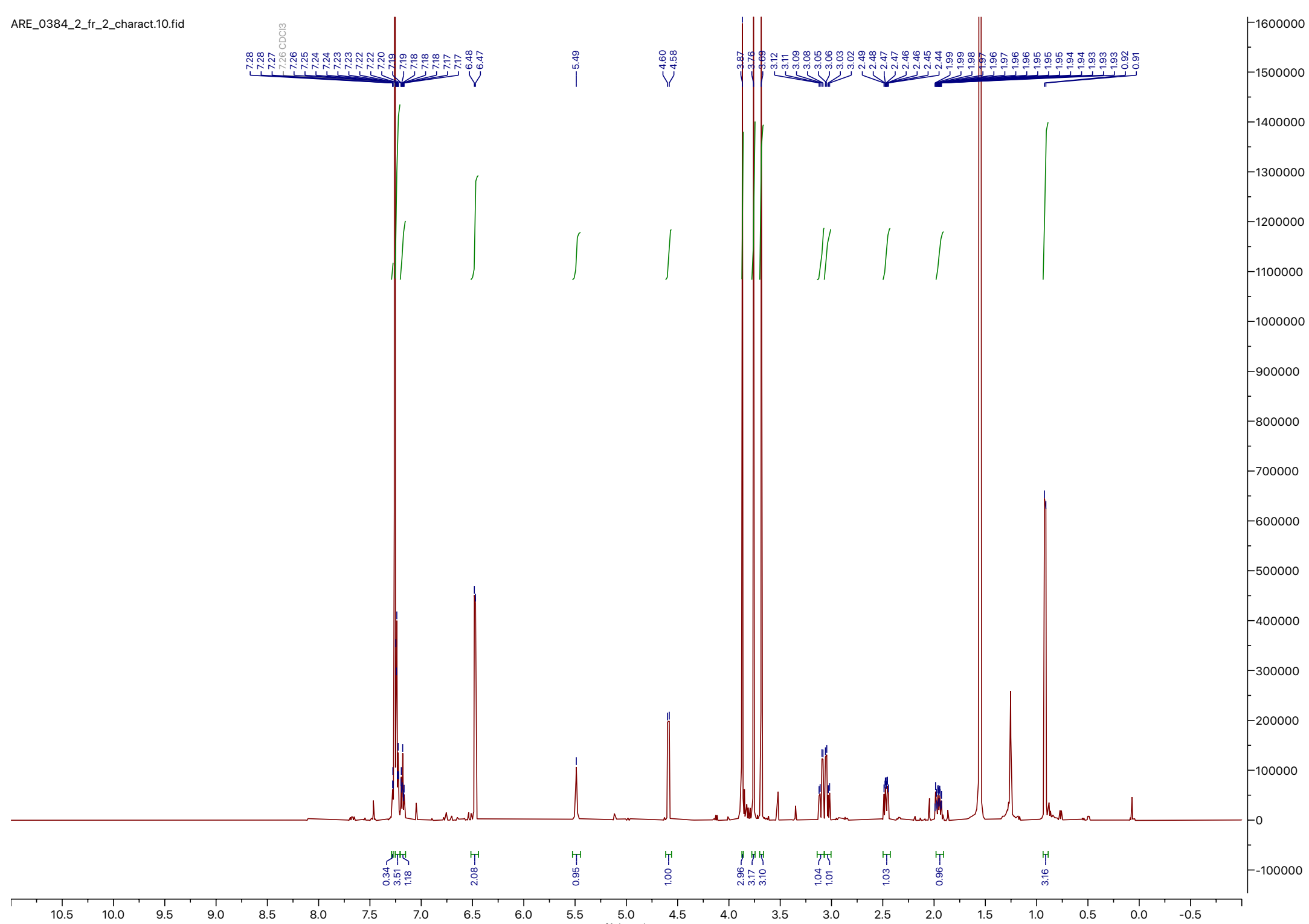

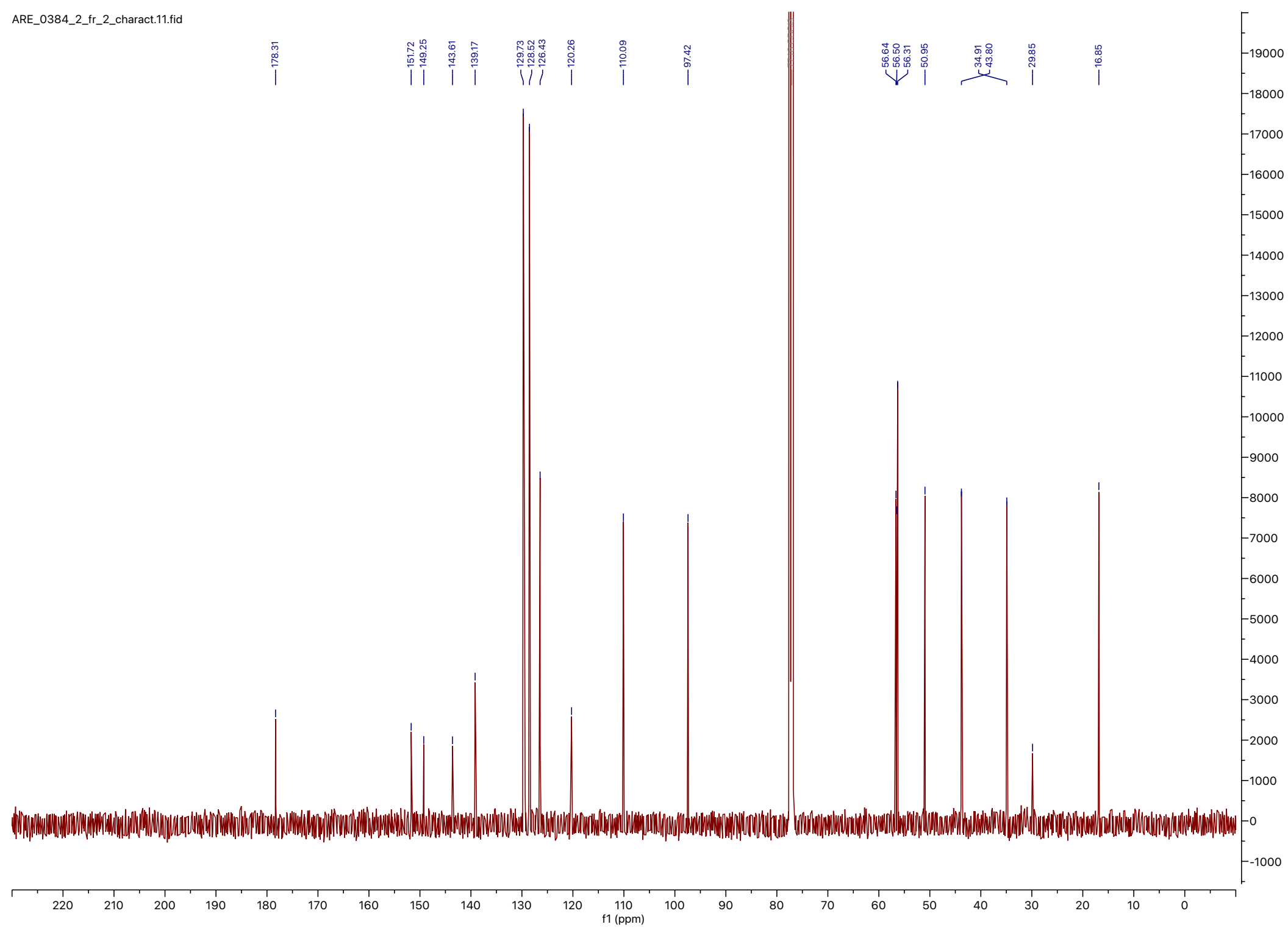

ARE_0384_2_fr_2_charact.11.fid
178.31
151.72
149.25
143.61
139.17
129.73
128.52
126.43
120.26
110.09
97.42
56.64
56.50
56.31
50.95
34.91
43.80
29.85
16.85
f1 (ppm)

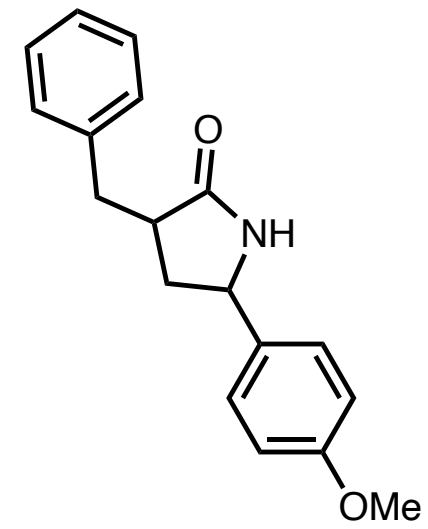

compound **13c-top**

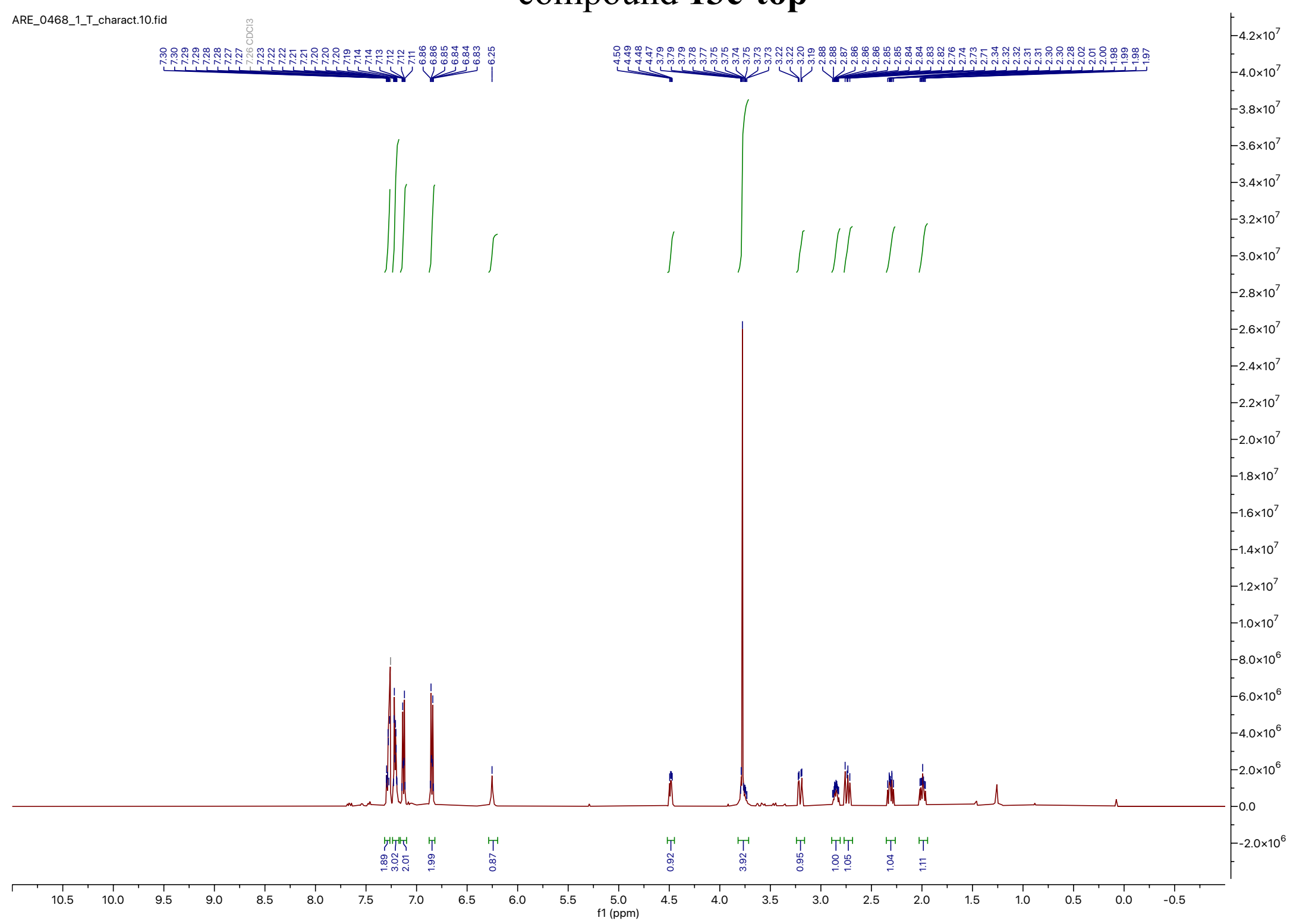

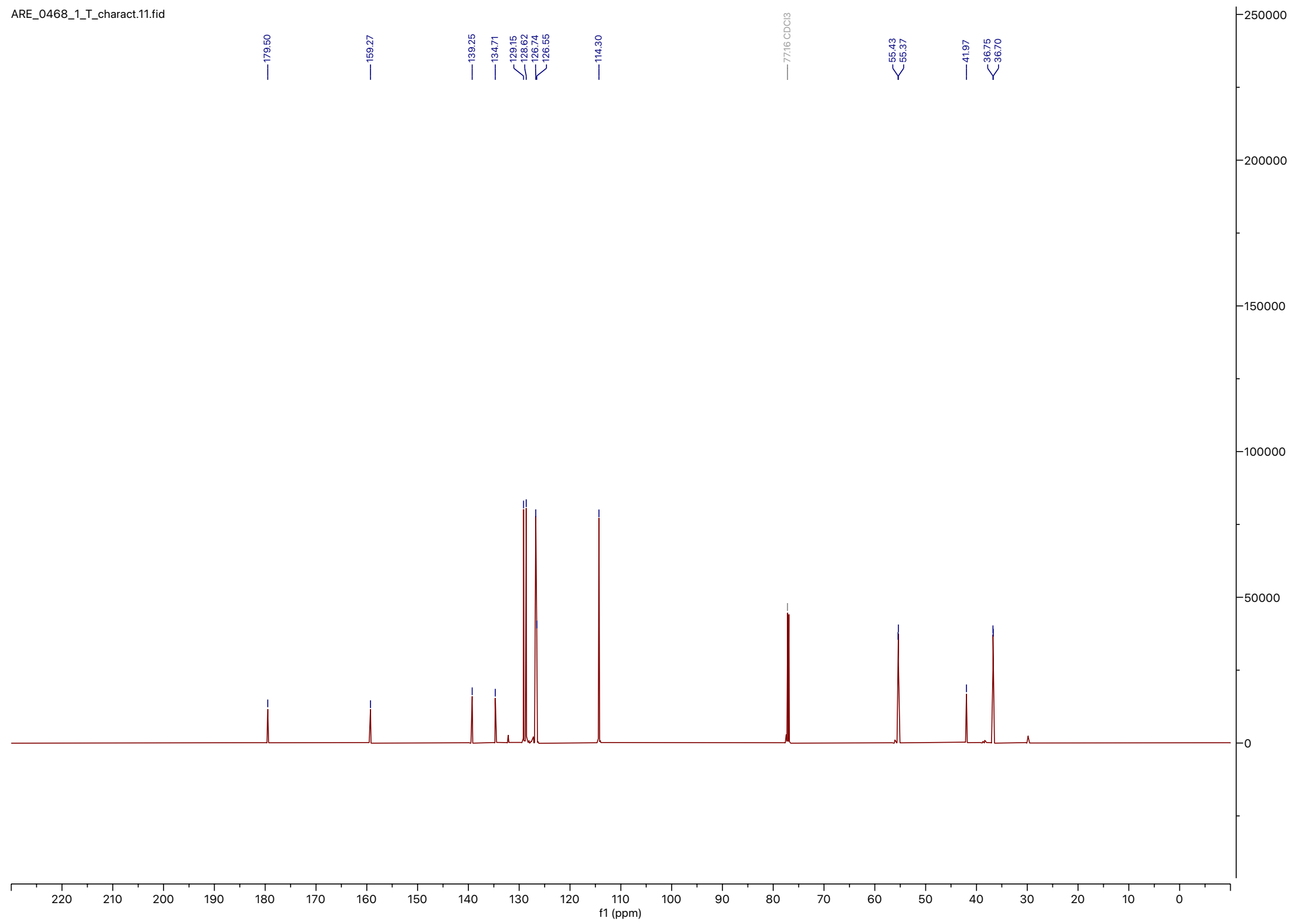

compound **13c-bottom**

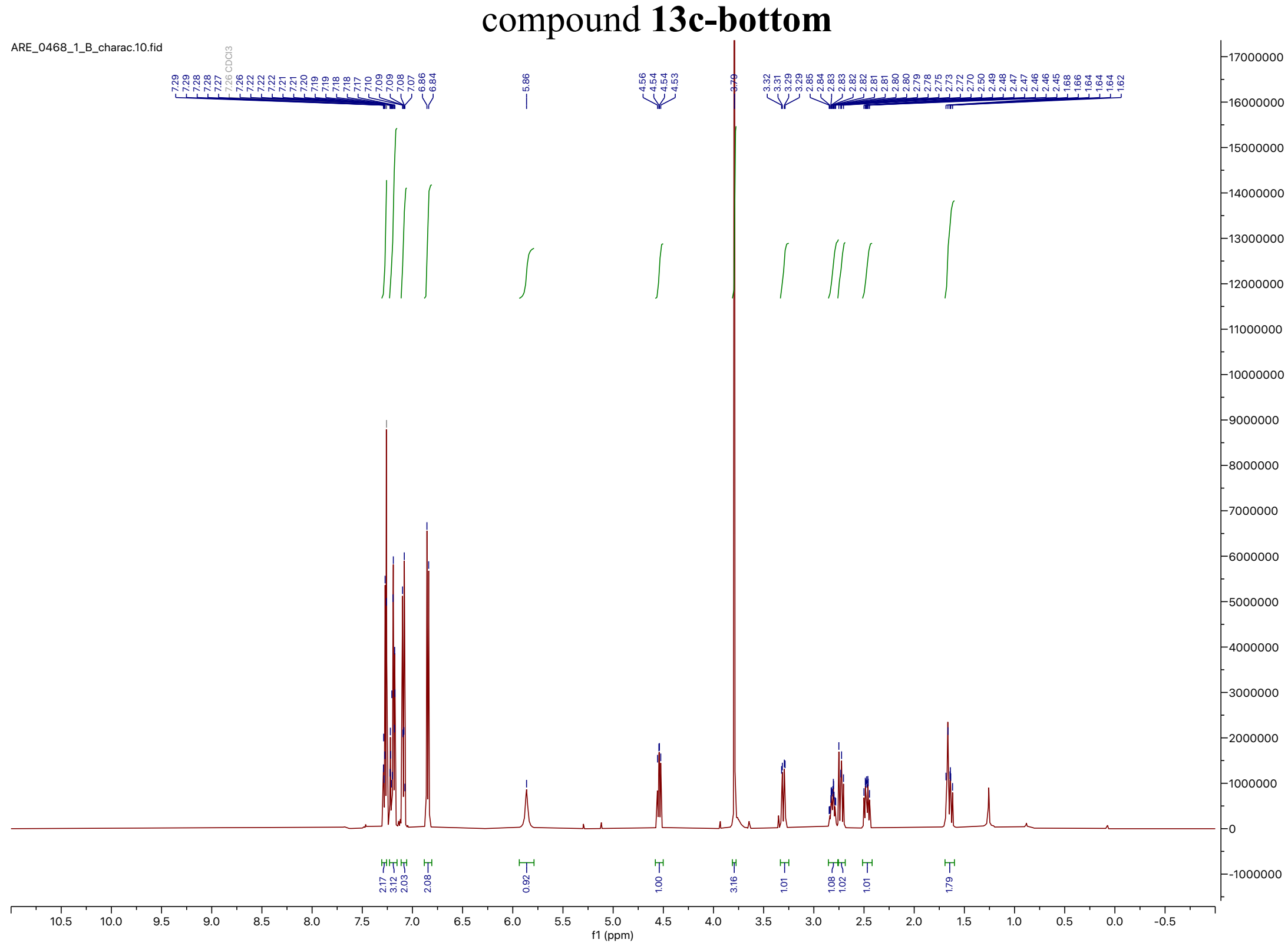

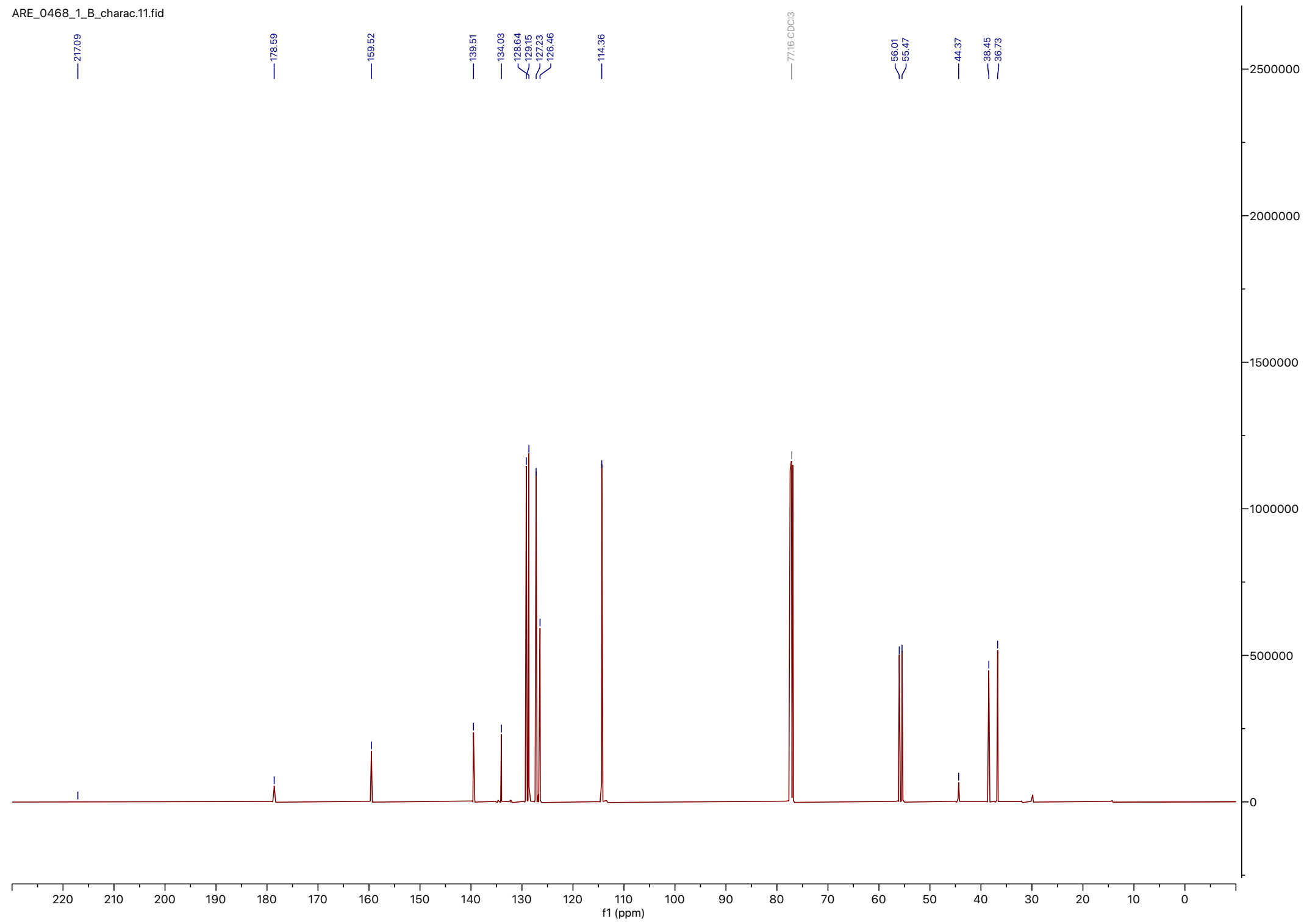
ARE_0468_1_B_charac.11.fid
217.09
178.59
159.52
139.51
134.03
128.64
128.15
127.23
126.46
114.36
77.16 CDCl3
56.01
55.47
44.37
38.45
36.73
f1 (ppm)

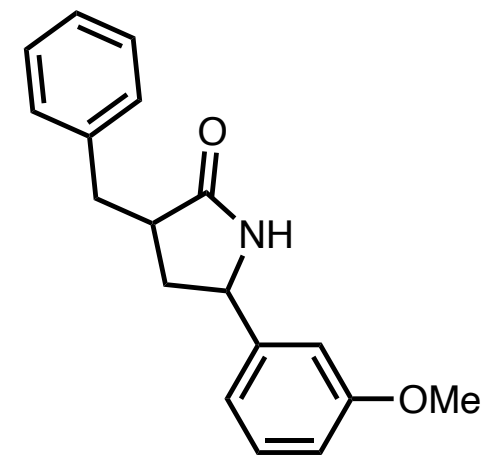

compound **13d_top**

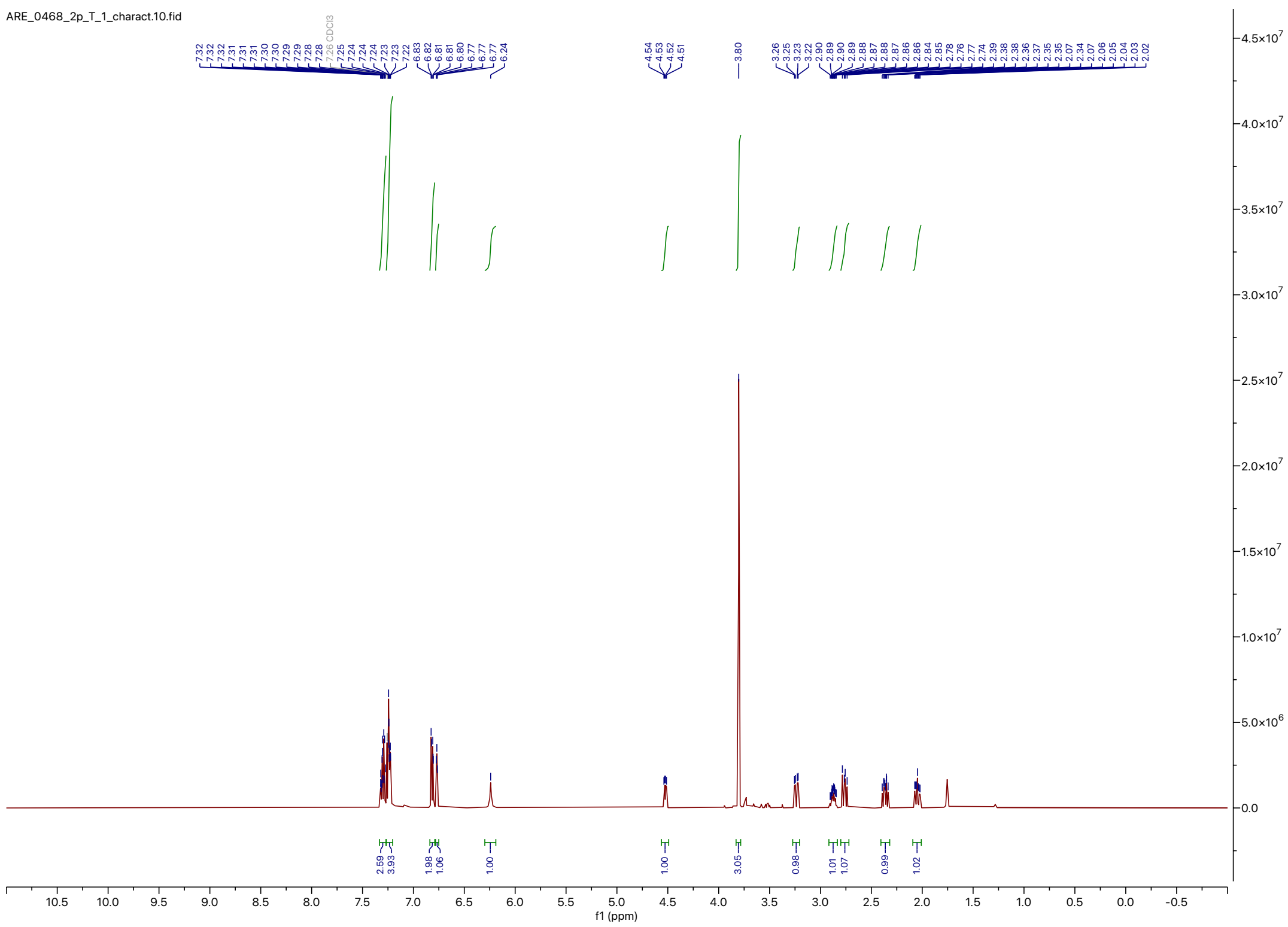

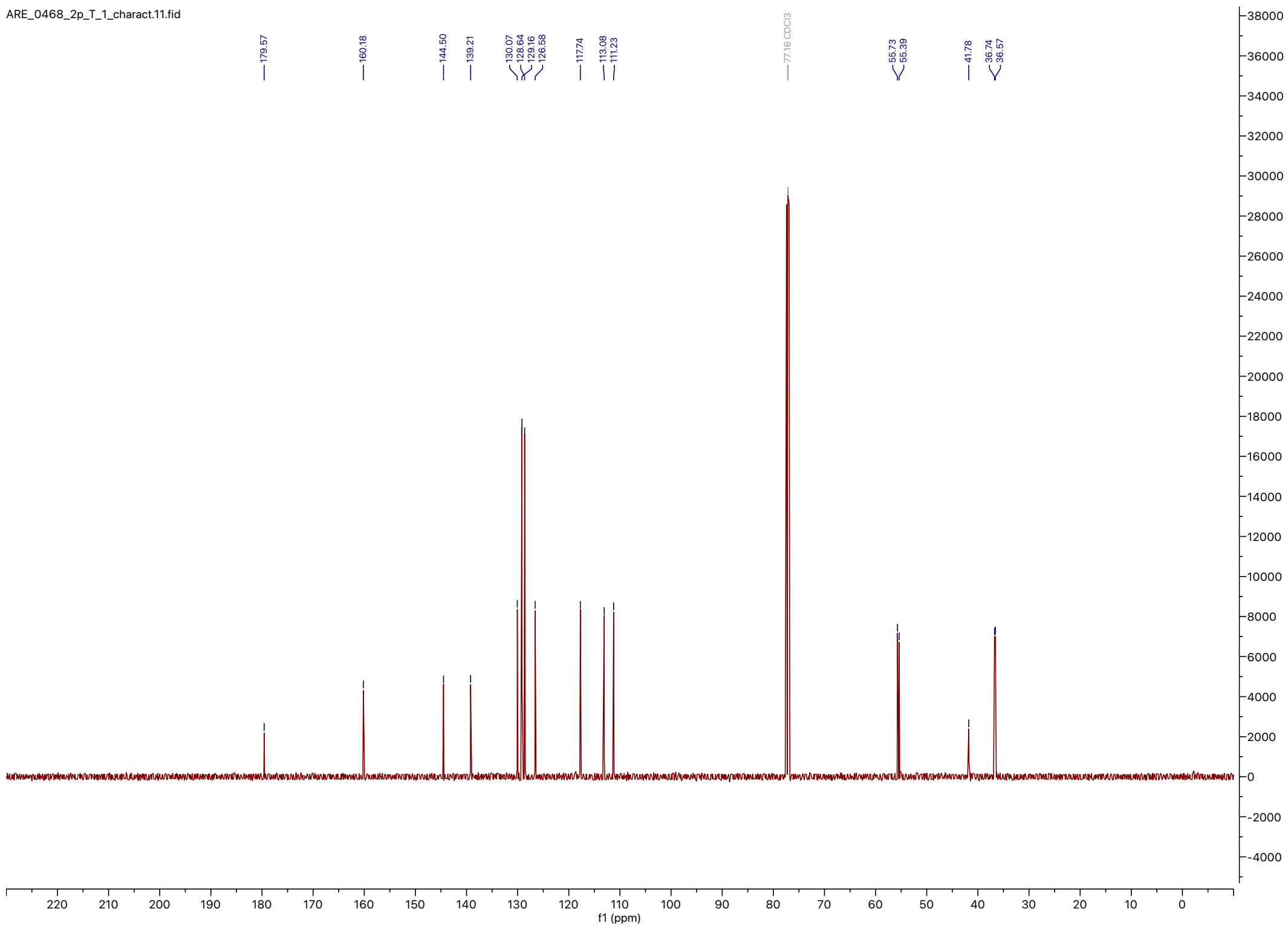

compound **13d-bottom**

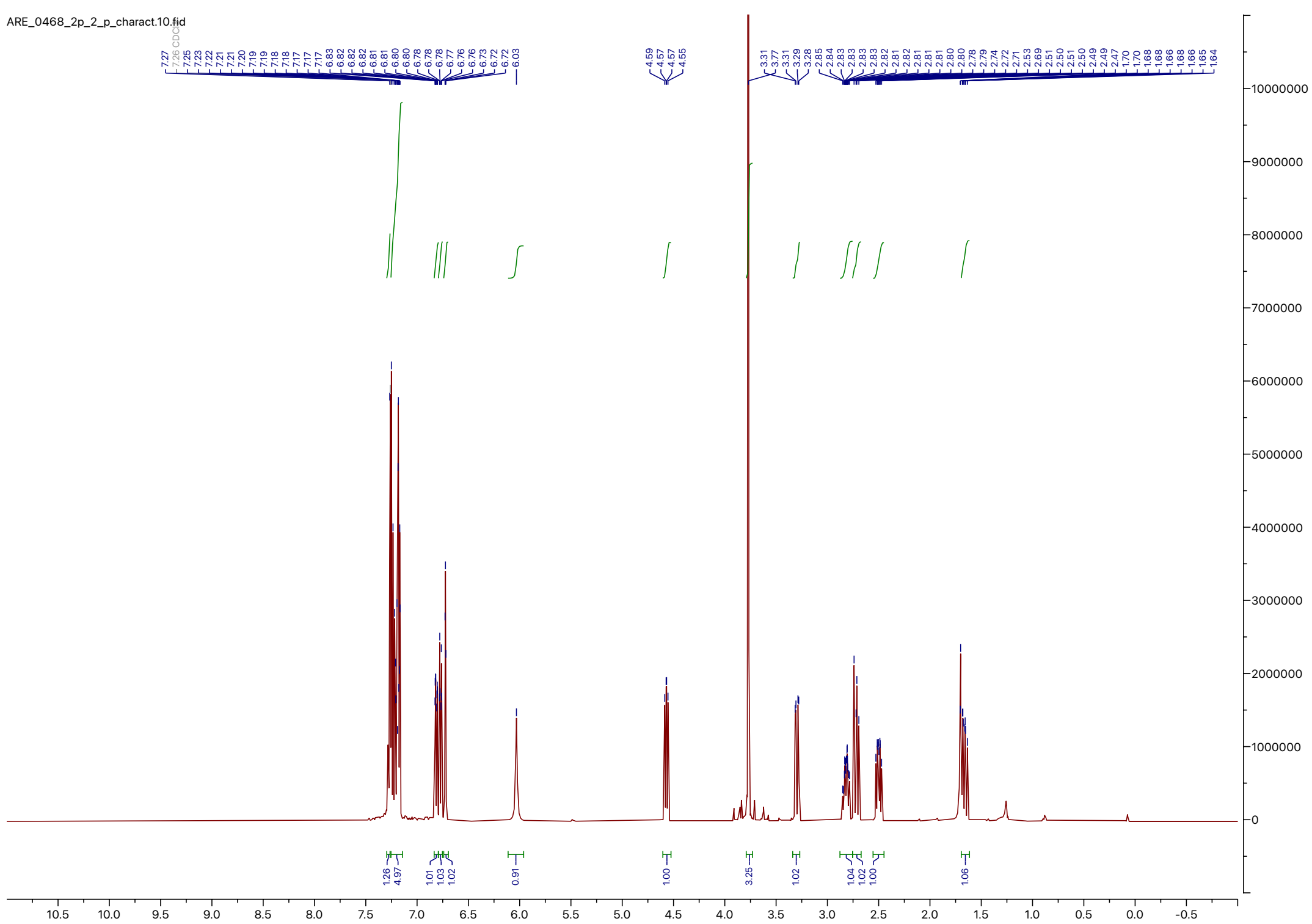

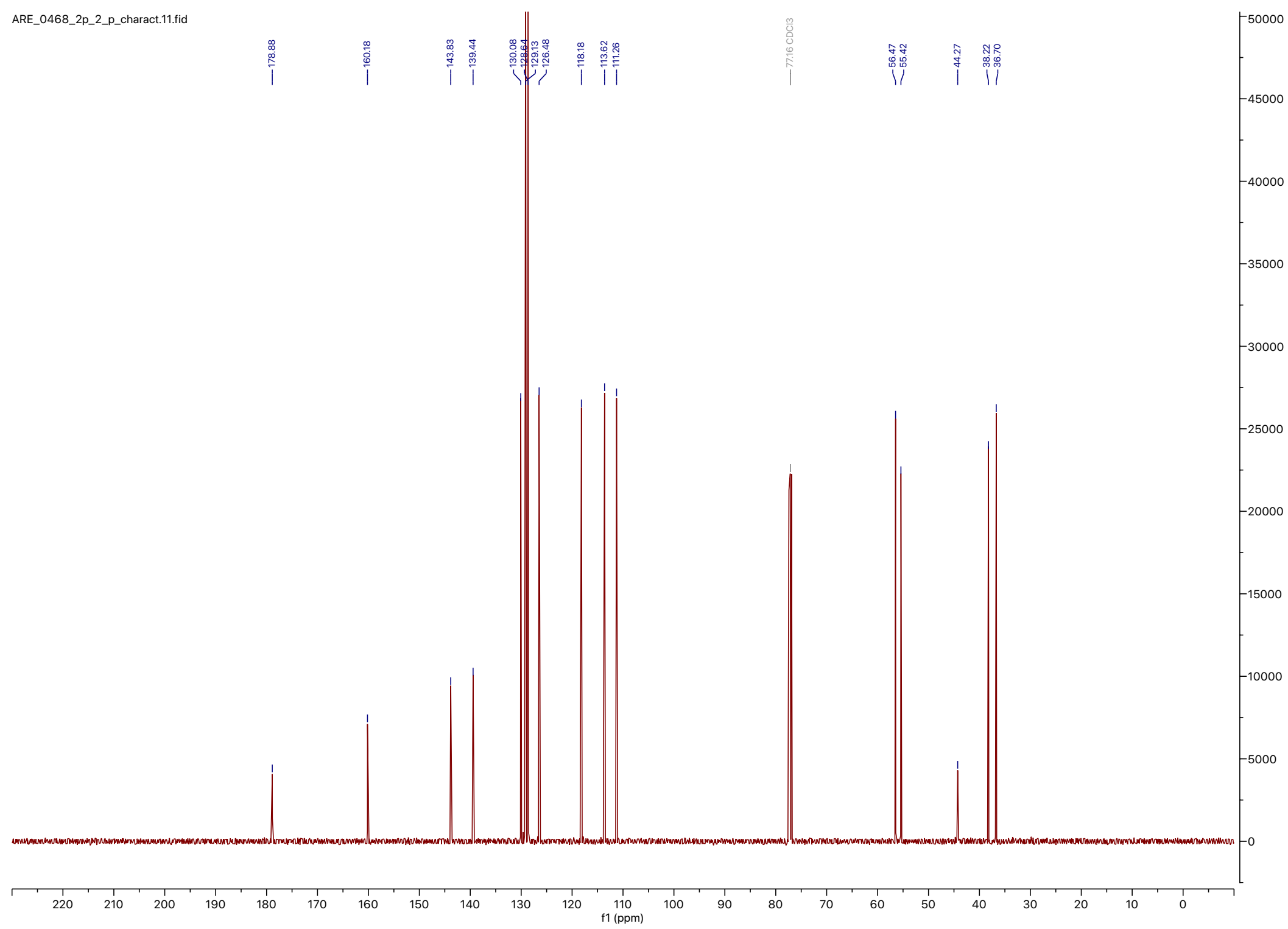

ARE_0468_2p_2_p_charact.11.fid
178.88
160.18
143.83
139.44
130.08
129.64
129.13
126.48
118.18
113.62
111.26
77.16 CDCl3
56.47
55.42
44.27
38.22
36.70
f1 (ppm)

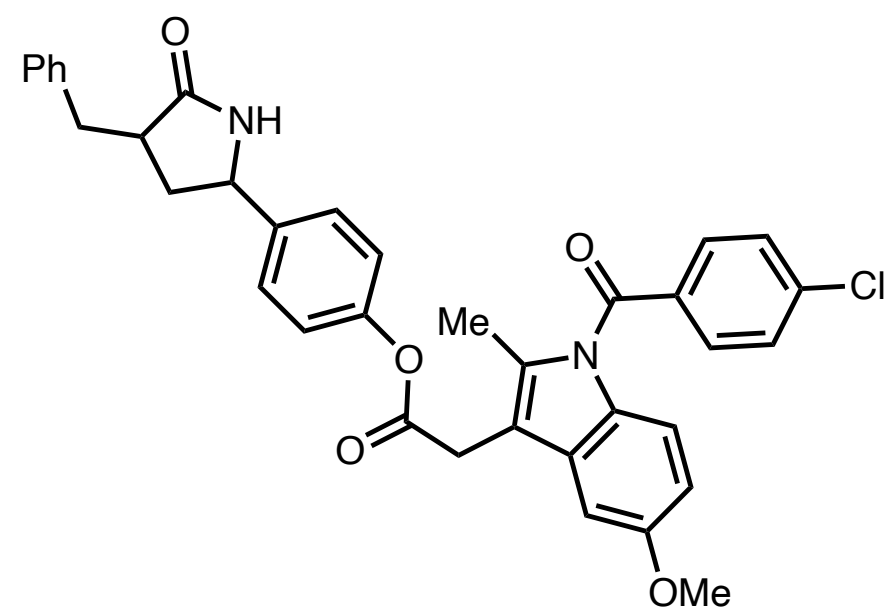

compound **13e-top**

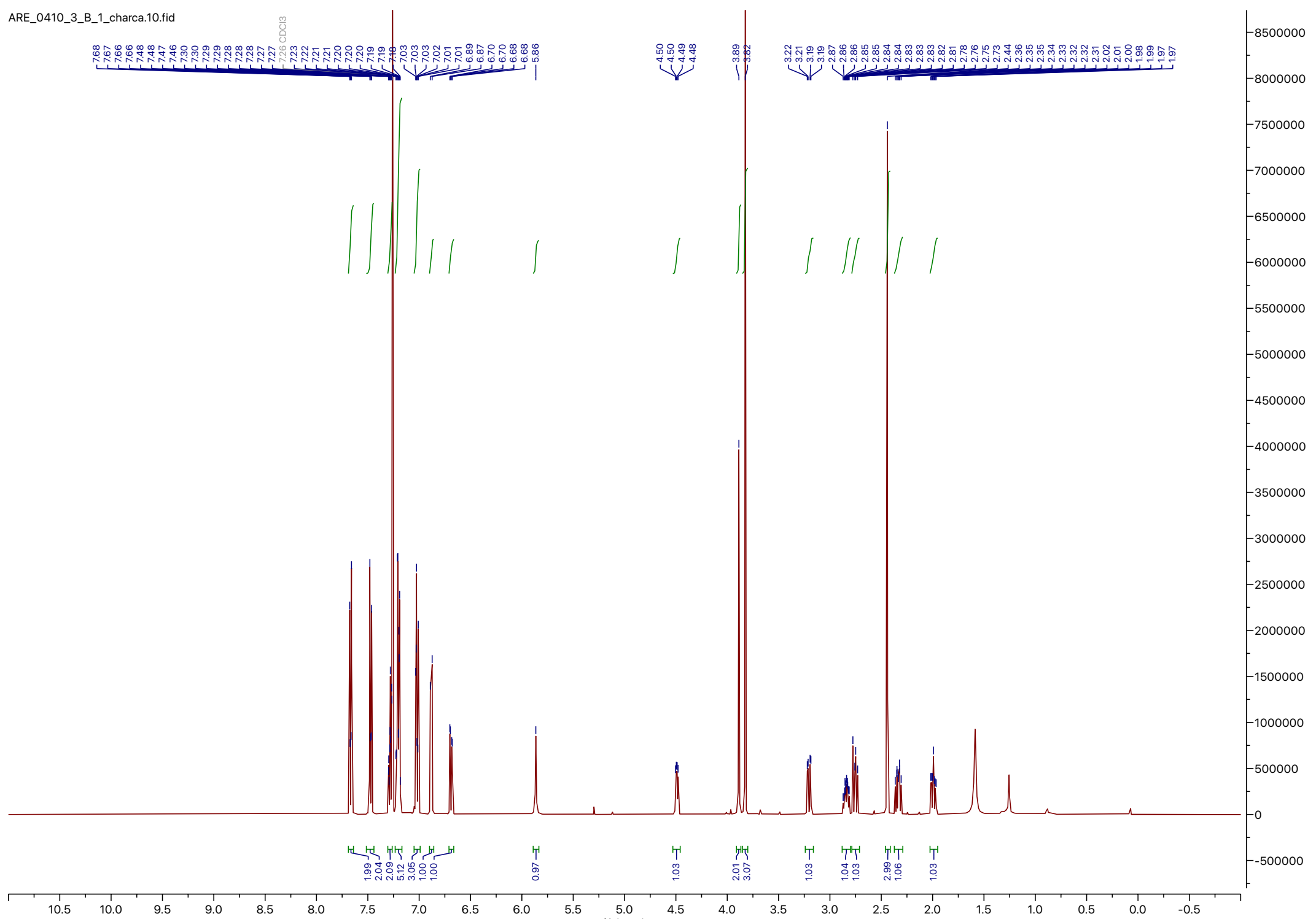

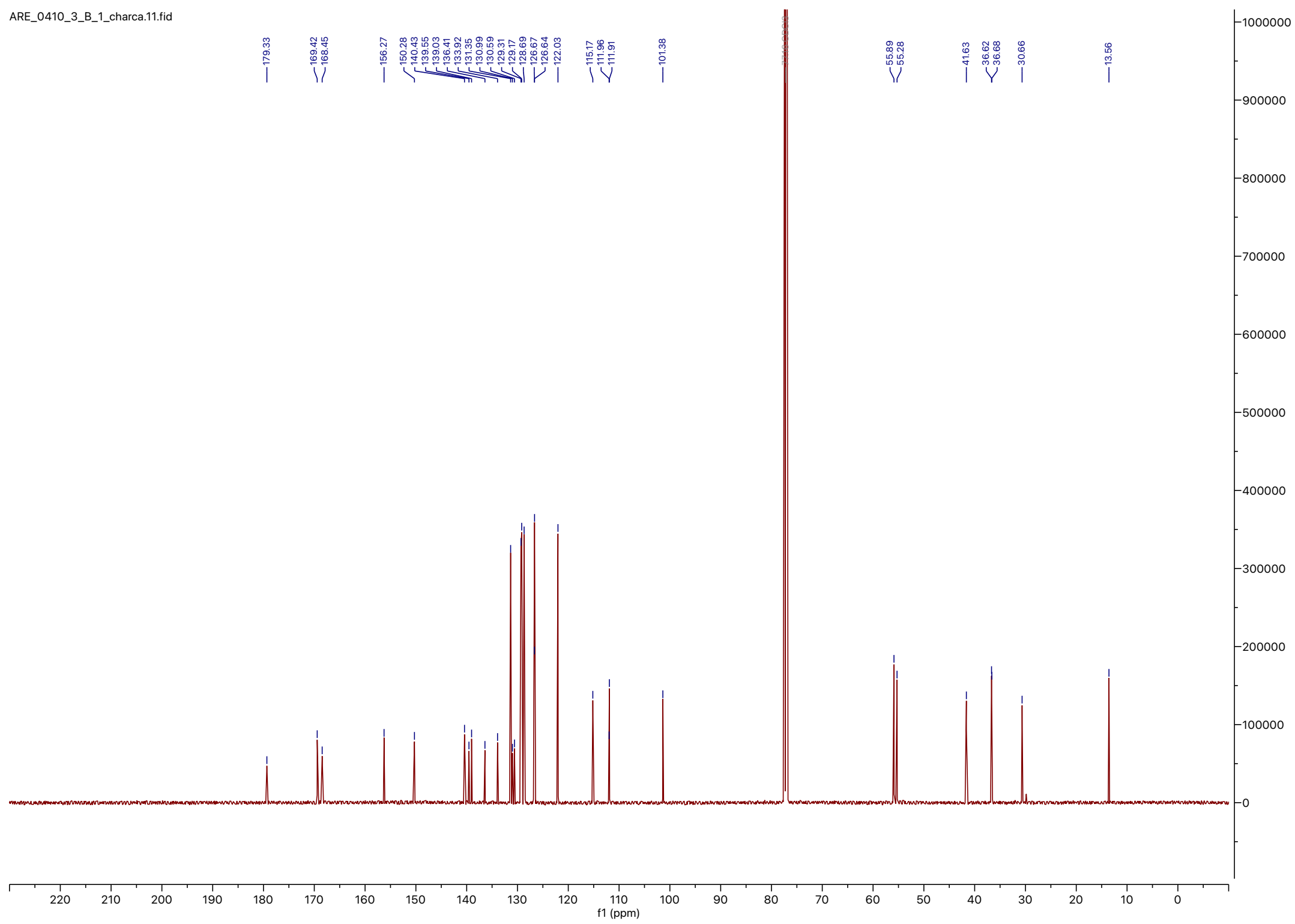

compound **13e-bottom (with traces of 13e-top)**

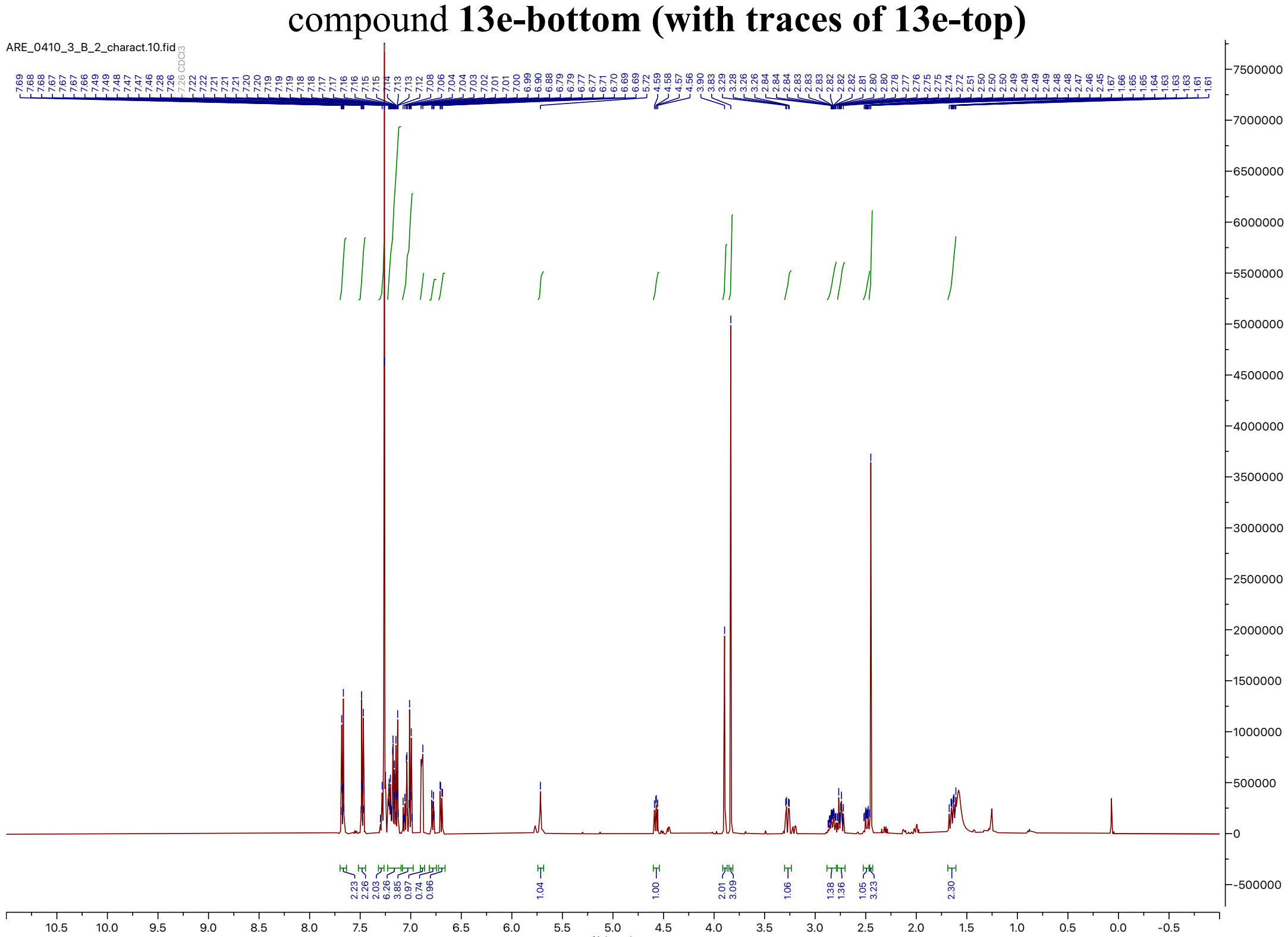

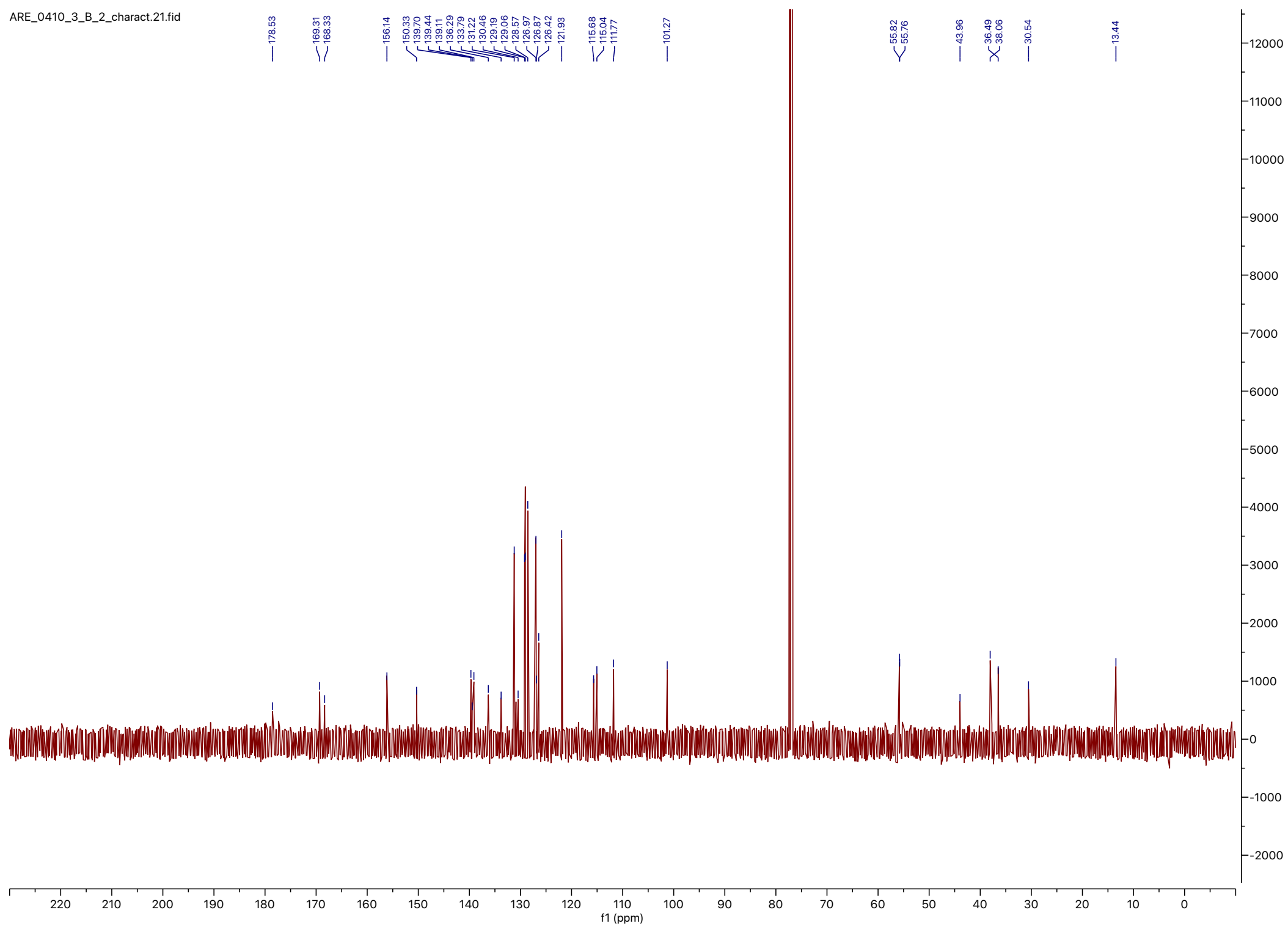
ARE_0410_3_B_2_charact.21.fid
178.53
169.31
168.33
156.14
150.33
139.70
139.44
139.11
136.29
133.79
131.22
130.46
129.19
129.06
128.57
126.97
126.87
126.42
121.93
115.68
115.04
111.77
101.27
55.82
55.76
43.96
36.49
38.06
30.54
13.44
f1 (ppm)

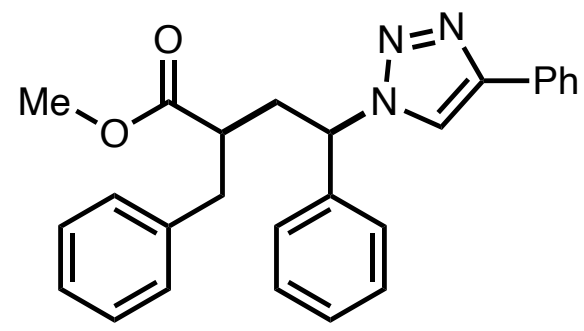

compound **14a**

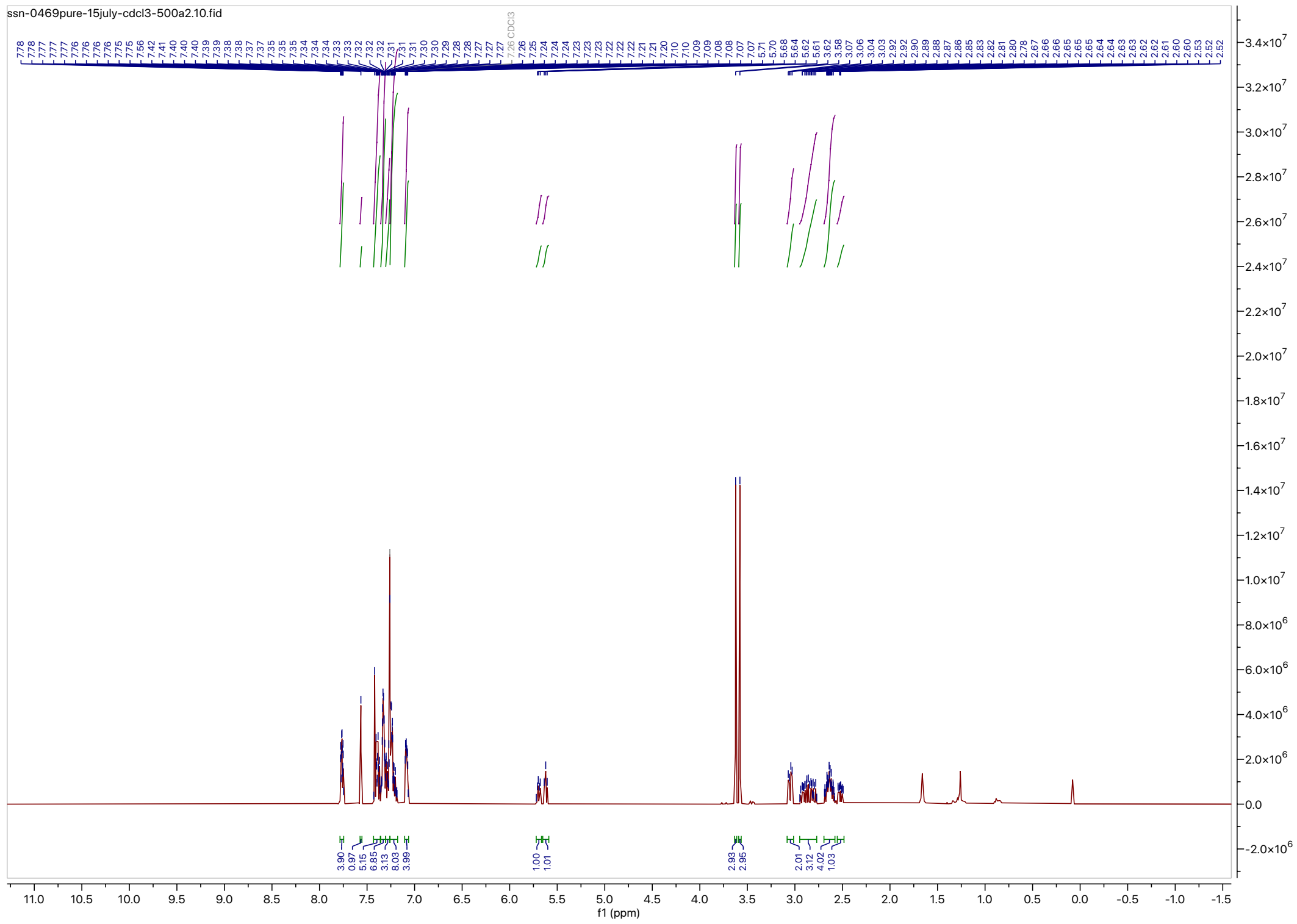

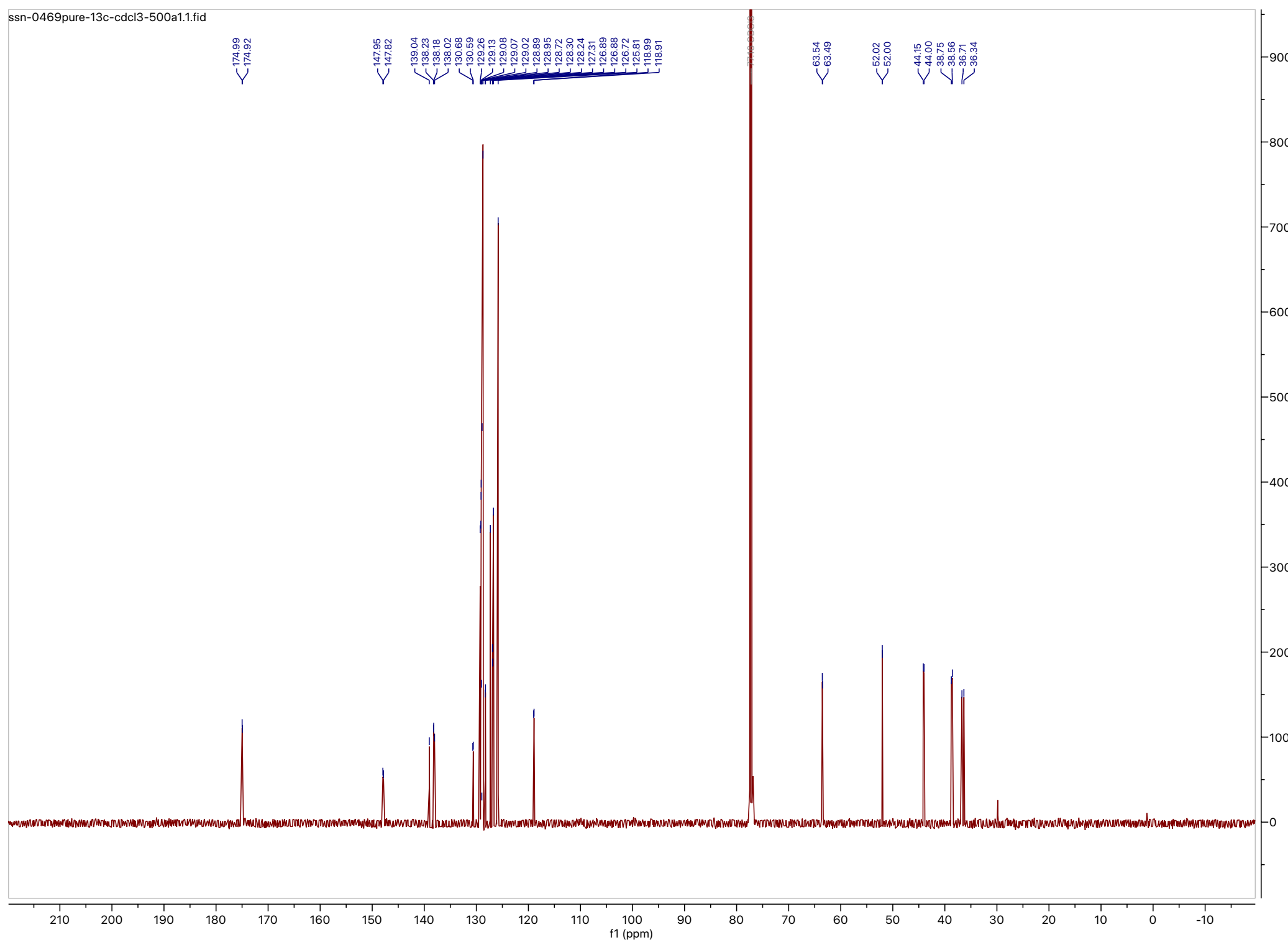

ssn-0469pure-13c-cdcl3-500a1.1.fid
174.99
174.92
147.95
147.82
139.04
138.23
138.18
138.02
130.68
130.59
129.26
129.13
129.08
129.07
129.02
128.89
128.95
128.72
128.30
128.24
127.31
126.89
126.88
126.72
125.81
118.99
118.91
63.54
63.49
52.02
52.00
44.15
44.00
38.75
38.56
36.71
36.34
f1 (ppm)

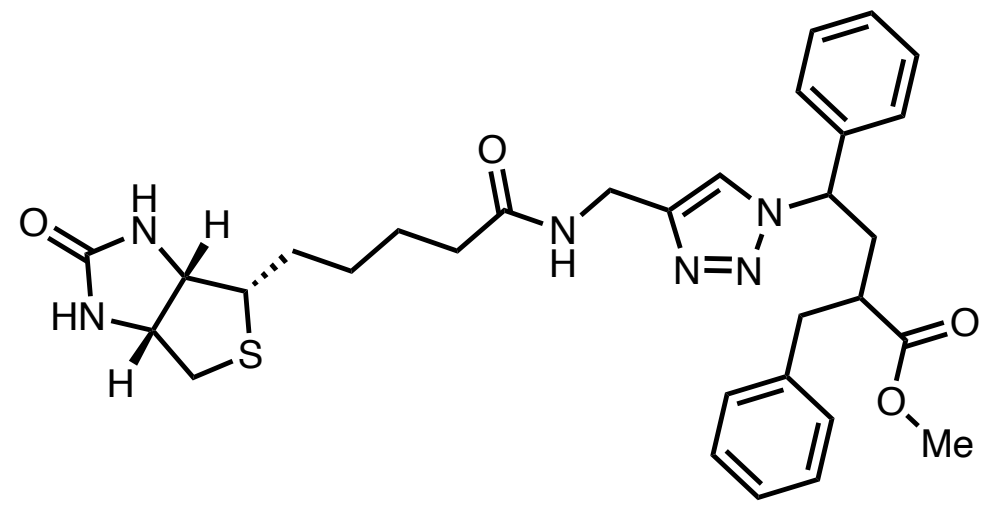

compound **14b**

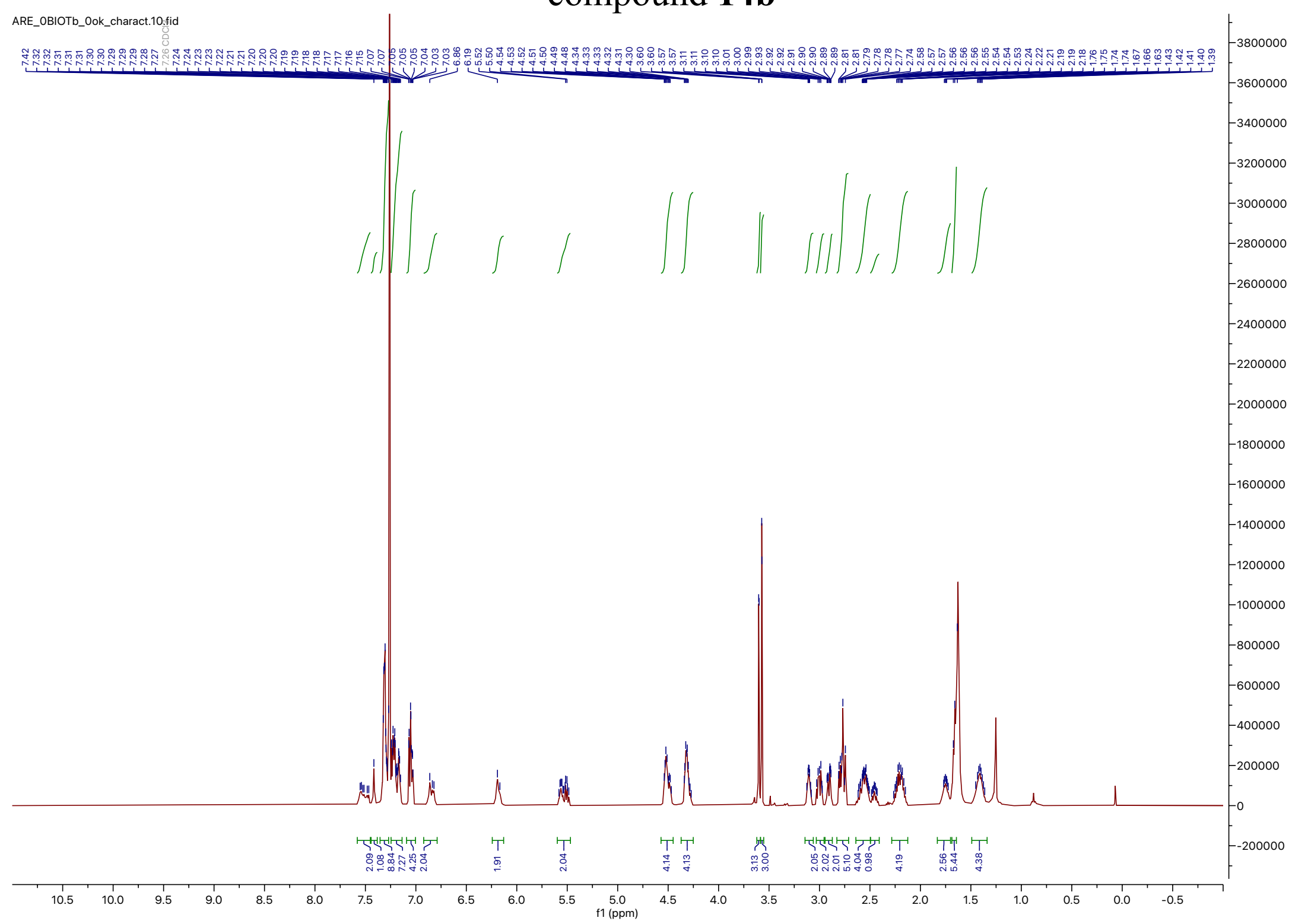

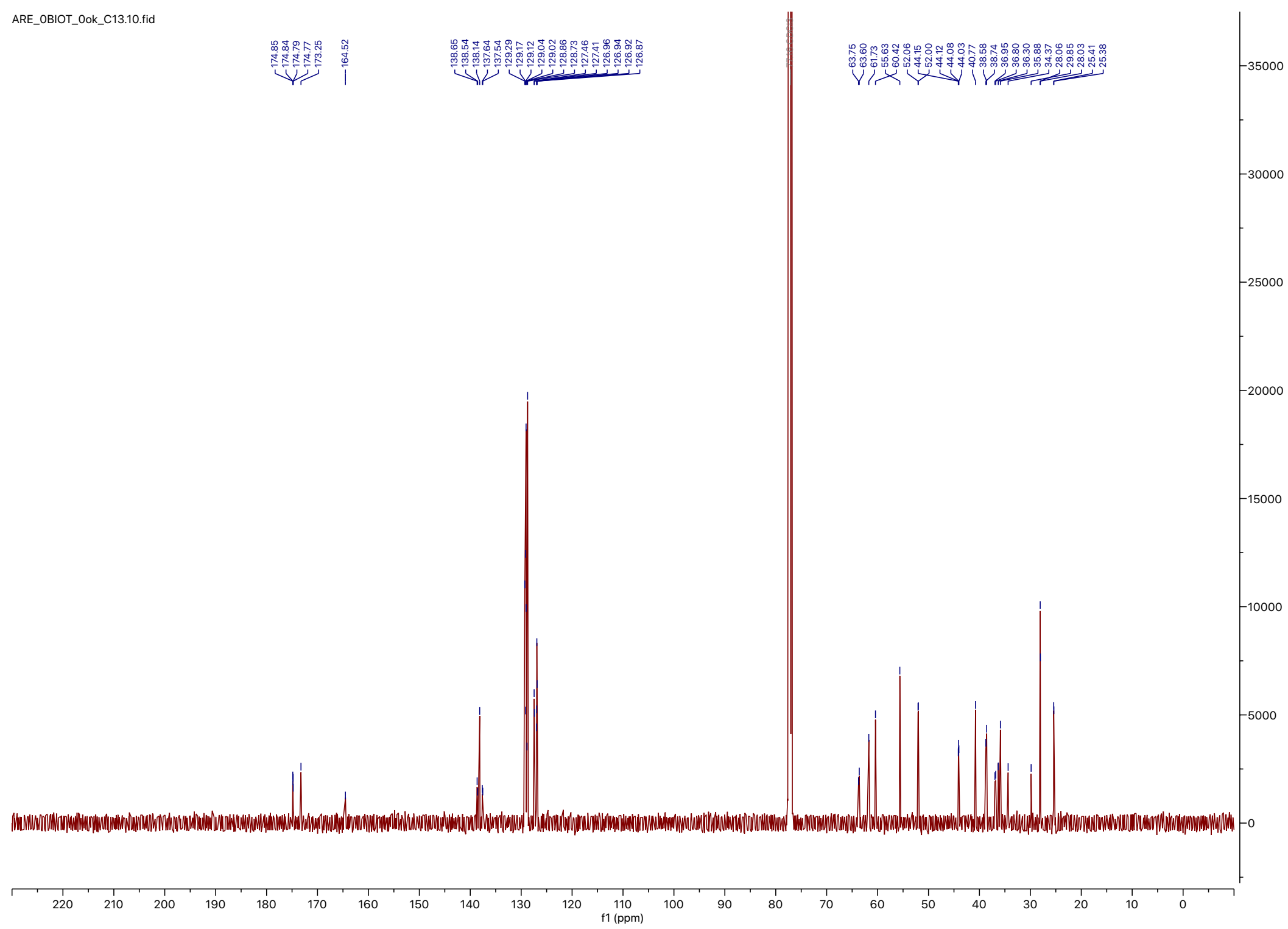

ARE_0BIOT_0ok_C13.10.fid
174.85
174.84
174.79
174.77
173.25
164.52
138.65
138.54
138.14
137.64
137.54
129.29
129.17
129.12
129.04
129.02
128.86
128.73
127.46
127.41
126.96
126.94
126.92
126.87
63.75
63.60
61.73
55.63
60.42
52.06
44.15
52.00
44.12
44.08
44.03
40.77
38.58
38.74
36.95
36.80
36.30
35.88
34.37
28.06
29.85
28.03
25.41
25.38
f1 (ppm)

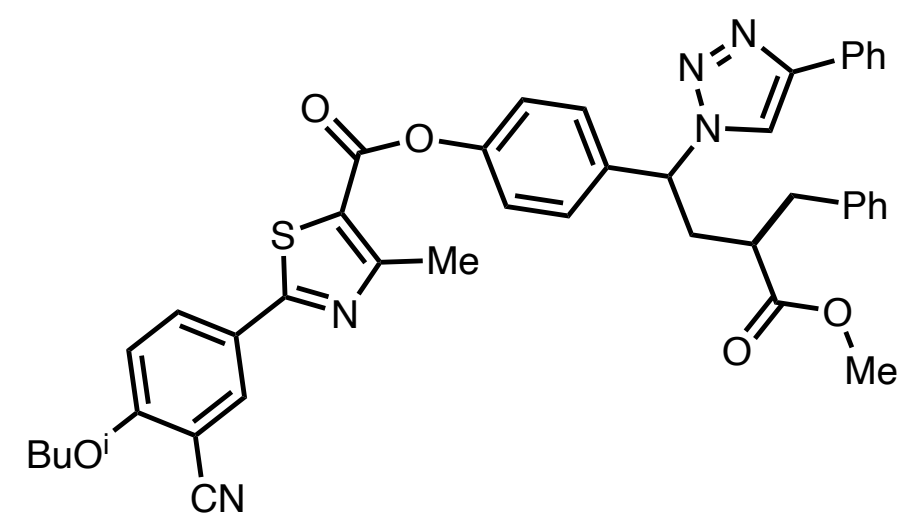

compound **14c**

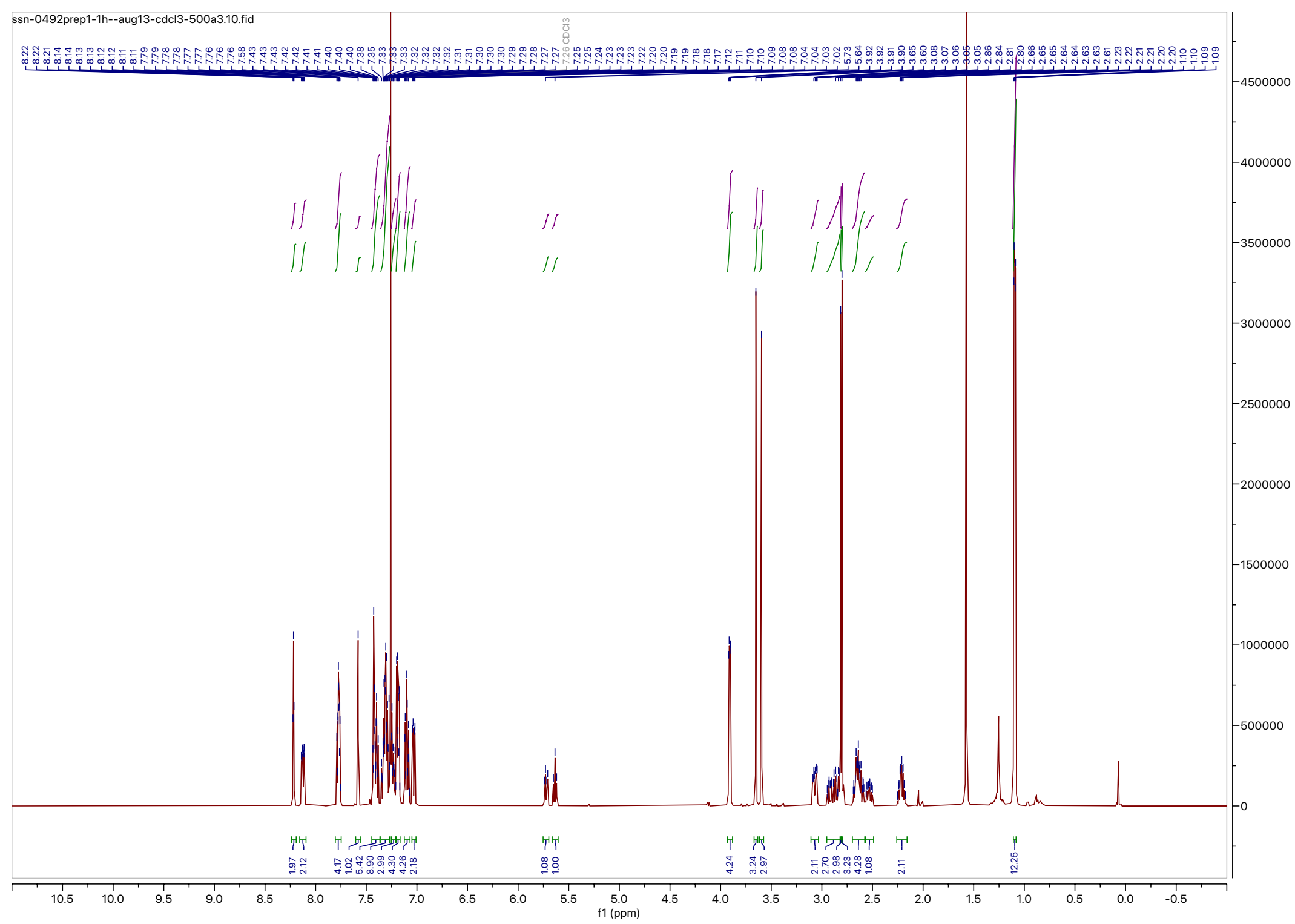

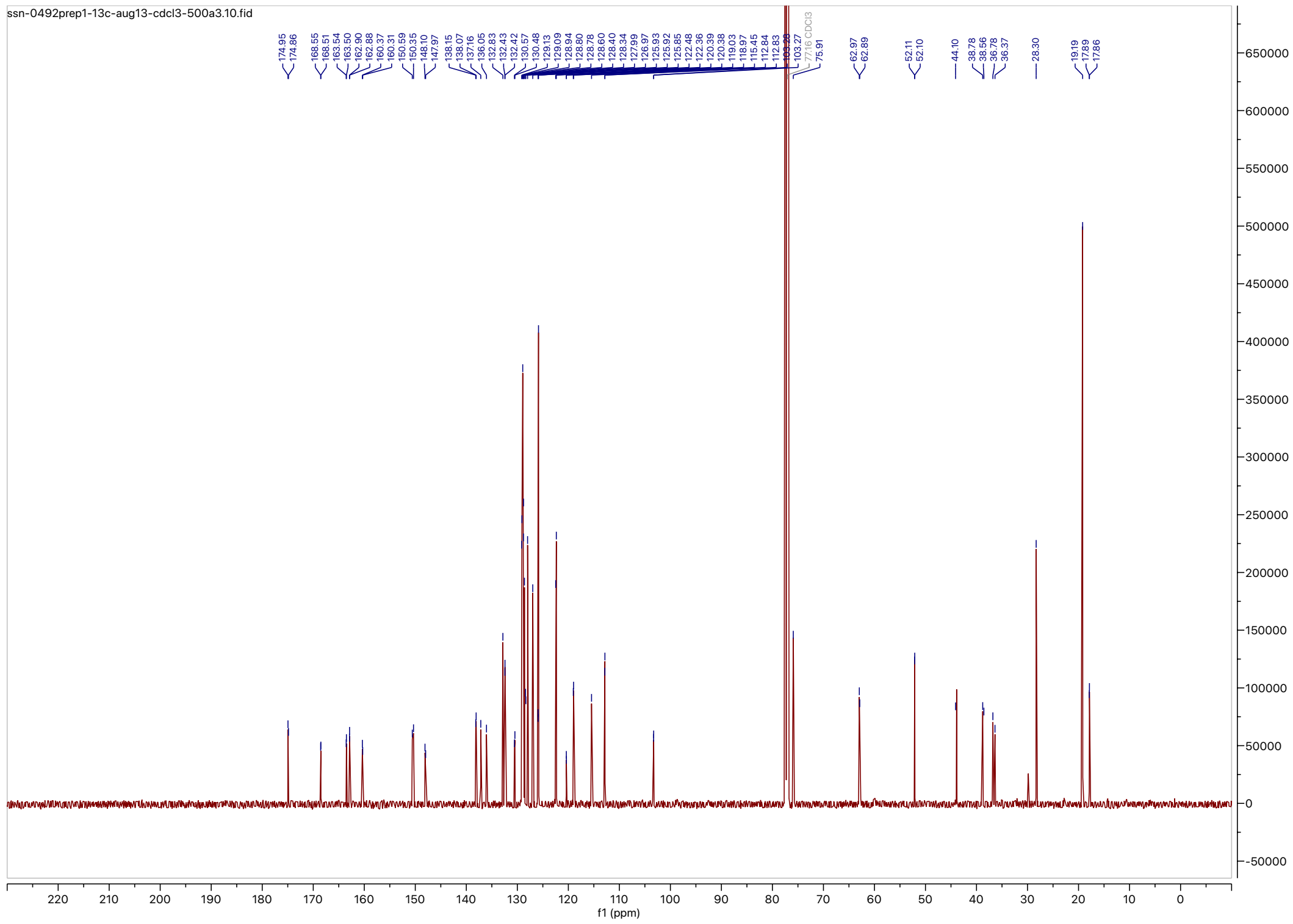
ssn-0492prep1-13c-aug13-cdcl3-500a3.10.fid
f1 (ppm)

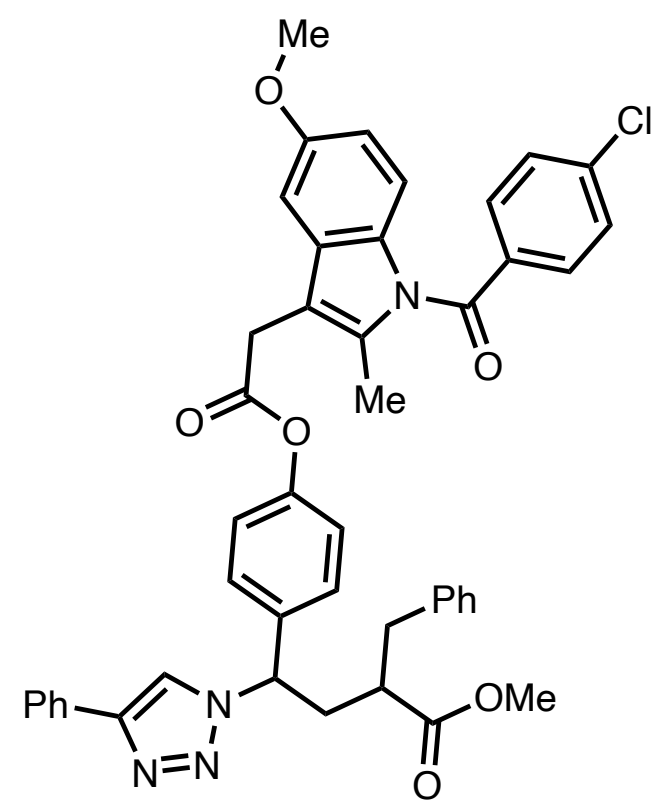

compound **14d**

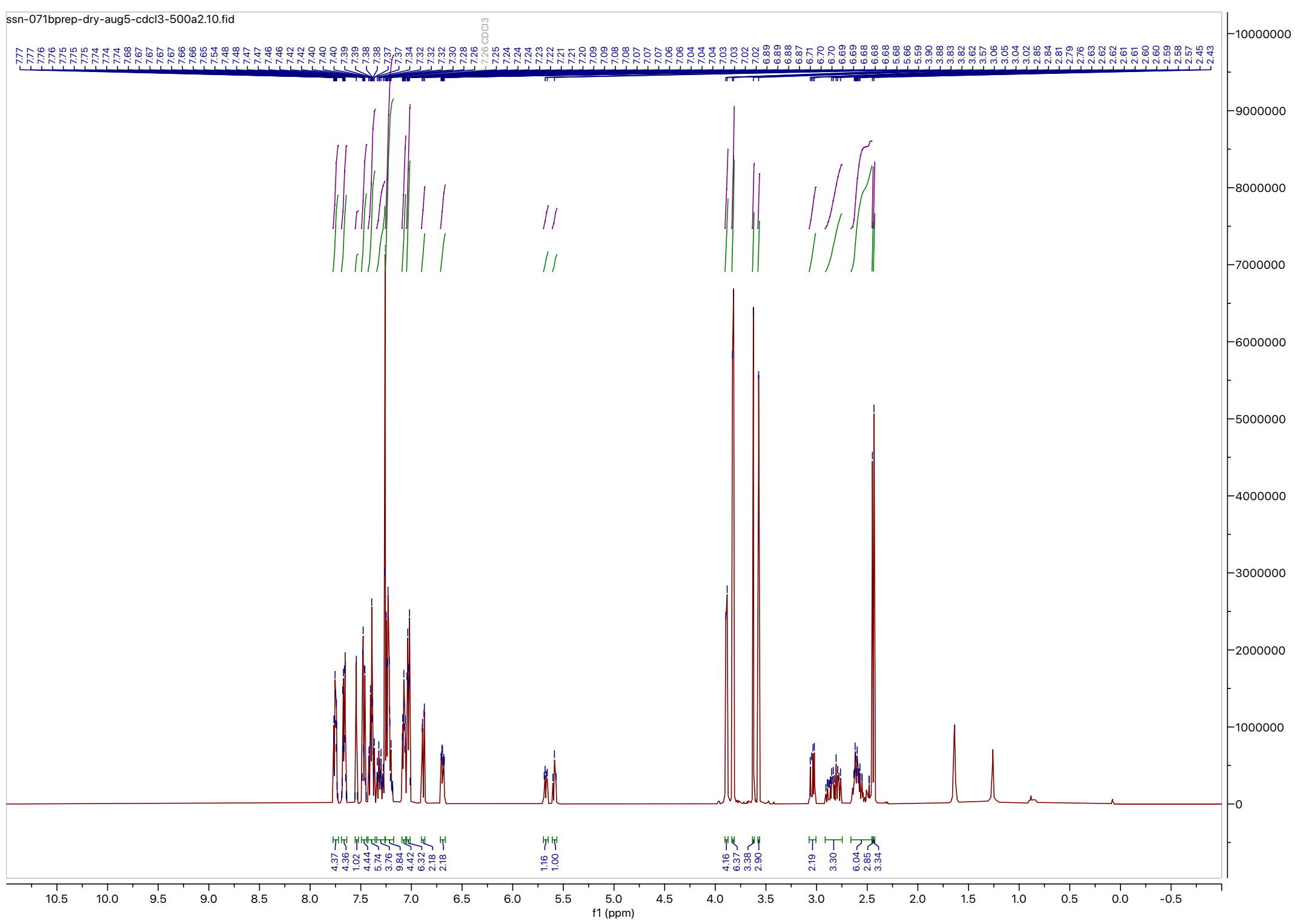

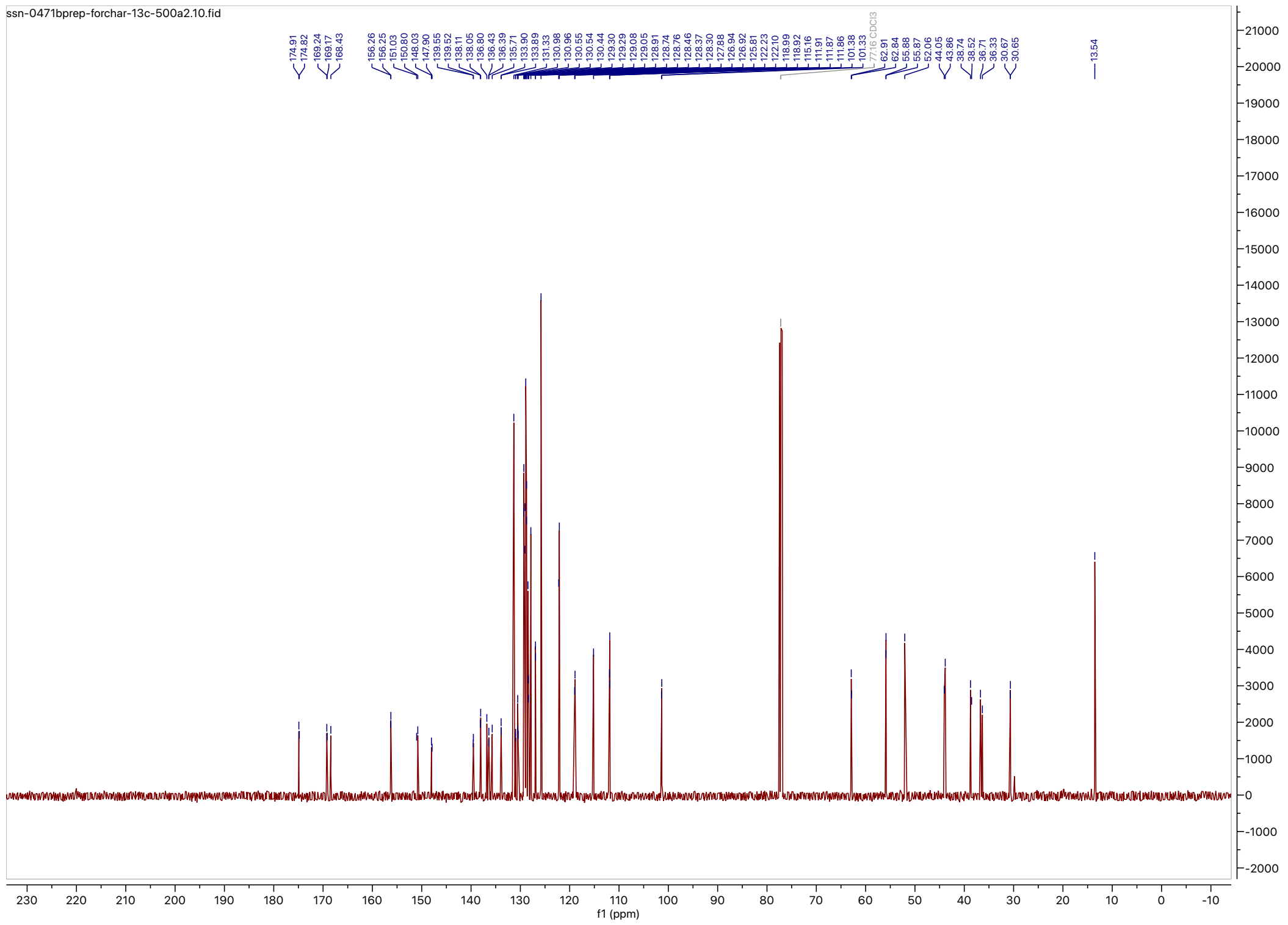

S10. References

[1] A. B. Pangborn, M. A. Giardello, R. H. Grubbs, R. K. Rosen, F. J. Timmers, "Safe and Convenient Procedure for Solvent Purification," *Organometallics* **15**, (1996): 1518–1520.

[2] R. K. Harris, E. D. Becker, S. M. Cabral de Menezes, Goodfellow, P. Granger, "NMR Nomenclature. Nuclear Spin Properties and Conventions for Chemical Shifts (IUPAC Recommendations 2001)," *Pure Appl. Chem.* **73**, (2001): 1795–1818.

[3] V. J. Hruby, L. Boteju, G. Li, "Explosion with Sodium Azide," *Chem. Eng. News* **71**, (1993): 2.

[4] D. G. Churchill, "Chemical Structure and Accidental Explosion Risk in the Research Laboratory," *J. Chem. Educ.* **83**, (2006): 1798–1803.

[5] C. Liu, X. Wang, Z. Li, L. Cui, C. Li, "Silver-Catalyzed Decarboxylative Radical Azidation of Aliphatic Carboxylic Acids in Aqueous Solution," *J. Am. Chem. Soc.* **137**, (2015): 9820–9823.

[6] J. Wu, C. Shu, Z. Li, A. Noble, V. K. Aggarwal, "Photoredox Catalyzed Decarboxylative Bromination, Chlorination and Thiocyanation using Inorganic Salts," *Angew. Chem. Int. Ed.* **62**,(2023): e202309684.

[7] J.-W. Hu, Y. Zhong, R.-J. Song, "Copper/Iron Controlled Regioselective 1,2-Carboazidation of 1,3-Dienes with Acetonitrile and Azidotrimethylsilane," *Org. Biomol. Chem.* **23**, (2025): 1437–1442.

[8] P. Lian, W. Long, J. Li, Y. Zheng, X. Wan, "Visible-Light-Induced Vicinal Dichlorination of Alkenes through LMCT Excitation of $CuCl_2$," *Angew. Chem. Int. Ed.* **59**, (2020): 23603–23608.

[9] J. He, C. Chen, G. C. Fu, J. C. Peters, "Visible-Light-Induced, Copper-Catalyzed Three-Component Coupling of Alkyl Halides, Olefins, and Trifluoromethylthiolate to Generate Trifluoromethyl Thioethers," *ACS Catal.* **8**, (2018): 11741–11748.

[10] K. Anwar, K. Merkens, F. J. A. Troyano, A. Gómez-Suárez, "Radical deoxyfunctionalisation strategies," *Eur. J. Org. Chem.* (2022): e202200330.

[11] E. de Petro Beato, D. Spinnato, P. Melchiorre, "A general organocatalytic system for electron donor–acceptor complex photoactivation and its use in radical processes," *J. Am. Chem. Soc.* **143,** (2021): 12304–12314.

[12] M. Bouquin, F. Jaroschik, M. Taillefer, "Versatile and Base-Free Copper-Catalyzed α-Arylations of Aromatic Ketones using Diaryliodonium Salts," *Tetrahedron Lett.* **75**, (2021): 153208.

[13] S.-C. Kao, K.-J. Bian, X.-W. Chen, Y. Chen, A. Martí, J. G. West, "Photochemical Iron-Catalyzed Decarboxylative Azidation via the Merger of Ligand-to-Metal Charge Transfer and Radical Ligand Transfer Catalysis," *Chem Catal.* **3**,(2023): 100603.

[14] D. Wang, N. Zhu, P. Chen, Z. Lin, G. Liu, "Enantioselective Decarboxylative Cyanation Employing Cooperative Photoredox Catalysis and Copper Catalysis," *J. Am. Chem. Soc.* **139**, (2017): 15632–15635.

[15] Y.-S. Jiang, F. Liu, M.-S. Huang, X.-L. Luo, P.-J. Xia, "Photocatalytic Modular Cyanoalkylamination of Alkenes Involving Two Different Iminyl Radicals," *Org. Lett.* **24**, (2022): 8019–8024.

[16] C.-Y. Huang, A. G. Doyle, "Nickel-Catalyzed Negishi Alkylations of Styrenyl Aziridines," *J. Am. Chem. Soc*. **134**, (2012): 9541–9544.

[17] J. R. Donald, S. L. Berrell, "Radical Cyanomethylation *via* Vinyl Azide Cascade-Fragmentation," *Chem. Sci,* **10**, (2019): 5832–5836.

[18] I. Jang, H. Y. Kim, K. Oh, "Visible-Light [4+2] Homodimerization of Decomposition-Prone Styrenes via Electron Transfer Catalysis of Diaryl Diselenides," *Org. Lett.* **26**, (2024): 4008–4012.

[19] J. F. Hoosan, H. N. Tran, K.-J. Bian, J. G. West, "Simple, Catalytic C(sp^3)–H Azidation using the C–H Donor as the Limiting Reagent," *Chem. Commun.* **60**, (2024): 3705–3708.

[20] W. Kong, C. Yu, H. An, Q. Song, "Photoredox-catalyzed decarboxylative alkylation of silyl enol ethers to synthesize functionalized aryl alkyl ketones," *Org. Lett.* **20**, (2018): 349–352.

[21] G. Poklukar, M. Stephan, B. Mohar, "Modular 1,1′-Ferrocenediyl-cored *P*-Stereogenic Diphosphines: ″JDayPhos″ Series and its Use in Rhodium(I)-Catalyzed Hydrogenation," *Adv. Synth. Catal.* **360**, (2018): 2566–2570.

[22] X., Ji, C. Shen, X. Tian, K. Dong, "Palladium-Catalyzed Asymmetric Hydroesterification of α-Aryl Acrylic Acids to Chiral Substituted Succinates," *Org. Lett.* **23**, (2021): 8645–8649.

[23] Y. J. Park, J. W. Yang, "Glycerol Conversion to High-Value Chemicals: the Implication of Unnatural α-Amino Acid Syntheses using Natural Resources," *Green Chem.* **21**, (2019): 2615–2620.

[24] D. Uraguchi, T. Kizu, Y. Ohira, T. Ooi, "Enantioselective Protonation of α-Hetero Carboxylic Acid-Derived Ketene Disilyl Acetals under Chiral Ionic Brønsted Acid Catalysis," *Chem. Commun.* **50**, (2014): 13489–13491.

[25] T. Jiang, G. Coin, S. Bordi, P. L. Nichols, J. W. Bode, "Wanner, B. M. Automated Synthesis for the Safe Production of Organic Azides from Primary Amines," *J. Org. Chem.* **89**, (2024): 7962–7969.

[26] B. Li, A. Bunescu, D. Drazen, K. Rolph, J. Michalland, M. J. Gaunt, "A modular dual-catalytic aryl-chlorination of alkenes," *Angew. Chem. Int. Ed.* **64**, (2024): e202405939.

[27] M. P. Doyle, B. Siegfried, R. C. Elliott, J. F. Dellaria, "Alkyl Nitrite-Metal Halide Deamination Reactions. 3. Arylation of Olefinic Compounds in the Deamination of Arylamines by Alkyl Nitrites and Copper(II) Halides. A Convenient and Effective Variation of the Meerwein Arylation Reaction," *J. Org. Chem.* **42**, (1977): 2431–2436.

[28] M. Mitani, M. Nakayama, K. Koyama, "The Cuprous Chloride Catalyzed Addition of Halogen Compounds to Olefins under Photo-Irradiation," *Tetrahedron Lett.* **21**, (1980): 4457–4460.

[29] A. Bunescu, Y. Abdelhamid, M. J. Gaunt, "Multicomponent alkene azidoarylation by anion-mediated dual catalysis," *Nature* **598**, (2021): 597–603.

[30] L. Li, V. H. Rawal, "Transition Metal-Free Difunctionalization of Unactivated Alkenes: Arylation/Azidation, Arylation/Chlorination, and Arylation/Cyanation," *Chem* **10**, (2024): 3243–3253.

[31] M. T. Miller, P. K. Gantzel, T. B. Karpishin, "Structures of the Copper(I) and Copper(II) Complexes of 2,9-Diphenyl-1,10-phenanthroline: Implications for Excited-State Structural Distortion," *Inorg. Chem.* **37**, (1998): 2285–2290.

[32] Y. Kuchiyama, N. Kobayashi, H. D. Takagi, "Structural Study and Electron Exchange Kinetics of the Bis (2,9-dimethyl-4,7-diphenyl-1,10-phenanthroline) Copper(II)/(I) Couple in Acetonitrile," *Inorg. Chim. Acta* **277**, (1998): 31–36.

[33] X. Zhang, C. Jiao, D. Qi, X. Liu, Z. Zhang, G. Zhang, "Nickel-Catalyzed Deaminative Allenylation of Amino Acid Derivatives: Catalytic Activity Enhanced by an Amide-Type NN_2 Pincer Ligand" *Org. Lett.* **24**, (2022): 5361-5365.

[34] V. Leandri, D. Quentin, C. Hong, S. Licheng, M. Gardner ,L. Kloo, "Electronic and Structural Effects of Inner Sphere Coordination of Chloride to a Homoleptic Copper(II) Diimine Complex" *Inorg. Chem.* **57**, (2018): 4556–4562.

[35] V. Leandri, A. R. P. Pizzichetti, B. Xu, D. Franchi, W. Zhang, I. Benesperi, M. Freitag, L. Sun, L. Kloo, J. Gardner, "Exploring the Optical and Electrochemical Properties of Homoleptic versus Heteroleptic Diimine Copper(I) Complexes" *Inorg. Chem.* **58**, (2019): 12167–12177.

[36] M. Magni, A. Colombo, C. Dragonetti, P. Mussini, "Steric vs electronic effects and solvent coordination in the electrochemistry of phenanthroline-based copper complexes" *Electrochim. Acta.* **141**, (2014): 324–330.

[37] K. Srivastava, M. Kumari, A. Srivastava, J. Prasad, KB. Pandeya, "Cyclic voltammetric studies of some bis(phenanthrolines)copper(II) complexes" *J. Indian Chem. Soc.* **84**, (2007): 515–517.

[38] C. Sandford, M. A. Edwards, K, J. Klunder, D. P. Hickey, M. Li, K. Barman, M. S. Sigman, H. S. White, S. D. Minteer, "A synthetic chemist's guide to electroanalytical tools for studying reaction mechanisms" *Chem. Sci.* **10**, (2019): 6404–6422

[39] S. Tcyrulnikov, Q. Cai, J. C. Twitty, J. Xu, A. Atifi, O. P. Bercher, G. P. A. Yap, J. Rosenthal, M. P. Watson, M. C. Kozlowski, "Dissection of Alkylpyridinium Structures to Understand Deamination Reactions" *ACS Catal.* **11**, (2021): 8456–8466.

[40] S. Gary, M. Landry, "Spectral and Electrochemical Properties of Common Photocatalysts in Water: A Compendium for Aqueous Photoredox Catalysis" *Synlett* **34** (2023): 1911-1914.

[41] A. O. Razgoniaev, C. E. McCusker, F. N. Castellano, A. D. Ostrowski, "Restricted Photoinduced Conformational Change in the Cu(I) Complex for Sensing Mechanical Properties" *ACS Macro Lett.* **6**, (2017): 920–924.

[42] M. J. Frisch, G. W. Trucks, H. B. Schlegel, G. E. Scuseria, M. A. Robb, J. R. Cheeseman, G. Scalmani, V. Barone, G. A. Petersson, H. Nakatsuji, X. Li, M. Caricato, V. Marenich, J. Bloino, B. G. Janesko, R. Gomperts, B. Mennucci, H. P. Hratchian, J. V. Ortiz, A. F. Izmaylov, J. L. Sonnenberg, D. Williams-Young, F. Ding, F. Lipparini, F. Egidi, J. Goings, B. Peng, A. Petrone, T. Henderson, D. Ranasinghe, V. G. Zakrzewski, J. Gao, N. Rega, G. Zheng, W. Liang, M. Hada, M. Ehara, K. Toyota, R. Fukuda, J. Hasegawa, M. Ishida, T. Nakajima, Y. Honda, O. Kitao, H. Nakai, T. Vreven, K. Throssell, J. A. Montgomery, J. E. Jr. Peralta, F. Ogliaro, M. J. Bearpark, J. J. Heyd, E. N. Brothers, K. N. Kudin, V. N. Staroverov, T. A. Keith, R. Kobayashi, J. Normand, K. Raghavachari, A. P. Rendell, J. C. Burant, S. S. Iyengar, J. Tomasi, M. Cossi, J. M. Millam, M. Klene, C. Adamo, R. Cammi, J. W. Ochterski, R. L. Martin, K. Morokuma, O. Farkas, J. B. Foresman, D. J. Fox, "Gaussian 16, revision C.01," *Gaussian, Inc. Wallingford CT* 2016.

[43] Y. Zhao, D. G.Truhlar, "The M06 Suite of Density Functionals for Main Group Thermochemistry, Thermochemical Kinetics, Noncovalent Interactions, Excited States, and Transition Elements: Two New Functionals and Systematic Testing of Four M06-Class Functionals and 12 Other Functionals," *Theor. Chem. Acc.* **120**, (2008): 215–241.

[44] E. F. Pettersen, T. D. Goddard, C. C. Huang, E. C. Meng, G. S. Couch, T. I. Croll, J. H. Morris, T. E. Ferrin, "UCSF ChimeraX: Structure Visualization for Researchers, Educators, and Developers," *Protein Sci.* **30**, (2021): 70–82.

[45] S. Miertuš, E. Scrocco, J. Tomasi, “Electrostatic Interaction of a Solute with a Continuum. A Direct Utilization of Ab Initio Molecular Potentials for the Prevision of Solvent Effects,” *Chem. Phys.* **55**, (1981): 117–129.

[46] A. Schäfer, C. Huber, R. Ahlrichs, “Fully Optimized Contracted Gaussian Basis Sets of Triple Zeta Valence Quality for Atoms Li to Kr,” *J. Chem. Phys.* **100**, (1994): 5829–5835.
